# Molecular Signatures of Proliferation and Quiescence in Hematopoietic Stem Cells

**DOI:** 10.1371/journal.pbio.0020301

**Published:** 2004-09-28

**Authors:** Teresa A Venezia, Akil A Merchant, Carlos A Ramos, Nathan L Whitehouse, Andrew S Young, Chad A Shaw, Margaret A Goodell

**Affiliations:** **1**Cell and Molecular Biology Program, Baylor College of MedicineHouston, TexasUnited States of America; **2**Center for Cell and Gene Therapy, Baylor College of MedicineHouston, TexasUnited States of America; **3**Department of Medicine, Baylor College of MedicineHouston, TexasUnited States of America; **4**Department of Human and Molecular Genetics, Baylor College of MedicineHouston, TexasUnited States of America; **5**Department of Pediatrics, Baylor College of MedicineHouston, TexasUnited States of America

## Abstract

Stem cells resident in adult tissues are principally quiescent, yet harbor enormous capacity for proliferation to achieve self renewal and to replenish their tissue constituents. Although a single hematopoietic stem cell (HSC) can generate sufficient primitive progeny to repopulate many recipients, little is known about the molecular mechanisms that maintain their potency or regulate their self renewal. Here we have examined the gene expression changes that occur over a time course when HSCs are induced to proliferate and return to quiescence in vivo. These data were compared to data representing differences between naturally proliferating fetal HSCs and their quiescent adult counterparts. Bioinformatic strategies were used to group time-ordered gene expression profiles generated from microarrays into signatures of quiescent and dividing stem cells. A novel method for calculating statistically significant enrichments in Gene Ontology groupings for our gene lists revealed elemental subgroups within the signatures that underlie HSC behavior, and allowed us to build a molecular model of the HSC activation cycle. Initially, quiescent HSCs evince a state of readiness. The proliferative signal induces a preparative state, which is followed by active proliferation divisible into early and late phases. Re-induction of quiescence involves changes in migratory molecule expression, prior to reestablishment of homeostasis. We also identified two genes that increase in both gene and protein expression during activation, and potentially represent new markers for proliferating stem cells. These data will be of use in attempts to recapitulate the HSC self renewal process for therapeutic expansion of stem cells, and our model may correlate with acquisition of self renewal characteristics by cancer stem cells.

## Abstract 2

This comprehensive study of gene expression in hematopoietic stem cells reveals some key cellular changes that occur when the stem cells transition from quiescence to proliferation and back again

## Introduction

Hematopoietic stem cells (HSCs) are the best-described adult stem cell population at a phenotypic and functional level. Recent attempts have been made to characterize their molecular regulation by comparing their gene expression profiles with those of other stem cell populations (Ivanova et al. 2002; Ramalho-Santos et al. 2002; Fortunel et al. 2003). These analyses of normal steady-state stem cells revealed so-called stem cell signatures, but the overlap of genes that universally defined “stemness” was extremely limited (Fortunel et al. 2003). Here, we have focused on HSCs alone in order to systematically examine one process, that of HSC self renewal, comprising a cycle of quiescence, proliferation, and reinduction of a dormant state.

In a normal adult, HSCs reside in the bone marrow, where they are relatively inactive. Long-term HSCs divide infrequently to produce more proliferative short-term HSCs, which in turn generate the lineage-committed progenitors that manufacture the billions of differentiated hematopoietic cells that daily enter the peripheral blood. One hallmark of HSCs is their ability to rapidly proliferate in response to stressors such as myelosuppressive chemotherapy or bone marrow transplantation in order to quickly generate work-horse progenitors as well as additional stem cells, which then return to quiescence (Dixon and Rosendaal 1981). While this expansion of HSCs occurs naturally in vivo, there is as yet little understanding of the genes that control this process. A full appreciation of the molecular regulation of stem cell self renewal could illuminate the development of cancers (Sherr 1996) as well as potentially inform strategies for in vitro stem cell expansion, which would have enormous clinical advantages. Thus, we sought to understand the molecular mechanisms by which HSCs respond to an activating trigger, initiate a program of cell division, and resume quiescence by suppression of cell division.

**Figure 1:**
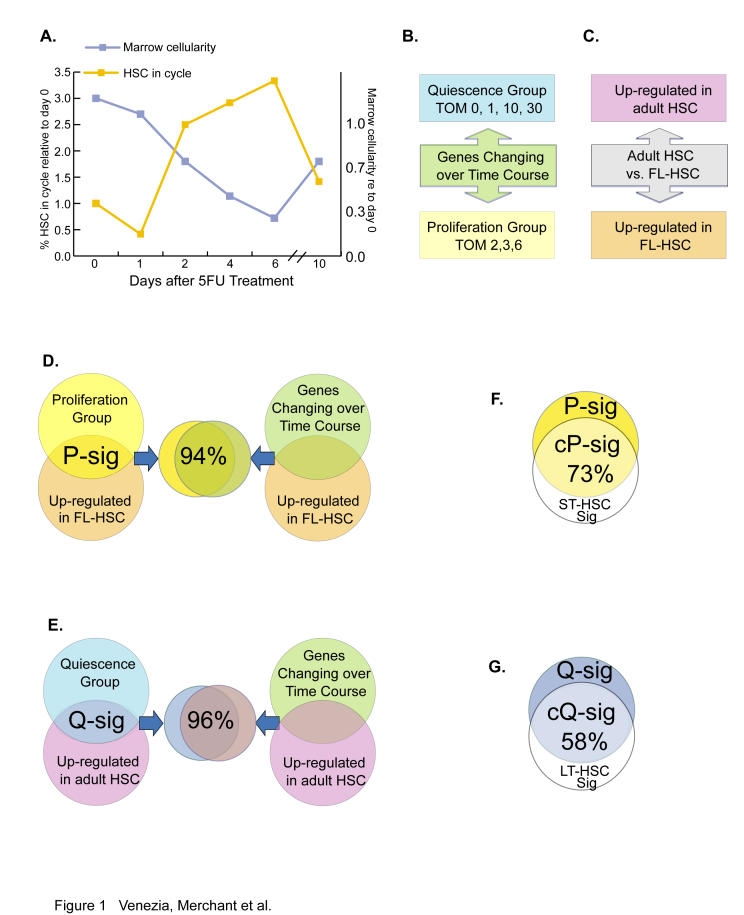
P-Sig and Q-Sig Defined by Gene Expression Levels in HSCs in Different Stages of Cell Cycle (A) Graphic depicting the changes in bone marrow cellularity and number of HSCs in cell cycle following 5FU treatment (adapted from Harrison and Lerner 1991; Randall and Weissman 1997)*.* (B) Schematic of 5FU-HSC time course analysis. The genes that change over the time course can be split into two groups based on the day of maximum expression (TOM). (C) Schematic of pair-wise comparison between quiescent adult HSCs and FL-HSCs, showing groups of genes either up-regulated in the quiescent adult cells or up-regulated in the cycling FL-HSCs. (D) Genes that were both up-regulated in FL-HSCs and were in the proliferation group composed the P-sig. The P-sig shows 94% overlap with the group of genes that were up-regulated in FL-HSCs and changed over the time course. (E) Genes that were both in the quiescence group and up-regulated in adult HSCs were termed the Q-sig. The Q-sig overlaps 96% with the set of genes that were up-regulated in adult HSCs and changed over the time course. (F) Overlap of the ST-HSC signature with P-sig revealed 73% in common, defining the common P-sig. (G) Overlap of the LT-HSC signature with Q-sig revealed 58% in common and defined the common Q-sig. This figure is interactive online, and provides contextual access to Tables S1–S11. Use your mouse to highight animated areas of the graphic. Click on these areas to link to related files.

Our approach was to examine the transcriptional profiles of purified adult HSCs throughout a time course of induced proliferation, and compare the gene expression in these cells to that of naturally dividing fetal liver HSCs (FL-HSCs). Normal adult HSCs are largely nondividing, with around 1%–3% in cycle and approximately 90% in G0 (Morrison and Weissman 1994; Goodell et al. 1996; Bradford et al. 1997; Cheshier et al. 1999). A single injection of the pyrimidine analog 5-fluorouracil (5FU) kills cycling hematopoietic cells, bringing the spared quiescent HSCs into cycle to repopulate the depleted bone marrow (Van Zant 1984; Harrison and Lerner 1991; Randall and Weissman 1997). HSC proliferation proceeds in a time-dependent manner, peaking 5 to 6 d after treatment, with approximately 20% of HSCs in cycle, before returning to normal around day 10 (Figures 1A and S1; Randall and Weissman 1997). Changes in the cell surface profile concomitant with cell cycle activation have been observed. The receptor tyrosine kinase c-Kit, normally expressed at high levels in quiescent HSCs, is down-regulated after 5FU treatment (Randall and Weissman 1997). Conversely, the markers Mac1 and AA4.1, absent on normal HSCs, are expressed at low levels after 5FU treatment (Szilvassy and Cory 1993; Randall and Weissman 1997).

During the latter part of mammalian embryonic development, HSCs reside in the fetal liver, where they undergo a massive expansion prior to entering the bone marrow. Approximately 30% of murine FL-HSCs are in cycle (Morrison et al. 1995), and similar to 5FU-activated HSCs (5FU-HSCs), they express AA4.1 and Mac1 (Jordan et al. 1990; Morrison et al. 1995). Given the similarities between 5FU-activated HSCs and FL-HSCs, we hypothesized that they would share similar gene expression profiles vis-à-vis proliferation and that simultaneous comparison of FL-HSCs, adult quiescent HSCs, and 5FU-HSCs would define genes specifically involved with HSC proliferation. Indeed, we defined both proliferation and quiescence signatures for HSCs, validated these groupings using Gene Ontology (GO) classifications, and created a model of the HSC proliferation cycle.

## Results

### Experimental Design

Our overall approach was to isolate highly purified HSCs in the states described above, obtain their gene expression profiles using Affymetrix microarrays, and apply statistical and bioinformatics methods to facilitate comparisons among the samples. To construct a profile of the time-dependent induction of HSC proliferation, 5FU-HSCs were isolated at days 0, 1, 2, 3, 6, 10, and 30 after treatment.

Adult quiescent HSCs and 5FU-HSCs were isolated according to Hoechst 33342 efflux, termed the side population (SP) and Sca1^+^ characteristics (Goodell et al. 1996) (Table 1; Figure S2A). Further analysis of these populations revealed them to be highly homogeneous with more than 97% having Sca1^+^/Lineage^−^ characteristics (Figure S3). Transplantation into lethally irradiated hosts, performed for both quiescent and 5FU-treated SP/Sca1^+^ cells, confirmed their stem cell activity (data not shown). FL-HSCs were isolated by FACS for AA4.1^+^, c-Kit^+^, Sca1^+^, and Lineage^−^ characteristics from embryos 13.5–14.5 d postcoitus (Jordan et al. 1990) (Table 1; Figure S2B.) RNA probes were prepared from HSCs using two rounds of in vitro transcription and applied to Affymetrix MGU74Av2 microarrays. Hybridization, scanning, and production of raw data files were performed according to standard protocols. To correct raw intensity values for systemic variables such as fragmentation efficiency, hybridization conditions, and scanner effects, microarrays were normalized before intensity values were converted to gene expression measures. Normalization and model-based expression measurements were performed with GeneChip Robust Multichip Analysis (Wu et al. 2003), which is more precise and accurate in estimating fold changes than Affymetrix MAS 5.0 and the recently published Robust Multichip Analysis method (Irizarry et al. 2003), and is available as part of the open-source Bioconductor project (http://www.bioconductor.org). Further statistical analysis was performed in R (http://www.r-project.org). Quality control was performed both pre- and postnormalization. Briefly, chips were inspected for spatial defects, intensity outliers, and amplification bias. After screening, the two chips representing biological replicates with the highest correlation (R^2^ = 0.97–0.99, average = 0.98) in each group or time point were selected for further analysis. Raw data and normalized expression data are available for download from Gene Expression Omnibus (http://ncbi.nlm.nih.gov/geo) or http://franklin.imgen.bcm.tmc.edu/SCGAP/downloads/SPTimecourse. Normalized expression data along with all filtering criteria used to obtain our gene lists are available in Table S46. A gene-by-gene query tool is available at http://franklin.imgen.bcm.tmc.edu/PLoS.

**Table 1:**
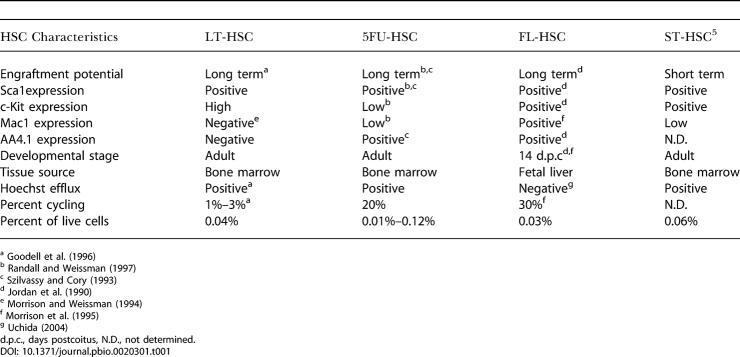
Comparison of Phenotypic and Functional Characteristics of HSC Populations

^a^ Goodell et al. (1996)

^b^ Randall and Weissman (1997)

^c^ Szilvassy and Cory (1993)

^d^ Jordan et al. (1990)

^e^ Morrison and Weissman (1994)

^f^ Morrison et al. (1995)

^g^ Uchida (2004)

d.p.c., days postcoitus, N.D., not determined

### Time of Maximum Grouping Reveals Strong Time Ordering to Expression Data

We began our analysis of the 5FU time course by identifying genes that varied over time. This was accomplished by fitting smooth curves to the expression profiles using regression analysis with time as a continuous variable. ANOVA on these profiles revealed 1,488 genes that showed a significant change over the time course (*p* < 0.05). Principle component analysis revealed that the time course data consisted of two major groups: genes that up-regulated and genes that down-regulated over the time course (data not shown). We further explored the expression data with unsupervised (k-means) clustering and observed that when the number of predefined groups was low (2–3), only the overall pattern of up- or down-regulation was discernable; however, as we increased the number of groups (4–8), more complex patterns with peaks early or late in the time course were visible (data not shown). Since the 5FU treatment consisted of a single dose administered at time zero, we speculated that the downstream effects of 5FU treatment would be represented by groups of genes whose gene expression profiles showed time-ordered peaks propagating through the time course. The expression profile of groups created by k-means clustering supported this hypothesis. Therefore, to more directly delineate these peaking subsets, we sorted the genes into groups by their time of maximum expression (TOM). Strikingly, these groups had two predominant patterns over the time course: one group up-regulated with 5FU treatment with a TOM at day 2, 3, or 6, and one group down-regulated, exhibiting TOM at day 0, 1, 10, or 30 (Figure 1). By correlating these patterns to HSC cell cycle status after 5FU treatment (Figure 1A), we assigned the up-regulated genes to the “proliferation” group (680 genes) and the down-regulated genes to the “quiescence” group (808 genes) (Figure 1B).

**Figure 2:**
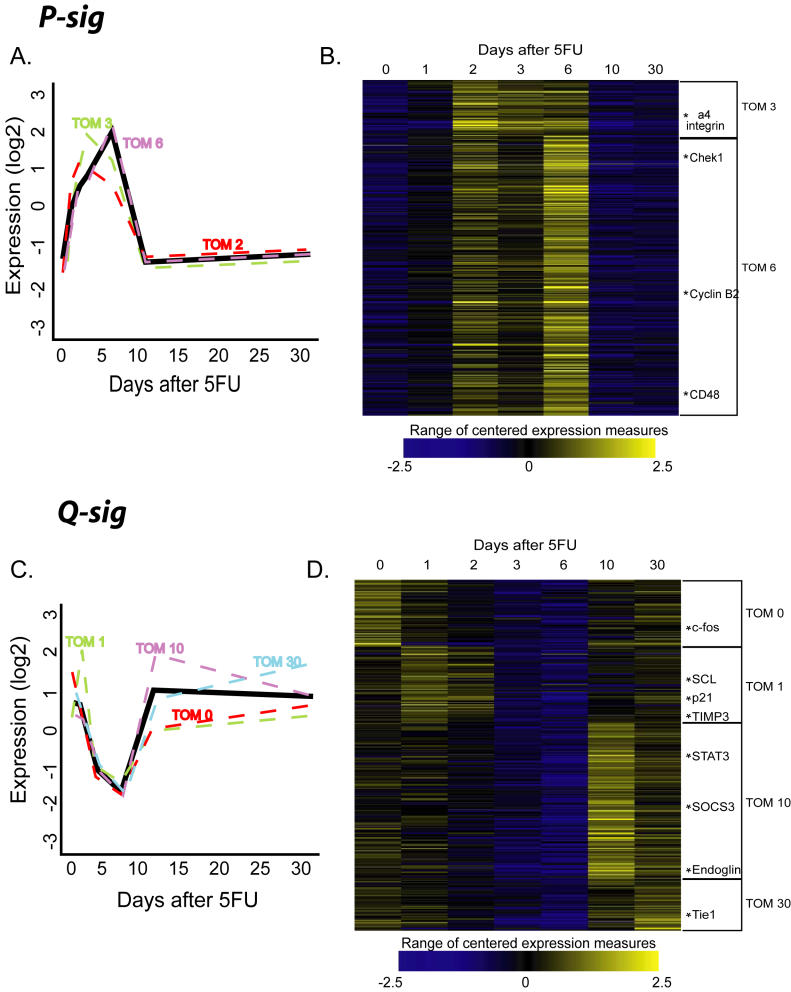
P-Sig and Q-Sig Show Patterns of Activation and Down-Regulation with Respect to Cell Cycle Status (A) Averaged pattern of P-sig gene expression over the 5FU time course plotted in solid lines, with the contributing TOM subgroups plotted in dashed lines. (B) Heat map of each gene in P-sig over the 5FU time course showing TOM subgroups in brackets. (C) Averaged pattern of Q-sig gene expression over the 5FU time course plotted in solid lines, with the contributing TOM subgroups plotted in dashed lines. (D) Heat map of each gene in Q-sig over the 5FU time course showing TOM subgroups in brackets. For both heat maps, relative expression levels are displayed according to color intensity, blue (lowest) to yellow (highest). This figure is interactive online, and provides contextual access to Tables S12–S18. Use your mouse to highight animated areas of the graphic. Click on these areas to link to related files.

To validate these time-dependent expression-pattern-based gene groupings, we compared our quiescence and proliferation groups to the genes differentially expressed between quiescent adult HSCs and FL-HSCs. The latter were identified in a pair-wise comparison between adult HSCs and FL-HSCs that revealed 1,772 genes that were at least 2-fold differentially expressed (Figure 1C). Since FL-HSCs are in cycle, as are 5FU-HSCs, a list of genes expressed in common between the time-course-defined proliferation group and those up-regulated in FL-HSCs should specifically contain genes involved in HSC proliferation, eliminating genes involved in interacting with their very different source environments. We designated this list of 338 genes our “proliferation signature” (P-sig; Figure 1D). Likewise, the 298 genes in common between the time-course-defined quiescence group and those up-regulated in adult HSCs relative to FL-HSCs defined our “quiescence signature” (Q-sig; Figure 1E). In Figure 2B and 2D, each gene within the P-sig and Q-sig is represented by a single line, and its relative expression along the time course is represented by the intensity of the colors on the heat map. Genes discussed later in the text are highlighted. To examine whether similar signatures could be generated without the TOM groupings (which could potentially introduce a bias), we examined the list of genes overlapping between the set of those up-regulated in FL-HSCs and the entire set of genes that change during the time course (see Figure 1D). A striking 94% of the P-sig overlaps with these genes. Similarly, 96% of genes in the Q-sig overlap with the set of genes that are up-regulated in adult HSCs and change over the entire time course (see Figure 1E). In other words, overlapping the pair-wise comparison with our expression-pattern-based groups, i.e., TOM groupings, identified essentially the same genes as did overlapping the time course with quiescent adult HSC and FL-HSC data, thus correlating, at the gene level, the TOM groupings to populations with known biological differences.

We then plotted the average pattern for the P-sig and Q-sig and examined their component TOM groups (see Figure 2). The patterns of genes in the TOM subgroups of the P-sig were very similar, with an overall off-on-off pattern that corresponded to the number of HSCs in cycle after 5FU treatment (see Figures 1A, 2A, and 2B). Although mutually exclusive gene lists, TOM 0 and 30 were almost identical in pattern and were highly similar at the functional level (see below). Genes in TOM 1 and 10 shared the overall pattern of down-regulation with the Q-sig, but showed early and late peaks, respectively, the significance of which is discussed below. Overall we found the individual TOM groups to be highly coherent with a high degree of correlation between the individual genes and the mean profile of each group (Table S47).

### Q-sig and P-sig Overlap with Published Data to Give “Common” Signature

Encouraged by these results, we performed a parallel analysis on a raw dataset from Akashi et al. (2003), who compared the transcriptional profiles of adult long-term HSCs (LT-HSCs) and short-term HSCs (ST-HSCs). Although isolated by different methods, the Rho^low^ KTSL cells isolated by Akashi et al. and our quiescent adult HSCs are functionally equivalent (Wolf et al. 1993; Goodell et al. 1996). ST-HSCs have the ability, as do LT-HSCs, to contribute to all lineages of the hematopoietic system, but are not able to maintain long-term engraftment in irradiated hosts. They are also more in cycle than LT-HSCs and express low levels of Mac1 (Table 1) (Morrison and Weissman 1994; Cheshier et al. 1999). We therefore suspected that the genes 2-fold differentially expressed between LT-HSCs and ST-HSCs, approximately 300 and 600 genes, respectively, would be enriched for quiescence and proliferation genes, respectively. When we compared these lists with the list of genes changing after 5FU treatment, we observed that almost all the genes in common between LT-HSC and time course lists were in the quiescence group list. Similarly, most of the genes in common between the ST-HSC and the time course lists were in the proliferation group list. This confirmed that many of the gene expression changes that occur between LT-HSCs and ST-HSCs are the same changes that occur after activation of HSC with 5FU, and we designated these list intersections as the LT-HSC signature and ST-HSC signature, respectively.

A natural question was whether the Q-sig and P-sig described above would have any overlap with the LT-HSC signature and ST-HSC signature groups. Remarkably, 58% of the genes were in common between LT-HSC signature and the Q-sig, and 73% of the genes were in common between ST-HSC signature and the P-sig. We named these highly selected lists (53 and 118 genes, respectively) the “common quiescence signature” (cQ-sig) and “common proliferation signature” (cP-sig) (Figure 1F and 1G). As we show below, these “common” signatures derived from the three-way intersection of 5FU-HSC data, adult-HSC-versus-FL-HSC data, and LT-HSC-versus-ST-HSC data were highly enriched for genes related to HSC proliferation.

### Novel Uses of Gene Ontologies Allowed Functional Validation of Gene Groupings

**Figure 3:**
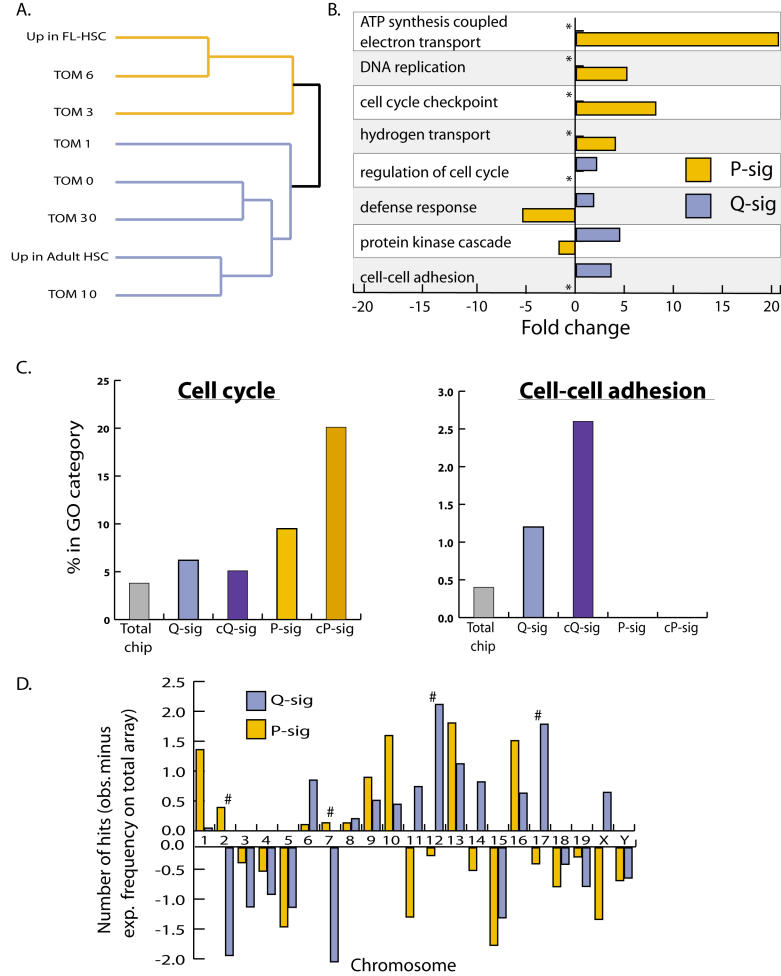
GO Analysis and Chromosomal Clustering (A) Dendrogram of gene lists clustered solely according to their similarity in GO content. (B) Bar graph showing enrichments of selected GO groups in the Q-sig and P-sig. Fold changes are relative to whole microarray (*p* < 0.05). Asterisk marks groups in which no genes were found (complete depletion). (C) Percentage of genes within each list that are in the GO groups “cell cycle” or “cell–cell adhesion.” (D) Distribution of hits within Q-sig and P-sig on each chromosome normalized for number of expected hits for whole microarray. Pound sign denotes significant differences between Q-sig and P-sig (*p* < 0.05). This figure is interactive online, and provides contextual access to Tables S19–S45. Use your mouse to highight animated areas of the graphic. Click on these areas to link to related files.

To investigate the biological significance of the groupings described above, we developed novel methods for utilizing the GO annotations (Ashburner et al. 2000) (http://www.geneontology.org) to analyze the content of gene lists. The GO is a controlled vocabulary that describes gene functions in their cellular context and is arranged in a quasi-hierarchical structure from more general to more specific. Since the vocabulary of annotations is fixed, it allows for functional comparisons of mutually exclusive gene lists, such at the TOM groups. We began by mapping each gene in the lists being analyzed to the GO tree structure. This allowed us to count the number of times each gene hit at or below any particular node in the GO structure. Once the lists were mapped, we were able (a) to calculate a measure of similarity (distance) between the lists using the distributions of each list across the various levels of the GO tree and (b) to calculate the enrichment of the various GO categories in each list (Figure 3A–3C).

We clustered the gene lists based on this distance metric (Figure 3A). As can be seen, GO-based clustering recapitulated the previous expression-pattern-based groupings: TOM days 0, 1, 10, and 30 clustered with the list of genes up-regulated in adult HSCs; and TOM days 3 and 6 clustered with those genes up-regulated in FL-HSCs. We calculated a probability of 0.003 that we could arrive at the grouping pattern shown by chance. Importantly, this indicated that the content of these clusters, as defined by their biological process using GO, was highly similar despite the nonoverlapping nature of the TOM groups. Although recapitulating the expression-pattern-based groupings, our GO-based clustering also revealed that TOM 1 has a unique signature amongst the quiescence cluster, suggesting a distinctive role for the genes in this group in governing HSC quiescence (Figure 3A).

Our strategy for mapping gene lists to the GO structure also allowed us to calculate statistically significant enrichments of particular GO categories within our gene lists. We achieved this by mapping the whole microarray (approximately 12,000 genes) onto the GO structure and then calculating fold enrichments for each GO category in our lists relative to the microarray. We expected to find differences between the Q-sig and P-sig in the frequencies of antiproliferative and proproliferative genes, and verification of this served as proof-of-principle for our experimental design. Indeed, we found the GO category “regulation of cell cycle” (containing genes like the antiproliferative genes *p21 [cyclin-dependent kinase inhibitor 1A]* and *GADD45β [Growth arrest and DNA-damage-inducible 45, beta]*) to be 2.1-fold increased in the Q-sig over the total array (Figure 3B). Moreover, the category “DNA replication” was about 5-fold greater in the P-sig, while this category was absent in Q-sig (Figure 3B).

Intriguingly, the GO group “defense response,” containing many of the H2 genes of the MHC class I family, was slightly enriched in the Q-sig, but was depleted by over 5-fold in the P-sig (Figure 3B). Signal transduction molecules such as those in the GO groups “protein kinase cascade” were enriched 4.3-fold in the Q-sig (Figure 3B). The GO group “ATP synthesis coupled electron transport” was enriched almost 21-fold in the P-sig, which correlates with the high energy requirements of cell division (Figure 3B).

As discussed above, our results and the data from Akashi et al. (2003) have remarkable overlap at the gene level. Using the common signature lists, we observed further refinements in key GO categories. For example, “cell cycle” genes were less than 4% of all genes on the chip, yet they represented 21% of the genes in the common P-sig (Figure 3C). Progressive enrichment in “cell–cell adhesion” was also observed (Figure 3C). Although almost 19% of the genes in our “common” signatures have no previously defined biological process, given the remarkable enrichment of proliferation-related genes in our common signatures, we can infer that they also may be involved in HSC proliferation.

### TOM Analysis Uncovered Orderly Progression of HSC Activation

We further utilized the GO-based analysis of the TOM groups within the Q-sig and P-sig to gain insight into the biological activities of HSCs at these time points. Because of the high similarity of TOM 0 and 30 in both expression pattern and GO categorization, we treated them as a single group. “Regulation of transcription” was enriched 1.5-fold in TOM 0 and 30 and comprised 16 genes, including several key transcriptional regulators of cell cycle such as the oncogenes *c-fos* and *c-maf,* as well as the global transcriptional repressor *histone deacetylase 5*.

The GO categories “regulation of cell cycle,” “cell–cell adhesion,” and “defense response” were specifically enriched in TOM 1 (approximately 4-fold each). Many genes in these groups are negative regulators of cell cycle, such as *p21, Tob 1/APRO6 (Transducer of ErbB2.1-1), Btg3/APRO4 (B-cell translocation gene 3), cyclin G1, GADD45β, and melanoma antigen, family D, 1*. Prior experiments have shown a decrease in the number of HSCs in cycle during the first day after 5FU treatment as compared to untreated HSCs (see Figure 1A; Randall and Weissman 1997). We therefore concluded that many of the genes in TOM 1 are responsible for this momentary pause in cell cycle, and this explained why these genes were initially up-regulated and then sharply down-regulated as rapid HSC proliferation began (see Figure 1A and 2C).

In the P-sig, TOM 3 and TOM 6 showed astonishingly different GO contents despite their similar expression patterns (see Figure 2A). Genes in the GO category “cell cycle” identified in the P-sig are concentrated in TOM 3. Specifically, genes in both “DNA replication” and “M phase” were enriched about 18-fold and 10-fold, respectively, indicating a preparation for cell division. TOM 6 was enriched almost 3-fold in genes involved with biosynthesis of many essential cellular components, such as ATP (8.8-fold), nucleotides (5.6-fold), and proteins (2.4-fold). These data suggest early and late phases of proliferation, represented by the genes in TOM 3 and TOM 6, respectively.

As discussed above, by day 10 after 5FU treatment the number of HSCs in cycle is reduced to near pretreatment levels (see Figure 1A). Although the signals responsible for restoring quiescence remain elusive, we believe that this process may be mediated by JAK/STAT and other signaling pathways. Overall, the GO category “signal transduction” showed approximately 2-fold enrichment in the TOM day 10 list. *SOCS3 (Suppressor of cytokine signaling 3),* whose product suppresses responses to growth factors in part by inhibiting JAK/STAT signaling, was most highly expressed at day 10, along with *STAT3* and *STAT6*. JAK/STAT signaling has been implicated in regulation of proliferation and differentiation of various hematopoietic cell types.

### Chromosomes 2, 7, 12, and 17 Contain HSC Proliferation Control Regions

Our expression data can be combined with data from the mouse genome projects to correlate gene expression changes observed after 5FU treatment with higher order genome-wide regulation. For example, we analyzed the contents of Q-sig and P-sig for clustering on particular chromosomes. Four chromosomes exhibited significant enrichment between the two signatures: Chromosomes 12 and 17 were enriched in the Q-sig, and Chromosomes 2 and 7 were enriched in the P-sig (see Figure 3D). Earlier work identified quantitative trait loci (QTL) on Chromosomes 17 and 7 associated with the control of HSC frequency and proliferation of hematopoietic progenitors, respectively (Phillips et al. 1992; Geiger et al. 2001). *p21,* a prototypic member of the Q-sig, was specifically found within a QTL on Chromosome 17 associated with regulating HSC frequency. This region is syntenic with human Chromosome 6p21, a known hot spot for translocations linked to leukemias and lymphomas (Huret et al. 1986; Johansson et al. 2002).

### Microarray Gene Expression Changes Reflect Changes in Protein Expression and HSC Behavior

**Figure 4:**
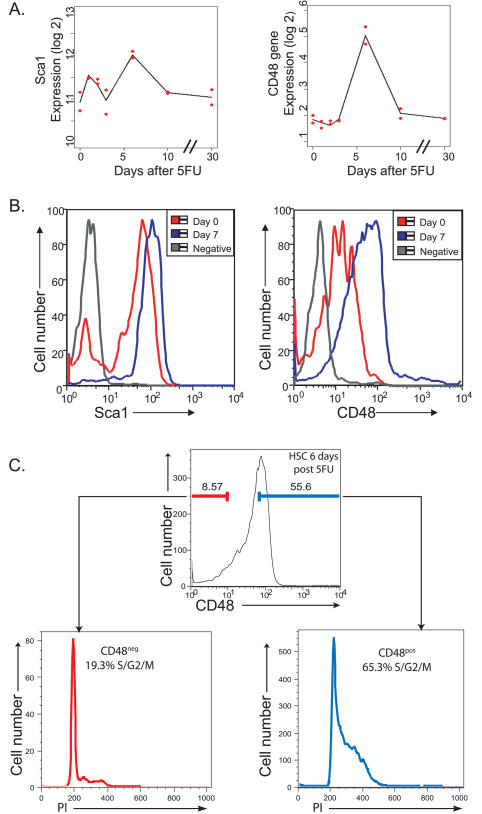
Gene Expression Profiles Correlate with Protein Expression on HSCs (A) Gene expression over time. The actual observed values of each replicate at each time point are shown in red, and the line connects the predicted expression value at each time point based on our regression analysis. (B) Antigen expression on HSCs measured by flow cytometry. Gray lines represent negative control, red lines represent protein expression at day 0, and blue lines represent protein expression at day 7. (C) Cell cycle analysis of CD48^−^ and CD48^+^ HSCs isolated 6 d post 5FU treatment.

In order to determine whether some of the observed gene expression changes were accompanied by measurable differences in protein expression, we identified two genes whose expression changed over time and whose product could be tracked using flow cytometry. Gene expression of *Sca1,* a known marker of HSCs, showed significant increase after 5FU treatment despite having a high starting level (Figure 4A). Flow cytometric analysis showed that Sca1 antigen expression was also distinctly higher after 5FU (Figure 4B). *Sca1*-null mice have a defect in HSC self renewal that has been interpreted as a loss of proliferative capacity (Ito et al. 2003). Our data support this finding since maximal expression of Sca1 both at the gene expression and protein level was at day 6/7 post 5FU treatment. We also analyzed CD48, a cell adhesion molecule previously associated with T-cell activation and proliferation (Kato et al. 1992; Chavin et al. 1994; Gonzalez-Cabrero et al. 1999), which peaked in gene expression 6 d after 5FU treatment (Figure 4A). By flow cytometry, CD48 antigen was detected on quiescent HSCs, but exhibited a substantially higher level of expression at the height of HSC proliferation (Figure 4B). To determine whether high levels of CD48 antigen on HSCs coordinated with proliferation in a similar fashion as on T-cells, we performed cell cycle analysis of CD48^+^ and CD48^−^ HSCs. Further characterization of CD48^+^ HSCs 6 d post 5FU revealed a greater than 3-fold enrichment in the number of cells in cycle over CD48^−^ HSCs (Figure 4C). This finding is the first report of a marker that enriches for cycling HSCs.

## Discussion

Here we have identified proliferation and quiescence signatures of HSCs. Our experimental design utilized a combination of pair-wise comparisons and time course microarray experiments. The pair-wise analysis allowed us to find the genes different between quiescent and cycling HSCs, while the time course data allowed us to order these genes in a time-dependent manner. The power of our overall methodology is reflected in the remarkable overlaps between the gene lists presented and those extracted from published data (Akashi et al. 2003), in particular the common P-sig and common Q-sig.

Applying a novel approach to utilizing the GO annotations, we calculated the statistical significance of the enrichment of particular GO categories in our lists. We also devised a new method for calculating the distance between gene lists based on the GO structure. This allows one to assess the functional similarity, in “GO space,” of gene lists that may not have any actual genes in common (such as our TOM groups). Furthermore, since the GO vocabulary is not specific to any species, this method allows for cross-species and cross-platform comparisons of gene lists. Re-analysis of data from previous studies may reveal a functional stem cell signature in GO space that was not evident at the gene level (Ivanova et al. 2002; Ramalho-Santos et al. 2002; Fortunel et al. 2003).

**Figure 5:**
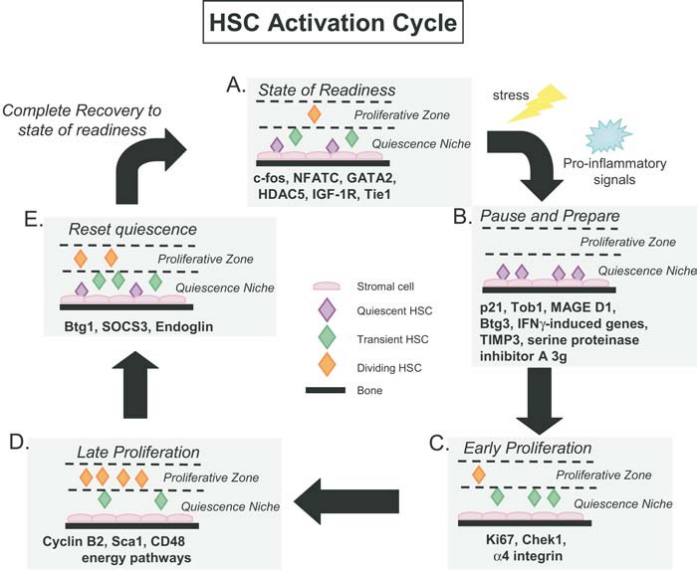
Model of HSC Activation Cycle (A) Normal HSCs reside in a quiescent niche in a “state of readiness” exemplified by the indicated genes. (B) Upon stress (5FU treatment), HSCs “pause” by remaining quiescent and in their niche while they “prepare” to proliferate. HSCs receive signals from proinflammatory cytokines at this point. The signals induce a proliferative state that is divisible into early (C) and late (D) phases. (C) “Early proliferation” is marked by an increase in expression of genes involved in DNA replication, repair, and cell migration molecules that allow movement of HSCs from the quiescence niche to the proliferative zone. (D) “Late proliferation” is marked by expression of many cell cycle genes as well as many energy pathway molecules. (E) Re-induction of quiescence involves changes in migratory molecule expression, which leads to return of cells to their quiescence niche, as well the expression of antiproliferative genes.

Applying GO analysis to the TOM groupings revealed elemental subgroups within the signature lists that allowed us to construct a molecular model of the HSC activation cycle. The majority of unperturbed HSCs reside in a quiescence niche and express receptors, for example the metabolism- and ageing-associated receptor IGF1R and the receptor tyrosine kinase Tie1, that allow them to respond to multiple mitogenic signals (Figure 5A). They also express high levels of transcription factors, such as c-fos and GATA-2, that enable swift activation of HSCs. This expression profile, found in the TOM 0 and 30 groups, suggests that although adult HSCs are quiescent, they are in a “state of readiness” to react to changes in their environment.

Immediately after activation is triggered (here by 5FU), HSCs enter a superquiescent “pause.” This state, found at TOM 1 and also observed by cell cycle analysis (Randall and Weissman 1997), is mediated by antiproliferative genes such as *Tob1, p21,* and *Btg3* (Figure 5B). Interestingly, *p21*-null mice have defects in HSC self renewal (Cheng et al. 2000). We observed up-regulation of TIMP3 and the serine proteinase inhibitor A-3 g, which inhibit cell migration (Qi et al. 2003). At least six interferon-γ-induced genes were also up-regulated at this point, suggesting that HSCs are responding to proinflammatory signals. We speculate that the pause in HSC proliferation and migration allows HSCs to survive 5FU cytotoxicity while the cells simultaneously “prepare” to proliferate and repopulate the bone marrow to ensure survival of the animal.

In the early phase of proliferation starting at day 3, when increased numbers of HSCs in cell cycle are first detected (Randall and Weissman 1997), HSCs have committed to cell division, as can be seen by the maximal expression of genes involved in DNA replication and repair (Figure 5C). At day 6, the late phase of proliferation, when the greatest number of HSCs are in cycle, we see expression of genes involved with energy production, indicating an overall increase in metabolic activity in the HSCs (Figure 5D). Prior work has linked HSC mobilization with proliferation (Wright et al. 2001; Heissig et al. 2002), and our data indicate that the opposite is also true: to proliferate, HSCs need to move out of their quiescence niche and into a proliferative zone (Figure 5C and 5D). We see the up-regulation of *α4-integrin* at day 3 followed by a dramatic decrease at day 6 post 5FU treatment. Experiments that block α4-integrin function by blocking antibodies or via knockout technology have previously shown that down-regulation induces increased mobilization and delays recovery after 5FU treatment (Craddock et al. 1997; Scott et al. 2003). The gene expression pattern displayed by α4-integrin predicts that down-regulation of α4-integrin is necessary for 5FU-induced proliferation. As stated above, down-regulation of α4-integrin is sufficient to alter recovery of bone marrow progenitors after 5FU treatment, supporting the link between HSC proliferation and migration in our model. Down-regulation of c-Kit has also been linked to mobilization of HSCs (Heissig et al. 2002), and its expression is lowest at day 6 post treatment.

In order to “reset quiescence,” HSCs need to return to their niche (Figure 5E). This process begins at day 10, when the number of cycling HSCs falls and HSCs express the high levels of specific antiproliferative genes such as *Btg1* and several components of the JAK/STAT signal transduction pathway. Both SOCS3 (Soriano et al. 2002) and STAT3 (Levy and Lee 2002) have been associated with both positive and negative regulation of proliferation and differentiation of various hematopoietic cell types. Simultaneous expression of *SOCS3, STAT3,* and *STAT6* suggests a complex regulation of HSC quiescence, but earlier work examining STAT signaling in other stem cell populations gave us insight into the role of JAK/STAT signaling in HSCs. Expression of STATs has been shown to establish and maintain stem cell pluripotency in embryonic stem cells (Raz et al. 1999). However in *Drosophila* testes, JAK/STAT activation is crucial for stem cell self renewal; perturbations by both loss and increase in expression lead to dramatic changes in the stem cell compartment (Kiger et al. 2001). Notably, activation of the JAK/STAT pathway by PKD1 induces cell cycle arrest through p21-dependent mechanisms (Bhunia et al. 2002). This supports our hypothesis that JAK/STAT signaling is important for inducing quiescence at day 10, since we have shown that p21 is likely involved in HSC cell cycle arrest. The involvement of JAK/STAT signaling in both stem cell pluripotency and HSC quiescence suggests that these processes may be linked in HSCs.

Endoglin, also found in the TOM 10 group, is known to be expressed on both murine (Chen et al. 2002) and human (Pierelli et al. 2001) HSCs, and has been shown to decrease cell migration by increasing cell–cell adhesion (Liu et al. 2002). Its expression pattern was negatively correlated with 5FU-HSC proliferation: it was lowest at day 6 after 5FU treatment, and highest at day 10. Our data suggest that HSC proliferation requires mobilization from the niche, and that restoration of quiescence is accompanied by a return to the niche. Endoglin's expression pattern makes it an ideal candidate for mediating HSC-to-niche homing and long-term association.

Our model derived from gene expression profiles correlates well with the literature on HSC cell cycle and mobilization. Although other models of HSC mobilization and 5FU treatment have previously been proposed (Heissig et al. 2002), our data allow association of specific genes with particular stages of HSC activation and recovery. Our model predicted that CD48 might preferentially mark cycling HSCs, and our cell cycle analysis of CD48^+^ and CD48^−^ HSCs confirmed this prediction. Our model also postulates the presence of “quiescence” and “proliferative” zones in the bone marrow; osteoblasts may be a component of this quiescence niche (Calvi et al. 2003; Zhang et al. 2003).

In summary, we present proliferation and quiescence signatures for HSCs that show remarkable overlap with published literature. In addition, this study revealed new, uncharacterized genes whose role in HSC self renewal needs to be explored: some of these genes may play as yet undiscovered roles in the development of cancer or may aid in the manipulation of stem cells for therapeutic uses. Finally, harnessing the GO using novel bioinformatics approaches to analyze our data at a global level allowed us to propose a model of the HSC activation cycle.

## Materials and Methods

### 

#### Flow cytometry

For quiescent adult HSCs and 5FU-HSCs, whole bone marrow (WBM) was collected from the femurs and tibias of ten to fifteen 8- to12-wk-old normal or 5FU-treated C57Bl/6 mice. For 5FU treatment, mice were injected intravenously with a single dose of 5FU (150 mg/kg body weight; Sigma, St. Louis, Missouri, United States) and killed at day 0, 1, 2, 3, 6, 10, or 30 after injection. Day 0 mice were untreated, and day 1 WBM was isolated 17–19 h after injection; all subsequent days were in 24-h increments. WBM was stained with Hoechst 33342 to identify the SP cells (Goodell et al. 1996) and then magnetically enriched for Sca1^+^ cells (autoMACS; Miltenyi Biotec, Sunnyvale, California, United States). Cells were stained with a biotinylated Sca1 antibody (clone E13–161.7; BD Pharmingen, San Diego, California, United States) and visualized with strepavidin-PE (Molecular Probes, Eugene, Oregon, United States). Sca1-enriched WBM was sorted for the SP profile and Sca1 positivity on a MoFlo (Cytomation, Fort Collins, Colorado, United States). Representative flow diagrams of cell sorting can be found in Figure S2A. Phenotypic purity was typically 95% or greater. Regarding functional purity of the sorted populations, evidence from multiple sources in our lab and others suggests that both normal bone marrow and 5FU-treated SP cells are very highly enriched for HSCs. The whole SP contains both LT-HSCs and ST-HSCs, but has very limited contamination from committed progenitors or differentiated hematopoietic cells.

For FL-HSCs, fetal livers were removed from embryos 13.5–14.5 d postcoitus and dissociated (Jordan et al. 1990). Fetal liver cells were magnetically enriched for c-Kit^+^ cells using c-Kit-biotin (clone 2B8, BD Pharmingen) and visualized with strepavidin-APC (Molecular Probes). The c-Kit-enriched cells were stained with a lineage cocktail consisting of cychrome-conjugated CD4 (L3T4), CD8 (53–6.7), B220 (RA3–6B2), GR1 (RB6–8C5), and Ter119 (Ter119) as well as Sca1-PE, and AA4.1-FITC (all antibodies from BD Pharmingen). FL-HSCs were identified as negative for the lineage markers and positive for Sca1, c-Kit, and AA4.1 (see Figure S2B). Percentage of enriched cells was between 0.02% and 0.04% of total cells, with a purity of approximately 90%.

For protein expression validation, SP cells from days 0 and 7 post 5FU treatment were analyzed for expression of Sca1-FITC, c-Kit-APC, and CD48-PE (HM48–1, BD Pharmingen) by flow cytometry.

#### RNA isolation and amplification

Total RNA was isolated from approximately 35,000–70,000 sorted HSCs using the RNaqeuous kit (Ambion, Austin, Texas, United States). All samples were then digested with DNaseI to eliminate residual genomic DNA, and extracted with phenol:chloroform. Total RNA was then subjected to two rounds of linear amplification using T7-based in vitro transcription (IVT) (MessageAmp, Ambion). Briefly, total RNA was reverse transcribed with an oligo-dT primer containing a T7 promoter sequence at the 5′ end (oligo-dT-T7 primer). To prime second-strand synthesis, RNA–cDNA hybrids were digested with RNaseH, producing patches of single-stranded cDNA. The second strand was filled in by DNA polymerase. The double-stranded cDNA served as a template for T7 RNA polymerase-driven IVT, which yielded up to 100× the starting mRNA pool. RNA probes were labeled in the second round of IVT with biotinylated nucleotides (Enzo Biotech, Farmington, Connecticut, United States). The second round of amplification was performed using random primers for first-strand synthesis and the oligo-dT-T7 primer to prime second-strand synthesis. Overall amplification was estimated to be 10,000-fold or greater (Gallardo et al. 2003).

#### Microarray hybridization

Affymetrix (Santa Clara, California, United States) MG-U74Av2 chips were hybridized with fragmented, biotinylated aRNA according to standard protocols. Chips were then washed and counterstained using PE-conjugated strepavidin. Signal was amplified using the Affymetrix protocol for antibody amplification. The raw image (.DAT) and intensity (.CEL) files were generated using MAS 5.0 software (http://www.affymetrix.com).

#### Microarray analysis

Chip quality was assessed using various parameters outputted by a combination of the following software packages: MAS 5.0 (http://www.affymetrix.com, BRB Array tools (http://linus.nci.nih.gov/BRB-ArrayTools.html, and Bioconductor version 1.2 (http://www.bioconductor.org). Twenty-one chips were hybridized and analyzed, but only 16 (approximately 75%) passed our quality control standards (scale factor ≤ 10, 3′-to-5′ ratio ≤ 25, R^2^ ≥ 0.97). Normalization and model-based expression values were calculated using the GeneChip Robust Multichip Analysis method (Wu et al. 2003), available as part of the Bioconductor package.

#### Statistical analysis

Time-dependent expression profiles for each gene were analyzed by regressing the normalized expression values using polynomial least squares regression. ANOVA was performed on the coefficients of regression to identify genes with significant time patterns (*p* < 0.05). The smooth curve fitting assumed that the expression trajectory for each gene followed a continuous time pattern. The class of fifth-degree polynomials was chosen for the fits, because it was the highest degree polynomial that did not interpolate the time point means. Analysis was performed in R 1.7.1 (http://www.r-project.org) using the Bioconductor suite of R packages. Source code for the analysis, including the curve fitting procedure, is available in Protocol S1.

#### GO analysis

GO analysis was performed using the 1 October 2003 build of the gene ontologies (http://www.geneontology.org) and the GO annotations for each probe set on the MGU74Av2 chip, provided by Affymetrix (http://www.affymetrix.com, downloaded 8 October 2003). The GO vocabulary structure was then instantiated as a directed acyclic graph and traversed to obtain hit counts for the genes in our lists that mapped at or below each node in the GO structure. To assess the significance of gene counts at each term, the annotations for the entire array were mapped to the GO structure, and counts for the whole array were obtained at each GO term. The significance of counts in particular categories was obtained via a sampling-without-replacement statistical model for the gene counts in each GO category.

The probability of a count of *k* genes to a GO node at some level of the GO hierarchy was modeled according to the hypergeometric probability law:



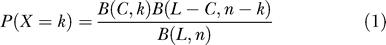



In the formula, *B(x,y)* is the binomial coefficient for *x* choose *y*. The value *C* is the total number of genes annotated to the GO node under consideration for the entire gene set. The value of *L* is the number of genes annotated to all nodes at the same level of the GO hierarchy, again considering the entire arrayed gene set. The value *n* is the number of genes annotated to terms at the same GO level for the gene list under consideration. The *p* value (one sided) for the node under consideration is obtained by summing probabilities as determined by the formula for all values of *X* from *k* to *n*. A web-based tool to perform this analysis on any gene list is available at http://franklin.imgen.bcm.tmc.edu/OntologyTraverser.

The list distance metric was determined from the estimated joint distribution of probe counts across the GO structure for each gene list. This joint distribution was estimated by obtaining the counts at each GO node at each level. Only those nodes with non-zero counts in at least one list were included in the calculations. Relative frequencies at each GO node at each GO level were obtained by normalizing to the total counts at each level for each list. Once the frequency distribution at each level was determined, a Kullback–Leibler-like distance metric was constructed. Briefly, the distance metric is a weighted average of Kullback–Leibler distances at each level of the GO. The formula for computing distance between a pair of lists is



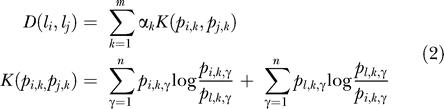



The weights α*_k_* were normalized to sum to one and were drawn from the Poisson mass function with a mean of four. Since the GO levels are ordered in terms of increasing specificity, the contribution of each level was weighted differently: positive weight was applied to the middle of the GO hierarchy (levels 3–8), and weights for levels lower than 3 and higher than 8 were set to 0. The indices *i* and *j* in the formula indicate the lists being compared. The index *k* indicates the level of the GO under consideration, and the index *γ* considers each GO node at the level *k.*

To compute the significance of our list dendrogram we determined the probability that we could arrive at the grouping pattern by chance. We determined the number of dendrograms with the “two group” pattern divided by the total number of labeled dendrograms. For our case, our “two group” dendrogram consisted of two subtrees with three and five arms, respectively. The total number of labeled dendrograms was the product of the number of labeled three-leaf dendrograms (three) and the number of labeled five-leaf dendrograms (105), which is 415. We divided this number by the total number of eight-leaf dendrograms (135,135) to attain the 0.003 probability. An R function for making this calculation is contained in the R script provided in Protocol S1.

#### Chromosome analysis

Gene hits per chromosome were counted for Q-sig and P-sig as well as the total MGU74Av2 chip. Number of hits in our signatures was centered to the expected frequency of the number of hits on the total chip using the following equation. The number of hits above/below expected equals *X* − *nP_i_,* where *X* equals the number of genes in list on chromosome *i, n* equals the total number of genes in list, and *P_i_* equals the frequency of chromosome *i* hits on total chip (which equals the number of genes on total chip on chromosome *i* divided by the number of genes on total chip with known chromosome position).

To determine the significance of enrichments and depletions of gene hits on each chromosome, we calculated a *Z*-score with the following equation.



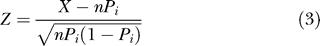



Chromosome enrichment or depletion between signatures was considered significant if the additive *Z*-score of Q-sig and P-sig was significant to 0.02 < α < 0.05.

## Supporting Information

Figure S1 Cell Cycle Analysis of HSCs Cell cycle analysis of bone marrow SP cells before (left) and 6 d post (right) 5FU treatment. Before treatment, approximately 2% of adult quiescent HSCs are in cycle; 6 d after 5FU treatment, approximately 22% of HSCs are in cycle.
(2.0 MB EPS).

Figure S2 FACS Isolation of HSCs (A) Representative flow cytometry plots of bone marrow enriched for Sca1^+^ cells at each time point. The indicated regions contain the SP cells. The table shows prevalence and purity from several isolations.
(B) Representative flow cytometry analysis of fetal liver enriched for c-Kit^+^ cells.
(2.9 MB EPS).

Figure S3 Homogeneity of SP Cells SP profile of adult HSCs and 5FU-HSCs 6 d post 5FU treatment. Arrows point to analysis in SP cells of Sca1 and lineage marker expression showing greater than 97% homogeneity for Sca1^+^ and Lineage^−^ expression.
For analysis of adult HSCs on day 0, the lineage markers used were Mac1, CD4, CD8, B220, GR1, and Ter119. For analysis of 5FU-HSCs on day 6, all of the above markers were used except for Mac1, because of its low level expression on HSCs after 5FU treatment.
(2.2 MB EPS).

Protocol S1 R Script for Constructing Gene Lists (8 KB TXT).

Table S1 Genes Up-Regulated in FL-HSCs (800 KB HTML).

Table S2 Genes in Proliferation Group (666 KB HTML).

Table S3 Genes That Change over 5FU Treatment Time Course (1.4 MB HTML).

Table S4 Genes in Quiescence Group (790 KB HTML).

Table S5 Genes Up-Regulated in Adult HSCs (933 KB HTML).

Table S6 Genes in P-Sig (336 KB HTML).

Table S7 Genes in Q-Sig (296 KB HTML).

Table S8 Genes in ST-HSC Signature (164 KB HTML).

Table S9 Genes in LT-HSC Signature (77 KB HTML).

Table S10 Genes in Common P-Sig (122 KB HTML).

Table S11 Genes in Common Q-Sig (59 KB HTML).

Table S12 Genes in TOM0 Group of Q-Sig (62 KB HTML).

Table S13 Genes in TOM1 Group of Q-Sig (70 KB HTML).

Table S14 Genes in TOM10 Group of Q-Sig (169 KB HTML).

Table S15 Genes in TOM30 Group of Q-Sig (45 KB HTML).

Table S16 Genes in TOM3 Group of P-Sig (52 KB HTML).

Table S17 Genes in TOM2 Group of P-Sig (18 KB HTML).

Table S18 Genes in TOM6 Group of P-Sig (280 KB HTML).

Table S19 Lists of GO Groups Enriched in Adult HSCs, FL-HSCs, and TOM Groups (2 KB HTML).

Table S20 Genes in Q-Sig, P-Sig, Common Q-Sig, and Common P-Sig in the GO Category “Cell Cycle” (7 KB HTML).

Table S21 Genes in Q-Sig, P-Sig, Common Q-Sig, and Common P-Sig in the GO Category “Cell–Cell Adhesion” (3 KB HTML).

Table S22 Genes in P-Sig in the GO Category “ATP-Synthesis-Coupled Electron Transport” (6 KB HTML).

Table S23 Genes in P-Sig in the GO Category “DNA Replication” (8 KB HTML).

Table S24 Genes in P-Sig in the GO Category “Cell Cycle Checkpoint” (6 KB HTML).

Table S25 Genes in P-Sig in the GO Category “Hydrogen Transport” (7 KB HTML).

Table S26 Genes in Q-Sig in the GO Category “Regulation of Cell Cycle” (7 KB HTML).

Table S27 Genes in Q-Sig in the GO Category “Defense Response” (9 KB HTML).

Table S28 Genes in Q-Sig in the GO Category “Protein Kinase Cascade” (7 KB HTML).

Table S29 Genes in Q-Sig in the GO Category “Cell–Cell Adhesion” (6 KB HTML).

Table S30 TOM1 Genes within GO Categories and the Fold Enrichment of Each Category (92 KB HTML).

Table S31 TOM1 Genes within GO Categories That Were Significantly Enriched (31 KB HTML).

Table S32 TOM10 Genes within GO Categories and the Fold Enrichment of Each Category (136 KB HTML).

Table S33 TOM10 Genes within GO Categories That Were Significantly Enriched (45 KB HTML).

Table S34 Genes Up-Regulated in Adult HSCs within GO Categories and the Fold Enrichment of Each Category (244 KB HTML).

Table S35 Genes Up-Regulated in Adult HSCs within GO Categories That Were Significantly Enriched (14 KB HTML).

Table S36 TOM0 Genes within GO Categories and the Fold Enrichment of Each Category (52 KB HTML).

Table S37 TOM0 Genes within GO Categories That Were Significantly Enriched (8 KB HTML).

Table S38 TOM30 Genes within GO Categories and the Fold Enrichment of Each Category (61 KB HTML).

Table S39 TOM30 Genes within GO Categories That Were Significantly Enriched (14 KB HTML).

Table S40 TOM3 Genes within GO Categories and the Fold Enrichment of Each Category (65 KB HTML).

Table S41 TOM3 Genes within GO Categories That Were Significantly Enriched (32 KB HTML).

Table S42 TOM6 Genes within GO Categories and the Fold Enrichment of Each Category (215 KB HTML).

Table S43 Lists of TOM6 Genes within GO Categories That Were Significantly Enriched (199 KB HTML).

Table S44 Lists of Genes Up-Regulated in FL-HSCs within GO Categories and the Fold Enrichment of Each Category (244 KB HTML).

Table S45 Genes Up-Regulated in FL-HSCs within GO Categories That Were Significantly Enriched (14 KB HTML).

Table S46 GeneChip Robust Multichip Analysis Normalized Data and Filtering Information (9.7 MB XLS).

Table S47 Goodness of Fit within Each TOM Group This table gives the 0.25, 0.5, and 0.75 quartile of the gene correlations (Pearson's) to their TOM group mean shown in Figure 2A and 2C.
(27 KB DOC).

### Accession Numbers

The LocusLink (http://www.ncbi.nlm.nih.gov/LocusLink/) accession numbers for the genes and gene products discussed in this paper are BTG1 (Locuslink 12226), Btg3/APRO4 (Locuslink 12228), CD48 (Locuslink 12506), c-fos (Locuslink 14281), c-maf (Locuslink 17134), cyclin G1 (Locuslink 12450), Endoglin (Locuslink 13805), GADD45β (Locuslink 17873), GATA-2 (Locuslink 14461), histone deacetylase 5 (Locuslink 15184), IGF1R (Locuslink 16001), melanoma antigen, family D, 1 (Locuslink 94275), p21 (Locuslink12575), receptor tyrosine kinase Tie1 (Locuslink 21846), serine proteinase inhibitor A-3 g (Locuslink 20715), SOCS3 (Locuslink 12702), STAT3 (Locuslink 20848), STAT6 (Locuslink 20852), Suppressor of cytokine signaling 3 (Locuslink 12702), TIMP3 (Locuslink 21859), Tob 1/APRO6 (Locuslink 22057), and α4-integrin (Locuslink 16401). The GEO (www.ncbi.nlm.nih.gov/geo) accession numbers for microarrays discussed in this paper are GSM26734-GSM26749.

## Abbreviations

5FU: 5-fluorouracil
5FU-HSC: 5-fluorouracil-activated hematopoietic stem cell
FL-HSC: fetal liver hematopoietic stem cell
GO: Gene Ontology
HSC: hematopoietic stem cell
IVT: in vitro transcription
LT-HSC: long-term hematopoietic stem cell
P-sig: Proliferation Signature
Q-sig: Quiescence Signature
QTL: quantitative trait loci
SP: side population
ST-HSC: short-term hematopoietic stem cell
TOM: time of maximum expression
WBM: whole bone marrow

## References

[pbio-0020301-Akashi1] Akashi K, He X, Chen J, Iwasaki H, Niu C (2003). Transcriptional accessibility for genes of multiple tissues and hematopoietic lineages is hierarchically controlled during early hematopoiesis. Blood.

[pbio-0020301-Ashburner1] Ashburner M, Ball CA, Blake JA, Botstein D, Butler H (2000). Gene ontology: Tool for the unification of biology. The Gene Ontology Consortium. Nat Genet.

[pbio-0020301-Bhunia1] Bhunia AK, Piontek K, Boletta A, Liu L, Qian F (2002). PKD1 induces p21(waf1) and regulation of the cell cycle via direct activation of the JAK-STAT signaling pathway in a process requiring PKD2. Cell.

[pbio-0020301-Bradford1] Bradford GB, Williams B, Rossi R, Bertoncello I (1997). Quiescence, cycling, and turnover in the primitive hematopoietic stem cell compartment. Exp Hematol.

[pbio-0020301-Calvi1] Calvi LM, Adams GB, Weibrecht KW, Weber JM, Olson DP (2003). Osteoblastic cells regulate the haematopoietic stem cell niche. Nature.

[pbio-0020301-Chavin1] Chavin KD, Qin L, Lin J, Woodward J, Baliga P (1994). Anti-CD48 (murine CD2 ligand) mAbs suppress cell mediated immunity in vivo. Int Immunol.

[pbio-0020301-Chen1] Chen CZ, Li M, de Graaf D, Monti S, Gottgens B (2002). Identification of endoglin as a functional marker that defines long-term repopulating hematopoietic stem cells. Proc Natl Acad Sci U S A.

[pbio-0020301-Cheng1] Cheng T, Rodrigues N, Shen H, Yang Y, Dombkowski D (2000). Hematopoietic stem cell quiescence maintained by p21cip1/waf1. Science.

[pbio-0020301-Cheshier1] Cheshier SH, Morrison SJ, Liao X, Weissman IL (1999). In vivo proliferation and cell cycle kinetics of long-term self-renewing hematopoietic stem cells. Proc Natl Acad Sci U S A.

[pbio-0020301-Craddock1] Craddock CF, Nakamoto B, Andrews RG, Priestley GV, Papayannopoulou T (1997). Antibodies to VLA4 integrin mobilize long-term repopulating cells and augment cytokine-induced mobilization in primates and mice. Blood.

[pbio-0020301-Dixon1] Dixon R, Rosendaal M (1981). Contrasts between the response of the mouse haemopoietic system to 5-fluorouracil and irradiation. Blood Cells.

[pbio-0020301-Fortunel1] Fortunel NO, Otu HH, Ng HH, Chen J, Mu X (2003). Comment on “‘Stemness’: Transcriptional profiling of embryonic and adult stem cells” and “A stem cell molecular signature.”. Science.

[pbio-0020301-Gallardo1] Gallardo TD, Hammer RE, Garry DJ (2003). RNA amplification and transcriptional profiling for analysis of stem cell populations. Genesis.

[pbio-0020301-Geiger1] Geiger H, True JM, de Haan G, Van Zant G (2001). Age- and stage-specific regulation patterns in the hematopoietic stem cell hierarchy. Blood.

[pbio-0020301-Gonzalez-Cabrero1] Gonzalez-Cabrero J, Wise CJ, Latchman Y, Freeman GJ, Sharpe AH (1999). CD48–deficient mice have a pronounced defect in CD4(+) T cell activation. Proc Natl Acad Sci U S A.

[pbio-0020301-Goodell1] Goodell MA, Brose K, Paradis G, Conner AS, Mulligan RC (1996). Isolation and functional properties of murine hematopoietic stem cells that are replicating in vivo. J Exp Med.

[pbio-0020301-Harrison1] Harrison DE, Lerner CP (1991). Most primitive hematopoietic stem cells are stimulated to cycle rapidly after treatment with 5-fluorouracil. Blood.

[pbio-0020301-Heissig1] Heissig B, Hattori K, Dias S, Friedrich M, Ferris B (2002). Recruitment of stem and progenitor cells from the bone marrow niche requires MMP-9 mediated release of kit-ligand. Cell.

[pbio-0020301-Huret1] Huret JL, Tanzer J, Henry-Amar M (1986). Aberrant breakpoints in chronic myelogenous leukaemia; oncogenes and fragile sites. Hum Genet.

[pbio-0020301-Irizarry1] Irizarry RA, Bolstad BM, Collin F, Cope LM, Hobbs B (2003). Summaries of Affymetrix GeneChip probe level data. Nucleic Acids Res.

[pbio-0020301-Ito1] Ito CY, Li CY, Bernstein A, Dick JE, Stanford WL (2003). Hematopoietic stem cell and progenitor defects in Sca-1/Ly-6A-null mice. Blood.

[pbio-0020301-Ivanova1] Ivanova NB, Dimos JT, Schaniel C, Hackney JA, Moore KA (2002). A stem cell molecular signature. Science.

[pbio-0020301-Johansson1] Johansson B, Fioretos T, Mitelman F (2002). Cytogenetic and molecular genetic evolution of chronic myeloid leukemia. Acta Haematol.

[pbio-0020301-Jordan1] Jordan CT, McKearn JP, Lemischka IR (1990). Cellular and developmental properties of fetal hematopoietic stem cells. Cell.

[pbio-0020301-Kato1] Kato K, Koyanagi M, Okada H, Takanashi T, Wong YW (1992). CD48 is a counter-receptor for mouse CD2 and is involved in T cell activation. J Exp Med.

[pbio-0020301-Kiger1] Kiger AA, Jones DL, Schulz C, Rogers MB, Fuller MT (2001). Stem cell self-renewal specified by JAK-STAT activation in response to a support cell cue. Science.

[pbio-0020301-Levy1] Levy DE, Lee CK (2002). What does Stat3 do?. J Clin Invest.

[pbio-0020301-Liu1] Liu Y, Jovanovic B, Pins M, Lee C, Bergan RC (2002). Over expression of endoglin in human prostate cancer suppresses cell detachment, migration and invasion. Oncogene.

[pbio-0020301-Morrison1] Morrison SJ, Weissman IL (1994). The long-term repopulating subset of hematopoietic stem cells is deterministic and isolatable by phenotype. Immunity.

[pbio-0020301-Morrison2] Morrison SJ, Hemmati HD, Wandycz AM, Weissman IL (1995). The purification and characterization of fetal liver hematopoietic stem cells. Proc Natl Acad Sci U S A.

[pbio-0020301-Phillips1] Phillips RL, Reinhart AJ, Van Zant G (1992). Genetic control of murine hematopoietic stem cell pool sizes and cycling kinetics. Proc Natl Acad Sci U S A.

[pbio-0020301-Pierelli1] Pierelli L, Bonanno G, Rutella S, Marone M, Scambia G (2001). CD105 (endoglin) expression on hematopoietic stem/progenitor cells. Leuk Lymphoma.

[pbio-0020301-Qi1] Qi JH, Ebrahem Q, Moore N, Murphy G, Claesson-Welsh L (2003). A novel function for tissue inhibitor of metalloproteinases-3 (TIMP3): Inhibition of angiogenesis by blockage of VEGF binding to VEGF receptor-2. Nat Med.

[pbio-0020301-Ramalho-Santos1] Ramalho-Santos M, Yoon S, Matsuzaki Y, Mulligan RC, Melton DA (2002). “Stemness”: Transcriptional profiling of embryonic and adult stem cells. Science.

[pbio-0020301-Randall1] Randall TD, Weissman IL (1997). Phenotypic and functional changes induced at the clonal level in hematopoietic stem cells after 5-fluorouracil treatment. Blood.

[pbio-0020301-Raz1] Raz R, Lee CK, Cannizzaro LA, d'Eustachio P, Levy DE (1999). Essential role of STAT3 for embryonic stem cell pluripotency. Proc Natl Acad Sci U S A.

[pbio-0020301-Scott1] Scott LM, Priestley GV, Papayannopoulou T (2003). Deletion of alpha4 integrins from adult hematopoietic cells reveals roles in homeostasis, regeneration, and homing. Mol Cell Biol.

[pbio-0020301-Sherr1] Sherr CJ (1996). Cancer cell cycles. Science.

[pbio-0020301-Soriano1] Soriano SF, Hernanz-Falcon P, Rodriguez-Frade JM, De Ana AM, Garzon R (2002). Functional inactivation of CXC chemokine receptor 4-mediated responses through SOCS3 up-regulation. J Exp Med.

[pbio-0020301-Szilvassy1] Szilvassy SJ, Cory S (1993). Phenotypic and functional characterization of competitive long-term repopulating hematopoietic stem cells enriched from 5-fluorouracil-treated murine marrow. Blood.

[pbio-0020301-Uchida1] Uchida N, Dykstra B, Lyons K, Leung F, Kristiansen M (2004). ABC transporter activities of murine hematopoietic stem cells vary according to their developmental and activation status. Blood.

[pbio-0020301-Van1] Van Zant G (1984). Studies of hematopoietic stem cells spared by 5-fluorouracil. J Exp Med.

[pbio-0020301-Wolf1] Wolf NS, Kone A, Priestley GV, Bartelmez SH (1993). In vivo and in vitro characterization of long-term repopulating primitive hematopoietic cells isolated by sequential Hoechst 33342-rhodamine 123 FACS selection. Exp Hematol.

[pbio-0020301-Wright1] Wright DE, Cheshier SH, Wagers AJ, Randall TD, Christensen JL (2001). Cyclophosphamide/granulocyte colony-stimulating factor causes selective mobilization of bone marrow hematopoietic stem cells into the blood after M phase of the cell cycle. Blood.

[pbio-0020301-Wu1] Wu Z, Irizarry RA, Gentleman R, Martinez Murillo F, Spencer F (2003). A model based background adjustment for oligonucleotide expression arrays. Johns Hopkins University, Department of Biostatistics Working Papers. Working Paper 1 Available: http://www.bepress.com/jhubiostat/paper1 via the Internet. http://www.bepress.com/jhubiostat/paper1.

[pbio-0020301-Zhang1] Zhang J, Niu C, Ye L, Huang H, He X (2003). Identification of the haematopoietic stem cell niche and control of the niche size. Nature.

