# Supplementary material for: Molecular Signatures of Proliferation and Quiescence in Hematopoietic Stem Cells
Source: PLoS Biol. 2004 Sep 28;2(10):e301. doi: 10.1371/journal.pbio.0020301 (PMC520599; doi:10.1371/journal.pbio.0020301)
Supplement: Table S1 — (800 KB HTML). [file pbio.0020301.st001.html]

|  |  | Up-regulated in FL-HSC |  |  |  |  |  |  |  |  |
| Probe Set ID | Gene Symbol | Gene name | Chromosome | Log2 Fold Change (FL-HSC vs Adult HSC)\* | Day of max (TOM) | p-value of ANOVA (time course) |  | | | |
| 102282\_g\_at | Tnfrsf7 | tumor necrosis factor receptor superfamily, member 7 | --- | 1.004 | 0 | 0.219 |  | | | |
| 103048\_at | Nmyc1 | neuroblastoma myc-related oncogene 1 | chr12 | 3.322 | 0 | 0.634 |  | | | |
| 103299\_at | AI132321 | expressed sequence AI132321 | chr12 | 1.067 | 0 | 0.079 |  | | | |
| 103515\_at | Shapy-pending | Ca2+-dependent endoplasmic reticulum nucleoside diphosphatase | chr11 | 1.938 | 0 | 0.558 |  | | | |
| 104559\_at | BC003940 | cDNA sequence BC003940 | chr11 | 2.084 | 0 | 0.34 |  | | | |
| 161914\_s\_at | Lsp1 | lymphocyte specific 1 | chr7 | 1.075 | 0 | 0.909 |  | | | |
| 162457\_f\_at | Hba-a1 | hemoglobin alpha, adult chain 1 | --- | 1.419 | 0 | 0.34 |  | | | |
| 92259\_at | Tbl3 | transducin (beta)-like 3 | chr17 | 1.357 | 0 | 0.492 |  | | | |
| 92934\_at | Zfp90 | zinc finger protein 90 | --- | 1.323 | 0 | 0.231 |  | | | |
| 93077\_s\_at | Ly6c | lymphocyte antigen 6 complex, locus C | chr15 | 1.151 | 0 | 0.244 |  | | | |
| 93500\_at | Alas1 | aminolevulinic acid synthase 1 | chr9 | 2.837 | 0 | 0.047 |  | | | |
| 93584\_at | Igh-6 | immunoglobulin heavy chain 6 (heavy chain of IgM) | chr12 | 1.572 | 0 | 0.006 |  | | | |
| 93758\_at | Incenp | inner centromere protein | chr19 | 2.166 | 0 | 0.082 |  | | | |
| 94398\_s\_at | Inpp5b | inositol polyphosphate-5-phosphatase B | chr4 | 1.124 | 0 | 0.218 |  | | | |
| 94399\_at | Inpp5b | inositol polyphosphate-5-phosphatase B | chr4 | 1.51 | 0 | 0.101 |  | | | |
| 95514\_at | 2610510H01Rik | RIKEN cDNA 2610510H01 gene | chr4 | 2.6 | 0 | 0.136 |  | | | |
| 95556\_at | Mrpl45 | mitochondrial ribosomal protein L45 | --- | 1.22 | 0 | 0.188 |  | | | |
| 96865\_at | Marcks | myristoylated alanine rich protein kinase C substrate | chr10 | 1.824 | 0 | 0.059 |  | | | |
| 97405\_at | Rps6ka1 | ribosomal protein S6 kinase polypeptide 1 | chr4 | 1.373 | 0 | 0.899 |  | | | |
| 98433\_at | Bid | BH3 interacting domain death agonist | chr6 | 2.124 | 0 | 0.153 |  | | | |
| 98623\_g\_at | Igf2 | insulin-like growth factor 2 | chr7 | 4.435 | 0 | 0.037 |  | | | |
| 100040\_at | Mrpl17 | mitochondrial ribosomal protein L17 | chr7 | 1.316 | 1 | 0.127 |  | | | |
| 100343\_f\_at | Tuba1 | tubulin, alpha 1 | chr15 | 1.066 | 1 | 0.397 |  | | | |
| 100540\_at | Lta4h | leukotriene A4 hydrolase | --- | 1.391 | 1 | 0.861 |  | | | |
| 100903\_at | Adprtl2 | ADP-ribosyltransferase (NAD+; poly(ADP-ribose) polymerase)-like 2 | chr14 | 1.283 | 1 | 0.318 |  | | | |
| 100910\_at | NoneAvailable | --- | chr2 | 1.379 | 1 | 0.319 |  | | | |
| 101393\_at | Anxa3 | annexin A3 | chr5 | 2.467 | 1 | 0.136 |  | | | |
| 101507\_at | Scnm1 | sodium channel modifier 1 | chr3 | 1.005 | 1 | 0.609 |  | | | |
| 101561\_at | Mt2 | metallothionein 2 | chr8 | 4.626 | 1 | 0.302 |  | | | |
| 102120\_f\_at | NoneAvailable | Mus musculus transcribed sequence with moderate similarity to protein pir:S47565 (H.sapiens) S47565 calcium-binding protein BDR-1 - human | --- | 1.013 | 1 | 0.569 |  | | | |
| 102627\_at | Igf2bp1 | insulin-like growth factor 2, binding protein 1 | chr11 | 6.314 | 1 | 0.012 |  | | | |
| 103070\_at | Ptpns1 | protein tyrosine phosphatase, non-receptor type substrate 1 | chr2 | 1.671 | 1 | 0.159 |  | | | |
| 103257\_at | C730036B01Rik | RIKEN cDNA C730036B01 gene | chr3 | 2.122 | 1 | 0.004 |  | | | |
| 103447\_at | C030006K11Rik | RIKEN cDNA C030006K11 gene | chr15 | 2.585 | 1 | 0.436 |  | | | |
| 103630\_at | Lars | leucyl-tRNA synthetase | chr18 | 1.115 | 1 | 0.424 |  | | | |
| 104288\_at | Cul4a | cullin 4A | chr8 | 1.075 | 1 | 0.771 |  | | | |
| 104331\_at | Smarcf1 | SWI/SNF related, matrix associated, actin dependent regulator of chromatin, subfamily f, member 1 | chr4 | 1.408 | 1 | 0.854 |  | | | |
| 104549\_at | Aqr | aquarius | chr2 | 1.015 | 1 | 0.095 |  | | | |
| 160150\_f\_at | Cnn3 | calponin 3, acidic | chr3 | 1.234 | 1 | 0.112 |  | | | |
| 160197\_at | 1110058B13Rik | RIKEN cDNA 1110058B13 gene | chr15 | 1.846 | 1 | 0.03 |  | | | |
| 160227\_s\_at | Bysl | bystin-like | chr17 | 1.211 | 1 | 0 |  | | | |
| 160282\_at | 4931406I20Rik | RIKEN cDNA 4931406I20 gene | chr4 | 1.032 | 1 | 0.241 |  | | | |
| 160337\_at | 1300017C10Rik | RIKEN cDNA 1300017C10 gene | chr8 | 1.306 | 1 | 0.247 |  | | | |
| 160386\_at | C130052I12Rik | RIKEN cDNA C130052I12 gene | chr13 | 2.018 | 1 | 0.328 |  | | | |
| 160395\_at | D11Ertd603e | DNA segment, Chr 11, ERATO Doi 603, expressed | chr11 | 1.698 | 1 | 0.067 |  | | | |
| 160583\_at | Xlkd1 | extra cellular link domain-containing 1 | chr7 | 1.037 | 1 | 0.235 |  | | | |
| 160666\_at | 2010200J04Rik | RIKEN cDNA 2010200J04 gene | chr10 | 1.27 | 1 | 0.533 |  | | | |
| 160970\_at | Odf2 | outer dense fiber of sperm tails 2 | chr2 | 1.149 | 1 | 0.39 |  | | | |
| 161968\_f\_at | Ccr5 | chemokine (C-C motif) receptor 5 | chr9 | 3.191 | 1 | 0.739 |  | | | |
| 92468\_at | Gbif-pending | globin inducing factor, fetal | chr9 | 2.692 | 1 | 0.09 |  | | | |
| 92607\_at | Mest | mesoderm specific transcript | --- | 1.189 | 1 | 0.268 |  | | | |
| 93356\_at | Mcm7 | minichromosome maintenance deficient 7 (S. cerevisiae) | chr16 | 1.7 | 1 | 0.458 |  | | | |
| 93683\_at | Rag1 | recombination activating gene 1 | --- | 2.941 | 1 | 0.457 |  | | | |
| 93859\_at | Mtif2 | mitochondrial translational initiation factor 2 | chr11 | 1.248 | 1 | 0.688 |  | | | |
| 93973\_at | Eif3s9 | eukaryotic translation initiation factor 3, subunit 9 (eta) | chr5 | 1.876 | 1 | 0.224 |  | | | |
| 94048\_at | Cdc34 | cell division cycle 34 homolog (S. cerevisiae) | chr10 | 1.025 | 1 | 0.206 |  | | | |
| 94073\_at | Polr2g | polymerase (RNA) II (DNA directed) polypeptide G | chr19 | 1.881 | 1 | 0.001 |  | | | |
| 94274\_at | 6720465F12Rik | RIKEN cDNA 6720465F12 gene | --- | 1.041 | 1 | 0.001 |  | | | |
| 94781\_at | Hba-a1 | hemoglobin alpha, adult chain 1 | chr11 | 6.743 | 1 | 0.336 |  | | | |
| 95914\_at | 6720461J16Rik | RIKEN cDNA 6720461J16 gene | chr2 | 1.582 | 1 | 0.206 |  | | | |
| 96124\_at | NoneAvailable | Mus musculus, Similar to KIAA1042 protein, clone IMAGE:4217060, mRNA | chr9 | 1.025 | 1 | 0.909 |  | | | |
| 96155\_at | Cdk5rap3 | CDK5 regulatory subunit associated protein 3 | chr11 | 1.562 | 1 | 0.221 |  | | | |
| 96604\_at | 1110032N12Rik | RIKEN cDNA 1110032N12 gene | chr11 | 1.191 | 1 | 0.754 |  | | | |
| 96864\_at | NoneAvailable | Mus musculus similar to 5OT-EST protein, mRNA (cDNA clone MGC:58411 IMAGE:6706680), complete cds | chr2 | 1.248 | 1 | 0.385 |  | | | |
| 96943\_at | Gps1 | G protein pathway suppressor 1 | chr11 | 1.289 | 1 | 0.11 |  | | | |
| 97462\_at | 3110006P09Rik | RIKEN cDNA 3110006P09 gene | --- | 1.641 | 1 | 0.348 |  | | | |
| 97667\_at | NoneAvailable | --- | --- | 1.391 | 1 | 0.452 |  | | | |
| 98061\_at | 1110001K21Rik | RIKEN cDNA 1110001K21 gene | chr16 | 1.343 | 1 | 0.155 |  | | | |
| 98106\_at | Timm44 | translocator of inner mitochondrial membrane 44 | chr8 | 1.12 | 1 | 0.106 |  | | | |
| 98901\_at | 1110008B24Rik | RIKEN cDNA 1110008B24 gene | chr4 | 1.065 | 1 | 0.057 |  | | | |
| 99101\_at | Eif3s7 | eukaryotic translation initiation factor 3, subunit 7 (zeta) | chr15 | 2.233 | 1 | 0.534 |  | | | |
| 99167\_at | 1110039H05Rik | RIKEN cDNA 1110039H05 gene | chr9 | 1.066 | 1 | 0.133 |  | | | |
| 100066\_at | Gart | phosphoribosylglycinamide formyltransferase | chr16 | 1.415 | 2 | 0.007 |  | | | |
| 100461\_at | Polr2j | polymerase (RNA) II (DNA directed) polypeptide J | chr5 | 1.36 | 2 | 0.076 |  | | | |
| 100955\_at | 2700084L22Rik | RIKEN cDNA 2700084L22 gene | chr1 | 1.283 | 2 | 0.082 |  | | | |
| 101074\_at | Ddost | dolichyl-di-phosphooligosaccharide-protein glycotransferase | chr4 | 1.134 | 2 | 0.054 |  | | | |
| 101867\_at | Gpam | glycerol-3-phosphate acyltransferase, mitochondrial | chr19 | 1.488 | 2 | 0.906 |  | | | |
| 101959\_r\_at | Tfdp1 | transcription factor Dp 1 | chr8 | 1.111 | 2 | 0.004 |  | | | |
| 102375\_at | Smyd5 | SET and MYND domain containing 5 | chr6 | 1.585 | 2 | 0.702 |  | | | |
| 103057\_at | Pold1 | polymerase (DNA directed), delta 1, catalytic subunit | chr7 | 3.176 | 2 | 0.034 |  | | | |
| 104303\_i\_at | 1500004O14Rik | RIKEN cDNA 1500004O14 gene | chr2 | 1.473 | 2 | 0.007 |  | | | |
| 104305\_at | Rarsl | arginyl-tRNA synthetase-like | chr4 | 2.295 | 2 | 0.005 |  | | | |
| 104716\_at | Rbp1 | retinol binding protein 1, cellular | --- | 1.474 | 2 | 0.065 |  | | | |
| 160341\_at | Jtv1-pending | JTV1 gene | chr5 | 2.15 | 2 | 0.033 |  | | | |
| 92496\_at | Vamp5 | vesicle-associated membrane protein 5 | --- | 1.191 | 2 | 0.088 |  | | | |
| 92610\_at | Rdbp | RD RNA-binding protein | chr17 | 1.118 | 2 | 0.842 |  | | | |
| 92794\_f\_at | Nme1 | expressed in non-metastatic cells 1, protein | chr11 | 2.156 | 2 | 0 |  | | | |
| 93019\_at | H2afx | H2A histone family, member X | chr9 | 1.291 | 2 | 0.366 |  | | | |
| 93397\_at | Ccr2 | chemokine (C-C) receptor 2 | chr9 | 2.455 | 2 | 0.801 |  | | | |
| 94219\_at | Psmb2 | proteasome (prosome, macropain) subunit, beta type 2 | chr4 | 1.362 | 2 | 0.155 |  | | | |
| 94450\_at | D13Wsu123e | DNA segment, Chr 13, Wayne State University 123, expressed | chr13 | 1.019 | 2 | 0.014 |  | | | |
| 94982\_f\_at | Nme3 | expressed in non-metastatic cells 3 | chr17 | 1.55 | 2 | 0.242 |  | | | |
| 95025\_at | D16H22S680E | DNA segment, Chr 16, human D22S680E, expressed | chr16 | 1.125 | 2 | 0.074 |  | | | |
| 95066\_at | Taldo1 | transaldolase 1 | chr7 | 1.157 | 2 | 0.129 |  | | | |
| 96626\_at | 2300002G02Rik | RIKEN cDNA 2300002G02 gene | --- | 1.029 | 2 | 0.712 |  | | | |
| 96765\_at | Peg3 | paternally expressed 3 | chr7 | 3.05 | 2 | 0.734 |  | | | |
| 96827\_at | Cad | carbamoyl-phosphate synthetase 2, aspartate transcarbamylase, and dihydroorotase | chr5 | 1.06 | 2 | 0.014 |  | | | |
| 97238\_at | Tacc3 | transforming, acidic coiled-coil containing protein 3 | chr5 | 1.439 | 2 | 0.833 |  | | | |
| 98573\_r\_at | Ranbp1 | RAN binding protein 1 | chr16 | 1.404 | 2 | 0.046 |  | | | |
| 99158\_at | Sh3d3 | SH3 domain protein 3 | chr19 | 1.284 | 2 | 0.048 |  | | | |
| 100062\_at | Mcm3 | minichromosome maintenance deficient 3 (S. cerevisiae) | chr1 | 1.792 | 3 | 0.005 |  | | | |
| 100440\_f\_at | Ank1 | ankyrin 1, erythroid | chr8 | 1.093 | 3 | 0.65 |  | | | |
| 100452\_at | Klf1 | Kruppel-like factor 1 (erythroid) | chr8 | 1.591 | 3 | 0.093 |  | | | |
| 100885\_at | Nek2 | NIMA (never in mitosis gene a)-related expressed kinase 2 | chr1 | 2.649 | 3 | 0.148 |  | | | |
| 100894\_at | Orc5l | origin recognition complex, subunit 5-like (S. cerevisiae) | chr5 | 1.174 | 3 | 0.205 |  | | | |
| 101003\_at | Sfrs3 | splicing factor, arginine/serine-rich 3 (SRp20) | chr17 | 1.314 | 3 | 0.219 |  | | | |
| 101019\_at | Ctsc | cathepsin C | chr7 | 1.035 | 3 | 0.185 |  | | | |
| 101372\_at | Trip13 | thyroid hormone receptor interactor 13 | chr13 | 1.578 | 3 | 0.014 |  | | | |
| 101920\_at | Pole2 | polymerase (DNA directed), epsilon 2 (p59 subunit) | --- | 1.432 | 3 | 0.025 |  | | | |
| 102047\_at | Nmt1 | N-myristoyltransferase 1 | chr11 | 1.098 | 3 | 0.008 |  | | | |
| 102103\_f\_at | NoneAvailable | --- | --- | 1.173 | 3 | 0 |  | | | |
| 102128\_f\_at | Mrps25 | mitochondrial ribosomal protein S25 | --- | 1.148 | 3 | 0.01 |  | | | |
| 102411\_at | Zfp239 | zinc finger protein 239 | --- | 1.962 | 3 | 0.104 |  | | | |
| 102632\_at | Calmbp1 | calmodulin binding protein 1 | chr1 | 1.663 | 3 | 0.253 |  | | | |
| 102879\_s\_at | Fcgr1 | Fc receptor, IgG, high affinity I | chr3 | 1 | 3 | 0.271 |  | | | |
| 102911\_at | Brca2 | breast cancer 2 | chr5 | 1.267 | 3 | 0.114 |  | | | |
| 102935\_at | Cdc25c | cell division cycle 25 homolog C (S. cerevisiae) | chr18 | 3.661 | 3 | 0.384 |  | | | |
| 102942\_at | 2810012G08Rik | RIKEN cDNA 2810012G08 gene | chr11 | 1.329 | 3 | 0.868 |  | | | |
| 102976\_at | Brca1 | breast cancer 1 | chr11 | 2.649 | 3 | 0.06 |  | | | |
| 103036\_at | G22p1 | thyroid autoantigen | chr15 | 2.055 | 3 | 0.365 |  | | | |
| 103064\_at | Chek1 | checkpoint kinase 1 homolog (S. pombe) | chr9 | 1.251 | 3 | 0.01 |  | | | |
| 103201\_at | Ttk | Ttk protein kinase | chr9 | 2.66 | 3 | 0.018 |  | | | |
| 103203\_f\_at | NoneAvailable | Mus musculus transcribed sequence with moderate similarity to protein ref:NP\_078956.1 (H.sapiens)  hypothetical protein FLJ23311 [Homo sapiens] | chr7 | 2.324 | 3 | 0.019 |  | | | |
| 103204\_r\_at | NoneAvailable | Mus musculus transcribed sequence with moderate similarity to protein ref:NP\_078956.1 (H.sapiens)  hypothetical protein FLJ23311 [Homo sapiens] | chr7 | 1.738 | 3 | 0.048 |  | | | |
| 103207\_at | Pola1 | polymerase (DNA directed), alpha 1 | chrX | 2.502 | 3 | 0.025 |  | | | |
| 103212\_at | BC006933 | cDNA sequence BC006933 | chr12 | 1.151 | 3 | 0.045 |  | | | |
| 103221\_at | 5031425D22Rik | RIKEN cDNA 5031425D22 gene | chr7 | 1.533 | 3 | 0.077 |  | | | |
| 103341\_at | Ctps | cytidine 5'-triphosphate synthase | chr4 | 1.417 | 3 | 0.121 |  | | | |
| 103385\_at | Tera-pending | teratocarcinoma expressed, serine rich | chr14 | 1.152 | 3 | 0.537 |  | | | |
| 103424\_at | 6330442E10Rik | RIKEN cDNA 6330442E10 gene | chr12 | 1.324 | 3 | 0.977 |  | | | |
| 103444\_at | E130315B21Rik | RIKEN cDNA E130315B21 gene | chr10 | 1.296 | 3 | 0.016 |  | | | |
| 103553\_at | 2410041F14Rik | RIKEN cDNA 2410041F14 gene | chr2 | 1.235 | 3 | 0.407 |  | | | |
| 103635\_at | 2610025M23Rik | RIKEN cDNA 2610025M23 gene | chr14 | 1.103 | 3 | 0.84 |  | | | |
| 103778\_at | NoneAvailable | Mus musculus transcribed sequences | --- | 1.259 | 3 | 0.273 |  | | | |
| 103821\_at | Cdc6 | cell division cycle 6 homolog (S. cerevisiae) | --- | 2.833 | 3 | 0.016 |  | | | |
| 104230\_at | Dclre1a | DNA cross-link repair 1A, PSO2 homolog (S. cerevisiae) | chr19 | 1.15 | 3 | 0.001 |  | | | |
| 104644\_at | Kif4 | kinesin family member 4 | chrX | 2.262 | 3 | 0.233 |  | | | |
| 160069\_at | Gmnn | geminin | chr13 | 2.598 | 3 | 0.009 |  | | | |
| 160496\_s\_at | Mcm3 | minichromosome maintenance deficient 3 (S. cerevisiae) | chr1 | 1.482 | 3 | 0.021 |  | | | |
| 160625\_f\_at | BC004636 | cDNA sequence BC004636 | --- | 2.931 | 3 | 0.223 |  | | | |
| 160667\_at | Evl | Ena-vasodilator stimulated phosphoprotein | chr12 | 1.348 | 3 | 0.366 |  | | | |
| 160755\_at | Kif2c | kinesin family member 2C | chr4 | 1.965 | 3 | 0.183 |  | | | |
| 161122\_f\_at | Ndufab1 | NADH dehydrogenase (ubiquinone) 1, alpha/beta subcomplex, 1 | --- | 1.68 | 3 | 0.003 |  | | | |
| 161292\_f\_at | NoneAvailable | Mus musculus transcribed sequences | chr7 | 1.403 | 3 | 0.006 |  | | | |
| 161808\_f\_at | Evl | Ena-vasodilator stimulated phosphoprotein | chr12 | 2.01 | 3 | 0.104 |  | | | |
| 161856\_f\_at | Kif20a | kinesin family member 20A | chr18 | 4.921 | 3 | 0.071 |  | | | |
| 92481\_at | Chek2 | CHK2 checkpoint homolog (S. pombe) | --- | 1.472 | 3 | 0.145 |  | | | |
| 92551\_at | Lig1 | ligase I, DNA, ATP-dependent | chr7 | 1.152 | 3 | 0.033 |  | | | |
| 92569\_f\_at | Nol5 | nucleolar protein 5 | chr1 | 1.083 | 3 | 0.108 |  | | | |
| 92593\_at | Osf2-pending | osteoblast specific factor 2 (fasciclin I-like) | chr3 | 1.316 | 3 | 0.006 |  | | | |
| 92632\_at | Calm2 | calmodulin 2 | chr7 | 1.468 | 3 | 0.188 |  | | | |
| 93028\_at | H19 | H19 fetal liver mRNA | chr7 | 4.926 | 3 | 0.186 |  | | | |
| 93119\_at | Cox5b | cytochrome c oxidase, subunit Vb | chr1 | 1.423 | 3 | 0.059 |  | | | |
| 93445\_at | Cd5l | CD5 antigen-like | --- | 1.122 | 3 | 0.013 |  | | | |
| 94024\_at | Ris2 | retroviral integration site 2 | chr8 | 1.667 | 3 | 0.134 |  | | | |
| 94228\_at | Xpo1 | exportin 1, CRM1 homolog (yeast) | chr11 | 1.337 | 3 | 0.001 |  | | | |
| 94281\_at | Cnot2 | CCR4-NOT transcription complex, subunit 2 | chr10 | 1.086 | 3 | 0.213 |  | | | |
| 94376\_s\_at | Mre11a | meiotic recombination 11 homolog A (S. cerevisiae) | chr9 | 2.127 | 3 | 0.035 |  | | | |
| 94784\_at | NoneAvailable | Mus musculus cDNA clone MGC:32192 IMAGE:5006129, complete cds | chr3 | 1.535 | 3 | 0.057 |  | | | |
| 94788\_f\_at | Tubb5 | tubulin, beta 5 | chr17 | 1.367 | 3 | 0.026 |  | | | |
| 94907\_f\_at | 1110001J03Rik | RIKEN cDNA 1110001J03 gene | chr6 | 1.71 | 3 | 0.003 |  | | | |
| 94952\_at | C330012H03Rik | RIKEN cDNA C330012H03 gene | chr16 | 1.807 | 3 | 0.082 |  | | | |
| 95063\_at | 2310021G01Rik | RIKEN cDNA 2310021G01 gene | chr2 | 2.4 | 3 | 0.041 |  | | | |
| 95084\_f\_at | Grhpr | glyoxylate reductase/hydroxypyruvate reductase | chr4 | 2.569 | 3 | 0.006 |  | | | |
| 95131\_f\_at | Ndufb2 | NADH dehydrogenase (ubiquinone) 1 beta subcomplex, 2 | chr2 | 1.121 | 3 | 0.003 |  | | | |
| 95292\_at | Itga4 | integrin alpha 4 | chr2 | 1.618 | 3 | 0.02 |  | | | |
| 95419\_at | H1f0 | H1 histone family, member 0 | chr15 | 1.552 | 3 | 0.357 |  | | | |
| 95456\_r\_at | Shfdg1 | split hand/foot deleted gene 1 | chr6 | 1.322 | 3 | 0.026 |  | | | |
| 95462\_at | Bzw2 | basic leucine zipper and W2 domains 2 | chr12 | 1.226 | 3 | 0.049 |  | | | |
| 95527\_at | Chaf1a | chromatin assembly factor 1, subunit A (p150) | chr17 | 1.001 | 3 | 0 |  | | | |
| 95549\_at | Prim2 | DNA primase, p58 subunit | chr1 | 1.442 | 3 | 0.242 |  | | | |
| 95612\_at | Rfc5 | replication factor C (activator 1) 5 | --- | 2.137 | 3 | 0.009 |  | | | |
| 95732\_at | 1110005L13Rik | RIKEN cDNA 1110005L13 gene | chr10 | 2.644 | 3 | 0.028 |  | | | |
| 95927\_f\_at | 2610201A13Rik | RIKEN cDNA 2610201A13 gene | chr11 | 1.536 | 3 | 0.02 |  | | | |
| 96010\_at | Kpna3 | karyopherin (importin) alpha 3 | chr14 | 1.32 | 3 | 0.111 |  | | | |
| 96026\_at | Ahcy | S-adenosylhomocysteine hydrolase | --- | 1.133 | 3 | 0.506 |  | | | |
| 96050\_at | Smarcb1 | SWI/SNF related, matrix associated, actin dependent regulator of chromatin, subfamily b, member 1 | chr10 | 1.737 | 3 | 0.102 |  | | | |
| 96092\_at | Hp | haptoglobin | chr8 | 2.669 | 3 | 0.478 |  | | | |
| 96168\_at | Kif23 | kinesin family member 23 | chr9 | 1.012 | 3 | 0.353 |  | | | |
| 96258\_at | Mgst3 | microsomal glutathione S-transferase 3 | --- | 1.139 | 3 | 0.303 |  | | | |
| 96625\_at | D630024B06Rik | RIKEN cDNA D630024B06 gene | chr14 | 1.795 | 3 | 0.01 |  | | | |
| 96686\_i\_at | 2010100O12Rik | RIKEN cDNA 2010100O12 gene | chr2 | 1.279 | 3 | 0.005 |  | | | |
| 96687\_f\_at | 2010100O12Rik | RIKEN cDNA 2010100O12 gene | chr2 | 1.093 | 3 | 0 |  | | | |
| 97095\_at | Bub1 | budding uninhibited by benzimidazoles 1 homolog (S. cerevisiae) | --- | 3.241 | 3 | 0.008 |  | | | |
| 97271\_at | 2600017H02Rik | RIKEN cDNA 2600017H02 gene | chr11 | 1.179 | 3 | 0.068 |  | | | |
| 97393\_at | Vrk1 | vaccinia related kinase 1 | chr12 | 1.381 | 3 | 0.035 |  | | | |
| 97411\_at | Ect2 | ect2 oncogene | chr3 | 2.597 | 3 | 0.019 |  | | | |
| 97445\_at | Ppid | peptidylprolyl isomerase D (cyclophilin D) | chr3 | 1.05 | 3 | 0.085 |  | | | |
| 97486\_at | U2af1 | U2 small nuclear ribonucleoprotein auxiliary factor (U2AF) 1 | chr17 | 1.144 | 3 | 0.257 |  | | | |
| 97868\_at | Dnaja3 | DnaJ (Hsp40) homolog, subfamily A, member 3 | chr16 | 1.04 | 3 | 0.858 |  | | | |
| 98111\_at | Hsp105 | heat shock protein 105 | chr5 | 1.007 | 3 | 0.967 |  | | | |
| 98400\_at | Solt | SoxLZ/Sox6 leucine zipper binding protein in testis | chr13 | 2.42 | 3 | 0.101 |  | | | |
| 98469\_at | Aurkb | aurora kinase B | --- | 1.496 | 3 | 0.378 |  | | | |
| 98550\_at | Set | SET translocation | chr1 | 1.359 | 3 | 0.045 |  | | | |
| 98618\_at | Dtymk | deoxythymidylate kinase | chr1 | 1.551 | 3 | 0.004 |  | | | |
| 98929\_at | 1110018B13Rik | RIKEN cDNA 1110018B13 gene | chr13 | 1.14 | 3 | 0.032 |  | | | |
| 98982\_at | Tmpo | thymopoietin | chr10 | 1.765 | 3 | 0.089 |  | | | |
| 98999\_at | Adsl | adenylosuccinate lyase | chr15 | 2.382 | 3 | 0.036 |  | | | |
| 99186\_at | Ccna2 | cyclin A2 | chr3 | 1.908 | 3 | 0.201 |  | | | |
| 99345\_at | 9630025B04Rik | RIKEN cDNA 9630025B04 gene | --- | 1.906 | 3 | 0.097 |  | | | |
| 99457\_at | Mki67 | antigen identified by monoclonal antibody Ki 67 | chr7 | 2.708 | 3 | 0.002 |  | | | |
| 99541\_at | Kif11 | kinesin family member 11 | --- | 2.6 | 3 | 0.273 |  | | | |
| 99564\_at | Np95 | nuclear protein 95 | chr17 | 1.058 | 3 | 0.073 |  | | | |
| 99581\_at | Hint | histidine triad nucleotide binding protein | --- | 1.503 | 3 | 0.038 |  | | | |
| 99662\_at | Pcnt2 | pericentrin 2 | chr11 | 1.386 | 3 | 0.116 |  | | | |
| 99663\_g\_at | Pcnt2 | pericentrin 2 | chr11 | 2.458 | 3 | 0.118 |  | | | |
| 99917\_at | Ezh2 | enhancer of zeste homolog 2 (Drosophila) | chr6 | 2.33 | 3 | 0.147 |  | | | |
| 100033\_at | Msh2 | mutS homolog 2 (E. coli) | chr17 | 2.961 | 6 | 0.073 |  | | | |
| 100039\_at | Tmem4 | transmembrane protein 4 | chr10 | 1.81 | 6 | 0.003 |  | | | |
| 100057\_at | 2510027N19Rik | RIKEN cDNA 2510027N19 gene | chr7 | 3.001 | 6 | 0 |  | | | |
| 100059\_at | Cyba | cytochrome b-245, alpha polypeptide | chr8 | 1.77 | 6 | 0.038 |  | | | |
| 100073\_at | 2510005D08Rik | RIKEN cDNA 2510005D08 gene | chr14 | 2.121 | 6 | 0.012 |  | | | |
| 100079\_at | Ndufb9 | NADH dehydrogenase (ubiquinone) 1 beta subcomplex, 9 | chr15 | 1.058 | 6 | 0.013 |  | | | |
| 100116\_at | 2810417H13Rik | RIKEN cDNA 2810417H13 gene | chr1 | 2.111 | 6 | 0.026 |  | | | |
| 100128\_at | Cdc2a | cell division cycle 2 homolog A (S. pombe) | --- | 4.133 | 6 | 0.002 |  | | | |
| 100156\_at | Mcm5 | minichromosome maintenance deficient 5, cell division cycle 46 (S. cerevisiae) | chr8 | 5.137 | 6 | 0.012 |  | | | |
| 100331\_g\_at | Prdx2 | peroxiredoxin 2 | chr1 | 1.095 | 6 | 0.001 |  | | | |
| 100404\_at | TgN737Rpw | transgene insert site 737, insertional mutation, polycystic kidney disease | --- | 1.648 | 6 | 0.143 |  | | | |
| 100459\_at | Rad50 | RAD50 homolog (S. cerevisiae) | --- | 2.337 | 6 | 0.009 |  | | | |
| 100467\_at | Lyl1 | lymphoblastomic leukemia | chr8 | 2.596 | 6 | 0.549 |  | | | |
| 100512\_at | Uchl5 | ubiquitin carboxyl-terminal esterase L5 | chr1 | 1.055 | 6 | 0.006 |  | | | |
| 100527\_at | D11Ertd99e | DNA segment, Chr 11, ERATO Doi 99, expressed | chr11 | 2.559 | 6 | 0.038 |  | | | |
| 100543\_s\_at | Brd7 | bromodomain containing 7 | chr8 | 1.094 | 6 | 0.003 |  | | | |
| 100553\_at | Trim27 | tripartite motif protein 27 | chr13 | 1.095 | 6 | 0.823 |  | | | |
| 100568\_at | Abce1 | ATP-binding cassette, sub-family E (OABP), member 1 | --- | 1.485 | 6 | 0.011 |  | | | |
| 100575\_at | Ard1 | N-acetyltransferase ARD1 homolog (S. cerevisiae) | chrX | 1.841 | 6 | 0.337 |  | | | |
| 100576\_at | Pafah1b3 | platelet-activating factor acetylhydrolase, isoform 1b, alpha1 subunit | --- | 1.257 | 6 | 0.004 |  | | | |
| 100577\_at | Snrpd1 | small nuclear ribonucleoprotein D1 | chr18 | 1.295 | 6 | 0 |  | | | |
| 100579\_s\_at | Clta | clathrin, light polypeptide (Lca) | chr4 | 1.455 | 6 | 0.077 |  | | | |
| 100592\_at | Ghitm | growth hormone inducible transmembrane protein | chr14 | 1.179 | 6 | 0.024 |  | | | |
| 100612\_at | Rrm1 | ribonucleotide reductase M1 | --- | 1.929 | 6 | 0.045 |  | | | |
| 100618\_f\_at | Slc25a5 | solute carrier family 25 (mitochondrial carrier; adenine nucleotide translocator), member 5 | --- | 1.078 | 6 | 0.012 |  | | | |
| 100628\_at | Ndufc1 | NADH dehydrogenase (ubiquinone) 1, subcomplex unknown, 1 | --- | 1.336 | 6 | 0.006 |  | | | |
| 100733\_at | Psma2 | proteasome (prosome, macropain) subunit, alpha type 2 | chr13 | 1.64 | 6 | 0.006 |  | | | |
| 100886\_f\_at | Mrpl45 | mitochondrial ribosomal protein L45 | chr11 | 1.086 | 6 | 0.178 |  | | | |
| 100892\_at | Ndufaf1 | NADH dehydrogenase (ubiquinone) 1 alpha subcomplex, assembly factor 1 | chr2 | 1.231 | 6 | 0.392 |  | | | |
| 100917\_at | NoneAvailable | --- | chr18 | 1.316 | 6 | 0.037 |  | | | |
| 100952\_at | Stim1 | stromal interaction molecule 1 | chr7 | 1.732 | 6 | 0.242 |  | | | |
| 101017\_at | Cdk4 | cyclin-dependent kinase 4 | --- | 1.288 | 6 | 0.191 |  | | | |
| 101044\_at | Alad | aminolevulinate, delta-, dehydratase | chr4 | 1.12 | 6 | 0.488 |  | | | |
| 101045\_at | Hadh2 | hydroxyacyl-Coenzyme A dehydrogenase type II | chrX | 1.101 | 6 | 0.087 |  | | | |
| 101061\_at | Ssr2 | signal sequence receptor, beta | chr3 | 1.296 | 6 | 0.034 |  | | | |
| 101085\_at | Mrps24 | mitochondrial ribosomal protein S24 | chr11 | 1.805 | 6 | 0.065 |  | | | |
| 101096\_s\_at | Hs1bp1 | HS1 binding protein | chr2 | 1.123 | 6 | 0.018 |  | | | |
| 101105\_at | Banf1 | barrier to autointegration factor 1 | chr19 | 2.053 | 6 | 0.002 |  | | | |
| 101214\_f\_at | Gapd | glyceraldehyde-3-phosphate dehydrogenase | chr1 | 1.313 | 6 | 0.055 |  | | | |
| 101254\_at | Ran | RAN, member RAS oncogene family | chr2 | 1.149 | 6 | 0.002 |  | | | |
| 101350\_g\_at | NoneAvailable | --- | chr5 | 3.496 | 6 | 0.223 |  | | | |
| 101407\_at | Frda | Friedreich ataxia | chr19 | 1.288 | 6 | 0.027 |  | | | |
| 101421\_at | Rnf5 | ring finger protein 5 | chr17 | 1.067 | 6 | 0.044 |  | | | |
| 101444\_at | Gt(ROSA)26asSor | gene trap ROSA 26 antisense, Philippe Soriano | chr6 | 2.567 | 6 | 0.757 |  | | | |
| 101486\_at | Psmb10 | proteasome (prosome, macropain) subunit, beta type 10 | chr8 | 1.623 | 6 | 0.012 |  | | | |
| 101506\_at | Snrpa1 | small nuclear ribonucleoprotein polypeptide A' | chr7 | 1.197 | 6 | 0.003 |  | | | |
| 101521\_at | Birc5 | baculoviral IAP repeat-containing 5 | chr11 | 2.475 | 6 | 0.01 |  | | | |
| 101540\_at | Tdg | thymine DNA glycosylase | chr10 | 1.209 | 6 | 0.813 |  | | | |
| 101543\_f\_at | Tuba6 | tubulin, alpha 6 | chr15 | 1.399 | 6 | 0.189 |  | | | |
| 101558\_s\_at | Psmb5 | proteasome (prosome, macropain) subunit, beta type 5 | chr14 | 1.051 | 6 | 0.018 |  | | | |
| 101562\_at | Hsp70-4 | heat shock protein 4 | chr2 | 2.149 | 6 | 0.047 |  | | | |
| 101580\_at | Cox7b | cytochrome c oxidase subunit VIIb | chr1 | 1.791 | 6 | 0 |  | | | |
| 101753\_s\_at | Lzp-s | P lysozyme structural | --- | 1.567 | 6 | 0.299 |  | | | |
| 101869\_s\_at | NoneAvailable | --- | chr7 | 4.172 | 6 | 0.055 |  | | | |
| 101890\_f\_at | Dnajc2 | DnaJ (Hsp40) homolog, subfamily C, member 2 | chr5 | 1.708 | 6 | 0.027 |  | | | |
| 101944\_at | Lypla1 | lysophospholipase 1 | --- | 1.316 | 6 | 0.107 |  | | | |
| 101961\_at | Bub3 | budding uninhibited by benzimidazoles 3 homolog (S. cerevisiae) | chr7 | 1.693 | 6 | 0.113 |  | | | |
| 101964\_at | Tkt | transketolase | chr14 | 1.087 | 6 | 0.001 |  | | | |
| 101989\_at | Uqcrc1 | ubiquinol-cytochrome c reductase core protein 1 | chr9 | 1.915 | 6 | 0.05 |  | | | |
| 101992\_at | Psmb6 | proteasome (prosome, macropain) subunit, beta type 6 | chr11 | 1.47 | 6 | 0.016 |  | | | |
| 102001\_at | Rrm2 | ribonucleotide reductase M2 | chr12 | 1.74 | 6 | 0.02 |  | | | |
| 102019\_at | Mrpl13 | mitochondrial ribosomal protein L13 | chr15 | 1.841 | 6 | 0.001 |  | | | |
| 102022\_at | 1110007A04Rik | RIKEN cDNA 1110007A04 gene | --- | 1.16 | 6 | 0.009 |  | | | |
| 102039\_at | Gtf2h4 | general transcription factor II H, polypeptide 4 | chr17 | 2.601 | 6 | 0.058 |  | | | |
| 102193\_at | Sah | SA rat hypertension-associated homolog | chr7 | 1.462 | 6 | 0.223 |  | | | |
| 102409\_at | Lsm8 | LSM8 homolog, U6 small nuclear RNA associated (S. cerevisiae) | chr6 | 1.406 | 6 | 0.001 |  | | | |
| 102412\_at | AW541137 | expressed sequence AW541137 | chr10 | 2.049 | 6 | 0.032 |  | | | |
| 102631\_at | Blm | Bloom syndrome homolog (human) | chr7 | 1.408 | 6 | 0.003 |  | | | |
| 102821\_s\_at | Rasl2-9 | RAS-like, family 2, locus 9 | chr2 | 1.55 | 6 | 0 |  | | | |
| 102853\_at | Cspg6 | chondroitin sulfate proteoglycan 6 | chr19 | 1.265 | 6 | 0.037 |  | | | |
| 102934\_s\_at | Cdc25c | cell division cycle 25 homolog C (S. cerevisiae) | chr18 | 4.127 | 6 | 0.201 |  | | | |
| 102944\_at | C87777 | expressed sequence C87777 | chr11 | 2.448 | 6 | 0.841 |  | | | |
| 103089\_at | Cd48 | CD48 antigen | chr1 | 7.012 | 6 | 0 |  | | | |
| 103334\_at | Crcp | calcitonin gene-related peptide-receptor component protein | chr5 | 1.088 | 6 | 0.015 |  | | | |
| 103335\_at | Lgals9 | lectin, galactose binding, soluble 9 | chr11 | 2.142 | 6 | 0.019 |  | | | |
| 103352\_at | Dpagt1 | dolichyl-phosphate (UDP-N-acetylglucosamine) acetylglucosaminephosphotransferase 1 (GlcNAc-1-P transferase) | chr9 | 1.847 | 6 | 0.054 |  | | | |
| 103418\_at | Rfc4 | replication factor C (activator 1) 4 | chr16 | 2.864 | 6 | 0.041 |  | | | |
| 103442\_at | BC003479 | cDNA sequence BC003479 | chr11 | 1.965 | 6 | 0.007 |  | | | |
| 103468\_at | Mns1 | meiosis-specific nuclear structural protein 1 | chr9 | 3.961 | 6 | 0.026 |  | | | |
| 103481\_at | 6720457D02Rik | RIKEN cDNA 6720457D02 gene | --- | 2.592 | 6 | 0.827 |  | | | |
| 103534\_at | NoneAvailable | --- | chr7 | 2.111 | 6 | 0.006 |  | | | |
| 103565\_at | 1810009A15Rik | RIKEN cDNA 1810009A15 gene | chr19 | 2.87 | 6 | 0.07 |  | | | |
| 103571\_at | Lst1 | leukocyte specific transcript 1 | --- | 2.254 | 6 | 0.536 |  | | | |
| 103579\_at | Rac2 | RAS-related C3 botulinum substrate 2 | chr15 | 1.62 | 6 | 0.336 |  | | | |
| 103592\_at | Map2k5 | mitogen activated protein kinase kinase 5 | chr9 | 1.154 | 6 | 0.528 |  | | | |
| 103619\_at | 1810044O22Rik | RIKEN cDNA 1810044O22 gene | chr8 | 1.634 | 6 | 0.009 |  | | | |
| 103620\_s\_at | Smn | survival motor neuron | chr13 | 1.002 | 6 | 0.477 |  | | | |
| 103683\_at | Dhodh | dihydroorotate dehydrogenase | chr8 | 1.175 | 6 | 0.041 |  | | | |
| 103790\_at | Alg3 | asparagine-linked glycosylation 3 homolog (yeast, alpha-1,3-mannosyltransferase) | chr16 | 1.699 | 6 | 0.062 |  | | | |
| 103879\_at | BC024806 | cDNA sequence BC024806 | chr9 | 1.577 | 6 | 0.609 |  | | | |
| 103881\_at | 1110013G13Rik | RIKEN cDNA 1110013G13 gene | chr3 | 1.342 | 6 | 0.006 |  | | | |
| 103885\_at | 1500019O16Rik | RIKEN cDNA 1500019O16 gene | chr7 | 5.88 | 6 | 0.011 |  | | | |
| 103911\_at | AI463102 | EST AI463102 | --- | 2.426 | 6 | 0.076 |  | | | |
| 104042\_at | Slc35b1 | solute carrier family 35, member B1 | chr17 | 1.214 | 6 | 0.037 |  | | | |
| 104044\_at | 1300006N24Rik | RIKEN cDNA 1300006N24 gene | chr9 | 1.315 | 6 | 0.044 |  | | | |
| 104057\_at | Grpel1 | GrpE-like 1, mitochondrial | chr5 | 1.548 | 6 | 0.026 |  | | | |
| 104077\_at | 1110049G11Rik | RIKEN cDNA 1110049G11 gene | chrX | 1.238 | 6 | 0.01 |  | | | |
| 104078\_g\_at | 1110049G11Rik | RIKEN cDNA 1110049G11 gene | chrX | 3.329 | 6 | 0.001 |  | | | |
| 104080\_at | Pdap1 | PDGFA associated protein 1 | chr5 | 2.918 | 6 | 0.01 |  | | | |
| 104097\_at | Bub1 | budding uninhibited by benzimidazoles 1 homolog (S. cerevisiae) | chr2 | 3.068 | 6 | 0.067 |  | | | |
| 104102\_at | Prss25 | protease, serine, 25 | chr6 | 1.763 | 6 | 0.805 |  | | | |
| 104120\_at | 5330431N19Rik | RIKEN cDNA 5330431N19 gene | chr19 | 1.196 | 6 | 0.13 |  | | | |
| 104132\_at | Noc4 | neighbor of Cox4 | chr8 | 1.779 | 6 | 0.119 |  | | | |
| 104145\_at | Tcof1 | Treacher Collins Franceschetti syndrome 1, homolog | chr18 | 1.073 | 6 | 0.043 |  | | | |
| 104147\_at | Nans | N-acetylneuraminic acid synthase (sialic acid synthase) | chr4 | 1.196 | 6 | 0.022 |  | | | |
| 104234\_at | Mrps25 | mitochondrial ribosomal protein S25 | chr6 | 1.243 | 6 | 0.029 |  | | | |
| 104237\_at | 2700061N24Rik | RIKEN cDNA 2700061N24 gene | chr13 | 1.881 | 6 | 0.016 |  | | | |
| 104259\_at | Cbx5 | chromobox homolog 5 (Drosophila HP1a) | chr15 | 1.163 | 6 | 0.082 |  | | | |
| 104279\_at | 1810060D16Rik | RIKEN cDNA 1810060D16 gene | chr15 | 2.539 | 6 | 0.087 |  | | | |
| 104297\_at | Ipo11 | importin 11 | chr13 | 1.447 | 6 | 0.02 |  | | | |
| 104301\_at | 2410018G20Rik | RIKEN cDNA 2410018G20 gene | chr16 | 1.631 | 6 | 0.002 |  | | | |
| 104306\_at | Dpp3 | dipeptidylpeptidase 3 | chr19 | 2.33 | 6 | 0.519 |  | | | |
| 104310\_at | 0710001K01Rik | RIKEN cDNA 0710001K01 gene | chr11 | 1.558 | 6 | 0.094 |  | | | |
| 104312\_at | 1110013B16Rik | RIKEN cDNA 1110013B16 gene | chr9 | 2.135 | 6 | 0.169 |  | | | |
| 104322\_at | Ckap2 | cytoskeleton associated protein 2 | chr8 | 1.598 | 6 | 0.003 |  | | | |
| 104330\_g\_at | Smarcf1 | SWI/SNF related, matrix associated, actin dependent regulator of chromatin, subfamily f, member 1 | chr4 | 1.362 | 6 | 0.405 |  | | | |
| 104356\_at | 4921516M08Rik | RIKEN cDNA 4921516M08 gene | --- | 1.385 | 6 | 0 |  | | | |
| 104390\_at | Anp32a | acidic (leucine-rich) nuclear phosphoprotein 32 family, member A | --- | 1.176 | 6 | 0.045 |  | | | |
| 104423\_at | 2810047L02Rik | RIKEN cDNA 2810047L02 gene | chr1 | 3.018 | 6 | 0.009 |  | | | |
| 104476\_at | Rbl1 | retinoblastoma-like 1 (p107) | chr2 | 1.983 | 6 | 0.011 |  | | | |
| 104541\_at | Prtn3 | proteinase 3 | chr10 | 1.916 | 6 | 0.042 |  | | | |
| 104567\_at | Mrpl46 | mitochondrial ribosomal protein L46 | chr7 | 1.995 | 6 | 0.031 |  | | | |
| 104573\_at | 1110025L05Rik | RIKEN cDNA 1110025L05 gene | chr7 | 1.475 | 6 | 0.191 |  | | | |
| 104583\_at | Zdhhc6 | zinc finger, DHHC domain containing 6 | chr18 | 1.361 | 6 | 0.239 |  | | | |
| 104693\_at | 2600001M11Rik | RIKEN cDNA 2600001M11 gene | chr2 | 1.999 | 6 | 0.161 |  | | | |
| 104738\_at | Zrf2 | zuotin related factor 2 | chr6 | 1.861 | 6 | 0.008 |  | | | |
| 104752\_at | Mmrp19-pending | monocyte macrophage 19 | chr2 | 1.082 | 6 | 0.071 |  | | | |
| 104760\_at | Ifrd2 | interferon-related developmental regulator 2 | chr9 | 1.009 | 6 | 0.689 |  | | | |
| 104762\_r\_at | 1500015J03Rik | RIKEN cDNA 1500015J03 gene | chr2 | 1.459 | 6 | 0.042 |  | | | |
| 104766\_at | Nola1 | nucleolar protein family A, member 1 (H/ACA small nucleolar RNPs) | --- | 1.457 | 6 | 0.002 |  | | | |
| 104767\_f\_at | Mrps18a | mitochondrial ribosomal protein S18A | chr17 | 1.768 | 6 | 0.118 |  | | | |
| 160071\_at | Rnasep2-pending | ribonuclease P2 | chr19 | 1.1 | 6 | 0.051 |  | | | |
| 160076\_at | Mtx2 | metaxin 2 | chr2 | 1.76 | 6 | 0.035 |  | | | |
| 160078\_at | Ppp1r14b | protein phosphatase 1, regulatory (inhibitor) subunit 14B | chr5 | 1.274 | 6 | 0.155 |  | | | |
| 160129\_at | Eef1d | eukaryotic translation elongation factor 1 delta (guanine nucleotide exchange protein) | chr15 | 1.637 | 6 | 0.097 |  | | | |
| 160135\_at | D16Ertd502e | DNA segment, Chr 16, ERATO Doi 502, expressed | chr16 | 1.284 | 6 | 0.007 |  | | | |
| 160152\_at | Psmc1 | protease (prosome, macropain) 26S subunit, ATPase 1 | chr16 | 1.442 | 6 | 0.02 |  | | | |
| 160156\_at | 0910001A06Rik | RIKEN cDNA 0910001A06 gene | chr15 | 1.21 | 6 | 0.688 |  | | | |
| 160159\_at | Ccnb1 | cyclin B1 | chr13 | 4.698 | 6 | 0.077 |  | | | |
| 160166\_r\_at | 2810409H07Rik | RIKEN cDNA 2810409H07 gene | chr2 | 2.421 | 6 | 0.09 |  | | | |
| 160176\_at | Hirip5 | histone cell cycle regulation defective interacting protein 5 | chr15 | 1.669 | 6 | 0 |  | | | |
| 160192\_at | Rbmxrt | RNA binding motif protein, X chromosome retrogene | chr8 | 2.14 | 6 | 0.132 |  | | | |
| 160211\_at | D17Wsu94e | DNA segment, Chr 17, Wayne State University 94, expressed | chr17 | 1.006 | 6 | 0.131 |  | | | |
| 160226\_at | Gfm | G elongation factor | chr3 | 1.221 | 6 | 0.083 |  | | | |
| 160230\_at | NoneAvailable | Mus musculus cDNA clone MGC:58837 IMAGE:6773943, complete cds | --- | 2.056 | 6 | 0.072 |  | | | |
| 160247\_at | Ube2v2 | ubiquitin-conjugating enzyme E2 variant 2 | chr15 | 1.044 | 6 | 0.003 |  | | | |
| 160270\_at | Lman1 | lectin, mannose-binding, 1 | chr18 | 1.146 | 6 | 0.148 |  | | | |
| 160293\_at | 2700038L12Rik | RIKEN cDNA 2700038L12 gene | chr9 | 1.194 | 6 | 0.048 |  | | | |
| 160297\_at | MGC36453 | hypothetical protein LOC381045 | chr14 | 2.932 | 6 | 0.003 |  | | | |
| 160299\_at | Rwdd1 | RWD domain containing 1 | chr10 | 1.08 | 6 | 0.034 |  | | | |
| 160310\_at | D19Bwg1357e | DNA segment, Chr 19, Brigham & Women's Genetics 1357 expressed | chr19 | 1.3 | 6 | 0.281 |  | | | |
| 160324\_at | Rpa3 | replication protein A3 | chr1 | 1.959 | 6 | 0.009 |  | | | |
| 160345\_at | Mrpl34 | mitochondrial ribosomal protein L34 | chr8 | 2.836 | 6 | 0.064 |  | | | |
| 160402\_at | Tceb2 | transcription elongation factor B (SIII), polypeptide 2 (18 kDa, elongin B) | --- | 1.288 | 6 | 0.263 |  | | | |
| 160422\_at | Ruvbl2 | RuvB-like protein 2 | chr7 | 2.221 | 6 | 0.062 |  | | | |
| 160424\_f\_at | Fdps | farnesyl diphosphate synthetase | chr10 | 1.737 | 6 | 0.136 |  | | | |
| 160431\_at | Mrpl12 | mitochondrial ribosomal protein L12 | chr11 | 2.077 | 6 | 0.009 |  | | | |
| 160432\_at | AW553050 | expressed sequence AW553050 | chr7 | 1.407 | 6 | 0.25 |  | | | |
| 160473\_at | Nme4 | expressed in non-metastatic cells 4, protein | chr17 | 3.929 | 6 | 0.278 |  | | | |
| 160501\_at | Kif20a | kinesin family member 20A | chr18 | 4.762 | 6 | 0.141 |  | | | |
| 160503\_at | Fbl | fibrillarin | chr17 | 1.886 | 6 | 0 |  | | | |
| 160538\_at | Cdk4 | cyclin-dependent kinase 4 | chr10 | 2.296 | 6 | 0.032 |  | | | |
| 160555\_r\_at | Snrpb | small nuclear ribonucleoprotein B | chr2 | 4.973 | 6 | 0.163 |  | | | |
| 160557\_at | Tssc4 | tumor-suppressing subchromosomal transferable fragment 4 | chr7 | 1.091 | 6 | 0.224 |  | | | |
| 160562\_at | Cct7 | chaperonin subunit 7 (eta) | chr6 | 1.527 | 6 | 0.138 |  | | | |
| 160568\_at | Eno1 | enolase 1, alpha non-neuron | --- | 2.405 | 6 | 0.102 |  | | | |
| 160569\_at | 2310008M10Rik | RIKEN cDNA 2310008M10 gene | chr3 | 1.341 | 6 | 0.006 |  | | | |
| 160585\_at | 2810470K21Rik | RIKEN cDNA 2810470K21 gene | --- | 2.814 | 6 | 0.011 |  | | | |
| 160648\_at | Fignl1 | fidgetin-like 1 | chr11 | 2.958 | 6 | 0.066 |  | | | |
| 160723\_at | 1500001M20Rik | RIKEN cDNA 1500001M20 gene | chr6 | 1.3 | 6 | 0.044 |  | | | |
| 160759\_at | Rfc2 | replication factor C (activator 1) 2 | --- | 1.312 | 6 | 0.077 |  | | | |
| 160811\_at | MGC56855 | hypothetical protein MGC56855 | chr5 | 1.032 | 6 | 0.948 |  | | | |
| 160812\_at | Gga2 | golgi associated, gamma adaptin ear containing, ARF binding protein 2 | chr7 | 1.278 | 6 | 0.165 |  | | | |
| 160869\_at | Sirt3 | sirtuin 3 (silent mating type information regulation 2, homolog) 3 (S. cerevisiae) | chr7 | 1.366 | 6 | 0.097 |  | | | |
| 160876\_at | Bcap29 | B-cell receptor-associated protein 29 | chr12 | 1.484 | 6 | 0.023 |  | | | |
| 160906\_i\_at | NoneAvailable | --- | --- | 3.071 | 6 | 0.425 |  | | | |
| 160955\_at | 2010309E21Rik | RIKEN cDNA 2010309E21 gene | chr6 | 2.304 | 6 | 0.634 |  | | | |
| 160962\_at | Bag2 | Bcl2-associated athanogene 2 | chr1 | 1.485 | 6 | 0.442 |  | | | |
| 161000\_i\_at | 2610201A12Rik | RIKEN cDNA 2610201A12 gene | chr2 | 2.414 | 6 | 0.15 |  | | | |
| 161129\_r\_at | Bag2 | Bcl2-associated athanogene 2 | --- | 1.996 | 6 | 0.084 |  | | | |
| 161147\_f\_at | 1110046L09Rik | RIKEN cDNA 1110046L09 gene | chr8 | 3.517 | 6 | 0.013 |  | | | |
| 161329\_f\_at | NoneAvailable | --- | --- | 1.419 | 6 | 0.55 |  | | | |
| 161361\_s\_at | Tnnt1 | troponin T1, skeletal, slow | chr7 | 2.025 | 6 | 0.12 |  | | | |
| 161872\_f\_at | 1110049G11Rik | RIKEN cDNA 1110049G11 gene | --- | 1.026 | 6 | 0.01 |  | | | |
| 162311\_f\_at | Slc35c2 | solute carrier family 35, member C2 | --- | 1.096 | 6 | 0.598 |  | | | |
| 162337\_f\_at | Alg3 | asparagine-linked glycosylation 3 homolog (yeast, alpha-1,3-mannosyltransferase) | chr16 | 2.101 | 6 | 0.265 |  | | | |
| 92191\_at | 2810410A08Rik | RIKEN cDNA 2810410A08 gene | chr2 | 1.448 | 6 | 0.117 |  | | | |
| 92540\_f\_at | Srm | spermidine synthase | chr4 | 6.091 | 6 | 0.001 |  | | | |
| 92555\_at | Tm4sf6 | transmembrane 4 superfamily member 6 | chrX | 1.293 | 6 | 0.053 |  | | | |
| 92574\_at | 3110001M13Rik | RIKEN cDNA 3110001M13 gene | chr9 | 1.899 | 6 | 0.011 |  | | | |
| 92615\_at | AI837181 | expressed sequence AI837181 | chr4 | 2.807 | 6 | 0.001 |  | | | |
| 92625\_at | Nme2 | expressed in non-metastatic cells 2, protein | chr10 | 1.732 | 6 | 0.004 |  | | | |
| 92630\_r\_at | Hdgf | hepatoma-derived growth factor | chr3 | 2.329 | 6 | 0.057 |  | | | |
| 92631\_f\_at | Calm3 | calmodulin 3 | chr7 | 1.825 | 6 | 0.062 |  | | | |
| 92636\_f\_at | Sec61g | SEC61, gamma subunit | chr1 | 1.533 | 6 | 0.004 |  | | | |
| 92646\_at | Mrpl23 | mitochondrial ribosomal protein L23 | chr2 | 2.152 | 6 | 0.028 |  | | | |
| 92668\_at | Btk | Bruton agammaglobulinemia tyrosine kinase | chrX | 1.527 | 6 | 0.365 |  | | | |
| 92788\_f\_at | Cetn3 | centrin 3 | chr13 | 1.678 | 6 | 0.018 |  | | | |
| 92790\_at | Kpna2 | karyopherin (importin) alpha 2 | chr17 | 2.534 | 6 | 0.582 |  | | | |
| 92798\_at | Atp5c1 | ATP synthase, H+ transporting, mitochondrial F1 complex, gamma polypeptide 1 | chrX | 1.327 | 6 | 0.003 |  | | | |
| 92799\_g\_at | Atp5c1 | ATP synthase, H+ transporting, mitochondrial F1 complex, gamma polypeptide 1 | chr2 | 1.541 | 6 | 0.001 |  | | | |
| 92808\_f\_at | Fkbp4 | FK506 binding protein 4 | chr6 | 1.527 | 6 | 0.18 |  | | | |
| 92824\_at | Nme6 | expressed in non-metastatic cells 6, protein | chr9 | 1.781 | 6 | 0.001 |  | | | |
| 92829\_at | Hspe1 | heat shock protein 1 (chaperonin 10) | chr1 | 1.429 | 6 | 0.001 |  | | | |
| 92831\_at | Sfxn1 | sideroflexin 1 | --- | 1.223 | 6 | 0.005 |  | | | |
| 92840\_at | 3110079L04Rik | RIKEN cDNA 3110079L04 gene | chr1 | 3.044 | 6 | 0.301 |  | | | |
| 92874\_f\_at | Cops7a | COP9 (constitutive photomorphogenic) homolog, subunit 7a (Arabidopsis thaliana) | chr6 | 1.098 | 6 | 0.003 |  | | | |
| 92924\_at | Ctsg | cathepsin G | chr14 | 4.674 | 6 | 0.914 |  | | | |
| 93008\_at | Lsm4 | LSM4 homolog, U6 small nuclear RNA associated (S. cerevisiae) | chr8 | 2.177 | 6 | 0.021 |  | | | |
| 93014\_at | Atp5l | ATP synthase, H+ transporting, mitochondrial F0 complex, subunit g | chr11 | 1.668 | 6 | 0.019 |  | | | |
| 93041\_at | Mcm4 | minichromosome maintenance deficient 4 homolog (S. cerevisiae) | chr16 | 1.922 | 6 | 0.039 |  | | | |
| 93048\_at | Clpp | caseinolytic protease, ATP-dependent, proteolytic subunit homolog (E. coli) | chr17 | 1.566 | 6 | 0.399 |  | | | |
| 93062\_at | Mrpl39 | mitochondrial ribosomal protein L39 | chr16 | 1.936 | 6 | 0.016 |  | | | |
| 93099\_f\_at | Plk | polo-like kinase (Drosophila) | chr7 | 5.772 | 6 | 0.27 |  | | | |
| 93105\_s\_at | Tcrb-V13 | T-cell receptor beta, variable 13 | chr6 | 1.339 | 6 | 0.893 |  | | | |
| 93112\_at | Mcm2 | minichromosome maintenance deficient 2 mitotin (S. cerevisiae) | chr6 | 2.139 | 6 | 0.018 |  | | | |
| 93130\_at | 2600005C20Rik | RIKEN cDNA 2600005C20 gene | --- | 1.646 | 6 | 0.477 |  | | | |
| 93144\_at | AI317237 | expressed sequence AI317237 | chr15 | 1.137 | 6 | 0.505 |  | | | |
| 93183\_at | NoneAvailable | Mus musculus, Similar to CDC91 cell division cycle 91-like 1 (S. cerevisiae), clone IMAGE:5374066, mRNA, partial cds | chr2 | 1.687 | 6 | 0.062 |  | | | |
| 93236\_s\_at | Tyms | thymidylate synthase | chr10 | 3.228 | 6 | 0.012 |  | | | |
| 93237\_s\_at | Tyms | thymidylate synthase | chr10 | 2.144 | 6 | 0.021 |  | | | |
| 93250\_r\_at | Hmgb2 | high mobility group box 2 | --- | 1.909 | 6 | 0.087 |  | | | |
| 93258\_at | Hmbs | hydroxymethylbilane synthase | chr9 | 2.914 | 6 | 0.046 |  | | | |
| 93277\_at | Hspd1 | heat shock protein 1 (chaperonin) | chr1 | 1.14 | 6 | 0.029 |  | | | |
| 93290\_at | Pnp | purine-nucleoside phosphorylase | chr14 | 1.213 | 6 | 0.044 |  | | | |
| 93518\_at | Rnps1 | ribonucleic acid binding protein S1 | chr17 | 1.642 | 6 | 0.146 |  | | | |
| 93519\_s\_at | Nedd8 | neural precursor cell expressed, developmentally down-regulated gene 8 | chr14 | 2.012 | 6 | 0.002 |  | | | |
| 93531\_at | Ndufa8 | NADH dehydrogenase (ubiquinone) 1 alpha subcomplex, 8 | chr2 | 1.477 | 6 | 0.042 |  | | | |
| 93533\_at | 1500011L16Rik | RIKEN cDNA 1500011L16 gene | --- | 2.198 | 6 | 0.022 |  | | | |
| 93539\_at | 1810004D07Rik | RIKEN cDNA 1810004D07 gene | --- | 1.406 | 6 | 0 |  | | | |
| 93542\_at | Pter | phosphotriesterase related | chr2 | 1.123 | 6 | 0.022 |  | | | |
| 93548\_at | Sec61b | Sec61 beta subunit | chr4 | 2.149 | 6 | 0.005 |  | | | |
| 93559\_at | Apex1 | apurinic/apyrimidinic endonuclease 1 | chr14 | 1.957 | 6 | 0.002 |  | | | |
| 93560\_at | Acyp1 | acylphosphatase 1, erythrocyte (common) type | chr12 | 3.035 | 6 | 0.029 |  | | | |
| 93572\_at | Ndufs1 | NADH dehydrogenase (ubiquinone) Fe-S protein 1 | chr1 | 1.372 | 6 | 0.122 |  | | | |
| 93573\_at | Mt1 | metallothionein 1 | chr8 | 2.351 | 6 | 0.398 |  | | | |
| 93579\_at | 5830427H10Rik | RIKEN cDNA 5830427H10 gene | chr6 | 1.438 | 6 | 0.034 |  | | | |
| 93581\_at | Ndufb8 | NADH dehydrogenase (ubiquinone) 1 beta subcomplex 8 | chr19 | 2.79 | 6 | 0.003 |  | | | |
| 93582\_at | Coq7 | demethyl-Q 7 | chr7 | 3.745 | 6 | 0.001 |  | | | |
| 93596\_i\_at | Atp5e | ATP synthase, H+ transporting, mitochondrial F1 complex, epsilon subunit | chr2 | 1.737 | 6 | 0.006 |  | | | |
| 93603\_at | Mrpl40 | mitochondrial ribosomal protein L40 | chr16 | 2.475 | 6 | 0.051 |  | | | |
| 93608\_at | Ebi3 | Epstein-Barr virus induced gene 3 | chr17 | 2.76 | 6 | 0.255 |  | | | |
| 93620\_at | Rpo1-4 | RNA polymerase 1-4 | chr6 | 2.127 | 6 | 0.222 |  | | | |
| 93676\_at | Rad51ap1 | RAD51 associated protein 1 | chr6 | 1.146 | 6 | 0.135 |  | | | |
| 93735\_f\_at | Psmc3 | proteasome (prosome, macropain) 26S subunit, ATPase 3 | chr2 | 1.365 | 6 | 0.011 |  | | | |
| 93762\_at | Ppp2r4 | protein phosphatase 2A, regulatory subunit B (PR 53) | chr2 | 2.487 | 6 | 0.265 |  | | | |
| 93780\_at | Them2 | thioesterase superfamily member 2 | chr13 | 1.504 | 6 | 0.012 |  | | | |
| 93784\_at | Cfdp | craniofacial development protein 1 | chr8 | 1.424 | 6 | 0.031 |  | | | |
| 93786\_i\_at | Mrpl18 | mitochondrial ribosomal protein L18 | chr17 | 2.577 | 6 | 0.013 |  | | | |
| 93787\_f\_at | Mrpl18 | mitochondrial ribosomal protein L18 | --- | 2.215 | 6 | 0.009 |  | | | |
| 93795\_at | Itpa | inosine triphosphatase (nucleoside triphosphate pyrophosphatase) | chr2 | 1.788 | 6 | 0.084 |  | | | |
| 93799\_at | AI316787 | expressed sequence AI316787 | chr11 | 1.925 | 6 | 0.053 |  | | | |
| 93805\_at | 1110003H09Rik | RIKEN cDNA 1110003H09 gene | chr2 | 2.548 | 6 | 0.254 |  | | | |
| 93812\_at | Clns1a | chloride channel, nucleotide-sensitive, 1A | chr7 | 1.373 | 6 | 0.031 |  | | | |
| 93820\_at | Cox7a2 | cytochrome c oxidase, subunit VIIa 2 | chr9 | 1.951 | 6 | 0.002 |  | | | |
| 93833\_s\_at | Hist1h2bc | histone 1, H2bc | chr13 | 1.06 | 6 | 0.043 |  | | | |
| 93838\_at | 2700038C09Rik | RIKEN cDNA 2700038C09 gene | chr2 | 3.273 | 6 | 0.025 |  | | | |
| 93844\_at | 1500040F11Rik | RIKEN cDNA 1500040F11 gene | chr11 | 2.937 | 6 | 0.067 |  | | | |
| 93918\_at | Taf9 | TAF9 RNA polymerase II, TATA box binding protein (TBP)-associated factor | chr6 | 1.138 | 6 | 0.146 |  | | | |
| 93924\_f\_at | Tuba7 | tubulin, alpha 7 | chr6 | 1.57 | 6 | 0.084 |  | | | |
| 93984\_at | Atpi | ATPase inhibitor | --- | 1.332 | 6 | 0 |  | | | |
| 93988\_at | Psma7 | proteasome (prosome, macropain) subunit, alpha type 7 | chr2 | 1.392 | 6 | 0.02 |  | | | |
| 93991\_at | Mor1 | malate dehydrogenase, mitochondrial | chr5 | 1.136 | 6 | 0.009 |  | | | |
| 93993\_at | Lman2 | lectin, mannose-binding 2 | chr13 | 1.647 | 6 | 0.002 |  | | | |
| 94014\_at | 2510048O06Rik | RIKEN cDNA 2510048O06 gene | --- | 1.798 | 6 | 0 |  | | | |
| 94020\_at | Ptk9l | PTK9 protein typrotein tyrosine kinase 9-like (A6-related protein) | chr9 | 1.253 | 6 | 0.473 |  | | | |
| 94025\_at | Psmb3 | proteasome (prosome, macropain) subunit, beta type 3 | chr16 | 1.932 | 6 | 0.035 |  | | | |
| 94030\_at | D3Ertd176e | DNA segment, Chr 3, ERATO Doi 176, expressed | chrX | 1.151 | 6 | 0.17 |  | | | |
| 94040\_at | Erh | enhancer of rudimentary homolog (Drosophila) | chr12 | 1.929 | 6 | 0.001 |  | | | |
| 94062\_at | Ndufv2 | NADH dehydrogenase (ubiquinone) flavoprotein 2 | chr17 | 1.3 | 6 | 0.045 |  | | | |
| 94067\_at | 1700001E16Rik | RIKEN cDNA 1700001E16 gene | chr9 | 2.68 | 6 | 0.117 |  | | | |
| 94111\_r\_at | NoneAvailable | Mus musculus 0 day neonate thymus cDNA, RIKEN full-length enriched library, clone:A430088G18 product:hypothetical Zinc finger, C2H2 type containing protein, full insert sequence | --- | 1.513 | 6 | 0.37 |  | | | |
| 94209\_g\_at | P5-pending | protein disulfide isomerase-related protein | chr12 | 2.132 | 6 | 0.144 |  | | | |
| 94210\_at | Timm9 | translocase of inner mitochondrial membrane 9 homolog (yeast) | chr10 | 3.713 | 6 | 0.022 |  | | | |
| 94217\_f\_at | Cdca3 | cell division cycle associated 3 | chr6 | 1.907 | 6 | 0.09 |  | | | |
| 94243\_at | 4930432B04Rik | RIKEN cDNA 4930432B04 gene | chr2 | 1.18 | 6 | 0.343 |  | | | |
| 94253\_at | Eif2s1 | eukaryotic translation initiation factor 2, subunit 1 alpha | chr12 | 1.884 | 6 | 0.063 |  | | | |
| 94258\_at | Arhgdib | Rho, GDP dissociation inhibitor (GDI) beta | chr6 | 1.003 | 6 | 0.138 |  | | | |
| 94263\_f\_at | Psmb7 | proteasome (prosome, macropain) subunit, beta type 7 | chr2 | 1.959 | 6 | 0.018 |  | | | |
| 94275\_at | Urod | uroporphyrinogen decarboxylase | chr4 | 1.492 | 6 | 0.015 |  | | | |
| 94277\_at | Mtx1 | metaxin 1 | chr3 | 1.276 | 6 | 0.007 |  | | | |
| 94294\_at | Ccnb2 | cyclin B2 | chr9 | 4.408 | 6 | 0.001 |  | | | |
| 94301\_at | Atp6v0e | ATPase, H+ transporting, V0 subunit | chr17 | 1.019 | 6 | 0.076 |  | | | |
| 94302\_at | Psmd4 | proteasome (prosome, macropain) 26S subunit, non-ATPase, 4 | chr3 | 1.486 | 6 | 0.208 |  | | | |
| 94321\_at | Krt1-10 | keratin complex 1, acidic, gene 10 | chr11 | 1.699 | 6 | 0.479 |  | | | |
| 94323\_at | D630012G11Rik | RIKEN cDNA D630012G11 gene | chr19 | 1.879 | 6 | 0 |  | | | |
| 94327\_at | Mrps18a | mitochondrial ribosomal protein S18A | --- | 1.219 | 6 | 0.187 |  | | | |
| 94360\_at | 2700029M09Rik | RIKEN cDNA 2700029M09 gene | chr8 | 1.272 | 6 | 0.009 |  | | | |
| 94366\_at | 2310079N02Rik | RIKEN cDNA 2310079N02 gene | chr8 | 1.138 | 6 | 0.072 |  | | | |
| 94372\_at | Nudt1 | nudix (nucleoside diphosphate linked moiety X)-type motif 1 | --- | 3.289 | 6 | 0.003 |  | | | |
| 94410\_f\_at | 2700094L05Rik | RIKEN cDNA 2700094L05 gene | chr14 | 1.38 | 6 | 0.144 |  | | | |
| 94451\_at | D13Wsu123e | DNA segment, Chr 13, Wayne State University 123, expressed | chr13 | 1.44 | 6 | 0.068 |  | | | |
| 94452\_g\_at | D13Wsu123e | DNA segment, Chr 13, Wayne State University 123, expressed | chr13 | 1.248 | 6 | 0.137 |  | | | |
| 94455\_at | Lsm3 | LSM3 homolog, U6 small nuclear RNA associated (S. cerevisiae) | chr6 | 2.688 | 6 | 0.002 |  | | | |
| 94462\_at | Eif2b1 | eukaryotic translation initiation factor 2B, subunit 1 (alpha) | chr5 | 1.441 | 6 | 0.278 |  | | | |
| 94488\_at | 1110059P08Rik | RIKEN cDNA 1110059P08 gene | chr10 | 1.567 | 6 | 0.795 |  | | | |
| 94494\_at | Farsl | phenylalanine-tRNA synthetase-like | chr1 | 1.832 | 6 | 0.008 |  | | | |
| 94503\_at | Mel | cell line NK14 derived transforming oncogene | --- | 1.07 | 6 | 0.246 |  | | | |
| 94518\_at | 0610033H09Rik | RIKEN cDNA 0610033H09 gene | --- | 1.398 | 6 | 0.263 |  | | | |
| 94526\_at | D10Ertd214e | DNA segment, Chr 10, ERATO Doi 214, expressed | chr10 | 2.227 | 6 | 0 |  | | | |
| 94531\_at | 2310005O14Rik | RIKEN cDNA 2310005O14 gene | --- | 1.02 | 6 | 0.33 |  | | | |
| 94548\_at | 1200003O06Rik | RIKEN cDNA 1200003O06 gene | chr3 | 1.452 | 6 | 0.079 |  | | | |
| 94558\_g\_at | Gtf3a | general transcription factor III A | chr5 | 1.943 | 6 | 0.011 |  | | | |
| 94789\_r\_at | Tubb5 | tubulin, beta 5 | chr17 | 3.275 | 6 | 0.044 |  | | | |
| 94805\_f\_at | NoneAvailable | --- | --- | 5.636 | 6 | 0.123 |  | | | |
| 94841\_at | Psma5 | proteasome (prosome, macropain) subunit, alpha type 5 | chr10 | 1.755 | 6 | 0.007 |  | | | |
| 94842\_at | Blmh | bleomycin hydrolase | chr11 | 1.008 | 6 | 0.068 |  | | | |
| 94870\_f\_at | 2310075M17Rik | RIKEN cDNA 2310075M17 gene | chr11 | 1.008 | 6 | 0.02 |  | | | |
| 94892\_r\_at | Mea1 | male enhanced antigen 1 | chr17 | 1.005 | 6 | 0.017 |  | | | |
| 94912\_at | Mrps21 | mitochondrial ribosomal protein S21 | chr3 | 1.449 | 6 | 0.017 |  | | | |
| 94931\_at | 1810045K17Rik | RIKEN cDNA 1810045K17 gene | chr3 | 1.276 | 6 | 0.034 |  | | | |
| 94933\_at | BC008155 | cDNA sequence BC008155 | chr17 | 1.315 | 6 | 0.039 |  | | | |
| 94971\_at | NoneAvailable | Mus musculus cDNA clone MGC:58593 IMAGE:6705916, complete cds | chr14 | 1.64 | 6 | 0.035 |  | | | |
| 95003\_at | Mt1a | metallothionein-I activator | chr15 | 1.535 | 6 | 0.051 |  | | | |
| 95018\_r\_at | AU021838 | expressed sequence AU021838 | chr12 | 1.114 | 6 | 0.17 |  | | | |
| 95045\_at | 0610012D09Rik | RIKEN cDNA 0610012D09 gene | chr7 | 1.41 | 6 | 0 |  | | | |
| 95049\_at | Snrpd2 | small nuclear ribonucleoprotein D2 | chr10 | 1.485 | 6 | 0.03 |  | | | |
| 95053\_s\_at | Sdhb | succinate dehydrogenase complex, subunit B, iron sulfur (Ip) | chr4 | 2.681 | 6 | 0.023 |  | | | |
| 95067\_at | Mrpl2 | mitochondrial ribosomal protein L2 | chr17 | 1.667 | 6 | 0.023 |  | | | |
| 95091\_at | Sec13r | SEC13 related gene (S. cerevisiae) | --- | 1.672 | 6 | 0.001 |  | | | |
| 95114\_s\_at | 0610039D01Rik | RIKEN cDNA 0610039D01 gene | chr17 | 2.751 | 6 | 0.077 |  | | | |
| 95132\_r\_at | Ndufb2 | NADH dehydrogenase (ubiquinone) 1 beta subcomplex, 2 | chr6 | 1.988 | 6 | 0.024 |  | | | |
| 95136\_at | Arl6ip4 | ADP-ribosylation factor-like 6 interacting protein 4 | chr5 | 1.084 | 6 | 0.886 |  | | | |
| 95137\_at | 1810014L12Rik | RIKEN cDNA 1810014L12 gene | chr11 | 2.422 | 6 | 0.308 |  | | | |
| 95148\_at | Ak2 | adenylate kinase 2 | chr4 | 1.085 | 6 | 0.273 |  | | | |
| 95149\_at | Copz1 | coatomer protein complex, subunit zeta 1 | chr15 | 1.495 | 6 | 0.324 |  | | | |
| 95159\_at | Mrps18b | mitochondrial ribosomal protein S18B | chr2 | 2.706 | 6 | 0.236 |  | | | |
| 95184\_f\_at | NoneAvailable | --- | --- | 1.131 | 6 | 0.148 |  | | | |
| 95406\_at | 1810037I17Rik | RIKEN cDNA 1810037I17 gene | --- | 1.17 | 6 | 0.561 |  | | | |
| 95409\_at | 1110019J04Rik | RIKEN cDNA 1110019J04 gene | chr4 | 1.897 | 6 | 0.354 |  | | | |
| 95420\_at | Pgd | phosphogluconate dehydrogenase | chr4 | 1.058 | 6 | 0.374 |  | | | |
| 95428\_at | D1Wsu40e | DNA segment, Chr 1, Wayne State University 40, expressed | chr1 | 1.673 | 6 | 0.136 |  | | | |
| 95441\_at | Timm23 | translocase of inner mitochondrial membrane 23 homolog (yeast) | chr1 | 1.675 | 6 | 0.042 |  | | | |
| 95448\_at | Psmc2 | proteasome (prosome, macropain) 26S subunit, ATPase 2 | chr5 | 1.408 | 6 | 0.017 |  | | | |
| 95460\_at | Cops5 | COP9 (constitutive photomorphogenic) homolog, subunit 5 (Arabidopsis thaliana) | chr1 | 1.564 | 6 | 0.025 |  | | | |
| 95480\_at | D11Wsu68e | DNA segment, Chr 11, Wayne State University 68, expressed | chr11 | 1.471 | 6 | 0.003 |  | | | |
| 95483\_at | Psmd1 | proteasome (prosome, macropain) 26S subunit, non-ATPase, 1 | chr1 | 1.459 | 6 | 0.095 |  | | | |
| 95491\_at | Park7 | Parkinson disease (autosomal recessive, early onset) 7 | chr4 | 1.248 | 6 | 0.002 |  | | | |
| 95497\_at | 1110005A05Rik | RIKEN cDNA 1110005A05 gene | chr9 | 3.276 | 6 | 0.011 |  | | | |
| 95498\_at | Mrps15 | mitochondrial ribosomal protein S15 | chr4 | 2.035 | 6 | 0.005 |  | | | |
| 95517\_i\_at | BC004004 | cDNA sequence BC004004 | chr17 | 3.826 | 6 | 0.116 |  | | | |
| 95523\_at | 6530401D17Rik | RIKEN cDNA 6530401D17 gene | chrX | 1.93 | 6 | 0.05 |  | | | |
| 95551\_at | 1700020M16Rik | RIKEN cDNA 1700020M16 gene | chr14 | 1.379 | 6 | 0.293 |  | | | |
| 95590\_at | Alg5 | asparagine-linked glycosylation 5 homolog (yeast, dolichyl-phosphate beta-glucosyltransferase) | chr3 | 1.43 | 6 | 0.003 |  | | | |
| 95593\_at | Golph2 | golgi phosphoprotein 2 | chr13 | 1.191 | 6 | 0.018 |  | | | |
| 95602\_at | Trpc4ap | transient receptor potential cation channel, subfamily C, member 4 associated protein | chr2 | 1.139 | 6 | 0.338 |  | | | |
| 95606\_at | Nsap1l-pending | NS1-associated protein 1-like | chr9 | 1.239 | 6 | 0.005 |  | | | |
| 95619\_at | Cct7 | chaperonin subunit 7 (eta) | chr6 | 1.327 | 6 | 0.063 |  | | | |
| 95629\_at | NoneAvailable | Mus musculus transcribed sequence with strong similarity to protein ref:NP\_067033.1 (H.sapiens)  MUM2 protein [Homo sapiens] | chr11 | 3.455 | 6 | 0.078 |  | | | |
| 95634\_at | 0610010K14Rik | RIKEN cDNA 0610010K14 gene | --- | 1.777 | 6 | 0.023 |  | | | |
| 95636\_at | 0610010K14Rik | RIKEN cDNA 0610010K14 gene | chr11 | 2.424 | 6 | 0.007 |  | | | |
| 95649\_at | Phf5a | PHD finger protein 5A | chr15 | 1.933 | 6 | 0.012 |  | | | |
| 95650\_at | Ssfa1 | sperm specific antigen 1 | chr15 | 1.573 | 6 | 0.108 |  | | | |
| 95656\_i\_at | D13Wsu177e | DNA segment, Chr 13, Wayne State University 177, expressed | chr13 | 2.603 | 6 | 0.011 |  | | | |
| 95657\_f\_at | D13Wsu177e | DNA segment, Chr 13, Wayne State University 177, expressed | chr13 | 1.618 | 6 | 0.086 |  | | | |
| 95660\_at | 0610025L15Rik | RIKEN cDNA 0610025L15 gene | chr7 | 2.58 | 6 | 0 |  | | | |
| 95677\_at | 0610009C03Rik | RIKEN cDNA 0610009C03 gene | --- | 2.789 | 6 | 0.026 |  | | | |
| 95685\_at | 5730436H21Rik | RIKEN cDNA 5730436H21 gene | chr19 | 1.92 | 6 | 0.565 |  | | | |
| 95690\_at | 1110030L07Rik | RIKEN cDNA 1110030L07 gene | chr15 | 3.584 | 6 | 0.007 |  | | | |
| 95693\_at | Idh2 | isocitrate dehydrogenase 2 (NADP+), mitochondrial | chr7 | 1.921 | 6 | 0.021 |  | | | |
| 95696\_at | Txnl2 | thioredoxin-like 2 | chr4 | 1.145 | 6 | 0.009 |  | | | |
| 95698\_at | Ndufb7 | NADH dehydrogenase (ubiquinone) 1 beta subcomplex, 7 | chr8 | 2.162 | 6 | 0.008 |  | | | |
| 95707\_at | 2900010M23Rik | RIKEN cDNA 2900010M23 gene | chr17 | 2.087 | 6 | 0.032 |  | | | |
| 95712\_at | Orc6l | origin recognition complex, subunit 6-like (S. cerevisiae) | chr8 | 2.028 | 6 | 0.066 |  | | | |
| 95714\_at | 0610009D07Rik | RIKEN cDNA 0610009D07 gene | chr12 | 1.473 | 6 | 0.027 |  | | | |
| 95717\_at | Elp3 | elongation protein 3 homolog (S. cerevisiae) | chr14 | 2.783 | 6 | 0.523 |  | | | |
| 95718\_f\_at | Usmg5 | upregulated during skeletal muscle growth 5 | chr19 | 1.302 | 6 | 0.006 |  | | | |
| 95730\_at | Mrps34 | mitochondrial ribosomal protein S34 | chr17 | 1.964 | 6 | 0.378 |  | | | |
| 95742\_at | Psmd13 | proteasome (prosome, macropain) 26S subunit, non-ATPase, 13 | --- | 2.006 | 6 | 0.053 |  | | | |
| 95760\_at | 1110011K10Rik | RIKEN cDNA 1110011K10 gene | chr9 | 2.372 | 6 | 0.002 |  | | | |
| 95891\_at | NoneAvailable | Mus musculus transcribed sequences | chr16 | 3.953 | 6 | 0.017 |  | | | |
| 95984\_at | NoneAvailable | Mus musculus transcribed sequences | --- | 1.143 | 6 | 0.323 |  | | | |
| 96016\_at | NoneAvailable | Mus musculus cDNA clone MGC:67366 IMAGE:5683334, complete cds | chr2 | 3.771 | 6 | 0.015 |  | | | |
| 96017\_at | 0610006I08Rik | RIKEN cDNA 0610006I08 gene | chr19 | 2.304 | 6 | 0.082 |  | | | |
| 96024\_at | Ahcy | S-adenosylhomocysteine hydrolase | chr16 | 1.032 | 6 | 0.066 |  | | | |
| 96029\_at | Sf3a3 | splicing factor 3a, subunit 3, 60kDa | chr4 | 2.16 | 6 | 0.047 |  | | | |
| 96052\_at | Acp1 | acid phosphatase 1, soluble | --- | 1.712 | 6 | 0.022 |  | | | |
| 96059\_at | D4Ertd786e | DNA segment, Chr 4, ERATO Doi 786, expressed | chr4 | 1.107 | 6 | 0.055 |  | | | |
| 96068\_at | 1500034J20Rik | RIKEN cDNA 1500034J20 gene | chr5 | 1.263 | 6 | 0.067 |  | | | |
| 96081\_at | Tk1 | thymidine kinase 1 | chr11 | 2.893 | 6 | 0.014 |  | | | |
| 96089\_at | 4931406C07Rik | RIKEN cDNA 4931406C07 gene | chr9 | 1.515 | 6 | 0.003 |  | | | |
| 96090\_g\_at | 4931406C07Rik | RIKEN cDNA 4931406C07 gene | chr9 | 1.088 | 6 | 0.103 |  | | | |
| 96098\_at | Mrpl36 | mitochondrial ribosomal protein L36 | chr13 | 2.885 | 6 | 0.062 |  | | | |
| 96106\_at | 2400006P09Rik | RIKEN cDNA 2400006P09 gene | chr8 | 1.766 | 6 | 0.08 |  | | | |
| 96112\_at | Etfa | electron transferring flavoprotein, alpha polypeptide | chr9 | 1.779 | 6 | 0.006 |  | | | |
| 96234\_at | Cpsf3 | cleavage and polyadenylation specificity factor 3 | --- | 1.301 | 6 | 0.153 |  | | | |
| 96256\_at | Prdx3 | peroxiredoxin 3 | chr19 | 1.229 | 6 | 0.198 |  | | | |
| 96261\_at | NoneAvailable | Mus musculus cDNA clone MGC:67622 IMAGE:6410794, complete cds | chr4 | 1.384 | 6 | 0 |  | | | |
| 96267\_at | Ndufv1 | NADH dehydrogenase (ubiquinone) flavoprotein 1 | chr19 | 1.004 | 6 | 0.016 |  | | | |
| 96268\_at | Suclg1 | succinate-CoA ligase, GDP-forming, alpha subunit | chr6 | 1.173 | 6 | 0.015 |  | | | |
| 96289\_at | Stoml2 | stomatin (Epb7.2)-like 2 | chr4 | 2.901 | 6 | 0.001 |  | | | |
| 96291\_f\_at | NoneAvailable | Mus musculus cDNA clone IMAGE:6772417, with apparent retained intron | chr13 | 2.298 | 6 | 0 |  | | | |
| 96292\_r\_at | NoneAvailable | Mus musculus cDNA clone IMAGE:6772417, with apparent retained intron | chr13 | 1.544 | 6 | 0.01 |  | | | |
| 96293\_at | 2410015N17Rik | RIKEN cDNA 2410015N17 gene | chr7 | 4.422 | 6 | 0.006 |  | | | |
| 96294\_s\_at | 2410015N17Rik | RIKEN cDNA 2410015N17 gene | chr7 | 1.904 | 6 | 0.129 |  | | | |
| 96296\_at | Mrpl15 | mitochondrial ribosomal protein L15 | chr1 | 1.134 | 6 | 0.051 |  | | | |
| 96297\_at | Ebna1bp2 | EBNA1 binding protein 2 | chr4 | 1.427 | 6 | 0.057 |  | | | |
| 96319\_at | Cdc20 | cell division cycle 20 homolog (S. cerevisiae) | --- | 4.591 | 6 | 0.004 |  | | | |
| 96321\_at | Ndufa9 | NADH dehydrogenase (ubiquinone) 1 alpha subcomplex, 9 | chr6 | 1.871 | 6 | 0.001 |  | | | |
| 96342\_at | 1700006C06Rik | RIKEN cDNA 1700006C06 gene | chr8 | 1.206 | 6 | 0.073 |  | | | |
| 96353\_at | 1110021D01Rik | RIKEN cDNA 1110021D01 gene | chr13 | 2.999 | 6 | 0.003 |  | | | |
| 96541\_at | X83328 | EST X83328 | --- | 1.322 | 6 | 0.061 |  | | | |
| 96579\_at | NoneAvailable | Mus musculus transcribed sequences | chr3 | 1.592 | 6 | 0.27 |  | | | |
| 96610\_at | Atp6v1h | ATPase, H+ transporting, lysosomal 50/57kDa, V1 subunit H | chr1 | 1.19 | 6 | 0.341 |  | | | |
| 96613\_at | 5730536A07Rik | RIKEN cDNA 5730536A07 gene | chr9 | 2.658 | 6 | 0.014 |  | | | |
| 96621\_at | 1110061L23Rik | RIKEN cDNA 1110061L23 gene | chr7 | 1.294 | 6 | 0.056 |  | | | |
| 96627\_at | Ebp | phenylalkylamine Ca2+ antagonist (emopamil) binding protein | chrX | 1.299 | 6 | 0.009 |  | | | |
| 96641\_at | Noc4 | neighbor of Cox4 | chr16 | 2.286 | 6 | 0.092 |  | | | |
| 96648\_at | Coro1a | coronin, actin binding protein 1A | chr7 | 1.079 | 6 | 0.668 |  | | | |
| 96652\_at | Mrpl28 | mitochondrial ribosomal protein L28 | chr17 | 1.368 | 6 | 0.026 |  | | | |
| 96658\_at | 2900010J23Rik | RIKEN cDNA 2900010J23 gene | chr12 | 2.213 | 6 | 0.051 |  | | | |
| 96668\_at | Timm17b | translocator of inner mitochondrial membrane b | chrX | 2.162 | 6 | 0.003 |  | | | |
| 96677\_at | 2410195B05Rik | RIKEN cDNA 2410195B05 gene | chr5 | 1.851 | 6 | 0.564 |  | | | |
| 96693\_at | NoneAvailable | Mus musculus transcribed sequence with moderate similarity to protein ref:NP\_002878.2 (H.sapiens)  arginyl-tRNA synthetase [Homo sapiens] | chr11 | 1.588 | 6 | 0.246 |  | | | |
| 96696\_at | Hrmt1l2 | heterogeneous nuclear ribonucleoproteins  methyltransferase-like 2 (S. cerevisiae) | chr7 | 2.608 | 6 | 0.013 |  | | | |
| 96698\_at | Psmd5 | proteasome (prosome, macropain) 26S subunit, non-ATPase, 5 | chr2 | 2.136 | 6 | 0.055 |  | | | |
| 96743\_at | Skiip | SKI interacting protein | --- | 1.897 | 6 | 0.005 |  | | | |
| 96772\_at | Prim1 | DNA primase, p49 subunit | chr10 | 1.016 | 6 | 0.058 |  | | | |
| 96778\_at | Rrs1 | RRS1 ribosome biogenesis regulator homolog (S. cerevisiae) | chr1 | 1.06 | 6 | 0.904 |  | | | |
| 96850\_at | 4833436O05 | hypothetical protein 4833436O05 | chr4 | 1.185 | 6 | 0.217 |  | | | |
| 96861\_at | Mrpl50 | mitochondrial ribosomal protein L50 | chr4 | 1.687 | 6 | 0.015 |  | | | |
| 96871\_at | 2310042G06Rik | RIKEN cDNA 2310042G06 gene | --- | 2.147 | 6 | 0.058 |  | | | |
| 96878\_at | 1810044O22Rik | RIKEN cDNA 1810044O22 gene | --- | 1.78 | 6 | 0.078 |  | | | |
| 96883\_at | Eif3s4 | eukaryotic translation initiation factor 3, subunit 4 (delta) | chr9 | 2.258 | 6 | 0.602 |  | | | |
| 96885\_at | 2510015F01Rik | RIKEN cDNA 2510015F01 gene | --- | 4.242 | 6 | 0.09 |  | | | |
| 96892\_at | Psma1 | proteasome (prosome, macropain) subunit, alpha type 1 | chr7 | 1.257 | 6 | 0.017 |  | | | |
| 96899\_at | Ndufs3 | NADH dehydrogenase (ubiquinone) Fe-S protein 3 | chr11 | 1.275 | 6 | 0.019 |  | | | |
| 96902\_at | 2900091E11Rik | RIKEN cDNA 2900091E11 gene | chr10 | 1.339 | 6 | 0.042 |  | | | |
| 96909\_at | Ndufab1 | NADH dehydrogenase (ubiquinone) 1, alpha/beta subcomplex, 1 | chr7 | 1.256 | 6 | 0.014 |  | | | |
| 96915\_f\_at | Ndufa3 | NADH dehydrogenase (ubiquinone) 1 alpha subcomplex, 3 | chr7 | 1.552 | 6 | 0.093 |  | | | |
| 96942\_at | Eif3s6ip | eukaryotic translation initiation factor 3, subunit 6 interacting protein | chr15 | 1.092 | 6 | 0.095 |  | | | |
| 96947\_at | Etfb | electron transferring flavoprotein, beta polypeptide | chr7 | 2.477 | 6 | 0 |  | | | |
| 96952\_at | Psma6 | proteasome (prosome, macropain) subunit, alpha type 6 | chr12 | 1.235 | 6 | 0.004 |  | | | |
| 97055\_s\_at | NoneAvailable | --- | chr16 | 1.087 | 6 | 0.01 |  | | | |
| 97164\_at | 2610207P08Rik | RIKEN cDNA 2610207P08 gene | --- | 3.233 | 6 | 0.011 |  | | | |
| 97179\_at | NoneAvailable | Mus musculus mRNA similar to putative c-Myc-responsive (cDNA clone MGC:54855 IMAGE:5388297), complete cds | --- | 2.605 | 6 | 0.001 |  | | | |
| 97201\_s\_at | Ndufa5 | NADH dehydrogenase (ubiquinone) 1 alpha subcomplex, 5 | chr6 | 1.857 | 6 | 0.001 |  | | | |
| 97220\_at | Dscr2 | Down syndrome critical region homolog 2 (human) | chr16 | 5.415 | 6 | 0 |  | | | |
| 97237\_at | 1810003N24Rik | RIKEN cDNA 1810003N24 gene | chr11 | 2.065 | 6 | 0.064 |  | | | |
| 97248\_at | Dbi | diazepam binding inhibitor | --- | 1.56 | 6 | 0 |  | | | |
| 97249\_at | D7Wsu180e | DNA segment, Chr 7, Wayne State University 180, expressed | chr7 | 1.852 | 6 | 0.006 |  | | | |
| 97251\_at | Mrps10 | mitochondrial ribosomal protein S10 | chr17 | 1.133 | 6 | 0.54 |  | | | |
| 97268\_i\_at | 0610010I12Rik | RIKEN cDNA 0610010I12 gene | chr3 | 1.265 | 6 | 0.003 |  | | | |
| 97274\_at | Psmd14 | proteasome (prosome, macropain) 26S subunit, non-ATPase, 14 | chr2 | 2.128 | 6 | 0.001 |  | | | |
| 97295\_at | D4Ertd421e | DNA segment, Chr 4, ERATO Doi 421, expressed | chr4 | 3.172 | 6 | 0.093 |  | | | |
| 97296\_at | Mrpl44 | mitochondrial ribosomal protein L44 | chr1 | 1.473 | 6 | 0.18 |  | | | |
| 97307\_f\_at | Ndufb5 | NADH dehydrogenase (ubiquinone) 1 beta subcomplex, 5 | --- | 1.376 | 6 | 0.003 |  | | | |
| 97342\_at | Mrps14 | mitochondrial ribosomal protein S14 | chr1 | 2.351 | 6 | 0.001 |  | | | |
| 97364\_at | Asf1b | ASF1 anti-silencing function 1 homolog B (S. cerevisiae) | chr8 | 1.409 | 6 | 0.389 |  | | | |
| 97374\_at | 2810025M15Rik | RIKEN cDNA 2810025M15 gene | chr1 | 2.124 | 6 | 0.003 |  | | | |
| 97390\_at | Cdc25a | cell division cycle 25 homolog A (S. cerevisiae) | chr9 | 3.04 | 6 | 0.263 |  | | | |
| 97407\_at | 1110033G07Rik | RIKEN cDNA 1110033G07 gene | chr2 | 1.896 | 6 | 0.06 |  | | | |
| 97412\_at | 3300001G02Rik | RIKEN cDNA 3300001G02 gene | chr11 | 2.545 | 6 | 0.004 |  | | | |
| 97421\_at | Smc2l1 | SMC2 structural maintenance of chromosomes 2-like 1 (yeast) | chr4 | 1.218 | 6 | 0.139 |  | | | |
| 97444\_at | Ifi30 | interferon gamma inducible protein 30 | chr8 | 1.201 | 6 | 0.096 |  | | | |
| 97452\_at | H2afy | H2A histone family, member Y | --- | 1.491 | 6 | 0.098 |  | | | |
| 97459\_at | Psma4 | proteasome (prosome, macropain) subunit, alpha type 4 | chr18 | 1.676 | 6 | 0.03 |  | | | |
| 97468\_at | Cks1 | CDC28 protein kinase 1 | chr3 | 2.843 | 6 | 0 |  | | | |
| 97473\_at | NoneAvailable | --- | chr7 | 1.496 | 6 | 0.33 |  | | | |
| 97477\_at | Timm8b | translocase of inner mitochondrial membrane 8 homolog b (yeast) | chr9 | 1.26 | 6 | 0.04 |  | | | |
| 97479\_at | Ube2l3 | ubiquitin-conjugating enzyme E2L 3 | chr16 | 1.447 | 6 | 0.178 |  | | | |
| 97488\_at | 1200011O22Rik | RIKEN cDNA 1200011O22 gene | chr5 | 1.991 | 6 | 0.502 |  | | | |
| 97527\_at | Cks2 | CDC28 protein kinase regulatory subunit 2 | chr1 | 1.069 | 6 | 0.566 |  | | | |
| 97538\_at | Gus | beta-glucuronidase | chr5 | 1.607 | 6 | 0.033 |  | | | |
| 97554\_at | BC005624 | cDNA sequence BC005624 | chr2 | 1.271 | 6 | 0.235 |  | | | |
| 97758\_at | Prdx1 | peroxiredoxin 1 | chr8 | 1.211 | 6 | 0.01 |  | | | |
| 97807\_at | 1110021H02Rik | RIKEN cDNA 1110021H02 gene | chr1 | 1.1 | 6 | 0.015 |  | | | |
| 97820\_at | Galk1 | galactokinase 1 | chr11 | 2.703 | 6 | 0.004 |  | | | |
| 97824\_at | D11Ertd175e | DNA segment, Chr 11, ERATO Doi 175, expressed | chr11 | 1.494 | 6 | 0.006 |  | | | |
| 97828\_at | Siva-pending | Cd27 binding protein (Hindu God of destruction) | chr12 | 1.387 | 6 | 0 |  | | | |
| 97841\_at | 1500016L11Rik | RIKEN cDNA 1500016L11 gene | chr13 | 1.771 | 6 | 0.074 |  | | | |
| 97884\_at | Mrps11 | mitochondrial ribosomal protein S11 | chr7 | 1.959 | 6 | 0.019 |  | | | |
| 97900\_at | Apacd-pending | ATP binding protein associated with cell differentiation | chr1 | 2.307 | 6 | 0.281 |  | | | |
| 97907\_at | Lsm7 | LSM7 homolog, U6 small nuclear RNA associated (S. cerevisiae) | chr10 | 1.945 | 6 | 0.002 |  | | | |
| 97979\_at | Ppp1r7 | protein phosphatase 1, regulatory (inhibitor) subunit 7 | chr1 | 2.977 | 6 | 0.019 |  | | | |
| 98015\_at | Scamp3 | secretory carrier membrane protein 3 | chr3 | 1.112 | 6 | 0.426 |  | | | |
| 98020\_at | Drg1 | developmentally regulated GTP binding protein 1 | --- | 1.284 | 6 | 0.067 |  | | | |
| 98024\_at | Nfyb | nuclear transcription factor-Y beta | chr10 | 1.154 | 6 | 0.065 |  | | | |
| 98039\_at | 2410015M20Rik | RIKEN cDNA 2410015M20 gene | chr17 | 1.43 | 6 | 0.019 |  | | | |
| 98047\_at | 5730410I19Rik | RIKEN cDNA 5730410I19 gene | chr12 | 1.764 | 6 | 0.298 |  | | | |
| 98071\_f\_at | Dck | deoxycytidine kinase | chr5 | 1.717 | 6 | 0.147 |  | | | |
| 98075\_at | G431001I09Rik | RIKEN cDNA G431001I09 gene | chr2 | 3.225 | 6 | 0.001 |  | | | |
| 98076\_at | Erp29 | endoplasmic reticulum protein 29 | chr5 | 1.502 | 6 | 0.052 |  | | | |
| 98077\_at | Snrpd3 | small nuclear ribonucleoprotein D3 | chr10 | 1.794 | 6 | 0.001 |  | | | |
| 98092\_at | Plac8 | placenta-specific 8 | chr5 | 2.981 | 6 | 0.004 |  | | | |
| 98120\_at | Mrpl27 | mitochondrial ribosomal protein L27 | chr11 | 3.683 | 6 | 0.001 |  | | | |
| 98149\_s\_at | 1110033J19Rik | RIKEN cDNA 1110033J19 gene | chr6 | 1.378 | 6 | 0.083 |  | | | |
| 98153\_at | Cct3 | chaperonin subunit 3 (gamma) | chr3 | 1.573 | 6 | 0.02 |  | | | |
| 98155\_r\_at | Pold2 | polymerase (DNA directed), delta 2, regulatory subunit | chr11 | 3.192 | 6 | 0.126 |  | | | |
| 98440\_at | Ltb4dh | leukotriene B4 12-hydroxydehydrogenase | chr4 | 3.855 | 6 | 0 |  | | | |
| 98446\_s\_at | Ephb4 | Eph receptor B4 | chr3 | 2.478 | 6 | 0.056 |  | | | |
| 98459\_at | Shmt1 | serine hydroxymethyl transferase 1 (soluble) | chr11 | 2.747 | 6 | 0.206 |  | | | |
| 98492\_at | Cklfsf7 | chemokine-like factor super family 7 | chr9 | 1.661 | 6 | 0.036 |  | | | |
| 98502\_at | Dusp19 | dual specificity phosphatase 19 | chr2 | 1.305 | 6 | 0.241 |  | | | |
| 98511\_at | Raly | hnRNP-associated with lethal yellow | chr2 | 1.502 | 6 | 0.292 |  | | | |
| 98524\_f\_at | NoneAvailable | --- | --- | 2.603 | 6 | 0.008 |  | | | |
| 98527\_at | Dci | dodecenoyl-Coenzyme A delta isomerase (3,2 trans-enoyl-Coenyme A isomerase) | chr17 | 1.306 | 6 | 0.011 |  | | | |
| 98545\_at | Bcap37 | B-cell receptor-associated protein 37 | chr6 | 1.531 | 6 | 0.121 |  | | | |
| 98605\_at | Wars | tryptophanyl-tRNA synthetase | --- | 1.11 | 6 | 0.2 |  | | | |
| 98610\_at | Mrps28 | mitochondrial ribosomal protein S28 | chr3 | 1.473 | 6 | 0.04 |  | | | |
| 98625\_s\_at | Adh5 | alcohol dehydrogenase 5 (class III), chi polypeptide | chr3 | 1.022 | 6 | 0.171 |  | | | |
| 98626\_at | 1810017G16Rik | RIKEN cDNA 1810017G16 gene | chr8 | 2.261 | 6 | 0.051 |  | | | |
| 98759\_f\_at | Tuba2 | tubulin, alpha 2 | chr13 | 1.313 | 6 | 0.153 |  | | | |
| 98886\_at | 2810410M20Rik | RIKEN cDNA 2810410M20 gene | --- | 1.838 | 6 | 0.128 |  | | | |
| 98904\_at | Mrpl35 | mitochondrial ribosomal protein L35 | chr6 | 2.237 | 6 | 0.002 |  | | | |
| 98912\_at | AI256361 | expressed sequence AI256361 | --- | 1.082 | 6 | 0.267 |  | | | |
| 98914\_at | Asf1a | ASF1 anti-silencing function 1 homolog A (S. cerevisiae) | chr10 | 1.148 | 6 | 0.086 |  | | | |
| 98930\_at | Cope | coatomer protein complex, subunit epsilon | chr8 | 1.116 | 6 | 0.022 |  | | | |
| 98934\_at | 0610007P06Rik | RIKEN cDNA 0610007P06 gene | chr7 | 4.269 | 6 | 0 |  | | | |
| 98938\_at | 1500026D16Rik | RIKEN cDNA 1500026D16 gene | chr19 | 1.602 | 6 | 0.013 |  | | | |
| 98966\_at | Dbt | dihydrolipoamide branched chain transacylase E2 | chr3 | 1.257 | 6 | 0.033 |  | | | |
| 98979\_at | Mnat1 | menage a trois 1 | chr12 | 2.425 | 6 | 0.127 |  | | | |
| 98980\_at | Cd37 | CD37 antigen | chr7 | 1.921 | 6 | 0.274 |  | | | |
| 99053\_at | Icam2 | intercellular adhesion molecule 2 | chr11 | 1.567 | 6 | 0.081 |  | | | |
| 99054\_at | Llglh | lethal giant larvae homolog | chr11 | 1.104 | 6 | 0.582 |  | | | |
| 99098\_at | Fdps | farnesyl diphosphate synthetase | chr10 | 3.309 | 6 | 0.158 |  | | | |
| 99106\_at | Cops6 | COP9 (constitutive photomorphogenic) homolog, subunit 6 (Arabidopsis thaliana) | chr5 | 2.396 | 6 | 0.029 |  | | | |
| 99113\_at | Cops3 | COP9 (constitutive photomorphogenic) homolog, subunit 3 (Arabidopsis thaliana) | chr11 | 1.653 | 6 | 0.136 |  | | | |
| 99126\_at | Xist | inactive X specific transcripts | chrX | 7.746 | 6 | 0.329 |  | | | |
| 99127\_at | Sca10 | spinocerebellar ataxia 10 homolog (human) | chr15 | 1.401 | 6 | 0.155 |  | | | |
| 99128\_at | Atp5o | ATP synthase, H+ transporting, mitochondrial F1 complex, O subunit | chr16 | 1.704 | 6 | 0.021 |  | | | |
| 99129\_at | Clast3-pending | CD40 ligand-activated specific transcript 3 | chr18 | 1.773 | 6 | 0.039 |  | | | |
| 99135\_at | Cdc37 | cell division cycle 37 homolog (S. cerevisiae) | chr9 | 1.028 | 6 | 0.484 |  | | | |
| 99148\_at | Fh1 | fumarate hydratase 1 | chr1 | 2.824 | 6 | 0.009 |  | | | |
| 99149\_at | 2310035M22Rik | RIKEN cDNA 2310035M22 gene | chr3 | 2.829 | 6 | 0.471 |  | | | |
| 99151\_at | 2610002K22Rik | RIKEN cDNA 2610002K22 gene | --- | 3.469 | 6 | 0.004 |  | | | |
| 99164\_at | 2010111E04Rik | RIKEN cDNA 2010111E04 gene | chr3 | 2.906 | 6 | 0.006 |  | | | |
| 99365\_at | Coq3 | coenzyme Q3 homolog, methyltransferase (yeast) | chr4 | 1.105 | 6 | 0.333 |  | | | |
| 99490\_at | 2410044K02Rik | RIKEN cDNA 2410044K02 gene | chr13 | 1.346 | 6 | 0.234 |  | | | |
| 99537\_at | Ruvbl1 | RuvB-like protein 1 | chr1 | 3.012 | 6 | 0.022 |  | | | |
| 99546\_at | Fkbp2 | FK506 binding protein 2 | --- | 2.273 | 6 | 0.001 |  | | | |
| 99566\_at | Tpi | triosephosphate isomerase | chr6 | 1.133 | 6 | 0.048 |  | | | |
| 99578\_at | Top2a | topoisomerase (DNA) II alpha | chr11 | 2.325 | 6 | 0.11 |  | | | |
| 99583\_at | Gstp2 | glutathione S-transferase, pi 2 | chr19 | 1.142 | 6 | 0.003 |  | | | |
| 99594\_at | Mrpl51 | mitochondrial ribosomal protein L51 | chr6 | 2.427 | 6 | 0 |  | | | |
| 99618\_at | 0710008D09Rik | RIKEN cDNA 0710008D09 gene | chr10 | 2.5 | 6 | 0 |  | | | |
| 99632\_at | Mad2l1 | MAD2 (mitotic arrest deficient, homolog)-like 1 (yeast) | chr6 | 1.589 | 6 | 0.018 |  | | | |
| 99651\_at | 2610209M04Rik | RIKEN cDNA 2610209M04 gene | chr6 | 1.176 | 6 | 0.024 |  | | | |
| 99661\_r\_at | Cox7c | cytochrome c oxidase, subunit VIIc | chr11 | 1.254 | 6 | 0.177 |  | | | |
| 99668\_at | Bin1 | bridging integrator 1 | chr18 | 2.256 | 6 | 0.25 |  | | | |
| 99669\_at | Lgals1 | lectin, galactose binding, soluble 1 | chr15 | 3.437 | 6 | 0.079 |  | | | |
| 99856\_r\_at | Catnd2 | catenin delta 2 | chr15 | 1.044 | 6 | 0.805 |  | | | |
| 99924\_at | Tubg1 | tubulin, gamma 1 | chr11 | 1.051 | 6 | 0.121 |  | | | |
| 100348\_at | NoneAvailable | --- | --- | 2.608 | 10 | 0.029 |  | | | |
| 100397\_at | Tyrobp | TYRO protein tyrosine kinase binding protein | chr7 | 3.755 | 10 | 0.004 |  | | | |
| 100414\_s\_at | Mpo | myeloperoxidase | chr11 | 7.927 | 10 | 0.168 |  | | | |
| 100525\_at | NoneAvailable | Mus musculus mRNA similar to HpaII tiny fragments locus 9c (cDNA clone MGC:54707 IMAGE:6337243), complete cds | chr16 | 1.121 | 10 | 0.057 |  | | | |
| 100773\_at | Il12a | interleukin 12a | --- | 2.645 | 10 | 0.007 |  | | | |
| 100895\_at | D16Bwg1547e | DNA segment, Chr 16, Brigham & Women's Genetics 1547 expressed | chr16 | 1.098 | 10 | 0.096 |  | | | |
| 100912\_at | 2410012M04Rik | RIKEN cDNA 2410012M04 gene | chr3 | 1.038 | 10 | 0.237 |  | | | |
| 100968\_at | Cstf3 | cleavage stimulation factor, 3' pre-RNA, subunit 3 | chr2 | 1.516 | 10 | 0.3 |  | | | |
| 101382\_at | Pbx2 | pre B-cell leukemia transcription factor 2 | chr17 | 1.365 | 10 | 0.142 |  | | | |
| 102104\_f\_at | NoneAvailable | --- | chr19 | 1.072 | 10 | 0.038 |  | | | |
| 102397\_at | Cbfa2t3h | core-binding factor, runt domain, alpha subunit 2; translocated to, 3 homolog (human) | --- | 1.889 | 10 | 0.098 |  | | | |
| 102638\_at | Cst7 | cystatin F (leukocystatin) | chr2 | 2.255 | 10 | 0.11 |  | | | |
| 103259\_at | Gfi1 | growth factor independent 1 | --- | 1.14 | 10 | 0.031 |  | | | |
| 103448\_at | S100a8 | S100 calcium binding protein A8 (calgranulin A) | chr3 | 2.6 | 10 | 0.138 |  | | | |
| 103662\_at | Ncf4 | neutrophil cytosolic factor 4 | chr15 | 1.25 | 10 | 0.154 |  | | | |
| 103665\_at | Elovl6 | ELOVL family member 6, elongation of long chain fatty acids (yeast) | chr3 | 1.679 | 10 | 0.206 |  | | | |
| 103964\_at | Esrra | estrogen related receptor, alpha | chr19 | 1.072 | 10 | 0.19 |  | | | |
| 104227\_at | Gpr97 | G protein-coupled receptor 97 | --- | 1.634 | 10 | 0.094 |  | | | |
| 104354\_at | Csf1r | colony stimulating factor 1 receptor | chr18 | 4.153 | 10 | 0.711 |  | | | |
| 104388\_at | Ccl9 | chemokine (C-C motif) ligand 9 | chr11 | 1.346 | 10 | 0.03 |  | | | |
| 104415\_at | 3110052D19Rik | RIKEN cDNA 3110052D19 gene | chr6 | 1.6 | 10 | 0.632 |  | | | |
| 104525\_at | 2810423O19Rik | RIKEN cDNA 2810423O19 gene | chr9 | 1.026 | 10 | 0.438 |  | | | |
| 104592\_i\_at | Mef2c | myocyte enhancer factor 2C | chr13 | 1.227 | 10 | 0.29 |  | | | |
| 104606\_at | Cd52 | CD52 antigen | chr4 | 1.04 | 10 | 0.796 |  | | | |
| 160832\_at | Ldlr | low density lipoprotein receptor | chr9 | 1.302 | 10 | 0.036 |  | | | |
| 92225\_f\_at | Rpo1-2 | RNA polymerase 1-2 | chr2 | 1.349 | 10 | 0.298 |  | | | |
| 93488\_at | Srr | serine racemase | chr11 | 1.111 | 10 | 0.978 |  | | | |
| 93636\_at | Rttn | rotatin | chr18 | 1.145 | 10 | 0.483 |  | | | |
| 94113\_at | NoneAvailable | Mus musculus transcribed sequences | chr12 | 1.211 | 10 | 0.072 |  | | | |
| 94401\_s\_at | Hemgn | hemogen | --- | 1.555 | 10 | 0.069 |  | | | |
| 94713\_at | Myo7a | myosin VIIa | chr7 | 1.664 | 10 | 0.666 |  | | | |
| 95032\_at | Prc1 | protein regulator of cytokinesis 1 | chr7 | 2.137 | 10 | 0.571 |  | | | |
| 95433\_at | Ddx54 | DEAD (Asp-Glu-Ala-Asp) box polypeptide 54 | chr5 | 1.761 | 10 | 0.017 |  | | | |
| 95939\_i\_at | 9830126M18 | hypothetical protein 9830126M18 | chr15 | 1.69 | 10 | 0.448 |  | | | |
| 95940\_f\_at | 9830126M18 | hypothetical protein 9830126M18 | --- | 1.121 | 10 | 0.336 |  | | | |
| 96577\_i\_at | NoneAvailable | --- | chr3 | 1.694 | 10 | 0.033 |  | | | |
| 96784\_at | 2900037I21Rik | RIKEN cDNA 2900037I21 gene | chr9 | 1.467 | 10 | 0.045 |  | | | |
| 96907\_at | Cherp | calcium homeostasis endoplasmic reticulum protein | chr8 | 1.487 | 10 | 0.788 |  | | | |
| 97180\_f\_at | Hbb-y | hemoglobin Y, beta-like embryonic chain | chr7 | 3.55 | 10 | 0.166 |  | | | |
| 97497\_at | Notch1 | Notch gene homolog 1 (Drosophila) | chr2 | 1.19 | 10 | 0.011 |  | | | |
| 98002\_at | Icsbp1 | interferon consensus sequence binding protein 1 | chr8 | 1.28 | 10 | 0.01 |  | | | |
| 98489\_at | Hurp-pending | hepatoma up-regulated protein | chr14 | 2.72 | 10 | 0.369 |  | | | |
| 98991\_at | Smarcad1 | SWI/SNF-related, matrix-associated actin-dependent regulator of chromatin, subfamily a, containing DEAD/H box 1` | chr6 | 1.448 | 10 | 0.918 |  | | | |
| 99030\_at | Il7r | interleukin 7 receptor | chr15 | 2.713 | 10 | 0.276 |  | | | |
| 99058\_at | Hmga2 | high mobility group AT-hook 2 | chr10 | 3.006 | 10 | 0.377 |  | | | |
| 99073\_at | Ccnf | cyclin F | chr17 | 2.395 | 10 | 0.313 |  | | | |
| 99562\_at | Man2b1 | mannosidase 2, alpha B1 | chr8 | 1.3 | 10 | 0.299 |  | | | |
| 100054\_s\_at | D2Wsu81e | DNA segment, Chr 2, Wayne State University 81, expressed | chr2 | 1.237 | 30 | 0.508 |  | | | |
| 100415\_at | NoneAvailable | --- | --- | 3.392 | 30 | 0.259 |  | | | |
| 100425\_at | Syk | spleen tyrosine kinase | chr13 | 1.144 | 30 | 0.369 |  | | | |
| 100468\_g\_at | Lyl1 | lymphoblastomic leukemia | chr8 | 1.71 | 30 | 0.888 |  | | | |
| 101896\_at | Cd1d2 | CD1d2 antigen | --- | 1.04 | 30 | 0.465 |  | | | |
| 102762\_r\_at | Rhag | Rhesus blood group-associated A glycoprotein | chr17 | 1.816 | 30 | 0.541 |  | | | |
| 102993\_at | Ggta1 | glycoprotein galactosyltransferase alpha 1, 3 | chr2 | 2.731 | 30 | 0.244 |  | | | |
| 103340\_at | Rhced | Rhesus blood group CE and D | chr4 | 1.361 | 30 | 0.22 |  | | | |
| 103535\_at | Hbb-y | hemoglobin Y, beta-like embryonic chain | chr7 | 1.259 | 30 | 0.372 |  | | | |
| 104032\_at | NoneAvailable | Mus musculus cDNA clone MGC:37981 IMAGE:5137303, complete cds | chr8 | 1.979 | 30 | 0.227 |  | | | |
| 104093\_at | Lsp1 | lymphocyte specific 1 | chr7 | 1.065 | 30 | 0.463 |  | | | |
| 104577\_at | Mlh1 | mutL homolog 1 (E. coli) | chr9 | 1.197 | 30 | 0.316 |  | | | |
| 160957\_at | D12Ertd7e | DNA segment, Chr 12, ERATO Doi 7, expressed | chr12 | 1.614 | 30 | 0.04 |  | | | |
| 92639\_at | Stk6 | serine/threonine kinase 6 | chr2 | 3.476 | 30 | 0.224 |  | | | |
| 94425\_at | Ly86 | lymphocyte antigen 86 | chr13 | 3.109 | 30 | 0.226 |  | | | |
| 94559\_at | Gtf3a | general transcription factor III A | chr5 | 1.083 | 30 | 0.547 |  | | | |
| 95356\_at | Apoe | apolipoprotein E | chr7 | 1.217 | 30 | 0.222 |  | | | |
| 96553\_at | Gpcr25 | G-protein coupled receptor 25 | chr12 | 1.773 | 30 | 0.264 |  | | | |
| 96911\_at | Gnb2 | guanine nucleotide binding protein, beta 2 | chr5 | 1.137 | 30 | 0.031 |  | | | |
| 98525\_f\_at | Erdr1-pending | erythroid differentiation regulator 1 | --- | 1.497 | 30 | 0.879 |  | | | |
| \* Positive log2 fold changes represent genes expressed higher in FL-HSC; Negative log2 fold changes represent genes expressed higher in adult HSC (fold change=2 is equivalent to log2 fold change=1) | | | | | | | | | | |
|  |  |  |  |  |  |  |  |  |  |  |
