# Supplementary material for: Molecular Signatures of Proliferation and Quiescence in Hematopoietic Stem Cells
Source: PLoS Biol. 2004 Sep 28;2(10):e301. doi: 10.1371/journal.pbio.0020301 (PMC520599; doi:10.1371/journal.pbio.0020301)
Supplement: Table S2 — (666 KB HTML). [file pbio.0020301.st002.html]

|  |  | Proliferation group |  |  |  |  |  |  |  |  |
| Probe Set ID | Gene Symbol | Gene name | Chromosome | Log2 Fold Change (FL-HSC vs Adult HSC)\* | Day of max (TOM) | p-value of ANOVA (time course) |  | | | |
| 100066\_at | Gart | phosphoribosylglycinamide formyltransferase | chr16 | 1.415 | 2 | 0.007 |  | | | |
| 100574\_f\_at | Gpi1 | glucose phosphate isomerase 1 | chr7 | 0.171 | 2 | 0.041 |  | | | |
| 100753\_at | Atp5a1 | ATP synthase, H+ transporting, mitochondrial F1 complex, alpha subunit, isoform 1 | --- | -0.077 | 2 | 0.029 |  | | | |
| 101042\_f\_at | Pep4 | peptidase 4 | --- | 0.494 | 2 | 0.003 |  | | | |
| 101461\_f\_at | Pja1 | praja1, RING-H2 motif containing | chrX | 0.613 | 2 | 0.034 |  | | | |
| 101466\_at | Pigf | phosphatidylinositol glycan, class F | chr17 | 0.537 | 2 | 0.006 |  | | | |
| 101518\_at | 0610009H04Rik | RIKEN cDNA 0610009H04 gene | chr11 | 0.157 | 2 | 0.019 |  | | | |
| 101959\_r\_at | Tfdp1 | transcription factor Dp 1 | chr8 | 1.111 | 2 | 0.004 |  | | | |
| 101984\_at | Atox1 | ATX1 (antioxidant protein 1) homolog 1 (yeast) | chr11 | 0.652 | 2 | 0.025 |  | | | |
| 102124\_f\_at | Cox4i1 | cytochrome c oxidase subunit IV isoform 1 | chr8 | 0.279 | 2 | 0.035 |  | | | |
| 102649\_s\_at | Raet1c | retinoic acid early transcript gamma | --- | 0.219 | 2 | 0.038 |  | | | |
| 103057\_at | Pold1 | polymerase (DNA directed), delta 1, catalytic subunit | chr7 | 3.176 | 2 | 0.034 |  | | | |
| 103377\_at | Lrp2 | low density lipoprotein receptor-related protein 2 | chr2 | 0.013 | 2 | 0.037 |  | | | |
| 103862\_r\_at | D7Wsu128e | DNA segment, Chr 7, Wayne State University 128, expressed | chr7 | -0.099 | 2 | 0.042 |  | | | |
| 103873\_i\_at | 2310015N07Rik | RIKEN cDNA 2310015N07 gene | --- | 0.684 | 2 | 0.022 |  | | | |
| 103874\_r\_at | 2310015N07Rik | RIKEN cDNA 2310015N07 gene | --- | 0.141 | 2 | 0.016 |  | | | |
| 104089\_at | 2810026P18Rik | RIKEN cDNA 2810026P18 gene | chr9 | 0.457 | 2 | 0.023 |  | | | |
| 104303\_i\_at | 1500004O14Rik | RIKEN cDNA 1500004O14 gene | chr2 | 1.473 | 2 | 0.007 |  | | | |
| 104304\_r\_at | 1500004O14Rik | RIKEN cDNA 1500004O14 gene | --- | 0.905 | 2 | 0.011 |  | | | |
| 104305\_at | Rarsl | arginyl-tRNA synthetase-like | chr4 | 2.295 | 2 | 0.005 |  | | | |
| 104343\_f\_at | Pla2g12 | phospholipase A2, group XII | chr3 | 0.319 | 2 | 0.003 |  | | | |
| 104662\_at | Tnfrsf18 | tumor necrosis factor receptor superfamily, member 18 | chr4 | -0.049 | 2 | 0.002 |  | | | |
| 160291\_at | Sec61a1 | Sec61 alpha 1 subunit (S. cerevisiae) | chr6 | -0.097 | 2 | 0.007 |  | | | |
| 160341\_at | Jtv1-pending | JTV1 gene | chr5 | 2.15 | 2 | 0.033 |  | | | |
| 160633\_at | Refbp1 | RNA and export factor binding protein 1 | --- | 0.652 | 2 | 0.028 |  | | | |
| 160803\_at | D0H8S2298E | DNA segment, Human S2298E | chr8 | -0.598 | 2 | 0.002 |  | | | |
| 161386\_f\_at | Ddx1 | DEAD (Asp-Glu-Ala-Asp) box polypeptide 1 | --- | 0.123 | 2 | 0.048 |  | | | |
| 161400\_f\_at | NoneAvailable | --- | --- | 0.17 | 2 | 0.002 |  | | | |
| 161466\_r\_at | Asb3 | ankyrin repeat and SOCS box-containing protein 3 | --- | 0.163 | 2 | 0.046 |  | | | |
| 161635\_f\_at | 1110008H02Rik | RIKEN cDNA 1110008H02 gene | chr1 | 0.113 | 2 | 0.019 |  | | | |
| 161703\_f\_at | NoneAvailable | --- | --- | 0.175 | 2 | 0 |  | | | |
| 162131\_f\_at | NoneAvailable | Mus musculus transcribed sequence | --- | 0.274 | 2 | 0.026 |  | | | |
| 162301\_f\_at | 2310016M24Rik | RIKEN cDNA 2310016M24 gene | chr15 | -0.1 | 2 | 0.011 |  | | | |
| 92633\_at | Ctsz | cathepsin Z | chr2 | 0.866 | 2 | 0.003 |  | | | |
| 92794\_f\_at | Nme1 | expressed in non-metastatic cells 1, protein | chr11 | 2.156 | 2 | 0 |  | | | |
| 92810\_at | Pdk3 | pyruvate dehydrogenase kinase, isoenzyme 3 | chrX | -0.996 | 2 | 0.017 |  | | | |
| 93038\_f\_at | Anxa1 | annexin A1 | chr19 | 0.074 | 2 | 0 |  | | | |
| 93070\_at | Kpnb3 | karyopherin (importin) beta 3 | chr14 | 0.784 | 2 | 0.004 |  | | | |
| 93352\_at | Tgm2 | transglutaminase 2, C polypeptide | --- | -0.153 | 2 | 0.034 |  | | | |
| 93529\_at | 2310047C21Rik | RIKEN cDNA 2310047C21 gene | chr8 | 0.295 | 2 | 0.013 |  | | | |
| 93740\_at | Nsep1 | nuclease sensitive element binding protein 1 | --- | 0.564 | 2 | 0.038 |  | | | |
| 93822\_at | Rpl37a | ribosomal protein L37a | --- | 0.979 | 2 | 0.019 |  | | | |
| 93968\_at | Pdcd5 | programmed cell death 5 | chr7 | 0.125 | 2 | 0.024 |  | | | |
| 94450\_at | D13Wsu123e | DNA segment, Chr 13, Wayne State University 123, expressed | chr13 | 1.019 | 2 | 0.014 |  | | | |
| 95034\_f\_at | Ipo4 | importin 4 | chr14 | -0.595 | 2 | 0.002 |  | | | |
| 95709\_at | D7Wsu86e | DNA segment, Chr 7, Wayne State University 86, expressed | chr7 | -0.199 | 2 | 0.025 |  | | | |
| 95755\_at | Csda | cold shock domain protein A | --- | 0.101 | 2 | 0.02 |  | | | |
| 96042\_at | Sod2 | superoxide dismutase 2, mitochondrial | chr17 | 0.163 | 2 | 0.002 |  | | | |
| 96827\_at | Cad | carbamoyl-phosphate synthetase 2, aspartate transcarbamylase, and dihydroorotase | chr5 | 1.06 | 2 | 0.014 |  | | | |
| 96855\_at | 5730406I15Rik | RIKEN cDNA 5730406I15 gene | --- | 0.755 | 2 | 0.018 |  | | | |
| 97272\_at | Hnrpa1 | heterogeneous nuclear ribonucleoprotein A1 | --- | 0.019 | 2 | 0.041 |  | | | |
| 97496\_f\_at | 6330514M23Rik | RIKEN cDNA 6330514M23 gene | chr7 | -1.202 | 2 | 0.002 |  | | | |
| 98099\_at | Nudt9 | nudix (nucleoside diphosphate linked moiety X)-type motif 9 | --- | 0.494 | 2 | 0.048 |  | | | |
| 98127\_at | Capza2 | capping protein (actin filament) muscle Z-line, alpha 2 | chr6 | -1.156 | 2 | 0.03 |  | | | |
| 98573\_r\_at | Ranbp1 | RAN binding protein 1 | chr16 | 1.404 | 2 | 0.046 |  | | | |
| 99158\_at | Sh3d3 | SH3 domain protein 3 | chr19 | 1.284 | 2 | 0.048 |  | | | |
| 100062\_at | Mcm3 | minichromosome maintenance deficient 3 (S. cerevisiae) | chr1 | 1.792 | 3 | 0.005 |  | | | |
| 100225\_f\_at | Psmc3 | proteasome (prosome, macropain) 26S subunit, ATPase 3 | chr2 | 0.602 | 3 | 0.01 |  | | | |
| 100290\_f\_at | 6230416J20Rik | RIKEN cDNA 6230416J20 gene | chr4 | 0.452 | 3 | 0.022 |  | | | |
| 101001\_at | 5031439A09Rik | RIKEN cDNA 5031439A09 gene | chr3 | -1.852 | 3 | 0.044 |  | | | |
| 101004\_f\_at | Sfrs3 | splicing factor, arginine/serine-rich 3 (SRp20) | --- | 0.867 | 3 | 0.032 |  | | | |
| 101372\_at | Trip13 | thyroid hormone receptor interactor 13 | chr13 | 1.578 | 3 | 0.014 |  | | | |
| 101445\_at | Dnmt1 | DNA methyltransferase (cytosine-5) 1 | chr9 | 0.6 | 3 | 0.041 |  | | | |
| 101589\_at | Hmgn2 | high mobility group nucleosomal binding domain 2 | chr13 | 0.392 | 3 | 0.016 |  | | | |
| 101920\_at | Pole2 | polymerase (DNA directed), epsilon 2 (p59 subunit) | --- | 1.432 | 3 | 0.025 |  | | | |
| 101957\_f\_at | Adprt1 | ADP-ribosyltransferase (NAD+; poly (ADP-ribose) polymerase) 1 | chr1 | 0.266 | 3 | 0.007 |  | | | |
| 101958\_f\_at | Tfdp1 | transcription factor Dp 1 | chr8 | 0.364 | 3 | 0.003 |  | | | |
| 102047\_at | Nmt1 | N-myristoyltransferase 1 | chr11 | 1.098 | 3 | 0.008 |  | | | |
| 102103\_f\_at | NoneAvailable | --- | --- | 1.173 | 3 | 0 |  | | | |
| 102105\_f\_at | Ptgds | prostaglandin D2 synthase (brain) | chr2 | 0.54 | 3 | 0.024 |  | | | |
| 102128\_f\_at | Mrps25 | mitochondrial ribosomal protein S25 | --- | 1.148 | 3 | 0.01 |  | | | |
| 102134\_f\_at | Atp5g2 | ATP synthase, H+ transporting, mitochondrial F0 complex, subunit c (subunit 9), isoform 2 | --- | 0.56 | 3 | 0.011 |  | | | |
| 102280\_at | Pcdh7 | protocadherin 7 | chr5 | 0.597 | 3 | 0.032 |  | | | |
| 102848\_f\_at | 2610524H06Rik | RIKEN cDNA 2610524H06 gene | --- | 0.197 | 3 | 0.026 |  | | | |
| 103064\_at | Chek1 | checkpoint kinase 1 homolog (S. pombe) | chr9 | 1.251 | 3 | 0.01 |  | | | |
| 103094\_at | Serf1 | small EDRK-rich factor 1 | chr13 | 0.583 | 3 | 0.009 |  | | | |
| 103201\_at | Ttk | Ttk protein kinase | chr9 | 2.66 | 3 | 0.018 |  | | | |
| 103203\_f\_at | NoneAvailable | Mus musculus transcribed sequence with moderate similarity to protein ref:NP\_078956.1 (H.sapiens)  hypothetical protein FLJ23311 [Homo sapiens] | chr7 | 2.324 | 3 | 0.019 |  | | | |
| 103204\_r\_at | NoneAvailable | Mus musculus transcribed sequence with moderate similarity to protein ref:NP\_078956.1 (H.sapiens)  hypothetical protein FLJ23311 [Homo sapiens] | chr7 | 1.738 | 3 | 0.048 |  | | | |
| 103207\_at | Pola1 | polymerase (DNA directed), alpha 1 | chrX | 2.502 | 3 | 0.025 |  | | | |
| 103212\_at | BC006933 | cDNA sequence BC006933 | chr12 | 1.151 | 3 | 0.045 |  | | | |
| 103240\_f\_at | Rnase2 | ribonuclease, RNase A family, 2 | --- | 0.537 | 3 | 0.016 |  | | | |
| 103444\_at | E130315B21Rik | RIKEN cDNA E130315B21 gene | chr10 | 1.296 | 3 | 0.016 |  | | | |
| 103797\_at | Cdc7 | cell division cycle 7 (S. cerevisiae) | chr5 | 0.917 | 3 | 0.008 |  | | | |
| 103821\_at | Cdc6 | cell division cycle 6 homolog (S. cerevisiae) | --- | 2.833 | 3 | 0.016 |  | | | |
| 103910\_at | NoneAvailable | --- | chr10 | 0.788 | 3 | 0.045 |  | | | |
| 103944\_at | Rad51l1 | RAD51-like 1 (S. cerevisiae) | chr12 | 0.358 | 3 | 0.006 |  | | | |
| 104058\_at | 1110018J12Rik | RIKEN cDNA 1110018J12 gene | chr17 | 0.83 | 3 | 0.027 |  | | | |
| 104258\_at | Acyp2 | acylphosphatase 2, muscle type | chr11 | -0.071 | 3 | 0.046 |  | | | |
| 104313\_at | Pgm2 | phosphoglucomutase 2 | chr4 | 0.87 | 3 | 0 |  | | | |
| 160069\_at | Gmnn | geminin | chr13 | 2.598 | 3 | 0.009 |  | | | |
| 160377\_at | Tardbp | TAR DNA binding protein | chr4 | 0.603 | 3 | 0.017 |  | | | |
| 160496\_s\_at | Mcm3 | minichromosome maintenance deficient 3 (S. cerevisiae) | chr1 | 1.482 | 3 | 0.021 |  | | | |
| 160518\_at | 1110060F11Rik | RIKEN cDNA 1110060F11 gene | chr4 | 0.42 | 3 | 0.019 |  | | | |
| 160595\_at | 2310042P20Rik | RIKEN cDNA 2310042P20 gene | chr15 | 0.875 | 3 | 0.031 |  | | | |
| 160907\_at | C81234 | expressed sequence C81234 | chrX | 0.285 | 3 | 0.016 |  | | | |
| 160988\_r\_at | D3Ertd330e | DNA segment, Chr 3, ERATO Doi 330, expressed | chr3 | 0.265 | 3 | 0 |  | | | |
| 161038\_at | Prps2 | phosphoribosyl pyrophosphate synthetase 2 | chrX | 0.523 | 3 | 0.031 |  | | | |
| 161122\_f\_at | Ndufab1 | NADH dehydrogenase (ubiquinone) 1, alpha/beta subcomplex, 1 | --- | 1.68 | 3 | 0.003 |  | | | |
| 161134\_at | 2610201A13Rik | RIKEN cDNA 2610201A13 gene | --- | 0.08 | 3 | 0.01 |  | | | |
| 161232\_r\_at | Gpiap1 | GPI-anchored membrane protein 1 | --- | 0.227 | 3 | 0.019 |  | | | |
| 161268\_f\_at | Gcs1 | glucosidase 1 | --- | 0.563 | 3 | 0.036 |  | | | |
| 161492\_i\_at | NoneAvailable | --- | --- | 0.081 | 3 | 0.017 |  | | | |
| 161757\_f\_at | NoneAvailable | Mus musculus transcribed sequence | chr14 | -0.117 | 3 | 0.001 |  | | | |
| 161787\_f\_at | Ris2 | retroviral integration site 2 | chr8 | 0.401 | 3 | 0.018 |  | | | |
| 162009\_f\_at | Msh2 | mutS homolog 2 (E. coli) | --- | 0.28 | 3 | 0.03 |  | | | |
| 162327\_f\_at | Ndufv2 | NADH dehydrogenase (ubiquinone) flavoprotein 2 | --- | 0.242 | 3 | 0.022 |  | | | |
| 92478\_at | Stag1 | stromal antigen 1 | chr9 | 0.82 | 3 | 0.014 |  | | | |
| 92551\_at | Lig1 | ligase I, DNA, ATP-dependent | chr7 | 1.152 | 3 | 0.033 |  | | | |
| 92561\_at | Entpd5 | ectonucleoside triphosphate diphosphohydrolase 5 | chr12 | -0.37 | 3 | 0.036 |  | | | |
| 92593\_at | Osf2-pending | osteoblast specific factor 2 (fasciclin I-like) | chr3 | 1.316 | 3 | 0.006 |  | | | |
| 92647\_at | Rbbp4 | retinoblastoma binding protein 4 | chr4 | -0.165 | 3 | 0.028 |  | | | |
| 92782\_at | Tmpo | thymopoietin | chr10 | 0.653 | 3 | 0 |  | | | |
| 93228\_at | Hells | helicase, lymphoid specific | chr19 | 0.826 | 3 | 0.023 |  | | | |
| 93248\_at | D730042P09Rik | RIKEN cDNA D730042P09 gene | chr9 | 0.282 | 3 | 0.011 |  | | | |
| 93333\_at | Tbca | tubulin cofactor a | chr13 | 0.666 | 3 | 0.002 |  | | | |
| 93439\_f\_at | Pawr | PRKC, apoptosis, WT1, regulator | chr10 | -0.266 | 3 | 0.017 |  | | | |
| 93445\_at | Cd5l | CD5 antigen-like | --- | 1.122 | 3 | 0.013 |  | | | |
| 93459\_s\_at | Fzd4 | frizzled homolog 4 (Drosophila) | chr7 | -2.581 | 3 | 0.006 |  | | | |
| 94078\_at | 1110020P15Rik | RIKEN cDNA 1110020P15 gene | --- | 0.74 | 3 | 0.014 |  | | | |
| 94228\_at | Xpo1 | exportin 1, CRM1 homolog (yeast) | chr11 | 1.337 | 3 | 0.001 |  | | | |
| 94376\_s\_at | Mre11a | meiotic recombination 11 homolog A (S. cerevisiae) | chr9 | 2.127 | 3 | 0.035 |  | | | |
| 94534\_at | Idh3a | isocitrate dehydrogenase 3 (NAD+) alpha | chr9 | 0.587 | 3 | 0.023 |  | | | |
| 94788\_f\_at | Tubb5 | tubulin, beta 5 | chr17 | 1.367 | 3 | 0.026 |  | | | |
| 94907\_f\_at | 1110001J03Rik | RIKEN cDNA 1110001J03 gene | chr6 | 1.71 | 3 | 0.003 |  | | | |
| 95063\_at | 2310021G01Rik | RIKEN cDNA 2310021G01 gene | chr2 | 2.4 | 3 | 0.041 |  | | | |
| 95084\_f\_at | Grhpr | glyoxylate reductase/hydroxypyruvate reductase | chr4 | 2.569 | 3 | 0.006 |  | | | |
| 95118\_r\_at | Kif22 | kinesin family member 22 | chr7 | 0.237 | 3 | 0.034 |  | | | |
| 95131\_f\_at | Ndufb2 | NADH dehydrogenase (ubiquinone) 1 beta subcomplex, 2 | chr2 | 1.121 | 3 | 0.003 |  | | | |
| 95292\_at | Itga4 | integrin alpha 4 | chr2 | 1.618 | 3 | 0.02 |  | | | |
| 95456\_r\_at | Shfdg1 | split hand/foot deleted gene 1 | chr6 | 1.322 | 3 | 0.026 |  | | | |
| 95462\_at | Bzw2 | basic leucine zipper and W2 domains 2 | chr12 | 1.226 | 3 | 0.049 |  | | | |
| 95527\_at | Chaf1a | chromatin assembly factor 1, subunit A (p150) | chr17 | 1.001 | 3 | 0 |  | | | |
| 95611\_at | Lpl | lipoprotein lipase | chr8 | -0.268 | 3 | 0.028 |  | | | |
| 95612\_at | Rfc5 | replication factor C (activator 1) 5 | --- | 2.137 | 3 | 0.009 |  | | | |
| 95703\_at | Uble1a | ubiquitin-like 1 (sentrin) activating enzyme E1A | --- | 0.674 | 3 | 0.022 |  | | | |
| 95732\_at | 1110005L13Rik | RIKEN cDNA 1110005L13 gene | chr10 | 2.644 | 3 | 0.028 |  | | | |
| 95927\_f\_at | 2610201A13Rik | RIKEN cDNA 2610201A13 gene | chr11 | 1.536 | 3 | 0.02 |  | | | |
| 95967\_at | NoneAvailable | Mus musculus transcribed sequences | chr13 | -0.136 | 3 | 0.035 |  | | | |
| 96156\_at | 1110008H02Rik | RIKEN cDNA 1110008H02 gene | chr1 | 0.334 | 3 | 0.001 |  | | | |
| 96280\_at | Ndufa2 | NADH dehydrogenase (ubiquinone) 1 alpha subcomplex, 2 | chr18 | 0.957 | 3 | 0.002 |  | | | |
| 96625\_at | D630024B06Rik | RIKEN cDNA D630024B06 gene | chr14 | 1.795 | 3 | 0.01 |  | | | |
| 96629\_at | D7Rp2e | DNA segment, Chr 7, Roswell Park 2 complex, expressed | --- | 0.862 | 3 | 0.034 |  | | | |
| 96686\_i\_at | 2010100O12Rik | RIKEN cDNA 2010100O12 gene | chr2 | 1.279 | 3 | 0.005 |  | | | |
| 96687\_f\_at | 2010100O12Rik | RIKEN cDNA 2010100O12 gene | chr2 | 1.093 | 3 | 0 |  | | | |
| 96710\_at | C530002L11Rik | RIKEN cDNA C530002L11 gene | chr11 | 0.039 | 3 | 0.007 |  | | | |
| 96858\_at | Pdcd8 | programmed cell death 8 | chrX | 0.932 | 3 | 0.013 |  | | | |
| 96891\_at | Anp32b | acidic nuclear phosphoprotein 32 family, member B | chr4 | 0.977 | 3 | 0.024 |  | | | |
| 96916\_at | Mrpl33 | mitochondrial ribosomal protein L33 | chr5 | -0.061 | 3 | 0.009 |  | | | |
| 97095\_at | Bub1 | budding uninhibited by benzimidazoles 1 homolog (S. cerevisiae) | --- | 3.241 | 3 | 0.008 |  | | | |
| 97358\_at | Lphn1 | latrophilin 1 | chr8 | 0.169 | 3 | 0.021 |  | | | |
| 97393\_at | Vrk1 | vaccinia related kinase 1 | chr12 | 1.381 | 3 | 0.035 |  | | | |
| 97411\_at | Ect2 | ect2 oncogene | chr3 | 2.597 | 3 | 0.019 |  | | | |
| 97492\_at | 0610040B21Rik | RIKEN cDNA 0610040B21 gene | chr4 | 0.189 | 3 | 0.032 |  | | | |
| 97909\_at | Stmn1 | stathmin 1 | chr4 | 0.912 | 3 | 0.004 |  | | | |
| 98072\_r\_at | Dck | deoxycytidine kinase | chr5 | 0.4 | 3 | 0.018 |  | | | |
| 98140\_at | Cdh1 | cadherin 1 | --- | 0.145 | 3 | 0.017 |  | | | |
| 98550\_at | Set | SET translocation | chr1 | 1.359 | 3 | 0.045 |  | | | |
| 98618\_at | Dtymk | deoxythymidylate kinase | chr1 | 1.551 | 3 | 0.004 |  | | | |
| 98922\_at | Itm1 | intergral membrane protein 1 | chr9 | -0.602 | 3 | 0.01 |  | | | |
| 98929\_at | 1110018B13Rik | RIKEN cDNA 1110018B13 gene | chr13 | 1.14 | 3 | 0.032 |  | | | |
| 98956\_at | Tram1 | translocating chain-associating membrane protein 1 | chr1 | -0.019 | 3 | 0.049 |  | | | |
| 98993\_at | Ppp2r5c | protein phosphatase 2, regulatory subunit B (B56), gamma isoform | chr12 | -0.394 | 3 | 0.047 |  | | | |
| 98999\_at | Adsl | adenylosuccinate lyase | chr15 | 2.382 | 3 | 0.036 |  | | | |
| 99111\_at | Skd3 | suppressor of K+ transport defect 3 | chr7 | -0.206 | 3 | 0.017 |  | | | |
| 99119\_at | Cfl1 | cofilin 1, non-muscle | chr19 | 0.325 | 3 | 0.012 |  | | | |
| 99439\_at | Mas1 | MAS1 oncogene | chr17 | 0.063 | 3 | 0.048 |  | | | |
| 99457\_at | Mki67 | antigen identified by monoclonal antibody Ki 67 | chr7 | 2.708 | 3 | 0.002 |  | | | |
| 99475\_at | Socs2 | suppressor of cytokine signaling 2 | chr10 | -3.55 | 3 | 0.012 |  | | | |
| 99513\_at | Pla2g4a | phospholipase A2, group IVA (cytosolic, calcium-dependent) | chr1 | 0.589 | 3 | 0.032 |  | | | |
| 99581\_at | Hint | histidine triad nucleotide binding protein | --- | 1.503 | 3 | 0.038 |  | | | |
| 99631\_f\_at | Cox6a1 | cytochrome c oxidase, subunit VI a, polypeptide 1 | chr5 | 0.75 | 3 | 0.004 |  | | | |
| 99658\_f\_at | 1110025H10Rik | RIKEN cDNA 1110025H10 gene | chr17 | 0.702 | 3 | 0.043 |  | | | |
| 99660\_f\_at | Cox7c | cytochrome c oxidase, subunit VIIc | chr11 | 0.349 | 3 | 0.021 |  | | | |
| 99777\_s\_at | 6230416J20Rik | RIKEN cDNA 6230416J20 gene | chr4 | 0.516 | 3 | 0.01 |  | | | |
| 100039\_at | Tmem4 | transmembrane protein 4 | chr10 | 1.81 | 6 | 0.003 |  | | | |
| 100057\_at | 2510027N19Rik | RIKEN cDNA 2510027N19 gene | chr7 | 3.001 | 6 | 0 |  | | | |
| 100059\_at | Cyba | cytochrome b-245, alpha polypeptide | chr8 | 1.77 | 6 | 0.038 |  | | | |
| 100073\_at | 2510005D08Rik | RIKEN cDNA 2510005D08 gene | chr14 | 2.121 | 6 | 0.012 |  | | | |
| 100079\_at | Ndufb9 | NADH dehydrogenase (ubiquinone) 1 beta subcomplex, 9 | chr15 | 1.058 | 6 | 0.013 |  | | | |
| 100089\_at | Ppic | peptidylprolyl isomerase C | chr18 | -0.435 | 6 | 0.039 |  | | | |
| 100116\_at | 2810417H13Rik | RIKEN cDNA 2810417H13 gene | chr1 | 2.111 | 6 | 0.026 |  | | | |
| 100128\_at | Cdc2a | cell division cycle 2 homolog A (S. pombe) | --- | 4.133 | 6 | 0.002 |  | | | |
| 100156\_at | Mcm5 | minichromosome maintenance deficient 5, cell division cycle 46 (S. cerevisiae) | chr8 | 5.137 | 6 | 0.012 |  | | | |
| 100331\_g\_at | Prdx2 | peroxiredoxin 2 | chr1 | 1.095 | 6 | 0.001 |  | | | |
| 100400\_at | 4921531G14Rik | RIKEN cDNA 4921531G14 gene | chr15 | 0.81 | 6 | 0.045 |  | | | |
| 100459\_at | Rad50 | RAD50 homolog (S. cerevisiae) | --- | 2.337 | 6 | 0.009 |  | | | |
| 100512\_at | Uchl5 | ubiquitin carboxyl-terminal esterase L5 | chr1 | 1.055 | 6 | 0.006 |  | | | |
| 100527\_at | D11Ertd99e | DNA segment, Chr 11, ERATO Doi 99, expressed | chr11 | 2.559 | 6 | 0.038 |  | | | |
| 100539\_at | 2410041A17Rik | RIKEN cDNA 2410041A17 gene | chr4 | 0.756 | 6 | 0.007 |  | | | |
| 100543\_s\_at | Brd7 | bromodomain containing 7 | chr8 | 1.094 | 6 | 0.003 |  | | | |
| 100550\_f\_at | Cox6c | cytochrome c oxidase, subunit VIc | chr15 | 0.466 | 6 | 0 |  | | | |
| 100568\_at | Abce1 | ATP-binding cassette, sub-family E (OABP), member 1 | --- | 1.485 | 6 | 0.011 |  | | | |
| 100569\_at | Anxa2 | annexin A2 | chr9 | -0.123 | 6 | 0.003 |  | | | |
| 100576\_at | Pafah1b3 | platelet-activating factor acetylhydrolase, isoform 1b, alpha1 subunit | --- | 1.257 | 6 | 0.004 |  | | | |
| 100577\_at | Snrpd1 | small nuclear ribonucleoprotein D1 | chr18 | 1.295 | 6 | 0 |  | | | |
| 100588\_at | Psme2 | proteasome (prosome, macropain) 28 subunit, beta | chr14 | 0.648 | 6 | 0 |  | | | |
| 100592\_at | Ghitm | growth hormone inducible transmembrane protein | chr14 | 1.179 | 6 | 0.024 |  | | | |
| 100612\_at | Rrm1 | ribonucleotide reductase M1 | --- | 1.929 | 6 | 0.045 |  | | | |
| 100618\_f\_at | Slc25a5 | solute carrier family 25 (mitochondrial carrier; adenine nucleotide translocator), member 5 | --- | 1.078 | 6 | 0.012 |  | | | |
| 100628\_at | Ndufc1 | NADH dehydrogenase (ubiquinone) 1, subcomplex unknown, 1 | --- | 1.336 | 6 | 0.006 |  | | | |
| 100633\_at | 2810484M10Rik | RIKEN cDNA 2810484M10 gene | --- | -0.323 | 6 | 0.045 |  | | | |
| 100733\_at | Psma2 | proteasome (prosome, macropain) subunit, alpha type 2 | chr13 | 1.64 | 6 | 0.006 |  | | | |
| 100917\_at | NoneAvailable | --- | chr18 | 1.316 | 6 | 0.037 |  | | | |
| 100990\_g\_at | Itgb1bp1 | integrin beta 1 binding protein 1 | --- | 0.369 | 6 | 0.031 |  | | | |
| 101061\_at | Ssr2 | signal sequence receptor, beta | chr3 | 1.296 | 6 | 0.034 |  | | | |
| 101065\_at | Pcna | proliferating cell nuclear antigen | chr19 | 0.251 | 6 | 0.008 |  | | | |
| 101067\_at | 2010005E08Rik | RIKEN cDNA 2010005E08 gene | chr3 | 0.448 | 6 | 0.01 |  | | | |
| 101096\_s\_at | Hs1bp1 | HS1 binding protein | chr2 | 1.123 | 6 | 0.018 |  | | | |
| 101097\_at | BC028768 | cDNA sequence BC028768 | chr19 | 0.818 | 6 | 0.019 |  | | | |
| 101105\_at | Banf1 | barrier to autointegration factor 1 | chr19 | 2.053 | 6 | 0.002 |  | | | |
| 101207\_at | Ppia | peptidylprolyl isomerase A | chr11 | 0.267 | 6 | 0.011 |  | | | |
| 101254\_at | Ran | RAN, member RAS oncogene family | chr2 | 1.149 | 6 | 0.002 |  | | | |
| 101380\_at | Nudt14 | nudix (nucleoside diphosphate linked moiety X)-type motif 14 | chr12 | 0.228 | 6 | 0.033 |  | | | |
| 101407\_at | Frda | Friedreich ataxia | chr19 | 1.288 | 6 | 0.027 |  | | | |
| 101421\_at | Rnf5 | ring finger protein 5 | chr17 | 1.067 | 6 | 0.044 |  | | | |
| 101440\_at | 1110033L15Rik | RIKEN cDNA 1110033L15 gene | chr2 | 0.499 | 6 | 0.027 |  | | | |
| 101486\_at | Psmb10 | proteasome (prosome, macropain) subunit, beta type 10 | chr8 | 1.623 | 6 | 0.012 |  | | | |
| 101506\_at | Snrpa1 | small nuclear ribonucleoprotein polypeptide A' | chr7 | 1.197 | 6 | 0.003 |  | | | |
| 101521\_at | Birc5 | baculoviral IAP repeat-containing 5 | chr11 | 2.475 | 6 | 0.01 |  | | | |
| 101525\_at | Ndufb10 | NADH dehydrogenase (ubiquinone) 1 beta subcomplex, 10 | chr17 | 0.565 | 6 | 0.008 |  | | | |
| 101558\_s\_at | Psmb5 | proteasome (prosome, macropain) subunit, beta type 5 | chr14 | 1.051 | 6 | 0.018 |  | | | |
| 101562\_at | Hsp70-4 | heat shock protein 4 | chr2 | 2.149 | 6 | 0.047 |  | | | |
| 101579\_at | Srp9 | signal recognition particle 9 | chr1 | 0.56 | 6 | 0.028 |  | | | |
| 101580\_at | Cox7b | cytochrome c oxidase subunit VIIb | chr1 | 1.791 | 6 | 0 |  | | | |
| 101634\_at | Npm1 | nucleophosmin 1 | chr11 | 0.582 | 6 | 0.008 |  | | | |
| 101680\_at | Rpl27a | ribosomal protein L27a | chr13 | -0.081 | 6 | 0 |  | | | |
| 101741\_at | NoneAvailable | --- | chr11 | 0.952 | 6 | 0.004 |  | | | |
| 101866\_at | Arfrp1 | ADP-ribosylation factor related protein 1 | chr2 | 0.898 | 6 | 0.011 |  | | | |
| 101890\_f\_at | Dnajc2 | DnaJ (Hsp40) homolog, subfamily C, member 2 | chr5 | 1.708 | 6 | 0.027 |  | | | |
| 101954\_at | H2afz | H2A histone family, member Z | chr12 | 0.014 | 6 | 0.023 |  | | | |
| 101964\_at | Tkt | transketolase | chr14 | 1.087 | 6 | 0.001 |  | | | |
| 101992\_at | Psmb6 | proteasome (prosome, macropain) subunit, beta type 6 | chr11 | 1.47 | 6 | 0.016 |  | | | |
| 102001\_at | Rrm2 | ribonucleotide reductase M2 | chr12 | 1.74 | 6 | 0.02 |  | | | |
| 102019\_at | Mrpl13 | mitochondrial ribosomal protein L13 | chr15 | 1.841 | 6 | 0.001 |  | | | |
| 102022\_at | 1110007A04Rik | RIKEN cDNA 1110007A04 gene | --- | 1.16 | 6 | 0.009 |  | | | |
| 102126\_at | Rps12 | ribosomal protein S12 | chr10 | 0.076 | 6 | 0.03 |  | | | |
| 102194\_at | 2810432D09Rik | RIKEN cDNA 2810432D09 gene | chr4 | 0.91 | 6 | 0.031 |  | | | |
| 102197\_at | Nucb2 | nucleobindin 2 | chr7 | 0.931 | 6 | 0.019 |  | | | |
| 102409\_at | Lsm8 | LSM8 homolog, U6 small nuclear RNA associated (S. cerevisiae) | chr6 | 1.406 | 6 | 0.001 |  | | | |
| 102412\_at | AW541137 | expressed sequence AW541137 | chr10 | 2.049 | 6 | 0.032 |  | | | |
| 102631\_at | Blm | Bloom syndrome homolog (human) | chr7 | 1.408 | 6 | 0.003 |  | | | |
| 102791\_at | Psmb8 | proteosome (prosome, macropain) subunit, beta type 8 (large multifunctional protease 7) | chr17 | -0.163 | 6 | 0.016 |  | | | |
| 102821\_s\_at | Rasl2-9 | RAS-like, family 2, locus 9 | chr2 | 1.55 | 6 | 0 |  | | | |
| 102838\_at | Sell | selectin, lymphocyte | chr1 | 0.254 | 6 | 0.004 |  | | | |
| 102853\_at | Cspg6 | chondroitin sulfate proteoglycan 6 | chr19 | 1.265 | 6 | 0.037 |  | | | |
| 102970\_at | Psmc3ip | proteasome (prosome, macropain) 26S subunit, ATPase 3, interacting protein | chr11 | 0.86 | 6 | 0.028 |  | | | |
| 103038\_at | Guca1a | guanylate cyclase activator 1a (retina) | chr17 | -0.105 | 6 | 0.007 |  | | | |
| 103089\_at | Cd48 | CD48 antigen | chr1 | 7.012 | 6 | 0 |  | | | |
| 103319\_at | Psmd10 | proteasome (prosome, macropain) 26S subunit, non-ATPase, 10 | chrX | 0.731 | 6 | 0.01 |  | | | |
| 103334\_at | Crcp | calcitonin gene-related peptide-receptor component protein | chr5 | 1.088 | 6 | 0.015 |  | | | |
| 103335\_at | Lgals9 | lectin, galactose binding, soluble 9 | chr11 | 2.142 | 6 | 0.019 |  | | | |
| 103418\_at | Rfc4 | replication factor C (activator 1) 4 | chr16 | 2.864 | 6 | 0.041 |  | | | |
| 103442\_at | BC003479 | cDNA sequence BC003479 | chr11 | 1.965 | 6 | 0.007 |  | | | |
| 103468\_at | Mns1 | meiosis-specific nuclear structural protein 1 | chr9 | 3.961 | 6 | 0.026 |  | | | |
| 103534\_at | NoneAvailable | --- | chr7 | 2.111 | 6 | 0.006 |  | | | |
| 103581\_at | Cte1 | cytosolic acyl-CoA thioesterase 1 | chr12 | 0.169 | 6 | 0.01 |  | | | |
| 103598\_at | Dhx9 | DEAH (Asp-Glu-Ala-His) box polypeptide 9 | chr1 | 0.862 | 6 | 0.032 |  | | | |
| 103605\_g\_at | Rgs19 | regulator of G-protein signaling 19 | chr2 | 0.421 | 6 | 0.01 |  | | | |
| 103608\_at | 2810431B21Rik | RIKEN cDNA 2810431B21 gene | chr13 | 0.933 | 6 | 0.047 |  | | | |
| 103619\_at | 1810044O22Rik | RIKEN cDNA 1810044O22 gene | chr8 | 1.634 | 6 | 0.009 |  | | | |
| 103654\_at | Nsbp1 | nucleosome binding protein 1 | chrX | 0.032 | 6 | 0.02 |  | | | |
| 103671\_at | Htatip2 | HIV-1 tat interactive protein 2, homolog (human) | chr7 | 0.743 | 6 | 0.01 |  | | | |
| 103683\_at | Dhodh | dihydroorotate dehydrogenase | chr8 | 1.175 | 6 | 0.041 |  | | | |
| 103805\_at | Nbn | nibrin | chr4 | 0.182 | 6 | 0 |  | | | |
| 103881\_at | 1110013G13Rik | RIKEN cDNA 1110013G13 gene | chr3 | 1.342 | 6 | 0.006 |  | | | |
| 103885\_at | 1500019O16Rik | RIKEN cDNA 1500019O16 gene | chr7 | 5.88 | 6 | 0.011 |  | | | |
| 103939\_at | 2610509I15Rik | RIKEN cDNA 2610509I15 gene | --- | 0.597 | 6 | 0.008 |  | | | |
| 104042\_at | Slc35b1 | solute carrier family 35, member B1 | chr17 | 1.214 | 6 | 0.037 |  | | | |
| 104044\_at | 1300006N24Rik | RIKEN cDNA 1300006N24 gene | chr9 | 1.315 | 6 | 0.044 |  | | | |
| 104057\_at | Grpel1 | GrpE-like 1, mitochondrial | chr5 | 1.548 | 6 | 0.026 |  | | | |
| 104077\_at | 1110049G11Rik | RIKEN cDNA 1110049G11 gene | chrX | 1.238 | 6 | 0.01 |  | | | |
| 104078\_g\_at | 1110049G11Rik | RIKEN cDNA 1110049G11 gene | chrX | 3.329 | 6 | 0.001 |  | | | |
| 104080\_at | Pdap1 | PDGFA associated protein 1 | chr5 | 2.918 | 6 | 0.01 |  | | | |
| 104135\_at | Arl3 | ADP-ribosylation factor-like 3 | chr19 | 0.196 | 6 | 0.008 |  | | | |
| 104145\_at | Tcof1 | Treacher Collins Franceschetti syndrome 1, homolog | chr18 | 1.073 | 6 | 0.043 |  | | | |
| 104147\_at | Nans | N-acetylneuraminic acid synthase (sialic acid synthase) | chr4 | 1.196 | 6 | 0.022 |  | | | |
| 104212\_at | Lrpprc | leucine-rich PPR-motif containing | --- | 0.574 | 6 | 0.023 |  | | | |
| 104234\_at | Mrps25 | mitochondrial ribosomal protein S25 | chr6 | 1.243 | 6 | 0.029 |  | | | |
| 104237\_at | 2700061N24Rik | RIKEN cDNA 2700061N24 gene | chr13 | 1.881 | 6 | 0.016 |  | | | |
| 104248\_at | 0610038P07Rik | RIKEN cDNA 0610038P07 gene | chr3 | 0.168 | 6 | 0.046 |  | | | |
| 104297\_at | Ipo11 | importin 11 | chr13 | 1.447 | 6 | 0.02 |  | | | |
| 104301\_at | 2410018G20Rik | RIKEN cDNA 2410018G20 gene | chr16 | 1.631 | 6 | 0.002 |  | | | |
| 104322\_at | Ckap2 | cytoskeleton associated protein 2 | chr8 | 1.598 | 6 | 0.003 |  | | | |
| 104356\_at | 4921516M08Rik | RIKEN cDNA 4921516M08 gene | --- | 1.385 | 6 | 0 |  | | | |
| 104380\_at | Slc35a1 | solute carrier family 35 (CMP-sialic acid transporter), member 1 | chr4 | 0.449 | 6 | 0.002 |  | | | |
| 104390\_at | Anp32a | acidic (leucine-rich) nuclear phosphoprotein 32 family, member A | --- | 1.176 | 6 | 0.045 |  | | | |
| 104423\_at | 2810047L02Rik | RIKEN cDNA 2810047L02 gene | chr1 | 3.018 | 6 | 0.009 |  | | | |
| 104476\_at | Rbl1 | retinoblastoma-like 1 (p107) | chr2 | 1.983 | 6 | 0.011 |  | | | |
| 104541\_at | Prtn3 | proteinase 3 | chr10 | 1.916 | 6 | 0.042 |  | | | |
| 104567\_at | Mrpl46 | mitochondrial ribosomal protein L46 | chr7 | 1.995 | 6 | 0.031 |  | | | |
| 104733\_at | Cetn2 | centrin 2 | chrX | 0.012 | 6 | 0.015 |  | | | |
| 104738\_at | Zrf2 | zuotin related factor 2 | chr6 | 1.861 | 6 | 0.008 |  | | | |
| 104762\_r\_at | 1500015J03Rik | RIKEN cDNA 1500015J03 gene | chr2 | 1.459 | 6 | 0.042 |  | | | |
| 104766\_at | Nola1 | nucleolar protein family A, member 1 (H/ACA small nucleolar RNPs) | --- | 1.457 | 6 | 0.002 |  | | | |
| 160076\_at | Mtx2 | metaxin 2 | chr2 | 1.76 | 6 | 0.035 |  | | | |
| 160107\_at | Hprt | hypoxanthine guanine phosphoribosyl transferase | chrX | 0.226 | 6 | 0.028 |  | | | |
| 160125\_at | D11Ertd497e | DNA segment, Chr 11, ERATO Doi 497, expressed | --- | 0.876 | 6 | 0.007 |  | | | |
| 160126\_at | Map2k1ip1 | mitogen-activated protein kinase kinase 1 interacting protein 1 | chr3 | 0.372 | 6 | 0.049 |  | | | |
| 160135\_at | D16Ertd502e | DNA segment, Chr 16, ERATO Doi 502, expressed | chr16 | 1.284 | 6 | 0.007 |  | | | |
| 160152\_at | Psmc1 | protease (prosome, macropain) 26S subunit, ATPase 1 | chr16 | 1.442 | 6 | 0.02 |  | | | |
| 160165\_at | Ubc-rs2 | ubiquitin C, related sequence 2 | chr7 | 0.51 | 6 | 0.046 |  | | | |
| 160176\_at | Hirip5 | histone cell cycle regulation defective interacting protein 5 | chr15 | 1.669 | 6 | 0 |  | | | |
| 160193\_at | 5730551F12Rik | RIKEN cDNA 5730551F12 gene | chr10 | 0.6 | 6 | 0.002 |  | | | |
| 160203\_at | 5330419I01Rik | RIKEN cDNA 5330419I01 gene | chr1 | 0.873 | 6 | 0.017 |  | | | |
| 160214\_at | 1700003F10Rik | RIKEN cDNA 1700003F10 gene | chr6 | 0.072 | 6 | 0.039 |  | | | |
| 160218\_at | 2310056P07Rik | RIKEN cDNA 2310056P07 gene | chr16 | 0.971 | 6 | 0.002 |  | | | |
| 160225\_at | Gtf2b | general transcription factor IIB | chr3 | 0.477 | 6 | 0.012 |  | | | |
| 160235\_at | 5033425B17Rik | RIKEN cDNA 5033425B17 gene | chr7 | 0.992 | 6 | 0.006 |  | | | |
| 160247\_at | Ube2v2 | ubiquitin-conjugating enzyme E2 variant 2 | chr15 | 1.044 | 6 | 0.003 |  | | | |
| 160256\_at | 1110020J08Rik | RIKEN cDNA 1110020J08 gene | --- | 0.939 | 6 | 0.001 |  | | | |
| 160262\_at | Mtch2 | mitochondrial carrier homolog 2 (C. elegans) | chr2 | 0.99 | 6 | 0.01 |  | | | |
| 160266\_r\_at | 1110064N10Rik | RIKEN cDNA 1110064N10 gene | chr15 | 0.96 | 6 | 0.041 |  | | | |
| 160293\_at | 2700038L12Rik | RIKEN cDNA 2700038L12 gene | chr9 | 1.194 | 6 | 0.048 |  | | | |
| 160297\_at | MGC36453 | hypothetical protein LOC381045 | chr14 | 2.932 | 6 | 0.003 |  | | | |
| 160299\_at | Rwdd1 | RWD domain containing 1 | chr10 | 1.08 | 6 | 0.034 |  | | | |
| 160314\_at | 2010317E03Rik | RIKEN cDNA 2010317E03 gene | chr10 | 0.092 | 6 | 0.007 |  | | | |
| 160324\_at | Rpa3 | replication protein A3 | chr1 | 1.959 | 6 | 0.009 |  | | | |
| 160344\_at | Npc2 | Niemann Pick type C2 | chr12 | 0.888 | 6 | 0.036 |  | | | |
| 160360\_at | Sep15-pending | selenoprotein | chr3 | -0.129 | 6 | 0.012 |  | | | |
| 160412\_at | Pdcd2 | programmed cell death 2 | chr17 | 0.662 | 6 | 0.048 |  | | | |
| 160416\_at | Fkbp3 | FK506 binding protein 3 | chr12 | 0.512 | 6 | 0.006 |  | | | |
| 160426\_at | Rpo1-1 | RNA polymerase 1-1 | chr17 | 0.705 | 6 | 0.029 |  | | | |
| 160431\_at | Mrpl12 | mitochondrial ribosomal protein L12 | chr11 | 2.077 | 6 | 0.009 |  | | | |
| 160444\_at | 2010319C14Rik | RIKEN cDNA 2010319C14 gene | chr16 | 0.548 | 6 | 0.042 |  | | | |
| 160456\_at | 1100001J08Rik | RIKEN cDNA 1100001J08 gene | --- | 0.642 | 6 | 0.011 |  | | | |
| 160485\_r\_at | Ywhae | tyrosine 3-monooxygenase/tryptophan 5-monooxygenase activation protein, epsilon polypeptide | --- | 0.185 | 6 | 0.012 |  | | | |
| 160503\_at | Fbl | fibrillarin | chr17 | 1.886 | 6 | 0 |  | | | |
| 160531\_at | Grcc2f | gene rich cluster, C2f gene | chr6 | 0.73 | 6 | 0.036 |  | | | |
| 160536\_at | Hras1 | Harvey rat sarcoma virus oncogene 1 | --- | 0.429 | 6 | 0.031 |  | | | |
| 160538\_at | Cdk4 | cyclin-dependent kinase 4 | chr10 | 2.296 | 6 | 0.032 |  | | | |
| 160543\_at | Snx3 | sorting nexin 3 | chr10 | 0.395 | 6 | 0.023 |  | | | |
| 160550\_i\_at | Magoh | mago-nashi homolog, proliferation-associated (Drosophila) | chr4 | 0.676 | 6 | 0.016 |  | | | |
| 160551\_at | Vdac3 | voltage-dependent anion channel 3 | chr13 | 0.534 | 6 | 0.006 |  | | | |
| 160556\_at | 1810020G14Rik | RIKEN cDNA 1810020G14 gene | chr12 | 0.101 | 6 | 0.023 |  | | | |
| 160569\_at | 2310008M10Rik | RIKEN cDNA 2310008M10 gene | chr3 | 1.341 | 6 | 0.006 |  | | | |
| 160585\_at | 2810470K21Rik | RIKEN cDNA 2810470K21 gene | --- | 2.814 | 6 | 0.011 |  | | | |
| 160621\_at | Mrps22 | mitochondrial ribosomal protein S22 | chr9 | 0.891 | 6 | 0.017 |  | | | |
| 160659\_at | 2310057G13Rik | RIKEN cDNA 2310057G13 gene | chr10 | 0.89 | 6 | 0 |  | | | |
| 160709\_at | 1110001A16Rik | RIKEN cDNA 1110001A16 gene | chr17 | 0.843 | 6 | 0 |  | | | |
| 160711\_at | Decr1 | 2,4-dienoyl CoA reductase 1, mitochondrial | chr4 | 0.759 | 6 | 0.006 |  | | | |
| 160723\_at | 1500001M20Rik | RIKEN cDNA 1500001M20 gene | chr6 | 1.3 | 6 | 0.044 |  | | | |
| 160743\_at | Pole3 | polymerase (DNA directed), epsilon 3 (p17 subunit) | chr4 | 0.238 | 6 | 0.008 |  | | | |
| 160805\_s\_at | Mpdu1 | mannose-P-dolichol utilization defect 1 | chr11 | 0.143 | 6 | 0.011 |  | | | |
| 160844\_at | Pts | 6-pyruvoyl-tetrahydropterin synthase | chr9 | 0.175 | 6 | 0.049 |  | | | |
| 160856\_at | Ubl4 | ubiquitin-like 4 | --- | 0.36 | 6 | 0.007 |  | | | |
| 160872\_f\_at | 2310008H09Rik | RIKEN cDNA 2310008H09 gene | chr7 | 0.592 | 6 | 0.016 |  | | | |
| 160876\_at | Bcap29 | B-cell receptor-associated protein 29 | chr12 | 1.484 | 6 | 0.023 |  | | | |
| 161004\_at | 1700097N02Rik | RIKEN cDNA 1700097N02 gene | --- | 0.221 | 6 | 0.005 |  | | | |
| 161147\_f\_at | 1110046L09Rik | RIKEN cDNA 1110046L09 gene | chr8 | 3.517 | 6 | 0.013 |  | | | |
| 161359\_s\_at | Apoa1bp | apolipoprotein A-I binding protein | chr3 | 0.755 | 6 | 0.006 |  | | | |
| 161487\_f\_at | NoneAvailable | Mus musculus transcribed sequence with strong similarity to protein sp:P48201 (H.sapiens) AT93\_HUMAN ATP synthase lipid-binding protein, mitochondrial precursor (ATP synthase proteolipid P3) (ATPase protein 9) (ATPase subunit C) | --- | 0.986 | 6 | 0.005 |  | | | |
| 161522\_i\_at | Cst3 | cystatin C | chr2 | 0.465 | 6 | 0.046 |  | | | |
| 161872\_f\_at | 1110049G11Rik | RIKEN cDNA 1110049G11 gene | --- | 1.026 | 6 | 0.01 |  | | | |
| 161897\_f\_at | Prps1 | phosphoribosyl pyrophosphate synthetase 1 | chrX | 0.02 | 6 | 0.026 |  | | | |
| 162013\_f\_at | D4Ertd786e | DNA segment, Chr 4, ERATO Doi 786, expressed | chr7 | 0.907 | 6 | 0.006 |  | | | |
| 162235\_f\_at | D19Wsu55e | DNA segment, Chr 19, Wayne State University 55, expressed | chr19 | 0.378 | 6 | 0.003 |  | | | |
| 162417\_at | 1500001M20Rik | RIKEN cDNA 1500001M20 gene | --- | 0.537 | 6 | 0.003 |  | | | |
| 162466\_at | 1810004D07Rik | RIKEN cDNA 1810004D07 gene | --- | 0.295 | 6 | 0.031 |  | | | |
| 92388\_at | BC038311 | cDNA sequence BC038311 | chr5 | 0.478 | 6 | 0.025 |  | | | |
| 92540\_f\_at | Srm | spermidine synthase | chr4 | 6.091 | 6 | 0.001 |  | | | |
| 92547\_at | Hip2 | huntingtin interacting protein 2 | chr5 | 0.567 | 6 | 0.04 |  | | | |
| 92565\_at | 1110005A23Rik | RIKEN cDNA 1110005A23 gene | chr19 | 0.994 | 6 | 0.007 |  | | | |
| 92574\_at | 3110001M13Rik | RIKEN cDNA 3110001M13 gene | chr9 | 1.899 | 6 | 0.011 |  | | | |
| 92578\_at | Scye1 | small inducible cytokine subfamily E, member 1 | chr3 | 0.839 | 6 | 0.011 |  | | | |
| 92589\_at | Psph | phosphoserine phosphatase | chr5 | 0.853 | 6 | 0.013 |  | | | |
| 92596\_at | Cacybp | calcyclin binding protein | chr1 | 0.523 | 6 | 0.039 |  | | | |
| 92615\_at | AI837181 | expressed sequence AI837181 | chr4 | 2.807 | 6 | 0.001 |  | | | |
| 92625\_at | Nme2 | expressed in non-metastatic cells 2, protein | chr10 | 1.732 | 6 | 0.004 |  | | | |
| 92629\_f\_at | Hdgf | hepatoma-derived growth factor | --- | 0.521 | 6 | 0.019 |  | | | |
| 92636\_f\_at | Sec61g | SEC61, gamma subunit | chr1 | 1.533 | 6 | 0.004 |  | | | |
| 92646\_at | Mrpl23 | mitochondrial ribosomal protein L23 | chr2 | 2.152 | 6 | 0.028 |  | | | |
| 92770\_at | S100a6 | S100 calcium binding protein A6 (calcyclin) | chr3 | -3.204 | 6 | 0 |  | | | |
| 92778\_i\_at | NoneAvailable | --- | --- | -1.238 | 6 | 0.049 |  | | | |
| 92788\_f\_at | Cetn3 | centrin 3 | chr13 | 1.678 | 6 | 0.018 |  | | | |
| 92798\_at | Atp5c1 | ATP synthase, H+ transporting, mitochondrial F1 complex, gamma polypeptide 1 | chrX | 1.327 | 6 | 0.003 |  | | | |
| 92799\_g\_at | Atp5c1 | ATP synthase, H+ transporting, mitochondrial F1 complex, gamma polypeptide 1 | chr2 | 1.541 | 6 | 0.001 |  | | | |
| 92800\_i\_at | Atp5c1 | ATP synthase, H+ transporting, mitochondrial F1 complex, gamma polypeptide 1 | chr13 | 0.86 | 6 | 0 |  | | | |
| 92824\_at | Nme6 | expressed in non-metastatic cells 6, protein | chr9 | 1.781 | 6 | 0.001 |  | | | |
| 92826\_at | Gdap3 | ganglioside-induced differentiation-associated-protein 3 | --- | 0.209 | 6 | 0.003 |  | | | |
| 92829\_at | Hspe1 | heat shock protein 1 (chaperonin 10) | chr1 | 1.429 | 6 | 0.001 |  | | | |
| 92831\_at | Sfxn1 | sideroflexin 1 | --- | 1.223 | 6 | 0.005 |  | | | |
| 92874\_f\_at | Cops7a | COP9 (constitutive photomorphogenic) homolog, subunit 7a (Arabidopsis thaliana) | chr6 | 1.098 | 6 | 0.003 |  | | | |
| 92876\_at | Ndufs4 | NADH dehydrogenase (ubiquinone) Fe-S protein 4 | chr13 | 0.222 | 6 | 0.002 |  | | | |
| 93008\_at | Lsm4 | LSM4 homolog, U6 small nuclear RNA associated (S. cerevisiae) | chr8 | 2.177 | 6 | 0.021 |  | | | |
| 93014\_at | Atp5l | ATP synthase, H+ transporting, mitochondrial F0 complex, subunit g | chr11 | 1.668 | 6 | 0.019 |  | | | |
| 93029\_at | Idh3g | isocitrate dehydrogenase 3 (NAD+), gamma | chrX | 0.845 | 6 | 0.006 |  | | | |
| 93041\_at | Mcm4 | minichromosome maintenance deficient 4 homolog (S. cerevisiae) | chr16 | 1.922 | 6 | 0.039 |  | | | |
| 93042\_at | Bzrp | benzodiazepine receptor, peripheral | chr15 | 0.924 | 6 | 0.021 |  | | | |
| 93062\_at | Mrpl39 | mitochondrial ribosomal protein L39 | chr16 | 1.936 | 6 | 0.016 |  | | | |
| 93078\_at | Ly6a | lymphocyte antigen 6 complex, locus A | chr15 | -3.737 | 6 | 0.008 |  | | | |
| 93084\_at | Slc25a4 | solute carrier family 25 (mitochondrial carrier; adenine nucleotide translocator), member 4 | chr8 | 0.194 | 6 | 0.005 |  | | | |
| 93085\_at | Psmb9 | proteosome (prosome, macropain) subunit, beta type 9 (large multifunctional protease 2) | chr17 | 0.307 | 6 | 0.015 |  | | | |
| 93095\_at | Hmgb1 | high mobility group box 1 | chr11 | 0.963 | 6 | 0.03 |  | | | |
| 93101\_s\_at | Nedd4 | neural precursor cell expressed, developmentally down-regulted gene 4 | chr9 | -0.095 | 6 | 0.003 |  | | | |
| 93112\_at | Mcm2 | minichromosome maintenance deficient 2 mitotin (S. cerevisiae) | chr6 | 2.139 | 6 | 0.018 |  | | | |
| 93117\_at | Hnrpa2b1 | heterogeneous nuclear ribonucleoprotein A2/B1 | chr6 | 0.4 | 6 | 0.006 |  | | | |
| 93139\_at | 4432411H13Rik | RIKEN cDNA 4432411H13 gene | chr5 | -0.583 | 6 | 0.031 |  | | | |
| 93203\_f\_at | 3230402J05Rik | RIKEN cDNA 3230402J05 gene | chr17 | -0.003 | 6 | 0.01 |  | | | |
| 93236\_s\_at | Tyms | thymidylate synthase | chr10 | 3.228 | 6 | 0.012 |  | | | |
| 93237\_s\_at | Tyms | thymidylate synthase | chr10 | 2.144 | 6 | 0.021 |  | | | |
| 93251\_at | Nipsnap1 | 4-nitrophenylphosphatase domain and non-neuronal SNAP25-like protein homolog 1 (C. elegans) | chr11 | 0.772 | 6 | 0.027 |  | | | |
| 93258\_at | Hmbs | hydroxymethylbilane synthase | chr9 | 2.914 | 6 | 0.046 |  | | | |
| 93277\_at | Hspd1 | heat shock protein 1 (chaperonin) | chr1 | 1.14 | 6 | 0.029 |  | | | |
| 93290\_at | Pnp | purine-nucleoside phosphorylase | chr14 | 1.213 | 6 | 0.044 |  | | | |
| 93323\_at | Plp2 | proteolipid protein 2 | chr2 | -0.4 | 6 | 0.008 |  | | | |
| 93336\_at | 1110014C03Rik | RIKEN cDNA 1110014C03 gene | chr12 | 0.813 | 6 | 0.041 |  | | | |
| 93456\_r\_at | NoneAvailable | --- | chr14 | -0.076 | 6 | 0.01 |  | | | |
| 93495\_at | Prdx4 | peroxiredoxin 4 | chrX | 0.796 | 6 | 0.013 |  | | | |
| 93512\_f\_at | Adk | adenosine kinase | chr14 | 0.798 | 6 | 0 |  | | | |
| 93519\_s\_at | Nedd8 | neural precursor cell expressed, developmentally down-regulated gene 8 | chr14 | 2.012 | 6 | 0.002 |  | | | |
| 93531\_at | Ndufa8 | NADH dehydrogenase (ubiquinone) 1 alpha subcomplex, 8 | chr2 | 1.477 | 6 | 0.042 |  | | | |
| 93533\_at | 1500011L16Rik | RIKEN cDNA 1500011L16 gene | --- | 2.198 | 6 | 0.022 |  | | | |
| 93539\_at | 1810004D07Rik | RIKEN cDNA 1810004D07 gene | --- | 1.406 | 6 | 0 |  | | | |
| 93542\_at | Pter | phosphotriesterase related | chr2 | 1.123 | 6 | 0.022 |  | | | |
| 93548\_at | Sec61b | Sec61 beta subunit | chr4 | 2.149 | 6 | 0.005 |  | | | |
| 93551\_at | Tm4sf7 | transmembrane 4 superfamily member 7 | chr7 | 0.862 | 6 | 0.008 |  | | | |
| 93559\_at | Apex1 | apurinic/apyrimidinic endonuclease 1 | chr14 | 1.957 | 6 | 0.002 |  | | | |
| 93560\_at | Acyp1 | acylphosphatase 1, erythrocyte (common) type | chr12 | 3.035 | 6 | 0.029 |  | | | |
| 93562\_at | Ndufb3 | NADH dehydrogenase (ubiquinone) 1 beta subcomplex 3 | --- | 0.903 | 6 | 0.001 |  | | | |
| 93579\_at | 5830427H10Rik | RIKEN cDNA 5830427H10 gene | chr6 | 1.438 | 6 | 0.034 |  | | | |
| 93581\_at | Ndufb8 | NADH dehydrogenase (ubiquinone) 1 beta subcomplex 8 | chr19 | 2.79 | 6 | 0.003 |  | | | |
| 93582\_at | Coq7 | demethyl-Q 7 | chr7 | 3.745 | 6 | 0.001 |  | | | |
| 93593\_f\_at | Emp3 | epithelial membrane protein 3 | chr7 | 0.001 | 6 | 0.046 |  | | | |
| 93596\_i\_at | Atp5e | ATP synthase, H+ transporting, mitochondrial F1 complex, epsilon subunit | chr2 | 1.737 | 6 | 0.006 |  | | | |
| 93734\_i\_at | Psmc3 | proteasome (prosome, macropain) 26S subunit, ATPase 3 | chr2 | 0.698 | 6 | 0.002 |  | | | |
| 93735\_f\_at | Psmc3 | proteasome (prosome, macropain) 26S subunit, ATPase 3 | chr2 | 1.365 | 6 | 0.011 |  | | | |
| 93742\_at | 5730449L18Rik | RIKEN cDNA 5730449L18 gene | chr1 | 0.944 | 6 | 0.026 |  | | | |
| 93754\_at | Ech1 | enoyl coenzyme A hydratase 1, peroxisomal | chr7 | 0.716 | 6 | 0.015 |  | | | |
| 93764\_at | Grim19-pending | genes associated with retinoid-IFN-induced mortality 19 | --- | 0.229 | 6 | 0.005 |  | | | |
| 93780\_at | Them2 | thioesterase superfamily member 2 | chr13 | 1.504 | 6 | 0.012 |  | | | |
| 93784\_at | Cfdp | craniofacial development protein 1 | chr8 | 1.424 | 6 | 0.031 |  | | | |
| 93786\_i\_at | Mrpl18 | mitochondrial ribosomal protein L18 | chr17 | 2.577 | 6 | 0.013 |  | | | |
| 93787\_f\_at | Mrpl18 | mitochondrial ribosomal protein L18 | --- | 2.215 | 6 | 0.009 |  | | | |
| 93812\_at | Clns1a | chloride channel, nucleotide-sensitive, 1A | chr7 | 1.373 | 6 | 0.031 |  | | | |
| 93815\_at | 0610041L09Rik | RIKEN cDNA 0610041L09 gene | chr6 | 0.912 | 6 | 0.013 |  | | | |
| 93820\_at | Cox7a2 | cytochrome c oxidase, subunit VIIa 2 | chr9 | 1.951 | 6 | 0.002 |  | | | |
| 93833\_s\_at | Hist1h2bc | histone 1, H2bc | chr13 | 1.06 | 6 | 0.043 |  | | | |
| 93838\_at | 2700038C09Rik | RIKEN cDNA 2700038C09 gene | chr2 | 3.273 | 6 | 0.025 |  | | | |
| 93952\_r\_at | 2810417H13Rik | RIKEN cDNA 2810417H13 gene | --- | 0.619 | 6 | 0.014 |  | | | |
| 93970\_at | Ipo7 | importin 7 | chr7 | 0.551 | 6 | 0.018 |  | | | |
| 93984\_at | Atpi | ATPase inhibitor | --- | 1.332 | 6 | 0 |  | | | |
| 93988\_at | Psma7 | proteasome (prosome, macropain) subunit, alpha type 7 | chr2 | 1.392 | 6 | 0.02 |  | | | |
| 93991\_at | Mor1 | malate dehydrogenase, mitochondrial | chr5 | 1.136 | 6 | 0.009 |  | | | |
| 93993\_at | Lman2 | lectin, mannose-binding 2 | chr13 | 1.647 | 6 | 0.002 |  | | | |
| 93999\_at | Snrpg | small nuclear ribonucleoprotein polypeptide G | chr17 | 0.927 | 6 | 0.002 |  | | | |
| 94014\_at | 2510048O06Rik | RIKEN cDNA 2510048O06 gene | --- | 1.798 | 6 | 0 |  | | | |
| 94025\_at | Psmb3 | proteasome (prosome, macropain) subunit, beta type 3 | chr16 | 1.932 | 6 | 0.035 |  | | | |
| 94032\_at | Apoa1bp | apolipoprotein A-I binding protein | chr3 | 0.949 | 6 | 0.008 |  | | | |
| 94034\_at | Smfn | small fragment nuclease | chr9 | 0.778 | 6 | 0 |  | | | |
| 94040\_at | Erh | enhancer of rudimentary homolog (Drosophila) | chr12 | 1.929 | 6 | 0.001 |  | | | |
| 94062\_at | Ndufv2 | NADH dehydrogenase (ubiquinone) flavoprotein 2 | chr17 | 1.3 | 6 | 0.045 |  | | | |
| 94110\_f\_at | NoneAvailable | Mus musculus 0 day neonate thymus cDNA, RIKEN full-length enriched library, clone:A430088G18 product:hypothetical Zinc finger, C2H2 type containing protein, full insert sequence | --- | 0.488 | 6 | 0.033 |  | | | |
| 94206\_at | Grcc10 | gene rich cluster, C10 gene | chr6 | 0.906 | 6 | 0 |  | | | |
| 94210\_at | Timm9 | translocase of inner mitochondrial membrane 9 homolog (yeast) | chr10 | 3.713 | 6 | 0.022 |  | | | |
| 94252\_at | Eif2s3x | eukaryotic translation initiation factor 2, subunit 3, structural gene X-linked | chrX | 0.381 | 6 | 0.049 |  | | | |
| 94263\_f\_at | Psmb7 | proteasome (prosome, macropain) subunit, beta type 7 | chr2 | 1.959 | 6 | 0.018 |  | | | |
| 94267\_i\_at | NoneAvailable | --- | chr9 | 0.902 | 6 | 0.037 |  | | | |
| 94268\_f\_at | NoneAvailable | --- | chr9 | 0.316 | 6 | 0.004 |  | | | |
| 94275\_at | Urod | uroporphyrinogen decarboxylase | chr4 | 1.492 | 6 | 0.015 |  | | | |
| 94276\_at | Hsd17b12 | hydroxysteroid (17-beta) dehydrogenase 12 | chr2 | 0.315 | 6 | 0.041 |  | | | |
| 94277\_at | Mtx1 | metaxin 1 | chr3 | 1.276 | 6 | 0.007 |  | | | |
| 94278\_at | Lcp1 | lymphocyte cytosolic protein 1 | chr14 | -0.14 | 6 | 0.027 |  | | | |
| 94294\_at | Ccnb2 | cyclin B2 | chr9 | 4.408 | 6 | 0.001 |  | | | |
| 94313\_at | Snrp1c | U1 small nuclear ribonucleoprotein 1C | chr15 | -0.007 | 6 | 0.002 |  | | | |
| 94323\_at | D630012G11Rik | RIKEN cDNA D630012G11 gene | chr19 | 1.879 | 6 | 0 |  | | | |
| 94360\_at | 2700029M09Rik | RIKEN cDNA 2700029M09 gene | chr8 | 1.272 | 6 | 0.009 |  | | | |
| 94372\_at | Nudt1 | nudix (nucleoside diphosphate linked moiety X)-type motif 1 | --- | 3.289 | 6 | 0.003 |  | | | |
| 94394\_at | Rras | Harvey rat sarcoma oncogene, subgroup R | chr7 | -0.442 | 6 | 0.049 |  | | | |
| 94455\_at | Lsm3 | LSM3 homolog, U6 small nuclear RNA associated (S. cerevisiae) | chr6 | 2.688 | 6 | 0.002 |  | | | |
| 94494\_at | Farsl | phenylalanine-tRNA synthetase-like | chr1 | 1.832 | 6 | 0.008 |  | | | |
| 94506\_at | Cpsf5 | cleavage and polyadenylation specific factor 5 | chr3 | 0.914 | 6 | 0.007 |  | | | |
| 94526\_at | D10Ertd214e | DNA segment, Chr 10, ERATO Doi 214, expressed | chr10 | 2.227 | 6 | 0 |  | | | |
| 94558\_g\_at | Gtf3a | general transcription factor III A | chr5 | 1.943 | 6 | 0.011 |  | | | |
| 94789\_r\_at | Tubb5 | tubulin, beta 5 | chr17 | 3.275 | 6 | 0.044 |  | | | |
| 94841\_at | Psma5 | proteasome (prosome, macropain) subunit, alpha type 5 | chr10 | 1.755 | 6 | 0.007 |  | | | |
| 94862\_i\_at | Dncl2a | dynein, cytoplasmic, light chain 2A | --- | 0.742 | 6 | 0.043 |  | | | |
| 94870\_f\_at | 2310075M17Rik | RIKEN cDNA 2310075M17 gene | chr11 | 1.008 | 6 | 0.02 |  | | | |
| 94892\_r\_at | Mea1 | male enhanced antigen 1 | chr17 | 1.005 | 6 | 0.017 |  | | | |
| 94897\_at | Gpx4 | glutathione peroxidase 4 | --- | 0.101 | 6 | 0.046 |  | | | |
| 94908\_r\_at | 1110001J03Rik | RIKEN cDNA 1110001J03 gene | chr6 | 0.276 | 6 | 0.029 |  | | | |
| 94912\_at | Mrps21 | mitochondrial ribosomal protein S21 | chr3 | 1.449 | 6 | 0.017 |  | | | |
| 94925\_at | 1810055D05Rik | RIKEN cDNA 1810055D05 gene | chr3 | 0.976 | 6 | 0.021 |  | | | |
| 94931\_at | 1810045K17Rik | RIKEN cDNA 1810045K17 gene | chr3 | 1.276 | 6 | 0.034 |  | | | |
| 94933\_at | BC008155 | cDNA sequence BC008155 | chr17 | 1.315 | 6 | 0.039 |  | | | |
| 95015\_at | Akr1c13 | aldo-keto reductase family 1, member C13 | chr13 | 0.179 | 6 | 0.002 |  | | | |
| 95042\_at | C030002N13Rik | RIKEN cDNA C030002N13 gene | chr4 | 0.462 | 6 | 0.014 |  | | | |
| 95045\_at | 0610012D09Rik | RIKEN cDNA 0610012D09 gene | chr7 | 1.41 | 6 | 0 |  | | | |
| 95049\_at | Snrpd2 | small nuclear ribonucleoprotein D2 | chr10 | 1.485 | 6 | 0.03 |  | | | |
| 95052\_at | 1110035L05Rik | RIKEN cDNA 1110035L05 gene | chr4 | 0.243 | 6 | 0.025 |  | | | |
| 95053\_s\_at | Sdhb | succinate dehydrogenase complex, subunit B, iron sulfur (Ip) | chr4 | 2.681 | 6 | 0.023 |  | | | |
| 95061\_at | Bcas2 | breast carcinoma amplified sequence 2 | chr3 | 0.334 | 6 | 0 |  | | | |
| 95064\_at | Acaa2 | acetyl-Coenzyme A acyltransferase 2 (mitochondrial 3-oxoacyl-Coenzyme A thiolase) | chr18 | 0.479 | 6 | 0.016 |  | | | |
| 95067\_at | Mrpl2 | mitochondrial ribosomal protein L2 | chr17 | 1.667 | 6 | 0.023 |  | | | |
| 95091\_at | Sec13r | SEC13 related gene (S. cerevisiae) | --- | 1.672 | 6 | 0.001 |  | | | |
| 95105\_at | 2010110M21Rik | RIKEN cDNA 2010110M21 gene | chr13 | 0.297 | 6 | 0.025 |  | | | |
| 95132\_r\_at | Ndufb2 | NADH dehydrogenase (ubiquinone) 1 beta subcomplex, 2 | chr6 | 1.988 | 6 | 0.024 |  | | | |
| 95408\_at | 2310003F16Rik | RIKEN cDNA 2310003F16 gene | chr2 | 0.987 | 6 | 0.023 |  | | | |
| 95413\_at | 6030432N09Rik | RIKEN cDNA 6030432N09 gene | chr5 | 0.123 | 6 | 0.035 |  | | | |
| 95426\_at | Echs1 | enoyl Coenzyme A hydratase, short chain, 1, mitochondrial | chr7 | 0.838 | 6 | 0.02 |  | | | |
| 95441\_at | Timm23 | translocase of inner mitochondrial membrane 23 homolog (yeast) | chr1 | 1.675 | 6 | 0.042 |  | | | |
| 95448\_at | Psmc2 | proteasome (prosome, macropain) 26S subunit, ATPase 2 | chr5 | 1.408 | 6 | 0.017 |  | | | |
| 95460\_at | Cops5 | COP9 (constitutive photomorphogenic) homolog, subunit 5 (Arabidopsis thaliana) | chr1 | 1.564 | 6 | 0.025 |  | | | |
| 95472\_f\_at | Uqcrb | ubiquinol-cytochrome c reductase binding protein | chr13 | 0.734 | 6 | 0.006 |  | | | |
| 95479\_at | 1110036E10Rik | RIKEN cDNA 1110036E10 gene | chr11 | 0.426 | 6 | 0.049 |  | | | |
| 95480\_at | D11Wsu68e | DNA segment, Chr 11, Wayne State University 68, expressed | chr11 | 1.471 | 6 | 0.003 |  | | | |
| 95485\_at | Hadhsc | L-3-hydroxyacyl-Coenzyme A dehydrogenase, short chain | chr3 | 0.06 | 6 | 0.011 |  | | | |
| 95491\_at | Park7 | Parkinson disease (autosomal recessive, early onset) 7 | chr4 | 1.248 | 6 | 0.002 |  | | | |
| 95497\_at | 1110005A05Rik | RIKEN cDNA 1110005A05 gene | chr9 | 3.276 | 6 | 0.011 |  | | | |
| 95498\_at | Mrps15 | mitochondrial ribosomal protein S15 | chr4 | 2.035 | 6 | 0.005 |  | | | |
| 95561\_at | 1700013H19Rik | RIKEN cDNA 1700013H19 gene | chr8 | 0.899 | 6 | 0.003 |  | | | |
| 95590\_at | Alg5 | asparagine-linked glycosylation 5 homolog (yeast, dolichyl-phosphate beta-glucosyltransferase) | chr3 | 1.43 | 6 | 0.003 |  | | | |
| 95593\_at | Golph2 | golgi phosphoprotein 2 | chr13 | 1.191 | 6 | 0.018 |  | | | |
| 95606\_at | Nsap1l-pending | NS1-associated protein 1-like | chr9 | 1.239 | 6 | 0.005 |  | | | |
| 95620\_at | 2310016E22Rik | RIKEN cDNA 2310016E22 gene | chr12 | 0.069 | 6 | 0.004 |  | | | |
| 95634\_at | 0610010K14Rik | RIKEN cDNA 0610010K14 gene | --- | 1.777 | 6 | 0.023 |  | | | |
| 95635\_g\_at | 0610010K14Rik | RIKEN cDNA 0610010K14 gene | chr11 | 0.403 | 6 | 0.036 |  | | | |
| 95636\_at | 0610010K14Rik | RIKEN cDNA 0610010K14 gene | chr11 | 2.424 | 6 | 0.007 |  | | | |
| 95649\_at | Phf5a | PHD finger protein 5A | chr15 | 1.933 | 6 | 0.012 |  | | | |
| 95654\_at | Clic1 | chloride intracellular channel 1 | chr17 | -0.179 | 6 | 0.033 |  | | | |
| 95656\_i\_at | D13Wsu177e | DNA segment, Chr 13, Wayne State University 177, expressed | chr13 | 2.603 | 6 | 0.011 |  | | | |
| 95660\_at | 0610025L15Rik | RIKEN cDNA 0610025L15 gene | chr7 | 2.58 | 6 | 0 |  | | | |
| 95677\_at | 0610009C03Rik | RIKEN cDNA 0610009C03 gene | --- | 2.789 | 6 | 0.026 |  | | | |
| 95690\_at | 1110030L07Rik | RIKEN cDNA 1110030L07 gene | chr15 | 3.584 | 6 | 0.007 |  | | | |
| 95693\_at | Idh2 | isocitrate dehydrogenase 2 (NADP+), mitochondrial | chr7 | 1.921 | 6 | 0.021 |  | | | |
| 95696\_at | Txnl2 | thioredoxin-like 2 | chr4 | 1.145 | 6 | 0.009 |  | | | |
| 95698\_at | Ndufb7 | NADH dehydrogenase (ubiquinone) 1 beta subcomplex, 7 | chr8 | 2.162 | 6 | 0.008 |  | | | |
| 95707\_at | 2900010M23Rik | RIKEN cDNA 2900010M23 gene | chr17 | 2.087 | 6 | 0.032 |  | | | |
| 95714\_at | 0610009D07Rik | RIKEN cDNA 0610009D07 gene | chr12 | 1.473 | 6 | 0.027 |  | | | |
| 95718\_f\_at | Usmg5 | upregulated during skeletal muscle growth 5 | chr19 | 1.302 | 6 | 0.006 |  | | | |
| 95760\_at | 1110011K10Rik | RIKEN cDNA 1110011K10 gene | chr9 | 2.372 | 6 | 0.002 |  | | | |
| 95891\_at | NoneAvailable | Mus musculus transcribed sequences | chr16 | 3.953 | 6 | 0.017 |  | | | |
| 96016\_at | NoneAvailable | Mus musculus cDNA clone MGC:67366 IMAGE:5683334, complete cds | chr2 | 3.771 | 6 | 0.015 |  | | | |
| 96029\_at | Sf3a3 | splicing factor 3a, subunit 3, 60kDa | chr4 | 2.16 | 6 | 0.047 |  | | | |
| 96041\_at | Rbm3 | RNA binding motif protein 3 | chr1 | -0.448 | 6 | 0.038 |  | | | |
| 96048\_at | Hrsp12 | heat-responsive protein 12 | chr15 | 0.196 | 6 | 0.037 |  | | | |
| 96052\_at | Acp1 | acid phosphatase 1, soluble | --- | 1.712 | 6 | 0.022 |  | | | |
| 96081\_at | Tk1 | thymidine kinase 1 | chr11 | 2.893 | 6 | 0.014 |  | | | |
| 96089\_at | 4931406C07Rik | RIKEN cDNA 4931406C07 gene | chr9 | 1.515 | 6 | 0.003 |  | | | |
| 96093\_at | ORF11 | open reading frame 11 | --- | 0.678 | 6 | 0.01 |  | | | |
| 96112\_at | Etfa | electron transferring flavoprotein, alpha polypeptide | chr9 | 1.779 | 6 | 0.006 |  | | | |
| 96231\_at | 2010012D11Rik | RIKEN cDNA 2010012D11 gene | chr13 | 0.868 | 6 | 0.031 |  | | | |
| 96249\_at | Sep15-pending | selenoprotein | chr3 | 0.398 | 6 | 0.031 |  | | | |
| 96261\_at | NoneAvailable | Mus musculus cDNA clone MGC:67622 IMAGE:6410794, complete cds | chr4 | 1.384 | 6 | 0 |  | | | |
| 96267\_at | Ndufv1 | NADH dehydrogenase (ubiquinone) flavoprotein 1 | chr19 | 1.004 | 6 | 0.016 |  | | | |
| 96268\_at | Suclg1 | succinate-CoA ligase, GDP-forming, alpha subunit | chr6 | 1.173 | 6 | 0.015 |  | | | |
| 96289\_at | Stoml2 | stomatin (Epb7.2)-like 2 | chr4 | 2.901 | 6 | 0.001 |  | | | |
| 96291\_f\_at | NoneAvailable | Mus musculus cDNA clone IMAGE:6772417, with apparent retained intron | chr13 | 2.298 | 6 | 0 |  | | | |
| 96292\_r\_at | NoneAvailable | Mus musculus cDNA clone IMAGE:6772417, with apparent retained intron | chr13 | 1.544 | 6 | 0.01 |  | | | |
| 96293\_at | 2410015N17Rik | RIKEN cDNA 2410015N17 gene | chr7 | 4.422 | 6 | 0.006 |  | | | |
| 96319\_at | Cdc20 | cell division cycle 20 homolog (S. cerevisiae) | --- | 4.591 | 6 | 0.004 |  | | | |
| 96321\_at | Ndufa9 | NADH dehydrogenase (ubiquinone) 1 alpha subcomplex, 9 | chr6 | 1.871 | 6 | 0.001 |  | | | |
| 96336\_at | Gatm | glycine amidinotransferase (L-arginine:glycine amidinotransferase) | --- | 0.529 | 6 | 0.022 |  | | | |
| 96345\_at | Sdbcag84 | serologically defined breast cancer antigen 84 | chr2 | 0.341 | 6 | 0.049 |  | | | |
| 96353\_at | 1110021D01Rik | RIKEN cDNA 1110021D01 gene | chr13 | 2.999 | 6 | 0.003 |  | | | |
| 96355\_at | 2900055D03Rik | RIKEN cDNA 2900055D03 gene | --- | 0.736 | 6 | 0.01 |  | | | |
| 96605\_at | 0610011I04Rik | RIKEN cDNA 0610011I04 gene | chr6 | -1.492 | 6 | 0.012 |  | | | |
| 96611\_at | NoneAvailable | Mus musculus cDNA clone IMAGE:5694421, partial cds | chr17 | 0.414 | 6 | 0.001 |  | | | |
| 96613\_at | 5730536A07Rik | RIKEN cDNA 5730536A07 gene | chr9 | 2.658 | 6 | 0.014 |  | | | |
| 96627\_at | Ebp | phenylalkylamine Ca2+ antagonist (emopamil) binding protein | chrX | 1.299 | 6 | 0.009 |  | | | |
| 96634\_at | 5730469M10Rik | RIKEN cDNA 5730469M10 gene | --- | -0.064 | 6 | 0.037 |  | | | |
| 96652\_at | Mrpl28 | mitochondrial ribosomal protein L28 | chr17 | 1.368 | 6 | 0.026 |  | | | |
| 96668\_at | Timm17b | translocator of inner mitochondrial membrane b | chrX | 2.162 | 6 | 0.003 |  | | | |
| 96670\_at | Gstk1 | glutathione S-transferase kappa 1 | chr6 | -0.369 | 6 | 0.033 |  | | | |
| 96678\_at | Dhrs4 | dehydrogenase/reductase (SDR family) member 4 | chr14 | 0.742 | 6 | 0.006 |  | | | |
| 96696\_at | Hrmt1l2 | heterogeneous nuclear ribonucleoproteins  methyltransferase-like 2 (S. cerevisiae) | chr7 | 2.608 | 6 | 0.013 |  | | | |
| 96699\_at | Hmgn1 | high mobility group nucleosomal binding domain 1 | chr11 | 0.795 | 6 | 0.001 |  | | | |
| 96733\_at | Rap1gds1 | RAP1, GTP-GDP dissociation stimulator 1 | chr3 | 0.959 | 6 | 0.024 |  | | | |
| 96734\_at | Synj2bp | synaptojanin 2 binding protein | chr12 | 0.397 | 6 | 0.031 |  | | | |
| 96743\_at | Skiip | SKI interacting protein | --- | 1.897 | 6 | 0.005 |  | | | |
| 96775\_at | Cbx1 | chromobox homolog 1 (Drosophila HP1 beta) | chr11 | 0.466 | 6 | 0.008 |  | | | |
| 96840\_at | Gabarapl2 | GABA(A) receptor-associated protein like 2 | chr5 | -0.619 | 6 | 0.016 |  | | | |
| 96849\_at | Timm8a | translocase of inner mitochondrial membrane 8 homolog a (yeast) | chr10 | 0.859 | 6 | 0.048 |  | | | |
| 96861\_at | Mrpl50 | mitochondrial ribosomal protein L50 | chr4 | 1.687 | 6 | 0.015 |  | | | |
| 96888\_at | Akr1a4 | aldo-keto reductase family 1, member A4 (aldehyde reductase) | chr4 | 0.887 | 6 | 0.042 |  | | | |
| 96892\_at | Psma1 | proteasome (prosome, macropain) subunit, alpha type 1 | chr7 | 1.257 | 6 | 0.017 |  | | | |
| 96899\_at | Ndufs3 | NADH dehydrogenase (ubiquinone) Fe-S protein 3 | chr11 | 1.275 | 6 | 0.019 |  | | | |
| 96902\_at | 2900091E11Rik | RIKEN cDNA 2900091E11 gene | chr10 | 1.339 | 6 | 0.042 |  | | | |
| 96909\_at | Ndufab1 | NADH dehydrogenase (ubiquinone) 1, alpha/beta subcomplex, 1 | chr7 | 1.256 | 6 | 0.014 |  | | | |
| 96947\_at | Etfb | electron transferring flavoprotein, beta polypeptide | chr7 | 2.477 | 6 | 0 |  | | | |
| 96948\_at | Qdpr | quininoid dihydropteridine reductase | chr5 | 0.591 | 6 | 0.006 |  | | | |
| 96952\_at | Psma6 | proteasome (prosome, macropain) subunit, alpha type 6 | chr12 | 1.235 | 6 | 0.004 |  | | | |
| 96956\_at | 0610038D11Rik | RIKEN cDNA 0610038D11 gene | chr19 | 0.405 | 6 | 0.016 |  | | | |
| 97013\_f\_at | Cyba | cytochrome b-245, alpha polypeptide | chr8 | 0.992 | 6 | 0.013 |  | | | |
| 97055\_s\_at | NoneAvailable | --- | chr16 | 1.087 | 6 | 0.01 |  | | | |
| 97164\_at | 2610207P08Rik | RIKEN cDNA 2610207P08 gene | --- | 3.233 | 6 | 0.011 |  | | | |
| 97179\_at | NoneAvailable | Mus musculus mRNA similar to putative c-Myc-responsive (cDNA clone MGC:54855 IMAGE:5388297), complete cds | --- | 2.605 | 6 | 0.001 |  | | | |
| 97200\_f\_at | Snrpe | small nuclear ribonucleoprotein E | chr1 | 0.59 | 6 | 0.041 |  | | | |
| 97201\_s\_at | Ndufa5 | NADH dehydrogenase (ubiquinone) 1 alpha subcomplex, 5 | chr6 | 1.857 | 6 | 0.001 |  | | | |
| 97207\_f\_at | Lypla1 | lysophospholipase 1 | chr1 | 0.831 | 6 | 0.047 |  | | | |
| 97220\_at | Dscr2 | Down syndrome critical region homolog 2 (human) | chr16 | 5.415 | 6 | 0 |  | | | |
| 97248\_at | Dbi | diazepam binding inhibitor | --- | 1.56 | 6 | 0 |  | | | |
| 97268\_i\_at | 0610010I12Rik | RIKEN cDNA 0610010I12 gene | chr3 | 1.265 | 6 | 0.003 |  | | | |
| 97274\_at | Psmd14 | proteasome (prosome, macropain) 26S subunit, non-ATPase, 14 | chr2 | 2.128 | 6 | 0.001 |  | | | |
| 97276\_at | Ckap1 | cytoskeleton-associated protein 1 | --- | 0.402 | 6 | 0.044 |  | | | |
| 97279\_at | 6430402H10Rik | RIKEN cDNA 6430402H10 gene | chr6 | -0.199 | 6 | 0.016 |  | | | |
| 97307\_f\_at | Ndufb5 | NADH dehydrogenase (ubiquinone) 1 beta subcomplex, 5 | --- | 1.376 | 6 | 0.003 |  | | | |
| 97311\_at | 6720456B07Rik | RIKEN cDNA 6720456B07 gene | chr6 | 0.701 | 6 | 0 |  | | | |
| 97318\_at | Hars2 | histidyl tRNA synthetase 2 | chr2 | 0.864 | 6 | 0 |  | | | |
| 97342\_at | Mrps14 | mitochondrial ribosomal protein S14 | chr1 | 2.351 | 6 | 0.001 |  | | | |
| 97346\_at | 2610001J05Rik | RIKEN cDNA 2610001J05 gene | chr6 | 0.616 | 6 | 0.03 |  | | | |
| 97356\_at | 1810008O21Rik | RIKEN cDNA 1810008O21 gene | chr7 | 0.227 | 6 | 0.037 |  | | | |
| 97374\_at | 2810025M15Rik | RIKEN cDNA 2810025M15 gene | chr1 | 2.124 | 6 | 0.003 |  | | | |
| 97395\_at | D19Wsu55e | DNA segment, Chr 19, Wayne State University 55, expressed | --- | 0.449 | 6 | 0.015 |  | | | |
| 97412\_at | 3300001G02Rik | RIKEN cDNA 3300001G02 gene | chr11 | 2.545 | 6 | 0.004 |  | | | |
| 97419\_at | 2310010I22Rik | RIKEN cDNA 2310010I22 gene | chr2 | 0.687 | 6 | 0.007 |  | | | |
| 97424\_at | Arl6ip5 | ADP-ribosylation factor-like 6 interacting protein 5 | chr6 | 0.945 | 6 | 0.001 |  | | | |
| 97449\_at | Aldh7a1 | aldehyde dehydrogenase family 7, member A1 | chr18 | 0.856 | 6 | 0.043 |  | | | |
| 97459\_at | Psma4 | proteasome (prosome, macropain) subunit, alpha type 4 | chr18 | 1.676 | 6 | 0.03 |  | | | |
| 97460\_at | Ube2r2 | ubiquitin-conjugating enzyme E2R 2 | --- | 0.681 | 6 | 0.047 |  | | | |
| 97468\_at | Cks1 | CDC28 protein kinase 1 | chr3 | 2.843 | 6 | 0 |  | | | |
| 97477\_at | Timm8b | translocase of inner mitochondrial membrane 8 homolog b (yeast) | chr9 | 1.26 | 6 | 0.04 |  | | | |
| 97478\_at | C030004C14Rik | RIKEN cDNA C030004C14 gene | chr13 | 0.351 | 6 | 0.002 |  | | | |
| 97512\_at | 2010107E04Rik | RIKEN cDNA 2010107E04 gene | chr12 | 0.955 | 6 | 0.007 |  | | | |
| 97538\_at | Gus | beta-glucuronidase | chr5 | 1.607 | 6 | 0.033 |  | | | |
| 97751\_f\_at | NoneAvailable | --- | --- | 0.47 | 6 | 0.022 |  | | | |
| 97758\_at | Prdx1 | peroxiredoxin 1 | chr8 | 1.211 | 6 | 0.01 |  | | | |
| 97807\_at | 1110021H02Rik | RIKEN cDNA 1110021H02 gene | chr1 | 1.1 | 6 | 0.015 |  | | | |
| 97819\_at | Gsto1 | glutathione S-transferase omega 1 | chr19 | 0.024 | 6 | 0.001 |  | | | |
| 97820\_at | Galk1 | galactokinase 1 | chr11 | 2.703 | 6 | 0.004 |  | | | |
| 97824\_at | D11Ertd175e | DNA segment, Chr 11, ERATO Doi 175, expressed | chr11 | 1.494 | 6 | 0.006 |  | | | |
| 97828\_at | Siva-pending | Cd27 binding protein (Hindu God of destruction) | chr12 | 1.387 | 6 | 0 |  | | | |
| 97884\_at | Mrps11 | mitochondrial ribosomal protein S11 | chr7 | 1.959 | 6 | 0.019 |  | | | |
| 97885\_at | 1810009M01Rik | RIKEN cDNA 1810009M01 gene | chr6 | -0.938 | 6 | 0.043 |  | | | |
| 97907\_at | Lsm7 | LSM7 homolog, U6 small nuclear RNA associated (S. cerevisiae) | chr10 | 1.945 | 6 | 0.002 |  | | | |
| 97979\_at | Ppp1r7 | protein phosphatase 1, regulatory (inhibitor) subunit 7 | chr1 | 2.977 | 6 | 0.019 |  | | | |
| 98039\_at | 2410015M20Rik | RIKEN cDNA 2410015M20 gene | chr17 | 1.43 | 6 | 0.019 |  | | | |
| 98075\_at | G431001I09Rik | RIKEN cDNA G431001I09 gene | chr2 | 3.225 | 6 | 0.001 |  | | | |
| 98077\_at | Snrpd3 | small nuclear ribonucleoprotein D3 | chr10 | 1.794 | 6 | 0.001 |  | | | |
| 98081\_at | Rpo1-3 | RNA polymerase 1-3 | chr4 | 0.959 | 6 | 0.001 |  | | | |
| 98092\_at | Plac8 | placenta-specific 8 | chr5 | 2.981 | 6 | 0.004 |  | | | |
| 98120\_at | Mrpl27 | mitochondrial ribosomal protein L27 | chr11 | 3.683 | 6 | 0.001 |  | | | |
| 98153\_at | Cct3 | chaperonin subunit 3 (gamma) | chr3 | 1.573 | 6 | 0.02 |  | | | |
| 98440\_at | Ltb4dh | leukotriene B4 12-hydroxydehydrogenase | chr4 | 3.855 | 6 | 0 |  | | | |
| 98456\_at | Stk19 | serine/threonine kinase 19 | --- | 0.189 | 6 | 0.008 |  | | | |
| 98492\_at | Cklfsf7 | chemokine-like factor super family 7 | chr9 | 1.661 | 6 | 0.036 |  | | | |
| 98516\_at | NoneAvailable | Mus musculus cDNA clone MGC:67360 IMAGE:6823629, complete cds | chr8 | 0.615 | 6 | 0.016 |  | | | |
| 98524\_f\_at | NoneAvailable | --- | --- | 2.603 | 6 | 0.008 |  | | | |
| 98527\_at | Dci | dodecenoyl-Coenzyme A delta isomerase (3,2 trans-enoyl-Coenyme A isomerase) | chr17 | 1.306 | 6 | 0.011 |  | | | |
| 98557\_f\_at | Psmb4 | proteasome (prosome, macropain) subunit, beta type 4 | --- | 0.285 | 6 | 0.018 |  | | | |
| 98564\_f\_at | Rps26 | ribosomal protein S26 | chr10 | 0.016 | 6 | 0.025 |  | | | |
| 98587\_at | Nap1l1 | nucleosome assembly protein 1-like 1 | chr10 | 0.829 | 6 | 0.015 |  | | | |
| 98595\_at | Irak1 | interleukin-1 receptor-associated kinase 1 | --- | -0.518 | 6 | 0.02 |  | | | |
| 98610\_at | Mrps28 | mitochondrial ribosomal protein S28 | chr3 | 1.473 | 6 | 0.04 |  | | | |
| 98613\_at | 2700085E05Rik | RIKEN cDNA 2700085E05 gene | chr11 | 0.873 | 6 | 0.02 |  | | | |
| 98904\_at | Mrpl35 | mitochondrial ribosomal protein L35 | chr6 | 2.237 | 6 | 0.002 |  | | | |
| 98910\_at | D030012E24Rik | RIKEN cDNA D030012E24 gene | chr11 | 0.973 | 6 | 0.008 |  | | | |
| 98920\_g\_at | 2410018G23Rik | RIKEN cDNA 2410018G23 gene | chrX | -2.085 | 6 | 0.004 |  | | | |
| 98921\_at | 2410018G23Rik | RIKEN cDNA 2410018G23 gene | chr8 | -1.976 | 6 | 0.004 |  | | | |
| 98930\_at | Cope | coatomer protein complex, subunit epsilon | chr8 | 1.116 | 6 | 0.022 |  | | | |
| 98934\_at | 0610007P06Rik | RIKEN cDNA 0610007P06 gene | chr7 | 4.269 | 6 | 0 |  | | | |
| 98938\_at | 1500026D16Rik | RIKEN cDNA 1500026D16 gene | chr19 | 1.602 | 6 | 0.013 |  | | | |
| 98959\_at | 0610016J10Rik | RIKEN cDNA 0610016J10 gene | chr17 | 0.606 | 6 | 0.011 |  | | | |
| 98966\_at | Dbt | dihydrolipoamide branched chain transacylase E2 | chr3 | 1.257 | 6 | 0.033 |  | | | |
| 98975\_at | 2410008G02Rik | RIKEN cDNA 2410008G02 gene | chr8 | 0.732 | 6 | 0.033 |  | | | |
| 99056\_at | Pcbd | 6-pyruvoyl-tetrahydropterin synthase/dimerization cofactor of hepatocyte nuclear factor 1 alpha (TCF1) | chr10 | 0.111 | 6 | 0.008 |  | | | |
| 99106\_at | Cops6 | COP9 (constitutive photomorphogenic) homolog, subunit 6 (Arabidopsis thaliana) | chr5 | 2.396 | 6 | 0.029 |  | | | |
| 99128\_at | Atp5o | ATP synthase, H+ transporting, mitochondrial F1 complex, O subunit | chr16 | 1.704 | 6 | 0.021 |  | | | |
| 99129\_at | Clast3-pending | CD40 ligand-activated specific transcript 3 | chr18 | 1.773 | 6 | 0.039 |  | | | |
| 99147\_at | Znhit1 | zinc finger, HIT domain containing 1 | --- | 0.46 | 6 | 0.042 |  | | | |
| 99148\_at | Fh1 | fumarate hydratase 1 | chr1 | 2.824 | 6 | 0.009 |  | | | |
| 99151\_at | 2610002K22Rik | RIKEN cDNA 2610002K22 gene | --- | 3.469 | 6 | 0.004 |  | | | |
| 99156\_at | 2700099C19Rik | RIKEN cDNA 2700099C19 gene | chrX | 0.68 | 6 | 0.049 |  | | | |
| 99164\_at | 2010111E04Rik | RIKEN cDNA 2010111E04 gene | chr3 | 2.906 | 6 | 0.006 |  | | | |
| 99179\_at | 3010002G01Rik | RIKEN cDNA 3010002G01 gene | chr19 | 0.465 | 6 | 0.011 |  | | | |
| 99182\_at | 2610511E03Rik | RIKEN cDNA 2610511E03 gene | chr14 | 0.954 | 6 | 0.044 |  | | | |
| 99522\_at | Gsg2 | germ cell-specific gene 2 | --- | 0.308 | 6 | 0.008 |  | | | |
| 99537\_at | Ruvbl1 | RuvB-like protein 1 | chr1 | 3.012 | 6 | 0.022 |  | | | |
| 99544\_at | Dguok | deoxyguanosine kinase | chr6 | 0.647 | 6 | 0.034 |  | | | |
| 99546\_at | Fkbp2 | FK506 binding protein 2 | --- | 2.273 | 6 | 0.001 |  | | | |
| 99566\_at | Tpi | triosephosphate isomerase | chr6 | 1.133 | 6 | 0.048 |  | | | |
| 99583\_at | Gstp2 | glutathione S-transferase, pi 2 | chr19 | 1.142 | 6 | 0.003 |  | | | |
| 99586\_at | Cst3 | cystatin C | chr2 | 0.348 | 6 | 0.003 |  | | | |
| 99594\_at | Mrpl51 | mitochondrial ribosomal protein L51 | chr6 | 2.427 | 6 | 0 |  | | | |
| 99613\_at | Mut | methylmalonyl-Coenzyme A mutase | chr17 | 0.426 | 6 | 0.012 |  | | | |
| 99618\_at | 0710008D09Rik | RIKEN cDNA 0710008D09 gene | chr10 | 2.5 | 6 | 0 |  | | | |
| 99632\_at | Mad2l1 | MAD2 (mitotic arrest deficient, homolog)-like 1 (yeast) | chr6 | 1.589 | 6 | 0.018 |  | | | |
| 99651\_at | 2610209M04Rik | RIKEN cDNA 2610209M04 gene | chr6 | 1.176 | 6 | 0.024 |  | | | |
| 99655\_at | 1810012E07Rik | RIKEN cDNA 1810012E07 gene | chr7 | 0.653 | 6 | 0.001 |  | | | |
| 99991\_at | Il17r | interleukin 17 receptor | chr6 | 0.281 | 6 | 0.036 |  | | | |
| \* Positive log2 fold changes represent genes expressed higher in FL-HSC; Negative log2 fold changes represent genes expressed higher in adult HSC (fold change=2 is equivalent to log2 fold change=1) | | | | | | | | | | |
|  |  |  |  |  |  |  |  |  |  |  |
