# Supplementary material for: Molecular Signatures of Proliferation and Quiescence in Hematopoietic Stem Cells
Source: PLoS Biol. 2004 Sep 28;2(10):e301. doi: 10.1371/journal.pbio.0020301 (PMC520599; doi:10.1371/journal.pbio.0020301)
Supplement: Table S3 — (1.4 MB HTML). [file pbio.0020301.st003.html]

|  |  | Genes changing over time course | | |  |  |  |  |  |  |
| Probe Set ID | Gene Symbol | Gene name | Chromosome | Log2 Fold Change (FL-HSC vs Adult HSC)\* | Day of max (TOM) | p-value of ANOVA (time course) |  | | | |
| 100322\_at | Igh-4 | immunoglobulin heavy chain 4 (serum IgG1) | --- | -0.13 | 0 | 0.024 |  | | | |
| 100473\_at | 1200006I17Rik | RIKEN cDNA 1200006I17 gene | chr7 | -0.11 | 0 | 0.034 |  | | | |
| 100533\_s\_at | Crem | cAMP responsive element modulator | --- | -5.967 | 0 | 0.008 |  | | | |
| 100582\_at | Snx17 | sorting nexin 17 | chr5 | 0.273 | 0 | 0.012 |  | | | |
| 100914\_at | NoneAvailable | Mus musculus 13 days embryo spinal cord cDNA, RIKEN full-length enriched library, clone:G630020J15 product:unclassifiable, full insert sequence | --- | 0.433 | 0 | 0.046 |  | | | |
| 101072\_at | BC010348 | cDNA sequence BC010348 | chr10 | 0.306 | 0 | 0.025 |  | | | |
| 101426\_at | Cerk | ceramide kinase | chr15 | -0.726 | 0 | 0.022 |  | | | |
| 101515\_at | Acox1 | acyl-Coenzyme A oxidase 1, palmitoyl | --- | -3.857 | 0 | 0.018 |  | | | |
| 101897\_g\_at | Cd1d2 | CD1d2 antigen | chr3 | -0.949 | 0 | 0.024 |  | | | |
| 101940\_at | Smpd2 | sphingomyelin phosphodiesterase 2, neutral | chr10 | -0.013 | 0 | 0.039 |  | | | |
| 101962\_at | 2610007K22Rik | RIKEN cDNA 2610007K22 gene | chr15 | -0.339 | 0 | 0.015 |  | | | |
| 102021\_at | Il4ra | interleukin 4 receptor, alpha | chr7 | -0.384 | 0 | 0.033 |  | | | |
| 102156\_f\_at | NoneAvailable | --- | chr6 | -3.755 | 0 | 0.005 |  | | | |
| 102209\_at | Nfatc1 | nuclear factor of activated T-cells, cytoplasmic 1 | chr18 | -2.297 | 0 | 0.009 |  | | | |
| 102225\_at | 8430421H08Rik | RIKEN cDNA 8430421H08 gene | chr1 | 0.012 | 0 | 0.005 |  | | | |
| 102286\_at | Araf | raf-related oncogene | chrX | 0.464 | 0 | 0.035 |  | | | |
| 102326\_at | Ncf2 | neutrophil cytosolic factor 2 | chr1 | -1.48 | 0 | 0.013 |  | | | |
| 102356\_at | Wdr23 | WD repeat domain 23 | chr14 | -1.437 | 0 | 0.036 |  | | | |
| 102790\_at | Jtb | jumping translocation breakpoint | --- | -0.421 | 0 | 0.04 |  | | | |
| 102884\_at | Inpp5d | inositol polyphosphate-5-phosphatase D | chr1 | 0.265 | 0 | 0.013 |  | | | |
| 102896\_at | Dok1 | downstream of tyrosine kinase 1 | chr6 | -0.631 | 0 | 0.035 |  | | | |
| 102933\_at | Plxna3 | plexin A3 | chrX | -0.272 | 0 | 0.006 |  | | | |
| 102955\_at | Nfil3 | nuclear factor, interleukin 3, regulated | chr13 | -2.259 | 0 | 0.012 |  | | | |
| 103037\_at | Ctf1 | cardiotrophin 1 | --- | -1.295 | 0 | 0.012 |  | | | |
| 103217\_at | Cflar | CASP8 and FADD-like apoptosis regulator | --- | -0.404 | 0 | 0.01 |  | | | |
| 103226\_at | Mrc1 | mannose receptor, C type 1 | chr2 | -2.184 | 0 | 0.003 |  | | | |
| 103348\_at | 1110018F06Rik | RIKEN cDNA 1110018F06 gene | --- | -0.369 | 0 | 0.002 |  | | | |
| 103381\_at | 1810024J13Rik | RIKEN cDNA 1810024J13 gene | chr5 | 0.119 | 0 | 0.025 |  | | | |
| 103392\_at | Adcy7 | adenylate cyclase 7 | chr8 | -0.359 | 0 | 0.003 |  | | | |
| 103427\_at | Fbxl3a | F-box and leucine-rich repeat protein 3a | chr14 | -1.769 | 0 | 0.042 |  | | | |
| 103832\_at | Tfip11 | tuftelin interacting protein 11 | chr5 | -0.287 | 0 | 0.036 |  | | | |
| 103990\_at | Fosb | FBJ osteosarcoma oncogene B | chr7 | -4.608 | 0 | 0.012 |  | | | |
| 104146\_at | 2610025P08Rik | RIKEN cDNA 2610025P08 gene | chr7 | -0.243 | 0 | 0.035 |  | | | |
| 104252\_at | AU020206 | expressed sequence AU020206 | chr7 | -1.192 | 0 | 0.034 |  | | | |
| 104362\_at | B230113M03Rik | RIKEN cDNA B230113M03 gene | chr11 | -0.083 | 0 | 0.024 |  | | | |
| 104396\_at | Sh3glb1 | SH3-domain GRB2-like B1 (endophilin) | chr6 | -0.559 | 0 | 0.014 |  | | | |
| 104398\_at | 1300010A20Rik | RIKEN cDNA 1300010A20 gene | chr6 | 0.057 | 0 | 0.037 |  | | | |
| 104516\_at | Cldn5 | claudin 5 | chr16 | -1.272 | 0 | 0.048 |  | | | |
| 104562\_at | 5730403M16Rik | RIKEN cDNA 5730403M16 gene | chr7 | -1.253 | 0 | 0.009 |  | | | |
| 104598\_at | Dusp1 | dual specificity phosphatase 1 | chr17 | -2.35 | 0 | 0.018 |  | | | |
| 104625\_at | Dnajb6 | DnaJ (Hsp40) homolog, subfamily B, member 6 | chr16 | -1.482 | 0 | 0.041 |  | | | |
| 104719\_at | Slc12a7 | solute carrier family 12, member 7 | chr13 | -1.166 | 0 | 0.039 |  | | | |
| 160104\_at | Hsd3b7 | hydroxy-delta-5-steroid dehydrogenase, 3 beta- and steroid delta-isomerase 7 | chr7 | -1.424 | 0 | 0.022 |  | | | |
| 160273\_at | NoneAvailable | Mus musculus Brf2 gene, 3' UTR | chr17 | -0.145 | 0 | 0.008 |  | | | |
| 160346\_at | 6330580J24Rik | RIKEN cDNA 6330580J24 gene | chr9 | -0.152 | 0 | 0.003 |  | | | |
| 160362\_at | Mat2a | methionine adenosyltransferase II, alpha | chr6 | -0.381 | 0 | 0.016 |  | | | |
| 160526\_s\_at | Crem | cAMP responsive element modulator | --- | -5.103 | 0 | 0.005 |  | | | |
| 160547\_s\_at | Txnip | thioredoxin interacting protein | chr3 | -0.865 | 0 | 0 |  | | | |
| 160564\_at | Lcn2 | lipocalin 2 | chr2 | -0.186 | 0 | 0.038 |  | | | |
| 160573\_at | Hccs | holocytochrome c synthetase | --- | -2.08 | 0 | 0.025 |  | | | |
| 160664\_at | 1200004M23Rik | RIKEN cDNA 1200004M23 gene | chr19 | -0.403 | 0 | 0.005 |  | | | |
| 160714\_at | Gab1 | growth factor receptor bound protein 2-associated protein 1 | chr8 | -0.506 | 0 | 0.048 |  | | | |
| 160792\_at | Snap25bp | synaptosomal-associated protein 25 binding protein | --- | 0.342 | 0 | 0.044 |  | | | |
| 160901\_at | Fos | FBJ osteosarcoma oncogene | chr12 | -3.257 | 0 | 0.024 |  | | | |
| 161211\_r\_at | Sox6 | SRY-box containing gene 6 | --- | -0.328 | 0 | 0.017 |  | | | |
| 161683\_r\_at | Gtpbp1 | GTP binding protein 1 | --- | -0.546 | 0 | 0.035 |  | | | |
| 92248\_at | Nr4a2 | nuclear receptor subfamily 4, group A, member 2 | chr2 | -5.877 | 0 | 0.007 |  | | | |
| 92256\_at | Fdft1 | farnesyl diphosphate farnesyl transferase 1 | --- | -1.15 | 0 | 0.003 |  | | | |
| 92270\_at | Tro | trophinin | chrX | -1.72 | 0 | 0.006 |  | | | |
| 92310\_at | Snk | serum-inducible kinase | chr13 | -0.859 | 0 | 0.003 |  | | | |
| 92323\_at | Mapk12 | mitogen-activated protein kinase 12 | chr15 | -0.894 | 0 | 0.026 |  | | | |
| 92558\_at | Vcam1 | vascular cell adhesion molecule 1 | chr3 | -4.435 | 0 | 0.005 |  | | | |
| 92644\_s\_at | Myb | myeloblastosis oncogene | chr10 | -0.777 | 0 | 0.029 |  | | | |
| 92672\_at | 5530400H20Rik | RIKEN cDNA 5530400H20 gene | chr2 | -0.636 | 0 | 0.005 |  | | | |
| 92830\_s\_at | NoneAvailable | --- | --- | -3.484 | 0 | 0.001 |  | | | |
| 93093\_at | Mcl1 | myeloid cell leukemia sequence 1 | chr3 | -2.974 | 0 | 0.018 |  | | | |
| 93120\_f\_at | H2-K | histocompatibility 2, K region | chr17 | -3.637 | 0 | 0.003 |  | | | |
| 93179\_at | B830009D23Rik | RIKEN cDNA B830009D23 gene | chr2 | -1.837 | 0 | 0.017 |  | | | |
| 93326\_at | Tm4sf2 | transmembrane 4 superfamily member 2 | chrX | -1.259 | 0 | 0.02 |  | | | |
| 93498\_s\_at | Aplp2 | amyloid beta (A4) precursor-like protein 2 | chr9 | -2.613 | 0 | 0.02 |  | | | |
| 93500\_at | Alas1 | aminolevulinic acid synthase 1 | chr9 | 2.837 | 0 | 0.047 |  | | | |
| 93584\_at | Igh-6 | immunoglobulin heavy chain 6 (heavy chain of IgM) | chr12 | 1.572 | 0 | 0.006 |  | | | |
| 93682\_at | Ldb2 | LIM domain binding 2 | --- | 0.32 | 0 | 0.014 |  | | | |
| 93705\_at | Chrnb1 | cholinergic receptor, nicotinic, beta polypeptide 1 (muscle) | --- | -2.651 | 0 | 0.016 |  | | | |
| 93714\_f\_at | H2-Q7 | histocompatibility 2, Q region locus 7 | chr17 | -3.128 | 0 | 0.001 |  | | | |
| 93907\_f\_at | NoneAvailable | --- | --- | -1.754 | 0 | 0.001 |  | | | |
| 93964\_s\_at | Ddx6 | DEAD (Asp-Glu-Ala-Asp) box polypeptide 6 | chr9 | -3.009 | 0 | 0.03 |  | | | |
| 94288\_at | Hist1h1c | histone 1, H1c | chr13 | -0.134 | 0 | 0.019 |  | | | |
| 94345\_at | Il6st | interleukin 6 signal transducer | chr13 | -3.717 | 0 | 0 |  | | | |
| 94386\_at | Son | Son cell proliferation protein | chr16 | -0.788 | 0 | 0.028 |  | | | |
| 94428\_at | Ilvbl | ilvB (bacterial acetolactate synthase)-like | chr10 | -1.305 | 0 | 0.004 |  | | | |
| 94834\_at | Ctsh | cathepsin H | chr9 | -1.968 | 0 | 0.045 |  | | | |
| 94929\_at | Ptpn1 | protein tyrosine phosphatase, non-receptor type 1 | chr2 | -0.604 | 0 | 0.003 |  | | | |
| 94948\_at | Trip6 | thyroid hormone receptor interactor 6 | chr5 | -1.016 | 0 | 0.02 |  | | | |
| 94990\_at | Pl6-pending | PL6 protein | chr9 | -0.163 | 0 | 0.017 |  | | | |
| 94991\_at | Synpo | synaptopodin | chr18 | -1.276 | 0 | 0.018 |  | | | |
| 95031\_at | 1110059H15Rik | RIKEN cDNA 1110059H15 gene | chr2 | -0.433 | 0 | 0.022 |  | | | |
| 95102\_at | Scotin-pending | scotin gene | chr9 | -2.721 | 0 | 0.006 |  | | | |
| 95133\_at | Asns | asparagine synthetase | chr6 | -0.283 | 0 | 0.036 |  | | | |
| 95138\_at | 1110018O08Rik | RIKEN cDNA 1110018O08 gene | chr5 | -0.739 | 0 | 0.004 |  | | | |
| 95157\_at | B830022L21Rik | RIKEN cDNA B830022L21 gene | chr8 | -0.743 | 0 | 0.019 |  | | | |
| 95449\_at | 2310075G12Rik | RIKEN cDNA 2310075G12 gene | chr11 | -1.383 | 0 | 0.016 |  | | | |
| 95520\_at | 2310061B02Rik | RIKEN cDNA 2310061B02 gene | chr1 | -2.378 | 0 | 0.04 |  | | | |
| 95537\_at | Ulk2 | Unc-51 like kinase 2 (C. elegans) | chr11 | -0.43 | 0 | 0.011 |  | | | |
| 95893\_at | Blk | B lymphoid kinase | chr14 | -0.111 | 0 | 0.005 |  | | | |
| 96049\_at | Bgn | biglycan | --- | -1.681 | 0 | 0.008 |  | | | |
| 96186\_at | Lrp10 | low-density lipoprotein receptor-related protein 10 | chr14 | -3.157 | 0 | 0.013 |  | | | |
| 96310\_at | Mbp | myelin basic protein | chr18 | -0.13 | 0 | 0.036 |  | | | |
| 96530\_at | NoneAvailable | Mus musculus transcribed sequences | --- | -1.094 | 0 | 0.002 |  | | | |
| 96640\_at | 3110001A13Rik | RIKEN cDNA 3110001A13 gene | chr2 | -0.765 | 0 | 0.02 |  | | | |
| 96732\_at | 1500001L20Rik | RIKEN cDNA 1500001L20 gene | chr4 | -0.397 | 0 | 0.011 |  | | | |
| 96848\_at | Inpp5e | inositol polyphosphate-5-phosphatase E | chr2 | -0.985 | 0 | 0.001 |  | | | |
| 96886\_at | Stab1 | stabilin 1 | chr14 | -2.451 | 0 | 0.017 |  | | | |
| 96912\_s\_at | Ctla2a | cytotoxic T lymphocyte-associated protein 2 alpha | chr13 | -3.703 | 0 | 0.031 |  | | | |
| 96940\_at | Tead2 | TEA domain family member 2 | --- | -0.493 | 0 | 0.005 |  | | | |
| 97181\_f\_at | NoneAvailable | --- | --- | -1.659 | 0 | 0.002 |  | | | |
| 97336\_at | Ctsf | cathepsin F | chr19 | -1.534 | 0 | 0.002 |  | | | |
| 97420\_at | Lrg-pending | leucine-rich alpha-2-glycoprotein | chr17 | -0.609 | 0 | 0.026 |  | | | |
| 97448\_at | NoneAvailable | Mus musculus cDNA clone MGC:65558 IMAGE:6485174, complete cds | chr11 | -1.982 | 0 | 0.019 |  | | | |
| 97798\_at | 4930504E06Rik | RIKEN cDNA 4930504E06 gene | chr3 | -1.169 | 0 | 0.001 |  | | | |
| 97817\_at | Spec1-pending | small protein effector 1 of Cdc42 | chr3 | -0.912 | 0 | 0 |  | | | |
| 97943\_at | Capn6 | calpain 6 | chrX | 0.274 | 0 | 0.005 |  | | | |
| 98007\_at | Rps6ka2 | ribosomal protein S6 kinase, polypeptide 2 | chr17 | 0.051 | 0 | 0 |  | | | |
| 98045\_s\_at | Dab2 | disabled homolog 2 (Drosophila) | chr15 | -0.645 | 0 | 0.022 |  | | | |
| 98083\_at | Copeb | core promoter element binding protein | chr13 | -2.963 | 0 | 0 |  | | | |
| 98088\_at | Cd14 | CD14 antigen | --- | -3.218 | 0 | 0.018 |  | | | |
| 98254\_f\_at | NoneAvailable | --- | --- | -1.54 | 0 | 0.002 |  | | | |
| 98369\_f\_at | NoneAvailable | --- | --- | -1.265 | 0 | 0.002 |  | | | |
| 98418\_at | Dvl1 | dishevelled, dsh homolog 1 (Drosophila) | chr4 | -0.479 | 0 | 0.017 |  | | | |
| 98431\_at | Dusp12 | dual specificity phosphatase 12 | chr1 | 0.357 | 0 | 0.015 |  | | | |
| 98451\_at | Dnajb10 | DnaJ (Hsp40) homolog, subfamily B, member 10 | chr1 | -3.902 | 0 | 0.002 |  | | | |
| 98977\_at | Tinf2 | Terf1 (TRF1)-interacting nuclear factor 2 | chr14 | -0.816 | 0 | 0.027 |  | | | |
| 99071\_at | Mpeg1 | macrophage expressed gene 1 | chr19 | 0.525 | 0 | 0.019 |  | | | |
| 99451\_at | C230093N12Rik | RIKEN cDNA C230093N12 gene | chr2 | -0.334 | 0 | 0.041 |  | | | |
| 99640\_at | Minpp1 | multiple inositol polyphosphate histidine phosphatase 1 | chr19 | -0.507 | 0 | 0.033 |  | | | |
| 99945\_at | Cd19 | CD19 antigen | chr7 | 0.104 | 0 | 0.028 |  | | | |
| 100013\_at | 2010008K16Rik | RIKEN cDNA 2010008K16 gene | chr11 | 0.08 | 1 | 0.045 |  | | | |
| 100064\_f\_at | Gja1 | gap junction membrane channel protein alpha 1 | chr10 | -0.741 | 1 | 0.001 |  | | | |
| 100508\_at | Mfng | manic fringe homolog (Drosophila) | chr15 | -0.007 | 1 | 0.001 |  | | | |
| 100516\_at | Chk | choline kinase | chr19 | -0.342 | 1 | 0.033 |  | | | |
| 100535\_at | Eif4g2 | eukaryotic translation initiation factor 4, gamma 2 | chr7 | -0.125 | 1 | 0.013 |  | | | |
| 100564\_at | Ddt | D-dopachrome tautomerase | chr10 | -0.672 | 1 | 0.022 |  | | | |
| 100578\_at | Impdh2 | inosine 5'-phosphate dehydrogenase 2 | chr8 | 0.093 | 1 | 0.007 |  | | | |
| 100587\_f\_at | 5730403B10Rik | RIKEN cDNA 5730403B10 gene | chr16 | -2.058 | 1 | 0.009 |  | | | |
| 100611\_at | Lyzs | lysozyme | chr10 | -0.306 | 1 | 0.033 |  | | | |
| 100629\_at | Gstm5 | glutathione S-transferase, mu 5 | chr3 | 0.731 | 1 | 0.019 |  | | | |
| 100880\_at | NoneAvailable | Mus musculus diabetic nephropathy-related gene 1 mRNA, partial sequence | chr3 | -0.254 | 1 | 0.018 |  | | | |
| 100998\_at | H2-Ab1 | histocompatibility 2, class II antigen A, beta 1 | chr17 | -1.116 | 1 | 0.026 |  | | | |
| 101059\_at | Ndn | necdin | chr7 | -0.839 | 1 | 0.032 |  | | | |
| 101060\_at | Grp58 | glucose regulated protein | chr2 | 0.396 | 1 | 0.013 |  | | | |
| 101398\_at | Stxbp2 | syntaxin binding protein 2 | chr8 | 0.366 | 1 | 0.005 |  | | | |
| 101495\_at | Cd81 | CD 81 antigen | chr7 | -0.022 | 1 | 0.001 |  | | | |
| 101510\_at | Psme1 | proteasome (prosome, macropain) 28 subunit, alpha | chr14 | 0.356 | 1 | 0.019 |  | | | |
| 101554\_at | Nfkbia | nuclear factor of kappa light chain gene enhancer in B-cells inhibitor, alpha | chr12 | -0.607 | 1 | 0.005 |  | | | |
| 101568\_at | NoneAvailable | Mus musculus mRNA similar to proline synthetase co-transcribed (cDNA clone MGC:59396 IMAGE:6504579), complete cds | chr8 | -1.703 | 1 | 0.024 |  | | | |
| 101583\_at | Btg2 | B-cell translocation gene 2, anti-proliferative | chr1 | -0.846 | 1 | 0.013 |  | | | |
| 101584\_at | Rsu1 | Ras suppressor protein 1 | chr2 | 0.552 | 1 | 0.022 |  | | | |
| 101593\_at | Crip2 | cysteine rich protein 2 | --- | -0.033 | 1 | 0.004 |  | | | |
| 101963\_at | Ctsl | cathepsin L | chr13 | -2.894 | 1 | 0.022 |  | | | |
| 101971\_at | 2500002L14Rik | RIKEN cDNA 2500002L14 gene | --- | -1.663 | 1 | 0.005 |  | | | |
| 101979\_at | Gadd45g | growth arrest and DNA-damage-inducible 45 gamma | chr13 | -0.823 | 1 | 0.009 |  | | | |
| 101990\_at | Ldh2 | lactate dehydrogenase 2, B chain | chr6 | -1.289 | 1 | 0.001 |  | | | |
| 102332\_at | Ulk1 | Unc-51 like kinase 1 (C. elegans) | chr5 | -2.225 | 1 | 0.009 |  | | | |
| 102401\_at | Irf1 | interferon regulatory factor 1 | chr11 | -0.554 | 1 | 0.009 |  | | | |
| 102737\_at | Edn1 | endothelin 1 | chr13 | -0.111 | 1 | 0.04 |  | | | |
| 102779\_at | Gadd45b | growth arrest and DNA-damage-inducible 45 beta | chr10 | -0.245 | 1 | 0.038 |  | | | |
| 102809\_s\_at | Lck | lymphocyte protein tyrosine kinase | chr4 | -0.359 | 1 | 0.034 |  | | | |
| 102860\_at | Serpina3g | serine (or cysteine) proteinase inhibitor, clade A, member 3G | chr12 | -4.051 | 1 | 0.013 |  | | | |
| 102906\_at | Tgtp | T-cell specific GTPase | chr11 | -5.973 | 1 | 0.001 |  | | | |
| 102936\_at | B4galt6 | UDP-Gal:betaGlcNAc beta 1,4-galactosyltransferase, polypeptide 6 | --- | -0.027 | 1 | 0.001 |  | | | |
| 102960\_at | Rga | recombination activating gene 1 gene activation | chr3 | -1.165 | 1 | 0.049 |  | | | |
| 102965\_at | NoneAvailable | Mus musculus 13 days embryo forelimb cDNA, RIKEN full-length enriched library, clone:5930414A15 product:hypothetical EF-hand containing protein, full insert sequence | chr2 | -0.732 | 1 | 0 |  | | | |
| 103257\_at | C730036B01Rik | RIKEN cDNA C730036B01 gene | chr3 | 2.122 | 1 | 0.004 |  | | | |
| 103326\_at | E130107N23Rik | RIKEN cDNA E130107N23 gene | chr9 | -0.23 | 1 | 0.017 |  | | | |
| 103353\_f\_at | Cyp4b1 | cytochrome P450, family 4, subfamily b, polypeptide 1 | chr4 | -2.127 | 1 | 0.004 |  | | | |
| 103483\_at | Ercc5 | excision repair cross-complementing rodent repair deficiency,complementation group 5 | chr1 | -0.612 | 1 | 0 |  | | | |
| 103574\_at | Ablim1 | actin-binding LIM protein 1 | chr19 | -0.139 | 1 | 0.022 |  | | | |
| 103634\_at | Isgf3g | interferon dependent positive acting transcription factor 3 gamma | --- | -1.108 | 1 | 0.002 |  | | | |
| 103717\_at | Wwp2-pending | WW domain-containing protein 2 | chr8 | -0.376 | 1 | 0.013 |  | | | |
| 103726\_at | 2610311I19Rik | RIKEN cDNA 2610311I19 gene | chr18 | 0.024 | 1 | 0.021 |  | | | |
| 103899\_at | Atp11a | ATPase, class VI, type 11A | chr8 | -1.465 | 1 | 0.049 |  | | | |
| 104000\_at | 2210023G05Rik | RIKEN cDNA 2210023G05 gene | --- | -0.048 | 1 | 0.001 |  | | | |
| 104002\_at | Zfp275 | Zinc finger protein 275 | chrX | -0.643 | 1 | 0.039 |  | | | |
| 104041\_at | 1810009A16Rik | RIKEN cDNA 1810009A16 gene | --- | -0.783 | 1 | 0.025 |  | | | |
| 104063\_at | Srcasm | Src activating and signaling molecule | chr11 | 0.431 | 1 | 0.01 |  | | | |
| 104208\_at | Pik4ca | phosphatidylinositol 4-kinase, catalytic, alpha polypeptide | chr16 | -0.134 | 1 | 0.004 |  | | | |
| 104427\_at | Mtmr13 | myotubularin related protein 13 | chr7 | -0.132 | 1 | 0.009 |  | | | |
| 104463\_at | 3830408P06Rik | RIKEN cDNA 3830408P06 gene | chr10 | 0.576 | 1 | 0.004 |  | | | |
| 104597\_at | Gbp2 | guanylate nucleotide binding protein 2 | chr3 | -2.148 | 1 | 0.021 |  | | | |
| 104735\_at | AW538430 | expressed sequence AW538430 | chr14 | -1.525 | 1 | 0.049 |  | | | |
| 160081\_at | Rpl44 | ribosomal protein L44 | chr3 | -0.147 | 1 | 0.042 |  | | | |
| 160090\_f\_at | Aldo1 | aldolase 1, A isoform | chr7 | 0.192 | 1 | 0.019 |  | | | |
| 160127\_at | Ccng1 | cyclin G1 | chr11 | -1.675 | 1 | 0.007 |  | | | |
| 160197\_at | 1110058B13Rik | RIKEN cDNA 1110058B13 gene | chr15 | 1.846 | 1 | 0.03 |  | | | |
| 160227\_s\_at | Bysl | bystin-like | chr17 | 1.211 | 1 | 0 |  | | | |
| 160343\_at | Srp19 | signal recognition particle 19 | --- | 0.308 | 1 | 0.027 |  | | | |
| 160361\_at | Trappc4 | trafficking protein particle complex 4 | chr9 | 0.38 | 1 | 0.042 |  | | | |
| 160366\_at | NoneAvailable | Mus musculus 8 days embryo whole body cDNA, RIKEN full-length enriched library, clone:5730496F02 product:HYPOTHETICAL 8.0 KDA PROTEIN homolog [Mus musculus], full insert sequence | chr18 | 0.38 | 1 | 0.029 |  | | | |
| 160428\_at | Suclg2 | succinate-Coenzyme A ligase, GDP-forming, beta subunit | chr6 | 0.005 | 1 | 0.034 |  | | | |
| 160449\_at | Dr1 | down-regulator of transcription 1 | chr5 | -0.571 | 1 | 0.002 |  | | | |
| 160462\_f\_at | Tubb3 | tubulin, beta 3 | chr8 | 0.376 | 1 | 0.037 |  | | | |
| 160476\_f\_at | Rpl18 | ribosomal protein L18 | --- | 0.052 | 1 | 0.014 |  | | | |
| 160502\_at | Creg | cellular repressor of E1A-stimulated genes | --- | -2.676 | 1 | 0.03 |  | | | |
| 160519\_at | Timp3 | tissue inhibitor of metalloproteinase 3 | chr10 | -3.836 | 1 | 0.007 |  | | | |
| 160704\_at | 1110067D22Rik | RIKEN cDNA 1110067D22 gene | chr11 | -0.873 | 1 | 0.012 |  | | | |
| 160722\_at | 4833420N02Rik | RIKEN cDNA 4833420N02 gene | chr11 | 0.416 | 1 | 0.026 |  | | | |
| 160724\_at | Usp49 | ubiquitin specific protease 49 | --- | -2.112 | 1 | 0 |  | | | |
| 160749\_at | 1500011H22Rik | RIKEN cDNA 1500011H22 gene | --- | -0.59 | 1 | 0.013 |  | | | |
| 160783\_at | D14Ertd436e | DNA segment, Chr 14, ERATO Doi 436, expressed | chr14 | -1.857 | 1 | 0.033 |  | | | |
| 160885\_at | 2700010L10Rik | RIKEN cDNA 2700010L10 gene | chr1 | 0.691 | 1 | 0.045 |  | | | |
| 160898\_at | Abt1 | activator of basal transcription | chr13 | -0.064 | 1 | 0.034 |  | | | |
| 160933\_at | Igtp | interferon gamma induced GTPase | chr11 | -3.814 | 1 | 0 |  | | | |
| 160971\_at | AI842353 | expressed sequence AI842353 | chr19 | -0.561 | 1 | 0.008 |  | | | |
| 160982\_at | 4921526G09Rik | RIKEN cDNA 4921526G09 gene | chrX | -0.898 | 1 | 0.018 |  | | | |
| 160998\_at | D4Ertd89e | DNA segment, Chr 4, ERATO Doi 89, expressed | chr4 | -0.023 | 1 | 0.009 |  | | | |
| 161250\_at | Ipo4 | importin 4 | --- | -0.275 | 1 | 0.026 |  | | | |
| 161666\_f\_at | Gadd45b | growth arrest and DNA-damage-inducible 45 beta | chr10 | -2.691 | 1 | 0.042 |  | | | |
| 162044\_f\_at | Cyp4b1 | cytochrome P450, family 4, subfamily b, polypeptide 1 | --- | -2.532 | 1 | 0.007 |  | | | |
| 162125\_f\_at | Ubc | ubiquitin C | chr5 | -0.081 | 1 | 0.035 |  | | | |
| 92198\_s\_at | Daf2 | decay accelerating factor 2 | chr1 | 0.014 | 1 | 0.006 |  | | | |
| 92262\_at | Wig1 | wild-type p53-induced gene 1 | chr3 | -0.291 | 1 | 0 |  | | | |
| 92263\_at | Grcb | gene rich cluster, B gene | chr6 | -1.939 | 1 | 0.002 |  | | | |
| 92401\_at | Ltc4s | leukotriene C4 synthase | chr11 | -0.139 | 1 | 0.011 |  | | | |
| 92440\_at | Irf6 | interferon regulatory factor 6 | chr1 | -4.326 | 1 | 0.031 |  | | | |
| 92653\_at | D530037H12Rik | RIKEN cDNA D530037H12 gene | chr1 | -1.338 | 1 | 0.01 |  | | | |
| 92707\_at | Pvt1 | plasmacytoma variant translocation 1 | --- | 0.022 | 1 | 0.003 |  | | | |
| 92780\_f\_at | NoneAvailable | --- | --- | -2.077 | 1 | 0.017 |  | | | |
| 92847\_s\_at | M6pr | mannose-6-phosphate receptor, cation dependent | chr6 | -1.096 | 1 | 0.043 |  | | | |
| 92866\_at | H2-Aa | histocompatibility 2, class II antigen A, alpha | chr17 | -3.869 | 1 | 0.036 |  | | | |
| 92872\_at | 1200016B17Rik | RIKEN cDNA 1200016B17 gene | chr6 | -0.497 | 1 | 0.031 |  | | | |
| 92926\_at | Mpl | myeloproliferative leukemia virus oncogene | --- | -1.119 | 1 | 0.003 |  | | | |
| 93011\_at | Gabarapl1 | gamma-aminobutyric acid (GABA(A)) receptor-associated protein-like 1 | chr6 | -2.046 | 1 | 0.019 |  | | | |
| 93020\_at | Rex3 | reduced expression 3 | chrX | -1.019 | 1 | 0.009 |  | | | |
| 93021\_at | Drpla | dentatorubral pallidoluysian atrophy | chr11 | 0.351 | 1 | 0.024 |  | | | |
| 93039\_at | 1190003P12Rik | RIKEN cDNA 1190003P12 gene | chr15 | -1.465 | 1 | 0.029 |  | | | |
| 93252\_at | Bcap31 | B-cell receptor-associated protein 31 | chrX | 0.065 | 1 | 0.001 |  | | | |
| 93278\_at | Scp2 | sterol carrier protein 2, liver | chr4 | 0.017 | 1 | 0.024 |  | | | |
| 93294\_at | Ctgf | connective tissue growth factor | chr10 | -0.222 | 1 | 0.045 |  | | | |
| 93318\_at | Ninj1 | ninjurin 1 | chr13 | -0.93 | 1 | 0.031 |  | | | |
| 93324\_at | Zfp36l1 | zinc finger protein 36, C3H type-like 1 | chr12 | -2.308 | 1 | 0.025 |  | | | |
| 93347\_at | Rab24 | RAB24, member RAS oncogene family | chr13 | -0.043 | 1 | 0.019 |  | | | |
| 93536\_at | Bax | Bcl2-associated X protein | chr7 | 0.954 | 1 | 0.007 |  | | | |
| 93543\_f\_at | Gstm1 | glutathione S-transferase, mu 1 | chr5 | -1.363 | 1 | 0.008 |  | | | |
| 93751\_at | 8430421I07Rik | RIKEN cDNA 8430421I07 gene | chr9 | -0.171 | 1 | 0.026 |  | | | |
| 93776\_at | 1500001L15Rik | RIKEN cDNA 1500001L15 gene | chr14 | 0.001 | 1 | 0.01 |  | | | |
| 93782\_at | Rnf4 | ring finger protein 4 | chr5 | -0.534 | 1 | 0.013 |  | | | |
| 93789\_s\_at | Sin3b | transcriptional regulator, SIN3B (yeast) | chr8 | -0.358 | 1 | 0.015 |  | | | |
| 93835\_at | Fuca | fucosidase, alpha-L- 1, tissue | chr4 | -0.701 | 1 | 0.021 |  | | | |
| 93971\_f\_at | Psmd12 | proteasome (prosome, macropain) 26S subunit, non-ATPase, 12 | chr11 | -0.122 | 1 | 0.019 |  | | | |
| 94000\_at | Cd8b | CD8 antigen, beta chain | chr5 | 0.23 | 1 | 0.024 |  | | | |
| 94021\_at | Trim11 | tripartite motif protein 11 | chr11 | -0.105 | 1 | 0.031 |  | | | |
| 94068\_at | Rps19 | ribosomal protein S19 | --- | 0.057 | 1 | 0 |  | | | |
| 94073\_at | Polr2g | polymerase (RNA) II (DNA directed) polypeptide G | chr19 | 1.881 | 1 | 0.001 |  | | | |
| 94246\_at | Ets2 | E26 avian leukemia oncogene 2, 3' domain | --- | 0.191 | 1 | 0.038 |  | | | |
| 94269\_at | Rabac1 | Rab acceptor 1 (prenylated) | chr7 | -1.557 | 1 | 0.015 |  | | | |
| 94270\_at | Krt1-18 | keratin complex 1, acidic, gene 18 | --- | -3.531 | 1 | 0.013 |  | | | |
| 94274\_at | 6720465F12Rik | RIKEN cDNA 6720465F12 gene | --- | 1.041 | 1 | 0.001 |  | | | |
| 94285\_at | H2-Eb1 | histocompatibility 2, class II antigen E beta | chr17 | -0.946 | 1 | 0 |  | | | |
| 94502\_at | D13Wsu50e | DNA segment, Chr 13, Wayne State University 50, expressed | chr13 | -0.107 | 1 | 0.023 |  | | | |
| 94522\_at | Dctn3 | dynactin 3 | chr4 | 0.552 | 1 | 0.037 |  | | | |
| 94715\_at | Cyp1a1 | cytochrome P450, family 1, subfamily a, polypeptide 1 | --- | -0.097 | 1 | 0.005 |  | | | |
| 94799\_at | F8 | coagulation factor VIII | chrX | -0.375 | 1 | 0.028 |  | | | |
| 94821\_at | Xbp1 | X-box binding protein 1 | --- | -2.107 | 1 | 0.021 |  | | | |
| 94823\_at | NoneAvailable | Mus musculus, clone IMAGE:3586350, mRNA, partial cds | chr10 | -0.477 | 1 | 0.011 |  | | | |
| 94835\_f\_at | Tubb2 | tubulin, beta 2 | chr13 | -2.52 | 1 | 0.002 |  | | | |
| 94881\_at | Cdkn1a | cyclin-dependent kinase inhibitor 1A (P21) | chr17 | -2.727 | 1 | 0.002 |  | | | |
| 94941\_at | Eif2ak4 | eukaryotic translation initiation factor 2 alpha kinase 4 | chr2 | 0.14 | 1 | 0.002 |  | | | |
| 95478\_at | Deb1 | differentially expressed in B16F10 1 | chr9 | 0.513 | 1 | 0.012 |  | | | |
| 95496\_at | 5730409F23Rik | RIKEN cDNA 5730409F23 gene | chr11 | 0.428 | 1 | 0.035 |  | | | |
| 95505\_at | Tor1b | torsin family 1, member B | --- | -1.061 | 1 | 0.039 |  | | | |
| 95508\_at | Nckap1 | NCK-associated protein 1 | chr2 | -4.402 | 1 | 0.021 |  | | | |
| 95547\_at | Arhd | ras homolog gene family, member D | chr19 | -0.192 | 1 | 0.01 |  | | | |
| 95666\_at | 9430009J09Rik | RIKEN cDNA 9430009J09 gene | chr1 | -0.229 | 1 | 0.014 |  | | | |
| 95715\_at | 1200009C21Rik | RIKEN cDNA 1200009C21 gene | chr7 | 0.656 | 1 | 0.043 |  | | | |
| 95737\_at | 1200015A19Rik | RIKEN cDNA 1200015A19 gene | chr4 | -2.325 | 1 | 0.023 |  | | | |
| 95913\_at | C230056F04Rik | RIKEN cDNA C230056F04 gene | chrX | -0.335 | 1 | 0.042 |  | | | |
| 96058\_s\_at | Aldh2 | aldehyde dehydrogenase 2, mitochondrial | chr5 | -0.942 | 1 | 0.037 |  | | | |
| 96146\_at | Btg3 | B-cell translocation gene 3 | chr16 | -2.704 | 1 | 0.035 |  | | | |
| 96158\_at | BC017133 | cDNA sequence BC017133 | --- | -0.57 | 1 | 0.006 |  | | | |
| 96592\_at | Pik3r1 | phosphatidylinositol 3-kinase, regulatory subunit, polypeptide 1 (p85 alpha) | chr13 | 0.081 | 1 | 0.03 |  | | | |
| 96596\_at | Ndrl | N-myc downstream regulated-like | chr15 | -4.908 | 1 | 0.007 |  | | | |
| 96614\_at | 4933426M11Rik | RIKEN cDNA 4933426M11 gene | chr12 | -1.2 | 1 | 0.039 |  | | | |
| 96703\_at | Maged1 | melanoma antigen, family D, 1 | chrX | -2.002 | 1 | 0.003 |  | | | |
| 96728\_at | DXImx38e | DNA segment, Chr X, Immunex 38, expressed | chrX | -2.856 | 1 | 0.001 |  | | | |
| 96752\_at | Icam1 | intercellular adhesion molecule | chr9 | -2.045 | 1 | 0.024 |  | | | |
| 96764\_at | Iigp-pending | interferon-inducible GTPase | --- | -5.077 | 1 | 0.009 |  | | | |
| 96785\_at | 0610013D04Rik | RIKEN cDNA 0610013D04 gene | chr17 | -0.743 | 1 | 0.003 |  | | | |
| 96801\_at | Ak1 | adenylate kinase 1 | chr2 | -0.22 | 1 | 0.004 |  | | | |
| 96831\_at | Pdir-pending | protein disulfide isomerase-related | --- | 0.318 | 1 | 0 |  | | | |
| 96876\_at | Laptm4a | lysosomal-associated protein transmembrane 4A | chr12 | -1.037 | 1 | 0.039 |  | | | |
| 96884\_at | Carhsp1 | calcium regulated heat stable protein 1 | --- | -0.717 | 1 | 0 |  | | | |
| 96890\_at | 1300002A08Rik | RIKEN cDNA 1300002A08 gene | chr8 | -0.314 | 1 | 0.048 |  | | | |
| 96935\_at | 2700030M23Rik | RIKEN cDNA 2700030M23 gene | chr4 | -2.968 | 1 | 0.003 |  | | | |
| 96936\_at | Copg1 | coatomer protein complex, subunit gamma 1 | chr6 | -0.064 | 1 | 0.042 |  | | | |
| 97060\_at | Ywhaq | tyrosine 3-monooxygenase/tryptophan 5-monooxygenase activation protein, theta polypeptide | --- | 0.172 | 1 | 0.043 |  | | | |
| 97107\_at | 1700007D05Rik | RIKEN cDNA 1700007D05 gene | chr10 | -0.34 | 1 | 0.021 |  | | | |
| 97148\_at | NoneAvailable | Mus musculus transcribed sequences | --- | 0.197 | 1 | 0.022 |  | | | |
| 97320\_at | 1600025H15Rik | RIKEN cDNA 1600025H15 gene | chr15 | -0.215 | 1 | 0.002 |  | | | |
| 97352\_f\_at | Coxvib2 | cytochrome c oxidase subunit VIb, testes-specific | chr7 | -0.255 | 1 | 0.002 |  | | | |
| 97401\_at | 1300006C06Rik | RIKEN cDNA 1300006C06 gene | chr15 | -0.573 | 1 | 0.001 |  | | | |
| 97409\_at | Ifi1 | interferon inducible protein 1 | chr11 | -1.031 | 1 | 0.034 |  | | | |
| 97487\_at | Serpine2 | serine (or cysteine) proteinase inhibitor, clade E, member 2 | chr1 | -0.891 | 1 | 0.011 |  | | | |
| 97515\_at | Hsd17b4 | hydroxysteroid (17-beta) dehydrogenase 4 | chr18 | 0.524 | 1 | 0.006 |  | | | |
| 97540\_f\_at | H2-D1 | histocompatibility 2, D region locus 1 | --- | -3.787 | 1 | 0.002 |  | | | |
| 97549\_at | Cfl2 | cofilin 2, muscle | chr12 | -2.749 | 1 | 0.03 |  | | | |
| 97718\_at | Ctla4 | cytotoxic T-lymphocyte-associated protein 4 | chr1 | -0.129 | 1 | 0.043 |  | | | |
| 97880\_at | Dlst | dihydrolipoamide S-succinyltransferase (E2 component of 2-oxo-glutarate complex) | chr12 | -0.165 | 1 | 0.028 |  | | | |
| 97890\_at | Sgk | serum/glucocorticoid regulated kinase | chr10 | -3.498 | 1 | 0.048 |  | | | |
| 97908\_at | 1110007A06Rik | RIKEN cDNA 1110007A06 gene | chr6 | -1.119 | 1 | 0.04 |  | | | |
| 97973\_at | Tal1 | T-cell acute lymphocytic leukemia 1 | chr4 | -1.383 | 1 | 0.032 |  | | | |
| 97984\_i\_at | NoneAvailable | Mus musculus 0 day neonate thymus cDNA, RIKEN full-length enriched library, clone:A430045K06 product:hypothetical Type I antifreeze protein containing protein, full insert sequence | --- | 0.534 | 1 | 0.023 |  | | | |
| 98053\_at | Ywhab | tyrosine 3-monooxygenase/tryptophan 5-monooxygenase activation protein, beta polypeptide | --- | 0.019 | 1 | 0.049 |  | | | |
| 98056\_at | Phlda3 | pleckstrin homology-like domain, family A, member 3 | chr1 | 0.009 | 1 | 0.021 |  | | | |
| 98067\_at | Cdkn1a | cyclin-dependent kinase inhibitor 1A (P21) | chr17 | -2.741 | 1 | 0.001 |  | | | |
| 98410\_at | Gtpi-pending | interferon-g induced GTPase | chr11 | -2.836 | 1 | 0.046 |  | | | |
| 98472\_at | H2-T23 | histocompatibility 2, T region locus 23 | --- | -1.61 | 1 | 0 |  | | | |
| 98569\_at | 1110030N17Rik | RIKEN cDNA 1110030N17 gene | chr2 | -0.46 | 1 | 0.006 |  | | | |
| 98574\_at | Prpf8 | pre-mRNA processing factor 8 | chr11 | -0.114 | 1 | 0.024 |  | | | |
| 98600\_at | S100a11 | S100 calcium binding protein A11 (calizzarin) | chr4 | -0.349 | 1 | 0.024 |  | | | |
| 98633\_at | 1200015A22Rik | RIKEN cDNA 1200015A22 gene | chr6 | -0.347 | 1 | 0.007 |  | | | |
| 98887\_at | Napa | N-ethylmaleimide sensitive fusion protein attachment protein alpha | --- | -0.084 | 1 | 0.048 |  | | | |
| 99109\_at | Ier2 | immediate early response 2 | chr8 | -2.892 | 1 | 0.003 |  | | | |
| 99133\_at | Slc3a2 | solute carrier family 3 (activators of dibasic and neutral amino acid transport), member 2 | chr19 | -1.113 | 1 | 0.018 |  | | | |
| 99154\_s\_at | 1810020D17Rik | RIKEN cDNA 1810020D17 gene | chr7 | 0.132 | 1 | 0.003 |  | | | |
| 99340\_at | Gnb2-rs1 | guanine nucleotide binding protein, beta 2, related sequence 1 | chr11 | -0.042 | 1 | 0.039 |  | | | |
| 99366\_at | E030024M05Rik | RIKEN cDNA E030024M05 gene | chr12 | -2.996 | 1 | 0.014 |  | | | |
| 99532\_at | Tob1 | transducer of ErbB-2.1 | chr11 | -5.048 | 1 | 0.008 |  | | | |
| 99577\_at | Kitl | kit ligand | chr10 | -0.059 | 1 | 0.022 |  | | | |
| 99629\_at | Ei24 | etoposide induced 2.4 mRNA | chr9 | -0.596 | 1 | 0 |  | | | |
| 99909\_at | Trpc6 | transient receptor potential cation channel, subfamily C, member 6 | chr9 | -0.627 | 1 | 0.031 |  | | | |
| 100066\_at | Gart | phosphoribosylglycinamide formyltransferase | chr16 | 1.415 | 2 | 0.007 |  | | | |
| 100574\_f\_at | Gpi1 | glucose phosphate isomerase 1 | chr7 | 0.171 | 2 | 0.041 |  | | | |
| 100753\_at | Atp5a1 | ATP synthase, H+ transporting, mitochondrial F1 complex, alpha subunit, isoform 1 | --- | -0.077 | 2 | 0.029 |  | | | |
| 101042\_f\_at | Pep4 | peptidase 4 | --- | 0.494 | 2 | 0.003 |  | | | |
| 101461\_f\_at | Pja1 | praja1, RING-H2 motif containing | chrX | 0.613 | 2 | 0.034 |  | | | |
| 101466\_at | Pigf | phosphatidylinositol glycan, class F | chr17 | 0.537 | 2 | 0.006 |  | | | |
| 101518\_at | 0610009H04Rik | RIKEN cDNA 0610009H04 gene | chr11 | 0.157 | 2 | 0.019 |  | | | |
| 101959\_r\_at | Tfdp1 | transcription factor Dp 1 | chr8 | 1.111 | 2 | 0.004 |  | | | |
| 101984\_at | Atox1 | ATX1 (antioxidant protein 1) homolog 1 (yeast) | chr11 | 0.652 | 2 | 0.025 |  | | | |
| 102124\_f\_at | Cox4i1 | cytochrome c oxidase subunit IV isoform 1 | chr8 | 0.279 | 2 | 0.035 |  | | | |
| 102649\_s\_at | Raet1c | retinoic acid early transcript gamma | --- | 0.219 | 2 | 0.038 |  | | | |
| 103057\_at | Pold1 | polymerase (DNA directed), delta 1, catalytic subunit | chr7 | 3.176 | 2 | 0.034 |  | | | |
| 103377\_at | Lrp2 | low density lipoprotein receptor-related protein 2 | chr2 | 0.013 | 2 | 0.037 |  | | | |
| 103862\_r\_at | D7Wsu128e | DNA segment, Chr 7, Wayne State University 128, expressed | chr7 | -0.099 | 2 | 0.042 |  | | | |
| 103873\_i\_at | 2310015N07Rik | RIKEN cDNA 2310015N07 gene | --- | 0.684 | 2 | 0.022 |  | | | |
| 103874\_r\_at | 2310015N07Rik | RIKEN cDNA 2310015N07 gene | --- | 0.141 | 2 | 0.016 |  | | | |
| 104089\_at | 2810026P18Rik | RIKEN cDNA 2810026P18 gene | chr9 | 0.457 | 2 | 0.023 |  | | | |
| 104303\_i\_at | 1500004O14Rik | RIKEN cDNA 1500004O14 gene | chr2 | 1.473 | 2 | 0.007 |  | | | |
| 104304\_r\_at | 1500004O14Rik | RIKEN cDNA 1500004O14 gene | --- | 0.905 | 2 | 0.011 |  | | | |
| 104305\_at | Rarsl | arginyl-tRNA synthetase-like | chr4 | 2.295 | 2 | 0.005 |  | | | |
| 104343\_f\_at | Pla2g12 | phospholipase A2, group XII | chr3 | 0.319 | 2 | 0.003 |  | | | |
| 104662\_at | Tnfrsf18 | tumor necrosis factor receptor superfamily, member 18 | chr4 | -0.049 | 2 | 0.002 |  | | | |
| 160291\_at | Sec61a1 | Sec61 alpha 1 subunit (S. cerevisiae) | chr6 | -0.097 | 2 | 0.007 |  | | | |
| 160341\_at | Jtv1-pending | JTV1 gene | chr5 | 2.15 | 2 | 0.033 |  | | | |
| 160633\_at | Refbp1 | RNA and export factor binding protein 1 | --- | 0.652 | 2 | 0.028 |  | | | |
| 160803\_at | D0H8S2298E | DNA segment, Human S2298E | chr8 | -0.598 | 2 | 0.002 |  | | | |
| 161386\_f\_at | Ddx1 | DEAD (Asp-Glu-Ala-Asp) box polypeptide 1 | --- | 0.123 | 2 | 0.048 |  | | | |
| 161400\_f\_at | NoneAvailable | --- | --- | 0.17 | 2 | 0.002 |  | | | |
| 161466\_r\_at | Asb3 | ankyrin repeat and SOCS box-containing protein 3 | --- | 0.163 | 2 | 0.046 |  | | | |
| 161635\_f\_at | 1110008H02Rik | RIKEN cDNA 1110008H02 gene | chr1 | 0.113 | 2 | 0.019 |  | | | |
| 161703\_f\_at | NoneAvailable | --- | --- | 0.175 | 2 | 0 |  | | | |
| 162131\_f\_at | NoneAvailable | Mus musculus transcribed sequence | --- | 0.274 | 2 | 0.026 |  | | | |
| 162301\_f\_at | 2310016M24Rik | RIKEN cDNA 2310016M24 gene | chr15 | -0.1 | 2 | 0.011 |  | | | |
| 92633\_at | Ctsz | cathepsin Z | chr2 | 0.866 | 2 | 0.003 |  | | | |
| 92794\_f\_at | Nme1 | expressed in non-metastatic cells 1, protein | chr11 | 2.156 | 2 | 0 |  | | | |
| 92810\_at | Pdk3 | pyruvate dehydrogenase kinase, isoenzyme 3 | chrX | -0.996 | 2 | 0.017 |  | | | |
| 93038\_f\_at | Anxa1 | annexin A1 | chr19 | 0.074 | 2 | 0 |  | | | |
| 93070\_at | Kpnb3 | karyopherin (importin) beta 3 | chr14 | 0.784 | 2 | 0.004 |  | | | |
| 93352\_at | Tgm2 | transglutaminase 2, C polypeptide | --- | -0.153 | 2 | 0.034 |  | | | |
| 93529\_at | 2310047C21Rik | RIKEN cDNA 2310047C21 gene | chr8 | 0.295 | 2 | 0.013 |  | | | |
| 93740\_at | Nsep1 | nuclease sensitive element binding protein 1 | --- | 0.564 | 2 | 0.038 |  | | | |
| 93822\_at | Rpl37a | ribosomal protein L37a | --- | 0.979 | 2 | 0.019 |  | | | |
| 93968\_at | Pdcd5 | programmed cell death 5 | chr7 | 0.125 | 2 | 0.024 |  | | | |
| 94450\_at | D13Wsu123e | DNA segment, Chr 13, Wayne State University 123, expressed | chr13 | 1.019 | 2 | 0.014 |  | | | |
| 95034\_f\_at | Ipo4 | importin 4 | chr14 | -0.595 | 2 | 0.002 |  | | | |
| 95709\_at | D7Wsu86e | DNA segment, Chr 7, Wayne State University 86, expressed | chr7 | -0.199 | 2 | 0.025 |  | | | |
| 95755\_at | Csda | cold shock domain protein A | --- | 0.101 | 2 | 0.02 |  | | | |
| 96042\_at | Sod2 | superoxide dismutase 2, mitochondrial | chr17 | 0.163 | 2 | 0.002 |  | | | |
| 96827\_at | Cad | carbamoyl-phosphate synthetase 2, aspartate transcarbamylase, and dihydroorotase | chr5 | 1.06 | 2 | 0.014 |  | | | |
| 96855\_at | 5730406I15Rik | RIKEN cDNA 5730406I15 gene | --- | 0.755 | 2 | 0.018 |  | | | |
| 97272\_at | Hnrpa1 | heterogeneous nuclear ribonucleoprotein A1 | --- | 0.019 | 2 | 0.041 |  | | | |
| 97496\_f\_at | 6330514M23Rik | RIKEN cDNA 6330514M23 gene | chr7 | -1.202 | 2 | 0.002 |  | | | |
| 98099\_at | Nudt9 | nudix (nucleoside diphosphate linked moiety X)-type motif 9 | --- | 0.494 | 2 | 0.048 |  | | | |
| 98127\_at | Capza2 | capping protein (actin filament) muscle Z-line, alpha 2 | chr6 | -1.156 | 2 | 0.03 |  | | | |
| 98573\_r\_at | Ranbp1 | RAN binding protein 1 | chr16 | 1.404 | 2 | 0.046 |  | | | |
| 99158\_at | Sh3d3 | SH3 domain protein 3 | chr19 | 1.284 | 2 | 0.048 |  | | | |
| 100062\_at | Mcm3 | minichromosome maintenance deficient 3 (S. cerevisiae) | chr1 | 1.792 | 3 | 0.005 |  | | | |
| 100225\_f\_at | Psmc3 | proteasome (prosome, macropain) 26S subunit, ATPase 3 | chr2 | 0.602 | 3 | 0.01 |  | | | |
| 100290\_f\_at | 6230416J20Rik | RIKEN cDNA 6230416J20 gene | chr4 | 0.452 | 3 | 0.022 |  | | | |
| 101001\_at | 5031439A09Rik | RIKEN cDNA 5031439A09 gene | chr3 | -1.852 | 3 | 0.044 |  | | | |
| 101004\_f\_at | Sfrs3 | splicing factor, arginine/serine-rich 3 (SRp20) | --- | 0.867 | 3 | 0.032 |  | | | |
| 101372\_at | Trip13 | thyroid hormone receptor interactor 13 | chr13 | 1.578 | 3 | 0.014 |  | | | |
| 101445\_at | Dnmt1 | DNA methyltransferase (cytosine-5) 1 | chr9 | 0.6 | 3 | 0.041 |  | | | |
| 101589\_at | Hmgn2 | high mobility group nucleosomal binding domain 2 | chr13 | 0.392 | 3 | 0.016 |  | | | |
| 101920\_at | Pole2 | polymerase (DNA directed), epsilon 2 (p59 subunit) | --- | 1.432 | 3 | 0.025 |  | | | |
| 101957\_f\_at | Adprt1 | ADP-ribosyltransferase (NAD+; poly (ADP-ribose) polymerase) 1 | chr1 | 0.266 | 3 | 0.007 |  | | | |
| 101958\_f\_at | Tfdp1 | transcription factor Dp 1 | chr8 | 0.364 | 3 | 0.003 |  | | | |
| 102047\_at | Nmt1 | N-myristoyltransferase 1 | chr11 | 1.098 | 3 | 0.008 |  | | | |
| 102103\_f\_at | NoneAvailable | --- | --- | 1.173 | 3 | 0 |  | | | |
| 102105\_f\_at | Ptgds | prostaglandin D2 synthase (brain) | chr2 | 0.54 | 3 | 0.024 |  | | | |
| 102128\_f\_at | Mrps25 | mitochondrial ribosomal protein S25 | --- | 1.148 | 3 | 0.01 |  | | | |
| 102134\_f\_at | Atp5g2 | ATP synthase, H+ transporting, mitochondrial F0 complex, subunit c (subunit 9), isoform 2 | --- | 0.56 | 3 | 0.011 |  | | | |
| 102280\_at | Pcdh7 | protocadherin 7 | chr5 | 0.597 | 3 | 0.032 |  | | | |
| 102848\_f\_at | 2610524H06Rik | RIKEN cDNA 2610524H06 gene | --- | 0.197 | 3 | 0.026 |  | | | |
| 103064\_at | Chek1 | checkpoint kinase 1 homolog (S. pombe) | chr9 | 1.251 | 3 | 0.01 |  | | | |
| 103094\_at | Serf1 | small EDRK-rich factor 1 | chr13 | 0.583 | 3 | 0.009 |  | | | |
| 103201\_at | Ttk | Ttk protein kinase | chr9 | 2.66 | 3 | 0.018 |  | | | |
| 103203\_f\_at | NoneAvailable | Mus musculus transcribed sequence with moderate similarity to protein ref:NP\_078956.1 (H.sapiens)  hypothetical protein FLJ23311 [Homo sapiens] | chr7 | 2.324 | 3 | 0.019 |  | | | |
| 103204\_r\_at | NoneAvailable | Mus musculus transcribed sequence with moderate similarity to protein ref:NP\_078956.1 (H.sapiens)  hypothetical protein FLJ23311 [Homo sapiens] | chr7 | 1.738 | 3 | 0.048 |  | | | |
| 103207\_at | Pola1 | polymerase (DNA directed), alpha 1 | chrX | 2.502 | 3 | 0.025 |  | | | |
| 103212\_at | BC006933 | cDNA sequence BC006933 | chr12 | 1.151 | 3 | 0.045 |  | | | |
| 103240\_f\_at | Rnase2 | ribonuclease, RNase A family, 2 | --- | 0.537 | 3 | 0.016 |  | | | |
| 103444\_at | E130315B21Rik | RIKEN cDNA E130315B21 gene | chr10 | 1.296 | 3 | 0.016 |  | | | |
| 103797\_at | Cdc7 | cell division cycle 7 (S. cerevisiae) | chr5 | 0.917 | 3 | 0.008 |  | | | |
| 103821\_at | Cdc6 | cell division cycle 6 homolog (S. cerevisiae) | --- | 2.833 | 3 | 0.016 |  | | | |
| 103910\_at | NoneAvailable | --- | chr10 | 0.788 | 3 | 0.045 |  | | | |
| 103944\_at | Rad51l1 | RAD51-like 1 (S. cerevisiae) | chr12 | 0.358 | 3 | 0.006 |  | | | |
| 104058\_at | 1110018J12Rik | RIKEN cDNA 1110018J12 gene | chr17 | 0.83 | 3 | 0.027 |  | | | |
| 104258\_at | Acyp2 | acylphosphatase 2, muscle type | chr11 | -0.071 | 3 | 0.046 |  | | | |
| 104313\_at | Pgm2 | phosphoglucomutase 2 | chr4 | 0.87 | 3 | 0 |  | | | |
| 160069\_at | Gmnn | geminin | chr13 | 2.598 | 3 | 0.009 |  | | | |
| 160377\_at | Tardbp | TAR DNA binding protein | chr4 | 0.603 | 3 | 0.017 |  | | | |
| 160496\_s\_at | Mcm3 | minichromosome maintenance deficient 3 (S. cerevisiae) | chr1 | 1.482 | 3 | 0.021 |  | | | |
| 160518\_at | 1110060F11Rik | RIKEN cDNA 1110060F11 gene | chr4 | 0.42 | 3 | 0.019 |  | | | |
| 160595\_at | 2310042P20Rik | RIKEN cDNA 2310042P20 gene | chr15 | 0.875 | 3 | 0.031 |  | | | |
| 160907\_at | C81234 | expressed sequence C81234 | chrX | 0.285 | 3 | 0.016 |  | | | |
| 160988\_r\_at | D3Ertd330e | DNA segment, Chr 3, ERATO Doi 330, expressed | chr3 | 0.265 | 3 | 0 |  | | | |
| 161038\_at | Prps2 | phosphoribosyl pyrophosphate synthetase 2 | chrX | 0.523 | 3 | 0.031 |  | | | |
| 161122\_f\_at | Ndufab1 | NADH dehydrogenase (ubiquinone) 1, alpha/beta subcomplex, 1 | --- | 1.68 | 3 | 0.003 |  | | | |
| 161134\_at | 2610201A13Rik | RIKEN cDNA 2610201A13 gene | --- | 0.08 | 3 | 0.01 |  | | | |
| 161232\_r\_at | Gpiap1 | GPI-anchored membrane protein 1 | --- | 0.227 | 3 | 0.019 |  | | | |
| 161268\_f\_at | Gcs1 | glucosidase 1 | --- | 0.563 | 3 | 0.036 |  | | | |
| 161492\_i\_at | NoneAvailable | --- | --- | 0.081 | 3 | 0.017 |  | | | |
| 161757\_f\_at | NoneAvailable | Mus musculus transcribed sequence | chr14 | -0.117 | 3 | 0.001 |  | | | |
| 161787\_f\_at | Ris2 | retroviral integration site 2 | chr8 | 0.401 | 3 | 0.018 |  | | | |
| 162009\_f\_at | Msh2 | mutS homolog 2 (E. coli) | --- | 0.28 | 3 | 0.03 |  | | | |
| 162327\_f\_at | Ndufv2 | NADH dehydrogenase (ubiquinone) flavoprotein 2 | --- | 0.242 | 3 | 0.022 |  | | | |
| 92478\_at | Stag1 | stromal antigen 1 | chr9 | 0.82 | 3 | 0.014 |  | | | |
| 92551\_at | Lig1 | ligase I, DNA, ATP-dependent | chr7 | 1.152 | 3 | 0.033 |  | | | |
| 92561\_at | Entpd5 | ectonucleoside triphosphate diphosphohydrolase 5 | chr12 | -0.37 | 3 | 0.036 |  | | | |
| 92593\_at | Osf2-pending | osteoblast specific factor 2 (fasciclin I-like) | chr3 | 1.316 | 3 | 0.006 |  | | | |
| 92647\_at | Rbbp4 | retinoblastoma binding protein 4 | chr4 | -0.165 | 3 | 0.028 |  | | | |
| 92782\_at | Tmpo | thymopoietin | chr10 | 0.653 | 3 | 0 |  | | | |
| 93228\_at | Hells | helicase, lymphoid specific | chr19 | 0.826 | 3 | 0.023 |  | | | |
| 93248\_at | D730042P09Rik | RIKEN cDNA D730042P09 gene | chr9 | 0.282 | 3 | 0.011 |  | | | |
| 93333\_at | Tbca | tubulin cofactor a | chr13 | 0.666 | 3 | 0.002 |  | | | |
| 93439\_f\_at | Pawr | PRKC, apoptosis, WT1, regulator | chr10 | -0.266 | 3 | 0.017 |  | | | |
| 93445\_at | Cd5l | CD5 antigen-like | --- | 1.122 | 3 | 0.013 |  | | | |
| 93459\_s\_at | Fzd4 | frizzled homolog 4 (Drosophila) | chr7 | -2.581 | 3 | 0.006 |  | | | |
| 94078\_at | 1110020P15Rik | RIKEN cDNA 1110020P15 gene | --- | 0.74 | 3 | 0.014 |  | | | |
| 94228\_at | Xpo1 | exportin 1, CRM1 homolog (yeast) | chr11 | 1.337 | 3 | 0.001 |  | | | |
| 94376\_s\_at | Mre11a | meiotic recombination 11 homolog A (S. cerevisiae) | chr9 | 2.127 | 3 | 0.035 |  | | | |
| 94534\_at | Idh3a | isocitrate dehydrogenase 3 (NAD+) alpha | chr9 | 0.587 | 3 | 0.023 |  | | | |
| 94788\_f\_at | Tubb5 | tubulin, beta 5 | chr17 | 1.367 | 3 | 0.026 |  | | | |
| 94907\_f\_at | 1110001J03Rik | RIKEN cDNA 1110001J03 gene | chr6 | 1.71 | 3 | 0.003 |  | | | |
| 95063\_at | 2310021G01Rik | RIKEN cDNA 2310021G01 gene | chr2 | 2.4 | 3 | 0.041 |  | | | |
| 95084\_f\_at | Grhpr | glyoxylate reductase/hydroxypyruvate reductase | chr4 | 2.569 | 3 | 0.006 |  | | | |
| 95118\_r\_at | Kif22 | kinesin family member 22 | chr7 | 0.237 | 3 | 0.034 |  | | | |
| 95131\_f\_at | Ndufb2 | NADH dehydrogenase (ubiquinone) 1 beta subcomplex, 2 | chr2 | 1.121 | 3 | 0.003 |  | | | |
| 95292\_at | Itga4 | integrin alpha 4 | chr2 | 1.618 | 3 | 0.02 |  | | | |
| 95456\_r\_at | Shfdg1 | split hand/foot deleted gene 1 | chr6 | 1.322 | 3 | 0.026 |  | | | |
| 95462\_at | Bzw2 | basic leucine zipper and W2 domains 2 | chr12 | 1.226 | 3 | 0.049 |  | | | |
| 95527\_at | Chaf1a | chromatin assembly factor 1, subunit A (p150) | chr17 | 1.001 | 3 | 0 |  | | | |
| 95611\_at | Lpl | lipoprotein lipase | chr8 | -0.268 | 3 | 0.028 |  | | | |
| 95612\_at | Rfc5 | replication factor C (activator 1) 5 | --- | 2.137 | 3 | 0.009 |  | | | |
| 95703\_at | Uble1a | ubiquitin-like 1 (sentrin) activating enzyme E1A | --- | 0.674 | 3 | 0.022 |  | | | |
| 95732\_at | 1110005L13Rik | RIKEN cDNA 1110005L13 gene | chr10 | 2.644 | 3 | 0.028 |  | | | |
| 95927\_f\_at | 2610201A13Rik | RIKEN cDNA 2610201A13 gene | chr11 | 1.536 | 3 | 0.02 |  | | | |
| 95967\_at | NoneAvailable | Mus musculus transcribed sequences | chr13 | -0.136 | 3 | 0.035 |  | | | |
| 96156\_at | 1110008H02Rik | RIKEN cDNA 1110008H02 gene | chr1 | 0.334 | 3 | 0.001 |  | | | |
| 96280\_at | Ndufa2 | NADH dehydrogenase (ubiquinone) 1 alpha subcomplex, 2 | chr18 | 0.957 | 3 | 0.002 |  | | | |
| 96625\_at | D630024B06Rik | RIKEN cDNA D630024B06 gene | chr14 | 1.795 | 3 | 0.01 |  | | | |
| 96629\_at | D7Rp2e | DNA segment, Chr 7, Roswell Park 2 complex, expressed | --- | 0.862 | 3 | 0.034 |  | | | |
| 96686\_i\_at | 2010100O12Rik | RIKEN cDNA 2010100O12 gene | chr2 | 1.279 | 3 | 0.005 |  | | | |
| 96687\_f\_at | 2010100O12Rik | RIKEN cDNA 2010100O12 gene | chr2 | 1.093 | 3 | 0 |  | | | |
| 96710\_at | C530002L11Rik | RIKEN cDNA C530002L11 gene | chr11 | 0.039 | 3 | 0.007 |  | | | |
| 96858\_at | Pdcd8 | programmed cell death 8 | chrX | 0.932 | 3 | 0.013 |  | | | |
| 96891\_at | Anp32b | acidic nuclear phosphoprotein 32 family, member B | chr4 | 0.977 | 3 | 0.024 |  | | | |
| 96916\_at | Mrpl33 | mitochondrial ribosomal protein L33 | chr5 | -0.061 | 3 | 0.009 |  | | | |
| 97095\_at | Bub1 | budding uninhibited by benzimidazoles 1 homolog (S. cerevisiae) | --- | 3.241 | 3 | 0.008 |  | | | |
| 97358\_at | Lphn1 | latrophilin 1 | chr8 | 0.169 | 3 | 0.021 |  | | | |
| 97393\_at | Vrk1 | vaccinia related kinase 1 | chr12 | 1.381 | 3 | 0.035 |  | | | |
| 97411\_at | Ect2 | ect2 oncogene | chr3 | 2.597 | 3 | 0.019 |  | | | |
| 97492\_at | 0610040B21Rik | RIKEN cDNA 0610040B21 gene | chr4 | 0.189 | 3 | 0.032 |  | | | |
| 97909\_at | Stmn1 | stathmin 1 | chr4 | 0.912 | 3 | 0.004 |  | | | |
| 98072\_r\_at | Dck | deoxycytidine kinase | chr5 | 0.4 | 3 | 0.018 |  | | | |
| 98140\_at | Cdh1 | cadherin 1 | --- | 0.145 | 3 | 0.017 |  | | | |
| 98550\_at | Set | SET translocation | chr1 | 1.359 | 3 | 0.045 |  | | | |
| 98618\_at | Dtymk | deoxythymidylate kinase | chr1 | 1.551 | 3 | 0.004 |  | | | |
| 98922\_at | Itm1 | intergral membrane protein 1 | chr9 | -0.602 | 3 | 0.01 |  | | | |
| 98929\_at | 1110018B13Rik | RIKEN cDNA 1110018B13 gene | chr13 | 1.14 | 3 | 0.032 |  | | | |
| 98956\_at | Tram1 | translocating chain-associating membrane protein 1 | chr1 | -0.019 | 3 | 0.049 |  | | | |
| 98993\_at | Ppp2r5c | protein phosphatase 2, regulatory subunit B (B56), gamma isoform | chr12 | -0.394 | 3 | 0.047 |  | | | |
| 98999\_at | Adsl | adenylosuccinate lyase | chr15 | 2.382 | 3 | 0.036 |  | | | |
| 99111\_at | Skd3 | suppressor of K+ transport defect 3 | chr7 | -0.206 | 3 | 0.017 |  | | | |
| 99119\_at | Cfl1 | cofilin 1, non-muscle | chr19 | 0.325 | 3 | 0.012 |  | | | |
| 99439\_at | Mas1 | MAS1 oncogene | chr17 | 0.063 | 3 | 0.048 |  | | | |
| 99457\_at | Mki67 | antigen identified by monoclonal antibody Ki 67 | chr7 | 2.708 | 3 | 0.002 |  | | | |
| 99475\_at | Socs2 | suppressor of cytokine signaling 2 | chr10 | -3.55 | 3 | 0.012 |  | | | |
| 99513\_at | Pla2g4a | phospholipase A2, group IVA (cytosolic, calcium-dependent) | chr1 | 0.589 | 3 | 0.032 |  | | | |
| 99581\_at | Hint | histidine triad nucleotide binding protein | --- | 1.503 | 3 | 0.038 |  | | | |
| 99631\_f\_at | Cox6a1 | cytochrome c oxidase, subunit VI a, polypeptide 1 | chr5 | 0.75 | 3 | 0.004 |  | | | |
| 99658\_f\_at | 1110025H10Rik | RIKEN cDNA 1110025H10 gene | chr17 | 0.702 | 3 | 0.043 |  | | | |
| 99660\_f\_at | Cox7c | cytochrome c oxidase, subunit VIIc | chr11 | 0.349 | 3 | 0.021 |  | | | |
| 99777\_s\_at | 6230416J20Rik | RIKEN cDNA 6230416J20 gene | chr4 | 0.516 | 3 | 0.01 |  | | | |
| 100039\_at | Tmem4 | transmembrane protein 4 | chr10 | 1.81 | 6 | 0.003 |  | | | |
| 100057\_at | 2510027N19Rik | RIKEN cDNA 2510027N19 gene | chr7 | 3.001 | 6 | 0 |  | | | |
| 100059\_at | Cyba | cytochrome b-245, alpha polypeptide | chr8 | 1.77 | 6 | 0.038 |  | | | |
| 100073\_at | 2510005D08Rik | RIKEN cDNA 2510005D08 gene | chr14 | 2.121 | 6 | 0.012 |  | | | |
| 100079\_at | Ndufb9 | NADH dehydrogenase (ubiquinone) 1 beta subcomplex, 9 | chr15 | 1.058 | 6 | 0.013 |  | | | |
| 100089\_at | Ppic | peptidylprolyl isomerase C | chr18 | -0.435 | 6 | 0.039 |  | | | |
| 100116\_at | 2810417H13Rik | RIKEN cDNA 2810417H13 gene | chr1 | 2.111 | 6 | 0.026 |  | | | |
| 100128\_at | Cdc2a | cell division cycle 2 homolog A (S. pombe) | --- | 4.133 | 6 | 0.002 |  | | | |
| 100156\_at | Mcm5 | minichromosome maintenance deficient 5, cell division cycle 46 (S. cerevisiae) | chr8 | 5.137 | 6 | 0.012 |  | | | |
| 100331\_g\_at | Prdx2 | peroxiredoxin 2 | chr1 | 1.095 | 6 | 0.001 |  | | | |
| 100400\_at | 4921531G14Rik | RIKEN cDNA 4921531G14 gene | chr15 | 0.81 | 6 | 0.045 |  | | | |
| 100459\_at | Rad50 | RAD50 homolog (S. cerevisiae) | --- | 2.337 | 6 | 0.009 |  | | | |
| 100512\_at | Uchl5 | ubiquitin carboxyl-terminal esterase L5 | chr1 | 1.055 | 6 | 0.006 |  | | | |
| 100527\_at | D11Ertd99e | DNA segment, Chr 11, ERATO Doi 99, expressed | chr11 | 2.559 | 6 | 0.038 |  | | | |
| 100539\_at | 2410041A17Rik | RIKEN cDNA 2410041A17 gene | chr4 | 0.756 | 6 | 0.007 |  | | | |
| 100543\_s\_at | Brd7 | bromodomain containing 7 | chr8 | 1.094 | 6 | 0.003 |  | | | |
| 100550\_f\_at | Cox6c | cytochrome c oxidase, subunit VIc | chr15 | 0.466 | 6 | 0 |  | | | |
| 100568\_at | Abce1 | ATP-binding cassette, sub-family E (OABP), member 1 | --- | 1.485 | 6 | 0.011 |  | | | |
| 100569\_at | Anxa2 | annexin A2 | chr9 | -0.123 | 6 | 0.003 |  | | | |
| 100576\_at | Pafah1b3 | platelet-activating factor acetylhydrolase, isoform 1b, alpha1 subunit | --- | 1.257 | 6 | 0.004 |  | | | |
| 100577\_at | Snrpd1 | small nuclear ribonucleoprotein D1 | chr18 | 1.295 | 6 | 0 |  | | | |
| 100588\_at | Psme2 | proteasome (prosome, macropain) 28 subunit, beta | chr14 | 0.648 | 6 | 0 |  | | | |
| 100592\_at | Ghitm | growth hormone inducible transmembrane protein | chr14 | 1.179 | 6 | 0.024 |  | | | |
| 100612\_at | Rrm1 | ribonucleotide reductase M1 | --- | 1.929 | 6 | 0.045 |  | | | |
| 100618\_f\_at | Slc25a5 | solute carrier family 25 (mitochondrial carrier; adenine nucleotide translocator), member 5 | --- | 1.078 | 6 | 0.012 |  | | | |
| 100628\_at | Ndufc1 | NADH dehydrogenase (ubiquinone) 1, subcomplex unknown, 1 | --- | 1.336 | 6 | 0.006 |  | | | |
| 100633\_at | 2810484M10Rik | RIKEN cDNA 2810484M10 gene | --- | -0.323 | 6 | 0.045 |  | | | |
| 100733\_at | Psma2 | proteasome (prosome, macropain) subunit, alpha type 2 | chr13 | 1.64 | 6 | 0.006 |  | | | |
| 100917\_at | NoneAvailable | --- | chr18 | 1.316 | 6 | 0.037 |  | | | |
| 100990\_g\_at | Itgb1bp1 | integrin beta 1 binding protein 1 | --- | 0.369 | 6 | 0.031 |  | | | |
| 101061\_at | Ssr2 | signal sequence receptor, beta | chr3 | 1.296 | 6 | 0.034 |  | | | |
| 101065\_at | Pcna | proliferating cell nuclear antigen | chr19 | 0.251 | 6 | 0.008 |  | | | |
| 101067\_at | 2010005E08Rik | RIKEN cDNA 2010005E08 gene | chr3 | 0.448 | 6 | 0.01 |  | | | |
| 101096\_s\_at | Hs1bp1 | HS1 binding protein | chr2 | 1.123 | 6 | 0.018 |  | | | |
| 101097\_at | BC028768 | cDNA sequence BC028768 | chr19 | 0.818 | 6 | 0.019 |  | | | |
| 101105\_at | Banf1 | barrier to autointegration factor 1 | chr19 | 2.053 | 6 | 0.002 |  | | | |
| 101207\_at | Ppia | peptidylprolyl isomerase A | chr11 | 0.267 | 6 | 0.011 |  | | | |
| 101254\_at | Ran | RAN, member RAS oncogene family | chr2 | 1.149 | 6 | 0.002 |  | | | |
| 101380\_at | Nudt14 | nudix (nucleoside diphosphate linked moiety X)-type motif 14 | chr12 | 0.228 | 6 | 0.033 |  | | | |
| 101407\_at | Frda | Friedreich ataxia | chr19 | 1.288 | 6 | 0.027 |  | | | |
| 101421\_at | Rnf5 | ring finger protein 5 | chr17 | 1.067 | 6 | 0.044 |  | | | |
| 101440\_at | 1110033L15Rik | RIKEN cDNA 1110033L15 gene | chr2 | 0.499 | 6 | 0.027 |  | | | |
| 101486\_at | Psmb10 | proteasome (prosome, macropain) subunit, beta type 10 | chr8 | 1.623 | 6 | 0.012 |  | | | |
| 101506\_at | Snrpa1 | small nuclear ribonucleoprotein polypeptide A' | chr7 | 1.197 | 6 | 0.003 |  | | | |
| 101521\_at | Birc5 | baculoviral IAP repeat-containing 5 | chr11 | 2.475 | 6 | 0.01 |  | | | |
| 101525\_at | Ndufb10 | NADH dehydrogenase (ubiquinone) 1 beta subcomplex, 10 | chr17 | 0.565 | 6 | 0.008 |  | | | |
| 101558\_s\_at | Psmb5 | proteasome (prosome, macropain) subunit, beta type 5 | chr14 | 1.051 | 6 | 0.018 |  | | | |
| 101562\_at | Hsp70-4 | heat shock protein 4 | chr2 | 2.149 | 6 | 0.047 |  | | | |
| 101579\_at | Srp9 | signal recognition particle 9 | chr1 | 0.56 | 6 | 0.028 |  | | | |
| 101580\_at | Cox7b | cytochrome c oxidase subunit VIIb | chr1 | 1.791 | 6 | 0 |  | | | |
| 101634\_at | Npm1 | nucleophosmin 1 | chr11 | 0.582 | 6 | 0.008 |  | | | |
| 101680\_at | Rpl27a | ribosomal protein L27a | chr13 | -0.081 | 6 | 0 |  | | | |
| 101741\_at | NoneAvailable | --- | chr11 | 0.952 | 6 | 0.004 |  | | | |
| 101866\_at | Arfrp1 | ADP-ribosylation factor related protein 1 | chr2 | 0.898 | 6 | 0.011 |  | | | |
| 101890\_f\_at | Dnajc2 | DnaJ (Hsp40) homolog, subfamily C, member 2 | chr5 | 1.708 | 6 | 0.027 |  | | | |
| 101954\_at | H2afz | H2A histone family, member Z | chr12 | 0.014 | 6 | 0.023 |  | | | |
| 101964\_at | Tkt | transketolase | chr14 | 1.087 | 6 | 0.001 |  | | | |
| 101992\_at | Psmb6 | proteasome (prosome, macropain) subunit, beta type 6 | chr11 | 1.47 | 6 | 0.016 |  | | | |
| 102001\_at | Rrm2 | ribonucleotide reductase M2 | chr12 | 1.74 | 6 | 0.02 |  | | | |
| 102019\_at | Mrpl13 | mitochondrial ribosomal protein L13 | chr15 | 1.841 | 6 | 0.001 |  | | | |
| 102022\_at | 1110007A04Rik | RIKEN cDNA 1110007A04 gene | --- | 1.16 | 6 | 0.009 |  | | | |
| 102126\_at | Rps12 | ribosomal protein S12 | chr10 | 0.076 | 6 | 0.03 |  | | | |
| 102194\_at | 2810432D09Rik | RIKEN cDNA 2810432D09 gene | chr4 | 0.91 | 6 | 0.031 |  | | | |
| 102197\_at | Nucb2 | nucleobindin 2 | chr7 | 0.931 | 6 | 0.019 |  | | | |
| 102409\_at | Lsm8 | LSM8 homolog, U6 small nuclear RNA associated (S. cerevisiae) | chr6 | 1.406 | 6 | 0.001 |  | | | |
| 102412\_at | AW541137 | expressed sequence AW541137 | chr10 | 2.049 | 6 | 0.032 |  | | | |
| 102631\_at | Blm | Bloom syndrome homolog (human) | chr7 | 1.408 | 6 | 0.003 |  | | | |
| 102791\_at | Psmb8 | proteosome (prosome, macropain) subunit, beta type 8 (large multifunctional protease 7) | chr17 | -0.163 | 6 | 0.016 |  | | | |
| 102821\_s\_at | Rasl2-9 | RAS-like, family 2, locus 9 | chr2 | 1.55 | 6 | 0 |  | | | |
| 102838\_at | Sell | selectin, lymphocyte | chr1 | 0.254 | 6 | 0.004 |  | | | |
| 102853\_at | Cspg6 | chondroitin sulfate proteoglycan 6 | chr19 | 1.265 | 6 | 0.037 |  | | | |
| 102970\_at | Psmc3ip | proteasome (prosome, macropain) 26S subunit, ATPase 3, interacting protein | chr11 | 0.86 | 6 | 0.028 |  | | | |
| 103038\_at | Guca1a | guanylate cyclase activator 1a (retina) | chr17 | -0.105 | 6 | 0.007 |  | | | |
| 103089\_at | Cd48 | CD48 antigen | chr1 | 7.012 | 6 | 0 |  | | | |
| 103319\_at | Psmd10 | proteasome (prosome, macropain) 26S subunit, non-ATPase, 10 | chrX | 0.731 | 6 | 0.01 |  | | | |
| 103334\_at | Crcp | calcitonin gene-related peptide-receptor component protein | chr5 | 1.088 | 6 | 0.015 |  | | | |
| 103335\_at | Lgals9 | lectin, galactose binding, soluble 9 | chr11 | 2.142 | 6 | 0.019 |  | | | |
| 103418\_at | Rfc4 | replication factor C (activator 1) 4 | chr16 | 2.864 | 6 | 0.041 |  | | | |
| 103442\_at | BC003479 | cDNA sequence BC003479 | chr11 | 1.965 | 6 | 0.007 |  | | | |
| 103468\_at | Mns1 | meiosis-specific nuclear structural protein 1 | chr9 | 3.961 | 6 | 0.026 |  | | | |
| 103534\_at | NoneAvailable | --- | chr7 | 2.111 | 6 | 0.006 |  | | | |
| 103581\_at | Cte1 | cytosolic acyl-CoA thioesterase 1 | chr12 | 0.169 | 6 | 0.01 |  | | | |
| 103598\_at | Dhx9 | DEAH (Asp-Glu-Ala-His) box polypeptide 9 | chr1 | 0.862 | 6 | 0.032 |  | | | |
| 103605\_g\_at | Rgs19 | regulator of G-protein signaling 19 | chr2 | 0.421 | 6 | 0.01 |  | | | |
| 103608\_at | 2810431B21Rik | RIKEN cDNA 2810431B21 gene | chr13 | 0.933 | 6 | 0.047 |  | | | |
| 103619\_at | 1810044O22Rik | RIKEN cDNA 1810044O22 gene | chr8 | 1.634 | 6 | 0.009 |  | | | |
| 103654\_at | Nsbp1 | nucleosome binding protein 1 | chrX | 0.032 | 6 | 0.02 |  | | | |
| 103671\_at | Htatip2 | HIV-1 tat interactive protein 2, homolog (human) | chr7 | 0.743 | 6 | 0.01 |  | | | |
| 103683\_at | Dhodh | dihydroorotate dehydrogenase | chr8 | 1.175 | 6 | 0.041 |  | | | |
| 103805\_at | Nbn | nibrin | chr4 | 0.182 | 6 | 0 |  | | | |
| 103881\_at | 1110013G13Rik | RIKEN cDNA 1110013G13 gene | chr3 | 1.342 | 6 | 0.006 |  | | | |
| 103885\_at | 1500019O16Rik | RIKEN cDNA 1500019O16 gene | chr7 | 5.88 | 6 | 0.011 |  | | | |
| 103939\_at | 2610509I15Rik | RIKEN cDNA 2610509I15 gene | --- | 0.597 | 6 | 0.008 |  | | | |
| 104042\_at | Slc35b1 | solute carrier family 35, member B1 | chr17 | 1.214 | 6 | 0.037 |  | | | |
| 104044\_at | 1300006N24Rik | RIKEN cDNA 1300006N24 gene | chr9 | 1.315 | 6 | 0.044 |  | | | |
| 104057\_at | Grpel1 | GrpE-like 1, mitochondrial | chr5 | 1.548 | 6 | 0.026 |  | | | |
| 104077\_at | 1110049G11Rik | RIKEN cDNA 1110049G11 gene | chrX | 1.238 | 6 | 0.01 |  | | | |
| 104078\_g\_at | 1110049G11Rik | RIKEN cDNA 1110049G11 gene | chrX | 3.329 | 6 | 0.001 |  | | | |
| 104080\_at | Pdap1 | PDGFA associated protein 1 | chr5 | 2.918 | 6 | 0.01 |  | | | |
| 104135\_at | Arl3 | ADP-ribosylation factor-like 3 | chr19 | 0.196 | 6 | 0.008 |  | | | |
| 104145\_at | Tcof1 | Treacher Collins Franceschetti syndrome 1, homolog | chr18 | 1.073 | 6 | 0.043 |  | | | |
| 104147\_at | Nans | N-acetylneuraminic acid synthase (sialic acid synthase) | chr4 | 1.196 | 6 | 0.022 |  | | | |
| 104212\_at | Lrpprc | leucine-rich PPR-motif containing | --- | 0.574 | 6 | 0.023 |  | | | |
| 104234\_at | Mrps25 | mitochondrial ribosomal protein S25 | chr6 | 1.243 | 6 | 0.029 |  | | | |
| 104237\_at | 2700061N24Rik | RIKEN cDNA 2700061N24 gene | chr13 | 1.881 | 6 | 0.016 |  | | | |
| 104248\_at | 0610038P07Rik | RIKEN cDNA 0610038P07 gene | chr3 | 0.168 | 6 | 0.046 |  | | | |
| 104297\_at | Ipo11 | importin 11 | chr13 | 1.447 | 6 | 0.02 |  | | | |
| 104301\_at | 2410018G20Rik | RIKEN cDNA 2410018G20 gene | chr16 | 1.631 | 6 | 0.002 |  | | | |
| 104322\_at | Ckap2 | cytoskeleton associated protein 2 | chr8 | 1.598 | 6 | 0.003 |  | | | |
| 104356\_at | 4921516M08Rik | RIKEN cDNA 4921516M08 gene | --- | 1.385 | 6 | 0 |  | | | |
| 104380\_at | Slc35a1 | solute carrier family 35 (CMP-sialic acid transporter), member 1 | chr4 | 0.449 | 6 | 0.002 |  | | | |
| 104390\_at | Anp32a | acidic (leucine-rich) nuclear phosphoprotein 32 family, member A | --- | 1.176 | 6 | 0.045 |  | | | |
| 104423\_at | 2810047L02Rik | RIKEN cDNA 2810047L02 gene | chr1 | 3.018 | 6 | 0.009 |  | | | |
| 104476\_at | Rbl1 | retinoblastoma-like 1 (p107) | chr2 | 1.983 | 6 | 0.011 |  | | | |
| 104541\_at | Prtn3 | proteinase 3 | chr10 | 1.916 | 6 | 0.042 |  | | | |
| 104567\_at | Mrpl46 | mitochondrial ribosomal protein L46 | chr7 | 1.995 | 6 | 0.031 |  | | | |
| 104733\_at | Cetn2 | centrin 2 | chrX | 0.012 | 6 | 0.015 |  | | | |
| 104738\_at | Zrf2 | zuotin related factor 2 | chr6 | 1.861 | 6 | 0.008 |  | | | |
| 104762\_r\_at | 1500015J03Rik | RIKEN cDNA 1500015J03 gene | chr2 | 1.459 | 6 | 0.042 |  | | | |
| 104766\_at | Nola1 | nucleolar protein family A, member 1 (H/ACA small nucleolar RNPs) | --- | 1.457 | 6 | 0.002 |  | | | |
| 160076\_at | Mtx2 | metaxin 2 | chr2 | 1.76 | 6 | 0.035 |  | | | |
| 160107\_at | Hprt | hypoxanthine guanine phosphoribosyl transferase | chrX | 0.226 | 6 | 0.028 |  | | | |
| 160125\_at | D11Ertd497e | DNA segment, Chr 11, ERATO Doi 497, expressed | --- | 0.876 | 6 | 0.007 |  | | | |
| 160126\_at | Map2k1ip1 | mitogen-activated protein kinase kinase 1 interacting protein 1 | chr3 | 0.372 | 6 | 0.049 |  | | | |
| 160135\_at | D16Ertd502e | DNA segment, Chr 16, ERATO Doi 502, expressed | chr16 | 1.284 | 6 | 0.007 |  | | | |
| 160152\_at | Psmc1 | protease (prosome, macropain) 26S subunit, ATPase 1 | chr16 | 1.442 | 6 | 0.02 |  | | | |
| 160165\_at | Ubc-rs2 | ubiquitin C, related sequence 2 | chr7 | 0.51 | 6 | 0.046 |  | | | |
| 160176\_at | Hirip5 | histone cell cycle regulation defective interacting protein 5 | chr15 | 1.669 | 6 | 0 |  | | | |
| 160193\_at | 5730551F12Rik | RIKEN cDNA 5730551F12 gene | chr10 | 0.6 | 6 | 0.002 |  | | | |
| 160203\_at | 5330419I01Rik | RIKEN cDNA 5330419I01 gene | chr1 | 0.873 | 6 | 0.017 |  | | | |
| 160214\_at | 1700003F10Rik | RIKEN cDNA 1700003F10 gene | chr6 | 0.072 | 6 | 0.039 |  | | | |
| 160218\_at | 2310056P07Rik | RIKEN cDNA 2310056P07 gene | chr16 | 0.971 | 6 | 0.002 |  | | | |
| 160225\_at | Gtf2b | general transcription factor IIB | chr3 | 0.477 | 6 | 0.012 |  | | | |
| 160235\_at | 5033425B17Rik | RIKEN cDNA 5033425B17 gene | chr7 | 0.992 | 6 | 0.006 |  | | | |
| 160247\_at | Ube2v2 | ubiquitin-conjugating enzyme E2 variant 2 | chr15 | 1.044 | 6 | 0.003 |  | | | |
| 160256\_at | 1110020J08Rik | RIKEN cDNA 1110020J08 gene | --- | 0.939 | 6 | 0.001 |  | | | |
| 160262\_at | Mtch2 | mitochondrial carrier homolog 2 (C. elegans) | chr2 | 0.99 | 6 | 0.01 |  | | | |
| 160266\_r\_at | 1110064N10Rik | RIKEN cDNA 1110064N10 gene | chr15 | 0.96 | 6 | 0.041 |  | | | |
| 160293\_at | 2700038L12Rik | RIKEN cDNA 2700038L12 gene | chr9 | 1.194 | 6 | 0.048 |  | | | |
| 160297\_at | MGC36453 | hypothetical protein LOC381045 | chr14 | 2.932 | 6 | 0.003 |  | | | |
| 160299\_at | Rwdd1 | RWD domain containing 1 | chr10 | 1.08 | 6 | 0.034 |  | | | |
| 160314\_at | 2010317E03Rik | RIKEN cDNA 2010317E03 gene | chr10 | 0.092 | 6 | 0.007 |  | | | |
| 160324\_at | Rpa3 | replication protein A3 | chr1 | 1.959 | 6 | 0.009 |  | | | |
| 160344\_at | Npc2 | Niemann Pick type C2 | chr12 | 0.888 | 6 | 0.036 |  | | | |
| 160360\_at | Sep15-pending | selenoprotein | chr3 | -0.129 | 6 | 0.012 |  | | | |
| 160412\_at | Pdcd2 | programmed cell death 2 | chr17 | 0.662 | 6 | 0.048 |  | | | |
| 160416\_at | Fkbp3 | FK506 binding protein 3 | chr12 | 0.512 | 6 | 0.006 |  | | | |
| 160426\_at | Rpo1-1 | RNA polymerase 1-1 | chr17 | 0.705 | 6 | 0.029 |  | | | |
| 160431\_at | Mrpl12 | mitochondrial ribosomal protein L12 | chr11 | 2.077 | 6 | 0.009 |  | | | |
| 160444\_at | 2010319C14Rik | RIKEN cDNA 2010319C14 gene | chr16 | 0.548 | 6 | 0.042 |  | | | |
| 160456\_at | 1100001J08Rik | RIKEN cDNA 1100001J08 gene | --- | 0.642 | 6 | 0.011 |  | | | |
| 160485\_r\_at | Ywhae | tyrosine 3-monooxygenase/tryptophan 5-monooxygenase activation protein, epsilon polypeptide | --- | 0.185 | 6 | 0.012 |  | | | |
| 160503\_at | Fbl | fibrillarin | chr17 | 1.886 | 6 | 0 |  | | | |
| 160531\_at | Grcc2f | gene rich cluster, C2f gene | chr6 | 0.73 | 6 | 0.036 |  | | | |
| 160536\_at | Hras1 | Harvey rat sarcoma virus oncogene 1 | --- | 0.429 | 6 | 0.031 |  | | | |
| 160538\_at | Cdk4 | cyclin-dependent kinase 4 | chr10 | 2.296 | 6 | 0.032 |  | | | |
| 160543\_at | Snx3 | sorting nexin 3 | chr10 | 0.395 | 6 | 0.023 |  | | | |
| 160550\_i\_at | Magoh | mago-nashi homolog, proliferation-associated (Drosophila) | chr4 | 0.676 | 6 | 0.016 |  | | | |
| 160551\_at | Vdac3 | voltage-dependent anion channel 3 | chr13 | 0.534 | 6 | 0.006 |  | | | |
| 160556\_at | 1810020G14Rik | RIKEN cDNA 1810020G14 gene | chr12 | 0.101 | 6 | 0.023 |  | | | |
| 160569\_at | 2310008M10Rik | RIKEN cDNA 2310008M10 gene | chr3 | 1.341 | 6 | 0.006 |  | | | |
| 160585\_at | 2810470K21Rik | RIKEN cDNA 2810470K21 gene | --- | 2.814 | 6 | 0.011 |  | | | |
| 160621\_at | Mrps22 | mitochondrial ribosomal protein S22 | chr9 | 0.891 | 6 | 0.017 |  | | | |
| 160659\_at | 2310057G13Rik | RIKEN cDNA 2310057G13 gene | chr10 | 0.89 | 6 | 0 |  | | | |
| 160709\_at | 1110001A16Rik | RIKEN cDNA 1110001A16 gene | chr17 | 0.843 | 6 | 0 |  | | | |
| 160711\_at | Decr1 | 2,4-dienoyl CoA reductase 1, mitochondrial | chr4 | 0.759 | 6 | 0.006 |  | | | |
| 160723\_at | 1500001M20Rik | RIKEN cDNA 1500001M20 gene | chr6 | 1.3 | 6 | 0.044 |  | | | |
| 160743\_at | Pole3 | polymerase (DNA directed), epsilon 3 (p17 subunit) | chr4 | 0.238 | 6 | 0.008 |  | | | |
| 160805\_s\_at | Mpdu1 | mannose-P-dolichol utilization defect 1 | chr11 | 0.143 | 6 | 0.011 |  | | | |
| 160844\_at | Pts | 6-pyruvoyl-tetrahydropterin synthase | chr9 | 0.175 | 6 | 0.049 |  | | | |
| 160856\_at | Ubl4 | ubiquitin-like 4 | --- | 0.36 | 6 | 0.007 |  | | | |
| 160872\_f\_at | 2310008H09Rik | RIKEN cDNA 2310008H09 gene | chr7 | 0.592 | 6 | 0.016 |  | | | |
| 160876\_at | Bcap29 | B-cell receptor-associated protein 29 | chr12 | 1.484 | 6 | 0.023 |  | | | |
| 161004\_at | 1700097N02Rik | RIKEN cDNA 1700097N02 gene | --- | 0.221 | 6 | 0.005 |  | | | |
| 161147\_f\_at | 1110046L09Rik | RIKEN cDNA 1110046L09 gene | chr8 | 3.517 | 6 | 0.013 |  | | | |
| 161359\_s\_at | Apoa1bp | apolipoprotein A-I binding protein | chr3 | 0.755 | 6 | 0.006 |  | | | |
| 161487\_f\_at | NoneAvailable | Mus musculus transcribed sequence with strong similarity to protein sp:P48201 (H.sapiens) AT93\_HUMAN ATP synthase lipid-binding protein, mitochondrial precursor (ATP synthase proteolipid P3) (ATPase protein 9) (ATPase subunit C) | --- | 0.986 | 6 | 0.005 |  | | | |
| 161522\_i\_at | Cst3 | cystatin C | chr2 | 0.465 | 6 | 0.046 |  | | | |
| 161872\_f\_at | 1110049G11Rik | RIKEN cDNA 1110049G11 gene | --- | 1.026 | 6 | 0.01 |  | | | |
| 161897\_f\_at | Prps1 | phosphoribosyl pyrophosphate synthetase 1 | chrX | 0.02 | 6 | 0.026 |  | | | |
| 162013\_f\_at | D4Ertd786e | DNA segment, Chr 4, ERATO Doi 786, expressed | chr7 | 0.907 | 6 | 0.006 |  | | | |
| 162235\_f\_at | D19Wsu55e | DNA segment, Chr 19, Wayne State University 55, expressed | chr19 | 0.378 | 6 | 0.003 |  | | | |
| 162417\_at | 1500001M20Rik | RIKEN cDNA 1500001M20 gene | --- | 0.537 | 6 | 0.003 |  | | | |
| 162466\_at | 1810004D07Rik | RIKEN cDNA 1810004D07 gene | --- | 0.295 | 6 | 0.031 |  | | | |
| 92388\_at | BC038311 | cDNA sequence BC038311 | chr5 | 0.478 | 6 | 0.025 |  | | | |
| 92540\_f\_at | Srm | spermidine synthase | chr4 | 6.091 | 6 | 0.001 |  | | | |
| 92547\_at | Hip2 | huntingtin interacting protein 2 | chr5 | 0.567 | 6 | 0.04 |  | | | |
| 92565\_at | 1110005A23Rik | RIKEN cDNA 1110005A23 gene | chr19 | 0.994 | 6 | 0.007 |  | | | |
| 92574\_at | 3110001M13Rik | RIKEN cDNA 3110001M13 gene | chr9 | 1.899 | 6 | 0.011 |  | | | |
| 92578\_at | Scye1 | small inducible cytokine subfamily E, member 1 | chr3 | 0.839 | 6 | 0.011 |  | | | |
| 92589\_at | Psph | phosphoserine phosphatase | chr5 | 0.853 | 6 | 0.013 |  | | | |
| 92596\_at | Cacybp | calcyclin binding protein | chr1 | 0.523 | 6 | 0.039 |  | | | |
| 92615\_at | AI837181 | expressed sequence AI837181 | chr4 | 2.807 | 6 | 0.001 |  | | | |
| 92625\_at | Nme2 | expressed in non-metastatic cells 2, protein | chr10 | 1.732 | 6 | 0.004 |  | | | |
| 92629\_f\_at | Hdgf | hepatoma-derived growth factor | --- | 0.521 | 6 | 0.019 |  | | | |
| 92636\_f\_at | Sec61g | SEC61, gamma subunit | chr1 | 1.533 | 6 | 0.004 |  | | | |
| 92646\_at | Mrpl23 | mitochondrial ribosomal protein L23 | chr2 | 2.152 | 6 | 0.028 |  | | | |
| 92770\_at | S100a6 | S100 calcium binding protein A6 (calcyclin) | chr3 | -3.204 | 6 | 0 |  | | | |
| 92778\_i\_at | NoneAvailable | --- | --- | -1.238 | 6 | 0.049 |  | | | |
| 92788\_f\_at | Cetn3 | centrin 3 | chr13 | 1.678 | 6 | 0.018 |  | | | |
| 92798\_at | Atp5c1 | ATP synthase, H+ transporting, mitochondrial F1 complex, gamma polypeptide 1 | chrX | 1.327 | 6 | 0.003 |  | | | |
| 92799\_g\_at | Atp5c1 | ATP synthase, H+ transporting, mitochondrial F1 complex, gamma polypeptide 1 | chr2 | 1.541 | 6 | 0.001 |  | | | |
| 92800\_i\_at | Atp5c1 | ATP synthase, H+ transporting, mitochondrial F1 complex, gamma polypeptide 1 | chr13 | 0.86 | 6 | 0 |  | | | |
| 92824\_at | Nme6 | expressed in non-metastatic cells 6, protein | chr9 | 1.781 | 6 | 0.001 |  | | | |
| 92826\_at | Gdap3 | ganglioside-induced differentiation-associated-protein 3 | --- | 0.209 | 6 | 0.003 |  | | | |
| 92829\_at | Hspe1 | heat shock protein 1 (chaperonin 10) | chr1 | 1.429 | 6 | 0.001 |  | | | |
| 92831\_at | Sfxn1 | sideroflexin 1 | --- | 1.223 | 6 | 0.005 |  | | | |
| 92874\_f\_at | Cops7a | COP9 (constitutive photomorphogenic) homolog, subunit 7a (Arabidopsis thaliana) | chr6 | 1.098 | 6 | 0.003 |  | | | |
| 92876\_at | Ndufs4 | NADH dehydrogenase (ubiquinone) Fe-S protein 4 | chr13 | 0.222 | 6 | 0.002 |  | | | |
| 93008\_at | Lsm4 | LSM4 homolog, U6 small nuclear RNA associated (S. cerevisiae) | chr8 | 2.177 | 6 | 0.021 |  | | | |
| 93014\_at | Atp5l | ATP synthase, H+ transporting, mitochondrial F0 complex, subunit g | chr11 | 1.668 | 6 | 0.019 |  | | | |
| 93029\_at | Idh3g | isocitrate dehydrogenase 3 (NAD+), gamma | chrX | 0.845 | 6 | 0.006 |  | | | |
| 93041\_at | Mcm4 | minichromosome maintenance deficient 4 homolog (S. cerevisiae) | chr16 | 1.922 | 6 | 0.039 |  | | | |
| 93042\_at | Bzrp | benzodiazepine receptor, peripheral | chr15 | 0.924 | 6 | 0.021 |  | | | |
| 93062\_at | Mrpl39 | mitochondrial ribosomal protein L39 | chr16 | 1.936 | 6 | 0.016 |  | | | |
| 93078\_at | Ly6a | lymphocyte antigen 6 complex, locus A | chr15 | -3.737 | 6 | 0.008 |  | | | |
| 93084\_at | Slc25a4 | solute carrier family 25 (mitochondrial carrier; adenine nucleotide translocator), member 4 | chr8 | 0.194 | 6 | 0.005 |  | | | |
| 93085\_at | Psmb9 | proteosome (prosome, macropain) subunit, beta type 9 (large multifunctional protease 2) | chr17 | 0.307 | 6 | 0.015 |  | | | |
| 93095\_at | Hmgb1 | high mobility group box 1 | chr11 | 0.963 | 6 | 0.03 |  | | | |
| 93101\_s\_at | Nedd4 | neural precursor cell expressed, developmentally down-regulted gene 4 | chr9 | -0.095 | 6 | 0.003 |  | | | |
| 93112\_at | Mcm2 | minichromosome maintenance deficient 2 mitotin (S. cerevisiae) | chr6 | 2.139 | 6 | 0.018 |  | | | |
| 93117\_at | Hnrpa2b1 | heterogeneous nuclear ribonucleoprotein A2/B1 | chr6 | 0.4 | 6 | 0.006 |  | | | |
| 93139\_at | 4432411H13Rik | RIKEN cDNA 4432411H13 gene | chr5 | -0.583 | 6 | 0.031 |  | | | |
| 93203\_f\_at | 3230402J05Rik | RIKEN cDNA 3230402J05 gene | chr17 | -0.003 | 6 | 0.01 |  | | | |
| 93236\_s\_at | Tyms | thymidylate synthase | chr10 | 3.228 | 6 | 0.012 |  | | | |
| 93237\_s\_at | Tyms | thymidylate synthase | chr10 | 2.144 | 6 | 0.021 |  | | | |
| 93251\_at | Nipsnap1 | 4-nitrophenylphosphatase domain and non-neuronal SNAP25-like protein homolog 1 (C. elegans) | chr11 | 0.772 | 6 | 0.027 |  | | | |
| 93258\_at | Hmbs | hydroxymethylbilane synthase | chr9 | 2.914 | 6 | 0.046 |  | | | |
| 93277\_at | Hspd1 | heat shock protein 1 (chaperonin) | chr1 | 1.14 | 6 | 0.029 |  | | | |
| 93290\_at | Pnp | purine-nucleoside phosphorylase | chr14 | 1.213 | 6 | 0.044 |  | | | |
| 93323\_at | Plp2 | proteolipid protein 2 | chr2 | -0.4 | 6 | 0.008 |  | | | |
| 93336\_at | 1110014C03Rik | RIKEN cDNA 1110014C03 gene | chr12 | 0.813 | 6 | 0.041 |  | | | |
| 93456\_r\_at | NoneAvailable | --- | chr14 | -0.076 | 6 | 0.01 |  | | | |
| 93495\_at | Prdx4 | peroxiredoxin 4 | chrX | 0.796 | 6 | 0.013 |  | | | |
| 93512\_f\_at | Adk | adenosine kinase | chr14 | 0.798 | 6 | 0 |  | | | |
| 93519\_s\_at | Nedd8 | neural precursor cell expressed, developmentally down-regulated gene 8 | chr14 | 2.012 | 6 | 0.002 |  | | | |
| 93531\_at | Ndufa8 | NADH dehydrogenase (ubiquinone) 1 alpha subcomplex, 8 | chr2 | 1.477 | 6 | 0.042 |  | | | |
| 93533\_at | 1500011L16Rik | RIKEN cDNA 1500011L16 gene | --- | 2.198 | 6 | 0.022 |  | | | |
| 93539\_at | 1810004D07Rik | RIKEN cDNA 1810004D07 gene | --- | 1.406 | 6 | 0 |  | | | |
| 93542\_at | Pter | phosphotriesterase related | chr2 | 1.123 | 6 | 0.022 |  | | | |
| 93548\_at | Sec61b | Sec61 beta subunit | chr4 | 2.149 | 6 | 0.005 |  | | | |
| 93551\_at | Tm4sf7 | transmembrane 4 superfamily member 7 | chr7 | 0.862 | 6 | 0.008 |  | | | |
| 93559\_at | Apex1 | apurinic/apyrimidinic endonuclease 1 | chr14 | 1.957 | 6 | 0.002 |  | | | |
| 93560\_at | Acyp1 | acylphosphatase 1, erythrocyte (common) type | chr12 | 3.035 | 6 | 0.029 |  | | | |
| 93562\_at | Ndufb3 | NADH dehydrogenase (ubiquinone) 1 beta subcomplex 3 | --- | 0.903 | 6 | 0.001 |  | | | |
| 93579\_at | 5830427H10Rik | RIKEN cDNA 5830427H10 gene | chr6 | 1.438 | 6 | 0.034 |  | | | |
| 93581\_at | Ndufb8 | NADH dehydrogenase (ubiquinone) 1 beta subcomplex 8 | chr19 | 2.79 | 6 | 0.003 |  | | | |
| 93582\_at | Coq7 | demethyl-Q 7 | chr7 | 3.745 | 6 | 0.001 |  | | | |
| 93593\_f\_at | Emp3 | epithelial membrane protein 3 | chr7 | 0.001 | 6 | 0.046 |  | | | |
| 93596\_i\_at | Atp5e | ATP synthase, H+ transporting, mitochondrial F1 complex, epsilon subunit | chr2 | 1.737 | 6 | 0.006 |  | | | |
| 93734\_i\_at | Psmc3 | proteasome (prosome, macropain) 26S subunit, ATPase 3 | chr2 | 0.698 | 6 | 0.002 |  | | | |
| 93735\_f\_at | Psmc3 | proteasome (prosome, macropain) 26S subunit, ATPase 3 | chr2 | 1.365 | 6 | 0.011 |  | | | |
| 93742\_at | 5730449L18Rik | RIKEN cDNA 5730449L18 gene | chr1 | 0.944 | 6 | 0.026 |  | | | |
| 93754\_at | Ech1 | enoyl coenzyme A hydratase 1, peroxisomal | chr7 | 0.716 | 6 | 0.015 |  | | | |
| 93764\_at | Grim19-pending | genes associated with retinoid-IFN-induced mortality 19 | --- | 0.229 | 6 | 0.005 |  | | | |
| 93780\_at | Them2 | thioesterase superfamily member 2 | chr13 | 1.504 | 6 | 0.012 |  | | | |
| 93784\_at | Cfdp | craniofacial development protein 1 | chr8 | 1.424 | 6 | 0.031 |  | | | |
| 93786\_i\_at | Mrpl18 | mitochondrial ribosomal protein L18 | chr17 | 2.577 | 6 | 0.013 |  | | | |
| 93787\_f\_at | Mrpl18 | mitochondrial ribosomal protein L18 | --- | 2.215 | 6 | 0.009 |  | | | |
| 93812\_at | Clns1a | chloride channel, nucleotide-sensitive, 1A | chr7 | 1.373 | 6 | 0.031 |  | | | |
| 93815\_at | 0610041L09Rik | RIKEN cDNA 0610041L09 gene | chr6 | 0.912 | 6 | 0.013 |  | | | |
| 93820\_at | Cox7a2 | cytochrome c oxidase, subunit VIIa 2 | chr9 | 1.951 | 6 | 0.002 |  | | | |
| 93833\_s\_at | Hist1h2bc | histone 1, H2bc | chr13 | 1.06 | 6 | 0.043 |  | | | |
| 93838\_at | 2700038C09Rik | RIKEN cDNA 2700038C09 gene | chr2 | 3.273 | 6 | 0.025 |  | | | |
| 93952\_r\_at | 2810417H13Rik | RIKEN cDNA 2810417H13 gene | --- | 0.619 | 6 | 0.014 |  | | | |
| 93970\_at | Ipo7 | importin 7 | chr7 | 0.551 | 6 | 0.018 |  | | | |
| 93984\_at | Atpi | ATPase inhibitor | --- | 1.332 | 6 | 0 |  | | | |
| 93988\_at | Psma7 | proteasome (prosome, macropain) subunit, alpha type 7 | chr2 | 1.392 | 6 | 0.02 |  | | | |
| 93991\_at | Mor1 | malate dehydrogenase, mitochondrial | chr5 | 1.136 | 6 | 0.009 |  | | | |
| 93993\_at | Lman2 | lectin, mannose-binding 2 | chr13 | 1.647 | 6 | 0.002 |  | | | |
| 93999\_at | Snrpg | small nuclear ribonucleoprotein polypeptide G | chr17 | 0.927 | 6 | 0.002 |  | | | |
| 94014\_at | 2510048O06Rik | RIKEN cDNA 2510048O06 gene | --- | 1.798 | 6 | 0 |  | | | |
| 94025\_at | Psmb3 | proteasome (prosome, macropain) subunit, beta type 3 | chr16 | 1.932 | 6 | 0.035 |  | | | |
| 94032\_at | Apoa1bp | apolipoprotein A-I binding protein | chr3 | 0.949 | 6 | 0.008 |  | | | |
| 94034\_at | Smfn | small fragment nuclease | chr9 | 0.778 | 6 | 0 |  | | | |
| 94040\_at | Erh | enhancer of rudimentary homolog (Drosophila) | chr12 | 1.929 | 6 | 0.001 |  | | | |
| 94062\_at | Ndufv2 | NADH dehydrogenase (ubiquinone) flavoprotein 2 | chr17 | 1.3 | 6 | 0.045 |  | | | |
| 94110\_f\_at | NoneAvailable | Mus musculus 0 day neonate thymus cDNA, RIKEN full-length enriched library, clone:A430088G18 product:hypothetical Zinc finger, C2H2 type containing protein, full insert sequence | --- | 0.488 | 6 | 0.033 |  | | | |
| 94206\_at | Grcc10 | gene rich cluster, C10 gene | chr6 | 0.906 | 6 | 0 |  | | | |
| 94210\_at | Timm9 | translocase of inner mitochondrial membrane 9 homolog (yeast) | chr10 | 3.713 | 6 | 0.022 |  | | | |
| 94252\_at | Eif2s3x | eukaryotic translation initiation factor 2, subunit 3, structural gene X-linked | chrX | 0.381 | 6 | 0.049 |  | | | |
| 94263\_f\_at | Psmb7 | proteasome (prosome, macropain) subunit, beta type 7 | chr2 | 1.959 | 6 | 0.018 |  | | | |
| 94267\_i\_at | NoneAvailable | --- | chr9 | 0.902 | 6 | 0.037 |  | | | |
| 94268\_f\_at | NoneAvailable | --- | chr9 | 0.316 | 6 | 0.004 |  | | | |
| 94275\_at | Urod | uroporphyrinogen decarboxylase | chr4 | 1.492 | 6 | 0.015 |  | | | |
| 94276\_at | Hsd17b12 | hydroxysteroid (17-beta) dehydrogenase 12 | chr2 | 0.315 | 6 | 0.041 |  | | | |
| 94277\_at | Mtx1 | metaxin 1 | chr3 | 1.276 | 6 | 0.007 |  | | | |
| 94278\_at | Lcp1 | lymphocyte cytosolic protein 1 | chr14 | -0.14 | 6 | 0.027 |  | | | |
| 94294\_at | Ccnb2 | cyclin B2 | chr9 | 4.408 | 6 | 0.001 |  | | | |
| 94313\_at | Snrp1c | U1 small nuclear ribonucleoprotein 1C | chr15 | -0.007 | 6 | 0.002 |  | | | |
| 94323\_at | D630012G11Rik | RIKEN cDNA D630012G11 gene | chr19 | 1.879 | 6 | 0 |  | | | |
| 94360\_at | 2700029M09Rik | RIKEN cDNA 2700029M09 gene | chr8 | 1.272 | 6 | 0.009 |  | | | |
| 94372\_at | Nudt1 | nudix (nucleoside diphosphate linked moiety X)-type motif 1 | --- | 3.289 | 6 | 0.003 |  | | | |
| 94394\_at | Rras | Harvey rat sarcoma oncogene, subgroup R | chr7 | -0.442 | 6 | 0.049 |  | | | |
| 94455\_at | Lsm3 | LSM3 homolog, U6 small nuclear RNA associated (S. cerevisiae) | chr6 | 2.688 | 6 | 0.002 |  | | | |
| 94494\_at | Farsl | phenylalanine-tRNA synthetase-like | chr1 | 1.832 | 6 | 0.008 |  | | | |
| 94506\_at | Cpsf5 | cleavage and polyadenylation specific factor 5 | chr3 | 0.914 | 6 | 0.007 |  | | | |
| 94526\_at | D10Ertd214e | DNA segment, Chr 10, ERATO Doi 214, expressed | chr10 | 2.227 | 6 | 0 |  | | | |
| 94558\_g\_at | Gtf3a | general transcription factor III A | chr5 | 1.943 | 6 | 0.011 |  | | | |
| 94789\_r\_at | Tubb5 | tubulin, beta 5 | chr17 | 3.275 | 6 | 0.044 |  | | | |
| 94841\_at | Psma5 | proteasome (prosome, macropain) subunit, alpha type 5 | chr10 | 1.755 | 6 | 0.007 |  | | | |
| 94862\_i\_at | Dncl2a | dynein, cytoplasmic, light chain 2A | --- | 0.742 | 6 | 0.043 |  | | | |
| 94870\_f\_at | 2310075M17Rik | RIKEN cDNA 2310075M17 gene | chr11 | 1.008 | 6 | 0.02 |  | | | |
| 94892\_r\_at | Mea1 | male enhanced antigen 1 | chr17 | 1.005 | 6 | 0.017 |  | | | |
| 94897\_at | Gpx4 | glutathione peroxidase 4 | --- | 0.101 | 6 | 0.046 |  | | | |
| 94908\_r\_at | 1110001J03Rik | RIKEN cDNA 1110001J03 gene | chr6 | 0.276 | 6 | 0.029 |  | | | |
| 94912\_at | Mrps21 | mitochondrial ribosomal protein S21 | chr3 | 1.449 | 6 | 0.017 |  | | | |
| 94925\_at | 1810055D05Rik | RIKEN cDNA 1810055D05 gene | chr3 | 0.976 | 6 | 0.021 |  | | | |
| 94931\_at | 1810045K17Rik | RIKEN cDNA 1810045K17 gene | chr3 | 1.276 | 6 | 0.034 |  | | | |
| 94933\_at | BC008155 | cDNA sequence BC008155 | chr17 | 1.315 | 6 | 0.039 |  | | | |
| 95015\_at | Akr1c13 | aldo-keto reductase family 1, member C13 | chr13 | 0.179 | 6 | 0.002 |  | | | |
| 95042\_at | C030002N13Rik | RIKEN cDNA C030002N13 gene | chr4 | 0.462 | 6 | 0.014 |  | | | |
| 95045\_at | 0610012D09Rik | RIKEN cDNA 0610012D09 gene | chr7 | 1.41 | 6 | 0 |  | | | |
| 95049\_at | Snrpd2 | small nuclear ribonucleoprotein D2 | chr10 | 1.485 | 6 | 0.03 |  | | | |
| 95052\_at | 1110035L05Rik | RIKEN cDNA 1110035L05 gene | chr4 | 0.243 | 6 | 0.025 |  | | | |
| 95053\_s\_at | Sdhb | succinate dehydrogenase complex, subunit B, iron sulfur (Ip) | chr4 | 2.681 | 6 | 0.023 |  | | | |
| 95061\_at | Bcas2 | breast carcinoma amplified sequence 2 | chr3 | 0.334 | 6 | 0 |  | | | |
| 95064\_at | Acaa2 | acetyl-Coenzyme A acyltransferase 2 (mitochondrial 3-oxoacyl-Coenzyme A thiolase) | chr18 | 0.479 | 6 | 0.016 |  | | | |
| 95067\_at | Mrpl2 | mitochondrial ribosomal protein L2 | chr17 | 1.667 | 6 | 0.023 |  | | | |
| 95091\_at | Sec13r | SEC13 related gene (S. cerevisiae) | --- | 1.672 | 6 | 0.001 |  | | | |
| 95105\_at | 2010110M21Rik | RIKEN cDNA 2010110M21 gene | chr13 | 0.297 | 6 | 0.025 |  | | | |
| 95132\_r\_at | Ndufb2 | NADH dehydrogenase (ubiquinone) 1 beta subcomplex, 2 | chr6 | 1.988 | 6 | 0.024 |  | | | |
| 95408\_at | 2310003F16Rik | RIKEN cDNA 2310003F16 gene | chr2 | 0.987 | 6 | 0.023 |  | | | |
| 95413\_at | 6030432N09Rik | RIKEN cDNA 6030432N09 gene | chr5 | 0.123 | 6 | 0.035 |  | | | |
| 95426\_at | Echs1 | enoyl Coenzyme A hydratase, short chain, 1, mitochondrial | chr7 | 0.838 | 6 | 0.02 |  | | | |
| 95441\_at | Timm23 | translocase of inner mitochondrial membrane 23 homolog (yeast) | chr1 | 1.675 | 6 | 0.042 |  | | | |
| 95448\_at | Psmc2 | proteasome (prosome, macropain) 26S subunit, ATPase 2 | chr5 | 1.408 | 6 | 0.017 |  | | | |
| 95460\_at | Cops5 | COP9 (constitutive photomorphogenic) homolog, subunit 5 (Arabidopsis thaliana) | chr1 | 1.564 | 6 | 0.025 |  | | | |
| 95472\_f\_at | Uqcrb | ubiquinol-cytochrome c reductase binding protein | chr13 | 0.734 | 6 | 0.006 |  | | | |
| 95479\_at | 1110036E10Rik | RIKEN cDNA 1110036E10 gene | chr11 | 0.426 | 6 | 0.049 |  | | | |
| 95480\_at | D11Wsu68e | DNA segment, Chr 11, Wayne State University 68, expressed | chr11 | 1.471 | 6 | 0.003 |  | | | |
| 95485\_at | Hadhsc | L-3-hydroxyacyl-Coenzyme A dehydrogenase, short chain | chr3 | 0.06 | 6 | 0.011 |  | | | |
| 95491\_at | Park7 | Parkinson disease (autosomal recessive, early onset) 7 | chr4 | 1.248 | 6 | 0.002 |  | | | |
| 95497\_at | 1110005A05Rik | RIKEN cDNA 1110005A05 gene | chr9 | 3.276 | 6 | 0.011 |  | | | |
| 95498\_at | Mrps15 | mitochondrial ribosomal protein S15 | chr4 | 2.035 | 6 | 0.005 |  | | | |
| 95561\_at | 1700013H19Rik | RIKEN cDNA 1700013H19 gene | chr8 | 0.899 | 6 | 0.003 |  | | | |
| 95590\_at | Alg5 | asparagine-linked glycosylation 5 homolog (yeast, dolichyl-phosphate beta-glucosyltransferase) | chr3 | 1.43 | 6 | 0.003 |  | | | |
| 95593\_at | Golph2 | golgi phosphoprotein 2 | chr13 | 1.191 | 6 | 0.018 |  | | | |
| 95606\_at | Nsap1l-pending | NS1-associated protein 1-like | chr9 | 1.239 | 6 | 0.005 |  | | | |
| 95620\_at | 2310016E22Rik | RIKEN cDNA 2310016E22 gene | chr12 | 0.069 | 6 | 0.004 |  | | | |
| 95634\_at | 0610010K14Rik | RIKEN cDNA 0610010K14 gene | --- | 1.777 | 6 | 0.023 |  | | | |
| 95635\_g\_at | 0610010K14Rik | RIKEN cDNA 0610010K14 gene | chr11 | 0.403 | 6 | 0.036 |  | | | |
| 95636\_at | 0610010K14Rik | RIKEN cDNA 0610010K14 gene | chr11 | 2.424 | 6 | 0.007 |  | | | |
| 95649\_at | Phf5a | PHD finger protein 5A | chr15 | 1.933 | 6 | 0.012 |  | | | |
| 95654\_at | Clic1 | chloride intracellular channel 1 | chr17 | -0.179 | 6 | 0.033 |  | | | |
| 95656\_i\_at | D13Wsu177e | DNA segment, Chr 13, Wayne State University 177, expressed | chr13 | 2.603 | 6 | 0.011 |  | | | |
| 95660\_at | 0610025L15Rik | RIKEN cDNA 0610025L15 gene | chr7 | 2.58 | 6 | 0 |  | | | |
| 95677\_at | 0610009C03Rik | RIKEN cDNA 0610009C03 gene | --- | 2.789 | 6 | 0.026 |  | | | |
| 95690\_at | 1110030L07Rik | RIKEN cDNA 1110030L07 gene | chr15 | 3.584 | 6 | 0.007 |  | | | |
| 95693\_at | Idh2 | isocitrate dehydrogenase 2 (NADP+), mitochondrial | chr7 | 1.921 | 6 | 0.021 |  | | | |
| 95696\_at | Txnl2 | thioredoxin-like 2 | chr4 | 1.145 | 6 | 0.009 |  | | | |
| 95698\_at | Ndufb7 | NADH dehydrogenase (ubiquinone) 1 beta subcomplex, 7 | chr8 | 2.162 | 6 | 0.008 |  | | | |
| 95707\_at | 2900010M23Rik | RIKEN cDNA 2900010M23 gene | chr17 | 2.087 | 6 | 0.032 |  | | | |
| 95714\_at | 0610009D07Rik | RIKEN cDNA 0610009D07 gene | chr12 | 1.473 | 6 | 0.027 |  | | | |
| 95718\_f\_at | Usmg5 | upregulated during skeletal muscle growth 5 | chr19 | 1.302 | 6 | 0.006 |  | | | |
| 95760\_at | 1110011K10Rik | RIKEN cDNA 1110011K10 gene | chr9 | 2.372 | 6 | 0.002 |  | | | |
| 95891\_at | NoneAvailable | Mus musculus transcribed sequences | chr16 | 3.953 | 6 | 0.017 |  | | | |
| 96016\_at | NoneAvailable | Mus musculus cDNA clone MGC:67366 IMAGE:5683334, complete cds | chr2 | 3.771 | 6 | 0.015 |  | | | |
| 96029\_at | Sf3a3 | splicing factor 3a, subunit 3, 60kDa | chr4 | 2.16 | 6 | 0.047 |  | | | |
| 96041\_at | Rbm3 | RNA binding motif protein 3 | chr1 | -0.448 | 6 | 0.038 |  | | | |
| 96048\_at | Hrsp12 | heat-responsive protein 12 | chr15 | 0.196 | 6 | 0.037 |  | | | |
| 96052\_at | Acp1 | acid phosphatase 1, soluble | --- | 1.712 | 6 | 0.022 |  | | | |
| 96081\_at | Tk1 | thymidine kinase 1 | chr11 | 2.893 | 6 | 0.014 |  | | | |
| 96089\_at | 4931406C07Rik | RIKEN cDNA 4931406C07 gene | chr9 | 1.515 | 6 | 0.003 |  | | | |
| 96093\_at | ORF11 | open reading frame 11 | --- | 0.678 | 6 | 0.01 |  | | | |
| 96112\_at | Etfa | electron transferring flavoprotein, alpha polypeptide | chr9 | 1.779 | 6 | 0.006 |  | | | |
| 96231\_at | 2010012D11Rik | RIKEN cDNA 2010012D11 gene | chr13 | 0.868 | 6 | 0.031 |  | | | |
| 96249\_at | Sep15-pending | selenoprotein | chr3 | 0.398 | 6 | 0.031 |  | | | |
| 96261\_at | NoneAvailable | Mus musculus cDNA clone MGC:67622 IMAGE:6410794, complete cds | chr4 | 1.384 | 6 | 0 |  | | | |
| 96267\_at | Ndufv1 | NADH dehydrogenase (ubiquinone) flavoprotein 1 | chr19 | 1.004 | 6 | 0.016 |  | | | |
| 96268\_at | Suclg1 | succinate-CoA ligase, GDP-forming, alpha subunit | chr6 | 1.173 | 6 | 0.015 |  | | | |
| 96289\_at | Stoml2 | stomatin (Epb7.2)-like 2 | chr4 | 2.901 | 6 | 0.001 |  | | | |
| 96291\_f\_at | NoneAvailable | Mus musculus cDNA clone IMAGE:6772417, with apparent retained intron | chr13 | 2.298 | 6 | 0 |  | | | |
| 96292\_r\_at | NoneAvailable | Mus musculus cDNA clone IMAGE:6772417, with apparent retained intron | chr13 | 1.544 | 6 | 0.01 |  | | | |
| 96293\_at | 2410015N17Rik | RIKEN cDNA 2410015N17 gene | chr7 | 4.422 | 6 | 0.006 |  | | | |
| 96319\_at | Cdc20 | cell division cycle 20 homolog (S. cerevisiae) | --- | 4.591 | 6 | 0.004 |  | | | |
| 96321\_at | Ndufa9 | NADH dehydrogenase (ubiquinone) 1 alpha subcomplex, 9 | chr6 | 1.871 | 6 | 0.001 |  | | | |
| 96336\_at | Gatm | glycine amidinotransferase (L-arginine:glycine amidinotransferase) | --- | 0.529 | 6 | 0.022 |  | | | |
| 96345\_at | Sdbcag84 | serologically defined breast cancer antigen 84 | chr2 | 0.341 | 6 | 0.049 |  | | | |
| 96353\_at | 1110021D01Rik | RIKEN cDNA 1110021D01 gene | chr13 | 2.999 | 6 | 0.003 |  | | | |
| 96355\_at | 2900055D03Rik | RIKEN cDNA 2900055D03 gene | --- | 0.736 | 6 | 0.01 |  | | | |
| 96605\_at | 0610011I04Rik | RIKEN cDNA 0610011I04 gene | chr6 | -1.492 | 6 | 0.012 |  | | | |
| 96611\_at | NoneAvailable | Mus musculus cDNA clone IMAGE:5694421, partial cds | chr17 | 0.414 | 6 | 0.001 |  | | | |
| 96613\_at | 5730536A07Rik | RIKEN cDNA 5730536A07 gene | chr9 | 2.658 | 6 | 0.014 |  | | | |
| 96627\_at | Ebp | phenylalkylamine Ca2+ antagonist (emopamil) binding protein | chrX | 1.299 | 6 | 0.009 |  | | | |
| 96634\_at | 5730469M10Rik | RIKEN cDNA 5730469M10 gene | --- | -0.064 | 6 | 0.037 |  | | | |
| 96652\_at | Mrpl28 | mitochondrial ribosomal protein L28 | chr17 | 1.368 | 6 | 0.026 |  | | | |
| 96668\_at | Timm17b | translocator of inner mitochondrial membrane b | chrX | 2.162 | 6 | 0.003 |  | | | |
| 96670\_at | Gstk1 | glutathione S-transferase kappa 1 | chr6 | -0.369 | 6 | 0.033 |  | | | |
| 96678\_at | Dhrs4 | dehydrogenase/reductase (SDR family) member 4 | chr14 | 0.742 | 6 | 0.006 |  | | | |
| 96696\_at | Hrmt1l2 | heterogeneous nuclear ribonucleoproteins  methyltransferase-like 2 (S. cerevisiae) | chr7 | 2.608 | 6 | 0.013 |  | | | |
| 96699\_at | Hmgn1 | high mobility group nucleosomal binding domain 1 | chr11 | 0.795 | 6 | 0.001 |  | | | |
| 96733\_at | Rap1gds1 | RAP1, GTP-GDP dissociation stimulator 1 | chr3 | 0.959 | 6 | 0.024 |  | | | |
| 96734\_at | Synj2bp | synaptojanin 2 binding protein | chr12 | 0.397 | 6 | 0.031 |  | | | |
| 96743\_at | Skiip | SKI interacting protein | --- | 1.897 | 6 | 0.005 |  | | | |
| 96775\_at | Cbx1 | chromobox homolog 1 (Drosophila HP1 beta) | chr11 | 0.466 | 6 | 0.008 |  | | | |
| 96840\_at | Gabarapl2 | GABA(A) receptor-associated protein like 2 | chr5 | -0.619 | 6 | 0.016 |  | | | |
| 96849\_at | Timm8a | translocase of inner mitochondrial membrane 8 homolog a (yeast) | chr10 | 0.859 | 6 | 0.048 |  | | | |
| 96861\_at | Mrpl50 | mitochondrial ribosomal protein L50 | chr4 | 1.687 | 6 | 0.015 |  | | | |
| 96888\_at | Akr1a4 | aldo-keto reductase family 1, member A4 (aldehyde reductase) | chr4 | 0.887 | 6 | 0.042 |  | | | |
| 96892\_at | Psma1 | proteasome (prosome, macropain) subunit, alpha type 1 | chr7 | 1.257 | 6 | 0.017 |  | | | |
| 96899\_at | Ndufs3 | NADH dehydrogenase (ubiquinone) Fe-S protein 3 | chr11 | 1.275 | 6 | 0.019 |  | | | |
| 96902\_at | 2900091E11Rik | RIKEN cDNA 2900091E11 gene | chr10 | 1.339 | 6 | 0.042 |  | | | |
| 96909\_at | Ndufab1 | NADH dehydrogenase (ubiquinone) 1, alpha/beta subcomplex, 1 | chr7 | 1.256 | 6 | 0.014 |  | | | |
| 96947\_at | Etfb | electron transferring flavoprotein, beta polypeptide | chr7 | 2.477 | 6 | 0 |  | | | |
| 96948\_at | Qdpr | quininoid dihydropteridine reductase | chr5 | 0.591 | 6 | 0.006 |  | | | |
| 96952\_at | Psma6 | proteasome (prosome, macropain) subunit, alpha type 6 | chr12 | 1.235 | 6 | 0.004 |  | | | |
| 96956\_at | 0610038D11Rik | RIKEN cDNA 0610038D11 gene | chr19 | 0.405 | 6 | 0.016 |  | | | |
| 97013\_f\_at | Cyba | cytochrome b-245, alpha polypeptide | chr8 | 0.992 | 6 | 0.013 |  | | | |
| 97055\_s\_at | NoneAvailable | --- | chr16 | 1.087 | 6 | 0.01 |  | | | |
| 97164\_at | 2610207P08Rik | RIKEN cDNA 2610207P08 gene | --- | 3.233 | 6 | 0.011 |  | | | |
| 97179\_at | NoneAvailable | Mus musculus mRNA similar to putative c-Myc-responsive (cDNA clone MGC:54855 IMAGE:5388297), complete cds | --- | 2.605 | 6 | 0.001 |  | | | |
| 97200\_f\_at | Snrpe | small nuclear ribonucleoprotein E | chr1 | 0.59 | 6 | 0.041 |  | | | |
| 97201\_s\_at | Ndufa5 | NADH dehydrogenase (ubiquinone) 1 alpha subcomplex, 5 | chr6 | 1.857 | 6 | 0.001 |  | | | |
| 97207\_f\_at | Lypla1 | lysophospholipase 1 | chr1 | 0.831 | 6 | 0.047 |  | | | |
| 97220\_at | Dscr2 | Down syndrome critical region homolog 2 (human) | chr16 | 5.415 | 6 | 0 |  | | | |
| 97248\_at | Dbi | diazepam binding inhibitor | --- | 1.56 | 6 | 0 |  | | | |
| 97268\_i\_at | 0610010I12Rik | RIKEN cDNA 0610010I12 gene | chr3 | 1.265 | 6 | 0.003 |  | | | |
| 97274\_at | Psmd14 | proteasome (prosome, macropain) 26S subunit, non-ATPase, 14 | chr2 | 2.128 | 6 | 0.001 |  | | | |
| 97276\_at | Ckap1 | cytoskeleton-associated protein 1 | --- | 0.402 | 6 | 0.044 |  | | | |
| 97279\_at | 6430402H10Rik | RIKEN cDNA 6430402H10 gene | chr6 | -0.199 | 6 | 0.016 |  | | | |
| 97307\_f\_at | Ndufb5 | NADH dehydrogenase (ubiquinone) 1 beta subcomplex, 5 | --- | 1.376 | 6 | 0.003 |  | | | |
| 97311\_at | 6720456B07Rik | RIKEN cDNA 6720456B07 gene | chr6 | 0.701 | 6 | 0 |  | | | |
| 97318\_at | Hars2 | histidyl tRNA synthetase 2 | chr2 | 0.864 | 6 | 0 |  | | | |
| 97342\_at | Mrps14 | mitochondrial ribosomal protein S14 | chr1 | 2.351 | 6 | 0.001 |  | | | |
| 97346\_at | 2610001J05Rik | RIKEN cDNA 2610001J05 gene | chr6 | 0.616 | 6 | 0.03 |  | | | |
| 97356\_at | 1810008O21Rik | RIKEN cDNA 1810008O21 gene | chr7 | 0.227 | 6 | 0.037 |  | | | |
| 97374\_at | 2810025M15Rik | RIKEN cDNA 2810025M15 gene | chr1 | 2.124 | 6 | 0.003 |  | | | |
| 97395\_at | D19Wsu55e | DNA segment, Chr 19, Wayne State University 55, expressed | --- | 0.449 | 6 | 0.015 |  | | | |
| 97412\_at | 3300001G02Rik | RIKEN cDNA 3300001G02 gene | chr11 | 2.545 | 6 | 0.004 |  | | | |
| 97419\_at | 2310010I22Rik | RIKEN cDNA 2310010I22 gene | chr2 | 0.687 | 6 | 0.007 |  | | | |
| 97424\_at | Arl6ip5 | ADP-ribosylation factor-like 6 interacting protein 5 | chr6 | 0.945 | 6 | 0.001 |  | | | |
| 97449\_at | Aldh7a1 | aldehyde dehydrogenase family 7, member A1 | chr18 | 0.856 | 6 | 0.043 |  | | | |
| 97459\_at | Psma4 | proteasome (prosome, macropain) subunit, alpha type 4 | chr18 | 1.676 | 6 | 0.03 |  | | | |
| 97460\_at | Ube2r2 | ubiquitin-conjugating enzyme E2R 2 | --- | 0.681 | 6 | 0.047 |  | | | |
| 97468\_at | Cks1 | CDC28 protein kinase 1 | chr3 | 2.843 | 6 | 0 |  | | | |
| 97477\_at | Timm8b | translocase of inner mitochondrial membrane 8 homolog b (yeast) | chr9 | 1.26 | 6 | 0.04 |  | | | |
| 97478\_at | C030004C14Rik | RIKEN cDNA C030004C14 gene | chr13 | 0.351 | 6 | 0.002 |  | | | |
| 97512\_at | 2010107E04Rik | RIKEN cDNA 2010107E04 gene | chr12 | 0.955 | 6 | 0.007 |  | | | |
| 97538\_at | Gus | beta-glucuronidase | chr5 | 1.607 | 6 | 0.033 |  | | | |
| 97751\_f\_at | NoneAvailable | --- | --- | 0.47 | 6 | 0.022 |  | | | |
| 97758\_at | Prdx1 | peroxiredoxin 1 | chr8 | 1.211 | 6 | 0.01 |  | | | |
| 97807\_at | 1110021H02Rik | RIKEN cDNA 1110021H02 gene | chr1 | 1.1 | 6 | 0.015 |  | | | |
| 97819\_at | Gsto1 | glutathione S-transferase omega 1 | chr19 | 0.024 | 6 | 0.001 |  | | | |
| 97820\_at | Galk1 | galactokinase 1 | chr11 | 2.703 | 6 | 0.004 |  | | | |
| 97824\_at | D11Ertd175e | DNA segment, Chr 11, ERATO Doi 175, expressed | chr11 | 1.494 | 6 | 0.006 |  | | | |
| 97828\_at | Siva-pending | Cd27 binding protein (Hindu God of destruction) | chr12 | 1.387 | 6 | 0 |  | | | |
| 97884\_at | Mrps11 | mitochondrial ribosomal protein S11 | chr7 | 1.959 | 6 | 0.019 |  | | | |
| 97885\_at | 1810009M01Rik | RIKEN cDNA 1810009M01 gene | chr6 | -0.938 | 6 | 0.043 |  | | | |
| 97907\_at | Lsm7 | LSM7 homolog, U6 small nuclear RNA associated (S. cerevisiae) | chr10 | 1.945 | 6 | 0.002 |  | | | |
| 97979\_at | Ppp1r7 | protein phosphatase 1, regulatory (inhibitor) subunit 7 | chr1 | 2.977 | 6 | 0.019 |  | | | |
| 98039\_at | 2410015M20Rik | RIKEN cDNA 2410015M20 gene | chr17 | 1.43 | 6 | 0.019 |  | | | |
| 98075\_at | G431001I09Rik | RIKEN cDNA G431001I09 gene | chr2 | 3.225 | 6 | 0.001 |  | | | |
| 98077\_at | Snrpd3 | small nuclear ribonucleoprotein D3 | chr10 | 1.794 | 6 | 0.001 |  | | | |
| 98081\_at | Rpo1-3 | RNA polymerase 1-3 | chr4 | 0.959 | 6 | 0.001 |  | | | |
| 98092\_at | Plac8 | placenta-specific 8 | chr5 | 2.981 | 6 | 0.004 |  | | | |
| 98120\_at | Mrpl27 | mitochondrial ribosomal protein L27 | chr11 | 3.683 | 6 | 0.001 |  | | | |
| 98153\_at | Cct3 | chaperonin subunit 3 (gamma) | chr3 | 1.573 | 6 | 0.02 |  | | | |
| 98440\_at | Ltb4dh | leukotriene B4 12-hydroxydehydrogenase | chr4 | 3.855 | 6 | 0 |  | | | |
| 98456\_at | Stk19 | serine/threonine kinase 19 | --- | 0.189 | 6 | 0.008 |  | | | |
| 98492\_at | Cklfsf7 | chemokine-like factor super family 7 | chr9 | 1.661 | 6 | 0.036 |  | | | |
| 98516\_at | NoneAvailable | Mus musculus cDNA clone MGC:67360 IMAGE:6823629, complete cds | chr8 | 0.615 | 6 | 0.016 |  | | | |
| 98524\_f\_at | NoneAvailable | --- | --- | 2.603 | 6 | 0.008 |  | | | |
| 98527\_at | Dci | dodecenoyl-Coenzyme A delta isomerase (3,2 trans-enoyl-Coenyme A isomerase) | chr17 | 1.306 | 6 | 0.011 |  | | | |
| 98557\_f\_at | Psmb4 | proteasome (prosome, macropain) subunit, beta type 4 | --- | 0.285 | 6 | 0.018 |  | | | |
| 98564\_f\_at | Rps26 | ribosomal protein S26 | chr10 | 0.016 | 6 | 0.025 |  | | | |
| 98587\_at | Nap1l1 | nucleosome assembly protein 1-like 1 | chr10 | 0.829 | 6 | 0.015 |  | | | |
| 98595\_at | Irak1 | interleukin-1 receptor-associated kinase 1 | --- | -0.518 | 6 | 0.02 |  | | | |
| 98610\_at | Mrps28 | mitochondrial ribosomal protein S28 | chr3 | 1.473 | 6 | 0.04 |  | | | |
| 98613\_at | 2700085E05Rik | RIKEN cDNA 2700085E05 gene | chr11 | 0.873 | 6 | 0.02 |  | | | |
| 98904\_at | Mrpl35 | mitochondrial ribosomal protein L35 | chr6 | 2.237 | 6 | 0.002 |  | | | |
| 98910\_at | D030012E24Rik | RIKEN cDNA D030012E24 gene | chr11 | 0.973 | 6 | 0.008 |  | | | |
| 98920\_g\_at | 2410018G23Rik | RIKEN cDNA 2410018G23 gene | chrX | -2.085 | 6 | 0.004 |  | | | |
| 98921\_at | 2410018G23Rik | RIKEN cDNA 2410018G23 gene | chr8 | -1.976 | 6 | 0.004 |  | | | |
| 98930\_at | Cope | coatomer protein complex, subunit epsilon | chr8 | 1.116 | 6 | 0.022 |  | | | |
| 98934\_at | 0610007P06Rik | RIKEN cDNA 0610007P06 gene | chr7 | 4.269 | 6 | 0 |  | | | |
| 98938\_at | 1500026D16Rik | RIKEN cDNA 1500026D16 gene | chr19 | 1.602 | 6 | 0.013 |  | | | |
| 98959\_at | 0610016J10Rik | RIKEN cDNA 0610016J10 gene | chr17 | 0.606 | 6 | 0.011 |  | | | |
| 98966\_at | Dbt | dihydrolipoamide branched chain transacylase E2 | chr3 | 1.257 | 6 | 0.033 |  | | | |
| 98975\_at | 2410008G02Rik | RIKEN cDNA 2410008G02 gene | chr8 | 0.732 | 6 | 0.033 |  | | | |
| 99056\_at | Pcbd | 6-pyruvoyl-tetrahydropterin synthase/dimerization cofactor of hepatocyte nuclear factor 1 alpha (TCF1) | chr10 | 0.111 | 6 | 0.008 |  | | | |
| 99106\_at | Cops6 | COP9 (constitutive photomorphogenic) homolog, subunit 6 (Arabidopsis thaliana) | chr5 | 2.396 | 6 | 0.029 |  | | | |
| 99128\_at | Atp5o | ATP synthase, H+ transporting, mitochondrial F1 complex, O subunit | chr16 | 1.704 | 6 | 0.021 |  | | | |
| 99129\_at | Clast3-pending | CD40 ligand-activated specific transcript 3 | chr18 | 1.773 | 6 | 0.039 |  | | | |
| 99147\_at | Znhit1 | zinc finger, HIT domain containing 1 | --- | 0.46 | 6 | 0.042 |  | | | |
| 99148\_at | Fh1 | fumarate hydratase 1 | chr1 | 2.824 | 6 | 0.009 |  | | | |
| 99151\_at | 2610002K22Rik | RIKEN cDNA 2610002K22 gene | --- | 3.469 | 6 | 0.004 |  | | | |
| 99156\_at | 2700099C19Rik | RIKEN cDNA 2700099C19 gene | chrX | 0.68 | 6 | 0.049 |  | | | |
| 99164\_at | 2010111E04Rik | RIKEN cDNA 2010111E04 gene | chr3 | 2.906 | 6 | 0.006 |  | | | |
| 99179\_at | 3010002G01Rik | RIKEN cDNA 3010002G01 gene | chr19 | 0.465 | 6 | 0.011 |  | | | |
| 99182\_at | 2610511E03Rik | RIKEN cDNA 2610511E03 gene | chr14 | 0.954 | 6 | 0.044 |  | | | |
| 99522\_at | Gsg2 | germ cell-specific gene 2 | --- | 0.308 | 6 | 0.008 |  | | | |
| 99537\_at | Ruvbl1 | RuvB-like protein 1 | chr1 | 3.012 | 6 | 0.022 |  | | | |
| 99544\_at | Dguok | deoxyguanosine kinase | chr6 | 0.647 | 6 | 0.034 |  | | | |
| 99546\_at | Fkbp2 | FK506 binding protein 2 | --- | 2.273 | 6 | 0.001 |  | | | |
| 99566\_at | Tpi | triosephosphate isomerase | chr6 | 1.133 | 6 | 0.048 |  | | | |
| 99583\_at | Gstp2 | glutathione S-transferase, pi 2 | chr19 | 1.142 | 6 | 0.003 |  | | | |
| 99586\_at | Cst3 | cystatin C | chr2 | 0.348 | 6 | 0.003 |  | | | |
| 99594\_at | Mrpl51 | mitochondrial ribosomal protein L51 | chr6 | 2.427 | 6 | 0 |  | | | |
| 99613\_at | Mut | methylmalonyl-Coenzyme A mutase | chr17 | 0.426 | 6 | 0.012 |  | | | |
| 99618\_at | 0710008D09Rik | RIKEN cDNA 0710008D09 gene | chr10 | 2.5 | 6 | 0 |  | | | |
| 99632\_at | Mad2l1 | MAD2 (mitotic arrest deficient, homolog)-like 1 (yeast) | chr6 | 1.589 | 6 | 0.018 |  | | | |
| 99651\_at | 2610209M04Rik | RIKEN cDNA 2610209M04 gene | chr6 | 1.176 | 6 | 0.024 |  | | | |
| 99655\_at | 1810012E07Rik | RIKEN cDNA 1810012E07 gene | chr7 | 0.653 | 6 | 0.001 |  | | | |
| 99991\_at | Il17r | interleukin 17 receptor | chr6 | 0.281 | 6 | 0.036 |  | | | |
| 100030\_at | Upp1 | uridine phosphorylase 1 | chr11 | -1.097 | 10 | 0.005 |  | | | |
| 100094\_at | Supt5h | suppressor of Ty 5 homolog (S. cerevisiae) | chr7 | -0.649 | 10 | 0.037 |  | | | |
| 100134\_at | Eng | endoglin | chr2 | -1.282 | 10 | 0.012 |  | | | |
| 100136\_at | Lamp2 | lysosomal membrane glycoprotein 2 | chrX | -1.749 | 10 | 0.025 |  | | | |
| 100293\_at | Cd59b | CD59b antigen | --- | -0.374 | 10 | 0.011 |  | | | |
| 100325\_at | Gp49a | glycoprotein 49 A | chr10 | -0.358 | 10 | 0.001 |  | | | |
| 100333\_at | Saa2 | serum amyloid A 2 | chr7 | -0.09 | 10 | 0.007 |  | | | |
| 100348\_at | NoneAvailable | --- | --- | 2.608 | 10 | 0.029 |  | | | |
| 100397\_at | Tyrobp | TYRO protein tyrosine kinase binding protein | chr7 | 3.755 | 10 | 0.004 |  | | | |
| 100429\_at | Ppox | protoporphyrinogen oxidase | chr1 | -0.282 | 10 | 0 |  | | | |
| 100475\_at | Trim25 | tripartite motif protein 25 | --- | -1.645 | 10 | 0 |  | | | |
| 100486\_at | Ezh1 | enhancer of zeste homolog 1 (Drosophila) | chr11 | -0.745 | 10 | 0.003 |  | | | |
| 100492\_at | Ap2a2 | adaptor protein complex AP-2, alpha 2 subunit | chr7 | 0.607 | 10 | 0.035 |  | | | |
| 100606\_at | Prnp | prion protein | --- | -1.719 | 10 | 0.036 |  | | | |
| 100616\_at | Cenpa | centromere autoantigen A | --- | 0.979 | 10 | 0.013 |  | | | |
| 100635\_at | Sara | SAR1a gene homolog (S. cerevisiae) | chr10 | -1.295 | 10 | 0.009 |  | | | |
| 100710\_at | Vcp | valosin containing protein | chr4 | -0.461 | 10 | 0.033 |  | | | |
| 100877\_at | 1810058I24Rik | RIKEN cDNA 1810058I24 gene | chr6 | 0.025 | 10 | 0.005 |  | | | |
| 100921\_at | Tnni3 | troponin I, cardiac | chr7 | -0.848 | 10 | 0.002 |  | | | |
| 100939\_at | Zfp282 | zinc finger protein 282 | chr6 | -0.161 | 10 | 0.006 |  | | | |
| 100973\_i\_at | Ccl27 | chemokine (C-C motif) ligand 27 | --- | -3.092 | 10 | 0 |  | | | |
| 100988\_at | Bcl2l11 | BCL2-like 11 (apoptosis facilitator) | chr2 | -1.027 | 10 | 0.014 |  | | | |
| 101014\_at | Ifnar2 | interferon (alpha and beta) receptor 2 | chr16 | 0.225 | 10 | 0.032 |  | | | |
| 101036\_at | 1810060K07Rik | RIKEN cDNA 1810060K07 gene | --- | -0.149 | 10 | 0.019 |  | | | |
| 101073\_at | Lrp1 | low density lipoprotein receptor-related protein 1 | chr10 | -0.016 | 10 | 0.002 |  | | | |
| 101079\_at | Nxf1 | nuclear RNA export factor 1 homolog (S. cerevisiae) | chr19 | -2.424 | 10 | 0.003 |  | | | |
| 101144\_at | Il18r1 | interleukin 18 receptor 1 | --- | -1.522 | 10 | 0.037 |  | | | |
| 101186\_at | Ppnr-pending | per-pentamer repeat gene | chr19 | -1.941 | 10 | 0.019 |  | | | |
| 101432\_at | 2410019G02Rik | RIKEN cDNA 2410019G02 gene | chr11 | -0.055 | 10 | 0.022 |  | | | |
| 101441\_i\_at | Itpr5 | inositol 1,4,5-triphosphate receptor 5 | chr6 | -3.278 | 10 | 0.019 |  | | | |
| 101446\_at | Tpd52l1 | tumor protein D52-like 1 | chr10 | -0.068 | 10 | 0.04 |  | | | |
| 101449\_at | Trim41 | tripartite motif-containing 41 | chr11 | -0.832 | 10 | 0.009 |  | | | |
| 101697\_f\_at | NoneAvailable | --- | --- | -0.037 | 10 | 0.027 |  | | | |
| 101778\_at | Gja5 | gap junction membrane channel protein alpha 5 | chr3 | -0.024 | 10 | 0.013 |  | | | |
| 101835\_at | Lrmp | lymphoid-restricted membrane protein | chr6 | 0.24 | 10 | 0.027 |  | | | |
| 101836\_at | Ppm1b | protein phosphatase 1B, magnesium dependent, beta isoform | chr17 | -1.457 | 10 | 0.007 |  | | | |
| 101844\_at | Pipox | pipecolic acid oxidase | chr11 | -0.024 | 10 | 0.008 |  | | | |
| 101884\_at | Xlr4 | X-linked lymphocyte-regulated 4 | chrX | -2.072 | 10 | 0.038 |  | | | |
| 101889\_s\_at | Rora | RAR-related orphan receptor alpha | chr9 | -0.911 | 10 | 0.004 |  | | | |
| 101943\_at | Tceb3 | transcription elongation factor B (SIII), polypeptide 3 | chr4 | -1.496 | 10 | 0.002 |  | | | |
| 101947\_at | Nakap95-pending | neighbor of A-kinase anchoring protein 95 | chr17 | -1.799 | 10 | 0.001 |  | | | |
| 102024\_at | Ncoa3 | nuclear receptor coactivator 3 | chr2 | -0.905 | 10 | 0.014 |  | | | |
| 102063\_at | Pdpk1 | 3-phosphoinositide dependent protein kinase-1 | chr17 | -0.713 | 10 | 0.01 |  | | | |
| 102091\_f\_at | NoneAvailable | --- | --- | -0.219 | 10 | 0.003 |  | | | |
| 102101\_f\_at | NoneAvailable | --- | --- | -0.105 | 10 | 0.004 |  | | | |
| 102104\_f\_at | NoneAvailable | --- | chr19 | 1.072 | 10 | 0.038 |  | | | |
| 102242\_at | Per3 | period homolog 3 (Drosophila) | chr4 | -0.182 | 10 | 0.046 |  | | | |
| 102260\_at | Gfi1b | growth factor independent 1B | chr2 | 0.788 | 10 | 0.001 |  | | | |
| 102279\_at | 1300004C08Rik | RIKEN cDNA 1300004C08 gene | chr9 | -2.168 | 10 | 0.008 |  | | | |
| 102313\_at | Gch | GTP cyclohydrolase 1 | chr14 | -4.039 | 10 | 0.005 |  | | | |
| 102382\_at | Arntl | aryl hydrocarbon receptor nuclear translocator-like | chr7 | -0.06 | 10 | 0.004 |  | | | |
| 102580\_r\_at | NoneAvailable | --- | chr6 | 0.113 | 10 | 0.044 |  | | | |
| 102658\_at | Il1r2 | interleukin 1 receptor, type II | chr1 | -1.46 | 10 | 0 |  | | | |
| 102663\_at | Plaur | urokinase plasminogen activator receptor | --- | -2.014 | 10 | 0.036 |  | | | |
| 102781\_at | Ccnl2 | cyclin L2 | chr4 | -1.306 | 10 | 0.005 |  | | | |
| 102787\_at | Gpr56 | G protein-coupled receptor 56 | chr8 | -1.614 | 10 | 0.021 |  | | | |
| 102836\_at | Pps | putative phosphatase | chr11 | -1.69 | 10 | 0 |  | | | |
| 102895\_at | 4921518A06Rik | RIKEN cDNA 4921518A06 gene | chr15 | -0.554 | 10 | 0.029 |  | | | |
| 102980\_at | Nmt1 | N-myristoyltransferase 1 | chr11 | -0.153 | 10 | 0.045 |  | | | |
| 102991\_s\_at | H2-Ke6 | H2-K region expressed gene 6 | chr17 | -0.88 | 10 | 0.009 |  | | | |
| 103015\_at | Bcl6 | B-cell leukemia/lymphoma 6 | chr16 | -4.658 | 10 | 0.02 |  | | | |
| 103033\_at | C4 | complement component 4 (within H-2S) | chr17 | -0.069 | 10 | 0.04 |  | | | |
| 103051\_at | Expi | extracellular proteinase inhibitor | chr11 | -0.064 | 10 | 0 |  | | | |
| 103080\_at | Samhd1 | SAM domain and HD domain, 1 | chr2 | 0.08 | 10 | 0.003 |  | | | |
| 103210\_at | Csf2rb2 | colony stimulating factor 2 receptor, beta 2, low-affinity (granulocyte-macrophage) | chr15 | -1.2 | 10 | 0.02 |  | | | |
| 103218\_at | Slc10a3 | solute carrier family 10 (sodium/bile acid cotransporter family), member 3 | chrX | -0.604 | 10 | 0.045 |  | | | |
| 103251\_at | 2310010M10Rik | RIKEN cDNA 2310010M10 gene | chr14 | -0.107 | 10 | 0.004 |  | | | |
| 103254\_at | Fln29-pending | FLN29 gene product | chr5 | -2.179 | 10 | 0.011 |  | | | |
| 103259\_at | Gfi1 | growth factor independent 1 | --- | 1.14 | 10 | 0.031 |  | | | |
| 103314\_at | D13Ertd275e | DNA segment, Chr 13, ERATO Doi 275, expressed | chr13 | 0.006 | 10 | 0.002 |  | | | |
| 103366\_at | AU043488 | expressed sequence AU043488 | chr10 | 0.069 | 10 | 0.022 |  | | | |
| 103408\_at | AI325941 | expressed sequence AI325941 | chr7 | 0.021 | 10 | 0.004 |  | | | |
| 103414\_at | Skiv2l | superkiller viralicidic activity 2-like (S. cerevisiae ) | chr17 | 0.059 | 10 | 0.038 |  | | | |
| 103422\_at | Cd1d1 | CD1d1 antigen | chr3 | -2.191 | 10 | 0.001 |  | | | |
| 103443\_at | Aim1 | absent in melanoma 1 | chr10 | -1.49 | 10 | 0.005 |  | | | |
| 103504\_at | Ssbp2 | single-stranded DNA binding protein 2 | chr13 | 0.016 | 10 | 0.003 |  | | | |
| 103518\_at | Ctla2b | cytotoxic T lymphocyte-associated protein 2 beta | chr13 | -5.219 | 10 | 0.015 |  | | | |
| 103545\_at | 2610019E17Rik | RIKEN cDNA 2610019E17 gene | chr17 | -0.409 | 10 | 0.027 |  | | | |
| 103562\_f\_at | NoneAvailable | --- | --- | -0.059 | 10 | 0.018 |  | | | |
| 103582\_r\_at | 6130401J04Rik | RIKEN cDNA 6130401J04 gene | chr1 | -0.175 | 10 | 0.038 |  | | | |
| 103596\_at | Dgka | diacylglycerol kinase, alpha | chr10 | -3.305 | 10 | 0 |  | | | |
| 103656\_at | Lancl1 | LanC (bacterial lantibiotic synthetase component C)-like | chr1 | -0.561 | 10 | 0.005 |  | | | |
| 103672\_at | 2410141M05Rik | RIKEN cDNA 2410141M05 gene | chr11 | -1.198 | 10 | 0.001 |  | | | |
| 103690\_at | Wbscr5 | Williams-Beuren syndrome chromosome region 5 homolog (human) | --- | 0.578 | 10 | 0.028 |  | | | |
| 103704\_at | 2010305K11Rik | RIKEN cDNA 2010305K11 gene | chr8 | -0.023 | 10 | 0.043 |  | | | |
| 103773\_at | 1110020K19Rik | RIKEN cDNA 1110020K19 gene | chr17 | -0.139 | 10 | 0.04 |  | | | |
| 103812\_at | Clca1 | chloride channel calcium activated 1 | chr3 | -4.597 | 10 | 0.005 |  | | | |
| 103845\_at | Slc31a1 | solute carrier family 31, member 1 | chr4 | -0.413 | 10 | 0.033 |  | | | |
| 103890\_at | AW538196 | expressed sequence AW538196 | chr7 | -0.467 | 10 | 0.031 |  | | | |
| 103893\_at | 6030410I24Rik | RIKEN cDNA 6030410I24 gene | chr18 | 0.036 | 10 | 0.003 |  | | | |
| 103895\_at | AW549877 | expressed sequence AW549877 | chr15 | -1.037 | 10 | 0.03 |  | | | |
| 103988\_at | A830054M12 | hypothetical protein A830054M12 | chr19 | -0.467 | 10 | 0.004 |  | | | |
| 104048\_at | Cars | cysteinyl-tRNA synthetase | chr7 | -0.836 | 10 | 0.017 |  | | | |
| 104060\_at | 2700088M22Rik | RIKEN cDNA 2700088M22 gene | chr15 | 0.135 | 10 | 0.036 |  | | | |
| 104144\_at | Gtpbp2 | GTP binding protein 2 | chr17 | -1.316 | 10 | 0.016 |  | | | |
| 104150\_at | 2810008P14Rik | RIKEN cDNA 2810008P14 gene | chr5 | 0.012 | 10 | 0.018 |  | | | |
| 104165\_at | Vnn1 | vanin 1 | --- | -2.98 | 10 | 0.003 |  | | | |
| 104184\_at | Nppb | natriuretic peptide precursor type B | --- | -0.088 | 10 | 0.032 |  | | | |
| 104206\_at | 0610012A05Rik | RIKEN cDNA 0610012A05 gene | chr15 | -7.151 | 10 | 0.003 |  | | | |
| 104217\_at | 1110015E22Rik | RIKEN cDNA 1110015E22 gene | chr7 | -0.233 | 10 | 0.035 |  | | | |
| 104219\_f\_at | Pcbp2 | poly(rC) binding protein 2 | --- | -0.24 | 10 | 0.037 |  | | | |
| 104256\_at | Pscdbp | pleckstrin homology, Sec7 and coiled-coil domains, binding protein | chr2 | -2.279 | 10 | 0.022 |  | | | |
| 104257\_g\_at | Pscdbp | pleckstrin homology, Sec7 and coiled-coil domains, binding protein | chr2 | -2.012 | 10 | 0.004 |  | | | |
| 104263\_at | 9330177P20Rik | RIKEN cDNA 9330177P20 gene | chr4 | -1.251 | 10 | 0.004 |  | | | |
| 104285\_at | Hmgcr | 3-hydroxy-3-methylglutaryl-Coenzyme A reductase | chr13 | -0.78 | 10 | 0.045 |  | | | |
| 104311\_at | 1300013G12Rik | RIKEN cDNA 1300013G12 gene | chr1 | -1.417 | 10 | 0.024 |  | | | |
| 104324\_at | Masp2 | mannan-binding lectin serine protease 2 | chr4 | -0.813 | 10 | 0.01 |  | | | |
| 104340\_at | Mbd1 | methyl-CpG binding domain protein 1 | chr18 | -0.587 | 10 | 0.036 |  | | | |
| 104364\_at | Mapkapk5 | MAP kinase-activated protein kinase 5 | chr5 | -0.358 | 10 | 0.029 |  | | | |
| 104371\_at | Dgat1 | diacylglycerol O-acyltransferase 1 | chr15 | -3.266 | 10 | 0 |  | | | |
| 104388\_at | Ccl9 | chemokine (C-C motif) ligand 9 | chr11 | 1.346 | 10 | 0.03 |  | | | |
| 104425\_at | Cipp | channel-interacting PDZ domain protein | chr4 | -0.357 | 10 | 0.011 |  | | | |
| 104453\_at | NoneAvailable | Mus musculus cDNA clone IMAGE:6433799, partial cds | chr11 | -1.124 | 10 | 0.03 |  | | | |
| 104471\_at | Hdac6 | histone deacetylase 6 | chrX | -0.009 | 10 | 0.007 |  | | | |
| 104533\_at | Pim1 | proviral integration site 1 | chr17 | -0.582 | 10 | 0.038 |  | | | |
| 104572\_at | Etohd2 | ethanol decreased 2 | chr13 | -1.512 | 10 | 0.001 |  | | | |
| 104574\_at | 5730453I16Rik | RIKEN cDNA 5730453I16 gene | chr19 | -0.933 | 10 | 0.013 |  | | | |
| 104677\_at | LOC227619 | hypothetical protein LOC227619 | chr2 | -1.818 | 10 | 0.046 |  | | | |
| 104683\_at | AA407558 | expressed sequence AA407558 | --- | 0.247 | 10 | 0.021 |  | | | |
| 104692\_at | Selp | selectin, platelet | chr1 | -0.542 | 10 | 0.006 |  | | | |
| 104701\_at | Bhlhb2 | basic helix-loop-helix domain containing, class B2 | chr6 | -3.085 | 10 | 0.023 |  | | | |
| 104714\_at | AA959601 | expressed sequence AA959601 | chr14 | -0.945 | 10 | 0 |  | | | |
| 104741\_at | 9530098M12Rik | RIKEN cDNA 9530098M12 gene | chrX | -1.016 | 10 | 0.015 |  | | | |
| 104742\_at | Mgst2 | microsomal glutathione S-transferase 2 | chr3 | -0.5 | 10 | 0.033 |  | | | |
| 104745\_at | Arl6ip2 | ADP-ribosylation factor-like 6 interacting protein 2 | chr17 | -1.189 | 10 | 0.045 |  | | | |
| 160088\_at | Fmo5 | flavin containing monooxygenase 5 | chr3 | -1.409 | 10 | 0.019 |  | | | |
| 160099\_at | Lgals4 | lectin, galactose binding, soluble 4 | chr7 | -1.876 | 10 | 0.001 |  | | | |
| 160140\_at | Tbce | tubulin-specific chaperone e | chr13 | -0.152 | 10 | 0.007 |  | | | |
| 160151\_i\_at | 1200009B18Rik | RIKEN cDNA 1200009B18 gene | chr6 | -1.735 | 10 | 0.029 |  | | | |
| 160174\_at | 0610041O14Rik | RIKEN cDNA 0610041O14 gene | chr8 | -0.442 | 10 | 0.01 |  | | | |
| 160182\_at | Sfrs6 | splicing factor, arginine/serine-rich 6 | chr2 | -0.282 | 10 | 0.005 |  | | | |
| 160220\_at | Zfp110 | zinc finger protein 110 | --- | 0.113 | 10 | 0.013 |  | | | |
| 160264\_s\_at | 1500036F01Rik | RIKEN cDNA 1500036F01 gene | chr1 | -1.44 | 10 | 0.029 |  | | | |
| 160283\_at | 2410005K20Rik | RIKEN cDNA 2410005K20 gene | --- | 0.077 | 10 | 0.048 |  | | | |
| 160287\_at | Map1lc3 | microtubule-associated protein 1 light chain 3 | chr14 | -1.669 | 10 | 0.02 |  | | | |
| 160316\_at | NoneAvailable | Mus musculus 0 day neonate thymus cDNA, RIKEN full-length enriched library, clone:A430083K13 product:unknown EST, full insert sequence | --- | -0.182 | 10 | 0.003 |  | | | |
| 160393\_at | 4930555L11Rik | RIKEN cDNA 4930555L11 gene | chr6 | -2.283 | 10 | 0.001 |  | | | |
| 160394\_at | D930014A20Rik | RIKEN cDNA D930014A20 gene | chr12 | 0.071 | 10 | 0.039 |  | | | |
| 160396\_at | 0610013I17Rik | RIKEN cDNA 0610013I17 gene | --- | -0.866 | 10 | 0.015 |  | | | |
| 160425\_at | 2410017I18Rik | RIKEN cDNA 2410017I18 gene | chr13 | -0.393 | 10 | 0.043 |  | | | |
| 160461\_f\_at | 2310057H16Rik | RIKEN cDNA 2310057H16 gene | chr18 | -0.341 | 10 | 0.01 |  | | | |
| 160495\_at | Ahr | aryl-hydrocarbon receptor | chr12 | -3.189 | 10 | 0.004 |  | | | |
| 160512\_at | BC017643 | cDNA sequence BC017643 | chr11 | -0.317 | 10 | 0.028 |  | | | |
| 160592\_at | Tmc6 | transmembrane channel-like gene family 6 | --- | -0.646 | 10 | 0.016 |  | | | |
| 160624\_at | NoneAvailable | Mus musculus, clone IMAGE:5401580, mRNA | chr4 | -0.596 | 10 | 0.036 |  | | | |
| 160698\_s\_at | Prkcd | protein kinase C, delta | chr14 | -0.279 | 10 | 0.034 |  | | | |
| 160742\_at | Plod3 | procollagen-lysine, 2-oxoglutarate 5-dioxygenase 3 | --- | -0.075 | 10 | 0.043 |  | | | |
| 160762\_at | Abr | active BCR-related gene | chr11 | 0.372 | 10 | 0.006 |  | | | |
| 160781\_r\_at | Unc93b | unc-93 homolog B (C. elegans) | chr19 | -0.132 | 10 | 0.011 |  | | | |
| 160832\_at | Ldlr | low density lipoprotein receptor | chr9 | 1.302 | 10 | 0.036 |  | | | |
| 160834\_at | 1110032C13Rik | RIKEN cDNA 1110032C13 gene | chr7 | -4.49 | 10 | 0.04 |  | | | |
| 160920\_at | Bcl2l2 | Bcl2-like 2 | chr14 | -1.72 | 10 | 0.02 |  | | | |
| 160965\_at | AA793972 | EST AA793972 | chr5 | -3.294 | 10 | 0.001 |  | | | |
| 160977\_at | Arhgef5 | Rho guanine nucleotide exchange factor (GEF) 5 | chr6 | -3.899 | 10 | 0.031 |  | | | |
| 161005\_at | 5730420B22Rik | RIKEN cDNA 5730420B22 gene | --- | -0.111 | 10 | 0.015 |  | | | |
| 161039\_at | Adam22 | a disintegrin and metalloprotease domain 22 | chr5 | -0.147 | 10 | 0.018 |  | | | |
| 161060\_i\_at | 2310061O04Rik | RIKEN cDNA 2310061O04 gene | chr5 | -0.211 | 10 | 0.047 |  | | | |
| 161077\_f\_at | Smarcd2 | SWI/SNF related, matrix associated, actin dependent regulator of chromatin, subfamily d, member 2 | chr11 | -0.15 | 10 | 0.024 |  | | | |
| 161081\_at | Cpeb2 | cytoplasmic polyadenylation element binding protein 2 | chr5 | -3.809 | 10 | 0.02 |  | | | |
| 161083\_at | A130052D22 | hypothetical protein A130052D22 | --- | 0.607 | 10 | 0.002 |  | | | |
| 161109\_at | 1110017P05Rik | RIKEN cDNA 1110017P05 gene | --- | -1.394 | 10 | 0.011 |  | | | |
| 161112\_at | LOC214424 | hypothetical protein LOC214424 | chr9 | 0.183 | 10 | 0 |  | | | |
| 161113\_at | Esr1 | estrogen receptor 1 (alpha) | chr10 | -1.744 | 10 | 0.007 |  | | | |
| 161165\_f\_at | Lpin2 | lipin 2 | chr17 | -0.341 | 10 | 0.036 |  | | | |
| 161187\_f\_at | 5730589K01Rik | RIKEN cDNA 5730589K01 gene | --- | -0.457 | 10 | 0.002 |  | | | |
| 161333\_f\_at | D1Ertd161e | DNA segment, Chr 1, ERATO Doi 161, expressed | chr1 | -0.304 | 10 | 0.01 |  | | | |
| 161396\_f\_at | NoneAvailable | --- | --- | -0.498 | 10 | 0.011 |  | | | |
| 161530\_r\_at | Sema4a | sema domain, immunoglobulin domain (Ig), transmembrane domain (TM) and short cytoplasmic domain, (semaphorin) 4A | --- | -0.218 | 10 | 0.045 |  | | | |
| 161551\_f\_at | Riok3 | RIO kinase 3 (yeast) | --- | -1.464 | 10 | 0.012 |  | | | |
| 161610\_at | Ndr2 | N-myc downstream regulated 2 | --- | -4.394 | 10 | 0.001 |  | | | |
| 161667\_r\_at | 1110001M20Rik | RIKEN cDNA 1110001M20 gene | chr4 | 0.017 | 10 | 0.041 |  | | | |
| 161689\_f\_at | Il1r2 | interleukin 1 receptor, type II | chr1 | -3.446 | 10 | 0 |  | | | |
| 161745\_f\_at | Hspa4 | heat shock protein 4 | chr11 | -0.317 | 10 | 0.002 |  | | | |
| 161785\_f\_at | D5Wsu46e | DNA segment, Chr 5, Wayne State University 46, expressed | chr5 | -0.441 | 10 | 0.027 |  | | | |
| 161814\_f\_at | Rnf19 | ring finger protein (C3HC4 type) 19 | chr15 | -1.939 | 10 | 0.035 |  | | | |
| 161881\_f\_at | Zfp259 | zinc finger protein 259 | chr9 | -0.311 | 10 | 0.002 |  | | | |
| 161899\_f\_at | Wbscr5 | Williams-Beuren syndrome chromosome region 5 homolog (human) | chr5 | 0.701 | 10 | 0.002 |  | | | |
| 161980\_f\_at | Bag3 | Bcl2-associated athanogene 3 | chr7 | -4.017 | 10 | 0.037 |  | | | |
| 162041\_f\_at | NoneAvailable | --- | --- | -1.038 | 10 | 0.01 |  | | | |
| 162092\_f\_at | Ihpk1 | inositol hexaphosphate kinase 1 | chr9 | -0.407 | 10 | 0.044 |  | | | |
| 162114\_f\_at | Usp49 | ubiquitin specific protease 49 | chr17 | -0.67 | 10 | 0.008 |  | | | |
| 162204\_r\_at | Notch1 | Notch gene homolog 1 (Drosophila) | chr2 | -0.352 | 10 | 0.011 |  | | | |
| 162206\_f\_at | Socs3 | suppressor of cytokine signaling 3 | --- | -4.307 | 10 | 0.013 |  | | | |
| 162228\_f\_at | NoneAvailable | Mus musculus transcribed sequences | --- | -0.565 | 10 | 0.001 |  | | | |
| 162260\_at | 6330407G11Rik | RIKEN cDNA 6330407G11 gene | chr17 | -0.728 | 10 | 0.009 |  | | | |
| 162261\_f\_at | Zp2 | zona pellucida glycoprotein 2 | --- | -0.329 | 10 | 0.023 |  | | | |
| 162384\_f\_at | Ccrn4l | CCR4 carbon catabolite repression 4-like (S. cerevisiae) | --- | -0.633 | 10 | 0.025 |  | | | |
| 162424\_f\_at | 2610007K22Rik | RIKEN cDNA 2610007K22 gene | chr15 | -0.846 | 10 | 0.02 |  | | | |
| 162463\_at | Tpd52 | tumor protein D52 | --- | -0.325 | 10 | 0.047 |  | | | |
| 92208\_at | C1qdc1 | C1q domain containing 1 | chr6 | -0.521 | 10 | 0.003 |  | | | |
| 92217\_s\_at | NoneAvailable | --- | chr10 | 0.093 | 10 | 0.02 |  | | | |
| 92232\_at | Socs3 | suppressor of cytokine signaling 3 | chr11 | -0.365 | 10 | 0.003 |  | | | |
| 92233\_at | 1810007M14Rik | RIKEN cDNA 1810007M14 gene | chr16 | -0.913 | 10 | 0.015 |  | | | |
| 92300\_at | Mnt | max binding protein | chr11 | -0.573 | 10 | 0.007 |  | | | |
| 92318\_at | 2010301N04Rik | RIKEN cDNA 2010301N04 gene | chr6 | -0.432 | 10 | 0.019 |  | | | |
| 92339\_at | Taf1a | TATA box binding protein (Tbp)-associated factor, RNA polymerase I, A | --- | -0.038 | 10 | 0.002 |  | | | |
| 92400\_at | Ndst2 | N-deacetylase/N-sulfotransferase (heparan glucosaminyl) 2 | chr14 | -0.661 | 10 | 0.004 |  | | | |
| 92542\_at | D4Wsu53e | DNA segment, Chr 4, Wayne State University 53, expressed | chr4 | -1.236 | 10 | 0.014 |  | | | |
| 92571\_at | Hspa4 | heat shock protein 4 | chr11 | -0.736 | 10 | 0.002 |  | | | |
| 92634\_at | Dpp4 | dipeptidylpeptidase 4 | chr2 | -0.409 | 10 | 0.044 |  | | | |
| 92708\_at | 2810457M08Rik | RIKEN cDNA 2810457M08 gene | chr8 | -0.324 | 10 | 0.031 |  | | | |
| 92737\_at | Irf4 | interferon regulatory factor 4 | chr13 | -0.317 | 10 | 0.008 |  | | | |
| 92758\_at | Dusp2 | dual specificity phosphatase 2 | chr2 | -0.942 | 10 | 0.002 |  | | | |
| 92877\_at | Tgfbi | transforming growth factor, beta induced | chr13 | -1.788 | 10 | 0.029 |  | | | |
| 92918\_at | F7 | coagulation factor VII | chr8 | -0.006 | 10 | 0.04 |  | | | |
| 92945\_at | NoneAvailable | --- | chr3 | 0.395 | 10 | 0.005 |  | | | |
| 92986\_g\_at | Ptprj | protein tyrosine phosphatase, receptor type, J | chr12 | -0.245 | 10 | 0.028 |  | | | |
| 92991\_at | Sp4 | trans-acting transcription factor 4 | chr12 | -0.902 | 10 | 0.04 |  | | | |
| 92992\_i\_at | 5730497N03Rik | RIKEN cDNA 5730497N03 gene | chr12 | -1.16 | 10 | 0.027 |  | | | |
| 92993\_r\_at | 5730497N03Rik | RIKEN cDNA 5730497N03 gene | chr12 | -1.744 | 10 | 0.003 |  | | | |
| 93104\_at | Btg1 | B-cell translocation gene 1, anti-proliferative | chr10 | -3.713 | 10 | 0.005 |  | | | |
| 93193\_at | Adrb2 | adrenergic receptor, beta 2 | chr18 | -1.617 | 10 | 0.021 |  | | | |
| 93199\_at | NoneAvailable | M.musculus mRNA for L41 ribosomal like-protein | --- | -0.244 | 10 | 0.033 |  | | | |
| 93274\_at | Clk | CDC-like kinase | chr1 | -1.211 | 10 | 0.031 |  | | | |
| 93311\_at | Clk3 | CDC-like kinase 3 | chr9 | -1.389 | 10 | 0.003 |  | | | |
| 93315\_at | Map2k3 | mitogen activated protein kinase kinase 3 | chr11 | -2.248 | 10 | 0.044 |  | | | |
| 93319\_at | Rasa3 | RAS p21 protein activator 3 | chr8 | -0.464 | 10 | 0.004 |  | | | |
| 93321\_at | Ifi203 | interferon activated gene 203 | chr1 | -0.168 | 10 | 0.003 |  | | | |
| 93339\_at | Mdm4 | transformed mouse 3T3 cell double minute 4 | chr1 | -0.202 | 10 | 0.004 |  | | | |
| 93408\_at | NoneAvailable | Mus musculus transcribed sequences | chr8 | -0.44 | 10 | 0.001 |  | | | |
| 93414\_at | Abcb1b | ATP-binding cassette, sub-family B (MDR/TAP), member 1B | chr5 | -1.239 | 10 | 0.019 |  | | | |
| 93424\_at | NoneAvailable | Mus musculus, Similar to KIAA0916 protein, clone IMAGE:4022573, mRNA | chr14 | -1.14 | 10 | 0.024 |  | | | |
| 93440\_at | 4930564D15Rik | RIKEN cDNA 4930564D15 gene | chr3 | -1.001 | 10 | 0.017 |  | | | |
| 93492\_at | Pscd2 | pleckstrin homology, Sec7 and coiled-coil domains 2 | chr7 | -0.593 | 10 | 0.022 |  | | | |
| 93509\_at | Ube2b | ubiquitin-conjugating enzyme E2B, RAD6 homology (S. cerevisiae) | chr11 | -0.724 | 10 | 0.049 |  | | | |
| 93520\_at | Srrm1 | serine/arginine repetitive matrix 1 | --- | -1.338 | 10 | 0.018 |  | | | |
| 93557\_at | Sps2 | selenophosphate synthetase 2 | chr7 | -0.116 | 10 | 0.016 |  | | | |
| 93570\_at | Slc12a3 | solute carrier family 12, member 3 | chr8 | -0.004 | 10 | 0.011 |  | | | |
| 93627\_at | E430019N21Rik | RIKEN cDNA E430019N21 gene | chr14 | -0.592 | 10 | 0.002 |  | | | |
| 93701\_at | Smarca5 | SWI/SNF related, matrix associated, actin dependent regulator of chromatin, subfamily a, member 5 | chr4 | 0.12 | 10 | 0.01 |  | | | |
| 93718\_at | Rab23 | RAB23, member RAS oncogene family | --- | -0.035 | 10 | 0.016 |  | | | |
| 93744\_at | Calm4 | calmodulin 4 | chr13 | -0.05 | 10 | 0.041 |  | | | |
| 93753\_at | Litaf | LPS-induced TN factor | chr16 | -1.716 | 10 | 0.013 |  | | | |
| 93852\_at | Mef2a | myocyte enhancer factor 2A | chr7 | -2.066 | 10 | 0.044 |  | | | |
| 93909\_f\_at | NoneAvailable | --- | --- | -0.211 | 10 | 0.002 |  | | | |
| 93914\_at | Il1r1 | interleukin 1 receptor, type I | chr1 | -1.087 | 10 | 0.006 |  | | | |
| 93965\_r\_at | Ddx6 | DEAD (Asp-Glu-Ala-Asp) box polypeptide 6 | chr9 | -3.007 | 10 | 0.007 |  | | | |
| 93975\_at | 1300002F13Rik | RIKEN cDNA 1300002F13 gene | chr4 | -6.877 | 10 | 0 |  | | | |
| 93978\_at | D230016N13Rik | RIKEN cDNA D230016N13 gene | --- | -0.085 | 10 | 0.016 |  | | | |
| 93980\_at | BC019943 | cDNA sequence BC019943 | --- | 0.418 | 10 | 0.048 |  | | | |
| 94003\_at | NoneAvailable | Mus musculus cDNA clone IMAGE:6490905, partial cds | chr6 | -0.8 | 10 | 0.049 |  | | | |
| 94006\_at | Azi2 | 5-azacytidine induced gene 2 | chr16 | -0.771 | 10 | 0 |  | | | |
| 94011\_at | 3100004P22Rik | RIKEN cDNA 3100004P22 gene | chr7 | -0.355 | 10 | 0.011 |  | | | |
| 94012\_at | Timm13a | translocase of inner mitochondrial membrane 13 homolog a (yeast) | --- | 0.276 | 10 | 0.026 |  | | | |
| 94042\_f\_at | Gng5 | guanine nucleotide binding protein (G protein), gamma 5 subunit | chr15 | -0.485 | 10 | 0.006 |  | | | |
| 94192\_at | Gdap10 | ganglioside-induced differentiation-associated-protein 10 | chr12 | -2.38 | 10 | 0.047 |  | | | |
| 94224\_s\_at | Ifi205 | interferon activated gene 205 | chr1 | -0.908 | 10 | 0.002 |  | | | |
| 94236\_at | Nisch | nischarin | chr14 | -0.278 | 10 | 0.002 |  | | | |
| 94264\_at | Raf1 | v-raf-1 leukemia viral oncogene 1 | chr6 | -1.251 | 10 | 0.001 |  | | | |
| 94300\_f\_at | 2310042E05Rik | RIKEN cDNA 2310042E05 gene | chr10 | -0.68 | 10 | 0.001 |  | | | |
| 94331\_at | Stat6 | signal transducer and activator of transcription 6 | chr10 | -2.796 | 10 | 0 |  | | | |
| 94341\_at | Jmj | jumonji | chr13 | -0.648 | 10 | 0.036 |  | | | |
| 94397\_at | 1200014O24Rik | RIKEN cDNA 1200014O24 gene | chr10 | 0.361 | 10 | 0.002 |  | | | |
| 94483\_at | Csnk2a2 | casein kinase II, alpha 2, polypeptide | chr8 | -1.076 | 10 | 0.04 |  | | | |
| 94484\_at | Hbs1l | Hbs1-like (S. cerevisiae) | chr10 | 0.209 | 10 | 0.017 |  | | | |
| 94689\_at | C79248 | expressed sequence C79248 | --- | -1.2 | 10 | 0.015 |  | | | |
| 94780\_at | Zfp288 | zinc finger protein 288 | chr16 | -4.353 | 10 | 0.034 |  | | | |
| 94818\_at | Ogt | O-linked N-acetylglucosamine (GlcNAc) transferase (UDP-N-acetylglucosamine:polypeptide-N-acetylglucosaminyl transferase) | chrX | -1.371 | 10 | 0.004 |  | | | |
| 94830\_at | BC005537 | cDNA sequence BC005537 | chr13 | -1.83 | 10 | 0.008 |  | | | |
| 94899\_at | Rhoip3-pending | Rho interacting protein 3 | chr11 | -1.473 | 10 | 0.042 |  | | | |
| 94928\_at | Tnfrsf1b | tumor necrosis factor receptor superfamily, member 1b | --- | -1.859 | 10 | 0.009 |  | | | |
| 94932\_at | Pdgfa | platelet derived growth factor, alpha | chr5 | -0.678 | 10 | 0.022 |  | | | |
| 94939\_at | Cd53 | CD53 antigen | chr3 | -1.067 | 10 | 0.004 |  | | | |
| 94951\_at | 1810030A06Rik | RIKEN cDNA 1810030A06 gene | chrX | 0.853 | 10 | 0.03 |  | | | |
| 94979\_at | BC018507 | cDNA sequence BC018507 | chr13 | -0.219 | 10 | 0.021 |  | | | |
| 94980\_at | Dusp11 | dual specificity phosphatase 11 (RNA/RNP complex 1-interacting) | chr6 | -1.6 | 10 | 0.004 |  | | | |
| 95021\_at | 9430010O03Rik | RIKEN cDNA 9430010O03 gene | chr8 | -0.858 | 10 | 0.002 |  | | | |
| 95023\_at | BC023957 | cDNA sequence BC023957 | chr9 | -1.742 | 10 | 0 |  | | | |
| 95081\_at | P38ip-pending | transcription factor (p38 interacting protein) | chr3 | -0.31 | 10 | 0.03 |  | | | |
| 95095\_at | Flot1 | flotillin 1 | chr17 | 0.746 | 10 | 0.007 |  | | | |
| 95119\_at | 1110038D17Rik | RIKEN cDNA 1110038D17 gene | chr10 | -1.366 | 10 | 0.009 |  | | | |
| 95123\_at | 4930566A11Rik | RIKEN cDNA 4930566A11 gene | chr8 | -0.035 | 10 | 0.024 |  | | | |
| 95155\_at | B830022L21Rik | RIKEN cDNA B830022L21 gene | chr8 | -0.064 | 10 | 0.023 |  | | | |
| 95287\_at | NoneAvailable | Mus musculus RIKEN cDNA 4930471C18 gene, mRNA (cDNA clone IMAGE:4487650), partial cds | chr6 | -1.232 | 10 | 0.032 |  | | | |
| 95295\_s\_at | Flt3 | FMS-like tyrosine kinase 3 | chr5 | 0.496 | 10 | 0.006 |  | | | |
| 95355\_at | Agtrap | angiotensin II, type I receptor-associated protein | --- | -0.466 | 10 | 0.012 |  | | | |
| 95382\_at | NoneAvailable | Mus musculus transcribed sequences | --- | -0.751 | 10 | 0 |  | | | |
| 95387\_f\_at | Sema4b | sema domain, immunoglobulin domain (Ig), transmembrane domain (TM) and short cytoplasmic domain, (semaphorin) 4B | chr7 | 0.341 | 10 | 0.032 |  | | | |
| 95398\_at | NoneAvailable | Mus musculus transcribed sequences | chr1 | -0.096 | 10 | 0.047 |  | | | |
| 95433\_at | Ddx54 | DEAD (Asp-Glu-Ala-Asp) box polypeptide 54 | chr5 | 1.761 | 10 | 0.017 |  | | | |
| 95444\_at | 4930579A11Rik | RIKEN cDNA 4930579A11 gene | chr11 | -2.862 | 10 | 0.016 |  | | | |
| 95489\_at | Fliih | flightless I homolog (Drosophila) | chr11 | -1.074 | 10 | 0.042 |  | | | |
| 95501\_at | 2410001C21Rik | RIKEN cDNA 2410001C21 gene | --- | 0.09 | 10 | 0.016 |  | | | |
| 95502\_at | Sirt2 | sirtuin 2 (silent mating type information regulation 2, homolog) 2 (S. cerevisiae) | chr7 | -0.093 | 10 | 0.034 |  | | | |
| 95521\_s\_at | Zfp68 | Zinc finger protein 68 | chr5 | -2.132 | 10 | 0.035 |  | | | |
| 95533\_at | Zfp106 | zinc finger protein 106 | --- | -0.751 | 10 | 0.014 |  | | | |
| 95536\_at | Tceb3 | transcription elongation factor B (SIII), polypeptide 3 | chr4 | -0.823 | 10 | 0.003 |  | | | |
| 95564\_at | BC018601 | cDNA sequence BC018601 | chr11 | -2.789 | 10 | 0.032 |  | | | |
| 95573\_at | Baz2a | bromodomain adjacent to zinc finger domain, 2A | chr10 | -0.788 | 10 | 0 |  | | | |
| 95574\_f\_at | Cggbp1 | CGG triplet repeat binding protein 1 | --- | -0.136 | 10 | 0.035 |  | | | |
| 95586\_at | P2rx4 | purinergic receptor P2X, ligand-gated ion channel 4 | chr5 | -1.711 | 10 | 0 |  | | | |
| 95607\_at | Stard3 | START domain containing 3 | chr11 | -0.126 | 10 | 0.019 |  | | | |
| 95647\_f\_at | 4022402H07Rik | RIKEN cDNA 4022402H07 gene | chr6 | -0.734 | 10 | 0.01 |  | | | |
| 95655\_at | 5830411E10Rik | RIKEN cDNA 5830411E10 gene | chr1 | -1.472 | 10 | 0.036 |  | | | |
| 95686\_at | Rab14 | RAB14, member RAS oncogene family | --- | -0.214 | 10 | 0.01 |  | | | |
| 95694\_at | Top1 | topoisomerase (DNA) I | chr2 | 0.495 | 10 | 0.006 |  | | | |
| 95917\_at | NoneAvailable | Mus musculus transcribed sequences | chr8 | -4.563 | 10 | 0.041 |  | | | |
| 96056\_at | Arhc | ras homolog gene family, member C | chr3 | -0.212 | 10 | 0.043 |  | | | |
| 96176\_at | Arih2 | ariadne homolog 2 (Drosophila) | chr9 | -1.874 | 10 | 0.022 |  | | | |
| 96189\_at | 2410141K03Rik | RIKEN cDNA 2410141K03 gene | --- | -2.437 | 10 | 0.013 |  | | | |
| 96196\_i\_at | 5730589K01Rik | RIKEN cDNA 5730589K01 gene | --- | -0.202 | 10 | 0.021 |  | | | |
| 96197\_f\_at | 5730589K01Rik | RIKEN cDNA 5730589K01 gene | --- | -0.685 | 10 | 0.022 |  | | | |
| 96300\_f\_at | Rps27 | ribosomal protein S27 | chr3 | -0.761 | 10 | 0.034 |  | | | |
| 96333\_g\_at | Snx2 | sorting nexin 2 | chr18 | 0.109 | 10 | 0.041 |  | | | |
| 96481\_at | C80638 | expressed sequence C80638 | chr3 | -0.708 | 10 | 0.009 |  | | | |
| 96511\_s\_at | Vav1 | vav 1 oncogene | --- | 0.266 | 10 | 0.046 |  | | | |
| 96534\_at | Vldlr | very low density lipoprotein receptor | chr19 | -1.008 | 10 | 0 |  | | | |
| 96538\_at | Centb1 | centaurin, beta 1 | chr11 | -0.106 | 10 | 0.041 |  | | | |
| 96545\_s\_at | A730042J05Rik | RIKEN cDNA A730042J05 gene | chr8 | -0.115 | 10 | 0.018 |  | | | |
| 96577\_i\_at | NoneAvailable | --- | chr3 | 1.694 | 10 | 0.033 |  | | | |
| 96578\_r\_at | NoneAvailable | --- | --- | -0.683 | 10 | 0.046 |  | | | |
| 96618\_at | 0610037N01Rik | RIKEN cDNA 0610037N01 gene | chr17 | 0.03 | 10 | 0.009 |  | | | |
| 96650\_at | Auh | AU RNA binding protein/enoyl-coenzyme A hydratase | chr13 | 0.434 | 10 | 0.001 |  | | | |
| 96751\_at | 2810410P22Rik | RIKEN cDNA 2810410P22 gene | chr2 | 0.168 | 10 | 0.001 |  | | | |
| 96784\_at | 2900037I21Rik | RIKEN cDNA 2900037I21 gene | chr9 | 1.467 | 10 | 0.045 |  | | | |
| 96813\_f\_at | DXImx46e | DNA segment, Chr X, Immunex 46, expressed | chrX | -1.437 | 10 | 0.027 |  | | | |
| 96817\_at | 2700067D09Rik | RIKEN cDNA 2700067D09 gene | chr16 | -0.617 | 10 | 0.012 |  | | | |
| 96818\_at | Dtx2 | deltex 2 homolog (Drosophila) | chr5 | -0.13 | 10 | 0.039 |  | | | |
| 96845\_at | Slc30a9 | solute carrier family 30 (zinc transporter), member 9 | chr5 | -0.927 | 10 | 0.002 |  | | | |
| 97083\_at | Eif2s2 | eukaryotic translation initiation factor 2, subunit 2 (beta) | chr2 | -0.842 | 10 | 0.018 |  | | | |
| 97118\_at | 1810028B20Rik | RIKEN cDNA 1810028B20 gene | chr19 | -1.614 | 10 | 0.036 |  | | | |
| 97285\_f\_at | Ubxdc2 | UBX domain-containing 2 | chr17 | -2.548 | 10 | 0 |  | | | |
| 97297\_at | 1500036F01Rik | RIKEN cDNA 1500036F01 gene | chr1 | -1.772 | 10 | 0.001 |  | | | |
| 97319\_at | Rrad | Ras-related associated with diabetes | chr8 | -2.544 | 10 | 0.005 |  | | | |
| 97349\_at | 4930488L10Rik | RIKEN cDNA 4930488L10 gene | chr12 | -2.819 | 10 | 0.04 |  | | | |
| 97384\_at | Gmfg | glia maturation factor, gamma | chr7 | 0.552 | 10 | 0.004 |  | | | |
| 97386\_at | 1110032O19Rik | RIKEN cDNA 1110032O19 gene | chr8 | -0.598 | 10 | 0.031 |  | | | |
| 97429\_at | Snrk | SNF related kinase | chr9 | -1.935 | 10 | 0.021 |  | | | |
| 97497\_at | Notch1 | Notch gene homolog 1 (Drosophila) | chr2 | 1.19 | 10 | 0.011 |  | | | |
| 97509\_f\_at | Fgfr1 | fibroblast growth factor receptor 1 | chr8 | -0.746 | 10 | 0.003 |  | | | |
| 97593\_f\_at | Fliih | flightless I homolog (Drosophila) | chr11 | -0.685 | 10 | 0.014 |  | | | |
| 97710\_f\_at | C530046L02Rik | RIKEN cDNA C530046L02 gene | chr17 | -0.132 | 10 | 0 |  | | | |
| 97843\_at | Ncoa4 | nuclear receptor coactivator 4 | chr12 | -1.253 | 10 | 0.003 |  | | | |
| 97848\_at | Rbmx | RNA binding motif protein, X chromosome | chrX | -0.951 | 10 | 0.013 |  | | | |
| 97897\_at | NoneAvailable | Mus musculus, clone IMAGE:6430978, mRNA | chr13 | -1.708 | 10 | 0.027 |  | | | |
| 97936\_at | 2810407L07Rik | RIKEN cDNA 2810407L07 gene | chr6 | -0.421 | 10 | 0.034 |  | | | |
| 98000\_at | Ly64 | lymphocyte antigen 64 | chr16 | -2.084 | 10 | 0.036 |  | | | |
| 98002\_at | Icsbp1 | interferon consensus sequence binding protein 1 | chr8 | 1.28 | 10 | 0.01 |  | | | |
| 98018\_at | Procr | protein C receptor, endothelial | chr2 | -4.119 | 10 | 0.038 |  | | | |
| 98057\_at | 1110031E24Rik | RIKEN cDNA 1110031E24 gene | chr8 | -0.259 | 10 | 0.001 |  | | | |
| 98356\_at | NoneAvailable | Mus musculus transcribed sequences | chr16 | -0.627 | 10 | 0.03 |  | | | |
| 98435\_at | Adss | adenylosuccinate synthetase, muscle | chr12 | 0.249 | 10 | 0.027 |  | | | |
| 98461\_at | 1200014P03Rik | RIKEN cDNA 1200014P03 gene | chr17 | -1.307 | 10 | 0.007 |  | | | |
| 98465\_f\_at | Ifi204 | interferon activated gene 204 | chr1 | 0.141 | 10 | 0.002 |  | | | |
| 98495\_at | 5033414D02Rik | RIKEN cDNA 5033414D02 gene | chr19 | -0.739 | 10 | 0.006 |  | | | |
| 98533\_at | Cyb5 | cytochrome b-5 | chr18 | -1.221 | 10 | 0.003 |  | | | |
| 98534\_at | Sap18 | Sin3-associated polypeptide 18 | chr14 | -0.144 | 10 | 0.029 |  | | | |
| 98767\_at | Yy1 | YY1 transcription factor | chr12 | -0.558 | 10 | 0.008 |  | | | |
| 98818\_at | Nr3c1 | nuclear receptor subfamily 3, group C, member 1 | chr18 | 0.019 | 10 | 0.013 |  | | | |
| 98862\_at | Wnt10a | wingless related MMTV integration site 10a | chr1 | -0.233 | 10 | 0.011 |  | | | |
| 98882\_s\_at | Ndel1 | nuclear distribution gene E-like homolog 1 (A. nidulans) | chr11 | -1.562 | 10 | 0 |  | | | |
| 98884\_r\_at | Ndel1 | nuclear distribution gene E-like homolog 1 (A. nidulans) | chr11 | -2.864 | 10 | 0.022 |  | | | |
| 98926\_at | Vamp2 | vesicle-associated membrane protein 2 | chr11 | -2.233 | 10 | 0 |  | | | |
| 98951\_at | D8Ertd325e | DNA segment, Chr 8, ERATO Doi 325, expressed | chr8 | -1.148 | 10 | 0.009 |  | | | |
| 99045\_at | Eno2 | enolase 2, gamma neuronal | --- | -1.634 | 10 | 0.008 |  | | | |
| 99085\_at | Usp3 | ubiquitin specific protease 3 | chr9 | 0.285 | 10 | 0.034 |  | | | |
| 99086\_g\_at | Usp3 | ubiquitin specific protease 3 | chr9 | -0.289 | 10 | 0.017 |  | | | |
| 99100\_at | Stat3 | signal transducer and activator of transcription 3 | chr11 | -1.118 | 10 | 0.022 |  | | | |
| 99103\_at | Irf3 | interferon regulatory factor 3 | chr7 | -1.113 | 10 | 0.042 |  | | | |
| 99143\_at | Tgoln1 | trans-golgi network protein | chr6 | -2.457 | 10 | 0.021 |  | | | |
| 99160\_s\_at | 1110025J15Rik | RIKEN cDNA 1110025J15 gene | chr15 | -0.778 | 10 | 0.017 |  | | | |
| 99184\_at | Csad | cysteine sulfinic acid decarboxylase | --- | -2.259 | 10 | 0.001 |  | | | |
| 99187\_f\_at | 2010315L10Rik | RIKEN cDNA 2010315L10 gene | chr8 | -1.207 | 10 | 0.004 |  | | | |
| 99188\_at | 2010315L10Rik | RIKEN cDNA 2010315L10 gene | chr8 | -1.031 | 10 | 0.002 |  | | | |
| 99347\_f\_at | NoneAvailable | Mus musculus transcribed sequences | --- | -2.205 | 10 | 0.048 |  | | | |
| 99445\_at | 1110028E10Rik | RIKEN cDNA 1110028E10 gene | chr9 | -1.202 | 10 | 0.035 |  | | | |
| 99458\_i\_at | Mark2 | MAP/microtubule affinity-regulating kinase 2 | chr19 | -0.678 | 10 | 0.022 |  | | | |
| 99617\_at | LOC218811 | hypothetical protein LOC218811 | chr14 | -0.043 | 10 | 0.027 |  | | | |
| 99665\_at | Satb1 | special AT-rich sequence binding protein 1 | chr17 | 0.476 | 10 | 0.024 |  | | | |
| 99953\_at | Rgl2 | ral guanine nucleotide dissociation stimulator,-like 2 | chr17 | -0.019 | 10 | 0.009 |  | | | |
| 99985\_at | Txnrd1 | thioredoxin reductase 1 | chr10 | -1.205 | 10 | 0.045 |  | | | |
| 100115\_at | 2610028H07Rik | RIKEN cDNA 2610028H07 gene | chr9 | 0.017 | 30 | 0.036 |  | | | |
| 100462\_at | Arf6 | ADP-ribosylation factor 6 | chr12 | -0.219 | 30 | 0.018 |  | | | |
| 100482\_at | BC023040 | cDNA sequence BC023040 | chr17 | -1.285 | 30 | 0.005 |  | | | |
| 100583\_at | Igh-VJ558 | immunoglobulin heavy chain (J558 family) | chr12 | -1.148 | 30 | 0.003 |  | | | |
| 100696\_at | Pde6a | phosphodiesterase 6A, cGMP-specific, rod, alpha | --- | -0.117 | 30 | 0.041 |  | | | |
| 100972\_s\_at | Ccl27 | chemokine (C-C motif) ligand 27 | --- | -0.931 | 30 | 0.001 |  | | | |
| 101352\_g\_at | NoneAvailable | --- | chr7 | 0.015 | 30 | 0.005 |  | | | |
| 101843\_at | Sh2bpsm1 | SH2-B PH domain containing signaling mediator 1 | chr7 | -0.153 | 30 | 0.007 |  | | | |
| 102028\_at | Rassf5 | Ras association (RalGDS/AF-6) domain family 5 | chr1 | 0.398 | 30 | 0.014 |  | | | |
| 102152\_f\_at | NoneAvailable | --- | --- | -0.22 | 30 | 0.006 |  | | | |
| 102224\_at | Igf1r | insulin-like growth factor I receptor | --- | -1.399 | 30 | 0.011 |  | | | |
| 102302\_at | Bckdhb | branched chain ketoacid dehydrogenase E1, beta polypeptide | chr9 | -1.776 | 30 | 0.019 |  | | | |
| 102335\_at | Kcnk1 | potassium channel, subfamily K, member 1 | chr8 | -0.114 | 30 | 0.025 |  | | | |
| 102691\_at | Zfp385 | zinc finger protein 385 | chr15 | -0.099 | 30 | 0.045 |  | | | |
| 102789\_at | Gata2 | GATA binding protein 2 | chr6 | -1.995 | 30 | 0.048 |  | | | |
| 102873\_at | Tap2 | transporter 2, ATP-binding cassette, sub-family B (MDR/TAP) | chr17 | -0.371 | 30 | 0.007 |  | | | |
| 103052\_r\_at | Nr2f2 | nuclear receptor subfamily 2, group F, member 2 | chr7 | -0.983 | 30 | 0.018 |  | | | |
| 103091\_at | Relb | avian reticuloendotheliosis viral (v-rel) oncogene related B | chr7 | -0.368 | 30 | 0 |  | | | |
| 103236\_at | Ring1 | ring finger protein 1 | chr17 | -0.114 | 30 | 0.04 |  | | | |
| 103547\_at | Slc41a1 | solute carrier family 41, member 1 | chr1 | -2.728 | 30 | 0 |  | | | |
| 103666\_at | Hoxb5 | homeo box B5 | chr11 | -0.825 | 30 | 0.035 |  | | | |
| 103727\_at | D730048C23Rik | RIKEN cDNA D730048C23 gene | --- | -0.44 | 30 | 0.037 |  | | | |
| 103793\_at | Mvp | major vault protein | --- | -0.37 | 30 | 0.006 |  | | | |
| 103954\_at | Reg3a | regenerating islet-derived 3 alpha | chr6 | 0.122 | 30 | 0 |  | | | |
| 103958\_g\_at | Trfr | transferrin receptor | chr16 | -0.439 | 30 | 0.046 |  | | | |
| 104083\_at | Cdh5 | cadherin 5 | chr8 | -1.968 | 30 | 0.003 |  | | | |
| 104139\_at | P4ha1 | procollagen-proline, 2-oxoglutarate 4-dioxygenase (proline 4-hydroxylase), alpha 1 polypeptide | chr10 | -0.637 | 30 | 0.006 |  | | | |
| 104243\_r\_at | 4930578F06Rik | RIKEN cDNA 4930578F06 gene | --- | -0.225 | 30 | 0.036 |  | | | |
| 104287\_at | Smt3ip1-pending | smt3-specific isopeptidase 1 | chr11 | -0.382 | 30 | 0.03 |  | | | |
| 104292\_at | Eya2 | eyes absent 2 homolog (Drosophila) | chr2 | -0.122 | 30 | 0.042 |  | | | |
| 104363\_at | NoneAvailable | Mus musculus cDNA clone MGC:60763 IMAGE:30058959, complete cds | chr17 | -0.22 | 30 | 0.035 |  | | | |
| 104376\_at | Hdac5 | histone deacetylase 5 | chr11 | -2.263 | 30 | 0.006 |  | | | |
| 104417\_at | NoneAvailable | Mus musculus transcribed sequences | chr11 | -2.062 | 30 | 0.001 |  | | | |
| 104438\_at | Zfp30 | zinc finger protein 30 | chr7 | -0.926 | 30 | 0.018 |  | | | |
| 104645\_at | Klf7 | Kruppel-like factor 7 (ubiquitous) | chr1 | -1.19 | 30 | 0.003 |  | | | |
| 160228\_at | 1110019C08Rik | RIKEN cDNA 1110019C08 gene | chr16 | -1.025 | 30 | 0.025 |  | | | |
| 160244\_at | Fem1a | feminization 1 homolog a (C. elegans) | chr17 | 0 | 30 | 0.005 |  | | | |
| 160433\_at | 2400010D15Rik | RIKEN cDNA 2400010D15 gene | chr18 | -0.08 | 30 | 0 |  | | | |
| 160463\_at | Myd116 | myeloid differentiation primary response gene 116 | chr7 | -0.282 | 30 | 0.048 |  | | | |
| 160498\_at | Ldb1 | LIM domain binding 1 | chr19 | 0.031 | 30 | 0.041 |  | | | |
| 160606\_r\_at | Adamts1 | a disintegrin-like and metalloprotease (reprolysin type) with thrombospondin type 1 motif, 1 | chr16 | -0.354 | 30 | 0.009 |  | | | |
| 160651\_at | Tacstd2 | tumor-associated calcium signal transducer 2 | chr6 | -3.696 | 30 | 0 |  | | | |
| 160686\_at | 5730555F13Rik | RIKEN cDNA 5730555F13 gene | --- | -0.692 | 30 | 0.044 |  | | | |
| 160727\_at | 2410002F23Rik | RIKEN cDNA 2410002F23 gene | chr2 | -1.032 | 30 | 0.005 |  | | | |
| 160776\_at | D1Ucla4 | DNA segment, Chr 1, University of California at Los Angeles 4 | chr1 | -0.971 | 30 | 0.006 |  | | | |
| 160789\_at | 9530090G24Rik | RIKEN cDNA 9530090G24 gene | chr2 | 0.122 | 30 | 0.008 |  | | | |
| 160802\_at | Ppan | peter pan homolog (Drosophila) | chr9 | 0.48 | 30 | 0.015 |  | | | |
| 160915\_at | B4galt3 | UDP-Gal:betaGlcNAc beta 1,4-galactosyltransferase, polypeptide 3 | chr1 | -0.179 | 30 | 0.004 |  | | | |
| 160957\_at | D12Ertd7e | DNA segment, Chr 12, ERATO Doi 7, expressed | chr12 | 1.614 | 30 | 0.04 |  | | | |
| 161080\_f\_at | 1700012P16Rik | RIKEN cDNA 1700012P16 gene | chr5 | -1.148 | 30 | 0.008 |  | | | |
| 161148\_f\_at | Ing4 | inhibitor of growth family, member 4 | chr6 | -0.94 | 30 | 0.02 |  | | | |
| 161184\_f\_at | Tie1 | tyrosine kinase receptor 1 | chr4 | -1.63 | 30 | 0.006 |  | | | |
| 161348\_r\_at | Pdlim1 | PDZ and LIM domain 1 (elfin) | --- | 0.078 | 30 | 0.02 |  | | | |
| 161392\_f\_at | Lrpb7 | leucine rich protein, B7 gene | chr6 | -0.815 | 30 | 0.022 |  | | | |
| 161534\_f\_at | 1110038D17Rik | RIKEN cDNA 1110038D17 gene | --- | -0.194 | 30 | 0.034 |  | | | |
| 161576\_f\_at | Fln29-pending | FLN29 gene product | chr5 | -0.323 | 30 | 0.031 |  | | | |
| 161613\_at | 6330407G04Rik | RIKEN cDNA 6330407G04 gene | --- | -0.392 | 30 | 0.039 |  | | | |
| 161738\_f\_at | Ilvbl | ilvB (bacterial acetolactate synthase)-like | chr10 | -0.679 | 30 | 0.01 |  | | | |
| 161990\_f\_at | BC012974 | hypothetical gene supported by BC012974 | chr18 | -1.702 | 30 | 0.006 |  | | | |
| 162034\_r\_at | Antxr2 | anthrax toxin receptor 2 | --- | -0.317 | 30 | 0.008 |  | | | |
| 162080\_f\_at | 2900008M13Rik | RIKEN cDNA 2900008M13 gene | --- | -0.379 | 30 | 0.012 |  | | | |
| 92249\_g\_at | Nr4a2 | nuclear receptor subfamily 4, group A, member 2 | chr2 | -5.506 | 30 | 0.001 |  | | | |
| 92821\_at | Usp2 | ubiquitin specific protease 2 | chr9 | -1.119 | 30 | 0.004 |  | | | |
| 93382\_at | Pde1b | phosphodiesterase 1B, Ca2+-calmodulin dependent | chr15 | -0.176 | 30 | 0.034 |  | | | |
| 93425\_at | Irf5 | interferon regulatory factor 5 | chr6 | -0.888 | 30 | 0.031 |  | | | |
| 93702\_at | AI462446 | expressed sequence AI462446 | chr9 | -0.25 | 30 | 0.015 |  | | | |
| 93768\_f\_at | 2700059D21Rik | RIKEN cDNA 2700059D21 gene | chr4 | -0.412 | 30 | 0.006 |  | | | |
| 93875\_at | Hspa1a | heat shock protein 1A | chr17 | -1.082 | 30 | 0.006 |  | | | |
| 94022\_at | Gltscr2 | glioma tumor suppressor candidate region gene 2 | --- | -0.708 | 30 | 0.015 |  | | | |
| 94060\_at | Myo1h | myosin 1H | chr5 | -1.495 | 30 | 0.002 |  | | | |
| 94146\_at | Ccl4 | chemokine (C-C motif) ligand 4 | chr11 | 0.033 | 30 | 0.009 |  | | | |
| 94657\_at | NoneAvailable | Mus musculus transcribed sequences | chr8 | -5.096 | 30 | 0.006 |  | | | |
| 94796\_at | Psmd11 | proteasome (prosome, macropain) 26S subunit, non-ATPase, 11 | chr11 | 0.269 | 30 | 0.027 |  | | | |
| 94976\_at | AL022610 | expressed sequence AL022610 | chr7 | -1.012 | 30 | 0.029 |  | | | |
| 95001\_at | Akap8 | A kinase (PRKA) anchor protein 8 | chr17 | -0.175 | 30 | 0.019 |  | | | |
| 95002\_at | D17Wsu92e | DNA segment, Chr 17, Wayne State University 92, expressed | chr17 | -1.483 | 30 | 0.016 |  | | | |
| 95012\_at | Slc22a17 | solute carrier family 22 (organic cation transporter), member 17 | chr14 | -0.358 | 30 | 0.042 |  | | | |
| 95016\_at | Nrp | neuropilin | chr8 | 0.029 | 30 | 0.021 |  | | | |
| 95033\_at | Jmjd1 | jumonji domain containing 1 | chr6 | -1.446 | 30 | 0.007 |  | | | |
| 95291\_r\_at | NoneAvailable | Mus musculus transcribed sequences | chr11 | -0.592 | 30 | 0.027 |  | | | |
| 95599\_at | Siat4c | sialyltransferase 4C (beta-galactoside alpha-2,3-sialytransferase) | --- | -0.319 | 30 | 0.002 |  | | | |
| 95618\_at | D6Ertd32e | DNA segment, Chr 6, ERATO Doi 32, expressed | chr6 | -2.616 | 30 | 0.038 |  | | | |
| 95805\_at | Cdc2l2 | cell division cycle 2 homolog (S. pombe)-like 2 | chr4 | -1.429 | 30 | 0.02 |  | | | |
| 95961\_at | BC042396 | cDNA sequence BC042396 | chr9 | 0.125 | 30 | 0.003 |  | | | |
| 96076\_at | Stx5a | syntaxin 5A | chr19 | -1.273 | 30 | 0.008 |  | | | |
| 96088\_at | Ndr2 | N-myc downstream regulated 2 | chr14 | -1.173 | 30 | 0.019 |  | | | |
| 96147\_at | Mafg | v-maf musculoaponeurotic fibrosarcoma oncogene family, protein G (avian) | chr11 | -1.157 | 30 | 0.03 |  | | | |
| 96219\_at | 1810031K02Rik | RIKEN cDNA 1810031K02 gene | chr4 | -0.449 | 30 | 0.043 |  | | | |
| 96270\_at | D11Bwg0434e | DNA segment, Chr 11, Brigham & Women's Genetics 0434 expressed | chr11 | -0.318 | 30 | 0.014 |  | | | |
| 96367\_at | NoneAvailable | Mus musculus transcribed sequences | chr17 | -1.93 | 30 | 0.01 |  | | | |
| 96373\_at | NoneAvailable | --- | chr6 | -0.11 | 30 | 0.035 |  | | | |
| 96637\_at | Tbc1d1 | TBC1 domain family, member 1 | chr5 | 0.29 | 30 | 0.001 |  | | | |
| 96669\_at | 2400003C14Rik | RIKEN cDNA 2400003C14 gene | chr8 | -1.563 | 30 | 0.026 |  | | | |
| 96790\_f\_at | A530057M15Rik | RIKEN cDNA A530057M15 gene | --- | -0.632 | 30 | 0.007 |  | | | |
| 96829\_at | D19Wsu162e | DNA segment, Chr 19, Wayne State University 162, expressed | chr19 | -0.857 | 30 | 0.006 |  | | | |
| 96911\_at | Gnb2 | guanine nucleotide binding protein, beta 2 | chr5 | 1.137 | 30 | 0.031 |  | | | |
| 97125\_f\_at | LOC56628 | MHC (A.CA/J(H-2K-f) class I antigen | chr17 | -3.415 | 30 | 0.007 |  | | | |
| 97375\_at | Pkd1 | polycystic kidney disease 1 homolog | chr17 | -1.292 | 30 | 0.009 |  | | | |
| 97563\_f\_at | NoneAvailable | --- | --- | -0.019 | 30 | 0.03 |  | | | |
| 97684\_at | Prkcabp | protein kinase C, alpha binding protein | chr15 | 0.123 | 30 | 0.005 |  | | | |
| 97813\_at | Rela | v-rel reticuloendotheliosis viral oncogene homolog A (avian) | chr19 | -0.853 | 30 | 0.014 |  | | | |
| 97901\_at | Ubtf | upstream binding transcription factor, RNA polymerase I | chr11 | 0.558 | 30 | 0.013 |  | | | |
| 98065\_at | Ormdl3 | ORM1-like 3 (S. cerevisiae) | chr11 | -1.914 | 30 | 0.032 |  | | | |
| 98385\_at | Ptpn14 | protein tyrosine phosphatase, non-receptor type 14 | chr1 | -0.019 | 30 | 0.021 |  | | | |
| 98402\_at | Macf1 | microtubule-actin crosslinking factor 1 | chr4 | -0.533 | 30 | 0.031 |  | | | |
| 98426\_at | Ppt2 | palmitoyl-protein thioesterase 2 | chr17 | -0.1 | 30 | 0.013 |  | | | |
| 98438\_f\_at | H2-Q7 | histocompatibility 2, Q region locus 7 | chr17 | -3.59 | 30 | 0.001 |  | | | |
| 98861\_at | Sts | steroid sulfatase | --- | -0.975 | 30 | 0.046 |  | | | |
| 98906\_at | Fbxo9 | f-box only protein 9 | chr9 | -2.278 | 30 | 0.037 |  | | | |
| 99142\_at | Mpra-pending | membrane progestin receptor alpha | chr4 | 0.136 | 30 | 0.013 |  | | | |
| 99440\_at | Nfib | nuclear factor I/B | chr4 | -0.459 | 30 | 0.045 |  | | | |
| 99587\_at | Rab7 | RAB7, member RAS oncogene family | chr6 | -0.623 | 30 | 0.032 |  | | | |
| 99644\_at | Zfp289 | zinc finger protein 289 | chr2 | -0.157 | 30 | 0.022 |  | | | |
| 99961\_s\_at | Cdc2l2 | cell division cycle 2 homolog (S. pombe)-like 2 | chr4 | -1.587 | 30 | 0.025 |  | | | |
| 99964\_at | Vdr | vitamin D receptor | chr15 | -0.749 | 30 | 0.034 |  | | | |
| 99970\_at | Ptpn21 | protein tyrosine phosphatase, non-receptor type 21 | chr12 | -1.262 | 30 | 0.008 |  | | | |
| 99992\_at | Il17r | interleukin 17 receptor | chr6 | 0.087 | 30 | 0.02 |  | | | |
| \* Positive log2 fold changes represent genes expressed higher in FL-HSC; Negative log2 fold changes represent genes expressed higher in adult HSC (fold change=2 is equivalent to log2 fold change=1) | | | | | | | | | | |
|  |  |  |  |  |  |  |  |  |  |  |
