# Supplementary material for: Molecular Signatures of Proliferation and Quiescence in Hematopoietic Stem Cells
Source: PLoS Biol. 2004 Sep 28;2(10):e301. doi: 10.1371/journal.pbio.0020301 (PMC520599; doi:10.1371/journal.pbio.0020301)
Supplement: Table S4 — (790 KB HTML). [file pbio.0020301.st004.html]

|  |  | Quiescence group |  |  |  |  |  |  |  |  |
| Probe Set ID | Gene Symbol | Gene name | Chromosome | Log2 Fold Change (FL-HSC vs Adult HSC)\* | Day of max (TOM) | p-value of ANOVA (time course) |  | | | |
| 100322\_at | Igh-4 | immunoglobulin heavy chain 4 (serum IgG1) | --- | -0.13 | 0 | 0.024 |  | | | |
| 100473\_at | 1200006I17Rik | RIKEN cDNA 1200006I17 gene | chr7 | -0.11 | 0 | 0.034 |  | | | |
| 100533\_s\_at | Crem | cAMP responsive element modulator | --- | -5.967 | 0 | 0.008 |  | | | |
| 100582\_at | Snx17 | sorting nexin 17 | chr5 | 0.273 | 0 | 0.012 |  | | | |
| 100914\_at | NoneAvailable | Mus musculus 13 days embryo spinal cord cDNA, RIKEN full-length enriched library, clone:G630020J15 product:unclassifiable, full insert sequence | --- | 0.433 | 0 | 0.046 |  | | | |
| 101072\_at | BC010348 | cDNA sequence BC010348 | chr10 | 0.306 | 0 | 0.025 |  | | | |
| 101426\_at | Cerk | ceramide kinase | chr15 | -0.726 | 0 | 0.022 |  | | | |
| 101515\_at | Acox1 | acyl-Coenzyme A oxidase 1, palmitoyl | --- | -3.857 | 0 | 0.018 |  | | | |
| 101897\_g\_at | Cd1d2 | CD1d2 antigen | chr3 | -0.949 | 0 | 0.024 |  | | | |
| 101940\_at | Smpd2 | sphingomyelin phosphodiesterase 2, neutral | chr10 | -0.013 | 0 | 0.039 |  | | | |
| 101962\_at | 2610007K22Rik | RIKEN cDNA 2610007K22 gene | chr15 | -0.339 | 0 | 0.015 |  | | | |
| 102021\_at | Il4ra | interleukin 4 receptor, alpha | chr7 | -0.384 | 0 | 0.033 |  | | | |
| 102156\_f\_at | NoneAvailable | --- | chr6 | -3.755 | 0 | 0.005 |  | | | |
| 102209\_at | Nfatc1 | nuclear factor of activated T-cells, cytoplasmic 1 | chr18 | -2.297 | 0 | 0.009 |  | | | |
| 102225\_at | 8430421H08Rik | RIKEN cDNA 8430421H08 gene | chr1 | 0.012 | 0 | 0.005 |  | | | |
| 102286\_at | Araf | raf-related oncogene | chrX | 0.464 | 0 | 0.035 |  | | | |
| 102326\_at | Ncf2 | neutrophil cytosolic factor 2 | chr1 | -1.48 | 0 | 0.013 |  | | | |
| 102356\_at | Wdr23 | WD repeat domain 23 | chr14 | -1.437 | 0 | 0.036 |  | | | |
| 102790\_at | Jtb | jumping translocation breakpoint | --- | -0.421 | 0 | 0.04 |  | | | |
| 102884\_at | Inpp5d | inositol polyphosphate-5-phosphatase D | chr1 | 0.265 | 0 | 0.013 |  | | | |
| 102896\_at | Dok1 | downstream of tyrosine kinase 1 | chr6 | -0.631 | 0 | 0.035 |  | | | |
| 102933\_at | Plxna3 | plexin A3 | chrX | -0.272 | 0 | 0.006 |  | | | |
| 102955\_at | Nfil3 | nuclear factor, interleukin 3, regulated | chr13 | -2.259 | 0 | 0.012 |  | | | |
| 103037\_at | Ctf1 | cardiotrophin 1 | --- | -1.295 | 0 | 0.012 |  | | | |
| 103217\_at | Cflar | CASP8 and FADD-like apoptosis regulator | --- | -0.404 | 0 | 0.01 |  | | | |
| 103226\_at | Mrc1 | mannose receptor, C type 1 | chr2 | -2.184 | 0 | 0.003 |  | | | |
| 103348\_at | 1110018F06Rik | RIKEN cDNA 1110018F06 gene | --- | -0.369 | 0 | 0.002 |  | | | |
| 103381\_at | 1810024J13Rik | RIKEN cDNA 1810024J13 gene | chr5 | 0.119 | 0 | 0.025 |  | | | |
| 103392\_at | Adcy7 | adenylate cyclase 7 | chr8 | -0.359 | 0 | 0.003 |  | | | |
| 103427\_at | Fbxl3a | F-box and leucine-rich repeat protein 3a | chr14 | -1.769 | 0 | 0.042 |  | | | |
| 103832\_at | Tfip11 | tuftelin interacting protein 11 | chr5 | -0.287 | 0 | 0.036 |  | | | |
| 103990\_at | Fosb | FBJ osteosarcoma oncogene B | chr7 | -4.608 | 0 | 0.012 |  | | | |
| 104146\_at | 2610025P08Rik | RIKEN cDNA 2610025P08 gene | chr7 | -0.243 | 0 | 0.035 |  | | | |
| 104252\_at | AU020206 | expressed sequence AU020206 | chr7 | -1.192 | 0 | 0.034 |  | | | |
| 104362\_at | B230113M03Rik | RIKEN cDNA B230113M03 gene | chr11 | -0.083 | 0 | 0.024 |  | | | |
| 104396\_at | Sh3glb1 | SH3-domain GRB2-like B1 (endophilin) | chr6 | -0.559 | 0 | 0.014 |  | | | |
| 104398\_at | 1300010A20Rik | RIKEN cDNA 1300010A20 gene | chr6 | 0.057 | 0 | 0.037 |  | | | |
| 104516\_at | Cldn5 | claudin 5 | chr16 | -1.272 | 0 | 0.048 |  | | | |
| 104562\_at | 5730403M16Rik | RIKEN cDNA 5730403M16 gene | chr7 | -1.253 | 0 | 0.009 |  | | | |
| 104598\_at | Dusp1 | dual specificity phosphatase 1 | chr17 | -2.35 | 0 | 0.018 |  | | | |
| 104625\_at | Dnajb6 | DnaJ (Hsp40) homolog, subfamily B, member 6 | chr16 | -1.482 | 0 | 0.041 |  | | | |
| 104719\_at | Slc12a7 | solute carrier family 12, member 7 | chr13 | -1.166 | 0 | 0.039 |  | | | |
| 160104\_at | Hsd3b7 | hydroxy-delta-5-steroid dehydrogenase, 3 beta- and steroid delta-isomerase 7 | chr7 | -1.424 | 0 | 0.022 |  | | | |
| 160273\_at | NoneAvailable | Mus musculus Brf2 gene, 3' UTR | chr17 | -0.145 | 0 | 0.008 |  | | | |
| 160346\_at | 6330580J24Rik | RIKEN cDNA 6330580J24 gene | chr9 | -0.152 | 0 | 0.003 |  | | | |
| 160362\_at | Mat2a | methionine adenosyltransferase II, alpha | chr6 | -0.381 | 0 | 0.016 |  | | | |
| 160526\_s\_at | Crem | cAMP responsive element modulator | --- | -5.103 | 0 | 0.005 |  | | | |
| 160547\_s\_at | Txnip | thioredoxin interacting protein | chr3 | -0.865 | 0 | 0 |  | | | |
| 160564\_at | Lcn2 | lipocalin 2 | chr2 | -0.186 | 0 | 0.038 |  | | | |
| 160573\_at | Hccs | holocytochrome c synthetase | --- | -2.08 | 0 | 0.025 |  | | | |
| 160664\_at | 1200004M23Rik | RIKEN cDNA 1200004M23 gene | chr19 | -0.403 | 0 | 0.005 |  | | | |
| 160714\_at | Gab1 | growth factor receptor bound protein 2-associated protein 1 | chr8 | -0.506 | 0 | 0.048 |  | | | |
| 160792\_at | Snap25bp | synaptosomal-associated protein 25 binding protein | --- | 0.342 | 0 | 0.044 |  | | | |
| 160901\_at | Fos | FBJ osteosarcoma oncogene | chr12 | -3.257 | 0 | 0.024 |  | | | |
| 161211\_r\_at | Sox6 | SRY-box containing gene 6 | --- | -0.328 | 0 | 0.017 |  | | | |
| 161683\_r\_at | Gtpbp1 | GTP binding protein 1 | --- | -0.546 | 0 | 0.035 |  | | | |
| 92248\_at | Nr4a2 | nuclear receptor subfamily 4, group A, member 2 | chr2 | -5.877 | 0 | 0.007 |  | | | |
| 92256\_at | Fdft1 | farnesyl diphosphate farnesyl transferase 1 | --- | -1.15 | 0 | 0.003 |  | | | |
| 92270\_at | Tro | trophinin | chrX | -1.72 | 0 | 0.006 |  | | | |
| 92310\_at | Snk | serum-inducible kinase | chr13 | -0.859 | 0 | 0.003 |  | | | |
| 92323\_at | Mapk12 | mitogen-activated protein kinase 12 | chr15 | -0.894 | 0 | 0.026 |  | | | |
| 92558\_at | Vcam1 | vascular cell adhesion molecule 1 | chr3 | -4.435 | 0 | 0.005 |  | | | |
| 92644\_s\_at | Myb | myeloblastosis oncogene | chr10 | -0.777 | 0 | 0.029 |  | | | |
| 92672\_at | 5530400H20Rik | RIKEN cDNA 5530400H20 gene | chr2 | -0.636 | 0 | 0.005 |  | | | |
| 92830\_s\_at | NoneAvailable | --- | --- | -3.484 | 0 | 0.001 |  | | | |
| 93093\_at | Mcl1 | myeloid cell leukemia sequence 1 | chr3 | -2.974 | 0 | 0.018 |  | | | |
| 93120\_f\_at | H2-K | histocompatibility 2, K region | chr17 | -3.637 | 0 | 0.003 |  | | | |
| 93179\_at | B830009D23Rik | RIKEN cDNA B830009D23 gene | chr2 | -1.837 | 0 | 0.017 |  | | | |
| 93326\_at | Tm4sf2 | transmembrane 4 superfamily member 2 | chrX | -1.259 | 0 | 0.02 |  | | | |
| 93498\_s\_at | Aplp2 | amyloid beta (A4) precursor-like protein 2 | chr9 | -2.613 | 0 | 0.02 |  | | | |
| 93500\_at | Alas1 | aminolevulinic acid synthase 1 | chr9 | 2.837 | 0 | 0.047 |  | | | |
| 93584\_at | Igh-6 | immunoglobulin heavy chain 6 (heavy chain of IgM) | chr12 | 1.572 | 0 | 0.006 |  | | | |
| 93682\_at | Ldb2 | LIM domain binding 2 | --- | 0.32 | 0 | 0.014 |  | | | |
| 93705\_at | Chrnb1 | cholinergic receptor, nicotinic, beta polypeptide 1 (muscle) | --- | -2.651 | 0 | 0.016 |  | | | |
| 93714\_f\_at | H2-Q7 | histocompatibility 2, Q region locus 7 | chr17 | -3.128 | 0 | 0.001 |  | | | |
| 93907\_f\_at | NoneAvailable | --- | --- | -1.754 | 0 | 0.001 |  | | | |
| 93964\_s\_at | Ddx6 | DEAD (Asp-Glu-Ala-Asp) box polypeptide 6 | chr9 | -3.009 | 0 | 0.03 |  | | | |
| 94288\_at | Hist1h1c | histone 1, H1c | chr13 | -0.134 | 0 | 0.019 |  | | | |
| 94345\_at | Il6st | interleukin 6 signal transducer | chr13 | -3.717 | 0 | 0 |  | | | |
| 94386\_at | Son | Son cell proliferation protein | chr16 | -0.788 | 0 | 0.028 |  | | | |
| 94428\_at | Ilvbl | ilvB (bacterial acetolactate synthase)-like | chr10 | -1.305 | 0 | 0.004 |  | | | |
| 94834\_at | Ctsh | cathepsin H | chr9 | -1.968 | 0 | 0.045 |  | | | |
| 94929\_at | Ptpn1 | protein tyrosine phosphatase, non-receptor type 1 | chr2 | -0.604 | 0 | 0.003 |  | | | |
| 94948\_at | Trip6 | thyroid hormone receptor interactor 6 | chr5 | -1.016 | 0 | 0.02 |  | | | |
| 94990\_at | Pl6-pending | PL6 protein | chr9 | -0.163 | 0 | 0.017 |  | | | |
| 94991\_at | Synpo | synaptopodin | chr18 | -1.276 | 0 | 0.018 |  | | | |
| 95031\_at | 1110059H15Rik | RIKEN cDNA 1110059H15 gene | chr2 | -0.433 | 0 | 0.022 |  | | | |
| 95102\_at | Scotin-pending | scotin gene | chr9 | -2.721 | 0 | 0.006 |  | | | |
| 95133\_at | Asns | asparagine synthetase | chr6 | -0.283 | 0 | 0.036 |  | | | |
| 95138\_at | 1110018O08Rik | RIKEN cDNA 1110018O08 gene | chr5 | -0.739 | 0 | 0.004 |  | | | |
| 95157\_at | B830022L21Rik | RIKEN cDNA B830022L21 gene | chr8 | -0.743 | 0 | 0.019 |  | | | |
| 95449\_at | 2310075G12Rik | RIKEN cDNA 2310075G12 gene | chr11 | -1.383 | 0 | 0.016 |  | | | |
| 95520\_at | 2310061B02Rik | RIKEN cDNA 2310061B02 gene | chr1 | -2.378 | 0 | 0.04 |  | | | |
| 95537\_at | Ulk2 | Unc-51 like kinase 2 (C. elegans) | chr11 | -0.43 | 0 | 0.011 |  | | | |
| 95893\_at | Blk | B lymphoid kinase | chr14 | -0.111 | 0 | 0.005 |  | | | |
| 96049\_at | Bgn | biglycan | --- | -1.681 | 0 | 0.008 |  | | | |
| 96186\_at | Lrp10 | low-density lipoprotein receptor-related protein 10 | chr14 | -3.157 | 0 | 0.013 |  | | | |
| 96310\_at | Mbp | myelin basic protein | chr18 | -0.13 | 0 | 0.036 |  | | | |
| 96530\_at | NoneAvailable | Mus musculus transcribed sequences | --- | -1.094 | 0 | 0.002 |  | | | |
| 96640\_at | 3110001A13Rik | RIKEN cDNA 3110001A13 gene | chr2 | -0.765 | 0 | 0.02 |  | | | |
| 96732\_at | 1500001L20Rik | RIKEN cDNA 1500001L20 gene | chr4 | -0.397 | 0 | 0.011 |  | | | |
| 96848\_at | Inpp5e | inositol polyphosphate-5-phosphatase E | chr2 | -0.985 | 0 | 0.001 |  | | | |
| 96886\_at | Stab1 | stabilin 1 | chr14 | -2.451 | 0 | 0.017 |  | | | |
| 96912\_s\_at | Ctla2a | cytotoxic T lymphocyte-associated protein 2 alpha | chr13 | -3.703 | 0 | 0.031 |  | | | |
| 96940\_at | Tead2 | TEA domain family member 2 | --- | -0.493 | 0 | 0.005 |  | | | |
| 97181\_f\_at | NoneAvailable | --- | --- | -1.659 | 0 | 0.002 |  | | | |
| 97336\_at | Ctsf | cathepsin F | chr19 | -1.534 | 0 | 0.002 |  | | | |
| 97420\_at | Lrg-pending | leucine-rich alpha-2-glycoprotein | chr17 | -0.609 | 0 | 0.026 |  | | | |
| 97448\_at | NoneAvailable | Mus musculus cDNA clone MGC:65558 IMAGE:6485174, complete cds | chr11 | -1.982 | 0 | 0.019 |  | | | |
| 97798\_at | 4930504E06Rik | RIKEN cDNA 4930504E06 gene | chr3 | -1.169 | 0 | 0.001 |  | | | |
| 97817\_at | Spec1-pending | small protein effector 1 of Cdc42 | chr3 | -0.912 | 0 | 0 |  | | | |
| 97943\_at | Capn6 | calpain 6 | chrX | 0.274 | 0 | 0.005 |  | | | |
| 98007\_at | Rps6ka2 | ribosomal protein S6 kinase, polypeptide 2 | chr17 | 0.051 | 0 | 0 |  | | | |
| 98045\_s\_at | Dab2 | disabled homolog 2 (Drosophila) | chr15 | -0.645 | 0 | 0.022 |  | | | |
| 98083\_at | Copeb | core promoter element binding protein | chr13 | -2.963 | 0 | 0 |  | | | |
| 98088\_at | Cd14 | CD14 antigen | --- | -3.218 | 0 | 0.018 |  | | | |
| 98254\_f\_at | NoneAvailable | --- | --- | -1.54 | 0 | 0.002 |  | | | |
| 98369\_f\_at | NoneAvailable | --- | --- | -1.265 | 0 | 0.002 |  | | | |
| 98418\_at | Dvl1 | dishevelled, dsh homolog 1 (Drosophila) | chr4 | -0.479 | 0 | 0.017 |  | | | |
| 98431\_at | Dusp12 | dual specificity phosphatase 12 | chr1 | 0.357 | 0 | 0.015 |  | | | |
| 98451\_at | Dnajb10 | DnaJ (Hsp40) homolog, subfamily B, member 10 | chr1 | -3.902 | 0 | 0.002 |  | | | |
| 98977\_at | Tinf2 | Terf1 (TRF1)-interacting nuclear factor 2 | chr14 | -0.816 | 0 | 0.027 |  | | | |
| 99071\_at | Mpeg1 | macrophage expressed gene 1 | chr19 | 0.525 | 0 | 0.019 |  | | | |
| 99451\_at | C230093N12Rik | RIKEN cDNA C230093N12 gene | chr2 | -0.334 | 0 | 0.041 |  | | | |
| 99640\_at | Minpp1 | multiple inositol polyphosphate histidine phosphatase 1 | chr19 | -0.507 | 0 | 0.033 |  | | | |
| 99945\_at | Cd19 | CD19 antigen | chr7 | 0.104 | 0 | 0.028 |  | | | |
| 100013\_at | 2010008K16Rik | RIKEN cDNA 2010008K16 gene | chr11 | 0.08 | 1 | 0.045 |  | | | |
| 100064\_f\_at | Gja1 | gap junction membrane channel protein alpha 1 | chr10 | -0.741 | 1 | 0.001 |  | | | |
| 100508\_at | Mfng | manic fringe homolog (Drosophila) | chr15 | -0.007 | 1 | 0.001 |  | | | |
| 100516\_at | Chk | choline kinase | chr19 | -0.342 | 1 | 0.033 |  | | | |
| 100535\_at | Eif4g2 | eukaryotic translation initiation factor 4, gamma 2 | chr7 | -0.125 | 1 | 0.013 |  | | | |
| 100564\_at | Ddt | D-dopachrome tautomerase | chr10 | -0.672 | 1 | 0.022 |  | | | |
| 100578\_at | Impdh2 | inosine 5'-phosphate dehydrogenase 2 | chr8 | 0.093 | 1 | 0.007 |  | | | |
| 100587\_f\_at | 5730403B10Rik | RIKEN cDNA 5730403B10 gene | chr16 | -2.058 | 1 | 0.009 |  | | | |
| 100611\_at | Lyzs | lysozyme | chr10 | -0.306 | 1 | 0.033 |  | | | |
| 100629\_at | Gstm5 | glutathione S-transferase, mu 5 | chr3 | 0.731 | 1 | 0.019 |  | | | |
| 100880\_at | NoneAvailable | Mus musculus diabetic nephropathy-related gene 1 mRNA, partial sequence | chr3 | -0.254 | 1 | 0.018 |  | | | |
| 100998\_at | H2-Ab1 | histocompatibility 2, class II antigen A, beta 1 | chr17 | -1.116 | 1 | 0.026 |  | | | |
| 101059\_at | Ndn | necdin | chr7 | -0.839 | 1 | 0.032 |  | | | |
| 101060\_at | Grp58 | glucose regulated protein | chr2 | 0.396 | 1 | 0.013 |  | | | |
| 101398\_at | Stxbp2 | syntaxin binding protein 2 | chr8 | 0.366 | 1 | 0.005 |  | | | |
| 101495\_at | Cd81 | CD 81 antigen | chr7 | -0.022 | 1 | 0.001 |  | | | |
| 101510\_at | Psme1 | proteasome (prosome, macropain) 28 subunit, alpha | chr14 | 0.356 | 1 | 0.019 |  | | | |
| 101554\_at | Nfkbia | nuclear factor of kappa light chain gene enhancer in B-cells inhibitor, alpha | chr12 | -0.607 | 1 | 0.005 |  | | | |
| 101568\_at | NoneAvailable | Mus musculus mRNA similar to proline synthetase co-transcribed (cDNA clone MGC:59396 IMAGE:6504579), complete cds | chr8 | -1.703 | 1 | 0.024 |  | | | |
| 101583\_at | Btg2 | B-cell translocation gene 2, anti-proliferative | chr1 | -0.846 | 1 | 0.013 |  | | | |
| 101584\_at | Rsu1 | Ras suppressor protein 1 | chr2 | 0.552 | 1 | 0.022 |  | | | |
| 101593\_at | Crip2 | cysteine rich protein 2 | --- | -0.033 | 1 | 0.004 |  | | | |
| 101963\_at | Ctsl | cathepsin L | chr13 | -2.894 | 1 | 0.022 |  | | | |
| 101971\_at | 2500002L14Rik | RIKEN cDNA 2500002L14 gene | --- | -1.663 | 1 | 0.005 |  | | | |
| 101979\_at | Gadd45g | growth arrest and DNA-damage-inducible 45 gamma | chr13 | -0.823 | 1 | 0.009 |  | | | |
| 101990\_at | Ldh2 | lactate dehydrogenase 2, B chain | chr6 | -1.289 | 1 | 0.001 |  | | | |
| 102332\_at | Ulk1 | Unc-51 like kinase 1 (C. elegans) | chr5 | -2.225 | 1 | 0.009 |  | | | |
| 102401\_at | Irf1 | interferon regulatory factor 1 | chr11 | -0.554 | 1 | 0.009 |  | | | |
| 102737\_at | Edn1 | endothelin 1 | chr13 | -0.111 | 1 | 0.04 |  | | | |
| 102779\_at | Gadd45b | growth arrest and DNA-damage-inducible 45 beta | chr10 | -0.245 | 1 | 0.038 |  | | | |
| 102809\_s\_at | Lck | lymphocyte protein tyrosine kinase | chr4 | -0.359 | 1 | 0.034 |  | | | |
| 102860\_at | Serpina3g | serine (or cysteine) proteinase inhibitor, clade A, member 3G | chr12 | -4.051 | 1 | 0.013 |  | | | |
| 102906\_at | Tgtp | T-cell specific GTPase | chr11 | -5.973 | 1 | 0.001 |  | | | |
| 102936\_at | B4galt6 | UDP-Gal:betaGlcNAc beta 1,4-galactosyltransferase, polypeptide 6 | --- | -0.027 | 1 | 0.001 |  | | | |
| 102960\_at | Rga | recombination activating gene 1 gene activation | chr3 | -1.165 | 1 | 0.049 |  | | | |
| 102965\_at | NoneAvailable | Mus musculus 13 days embryo forelimb cDNA, RIKEN full-length enriched library, clone:5930414A15 product:hypothetical EF-hand containing protein, full insert sequence | chr2 | -0.732 | 1 | 0 |  | | | |
| 103257\_at | C730036B01Rik | RIKEN cDNA C730036B01 gene | chr3 | 2.122 | 1 | 0.004 |  | | | |
| 103326\_at | E130107N23Rik | RIKEN cDNA E130107N23 gene | chr9 | -0.23 | 1 | 0.017 |  | | | |
| 103353\_f\_at | Cyp4b1 | cytochrome P450, family 4, subfamily b, polypeptide 1 | chr4 | -2.127 | 1 | 0.004 |  | | | |
| 103483\_at | Ercc5 | excision repair cross-complementing rodent repair deficiency,complementation group 5 | chr1 | -0.612 | 1 | 0 |  | | | |
| 103574\_at | Ablim1 | actin-binding LIM protein 1 | chr19 | -0.139 | 1 | 0.022 |  | | | |
| 103634\_at | Isgf3g | interferon dependent positive acting transcription factor 3 gamma | --- | -1.108 | 1 | 0.002 |  | | | |
| 103717\_at | Wwp2-pending | WW domain-containing protein 2 | chr8 | -0.376 | 1 | 0.013 |  | | | |
| 103726\_at | 2610311I19Rik | RIKEN cDNA 2610311I19 gene | chr18 | 0.024 | 1 | 0.021 |  | | | |
| 103899\_at | Atp11a | ATPase, class VI, type 11A | chr8 | -1.465 | 1 | 0.049 |  | | | |
| 104000\_at | 2210023G05Rik | RIKEN cDNA 2210023G05 gene | --- | -0.048 | 1 | 0.001 |  | | | |
| 104002\_at | Zfp275 | Zinc finger protein 275 | chrX | -0.643 | 1 | 0.039 |  | | | |
| 104041\_at | 1810009A16Rik | RIKEN cDNA 1810009A16 gene | --- | -0.783 | 1 | 0.025 |  | | | |
| 104063\_at | Srcasm | Src activating and signaling molecule | chr11 | 0.431 | 1 | 0.01 |  | | | |
| 104208\_at | Pik4ca | phosphatidylinositol 4-kinase, catalytic, alpha polypeptide | chr16 | -0.134 | 1 | 0.004 |  | | | |
| 104427\_at | Mtmr13 | myotubularin related protein 13 | chr7 | -0.132 | 1 | 0.009 |  | | | |
| 104463\_at | 3830408P06Rik | RIKEN cDNA 3830408P06 gene | chr10 | 0.576 | 1 | 0.004 |  | | | |
| 104597\_at | Gbp2 | guanylate nucleotide binding protein 2 | chr3 | -2.148 | 1 | 0.021 |  | | | |
| 104735\_at | AW538430 | expressed sequence AW538430 | chr14 | -1.525 | 1 | 0.049 |  | | | |
| 160081\_at | Rpl44 | ribosomal protein L44 | chr3 | -0.147 | 1 | 0.042 |  | | | |
| 160090\_f\_at | Aldo1 | aldolase 1, A isoform | chr7 | 0.192 | 1 | 0.019 |  | | | |
| 160127\_at | Ccng1 | cyclin G1 | chr11 | -1.675 | 1 | 0.007 |  | | | |
| 160197\_at | 1110058B13Rik | RIKEN cDNA 1110058B13 gene | chr15 | 1.846 | 1 | 0.03 |  | | | |
| 160227\_s\_at | Bysl | bystin-like | chr17 | 1.211 | 1 | 0 |  | | | |
| 160343\_at | Srp19 | signal recognition particle 19 | --- | 0.308 | 1 | 0.027 |  | | | |
| 160361\_at | Trappc4 | trafficking protein particle complex 4 | chr9 | 0.38 | 1 | 0.042 |  | | | |
| 160366\_at | NoneAvailable | Mus musculus 8 days embryo whole body cDNA, RIKEN full-length enriched library, clone:5730496F02 product:HYPOTHETICAL 8.0 KDA PROTEIN homolog [Mus musculus], full insert sequence | chr18 | 0.38 | 1 | 0.029 |  | | | |
| 160428\_at | Suclg2 | succinate-Coenzyme A ligase, GDP-forming, beta subunit | chr6 | 0.005 | 1 | 0.034 |  | | | |
| 160449\_at | Dr1 | down-regulator of transcription 1 | chr5 | -0.571 | 1 | 0.002 |  | | | |
| 160462\_f\_at | Tubb3 | tubulin, beta 3 | chr8 | 0.376 | 1 | 0.037 |  | | | |
| 160476\_f\_at | Rpl18 | ribosomal protein L18 | --- | 0.052 | 1 | 0.014 |  | | | |
| 160502\_at | Creg | cellular repressor of E1A-stimulated genes | --- | -2.676 | 1 | 0.03 |  | | | |
| 160519\_at | Timp3 | tissue inhibitor of metalloproteinase 3 | chr10 | -3.836 | 1 | 0.007 |  | | | |
| 160704\_at | 1110067D22Rik | RIKEN cDNA 1110067D22 gene | chr11 | -0.873 | 1 | 0.012 |  | | | |
| 160722\_at | 4833420N02Rik | RIKEN cDNA 4833420N02 gene | chr11 | 0.416 | 1 | 0.026 |  | | | |
| 160724\_at | Usp49 | ubiquitin specific protease 49 | --- | -2.112 | 1 | 0 |  | | | |
| 160749\_at | 1500011H22Rik | RIKEN cDNA 1500011H22 gene | --- | -0.59 | 1 | 0.013 |  | | | |
| 160783\_at | D14Ertd436e | DNA segment, Chr 14, ERATO Doi 436, expressed | chr14 | -1.857 | 1 | 0.033 |  | | | |
| 160885\_at | 2700010L10Rik | RIKEN cDNA 2700010L10 gene | chr1 | 0.691 | 1 | 0.045 |  | | | |
| 160898\_at | Abt1 | activator of basal transcription | chr13 | -0.064 | 1 | 0.034 |  | | | |
| 160933\_at | Igtp | interferon gamma induced GTPase | chr11 | -3.814 | 1 | 0 |  | | | |
| 160971\_at | AI842353 | expressed sequence AI842353 | chr19 | -0.561 | 1 | 0.008 |  | | | |
| 160982\_at | 4921526G09Rik | RIKEN cDNA 4921526G09 gene | chrX | -0.898 | 1 | 0.018 |  | | | |
| 160998\_at | D4Ertd89e | DNA segment, Chr 4, ERATO Doi 89, expressed | chr4 | -0.023 | 1 | 0.009 |  | | | |
| 161250\_at | Ipo4 | importin 4 | --- | -0.275 | 1 | 0.026 |  | | | |
| 161666\_f\_at | Gadd45b | growth arrest and DNA-damage-inducible 45 beta | chr10 | -2.691 | 1 | 0.042 |  | | | |
| 162044\_f\_at | Cyp4b1 | cytochrome P450, family 4, subfamily b, polypeptide 1 | --- | -2.532 | 1 | 0.007 |  | | | |
| 162125\_f\_at | Ubc | ubiquitin C | chr5 | -0.081 | 1 | 0.035 |  | | | |
| 92198\_s\_at | Daf2 | decay accelerating factor 2 | chr1 | 0.014 | 1 | 0.006 |  | | | |
| 92262\_at | Wig1 | wild-type p53-induced gene 1 | chr3 | -0.291 | 1 | 0 |  | | | |
| 92263\_at | Grcb | gene rich cluster, B gene | chr6 | -1.939 | 1 | 0.002 |  | | | |
| 92401\_at | Ltc4s | leukotriene C4 synthase | chr11 | -0.139 | 1 | 0.011 |  | | | |
| 92440\_at | Irf6 | interferon regulatory factor 6 | chr1 | -4.326 | 1 | 0.031 |  | | | |
| 92653\_at | D530037H12Rik | RIKEN cDNA D530037H12 gene | chr1 | -1.338 | 1 | 0.01 |  | | | |
| 92707\_at | Pvt1 | plasmacytoma variant translocation 1 | --- | 0.022 | 1 | 0.003 |  | | | |
| 92780\_f\_at | NoneAvailable | --- | --- | -2.077 | 1 | 0.017 |  | | | |
| 92847\_s\_at | M6pr | mannose-6-phosphate receptor, cation dependent | chr6 | -1.096 | 1 | 0.043 |  | | | |
| 92866\_at | H2-Aa | histocompatibility 2, class II antigen A, alpha | chr17 | -3.869 | 1 | 0.036 |  | | | |
| 92872\_at | 1200016B17Rik | RIKEN cDNA 1200016B17 gene | chr6 | -0.497 | 1 | 0.031 |  | | | |
| 92926\_at | Mpl | myeloproliferative leukemia virus oncogene | --- | -1.119 | 1 | 0.003 |  | | | |
| 93011\_at | Gabarapl1 | gamma-aminobutyric acid (GABA(A)) receptor-associated protein-like 1 | chr6 | -2.046 | 1 | 0.019 |  | | | |
| 93020\_at | Rex3 | reduced expression 3 | chrX | -1.019 | 1 | 0.009 |  | | | |
| 93021\_at | Drpla | dentatorubral pallidoluysian atrophy | chr11 | 0.351 | 1 | 0.024 |  | | | |
| 93039\_at | 1190003P12Rik | RIKEN cDNA 1190003P12 gene | chr15 | -1.465 | 1 | 0.029 |  | | | |
| 93252\_at | Bcap31 | B-cell receptor-associated protein 31 | chrX | 0.065 | 1 | 0.001 |  | | | |
| 93278\_at | Scp2 | sterol carrier protein 2, liver | chr4 | 0.017 | 1 | 0.024 |  | | | |
| 93294\_at | Ctgf | connective tissue growth factor | chr10 | -0.222 | 1 | 0.045 |  | | | |
| 93318\_at | Ninj1 | ninjurin 1 | chr13 | -0.93 | 1 | 0.031 |  | | | |
| 93324\_at | Zfp36l1 | zinc finger protein 36, C3H type-like 1 | chr12 | -2.308 | 1 | 0.025 |  | | | |
| 93347\_at | Rab24 | RAB24, member RAS oncogene family | chr13 | -0.043 | 1 | 0.019 |  | | | |
| 93536\_at | Bax | Bcl2-associated X protein | chr7 | 0.954 | 1 | 0.007 |  | | | |
| 93543\_f\_at | Gstm1 | glutathione S-transferase, mu 1 | chr5 | -1.363 | 1 | 0.008 |  | | | |
| 93751\_at | 8430421I07Rik | RIKEN cDNA 8430421I07 gene | chr9 | -0.171 | 1 | 0.026 |  | | | |
| 93776\_at | 1500001L15Rik | RIKEN cDNA 1500001L15 gene | chr14 | 0.001 | 1 | 0.01 |  | | | |
| 93782\_at | Rnf4 | ring finger protein 4 | chr5 | -0.534 | 1 | 0.013 |  | | | |
| 93789\_s\_at | Sin3b | transcriptional regulator, SIN3B (yeast) | chr8 | -0.358 | 1 | 0.015 |  | | | |
| 93835\_at | Fuca | fucosidase, alpha-L- 1, tissue | chr4 | -0.701 | 1 | 0.021 |  | | | |
| 93971\_f\_at | Psmd12 | proteasome (prosome, macropain) 26S subunit, non-ATPase, 12 | chr11 | -0.122 | 1 | 0.019 |  | | | |
| 94000\_at | Cd8b | CD8 antigen, beta chain | chr5 | 0.23 | 1 | 0.024 |  | | | |
| 94021\_at | Trim11 | tripartite motif protein 11 | chr11 | -0.105 | 1 | 0.031 |  | | | |
| 94068\_at | Rps19 | ribosomal protein S19 | --- | 0.057 | 1 | 0 |  | | | |
| 94073\_at | Polr2g | polymerase (RNA) II (DNA directed) polypeptide G | chr19 | 1.881 | 1 | 0.001 |  | | | |
| 94246\_at | Ets2 | E26 avian leukemia oncogene 2, 3' domain | --- | 0.191 | 1 | 0.038 |  | | | |
| 94269\_at | Rabac1 | Rab acceptor 1 (prenylated) | chr7 | -1.557 | 1 | 0.015 |  | | | |
| 94270\_at | Krt1-18 | keratin complex 1, acidic, gene 18 | --- | -3.531 | 1 | 0.013 |  | | | |
| 94274\_at | 6720465F12Rik | RIKEN cDNA 6720465F12 gene | --- | 1.041 | 1 | 0.001 |  | | | |
| 94285\_at | H2-Eb1 | histocompatibility 2, class II antigen E beta | chr17 | -0.946 | 1 | 0 |  | | | |
| 94502\_at | D13Wsu50e | DNA segment, Chr 13, Wayne State University 50, expressed | chr13 | -0.107 | 1 | 0.023 |  | | | |
| 94522\_at | Dctn3 | dynactin 3 | chr4 | 0.552 | 1 | 0.037 |  | | | |
| 94715\_at | Cyp1a1 | cytochrome P450, family 1, subfamily a, polypeptide 1 | --- | -0.097 | 1 | 0.005 |  | | | |
| 94799\_at | F8 | coagulation factor VIII | chrX | -0.375 | 1 | 0.028 |  | | | |
| 94821\_at | Xbp1 | X-box binding protein 1 | --- | -2.107 | 1 | 0.021 |  | | | |
| 94823\_at | NoneAvailable | Mus musculus, clone IMAGE:3586350, mRNA, partial cds | chr10 | -0.477 | 1 | 0.011 |  | | | |
| 94835\_f\_at | Tubb2 | tubulin, beta 2 | chr13 | -2.52 | 1 | 0.002 |  | | | |
| 94881\_at | Cdkn1a | cyclin-dependent kinase inhibitor 1A (P21) | chr17 | -2.727 | 1 | 0.002 |  | | | |
| 94941\_at | Eif2ak4 | eukaryotic translation initiation factor 2 alpha kinase 4 | chr2 | 0.14 | 1 | 0.002 |  | | | |
| 95478\_at | Deb1 | differentially expressed in B16F10 1 | chr9 | 0.513 | 1 | 0.012 |  | | | |
| 95496\_at | 5730409F23Rik | RIKEN cDNA 5730409F23 gene | chr11 | 0.428 | 1 | 0.035 |  | | | |
| 95505\_at | Tor1b | torsin family 1, member B | --- | -1.061 | 1 | 0.039 |  | | | |
| 95508\_at | Nckap1 | NCK-associated protein 1 | chr2 | -4.402 | 1 | 0.021 |  | | | |
| 95547\_at | Arhd | ras homolog gene family, member D | chr19 | -0.192 | 1 | 0.01 |  | | | |
| 95666\_at | 9430009J09Rik | RIKEN cDNA 9430009J09 gene | chr1 | -0.229 | 1 | 0.014 |  | | | |
| 95715\_at | 1200009C21Rik | RIKEN cDNA 1200009C21 gene | chr7 | 0.656 | 1 | 0.043 |  | | | |
| 95737\_at | 1200015A19Rik | RIKEN cDNA 1200015A19 gene | chr4 | -2.325 | 1 | 0.023 |  | | | |
| 95913\_at | C230056F04Rik | RIKEN cDNA C230056F04 gene | chrX | -0.335 | 1 | 0.042 |  | | | |
| 96058\_s\_at | Aldh2 | aldehyde dehydrogenase 2, mitochondrial | chr5 | -0.942 | 1 | 0.037 |  | | | |
| 96146\_at | Btg3 | B-cell translocation gene 3 | chr16 | -2.704 | 1 | 0.035 |  | | | |
| 96158\_at | BC017133 | cDNA sequence BC017133 | --- | -0.57 | 1 | 0.006 |  | | | |
| 96592\_at | Pik3r1 | phosphatidylinositol 3-kinase, regulatory subunit, polypeptide 1 (p85 alpha) | chr13 | 0.081 | 1 | 0.03 |  | | | |
| 96596\_at | Ndrl | N-myc downstream regulated-like | chr15 | -4.908 | 1 | 0.007 |  | | | |
| 96614\_at | 4933426M11Rik | RIKEN cDNA 4933426M11 gene | chr12 | -1.2 | 1 | 0.039 |  | | | |
| 96703\_at | Maged1 | melanoma antigen, family D, 1 | chrX | -2.002 | 1 | 0.003 |  | | | |
| 96728\_at | DXImx38e | DNA segment, Chr X, Immunex 38, expressed | chrX | -2.856 | 1 | 0.001 |  | | | |
| 96752\_at | Icam1 | intercellular adhesion molecule | chr9 | -2.045 | 1 | 0.024 |  | | | |
| 96764\_at | Iigp-pending | interferon-inducible GTPase | --- | -5.077 | 1 | 0.009 |  | | | |
| 96785\_at | 0610013D04Rik | RIKEN cDNA 0610013D04 gene | chr17 | -0.743 | 1 | 0.003 |  | | | |
| 96801\_at | Ak1 | adenylate kinase 1 | chr2 | -0.22 | 1 | 0.004 |  | | | |
| 96831\_at | Pdir-pending | protein disulfide isomerase-related | --- | 0.318 | 1 | 0 |  | | | |
| 96876\_at | Laptm4a | lysosomal-associated protein transmembrane 4A | chr12 | -1.037 | 1 | 0.039 |  | | | |
| 96884\_at | Carhsp1 | calcium regulated heat stable protein 1 | --- | -0.717 | 1 | 0 |  | | | |
| 96890\_at | 1300002A08Rik | RIKEN cDNA 1300002A08 gene | chr8 | -0.314 | 1 | 0.048 |  | | | |
| 96935\_at | 2700030M23Rik | RIKEN cDNA 2700030M23 gene | chr4 | -2.968 | 1 | 0.003 |  | | | |
| 96936\_at | Copg1 | coatomer protein complex, subunit gamma 1 | chr6 | -0.064 | 1 | 0.042 |  | | | |
| 97060\_at | Ywhaq | tyrosine 3-monooxygenase/tryptophan 5-monooxygenase activation protein, theta polypeptide | --- | 0.172 | 1 | 0.043 |  | | | |
| 97107\_at | 1700007D05Rik | RIKEN cDNA 1700007D05 gene | chr10 | -0.34 | 1 | 0.021 |  | | | |
| 97148\_at | NoneAvailable | Mus musculus transcribed sequences | --- | 0.197 | 1 | 0.022 |  | | | |
| 97320\_at | 1600025H15Rik | RIKEN cDNA 1600025H15 gene | chr15 | -0.215 | 1 | 0.002 |  | | | |
| 97352\_f\_at | Coxvib2 | cytochrome c oxidase subunit VIb, testes-specific | chr7 | -0.255 | 1 | 0.002 |  | | | |
| 97401\_at | 1300006C06Rik | RIKEN cDNA 1300006C06 gene | chr15 | -0.573 | 1 | 0.001 |  | | | |
| 97409\_at | Ifi1 | interferon inducible protein 1 | chr11 | -1.031 | 1 | 0.034 |  | | | |
| 97487\_at | Serpine2 | serine (or cysteine) proteinase inhibitor, clade E, member 2 | chr1 | -0.891 | 1 | 0.011 |  | | | |
| 97515\_at | Hsd17b4 | hydroxysteroid (17-beta) dehydrogenase 4 | chr18 | 0.524 | 1 | 0.006 |  | | | |
| 97540\_f\_at | H2-D1 | histocompatibility 2, D region locus 1 | --- | -3.787 | 1 | 0.002 |  | | | |
| 97549\_at | Cfl2 | cofilin 2, muscle | chr12 | -2.749 | 1 | 0.03 |  | | | |
| 97718\_at | Ctla4 | cytotoxic T-lymphocyte-associated protein 4 | chr1 | -0.129 | 1 | 0.043 |  | | | |
| 97880\_at | Dlst | dihydrolipoamide S-succinyltransferase (E2 component of 2-oxo-glutarate complex) | chr12 | -0.165 | 1 | 0.028 |  | | | |
| 97890\_at | Sgk | serum/glucocorticoid regulated kinase | chr10 | -3.498 | 1 | 0.048 |  | | | |
| 97908\_at | 1110007A06Rik | RIKEN cDNA 1110007A06 gene | chr6 | -1.119 | 1 | 0.04 |  | | | |
| 97973\_at | Tal1 | T-cell acute lymphocytic leukemia 1 | chr4 | -1.383 | 1 | 0.032 |  | | | |
| 97984\_i\_at | NoneAvailable | Mus musculus 0 day neonate thymus cDNA, RIKEN full-length enriched library, clone:A430045K06 product:hypothetical Type I antifreeze protein containing protein, full insert sequence | --- | 0.534 | 1 | 0.023 |  | | | |
| 98053\_at | Ywhab | tyrosine 3-monooxygenase/tryptophan 5-monooxygenase activation protein, beta polypeptide | --- | 0.019 | 1 | 0.049 |  | | | |
| 98056\_at | Phlda3 | pleckstrin homology-like domain, family A, member 3 | chr1 | 0.009 | 1 | 0.021 |  | | | |
| 98067\_at | Cdkn1a | cyclin-dependent kinase inhibitor 1A (P21) | chr17 | -2.741 | 1 | 0.001 |  | | | |
| 98410\_at | Gtpi-pending | interferon-g induced GTPase | chr11 | -2.836 | 1 | 0.046 |  | | | |
| 98472\_at | H2-T23 | histocompatibility 2, T region locus 23 | --- | -1.61 | 1 | 0 |  | | | |
| 98569\_at | 1110030N17Rik | RIKEN cDNA 1110030N17 gene | chr2 | -0.46 | 1 | 0.006 |  | | | |
| 98574\_at | Prpf8 | pre-mRNA processing factor 8 | chr11 | -0.114 | 1 | 0.024 |  | | | |
| 98600\_at | S100a11 | S100 calcium binding protein A11 (calizzarin) | chr4 | -0.349 | 1 | 0.024 |  | | | |
| 98633\_at | 1200015A22Rik | RIKEN cDNA 1200015A22 gene | chr6 | -0.347 | 1 | 0.007 |  | | | |
| 98887\_at | Napa | N-ethylmaleimide sensitive fusion protein attachment protein alpha | --- | -0.084 | 1 | 0.048 |  | | | |
| 99109\_at | Ier2 | immediate early response 2 | chr8 | -2.892 | 1 | 0.003 |  | | | |
| 99133\_at | Slc3a2 | solute carrier family 3 (activators of dibasic and neutral amino acid transport), member 2 | chr19 | -1.113 | 1 | 0.018 |  | | | |
| 99154\_s\_at | 1810020D17Rik | RIKEN cDNA 1810020D17 gene | chr7 | 0.132 | 1 | 0.003 |  | | | |
| 99340\_at | Gnb2-rs1 | guanine nucleotide binding protein, beta 2, related sequence 1 | chr11 | -0.042 | 1 | 0.039 |  | | | |
| 99366\_at | E030024M05Rik | RIKEN cDNA E030024M05 gene | chr12 | -2.996 | 1 | 0.014 |  | | | |
| 99532\_at | Tob1 | transducer of ErbB-2.1 | chr11 | -5.048 | 1 | 0.008 |  | | | |
| 99577\_at | Kitl | kit ligand | chr10 | -0.059 | 1 | 0.022 |  | | | |
| 99629\_at | Ei24 | etoposide induced 2.4 mRNA | chr9 | -0.596 | 1 | 0 |  | | | |
| 99909\_at | Trpc6 | transient receptor potential cation channel, subfamily C, member 6 | chr9 | -0.627 | 1 | 0.031 |  | | | |
| 100030\_at | Upp1 | uridine phosphorylase 1 | chr11 | -1.097 | 10 | 0.005 |  | | | |
| 100094\_at | Supt5h | suppressor of Ty 5 homolog (S. cerevisiae) | chr7 | -0.649 | 10 | 0.037 |  | | | |
| 100134\_at | Eng | endoglin | chr2 | -1.282 | 10 | 0.012 |  | | | |
| 100136\_at | Lamp2 | lysosomal membrane glycoprotein 2 | chrX | -1.749 | 10 | 0.025 |  | | | |
| 100293\_at | Cd59b | CD59b antigen | --- | -0.374 | 10 | 0.011 |  | | | |
| 100325\_at | Gp49a | glycoprotein 49 A | chr10 | -0.358 | 10 | 0.001 |  | | | |
| 100333\_at | Saa2 | serum amyloid A 2 | chr7 | -0.09 | 10 | 0.007 |  | | | |
| 100348\_at | NoneAvailable | --- | --- | 2.608 | 10 | 0.029 |  | | | |
| 100397\_at | Tyrobp | TYRO protein tyrosine kinase binding protein | chr7 | 3.755 | 10 | 0.004 |  | | | |
| 100429\_at | Ppox | protoporphyrinogen oxidase | chr1 | -0.282 | 10 | 0 |  | | | |
| 100475\_at | Trim25 | tripartite motif protein 25 | --- | -1.645 | 10 | 0 |  | | | |
| 100486\_at | Ezh1 | enhancer of zeste homolog 1 (Drosophila) | chr11 | -0.745 | 10 | 0.003 |  | | | |
| 100492\_at | Ap2a2 | adaptor protein complex AP-2, alpha 2 subunit | chr7 | 0.607 | 10 | 0.035 |  | | | |
| 100606\_at | Prnp | prion protein | --- | -1.719 | 10 | 0.036 |  | | | |
| 100616\_at | Cenpa | centromere autoantigen A | --- | 0.979 | 10 | 0.013 |  | | | |
| 100635\_at | Sara | SAR1a gene homolog (S. cerevisiae) | chr10 | -1.295 | 10 | 0.009 |  | | | |
| 100710\_at | Vcp | valosin containing protein | chr4 | -0.461 | 10 | 0.033 |  | | | |
| 100877\_at | 1810058I24Rik | RIKEN cDNA 1810058I24 gene | chr6 | 0.025 | 10 | 0.005 |  | | | |
| 100921\_at | Tnni3 | troponin I, cardiac | chr7 | -0.848 | 10 | 0.002 |  | | | |
| 100939\_at | Zfp282 | zinc finger protein 282 | chr6 | -0.161 | 10 | 0.006 |  | | | |
| 100973\_i\_at | Ccl27 | chemokine (C-C motif) ligand 27 | --- | -3.092 | 10 | 0 |  | | | |
| 100988\_at | Bcl2l11 | BCL2-like 11 (apoptosis facilitator) | chr2 | -1.027 | 10 | 0.014 |  | | | |
| 101014\_at | Ifnar2 | interferon (alpha and beta) receptor 2 | chr16 | 0.225 | 10 | 0.032 |  | | | |
| 101036\_at | 1810060K07Rik | RIKEN cDNA 1810060K07 gene | --- | -0.149 | 10 | 0.019 |  | | | |
| 101073\_at | Lrp1 | low density lipoprotein receptor-related protein 1 | chr10 | -0.016 | 10 | 0.002 |  | | | |
| 101079\_at | Nxf1 | nuclear RNA export factor 1 homolog (S. cerevisiae) | chr19 | -2.424 | 10 | 0.003 |  | | | |
| 101144\_at | Il18r1 | interleukin 18 receptor 1 | --- | -1.522 | 10 | 0.037 |  | | | |
| 101186\_at | Ppnr-pending | per-pentamer repeat gene | chr19 | -1.941 | 10 | 0.019 |  | | | |
| 101432\_at | 2410019G02Rik | RIKEN cDNA 2410019G02 gene | chr11 | -0.055 | 10 | 0.022 |  | | | |
| 101441\_i\_at | Itpr5 | inositol 1,4,5-triphosphate receptor 5 | chr6 | -3.278 | 10 | 0.019 |  | | | |
| 101446\_at | Tpd52l1 | tumor protein D52-like 1 | chr10 | -0.068 | 10 | 0.04 |  | | | |
| 101449\_at | Trim41 | tripartite motif-containing 41 | chr11 | -0.832 | 10 | 0.009 |  | | | |
| 101697\_f\_at | NoneAvailable | --- | --- | -0.037 | 10 | 0.027 |  | | | |
| 101778\_at | Gja5 | gap junction membrane channel protein alpha 5 | chr3 | -0.024 | 10 | 0.013 |  | | | |
| 101835\_at | Lrmp | lymphoid-restricted membrane protein | chr6 | 0.24 | 10 | 0.027 |  | | | |
| 101836\_at | Ppm1b | protein phosphatase 1B, magnesium dependent, beta isoform | chr17 | -1.457 | 10 | 0.007 |  | | | |
| 101844\_at | Pipox | pipecolic acid oxidase | chr11 | -0.024 | 10 | 0.008 |  | | | |
| 101884\_at | Xlr4 | X-linked lymphocyte-regulated 4 | chrX | -2.072 | 10 | 0.038 |  | | | |
| 101889\_s\_at | Rora | RAR-related orphan receptor alpha | chr9 | -0.911 | 10 | 0.004 |  | | | |
| 101943\_at | Tceb3 | transcription elongation factor B (SIII), polypeptide 3 | chr4 | -1.496 | 10 | 0.002 |  | | | |
| 101947\_at | Nakap95-pending | neighbor of A-kinase anchoring protein 95 | chr17 | -1.799 | 10 | 0.001 |  | | | |
| 102024\_at | Ncoa3 | nuclear receptor coactivator 3 | chr2 | -0.905 | 10 | 0.014 |  | | | |
| 102063\_at | Pdpk1 | 3-phosphoinositide dependent protein kinase-1 | chr17 | -0.713 | 10 | 0.01 |  | | | |
| 102091\_f\_at | NoneAvailable | --- | --- | -0.219 | 10 | 0.003 |  | | | |
| 102101\_f\_at | NoneAvailable | --- | --- | -0.105 | 10 | 0.004 |  | | | |
| 102104\_f\_at | NoneAvailable | --- | chr19 | 1.072 | 10 | 0.038 |  | | | |
| 102242\_at | Per3 | period homolog 3 (Drosophila) | chr4 | -0.182 | 10 | 0.046 |  | | | |
| 102260\_at | Gfi1b | growth factor independent 1B | chr2 | 0.788 | 10 | 0.001 |  | | | |
| 102279\_at | 1300004C08Rik | RIKEN cDNA 1300004C08 gene | chr9 | -2.168 | 10 | 0.008 |  | | | |
| 102313\_at | Gch | GTP cyclohydrolase 1 | chr14 | -4.039 | 10 | 0.005 |  | | | |
| 102382\_at | Arntl | aryl hydrocarbon receptor nuclear translocator-like | chr7 | -0.06 | 10 | 0.004 |  | | | |
| 102580\_r\_at | NoneAvailable | --- | chr6 | 0.113 | 10 | 0.044 |  | | | |
| 102658\_at | Il1r2 | interleukin 1 receptor, type II | chr1 | -1.46 | 10 | 0 |  | | | |
| 102663\_at | Plaur | urokinase plasminogen activator receptor | --- | -2.014 | 10 | 0.036 |  | | | |
| 102781\_at | Ccnl2 | cyclin L2 | chr4 | -1.306 | 10 | 0.005 |  | | | |
| 102787\_at | Gpr56 | G protein-coupled receptor 56 | chr8 | -1.614 | 10 | 0.021 |  | | | |
| 102836\_at | Pps | putative phosphatase | chr11 | -1.69 | 10 | 0 |  | | | |
| 102895\_at | 4921518A06Rik | RIKEN cDNA 4921518A06 gene | chr15 | -0.554 | 10 | 0.029 |  | | | |
| 102980\_at | Nmt1 | N-myristoyltransferase 1 | chr11 | -0.153 | 10 | 0.045 |  | | | |
| 102991\_s\_at | H2-Ke6 | H2-K region expressed gene 6 | chr17 | -0.88 | 10 | 0.009 |  | | | |
| 103015\_at | Bcl6 | B-cell leukemia/lymphoma 6 | chr16 | -4.658 | 10 | 0.02 |  | | | |
| 103033\_at | C4 | complement component 4 (within H-2S) | chr17 | -0.069 | 10 | 0.04 |  | | | |
| 103051\_at | Expi | extracellular proteinase inhibitor | chr11 | -0.064 | 10 | 0 |  | | | |
| 103080\_at | Samhd1 | SAM domain and HD domain, 1 | chr2 | 0.08 | 10 | 0.003 |  | | | |
| 103210\_at | Csf2rb2 | colony stimulating factor 2 receptor, beta 2, low-affinity (granulocyte-macrophage) | chr15 | -1.2 | 10 | 0.02 |  | | | |
| 103218\_at | Slc10a3 | solute carrier family 10 (sodium/bile acid cotransporter family), member 3 | chrX | -0.604 | 10 | 0.045 |  | | | |
| 103251\_at | 2310010M10Rik | RIKEN cDNA 2310010M10 gene | chr14 | -0.107 | 10 | 0.004 |  | | | |
| 103254\_at | Fln29-pending | FLN29 gene product | chr5 | -2.179 | 10 | 0.011 |  | | | |
| 103259\_at | Gfi1 | growth factor independent 1 | --- | 1.14 | 10 | 0.031 |  | | | |
| 103314\_at | D13Ertd275e | DNA segment, Chr 13, ERATO Doi 275, expressed | chr13 | 0.006 | 10 | 0.002 |  | | | |
| 103366\_at | AU043488 | expressed sequence AU043488 | chr10 | 0.069 | 10 | 0.022 |  | | | |
| 103408\_at | AI325941 | expressed sequence AI325941 | chr7 | 0.021 | 10 | 0.004 |  | | | |
| 103414\_at | Skiv2l | superkiller viralicidic activity 2-like (S. cerevisiae ) | chr17 | 0.059 | 10 | 0.038 |  | | | |
| 103422\_at | Cd1d1 | CD1d1 antigen | chr3 | -2.191 | 10 | 0.001 |  | | | |
| 103443\_at | Aim1 | absent in melanoma 1 | chr10 | -1.49 | 10 | 0.005 |  | | | |
| 103504\_at | Ssbp2 | single-stranded DNA binding protein 2 | chr13 | 0.016 | 10 | 0.003 |  | | | |
| 103518\_at | Ctla2b | cytotoxic T lymphocyte-associated protein 2 beta | chr13 | -5.219 | 10 | 0.015 |  | | | |
| 103545\_at | 2610019E17Rik | RIKEN cDNA 2610019E17 gene | chr17 | -0.409 | 10 | 0.027 |  | | | |
| 103562\_f\_at | NoneAvailable | --- | --- | -0.059 | 10 | 0.018 |  | | | |
| 103582\_r\_at | 6130401J04Rik | RIKEN cDNA 6130401J04 gene | chr1 | -0.175 | 10 | 0.038 |  | | | |
| 103596\_at | Dgka | diacylglycerol kinase, alpha | chr10 | -3.305 | 10 | 0 |  | | | |
| 103656\_at | Lancl1 | LanC (bacterial lantibiotic synthetase component C)-like | chr1 | -0.561 | 10 | 0.005 |  | | | |
| 103672\_at | 2410141M05Rik | RIKEN cDNA 2410141M05 gene | chr11 | -1.198 | 10 | 0.001 |  | | | |
| 103690\_at | Wbscr5 | Williams-Beuren syndrome chromosome region 5 homolog (human) | --- | 0.578 | 10 | 0.028 |  | | | |
| 103704\_at | 2010305K11Rik | RIKEN cDNA 2010305K11 gene | chr8 | -0.023 | 10 | 0.043 |  | | | |
| 103773\_at | 1110020K19Rik | RIKEN cDNA 1110020K19 gene | chr17 | -0.139 | 10 | 0.04 |  | | | |
| 103812\_at | Clca1 | chloride channel calcium activated 1 | chr3 | -4.597 | 10 | 0.005 |  | | | |
| 103845\_at | Slc31a1 | solute carrier family 31, member 1 | chr4 | -0.413 | 10 | 0.033 |  | | | |
| 103890\_at | AW538196 | expressed sequence AW538196 | chr7 | -0.467 | 10 | 0.031 |  | | | |
| 103893\_at | 6030410I24Rik | RIKEN cDNA 6030410I24 gene | chr18 | 0.036 | 10 | 0.003 |  | | | |
| 103895\_at | AW549877 | expressed sequence AW549877 | chr15 | -1.037 | 10 | 0.03 |  | | | |
| 103988\_at | A830054M12 | hypothetical protein A830054M12 | chr19 | -0.467 | 10 | 0.004 |  | | | |
| 104048\_at | Cars | cysteinyl-tRNA synthetase | chr7 | -0.836 | 10 | 0.017 |  | | | |
| 104060\_at | 2700088M22Rik | RIKEN cDNA 2700088M22 gene | chr15 | 0.135 | 10 | 0.036 |  | | | |
| 104144\_at | Gtpbp2 | GTP binding protein 2 | chr17 | -1.316 | 10 | 0.016 |  | | | |
| 104150\_at | 2810008P14Rik | RIKEN cDNA 2810008P14 gene | chr5 | 0.012 | 10 | 0.018 |  | | | |
| 104165\_at | Vnn1 | vanin 1 | --- | -2.98 | 10 | 0.003 |  | | | |
| 104184\_at | Nppb | natriuretic peptide precursor type B | --- | -0.088 | 10 | 0.032 |  | | | |
| 104206\_at | 0610012A05Rik | RIKEN cDNA 0610012A05 gene | chr15 | -7.151 | 10 | 0.003 |  | | | |
| 104217\_at | 1110015E22Rik | RIKEN cDNA 1110015E22 gene | chr7 | -0.233 | 10 | 0.035 |  | | | |
| 104219\_f\_at | Pcbp2 | poly(rC) binding protein 2 | --- | -0.24 | 10 | 0.037 |  | | | |
| 104256\_at | Pscdbp | pleckstrin homology, Sec7 and coiled-coil domains, binding protein | chr2 | -2.279 | 10 | 0.022 |  | | | |
| 104257\_g\_at | Pscdbp | pleckstrin homology, Sec7 and coiled-coil domains, binding protein | chr2 | -2.012 | 10 | 0.004 |  | | | |
| 104263\_at | 9330177P20Rik | RIKEN cDNA 9330177P20 gene | chr4 | -1.251 | 10 | 0.004 |  | | | |
| 104285\_at | Hmgcr | 3-hydroxy-3-methylglutaryl-Coenzyme A reductase | chr13 | -0.78 | 10 | 0.045 |  | | | |
| 104311\_at | 1300013G12Rik | RIKEN cDNA 1300013G12 gene | chr1 | -1.417 | 10 | 0.024 |  | | | |
| 104324\_at | Masp2 | mannan-binding lectin serine protease 2 | chr4 | -0.813 | 10 | 0.01 |  | | | |
| 104340\_at | Mbd1 | methyl-CpG binding domain protein 1 | chr18 | -0.587 | 10 | 0.036 |  | | | |
| 104364\_at | Mapkapk5 | MAP kinase-activated protein kinase 5 | chr5 | -0.358 | 10 | 0.029 |  | | | |
| 104371\_at | Dgat1 | diacylglycerol O-acyltransferase 1 | chr15 | -3.266 | 10 | 0 |  | | | |
| 104388\_at | Ccl9 | chemokine (C-C motif) ligand 9 | chr11 | 1.346 | 10 | 0.03 |  | | | |
| 104425\_at | Cipp | channel-interacting PDZ domain protein | chr4 | -0.357 | 10 | 0.011 |  | | | |
| 104453\_at | NoneAvailable | Mus musculus cDNA clone IMAGE:6433799, partial cds | chr11 | -1.124 | 10 | 0.03 |  | | | |
| 104471\_at | Hdac6 | histone deacetylase 6 | chrX | -0.009 | 10 | 0.007 |  | | | |
| 104533\_at | Pim1 | proviral integration site 1 | chr17 | -0.582 | 10 | 0.038 |  | | | |
| 104572\_at | Etohd2 | ethanol decreased 2 | chr13 | -1.512 | 10 | 0.001 |  | | | |
| 104574\_at | 5730453I16Rik | RIKEN cDNA 5730453I16 gene | chr19 | -0.933 | 10 | 0.013 |  | | | |
| 104677\_at | LOC227619 | hypothetical protein LOC227619 | chr2 | -1.818 | 10 | 0.046 |  | | | |
| 104683\_at | AA407558 | expressed sequence AA407558 | --- | 0.247 | 10 | 0.021 |  | | | |
| 104692\_at | Selp | selectin, platelet | chr1 | -0.542 | 10 | 0.006 |  | | | |
| 104701\_at | Bhlhb2 | basic helix-loop-helix domain containing, class B2 | chr6 | -3.085 | 10 | 0.023 |  | | | |
| 104714\_at | AA959601 | expressed sequence AA959601 | chr14 | -0.945 | 10 | 0 |  | | | |
| 104741\_at | 9530098M12Rik | RIKEN cDNA 9530098M12 gene | chrX | -1.016 | 10 | 0.015 |  | | | |
| 104742\_at | Mgst2 | microsomal glutathione S-transferase 2 | chr3 | -0.5 | 10 | 0.033 |  | | | |
| 104745\_at | Arl6ip2 | ADP-ribosylation factor-like 6 interacting protein 2 | chr17 | -1.189 | 10 | 0.045 |  | | | |
| 160088\_at | Fmo5 | flavin containing monooxygenase 5 | chr3 | -1.409 | 10 | 0.019 |  | | | |
| 160099\_at | Lgals4 | lectin, galactose binding, soluble 4 | chr7 | -1.876 | 10 | 0.001 |  | | | |
| 160140\_at | Tbce | tubulin-specific chaperone e | chr13 | -0.152 | 10 | 0.007 |  | | | |
| 160151\_i\_at | 1200009B18Rik | RIKEN cDNA 1200009B18 gene | chr6 | -1.735 | 10 | 0.029 |  | | | |
| 160174\_at | 0610041O14Rik | RIKEN cDNA 0610041O14 gene | chr8 | -0.442 | 10 | 0.01 |  | | | |
| 160182\_at | Sfrs6 | splicing factor, arginine/serine-rich 6 | chr2 | -0.282 | 10 | 0.005 |  | | | |
| 160220\_at | Zfp110 | zinc finger protein 110 | --- | 0.113 | 10 | 0.013 |  | | | |
| 160264\_s\_at | 1500036F01Rik | RIKEN cDNA 1500036F01 gene | chr1 | -1.44 | 10 | 0.029 |  | | | |
| 160283\_at | 2410005K20Rik | RIKEN cDNA 2410005K20 gene | --- | 0.077 | 10 | 0.048 |  | | | |
| 160287\_at | Map1lc3 | microtubule-associated protein 1 light chain 3 | chr14 | -1.669 | 10 | 0.02 |  | | | |
| 160316\_at | NoneAvailable | Mus musculus 0 day neonate thymus cDNA, RIKEN full-length enriched library, clone:A430083K13 product:unknown EST, full insert sequence | --- | -0.182 | 10 | 0.003 |  | | | |
| 160393\_at | 4930555L11Rik | RIKEN cDNA 4930555L11 gene | chr6 | -2.283 | 10 | 0.001 |  | | | |
| 160394\_at | D930014A20Rik | RIKEN cDNA D930014A20 gene | chr12 | 0.071 | 10 | 0.039 |  | | | |
| 160396\_at | 0610013I17Rik | RIKEN cDNA 0610013I17 gene | --- | -0.866 | 10 | 0.015 |  | | | |
| 160425\_at | 2410017I18Rik | RIKEN cDNA 2410017I18 gene | chr13 | -0.393 | 10 | 0.043 |  | | | |
| 160461\_f\_at | 2310057H16Rik | RIKEN cDNA 2310057H16 gene | chr18 | -0.341 | 10 | 0.01 |  | | | |
| 160495\_at | Ahr | aryl-hydrocarbon receptor | chr12 | -3.189 | 10 | 0.004 |  | | | |
| 160512\_at | BC017643 | cDNA sequence BC017643 | chr11 | -0.317 | 10 | 0.028 |  | | | |
| 160592\_at | Tmc6 | transmembrane channel-like gene family 6 | --- | -0.646 | 10 | 0.016 |  | | | |
| 160624\_at | NoneAvailable | Mus musculus, clone IMAGE:5401580, mRNA | chr4 | -0.596 | 10 | 0.036 |  | | | |
| 160698\_s\_at | Prkcd | protein kinase C, delta | chr14 | -0.279 | 10 | 0.034 |  | | | |
| 160742\_at | Plod3 | procollagen-lysine, 2-oxoglutarate 5-dioxygenase 3 | --- | -0.075 | 10 | 0.043 |  | | | |
| 160762\_at | Abr | active BCR-related gene | chr11 | 0.372 | 10 | 0.006 |  | | | |
| 160781\_r\_at | Unc93b | unc-93 homolog B (C. elegans) | chr19 | -0.132 | 10 | 0.011 |  | | | |
| 160832\_at | Ldlr | low density lipoprotein receptor | chr9 | 1.302 | 10 | 0.036 |  | | | |
| 160834\_at | 1110032C13Rik | RIKEN cDNA 1110032C13 gene | chr7 | -4.49 | 10 | 0.04 |  | | | |
| 160920\_at | Bcl2l2 | Bcl2-like 2 | chr14 | -1.72 | 10 | 0.02 |  | | | |
| 160965\_at | AA793972 | EST AA793972 | chr5 | -3.294 | 10 | 0.001 |  | | | |
| 160977\_at | Arhgef5 | Rho guanine nucleotide exchange factor (GEF) 5 | chr6 | -3.899 | 10 | 0.031 |  | | | |
| 161005\_at | 5730420B22Rik | RIKEN cDNA 5730420B22 gene | --- | -0.111 | 10 | 0.015 |  | | | |
| 161039\_at | Adam22 | a disintegrin and metalloprotease domain 22 | chr5 | -0.147 | 10 | 0.018 |  | | | |
| 161060\_i\_at | 2310061O04Rik | RIKEN cDNA 2310061O04 gene | chr5 | -0.211 | 10 | 0.047 |  | | | |
| 161077\_f\_at | Smarcd2 | SWI/SNF related, matrix associated, actin dependent regulator of chromatin, subfamily d, member 2 | chr11 | -0.15 | 10 | 0.024 |  | | | |
| 161081\_at | Cpeb2 | cytoplasmic polyadenylation element binding protein 2 | chr5 | -3.809 | 10 | 0.02 |  | | | |
| 161083\_at | A130052D22 | hypothetical protein A130052D22 | --- | 0.607 | 10 | 0.002 |  | | | |
| 161109\_at | 1110017P05Rik | RIKEN cDNA 1110017P05 gene | --- | -1.394 | 10 | 0.011 |  | | | |
| 161112\_at | LOC214424 | hypothetical protein LOC214424 | chr9 | 0.183 | 10 | 0 |  | | | |
| 161113\_at | Esr1 | estrogen receptor 1 (alpha) | chr10 | -1.744 | 10 | 0.007 |  | | | |
| 161165\_f\_at | Lpin2 | lipin 2 | chr17 | -0.341 | 10 | 0.036 |  | | | |
| 161187\_f\_at | 5730589K01Rik | RIKEN cDNA 5730589K01 gene | --- | -0.457 | 10 | 0.002 |  | | | |
| 161333\_f\_at | D1Ertd161e | DNA segment, Chr 1, ERATO Doi 161, expressed | chr1 | -0.304 | 10 | 0.01 |  | | | |
| 161396\_f\_at | NoneAvailable | --- | --- | -0.498 | 10 | 0.011 |  | | | |
| 161530\_r\_at | Sema4a | sema domain, immunoglobulin domain (Ig), transmembrane domain (TM) and short cytoplasmic domain, (semaphorin) 4A | --- | -0.218 | 10 | 0.045 |  | | | |
| 161551\_f\_at | Riok3 | RIO kinase 3 (yeast) | --- | -1.464 | 10 | 0.012 |  | | | |
| 161610\_at | Ndr2 | N-myc downstream regulated 2 | --- | -4.394 | 10 | 0.001 |  | | | |
| 161667\_r\_at | 1110001M20Rik | RIKEN cDNA 1110001M20 gene | chr4 | 0.017 | 10 | 0.041 |  | | | |
| 161689\_f\_at | Il1r2 | interleukin 1 receptor, type II | chr1 | -3.446 | 10 | 0 |  | | | |
| 161745\_f\_at | Hspa4 | heat shock protein 4 | chr11 | -0.317 | 10 | 0.002 |  | | | |
| 161785\_f\_at | D5Wsu46e | DNA segment, Chr 5, Wayne State University 46, expressed | chr5 | -0.441 | 10 | 0.027 |  | | | |
| 161814\_f\_at | Rnf19 | ring finger protein (C3HC4 type) 19 | chr15 | -1.939 | 10 | 0.035 |  | | | |
| 161881\_f\_at | Zfp259 | zinc finger protein 259 | chr9 | -0.311 | 10 | 0.002 |  | | | |
| 161899\_f\_at | Wbscr5 | Williams-Beuren syndrome chromosome region 5 homolog (human) | chr5 | 0.701 | 10 | 0.002 |  | | | |
| 161980\_f\_at | Bag3 | Bcl2-associated athanogene 3 | chr7 | -4.017 | 10 | 0.037 |  | | | |
| 162041\_f\_at | NoneAvailable | --- | --- | -1.038 | 10 | 0.01 |  | | | |
| 162092\_f\_at | Ihpk1 | inositol hexaphosphate kinase 1 | chr9 | -0.407 | 10 | 0.044 |  | | | |
| 162114\_f\_at | Usp49 | ubiquitin specific protease 49 | chr17 | -0.67 | 10 | 0.008 |  | | | |
| 162204\_r\_at | Notch1 | Notch gene homolog 1 (Drosophila) | chr2 | -0.352 | 10 | 0.011 |  | | | |
| 162206\_f\_at | Socs3 | suppressor of cytokine signaling 3 | --- | -4.307 | 10 | 0.013 |  | | | |
| 162228\_f\_at | NoneAvailable | Mus musculus transcribed sequences | --- | -0.565 | 10 | 0.001 |  | | | |
| 162260\_at | 6330407G11Rik | RIKEN cDNA 6330407G11 gene | chr17 | -0.728 | 10 | 0.009 |  | | | |
| 162261\_f\_at | Zp2 | zona pellucida glycoprotein 2 | --- | -0.329 | 10 | 0.023 |  | | | |
| 162384\_f\_at | Ccrn4l | CCR4 carbon catabolite repression 4-like (S. cerevisiae) | --- | -0.633 | 10 | 0.025 |  | | | |
| 162424\_f\_at | 2610007K22Rik | RIKEN cDNA 2610007K22 gene | chr15 | -0.846 | 10 | 0.02 |  | | | |
| 162463\_at | Tpd52 | tumor protein D52 | --- | -0.325 | 10 | 0.047 |  | | | |
| 92208\_at | C1qdc1 | C1q domain containing 1 | chr6 | -0.521 | 10 | 0.003 |  | | | |
| 92217\_s\_at | NoneAvailable | --- | chr10 | 0.093 | 10 | 0.02 |  | | | |
| 92232\_at | Socs3 | suppressor of cytokine signaling 3 | chr11 | -0.365 | 10 | 0.003 |  | | | |
| 92233\_at | 1810007M14Rik | RIKEN cDNA 1810007M14 gene | chr16 | -0.913 | 10 | 0.015 |  | | | |
| 92300\_at | Mnt | max binding protein | chr11 | -0.573 | 10 | 0.007 |  | | | |
| 92318\_at | 2010301N04Rik | RIKEN cDNA 2010301N04 gene | chr6 | -0.432 | 10 | 0.019 |  | | | |
| 92339\_at | Taf1a | TATA box binding protein (Tbp)-associated factor, RNA polymerase I, A | --- | -0.038 | 10 | 0.002 |  | | | |
| 92400\_at | Ndst2 | N-deacetylase/N-sulfotransferase (heparan glucosaminyl) 2 | chr14 | -0.661 | 10 | 0.004 |  | | | |
| 92542\_at | D4Wsu53e | DNA segment, Chr 4, Wayne State University 53, expressed | chr4 | -1.236 | 10 | 0.014 |  | | | |
| 92571\_at | Hspa4 | heat shock protein 4 | chr11 | -0.736 | 10 | 0.002 |  | | | |
| 92634\_at | Dpp4 | dipeptidylpeptidase 4 | chr2 | -0.409 | 10 | 0.044 |  | | | |
| 92708\_at | 2810457M08Rik | RIKEN cDNA 2810457M08 gene | chr8 | -0.324 | 10 | 0.031 |  | | | |
| 92737\_at | Irf4 | interferon regulatory factor 4 | chr13 | -0.317 | 10 | 0.008 |  | | | |
| 92758\_at | Dusp2 | dual specificity phosphatase 2 | chr2 | -0.942 | 10 | 0.002 |  | | | |
| 92877\_at | Tgfbi | transforming growth factor, beta induced | chr13 | -1.788 | 10 | 0.029 |  | | | |
| 92918\_at | F7 | coagulation factor VII | chr8 | -0.006 | 10 | 0.04 |  | | | |
| 92945\_at | NoneAvailable | --- | chr3 | 0.395 | 10 | 0.005 |  | | | |
| 92986\_g\_at | Ptprj | protein tyrosine phosphatase, receptor type, J | chr12 | -0.245 | 10 | 0.028 |  | | | |
| 92991\_at | Sp4 | trans-acting transcription factor 4 | chr12 | -0.902 | 10 | 0.04 |  | | | |
| 92992\_i\_at | 5730497N03Rik | RIKEN cDNA 5730497N03 gene | chr12 | -1.16 | 10 | 0.027 |  | | | |
| 92993\_r\_at | 5730497N03Rik | RIKEN cDNA 5730497N03 gene | chr12 | -1.744 | 10 | 0.003 |  | | | |
| 93104\_at | Btg1 | B-cell translocation gene 1, anti-proliferative | chr10 | -3.713 | 10 | 0.005 |  | | | |
| 93193\_at | Adrb2 | adrenergic receptor, beta 2 | chr18 | -1.617 | 10 | 0.021 |  | | | |
| 93199\_at | NoneAvailable | M.musculus mRNA for L41 ribosomal like-protein | --- | -0.244 | 10 | 0.033 |  | | | |
| 93274\_at | Clk | CDC-like kinase | chr1 | -1.211 | 10 | 0.031 |  | | | |
| 93311\_at | Clk3 | CDC-like kinase 3 | chr9 | -1.389 | 10 | 0.003 |  | | | |
| 93315\_at | Map2k3 | mitogen activated protein kinase kinase 3 | chr11 | -2.248 | 10 | 0.044 |  | | | |
| 93319\_at | Rasa3 | RAS p21 protein activator 3 | chr8 | -0.464 | 10 | 0.004 |  | | | |
| 93321\_at | Ifi203 | interferon activated gene 203 | chr1 | -0.168 | 10 | 0.003 |  | | | |
| 93339\_at | Mdm4 | transformed mouse 3T3 cell double minute 4 | chr1 | -0.202 | 10 | 0.004 |  | | | |
| 93408\_at | NoneAvailable | Mus musculus transcribed sequences | chr8 | -0.44 | 10 | 0.001 |  | | | |
| 93414\_at | Abcb1b | ATP-binding cassette, sub-family B (MDR/TAP), member 1B | chr5 | -1.239 | 10 | 0.019 |  | | | |
| 93424\_at | NoneAvailable | Mus musculus, Similar to KIAA0916 protein, clone IMAGE:4022573, mRNA | chr14 | -1.14 | 10 | 0.024 |  | | | |
| 93440\_at | 4930564D15Rik | RIKEN cDNA 4930564D15 gene | chr3 | -1.001 | 10 | 0.017 |  | | | |
| 93492\_at | Pscd2 | pleckstrin homology, Sec7 and coiled-coil domains 2 | chr7 | -0.593 | 10 | 0.022 |  | | | |
| 93509\_at | Ube2b | ubiquitin-conjugating enzyme E2B, RAD6 homology (S. cerevisiae) | chr11 | -0.724 | 10 | 0.049 |  | | | |
| 93520\_at | Srrm1 | serine/arginine repetitive matrix 1 | --- | -1.338 | 10 | 0.018 |  | | | |
| 93557\_at | Sps2 | selenophosphate synthetase 2 | chr7 | -0.116 | 10 | 0.016 |  | | | |
| 93570\_at | Slc12a3 | solute carrier family 12, member 3 | chr8 | -0.004 | 10 | 0.011 |  | | | |
| 93627\_at | E430019N21Rik | RIKEN cDNA E430019N21 gene | chr14 | -0.592 | 10 | 0.002 |  | | | |
| 93701\_at | Smarca5 | SWI/SNF related, matrix associated, actin dependent regulator of chromatin, subfamily a, member 5 | chr4 | 0.12 | 10 | 0.01 |  | | | |
| 93718\_at | Rab23 | RAB23, member RAS oncogene family | --- | -0.035 | 10 | 0.016 |  | | | |
| 93744\_at | Calm4 | calmodulin 4 | chr13 | -0.05 | 10 | 0.041 |  | | | |
| 93753\_at | Litaf | LPS-induced TN factor | chr16 | -1.716 | 10 | 0.013 |  | | | |
| 93852\_at | Mef2a | myocyte enhancer factor 2A | chr7 | -2.066 | 10 | 0.044 |  | | | |
| 93909\_f\_at | NoneAvailable | --- | --- | -0.211 | 10 | 0.002 |  | | | |
| 93914\_at | Il1r1 | interleukin 1 receptor, type I | chr1 | -1.087 | 10 | 0.006 |  | | | |
| 93965\_r\_at | Ddx6 | DEAD (Asp-Glu-Ala-Asp) box polypeptide 6 | chr9 | -3.007 | 10 | 0.007 |  | | | |
| 93975\_at | 1300002F13Rik | RIKEN cDNA 1300002F13 gene | chr4 | -6.877 | 10 | 0 |  | | | |
| 93978\_at | D230016N13Rik | RIKEN cDNA D230016N13 gene | --- | -0.085 | 10 | 0.016 |  | | | |
| 93980\_at | BC019943 | cDNA sequence BC019943 | --- | 0.418 | 10 | 0.048 |  | | | |
| 94003\_at | NoneAvailable | Mus musculus cDNA clone IMAGE:6490905, partial cds | chr6 | -0.8 | 10 | 0.049 |  | | | |
| 94006\_at | Azi2 | 5-azacytidine induced gene 2 | chr16 | -0.771 | 10 | 0 |  | | | |
| 94011\_at | 3100004P22Rik | RIKEN cDNA 3100004P22 gene | chr7 | -0.355 | 10 | 0.011 |  | | | |
| 94012\_at | Timm13a | translocase of inner mitochondrial membrane 13 homolog a (yeast) | --- | 0.276 | 10 | 0.026 |  | | | |
| 94042\_f\_at | Gng5 | guanine nucleotide binding protein (G protein), gamma 5 subunit | chr15 | -0.485 | 10 | 0.006 |  | | | |
| 94192\_at | Gdap10 | ganglioside-induced differentiation-associated-protein 10 | chr12 | -2.38 | 10 | 0.047 |  | | | |
| 94224\_s\_at | Ifi205 | interferon activated gene 205 | chr1 | -0.908 | 10 | 0.002 |  | | | |
| 94236\_at | Nisch | nischarin | chr14 | -0.278 | 10 | 0.002 |  | | | |
| 94264\_at | Raf1 | v-raf-1 leukemia viral oncogene 1 | chr6 | -1.251 | 10 | 0.001 |  | | | |
| 94300\_f\_at | 2310042E05Rik | RIKEN cDNA 2310042E05 gene | chr10 | -0.68 | 10 | 0.001 |  | | | |
| 94331\_at | Stat6 | signal transducer and activator of transcription 6 | chr10 | -2.796 | 10 | 0 |  | | | |
| 94341\_at | Jmj | jumonji | chr13 | -0.648 | 10 | 0.036 |  | | | |
| 94397\_at | 1200014O24Rik | RIKEN cDNA 1200014O24 gene | chr10 | 0.361 | 10 | 0.002 |  | | | |
| 94483\_at | Csnk2a2 | casein kinase II, alpha 2, polypeptide | chr8 | -1.076 | 10 | 0.04 |  | | | |
| 94484\_at | Hbs1l | Hbs1-like (S. cerevisiae) | chr10 | 0.209 | 10 | 0.017 |  | | | |
| 94689\_at | C79248 | expressed sequence C79248 | --- | -1.2 | 10 | 0.015 |  | | | |
| 94780\_at | Zfp288 | zinc finger protein 288 | chr16 | -4.353 | 10 | 0.034 |  | | | |
| 94818\_at | Ogt | O-linked N-acetylglucosamine (GlcNAc) transferase (UDP-N-acetylglucosamine:polypeptide-N-acetylglucosaminyl transferase) | chrX | -1.371 | 10 | 0.004 |  | | | |
| 94830\_at | BC005537 | cDNA sequence BC005537 | chr13 | -1.83 | 10 | 0.008 |  | | | |
| 94899\_at | Rhoip3-pending | Rho interacting protein 3 | chr11 | -1.473 | 10 | 0.042 |  | | | |
| 94928\_at | Tnfrsf1b | tumor necrosis factor receptor superfamily, member 1b | --- | -1.859 | 10 | 0.009 |  | | | |
| 94932\_at | Pdgfa | platelet derived growth factor, alpha | chr5 | -0.678 | 10 | 0.022 |  | | | |
| 94939\_at | Cd53 | CD53 antigen | chr3 | -1.067 | 10 | 0.004 |  | | | |
| 94951\_at | 1810030A06Rik | RIKEN cDNA 1810030A06 gene | chrX | 0.853 | 10 | 0.03 |  | | | |
| 94979\_at | BC018507 | cDNA sequence BC018507 | chr13 | -0.219 | 10 | 0.021 |  | | | |
| 94980\_at | Dusp11 | dual specificity phosphatase 11 (RNA/RNP complex 1-interacting) | chr6 | -1.6 | 10 | 0.004 |  | | | |
| 95021\_at | 9430010O03Rik | RIKEN cDNA 9430010O03 gene | chr8 | -0.858 | 10 | 0.002 |  | | | |
| 95023\_at | BC023957 | cDNA sequence BC023957 | chr9 | -1.742 | 10 | 0 |  | | | |
| 95081\_at | P38ip-pending | transcription factor (p38 interacting protein) | chr3 | -0.31 | 10 | 0.03 |  | | | |
| 95095\_at | Flot1 | flotillin 1 | chr17 | 0.746 | 10 | 0.007 |  | | | |
| 95119\_at | 1110038D17Rik | RIKEN cDNA 1110038D17 gene | chr10 | -1.366 | 10 | 0.009 |  | | | |
| 95123\_at | 4930566A11Rik | RIKEN cDNA 4930566A11 gene | chr8 | -0.035 | 10 | 0.024 |  | | | |
| 95155\_at | B830022L21Rik | RIKEN cDNA B830022L21 gene | chr8 | -0.064 | 10 | 0.023 |  | | | |
| 95287\_at | NoneAvailable | Mus musculus RIKEN cDNA 4930471C18 gene, mRNA (cDNA clone IMAGE:4487650), partial cds | chr6 | -1.232 | 10 | 0.032 |  | | | |
| 95295\_s\_at | Flt3 | FMS-like tyrosine kinase 3 | chr5 | 0.496 | 10 | 0.006 |  | | | |
| 95355\_at | Agtrap | angiotensin II, type I receptor-associated protein | --- | -0.466 | 10 | 0.012 |  | | | |
| 95382\_at | NoneAvailable | Mus musculus transcribed sequences | --- | -0.751 | 10 | 0 |  | | | |
| 95387\_f\_at | Sema4b | sema domain, immunoglobulin domain (Ig), transmembrane domain (TM) and short cytoplasmic domain, (semaphorin) 4B | chr7 | 0.341 | 10 | 0.032 |  | | | |
| 95398\_at | NoneAvailable | Mus musculus transcribed sequences | chr1 | -0.096 | 10 | 0.047 |  | | | |
| 95433\_at | Ddx54 | DEAD (Asp-Glu-Ala-Asp) box polypeptide 54 | chr5 | 1.761 | 10 | 0.017 |  | | | |
| 95444\_at | 4930579A11Rik | RIKEN cDNA 4930579A11 gene | chr11 | -2.862 | 10 | 0.016 |  | | | |
| 95489\_at | Fliih | flightless I homolog (Drosophila) | chr11 | -1.074 | 10 | 0.042 |  | | | |
| 95501\_at | 2410001C21Rik | RIKEN cDNA 2410001C21 gene | --- | 0.09 | 10 | 0.016 |  | | | |
| 95502\_at | Sirt2 | sirtuin 2 (silent mating type information regulation 2, homolog) 2 (S. cerevisiae) | chr7 | -0.093 | 10 | 0.034 |  | | | |
| 95521\_s\_at | Zfp68 | Zinc finger protein 68 | chr5 | -2.132 | 10 | 0.035 |  | | | |
| 95533\_at | Zfp106 | zinc finger protein 106 | --- | -0.751 | 10 | 0.014 |  | | | |
| 95536\_at | Tceb3 | transcription elongation factor B (SIII), polypeptide 3 | chr4 | -0.823 | 10 | 0.003 |  | | | |
| 95564\_at | BC018601 | cDNA sequence BC018601 | chr11 | -2.789 | 10 | 0.032 |  | | | |
| 95573\_at | Baz2a | bromodomain adjacent to zinc finger domain, 2A | chr10 | -0.788 | 10 | 0 |  | | | |
| 95574\_f\_at | Cggbp1 | CGG triplet repeat binding protein 1 | --- | -0.136 | 10 | 0.035 |  | | | |
| 95586\_at | P2rx4 | purinergic receptor P2X, ligand-gated ion channel 4 | chr5 | -1.711 | 10 | 0 |  | | | |
| 95607\_at | Stard3 | START domain containing 3 | chr11 | -0.126 | 10 | 0.019 |  | | | |
| 95647\_f\_at | 4022402H07Rik | RIKEN cDNA 4022402H07 gene | chr6 | -0.734 | 10 | 0.01 |  | | | |
| 95655\_at | 5830411E10Rik | RIKEN cDNA 5830411E10 gene | chr1 | -1.472 | 10 | 0.036 |  | | | |
| 95686\_at | Rab14 | RAB14, member RAS oncogene family | --- | -0.214 | 10 | 0.01 |  | | | |
| 95694\_at | Top1 | topoisomerase (DNA) I | chr2 | 0.495 | 10 | 0.006 |  | | | |
| 95917\_at | NoneAvailable | Mus musculus transcribed sequences | chr8 | -4.563 | 10 | 0.041 |  | | | |
| 96056\_at | Arhc | ras homolog gene family, member C | chr3 | -0.212 | 10 | 0.043 |  | | | |
| 96176\_at | Arih2 | ariadne homolog 2 (Drosophila) | chr9 | -1.874 | 10 | 0.022 |  | | | |
| 96189\_at | 2410141K03Rik | RIKEN cDNA 2410141K03 gene | --- | -2.437 | 10 | 0.013 |  | | | |
| 96196\_i\_at | 5730589K01Rik | RIKEN cDNA 5730589K01 gene | --- | -0.202 | 10 | 0.021 |  | | | |
| 96197\_f\_at | 5730589K01Rik | RIKEN cDNA 5730589K01 gene | --- | -0.685 | 10 | 0.022 |  | | | |
| 96300\_f\_at | Rps27 | ribosomal protein S27 | chr3 | -0.761 | 10 | 0.034 |  | | | |
| 96333\_g\_at | Snx2 | sorting nexin 2 | chr18 | 0.109 | 10 | 0.041 |  | | | |
| 96481\_at | C80638 | expressed sequence C80638 | chr3 | -0.708 | 10 | 0.009 |  | | | |
| 96511\_s\_at | Vav1 | vav 1 oncogene | --- | 0.266 | 10 | 0.046 |  | | | |
| 96534\_at | Vldlr | very low density lipoprotein receptor | chr19 | -1.008 | 10 | 0 |  | | | |
| 96538\_at | Centb1 | centaurin, beta 1 | chr11 | -0.106 | 10 | 0.041 |  | | | |
| 96545\_s\_at | A730042J05Rik | RIKEN cDNA A730042J05 gene | chr8 | -0.115 | 10 | 0.018 |  | | | |
| 96577\_i\_at | NoneAvailable | --- | chr3 | 1.694 | 10 | 0.033 |  | | | |
| 96578\_r\_at | NoneAvailable | --- | --- | -0.683 | 10 | 0.046 |  | | | |
| 96618\_at | 0610037N01Rik | RIKEN cDNA 0610037N01 gene | chr17 | 0.03 | 10 | 0.009 |  | | | |
| 96650\_at | Auh | AU RNA binding protein/enoyl-coenzyme A hydratase | chr13 | 0.434 | 10 | 0.001 |  | | | |
| 96751\_at | 2810410P22Rik | RIKEN cDNA 2810410P22 gene | chr2 | 0.168 | 10 | 0.001 |  | | | |
| 96784\_at | 2900037I21Rik | RIKEN cDNA 2900037I21 gene | chr9 | 1.467 | 10 | 0.045 |  | | | |
| 96813\_f\_at | DXImx46e | DNA segment, Chr X, Immunex 46, expressed | chrX | -1.437 | 10 | 0.027 |  | | | |
| 96817\_at | 2700067D09Rik | RIKEN cDNA 2700067D09 gene | chr16 | -0.617 | 10 | 0.012 |  | | | |
| 96818\_at | Dtx2 | deltex 2 homolog (Drosophila) | chr5 | -0.13 | 10 | 0.039 |  | | | |
| 96845\_at | Slc30a9 | solute carrier family 30 (zinc transporter), member 9 | chr5 | -0.927 | 10 | 0.002 |  | | | |
| 97083\_at | Eif2s2 | eukaryotic translation initiation factor 2, subunit 2 (beta) | chr2 | -0.842 | 10 | 0.018 |  | | | |
| 97118\_at | 1810028B20Rik | RIKEN cDNA 1810028B20 gene | chr19 | -1.614 | 10 | 0.036 |  | | | |
| 97285\_f\_at | Ubxdc2 | UBX domain-containing 2 | chr17 | -2.548 | 10 | 0 |  | | | |
| 97297\_at | 1500036F01Rik | RIKEN cDNA 1500036F01 gene | chr1 | -1.772 | 10 | 0.001 |  | | | |
| 97319\_at | Rrad | Ras-related associated with diabetes | chr8 | -2.544 | 10 | 0.005 |  | | | |
| 97349\_at | 4930488L10Rik | RIKEN cDNA 4930488L10 gene | chr12 | -2.819 | 10 | 0.04 |  | | | |
| 97384\_at | Gmfg | glia maturation factor, gamma | chr7 | 0.552 | 10 | 0.004 |  | | | |
| 97386\_at | 1110032O19Rik | RIKEN cDNA 1110032O19 gene | chr8 | -0.598 | 10 | 0.031 |  | | | |
| 97429\_at | Snrk | SNF related kinase | chr9 | -1.935 | 10 | 0.021 |  | | | |
| 97497\_at | Notch1 | Notch gene homolog 1 (Drosophila) | chr2 | 1.19 | 10 | 0.011 |  | | | |
| 97509\_f\_at | Fgfr1 | fibroblast growth factor receptor 1 | chr8 | -0.746 | 10 | 0.003 |  | | | |
| 97593\_f\_at | Fliih | flightless I homolog (Drosophila) | chr11 | -0.685 | 10 | 0.014 |  | | | |
| 97710\_f\_at | C530046L02Rik | RIKEN cDNA C530046L02 gene | chr17 | -0.132 | 10 | 0 |  | | | |
| 97843\_at | Ncoa4 | nuclear receptor coactivator 4 | chr12 | -1.253 | 10 | 0.003 |  | | | |
| 97848\_at | Rbmx | RNA binding motif protein, X chromosome | chrX | -0.951 | 10 | 0.013 |  | | | |
| 97897\_at | NoneAvailable | Mus musculus, clone IMAGE:6430978, mRNA | chr13 | -1.708 | 10 | 0.027 |  | | | |
| 97936\_at | 2810407L07Rik | RIKEN cDNA 2810407L07 gene | chr6 | -0.421 | 10 | 0.034 |  | | | |
| 98000\_at | Ly64 | lymphocyte antigen 64 | chr16 | -2.084 | 10 | 0.036 |  | | | |
| 98002\_at | Icsbp1 | interferon consensus sequence binding protein 1 | chr8 | 1.28 | 10 | 0.01 |  | | | |
| 98018\_at | Procr | protein C receptor, endothelial | chr2 | -4.119 | 10 | 0.038 |  | | | |
| 98057\_at | 1110031E24Rik | RIKEN cDNA 1110031E24 gene | chr8 | -0.259 | 10 | 0.001 |  | | | |
| 98356\_at | NoneAvailable | Mus musculus transcribed sequences | chr16 | -0.627 | 10 | 0.03 |  | | | |
| 98435\_at | Adss | adenylosuccinate synthetase, muscle | chr12 | 0.249 | 10 | 0.027 |  | | | |
| 98461\_at | 1200014P03Rik | RIKEN cDNA 1200014P03 gene | chr17 | -1.307 | 10 | 0.007 |  | | | |
| 98465\_f\_at | Ifi204 | interferon activated gene 204 | chr1 | 0.141 | 10 | 0.002 |  | | | |
| 98495\_at | 5033414D02Rik | RIKEN cDNA 5033414D02 gene | chr19 | -0.739 | 10 | 0.006 |  | | | |
| 98533\_at | Cyb5 | cytochrome b-5 | chr18 | -1.221 | 10 | 0.003 |  | | | |
| 98534\_at | Sap18 | Sin3-associated polypeptide 18 | chr14 | -0.144 | 10 | 0.029 |  | | | |
| 98767\_at | Yy1 | YY1 transcription factor | chr12 | -0.558 | 10 | 0.008 |  | | | |
| 98818\_at | Nr3c1 | nuclear receptor subfamily 3, group C, member 1 | chr18 | 0.019 | 10 | 0.013 |  | | | |
| 98862\_at | Wnt10a | wingless related MMTV integration site 10a | chr1 | -0.233 | 10 | 0.011 |  | | | |
| 98882\_s\_at | Ndel1 | nuclear distribution gene E-like homolog 1 (A. nidulans) | chr11 | -1.562 | 10 | 0 |  | | | |
| 98884\_r\_at | Ndel1 | nuclear distribution gene E-like homolog 1 (A. nidulans) | chr11 | -2.864 | 10 | 0.022 |  | | | |
| 98926\_at | Vamp2 | vesicle-associated membrane protein 2 | chr11 | -2.233 | 10 | 0 |  | | | |
| 98951\_at | D8Ertd325e | DNA segment, Chr 8, ERATO Doi 325, expressed | chr8 | -1.148 | 10 | 0.009 |  | | | |
| 99045\_at | Eno2 | enolase 2, gamma neuronal | --- | -1.634 | 10 | 0.008 |  | | | |
| 99085\_at | Usp3 | ubiquitin specific protease 3 | chr9 | 0.285 | 10 | 0.034 |  | | | |
| 99086\_g\_at | Usp3 | ubiquitin specific protease 3 | chr9 | -0.289 | 10 | 0.017 |  | | | |
| 99100\_at | Stat3 | signal transducer and activator of transcription 3 | chr11 | -1.118 | 10 | 0.022 |  | | | |
| 99103\_at | Irf3 | interferon regulatory factor 3 | chr7 | -1.113 | 10 | 0.042 |  | | | |
| 99143\_at | Tgoln1 | trans-golgi network protein | chr6 | -2.457 | 10 | 0.021 |  | | | |
| 99160\_s\_at | 1110025J15Rik | RIKEN cDNA 1110025J15 gene | chr15 | -0.778 | 10 | 0.017 |  | | | |
| 99184\_at | Csad | cysteine sulfinic acid decarboxylase | --- | -2.259 | 10 | 0.001 |  | | | |
| 99187\_f\_at | 2010315L10Rik | RIKEN cDNA 2010315L10 gene | chr8 | -1.207 | 10 | 0.004 |  | | | |
| 99188\_at | 2010315L10Rik | RIKEN cDNA 2010315L10 gene | chr8 | -1.031 | 10 | 0.002 |  | | | |
| 99347\_f\_at | NoneAvailable | Mus musculus transcribed sequences | --- | -2.205 | 10 | 0.048 |  | | | |
| 99445\_at | 1110028E10Rik | RIKEN cDNA 1110028E10 gene | chr9 | -1.202 | 10 | 0.035 |  | | | |
| 99458\_i\_at | Mark2 | MAP/microtubule affinity-regulating kinase 2 | chr19 | -0.678 | 10 | 0.022 |  | | | |
| 99617\_at | LOC218811 | hypothetical protein LOC218811 | chr14 | -0.043 | 10 | 0.027 |  | | | |
| 99665\_at | Satb1 | special AT-rich sequence binding protein 1 | chr17 | 0.476 | 10 | 0.024 |  | | | |
| 99953\_at | Rgl2 | ral guanine nucleotide dissociation stimulator,-like 2 | chr17 | -0.019 | 10 | 0.009 |  | | | |
| 99985\_at | Txnrd1 | thioredoxin reductase 1 | chr10 | -1.205 | 10 | 0.045 |  | | | |
| 100115\_at | 2610028H07Rik | RIKEN cDNA 2610028H07 gene | chr9 | 0.017 | 30 | 0.036 |  | | | |
| 100462\_at | Arf6 | ADP-ribosylation factor 6 | chr12 | -0.219 | 30 | 0.018 |  | | | |
| 100482\_at | BC023040 | cDNA sequence BC023040 | chr17 | -1.285 | 30 | 0.005 |  | | | |
| 100583\_at | Igh-VJ558 | immunoglobulin heavy chain (J558 family) | chr12 | -1.148 | 30 | 0.003 |  | | | |
| 100696\_at | Pde6a | phosphodiesterase 6A, cGMP-specific, rod, alpha | --- | -0.117 | 30 | 0.041 |  | | | |
| 100972\_s\_at | Ccl27 | chemokine (C-C motif) ligand 27 | --- | -0.931 | 30 | 0.001 |  | | | |
| 101352\_g\_at | NoneAvailable | --- | chr7 | 0.015 | 30 | 0.005 |  | | | |
| 101843\_at | Sh2bpsm1 | SH2-B PH domain containing signaling mediator 1 | chr7 | -0.153 | 30 | 0.007 |  | | | |
| 102028\_at | Rassf5 | Ras association (RalGDS/AF-6) domain family 5 | chr1 | 0.398 | 30 | 0.014 |  | | | |
| 102152\_f\_at | NoneAvailable | --- | --- | -0.22 | 30 | 0.006 |  | | | |
| 102224\_at | Igf1r | insulin-like growth factor I receptor | --- | -1.399 | 30 | 0.011 |  | | | |
| 102302\_at | Bckdhb | branched chain ketoacid dehydrogenase E1, beta polypeptide | chr9 | -1.776 | 30 | 0.019 |  | | | |
| 102335\_at | Kcnk1 | potassium channel, subfamily K, member 1 | chr8 | -0.114 | 30 | 0.025 |  | | | |
| 102691\_at | Zfp385 | zinc finger protein 385 | chr15 | -0.099 | 30 | 0.045 |  | | | |
| 102789\_at | Gata2 | GATA binding protein 2 | chr6 | -1.995 | 30 | 0.048 |  | | | |
| 102873\_at | Tap2 | transporter 2, ATP-binding cassette, sub-family B (MDR/TAP) | chr17 | -0.371 | 30 | 0.007 |  | | | |
| 103052\_r\_at | Nr2f2 | nuclear receptor subfamily 2, group F, member 2 | chr7 | -0.983 | 30 | 0.018 |  | | | |
| 103091\_at | Relb | avian reticuloendotheliosis viral (v-rel) oncogene related B | chr7 | -0.368 | 30 | 0 |  | | | |
| 103236\_at | Ring1 | ring finger protein 1 | chr17 | -0.114 | 30 | 0.04 |  | | | |
| 103547\_at | Slc41a1 | solute carrier family 41, member 1 | chr1 | -2.728 | 30 | 0 |  | | | |
| 103666\_at | Hoxb5 | homeo box B5 | chr11 | -0.825 | 30 | 0.035 |  | | | |
| 103727\_at | D730048C23Rik | RIKEN cDNA D730048C23 gene | --- | -0.44 | 30 | 0.037 |  | | | |
| 103793\_at | Mvp | major vault protein | --- | -0.37 | 30 | 0.006 |  | | | |
| 103954\_at | Reg3a | regenerating islet-derived 3 alpha | chr6 | 0.122 | 30 | 0 |  | | | |
| 103958\_g\_at | Trfr | transferrin receptor | chr16 | -0.439 | 30 | 0.046 |  | | | |
| 104083\_at | Cdh5 | cadherin 5 | chr8 | -1.968 | 30 | 0.003 |  | | | |
| 104139\_at | P4ha1 | procollagen-proline, 2-oxoglutarate 4-dioxygenase (proline 4-hydroxylase), alpha 1 polypeptide | chr10 | -0.637 | 30 | 0.006 |  | | | |
| 104243\_r\_at | 4930578F06Rik | RIKEN cDNA 4930578F06 gene | --- | -0.225 | 30 | 0.036 |  | | | |
| 104287\_at | Smt3ip1-pending | smt3-specific isopeptidase 1 | chr11 | -0.382 | 30 | 0.03 |  | | | |
| 104292\_at | Eya2 | eyes absent 2 homolog (Drosophila) | chr2 | -0.122 | 30 | 0.042 |  | | | |
| 104363\_at | NoneAvailable | Mus musculus cDNA clone MGC:60763 IMAGE:30058959, complete cds | chr17 | -0.22 | 30 | 0.035 |  | | | |
| 104376\_at | Hdac5 | histone deacetylase 5 | chr11 | -2.263 | 30 | 0.006 |  | | | |
| 104417\_at | NoneAvailable | Mus musculus transcribed sequences | chr11 | -2.062 | 30 | 0.001 |  | | | |
| 104438\_at | Zfp30 | zinc finger protein 30 | chr7 | -0.926 | 30 | 0.018 |  | | | |
| 104645\_at | Klf7 | Kruppel-like factor 7 (ubiquitous) | chr1 | -1.19 | 30 | 0.003 |  | | | |
| 160228\_at | 1110019C08Rik | RIKEN cDNA 1110019C08 gene | chr16 | -1.025 | 30 | 0.025 |  | | | |
| 160244\_at | Fem1a | feminization 1 homolog a (C. elegans) | chr17 | 0 | 30 | 0.005 |  | | | |
| 160433\_at | 2400010D15Rik | RIKEN cDNA 2400010D15 gene | chr18 | -0.08 | 30 | 0 |  | | | |
| 160463\_at | Myd116 | myeloid differentiation primary response gene 116 | chr7 | -0.282 | 30 | 0.048 |  | | | |
| 160498\_at | Ldb1 | LIM domain binding 1 | chr19 | 0.031 | 30 | 0.041 |  | | | |
| 160606\_r\_at | Adamts1 | a disintegrin-like and metalloprotease (reprolysin type) with thrombospondin type 1 motif, 1 | chr16 | -0.354 | 30 | 0.009 |  | | | |
| 160651\_at | Tacstd2 | tumor-associated calcium signal transducer 2 | chr6 | -3.696 | 30 | 0 |  | | | |
| 160686\_at | 5730555F13Rik | RIKEN cDNA 5730555F13 gene | --- | -0.692 | 30 | 0.044 |  | | | |
| 160727\_at | 2410002F23Rik | RIKEN cDNA 2410002F23 gene | chr2 | -1.032 | 30 | 0.005 |  | | | |
| 160776\_at | D1Ucla4 | DNA segment, Chr 1, University of California at Los Angeles 4 | chr1 | -0.971 | 30 | 0.006 |  | | | |
| 160789\_at | 9530090G24Rik | RIKEN cDNA 9530090G24 gene | chr2 | 0.122 | 30 | 0.008 |  | | | |
| 160802\_at | Ppan | peter pan homolog (Drosophila) | chr9 | 0.48 | 30 | 0.015 |  | | | |
| 160915\_at | B4galt3 | UDP-Gal:betaGlcNAc beta 1,4-galactosyltransferase, polypeptide 3 | chr1 | -0.179 | 30 | 0.004 |  | | | |
| 160957\_at | D12Ertd7e | DNA segment, Chr 12, ERATO Doi 7, expressed | chr12 | 1.614 | 30 | 0.04 |  | | | |
| 161080\_f\_at | 1700012P16Rik | RIKEN cDNA 1700012P16 gene | chr5 | -1.148 | 30 | 0.008 |  | | | |
| 161148\_f\_at | Ing4 | inhibitor of growth family, member 4 | chr6 | -0.94 | 30 | 0.02 |  | | | |
| 161184\_f\_at | Tie1 | tyrosine kinase receptor 1 | chr4 | -1.63 | 30 | 0.006 |  | | | |
| 161348\_r\_at | Pdlim1 | PDZ and LIM domain 1 (elfin) | --- | 0.078 | 30 | 0.02 |  | | | |
| 161392\_f\_at | Lrpb7 | leucine rich protein, B7 gene | chr6 | -0.815 | 30 | 0.022 |  | | | |
| 161534\_f\_at | 1110038D17Rik | RIKEN cDNA 1110038D17 gene | --- | -0.194 | 30 | 0.034 |  | | | |
| 161576\_f\_at | Fln29-pending | FLN29 gene product | chr5 | -0.323 | 30 | 0.031 |  | | | |
| 161613\_at | 6330407G04Rik | RIKEN cDNA 6330407G04 gene | --- | -0.392 | 30 | 0.039 |  | | | |
| 161738\_f\_at | Ilvbl | ilvB (bacterial acetolactate synthase)-like | chr10 | -0.679 | 30 | 0.01 |  | | | |
| 161990\_f\_at | BC012974 | hypothetical gene supported by BC012974 | chr18 | -1.702 | 30 | 0.006 |  | | | |
| 162034\_r\_at | Antxr2 | anthrax toxin receptor 2 | --- | -0.317 | 30 | 0.008 |  | | | |
| 162080\_f\_at | 2900008M13Rik | RIKEN cDNA 2900008M13 gene | --- | -0.379 | 30 | 0.012 |  | | | |
| 92249\_g\_at | Nr4a2 | nuclear receptor subfamily 4, group A, member 2 | chr2 | -5.506 | 30 | 0.001 |  | | | |
| 92821\_at | Usp2 | ubiquitin specific protease 2 | chr9 | -1.119 | 30 | 0.004 |  | | | |
| 93382\_at | Pde1b | phosphodiesterase 1B, Ca2+-calmodulin dependent | chr15 | -0.176 | 30 | 0.034 |  | | | |
| 93425\_at | Irf5 | interferon regulatory factor 5 | chr6 | -0.888 | 30 | 0.031 |  | | | |
| 93702\_at | AI462446 | expressed sequence AI462446 | chr9 | -0.25 | 30 | 0.015 |  | | | |
| 93768\_f\_at | 2700059D21Rik | RIKEN cDNA 2700059D21 gene | chr4 | -0.412 | 30 | 0.006 |  | | | |
| 93875\_at | Hspa1a | heat shock protein 1A | chr17 | -1.082 | 30 | 0.006 |  | | | |
| 94022\_at | Gltscr2 | glioma tumor suppressor candidate region gene 2 | --- | -0.708 | 30 | 0.015 |  | | | |
| 94060\_at | Myo1h | myosin 1H | chr5 | -1.495 | 30 | 0.002 |  | | | |
| 94146\_at | Ccl4 | chemokine (C-C motif) ligand 4 | chr11 | 0.033 | 30 | 0.009 |  | | | |
| 94657\_at | NoneAvailable | Mus musculus transcribed sequences | chr8 | -5.096 | 30 | 0.006 |  | | | |
| 94796\_at | Psmd11 | proteasome (prosome, macropain) 26S subunit, non-ATPase, 11 | chr11 | 0.269 | 30 | 0.027 |  | | | |
| 94976\_at | AL022610 | expressed sequence AL022610 | chr7 | -1.012 | 30 | 0.029 |  | | | |
| 95001\_at | Akap8 | A kinase (PRKA) anchor protein 8 | chr17 | -0.175 | 30 | 0.019 |  | | | |
| 95002\_at | D17Wsu92e | DNA segment, Chr 17, Wayne State University 92, expressed | chr17 | -1.483 | 30 | 0.016 |  | | | |
| 95012\_at | Slc22a17 | solute carrier family 22 (organic cation transporter), member 17 | chr14 | -0.358 | 30 | 0.042 |  | | | |
| 95016\_at | Nrp | neuropilin | chr8 | 0.029 | 30 | 0.021 |  | | | |
| 95033\_at | Jmjd1 | jumonji domain containing 1 | chr6 | -1.446 | 30 | 0.007 |  | | | |
| 95291\_r\_at | NoneAvailable | Mus musculus transcribed sequences | chr11 | -0.592 | 30 | 0.027 |  | | | |
| 95599\_at | Siat4c | sialyltransferase 4C (beta-galactoside alpha-2,3-sialytransferase) | --- | -0.319 | 30 | 0.002 |  | | | |
| 95618\_at | D6Ertd32e | DNA segment, Chr 6, ERATO Doi 32, expressed | chr6 | -2.616 | 30 | 0.038 |  | | | |
| 95805\_at | Cdc2l2 | cell division cycle 2 homolog (S. pombe)-like 2 | chr4 | -1.429 | 30 | 0.02 |  | | | |
| 95961\_at | BC042396 | cDNA sequence BC042396 | chr9 | 0.125 | 30 | 0.003 |  | | | |
| 96076\_at | Stx5a | syntaxin 5A | chr19 | -1.273 | 30 | 0.008 |  | | | |
| 96088\_at | Ndr2 | N-myc downstream regulated 2 | chr14 | -1.173 | 30 | 0.019 |  | | | |
| 96147\_at | Mafg | v-maf musculoaponeurotic fibrosarcoma oncogene family, protein G (avian) | chr11 | -1.157 | 30 | 0.03 |  | | | |
| 96219\_at | 1810031K02Rik | RIKEN cDNA 1810031K02 gene | chr4 | -0.449 | 30 | 0.043 |  | | | |
| 96270\_at | D11Bwg0434e | DNA segment, Chr 11, Brigham & Women's Genetics 0434 expressed | chr11 | -0.318 | 30 | 0.014 |  | | | |
| 96367\_at | NoneAvailable | Mus musculus transcribed sequences | chr17 | -1.93 | 30 | 0.01 |  | | | |
| 96373\_at | NoneAvailable | --- | chr6 | -0.11 | 30 | 0.035 |  | | | |
| 96637\_at | Tbc1d1 | TBC1 domain family, member 1 | chr5 | 0.29 | 30 | 0.001 |  | | | |
| 96669\_at | 2400003C14Rik | RIKEN cDNA 2400003C14 gene | chr8 | -1.563 | 30 | 0.026 |  | | | |
| 96790\_f\_at | A530057M15Rik | RIKEN cDNA A530057M15 gene | --- | -0.632 | 30 | 0.007 |  | | | |
| 96829\_at | D19Wsu162e | DNA segment, Chr 19, Wayne State University 162, expressed | chr19 | -0.857 | 30 | 0.006 |  | | | |
| 96911\_at | Gnb2 | guanine nucleotide binding protein, beta 2 | chr5 | 1.137 | 30 | 0.031 |  | | | |
| 97125\_f\_at | LOC56628 | MHC (A.CA/J(H-2K-f) class I antigen | chr17 | -3.415 | 30 | 0.007 |  | | | |
| 97375\_at | Pkd1 | polycystic kidney disease 1 homolog | chr17 | -1.292 | 30 | 0.009 |  | | | |
| 97563\_f\_at | NoneAvailable | --- | --- | -0.019 | 30 | 0.03 |  | | | |
| 97684\_at | Prkcabp | protein kinase C, alpha binding protein | chr15 | 0.123 | 30 | 0.005 |  | | | |
| 97813\_at | Rela | v-rel reticuloendotheliosis viral oncogene homolog A (avian) | chr19 | -0.853 | 30 | 0.014 |  | | | |
| 97901\_at | Ubtf | upstream binding transcription factor, RNA polymerase I | chr11 | 0.558 | 30 | 0.013 |  | | | |
| 98065\_at | Ormdl3 | ORM1-like 3 (S. cerevisiae) | chr11 | -1.914 | 30 | 0.032 |  | | | |
| 98385\_at | Ptpn14 | protein tyrosine phosphatase, non-receptor type 14 | chr1 | -0.019 | 30 | 0.021 |  | | | |
| 98402\_at | Macf1 | microtubule-actin crosslinking factor 1 | chr4 | -0.533 | 30 | 0.031 |  | | | |
| 98426\_at | Ppt2 | palmitoyl-protein thioesterase 2 | chr17 | -0.1 | 30 | 0.013 |  | | | |
| 98438\_f\_at | H2-Q7 | histocompatibility 2, Q region locus 7 | chr17 | -3.59 | 30 | 0.001 |  | | | |
| 98861\_at | Sts | steroid sulfatase | --- | -0.975 | 30 | 0.046 |  | | | |
| 98906\_at | Fbxo9 | f-box only protein 9 | chr9 | -2.278 | 30 | 0.037 |  | | | |
| 99142\_at | Mpra-pending | membrane progestin receptor alpha | chr4 | 0.136 | 30 | 0.013 |  | | | |
| 99440\_at | Nfib | nuclear factor I/B | chr4 | -0.459 | 30 | 0.045 |  | | | |
| 99587\_at | Rab7 | RAB7, member RAS oncogene family | chr6 | -0.623 | 30 | 0.032 |  | | | |
| 99644\_at | Zfp289 | zinc finger protein 289 | chr2 | -0.157 | 30 | 0.022 |  | | | |
| 99961\_s\_at | Cdc2l2 | cell division cycle 2 homolog (S. pombe)-like 2 | chr4 | -1.587 | 30 | 0.025 |  | | | |
| 99964\_at | Vdr | vitamin D receptor | chr15 | -0.749 | 30 | 0.034 |  | | | |
| 99970\_at | Ptpn21 | protein tyrosine phosphatase, non-receptor type 21 | chr12 | -1.262 | 30 | 0.008 |  | | | |
| 99992\_at | Il17r | interleukin 17 receptor | chr6 | 0.087 | 30 | 0.02 |  | | | |
| \* Positive log2 fold changes represent genes expressed higher in FL-HSC; Negative log2 fold changes represent genes expressed higher in adult HSC (fold change=2 is equivalent to log2 fold change=1) | | | | | | | | | | |
|  |  |  |  |  |  |  |  |  |  |  |
