# Supplementary material for: Molecular Signatures of Proliferation and Quiescence in Hematopoietic Stem Cells
Source: PLoS Biol. 2004 Sep 28;2(10):e301. doi: 10.1371/journal.pbio.0020301 (PMC520599; doi:10.1371/journal.pbio.0020301)
Supplement: Table S5 — (933 KB HTML). [file pbio.0020301.st005.html]

|  |  | Up-regulated in Adult HSC | |  |  |  |  |  |  |  |
| Probe Set ID | Gene Symbol | Gene name | Chromosome | Log2 Fold Change (FL-HSC vs Adult HSC)\* | Day of max (TOM) | p-value of ANOVA (time course) |  | | | |
| 100122\_at | Gnb5 | guanine nucleotide binding protein, beta 5 | chr9 | -1.744 | 0 | 0.812 |  | | | |
| 100130\_at | Jun | Jun oncogene | chr4 | -2.137 | 0 | 0.111 |  | | | |
| 100417\_at | AI643885 | expressed sequence AI643885 | chr8 | -1.255 | 0 | 0.23 |  | | | |
| 100464\_at | 3110043O21Rik | RIKEN cDNA 3110043O21 gene | chr4 | -2.68 | 0 | 0.478 |  | | | |
| 100509\_at | Rnf19 | ring finger protein (C3HC4 type) 19 | chr15 | -2.123 | 0 | 0.127 |  | | | |
| 100533\_s\_at | Crem | cAMP responsive element modulator | --- | -5.967 | 0 | 0.008 |  | | | |
| 100584\_at | Anxa4 | annexin A4 | chr6 | -1.405 | 0 | 0.222 |  | | | |
| 100600\_at | Cd24a | CD24a antigen | chr10 | -2.172 | 0 | 0.595 |  | | | |
| 100951\_at | Pkd2 | polycystic kidney disease 2 | chr5 | -1.903 | 0 | 0.432 |  | | | |
| 101070\_at | Mkrn1 | makorin, ring finger protein, 1 | chr6 | -2.238 | 0 | 0.286 |  | | | |
| 101484\_at | Nbr1 | neighbor of Brca1 gene 1 | chr11 | -1.113 | 0 | 0.686 |  | | | |
| 101515\_at | Acox1 | acyl-Coenzyme A oxidase 1, palmitoyl | --- | -3.857 | 0 | 0.018 |  | | | |
| 101542\_f\_at | Ddx3x | DEAD/H (Asp-Glu-Ala-Asp/His) box polypeptide 3, X-linked | chrX | -1.168 | 0 | 0.519 |  | | | |
| 101590\_at | Lamp2 | lysosomal membrane glycoprotein 2 | chrX | -1.32 | 0 | 0.994 |  | | | |
| 101681\_f\_at | NoneAvailable | --- | --- | -1.555 | 0 | 0.052 |  | | | |
| 101694\_f\_at | Myst2 | MYST histone acetyltransferase 2 | --- | -1.716 | 0 | 0.057 |  | | | |
| 101878\_at | Cd72 | CD72 antigen | chr4 | -1.325 | 0 | 0.319 |  | | | |
| 102032\_at | Twsg1 | twisted gastrulation homolog 1 (Drosophila) | chr17 | -2.079 | 0 | 0.916 |  | | | |
| 102069\_at | Mtf2 | metal response element binding transcription factor 2 | chr5 | -2.333 | 0 | 0.63 |  | | | |
| 102156\_f\_at | NoneAvailable | --- | chr6 | -3.755 | 0 | 0.005 |  | | | |
| 102209\_at | Nfatc1 | nuclear factor of activated T-cells, cytoplasmic 1 | chr18 | -2.297 | 0 | 0.009 |  | | | |
| 102255\_at | Osmr | oncostatin M receptor | chr15 | -1.198 | 0 | 0.167 |  | | | |
| 102326\_at | Ncf2 | neutrophil cytosolic factor 2 | chr1 | -1.48 | 0 | 0.013 |  | | | |
| 102356\_at | Wdr23 | WD repeat domain 23 | chr14 | -1.437 | 0 | 0.036 |  | | | |
| 102364\_at | Jund1 | Jun proto-oncogene related gene d1 | --- | -2.597 | 0 | 0.382 |  | | | |
| 102657\_at | Hlx | H2.0-like homeo box gene | chr1 | -1.217 | 0 | 0.051 |  | | | |
| 102750\_at | Apba3 | amyloid beta (A4) precursor protein-binding, family A, member 3 | chr10 | -1.172 | 0 | 0.066 |  | | | |
| 102813\_f\_at | 5730525G14Rik | RIKEN cDNA 5730525G14 gene | chr9 | -1.094 | 0 | 0.459 |  | | | |
| 102824\_g\_at | MGC68300 | Unknown (protein for MGC:68300) | chr12 | -2.221 | 0 | 0.404 |  | | | |
| 102839\_at | Plscr1 | phospholipid scramblase 1 | chr1 | -2.355 | 0 | 0.458 |  | | | |
| 102907\_at | C820004H04Rik | RIKEN cDNA C820004H04 gene | chr16 | -1.259 | 0 | 0.054 |  | | | |
| 102929\_s\_at | Flt3l | FMS-like tyrosine kinase 3 ligand | --- | -1.079 | 0 | 0.089 |  | | | |
| 102955\_at | Nfil3 | nuclear factor, interleukin 3, regulated | chr13 | -2.259 | 0 | 0.012 |  | | | |
| 102983\_at | Madh1 | MAD homolog 1 (Drosophila) | chr8 | -1.497 | 0 | 0.234 |  | | | |
| 102994\_at | Stat4 | signal transducer and activator of transcription 4 | chr1 | -1.69 | 0 | 0.092 |  | | | |
| 103037\_at | Ctf1 | cardiotrophin 1 | --- | -1.295 | 0 | 0.012 |  | | | |
| 103065\_at | Slc20a1 | solute carrier family 20, member 1 | chr2 | -2.299 | 0 | 0.337 |  | | | |
| 103226\_at | Mrc1 | mannose receptor, C type 1 | chr2 | -2.184 | 0 | 0.003 |  | | | |
| 103288\_at | Nrip1 | nuclear receptor interacting protein 1 | chr16 | -1.714 | 0 | 0.142 |  | | | |
| 103416\_at | Mapk6 | mitogen-activated protein kinase 6 | chr9 | -1.349 | 0 | 0.806 |  | | | |
| 103427\_at | Fbxl3a | F-box and leucine-rich repeat protein 3a | chr14 | -1.769 | 0 | 0.042 |  | | | |
| 103467\_g\_at | NoneAvailable | Mus musculus mRNA for mKIAA0496 protein | chr15 | -1.251 | 0 | 0.704 |  | | | |
| 103693\_at | C130027E04Rik | RIKEN cDNA C130027E04 gene | --- | -1.673 | 0 | 0.051 |  | | | |
| 103699\_i\_at | Frat2 | frequently rearranged in advanced T-cell lymphomas 2 | --- | -1.204 | 0 | 0.889 |  | | | |
| 103734\_at | Ahi1 | Abelson helper integration site | chr10 | -1.414 | 0 | 0.422 |  | | | |
| 103774\_at | 4933432B13Rik | RIKEN cDNA 4933432B13 gene | chr18 | -1.02 | 0 | 0.449 |  | | | |
| 103816\_at | F11r | F11 receptor | chr1 | -2.918 | 0 | 0.71 |  | | | |
| 103901\_at | 4930451A13Rik | RIKEN cDNA 4930451A13 gene | chr16 | -1.356 | 0 | 0.081 |  | | | |
| 103935\_at | Atp2a3 | ATPase, Ca++ transporting, ubiquitous | chr11 | -1.655 | 0 | 0.064 |  | | | |
| 103990\_at | Fosb | FBJ osteosarcoma oncogene B | chr7 | -4.608 | 0 | 0.012 |  | | | |
| 104108\_at | Rab6ip1 | Rab6 interacting protein 1 | chr7 | -1.723 | 0 | 0.104 |  | | | |
| 104114\_at | 2310050N11Rik | RIKEN cDNA 2310050N11 gene | chr1 | -1.601 | 0 | 0.607 |  | | | |
| 104156\_r\_at | Atf3 | activating transcription factor 3 | chr1 | -1.127 | 0 | 0.775 |  | | | |
| 104252\_at | AU020206 | expressed sequence AU020206 | chr7 | -1.192 | 0 | 0.034 |  | | | |
| 104327\_at | 9030612M13Rik | RIKEN cDNA 9030612M13 gene | --- | -1.029 | 0 | 0.689 |  | | | |
| 104461\_at | 6330412C24Rik | RIKEN cDNA 6330412C24 gene | chr3 | -1.082 | 0 | 0.069 |  | | | |
| 104516\_at | Cldn5 | claudin 5 | chr16 | -1.272 | 0 | 0.048 |  | | | |
| 104522\_at | Itsn | intersectin (SH3 domain protein 1A) | --- | -2.718 | 0 | 0.503 |  | | | |
| 104562\_at | 5730403M16Rik | RIKEN cDNA 5730403M16 gene | chr7 | -1.253 | 0 | 0.009 |  | | | |
| 104598\_at | Dusp1 | dual specificity phosphatase 1 | chr17 | -2.35 | 0 | 0.018 |  | | | |
| 104612\_g\_at | Wdr26 | WD repeat domain 26 | chr1 | -1.512 | 0 | 0.725 |  | | | |
| 104625\_at | Dnajb6 | DnaJ (Hsp40) homolog, subfamily B, member 6 | chr16 | -1.482 | 0 | 0.041 |  | | | |
| 104719\_at | Slc12a7 | solute carrier family 12, member 7 | chr13 | -1.166 | 0 | 0.039 |  | | | |
| 160082\_s\_at | Arf4 | ADP-ribosylation factor 4 | chr14 | -1.223 | 0 | 0.206 |  | | | |
| 160104\_at | Hsd3b7 | hydroxy-delta-5-steroid dehydrogenase, 3 beta- and steroid delta-isomerase 7 | chr7 | -1.424 | 0 | 0.022 |  | | | |
| 160111\_at | 1500010B24Rik | RIKEN cDNA 1500010B24 gene | chrX | -1.004 | 0 | 0.871 |  | | | |
| 160137\_at | B3gnt1 | UDP-GlcNAc:betaGal beta-1,3-N-acetylglucosaminyltransferase 1 | chr11 | -1.201 | 0 | 0.214 |  | | | |
| 160149\_at | Rab10 | RAB10, member RAS oncogene family | chr12 | -1.267 | 0 | 0.823 |  | | | |
| 160200\_at | 3230401D17Rik | RIKEN cDNA 3230401D17 gene | chr11 | -1.904 | 0 | 0.999 |  | | | |
| 160202\_at | Atp6ap2 | ATPase, H+ transporting, lysosomal accessory protein 2 | chrX | -1.533 | 0 | 0.927 |  | | | |
| 160261\_i\_at | Lats2 | large tumor suppressor 2 | chr14 | -3.911 | 0 | 0.434 |  | | | |
| 160288\_at | Map1lc3 | microtubule-associated protein 1 light chain 3 | chr8 | -3.072 | 0 | 0.28 |  | | | |
| 160301\_at | Riok3 | RIO kinase 3 (yeast) | chr18 | -1.331 | 0 | 0.119 |  | | | |
| 160321\_at | Zfp216 | zinc finger protein 216 | chr19 | -2.29 | 0 | 0.239 |  | | | |
| 160391\_at | 0710001O03Rik | RIKEN cDNA 0710001O03 gene | chr19 | -1.04 | 0 | 0.147 |  | | | |
| 160430\_at | Catnb | catenin beta | chr9 | -1.057 | 0 | 0.883 |  | | | |
| 160490\_at | Def8 | differentially expressed in FDCP 8 | chr8 | -1.446 | 0 | 0.053 |  | | | |
| 160526\_s\_at | Crem | cAMP responsive element modulator | --- | -5.103 | 0 | 0.005 |  | | | |
| 160573\_at | Hccs | holocytochrome c synthetase | --- | -2.08 | 0 | 0.025 |  | | | |
| 160575\_at | Cggbp1 | CGG triplet repeat binding protein 1 | chr16 | -1.18 | 0 | 0.145 |  | | | |
| 160679\_at | NoneAvailable | Mus musculus 18-day embryo whole body cDNA, RIKEN full-length enriched library, clone:1110061F19 product:unknown EST, full insert sequence | --- | -1.153 | 0 | 0.467 |  | | | |
| 160684\_at | 8430410A17Rik | RIKEN cDNA 8430410A17 gene | chr6 | -1.855 | 0 | 0.111 |  | | | |
| 160710\_at | Usp33 | ubiquitin specific protease 33 | chr3 | -1.243 | 0 | 0.939 |  | | | |
| 160718\_at | Capn7 | calpain 7 | chr14 | -1.384 | 0 | 0.877 |  | | | |
| 160896\_at | Rcn | reticulocalbin | chr2 | -1.046 | 0 | 0.933 |  | | | |
| 160901\_at | Fos | FBJ osteosarcoma oncogene | chr12 | -3.257 | 0 | 0.024 |  | | | |
| 160911\_at | Sos1 | Son of sevenless homolog 1 (Drosophila) | chr17 | -1.303 | 0 | 0.783 |  | | | |
| 160941\_at | Pde8a | phosphodiesterase 8A | chr7 | -1.266 | 0 | 0.723 |  | | | |
| 160963\_at | 9630050M13Rik | RIKEN cDNA 9630050M13 gene | chr2 | -1.984 | 0 | 0.524 |  | | | |
| 160976\_at | BC012974 | hypothetical gene supported by BC012974 | chr18 | -3.126 | 0 | 0.077 |  | | | |
| 161056\_at | NoneAvailable | Mus musculus, clone IMAGE:1264290, mRNA | --- | -2.081 | 0 | 0.377 |  | | | |
| 161847\_r\_at | NoneAvailable | --- | --- | -1.169 | 0 | 0.077 |  | | | |
| 161951\_f\_at | D130005A03 | hypothetical protein D130005A03 | chr4 | -1.319 | 0 | 0.936 |  | | | |
| 92248\_at | Nr4a2 | nuclear receptor subfamily 4, group A, member 2 | chr2 | -5.877 | 0 | 0.007 |  | | | |
| 92256\_at | Fdft1 | farnesyl diphosphate farnesyl transferase 1 | --- | -1.15 | 0 | 0.003 |  | | | |
| 92270\_at | Tro | trophinin | chrX | -1.72 | 0 | 0.006 |  | | | |
| 92316\_f\_at | LOC207685 | hypothetical protein LOC207685 | chr16 | -1.549 | 0 | 0.272 |  | | | |
| 92392\_at | NoneAvailable | Mus musculus transcribed sequence with strong similarity to protein sp:P22001 (H.sapiens) CIK3\_HUMAN Voltage-gated potassium channel protein Kv1.3 (HPCN3) (HGK5) (HUKIII) (HLK3) | chr3 | -2.836 | 0 | 0.301 |  | | | |
| 92558\_at | Vcam1 | vascular cell adhesion molecule 1 | chr3 | -4.435 | 0 | 0.005 |  | | | |
| 92655\_at | Gcnt1 | glucosaminyl (N-acetyl) transferase 1, core 2 | chr19 | -1.161 | 0 | 0.274 |  | | | |
| 92805\_s\_at | Arl4 | ADP-ribosylation factor-like 4 | chr12 | -1.414 | 0 | 0.294 |  | | | |
| 92830\_s\_at | NoneAvailable | --- | --- | -3.484 | 0 | 0.001 |  | | | |
| 92854\_at | Rab11a | RAB11a, member RAS oncogene family | chr9 | -1.807 | 0 | 0.951 |  | | | |
| 93016\_at | Ywhaq | tyrosine 3-monooxygenase/tryptophan 5-monooxygenase activation protein, theta polypeptide | --- | -1.361 | 0 | 0.429 |  | | | |
| 93025\_at | Ndfip1 | Nedd4 family interacting protein 1 | chr18 | -1.578 | 0 | 0.832 |  | | | |
| 93086\_at | Igk-V8 | immunoglobulin kappa chain variable 8 (V8) | chr6 | -6.147 | 0 | 0.075 |  | | | |
| 93093\_at | Mcl1 | myeloid cell leukemia sequence 1 | chr3 | -2.974 | 0 | 0.018 |  | | | |
| 93120\_f\_at | H2-K | histocompatibility 2, K region | chr17 | -3.637 | 0 | 0.003 |  | | | |
| 93165\_at | 2610022K04Rik | RIKEN cDNA 2610022K04 gene | chr3 | -1.057 | 0 | 0.173 |  | | | |
| 93179\_at | B830009D23Rik | RIKEN cDNA B830009D23 gene | chr2 | -1.837 | 0 | 0.017 |  | | | |
| 93267\_at | Rnpc2 | RNA-binding region (RNP1, RRM) containing 2 | chr2 | -1.572 | 0 | 0.624 |  | | | |
| 93326\_at | Tm4sf2 | transmembrane 4 superfamily member 2 | chrX | -1.259 | 0 | 0.02 |  | | | |
| 93462\_at | 1810054D07Rik | RIKEN cDNA 1810054D07 gene | chr4 | -1.112 | 0 | 0.762 |  | | | |
| 93482\_at | 9530072E15Rik | RIKEN cDNA 9530072E15 gene | chr16 | -3.129 | 0 | 0.15 |  | | | |
| 93493\_at | Ddx5 | DEAD (Asp-Glu-Ala-Asp) box polypeptide 5 | chr11 | -1.312 | 0 | 0.469 |  | | | |
| 93498\_s\_at | Aplp2 | amyloid beta (A4) precursor-like protein 2 | chr9 | -2.613 | 0 | 0.02 |  | | | |
| 93528\_s\_at | Bteb1 | basic transcription element binding protein 1 | chr19 | -3.735 | 0 | 0.838 |  | | | |
| 93666\_at | Lmo2 | LIM domain only 2 | chr2 | -1.002 | 0 | 0.117 |  | | | |
| 93705\_at | Chrnb1 | cholinergic receptor, nicotinic, beta polypeptide 1 (muscle) | --- | -2.651 | 0 | 0.016 |  | | | |
| 93714\_f\_at | H2-Q7 | histocompatibility 2, Q region locus 7 | chr17 | -3.128 | 0 | 0.001 |  | | | |
| 93728\_at | Tgfb1i4 | transforming growth factor beta 1 induced transcript 4 | chr14 | -3.15 | 0 | 0.097 |  | | | |
| 93793\_at | Lasp1 | LIM and SH3 protein 1 | chr11 | -1.092 | 0 | 0.786 |  | | | |
| 93907\_f\_at | NoneAvailable | --- | --- | -1.754 | 0 | 0.001 |  | | | |
| 93964\_s\_at | Ddx6 | DEAD (Asp-Glu-Ala-Asp) box polypeptide 6 | chr9 | -3.009 | 0 | 0.03 |  | | | |
| 93997\_at | Ifrg15-pending | interferon alpha responsive gene | chr1 | -1.408 | 0 | 0.877 |  | | | |
| 94004\_at | Cnn2 | calponin 2 | chr10 | -1.454 | 0 | 0.213 |  | | | |
| 94031\_at | Rab2 | RAB2, member RAS oncogene family | chr4 | -1.126 | 0 | 0.819 |  | | | |
| 94109\_at | LOC226442 | hypothetical protein LOC226442 | chr1 | -1.228 | 0 | 0.068 |  | | | |
| 94286\_at | 9130011J15Rik | RIKEN cDNA 9130011J15 gene | chr8 | -1.82 | 0 | 0.233 |  | | | |
| 94345\_at | Il6st | interleukin 6 signal transducer | chr13 | -3.717 | 0 | 0 |  | | | |
| 94359\_at | AA960558 | expressed sequence AA960558 | chr7 | -1.173 | 0 | 0.866 |  | | | |
| 94428\_at | Ilvbl | ilvB (bacterial acetolactate synthase)-like | chr10 | -1.305 | 0 | 0.004 |  | | | |
| 94448\_at | Bcl10 | B-cell leukemia/lymphoma 10 | chr3 | -1.934 | 0 | 0.355 |  | | | |
| 94454\_at | Dazap2 | DAZ associated protein 2 | chr15 | -1.095 | 0 | 0.069 |  | | | |
| 94460\_at | Stk38 | serine/threonine kinase 38 | chr17 | -1.608 | 0 | 0.632 |  | | | |
| 94461\_at | Pbef-pending | pre-B-cell colony-enhancing factor | chr12 | -1.477 | 0 | 0.103 |  | | | |
| 94469\_at | 2410018D16Rik | RIKEN cDNA 2410018D16 gene | chr11 | -1.032 | 0 | 0.79 |  | | | |
| 94489\_at | Ptp4a1 | protein tyrosine phosphatase 4a1 | chr1 | -1.816 | 0 | 0.749 |  | | | |
| 94499\_at | Mgea5 | meningioma expressed antigen 5 (hyaluronidase) | chr19 | -1.029 | 0 | 0.213 |  | | | |
| 94834\_at | Ctsh | cathepsin H | chr9 | -1.968 | 0 | 0.045 |  | | | |
| 94882\_at | 2010200I23Rik | RIKEN cDNA 2010200I23 gene | chr9 | -1.775 | 0 | 0.415 |  | | | |
| 94898\_at | 2010004P11Rik | RIKEN cDNA 2010004P11 gene | chr2 | -1.56 | 0 | 0.776 |  | | | |
| 94948\_at | Trip6 | thyroid hormone receptor interactor 6 | chr5 | -1.016 | 0 | 0.02 |  | | | |
| 94991\_at | Synpo | synaptopodin | chr18 | -1.276 | 0 | 0.018 |  | | | |
| 95057\_at | Herpud1 | homocysteine-inducible, endoplasmic reticulum stress-inducible, ubiquitin-like domain member 1 | chr8 | -2.784 | 0 | 0.608 |  | | | |
| 95062\_at | Cast | calpastatin | chr13 | -1.651 | 0 | 0.222 |  | | | |
| 95070\_at | Nars | asparaginyl-tRNA synthetase | chr18 | -1.983 | 0 | 0.952 |  | | | |
| 95101\_at | Tde2 | tumor differentially expressed 2 | chr10 | -1.249 | 0 | 0.769 |  | | | |
| 95102\_at | Scotin-pending | scotin gene | chr9 | -2.721 | 0 | 0.006 |  | | | |
| 95135\_at | 3110038L01Rik | RIKEN cDNA 3110038L01 gene | chrX | -1.623 | 0 | 0.857 |  | | | |
| 95146\_at | 1810045K07Rik | RIKEN cDNA 1810045K07 gene | chr8 | -3.602 | 0 | 0.365 |  | | | |
| 95360\_at | 6030448M23Rik | RIKEN cDNA 6030448M23 gene | --- | -2.433 | 0 | 0.694 |  | | | |
| 95440\_at | 2810432L12Rik | RIKEN cDNA 2810432L12 gene | chr4 | -1.235 | 0 | 0.164 |  | | | |
| 95449\_at | 2310075G12Rik | RIKEN cDNA 2310075G12 gene | chr11 | -1.383 | 0 | 0.016 |  | | | |
| 95520\_at | 2310061B02Rik | RIKEN cDNA 2310061B02 gene | chr1 | -2.378 | 0 | 0.04 |  | | | |
| 95673\_s\_at | Basp1 | brain abundant, membrane attached signal protein 1 | chr15 | -1.426 | 0 | 0.851 |  | | | |
| 95955\_at | NoneAvailable | Mus musculus adult male urinary bladder cDNA, RIKEN full-length enriched library, clone:9530006N05 product:unknown EST, full insert sequence | --- | -1.923 | 0 | 0.261 |  | | | |
| 96049\_at | Bgn | biglycan | --- | -1.681 | 0 | 0.008 |  | | | |
| 96079\_at | 0610010K06Rik | RIKEN cDNA 0610010K06 gene | chr6 | -1.137 | 0 | 0.139 |  | | | |
| 96120\_at | Dnajb6 | DnaJ (Hsp40) homolog, subfamily B, member 6 | chr12 | -2.15 | 0 | 0.685 |  | | | |
| 96165\_at | 4732477C12Rik | RIKEN cDNA 4732477C12 gene | chr1 | -1.011 | 0 | 0.505 |  | | | |
| 96186\_at | Lrp10 | low-density lipoprotein receptor-related protein 10 | chr14 | -3.157 | 0 | 0.013 |  | | | |
| 96238\_at | Rab11a | RAB11a, member RAS oncogene family | chr9 | -1.896 | 0 | 0.595 |  | | | |
| 96255\_at | Bnip3l | BCL2/adenovirus E1B 19kDa-interacting protein 3-like | chr14 | -1.839 | 0 | 0.72 |  | | | |
| 96278\_at | 1110020C13Rik | RIKEN cDNA 1110020C13 gene | chr15 | -1.51 | 0 | 0.369 |  | | | |
| 96299\_at | 2900097C17Rik | RIKEN cDNA 2900097C17 gene | --- | -1.146 | 0 | 0.731 |  | | | |
| 96325\_at | 2510039O18Rik | RIKEN cDNA 2510039O18 gene | chr4 | -1.218 | 0 | 0.335 |  | | | |
| 96340\_at | B230114J08Rik | RIKEN cDNA B230114J08 gene | --- | -2.264 | 0 | 0.215 |  | | | |
| 96530\_at | NoneAvailable | Mus musculus transcribed sequences | --- | -1.094 | 0 | 0.002 |  | | | |
| 96609\_at | 2610019N13Rik | RIKEN cDNA 2610019N13 gene | chr3 | -1.054 | 0 | 0.858 |  | | | |
| 96657\_at | Sat1 | spermidine/spermine N1-acetyl transferase 1 | chr5 | -2.345 | 0 | 0.101 |  | | | |
| 96810\_at | Lmo2 | LIM domain only 2 | chr2 | -1.938 | 0 | 0.076 |  | | | |
| 96852\_at | Prkar1a | protein kinase, cAMP dependent regulatory, type I, alpha | chr11 | -1.528 | 0 | 0.532 |  | | | |
| 96874\_g\_at | D6Ertd772e | DNA segment, Chr 6, ERATO Doi 772, expressed | chr6 | -1.501 | 0 | 0.095 |  | | | |
| 96886\_at | Stab1 | stabilin 1 | chr14 | -2.451 | 0 | 0.017 |  | | | |
| 96912\_s\_at | Ctla2a | cytotoxic T lymphocyte-associated protein 2 alpha | chr13 | -3.703 | 0 | 0.031 |  | | | |
| 97181\_f\_at | NoneAvailable | --- | --- | -1.659 | 0 | 0.002 |  | | | |
| 97203\_at | Mlp | MARCKS-like protein | chr4 | -1.185 | 0 | 0.531 |  | | | |
| 97211\_at | 3230401N03Rik | RIKEN cDNA 3230401N03 gene | chrX | -1.874 | 0 | 0.059 |  | | | |
| 97312\_at | Cd164 | CD164 antigen | chr10 | -1.028 | 0 | 0.208 |  | | | |
| 97336\_at | Ctsf | cathepsin F | chr19 | -1.534 | 0 | 0.002 |  | | | |
| 97355\_at | AW050020 | expressed sequence AW050020 | chr11 | -1.022 | 0 | 0.145 |  | | | |
| 97448\_at | NoneAvailable | Mus musculus cDNA clone MGC:65558 IMAGE:6485174, complete cds | chr11 | -1.982 | 0 | 0.019 |  | | | |
| 97458\_at | Gnb1 | guanine nucleotide binding protein, beta 1 | --- | -1.081 | 0 | 0.292 |  | | | |
| 97504\_at | Ccnd2 | cyclin D2 | chr6 | -1.325 | 0 | 0.162 |  | | | |
| 97740\_at | Dusp16 | dual specificity phosphatase 16 | chr6 | -2.544 | 0 | 0.548 |  | | | |
| 97798\_at | 4930504E06Rik | RIKEN cDNA 4930504E06 gene | chr3 | -1.169 | 0 | 0.001 |  | | | |
| 97859\_at | LOC212111 | hypothetical protein LOC212111 | chr7 | -1.348 | 0 | 0.455 |  | | | |
| 97915\_at | Plekhb2 | pleckstrin homology domain containing, family B (evectins) member 2 | chr1 | -1.257 | 0 | 0.05 |  | | | |
| 98011\_at | Gabbr1 | gamma-aminobutyric acid (GABA-B) receptor, 1 | --- | -3.575 | 0 | 0.272 |  | | | |
| 98055\_at | Blcap | bladder cancer associated protein homolog (human) | --- | -2.841 | 0 | 0.153 |  | | | |
| 98083\_at | Copeb | core promoter element binding protein | chr13 | -2.963 | 0 | 0 |  | | | |
| 98088\_at | Cd14 | CD14 antigen | --- | -3.218 | 0 | 0.018 |  | | | |
| 98254\_f\_at | NoneAvailable | --- | --- | -1.54 | 0 | 0.002 |  | | | |
| 98369\_f\_at | NoneAvailable | --- | --- | -1.265 | 0 | 0.002 |  | | | |
| 98427\_s\_at | Nfkb1 | nuclear factor of kappa light chain gene enhancer in B-cells 1, p105 | chr3 | -1.548 | 0 | 0.166 |  | | | |
| 98451\_at | Dnajb10 | DnaJ (Hsp40) homolog, subfamily B, member 10 | chr1 | -3.902 | 0 | 0.002 |  | | | |
| 98927\_at | Rab6 | RAB6, member RAS oncogene family | chr7 | -1.457 | 0 | 0.071 |  | | | |
| 98946\_at | Wsb1-pending | WD-40-repeat-containing protein with a SOCS box 1 | chr11 | -2.94 | 0 | 0.751 |  | | | |
| 98988\_at | Mail-pending | molecule possessing ankyrin-repeats induced by lipopolysaccharide | chr16 | -3.621 | 0 | 0.531 |  | | | |
| 99076\_at | Nr1d2 | nuclear receptor subfamily 1, group D, member 2 | chr14 | -2.902 | 0 | 0.121 |  | | | |
| 99358\_at | NoneAvailable | Mus musculus transcribed sequences | --- | -1.376 | 0 | 0.328 |  | | | |
| 99603\_g\_at | Tieg1 | TGFB inducible early growth response 1 | --- | -1.867 | 0 | 0.968 |  | | | |
| 99610\_at | Ss18 | synovial sarcoma translocation, Chromosome 18 | chr18 | -1.19 | 0 | 0.838 |  | | | |
| 99849\_at | NoneAvailable | --- | --- | -2.35 | 0 | 0.12 |  | | | |
| 100068\_at | Aldh1a1 | aldehyde dehydrogenase family 1, subfamily A1 | chr19 | -3.752 | 1 | 0.184 |  | | | |
| 100088\_at | Ppp1cb | protein phosphatase 1, catalytic subunit, beta isoform | chr5 | -1.671 | 1 | 0.186 |  | | | |
| 100138\_f\_at | Rbm14 | RNA binding motif protein 14 | chr19 | -1.11 | 1 | 0.181 |  | | | |
| 100151\_at | Tde1 | tumor differentially expressed 1 | chr2 | -1.338 | 1 | 0.799 |  | | | |
| 100154\_at | Tapbp | TAP binding protein | --- | -2.541 | 1 | 0.1 |  | | | |
| 100306\_at | 2700007P21Rik | RIKEN cDNA 2700007P21 gene | --- | -1.654 | 1 | 0.276 |  | | | |
| 100342\_i\_at | Tuba1 | tubulin, alpha 1 | chr15 | -2.172 | 1 | 0.05 |  | | | |
| 100499\_at | Stx3 | syntaxin 3 | chr19 | -3.612 | 1 | 0.155 |  | | | |
| 100523\_r\_at | Wbp5 | WW domain binding protein 5 | chrX | -1.129 | 1 | 0.334 |  | | | |
| 100587\_f\_at | 5730403B10Rik | RIKEN cDNA 5730403B10 gene | chr16 | -2.058 | 1 | 0.009 |  | | | |
| 100708\_at | H3f3b | H3 histone, family 3B | chr11 | -1.412 | 1 | 0.393 |  | | | |
| 100944\_at | NoneAvailable | Mus musculus transcribed sequences | --- | -3.522 | 1 | 0.158 |  | | | |
| 100998\_at | H2-Ab1 | histocompatibility 2, class II antigen A, beta 1 | chr17 | -1.116 | 1 | 0.026 |  | | | |
| 101000\_at | Oaz2 | ornithine decarboxylase antizyme 2 | chr3 | -1.996 | 1 | 0.138 |  | | | |
| 101123\_at | Itm2b | integral membrane protein 2B | chr14 | -1.825 | 1 | 0.203 |  | | | |
| 101465\_at | Stat1 | signal transducer and activator of transcription 1 | chr1 | -2.066 | 1 | 0.537 |  | | | |
| 101475\_at | Bmi1 | B lymphoma Mo-MLV insertion region 1 | chr2 | -1.056 | 1 | 0.363 |  | | | |
| 101490\_at | 1810010A06Rik | RIKEN cDNA 1810010A06 gene | --- | -2.706 | 1 | 0.541 |  | | | |
| 101568\_at | NoneAvailable | Mus musculus mRNA similar to proline synthetase co-transcribed (cDNA clone MGC:59396 IMAGE:6504579), complete cds | chr8 | -1.703 | 1 | 0.024 |  | | | |
| 101585\_at | Pgrmc1 | progesterone receptor membrane component 1 | chrX | -1.463 | 1 | 0.831 |  | | | |
| 101876\_s\_at | H2-T17 | histocompatibility 2, T region locus 17 | --- | -3.394 | 1 | 0.149 |  | | | |
| 101886\_f\_at | H2-D1 | histocompatibility 2, D region locus 1 | chr17 | -2.762 | 1 | 0.066 |  | | | |
| 101930\_at | Nfix | nuclear factor I/X | --- | -1.559 | 1 | 0.136 |  | | | |
| 101955\_at | Hspa5 | heat shock 70kD protein 5 (glucose-regulated protein) | chr2 | -1.156 | 1 | 0.413 |  | | | |
| 101963\_at | Ctsl | cathepsin L | chr13 | -2.894 | 1 | 0.022 |  | | | |
| 101971\_at | 2500002L14Rik | RIKEN cDNA 2500002L14 gene | --- | -1.663 | 1 | 0.005 |  | | | |
| 101990\_at | Ldh2 | lactate dehydrogenase 2, B chain | chr6 | -1.289 | 1 | 0.001 |  | | | |
| 102064\_at | Casp1 | caspase 1 | chr9 | -1.913 | 1 | 0.371 |  | | | |
| 102094\_f\_at | NoneAvailable | --- | --- | -1.908 | 1 | 0.051 |  | | | |
| 102125\_f\_at | NoneAvailable | --- | --- | -1.486 | 1 | 0.204 |  | | | |
| 102161\_f\_at | NoneAvailable | --- | --- | -2.38 | 1 | 0.136 |  | | | |
| 102292\_at | Gadd45a | growth arrest and DNA-damage-inducible 45 alpha | chr6 | -1.887 | 1 | 0.055 |  | | | |
| 102332\_at | Ulk1 | Unc-51 like kinase 1 (C. elegans) | chr5 | -2.225 | 1 | 0.009 |  | | | |
| 102384\_at | 2610209L14Rik | RIKEN cDNA 2610209L14 gene | chr19 | -4.341 | 1 | 0.225 |  | | | |
| 102644\_at | Kdt1 | kidney cell line derived transcript 1 | chr6 | -3.548 | 1 | 0.438 |  | | | |
| 102860\_at | Serpina3g | serine (or cysteine) proteinase inhibitor, clade A, member 3G | chr12 | -4.051 | 1 | 0.013 |  | | | |
| 102905\_at | Casp4 | caspase 4, apoptosis-related cysteine protease | --- | -2.328 | 1 | 0.52 |  | | | |
| 102906\_at | Tgtp | T-cell specific GTPase | chr11 | -5.973 | 1 | 0.001 |  | | | |
| 102960\_at | Rga | recombination activating gene 1 gene activation | chr3 | -1.165 | 1 | 0.049 |  | | | |
| 102984\_g\_at | Madh1 | MAD homolog 1 (Drosophila) | chr8 | -1.18 | 1 | 0.089 |  | | | |
| 103007\_at | Efna1 | ephrin A1 | chr3 | -1.146 | 1 | 0.172 |  | | | |
| 103035\_at | Tap1 | transporter 1, ATP-binding cassette, sub-family B (MDR/TAP) | chr17 | -2.277 | 1 | 0.303 |  | | | |
| 103202\_at | Gbp3 | guanylate nucleotide binding protein 3 | chr3 | -3.37 | 1 | 0.56 |  | | | |
| 103353\_f\_at | Cyp4b1 | cytochrome P450, family 4, subfamily b, polypeptide 1 | chr4 | -2.127 | 1 | 0.004 |  | | | |
| 103362\_at | Ptger4 | prostaglandin E receptor 4 (subtype EP4) | chr15 | -3.361 | 1 | 0.095 |  | | | |
| 103440\_at | Gabpa | GA repeat binding protein, alpha | chr16 | -1.107 | 1 | 0.803 |  | | | |
| 103497\_at | BC025546 | cDNA sequence BC025546 | chr8 | -1.103 | 1 | 0.059 |  | | | |
| 103501\_at | Pura | purine rich element binding protein A | --- | -3.373 | 1 | 0.247 |  | | | |
| 103634\_at | Isgf3g | interferon dependent positive acting transcription factor 3 gamma | --- | -1.108 | 1 | 0.002 |  | | | |
| 103658\_r\_at | 9230112N11Rik | RIKEN cDNA 9230112N11 gene | chr5 | -1.06 | 1 | 0.486 |  | | | |
| 103739\_at | 1110017N23Rik | RIKEN cDNA 1110017N23 gene | chr9 | -1.318 | 1 | 0.736 |  | | | |
| 103899\_at | Atp11a | ATPase, class VI, type 11A | chr8 | -1.465 | 1 | 0.049 |  | | | |
| 103913\_at | Sec61a2 | Sec61, alpha subunit 2 (S. cerevisiae) | chr2 | -1.013 | 1 | 0.733 |  | | | |
| 103916\_at | 8430420C20Rik | RIKEN cDNA 8430420C20 gene | chr7 | -3.057 | 1 | 0.172 |  | | | |
| 104019\_at | Ubl4 | ubiquitin-like 4 | chrX | -1.437 | 1 | 0.368 |  | | | |
| 104100\_at | 2310075E07Rik | RIKEN cDNA 2310075E07 gene | chr11 | -2.778 | 1 | 0.396 |  | | | |
| 104155\_f\_at | Atf3 | activating transcription factor 3 | chr1 | -4.531 | 1 | 0.142 |  | | | |
| 104186\_at | E130103E02Rik | RIKEN cDNA E130103E02 gene | chr9 | -1.247 | 1 | 0.626 |  | | | |
| 104477\_at | NoneAvailable | Mus musculus transcribed sequences | chr3 | -2.718 | 1 | 0.413 |  | | | |
| 104597\_at | Gbp2 | guanylate nucleotide binding protein 2 | chr3 | -2.148 | 1 | 0.021 |  | | | |
| 104618\_at | Rbbp9 | retinoblastoma binding protein 9 | --- | -1.148 | 1 | 0.898 |  | | | |
| 104694\_at | LOC224598 | similar to zinc finger protein 40 | --- | -1.587 | 1 | 0.274 |  | | | |
| 104735\_at | AW538430 | expressed sequence AW538430 | chr14 | -1.525 | 1 | 0.049 |  | | | |
| 104750\_at | Ifi47 | interferon gamma inducible protein | chr11 | -1.691 | 1 | 0.151 |  | | | |
| 160084\_at | Odc | ornithine decarboxylase, structural | chr12 | -1.134 | 1 | 0.332 |  | | | |
| 160092\_at | Ifrd1 | interferon-related developmental regulator 1 | chr12 | -2.637 | 1 | 0.369 |  | | | |
| 160112\_at | 1190006E07Rik | RIKEN cDNA 1190006E07 gene | chr16 | -1.604 | 1 | 0.652 |  | | | |
| 160127\_at | Ccng1 | cyclin G1 | chr11 | -1.675 | 1 | 0.007 |  | | | |
| 160138\_at | Mxi1 | Max interacting protein 1 | chr19 | -1.94 | 1 | 0.725 |  | | | |
| 160199\_at | Hnrpc | heterogeneous nuclear ribonucleoprotein C | chr14 | -1.233 | 1 | 0.466 |  | | | |
| 160205\_f\_at | Rnf11 | ring finger protein 11 | chr4 | -1.893 | 1 | 0.079 |  | | | |
| 160236\_at | 9630044O09Rik | RIKEN cDNA 9630044O09 gene | --- | -3.328 | 1 | 0.41 |  | | | |
| 160240\_at | 1110003E01Rik | RIKEN cDNA 1110003E01 gene | chr5 | -1.376 | 1 | 0.222 |  | | | |
| 160296\_at | Wsb2-pending | WD-40-repeat-containing protein with a SOCS box 2 | chr5 | -2.532 | 1 | 0.718 |  | | | |
| 160308\_at | Msn | moesin | chrX | -1.455 | 1 | 0.643 |  | | | |
| 160338\_at | 1100001D10Rik | RIKEN cDNA 1100001D10 gene | chr5 | -1.249 | 1 | 0.297 |  | | | |
| 160373\_i\_at | Sdpr | serum deprivation response | chr1 | -1.337 | 1 | 0.228 |  | | | |
| 160403\_at | Selk-pending | selenoprotein K | chr14 | -1.434 | 1 | 0.133 |  | | | |
| 160434\_at | 2310004K06Rik | RIKEN cDNA 2310004K06 gene | chr7 | -1.365 | 1 | 0.317 |  | | | |
| 160464\_s\_at | Ndr1 | N-myc downstream regulated 1 | chr15 | -4.394 | 1 | 0.054 |  | | | |
| 160493\_at | Cd63 | Cd63 antigen | chr18 | -2.846 | 1 | 0.078 |  | | | |
| 160502\_at | Creg | cellular repressor of E1A-stimulated genes | --- | -2.676 | 1 | 0.03 |  | | | |
| 160519\_at | Timp3 | tissue inhibitor of metalloproteinase 3 | chr10 | -3.836 | 1 | 0.007 |  | | | |
| 160520\_at | Yap | yes-associated protein | --- | -1.51 | 1 | 0.526 |  | | | |
| 160530\_at | Ghitm | growth hormone inducible transmembrane protein | chr14 | -1.065 | 1 | 0.747 |  | | | |
| 160611\_at | Cyp4v3 | cytochrome P450, family 4, subfamily v, polypeptide 3 | chr8 | -1.958 | 1 | 0.096 |  | | | |
| 160676\_at | NoneAvailable | Mus musculus, clone IMAGE:2647796, mRNA | chr16 | -1.374 | 1 | 0.053 |  | | | |
| 160715\_at | AW146242 | expressed sequence AW146242 | chr6 | -1.781 | 1 | 0.488 |  | | | |
| 160724\_at | Usp49 | ubiquitin specific protease 49 | --- | -2.112 | 1 | 0 |  | | | |
| 160783\_at | D14Ertd436e | DNA segment, Chr 14, ERATO Doi 436, expressed | chr14 | -1.857 | 1 | 0.033 |  | | | |
| 160933\_at | Igtp | interferon gamma induced GTPase | chr11 | -3.814 | 1 | 0 |  | | | |
| 161026\_s\_at | Sytl4 | synaptotagmin-like 4 | chrX | -1.66 | 1 | 0.629 |  | | | |
| 161036\_at | AA536730 | expressed sequence AA536730 | chr10 | -1.272 | 1 | 0.305 |  | | | |
| 161281\_f\_at | NoneAvailable | --- | --- | -2.519 | 1 | 0.517 |  | | | |
| 161666\_f\_at | Gadd45b | growth arrest and DNA-damage-inducible 45 beta | chr10 | -2.691 | 1 | 0.042 |  | | | |
| 162044\_f\_at | Cyp4b1 | cytochrome P450, family 4, subfamily b, polypeptide 1 | --- | -2.532 | 1 | 0.007 |  | | | |
| 162420\_r\_at | NoneAvailable | --- | --- | -1.492 | 1 | 0.614 |  | | | |
| 92263\_at | Grcb | gene rich cluster, B gene | chr6 | -1.939 | 1 | 0.002 |  | | | |
| 92440\_at | Irf6 | interferon regulatory factor 6 | chr1 | -4.326 | 1 | 0.031 |  | | | |
| 92559\_at | Vcam1 | vascular cell adhesion molecule 1 | chr3 | -1.63 | 1 | 0.057 |  | | | |
| 92564\_at | Lrrfip1 | leucine rich repeat (in FLII) interacting protein 1 | chr1 | -2.362 | 1 | 0.597 |  | | | |
| 92579\_at | Ssb | Sjogren syndrome antigen B | chr2 | -1.238 | 1 | 0.078 |  | | | |
| 92586\_at | Glud | glutamate dehydrogenase | chr14 | -1.053 | 1 | 0.146 |  | | | |
| 92648\_at | Stxbp3 | syntaxin binding protein 3 | chr3 | -1.895 | 1 | 0.642 |  | | | |
| 92653\_at | D530037H12Rik | RIKEN cDNA D530037H12 gene | chr1 | -1.338 | 1 | 0.01 |  | | | |
| 92780\_f\_at | NoneAvailable | --- | --- | -2.077 | 1 | 0.017 |  | | | |
| 92847\_s\_at | M6pr | mannose-6-phosphate receptor, cation dependent | chr6 | -1.096 | 1 | 0.043 |  | | | |
| 92855\_at | Sui1-rs1 | suppressor of initiator codon mutations, related sequence 1 (S. cerevisiae) | chr11 | -1.235 | 1 | 0.155 |  | | | |
| 92866\_at | H2-Aa | histocompatibility 2, class II antigen A, alpha | chr17 | -3.869 | 1 | 0.036 |  | | | |
| 92926\_at | Mpl | myeloproliferative leukemia virus oncogene | --- | -1.119 | 1 | 0.003 |  | | | |
| 93011\_at | Gabarapl1 | gamma-aminobutyric acid (GABA(A)) receptor-associated protein-like 1 | chr6 | -2.046 | 1 | 0.019 |  | | | |
| 93017\_at | Sdcbp | syndecan binding protein | chr4 | -1.96 | 1 | 0.582 |  | | | |
| 93020\_at | Rex3 | reduced expression 3 | chrX | -1.019 | 1 | 0.009 |  | | | |
| 93039\_at | 1190003P12Rik | RIKEN cDNA 1190003P12 gene | chr15 | -1.465 | 1 | 0.029 |  | | | |
| 93043\_at | Sdfr1 | stromal cell derived factor receptor 1 | chr9 | -1.79 | 1 | 0.712 |  | | | |
| 93063\_at | App | amyloid beta (A4) precursor protein | chr16 | -2.104 | 1 | 0.371 |  | | | |
| 93088\_at | B2m | beta-2 microglobulin | chr2 | -1.669 | 1 | 0.553 |  | | | |
| 93187\_at | 2210023F24Rik | RIKEN cDNA 2210023F24 gene | chr11 | -1.565 | 1 | 0.113 |  | | | |
| 93281\_at | Rcn2 | reticulocalbin 2 | chr9 | -1.366 | 1 | 0.848 |  | | | |
| 93285\_at | Dusp6 | dual specificity phosphatase 6 | chr10 | -3.965 | 1 | 0.236 |  | | | |
| 93316\_at | Osbpl1a | oxysterol binding protein-like 1A | chr18 | -2.393 | 1 | 0.804 |  | | | |
| 93324\_at | Zfp36l1 | zinc finger protein 36, C3H type-like 1 | chr12 | -2.308 | 1 | 0.025 |  | | | |
| 93511\_at | Itm2a | integral membrane protein 2A | chrX | -2.937 | 1 | 0.259 |  | | | |
| 93543\_f\_at | Gstm1 | glutathione S-transferase, mu 1 | chr5 | -1.363 | 1 | 0.008 |  | | | |
| 93588\_at | Gtl3 | gene trap locus 3 | chr8 | -1.315 | 1 | 0.337 |  | | | |
| 93626\_at | Abcg2 | ATP-binding cassette, sub-family G (WHITE), member 2 | chr6 | -1.979 | 1 | 0.053 |  | | | |
| 93729\_at | Mtap7 | microtubule-associated protein 7 | chr10 | -1.028 | 1 | 0.16 |  | | | |
| 93731\_at | Fkbp9 | FK506 binding protein 9 | chr6 | -2.146 | 1 | 0.169 |  | | | |
| 93865\_s\_at | H2-T10 | histocompatibility 2, T region locus 10 | chr17 | -3.212 | 1 | 0.133 |  | | | |
| 94041\_at | Hnrpk | heterogeneous nuclear ribonucleoprotein K | chr13 | -1.33 | 1 | 0.81 |  | | | |
| 94043\_at | Atp6ap1 | ATPase, H+ transporting, lysosomal accessory protein 1 | chrX | -1.107 | 1 | 0.219 |  | | | |
| 94085\_at | Prg | proteoglycan, secretory granule | --- | -2.082 | 1 | 0.521 |  | | | |
| 94269\_at | Rabac1 | Rab acceptor 1 (prenylated) | chr7 | -1.557 | 1 | 0.015 |  | | | |
| 94270\_at | Krt1-18 | keratin complex 1, acidic, gene 18 | --- | -3.531 | 1 | 0.013 |  | | | |
| 94330\_at | Npl | N-acetylneuraminate pyruvate lyase | chr1 | -1.211 | 1 | 0.235 |  | | | |
| 94383\_at | Tfpi2 | tissue factor pathway inhibitor 2 | chr6 | -1.154 | 1 | 0.094 |  | | | |
| 94384\_at | Ier3 | immediate early response 3 | chr17 | -1.292 | 1 | 0.785 |  | | | |
| 94473\_at | 1810010L20Rik | RIKEN cDNA 1810010L20 gene | chr10 | -2.207 | 1 | 0.096 |  | | | |
| 94505\_at | Peli1 | pellino 1 | chr11 | -1.62 | 1 | 0.412 |  | | | |
| 94537\_at | 1500001M02Rik | RIKEN cDNA 1500001M02 gene | chr17 | -1.32 | 1 | 0.793 |  | | | |
| 94815\_at | Bpgm | 2,3-bisphosphoglycerate mutase | chr6 | -2.101 | 1 | 0.311 |  | | | |
| 94817\_at | Serpinh1 | serine (or cysteine) proteinase inhibitor, clade H, member 1 | chr7 | -1.251 | 1 | 0.289 |  | | | |
| 94821\_at | Xbp1 | X-box binding protein 1 | --- | -2.107 | 1 | 0.021 |  | | | |
| 94832\_at | Hnrph2 | heterogeneous nuclear ribonucleoprotein H2 | chrX | -1.589 | 1 | 0.701 |  | | | |
| 94835\_f\_at | Tubb2 | tubulin, beta 2 | chr13 | -2.52 | 1 | 0.002 |  | | | |
| 94839\_at | Nucb | nucleobindin | chr7 | -1.601 | 1 | 0.105 |  | | | |
| 94881\_at | Cdkn1a | cyclin-dependent kinase inhibitor 1A (P21) | chr17 | -2.727 | 1 | 0.002 |  | | | |
| 95348\_at | Cxcl1 | chemokine (C-X-C motif) ligand 1 | chr5 | -1.203 | 1 | 0.366 |  | | | |
| 95377\_at | NoneAvailable | Mus musculus transcribed sequence with strong similarity to protein sp:Q92914 (H.sapiens) FGFB\_HUMAN Fibroblast growth factor-11 (FGF-11) (Fibroblast growth factor homologous factor 3) (FHF-3) | chr11 | -1.775 | 1 | 0.416 |  | | | |
| 95395\_at | 9130022A11Rik | RIKEN cDNA 9130022A11 gene | chr3 | -1.643 | 1 | 0.286 |  | | | |
| 95412\_at | Pdcd6 | programmed cell death 6 | chr13 | -1.094 | 1 | 0.412 |  | | | |
| 95505\_at | Tor1b | torsin family 1, member B | --- | -1.061 | 1 | 0.039 |  | | | |
| 95508\_at | Nckap1 | NCK-associated protein 1 | chr2 | -4.402 | 1 | 0.021 |  | | | |
| 95522\_i\_at | Zfp68 | Zinc finger protein 68 | --- | -2.21 | 1 | 0.054 |  | | | |
| 95617\_at | Rbl2 | retinoblastoma-like 2 | chr8 | -1.456 | 1 | 0.316 |  | | | |
| 95737\_at | 1200015A19Rik | RIKEN cDNA 1200015A19 gene | chr4 | -2.325 | 1 | 0.023 |  | | | |
| 95743\_at | 2310050K10Rik | RIKEN cDNA 2310050K10 gene | chr18 | -1.586 | 1 | 0.277 |  | | | |
| 96146\_at | Btg3 | B-cell translocation gene 3 | chr16 | -2.704 | 1 | 0.035 |  | | | |
| 96252\_at | Pdcd6ip | programmed cell death 6 interacting protein | chr9 | -1.212 | 1 | 0.279 |  | | | |
| 96283\_at | Itm2c | integral membrane protein 2C | chr1 | -1.109 | 1 | 0.05 |  | | | |
| 96341\_at | Gcipip-pending | GCIP-interacting protein p29 | chr4 | -1.216 | 1 | 0.123 |  | | | |
| 96596\_at | Ndrl | N-myc downstream regulated-like | chr15 | -4.908 | 1 | 0.007 |  | | | |
| 96614\_at | 4933426M11Rik | RIKEN cDNA 4933426M11 gene | chr12 | -1.2 | 1 | 0.039 |  | | | |
| 96633\_s\_at | Morf4l2 | mortality factor 4 like 2 | chr4 | -1.355 | 1 | 0.14 |  | | | |
| 96680\_at | Dnajb9 | DnaJ (Hsp40) homolog, subfamily B, member 9 | chr12 | -3.316 | 1 | 0.179 |  | | | |
| 96688\_at | 2610318G18Rik | RIKEN cDNA 2610318G18 gene | --- | -1.02 | 1 | 0.194 |  | | | |
| 96703\_at | Maged1 | melanoma antigen, family D, 1 | chrX | -2.002 | 1 | 0.003 |  | | | |
| 96728\_at | DXImx38e | DNA segment, Chr X, Immunex 38, expressed | chrX | -2.856 | 1 | 0.001 |  | | | |
| 96736\_at | 9430020K16Rik | RIKEN cDNA 9430020K16 gene | chr16 | -1.075 | 1 | 0.065 |  | | | |
| 96752\_at | Icam1 | intercellular adhesion molecule | chr9 | -2.045 | 1 | 0.024 |  | | | |
| 96764\_at | Iigp-pending | interferon-inducible GTPase | --- | -5.077 | 1 | 0.009 |  | | | |
| 96876\_at | Laptm4a | lysosomal-associated protein transmembrane 4A | chr12 | -1.037 | 1 | 0.039 |  | | | |
| 96935\_at | 2700030M23Rik | RIKEN cDNA 2700030M23 gene | chr4 | -2.968 | 1 | 0.003 |  | | | |
| 97104\_g\_at | 0610038L10Rik | RIKEN cDNA 0610038L10 gene | chr19 | -1.236 | 1 | 0.877 |  | | | |
| 97409\_at | Ifi1 | interferon inducible protein 1 | chr11 | -1.031 | 1 | 0.034 |  | | | |
| 97451\_at | Mcfd2 | multiple coagulation factor deficiency 2 | chr17 | -1.202 | 1 | 0.094 |  | | | |
| 97540\_f\_at | H2-D1 | histocompatibility 2, D region locus 1 | --- | -3.787 | 1 | 0.002 |  | | | |
| 97549\_at | Cfl2 | cofilin 2, muscle | chr12 | -2.749 | 1 | 0.03 |  | | | |
| 97825\_at | Perp-pending | p53 apoptosis effector related to Pmp22 | chr10 | -3.39 | 1 | 0.062 |  | | | |
| 97834\_g\_at | Pfkp | phosphofructokinase, platelet | chr13 | -1.277 | 1 | 0.329 |  | | | |
| 97890\_at | Sgk | serum/glucocorticoid regulated kinase | chr10 | -3.498 | 1 | 0.048 |  | | | |
| 97908\_at | 1110007A06Rik | RIKEN cDNA 1110007A06 gene | chr6 | -1.119 | 1 | 0.04 |  | | | |
| 97949\_at | Fgl2 | fibrinogen-like protein 2 | chr5 | -1.99 | 1 | 0.085 |  | | | |
| 97972\_at | Rnf103 | ring finger protein 103 | chr6 | -3.704 | 1 | 0.488 |  | | | |
| 97973\_at | Tal1 | T-cell acute lymphocytic leukemia 1 | chr4 | -1.383 | 1 | 0.032 |  | | | |
| 98067\_at | Cdkn1a | cyclin-dependent kinase inhibitor 1A (P21) | chr17 | -2.741 | 1 | 0.001 |  | | | |
| 98410\_at | Gtpi-pending | interferon-g induced GTPase | chr11 | -2.836 | 1 | 0.046 |  | | | |
| 98472\_at | H2-T23 | histocompatibility 2, T region locus 23 | --- | -1.61 | 1 | 0 |  | | | |
| 98508\_s\_at | Ppap2a | phosphatidic acid phosphatase 2a | chr13 | -1.267 | 1 | 0.512 |  | | | |
| 99032\_at | Rasd1 | RAS, dexamethasone-induced 1 | chr11 | -3.559 | 1 | 0.365 |  | | | |
| 99109\_at | Ier2 | immediate early response 2 | chr8 | -2.892 | 1 | 0.003 |  | | | |
| 99133\_at | Slc3a2 | solute carrier family 3 (activators of dibasic and neutral amino acid transport), member 2 | chr19 | -1.113 | 1 | 0.018 |  | | | |
| 99366\_at | E030024M05Rik | RIKEN cDNA E030024M05 gene | chr12 | -2.996 | 1 | 0.014 |  | | | |
| 99532\_at | Tob1 | transducer of ErbB-2.1 | chr11 | -5.048 | 1 | 0.008 |  | | | |
| 99602\_at | Tieg1 | TGFB inducible early growth response 1 | chr15 | -2.141 | 1 | 0.807 |  | | | |
| 99622\_at | Klf4 | Kruppel-like factor 4 (gut) | chr4 | -4.599 | 1 | 0.057 |  | | | |
| 99959\_at | Ak4 | adenylate kinase 4 | chr4 | -2.208 | 1 | 0.586 |  | | | |
| 100024\_at | Shrm | shroom | chr5 | -1.937 | 2 | 0.086 |  | | | |
| 100959\_at | S100a13 | S100 calcium binding protein A13 | chr3 | -1.165 | 2 | 0.756 |  | | | |
| 101015\_s\_at | Ifnar2 | interferon (alpha and beta) receptor 2 | chr16 | -1.038 | 2 | 0.802 |  | | | |
| 101023\_f\_at | 0610010E21Rik | RIKEN cDNA 0610010E21 gene | chr7 | -1.262 | 2 | 0.51 |  | | | |
| 103223\_at | 5830406C15Rik | RIKEN cDNA 5830406C15 gene | chr17 | -1.812 | 2 | 0.959 |  | | | |
| 103460\_at | 5830413E08Rik | RIKEN cDNA 5830413E08 gene | chr10 | -6.025 | 2 | 0.747 |  | | | |
| 104149\_at | Nfkbia | nuclear factor of kappa light chain gene enhancer in B-cells inhibitor, alpha | chr12 | -2.483 | 2 | 0.271 |  | | | |
| 104337\_f\_at | 1200008D14Rik | RIKEN cDNA 1200008D14 gene | chr16 | -3.492 | 2 | 0.085 |  | | | |
| 160065\_s\_at | Csrp1 | cysteine and glycine-rich protein 1 | chr1 | -1.548 | 2 | 0.472 |  | | | |
| 160359\_at | 1190002H23Rik | RIKEN cDNA 1190002H23 gene | chr14 | -1.318 | 2 | 0.095 |  | | | |
| 162138\_s\_at | Nptxr | neuronal pentraxin receptor | chr15 | -1.433 | 2 | 0.058 |  | | | |
| 93874\_s\_at | Il11ra2 | interleukin 11 receptor, alpha chain 2 | --- | -1.619 | 2 | 0.081 |  | | | |
| 94445\_at | Pls3 | plastin 3 (T-isoform) | chrX | -1.992 | 2 | 0.521 |  | | | |
| 94820\_r\_at | Ccni | cyclin I | chr14 | -1.432 | 2 | 0.952 |  | | | |
| 94917\_at | Fbxo8 | F-box only protein 8 | chr8 | -1.235 | 2 | 0.253 |  | | | |
| 95418\_at | 1190017B18Rik | RIKEN cDNA 1190017B18 gene | chr5 | -2.563 | 2 | 0.159 |  | | | |
| 95430\_f\_at | D9Wsu18e | DNA segment, Chr 9, Wayne State University 18, expressed | chr9 | -1.061 | 2 | 0.119 |  | | | |
| 95453\_f\_at | S100a1 | S100 calcium binding protein A1 | chr3 | -2.675 | 2 | 0.933 |  | | | |
| 95661\_at | Cd9 | CD9 antigen | chr6 | -2.1 | 2 | 0.426 |  | | | |
| 95681\_f\_at | Ppp1r2 | protein phosphatase 1, regulatory (inhibitor) subunit 2 | chr16 | -1.034 | 2 | 0.257 |  | | | |
| 96060\_at | Serpinb6a | serine (or cysteine) proteinase inhibitor, clade B, member 6a | chr13 | -1.432 | 2 | 0.082 |  | | | |
| 96779\_f\_at | 2410022L05Rik | RIKEN cDNA 2410022L05 gene | chr8 | -1.408 | 2 | 0.125 |  | | | |
| 97496\_f\_at | 6330514M23Rik | RIKEN cDNA 6330514M23 gene | chr7 | -1.202 | 2 | 0.002 |  | | | |
| 97498\_at | Fhl1 | four and a half LIM domains 1 | chrX | -1.276 | 2 | 0.161 |  | | | |
| 98122\_at | Lmo4 | LIM domain only 4 | chr3 | -1.024 | 2 | 0.555 |  | | | |
| 98127\_at | Capza2 | capping protein (actin filament) muscle Z-line, alpha 2 | chr6 | -1.156 | 2 | 0.03 |  | | | |
| 99607\_at | Skp1a | S-phase kinase-associated protein 1A | chr11 | -1.159 | 2 | 0.254 |  | | | |
| 100307\_at | Nfix | nuclear factor I/X | chr8 | -1.498 | 3 | 0.272 |  | | | |
| 101001\_at | 5031439A09Rik | RIKEN cDNA 5031439A09 gene | chr3 | -1.852 | 3 | 0.044 |  | | | |
| 101578\_f\_at | Actb | actin, beta, cytoplasmic | --- | -1.214 | 3 | 0.46 |  | | | |
| 102752\_at | Shyc | selective hybridizing clone | chr7 | -1.071 | 3 | 0.161 |  | | | |
| 102957\_at | Lcp2 | lymphocyte cytosolic protein 2 | chr11 | -1.005 | 3 | 0.444 |  | | | |
| 103021\_r\_at | Map3k1 | mitogen activated protein kinase kinase kinase 1 | --- | -1.094 | 3 | 0.205 |  | | | |
| 103086\_at | Hoxa5 | homeo box A5 | chr6 | -1.175 | 3 | 0.792 |  | | | |
| 104177\_at | Vig1-pending | viral hemorrhagic septicemia virus(VHSV) induced gene 1 | chr12 | -1.432 | 3 | 0.397 |  | | | |
| 160124\_r\_at | Atp6v1c1 | ATPase, H+ transporting, V1 subunit C, isoform 1 | chr15 | -1.186 | 3 | 0.983 |  | | | |
| 160263\_r\_at | Ndfip2 | Nedd4 family interacting protein 2 | chr14 | -2.124 | 3 | 0.35 |  | | | |
| 161270\_i\_at | LOC269796 | hypothetical protein LOC269796 | chr6 | -2.032 | 3 | 0.445 |  | | | |
| 92531\_at | 3100002M17Rik | RIKEN cDNA 3100002M17 gene | chr3 | -1.032 | 3 | 0.342 |  | | | |
| 93241\_r\_at | NoneAvailable | Mus musculus 11 days embryo gonad cDNA, RIKEN full-length enriched library, clone:7030422K17 product:unclassifiable, full insert sequence | chrX | -1.686 | 3 | 0.905 |  | | | |
| 93421\_at | Pftk1 | PFTAIRE protein kinase 1 | chr5 | -1.167 | 3 | 0.514 |  | | | |
| 93459\_s\_at | Fzd4 | frizzled homolog 4 (Drosophila) | chr7 | -2.581 | 3 | 0.006 |  | | | |
| 94556\_at | Snx10 | sorting nexin 10 | chr6 | -1.37 | 3 | 0.656 |  | | | |
| 95058\_f\_at | 2610205H19Rik | RIKEN cDNA 2610205H19 gene | chr1 | -1.256 | 3 | 0.202 |  | | | |
| 95474\_at | F2r | coagulation factor II (thrombin) receptor | chr13 | -2.462 | 3 | 0.643 |  | | | |
| 95944\_at | Dhx36 | DEAH (Asp-Glu-Ala-His) box polypeptide 36 | chr3 | -1.173 | 3 | 0.629 |  | | | |
| 96019\_at | Sypl | synaptophysin-like protein | chr12 | -1.41 | 3 | 0.558 |  | | | |
| 96836\_r\_at | Zfp161 | zinc finger protein 161 | chr17 | -1.443 | 3 | 0.35 |  | | | |
| 97111\_at | 4733401O11Rik | RIKEN cDNA 4733401O11 gene | --- | -1.064 | 3 | 0.067 |  | | | |
| 97136\_at | NoneAvailable | Mus musculus transcribed sequence with weak  similarity to protein ref:NP\_060132.2 (H.sapiens)  transient receptor potential cation channel, subfamily M, member 6 [Homo sapiens] | --- | -1.341 | 3 | 0.793 |  | | | |
| 97844\_at | Rgs2 | regulator of G-protein signaling 2 | chr1 | -2.954 | 3 | 0.368 |  | | | |
| 97918\_at | AA536743 | expressed sequence AA536743 | chr5 | -2.906 | 3 | 0.227 |  | | | |
| 98436\_s\_at | Casp3 | caspase 3, apoptosis related cysteine protease | chr8 | -3.026 | 3 | 0.283 |  | | | |
| 98468\_r\_at | Brd1 | bromodomain containing 1 | chr15 | -1.606 | 3 | 0.188 |  | | | |
| 98478\_at | Ccng2 | cyclin G2 | chr5 | -1.73 | 3 | 0.576 |  | | | |
| 98968\_at | Myo5a | myosin Va | chr9 | -1.509 | 3 | 0.501 |  | | | |
| 99475\_at | Socs2 | suppressor of cytokine signaling 2 | chr10 | -3.55 | 3 | 0.012 |  | | | |
| 99500\_at | Slc12a2 | solute carrier family 12, member 2 | chr18 | -1.592 | 3 | 0.91 |  | | | |
| 100595\_at | Ptp4a2 | protein tyrosine phosphatase 4a2 | --- | -1.053 | 6 | 0.735 |  | | | |
| 104202\_at | NoneAvailable | Mus musculus, Similar to transmembrane protein 1, clone IMAGE:3989239, mRNA, partial cds | chr10 | -1.058 | 6 | 0.842 |  | | | |
| 104316\_at | NoneAvailable | Mus musculus, clone IMAGE:1379624, mRNA, partial cds | chr11 | -1.46 | 6 | 0.394 |  | | | |
| 160579\_at | Man1a | mannosidase 1, alpha | chr10 | -1.33 | 6 | 0.252 |  | | | |
| 160632\_at | Prkcn | protein kinase C, nu | chr17 | -1.104 | 6 | 0.975 |  | | | |
| 160795\_at | Scamp1 | secretory carrier membrane protein 1 | chr13 | -1.292 | 6 | 0.655 |  | | | |
| 92660\_f\_at | Ube2e1 | ubiquitin-conjugating enzyme E2E 1, UBC4/5 homolog (yeast) | chr14 | -1.11 | 6 | 0.328 |  | | | |
| 92770\_at | S100a6 | S100 calcium binding protein A6 (calcyclin) | chr3 | -3.204 | 6 | 0 |  | | | |
| 92778\_i\_at | NoneAvailable | --- | --- | -1.238 | 6 | 0.049 |  | | | |
| 93078\_at | Ly6a | lymphocyte antigen 6 complex, locus A | chr15 | -3.737 | 6 | 0.008 |  | | | |
| 93083\_at | Anxa5 | annexin A5 | chr3 | -1.415 | 6 | 0.083 |  | | | |
| 93320\_at | Cpt1a | carnitine palmitoyltransferase 1, liver | chr19 | -2.188 | 6 | 0.698 |  | | | |
| 93550\_at | Csrp2 | cysteine and glycine-rich protein 2 | chr10 | -1.277 | 6 | 0.591 |  | | | |
| 93775\_at | D12Ertd647e | DNA segment, Chr 12, ERATO Doi 647, expressed | chr12 | -1.118 | 6 | 0.067 |  | | | |
| 94256\_at | D0Jmb3 | DNA segment, Jeremy M. Boss 3 | chr3 | -1.929 | 6 | 0.876 |  | | | |
| 94346\_at | Wtap | Wilms' tumour 1-associating protein | chr17 | -1.134 | 6 | 0.982 |  | | | |
| 94408\_at | Nab1 | Ngfi-A binding protein 1 | --- | -1.046 | 6 | 0.093 |  | | | |
| 95425\_at | Acadl | acetyl-Coenzyme A dehydrogenase, long-chain | chr1 | -2.569 | 6 | 0.259 |  | | | |
| 95477\_at | 1110001M20Rik | RIKEN cDNA 1110001M20 gene | chr4 | -1.466 | 6 | 0.628 |  | | | |
| 95671\_at | Hey1 | hairy/enhancer-of-split related with YRPW motif 1 | chr3 | -1.012 | 6 | 0.269 |  | | | |
| 95695\_at | Slc25a20 | solute carrier family 25 (mitochondrial carnitine/acylcarnitine translocase), member 20 | chr9 | -1.141 | 6 | 0.29 |  | | | |
| 95701\_at | 4930415K17Rik | RIKEN cDNA 4930415K17 gene | chr18 | -1.759 | 6 | 0.211 |  | | | |
| 96271\_at | 2310075C12Rik | RIKEN cDNA 2310075C12 gene | chr9 | -1.203 | 6 | 0.523 |  | | | |
| 96603\_at | Qscn6 | quiescin Q6 | chr1 | -1.269 | 6 | 0.428 |  | | | |
| 96605\_at | 0610011I04Rik | RIKEN cDNA 0610011I04 gene | chr6 | -1.492 | 6 | 0.012 |  | | | |
| 96771\_at | Erbb3 | v-erb-b2 erythroblastic leukemia viral oncogene homolog 3 (avian) | --- | -2.18 | 6 | 0.228 |  | | | |
| 97519\_at | Spp1 | secreted phosphoprotein 1 | chr5 | -1.453 | 6 | 0.539 |  | | | |
| 97930\_f\_at | Cd151 | CD151 antigen | --- | -1.656 | 6 | 0.565 |  | | | |
| 98920\_g\_at | 2410018G23Rik | RIKEN cDNA 2410018G23 gene | chrX | -2.085 | 6 | 0.004 |  | | | |
| 98921\_at | 2410018G23Rik | RIKEN cDNA 2410018G23 gene | chr8 | -1.976 | 6 | 0.004 |  | | | |
| 99378\_f\_at | H2-Q1 | histocompatibility 2, Q region locus 1 | chr17 | -2.066 | 6 | 0.1 |  | | | |
| 99379\_f\_at | H2-K | histocompatibility 2, K region | chr17 | -1.443 | 6 | 0.077 |  | | | |
| 100011\_at | Klf3 | Kruppel-like factor 3 (basic) | chr5 | -1.992 | 10 | 0.399 |  | | | |
| 100030\_at | Upp1 | uridine phosphorylase 1 | chr11 | -1.097 | 10 | 0.005 |  | | | |
| 100032\_at | Sp1 | trans-acting transcription factor 1 | chr15 | -1.149 | 10 | 0.076 |  | | | |
| 100134\_at | Eng | endoglin | chr2 | -1.282 | 10 | 0.012 |  | | | |
| 100136\_at | Lamp2 | lysosomal membrane glycoprotein 2 | chrX | -1.749 | 10 | 0.025 |  | | | |
| 100297\_at | Wdr26 | WD repeat domain 26 | chr1 | -1.703 | 10 | 0.286 |  | | | |
| 100327\_at | NoneAvailable | --- | chr17 | -1.138 | 10 | 0.666 |  | | | |
| 100475\_at | Trim25 | tripartite motif protein 25 | --- | -1.645 | 10 | 0 |  | | | |
| 100514\_at | Gna13 | guanine nucleotide binding protein, alpha 13 | chr11 | -2.439 | 10 | 0.438 |  | | | |
| 100522\_s\_at | Wbp5 | WW domain binding protein 5 | --- | -1.397 | 10 | 0.503 |  | | | |
| 100561\_at | Iqgap1 | IQ motif containing GTPase activating protein 1 | chr7 | -1.033 | 10 | 0.841 |  | | | |
| 100606\_at | Prnp | prion protein | --- | -1.719 | 10 | 0.036 |  | | | |
| 100635\_at | Sara | SAR1a gene homolog (S. cerevisiae) | chr10 | -1.295 | 10 | 0.009 |  | | | |
| 100905\_at | 1700056O17Rik | RIKEN cDNA 1700056O17 gene | chr10 | -1.753 | 10 | 0.106 |  | | | |
| 100973\_i\_at | Ccl27 | chemokine (C-C motif) ligand 27 | --- | -3.092 | 10 | 0 |  | | | |
| 100988\_at | Bcl2l11 | BCL2-like 11 (apoptosis facilitator) | chr2 | -1.027 | 10 | 0.014 |  | | | |
| 100992\_at | Edr1 | early development regulator 1 (homolog of polyhomeotic 1) | chr6 | -1.634 | 10 | 0.117 |  | | | |
| 101079\_at | Nxf1 | nuclear RNA export factor 1 homolog (S. cerevisiae) | chr19 | -2.424 | 10 | 0.003 |  | | | |
| 101144\_at | Il18r1 | interleukin 18 receptor 1 | --- | -1.522 | 10 | 0.037 |  | | | |
| 101186\_at | Ppnr-pending | per-pentamer repeat gene | chr19 | -1.941 | 10 | 0.019 |  | | | |
| 101217\_at | BC023892 | cDNA sequence BC023892 | chr9 | -1.335 | 10 | 0.112 |  | | | |
| 101226\_at | NoneAvailable | Mus musculus transcribed sequences | chr8 | -1.89 | 10 | 0.151 |  | | | |
| 101441\_i\_at | Itpr5 | inositol 1,4,5-triphosphate receptor 5 | chr6 | -3.278 | 10 | 0.019 |  | | | |
| 101502\_at | Tgif | TG interacting factor | chr17 | -1.725 | 10 | 0.39 |  | | | |
| 101687\_r\_at | NoneAvailable | --- | --- | -1.273 | 10 | 0.167 |  | | | |
| 101700\_at | Phxr4 | per-hexamer repeat gene 4 | chr9 | -2.041 | 10 | 0.08 |  | | | |
| 101787\_f\_at | NoneAvailable | --- | --- | -1.24 | 10 | 0.064 |  | | | |
| 101836\_at | Ppm1b | protein phosphatase 1B, magnesium dependent, beta isoform | chr17 | -1.457 | 10 | 0.007 |  | | | |
| 101848\_g\_at | Sp100 | nuclear antigen Sp100 | chr1 | -1.234 | 10 | 0.359 |  | | | |
| 101884\_at | Xlr4 | X-linked lymphocyte-regulated 4 | chrX | -2.072 | 10 | 0.038 |  | | | |
| 101934\_at | Fez2 | fasciculation and elongation protein zeta 2 (zygin II) | chr17 | -1.364 | 10 | 0.41 |  | | | |
| 101936\_at | Clk4 | CDC like kinase 4 | chr11 | -1.28 | 10 | 0.136 |  | | | |
| 101943\_at | Tceb3 | transcription elongation factor B (SIII), polypeptide 3 | chr4 | -1.496 | 10 | 0.002 |  | | | |
| 101947\_at | Nakap95-pending | neighbor of A-kinase anchoring protein 95 | chr17 | -1.799 | 10 | 0.001 |  | | | |
| 101998\_at | 4833420G17Rik | RIKEN cDNA 4833420G17 gene | chr13 | -1.142 | 10 | 0.095 |  | | | |
| 102009\_at | Cyfip2 | cytoplasmic FMR1 interacting protein 2 | --- | -1.919 | 10 | 0.212 |  | | | |
| 102237\_at | Cd28 | CD28 antigen | chr1 | -1.282 | 10 | 0.093 |  | | | |
| 102279\_at | 1300004C08Rik | RIKEN cDNA 1300004C08 gene | chr9 | -2.168 | 10 | 0.008 |  | | | |
| 102313\_at | Gch | GTP cyclohydrolase 1 | chr14 | -4.039 | 10 | 0.005 |  | | | |
| 102321\_at | Adcy6 | adenylate cyclase 6 | chr15 | -1.276 | 10 | 0.052 |  | | | |
| 102322\_at | Ugdh | UDP-glucose dehydrogenase | chr5 | -1.169 | 10 | 0.416 |  | | | |
| 102360\_at | Mthfr | 5,10-methylenetetrahydrofolate reductase | --- | -2.959 | 10 | 0.117 |  | | | |
| 102372\_at | Igj | immunoglobulin joining chain | chr5 | -1.202 | 10 | 0.473 |  | | | |
| 102381\_at | Facl4 | fatty acid-Coenzyme A ligase, long chain 4 | chrX | -2.044 | 10 | 0.731 |  | | | |
| 102414\_i\_at | Dnajc3 | DnaJ (Hsp40) homolog, subfamily C, member 3 | chr14 | -1.165 | 10 | 0.868 |  | | | |
| 102415\_r\_at | Dnajc3 | DnaJ (Hsp40) homolog, subfamily C, member 3 | chr14 | -1.209 | 10 | 0.752 |  | | | |
| 102425\_at | Tle1 | transducin-like enhancer of split 1, homolog of Drosophila E(spl) | chr4 | -2.078 | 10 | 0.227 |  | | | |
| 102658\_at | Il1r2 | interleukin 1 receptor, type II | chr1 | -1.46 | 10 | 0 |  | | | |
| 102663\_at | Plaur | urokinase plasminogen activator receptor | --- | -2.014 | 10 | 0.036 |  | | | |
| 102734\_at | Birc3 | baculoviral IAP repeat-containing 3 | --- | -1.771 | 10 | 0.229 |  | | | |
| 102768\_i\_at | Sc5d | sterol-C5-desaturase (fungal ERG3, delta-5-desaturase) homolog (S. cerevisae) | chr9 | -2.488 | 10 | 0.122 |  | | | |
| 102776\_at | 4833420O05Rik | RIKEN cDNA 4833420O05 gene | chr8 | -1.485 | 10 | 0.085 |  | | | |
| 102781\_at | Ccnl2 | cyclin L2 | chr4 | -1.306 | 10 | 0.005 |  | | | |
| 102787\_at | Gpr56 | G protein-coupled receptor 56 | chr8 | -1.614 | 10 | 0.021 |  | | | |
| 102794\_at | Cxcr4 | chemokine (C-X-C motif) receptor 4 | chr1 | -4.356 | 10 | 0.599 |  | | | |
| 102836\_at | Pps | putative phosphatase | chr11 | -1.69 | 10 | 0 |  | | | |
| 102854\_s\_at | Atp7a | ATPase, Cu++ transporting, alpha polypeptide | chrX | -1.236 | 10 | 0.131 |  | | | |
| 102912\_at | 5430432P15Rik | RIKEN cDNA 5430432P15 gene | chr19 | -1.21 | 10 | 0.382 |  | | | |
| 102920\_at | 9130422G05Rik | RIKEN cDNA 9130422G05 gene | chr4 | -1.625 | 10 | 0.33 |  | | | |
| 103015\_at | Bcl6 | B-cell leukemia/lymphoma 6 | chr16 | -4.658 | 10 | 0.02 |  | | | |
| 103017\_at | Tm7sf1 | transmembrane 7 superfamily member 1 | chr13 | -1.252 | 10 | 0.291 |  | | | |
| 103029\_at | Pdcd4 | programmed cell death 4 | chr19 | -1.951 | 10 | 0.525 |  | | | |
| 103062\_at | Rab33b | RAB33B, member of RAS oncogene family | chr3 | -1.412 | 10 | 0.113 |  | | | |
| 103210\_at | Csf2rb2 | colony stimulating factor 2 receptor, beta 2, low-affinity (granulocyte-macrophage) | chr15 | -1.2 | 10 | 0.02 |  | | | |
| 103222\_at | Eps8 | epidermal growth factor receptor pathway substrate 8 | chr6 | -1.718 | 10 | 0.326 |  | | | |
| 103254\_at | Fln29-pending | FLN29 gene product | chr5 | -2.179 | 10 | 0.011 |  | | | |
| 103258\_at | Ly75 | lymphocyte antigen 75 | chr2 | -2.605 | 10 | 0.615 |  | | | |
| 103321\_at | B230364F10 | hypothetical protein B230364F10 | chr5 | -1.666 | 10 | 0.198 |  | | | |
| 103328\_at | Tank | TRAF family member-associated Nf-kappa B activator | chr2 | -1.263 | 10 | 0.161 |  | | | |
| 103376\_s\_at | Pitpnm | phosphatidylinositol membrane-associated | chr19 | -1.877 | 10 | 0.108 |  | | | |
| 103422\_at | Cd1d1 | CD1d1 antigen | chr3 | -2.191 | 10 | 0.001 |  | | | |
| 103443\_at | Aim1 | absent in melanoma 1 | chr10 | -1.49 | 10 | 0.005 |  | | | |
| 103446\_at | 9130009C22Rik | RIKEN cDNA 9130009C22 gene | --- | -2.406 | 10 | 0.101 |  | | | |
| 103451\_at | Ptk2b | PTK2 protein tyrosine kinase 2 beta | chr14 | -1.201 | 10 | 0.081 |  | | | |
| 103471\_at | 4432405K22Rik | RIKEN cDNA 4432405K22 gene | chr10 | -1.18 | 10 | 0.081 |  | | | |
| 103518\_at | Ctla2b | cytotoxic T lymphocyte-associated protein 2 beta | chr13 | -5.219 | 10 | 0.015 |  | | | |
| 103531\_f\_at | ero1-beta | endoplasmic oxidoreductase 1 beta | --- | -2.112 | 10 | 0.205 |  | | | |
| 103584\_at | 5830471E12Rik | RIKEN cDNA 5830471E12 gene | --- | -1.276 | 10 | 0.085 |  | | | |
| 103596\_at | Dgka | diacylglycerol kinase, alpha | chr10 | -3.305 | 10 | 0 |  | | | |
| 103672\_at | 2410141M05Rik | RIKEN cDNA 2410141M05 gene | chr11 | -1.198 | 10 | 0.001 |  | | | |
| 103674\_f\_at | Eif2s3y | eukaryotic translation initiation factor 2, subunit 3, structural gene Y-linked | --- | -3.292 | 10 | 0.274 |  | | | |
| 103686\_at | Mcoln2 | mucolipin 2 | chr3 | -1.47 | 10 | 0.057 |  | | | |
| 103720\_at | Rest | RE1-silencing transcription factor | chr5 | -3.222 | 10 | 0.377 |  | | | |
| 103736\_at | 2500002E12Rik | RIKEN cDNA 2500002E12 gene | chr10 | -1.557 | 10 | 0.186 |  | | | |
| 103812\_at | Clca1 | chloride channel calcium activated 1 | chr3 | -4.597 | 10 | 0.005 |  | | | |
| 103842\_at | Ddx3y | DEAD (Asp-Glu-Ala-Asp) box polypeptide 3, Y-linked | --- | -4.323 | 10 | 0.192 |  | | | |
| 103891\_i\_at | Ell2 | elongation factor RNA polymerase II 2 | chr13 | -2.946 | 10 | 0.07 |  | | | |
| 103892\_r\_at | Ell2 | elongation factor RNA polymerase II 2 | chr13 | -2.911 | 10 | 0.209 |  | | | |
| 103895\_at | AW549877 | expressed sequence AW549877 | chr15 | -1.037 | 10 | 0.03 |  | | | |
| 103922\_f\_at | 1500005G05Rik | RIKEN cDNA 1500005G05 gene | chr1 | -1.877 | 10 | 0.12 |  | | | |
| 103930\_at | N4bp1-pending | Nedd4 binding protein 1 | chr8 | -1.676 | 10 | 0.067 |  | | | |
| 103933\_at | Ysg2 | yolk sac gene 2 | chr9 | -1.342 | 10 | 0.056 |  | | | |
| 103959\_at | Phf13 | PHD finger protein 13 | chr4 | -3.552 | 10 | 0.062 |  | | | |
| 103994\_at | Eif2c2 | eukaryotic translation initiation factor 2C, 2 | chr11 | -1.478 | 10 | 0.09 |  | | | |
| 104049\_at | 1200013I08Rik | RIKEN cDNA 1200013I08 gene | --- | -1.026 | 10 | 0.432 |  | | | |
| 104070\_at | Pcaf | p300/CBP-associated factor | chr13 | -1.065 | 10 | 0.744 |  | | | |
| 104106\_at | Sbno1 | sno, strawberry notch homolog 1 (Drosophila) | chr5 | -1.711 | 10 | 0.411 |  | | | |
| 104117\_at | 4930421J07Rik | RIKEN cDNA 4930421J07 gene | chr12 | -1.262 | 10 | 0.14 |  | | | |
| 104119\_at | AW060714 | expressed sequence AW060714 | chr18 | -2.011 | 10 | 0.101 |  | | | |
| 104144\_at | Gtpbp2 | GTP binding protein 2 | chr17 | -1.316 | 10 | 0.016 |  | | | |
| 104165\_at | Vnn1 | vanin 1 | --- | -2.98 | 10 | 0.003 |  | | | |
| 104179\_at | Arf6 | ADP-ribosylation factor 6 | chr12 | -1.239 | 10 | 0.694 |  | | | |
| 104188\_at | Notch2 | Notch gene homolog 2 (Drosophila) | chr3 | -1.26 | 10 | 0.963 |  | | | |
| 104206\_at | 0610012A05Rik | RIKEN cDNA 0610012A05 gene | chr15 | -7.151 | 10 | 0.003 |  | | | |
| 104250\_at | NoneAvailable | Mus musculus, clone IMAGE:5290106, mRNA | chr2 | -1.787 | 10 | 0.136 |  | | | |
| 104256\_at | Pscdbp | pleckstrin homology, Sec7 and coiled-coil domains, binding protein | chr2 | -2.279 | 10 | 0.022 |  | | | |
| 104257\_g\_at | Pscdbp | pleckstrin homology, Sec7 and coiled-coil domains, binding protein | chr2 | -2.012 | 10 | 0.004 |  | | | |
| 104263\_at | 9330177P20Rik | RIKEN cDNA 9330177P20 gene | chr4 | -1.251 | 10 | 0.004 |  | | | |
| 104311\_at | 1300013G12Rik | RIKEN cDNA 1300013G12 gene | chr1 | -1.417 | 10 | 0.024 |  | | | |
| 104371\_at | Dgat1 | diacylglycerol O-acyltransferase 1 | chr15 | -3.266 | 10 | 0 |  | | | |
| 104386\_f\_at | 1110004F14Rik | RIKEN cDNA 1110004F14 gene | chr2 | -2.602 | 10 | 0.153 |  | | | |
| 104404\_at | 5730533P17Rik | RIKEN cDNA 5730533P17 gene | chr14 | -1.076 | 10 | 0.484 |  | | | |
| 104407\_at | Alcam | activated leukocyte cell adhesion molecule | chr16 | -4.923 | 10 | 0.155 |  | | | |
| 104442\_at | A830021G03Rik | RIKEN cDNA A830021G03 gene | --- | -1.281 | 10 | 0.133 |  | | | |
| 104445\_at | 4631408O11Rik | RIKEN cDNA 4631408O11 gene | chr2 | -2.355 | 10 | 0.666 |  | | | |
| 104453\_at | NoneAvailable | Mus musculus cDNA clone IMAGE:6433799, partial cds | chr11 | -1.124 | 10 | 0.03 |  | | | |
| 104456\_at | Mettl3 | methyltransferase-like 3 | chr14 | -1.939 | 10 | 0.209 |  | | | |
| 104534\_at | Pgm1 | phosphoglucomutase 1 | chr5 | -1.635 | 10 | 0.547 |  | | | |
| 104572\_at | Etohd2 | ethanol decreased 2 | chr13 | -1.512 | 10 | 0.001 |  | | | |
| 104605\_at | 1110001I14Rik | RIKEN cDNA 1110001I14 gene | chr6 | -1.289 | 10 | 0.102 |  | | | |
| 104677\_at | LOC227619 | hypothetical protein LOC227619 | chr2 | -1.818 | 10 | 0.046 |  | | | |
| 104701\_at | Bhlhb2 | basic helix-loop-helix domain containing, class B2 | chr6 | -3.085 | 10 | 0.023 |  | | | |
| 104741\_at | 9530098M12Rik | RIKEN cDNA 9530098M12 gene | chrX | -1.016 | 10 | 0.015 |  | | | |
| 104745\_at | Arl6ip2 | ADP-ribosylation factor-like 6 interacting protein 2 | chr17 | -1.189 | 10 | 0.045 |  | | | |
| 104761\_at | Antxr2 | anthrax toxin receptor 2 | chr5 | -1.717 | 10 | 0.055 |  | | | |
| 160079\_i\_at | Wwp4-pending | WW domain-containing protein 4 | chr18 | -1.299 | 10 | 0.227 |  | | | |
| 160088\_at | Fmo5 | flavin containing monooxygenase 5 | chr3 | -1.409 | 10 | 0.019 |  | | | |
| 160099\_at | Lgals4 | lectin, galactose binding, soluble 4 | chr7 | -1.876 | 10 | 0.001 |  | | | |
| 160103\_at | Axot | axotrophin | chr2 | -1.214 | 10 | 0.471 |  | | | |
| 160130\_at | Wdr26 | WD repeat domain 26 | chr1 | -1.964 | 10 | 0.093 |  | | | |
| 160151\_i\_at | 1200009B18Rik | RIKEN cDNA 1200009B18 gene | chr6 | -1.735 | 10 | 0.029 |  | | | |
| 160246\_at | E130304C20Rik | RIKEN cDNA E130304C20 gene | chr18 | -1.316 | 10 | 0.426 |  | | | |
| 160249\_at | Tpd52 | tumor protein D52 | chr3 | -1.593 | 10 | 0.241 |  | | | |
| 160264\_s\_at | 1500036F01Rik | RIKEN cDNA 1500036F01 gene | chr1 | -1.44 | 10 | 0.029 |  | | | |
| 160285\_at | Dhx40 | DEAH (Asp-Glu-Ala-His) box polypeptide 40 | chr11 | -1.793 | 10 | 0.225 |  | | | |
| 160287\_at | Map1lc3 | microtubule-associated protein 1 light chain 3 | chr14 | -1.669 | 10 | 0.02 |  | | | |
| 160313\_at | A730098D12Rik | RIKEN cDNA A730098D12 gene | chr5 | -1.268 | 10 | 0.125 |  | | | |
| 160335\_at | Gclm | glutamate-cysteine ligase , modifier subunit | chr3 | -2.391 | 10 | 0.304 |  | | | |
| 160374\_r\_at | Ptbp2 | polypyrimidine tract binding protein 2 | chr3 | -1.112 | 10 | 0.303 |  | | | |
| 160383\_at | Cox7a2l | cytochrome c oxidase subunit VIIa polypeptide 2-like | chr12 | -1.082 | 10 | 0.082 |  | | | |
| 160393\_at | 4930555L11Rik | RIKEN cDNA 4930555L11 gene | chr6 | -2.283 | 10 | 0.001 |  | | | |
| 160397\_at | Ik | IK cytokine | chr18 | -1.163 | 10 | 0.566 |  | | | |
| 160399\_r\_at | H2afy | H2A histone family, member Y | chr13 | -1.179 | 10 | 0.164 |  | | | |
| 160440\_at | Madh4 | MAD homolog 4 (Drosophila) | chr18 | -1.348 | 10 | 0.278 |  | | | |
| 160469\_at | Thbs1 | thrombospondin 1 | chr2 | -4.169 | 10 | 0.085 |  | | | |
| 160483\_at | Tcf4 | transcription factor 4 | chr18 | -1.191 | 10 | 0.38 |  | | | |
| 160495\_at | Ahr | aryl-hydrocarbon receptor | chr12 | -3.189 | 10 | 0.004 |  | | | |
| 160507\_at | 0610013E23Rik | RIKEN cDNA 0610013E23 gene | chr11 | -2.005 | 10 | 0.224 |  | | | |
| 160511\_at | Cxcl12 | chemokine (C-X-C motif) ligand 12 | chr6 | -1.569 | 10 | 0.451 |  | | | |
| 160588\_at | Zfp131 | zinc finger protein 131 | --- | -1.358 | 10 | 0.31 |  | | | |
| 160605\_s\_at | 4833420O05Rik | RIKEN cDNA 4833420O05 gene | --- | -2.248 | 10 | 0.091 |  | | | |
| 160615\_at | Pias3 | protein inhibitor of activated STAT 3 | chr3 | -1.638 | 10 | 0.073 |  | | | |
| 160620\_at | Gt(ROSA)26Sor | gene trap ROSA 26, Philippe Soriano | --- | -1.334 | 10 | 0.617 |  | | | |
| 160655\_at | Cpd | carboxypeptidase D | chr11 | -1.361 | 10 | 0.269 |  | | | |
| 160683\_at | C330005L02Rik | RIKEN cDNA C330005L02 gene | chr9 | -1.353 | 10 | 0.068 |  | | | |
| 160688\_at | Golph3 | golgi phosphoprotein 3 | chr15 | -1.744 | 10 | 0.492 |  | | | |
| 160726\_at | Qk | quaking | --- | -1.007 | 10 | 0.368 |  | | | |
| 160757\_at | 2610202O07Rik | RIKEN cDNA 2610202O07 gene | chr4 | -1.034 | 10 | 0.081 |  | | | |
| 160772\_i\_at | Slu7-pending | step II splicing factor SLU7 | --- | -1.233 | 10 | 0.143 |  | | | |
| 160834\_at | 1110032C13Rik | RIKEN cDNA 1110032C13 gene | chr7 | -4.49 | 10 | 0.04 |  | | | |
| 160857\_at | Efnb2 | ephrin B2 | chr8 | -2.391 | 10 | 0.363 |  | | | |
| 160889\_at | 8430437G11Rik | RIKEN cDNA 8430437G11 gene | chr6 | -1.07 | 10 | 0.772 |  | | | |
| 160894\_at | Cebpd | CCAAT/enhancer binding protein (C/EBP), delta | chr16 | -1.36 | 10 | 0.05 |  | | | |
| 160920\_at | Bcl2l2 | Bcl2-like 2 | chr14 | -1.72 | 10 | 0.02 |  | | | |
| 160965\_at | AA793972 | EST AA793972 | chr5 | -3.294 | 10 | 0.001 |  | | | |
| 160977\_at | Arhgef5 | Rho guanine nucleotide exchange factor (GEF) 5 | chr6 | -3.899 | 10 | 0.031 |  | | | |
| 161070\_at | Spred2 | sprouty protein with EVH-1 domain 2, related sequence | chr11 | -2.044 | 10 | 0.727 |  | | | |
| 161081\_at | Cpeb2 | cytoplasmic polyadenylation element binding protein 2 | chr5 | -3.809 | 10 | 0.02 |  | | | |
| 161084\_at | B230354B21Rik | RIKEN cDNA B230354B21 gene | --- | -1.148 | 10 | 0.183 |  | | | |
| 161109\_at | 1110017P05Rik | RIKEN cDNA 1110017P05 gene | --- | -1.394 | 10 | 0.011 |  | | | |
| 161113\_at | Esr1 | estrogen receptor 1 (alpha) | chr10 | -1.744 | 10 | 0.007 |  | | | |
| 161401\_f\_at | Aldh3a2 | aldehyde dehydrogenase family 3, subfamily A2 | --- | -1.25 | 10 | 0.845 |  | | | |
| 161436\_s\_at | Adarb1 | adenosine deaminase, RNA-specific, B1 | chr10 | -2.118 | 10 | 0.145 |  | | | |
| 161551\_f\_at | Riok3 | RIO kinase 3 (yeast) | --- | -1.464 | 10 | 0.012 |  | | | |
| 161609\_at | Rgs16 | regulator of G-protein signaling 16 | chr1 | -1.687 | 10 | 0.086 |  | | | |
| 161610\_at | Ndr2 | N-myc downstream regulated 2 | --- | -4.394 | 10 | 0.001 |  | | | |
| 161615\_f\_at | Map3k11 | mitogen activated protein kinase kinase kinase 11 | --- | -1.291 | 10 | 0.116 |  | | | |
| 161689\_f\_at | Il1r2 | interleukin 1 receptor, type II | chr1 | -3.446 | 10 | 0 |  | | | |
| 161696\_f\_at | C77080 | expressed sequence C77080 | chr4 | -3.068 | 10 | 0.144 |  | | | |
| 161760\_s\_at | C030034J04Rik | RIKEN cDNA C030034J04 gene | chr7 | -4.514 | 10 | 0.177 |  | | | |
| 161788\_f\_at | Edg1 | endothelial differentiation sphingolipid G-protein-coupled receptor 1 | chr3 | -1.5 | 10 | 0.087 |  | | | |
| 161814\_f\_at | Rnf19 | ring finger protein (C3HC4 type) 19 | chr15 | -1.939 | 10 | 0.035 |  | | | |
| 161946\_r\_at | Sorbs1 | sorbin and SH3 domain containing 1 | --- | -1.057 | 10 | 0.236 |  | | | |
| 161980\_f\_at | Bag3 | Bcl2-associated athanogene 3 | chr7 | -4.017 | 10 | 0.037 |  | | | |
| 161994\_f\_at | Pias3 | protein inhibitor of activated STAT 3 | chr3 | -1.226 | 10 | 0.161 |  | | | |
| 162010\_r\_at | Atf2 | activating transcription factor 2 | chr2 | -1.22 | 10 | 0.23 |  | | | |
| 162041\_f\_at | NoneAvailable | --- | --- | -1.038 | 10 | 0.01 |  | | | |
| 162206\_f\_at | Socs3 | suppressor of cytokine signaling 3 | --- | -4.307 | 10 | 0.013 |  | | | |
| 162313\_f\_at | Galnt3 | UDP-N-acetyl-alpha-D-galactosamine:polypeptide N-acetylgalactosaminyltransferase 3 | chr2 | -1.95 | 10 | 0.242 |  | | | |
| 162462\_r\_at | 9130022B02Rik | RIKEN cDNA 9130022B02 gene | --- | -1.715 | 10 | 0.053 |  | | | |
| 162496\_r\_at | Ndufa3 | NADH dehydrogenase (ubiquinone) 1 alpha subcomplex, 3 | --- | -1.105 | 10 | 0.099 |  | | | |
| 92185\_at | A630084M22Rik | RIKEN cDNA A630084M22 gene | chr1 | -4.242 | 10 | 0.095 |  | | | |
| 92195\_at | Cebpg | CCAAT/enhancer binding protein (C/EBP), gamma | chr7 | -2.092 | 10 | 0.255 |  | | | |
| 92265\_f\_at | Ssa2 | Sjogren syndrome antigen A2 | --- | -1.291 | 10 | 0.096 |  | | | |
| 92268\_at | 2700007P21Rik | RIKEN cDNA 2700007P21 gene | chr2 | -1.269 | 10 | 0.707 |  | | | |
| 92302\_at | Sos2 | Son of sevenless homolog 2 (Drosophila) | chr12 | -1.846 | 10 | 0.838 |  | | | |
| 92344\_at | Smarca3 | SWI/SNF related, matrix associated, actin dependent regulator of chromatin, subfamily a, member 3 | chr3 | -1.402 | 10 | 0.162 |  | | | |
| 92484\_at | Hivep2 | human immunodeficiency virus type I enhancer binding protein 2 | chr10 | -1.852 | 10 | 0.264 |  | | | |
| 92542\_at | D4Wsu53e | DNA segment, Chr 4, Wayne State University 53, expressed | chr4 | -1.236 | 10 | 0.014 |  | | | |
| 92562\_at | Nfe2l2 | nuclear, factor, erythroid derived 2, like 2 | chr2 | -1.243 | 10 | 0.109 |  | | | |
| 92568\_at | Tfb2m | transcription factor B2, mitochondrial | --- | -1.763 | 10 | 0.072 |  | | | |
| 92638\_at | Ppp2ca | protein phosphatase 2a, catalytic subunit, alpha isoform | chr11 | -1.373 | 10 | 0.725 |  | | | |
| 92877\_at | Tgfbi | transforming growth factor, beta induced | chr13 | -1.788 | 10 | 0.029 |  | | | |
| 92888\_s\_at | Styx | phosphoserine/threonine/tyrosine interaction protein | chr14 | -1.212 | 10 | 0.792 |  | | | |
| 92925\_at | Cebpb | CCAAT/enhancer binding protein (C/EBP), beta | chr2 | -1.175 | 10 | 0.113 |  | | | |
| 92992\_i\_at | 5730497N03Rik | RIKEN cDNA 5730497N03 gene | chr12 | -1.16 | 10 | 0.027 |  | | | |
| 92993\_r\_at | 5730497N03Rik | RIKEN cDNA 5730497N03 gene | chr12 | -1.744 | 10 | 0.003 |  | | | |
| 93026\_at | Mgst1 | microsomal glutathione S-transferase 1 | chr6 | -1.115 | 10 | 0.158 |  | | | |
| 93064\_at | Bnip2 | BCL2/adenovirus E1B 19kDa-interacting protein 1, NIP2 | chr9 | -1.051 | 10 | 0.351 |  | | | |
| 93104\_at | Btg1 | B-cell translocation gene 1, anti-proliferative | chr10 | -3.713 | 10 | 0.005 |  | | | |
| 93193\_at | Adrb2 | adrenergic receptor, beta 2 | chr18 | -1.617 | 10 | 0.021 |  | | | |
| 93274\_at | Clk | CDC-like kinase | chr1 | -1.211 | 10 | 0.031 |  | | | |
| 93311\_at | Clk3 | CDC-like kinase 3 | chr9 | -1.389 | 10 | 0.003 |  | | | |
| 93315\_at | Map2k3 | mitogen activated protein kinase kinase 3 | chr11 | -2.248 | 10 | 0.044 |  | | | |
| 93414\_at | Abcb1b | ATP-binding cassette, sub-family B (MDR/TAP), member 1B | chr5 | -1.239 | 10 | 0.019 |  | | | |
| 93424\_at | NoneAvailable | Mus musculus, Similar to KIAA0916 protein, clone IMAGE:4022573, mRNA | chr14 | -1.14 | 10 | 0.024 |  | | | |
| 93440\_at | 4930564D15Rik | RIKEN cDNA 4930564D15 gene | chr3 | -1.001 | 10 | 0.017 |  | | | |
| 93464\_at | Akap9 | A kinase (PRKA) anchor protein (yotiao) 9 | chr5 | -1.62 | 10 | 0.231 |  | | | |
| 93471\_at | Slc4a7 | solute carrier family 4, sodium bicarbonate cotransporter, member 7 | chr14 | -1.068 | 10 | 0.223 |  | | | |
| 93520\_at | Srrm1 | serine/arginine repetitive matrix 1 | --- | -1.338 | 10 | 0.018 |  | | | |
| 93753\_at | Litaf | LPS-induced TN factor | chr16 | -1.716 | 10 | 0.013 |  | | | |
| 93852\_at | Mef2a | myocyte enhancer factor 2A | chr7 | -2.066 | 10 | 0.044 |  | | | |
| 93914\_at | Il1r1 | interleukin 1 receptor, type I | chr1 | -1.087 | 10 | 0.006 |  | | | |
| 93963\_at | AI661017 | expressed sequence AI661017 | chr15 | -2.487 | 10 | 0.22 |  | | | |
| 93965\_r\_at | Ddx6 | DEAD (Asp-Glu-Ala-Asp) box polypeptide 6 | chr9 | -3.007 | 10 | 0.007 |  | | | |
| 93975\_at | 1300002F13Rik | RIKEN cDNA 1300002F13 gene | chr4 | -6.877 | 10 | 0 |  | | | |
| 93985\_at | AW558171 | expressed sequence AW558171 | chr3 | -2.596 | 10 | 0.594 |  | | | |
| 94018\_at | Ubl3 | ubiquitin-like 3 | chr5 | -2.685 | 10 | 0.808 |  | | | |
| 94106\_at | NoneAvailable | Mus musculus transcribed sequences | chr18 | -1.04 | 10 | 0.226 |  | | | |
| 94192\_at | Gdap10 | ganglioside-induced differentiation-associated-protein 10 | chr12 | -2.38 | 10 | 0.047 |  | | | |
| 94255\_g\_at | Clic4 | chloride intracellular channel 4 (mitochondrial) | chr4 | -2.625 | 10 | 0.25 |  | | | |
| 94264\_at | Raf1 | v-raf-1 leukemia viral oncogene 1 | chr6 | -1.251 | 10 | 0.001 |  | | | |
| 94319\_at | Rab18 | RAB18, member RAS oncogene family | chr18 | -1.068 | 10 | 0.581 |  | | | |
| 94331\_at | Stat6 | signal transducer and activator of transcription 6 | chr10 | -2.796 | 10 | 0 |  | | | |
| 94374\_at | Wdr13 | WD repeat domain 13 | chrX | -1.734 | 10 | 0.137 |  | | | |
| 94415\_at | 6230421P05Rik | RIKEN cDNA 6230421P05 gene | chr16 | -1.852 | 10 | 0.266 |  | | | |
| 94439\_at | Osbpl11 | oxysterol binding protein-like 11 | chr16 | -1.618 | 10 | 0.713 |  | | | |
| 94464\_at | Clcn3 | chloride channel 3 | chr8 | -1.229 | 10 | 0.259 |  | | | |
| 94465\_g\_at | Clcn3 | chloride channel 3 | chr8 | -1.312 | 10 | 0.342 |  | | | |
| 94483\_at | Csnk2a2 | casein kinase II, alpha 2, polypeptide | chr8 | -1.076 | 10 | 0.04 |  | | | |
| 94689\_at | C79248 | expressed sequence C79248 | --- | -1.2 | 10 | 0.015 |  | | | |
| 94695\_at | NoneAvailable | Mus musculus transcribed sequences | chr3 | -1.132 | 10 | 0.419 |  | | | |
| 94780\_at | Zfp288 | zinc finger protein 288 | chr16 | -4.353 | 10 | 0.034 |  | | | |
| 94809\_at | Tsg101 | tumor susceptibility gene 101 | --- | -1.094 | 10 | 0.756 |  | | | |
| 94818\_at | Ogt | O-linked N-acetylglucosamine (GlcNAc) transferase (UDP-N-acetylglucosamine:polypeptide-N-acetylglucosaminyl transferase) | chrX | -1.371 | 10 | 0.004 |  | | | |
| 94830\_at | BC005537 | cDNA sequence BC005537 | chr13 | -1.83 | 10 | 0.008 |  | | | |
| 94872\_at | 0610010C24Rik | RIKEN cDNA 0610010C24 gene | chr10 | -2.579 | 10 | 0.155 |  | | | |
| 94899\_at | Rhoip3-pending | Rho interacting protein 3 | chr11 | -1.473 | 10 | 0.042 |  | | | |
| 94928\_at | Tnfrsf1b | tumor necrosis factor receptor superfamily, member 1b | --- | -1.859 | 10 | 0.009 |  | | | |
| 94939\_at | Cd53 | CD53 antigen | chr3 | -1.067 | 10 | 0.004 |  | | | |
| 94980\_at | Dusp11 | dual specificity phosphatase 11 (RNA/RNP complex 1-interacting) | chr6 | -1.6 | 10 | 0.004 |  | | | |
| 95023\_at | BC023957 | cDNA sequence BC023957 | chr9 | -1.742 | 10 | 0 |  | | | |
| 95092\_at | Ppp3ca | protein phosphatase 3, catalytic subunit, alpha isoform | chr3 | -1.105 | 10 | 0.709 |  | | | |
| 95119\_at | 1110038D17Rik | RIKEN cDNA 1110038D17 gene | chr10 | -1.366 | 10 | 0.009 |  | | | |
| 95120\_at | Tm4sf13 | transmembrane 4 superfamily member 13 | chr12 | -1.946 | 10 | 0.326 |  | | | |
| 95287\_at | NoneAvailable | Mus musculus RIKEN cDNA 4930471C18 gene, mRNA (cDNA clone IMAGE:4487650), partial cds | chr6 | -1.232 | 10 | 0.032 |  | | | |
| 95444\_at | 4930579A11Rik | RIKEN cDNA 4930579A11 gene | chr11 | -2.862 | 10 | 0.016 |  | | | |
| 95489\_at | Fliih | flightless I homolog (Drosophila) | chr11 | -1.074 | 10 | 0.042 |  | | | |
| 95511\_at | Itga6 | integrin alpha 6 | chr2 | -1.684 | 10 | 0.301 |  | | | |
| 95521\_s\_at | Zfp68 | Zinc finger protein 68 | chr5 | -2.132 | 10 | 0.035 |  | | | |
| 95539\_at | Gtpat12 | gene trap PAT 12 | chr10 | -1.196 | 10 | 0.441 |  | | | |
| 95564\_at | BC018601 | cDNA sequence BC018601 | chr11 | -2.789 | 10 | 0.032 |  | | | |
| 95586\_at | P2rx4 | purinergic receptor P2X, ligand-gated ion channel 4 | chr5 | -1.711 | 10 | 0 |  | | | |
| 95655\_at | 5830411E10Rik | RIKEN cDNA 5830411E10 gene | chr1 | -1.472 | 10 | 0.036 |  | | | |
| 95688\_at | Degs | degenerative spermatocyte homolog (Drosophila) | chr1 | -2.305 | 10 | 0.306 |  | | | |
| 95862\_at | NoneAvailable | --- | chr2 | -2.045 | 10 | 0.125 |  | | | |
| 95869\_at | NoneAvailable | Mus musculus transcribed sequences | --- | -1.315 | 10 | 0.578 |  | | | |
| 95872\_at | NoneAvailable | Mus musculus transcribed sequences | --- | -1.216 | 10 | 0.063 |  | | | |
| 95917\_at | NoneAvailable | Mus musculus transcribed sequences | chr8 | -4.563 | 10 | 0.041 |  | | | |
| 95962\_at | NoneAvailable | Mus musculus transcribed sequence with weak  similarity to protein ref:NP\_081764.1 (M.musculus)  RIKEN cDNA 5730493B19 [Mus musculus] | chr7 | -1.092 | 10 | 0.223 |  | | | |
| 96157\_at | Zfp91 | zinc finger protein 91 | chr19 | -1.113 | 10 | 0.065 |  | | | |
| 96176\_at | Arih2 | ariadne homolog 2 (Drosophila) | chr9 | -1.874 | 10 | 0.022 |  | | | |
| 96189\_at | 2410141K03Rik | RIKEN cDNA 2410141K03 gene | --- | -2.437 | 10 | 0.013 |  | | | |
| 96192\_at | Sp3 | trans-acting transcription factor 3 | chr2 | -1.189 | 10 | 0.051 |  | | | |
| 96414\_at | Nsccn1 | non-selective cation channel 1 | --- | -1.133 | 10 | 0.598 |  | | | |
| 96488\_at | NoneAvailable | Mus musculus transcribed sequence with moderate similarity to protein sp:O60674 (H.sapiens) JAK2\_HUMAN Tyrosine-protein kinase JAK2 (Janus kinase 2) (JAK-2) | --- | -2.509 | 10 | 0.158 |  | | | |
| 96513\_at | NoneAvailable | --- | chr11 | -2.8 | 10 | 0.559 |  | | | |
| 96532\_at | NoneAvailable | Mus musculus 12 days embryo male wolffian duct includes surrounding region cDNA, RIKEN full-length enriched library, clone:6720481A07 product:nucleolar protein GU2, full insert sequence | chr10 | -2.148 | 10 | 0.337 |  | | | |
| 96534\_at | Vldlr | very low density lipoprotein receptor | chr19 | -1.008 | 10 | 0 |  | | | |
| 96561\_at | Nfatc2ip | nuclear factor of activated T-cells, cytoplasmic 2 interacting protein | --- | -1.522 | 10 | 0.179 |  | | | |
| 96600\_at | NoneAvailable | Mus musculus transcribed sequence with moderate similarity to protein ref:NP\_004806.1 (H.sapiens)  PTPL1-associated RhoGAP 1 [Homo sapiens] | chr3 | -2.473 | 10 | 0.607 |  | | | |
| 96615\_at | 0610043B10Rik | RIKEN cDNA 0610043B10 gene | chr7 | -1.733 | 10 | 0.102 |  | | | |
| 96628\_at | Eprs | glutamyl-prolyl-tRNA synthetase | chr1 | -1.313 | 10 | 0.054 |  | | | |
| 96725\_at | Cic | capicua homolog (Drosophila) | chr7 | -1.047 | 10 | 0.089 |  | | | |
| 96738\_at | Adam9 | a disintegrin and metalloproteinase domain 9 (meltrin gamma) | chr8 | -3.478 | 10 | 0.291 |  | | | |
| 96777\_at | Sf3b1 | splicing factor 3b, subunit 1 | chr1 | -1.402 | 10 | 0.053 |  | | | |
| 96806\_at | Lpin2 | lipin 2 | chr17 | -1.054 | 10 | 0.557 |  | | | |
| 96813\_f\_at | DXImx46e | DNA segment, Chr X, Immunex 46, expressed | chrX | -1.437 | 10 | 0.027 |  | | | |
| 96875\_r\_at | D6Ertd772e | DNA segment, Chr 6, ERATO Doi 772, expressed | chr6 | -2.157 | 10 | 0.092 |  | | | |
| 96945\_at | Snap23 | synaptosomal-associated protein 23 | chr2 | -1.319 | 10 | 0.127 |  | | | |
| 96951\_at | Atp6v1d | ATPase, H+ transporting, V1 subunit D | chr12 | -1.976 | 10 | 0.173 |  | | | |
| 97058\_f\_at | Rab33b | RAB33B, member of RAS oncogene family | chr3 | -1.332 | 10 | 0.147 |  | | | |
| 97105\_at | A230108E06 | hypothetical protein A230108E06 | chr9 | -1.925 | 10 | 0.221 |  | | | |
| 97118\_at | 1810028B20Rik | RIKEN cDNA 1810028B20 gene | chr19 | -1.614 | 10 | 0.036 |  | | | |
| 97285\_f\_at | Ubxdc2 | UBX domain-containing 2 | chr17 | -2.548 | 10 | 0 |  | | | |
| 97297\_at | 1500036F01Rik | RIKEN cDNA 1500036F01 gene | chr1 | -1.772 | 10 | 0.001 |  | | | |
| 97302\_at | Ivns1abp | influenza virus NS1A binding protein | chr1 | -1.88 | 10 | 0.333 |  | | | |
| 97304\_at | Ubp1 | upstream binding protein 1 | chr9 | -1.378 | 10 | 0.21 |  | | | |
| 97319\_at | Rrad | Ras-related associated with diabetes | chr8 | -2.544 | 10 | 0.005 |  | | | |
| 97349\_at | 4930488L10Rik | RIKEN cDNA 4930488L10 gene | chr12 | -2.819 | 10 | 0.04 |  | | | |
| 97357\_at | 5430401D19Rik | RIKEN cDNA 5430401D19 gene | chr13 | -1.57 | 10 | 0.561 |  | | | |
| 97398\_at | 9130022B02Rik | RIKEN cDNA 9130022B02 gene | chr14 | -1.212 | 10 | 0.49 |  | | | |
| 97426\_at | Emp1 | epithelial membrane protein 1 | chr6 | -2.305 | 10 | 0.086 |  | | | |
| 97429\_at | Snrk | SNF related kinase | chr9 | -1.935 | 10 | 0.021 |  | | | |
| 97434\_at | 2810405F18Rik | RIKEN cDNA 2810405F18 gene | chr4 | -1.181 | 10 | 0.655 |  | | | |
| 97447\_at | 1010001H21Rik | RIKEN cDNA 1010001H21 gene | chr2 | -1.365 | 10 | 0.69 |  | | | |
| 97484\_at | 2210402G22Rik | RIKEN cDNA 2210402G22 gene | chr13 | -1.114 | 10 | 0.085 |  | | | |
| 97548\_at | LOC328110 | hypothetical protein LOC328110 | --- | -1.863 | 10 | 0.361 |  | | | |
| 97551\_at | Hip1r | huntingtin interacting protein 1 related | chr5 | -1.274 | 10 | 0.068 |  | | | |
| 97665\_i\_at | NoneAvailable | Mus musculus transcribed sequence with moderate similarity to protein pir:S12207 (M.musculus) S12207 hypothetical protein (B2 element) - mouse | --- | -1.185 | 10 | 0.185 |  | | | |
| 97812\_at | Ranbp9 | RAN binding protein 9 | chr13 | -1.969 | 10 | 0.243 |  | | | |
| 97843\_at | Ncoa4 | nuclear receptor coactivator 4 | chr12 | -1.253 | 10 | 0.003 |  | | | |
| 97897\_at | NoneAvailable | Mus musculus, clone IMAGE:6430978, mRNA | chr13 | -1.708 | 10 | 0.027 |  | | | |
| 97925\_at | Csnk1e | casein kinase 1, epsilon | chr15 | -1.783 | 10 | 0.061 |  | | | |
| 98000\_at | Ly64 | lymphocyte antigen 64 | chr16 | -2.084 | 10 | 0.036 |  | | | |
| 98018\_at | Procr | protein C receptor, endothelial | chr2 | -4.119 | 10 | 0.038 |  | | | |
| 98110\_at | Mdm2 | transformed mouse 3T3 cell double minute 2 | chr10 | -1.298 | 10 | 0.73 |  | | | |
| 98114\_at | Npc1 | Niemann Pick type C1 | --- | -1.887 | 10 | 0.063 |  | | | |
| 98324\_at | Foxa3 | forkhead box A3 | --- | -1.298 | 10 | 0.174 |  | | | |
| 98461\_at | 1200014P03Rik | RIKEN cDNA 1200014P03 gene | chr17 | -1.307 | 10 | 0.007 |  | | | |
| 98533\_at | Cyb5 | cytochrome b-5 | chr18 | -1.221 | 10 | 0.003 |  | | | |
| 98580\_at | Ppm1a | protein phosphatase 1A, magnesium dependent, alpha isoform | chr12 | -1.739 | 10 | 0.095 |  | | | |
| 98756\_at | 2810043O03Rik | RIKEN cDNA 2810043O03 gene | --- | -1.374 | 10 | 0.168 |  | | | |
| 98849\_at | NoneAvailable | Mus musculus transcribed sequences | chr8 | -1.564 | 10 | 0.088 |  | | | |
| 98855\_r\_at | NoneAvailable | Mus musculus transcribed sequence with weak  similarity to protein ref:NP\_001888.1 (H.sapiens)  melanoma-associated chondroitin sulfate proteoglycan 4 [Homo sapiens] | chr17 | -2.775 | 10 | 0.05 |  | | | |
| 98882\_s\_at | Ndel1 | nuclear distribution gene E-like homolog 1 (A. nidulans) | chr11 | -1.562 | 10 | 0 |  | | | |
| 98884\_r\_at | Ndel1 | nuclear distribution gene E-like homolog 1 (A. nidulans) | chr11 | -2.864 | 10 | 0.022 |  | | | |
| 98926\_at | Vamp2 | vesicle-associated membrane protein 2 | chr11 | -2.233 | 10 | 0 |  | | | |
| 98931\_at | 2610016K11Rik | RIKEN cDNA 2610016K11 gene | chr10 | -1.837 | 10 | 0.13 |  | | | |
| 98942\_r\_at | 2310032D16Rik | RIKEN cDNA 2310032D16 gene | chr2 | -1.287 | 10 | 0.105 |  | | | |
| 98945\_at | Sh3glb1 | SH3-domain GRB2-like B1 (endophilin) | --- | -1.415 | 10 | 0.418 |  | | | |
| 98951\_at | D8Ertd325e | DNA segment, Chr 8, ERATO Doi 325, expressed | chr8 | -1.148 | 10 | 0.009 |  | | | |
| 99025\_at | Ddx19 | DEAD (Asp-Glu-Ala-Asp) box polypeptide 19 | chr8 | -1.285 | 10 | 0.054 |  | | | |
| 99045\_at | Eno2 | enolase 2, gamma neuronal | --- | -1.634 | 10 | 0.008 |  | | | |
| 99070\_at | Chuk | conserved helix-loop-helix ubiquitous kinase | --- | -1.291 | 10 | 0.245 |  | | | |
| 99100\_at | Stat3 | signal transducer and activator of transcription 3 | chr11 | -1.118 | 10 | 0.022 |  | | | |
| 99103\_at | Irf3 | interferon regulatory factor 3 | chr7 | -1.113 | 10 | 0.042 |  | | | |
| 99143\_at | Tgoln1 | trans-golgi network protein | chr6 | -2.457 | 10 | 0.021 |  | | | |
| 99184\_at | Csad | cysteine sulfinic acid decarboxylase | --- | -2.259 | 10 | 0.001 |  | | | |
| 99187\_f\_at | 2010315L10Rik | RIKEN cDNA 2010315L10 gene | chr8 | -1.207 | 10 | 0.004 |  | | | |
| 99188\_at | 2010315L10Rik | RIKEN cDNA 2010315L10 gene | chr8 | -1.031 | 10 | 0.002 |  | | | |
| 99347\_f\_at | NoneAvailable | Mus musculus transcribed sequences | --- | -2.205 | 10 | 0.048 |  | | | |
| 99350\_at | Sec63 | SEC63-like (S. cerevisiae) | chr10 | -1.038 | 10 | 0.133 |  | | | |
| 99354\_s\_at | NoneAvailable | Mus musculus transcribed sequence with weak  similarity to protein ref:NP\_081764.1 (M.musculus)  RIKEN cDNA 5730493B19 [Mus musculus] | chr2 | -1.079 | 10 | 0.311 |  | | | |
| 99364\_at | C030034J04Rik | RIKEN cDNA C030034J04 gene | --- | -3.368 | 10 | 0.586 |  | | | |
| 99416\_at | NoneAvailable | Mus musculus transcribed sequence with moderate similarity to protein pir:S12207 (M.musculus) S12207 hypothetical protein (B2 element) - mouse | chr4 | -3.122 | 10 | 0.144 |  | | | |
| 99445\_at | 1110028E10Rik | RIKEN cDNA 1110028E10 gene | chr9 | -1.202 | 10 | 0.035 |  | | | |
| 99467\_at | Rasa1 | RAS p21 protein activator 1 | chr13 | -1.422 | 10 | 0.728 |  | | | |
| 99489\_at | Osp94 | osmotic stress protein | chr3 | -1.598 | 10 | 0.257 |  | | | |
| 99503\_at | 9130017A15Rik | RIKEN cDNA 9130017A15 gene | chr5 | -1.042 | 10 | 0.315 |  | | | |
| 99529\_f\_at | Rnf138 | ring finger protein 138 | --- | -2.231 | 10 | 0.072 |  | | | |
| 99535\_at | Ccrn4l | CCR4 carbon catabolite repression 4-like (S. cerevisiae) | chr3 | -1.202 | 10 | 0.428 |  | | | |
| 99963\_at | Zfp101 | zinc finger protein 101 | --- | -1.239 | 10 | 0.234 |  | | | |
| 99985\_at | Txnrd1 | thioredoxin reductase 1 | chr10 | -1.205 | 10 | 0.045 |  | | | |
| 100482\_at | BC023040 | cDNA sequence BC023040 | chr17 | -1.285 | 30 | 0.005 |  | | | |
| 100552\_at | Ifngr | interferon gamma receptor | chr10 | -1.093 | 30 | 0.436 |  | | | |
| 100567\_at | Fabp4 | fatty acid binding protein 4, adipocyte | --- | -1.177 | 30 | 0.051 |  | | | |
| 100583\_at | Igh-VJ558 | immunoglobulin heavy chain (J558 family) | chr12 | -1.148 | 30 | 0.003 |  | | | |
| 100622\_at | Prdx6 | peroxiredoxin 6 | chr1 | -2.147 | 30 | 0.05 |  | | | |
| 100924\_at | Gata3 | GATA binding protein 3 | chr2 | -2.504 | 30 | 0.215 |  | | | |
| 101030\_at | Arhb | ras homolog gene family, member AB | --- | -3.02 | 30 | 0.61 |  | | | |
| 101050\_at | 0610038L10Rik | RIKEN cDNA 0610038L10 gene | chr19 | -1.261 | 30 | 0.562 |  | | | |
| 101118\_at | NoneAvailable | Mus musculus transcribed sequences | chr12 | -1.241 | 30 | 0.437 |  | | | |
| 101392\_at | 6330407G04Rik | RIKEN cDNA 6330407G04 gene | chr14 | -1.128 | 30 | 0.107 |  | | | |
| 101422\_at | Fnbp4 | formin binding protein 4 | chr2 | -1.557 | 30 | 0.284 |  | | | |
| 101457\_at | Jak2 | Janus kinase 2 | chr19 | -1.515 | 30 | 0.487 |  | | | |
| 101982\_at | Vasp | vasodilator-stimulated phosphoprotein | chr7 | -2.065 | 30 | 0.059 |  | | | |
| 101995\_at | Sqstm1 | sequestosome 1 | chr11 | -2.505 | 30 | 0.103 |  | | | |
| 102224\_at | Igf1r | insulin-like growth factor I receptor | --- | -1.399 | 30 | 0.011 |  | | | |
| 102302\_at | Bckdhb | branched chain ketoacid dehydrogenase E1, beta polypeptide | chr9 | -1.776 | 30 | 0.019 |  | | | |
| 102371\_at | Nr4a1 | nuclear receptor subfamily 4, group A, member 1 | chr15 | -6.781 | 30 | 0.201 |  | | | |
| 102789\_at | Gata2 | GATA binding protein 2 | chr6 | -1.995 | 30 | 0.048 |  | | | |
| 102823\_at | MGC68300 | Unknown (protein for MGC:68300) | chr12 | -2.578 | 30 | 0.543 |  | | | |
| 102940\_at | Ltb | lymphotoxin B | chr17 | -1.553 | 30 | 0.653 |  | | | |
| 102966\_at | NoneAvailable | Mus musculus cDNA clone IMAGE:6410537, partial cds | chr5 | -1.197 | 30 | 0.351 |  | | | |
| 102996\_at | Ell | elongation factor RNA polymerase II | chr8 | -1.456 | 30 | 0.129 |  | | | |
| 103020\_s\_at | Map3k1 | mitogen activated protein kinase kinase kinase 1 | chr13 | -1.472 | 30 | 0.07 |  | | | |
| 103343\_at | 5430432M24Rik | RIKEN cDNA 5430432M24 gene | chr2 | -1.967 | 30 | 0.109 |  | | | |
| 103499\_at | Vwf | Von Willebrand factor homolog | chr6 | -1.515 | 30 | 0.297 |  | | | |
| 103500\_at | Orc4l | origin recognition complex, subunit 4-like (S. cerevisiae) | chr2 | -1.33 | 30 | 0.111 |  | | | |
| 103547\_at | Slc41a1 | solute carrier family 41, member 1 | chr1 | -2.728 | 30 | 0 |  | | | |
| 103713\_at | Usp9x | ubiquitin specific protease 9, X chromosome | chrX | -1.016 | 30 | 0.248 |  | | | |
| 103969\_at | Srpk2 | serine/arginine-rich protein specific kinase 2 | chr5 | -1.193 | 30 | 0.648 |  | | | |
| 104083\_at | Cdh5 | cadherin 5 | chr8 | -1.968 | 30 | 0.003 |  | | | |
| 104376\_at | Hdac5 | histone deacetylase 5 | chr11 | -2.263 | 30 | 0.006 |  | | | |
| 104389\_at | 1700017B05Rik | RIKEN cDNA 1700017B05 gene | --- | -1.287 | 30 | 0.056 |  | | | |
| 104417\_at | NoneAvailable | Mus musculus transcribed sequences | chr11 | -2.062 | 30 | 0.001 |  | | | |
| 104576\_at | Ski | Sloan-Kettering viral oncogene homolog | chr4 | -3.928 | 30 | 0.091 |  | | | |
| 104610\_at | BC013720 | cDNA sequence BC013720 | chr19 | -1.093 | 30 | 0.109 |  | | | |
| 104645\_at | Klf7 | Kruppel-like factor 7 (ubiquitous) | chr1 | -1.19 | 30 | 0.003 |  | | | |
| 104725\_at | Arhq | ras homolog gene family, member Q | chr17 | -3.36 | 30 | 0.128 |  | | | |
| 160228\_at | 1110019C08Rik | RIKEN cDNA 1110019C08 gene | chr16 | -1.025 | 30 | 0.025 |  | | | |
| 160252\_at | Cltc | clathrin, heavy polypeptide (Hc) | chr11 | -1.043 | 30 | 0.369 |  | | | |
| 160255\_at | 1110004P15Rik | RIKEN cDNA 1110004P15 gene | chr19 | -3.493 | 30 | 0.137 |  | | | |
| 160603\_at | Pparbp | peroxisome proliferator activated receptor binding protein | chr11 | -1.074 | 30 | 0.417 |  | | | |
| 160651\_at | Tacstd2 | tumor-associated calcium signal transducer 2 | chr6 | -3.696 | 30 | 0 |  | | | |
| 160727\_at | 2410002F23Rik | RIKEN cDNA 2410002F23 gene | chr2 | -1.032 | 30 | 0.005 |  | | | |
| 160739\_at | LOC269796 | hypothetical protein LOC269796 | chr6 | -1.672 | 30 | 0.602 |  | | | |
| 160829\_at | Phlda1 | pleckstrin homology-like domain, family A, member 1 | chr10 | -3.625 | 30 | 0.17 |  | | | |
| 160993\_at | NoneAvailable | Mus musculus 3 days neonate thymus cDNA, RIKEN full-length enriched library, clone:A630086H07 product:RAS GTPASE-ACTIVATING-LIKE PROTEIN IQGAP2 homolog [Homo sapiens], full insert sequence | chr13 | -2.171 | 30 | 0.108 |  | | | |
| 161067\_at | Ifld2 | induced in fatty liver dystrophy 2 | chr2 | -2.044 | 30 | 0.167 |  | | | |
| 161074\_at | Pcyt1a | phosphate cytidylyltransferase 1, choline, alpha isoform | chr16 | -1.541 | 30 | 0.084 |  | | | |
| 161080\_f\_at | 1700012P16Rik | RIKEN cDNA 1700012P16 gene | chr5 | -1.148 | 30 | 0.008 |  | | | |
| 161103\_at | Lysal2 | lysosomal apyrase-like 2 | chr19 | -1.122 | 30 | 0.775 |  | | | |
| 161184\_f\_at | Tie1 | tyrosine kinase receptor 1 | chr4 | -1.63 | 30 | 0.006 |  | | | |
| 161342\_r\_at | 2310046H11Rik | RIKEN cDNA 2310046H11 gene | --- | -1.175 | 30 | 0.533 |  | | | |
| 161990\_f\_at | BC012974 | hypothetical gene supported by BC012974 | chr18 | -1.702 | 30 | 0.006 |  | | | |
| 92216\_at | Madh7 | MAD homolog 7 (Drosophila) | chr18 | -2.696 | 30 | 0.13 |  | | | |
| 92249\_g\_at | Nr4a2 | nuclear receptor subfamily 4, group A, member 2 | chr2 | -5.506 | 30 | 0.001 |  | | | |
| 92294\_at | 2810410P22Rik | RIKEN cDNA 2810410P22 gene | chr2 | -1.24 | 30 | 0.577 |  | | | |
| 92821\_at | Usp2 | ubiquitin specific protease 2 | chr9 | -1.119 | 30 | 0.004 |  | | | |
| 92870\_at | Sel1h | Sel1 (suppressor of lin-12) 1 homolog (C. elegans) | chr12 | -1.228 | 30 | 0.472 |  | | | |
| 92908\_at | Hivep1 | human immunodeficiency virus type I enhancer binding protein 1 | chr13 | -1.609 | 30 | 0.205 |  | | | |
| 93177\_at | Ctbp2 | C-terminal binding protein 2 | chr7 | -1.119 | 30 | 0.187 |  | | | |
| 93191\_at | Vamp4 | vesicle-associated membrane protein 4 | chr1 | -2.06 | 30 | 0.077 |  | | | |
| 93619\_at | Per1 | period homolog 1 (Drosophila) | chr11 | -1.237 | 30 | 0.105 |  | | | |
| 93875\_at | Hspa1a | heat shock protein 1A | chr17 | -1.082 | 30 | 0.006 |  | | | |
| 94060\_at | Myo1h | myosin 1H | chr5 | -1.495 | 30 | 0.002 |  | | | |
| 94254\_at | Clic4 | chloride intracellular channel 4 (mitochondrial) | --- | -1.337 | 30 | 0.404 |  | | | |
| 94343\_at | Dnajc3 | DnaJ (Hsp40) homolog, subfamily C, member 3 | chr14 | -1.392 | 30 | 0.64 |  | | | |
| 94356\_at | Trp53bp1 | transformation related protein 53 binding protein 1 | chr2 | -1.038 | 30 | 0.108 |  | | | |
| 94420\_f\_at | Cry1 | cryptochrome 1 (photolyase-like) | chr10 | -1.369 | 30 | 0.052 |  | | | |
| 94433\_at | Slc38a2 | solute carrier family 38, member 2 | chr15 | -2.857 | 30 | 0.513 |  | | | |
| 94657\_at | NoneAvailable | Mus musculus transcribed sequences | chr8 | -5.096 | 30 | 0.006 |  | | | |
| 94752\_s\_at | Skil | SKI-like | chr3 | -1.931 | 30 | 0.237 |  | | | |
| 94770\_at | DXImx46e | DNA segment, Chr X, Immunex 46, expressed | --- | -1.61 | 30 | 0.093 |  | | | |
| 94861\_at | 4930453N24Rik | RIKEN cDNA 4930453N24 gene | chr16 | -1.288 | 30 | 0.653 |  | | | |
| 94976\_at | AL022610 | expressed sequence AL022610 | chr7 | -1.012 | 30 | 0.029 |  | | | |
| 95002\_at | D17Wsu92e | DNA segment, Chr 17, Wayne State University 92, expressed | chr17 | -1.483 | 30 | 0.016 |  | | | |
| 95033\_at | Jmjd1 | jumonji domain containing 1 | chr6 | -1.446 | 30 | 0.007 |  | | | |
| 95139\_at | 1110018O08Rik | RIKEN cDNA 1110018O08 gene | chr5 | -1.084 | 30 | 0.142 |  | | | |
| 95471\_at | Cdkn1c | cyclin-dependent kinase inhibitor 1C (P57) | chr7 | -1.401 | 30 | 0.062 |  | | | |
| 95563\_at | Arih1 | ariadne ubiquitin-conjugating enzyme E2 binding protein homolog 1 (Drosophila) | chr9 | -1.514 | 30 | 0.588 |  | | | |
| 95613\_at | 2010200I23Rik | RIKEN cDNA 2010200I23 gene | chr9 | -2.202 | 30 | 0.299 |  | | | |
| 95618\_at | D6Ertd32e | DNA segment, Chr 6, ERATO Doi 32, expressed | chr6 | -2.616 | 30 | 0.038 |  | | | |
| 95805\_at | Cdc2l2 | cell division cycle 2 homolog (S. pombe)-like 2 | chr4 | -1.429 | 30 | 0.02 |  | | | |
| 95858\_at | NoneAvailable | Mus musculus transcribed sequences | chr16 | -1.057 | 30 | 0.287 |  | | | |
| 96076\_at | Stx5a | syntaxin 5A | chr19 | -1.273 | 30 | 0.008 |  | | | |
| 96088\_at | Ndr2 | N-myc downstream regulated 2 | chr14 | -1.173 | 30 | 0.019 |  | | | |
| 96147\_at | Mafg | v-maf musculoaponeurotic fibrosarcoma oncogene family, protein G (avian) | chr11 | -1.157 | 30 | 0.03 |  | | | |
| 96367\_at | NoneAvailable | Mus musculus transcribed sequences | chr17 | -1.93 | 30 | 0.01 |  | | | |
| 96489\_at | NoneAvailable | --- | --- | -2.438 | 30 | 0.487 |  | | | |
| 96669\_at | 2400003C14Rik | RIKEN cDNA 2400003C14 gene | chr8 | -1.563 | 30 | 0.026 |  | | | |
| 96961\_at | Zfp144 | zinc finger protein 144 | chr11 | -2.635 | 30 | 0.275 |  | | | |
| 97125\_f\_at | LOC56628 | MHC (A.CA/J(H-2K-f) class I antigen | chr17 | -3.415 | 30 | 0.007 |  | | | |
| 97224\_at | Pnrc1 | proline-rich nuclear receptor coactivator 1 | --- | -1.392 | 30 | 0.135 |  | | | |
| 97375\_at | Pkd1 | polycystic kidney disease 1 homolog | chr17 | -1.292 | 30 | 0.009 |  | | | |
| 97541\_f\_at | H2-D1 | histocompatibility 2, D region locus 1 | --- | -4.349 | 30 | 0.053 |  | | | |
| 97829\_at | Cdipt | CDP-diacylglycerol--inositol 3-phosphatidyltransferase (phosphatidylinositol synthase) | chr7 | -1.346 | 30 | 0.076 |  | | | |
| 97833\_at | Pfkp | phosphofructokinase, platelet | chr13 | -1.17 | 30 | 0.504 |  | | | |
| 98059\_s\_at | Lmna | lamin A | chr3 | -3.624 | 30 | 0.108 |  | | | |
| 98065\_at | Ormdl3 | ORM1-like 3 (S. cerevisiae) | chr11 | -1.914 | 30 | 0.032 |  | | | |
| 98438\_f\_at | H2-Q7 | histocompatibility 2, Q region locus 7 | chr17 | -3.59 | 30 | 0.001 |  | | | |
| 98504\_at | Rock2 | Rho-associated coiled-coil forming kinase 2 | chr12 | -1.899 | 30 | 0.332 |  | | | |
| 98868\_at | Bcl2 | B-cell leukemia/lymphoma 2 | chr1 | -1.998 | 30 | 0.069 |  | | | |
| 98906\_at | Fbxo9 | f-box only protein 9 | chr9 | -2.278 | 30 | 0.037 |  | | | |
| 98984\_f\_at | Gpd2 | glycerol phosphate dehydrogenase 2, mitochondrial | chr2 | -1.107 | 30 | 0.416 |  | | | |
| 99013\_f\_at | Tmod3 | tropomodulin 3 | chr9 | -1.021 | 30 | 0.351 |  | | | |
| 99024\_at | Mad4 | Max dimerization protein 4 | --- | -1.293 | 30 | 0.265 |  | | | |
| 99102\_at | Usp9x | ubiquitin specific protease 9, X chromosome | chrX | -1.037 | 30 | 0.363 |  | | | |
| 99600\_at | Ptov1 | prostate tumor over expressed gene 1 | chr7 | -1.047 | 30 | 0.078 |  | | | |
| 99961\_s\_at | Cdc2l2 | cell division cycle 2 homolog (S. pombe)-like 2 | chr4 | -1.587 | 30 | 0.025 |  | | | |
| 99970\_at | Ptpn21 | protein tyrosine phosphatase, non-receptor type 21 | chr12 | -1.262 | 30 | 0.008 |  | | | |
| \* Positive log2 fold changes represent genes expressed higher in FL-HSC; Negative log2 fold changes represent genes expressed higher in adult HSC (fold change=2 is equivalent to log2 fold change=1) | | | | | | | | | | |
|  |  |  |  |  |  |  |  |  |  |  |
