# Supplementary material for: Molecular Signatures of Proliferation and Quiescence in Hematopoietic Stem Cells
Source: PLoS Biol. 2004 Sep 28;2(10):e301. doi: 10.1371/journal.pbio.0020301 (PMC520599; doi:10.1371/journal.pbio.0020301)
Supplement: Table S7 — (296 KB HTML). [file pbio.0020301.st007.html]

|  |  | Q-sig |  |  |  |  |  |  |  |  |
| Probe Set ID | Gene Symbol | Gene name | Chromosome | Log2 Fold Change (FL-HSC vs Adult HSC)\* | Day of max (TOM) | p-value of ANOVA (time course) |  | | | |
| 100533\_s\_at | Crem | cAMP responsive element modulator | --- | -5.967 | 0 | 0.008 |  | | | |
| 101515\_at | Acox1 | acyl-Coenzyme A oxidase 1, palmitoyl | --- | -3.857 | 0 | 0.018 |  | | | |
| 102156\_f\_at | NoneAvailable | --- | chr6 | -3.755 | 0 | 0.005 |  | | | |
| 102209\_at | Nfatc1 | nuclear factor of activated T-cells, cytoplasmic 1 | chr18 | -2.297 | 0 | 0.009 |  | | | |
| 102326\_at | Ncf2 | neutrophil cytosolic factor 2 | chr1 | -1.48 | 0 | 0.013 |  | | | |
| 102356\_at | Wdr23 | WD repeat domain 23 | chr14 | -1.437 | 0 | 0.036 |  | | | |
| 102955\_at | Nfil3 | nuclear factor, interleukin 3, regulated | chr13 | -2.259 | 0 | 0.012 |  | | | |
| 103037\_at | Ctf1 | cardiotrophin 1 | --- | -1.295 | 0 | 0.012 |  | | | |
| 103226\_at | Mrc1 | mannose receptor, C type 1 | chr2 | -2.184 | 0 | 0.003 |  | | | |
| 103427\_at | Fbxl3a | F-box and leucine-rich repeat protein 3a | chr14 | -1.769 | 0 | 0.042 |  | | | |
| 103990\_at | Fosb | FBJ osteosarcoma oncogene B | chr7 | -4.608 | 0 | 0.012 |  | | | |
| 104252\_at | AU020206 | expressed sequence AU020206 | chr7 | -1.192 | 0 | 0.034 |  | | | |
| 104516\_at | Cldn5 | claudin 5 | chr16 | -1.272 | 0 | 0.048 |  | | | |
| 104562\_at | 5730403M16Rik | RIKEN cDNA 5730403M16 gene | chr7 | -1.253 | 0 | 0.009 |  | | | |
| 104598\_at | Dusp1 | dual specificity phosphatase 1 | chr17 | -2.35 | 0 | 0.018 |  | | | |
| 104625\_at | Dnajb6 | DnaJ (Hsp40) homolog, subfamily B, member 6 | chr16 | -1.482 | 0 | 0.041 |  | | | |
| 104719\_at | Slc12a7 | solute carrier family 12, member 7 | chr13 | -1.166 | 0 | 0.039 |  | | | |
| 160104\_at | Hsd3b7 | hydroxy-delta-5-steroid dehydrogenase, 3 beta- and steroid delta-isomerase 7 | chr7 | -1.424 | 0 | 0.022 |  | | | |
| 160526\_s\_at | Crem | cAMP responsive element modulator | --- | -5.103 | 0 | 0.005 |  | | | |
| 160573\_at | Hccs | holocytochrome c synthetase | --- | -2.08 | 0 | 0.025 |  | | | |
| 160901\_at | Fos | FBJ osteosarcoma oncogene | chr12 | -3.257 | 0 | 0.024 |  | | | |
| 92248\_at | Nr4a2 | nuclear receptor subfamily 4, group A, member 2 | chr2 | -5.877 | 0 | 0.007 |  | | | |
| 92256\_at | Fdft1 | farnesyl diphosphate farnesyl transferase 1 | --- | -1.15 | 0 | 0.003 |  | | | |
| 92270\_at | Tro | trophinin | chrX | -1.72 | 0 | 0.006 |  | | | |
| 92558\_at | Vcam1 | vascular cell adhesion molecule 1 | chr3 | -4.435 | 0 | 0.005 |  | | | |
| 92830\_s\_at | NoneAvailable | --- | --- | -3.484 | 0 | 0.001 |  | | | |
| 93093\_at | Mcl1 | myeloid cell leukemia sequence 1 | chr3 | -2.974 | 0 | 0.018 |  | | | |
| 93120\_f\_at | H2-K | histocompatibility 2, K region | chr17 | -3.637 | 0 | 0.003 |  | | | |
| 93179\_at | B830009D23Rik | RIKEN cDNA B830009D23 gene | chr2 | -1.837 | 0 | 0.017 |  | | | |
| 93326\_at | Tm4sf2 | transmembrane 4 superfamily member 2 | chrX | -1.259 | 0 | 0.02 |  | | | |
| 93498\_s\_at | Aplp2 | amyloid beta (A4) precursor-like protein 2 | chr9 | -2.613 | 0 | 0.02 |  | | | |
| 93705\_at | Chrnb1 | cholinergic receptor, nicotinic, beta polypeptide 1 (muscle) | --- | -2.651 | 0 | 0.016 |  | | | |
| 93714\_f\_at | H2-Q7 | histocompatibility 2, Q region locus 7 | chr17 | -3.128 | 0 | 0.001 |  | | | |
| 93907\_f\_at | NoneAvailable | --- | --- | -1.754 | 0 | 0.001 |  | | | |
| 93964\_s\_at | Ddx6 | DEAD (Asp-Glu-Ala-Asp) box polypeptide 6 | chr9 | -3.009 | 0 | 0.03 |  | | | |
| 94345\_at | Il6st | interleukin 6 signal transducer | chr13 | -3.717 | 0 | 0 |  | | | |
| 94428\_at | Ilvbl | ilvB (bacterial acetolactate synthase)-like | chr10 | -1.305 | 0 | 0.004 |  | | | |
| 94834\_at | Ctsh | cathepsin H | chr9 | -1.968 | 0 | 0.045 |  | | | |
| 94948\_at | Trip6 | thyroid hormone receptor interactor 6 | chr5 | -1.016 | 0 | 0.02 |  | | | |
| 94991\_at | Synpo | synaptopodin | chr18 | -1.276 | 0 | 0.018 |  | | | |
| 95102\_at | Scotin-pending | scotin gene | chr9 | -2.721 | 0 | 0.006 |  | | | |
| 95449\_at | 2310075G12Rik | RIKEN cDNA 2310075G12 gene | chr11 | -1.383 | 0 | 0.016 |  | | | |
| 95520\_at | 2310061B02Rik | RIKEN cDNA 2310061B02 gene | chr1 | -2.378 | 0 | 0.04 |  | | | |
| 96049\_at | Bgn | biglycan | --- | -1.681 | 0 | 0.008 |  | | | |
| 96186\_at | Lrp10 | low-density lipoprotein receptor-related protein 10 | chr14 | -3.157 | 0 | 0.013 |  | | | |
| 96530\_at | NoneAvailable | Mus musculus transcribed sequences | --- | -1.094 | 0 | 0.002 |  | | | |
| 96886\_at | Stab1 | stabilin 1 | chr14 | -2.451 | 0 | 0.017 |  | | | |
| 96912\_s\_at | Ctla2a | cytotoxic T lymphocyte-associated protein 2 alpha | chr13 | -3.703 | 0 | 0.031 |  | | | |
| 97181\_f\_at | NoneAvailable | --- | --- | -1.659 | 0 | 0.002 |  | | | |
| 97336\_at | Ctsf | cathepsin F | chr19 | -1.534 | 0 | 0.002 |  | | | |
| 97448\_at | NoneAvailable | Mus musculus cDNA clone MGC:65558 IMAGE:6485174, complete cds | chr11 | -1.982 | 0 | 0.019 |  | | | |
| 97798\_at | 4930504E06Rik | RIKEN cDNA 4930504E06 gene | chr3 | -1.169 | 0 | 0.001 |  | | | |
| 98083\_at | Copeb | core promoter element binding protein | chr13 | -2.963 | 0 | 0 |  | | | |
| 98088\_at | Cd14 | CD14 antigen | --- | -3.218 | 0 | 0.018 |  | | | |
| 98254\_f\_at | NoneAvailable | --- | --- | -1.54 | 0 | 0.002 |  | | | |
| 98369\_f\_at | NoneAvailable | --- | --- | -1.265 | 0 | 0.002 |  | | | |
| 98451\_at | Dnajb10 | DnaJ (Hsp40) homolog, subfamily B, member 10 | chr1 | -3.902 | 0 | 0.002 |  | | | |
| 100587\_f\_at | 5730403B10Rik | RIKEN cDNA 5730403B10 gene | chr16 | -2.058 | 1 | 0.009 |  | | | |
| 100998\_at | H2-Ab1 | histocompatibility 2, class II antigen A, beta 1 | chr17 | -1.116 | 1 | 0.026 |  | | | |
| 101568\_at | NoneAvailable | Mus musculus mRNA similar to proline synthetase co-transcribed (cDNA clone MGC:59396 IMAGE:6504579), complete cds | chr8 | -1.703 | 1 | 0.024 |  | | | |
| 101963\_at | Ctsl | cathepsin L | chr13 | -2.894 | 1 | 0.022 |  | | | |
| 101971\_at | 2500002L14Rik | RIKEN cDNA 2500002L14 gene | --- | -1.663 | 1 | 0.005 |  | | | |
| 101990\_at | Ldh2 | lactate dehydrogenase 2, B chain | chr6 | -1.289 | 1 | 0.001 |  | | | |
| 102332\_at | Ulk1 | Unc-51 like kinase 1 (C. elegans) | chr5 | -2.225 | 1 | 0.009 |  | | | |
| 102860\_at | Serpina3g | serine (or cysteine) proteinase inhibitor, clade A, member 3G | chr12 | -4.051 | 1 | 0.013 |  | | | |
| 102906\_at | Tgtp | T-cell specific GTPase | chr11 | -5.973 | 1 | 0.001 |  | | | |
| 102960\_at | Rga | recombination activating gene 1 gene activation | chr3 | -1.165 | 1 | 0.049 |  | | | |
| 103353\_f\_at | Cyp4b1 | cytochrome P450, family 4, subfamily b, polypeptide 1 | chr4 | -2.127 | 1 | 0.004 |  | | | |
| 103634\_at | Isgf3g | interferon dependent positive acting transcription factor 3 gamma | --- | -1.108 | 1 | 0.002 |  | | | |
| 103899\_at | Atp11a | ATPase, class VI, type 11A | chr8 | -1.465 | 1 | 0.049 |  | | | |
| 104597\_at | Gbp2 | guanylate nucleotide binding protein 2 | chr3 | -2.148 | 1 | 0.021 |  | | | |
| 104735\_at | AW538430 | expressed sequence AW538430 | chr14 | -1.525 | 1 | 0.049 |  | | | |
| 160127\_at | Ccng1 | cyclin G1 | chr11 | -1.675 | 1 | 0.007 |  | | | |
| 160502\_at | Creg | cellular repressor of E1A-stimulated genes | --- | -2.676 | 1 | 0.03 |  | | | |
| 160519\_at | Timp3 | tissue inhibitor of metalloproteinase 3 | chr10 | -3.836 | 1 | 0.007 |  | | | |
| 160724\_at | Usp49 | ubiquitin specific protease 49 | --- | -2.112 | 1 | 0 |  | | | |
| 160783\_at | D14Ertd436e | DNA segment, Chr 14, ERATO Doi 436, expressed | chr14 | -1.857 | 1 | 0.033 |  | | | |
| 160933\_at | Igtp | interferon gamma induced GTPase | chr11 | -3.814 | 1 | 0 |  | | | |
| 161666\_f\_at | Gadd45b | growth arrest and DNA-damage-inducible 45 beta | chr10 | -2.691 | 1 | 0.042 |  | | | |
| 162044\_f\_at | Cyp4b1 | cytochrome P450, family 4, subfamily b, polypeptide 1 | --- | -2.532 | 1 | 0.007 |  | | | |
| 92263\_at | Grcb | gene rich cluster, B gene | chr6 | -1.939 | 1 | 0.002 |  | | | |
| 92440\_at | Irf6 | interferon regulatory factor 6 | chr1 | -4.326 | 1 | 0.031 |  | | | |
| 92653\_at | D530037H12Rik | RIKEN cDNA D530037H12 gene | chr1 | -1.338 | 1 | 0.01 |  | | | |
| 92780\_f\_at | NoneAvailable | --- | --- | -2.077 | 1 | 0.017 |  | | | |
| 92847\_s\_at | M6pr | mannose-6-phosphate receptor, cation dependent | chr6 | -1.096 | 1 | 0.043 |  | | | |
| 92866\_at | H2-Aa | histocompatibility 2, class II antigen A, alpha | chr17 | -3.869 | 1 | 0.036 |  | | | |
| 92926\_at | Mpl | myeloproliferative leukemia virus oncogene | --- | -1.119 | 1 | 0.003 |  | | | |
| 93011\_at | Gabarapl1 | gamma-aminobutyric acid (GABA(A)) receptor-associated protein-like 1 | chr6 | -2.046 | 1 | 0.019 |  | | | |
| 93020\_at | Rex3 | reduced expression 3 | chrX | -1.019 | 1 | 0.009 |  | | | |
| 93039\_at | 1190003P12Rik | RIKEN cDNA 1190003P12 gene | chr15 | -1.465 | 1 | 0.029 |  | | | |
| 93324\_at | Zfp36l1 | zinc finger protein 36, C3H type-like 1 | chr12 | -2.308 | 1 | 0.025 |  | | | |
| 93543\_f\_at | Gstm1 | glutathione S-transferase, mu 1 | chr5 | -1.363 | 1 | 0.008 |  | | | |
| 94269\_at | Rabac1 | Rab acceptor 1 (prenylated) | chr7 | -1.557 | 1 | 0.015 |  | | | |
| 94270\_at | Krt1-18 | keratin complex 1, acidic, gene 18 | --- | -3.531 | 1 | 0.013 |  | | | |
| 94821\_at | Xbp1 | X-box binding protein 1 | --- | -2.107 | 1 | 0.021 |  | | | |
| 94835\_f\_at | Tubb2 | tubulin, beta 2 | chr13 | -2.52 | 1 | 0.002 |  | | | |
| 94881\_at | Cdkn1a | cyclin-dependent kinase inhibitor 1A (P21) | chr17 | -2.727 | 1 | 0.002 |  | | | |
| 95505\_at | Tor1b | torsin family 1, member B | --- | -1.061 | 1 | 0.039 |  | | | |
| 95508\_at | Nckap1 | NCK-associated protein 1 | chr2 | -4.402 | 1 | 0.021 |  | | | |
| 95737\_at | 1200015A19Rik | RIKEN cDNA 1200015A19 gene | chr4 | -2.325 | 1 | 0.023 |  | | | |
| 96146\_at | Btg3 | B-cell translocation gene 3 | chr16 | -2.704 | 1 | 0.035 |  | | | |
| 96596\_at | Ndrl | N-myc downstream regulated-like | chr15 | -4.908 | 1 | 0.007 |  | | | |
| 96614\_at | 4933426M11Rik | RIKEN cDNA 4933426M11 gene | chr12 | -1.2 | 1 | 0.039 |  | | | |
| 96703\_at | Maged1 | melanoma antigen, family D, 1 | chrX | -2.002 | 1 | 0.003 |  | | | |
| 96728\_at | DXImx38e | DNA segment, Chr X, Immunex 38, expressed | chrX | -2.856 | 1 | 0.001 |  | | | |
| 96752\_at | Icam1 | intercellular adhesion molecule | chr9 | -2.045 | 1 | 0.024 |  | | | |
| 96764\_at | Iigp-pending | interferon-inducible GTPase | --- | -5.077 | 1 | 0.009 |  | | | |
| 96876\_at | Laptm4a | lysosomal-associated protein transmembrane 4A | chr12 | -1.037 | 1 | 0.039 |  | | | |
| 96935\_at | 2700030M23Rik | RIKEN cDNA 2700030M23 gene | chr4 | -2.968 | 1 | 0.003 |  | | | |
| 97409\_at | Ifi1 | interferon inducible protein 1 | chr11 | -1.031 | 1 | 0.034 |  | | | |
| 97540\_f\_at | H2-D1 | histocompatibility 2, D region locus 1 | --- | -3.787 | 1 | 0.002 |  | | | |
| 97549\_at | Cfl2 | cofilin 2, muscle | chr12 | -2.749 | 1 | 0.03 |  | | | |
| 97890\_at | Sgk | serum/glucocorticoid regulated kinase | chr10 | -3.498 | 1 | 0.048 |  | | | |
| 97908\_at | 1110007A06Rik | RIKEN cDNA 1110007A06 gene | chr6 | -1.119 | 1 | 0.04 |  | | | |
| 97973\_at | Tal1 | T-cell acute lymphocytic leukemia 1 | chr4 | -1.383 | 1 | 0.032 |  | | | |
| 98067\_at | Cdkn1a | cyclin-dependent kinase inhibitor 1A (P21) | chr17 | -2.741 | 1 | 0.001 |  | | | |
| 98410\_at | Gtpi-pending | interferon-g induced GTPase | chr11 | -2.836 | 1 | 0.046 |  | | | |
| 98472\_at | H2-T23 | histocompatibility 2, T region locus 23 | --- | -1.61 | 1 | 0 |  | | | |
| 99109\_at | Ier2 | immediate early response 2 | chr8 | -2.892 | 1 | 0.003 |  | | | |
| 99133\_at | Slc3a2 | solute carrier family 3 (activators of dibasic and neutral amino acid transport), member 2 | chr19 | -1.113 | 1 | 0.018 |  | | | |
| 99366\_at | E030024M05Rik | RIKEN cDNA E030024M05 gene | chr12 | -2.996 | 1 | 0.014 |  | | | |
| 99532\_at | Tob1 | transducer of ErbB-2.1 | chr11 | -5.048 | 1 | 0.008 |  | | | |
| 100030\_at | Upp1 | uridine phosphorylase 1 | chr11 | -1.097 | 10 | 0.005 |  | | | |
| 100134\_at | Eng | endoglin | chr2 | -1.282 | 10 | 0.012 |  | | | |
| 100136\_at | Lamp2 | lysosomal membrane glycoprotein 2 | chrX | -1.749 | 10 | 0.025 |  | | | |
| 100475\_at | Trim25 | tripartite motif protein 25 | --- | -1.645 | 10 | 0 |  | | | |
| 100606\_at | Prnp | prion protein | --- | -1.719 | 10 | 0.036 |  | | | |
| 100635\_at | Sara | SAR1a gene homolog (S. cerevisiae) | chr10 | -1.295 | 10 | 0.009 |  | | | |
| 100973\_i\_at | Ccl27 | chemokine (C-C motif) ligand 27 | --- | -3.092 | 10 | 0 |  | | | |
| 100988\_at | Bcl2l11 | BCL2-like 11 (apoptosis facilitator) | chr2 | -1.027 | 10 | 0.014 |  | | | |
| 101079\_at | Nxf1 | nuclear RNA export factor 1 homolog (S. cerevisiae) | chr19 | -2.424 | 10 | 0.003 |  | | | |
| 101144\_at | Il18r1 | interleukin 18 receptor 1 | --- | -1.522 | 10 | 0.037 |  | | | |
| 101186\_at | Ppnr-pending | per-pentamer repeat gene | chr19 | -1.941 | 10 | 0.019 |  | | | |
| 101441\_i\_at | Itpr5 | inositol 1,4,5-triphosphate receptor 5 | chr6 | -3.278 | 10 | 0.019 |  | | | |
| 101836\_at | Ppm1b | protein phosphatase 1B, magnesium dependent, beta isoform | chr17 | -1.457 | 10 | 0.007 |  | | | |
| 101884\_at | Xlr4 | X-linked lymphocyte-regulated 4 | chrX | -2.072 | 10 | 0.038 |  | | | |
| 101943\_at | Tceb3 | transcription elongation factor B (SIII), polypeptide 3 | chr4 | -1.496 | 10 | 0.002 |  | | | |
| 101947\_at | Nakap95-pending | neighbor of A-kinase anchoring protein 95 | chr17 | -1.799 | 10 | 0.001 |  | | | |
| 102279\_at | 1300004C08Rik | RIKEN cDNA 1300004C08 gene | chr9 | -2.168 | 10 | 0.008 |  | | | |
| 102313\_at | Gch | GTP cyclohydrolase 1 | chr14 | -4.039 | 10 | 0.005 |  | | | |
| 102658\_at | Il1r2 | interleukin 1 receptor, type II | chr1 | -1.46 | 10 | 0 |  | | | |
| 102663\_at | Plaur | urokinase plasminogen activator receptor | --- | -2.014 | 10 | 0.036 |  | | | |
| 102781\_at | Ccnl2 | cyclin L2 | chr4 | -1.306 | 10 | 0.005 |  | | | |
| 102787\_at | Gpr56 | G protein-coupled receptor 56 | chr8 | -1.614 | 10 | 0.021 |  | | | |
| 102836\_at | Pps | putative phosphatase | chr11 | -1.69 | 10 | 0 |  | | | |
| 103015\_at | Bcl6 | B-cell leukemia/lymphoma 6 | chr16 | -4.658 | 10 | 0.02 |  | | | |
| 103210\_at | Csf2rb2 | colony stimulating factor 2 receptor, beta 2, low-affinity (granulocyte-macrophage) | chr15 | -1.2 | 10 | 0.02 |  | | | |
| 103254\_at | Fln29-pending | FLN29 gene product | chr5 | -2.179 | 10 | 0.011 |  | | | |
| 103422\_at | Cd1d1 | CD1d1 antigen | chr3 | -2.191 | 10 | 0.001 |  | | | |
| 103443\_at | Aim1 | absent in melanoma 1 | chr10 | -1.49 | 10 | 0.005 |  | | | |
| 103518\_at | Ctla2b | cytotoxic T lymphocyte-associated protein 2 beta | chr13 | -5.219 | 10 | 0.015 |  | | | |
| 103596\_at | Dgka | diacylglycerol kinase, alpha | chr10 | -3.305 | 10 | 0 |  | | | |
| 103672\_at | 2410141M05Rik | RIKEN cDNA 2410141M05 gene | chr11 | -1.198 | 10 | 0.001 |  | | | |
| 103812\_at | Clca1 | chloride channel calcium activated 1 | chr3 | -4.597 | 10 | 0.005 |  | | | |
| 103895\_at | AW549877 | expressed sequence AW549877 | chr15 | -1.037 | 10 | 0.03 |  | | | |
| 104144\_at | Gtpbp2 | GTP binding protein 2 | chr17 | -1.316 | 10 | 0.016 |  | | | |
| 104165\_at | Vnn1 | vanin 1 | --- | -2.98 | 10 | 0.003 |  | | | |
| 104206\_at | 0610012A05Rik | RIKEN cDNA 0610012A05 gene | chr15 | -7.151 | 10 | 0.003 |  | | | |
| 104256\_at | Pscdbp | pleckstrin homology, Sec7 and coiled-coil domains, binding protein | chr2 | -2.279 | 10 | 0.022 |  | | | |
| 104257\_g\_at | Pscdbp | pleckstrin homology, Sec7 and coiled-coil domains, binding protein | chr2 | -2.012 | 10 | 0.004 |  | | | |
| 104263\_at | 9330177P20Rik | RIKEN cDNA 9330177P20 gene | chr4 | -1.251 | 10 | 0.004 |  | | | |
| 104311\_at | 1300013G12Rik | RIKEN cDNA 1300013G12 gene | chr1 | -1.417 | 10 | 0.024 |  | | | |
| 104371\_at | Dgat1 | diacylglycerol O-acyltransferase 1 | chr15 | -3.266 | 10 | 0 |  | | | |
| 104453\_at | NoneAvailable | Mus musculus cDNA clone IMAGE:6433799, partial cds | chr11 | -1.124 | 10 | 0.03 |  | | | |
| 104572\_at | Etohd2 | ethanol decreased 2 | chr13 | -1.512 | 10 | 0.001 |  | | | |
| 104677\_at | LOC227619 | hypothetical protein LOC227619 | chr2 | -1.818 | 10 | 0.046 |  | | | |
| 104701\_at | Bhlhb2 | basic helix-loop-helix domain containing, class B2 | chr6 | -3.085 | 10 | 0.023 |  | | | |
| 104741\_at | 9530098M12Rik | RIKEN cDNA 9530098M12 gene | chrX | -1.016 | 10 | 0.015 |  | | | |
| 104745\_at | Arl6ip2 | ADP-ribosylation factor-like 6 interacting protein 2 | chr17 | -1.189 | 10 | 0.045 |  | | | |
| 160088\_at | Fmo5 | flavin containing monooxygenase 5 | chr3 | -1.409 | 10 | 0.019 |  | | | |
| 160099\_at | Lgals4 | lectin, galactose binding, soluble 4 | chr7 | -1.876 | 10 | 0.001 |  | | | |
| 160151\_i\_at | 1200009B18Rik | RIKEN cDNA 1200009B18 gene | chr6 | -1.735 | 10 | 0.029 |  | | | |
| 160264\_s\_at | 1500036F01Rik | RIKEN cDNA 1500036F01 gene | chr1 | -1.44 | 10 | 0.029 |  | | | |
| 160287\_at | Map1lc3 | microtubule-associated protein 1 light chain 3 | chr14 | -1.669 | 10 | 0.02 |  | | | |
| 160393\_at | 4930555L11Rik | RIKEN cDNA 4930555L11 gene | chr6 | -2.283 | 10 | 0.001 |  | | | |
| 160495\_at | Ahr | aryl-hydrocarbon receptor | chr12 | -3.189 | 10 | 0.004 |  | | | |
| 160834\_at | 1110032C13Rik | RIKEN cDNA 1110032C13 gene | chr7 | -4.49 | 10 | 0.04 |  | | | |
| 160920\_at | Bcl2l2 | Bcl2-like 2 | chr14 | -1.72 | 10 | 0.02 |  | | | |
| 160965\_at | AA793972 | EST AA793972 | chr5 | -3.294 | 10 | 0.001 |  | | | |
| 160977\_at | Arhgef5 | Rho guanine nucleotide exchange factor (GEF) 5 | chr6 | -3.899 | 10 | 0.031 |  | | | |
| 161081\_at | Cpeb2 | cytoplasmic polyadenylation element binding protein 2 | chr5 | -3.809 | 10 | 0.02 |  | | | |
| 161109\_at | 1110017P05Rik | RIKEN cDNA 1110017P05 gene | --- | -1.394 | 10 | 0.011 |  | | | |
| 161113\_at | Esr1 | estrogen receptor 1 (alpha) | chr10 | -1.744 | 10 | 0.007 |  | | | |
| 161551\_f\_at | Riok3 | RIO kinase 3 (yeast) | --- | -1.464 | 10 | 0.012 |  | | | |
| 161610\_at | Ndr2 | N-myc downstream regulated 2 | --- | -4.394 | 10 | 0.001 |  | | | |
| 161689\_f\_at | Il1r2 | interleukin 1 receptor, type II | chr1 | -3.446 | 10 | 0 |  | | | |
| 161814\_f\_at | Rnf19 | ring finger protein (C3HC4 type) 19 | chr15 | -1.939 | 10 | 0.035 |  | | | |
| 161980\_f\_at | Bag3 | Bcl2-associated athanogene 3 | chr7 | -4.017 | 10 | 0.037 |  | | | |
| 162041\_f\_at | NoneAvailable | --- | --- | -1.038 | 10 | 0.01 |  | | | |
| 162206\_f\_at | Socs3 | suppressor of cytokine signaling 3 | --- | -4.307 | 10 | 0.013 |  | | | |
| 92542\_at | D4Wsu53e | DNA segment, Chr 4, Wayne State University 53, expressed | chr4 | -1.236 | 10 | 0.014 |  | | | |
| 92877\_at | Tgfbi | transforming growth factor, beta induced | chr13 | -1.788 | 10 | 0.029 |  | | | |
| 92992\_i\_at | 5730497N03Rik | RIKEN cDNA 5730497N03 gene | chr12 | -1.16 | 10 | 0.027 |  | | | |
| 92993\_r\_at | 5730497N03Rik | RIKEN cDNA 5730497N03 gene | chr12 | -1.744 | 10 | 0.003 |  | | | |
| 93104\_at | Btg1 | B-cell translocation gene 1, anti-proliferative | chr10 | -3.713 | 10 | 0.005 |  | | | |
| 93193\_at | Adrb2 | adrenergic receptor, beta 2 | chr18 | -1.617 | 10 | 0.021 |  | | | |
| 93274\_at | Clk | CDC-like kinase | chr1 | -1.211 | 10 | 0.031 |  | | | |
| 93311\_at | Clk3 | CDC-like kinase 3 | chr9 | -1.389 | 10 | 0.003 |  | | | |
| 93315\_at | Map2k3 | mitogen activated protein kinase kinase 3 | chr11 | -2.248 | 10 | 0.044 |  | | | |
| 93414\_at | Abcb1b | ATP-binding cassette, sub-family B (MDR/TAP), member 1B | chr5 | -1.239 | 10 | 0.019 |  | | | |
| 93424\_at | NoneAvailable | Mus musculus, Similar to KIAA0916 protein, clone IMAGE:4022573, mRNA | chr14 | -1.14 | 10 | 0.024 |  | | | |
| 93440\_at | 4930564D15Rik | RIKEN cDNA 4930564D15 gene | chr3 | -1.001 | 10 | 0.017 |  | | | |
| 93520\_at | Srrm1 | serine/arginine repetitive matrix 1 | --- | -1.338 | 10 | 0.018 |  | | | |
| 93753\_at | Litaf | LPS-induced TN factor | chr16 | -1.716 | 10 | 0.013 |  | | | |
| 93852\_at | Mef2a | myocyte enhancer factor 2A | chr7 | -2.066 | 10 | 0.044 |  | | | |
| 93914\_at | Il1r1 | interleukin 1 receptor, type I | chr1 | -1.087 | 10 | 0.006 |  | | | |
| 93965\_r\_at | Ddx6 | DEAD (Asp-Glu-Ala-Asp) box polypeptide 6 | chr9 | -3.007 | 10 | 0.007 |  | | | |
| 93975\_at | 1300002F13Rik | RIKEN cDNA 1300002F13 gene | chr4 | -6.877 | 10 | 0 |  | | | |
| 94192\_at | Gdap10 | ganglioside-induced differentiation-associated-protein 10 | chr12 | -2.38 | 10 | 0.047 |  | | | |
| 94264\_at | Raf1 | v-raf-1 leukemia viral oncogene 1 | chr6 | -1.251 | 10 | 0.001 |  | | | |
| 94331\_at | Stat6 | signal transducer and activator of transcription 6 | chr10 | -2.796 | 10 | 0 |  | | | |
| 94483\_at | Csnk2a2 | casein kinase II, alpha 2, polypeptide | chr8 | -1.076 | 10 | 0.04 |  | | | |
| 94689\_at | C79248 | expressed sequence C79248 | --- | -1.2 | 10 | 0.015 |  | | | |
| 94780\_at | Zfp288 | zinc finger protein 288 | chr16 | -4.353 | 10 | 0.034 |  | | | |
| 94818\_at | Ogt | O-linked N-acetylglucosamine (GlcNAc) transferase (UDP-N-acetylglucosamine:polypeptide-N-acetylglucosaminyl transferase) | chrX | -1.371 | 10 | 0.004 |  | | | |
| 94830\_at | BC005537 | cDNA sequence BC005537 | chr13 | -1.83 | 10 | 0.008 |  | | | |
| 94899\_at | Rhoip3-pending | Rho interacting protein 3 | chr11 | -1.473 | 10 | 0.042 |  | | | |
| 94928\_at | Tnfrsf1b | tumor necrosis factor receptor superfamily, member 1b | --- | -1.859 | 10 | 0.009 |  | | | |
| 94939\_at | Cd53 | CD53 antigen | chr3 | -1.067 | 10 | 0.004 |  | | | |
| 94980\_at | Dusp11 | dual specificity phosphatase 11 (RNA/RNP complex 1-interacting) | chr6 | -1.6 | 10 | 0.004 |  | | | |
| 95023\_at | BC023957 | cDNA sequence BC023957 | chr9 | -1.742 | 10 | 0 |  | | | |
| 95119\_at | 1110038D17Rik | RIKEN cDNA 1110038D17 gene | chr10 | -1.366 | 10 | 0.009 |  | | | |
| 95287\_at | NoneAvailable | Mus musculus RIKEN cDNA 4930471C18 gene, mRNA (cDNA clone IMAGE:4487650), partial cds | chr6 | -1.232 | 10 | 0.032 |  | | | |
| 95444\_at | 4930579A11Rik | RIKEN cDNA 4930579A11 gene | chr11 | -2.862 | 10 | 0.016 |  | | | |
| 95489\_at | Fliih | flightless I homolog (Drosophila) | chr11 | -1.074 | 10 | 0.042 |  | | | |
| 95521\_s\_at | Zfp68 | Zinc finger protein 68 | chr5 | -2.132 | 10 | 0.035 |  | | | |
| 95564\_at | BC018601 | cDNA sequence BC018601 | chr11 | -2.789 | 10 | 0.032 |  | | | |
| 95586\_at | P2rx4 | purinergic receptor P2X, ligand-gated ion channel 4 | chr5 | -1.711 | 10 | 0 |  | | | |
| 95655\_at | 5830411E10Rik | RIKEN cDNA 5830411E10 gene | chr1 | -1.472 | 10 | 0.036 |  | | | |
| 95917\_at | NoneAvailable | Mus musculus transcribed sequences | chr8 | -4.563 | 10 | 0.041 |  | | | |
| 96176\_at | Arih2 | ariadne homolog 2 (Drosophila) | chr9 | -1.874 | 10 | 0.022 |  | | | |
| 96189\_at | 2410141K03Rik | RIKEN cDNA 2410141K03 gene | --- | -2.437 | 10 | 0.013 |  | | | |
| 96534\_at | Vldlr | very low density lipoprotein receptor | chr19 | -1.008 | 10 | 0 |  | | | |
| 96813\_f\_at | DXImx46e | DNA segment, Chr X, Immunex 46, expressed | chrX | -1.437 | 10 | 0.027 |  | | | |
| 97118\_at | 1810028B20Rik | RIKEN cDNA 1810028B20 gene | chr19 | -1.614 | 10 | 0.036 |  | | | |
| 97285\_f\_at | Ubxdc2 | UBX domain-containing 2 | chr17 | -2.548 | 10 | 0 |  | | | |
| 97297\_at | 1500036F01Rik | RIKEN cDNA 1500036F01 gene | chr1 | -1.772 | 10 | 0.001 |  | | | |
| 97319\_at | Rrad | Ras-related associated with diabetes | chr8 | -2.544 | 10 | 0.005 |  | | | |
| 97349\_at | 4930488L10Rik | RIKEN cDNA 4930488L10 gene | chr12 | -2.819 | 10 | 0.04 |  | | | |
| 97429\_at | Snrk | SNF related kinase | chr9 | -1.935 | 10 | 0.021 |  | | | |
| 97843\_at | Ncoa4 | nuclear receptor coactivator 4 | chr12 | -1.253 | 10 | 0.003 |  | | | |
| 97897\_at | NoneAvailable | Mus musculus, clone IMAGE:6430978, mRNA | chr13 | -1.708 | 10 | 0.027 |  | | | |
| 98000\_at | Ly64 | lymphocyte antigen 64 | chr16 | -2.084 | 10 | 0.036 |  | | | |
| 98018\_at | Procr | protein C receptor, endothelial | chr2 | -4.119 | 10 | 0.038 |  | | | |
| 98461\_at | 1200014P03Rik | RIKEN cDNA 1200014P03 gene | chr17 | -1.307 | 10 | 0.007 |  | | | |
| 98533\_at | Cyb5 | cytochrome b-5 | chr18 | -1.221 | 10 | 0.003 |  | | | |
| 98882\_s\_at | Ndel1 | nuclear distribution gene E-like homolog 1 (A. nidulans) | chr11 | -1.562 | 10 | 0 |  | | | |
| 98884\_r\_at | Ndel1 | nuclear distribution gene E-like homolog 1 (A. nidulans) | chr11 | -2.864 | 10 | 0.022 |  | | | |
| 98926\_at | Vamp2 | vesicle-associated membrane protein 2 | chr11 | -2.233 | 10 | 0 |  | | | |
| 98951\_at | D8Ertd325e | DNA segment, Chr 8, ERATO Doi 325, expressed | chr8 | -1.148 | 10 | 0.009 |  | | | |
| 99045\_at | Eno2 | enolase 2, gamma neuronal | --- | -1.634 | 10 | 0.008 |  | | | |
| 99100\_at | Stat3 | signal transducer and activator of transcription 3 | chr11 | -1.118 | 10 | 0.022 |  | | | |
| 99103\_at | Irf3 | interferon regulatory factor 3 | chr7 | -1.113 | 10 | 0.042 |  | | | |
| 99143\_at | Tgoln1 | trans-golgi network protein | chr6 | -2.457 | 10 | 0.021 |  | | | |
| 99184\_at | Csad | cysteine sulfinic acid decarboxylase | --- | -2.259 | 10 | 0.001 |  | | | |
| 99187\_f\_at | 2010315L10Rik | RIKEN cDNA 2010315L10 gene | chr8 | -1.207 | 10 | 0.004 |  | | | |
| 99188\_at | 2010315L10Rik | RIKEN cDNA 2010315L10 gene | chr8 | -1.031 | 10 | 0.002 |  | | | |
| 99347\_f\_at | NoneAvailable | Mus musculus transcribed sequences | --- | -2.205 | 10 | 0.048 |  | | | |
| 99445\_at | 1110028E10Rik | RIKEN cDNA 1110028E10 gene | chr9 | -1.202 | 10 | 0.035 |  | | | |
| 99985\_at | Txnrd1 | thioredoxin reductase 1 | chr10 | -1.205 | 10 | 0.045 |  | | | |
| 100482\_at | BC023040 | cDNA sequence BC023040 | chr17 | -1.285 | 30 | 0.005 |  | | | |
| 100583\_at | Igh-VJ558 | immunoglobulin heavy chain (J558 family) | chr12 | -1.148 | 30 | 0.003 |  | | | |
| 102224\_at | Igf1r | insulin-like growth factor I receptor | --- | -1.399 | 30 | 0.011 |  | | | |
| 102302\_at | Bckdhb | branched chain ketoacid dehydrogenase E1, beta polypeptide | chr9 | -1.776 | 30 | 0.019 |  | | | |
| 102789\_at | Gata2 | GATA binding protein 2 | chr6 | -1.995 | 30 | 0.048 |  | | | |
| 103547\_at | Slc41a1 | solute carrier family 41, member 1 | chr1 | -2.728 | 30 | 0 |  | | | |
| 104083\_at | Cdh5 | cadherin 5 | chr8 | -1.968 | 30 | 0.003 |  | | | |
| 104376\_at | Hdac5 | histone deacetylase 5 | chr11 | -2.263 | 30 | 0.006 |  | | | |
| 104417\_at | NoneAvailable | Mus musculus transcribed sequences | chr11 | -2.062 | 30 | 0.001 |  | | | |
| 104645\_at | Klf7 | Kruppel-like factor 7 (ubiquitous) | chr1 | -1.19 | 30 | 0.003 |  | | | |
| 160228\_at | 1110019C08Rik | RIKEN cDNA 1110019C08 gene | chr16 | -1.025 | 30 | 0.025 |  | | | |
| 160651\_at | Tacstd2 | tumor-associated calcium signal transducer 2 | chr6 | -3.696 | 30 | 0 |  | | | |
| 160727\_at | 2410002F23Rik | RIKEN cDNA 2410002F23 gene | chr2 | -1.032 | 30 | 0.005 |  | | | |
| 161080\_f\_at | 1700012P16Rik | RIKEN cDNA 1700012P16 gene | chr5 | -1.148 | 30 | 0.008 |  | | | |
| 161184\_f\_at | Tie1 | tyrosine kinase receptor 1 | chr4 | -1.63 | 30 | 0.006 |  | | | |
| 161990\_f\_at | BC012974 | hypothetical gene supported by BC012974 | chr18 | -1.702 | 30 | 0.006 |  | | | |
| 92249\_g\_at | Nr4a2 | nuclear receptor subfamily 4, group A, member 2 | chr2 | -5.506 | 30 | 0.001 |  | | | |
| 92821\_at | Usp2 | ubiquitin specific protease 2 | chr9 | -1.119 | 30 | 0.004 |  | | | |
| 93875\_at | Hspa1a | heat shock protein 1A | chr17 | -1.082 | 30 | 0.006 |  | | | |
| 94060\_at | Myo1h | myosin 1H | chr5 | -1.495 | 30 | 0.002 |  | | | |
| 94657\_at | NoneAvailable | Mus musculus transcribed sequences | chr8 | -5.096 | 30 | 0.006 |  | | | |
| 94976\_at | AL022610 | expressed sequence AL022610 | chr7 | -1.012 | 30 | 0.029 |  | | | |
| 95002\_at | D17Wsu92e | DNA segment, Chr 17, Wayne State University 92, expressed | chr17 | -1.483 | 30 | 0.016 |  | | | |
| 95033\_at | Jmjd1 | jumonji domain containing 1 | chr6 | -1.446 | 30 | 0.007 |  | | | |
| 95618\_at | D6Ertd32e | DNA segment, Chr 6, ERATO Doi 32, expressed | chr6 | -2.616 | 30 | 0.038 |  | | | |
| 95805\_at | Cdc2l2 | cell division cycle 2 homolog (S. pombe)-like 2 | chr4 | -1.429 | 30 | 0.02 |  | | | |
| 96076\_at | Stx5a | syntaxin 5A | chr19 | -1.273 | 30 | 0.008 |  | | | |
| 96088\_at | Ndr2 | N-myc downstream regulated 2 | chr14 | -1.173 | 30 | 0.019 |  | | | |
| 96147\_at | Mafg | v-maf musculoaponeurotic fibrosarcoma oncogene family, protein G (avian) | chr11 | -1.157 | 30 | 0.03 |  | | | |
| 96367\_at | NoneAvailable | Mus musculus transcribed sequences | chr17 | -1.93 | 30 | 0.01 |  | | | |
| 96669\_at | 2400003C14Rik | RIKEN cDNA 2400003C14 gene | chr8 | -1.563 | 30 | 0.026 |  | | | |
| 97125\_f\_at | LOC56628 | MHC (A.CA/J(H-2K-f) class I antigen | chr17 | -3.415 | 30 | 0.007 |  | | | |
| 97375\_at | Pkd1 | polycystic kidney disease 1 homolog | chr17 | -1.292 | 30 | 0.009 |  | | | |
| 98065\_at | Ormdl3 | ORM1-like 3 (S. cerevisiae) | chr11 | -1.914 | 30 | 0.032 |  | | | |
| 98438\_f\_at | H2-Q7 | histocompatibility 2, Q region locus 7 | chr17 | -3.59 | 30 | 0.001 |  | | | |
| 98906\_at | Fbxo9 | f-box only protein 9 | chr9 | -2.278 | 30 | 0.037 |  | | | |
| 99961\_s\_at | Cdc2l2 | cell division cycle 2 homolog (S. pombe)-like 2 | chr4 | -1.587 | 30 | 0.025 |  | | | |
| 99970\_at | Ptpn21 | protein tyrosine phosphatase, non-receptor type 21 | chr12 | -1.262 | 30 | 0.008 |  | | | |
| \* Positive log2 fold changes represent genes expressed higher in FL-HSC; Negative log2 fold changes represent genes expressed higher in adult HSC (fold change=2 is equivalent to log2 fold change=1) | | | | | | | | | | |
|  |  |  |  |  |  |  |  |  |  |  |
