# Supplementary material for: Molecular Signatures of Proliferation and Quiescence in Hematopoietic Stem Cells
Source: PLoS Biol. 2004 Sep 28;2(10):e301. doi: 10.1371/journal.pbio.0020301 (PMC520599; doi:10.1371/journal.pbio.0020301)
Supplement: Table S8 — (164 KB HTML). [file pbio.0020301.st008.html]

|  |  | ST-HSC sig |  |  |  |  |  |  |  |  |
| Probe Set ID | Gene Symbol | Gene name | Chromosome | Log2 Fold Change (FL-HSC vs Adult HSC)\* | Day of max (TOM) | p-value of ANOVA (time course) |  | | | |
| 101959\_r\_at | Tfdp1 | transcription factor Dp 1 | chr8 | 1.111 | 2 | 0.004 |  | | | |
| 103057\_at | Pold1 | polymerase (DNA directed), delta 1, catalytic subunit | chr7 | 3.176 | 2 | 0.034 |  | | | |
| 104303\_i\_at | 1500004O14Rik | RIKEN cDNA 1500004O14 gene | chr2 | 1.473 | 2 | 0.007 |  | | | |
| 160633\_at | Refbp1 | RNA and export factor binding protein 1 | --- | 0.652 | 2 | 0.028 |  | | | |
| 161400\_f\_at | NoneAvailable | --- | --- | 0.17 | 2 | 0.002 |  | | | |
| 92633\_at | Ctsz | cathepsin Z | chr2 | 0.866 | 2 | 0.003 |  | | | |
| 92794\_f\_at | Nme1 | expressed in non-metastatic cells 1, protein | chr11 | 2.156 | 2 | 0 |  | | | |
| 93070\_at | Kpnb3 | karyopherin (importin) beta 3 | chr14 | 0.784 | 2 | 0.004 |  | | | |
| 94450\_at | D13Wsu123e | DNA segment, Chr 13, Wayne State University 123, expressed | chr13 | 1.019 | 2 | 0.014 |  | | | |
| 95755\_at | Csda | cold shock domain protein A | --- | 0.101 | 2 | 0.02 |  | | | |
| 100062\_at | Mcm3 | minichromosome maintenance deficient 3 (S. cerevisiae) | chr1 | 1.792 | 3 | 0.005 |  | | | |
| 100290\_f\_at | 6230416J20Rik | RIKEN cDNA 6230416J20 gene | chr4 | 0.452 | 3 | 0.022 |  | | | |
| 101445\_at | Dnmt1 | DNA methyltransferase (cytosine-5) 1 | chr9 | 0.6 | 3 | 0.041 |  | | | |
| 101920\_at | Pole2 | polymerase (DNA directed), epsilon 2 (p59 subunit) | --- | 1.432 | 3 | 0.025 |  | | | |
| 101958\_f\_at | Tfdp1 | transcription factor Dp 1 | chr8 | 0.364 | 3 | 0.003 |  | | | |
| 102128\_f\_at | Mrps25 | mitochondrial ribosomal protein S25 | --- | 1.148 | 3 | 0.01 |  | | | |
| 103064\_at | Chek1 | checkpoint kinase 1 homolog (S. pombe) | chr9 | 1.251 | 3 | 0.01 |  | | | |
| 103201\_at | Ttk | Ttk protein kinase | chr9 | 2.66 | 3 | 0.018 |  | | | |
| 103203\_f\_at | NoneAvailable | Mus musculus transcribed sequence with moderate similarity to protein ref:NP\_078956.1 (H.sapiens)  hypothetical protein FLJ23311 [Homo sapiens] | chr7 | 2.324 | 3 | 0.019 |  | | | |
| 103204\_r\_at | NoneAvailable | Mus musculus transcribed sequence with moderate similarity to protein ref:NP\_078956.1 (H.sapiens)  hypothetical protein FLJ23311 [Homo sapiens] | chr7 | 1.738 | 3 | 0.048 |  | | | |
| 103207\_at | Pola1 | polymerase (DNA directed), alpha 1 | chrX | 2.502 | 3 | 0.025 |  | | | |
| 103444\_at | E130315B21Rik | RIKEN cDNA E130315B21 gene | chr10 | 1.296 | 3 | 0.016 |  | | | |
| 103797\_at | Cdc7 | cell division cycle 7 (S. cerevisiae) | chr5 | 0.917 | 3 | 0.008 |  | | | |
| 103821\_at | Cdc6 | cell division cycle 6 homolog (S. cerevisiae) | --- | 2.833 | 3 | 0.016 |  | | | |
| 160069\_at | Gmnn | geminin | chr13 | 2.598 | 3 | 0.009 |  | | | |
| 160377\_at | Tardbp | TAR DNA binding protein | chr4 | 0.603 | 3 | 0.017 |  | | | |
| 160496\_s\_at | Mcm3 | minichromosome maintenance deficient 3 (S. cerevisiae) | chr1 | 1.482 | 3 | 0.021 |  | | | |
| 160988\_r\_at | D3Ertd330e | DNA segment, Chr 3, ERATO Doi 330, expressed | chr3 | 0.265 | 3 | 0 |  | | | |
| 161122\_f\_at | Ndufab1 | NADH dehydrogenase (ubiquinone) 1, alpha/beta subcomplex, 1 | --- | 1.68 | 3 | 0.003 |  | | | |
| 161787\_f\_at | Ris2 | retroviral integration site 2 | chr8 | 0.401 | 3 | 0.018 |  | | | |
| 92551\_at | Lig1 | ligase I, DNA, ATP-dependent | chr7 | 1.152 | 3 | 0.033 |  | | | |
| 92647\_at | Rbbp4 | retinoblastoma binding protein 4 | chr4 | -0.165 | 3 | 0.028 |  | | | |
| 92782\_at | Tmpo | thymopoietin | chr10 | 0.653 | 3 | 0 |  | | | |
| 93228\_at | Hells | helicase, lymphoid specific | chr19 | 0.826 | 3 | 0.023 |  | | | |
| 94376\_s\_at | Mre11a | meiotic recombination 11 homolog A (S. cerevisiae) | chr9 | 2.127 | 3 | 0.035 |  | | | |
| 94788\_f\_at | Tubb5 | tubulin, beta 5 | chr17 | 1.367 | 3 | 0.026 |  | | | |
| 95063\_at | 2310021G01Rik | RIKEN cDNA 2310021G01 gene | chr2 | 2.4 | 3 | 0.041 |  | | | |
| 95612\_at | Rfc5 | replication factor C (activator 1) 5 | --- | 2.137 | 3 | 0.009 |  | | | |
| 95703\_at | Uble1a | ubiquitin-like 1 (sentrin) activating enzyme E1A | --- | 0.674 | 3 | 0.022 |  | | | |
| 95732\_at | 1110005L13Rik | RIKEN cDNA 1110005L13 gene | chr10 | 2.644 | 3 | 0.028 |  | | | |
| 96625\_at | D630024B06Rik | RIKEN cDNA D630024B06 gene | chr14 | 1.795 | 3 | 0.01 |  | | | |
| 96687\_f\_at | 2010100O12Rik | RIKEN cDNA 2010100O12 gene | chr2 | 1.093 | 3 | 0 |  | | | |
| 97411\_at | Ect2 | ect2 oncogene | chr3 | 2.597 | 3 | 0.019 |  | | | |
| 97909\_at | Stmn1 | stathmin 1 | chr4 | 0.912 | 3 | 0.004 |  | | | |
| 98922\_at | Itm1 | intergral membrane protein 1 | chr9 | -0.602 | 3 | 0.01 |  | | | |
| 98956\_at | Tram1 | translocating chain-associating membrane protein 1 | chr1 | -0.019 | 3 | 0.049 |  | | | |
| 99457\_at | Mki67 | antigen identified by monoclonal antibody Ki 67 | chr7 | 2.708 | 3 | 0.002 |  | | | |
| 99581\_at | Hint | histidine triad nucleotide binding protein | --- | 1.503 | 3 | 0.038 |  | | | |
| 99777\_s\_at | 6230416J20Rik | RIKEN cDNA 6230416J20 gene | chr4 | 0.516 | 3 | 0.01 |  | | | |
| 100057\_at | 2510027N19Rik | RIKEN cDNA 2510027N19 gene | chr7 | 3.001 | 6 | 0 |  | | | |
| 100116\_at | 2810417H13Rik | RIKEN cDNA 2810417H13 gene | chr1 | 2.111 | 6 | 0.026 |  | | | |
| 100128\_at | Cdc2a | cell division cycle 2 homolog A (S. pombe) | --- | 4.133 | 6 | 0.002 |  | | | |
| 100156\_at | Mcm5 | minichromosome maintenance deficient 5, cell division cycle 46 (S. cerevisiae) | chr8 | 5.137 | 6 | 0.012 |  | | | |
| 100512\_at | Uchl5 | ubiquitin carboxyl-terminal esterase L5 | chr1 | 1.055 | 6 | 0.006 |  | | | |
| 100527\_at | D11Ertd99e | DNA segment, Chr 11, ERATO Doi 99, expressed | chr11 | 2.559 | 6 | 0.038 |  | | | |
| 100612\_at | Rrm1 | ribonucleotide reductase M1 | --- | 1.929 | 6 | 0.045 |  | | | |
| 100618\_f\_at | Slc25a5 | solute carrier family 25 (mitochondrial carrier; adenine nucleotide translocator), member 5 | --- | 1.078 | 6 | 0.012 |  | | | |
| 101065\_at | Pcna | proliferating cell nuclear antigen | chr19 | 0.251 | 6 | 0.008 |  | | | |
| 101105\_at | Banf1 | barrier to autointegration factor 1 | chr19 | 2.053 | 6 | 0.002 |  | | | |
| 101254\_at | Ran | RAN, member RAS oncogene family | chr2 | 1.149 | 6 | 0.002 |  | | | |
| 101421\_at | Rnf5 | ring finger protein 5 | chr17 | 1.067 | 6 | 0.044 |  | | | |
| 101506\_at | Snrpa1 | small nuclear ribonucleoprotein polypeptide A' | chr7 | 1.197 | 6 | 0.003 |  | | | |
| 101521\_at | Birc5 | baculoviral IAP repeat-containing 5 | chr11 | 2.475 | 6 | 0.01 |  | | | |
| 101558\_s\_at | Psmb5 | proteasome (prosome, macropain) subunit, beta type 5 | chr14 | 1.051 | 6 | 0.018 |  | | | |
| 101562\_at | Hsp70-4 | heat shock protein 4 | chr2 | 2.149 | 6 | 0.047 |  | | | |
| 101964\_at | Tkt | transketolase | chr14 | 1.087 | 6 | 0.001 |  | | | |
| 102001\_at | Rrm2 | ribonucleotide reductase M2 | chr12 | 1.74 | 6 | 0.02 |  | | | |
| 102412\_at | AW541137 | expressed sequence AW541137 | chr10 | 2.049 | 6 | 0.032 |  | | | |
| 102821\_s\_at | Rasl2-9 | RAS-like, family 2, locus 9 | chr2 | 1.55 | 6 | 0 |  | | | |
| 102853\_at | Cspg6 | chondroitin sulfate proteoglycan 6 | chr19 | 1.265 | 6 | 0.037 |  | | | |
| 103089\_at | Cd48 | CD48 antigen | chr1 | 7.012 | 6 | 0 |  | | | |
| 103335\_at | Lgals9 | lectin, galactose binding, soluble 9 | chr11 | 2.142 | 6 | 0.019 |  | | | |
| 103418\_at | Rfc4 | replication factor C (activator 1) 4 | chr16 | 2.864 | 6 | 0.041 |  | | | |
| 103468\_at | Mns1 | meiosis-specific nuclear structural protein 1 | chr9 | 3.961 | 6 | 0.026 |  | | | |
| 103534\_at | NoneAvailable | --- | chr7 | 2.111 | 6 | 0.006 |  | | | |
| 103598\_at | Dhx9 | DEAH (Asp-Glu-Ala-His) box polypeptide 9 | chr1 | 0.862 | 6 | 0.032 |  | | | |
| 103654\_at | Nsbp1 | nucleosome binding protein 1 | chrX | 0.032 | 6 | 0.02 |  | | | |
| 103683\_at | Dhodh | dihydroorotate dehydrogenase | chr8 | 1.175 | 6 | 0.041 |  | | | |
| 104147\_at | Nans | N-acetylneuraminic acid synthase (sialic acid synthase) | chr4 | 1.196 | 6 | 0.022 |  | | | |
| 104322\_at | Ckap2 | cytoskeleton associated protein 2 | chr8 | 1.598 | 6 | 0.003 |  | | | |
| 104423\_at | 2810047L02Rik | RIKEN cDNA 2810047L02 gene | chr1 | 3.018 | 6 | 0.009 |  | | | |
| 104476\_at | Rbl1 | retinoblastoma-like 1 (p107) | chr2 | 1.983 | 6 | 0.011 |  | | | |
| 104541\_at | Prtn3 | proteinase 3 | chr10 | 1.916 | 6 | 0.042 |  | | | |
| 104738\_at | Zrf2 | zuotin related factor 2 | chr6 | 1.861 | 6 | 0.008 |  | | | |
| 160107\_at | Hprt | hypoxanthine guanine phosphoribosyl transferase | chrX | 0.226 | 6 | 0.028 |  | | | |
| 160203\_at | 5330419I01Rik | RIKEN cDNA 5330419I01 gene | chr1 | 0.873 | 6 | 0.017 |  | | | |
| 160262\_at | Mtch2 | mitochondrial carrier homolog 2 (C. elegans) | chr2 | 0.99 | 6 | 0.01 |  | | | |
| 160324\_at | Rpa3 | replication protein A3 | chr1 | 1.959 | 6 | 0.009 |  | | | |
| 160503\_at | Fbl | fibrillarin | chr17 | 1.886 | 6 | 0 |  | | | |
| 160550\_i\_at | Magoh | mago-nashi homolog, proliferation-associated (Drosophila) | chr4 | 0.676 | 6 | 0.016 |  | | | |
| 161147\_f\_at | 1110046L09Rik | RIKEN cDNA 1110046L09 gene | chr8 | 3.517 | 6 | 0.013 |  | | | |
| 92540\_f\_at | Srm | spermidine synthase | chr4 | 6.091 | 6 | 0.001 |  | | | |
| 92596\_at | Cacybp | calcyclin binding protein | chr1 | 0.523 | 6 | 0.039 |  | | | |
| 92615\_at | AI837181 | expressed sequence AI837181 | chr4 | 2.807 | 6 | 0.001 |  | | | |
| 92625\_at | Nme2 | expressed in non-metastatic cells 2, protein | chr10 | 1.732 | 6 | 0.004 |  | | | |
| 92629\_f\_at | Hdgf | hepatoma-derived growth factor | --- | 0.521 | 6 | 0.019 |  | | | |
| 92829\_at | Hspe1 | heat shock protein 1 (chaperonin 10) | chr1 | 1.429 | 6 | 0.001 |  | | | |
| 93041\_at | Mcm4 | minichromosome maintenance deficient 4 homolog (S. cerevisiae) | chr16 | 1.922 | 6 | 0.039 |  | | | |
| 93095\_at | Hmgb1 | high mobility group box 1 | chr11 | 0.963 | 6 | 0.03 |  | | | |
| 93112\_at | Mcm2 | minichromosome maintenance deficient 2 mitotin (S. cerevisiae) | chr6 | 2.139 | 6 | 0.018 |  | | | |
| 93236\_s\_at | Tyms | thymidylate synthase | chr10 | 3.228 | 6 | 0.012 |  | | | |
| 93237\_s\_at | Tyms | thymidylate synthase | chr10 | 2.144 | 6 | 0.021 |  | | | |
| 93512\_f\_at | Adk | adenosine kinase | chr14 | 0.798 | 6 | 0 |  | | | |
| 93581\_at | Ndufb8 | NADH dehydrogenase (ubiquinone) 1 beta subcomplex 8 | chr19 | 2.79 | 6 | 0.003 |  | | | |
| 93734\_i\_at | Psmc3 | proteasome (prosome, macropain) 26S subunit, ATPase 3 | chr2 | 0.698 | 6 | 0.002 |  | | | |
| 93786\_i\_at | Mrpl18 | mitochondrial ribosomal protein L18 | chr17 | 2.577 | 6 | 0.013 |  | | | |
| 93787\_f\_at | Mrpl18 | mitochondrial ribosomal protein L18 | --- | 2.215 | 6 | 0.009 |  | | | |
| 93970\_at | Ipo7 | importin 7 | chr7 | 0.551 | 6 | 0.018 |  | | | |
| 94034\_at | Smfn | small fragment nuclease | chr9 | 0.778 | 6 | 0 |  | | | |
| 94040\_at | Erh | enhancer of rudimentary homolog (Drosophila) | chr12 | 1.929 | 6 | 0.001 |  | | | |
| 94062\_at | Ndufv2 | NADH dehydrogenase (ubiquinone) flavoprotein 2 | chr17 | 1.3 | 6 | 0.045 |  | | | |
| 94267\_i\_at | NoneAvailable | --- | chr9 | 0.902 | 6 | 0.037 |  | | | |
| 94294\_at | Ccnb2 | cyclin B2 | chr9 | 4.408 | 6 | 0.001 |  | | | |
| 94372\_at | Nudt1 | nudix (nucleoside diphosphate linked moiety X)-type motif 1 | --- | 3.289 | 6 | 0.003 |  | | | |
| 94494\_at | Farsl | phenylalanine-tRNA synthetase-like | chr1 | 1.832 | 6 | 0.008 |  | | | |
| 94789\_r\_at | Tubb5 | tubulin, beta 5 | chr17 | 3.275 | 6 | 0.044 |  | | | |
| 94912\_at | Mrps21 | mitochondrial ribosomal protein S21 | chr3 | 1.449 | 6 | 0.017 |  | | | |
| 94925\_at | 1810055D05Rik | RIKEN cDNA 1810055D05 gene | chr3 | 0.976 | 6 | 0.021 |  | | | |
| 95441\_at | Timm23 | translocase of inner mitochondrial membrane 23 homolog (yeast) | chr1 | 1.675 | 6 | 0.042 |  | | | |
| 95460\_at | Cops5 | COP9 (constitutive photomorphogenic) homolog, subunit 5 (Arabidopsis thaliana) | chr1 | 1.564 | 6 | 0.025 |  | | | |
| 95497\_at | 1110005A05Rik | RIKEN cDNA 1110005A05 gene | chr9 | 3.276 | 6 | 0.011 |  | | | |
| 95561\_at | 1700013H19Rik | RIKEN cDNA 1700013H19 gene | chr8 | 0.899 | 6 | 0.003 |  | | | |
| 95634\_at | 0610010K14Rik | RIKEN cDNA 0610010K14 gene | --- | 1.777 | 6 | 0.023 |  | | | |
| 95636\_at | 0610010K14Rik | RIKEN cDNA 0610010K14 gene | chr11 | 2.424 | 6 | 0.007 |  | | | |
| 95677\_at | 0610009C03Rik | RIKEN cDNA 0610009C03 gene | --- | 2.789 | 6 | 0.026 |  | | | |
| 95690\_at | 1110030L07Rik | RIKEN cDNA 1110030L07 gene | chr15 | 3.584 | 6 | 0.007 |  | | | |
| 95891\_at | NoneAvailable | Mus musculus transcribed sequences | chr16 | 3.953 | 6 | 0.017 |  | | | |
| 96081\_at | Tk1 | thymidine kinase 1 | chr11 | 2.893 | 6 | 0.014 |  | | | |
| 96112\_at | Etfa | electron transferring flavoprotein, alpha polypeptide | chr9 | 1.779 | 6 | 0.006 |  | | | |
| 96293\_at | 2410015N17Rik | RIKEN cDNA 2410015N17 gene | chr7 | 4.422 | 6 | 0.006 |  | | | |
| 96319\_at | Cdc20 | cell division cycle 20 homolog (S. cerevisiae) | --- | 4.591 | 6 | 0.004 |  | | | |
| 96336\_at | Gatm | glycine amidinotransferase (L-arginine:glycine amidinotransferase) | --- | 0.529 | 6 | 0.022 |  | | | |
| 96353\_at | 1110021D01Rik | RIKEN cDNA 1110021D01 gene | chr13 | 2.999 | 6 | 0.003 |  | | | |
| 96611\_at | NoneAvailable | Mus musculus cDNA clone IMAGE:5694421, partial cds | chr17 | 0.414 | 6 | 0.001 |  | | | |
| 96696\_at | Hrmt1l2 | heterogeneous nuclear ribonucleoproteins  methyltransferase-like 2 (S. cerevisiae) | chr7 | 2.608 | 6 | 0.013 |  | | | |
| 96743\_at | Skiip | SKI interacting protein | --- | 1.897 | 6 | 0.005 |  | | | |
| 96849\_at | Timm8a | translocase of inner mitochondrial membrane 8 homolog a (yeast) | chr10 | 0.859 | 6 | 0.048 |  | | | |
| 96892\_at | Psma1 | proteasome (prosome, macropain) subunit, alpha type 1 | chr7 | 1.257 | 6 | 0.017 |  | | | |
| 96909\_at | Ndufab1 | NADH dehydrogenase (ubiquinone) 1, alpha/beta subcomplex, 1 | chr7 | 1.256 | 6 | 0.014 |  | | | |
| 96947\_at | Etfb | electron transferring flavoprotein, beta polypeptide | chr7 | 2.477 | 6 | 0 |  | | | |
| 97164\_at | 2610207P08Rik | RIKEN cDNA 2610207P08 gene | --- | 3.233 | 6 | 0.011 |  | | | |
| 97179\_at | NoneAvailable | Mus musculus mRNA similar to putative c-Myc-responsive (cDNA clone MGC:54855 IMAGE:5388297), complete cds | --- | 2.605 | 6 | 0.001 |  | | | |
| 97248\_at | Dbi | diazepam binding inhibitor | --- | 1.56 | 6 | 0 |  | | | |
| 97268\_i\_at | 0610010I12Rik | RIKEN cDNA 0610010I12 gene | chr3 | 1.265 | 6 | 0.003 |  | | | |
| 97468\_at | Cks1 | CDC28 protein kinase 1 | chr3 | 2.843 | 6 | 0 |  | | | |
| 97538\_at | Gus | beta-glucuronidase | chr5 | 1.607 | 6 | 0.033 |  | | | |
| 97758\_at | Prdx1 | peroxiredoxin 1 | chr8 | 1.211 | 6 | 0.01 |  | | | |
| 97820\_at | Galk1 | galactokinase 1 | chr11 | 2.703 | 6 | 0.004 |  | | | |
| 97824\_at | D11Ertd175e | DNA segment, Chr 11, ERATO Doi 175, expressed | chr11 | 1.494 | 6 | 0.006 |  | | | |
| 97828\_at | Siva-pending | Cd27 binding protein (Hindu God of destruction) | chr12 | 1.387 | 6 | 0 |  | | | |
| 97884\_at | Mrps11 | mitochondrial ribosomal protein S11 | chr7 | 1.959 | 6 | 0.019 |  | | | |
| 98440\_at | Ltb4dh | leukotriene B4 12-hydroxydehydrogenase | chr4 | 3.855 | 6 | 0 |  | | | |
| 98516\_at | NoneAvailable | Mus musculus cDNA clone MGC:67360 IMAGE:6823629, complete cds | chr8 | 0.615 | 6 | 0.016 |  | | | |
| 98524\_f\_at | NoneAvailable | --- | --- | 2.603 | 6 | 0.008 |  | | | |
| 98587\_at | Nap1l1 | nucleosome assembly protein 1-like 1 | chr10 | 0.829 | 6 | 0.015 |  | | | |
| 98904\_at | Mrpl35 | mitochondrial ribosomal protein L35 | chr6 | 2.237 | 6 | 0.002 |  | | | |
| 98938\_at | 1500026D16Rik | RIKEN cDNA 1500026D16 gene | chr19 | 1.602 | 6 | 0.013 |  | | | |
| 98975\_at | 2410008G02Rik | RIKEN cDNA 2410008G02 gene | chr8 | 0.732 | 6 | 0.033 |  | | | |
| 99129\_at | Clast3-pending | CD40 ligand-activated specific transcript 3 | chr18 | 1.773 | 6 | 0.039 |  | | | |
| 99546\_at | Fkbp2 | FK506 binding protein 2 | --- | 2.273 | 6 | 0.001 |  | | | |
| 99632\_at | Mad2l1 | MAD2 (mitotic arrest deficient, homolog)-like 1 (yeast) | chr6 | 1.589 | 6 | 0.018 |  | | | |
| \* Positive log2 fold changes represent genes expressed higher in FL-HSC; Negative log2 fold changes represent genes expressed higher in adult HSC (fold change=2 is equivalent to log2 fold change=1) | | | | | | | | | | |
|  |  |  |  |  |  |  |  |  |  |  |
