# Supplementary material for: Molecular Signatures of Proliferation and Quiescence in Hematopoietic Stem Cells
Source: PLoS Biol. 2004 Sep 28;2(10):e301. doi: 10.1371/journal.pbio.0020301 (PMC520599; doi:10.1371/journal.pbio.0020301)
Supplement: Table S9 — (77 KB HTML). [file pbio.0020301.st009.html]

|  |  | LT-HSC sig |  |  |  |  |  |  |  |  |
| Probe Set ID | Gene Symbol | Gene name | Chromosome | Log2 Fold Change (FL-HSC vs Adult HSC)\* | Day of max (TOM) | p-value of ANOVA (time course) |  | | | |
| 101897\_g\_at | Cd1d2 | CD1d2 antigen | chr3 | -0.949 | 0 | 0.024 |  | | | |
| 101940\_at | Smpd2 | sphingomyelin phosphodiesterase 2, neutral | chr10 | -0.013 | 0 | 0.039 |  | | | |
| 101962\_at | 2610007K22Rik | RIKEN cDNA 2610007K22 gene | chr15 | -0.339 | 0 | 0.015 |  | | | |
| 102021\_at | Il4ra | interleukin 4 receptor, alpha | chr7 | -0.384 | 0 | 0.033 |  | | | |
| 102286\_at | Araf | raf-related oncogene | chrX | 0.464 | 0 | 0.035 |  | | | |
| 102884\_at | Inpp5d | inositol polyphosphate-5-phosphatase D | chr1 | 0.265 | 0 | 0.013 |  | | | |
| 160346\_at | 6330580J24Rik | RIKEN cDNA 6330580J24 gene | chr9 | -0.152 | 0 | 0.003 |  | | | |
| 160547\_s\_at | Txnip | thioredoxin interacting protein | chr3 | -0.865 | 0 | 0 |  | | | |
| 160714\_at | Gab1 | growth factor receptor bound protein 2-associated protein 1 | chr8 | -0.506 | 0 | 0.048 |  | | | |
| 92830\_s\_at | NoneAvailable | --- | --- | -3.484 | 0 | 0.001 |  | | | |
| 94288\_at | Hist1h1c | histone 1, H1c | chr13 | -0.134 | 0 | 0.019 |  | | | |
| 95102\_at | Scotin-pending | scotin gene | chr9 | -2.721 | 0 | 0.006 |  | | | |
| 96049\_at | Bgn | biglycan | --- | -1.681 | 0 | 0.008 |  | | | |
| 97336\_at | Ctsf | cathepsin F | chr19 | -1.534 | 0 | 0.002 |  | | | |
| 100508\_at | Mfng | manic fringe homolog (Drosophila) | chr15 | -0.007 | 1 | 0.001 |  | | | |
| 100998\_at | H2-Ab1 | histocompatibility 2, class II antigen A, beta 1 | chr17 | -1.116 | 1 | 0.026 |  | | | |
| 101059\_at | Ndn | necdin | chr7 | -0.839 | 1 | 0.032 |  | | | |
| 101554\_at | Nfkbia | nuclear factor of kappa light chain gene enhancer in B-cells inhibitor, alpha | chr12 | -0.607 | 1 | 0.005 |  | | | |
| 101568\_at | NoneAvailable | Mus musculus mRNA similar to proline synthetase co-transcribed (cDNA clone MGC:59396 IMAGE:6504579), complete cds | chr8 | -1.703 | 1 | 0.024 |  | | | |
| 101963\_at | Ctsl | cathepsin L | chr13 | -2.894 | 1 | 0.022 |  | | | |
| 101990\_at | Ldh2 | lactate dehydrogenase 2, B chain | chr6 | -1.289 | 1 | 0.001 |  | | | |
| 102332\_at | Ulk1 | Unc-51 like kinase 1 (C. elegans) | chr5 | -2.225 | 1 | 0.009 |  | | | |
| 102401\_at | Irf1 | interferon regulatory factor 1 | chr11 | -0.554 | 1 | 0.009 |  | | | |
| 102906\_at | Tgtp | T-cell specific GTPase | chr11 | -5.973 | 1 | 0.001 |  | | | |
| 160933\_at | Igtp | interferon gamma induced GTPase | chr11 | -3.814 | 1 | 0 |  | | | |
| 160982\_at | 4921526G09Rik | RIKEN cDNA 4921526G09 gene | chrX | -0.898 | 1 | 0.018 |  | | | |
| 161666\_f\_at | Gadd45b | growth arrest and DNA-damage-inducible 45 beta | chr10 | -2.691 | 1 | 0.042 |  | | | |
| 92263\_at | Grcb | gene rich cluster, B gene | chr6 | -1.939 | 1 | 0.002 |  | | | |
| 92401\_at | Ltc4s | leukotriene C4 synthase | chr11 | -0.139 | 1 | 0.011 |  | | | |
| 92440\_at | Irf6 | interferon regulatory factor 6 | chr1 | -4.326 | 1 | 0.031 |  | | | |
| 92866\_at | H2-Aa | histocompatibility 2, class II antigen A, alpha | chr17 | -3.869 | 1 | 0.036 |  | | | |
| 93020\_at | Rex3 | reduced expression 3 | chrX | -1.019 | 1 | 0.009 |  | | | |
| 93751\_at | 8430421I07Rik | RIKEN cDNA 8430421I07 gene | chr9 | -0.171 | 1 | 0.026 |  | | | |
| 94270\_at | Krt1-18 | keratin complex 1, acidic, gene 18 | --- | -3.531 | 1 | 0.013 |  | | | |
| 94285\_at | H2-Eb1 | histocompatibility 2, class II antigen E beta | chr17 | -0.946 | 1 | 0 |  | | | |
| 96592\_at | Pik3r1 | phosphatidylinositol 3-kinase, regulatory subunit, polypeptide 1 (p85 alpha) | chr13 | 0.081 | 1 | 0.03 |  | | | |
| 96703\_at | Maged1 | melanoma antigen, family D, 1 | chrX | -2.002 | 1 | 0.003 |  | | | |
| 96728\_at | DXImx38e | DNA segment, Chr X, Immunex 38, expressed | chrX | -2.856 | 1 | 0.001 |  | | | |
| 96764\_at | Iigp-pending | interferon-inducible GTPase | --- | -5.077 | 1 | 0.009 |  | | | |
| 96935\_at | 2700030M23Rik | RIKEN cDNA 2700030M23 gene | chr4 | -2.968 | 1 | 0.003 |  | | | |
| 98410\_at | Gtpi-pending | interferon-g induced GTPase | chr11 | -2.836 | 1 | 0.046 |  | | | |
| 99366\_at | E030024M05Rik | RIKEN cDNA E030024M05 gene | chr12 | -2.996 | 1 | 0.014 |  | | | |
| 99532\_at | Tob1 | transducer of ErbB-2.1 | chr11 | -5.048 | 1 | 0.008 |  | | | |
| 100606\_at | Prnp | prion protein | --- | -1.719 | 10 | 0.036 |  | | | |
| 101432\_at | 2410019G02Rik | RIKEN cDNA 2410019G02 gene | chr11 | -0.055 | 10 | 0.022 |  | | | |
| 102279\_at | 1300004C08Rik | RIKEN cDNA 1300004C08 gene | chr9 | -2.168 | 10 | 0.008 |  | | | |
| 103080\_at | Samhd1 | SAM domain and HD domain, 1 | chr2 | 0.08 | 10 | 0.003 |  | | | |
| 103254\_at | Fln29-pending | FLN29 gene product | chr5 | -2.179 | 10 | 0.011 |  | | | |
| 103408\_at | AI325941 | expressed sequence AI325941 | chr7 | 0.021 | 10 | 0.004 |  | | | |
| 103422\_at | Cd1d1 | CD1d1 antigen | chr3 | -2.191 | 10 | 0.001 |  | | | |
| 103443\_at | Aim1 | absent in melanoma 1 | chr10 | -1.49 | 10 | 0.005 |  | | | |
| 103518\_at | Ctla2b | cytotoxic T lymphocyte-associated protein 2 beta | chr13 | -5.219 | 10 | 0.015 |  | | | |
| 103890\_at | AW538196 | expressed sequence AW538196 | chr7 | -0.467 | 10 | 0.031 |  | | | |
| 104256\_at | Pscdbp | pleckstrin homology, Sec7 and coiled-coil domains, binding protein | chr2 | -2.279 | 10 | 0.022 |  | | | |
| 160287\_at | Map1lc3 | microtubule-associated protein 1 light chain 3 | chr14 | -1.669 | 10 | 0.02 |  | | | |
| 160832\_at | Ldlr | low density lipoprotein receptor | chr9 | 1.302 | 10 | 0.036 |  | | | |
| 160834\_at | 1110032C13Rik | RIKEN cDNA 1110032C13 gene | chr7 | -4.49 | 10 | 0.04 |  | | | |
| 162206\_f\_at | Socs3 | suppressor of cytokine signaling 3 | --- | -4.307 | 10 | 0.013 |  | | | |
| 162260\_at | 6330407G11Rik | RIKEN cDNA 6330407G11 gene | chr17 | -0.728 | 10 | 0.009 |  | | | |
| 92542\_at | D4Wsu53e | DNA segment, Chr 4, Wayne State University 53, expressed | chr4 | -1.236 | 10 | 0.014 |  | | | |
| 92758\_at | Dusp2 | dual specificity phosphatase 2 | chr2 | -0.942 | 10 | 0.002 |  | | | |
| 93274\_at | Clk | CDC-like kinase | chr1 | -1.211 | 10 | 0.031 |  | | | |
| 93311\_at | Clk3 | CDC-like kinase 3 | chr9 | -1.389 | 10 | 0.003 |  | | | |
| 93909\_f\_at | NoneAvailable | --- | --- | -0.211 | 10 | 0.002 |  | | | |
| 94689\_at | C79248 | expressed sequence C79248 | --- | -1.2 | 10 | 0.015 |  | | | |
| 94780\_at | Zfp288 | zinc finger protein 288 | chr16 | -4.353 | 10 | 0.034 |  | | | |
| 94928\_at | Tnfrsf1b | tumor necrosis factor receptor superfamily, member 1b | --- | -1.859 | 10 | 0.009 |  | | | |
| 95387\_f\_at | Sema4b | sema domain, immunoglobulin domain (Ig), transmembrane domain (TM) and short cytoplasmic domain, (semaphorin) 4B | chr7 | 0.341 | 10 | 0.032 |  | | | |
| 95433\_at | Ddx54 | DEAD (Asp-Glu-Ala-Asp) box polypeptide 54 | chr5 | 1.761 | 10 | 0.017 |  | | | |
| 95501\_at | 2410001C21Rik | RIKEN cDNA 2410001C21 gene | --- | 0.09 | 10 | 0.016 |  | | | |
| 95586\_at | P2rx4 | purinergic receptor P2X, ligand-gated ion channel 4 | chr5 | -1.711 | 10 | 0 |  | | | |
| 96176\_at | Arih2 | ariadne homolog 2 (Drosophila) | chr9 | -1.874 | 10 | 0.022 |  | | | |
| 97285\_f\_at | Ubxdc2 | UBX domain-containing 2 | chr17 | -2.548 | 10 | 0 |  | | | |
| 97710\_f\_at | C530046L02Rik | RIKEN cDNA C530046L02 gene | chr17 | -0.132 | 10 | 0 |  | | | |
| 98018\_at | Procr | protein C receptor, endothelial | chr2 | -4.119 | 10 | 0.038 |  | | | |
| 98461\_at | 1200014P03Rik | RIKEN cDNA 1200014P03 gene | chr17 | -1.307 | 10 | 0.007 |  | | | |
| 99100\_at | Stat3 | signal transducer and activator of transcription 3 | chr11 | -1.118 | 10 | 0.022 |  | | | |
| 99184\_at | Csad | cysteine sulfinic acid decarboxylase | --- | -2.259 | 10 | 0.001 |  | | | |
| 99187\_f\_at | 2010315L10Rik | RIKEN cDNA 2010315L10 gene | chr8 | -1.207 | 10 | 0.004 |  | | | |
| 99347\_f\_at | NoneAvailable | Mus musculus transcribed sequences | --- | -2.205 | 10 | 0.048 |  | | | |
| 102873\_at | Tap2 | transporter 2, ATP-binding cassette, sub-family B (MDR/TAP) | chr17 | -0.371 | 30 | 0.007 |  | | | |
| 104292\_at | Eya2 | eyes absent 2 homolog (Drosophila) | chr2 | -0.122 | 30 | 0.042 |  | | | |
| 104376\_at | Hdac5 | histone deacetylase 5 | chr11 | -2.263 | 30 | 0.006 |  | | | |
| 160463\_at | Myd116 | myeloid differentiation primary response gene 116 | chr7 | -0.282 | 30 | 0.048 |  | | | |
| 161184\_f\_at | Tie1 | tyrosine kinase receptor 1 | chr4 | -1.63 | 30 | 0.006 |  | | | |
| 92249\_g\_at | Nr4a2 | nuclear receptor subfamily 4, group A, member 2 | chr2 | -5.506 | 30 | 0.001 |  | | | |
| 95012\_at | Slc22a17 | solute carrier family 22 (organic cation transporter), member 17 | chr14 | -0.358 | 30 | 0.042 |  | | | |
| 95961\_at | BC042396 | cDNA sequence BC042396 | chr9 | 0.125 | 30 | 0.003 |  | | | |
| 96829\_at | D19Wsu162e | DNA segment, Chr 19, Wayne State University 162, expressed | chr19 | -0.857 | 30 | 0.006 |  | | | |
| 97375\_at | Pkd1 | polycystic kidney disease 1 homolog | chr17 | -1.292 | 30 | 0.009 |  | | | |
| 97684\_at | Prkcabp | protein kinase C, alpha binding protein | chr15 | 0.123 | 30 | 0.005 |  | | | |
| 99142\_at | Mpra-pending | membrane progestin receptor alpha | chr4 | 0.136 | 30 | 0.013 |  | | | |
| \* Positive log2 fold changes represent genes expressed higher in FL-HSC; Negative log2 fold changes represent genes expressed higher in adult HSC (fold change=2 is equivalent to log2 fold change=1) | | | | | | | | | | |
|  |  |  |  |  |  |  |  |  |  |  |
