# Supplementary material for: Molecular Signatures of Proliferation and Quiescence in Hematopoietic Stem Cells
Source: PLoS Biol. 2004 Sep 28;2(10):e301. doi: 10.1371/journal.pbio.0020301 (PMC520599; doi:10.1371/journal.pbio.0020301)
Supplement: Table S12 — (62 KB HTML). [file pbio.0020301.st012.html]

|  |  | Q-sig TOM 0 |  |  |  |  |  |  |  |  |
| Probe Set ID | Gene Symbol | Gene name | Chromosome | Log2 Fold Change (FL-HSC vs Adult HSC)\* | Day of max (TOM) | p-value of ANOVA (time course) |  | | | |
| 100533\_s\_at | Crem | cAMP responsive element modulator | --- | -5.967 | 0 | 0.008 |  | | | |
| 101515\_at | Acox1 | acyl-Coenzyme A oxidase 1, palmitoyl | --- | -3.857 | 0 | 0.018 |  | | | |
| 102156\_f\_at | NoneAvailable | --- | chr6 | -3.755 | 0 | 0.005 |  | | | |
| 102209\_at | Nfatc1 | nuclear factor of activated T-cells, cytoplasmic 1 | chr18 | -2.297 | 0 | 0.009 |  | | | |
| 102326\_at | Ncf2 | neutrophil cytosolic factor 2 | chr1 | -1.48 | 0 | 0.013 |  | | | |
| 102356\_at | Wdr23 | WD repeat domain 23 | chr14 | -1.437 | 0 | 0.036 |  | | | |
| 102955\_at | Nfil3 | nuclear factor, interleukin 3, regulated | chr13 | -2.259 | 0 | 0.012 |  | | | |
| 103037\_at | Ctf1 | cardiotrophin 1 | --- | -1.295 | 0 | 0.012 |  | | | |
| 103226\_at | Mrc1 | mannose receptor, C type 1 | chr2 | -2.184 | 0 | 0.003 |  | | | |
| 103427\_at | Fbxl3a | F-box and leucine-rich repeat protein 3a | chr14 | -1.769 | 0 | 0.042 |  | | | |
| 103990\_at | Fosb | FBJ osteosarcoma oncogene B | chr7 | -4.608 | 0 | 0.012 |  | | | |
| 104252\_at | AU020206 | expressed sequence AU020206 | chr7 | -1.192 | 0 | 0.034 |  | | | |
| 104516\_at | Cldn5 | claudin 5 | chr16 | -1.272 | 0 | 0.048 |  | | | |
| 104562\_at | 5730403M16Rik | RIKEN cDNA 5730403M16 gene | chr7 | -1.253 | 0 | 0.009 |  | | | |
| 104598\_at | Dusp1 | dual specificity phosphatase 1 | chr17 | -2.35 | 0 | 0.018 |  | | | |
| 104625\_at | Dnajb6 | DnaJ (Hsp40) homolog, subfamily B, member 6 | chr16 | -1.482 | 0 | 0.041 |  | | | |
| 104719\_at | Slc12a7 | solute carrier family 12, member 7 | chr13 | -1.166 | 0 | 0.039 |  | | | |
| 160104\_at | Hsd3b7 | hydroxy-delta-5-steroid dehydrogenase, 3 beta- and steroid delta-isomerase 7 | chr7 | -1.424 | 0 | 0.022 |  | | | |
| 160526\_s\_at | Crem | cAMP responsive element modulator | --- | -5.103 | 0 | 0.005 |  | | | |
| 160573\_at | Hccs | holocytochrome c synthetase | --- | -2.08 | 0 | 0.025 |  | | | |
| 160901\_at | Fos | FBJ osteosarcoma oncogene | chr12 | -3.257 | 0 | 0.024 |  | | | |
| 92248\_at | Nr4a2 | nuclear receptor subfamily 4, group A, member 2 | chr2 | -5.877 | 0 | 0.007 |  | | | |
| 92256\_at | Fdft1 | farnesyl diphosphate farnesyl transferase 1 | --- | -1.15 | 0 | 0.003 |  | | | |
| 92270\_at | Tro | trophinin | chrX | -1.72 | 0 | 0.006 |  | | | |
| 92558\_at | Vcam1 | vascular cell adhesion molecule 1 | chr3 | -4.435 | 0 | 0.005 |  | | | |
| 92830\_s\_at | NoneAvailable | --- | --- | -3.484 | 0 | 0.001 |  | | | |
| 93093\_at | Mcl1 | myeloid cell leukemia sequence 1 | chr3 | -2.974 | 0 | 0.018 |  | | | |
| 93120\_f\_at | H2-K | histocompatibility 2, K region | chr17 | -3.637 | 0 | 0.003 |  | | | |
| 93179\_at | B830009D23Rik | RIKEN cDNA B830009D23 gene | chr2 | -1.837 | 0 | 0.017 |  | | | |
| 93326\_at | Tm4sf2 | transmembrane 4 superfamily member 2 | chrX | -1.259 | 0 | 0.02 |  | | | |
| 93498\_s\_at | Aplp2 | amyloid beta (A4) precursor-like protein 2 | chr9 | -2.613 | 0 | 0.02 |  | | | |
| 93705\_at | Chrnb1 | cholinergic receptor, nicotinic, beta polypeptide 1 (muscle) | --- | -2.651 | 0 | 0.016 |  | | | |
| 93714\_f\_at | H2-Q7 | histocompatibility 2, Q region locus 7 | chr17 | -3.128 | 0 | 0.001 |  | | | |
| 93907\_f\_at | NoneAvailable | --- | --- | -1.754 | 0 | 0.001 |  | | | |
| 93964\_s\_at | Ddx6 | DEAD (Asp-Glu-Ala-Asp) box polypeptide 6 | chr9 | -3.009 | 0 | 0.03 |  | | | |
| 94345\_at | Il6st | interleukin 6 signal transducer | chr13 | -3.717 | 0 | 0 |  | | | |
| 94428\_at | Ilvbl | ilvB (bacterial acetolactate synthase)-like | chr10 | -1.305 | 0 | 0.004 |  | | | |
| 94834\_at | Ctsh | cathepsin H | chr9 | -1.968 | 0 | 0.045 |  | | | |
| 94948\_at | Trip6 | thyroid hormone receptor interactor 6 | chr5 | -1.016 | 0 | 0.02 |  | | | |
| 94991\_at | Synpo | synaptopodin | chr18 | -1.276 | 0 | 0.018 |  | | | |
| 95102\_at | Scotin-pending | scotin gene | chr9 | -2.721 | 0 | 0.006 |  | | | |
| 95449\_at | 2310075G12Rik | RIKEN cDNA 2310075G12 gene | chr11 | -1.383 | 0 | 0.016 |  | | | |
| 95520\_at | 2310061B02Rik | RIKEN cDNA 2310061B02 gene | chr1 | -2.378 | 0 | 0.04 |  | | | |
| 96049\_at | Bgn | biglycan | --- | -1.681 | 0 | 0.008 |  | | | |
| 96186\_at | Lrp10 | low-density lipoprotein receptor-related protein 10 | chr14 | -3.157 | 0 | 0.013 |  | | | |
| 96530\_at | NoneAvailable | Mus musculus transcribed sequences | --- | -1.094 | 0 | 0.002 |  | | | |
| 96886\_at | Stab1 | stabilin 1 | chr14 | -2.451 | 0 | 0.017 |  | | | |
| 96912\_s\_at | Ctla2a | cytotoxic T lymphocyte-associated protein 2 alpha | chr13 | -3.703 | 0 | 0.031 |  | | | |
| 97181\_f\_at | NoneAvailable | --- | --- | -1.659 | 0 | 0.002 |  | | | |
| 97336\_at | Ctsf | cathepsin F | chr19 | -1.534 | 0 | 0.002 |  | | | |
| 97448\_at | NoneAvailable | Mus musculus cDNA clone MGC:65558 IMAGE:6485174, complete cds | chr11 | -1.982 | 0 | 0.019 |  | | | |
| 97798\_at | 4930504E06Rik | RIKEN cDNA 4930504E06 gene | chr3 | -1.169 | 0 | 0.001 |  | | | |
| 98083\_at | Copeb | core promoter element binding protein | chr13 | -2.963 | 0 | 0 |  | | | |
| 98088\_at | Cd14 | CD14 antigen | --- | -3.218 | 0 | 0.018 |  | | | |
| 98254\_f\_at | NoneAvailable | --- | --- | -1.54 | 0 | 0.002 |  | | | |
| 98369\_f\_at | NoneAvailable | --- | --- | -1.265 | 0 | 0.002 |  | | | |
| 98451\_at | Dnajb10 | DnaJ (Hsp40) homolog, subfamily B, member 10 | chr1 | -3.902 | 0 | 0.002 |  | | | |
| \* Positive log2 fold changes represent genes expressed higher in FL-HSC; Negative log2 fold changes represent genes expressed higher in adult HSC (fold change=2 is equivalent to log2 fold change=1) | | | | | | | | | | |
|  |  |  |  |  |  |  |  |  |  |  |
