# Supplementary material for: Molecular Signatures of Proliferation and Quiescence in Hematopoietic Stem Cells
Source: PLoS Biol. 2004 Sep 28;2(10):e301. doi: 10.1371/journal.pbio.0020301 (PMC520599; doi:10.1371/journal.pbio.0020301)
Supplement: Table S13 — (70 KB HTML). [file pbio.0020301.st013.html]

|  |  | Q-sig TOM 1 |  |  |  |  |  |  |  |  |
| Probe Set ID | Gene Symbol | Gene name | Chromosome | Log2 Fold Change (FL-HSC vs Adult HSC)\* | Day of max (TOM) | p-value of ANOVA (time course) |  | | | |
| 100587\_f\_at | 5730403B10Rik | RIKEN cDNA 5730403B10 gene | chr16 | -2.058 | 1 | 0.009 |  | | | |
| 100998\_at | H2-Ab1 | histocompatibility 2, class II antigen A, beta 1 | chr17 | -1.116 | 1 | 0.026 |  | | | |
| 101568\_at | NoneAvailable | Mus musculus mRNA similar to proline synthetase co-transcribed (cDNA clone MGC:59396 IMAGE:6504579), complete cds | chr8 | -1.703 | 1 | 0.024 |  | | | |
| 101963\_at | Ctsl | cathepsin L | chr13 | -2.894 | 1 | 0.022 |  | | | |
| 101971\_at | 2500002L14Rik | RIKEN cDNA 2500002L14 gene | --- | -1.663 | 1 | 0.005 |  | | | |
| 101990\_at | Ldh2 | lactate dehydrogenase 2, B chain | chr6 | -1.289 | 1 | 0.001 |  | | | |
| 102332\_at | Ulk1 | Unc-51 like kinase 1 (C. elegans) | chr5 | -2.225 | 1 | 0.009 |  | | | |
| 102860\_at | Serpina3g | serine (or cysteine) proteinase inhibitor, clade A, member 3G | chr12 | -4.051 | 1 | 0.013 |  | | | |
| 102906\_at | Tgtp | T-cell specific GTPase | chr11 | -5.973 | 1 | 0.001 |  | | | |
| 102960\_at | Rga | recombination activating gene 1 gene activation | chr3 | -1.165 | 1 | 0.049 |  | | | |
| 103353\_f\_at | Cyp4b1 | cytochrome P450, family 4, subfamily b, polypeptide 1 | chr4 | -2.127 | 1 | 0.004 |  | | | |
| 103634\_at | Isgf3g | interferon dependent positive acting transcription factor 3 gamma | --- | -1.108 | 1 | 0.002 |  | | | |
| 103899\_at | Atp11a | ATPase, class VI, type 11A | chr8 | -1.465 | 1 | 0.049 |  | | | |
| 104597\_at | Gbp2 | guanylate nucleotide binding protein 2 | chr3 | -2.148 | 1 | 0.021 |  | | | |
| 104735\_at | AW538430 | expressed sequence AW538430 | chr14 | -1.525 | 1 | 0.049 |  | | | |
| 160127\_at | Ccng1 | cyclin G1 | chr11 | -1.675 | 1 | 0.007 |  | | | |
| 160502\_at | Creg | cellular repressor of E1A-stimulated genes | --- | -2.676 | 1 | 0.03 |  | | | |
| 160519\_at | Timp3 | tissue inhibitor of metalloproteinase 3 | chr10 | -3.836 | 1 | 0.007 |  | | | |
| 160724\_at | Usp49 | ubiquitin specific protease 49 | --- | -2.112 | 1 | 0 |  | | | |
| 160783\_at | D14Ertd436e | DNA segment, Chr 14, ERATO Doi 436, expressed | chr14 | -1.857 | 1 | 0.033 |  | | | |
| 160933\_at | Igtp | interferon gamma induced GTPase | chr11 | -3.814 | 1 | 0 |  | | | |
| 161666\_f\_at | Gadd45b | growth arrest and DNA-damage-inducible 45 beta | chr10 | -2.691 | 1 | 0.042 |  | | | |
| 162044\_f\_at | Cyp4b1 | cytochrome P450, family 4, subfamily b, polypeptide 1 | --- | -2.532 | 1 | 0.007 |  | | | |
| 92263\_at | Grcb | gene rich cluster, B gene | chr6 | -1.939 | 1 | 0.002 |  | | | |
| 92440\_at | Irf6 | interferon regulatory factor 6 | chr1 | -4.326 | 1 | 0.031 |  | | | |
| 92653\_at | D530037H12Rik | RIKEN cDNA D530037H12 gene | chr1 | -1.338 | 1 | 0.01 |  | | | |
| 92780\_f\_at | NoneAvailable | --- | --- | -2.077 | 1 | 0.017 |  | | | |
| 92847\_s\_at | M6pr | mannose-6-phosphate receptor, cation dependent | chr6 | -1.096 | 1 | 0.043 |  | | | |
| 92866\_at | H2-Aa | histocompatibility 2, class II antigen A, alpha | chr17 | -3.869 | 1 | 0.036 |  | | | |
| 92926\_at | Mpl | myeloproliferative leukemia virus oncogene | --- | -1.119 | 1 | 0.003 |  | | | |
| 93011\_at | Gabarapl1 | gamma-aminobutyric acid (GABA(A)) receptor-associated protein-like 1 | chr6 | -2.046 | 1 | 0.019 |  | | | |
| 93020\_at | Rex3 | reduced expression 3 | chrX | -1.019 | 1 | 0.009 |  | | | |
| 93039\_at | 1190003P12Rik | RIKEN cDNA 1190003P12 gene | chr15 | -1.465 | 1 | 0.029 |  | | | |
| 93324\_at | Zfp36l1 | zinc finger protein 36, C3H type-like 1 | chr12 | -2.308 | 1 | 0.025 |  | | | |
| 93543\_f\_at | Gstm1 | glutathione S-transferase, mu 1 | chr5 | -1.363 | 1 | 0.008 |  | | | |
| 94269\_at | Rabac1 | Rab acceptor 1 (prenylated) | chr7 | -1.557 | 1 | 0.015 |  | | | |
| 94270\_at | Krt1-18 | keratin complex 1, acidic, gene 18 | --- | -3.531 | 1 | 0.013 |  | | | |
| 94821\_at | Xbp1 | X-box binding protein 1 | --- | -2.107 | 1 | 0.021 |  | | | |
| 94835\_f\_at | Tubb2 | tubulin, beta 2 | chr13 | -2.52 | 1 | 0.002 |  | | | |
| 94881\_at | Cdkn1a | cyclin-dependent kinase inhibitor 1A (P21) | chr17 | -2.727 | 1 | 0.002 |  | | | |
| 95505\_at | Tor1b | torsin family 1, member B | --- | -1.061 | 1 | 0.039 |  | | | |
| 95508\_at | Nckap1 | NCK-associated protein 1 | chr2 | -4.402 | 1 | 0.021 |  | | | |
| 95737\_at | 1200015A19Rik | RIKEN cDNA 1200015A19 gene | chr4 | -2.325 | 1 | 0.023 |  | | | |
| 96146\_at | Btg3 | B-cell translocation gene 3 | chr16 | -2.704 | 1 | 0.035 |  | | | |
| 96596\_at | Ndrl | N-myc downstream regulated-like | chr15 | -4.908 | 1 | 0.007 |  | | | |
| 96614\_at | 4933426M11Rik | RIKEN cDNA 4933426M11 gene | chr12 | -1.2 | 1 | 0.039 |  | | | |
| 96703\_at | Maged1 | melanoma antigen, family D, 1 | chrX | -2.002 | 1 | 0.003 |  | | | |
| 96728\_at | DXImx38e | DNA segment, Chr X, Immunex 38, expressed | chrX | -2.856 | 1 | 0.001 |  | | | |
| 96752\_at | Icam1 | intercellular adhesion molecule | chr9 | -2.045 | 1 | 0.024 |  | | | |
| 96764\_at | Iigp-pending | interferon-inducible GTPase | --- | -5.077 | 1 | 0.009 |  | | | |
| 96876\_at | Laptm4a | lysosomal-associated protein transmembrane 4A | chr12 | -1.037 | 1 | 0.039 |  | | | |
| 96935\_at | 2700030M23Rik | RIKEN cDNA 2700030M23 gene | chr4 | -2.968 | 1 | 0.003 |  | | | |
| 97409\_at | Ifi1 | interferon inducible protein 1 | chr11 | -1.031 | 1 | 0.034 |  | | | |
| 97540\_f\_at | H2-D1 | histocompatibility 2, D region locus 1 | --- | -3.787 | 1 | 0.002 |  | | | |
| 97549\_at | Cfl2 | cofilin 2, muscle | chr12 | -2.749 | 1 | 0.03 |  | | | |
| 97890\_at | Sgk | serum/glucocorticoid regulated kinase | chr10 | -3.498 | 1 | 0.048 |  | | | |
| 97908\_at | 1110007A06Rik | RIKEN cDNA 1110007A06 gene | chr6 | -1.119 | 1 | 0.04 |  | | | |
| 97973\_at | Tal1 | T-cell acute lymphocytic leukemia 1 | chr4 | -1.383 | 1 | 0.032 |  | | | |
| 98067\_at | Cdkn1a | cyclin-dependent kinase inhibitor 1A (P21) | chr17 | -2.741 | 1 | 0.001 |  | | | |
| 98410\_at | Gtpi-pending | interferon-g induced GTPase | chr11 | -2.836 | 1 | 0.046 |  | | | |
| 98472\_at | H2-T23 | histocompatibility 2, T region locus 23 | --- | -1.61 | 1 | 0 |  | | | |
| 99109\_at | Ier2 | immediate early response 2 | chr8 | -2.892 | 1 | 0.003 |  | | | |
| 99133\_at | Slc3a2 | solute carrier family 3 (activators of dibasic and neutral amino acid transport), member 2 | chr19 | -1.113 | 1 | 0.018 |  | | | |
| 99366\_at | E030024M05Rik | RIKEN cDNA E030024M05 gene | chr12 | -2.996 | 1 | 0.014 |  | | | |
| 99532\_at | Tob1 | transducer of ErbB-2.1 | chr11 | -5.048 | 1 | 0.008 |  | | | |
| \* Positive log2 fold changes represent genes expressed higher in FL-HSC; Negative log2 fold changes represent genes expressed higher in adult HSC (fold change=2 is equivalent to log2 fold change=1) | | | | | | | | | | |
|  |  |  |  |  |  |  |  |  |  |  |
