# Supplementary material for: Molecular Signatures of Proliferation and Quiescence in Hematopoietic Stem Cells
Source: PLoS Biol. 2004 Sep 28;2(10):e301. doi: 10.1371/journal.pbio.0020301 (PMC520599; doi:10.1371/journal.pbio.0020301)
Supplement: Table S14 — (169 KB HTML). [file pbio.0020301.st014.html]

|  |  | Q-sig TOM 10 |  |  |  |  |  |  |  |  |
| Probe Set ID | Gene Symbol | Gene name | Chromosome | Genome Alignment | Log2 Fold Change (FL-HSC vs Adult HSC)\* | Day of max (TOM) | p-value of ANOVA (time course) |  | | |
| 100030\_at | Upp1 | uridine phosphorylase 1 | chr11 | 9209419-9211354 | -1.097 | 10 | 0.005 |  | | |
| 100134\_at | Eng | endoglin | chr2 | 32952268-32952660 | -1.282 | 10 | 0.012 |  | | |
| 100136\_at | Lamp2 | lysosomal membrane glycoprotein 2 | chrX | 24463512-24482695 | -1.749 | 10 | 0.025 |  | | |
| 100475\_at | Trim25 | tripartite motif protein 25 | --- | --- | -1.645 | 10 | 0 |  | | |
| 100606\_at | Prnp | prion protein | --- | --- | -1.719 | 10 | 0.036 |  | | |
| 100635\_at | Sara | SAR1a gene homolog (S. cerevisiae) | chr10 | 61340995-61341281 | -1.295 | 10 | 0.009 |  | | |
| 100973\_i\_at | Ccl27 | chemokine (C-C motif) ligand 27 | --- | --- | -3.092 | 10 | 0 |  | | |
| 100988\_at | Bcl2l11 | BCL2-like 11 (apoptosis facilitator) | chr2 | 129808315-129808820 | -1.027 | 10 | 0.014 |  | | |
| 101079\_at | Nxf1 | nuclear RNA export factor 1 homolog (S. cerevisiae) | chr19 | 7912136-7913991 | -2.424 | 10 | 0.003 |  | | |
| 101144\_at | Il18r1 | interleukin 18 receptor 1 | --- | --- | -1.522 | 10 | 0.037 |  | | |
| 101186\_at | Ppnr-pending | per-pentamer repeat gene | chr19 | 55615487-55616006 | -1.941 | 10 | 0.019 |  | | |
| 101441\_i\_at | Itpr5 | inositol 1,4,5-triphosphate receptor 5 | chr6 | 146465989-146466030 | -3.278 | 10 | 0.019 |  | | |
| 101836\_at | Ppm1b | protein phosphatase 1B, magnesium dependent, beta isoform | chr17 | 83686970-83687694 | -1.457 | 10 | 0.007 |  | | |
| 101884\_at | Xlr4 | X-linked lymphocyte-regulated 4 | chrX | 57370997-57371163 | -2.072 | 10 | 0.038 |  | | |
| 101943\_at | Tceb3 | transcription elongation factor B (SIII), polypeptide 3 | chr4 | 134065094-134065486 | -1.496 | 10 | 0.002 |  | | |
| 101947\_at | Nakap95-pending | neighbor of A-kinase anchoring protein 95 | chr17 | 31035727-31046959 | -1.799 | 10 | 0.001 |  | | |
| 102279\_at | 1300004C08Rik | RIKEN cDNA 1300004C08 gene | chr9 | 108160963-108162838 | -2.168 | 10 | 0.008 |  | | |
| 102313\_at | Gch | GTP cyclohydrolase 1 | chr14 | 38490650-38503128 | -4.039 | 10 | 0.005 |  | | |
| 102658\_at | Il1r2 | interleukin 1 receptor, type II | chr1 | 40543496-40551371 | -1.46 | 10 | 0 |  | | |
| 102663\_at | Plaur | urokinase plasminogen activator receptor | --- | --- | -2.014 | 10 | 0.036 |  | | |
| 102781\_at | Ccnl2 | cyclin L2 | chr4 | 152475875-152476592 | -1.306 | 10 | 0.005 |  | | |
| 102787\_at | Gpr56 | G protein-coupled receptor 56 | chr8 | 94375751-94376164 | -1.614 | 10 | 0.021 |  | | |
| 102836\_at | Pps | putative phosphatase | chr11 | 76433585-76434123 | -1.69 | 10 | 0 |  | | |
| 103015\_at | Bcl6 | B-cell leukemia/lymphoma 6 | chr16 | 23727212-23727474 | -4.658 | 10 | 0.02 |  | | |
| 103210\_at | Csf2rb2 | colony stimulating factor 2 receptor, beta 2, low-affinity (granulocyte-macrophage) | chr15 | 78517027-78517468 | -1.2 | 10 | 0.02 |  | | |
| 103254\_at | Fln29-pending | FLN29 gene product | chr5 | 118699646-118699842 | -2.179 | 10 | 0.011 |  | | |
| 103422\_at | Cd1d1 | CD1d1 antigen | chr3 | 88030604-88030951 | -2.191 | 10 | 0.001 |  | | |
| 103443\_at | Aim1 | absent in melanoma 1 | chr10 | 43795793-43796259 | -1.49 | 10 | 0.005 |  | | |
| 103518\_at | Ctla2b | cytotoxic T lymphocyte-associated protein 2 beta | chr13 | 60062176-60062776 | -5.219 | 10 | 0.015 |  | | |
| 103596\_at | Dgka | diacylglycerol kinase, alpha | chr10 | 128747738-128748578 | -3.305 | 10 | 0 |  | | |
| 103672\_at | 2410141M05Rik | RIKEN cDNA 2410141M05 gene | chr11 | 70658794-70659302 | -1.198 | 10 | 0.001 |  | | |
| 103812\_at | Clca1 | chloride channel calcium activated 1 | chr3 | 145384040-145384431 | -4.597 | 10 | 0.005 |  | | |
| 103895\_at | AW549877 | expressed sequence AW549877 | chr15 | 3807086-3807665 | -1.037 | 10 | 0.03 |  | | |
| 104144\_at | Gtpbp2 | GTP binding protein 2 | chr17 | 44421514-44422003 | -1.316 | 10 | 0.016 |  | | |
| 104165\_at | Vnn1 | vanin 1 | --- | --- | -2.98 | 10 | 0.003 |  | | |
| 104206\_at | 0610012A05Rik | RIKEN cDNA 0610012A05 gene | chr15 | 31281756-31282019 | -7.151 | 10 | 0.003 |  | | |
| 104256\_at | Pscdbp | pleckstrin homology, Sec7 and coiled-coil domains, binding protein | chr2 | 58944141-58944498 | -2.279 | 10 | 0.022 |  | | |
| 104257\_g\_at | Pscdbp | pleckstrin homology, Sec7 and coiled-coil domains, binding protein | chr2 | 58944053-58944117 | -2.012 | 10 | 0.004 |  | | |
| 104263\_at | 9330177P20Rik | RIKEN cDNA 9330177P20 gene | chr4 | 125143351-125143783 | -1.251 | 10 | 0.004 |  | | |
| 104311\_at | 1300013G12Rik | RIKEN cDNA 1300013G12 gene | chr1 | 128531600-128532068 | -1.417 | 10 | 0.024 |  | | |
| 104371\_at | Dgat1 | diacylglycerol O-acyltransferase 1 | chr15 | 76735158-76735691 | -3.266 | 10 | 0 |  | | |
| 104453\_at | NoneAvailable | Mus musculus cDNA clone IMAGE:6433799, partial cds | chr11 | 58435225-58437369 | -1.124 | 10 | 0.03 |  | | |
| 104572\_at | Etohd2 | ethanol decreased 2 | chr13 | 58940237-58940789 | -1.512 | 10 | 0.001 |  | | |
| 104677\_at | LOC227619 | hypothetical protein LOC227619 | chr2 | 25616701-25617000 | -1.818 | 10 | 0.046 |  | | |
| 104701\_at | Bhlhb2 | basic helix-loop-helix domain containing, class B2 | chr6 | 109333019-109333316 | -3.085 | 10 | 0.023 |  | | |
| 104741\_at | 9530098M12Rik | RIKEN cDNA 9530098M12 gene | chrX | 34427112-34427480 | -1.016 | 10 | 0.015 |  | | |
| 104745\_at | Arl6ip2 | ADP-ribosylation factor-like 6 interacting protein 2 | chr17 | 78479078-78479528 | -1.189 | 10 | 0.045 |  | | |
| 160088\_at | Fmo5 | flavin containing monooxygenase 5 | chr3 | 97993333-97993612 | -1.409 | 10 | 0.019 |  | | |
| 160099\_at | Lgals4 | lectin, galactose binding, soluble 4 | chr7 | 20439788-20440888 | -1.876 | 10 | 0.001 |  | | |
| 160151\_i\_at | 1200009B18Rik | RIKEN cDNA 1200009B18 gene | chr6 | 148557898-148558295 | -1.735 | 10 | 0.029 |  | | |
| 160264\_s\_at | 1500036F01Rik | RIKEN cDNA 1500036F01 gene | chr1 | 171916592-171916642 | -1.44 | 10 | 0.029 |  | | |
| 160287\_at | Map1lc3 | microtubule-associated protein 1 light chain 3 | chr14 | 39435970-39436212 | -1.669 | 10 | 0.02 |  | | |
| 160393\_at | 4930555L11Rik | RIKEN cDNA 4930555L11 gene | chr6 | 143588259-143588684 | -2.283 | 10 | 0.001 |  | | |
| 160495\_at | Ahr | aryl-hydrocarbon receptor | chr12 | 29655365-29655850 | -3.189 | 10 | 0.004 |  | | |
| 160834\_at | 1110032C13Rik | RIKEN cDNA 1110032C13 gene | chr7 | 19063567-19063694 | -4.49 | 10 | 0.04 |  | | |
| 160920\_at | Bcl2l2 | Bcl2-like 2 | chr14 | 45542417-45542930 | -1.72 | 10 | 0.02 |  | | |
| 160965\_at | AA793972 | EST AA793972 | chr5 | 133723946-133724003 | -3.294 | 10 | 0.001 |  | | |
| 160977\_at | Arhgef5 | Rho guanine nucleotide exchange factor (GEF) 5 | chr6 | 43299930-43300335 | -3.899 | 10 | 0.031 |  | | |
| 161081\_at | Cpeb2 | cytoplasmic polyadenylation element binding protein 2 | chr5 | 42154100-42154394 | -3.809 | 10 | 0.02 |  | | |
| 161109\_at | 1110017P05Rik | RIKEN cDNA 1110017P05 gene | --- | --- | -1.394 | 10 | 0.011 |  | | |
| 161113\_at | Esr1 | estrogen receptor 1 (alpha) | chr10 | 4723550-4723860 | -1.744 | 10 | 0.007 |  | | |
| 161551\_f\_at | Riok3 | RIO kinase 3 (yeast) | --- | --- | -1.464 | 10 | 0.012 |  | | |
| 161610\_at | Ndr2 | N-myc downstream regulated 2 | --- | --- | -4.394 | 10 | 0.001 |  | | |
| 161689\_f\_at | Il1r2 | interleukin 1 receptor, type II | chr1 | 40553181-40553322 | -3.446 | 10 | 0 |  | | |
| 161814\_f\_at | Rnf19 | ring finger protein (C3HC4 type) 19 | chr15 | 36269490-36269681 | -1.939 | 10 | 0.035 |  | | |
| 161980\_f\_at | Bag3 | Bcl2-associated athanogene 3 | chr7 | 117272836-117272952 | -4.017 | 10 | 0.037 |  | | |
| 162041\_f\_at | NoneAvailable | --- | --- | --- | -1.038 | 10 | 0.01 |  | | |
| 162206\_f\_at | Socs3 | suppressor of cytokine signaling 3 | --- | --- | -4.307 | 10 | 0.013 |  | | |
| 92542\_at | D4Wsu53e | DNA segment, Chr 4, Wayne State University 53, expressed | chr4 | 132986942-132987331 | -1.236 | 10 | 0.014 |  | | |
| 92877\_at | Tgfbi | transforming growth factor, beta induced | chr13 | 55768200-55768503 | -1.788 | 10 | 0.029 |  | | |
| 92992\_i\_at | 5730497N03Rik | RIKEN cDNA 5730497N03 gene | chr12 | 112670289-112670488 | -1.16 | 10 | 0.027 |  | | |
| 92993\_r\_at | 5730497N03Rik | RIKEN cDNA 5730497N03 gene | chr12 | 112670254-112670526 | -1.744 | 10 | 0.003 |  | | |
| 93104\_at | Btg1 | B-cell translocation gene 1, anti-proliferative | chr10 | 96141082-96141550 | -3.713 | 10 | 0.005 |  | | |
| 93193\_at | Adrb2 | adrenergic receptor, beta 2 | chr18 | 62546411-62546798 | -1.617 | 10 | 0.021 |  | | |
| 93274\_at | Clk | CDC-like kinase | chr1 | 59027649-59028843 | -1.211 | 10 | 0.031 |  | | |
| 93311\_at | Clk3 | CDC-like kinase 3 | chr9 | 57921213-57921503 | -1.389 | 10 | 0.003 |  | | |
| 93315\_at | Map2k3 | mitogen activated protein kinase kinase 3 | chr11 | 61476029-61476328 | -2.248 | 10 | 0.044 |  | | |
| 93414\_at | Abcb1b | ATP-binding cassette, sub-family B (MDR/TAP), member 1B | chr5 | 4999451-4999680 | -1.239 | 10 | 0.019 |  | | |
| 93424\_at | NoneAvailable | Mus musculus, Similar to KIAA0916 protein, clone IMAGE:4022573, mRNA | chr14 | 94157209-94157678 | -1.14 | 10 | 0.024 |  | | |
| 93440\_at | 4930564D15Rik | RIKEN cDNA 4930564D15 gene | chr3 | 98267531-98268063 | -1.001 | 10 | 0.017 |  | | |
| 93520\_at | Srrm1 | serine/arginine repetitive matrix 1 | --- | --- | -1.338 | 10 | 0.018 |  | | |
| 93753\_at | Litaf | LPS-induced TN factor | chr16 | 10444609-10445040 | -1.716 | 10 | 0.013 |  | | |
| 93852\_at | Mef2a | myocyte enhancer factor 2A | chr7 | 55360882-55361284 | -2.066 | 10 | 0.044 |  | | |
| 93914\_at | Il1r1 | interleukin 1 receptor, type I | chr1 | 40742717-40743273 | -1.087 | 10 | 0.006 |  | | |
| 93965\_r\_at | Ddx6 | DEAD (Asp-Glu-Ala-Asp) box polypeptide 6 | chr9 | 44683122-44683466 | -3.007 | 10 | 0.007 |  | | |
| 93975\_at | 1300002F13Rik | RIKEN cDNA 1300002F13 gene | chr4 | 147505100-147505327 | -6.877 | 10 | 0 |  | | |
| 94192\_at | Gdap10 | ganglioside-induced differentiation-associated-protein 10 | chr12 | 26966010-26966574 | -2.38 | 10 | 0.047 |  | | |
| 94264\_at | Raf1 | v-raf-1 leukemia viral oncogene 1 | chr6 | 116298435-116298833 | -1.251 | 10 | 0.001 |  | | |
| 94331\_at | Stat6 | signal transducer and activator of transcription 6 | chr10 | 127679531-127679934 | -2.796 | 10 | 0 |  | | |
| 94483\_at | Csnk2a2 | casein kinase II, alpha 2, polypeptide | chr8 | 94811181-94811405 | -1.076 | 10 | 0.04 |  | | |
| 94689\_at | C79248 | expressed sequence C79248 | --- | --- | -1.2 | 10 | 0.015 |  | | |
| 94780\_at | Zfp288 | zinc finger protein 288 | chr16 | 43537209-43537463 | -4.353 | 10 | 0.034 |  | | |
| 94818\_at | Ogt | O-linked N-acetylglucosamine (GlcNAc) transferase (UDP-N-acetylglucosamine:polypeptide-N-acetylglucosaminyl transferase) | chrX | 86499690-86499987 | -1.371 | 10 | 0.004 |  | | |
| 94830\_at | BC005537 | cDNA sequence BC005537 | chr13 | 24258669-24258959 | -1.83 | 10 | 0.008 |  | | |
| 94899\_at | Rhoip3-pending | Rho interacting protein 3 | chr11 | 60406863-60407397 | -1.473 | 10 | 0.042 |  | | |
| 94928\_at | Tnfrsf1b | tumor necrosis factor receptor superfamily, member 1b | --- | --- | -1.859 | 10 | 0.009 |  | | |
| 94939\_at | Cd53 | CD53 antigen | chr3 | 107167036-107167527 | -1.067 | 10 | 0.004 |  | | |
| 94980\_at | Dusp11 | dual specificity phosphatase 11 (RNA/RNP complex 1-interacting) | chr6 | 86446667-86448863 | -1.6 | 10 | 0.004 |  | | |
| 95023\_at | BC023957 | cDNA sequence BC023957 | chr9 | 45986784-45987315 | -1.742 | 10 | 0 |  | | |
| 95119\_at | 1110038D17Rik | RIKEN cDNA 1110038D17 gene | chr10 | 75299957-75300258 | -1.366 | 10 | 0.009 |  | | |
| 95287\_at | NoneAvailable | Mus musculus RIKEN cDNA 4930471C18 gene, mRNA (cDNA clone IMAGE:4487650), partial cds | chr6 | 38590511-38590909 | -1.232 | 10 | 0.032 |  | | |
| 95444\_at | 4930579A11Rik | RIKEN cDNA 4930579A11 gene | chr11 | 87361607-87362046 | -2.862 | 10 | 0.016 |  | | |
| 95489\_at | Fliih | flightless I homolog (Drosophila) | chr11 | 61239118-61239333 | -1.074 | 10 | 0.042 |  | | |
| 95521\_s\_at | Zfp68 | Zinc finger protein 68 | chr5 | 136163790-136163879 | -2.132 | 10 | 0.035 |  | | |
| 95564\_at | BC018601 | cDNA sequence BC018601 | chr11 | 5406382-5406767 | -2.789 | 10 | 0.032 |  | | |
| 95586\_at | P2rx4 | purinergic receptor P2X, ligand-gated ion channel 4 | chr5 | 120066833-120067054 | -1.711 | 10 | 0 |  | | |
| 95655\_at | 5830411E10Rik | RIKEN cDNA 5830411E10 gene | chr1 | 52056688-52057166 | -1.472 | 10 | 0.036 |  | | |
| 95917\_at | NoneAvailable | Mus musculus transcribed sequences | chr8 | 46984739-46985041 | -4.563 | 10 | 0.041 |  | | |
| 96176\_at | Arih2 | ariadne homolog 2 (Drosophila) | chr9 | 108767407-108767852 | -1.874 | 10 | 0.022 |  | | |
| 96189\_at | 2410141K03Rik | RIKEN cDNA 2410141K03 gene | --- | --- | -2.437 | 10 | 0.013 |  | | |
| 96534\_at | Vldlr | very low density lipoprotein receptor | chr19 | 26612865-26613082 | -1.008 | 10 | 0 |  | | |
| 96813\_f\_at | DXImx46e | DNA segment, Chr X, Immunex 46, expressed | chrX | 4042864-4043370 | -1.437 | 10 | 0.027 |  | | |
| 97118\_at | 1810028B20Rik | RIKEN cDNA 1810028B20 gene | chr19 | 56564259-56564753 | -1.614 | 10 | 0.036 |  | | |
| 97285\_f\_at | Ubxdc2 | UBX domain-containing 2 | chr17 | 54394270-54394653 | -2.548 | 10 | 0 |  | | |
| 97297\_at | 1500036F01Rik | RIKEN cDNA 1500036F01 gene | chr1 | 171916344-171916980 | -1.772 | 10 | 0.001 |  | | |
| 97319\_at | Rrad | Ras-related associated with diabetes | chr8 | 104140459-104140834 | -2.544 | 10 | 0.005 |  | | |
| 97349\_at | 4930488L10Rik | RIKEN cDNA 4930488L10 gene | chr12 | 65534848-65535148 | -2.819 | 10 | 0.04 |  | | |
| 97429\_at | Snrk | SNF related kinase | chr9 | 122461382-122461872 | -1.935 | 10 | 0.021 |  | | |
| 97843\_at | Ncoa4 | nuclear receptor coactivator 4 | chr12 | 113698051-113698407 | -1.253 | 10 | 0.003 |  | | |
| 97897\_at | NoneAvailable | Mus musculus, clone IMAGE:6430978, mRNA | chr13 | 46204847-46205403 | -1.708 | 10 | 0.027 |  | | |
| 98000\_at | Ly64 | lymphocyte antigen 64 | chr16 | 33608260-33608471 | -2.084 | 10 | 0.036 |  | | |
| 98018\_at | Procr | protein C receptor, endothelial | chr2 | 157601104-157601645 | -4.119 | 10 | 0.038 |  | | |
| 98461\_at | 1200014P03Rik | RIKEN cDNA 1200014P03 gene | chr17 | 44882317-44883629 | -1.307 | 10 | 0.007 |  | | |
| 98533\_at | Cyb5 | cytochrome b-5 | chr18 | 85330349-85330507 | -1.221 | 10 | 0.003 |  | | |
| 98882\_s\_at | Ndel1 | nuclear distribution gene E-like homolog 1 (A. nidulans) | chr11 | 69481044-69481233 | -1.562 | 10 | 0 |  | | |
| 98884\_r\_at | Ndel1 | nuclear distribution gene E-like homolog 1 (A. nidulans) | chr11 | 69480844-69481009 | -2.864 | 10 | 0.022 |  | | |
| 98926\_at | Vamp2 | vesicle-associated membrane protein 2 | chr11 | 69751167-69751707 | -2.233 | 10 | 0 |  | | |
| 98951\_at | D8Ertd325e | DNA segment, Chr 8, ERATO Doi 325, expressed | chr8 | 123096713-123097108 | -1.148 | 10 | 0.009 |  | | |
| 99045\_at | Eno2 | enolase 2, gamma neuronal | --- | --- | -1.634 | 10 | 0.008 |  | | |
| 99100\_at | Stat3 | signal transducer and activator of transcription 3 | chr11 | 101729696-101729826 | -1.118 | 10 | 0.022 |  | | |
| 99103\_at | Irf3 | interferon regulatory factor 3 | chr7 | 33630181-33631159 | -1.113 | 10 | 0.042 |  | | |
| 99143\_at | Tgoln1 | trans-golgi network protein | chr6 | 73076301-73076691 | -2.457 | 10 | 0.021 |  | | |
| 99184\_at | Csad | cysteine sulfinic acid decarboxylase | --- | --- | -2.259 | 10 | 0.001 |  | | |
| 99187\_f\_at | 2010315L10Rik | RIKEN cDNA 2010315L10 gene | chr8 | 70416051-70416494 | -1.207 | 10 | 0.004 |  | | |
| 99188\_at | 2010315L10Rik | RIKEN cDNA 2010315L10 gene | chr8 | 70414749-70416172 | -1.031 | 10 | 0.002 |  | | |
| 99347\_f\_at | NoneAvailable | Mus musculus transcribed sequences | --- | --- | -2.205 | 10 | 0.048 |  | | |
| 99445\_at | 1110028E10Rik | RIKEN cDNA 1110028E10 gene | chr9 | 21239942-21240316 | -1.202 | 10 | 0.035 |  | | |
| 99985\_at | Txnrd1 | thioredoxin reductase 1 | chr10 | 82477581-82478216 | -1.205 | 10 | 0.045 |  | | |
| \* Positive log2 fold changes represent genes expressed higher in FL-HSC; Negative log2 fold changes represent genes expressed higher in adult HSC (fold change=2 is equivalent to log2 fold change=1) | | | | | | | | | | |
|  |  |  |  |  |  |  |  |  |  |  |
