# Supplementary material for: Molecular Signatures of Proliferation and Quiescence in Hematopoietic Stem Cells
Source: PLoS Biol. 2004 Sep 28;2(10):e301. doi: 10.1371/journal.pbio.0020301 (PMC520599; doi:10.1371/journal.pbio.0020301)
Supplement: Table S15 — (45 KB HTML). [file pbio.0020301.st015.html]

|  |  | Q-sig TOM 30 |  |  |  |  |  |  |  |  |
| Probe Set ID | Gene Symbol | Gene name | Chromosome | Log2 Fold Change (FL-HSC vs Adult HSC)\* | Day of max (TOM) | p-value of ANOVA (time course) |  | | | |
| 100482\_at | BC023040 | cDNA sequence BC023040 | chr17 | -1.285 | 30 | 0.005 |  | | | |
| 100583\_at | Igh-VJ558 | immunoglobulin heavy chain (J558 family) | chr12 | -1.148 | 30 | 0.003 |  | | | |
| 102224\_at | Igf1r | insulin-like growth factor I receptor | --- | -1.399 | 30 | 0.011 |  | | | |
| 102302\_at | Bckdhb | branched chain ketoacid dehydrogenase E1, beta polypeptide | chr9 | -1.776 | 30 | 0.019 |  | | | |
| 102789\_at | Gata2 | GATA binding protein 2 | chr6 | -1.995 | 30 | 0.048 |  | | | |
| 103547\_at | Slc41a1 | solute carrier family 41, member 1 | chr1 | -2.728 | 30 | 0 |  | | | |
| 104083\_at | Cdh5 | cadherin 5 | chr8 | -1.968 | 30 | 0.003 |  | | | |
| 104376\_at | Hdac5 | histone deacetylase 5 | chr11 | -2.263 | 30 | 0.006 |  | | | |
| 104417\_at | NoneAvailable | Mus musculus transcribed sequences | chr11 | -2.062 | 30 | 0.001 |  | | | |
| 104645\_at | Klf7 | Kruppel-like factor 7 (ubiquitous) | chr1 | -1.19 | 30 | 0.003 |  | | | |
| 160228\_at | 1110019C08Rik | RIKEN cDNA 1110019C08 gene | chr16 | -1.025 | 30 | 0.025 |  | | | |
| 160651\_at | Tacstd2 | tumor-associated calcium signal transducer 2 | chr6 | -3.696 | 30 | 0 |  | | | |
| 160727\_at | 2410002F23Rik | RIKEN cDNA 2410002F23 gene | chr2 | -1.032 | 30 | 0.005 |  | | | |
| 161080\_f\_at | 1700012P16Rik | RIKEN cDNA 1700012P16 gene | chr5 | -1.148 | 30 | 0.008 |  | | | |
| 161184\_f\_at | Tie1 | tyrosine kinase receptor 1 | chr4 | -1.63 | 30 | 0.006 |  | | | |
| 161990\_f\_at | BC012974 | hypothetical gene supported by BC012974 | chr18 | -1.702 | 30 | 0.006 |  | | | |
| 92249\_g\_at | Nr4a2 | nuclear receptor subfamily 4, group A, member 2 | chr2 | -5.506 | 30 | 0.001 |  | | | |
| 92821\_at | Usp2 | ubiquitin specific protease 2 | chr9 | -1.119 | 30 | 0.004 |  | | | |
| 93875\_at | Hspa1a | heat shock protein 1A | chr17 | -1.082 | 30 | 0.006 |  | | | |
| 94060\_at | Myo1h | myosin 1H | chr5 | -1.495 | 30 | 0.002 |  | | | |
| 94657\_at | NoneAvailable | Mus musculus transcribed sequences | chr8 | -5.096 | 30 | 0.006 |  | | | |
| 94976\_at | AL022610 | expressed sequence AL022610 | chr7 | -1.012 | 30 | 0.029 |  | | | |
| 95002\_at | D17Wsu92e | DNA segment, Chr 17, Wayne State University 92, expressed | chr17 | -1.483 | 30 | 0.016 |  | | | |
| 95033\_at | Jmjd1 | jumonji domain containing 1 | chr6 | -1.446 | 30 | 0.007 |  | | | |
| 95618\_at | D6Ertd32e | DNA segment, Chr 6, ERATO Doi 32, expressed | chr6 | -2.616 | 30 | 0.038 |  | | | |
| 95805\_at | Cdc2l2 | cell division cycle 2 homolog (S. pombe)-like 2 | chr4 | -1.429 | 30 | 0.02 |  | | | |
| 96076\_at | Stx5a | syntaxin 5A | chr19 | -1.273 | 30 | 0.008 |  | | | |
| 96088\_at | Ndr2 | N-myc downstream regulated 2 | chr14 | -1.173 | 30 | 0.019 |  | | | |
| 96147\_at | Mafg | v-maf musculoaponeurotic fibrosarcoma oncogene family, protein G (avian) | chr11 | -1.157 | 30 | 0.03 |  | | | |
| 96367\_at | NoneAvailable | Mus musculus transcribed sequences | chr17 | -1.93 | 30 | 0.01 |  | | | |
| 96669\_at | 2400003C14Rik | RIKEN cDNA 2400003C14 gene | chr8 | -1.563 | 30 | 0.026 |  | | | |
| 97125\_f\_at | LOC56628 | MHC (A.CA/J(H-2K-f) class I antigen | chr17 | -3.415 | 30 | 0.007 |  | | | |
| 97375\_at | Pkd1 | polycystic kidney disease 1 homolog | chr17 | -1.292 | 30 | 0.009 |  | | | |
| 98065\_at | Ormdl3 | ORM1-like 3 (S. cerevisiae) | chr11 | -1.914 | 30 | 0.032 |  | | | |
| 98438\_f\_at | H2-Q7 | histocompatibility 2, Q region locus 7 | chr17 | -3.59 | 30 | 0.001 |  | | | |
| 98906\_at | Fbxo9 | f-box only protein 9 | chr9 | -2.278 | 30 | 0.037 |  | | | |
| 99961\_s\_at | Cdc2l2 | cell division cycle 2 homolog (S. pombe)-like 2 | chr4 | -1.587 | 30 | 0.025 |  | | | |
| 99970\_at | Ptpn21 | protein tyrosine phosphatase, non-receptor type 21 | chr12 | -1.262 | 30 | 0.008 |  | | | |
| \* Positive log2 fold changes represent genes expressed higher in FL-HSC; Negative log2 fold changes represent genes expressed higher in adult HSC (fold change=2 is equivalent to log2 fold change=1) | | | | | | | | | | |
|  |  |  |  |  |  |  |  |  |  |  |
