# Supplementary material for: Molecular Signatures of Proliferation and Quiescence in Hematopoietic Stem Cells
Source: PLoS Biol. 2004 Sep 28;2(10):e301. doi: 10.1371/journal.pbio.0020301 (PMC520599; doi:10.1371/journal.pbio.0020301)
Supplement: Table S16 — (52 KB HTML). [file pbio.0020301.st016.html]

|  |  | P-sig TOM 3 |  |  |  |  |  |  |  |  |  |
| Probe Set ID | Gene Symbol | Gene name | Chromosome | Log2 Fold Change (FL-HSC vs Adult HSC)\* | Day of max (TOM) | p-value of ANOVA (time course) |  | | | | |
| 100062\_at | Mcm3 | minichromosome maintenance deficient 3 (S. cerevisiae) | chr1 | 1.792 | 3 | 0.005 |  | | | | |
| 101372\_at | Trip13 | thyroid hormone receptor interactor 13 | chr13 | 1.578 | 3 | 0.014 |  | | | | |
| 101920\_at | Pole2 | polymerase (DNA directed), epsilon 2 (p59 subunit) | --- | 1.432 | 3 | 0.025 |  | | | | |
| 102047\_at | Nmt1 | N-myristoyltransferase 1 | chr11 | 1.098 | 3 | 0.008 |  | | | | |
| 102103\_f\_at | NoneAvailable | --- | --- | 1.173 | 3 | 0 |  | | | | |
| 102128\_f\_at | Mrps25 | mitochondrial ribosomal protein S25 | --- | 1.148 | 3 | 0.01 |  | | | | |
| 103064\_at | Chek1 | checkpoint kinase 1 homolog (S. pombe) | chr9 | 1.251 | 3 | 0.01 |  | | | | |
| 103201\_at | Ttk | Ttk protein kinase | chr9 | 2.66 | 3 | 0.018 |  | | | | |
| 103203\_f\_at | NoneAvailable | Mus musculus transcribed sequence with moderate similarity to protein ref:NP\_078956.1 (H.sapiens)  hypothetical protein FLJ23311 [Homo sapiens] | chr7 | 2.324 | 3 | 0.019 |  | | | | |
| 103204\_r\_at | NoneAvailable | Mus musculus transcribed sequence with moderate similarity to protein ref:NP\_078956.1 (H.sapiens)  hypothetical protein FLJ23311 [Homo sapiens] | chr7 | 1.738 | 3 | 0.048 |  | | | | |
| 103207\_at | Pola1 | polymerase (DNA directed), alpha 1 | chrX | 2.502 | 3 | 0.025 |  | | | | |
| 103212\_at | BC006933 | cDNA sequence BC006933 | chr12 | 1.151 | 3 | 0.045 |  | | | | |
| 103444\_at | E130315B21Rik | RIKEN cDNA E130315B21 gene | chr10 | 1.296 | 3 | 0.016 |  | | | | |
| 103821\_at | Cdc6 | cell division cycle 6 homolog (S. cerevisiae) | --- | 2.833 | 3 | 0.016 |  | | | | |
| 160069\_at | Gmnn | geminin | chr13 | 2.598 | 3 | 0.009 |  | | | | |
| 160496\_s\_at | Mcm3 | minichromosome maintenance deficient 3 (S. cerevisiae) | chr1 | 1.482 | 3 | 0.021 |  | | | | |
| 161122\_f\_at | Ndufab1 | NADH dehydrogenase (ubiquinone) 1, alpha/beta subcomplex, 1 | --- | 1.68 | 3 | 0.003 |  | | | | |
| 92551\_at | Lig1 | ligase I, DNA, ATP-dependent | chr7 | 1.152 | 3 | 0.033 |  | | | | |
| 92593\_at | Osf2-pending | osteoblast specific factor 2 (fasciclin I-like) | chr3 | 1.316 | 3 | 0.006 |  | | | | |
| 93445\_at | Cd5l | CD5 antigen-like | --- | 1.122 | 3 | 0.013 |  | | | | |
| 94228\_at | Xpo1 | exportin 1, CRM1 homolog (yeast) | chr11 | 1.337 | 3 | 0.001 |  | | | | |
| 94376\_s\_at | Mre11a | meiotic recombination 11 homolog A (S. cerevisiae) | chr9 | 2.127 | 3 | 0.035 |  | | | | |
| 94788\_f\_at | Tubb5 | tubulin, beta 5 | chr17 | 1.367 | 3 | 0.026 |  | | | | |
| 94907\_f\_at | 1110001J03Rik | RIKEN cDNA 1110001J03 gene | chr6 | 1.71 | 3 | 0.003 |  | | | | |
| 95063\_at | 2310021G01Rik | RIKEN cDNA 2310021G01 gene | chr2 | 2.4 | 3 | 0.041 |  | | | | |
| 95084\_f\_at | Grhpr | glyoxylate reductase/hydroxypyruvate reductase | chr4 | 2.569 | 3 | 0.006 |  | | | | |
| 95131\_f\_at | Ndufb2 | NADH dehydrogenase (ubiquinone) 1 beta subcomplex, 2 | chr2 | 1.121 | 3 | 0.003 |  | | | | |
| 95292\_at | Itga4 | integrin alpha 4 | chr2 | 1.618 | 3 | 0.02 |  | | | | |
| 95456\_r\_at | Shfdg1 | split hand/foot deleted gene 1 | chr6 | 1.322 | 3 | 0.026 |  | | | | |
| 95462\_at | Bzw2 | basic leucine zipper and W2 domains 2 | chr12 | 1.226 | 3 | 0.049 |  | | | | |
| 95527\_at | Chaf1a | chromatin assembly factor 1, subunit A (p150) | chr17 | 1.001 | 3 | 0 |  | | | | |
| 95612\_at | Rfc5 | replication factor C (activator 1) 5 | --- | 2.137 | 3 | 0.009 |  | | | | |
| 95732\_at | 1110005L13Rik | RIKEN cDNA 1110005L13 gene | chr10 | 2.644 | 3 | 0.028 |  | | | | |
| 95927\_f\_at | 2610201A13Rik | RIKEN cDNA 2610201A13 gene | chr11 | 1.536 | 3 | 0.02 |  | | | | |
| 96625\_at | D630024B06Rik | RIKEN cDNA D630024B06 gene | chr14 | 1.795 | 3 | 0.01 |  | | | | |
| 96686\_i\_at | 2010100O12Rik | RIKEN cDNA 2010100O12 gene | chr2 | 1.279 | 3 | 0.005 |  | | | | |
| 96687\_f\_at | 2010100O12Rik | RIKEN cDNA 2010100O12 gene | chr2 | 1.093 | 3 | 0 |  | | | | |
| 97095\_at | Bub1 | budding uninhibited by benzimidazoles 1 homolog (S. cerevisiae) | --- | 3.241 | 3 | 0.008 |  | | | | |
| 97393\_at | Vrk1 | vaccinia related kinase 1 | chr12 | 1.381 | 3 | 0.035 |  | | | | |
| 97411\_at | Ect2 | ect2 oncogene | chr3 | 2.597 | 3 | 0.019 |  | | | | |
| 98550\_at | Set | SET translocation | chr1 | 1.359 | 3 | 0.045 |  | | | | |
| 98618\_at | Dtymk | deoxythymidylate kinase | chr1 | 1.551 | 3 | 0.004 |  | | | | |
| 98929\_at | 1110018B13Rik | RIKEN cDNA 1110018B13 gene | chr13 | 1.14 | 3 | 0.032 |  | | | | |
| 98999\_at | Adsl | adenylosuccinate lyase | chr15 | 2.382 | 3 | 0.036 |  | | | | |
| 99457\_at | Mki67 | antigen identified by monoclonal antibody Ki 67 | chr7 | 2.708 | 3 | 0.002 |  | | | | |
| 99581\_at | Hint | histidine triad nucleotide binding protein | --- | 1.503 | 3 | 0.038 |  | | | | |
| \* Positive log2 fold changes represent genes expressed higher in FL-HSC; Negative log2 fold changes represent genes expressed higher in adult HSC (fold change=2 is equivalent to log2 fold change=1) | | | | | | | | | | | |
|  |  |  |  |  |  |  |  |  |  |  |  |
