# Supplementary material for: Molecular Signatures of Proliferation and Quiescence in Hematopoietic Stem Cells
Source: PLoS Biol. 2004 Sep 28;2(10):e301. doi: 10.1371/journal.pbio.0020301 (PMC520599; doi:10.1371/journal.pbio.0020301)
Supplement: Table S17 — (18 KB HTML). [file pbio.0020301.st017.html]

|  |  | P-sig TOM 2 |  |  |  |  |  |  |  |  |
| Probe Set ID | Gene Symbol | Gene name | Chromosome | Log2 Fold Change (FL-HSC vs Adult HSC)\* | Day of max (TOM) | p-value of ANOVA (time course) |  | | | |
| 100066\_at | Gart | phosphoribosylglycinamide formyltransferase | chr16 | 1.415 | 2 | 0.007 |  | | | |
| 101959\_r\_at | Tfdp1 | transcription factor Dp 1 | chr8 | 1.111 | 2 | 0.004 |  | | | |
| 103057\_at | Pold1 | polymerase (DNA directed), delta 1, catalytic subunit | chr7 | 3.176 | 2 | 0.034 |  | | | |
| 104303\_i\_at | 1500004O14Rik | RIKEN cDNA 1500004O14 gene | chr2 | 1.473 | 2 | 0.007 |  | | | |
| 104305\_at | Rarsl | arginyl-tRNA synthetase-like | chr4 | 2.295 | 2 | 0.005 |  | | | |
| 160341\_at | Jtv1-pending | JTV1 gene | chr5 | 2.15 | 2 | 0.033 |  | | | |
| 92794\_f\_at | Nme1 | expressed in non-metastatic cells 1, protein | chr11 | 2.156 | 2 | 0 |  | | | |
| 94450\_at | D13Wsu123e | DNA segment, Chr 13, Wayne State University 123, expressed | chr13 | 1.019 | 2 | 0.014 |  | | | |
| 96827\_at | Cad | carbamoyl-phosphate synthetase 2, aspartate transcarbamylase, and dihydroorotase | chr5 | 1.06 | 2 | 0.014 |  | | | |
| 98573\_r\_at | Ranbp1 | RAN binding protein 1 | chr16 | 1.404 | 2 | 0.046 |  | | | |
| 99158\_at | Sh3d3 | SH3 domain protein 3 | chr19 | 1.284 | 2 | 0.048 |  | | | |
| \* Positive log2 fold changes represent genes expressed higher in FL-HSC; Negative log2 fold changes represent genes expressed higher in adult HSC (fold change=2 is equivalent to log2 fold change=1) | | | | | | | | | | |
|  |  |  |  |  |  |  |  |  |  |  |
