# Supplementary material for: Molecular Signatures of Proliferation and Quiescence in Hematopoietic Stem Cells
Source: PLoS Biol. 2004 Sep 28;2(10):e301. doi: 10.1371/journal.pbio.0020301 (PMC520599; doi:10.1371/journal.pbio.0020301)
Supplement: Table S18 — (280 KB HTML). [file pbio.0020301.st018.html]

|  |  | P-sig TOM 6 |  |  |  |  |  |  |  |  |
| Probe Set ID | Gene Symbol | Gene name | Chromosome | Log2 Fold Change (FL-HSC vs Adult HSC)\* | Day of max (TOM) | p-value of ANOVA (time course) |  | | | |
| 100039\_at | Tmem4 | transmembrane protein 4 | chr10 | 1.81 | 6 | 0.003 |  | | | |
| 100057\_at | 2510027N19Rik | RIKEN cDNA 2510027N19 gene | chr7 | 3.001 | 6 | 0 |  | | | |
| 100059\_at | Cyba | cytochrome b-245, alpha polypeptide | chr8 | 1.77 | 6 | 0.038 |  | | | |
| 100073\_at | 2510005D08Rik | RIKEN cDNA 2510005D08 gene | chr14 | 2.121 | 6 | 0.012 |  | | | |
| 100079\_at | Ndufb9 | NADH dehydrogenase (ubiquinone) 1 beta subcomplex, 9 | chr15 | 1.058 | 6 | 0.013 |  | | | |
| 100116\_at | 2810417H13Rik | RIKEN cDNA 2810417H13 gene | chr1 | 2.111 | 6 | 0.026 |  | | | |
| 100128\_at | Cdc2a | cell division cycle 2 homolog A (S. pombe) | --- | 4.133 | 6 | 0.002 |  | | | |
| 100156\_at | Mcm5 | minichromosome maintenance deficient 5, cell division cycle 46 (S. cerevisiae) | chr8 | 5.137 | 6 | 0.012 |  | | | |
| 100331\_g\_at | Prdx2 | peroxiredoxin 2 | chr1 | 1.095 | 6 | 0.001 |  | | | |
| 100459\_at | Rad50 | RAD50 homolog (S. cerevisiae) | --- | 2.337 | 6 | 0.009 |  | | | |
| 100512\_at | Uchl5 | ubiquitin carboxyl-terminal esterase L5 | chr1 | 1.055 | 6 | 0.006 |  | | | |
| 100527\_at | D11Ertd99e | DNA segment, Chr 11, ERATO Doi 99, expressed | chr11 | 2.559 | 6 | 0.038 |  | | | |
| 100543\_s\_at | Brd7 | bromodomain containing 7 | chr8 | 1.094 | 6 | 0.003 |  | | | |
| 100568\_at | Abce1 | ATP-binding cassette, sub-family E (OABP), member 1 | --- | 1.485 | 6 | 0.011 |  | | | |
| 100576\_at | Pafah1b3 | platelet-activating factor acetylhydrolase, isoform 1b, alpha1 subunit | --- | 1.257 | 6 | 0.004 |  | | | |
| 100577\_at | Snrpd1 | small nuclear ribonucleoprotein D1 | chr18 | 1.295 | 6 | 0 |  | | | |
| 100592\_at | Ghitm | growth hormone inducible transmembrane protein | chr14 | 1.179 | 6 | 0.024 |  | | | |
| 100612\_at | Rrm1 | ribonucleotide reductase M1 | --- | 1.929 | 6 | 0.045 |  | | | |
| 100618\_f\_at | Slc25a5 | solute carrier family 25 (mitochondrial carrier; adenine nucleotide translocator), member 5 | --- | 1.078 | 6 | 0.012 |  | | | |
| 100628\_at | Ndufc1 | NADH dehydrogenase (ubiquinone) 1, subcomplex unknown, 1 | --- | 1.336 | 6 | 0.006 |  | | | |
| 100733\_at | Psma2 | proteasome (prosome, macropain) subunit, alpha type 2 | chr13 | 1.64 | 6 | 0.006 |  | | | |
| 100917\_at | NoneAvailable | --- | chr18 | 1.316 | 6 | 0.037 |  | | | |
| 101061\_at | Ssr2 | signal sequence receptor, beta | chr3 | 1.296 | 6 | 0.034 |  | | | |
| 101096\_s\_at | Hs1bp1 | HS1 binding protein | chr2 | 1.123 | 6 | 0.018 |  | | | |
| 101105\_at | Banf1 | barrier to autointegration factor 1 | chr19 | 2.053 | 6 | 0.002 |  | | | |
| 101254\_at | Ran | RAN, member RAS oncogene family | chr2 | 1.149 | 6 | 0.002 |  | | | |
| 101407\_at | Frda | Friedreich ataxia | chr19 | 1.288 | 6 | 0.027 |  | | | |
| 101421\_at | Rnf5 | ring finger protein 5 | chr17 | 1.067 | 6 | 0.044 |  | | | |
| 101486\_at | Psmb10 | proteasome (prosome, macropain) subunit, beta type 10 | chr8 | 1.623 | 6 | 0.012 |  | | | |
| 101506\_at | Snrpa1 | small nuclear ribonucleoprotein polypeptide A' | chr7 | 1.197 | 6 | 0.003 |  | | | |
| 101521\_at | Birc5 | baculoviral IAP repeat-containing 5 | chr11 | 2.475 | 6 | 0.01 |  | | | |
| 101558\_s\_at | Psmb5 | proteasome (prosome, macropain) subunit, beta type 5 | chr14 | 1.051 | 6 | 0.018 |  | | | |
| 101562\_at | Hsp70-4 | heat shock protein 4 | chr2 | 2.149 | 6 | 0.047 |  | | | |
| 101580\_at | Cox7b | cytochrome c oxidase subunit VIIb | chr1 | 1.791 | 6 | 0 |  | | | |
| 101890\_f\_at | Dnajc2 | DnaJ (Hsp40) homolog, subfamily C, member 2 | chr5 | 1.708 | 6 | 0.027 |  | | | |
| 101964\_at | Tkt | transketolase | chr14 | 1.087 | 6 | 0.001 |  | | | |
| 101992\_at | Psmb6 | proteasome (prosome, macropain) subunit, beta type 6 | chr11 | 1.47 | 6 | 0.016 |  | | | |
| 102001\_at | Rrm2 | ribonucleotide reductase M2 | chr12 | 1.74 | 6 | 0.02 |  | | | |
| 102019\_at | Mrpl13 | mitochondrial ribosomal protein L13 | chr15 | 1.841 | 6 | 0.001 |  | | | |
| 102022\_at | 1110007A04Rik | RIKEN cDNA 1110007A04 gene | --- | 1.16 | 6 | 0.009 |  | | | |
| 102409\_at | Lsm8 | LSM8 homolog, U6 small nuclear RNA associated (S. cerevisiae) | chr6 | 1.406 | 6 | 0.001 |  | | | |
| 102412\_at | AW541137 | expressed sequence AW541137 | chr10 | 2.049 | 6 | 0.032 |  | | | |
| 102631\_at | Blm | Bloom syndrome homolog (human) | chr7 | 1.408 | 6 | 0.003 |  | | | |
| 102821\_s\_at | Rasl2-9 | RAS-like, family 2, locus 9 | chr2 | 1.55 | 6 | 0 |  | | | |
| 102853\_at | Cspg6 | chondroitin sulfate proteoglycan 6 | chr19 | 1.265 | 6 | 0.037 |  | | | |
| 103089\_at | Cd48 | CD48 antigen | chr1 | 7.012 | 6 | 0 |  | | | |
| 103334\_at | Crcp | calcitonin gene-related peptide-receptor component protein | chr5 | 1.088 | 6 | 0.015 |  | | | |
| 103335\_at | Lgals9 | lectin, galactose binding, soluble 9 | chr11 | 2.142 | 6 | 0.019 |  | | | |
| 103418\_at | Rfc4 | replication factor C (activator 1) 4 | chr16 | 2.864 | 6 | 0.041 |  | | | |
| 103442\_at | BC003479 | cDNA sequence BC003479 | chr11 | 1.965 | 6 | 0.007 |  | | | |
| 103468\_at | Mns1 | meiosis-specific nuclear structural protein 1 | chr9 | 3.961 | 6 | 0.026 |  | | | |
| 103534\_at | NoneAvailable | --- | chr7 | 2.111 | 6 | 0.006 |  | | | |
| 103619\_at | 1810044O22Rik | RIKEN cDNA 1810044O22 gene | chr8 | 1.634 | 6 | 0.009 |  | | | |
| 103683\_at | Dhodh | dihydroorotate dehydrogenase | chr8 | 1.175 | 6 | 0.041 |  | | | |
| 103881\_at | 1110013G13Rik | RIKEN cDNA 1110013G13 gene | chr3 | 1.342 | 6 | 0.006 |  | | | |
| 103885\_at | 1500019O16Rik | RIKEN cDNA 1500019O16 gene | chr7 | 5.88 | 6 | 0.011 |  | | | |
| 104042\_at | Slc35b1 | solute carrier family 35, member B1 | chr17 | 1.214 | 6 | 0.037 |  | | | |
| 104044\_at | 1300006N24Rik | RIKEN cDNA 1300006N24 gene | chr9 | 1.315 | 6 | 0.044 |  | | | |
| 104057\_at | Grpel1 | GrpE-like 1, mitochondrial | chr5 | 1.548 | 6 | 0.026 |  | | | |
| 104077\_at | 1110049G11Rik | RIKEN cDNA 1110049G11 gene | chrX | 1.238 | 6 | 0.01 |  | | | |
| 104078\_g\_at | 1110049G11Rik | RIKEN cDNA 1110049G11 gene | chrX | 3.329 | 6 | 0.001 |  | | | |
| 104080\_at | Pdap1 | PDGFA associated protein 1 | chr5 | 2.918 | 6 | 0.01 |  | | | |
| 104145\_at | Tcof1 | Treacher Collins Franceschetti syndrome 1, homolog | chr18 | 1.073 | 6 | 0.043 |  | | | |
| 104147\_at | Nans | N-acetylneuraminic acid synthase (sialic acid synthase) | chr4 | 1.196 | 6 | 0.022 |  | | | |
| 104234\_at | Mrps25 | mitochondrial ribosomal protein S25 | chr6 | 1.243 | 6 | 0.029 |  | | | |
| 104237\_at | 2700061N24Rik | RIKEN cDNA 2700061N24 gene | chr13 | 1.881 | 6 | 0.016 |  | | | |
| 104297\_at | Ipo11 | importin 11 | chr13 | 1.447 | 6 | 0.02 |  | | | |
| 104301\_at | 2410018G20Rik | RIKEN cDNA 2410018G20 gene | chr16 | 1.631 | 6 | 0.002 |  | | | |
| 104322\_at | Ckap2 | cytoskeleton associated protein 2 | chr8 | 1.598 | 6 | 0.003 |  | | | |
| 104356\_at | 4921516M08Rik | RIKEN cDNA 4921516M08 gene | --- | 1.385 | 6 | 0 |  | | | |
| 104390\_at | Anp32a | acidic (leucine-rich) nuclear phosphoprotein 32 family, member A | --- | 1.176 | 6 | 0.045 |  | | | |
| 104423\_at | 2810047L02Rik | RIKEN cDNA 2810047L02 gene | chr1 | 3.018 | 6 | 0.009 |  | | | |
| 104476\_at | Rbl1 | retinoblastoma-like 1 (p107) | chr2 | 1.983 | 6 | 0.011 |  | | | |
| 104541\_at | Prtn3 | proteinase 3 | chr10 | 1.916 | 6 | 0.042 |  | | | |
| 104567\_at | Mrpl46 | mitochondrial ribosomal protein L46 | chr7 | 1.995 | 6 | 0.031 |  | | | |
| 104738\_at | Zrf2 | zuotin related factor 2 | chr6 | 1.861 | 6 | 0.008 |  | | | |
| 104762\_r\_at | 1500015J03Rik | RIKEN cDNA 1500015J03 gene | chr2 | 1.459 | 6 | 0.042 |  | | | |
| 104766\_at | Nola1 | nucleolar protein family A, member 1 (H/ACA small nucleolar RNPs) | --- | 1.457 | 6 | 0.002 |  | | | |
| 160076\_at | Mtx2 | metaxin 2 | chr2 | 1.76 | 6 | 0.035 |  | | | |
| 160135\_at | D16Ertd502e | DNA segment, Chr 16, ERATO Doi 502, expressed | chr16 | 1.284 | 6 | 0.007 |  | | | |
| 160152\_at | Psmc1 | protease (prosome, macropain) 26S subunit, ATPase 1 | chr16 | 1.442 | 6 | 0.02 |  | | | |
| 160176\_at | Hirip5 | histone cell cycle regulation defective interacting protein 5 | chr15 | 1.669 | 6 | 0 |  | | | |
| 160247\_at | Ube2v2 | ubiquitin-conjugating enzyme E2 variant 2 | chr15 | 1.044 | 6 | 0.003 |  | | | |
| 160293\_at | 2700038L12Rik | RIKEN cDNA 2700038L12 gene | chr9 | 1.194 | 6 | 0.048 |  | | | |
| 160297\_at | MGC36453 | hypothetical protein LOC381045 | chr14 | 2.932 | 6 | 0.003 |  | | | |
| 160299\_at | Rwdd1 | RWD domain containing 1 | chr10 | 1.08 | 6 | 0.034 |  | | | |
| 160324\_at | Rpa3 | replication protein A3 | chr1 | 1.959 | 6 | 0.009 |  | | | |
| 160431\_at | Mrpl12 | mitochondrial ribosomal protein L12 | chr11 | 2.077 | 6 | 0.009 |  | | | |
| 160503\_at | Fbl | fibrillarin | chr17 | 1.886 | 6 | 0 |  | | | |
| 160538\_at | Cdk4 | cyclin-dependent kinase 4 | chr10 | 2.296 | 6 | 0.032 |  | | | |
| 160569\_at | 2310008M10Rik | RIKEN cDNA 2310008M10 gene | chr3 | 1.341 | 6 | 0.006 |  | | | |
| 160585\_at | 2810470K21Rik | RIKEN cDNA 2810470K21 gene | --- | 2.814 | 6 | 0.011 |  | | | |
| 160723\_at | 1500001M20Rik | RIKEN cDNA 1500001M20 gene | chr6 | 1.3 | 6 | 0.044 |  | | | |
| 160876\_at | Bcap29 | B-cell receptor-associated protein 29 | chr12 | 1.484 | 6 | 0.023 |  | | | |
| 161147\_f\_at | 1110046L09Rik | RIKEN cDNA 1110046L09 gene | chr8 | 3.517 | 6 | 0.013 |  | | | |
| 161872\_f\_at | 1110049G11Rik | RIKEN cDNA 1110049G11 gene | --- | 1.026 | 6 | 0.01 |  | | | |
| 92540\_f\_at | Srm | spermidine synthase | chr4 | 6.091 | 6 | 0.001 |  | | | |
| 92574\_at | 3110001M13Rik | RIKEN cDNA 3110001M13 gene | chr9 | 1.899 | 6 | 0.011 |  | | | |
| 92615\_at | AI837181 | expressed sequence AI837181 | chr4 | 2.807 | 6 | 0.001 |  | | | |
| 92625\_at | Nme2 | expressed in non-metastatic cells 2, protein | chr10 | 1.732 | 6 | 0.004 |  | | | |
| 92636\_f\_at | Sec61g | SEC61, gamma subunit | chr1 | 1.533 | 6 | 0.004 |  | | | |
| 92646\_at | Mrpl23 | mitochondrial ribosomal protein L23 | chr2 | 2.152 | 6 | 0.028 |  | | | |
| 92788\_f\_at | Cetn3 | centrin 3 | chr13 | 1.678 | 6 | 0.018 |  | | | |
| 92798\_at | Atp5c1 | ATP synthase, H+ transporting, mitochondrial F1 complex, gamma polypeptide 1 | chrX | 1.327 | 6 | 0.003 |  | | | |
| 92799\_g\_at | Atp5c1 | ATP synthase, H+ transporting, mitochondrial F1 complex, gamma polypeptide 1 | chr2 | 1.541 | 6 | 0.001 |  | | | |
| 92824\_at | Nme6 | expressed in non-metastatic cells 6, protein | chr9 | 1.781 | 6 | 0.001 |  | | | |
| 92829\_at | Hspe1 | heat shock protein 1 (chaperonin 10) | chr1 | 1.429 | 6 | 0.001 |  | | | |
| 92831\_at | Sfxn1 | sideroflexin 1 | --- | 1.223 | 6 | 0.005 |  | | | |
| 92874\_f\_at | Cops7a | COP9 (constitutive photomorphogenic) homolog, subunit 7a (Arabidopsis thaliana) | chr6 | 1.098 | 6 | 0.003 |  | | | |
| 93008\_at | Lsm4 | LSM4 homolog, U6 small nuclear RNA associated (S. cerevisiae) | chr8 | 2.177 | 6 | 0.021 |  | | | |
| 93014\_at | Atp5l | ATP synthase, H+ transporting, mitochondrial F0 complex, subunit g | chr11 | 1.668 | 6 | 0.019 |  | | | |
| 93041\_at | Mcm4 | minichromosome maintenance deficient 4 homolog (S. cerevisiae) | chr16 | 1.922 | 6 | 0.039 |  | | | |
| 93062\_at | Mrpl39 | mitochondrial ribosomal protein L39 | chr16 | 1.936 | 6 | 0.016 |  | | | |
| 93112\_at | Mcm2 | minichromosome maintenance deficient 2 mitotin (S. cerevisiae) | chr6 | 2.139 | 6 | 0.018 |  | | | |
| 93236\_s\_at | Tyms | thymidylate synthase | chr10 | 3.228 | 6 | 0.012 |  | | | |
| 93237\_s\_at | Tyms | thymidylate synthase | chr10 | 2.144 | 6 | 0.021 |  | | | |
| 93258\_at | Hmbs | hydroxymethylbilane synthase | chr9 | 2.914 | 6 | 0.046 |  | | | |
| 93277\_at | Hspd1 | heat shock protein 1 (chaperonin) | chr1 | 1.14 | 6 | 0.029 |  | | | |
| 93290\_at | Pnp | purine-nucleoside phosphorylase | chr14 | 1.213 | 6 | 0.044 |  | | | |
| 93519\_s\_at | Nedd8 | neural precursor cell expressed, developmentally down-regulated gene 8 | chr14 | 2.012 | 6 | 0.002 |  | | | |
| 93531\_at | Ndufa8 | NADH dehydrogenase (ubiquinone) 1 alpha subcomplex, 8 | chr2 | 1.477 | 6 | 0.042 |  | | | |
| 93533\_at | 1500011L16Rik | RIKEN cDNA 1500011L16 gene | --- | 2.198 | 6 | 0.022 |  | | | |
| 93539\_at | 1810004D07Rik | RIKEN cDNA 1810004D07 gene | --- | 1.406 | 6 | 0 |  | | | |
| 93542\_at | Pter | phosphotriesterase related | chr2 | 1.123 | 6 | 0.022 |  | | | |
| 93548\_at | Sec61b | Sec61 beta subunit | chr4 | 2.149 | 6 | 0.005 |  | | | |
| 93559\_at | Apex1 | apurinic/apyrimidinic endonuclease 1 | chr14 | 1.957 | 6 | 0.002 |  | | | |
| 93560\_at | Acyp1 | acylphosphatase 1, erythrocyte (common) type | chr12 | 3.035 | 6 | 0.029 |  | | | |
| 93579\_at | 5830427H10Rik | RIKEN cDNA 5830427H10 gene | chr6 | 1.438 | 6 | 0.034 |  | | | |
| 93581\_at | Ndufb8 | NADH dehydrogenase (ubiquinone) 1 beta subcomplex 8 | chr19 | 2.79 | 6 | 0.003 |  | | | |
| 93582\_at | Coq7 | demethyl-Q 7 | chr7 | 3.745 | 6 | 0.001 |  | | | |
| 93596\_i\_at | Atp5e | ATP synthase, H+ transporting, mitochondrial F1 complex, epsilon subunit | chr2 | 1.737 | 6 | 0.006 |  | | | |
| 93735\_f\_at | Psmc3 | proteasome (prosome, macropain) 26S subunit, ATPase 3 | chr2 | 1.365 | 6 | 0.011 |  | | | |
| 93780\_at | Them2 | thioesterase superfamily member 2 | chr13 | 1.504 | 6 | 0.012 |  | | | |
| 93784\_at | Cfdp | craniofacial development protein 1 | chr8 | 1.424 | 6 | 0.031 |  | | | |
| 93786\_i\_at | Mrpl18 | mitochondrial ribosomal protein L18 | chr17 | 2.577 | 6 | 0.013 |  | | | |
| 93787\_f\_at | Mrpl18 | mitochondrial ribosomal protein L18 | --- | 2.215 | 6 | 0.009 |  | | | |
| 93812\_at | Clns1a | chloride channel, nucleotide-sensitive, 1A | chr7 | 1.373 | 6 | 0.031 |  | | | |
| 93820\_at | Cox7a2 | cytochrome c oxidase, subunit VIIa 2 | chr9 | 1.951 | 6 | 0.002 |  | | | |
| 93833\_s\_at | Hist1h2bc | histone 1, H2bc | chr13 | 1.06 | 6 | 0.043 |  | | | |
| 93838\_at | 2700038C09Rik | RIKEN cDNA 2700038C09 gene | chr2 | 3.273 | 6 | 0.025 |  | | | |
| 93984\_at | Atpi | ATPase inhibitor | --- | 1.332 | 6 | 0 |  | | | |
| 93988\_at | Psma7 | proteasome (prosome, macropain) subunit, alpha type 7 | chr2 | 1.392 | 6 | 0.02 |  | | | |
| 93991\_at | Mor1 | malate dehydrogenase, mitochondrial | chr5 | 1.136 | 6 | 0.009 |  | | | |
| 93993\_at | Lman2 | lectin, mannose-binding 2 | chr13 | 1.647 | 6 | 0.002 |  | | | |
| 94014\_at | 2510048O06Rik | RIKEN cDNA 2510048O06 gene | --- | 1.798 | 6 | 0 |  | | | |
| 94025\_at | Psmb3 | proteasome (prosome, macropain) subunit, beta type 3 | chr16 | 1.932 | 6 | 0.035 |  | | | |
| 94040\_at | Erh | enhancer of rudimentary homolog (Drosophila) | chr12 | 1.929 | 6 | 0.001 |  | | | |
| 94062\_at | Ndufv2 | NADH dehydrogenase (ubiquinone) flavoprotein 2 | chr17 | 1.3 | 6 | 0.045 |  | | | |
| 94210\_at | Timm9 | translocase of inner mitochondrial membrane 9 homolog (yeast) | chr10 | 3.713 | 6 | 0.022 |  | | | |
| 94263\_f\_at | Psmb7 | proteasome (prosome, macropain) subunit, beta type 7 | chr2 | 1.959 | 6 | 0.018 |  | | | |
| 94275\_at | Urod | uroporphyrinogen decarboxylase | chr4 | 1.492 | 6 | 0.015 |  | | | |
| 94277\_at | Mtx1 | metaxin 1 | chr3 | 1.276 | 6 | 0.007 |  | | | |
| 94294\_at | Ccnb2 | cyclin B2 | chr9 | 4.408 | 6 | 0.001 |  | | | |
| 94323\_at | D630012G11Rik | RIKEN cDNA D630012G11 gene | chr19 | 1.879 | 6 | 0 |  | | | |
| 94360\_at | 2700029M09Rik | RIKEN cDNA 2700029M09 gene | chr8 | 1.272 | 6 | 0.009 |  | | | |
| 94372\_at | Nudt1 | nudix (nucleoside diphosphate linked moiety X)-type motif 1 | --- | 3.289 | 6 | 0.003 |  | | | |
| 94455\_at | Lsm3 | LSM3 homolog, U6 small nuclear RNA associated (S. cerevisiae) | chr6 | 2.688 | 6 | 0.002 |  | | | |
| 94494\_at | Farsl | phenylalanine-tRNA synthetase-like | chr1 | 1.832 | 6 | 0.008 |  | | | |
| 94526\_at | D10Ertd214e | DNA segment, Chr 10, ERATO Doi 214, expressed | chr10 | 2.227 | 6 | 0 |  | | | |
| 94558\_g\_at | Gtf3a | general transcription factor III A | chr5 | 1.943 | 6 | 0.011 |  | | | |
| 94789\_r\_at | Tubb5 | tubulin, beta 5 | chr17 | 3.275 | 6 | 0.044 |  | | | |
| 94841\_at | Psma5 | proteasome (prosome, macropain) subunit, alpha type 5 | chr10 | 1.755 | 6 | 0.007 |  | | | |
| 94870\_f\_at | 2310075M17Rik | RIKEN cDNA 2310075M17 gene | chr11 | 1.008 | 6 | 0.02 |  | | | |
| 94892\_r\_at | Mea1 | male enhanced antigen 1 | chr17 | 1.005 | 6 | 0.017 |  | | | |
| 94912\_at | Mrps21 | mitochondrial ribosomal protein S21 | chr3 | 1.449 | 6 | 0.017 |  | | | |
| 94931\_at | 1810045K17Rik | RIKEN cDNA 1810045K17 gene | chr3 | 1.276 | 6 | 0.034 |  | | | |
| 94933\_at | BC008155 | cDNA sequence BC008155 | chr17 | 1.315 | 6 | 0.039 |  | | | |
| 95045\_at | 0610012D09Rik | RIKEN cDNA 0610012D09 gene | chr7 | 1.41 | 6 | 0 |  | | | |
| 95049\_at | Snrpd2 | small nuclear ribonucleoprotein D2 | chr10 | 1.485 | 6 | 0.03 |  | | | |
| 95053\_s\_at | Sdhb | succinate dehydrogenase complex, subunit B, iron sulfur (Ip) | chr4 | 2.681 | 6 | 0.023 |  | | | |
| 95067\_at | Mrpl2 | mitochondrial ribosomal protein L2 | chr17 | 1.667 | 6 | 0.023 |  | | | |
| 95091\_at | Sec13r | SEC13 related gene (S. cerevisiae) | --- | 1.672 | 6 | 0.001 |  | | | |
| 95132\_r\_at | Ndufb2 | NADH dehydrogenase (ubiquinone) 1 beta subcomplex, 2 | chr6 | 1.988 | 6 | 0.024 |  | | | |
| 95441\_at | Timm23 | translocase of inner mitochondrial membrane 23 homolog (yeast) | chr1 | 1.675 | 6 | 0.042 |  | | | |
| 95448\_at | Psmc2 | proteasome (prosome, macropain) 26S subunit, ATPase 2 | chr5 | 1.408 | 6 | 0.017 |  | | | |
| 95460\_at | Cops5 | COP9 (constitutive photomorphogenic) homolog, subunit 5 (Arabidopsis thaliana) | chr1 | 1.564 | 6 | 0.025 |  | | | |
| 95480\_at | D11Wsu68e | DNA segment, Chr 11, Wayne State University 68, expressed | chr11 | 1.471 | 6 | 0.003 |  | | | |
| 95491\_at | Park7 | Parkinson disease (autosomal recessive, early onset) 7 | chr4 | 1.248 | 6 | 0.002 |  | | | |
| 95497\_at | 1110005A05Rik | RIKEN cDNA 1110005A05 gene | chr9 | 3.276 | 6 | 0.011 |  | | | |
| 95498\_at | Mrps15 | mitochondrial ribosomal protein S15 | chr4 | 2.035 | 6 | 0.005 |  | | | |
| 95590\_at | Alg5 | asparagine-linked glycosylation 5 homolog (yeast, dolichyl-phosphate beta-glucosyltransferase) | chr3 | 1.43 | 6 | 0.003 |  | | | |
| 95593\_at | Golph2 | golgi phosphoprotein 2 | chr13 | 1.191 | 6 | 0.018 |  | | | |
| 95606\_at | Nsap1l-pending | NS1-associated protein 1-like | chr9 | 1.239 | 6 | 0.005 |  | | | |
| 95634\_at | 0610010K14Rik | RIKEN cDNA 0610010K14 gene | --- | 1.777 | 6 | 0.023 |  | | | |
| 95636\_at | 0610010K14Rik | RIKEN cDNA 0610010K14 gene | chr11 | 2.424 | 6 | 0.007 |  | | | |
| 95649\_at | Phf5a | PHD finger protein 5A | chr15 | 1.933 | 6 | 0.012 |  | | | |
| 95656\_i\_at | D13Wsu177e | DNA segment, Chr 13, Wayne State University 177, expressed | chr13 | 2.603 | 6 | 0.011 |  | | | |
| 95660\_at | 0610025L15Rik | RIKEN cDNA 0610025L15 gene | chr7 | 2.58 | 6 | 0 |  | | | |
| 95677\_at | 0610009C03Rik | RIKEN cDNA 0610009C03 gene | --- | 2.789 | 6 | 0.026 |  | | | |
| 95690\_at | 1110030L07Rik | RIKEN cDNA 1110030L07 gene | chr15 | 3.584 | 6 | 0.007 |  | | | |
| 95693\_at | Idh2 | isocitrate dehydrogenase 2 (NADP+), mitochondrial | chr7 | 1.921 | 6 | 0.021 |  | | | |
| 95696\_at | Txnl2 | thioredoxin-like 2 | chr4 | 1.145 | 6 | 0.009 |  | | | |
| 95698\_at | Ndufb7 | NADH dehydrogenase (ubiquinone) 1 beta subcomplex, 7 | chr8 | 2.162 | 6 | 0.008 |  | | | |
| 95707\_at | 2900010M23Rik | RIKEN cDNA 2900010M23 gene | chr17 | 2.087 | 6 | 0.032 |  | | | |
| 95714\_at | 0610009D07Rik | RIKEN cDNA 0610009D07 gene | chr12 | 1.473 | 6 | 0.027 |  | | | |
| 95718\_f\_at | Usmg5 | upregulated during skeletal muscle growth 5 | chr19 | 1.302 | 6 | 0.006 |  | | | |
| 95760\_at | 1110011K10Rik | RIKEN cDNA 1110011K10 gene | chr9 | 2.372 | 6 | 0.002 |  | | | |
| 95891\_at | NoneAvailable | Mus musculus transcribed sequences | chr16 | 3.953 | 6 | 0.017 |  | | | |
| 96016\_at | NoneAvailable | Mus musculus cDNA clone MGC:67366 IMAGE:5683334, complete cds | chr2 | 3.771 | 6 | 0.015 |  | | | |
| 96029\_at | Sf3a3 | splicing factor 3a, subunit 3, 60kDa | chr4 | 2.16 | 6 | 0.047 |  | | | |
| 96052\_at | Acp1 | acid phosphatase 1, soluble | --- | 1.712 | 6 | 0.022 |  | | | |
| 96081\_at | Tk1 | thymidine kinase 1 | chr11 | 2.893 | 6 | 0.014 |  | | | |
| 96089\_at | 4931406C07Rik | RIKEN cDNA 4931406C07 gene | chr9 | 1.515 | 6 | 0.003 |  | | | |
| 96112\_at | Etfa | electron transferring flavoprotein, alpha polypeptide | chr9 | 1.779 | 6 | 0.006 |  | | | |
| 96261\_at | NoneAvailable | Mus musculus cDNA clone MGC:67622 IMAGE:6410794, complete cds | chr4 | 1.384 | 6 | 0 |  | | | |
| 96267\_at | Ndufv1 | NADH dehydrogenase (ubiquinone) flavoprotein 1 | chr19 | 1.004 | 6 | 0.016 |  | | | |
| 96268\_at | Suclg1 | succinate-CoA ligase, GDP-forming, alpha subunit | chr6 | 1.173 | 6 | 0.015 |  | | | |
| 96289\_at | Stoml2 | stomatin (Epb7.2)-like 2 | chr4 | 2.901 | 6 | 0.001 |  | | | |
| 96291\_f\_at | NoneAvailable | Mus musculus cDNA clone IMAGE:6772417, with apparent retained intron | chr13 | 2.298 | 6 | 0 |  | | | |
| 96292\_r\_at | NoneAvailable | Mus musculus cDNA clone IMAGE:6772417, with apparent retained intron | chr13 | 1.544 | 6 | 0.01 |  | | | |
| 96293\_at | 2410015N17Rik | RIKEN cDNA 2410015N17 gene | chr7 | 4.422 | 6 | 0.006 |  | | | |
| 96319\_at | Cdc20 | cell division cycle 20 homolog (S. cerevisiae) | --- | 4.591 | 6 | 0.004 |  | | | |
| 96321\_at | Ndufa9 | NADH dehydrogenase (ubiquinone) 1 alpha subcomplex, 9 | chr6 | 1.871 | 6 | 0.001 |  | | | |
| 96353\_at | 1110021D01Rik | RIKEN cDNA 1110021D01 gene | chr13 | 2.999 | 6 | 0.003 |  | | | |
| 96613\_at | 5730536A07Rik | RIKEN cDNA 5730536A07 gene | chr9 | 2.658 | 6 | 0.014 |  | | | |
| 96627\_at | Ebp | phenylalkylamine Ca2+ antagonist (emopamil) binding protein | chrX | 1.299 | 6 | 0.009 |  | | | |
| 96652\_at | Mrpl28 | mitochondrial ribosomal protein L28 | chr17 | 1.368 | 6 | 0.026 |  | | | |
| 96668\_at | Timm17b | translocator of inner mitochondrial membrane b | chrX | 2.162 | 6 | 0.003 |  | | | |
| 96696\_at | Hrmt1l2 | heterogeneous nuclear ribonucleoproteins  methyltransferase-like 2 (S. cerevisiae) | chr7 | 2.608 | 6 | 0.013 |  | | | |
| 96743\_at | Skiip | SKI interacting protein | --- | 1.897 | 6 | 0.005 |  | | | |
| 96861\_at | Mrpl50 | mitochondrial ribosomal protein L50 | chr4 | 1.687 | 6 | 0.015 |  | | | |
| 96892\_at | Psma1 | proteasome (prosome, macropain) subunit, alpha type 1 | chr7 | 1.257 | 6 | 0.017 |  | | | |
| 96899\_at | Ndufs3 | NADH dehydrogenase (ubiquinone) Fe-S protein 3 | chr11 | 1.275 | 6 | 0.019 |  | | | |
| 96902\_at | 2900091E11Rik | RIKEN cDNA 2900091E11 gene | chr10 | 1.339 | 6 | 0.042 |  | | | |
| 96909\_at | Ndufab1 | NADH dehydrogenase (ubiquinone) 1, alpha/beta subcomplex, 1 | chr7 | 1.256 | 6 | 0.014 |  | | | |
| 96947\_at | Etfb | electron transferring flavoprotein, beta polypeptide | chr7 | 2.477 | 6 | 0 |  | | | |
| 96952\_at | Psma6 | proteasome (prosome, macropain) subunit, alpha type 6 | chr12 | 1.235 | 6 | 0.004 |  | | | |
| 97055\_s\_at | NoneAvailable | --- | chr16 | 1.087 | 6 | 0.01 |  | | | |
| 97164\_at | 2610207P08Rik | RIKEN cDNA 2610207P08 gene | --- | 3.233 | 6 | 0.011 |  | | | |
| 97179\_at | NoneAvailable | Mus musculus mRNA similar to putative c-Myc-responsive (cDNA clone MGC:54855 IMAGE:5388297), complete cds | --- | 2.605 | 6 | 0.001 |  | | | |
| 97201\_s\_at | Ndufa5 | NADH dehydrogenase (ubiquinone) 1 alpha subcomplex, 5 | chr6 | 1.857 | 6 | 0.001 |  | | | |
| 97220\_at | Dscr2 | Down syndrome critical region homolog 2 (human) | chr16 | 5.415 | 6 | 0 |  | | | |
| 97248\_at | Dbi | diazepam binding inhibitor | --- | 1.56 | 6 | 0 |  | | | |
| 97268\_i\_at | 0610010I12Rik | RIKEN cDNA 0610010I12 gene | chr3 | 1.265 | 6 | 0.003 |  | | | |
| 97274\_at | Psmd14 | proteasome (prosome, macropain) 26S subunit, non-ATPase, 14 | chr2 | 2.128 | 6 | 0.001 |  | | | |
| 97307\_f\_at | Ndufb5 | NADH dehydrogenase (ubiquinone) 1 beta subcomplex, 5 | --- | 1.376 | 6 | 0.003 |  | | | |
| 97342\_at | Mrps14 | mitochondrial ribosomal protein S14 | chr1 | 2.351 | 6 | 0.001 |  | | | |
| 97374\_at | 2810025M15Rik | RIKEN cDNA 2810025M15 gene | chr1 | 2.124 | 6 | 0.003 |  | | | |
| 97412\_at | 3300001G02Rik | RIKEN cDNA 3300001G02 gene | chr11 | 2.545 | 6 | 0.004 |  | | | |
| 97459\_at | Psma4 | proteasome (prosome, macropain) subunit, alpha type 4 | chr18 | 1.676 | 6 | 0.03 |  | | | |
| 97468\_at | Cks1 | CDC28 protein kinase 1 | chr3 | 2.843 | 6 | 0 |  | | | |
| 97477\_at | Timm8b | translocase of inner mitochondrial membrane 8 homolog b (yeast) | chr9 | 1.26 | 6 | 0.04 |  | | | |
| 97538\_at | Gus | beta-glucuronidase | chr5 | 1.607 | 6 | 0.033 |  | | | |
| 97758\_at | Prdx1 | peroxiredoxin 1 | chr8 | 1.211 | 6 | 0.01 |  | | | |
| 97807\_at | 1110021H02Rik | RIKEN cDNA 1110021H02 gene | chr1 | 1.1 | 6 | 0.015 |  | | | |
| 97820\_at | Galk1 | galactokinase 1 | chr11 | 2.703 | 6 | 0.004 |  | | | |
| 97824\_at | D11Ertd175e | DNA segment, Chr 11, ERATO Doi 175, expressed | chr11 | 1.494 | 6 | 0.006 |  | | | |
| 97828\_at | Siva-pending | Cd27 binding protein (Hindu God of destruction) | chr12 | 1.387 | 6 | 0 |  | | | |
| 97884\_at | Mrps11 | mitochondrial ribosomal protein S11 | chr7 | 1.959 | 6 | 0.019 |  | | | |
| 97907\_at | Lsm7 | LSM7 homolog, U6 small nuclear RNA associated (S. cerevisiae) | chr10 | 1.945 | 6 | 0.002 |  | | | |
| 97979\_at | Ppp1r7 | protein phosphatase 1, regulatory (inhibitor) subunit 7 | chr1 | 2.977 | 6 | 0.019 |  | | | |
| 98039\_at | 2410015M20Rik | RIKEN cDNA 2410015M20 gene | chr17 | 1.43 | 6 | 0.019 |  | | | |
| 98075\_at | G431001I09Rik | RIKEN cDNA G431001I09 gene | chr2 | 3.225 | 6 | 0.001 |  | | | |
| 98077\_at | Snrpd3 | small nuclear ribonucleoprotein D3 | chr10 | 1.794 | 6 | 0.001 |  | | | |
| 98092\_at | Plac8 | placenta-specific 8 | chr5 | 2.981 | 6 | 0.004 |  | | | |
| 98120\_at | Mrpl27 | mitochondrial ribosomal protein L27 | chr11 | 3.683 | 6 | 0.001 |  | | | |
| 98153\_at | Cct3 | chaperonin subunit 3 (gamma) | chr3 | 1.573 | 6 | 0.02 |  | | | |
| 98440\_at | Ltb4dh | leukotriene B4 12-hydroxydehydrogenase | chr4 | 3.855 | 6 | 0 |  | | | |
| 98492\_at | Cklfsf7 | chemokine-like factor super family 7 | chr9 | 1.661 | 6 | 0.036 |  | | | |
| 98524\_f\_at | NoneAvailable | --- | --- | 2.603 | 6 | 0.008 |  | | | |
| 98527\_at | Dci | dodecenoyl-Coenzyme A delta isomerase (3,2 trans-enoyl-Coenyme A isomerase) | chr17 | 1.306 | 6 | 0.011 |  | | | |
| 98610\_at | Mrps28 | mitochondrial ribosomal protein S28 | chr3 | 1.473 | 6 | 0.04 |  | | | |
| 98904\_at | Mrpl35 | mitochondrial ribosomal protein L35 | chr6 | 2.237 | 6 | 0.002 |  | | | |
| 98930\_at | Cope | coatomer protein complex, subunit epsilon | chr8 | 1.116 | 6 | 0.022 |  | | | |
| 98934\_at | 0610007P06Rik | RIKEN cDNA 0610007P06 gene | chr7 | 4.269 | 6 | 0 |  | | | |
| 98938\_at | 1500026D16Rik | RIKEN cDNA 1500026D16 gene | chr19 | 1.602 | 6 | 0.013 |  | | | |
| 98966\_at | Dbt | dihydrolipoamide branched chain transacylase E2 | chr3 | 1.257 | 6 | 0.033 |  | | | |
| 99106\_at | Cops6 | COP9 (constitutive photomorphogenic) homolog, subunit 6 (Arabidopsis thaliana) | chr5 | 2.396 | 6 | 0.029 |  | | | |
| 99128\_at | Atp5o | ATP synthase, H+ transporting, mitochondrial F1 complex, O subunit | chr16 | 1.704 | 6 | 0.021 |  | | | |
| 99129\_at | Clast3-pending | CD40 ligand-activated specific transcript 3 | chr18 | 1.773 | 6 | 0.039 |  | | | |
| 99148\_at | Fh1 | fumarate hydratase 1 | chr1 | 2.824 | 6 | 0.009 |  | | | |
| 99151\_at | 2610002K22Rik | RIKEN cDNA 2610002K22 gene | --- | 3.469 | 6 | 0.004 |  | | | |
| 99164\_at | 2010111E04Rik | RIKEN cDNA 2010111E04 gene | chr3 | 2.906 | 6 | 0.006 |  | | | |
| 99537\_at | Ruvbl1 | RuvB-like protein 1 | chr1 | 3.012 | 6 | 0.022 |  | | | |
| 99546\_at | Fkbp2 | FK506 binding protein 2 | --- | 2.273 | 6 | 0.001 |  | | | |
| 99566\_at | Tpi | triosephosphate isomerase | chr6 | 1.133 | 6 | 0.048 |  | | | |
| 99583\_at | Gstp2 | glutathione S-transferase, pi 2 | chr19 | 1.142 | 6 | 0.003 |  | | | |
| 99594\_at | Mrpl51 | mitochondrial ribosomal protein L51 | chr6 | 2.427 | 6 | 0 |  | | | |
| 99618\_at | 0710008D09Rik | RIKEN cDNA 0710008D09 gene | chr10 | 2.5 | 6 | 0 |  | | | |
| 99632\_at | Mad2l1 | MAD2 (mitotic arrest deficient, homolog)-like 1 (yeast) | chr6 | 1.589 | 6 | 0.018 |  | | | |
| 99651\_at | 2610209M04Rik | RIKEN cDNA 2610209M04 gene | chr6 | 1.176 | 6 | 0.024 |  | | | |
| \* Positive log2 fold changes represent genes expressed higher in FL-HSC; Negative log2 fold changes represent genes expressed higher in adult HSC (fold change=2 is equivalent to log2 fold change=1) | | | | | | | | | | |
|  |  |  |  |  |  |  |  |  |  |  |
