# Supplementary material for: Molecular Signatures of Proliferation and Quiescence in Hematopoietic Stem Cells
Source: PLoS Biol. 2004 Sep 28;2(10):e301. doi: 10.1371/journal.pbio.0020301 (PMC520599; doi:10.1371/journal.pbio.0020301)
Supplement: Table S19 — (2 KB HTML). [file pbio.0020301.st019.html]

| Gene lists GO results |  |  |
| TOM Day 1 | Full | Enriched |
| TOM Day 10 | Full | Enriched |
| Up in Adult HSC | Full | Enriched |
| TOM Day 0 | Full | Enriched |
| TOM Day 30 | Full | Enriched |
| TOM Day 3 | Full | Enriched |
| TOM Day 6 | Full | Enriched |
| Up in Fetal Liver | Full | Enriched |
|  |  |  |
