# Supplementary material for: Molecular Signatures of Proliferation and Quiescence in Hematopoietic Stem Cells
Source: PLoS Biol. 2004 Sep 28;2(10):e301. doi: 10.1371/journal.pbio.0020301 (PMC520599; doi:10.1371/journal.pbio.0020301)
Supplement: Table S20 — (7 KB HTML). [file pbio.0020301.st020.html]

| GO category: cell cycle |  |
| Q-sig |  |
| 102781\_at,104598\_at,160127\_at,160495\_at,94881\_at,95618\_at,95805\_at,96728\_at,98067\_at,99187\_f\_at,99188\_at,104735\_at,95917\_at,103990\_at,160901\_at,92926\_at,94264\_at,94483\_at,96147\_at,97375\_at | cyclin L2,dual specificity phosphatase 1,cyclin G1,aryl-hydrocarbon receptor,cyclin-dependent kinase inhibitor 1A (P21),DNA segment, Chr 6, ERATO Doi 32, expressed,cell division cycle 2 homolog (S. pombe)-like 1,DNA segment, Chr X, Immunex 38, expressed,cyclin-dependent kinase inhibitor 1A (P21),RIKEN cDNA 2010315L10 gene,RIKEN cDNA 2010315L10 gene,expressed sequence AW538430,expressed sequence AA407132,FBJ osteosarcoma oncogene B,FBJ osteosarcoma oncogene,myeloproliferative leukemia virus oncogene,v-raf-1 leukemia viral oncogene 1,casein kinase II, alpha 2, polypeptide,v-maf musculoaponeurotic fibrosarcoma oncogene family, protein G (avian),polycystic kidney disease 1 homolog |
|  |  |
| cQ-sig |  |
| 96728\_at,99187\_f\_at | DNA segment, Chr X, Immunex 38, expressed,RIKEN cDNA 2010315L10 gene |
|  |  |
| P-sig |  |
| 100062\_at,100128\_at,101959\_r\_at,102853\_at,103064\_at,103821\_at,104476\_at,160069\_at,160496\_s\_at,160538\_at,93112\_at,94294\_at,95527\_at,97095\_at,99632\_at,100156\_at,100612\_at,101920\_at,102001\_at,102631\_at,103057\_at,103207\_at,103418\_at,104738\_at,92551\_at,93041\_at,95612\_at,96289\_at,98550\_at,101254\_at,92788\_f\_at,99129\_at,94376\_s\_at,99457\_at,94933\_at,95891\_at,97411\_at,97412\_at | minichromosome maintenance deficient 3 (S. cerevisiae),cell division cycle 2 homolog A (S. pombe),transcription factor Dp 1,chondroitin sulfate proteoglycan 6,checkpoint kinase 1 homolog (S. pombe),cell division cycle 6 homolog (S. cerevisiae),retinoblastoma-like 1 (p107),geminin,minichromosome maintenance deficient 3 (S. cerevisiae),cyclin-dependent kinase 4,minichromosome maintenance deficient 2 mitotin (S. cerevisiae),cyclin B2,chromatin assembly factor 1, subunit A (p150),budding uninhibited by benzimidazoles 1 homolog (S. cerevisiae),MAD2 (mitotic arrest deficient, homolog)-like 1 (yeast),minichromosome maintenance deficient 5, cell division cycle 46 (S. cerevisiae),ribonucleotide reductase M1,polymerase (DNA directed), epsilon 2 (p59 subunit),ribonucleotide reductase M2,Bloom syndrome homolog (human),polymerase (DNA directed), delta 1, catalytic subunit,polymerase (DNA directed), alpha 1,expressed sequence AU040575,zuotin related factor 2,ligase I, DNA, ATP-dependent,minichromosome maintenance deficient 4 homolog (S. cerevisiae),replication factor C (activator 1) 5,stomatin (Epb7.2)-like 2,SET translocation,RAN, member RAS oncogene family,centrin 3,CD40 ligand-activated specific transcript 3,meiotic recombination 11 homolog A (S. cerevisiae),antigen identified by monoclonal antibody Ki 67,cDNA sequence BC008155,NA,ect2 oncogene,RIKEN cDNA 3300001G02 gene |
|  |  |
| cP-sig |  |
| 100062\_at,100128\_at,101959\_r\_at,102853\_at,103064\_at,103821\_at,104476\_at,160069\_at,160496\_s\_at,93112\_at,94294\_at,99632\_at,100156\_at,100612\_at,101920\_at,102001\_at,103057\_at,103207\_at,103418\_at,104738\_at,92551\_at,93041\_at,95612\_at,101254\_at,99129\_at,94376\_s\_at,99457\_at,95891\_at,97411\_at | minichromosome maintenance deficient 3 (S. cerevisiae),cell division cycle 2 homolog A (S. pombe),transcription factor Dp 1,chondroitin sulfate proteoglycan 6,checkpoint kinase 1 homolog (S. pombe),cell division cycle 6 homolog (S. cerevisiae),retinoblastoma-like 1 (p107),geminin,minichromosome maintenance deficient 3 (S. cerevisiae),minichromosome maintenance deficient 2 mitotin (S. cerevisiae),cyclin B2,MAD2 (mitotic arrest deficient, homolog)-like 1 (yeast),minichromosome maintenance deficient 5, cell division cycle 46 (S. cerevisiae),ribonucleotide reductase M1,polymerase (DNA directed), epsilon 2 (p59 subunit),ribonucleotide reductase M2,polymerase (DNA directed), delta 1, catalytic subunit,polymerase (DNA directed), alpha 1,expressed sequence AU040575,zuotin related factor 2,ligase I, DNA, ATP-dependent,minichromosome maintenance deficient 4 homolog (S. cerevisiae),replication factor C (activator 1) 5,RAN, member RAS oncogene family,CD40 ligand-activated specific transcript 3,meiotic recombination 11 homolog A (S. cerevisiae),antigen identified by monoclonal antibody Ki 67,NA,ect2 oncogene |
