# Supplementary material for: Molecular Signatures of Proliferation and Quiescence in Hematopoietic Stem Cells
Source: PLoS Biol. 2004 Sep 28;2(10):e301. doi: 10.1371/journal.pbio.0020301 (PMC520599; doi:10.1371/journal.pbio.0020301)
Supplement: Table S21 — (3 KB HTML). [file pbio.0020301.st021.html]

| GO category: cell-cell adhesion |  |
|  |  |
| Q-sig |  |
| 92558\_at,96752\_at,103518\_at,104083\_at | vascular cell adhesion molecule 1,intercellular adhesion molecule,cytotoxic T lymphocyte-associated protein 2 beta,expressed sequence AA408225 |
|  |  |
| cQ-sig |  |
| 103518\_at | cytotoxic T lymphocyte-associated protein 2 beta |
|  |  |
| P-sig |  |
| none |  |
|  |  |
| cP-sig |  |
| none |  |
