# Supplementary material for: Molecular Signatures of Proliferation and Quiescence in Hematopoietic Stem Cells
Source: PLoS Biol. 2004 Sep 28;2(10):e301. doi: 10.1371/journal.pbio.0020301 (PMC520599; doi:10.1371/journal.pbio.0020301)
Supplement: Table S22 — (6 KB HTML). [file pbio.0020301.st022.html]

|  | GO category enrichment in P-sig | | |
| GO category | Gene name | Probe set ID |  |
| ATP synthesis coupled electron transport | NADH dehydrogenase (ubiquinone) 1 beta subcomplex 9 | 100079\_at |  |
|  | NADH dehydrogenase (ubiquinone) flavoprotein 2 | 94062\_at |  |
|  | NADH dehydrogenase (ubiquinone) flavoprotein 1 | 96267\_at |  |
|  | NADH dehydrogenase (ubiquinone) Fe-S protein 3 | 96899\_at |  |
|  | RIKEN cDNA 2900091E11 gene | 96902\_at |  |
|  |  |  |  |
