# Supplementary material for: Molecular Signatures of Proliferation and Quiescence in Hematopoietic Stem Cells
Source: PLoS Biol. 2004 Sep 28;2(10):e301. doi: 10.1371/journal.pbio.0020301 (PMC520599; doi:10.1371/journal.pbio.0020301)
Supplement: Table S23 — (8 KB HTML). [file pbio.0020301.st023.html]

|  | GO category enrichment in P-sig | |
| GO category | Gene name | Probe set ID |
| DNA replication | minichromosome maintenance deficient 3 (S. cerevisiae) | 100062\_at |
|  | minichromosome maintenance deficient 5 | 100156\_at |
|  | �cell division cycle 46 (S. cerevisiae) | 100612\_at |
|  | ribonucleotide reductase M1 | 101920\_at |
|  | polymerase (DNA directed) epsilon 2 (p59 subunit) | 102001\_at |
|  | ribonucleotide reductase M2 | 102631\_at |
|  | Bloom syndrome homolog (human) | 103057\_at |
|  | polymerase (DNA directed) delta 1 catalytic subunit | 103207\_at |
|  | polymerase (DNA directed) alpha 1 | 103418\_at |
|  | expressed sequence AU040575 | 104738\_at |
|  | zuotin related factor 2 | 160496\_s\_at |
|  | minichromosome maintenance deficient 3 (S. cerevisiae) | 92551\_at |
|  | ligase I DNA ATP-dependent | 93041\_at |
|  | minichromosome maintenance deficient 4 homolog (S. cerevisiae) | 93112\_at |
|  | minichromosome maintenance deficient 2 mitotin (S. cerevisiae) | 95527\_at |
|  | chromatin assembly factor 1 subunit A (p150) | 95612\_at |
|  | replication factor C (activator 1) 5 | 96289\_at |
|  | stomatin (Epb7.2)-like 2SET translocation | 98550\_at |
|  |  |  |
