# Supplementary material for: Molecular Signatures of Proliferation and Quiescence in Hematopoietic Stem Cells
Source: PLoS Biol. 2004 Sep 28;2(10):e301. doi: 10.1371/journal.pbio.0020301 (PMC520599; doi:10.1371/journal.pbio.0020301)
Supplement: Table S24 — (6 KB HTML). [file pbio.0020301.st024.html]

|  | GO category enrichment in P-sig | | |
| GO category� | Gene name | Probe set ID |  |
| Cell cycle checkpoint | CD40 ligand-activated specific transcript 3 | 99129\_at |  |
|  | MAD2 (mitotic arrest deficient, homolog)-like 1 (yeast) | 99632\_at |  |
|  | checkpoint kinase 1 homolog (S. pombe) | 103064\_at |  |
|  |  |  |  |
