# Supplementary material for: Molecular Signatures of Proliferation and Quiescence in Hematopoietic Stem Cells
Source: PLoS Biol. 2004 Sep 28;2(10):e301. doi: 10.1371/journal.pbio.0020301 (PMC520599; doi:10.1371/journal.pbio.0020301)
Supplement: Table S25 — (7 KB HTML). [file pbio.0020301.st025.html]

|  | GO category enrichment in P-sig |  |
| GO category | Gene name | Probe set ID |
| Hydrogen transport | RIKEN cDNA 1500011L16 gene | 93533\_at |
|  | ATP synthase, H+ transporting, mitochondrial F1 complex, O subunit | 99128\_at |
|  | ATP synthase, H+ transporting, mitochondrial F1 complex, gamma polypeptide 1 | 92798\_at |
|  | ATP synthase, H+ transporting, mitochondrial F1 complex, gamma polypeptide 1 | 92799\_g\_at |
|  | RIKEN cDNA 2410043G19 gene | 93596\_i\_at |
|  | ATPase, H+ transporting, V1 subunit F | 95656\_i\_at |
|  | ATP synthase, H+ transporting, mitochondrial F0 complex, subunit g | 93014\_at |
|  |  |  |
