# Supplementary material for: Molecular Signatures of Proliferation and Quiescence in Hematopoietic Stem Cells
Source: PLoS Biol. 2004 Sep 28;2(10):e301. doi: 10.1371/journal.pbio.0020301 (PMC520599; doi:10.1371/journal.pbio.0020301)
Supplement: Table S26 — (7 KB HTML). [file pbio.0020301.st026.html]

|  | GO category enrichment in Q-sig |  |
| GO category | Gene name | Probe set ID |
| Regulation of cell cycle | FBJ osteosarcoma oncogene B | 103990\_at |
|  | cyclin G1 | 160127\_at |
|  | FBJ osteosarcoma oncogene | 160901\_at |
|  | myeloproliferative leukemia virus oncogene | 92926\_at |
|  | v-raf-1 leukemia viral oncogene 1 | 94264\_at |
|  | casein kinase II alpha 2 polypeptide | 94483\_at |
|  | v-maf musculoaponeurotic fibrosarcoma oncogene family protein G (avian) | 96147\_at |
|  | RIKEN cDNA 2010315L10 gene | 99187\_f\_at |
|  | RIKEN cDNA 2010315L10 gene | 99188\_at |
|  | cyclin-dependent kinase inhibitor 1A (P21) | 94881\_at |
|  | polycystic kidney disease 1 homolog | 97375\_at |
|  | cyclin-dependent kinase inhibitor 1A (P21) | 98067\_at |
|  |  |  |
