# Supplementary material for: Molecular Signatures of Proliferation and Quiescence in Hematopoietic Stem Cells
Source: PLoS Biol. 2004 Sep 28;2(10):e301. doi: 10.1371/journal.pbio.0020301 (PMC520599; doi:10.1371/journal.pbio.0020301)
Supplement: Table S27 — (9 KB HTML). [file pbio.0020301.st027.html]

|  | GO category enrichment in Q-sig |  |
| GO category | Gene name | Probe set ID |
| Defense response | histocompatibility 2 class II antigen A beta 1 | 100998\_at |
|  | CD1d1 antigen | 103422\_at |
|  | tumor-associated calcium signal transducer 2 | 160651\_at |
|  | histocompatibility 2 class II antigen A alpha | 92866\_at |
|  | histocompatibility 2 K region | 93120\_f\_at |
|  | CCR4 carbon catabolite repression 4-like (S. cerevisiae) | 93907\_f\_at |
|  | intercellular adhesion molecule | 96752\_at |
|  | MHC (A.CA/J(H-2K-f) class I antigen | 97125\_f\_at |
|  | histocompatibility 2 D region locus 1 | 97540\_f\_at |
|  | lymphocyte antigen 64 | 98000\_at |
|  | histocompatibility 2 Q region locus 7 | 98438\_f\_at |
|  | histocompatibility 2 T region locus 23 | 98472\_at |
|  | chemokine (C-C motif) ligand 27 | 100973\_i\_at |
|  | interferon dependent positive acting transcription factor 3 gamma | 103634\_at |
|  | ethanol decreased 2 | 104572\_at |
|  | guanylate nucleotide binding protein 2 | 104597\_at |
|  | CD14 antigen | 98088\_at |
|  | signal transducer and activator of transcription 3 | 99100\_at |
|  | NA | 100583\_at |
|  | immunoglobulin kappa chain variable 21 (V21) | 102156\_f\_at |
|  | proline synthetase co-transcribed | 101568\_at |
|  | mitogen activated protein kinase kinase 3 | 93315\_at |
|  | tumor necrosis factor receptor superfamily member 1b | 94928\_at |
|  |  |  |
