# Supplementary material for: Molecular Signatures of Proliferation and Quiescence in Hematopoietic Stem Cells
Source: PLoS Biol. 2004 Sep 28;2(10):e301. doi: 10.1371/journal.pbio.0020301 (PMC520599; doi:10.1371/journal.pbio.0020301)
Supplement: Table S28 — (7 KB HTML). [file pbio.0020301.st028.html]

|  | GO category enrichment in Q-sig | | | |
| GO category | Gene name | Probe set ID |  | |
| Protein kinase cascade | v-raf-1 leukemia viral oncogene 1 | 94264\_at |  | |
|  | polycystic kidney disease 1 homolog | 97375\_at |  | |
|  | signal transducer and activator of transcription 3 | 99100\_at |  | |
|  | expressed sequence AW536343 | 94060\_at |  | |
|  | mitogen activated protein kinase kinase 3 | 93315\_at |  | |
|  | growth arrest and DNA-damage-inducible 45 beta | 161666\_f\_at |  | |
|  |  |  |  |  |
