# Supplementary material for: Molecular Signatures of Proliferation and Quiescence in Hematopoietic Stem Cells
Source: PLoS Biol. 2004 Sep 28;2(10):e301. doi: 10.1371/journal.pbio.0020301 (PMC520599; doi:10.1371/journal.pbio.0020301)
Supplement: Table S29 — (6 KB HTML). [file pbio.0020301.st029.html]

|  | GO category enrichment in Q-sig | | |
| GO category | Gene name | Probe set ID |  |
| cell-cell adhesion | vascular cell adhesion molecule 1 | 92558\_at |  |
|  | intercellular adhesion molecule� | 96752\_at |  |
|  | cytotoxic T lymphocyte-associated protein 2 beta� | 103518\_at |  |
|  | expressed sequence AA408225 | 104083\_at |  |
|  |  |  |  |
