# Supplementary material for: Molecular Signatures of Proliferation and Quiescence in Hematopoietic Stem Cells
Source: PLoS Biol. 2004 Sep 28;2(10):e301. doi: 10.1371/journal.pbio.0020301 (PMC520599; doi:10.1371/journal.pbio.0020301)
Supplement: Table S30 — (92 KB HTML). [file pbio.0020301.st030.html]

   Full Tom Day 1   

# Full Tom Day 1

|  |  |  |  |  |  |  |  |  |  |  |
| --- | --- | --- | --- | --- | --- | --- | --- | --- | --- | --- |
| GOLevel | GOTerm | ProbeCount | ArrayCount | ListGOLevelCount | ArrayGoLevelCount | ListFq | ArrayFq | FoldChange | H-Pvalue | ProbeIds |
| 0 | Gene\_Ontology | 138 | NA | 138 | 0 | 1 | NA | NA | NA | 100064\_f\_at,104041\_at,104208\_at,160227\_s\_at,93294\_at,93318\_at,94799\_at,96752\_at,96785\_at,99577\_at,160885\_at,97060\_at,99340\_at,96764\_at,99532\_at,104063\_at,102737\_at,95478\_at,99909\_at,99366\_at,102809\_s\_at,104427\_at,160749\_at,96592\_at,101979\_at,102779\_at,161666\_f\_at,93347\_at,95547\_at,101584\_at,93252\_at,93536\_at,94502\_at,97890\_at,92653\_at,96596\_at,97973\_at,92926\_at,94246\_at,97107\_at,101059\_at,103899\_at,92780\_f\_at,92262\_at,92263\_at,94270\_at,160462\_f\_at,94522\_at,94835\_f\_at,160343\_at,93278\_at,160081\_at,94068\_at,97409\_at,160998\_at,93020\_at,93021\_at,160127\_at,94881\_at,96728\_at,98067\_at,104735\_at,95913\_at,96801\_at,101554\_at,101593\_at,101568\_at,160361\_at,92847\_s\_at,96876\_at,96936\_at,98569\_at,98887\_at,101398\_at,97984\_i\_at,97515\_at,161250\_at,100880\_at,100508\_at,104463\_at,100564\_at,160428\_at,96058\_s\_at,101990\_at,160090\_f\_at,103326\_at,92401\_at,102936\_at,100535\_at,160366\_at,95715\_at,95737\_at,95496\_at,100578\_at,100611\_at,99133\_at,101963\_at,96890\_at,101583\_at,93751\_at,101060\_at,103353\_f\_at,103574\_at,162044\_f\_at,94715\_at,96831\_at,103483\_at,94073\_at,160722\_at,160449\_at,102401\_at,103634\_at,104002\_at,160502\_at,160724\_at,160783\_at,92440\_at,93782\_at,93789\_s\_at,94821\_at,94823\_at,96703\_at,160898\_at,100629\_at,93543\_f\_at,102332\_at,94941\_at,101510\_at,103717\_at,94274\_at,100998\_at,92866\_at,94000\_at,94285\_at,97540\_f\_at,98472\_at,104597\_at,92198\_s\_at |
| 1 | biological\_process | 138 | 6769 | 138 | 6769 | 1 | 1 | 1 | 1 | 100064\_f\_at,104041\_at,104208\_at,160227\_s\_at,93294\_at,93318\_at,94799\_at,96752\_at,96785\_at,99577\_at,160885\_at,97060\_at,99340\_at,96764\_at,99532\_at,104063\_at,102737\_at,95478\_at,99909\_at,99366\_at,102809\_s\_at,104427\_at,160749\_at,96592\_at,101979\_at,102779\_at,161666\_f\_at,93347\_at,95547\_at,101584\_at,93252\_at,93536\_at,94502\_at,97890\_at,92653\_at,96596\_at,97973\_at,92926\_at,94246\_at,97107\_at,101059\_at,103899\_at,92780\_f\_at,92262\_at,92263\_at,94270\_at,160462\_f\_at,94522\_at,94835\_f\_at,160343\_at,93278\_at,160081\_at,94068\_at,97409\_at,160998\_at,93020\_at,93021\_at,160127\_at,94881\_at,96728\_at,98067\_at,104735\_at,95913\_at,96801\_at,101554\_at,101593\_at,101568\_at,160361\_at,92847\_s\_at,96876\_at,96936\_at,98569\_at,98887\_at,101398\_at,97984\_i\_at,97515\_at,161250\_at,100880\_at,100508\_at,104463\_at,100564\_at,160428\_at,96058\_s\_at,101990\_at,160090\_f\_at,103326\_at,92401\_at,102936\_at,100535\_at,160366\_at,95715\_at,95737\_at,95496\_at,100578\_at,100611\_at,99133\_at,101963\_at,96890\_at,101583\_at,93751\_at,101060\_at,103353\_f\_at,103574\_at,162044\_f\_at,94715\_at,96831\_at,103483\_at,94073\_at,160722\_at,160449\_at,102401\_at,103634\_at,104002\_at,160502\_at,160724\_at,160783\_at,92440\_at,93782\_at,93789\_s\_at,94821\_at,94823\_at,96703\_at,160898\_at,100629\_at,93543\_f\_at,102332\_at,94941\_at,101510\_at,103717\_at,94274\_at,100998\_at,92866\_at,94000\_at,94285\_at,97540\_f\_at,98472\_at,104597\_at,92198\_s\_at |
| 2 | cellular process | 78 | 3616 | 218 | 10540 | 0.358 | 0.343 | 1.043 | 0.346 | 100064\_f\_at,104041\_at,104208\_at,160227\_s\_at,93294\_at,93318\_at,94799\_at,96752\_at,96785\_at,99577\_at,160885\_at,97060\_at,99340\_at,96764\_at,99532\_at,104063\_at,102737\_at,95478\_at,99909\_at,99366\_at,102809\_s\_at,104427\_at,160749\_at,96592\_at,101979\_at,102779\_at,161666\_f\_at,93347\_at,95547\_at,101584\_at,93252\_at,93536\_at,94502\_at,97890\_at,92653\_at,96596\_at,97973\_at,92926\_at,94246\_at,97107\_at,101059\_at,103899\_at,92780\_f\_at,92262\_at,92263\_at,94270\_at,160462\_f\_at,94522\_at,94835\_f\_at,160343\_at,93278\_at,160081\_at,94068\_at,97409\_at,160998\_at,93020\_at,93021\_at,160127\_at,94881\_at,96728\_at,98067\_at,104735\_at,95913\_at,96801\_at,101554\_at,101593\_at,101568\_at,160361\_at,92847\_s\_at,96876\_at,96936\_at,98569\_at,98887\_at,101398\_at,97984\_i\_at,97515\_at,161250\_at,100880\_at |
| 3 | cell communication | 30 | 1550 | 214 | 10726 | 0.14 | 0.145 | 0.97 | 0.602 | 100064\_f\_at,104041\_at,104208\_at,160227\_s\_at,93294\_at,93318\_at,94799\_at,96752\_at,96785\_at,99577\_at,160885\_at,97060\_at,99340\_at,96764\_at,99532\_at,104063\_at,102737\_at,95478\_at,99909\_at,99366\_at,102809\_s\_at,104427\_at,160749\_at,96592\_at,101979\_at,102779\_at,161666\_f\_at,93347\_at,95547\_at,101584\_at |
| 4 | cell adhesion | 9 | 322 | 248 | 13100 | 0.036 | 0.025 | 1.476 | 0.158 | 104041\_at,104208\_at,160227\_s\_at,93294\_at,93318\_at,94799\_at,96752\_at,96785\_at,99577\_at |
| 5 | cell-cell adhesion | 3 | 44 | 209 | 11544 | 0.014 | 0.004 | 3.766 | 0.045 | 104041\_at,96752\_at,96785\_at |
| 6 | homophilic cell adhesion | 1 | 31 | 170 | 9498 | 0.006 | 0.003 | 1.804 | 0.429 | 96785\_at |
| 5 | cell-matrix adhesion | 1 | 50 | 209 | 11544 | 0.005 | 0.004 | 1.104 | 0.6 | 93294\_at |
| 4 | cell-cell signaling | 1 | 123 | 248 | 13100 | 0.004 | 0.009 | 0.429 | 0.906 | 100064\_f\_at |
| 4 | signal transduction | 21 | 1199 | 248 | 13100 | 0.085 | 0.092 | 0.925 | 0.679 | 160885\_at,97060\_at,99340\_at,96764\_at,99532\_at,104063\_at,93294\_at,102737\_at,95478\_at,99909\_at,99366\_at,102809\_s\_at,104427\_at,160749\_at,96592\_at,101979\_at,102779\_at,161666\_f\_at,93347\_at,95547\_at,101584\_at |
| 5 | cell surface receptor linked signal transduction | 9 | 621 | 209 | 11544 | 0.043 | 0.054 | 0.801 | 0.799 | 96764\_at,99532\_at,104063\_at,93294\_at,102737\_at,95478\_at,99909\_at,99340\_at,99366\_at |
| 6 | cytokine and chemokine mediated signaling pathway | 1 | 9 | 170 | 9498 | 0.006 | 0.001 | 6.189 | 0.15 | 96764\_at |
| 6 | enzyme linked receptor protein signaling pathway | 3 | 131 | 170 | 9498 | 0.018 | 0.014 | 1.28 | 0.417 | 99532\_at,104063\_at,93294\_at |
| 7 | transmembrane receptor protein serine/threonine kinase signaling pathway | 1 | 39 | 107 | 6246 | 0.009 | 0.006 | 1.498 | 0.491 | 99532\_at |
| 8 | TGFbeta receptor signaling pathway | 1 | 31 | 51 | 2164 | 0.02 | 0.014 | 1.368 | 0.525 | 99532\_at |
| 9 | BMP receptor signaling pathway | 1 | 5 | 22 | 911 | 0.045 | 0.005 | 8.279 | 0.115 | 99532\_at |
| 10 | regulation of BMP signaling pathway | 1 | 2 | 4 | 197 | 0.25 | 0.01 | 24.631 | 0.04 | 99532\_at |
| 11 | negative regulation of BMP signaling pathway | 1 | 2 | 1 | 34 | 1 | 0.059 | 17.001 | 0.059 | 99532\_at |
| 9 | SMAD protein nuclear translocation | 1 | 3 | 22 | 911 | 0.045 | 0.003 | 13.815 | 0.071 | 99532\_at |
| 7 | transmembrane receptor protein tyrosine kinase signaling pathway | 2 | 61 | 107 | 6246 | 0.019 | 0.01 | 1.913 | 0.281 | 104063\_at,93294\_at |
| 8 | FGF receptor signaling pathway | 1 | 2 | 51 | 2164 | 0.02 | 0.001 | 21.315 | 0.047 | 93294\_at |
| 6 | G-protein coupled receptor protein signaling pathway | 4 | 355 | 170 | 9498 | 0.024 | 0.037 | 0.629 | 0.885 | 102737\_at,95478\_at,99909\_at,99340\_at |
| 7 | G-protein signaling, coupled to IP3 second messenger (phospholipase C activating) | 2 | 13 | 107 | 6246 | 0.019 | 0.002 | 8.986 | 0.02 | 99909\_at,99340\_at |
| 8 | cytosolic calcium ion concentration elevation | 1 | 4 | 51 | 2164 | 0.02 | 0.002 | 10.6 | 0.091 | 99909\_at |
| 8 | protein kinase C activation | 1 | 5 | 51 | 2164 | 0.02 | 0.002 | 8.489 | 0.113 | 99340\_at |
| 6 | integrin-mediated signaling pathway | 1 | 45 | 170 | 9498 | 0.006 | 0.005 | 1.241 | 0.557 | 93294\_at |
| 6 | Wnt receptor signaling pathway | 1 | 30 | 170 | 9498 | 0.006 | 0.003 | 1.861 | 0.419 | 99366\_at |
| 7 | frizzled-2 signaling pathway | 1 | 16 | 107 | 6246 | 0.009 | 0.003 | 3.652 | 0.242 | 99366\_at |
| 5 | intracellular signaling cascade | 12 | 485 | 209 | 11544 | 0.057 | 0.042 | 1.367 | 0.17 | 102809\_s\_at,104427\_at,160749\_at,96592\_at,99340\_at,101979\_at,102779\_at,161666\_f\_at,93347\_at,95547\_at,97060\_at,101584\_at |
| 6 | protein kinase cascade | 3 | 50 | 170 | 9498 | 0.018 | 0.005 | 3.356 | 0.06 | 101979\_at,102779\_at,161666\_f\_at |
| 7 | MAPKKK cascade | 3 | 18 | 107 | 6246 | 0.028 | 0.003 | 9.736 | 0.003 | 101979\_at,102779\_at,161666\_f\_at |
| 8 | activation of MAPKK | 3 | 3 | 51 | 2164 | 0.059 | 0.001 | 42.317 | 0 | 101979\_at,102779\_at,161666\_f\_at |
| 6 | small GTPase mediated signal transduction | 4 | 135 | 170 | 9498 | 0.024 | 0.014 | 1.656 | 0.223 | 93347\_at,95547\_at,97060\_at,101584\_at |
| 7 | RAS protein signal transduction | 1 | 15 | 107 | 6246 | 0.009 | 0.002 | 3.896 | 0.229 | 101584\_at |
| 3 | cell death | 7 | 207 | 214 | 10726 | 0.033 | 0.019 | 1.695 | 0.121 | 101979\_at,102779\_at,161666\_f\_at,93252\_at,93536\_at,94502\_at,97890\_at |
| 4 | programmed cell death | 7 | 192 | 248 | 13100 | 0.028 | 0.015 | 1.926 | 0.073 | 101979\_at,102779\_at,161666\_f\_at,93252\_at,93536\_at,94502\_at,97890\_at |
| 5 | apoptosis | 7 | 192 | 209 | 11544 | 0.033 | 0.017 | 2.014 | 0.06 | 101979\_at,102779\_at,161666\_f\_at,93252\_at,93536\_at,94502\_at,97890\_at |
| 6 | apoptotic program | 1 | 16 | 170 | 9498 | 0.006 | 0.002 | 3.5 | 0.251 | 93536\_at |
| 7 | apoptotic mitochondrial changes | 1 | 4 | 107 | 6246 | 0.009 | 0.001 | 14.609 | 0.067 | 93536\_at |
| 7 | caspase activation | 1 | 7 | 107 | 6246 | 0.009 | 0.001 | 8.348 | 0.114 | 93536\_at |
| 8 | caspase activation via cytochrome c | 1 | 4 | 51 | 2164 | 0.02 | 0.002 | 10.6 | 0.091 | 93536\_at |
| 3 | cell differentiation | 8 | 137 | 214 | 10726 | 0.037 | 0.013 | 2.927 | 0.006 | 101979\_at,102779\_at,161666\_f\_at,92653\_at,96596\_at,97973\_at,96592\_at,99532\_at |
| 4 | cell fate commitment | 1 | 13 | 248 | 13100 | 0.004 | 0.001 | 4.071 | 0.22 | 97973\_at |
| 4 | lymphocytic blood cell differentiation | 2 | 6 | 248 | 13100 | 0.008 | 0 | 17.522 | 0.005 | 96592\_at,101979\_at |
| 5 | B-cell differentiation | 1 | 1 | 209 | 11544 | 0.005 | 0 | 53.111 | 0.018 | 96592\_at |
| 5 | T-cell differentiation | 1 | 2 | 209 | 11544 | 0.005 | 0 | 28.118 | 0.036 | 101979\_at |
| 6 | T-helper cell differentiation | 1 | 1 | 170 | 9498 | 0.006 | 0 | 53.455 | 0.018 | 101979\_at |
| 7 | T-helper 1 cell differentiation | 1 | 1 | 107 | 6246 | 0.009 | 0 | 58.438 | 0.017 | 101979\_at |
| 4 | myeloid blood cell differentiation | 1 | 7 | 248 | 13100 | 0.004 | 0.001 | 7.604 | 0.125 | 97973\_at |
| 5 | erythrocyte differentiation | 1 | 3 | 209 | 11544 | 0.005 | 0 | 18.385 | 0.053 | 97973\_at |
| 4 | osteoblast differentiation | 1 | 1 | 248 | 13100 | 0.004 | 0 | 50.375 | 0.019 | 99532\_at |
| 5 | regulation of osteoblast differentiation | 1 | 1 | 209 | 11544 | 0.005 | 0 | 53.111 | 0.018 | 99532\_at |
| 6 | negative regulation of osteoblast differentiation | 1 | 1 | 170 | 9498 | 0.006 | 0 | 53.455 | 0.018 | 99532\_at |
| 3 | cell growth and/or maintenance | 50 | 2128 | 214 | 10726 | 0.234 | 0.198 | 1.178 | 0.113 | 102809\_s\_at,92653\_at,92926\_at,94246\_at,97107\_at,97973\_at,93347\_at,101059\_at,103899\_at,92780\_f\_at,93294\_at,92262\_at,92263\_at,102737\_at,94270\_at,160462\_f\_at,94522\_at,94835\_f\_at,160343\_at,93278\_at,160081\_at,94068\_at,97409\_at,160998\_at,93020\_at,93021\_at,160127\_at,94881\_at,96728\_at,98067\_at,104735\_at,95913\_at,96801\_at,101554\_at,101593\_at,101568\_at,104063\_at,160361\_at,160885\_at,92847\_s\_at,93252\_at,96876\_at,96936\_at,98569\_at,98887\_at,101398\_at,97984\_i\_at,97515\_at,161250\_at,99909\_at |
| 4 | autophagy | 1 | 6 | 248 | 13100 | 0.004 | 0 | 8.761 | 0.108 | 93347\_at |
| 4 | cell growth | 6 | 51 | 248 | 13100 | 0.024 | 0.004 | 6.219 | 0 | 101059\_at,103899\_at,92780\_f\_at,93294\_at,92262\_at,92263\_at |
| 5 | regulation of cell growth | 6 | 38 | 209 | 11544 | 0.029 | 0.003 | 8.726 | 0 | 101059\_at,103899\_at,92780\_f\_at,93294\_at,92262\_at,92263\_at |
| 6 | negative regulation of cell growth | 2 | 2 | 170 | 9498 | 0.012 | 0 | 56 | 0 | 92262\_at,92263\_at |
| 4 | cell homeostasis | 2 | 41 | 248 | 13100 | 0.008 | 0.003 | 2.575 | 0.182 | 101059\_at,102737\_at |
| 5 | cell ion homeostasis | 2 | 37 | 209 | 11544 | 0.01 | 0.003 | 2.981 | 0.144 | 101059\_at,102737\_at |
| 6 | cation homeostasis | 2 | 36 | 170 | 9498 | 0.012 | 0.004 | 3.103 | 0.135 | 101059\_at,102737\_at |
| 7 | di-, tri-valent inorganic cation homeostasis | 1 | 29 | 107 | 6246 | 0.009 | 0.005 | 2.015 | 0.395 | 101059\_at |
| 8 | calcium ion homeostasis | 1 | 13 | 51 | 2164 | 0.02 | 0.006 | 3.263 | 0.267 | 101059\_at |
| 7 | monovalent inorganic cation homeostasis | 1 | 7 | 107 | 6246 | 0.009 | 0.001 | 8.348 | 0.114 | 102737\_at |
| 8 | hydrogen ion homeostasis | 1 | 7 | 51 | 2164 | 0.02 | 0.003 | 6.071 | 0.154 | 102737\_at |
| 9 | regulation of pH | 1 | 7 | 22 | 911 | 0.045 | 0.008 | 5.918 | 0.158 | 102737\_at |
| 4 | cell organization and biogenesis | 12 | 530 | 248 | 13100 | 0.048 | 0.04 | 1.196 | 0.304 | 94270\_at,160462\_f\_at,94522\_at,94835\_f\_at,160343\_at,93278\_at,160081\_at,94068\_at,97409\_at,160998\_at,93020\_at,93021\_at |
| 5 | cytoplasm organization and biogenesis | 9 | 380 | 209 | 11544 | 0.043 | 0.033 | 1.308 | 0.252 | 94270\_at,160462\_f\_at,94522\_at,94835\_f\_at,160343\_at,93278\_at,160081\_at,94068\_at,97409\_at |
| 6 | organelle organization and biogenesis | 6 | 318 | 170 | 9498 | 0.035 | 0.033 | 1.054 | 0.507 | 94270\_at,160462\_f\_at,94522\_at,94835\_f\_at,160343\_at,93278\_at |
| 7 | cytoskeleton organization and biogenesis | 4 | 262 | 107 | 6246 | 0.037 | 0.042 | 0.891 | 0.663 | 94270\_at,160462\_f\_at,94522\_at,94835\_f\_at |
| 8 | microtubule-based process | 3 | 119 | 51 | 2164 | 0.059 | 0.055 | 1.07 | 0.539 | 160462\_f\_at,94522\_at,94835\_f\_at |
| 9 | microtubule-based movement | 1 | 38 | 22 | 911 | 0.045 | 0.042 | 1.09 | 0.613 | 160462\_f\_at |
| 7 | ER organization and biogenesis | 1 | 6 | 107 | 6246 | 0.009 | 0.001 | 9.74 | 0.099 | 160343\_at |
| 8 | protein-ER targeting | 1 | 6 | 51 | 2164 | 0.02 | 0.003 | 7.079 | 0.133 | 160343\_at |
| 9 | cotranslational membrane targeting | 1 | 3 | 22 | 911 | 0.045 | 0.003 | 13.815 | 0.071 | 160343\_at |
| 7 | peroxisome organization and biogenesis | 1 | 17 | 107 | 6246 | 0.009 | 0.003 | 3.438 | 0.255 | 93278\_at |
| 6 | ribosome biogenesis and assembly | 3 | 60 | 170 | 9498 | 0.018 | 0.006 | 2.793 | 0.092 | 160081\_at,94068\_at,97409\_at |
| 7 | ribosome biogenesis | 3 | 60 | 107 | 6246 | 0.028 | 0.01 | 2.918 | 0.083 | 160081\_at,94068\_at,97409\_at |
| 5 | nuclear organization and biogenesis | 3 | 112 | 209 | 11544 | 0.014 | 0.01 | 1.479 | 0.331 | 160998\_at,93020\_at,93021\_at |
| 6 | chromosome organization and biogenesis (sensu Eukarya) | 3 | 108 | 170 | 9498 | 0.018 | 0.011 | 1.552 | 0.305 | 160998\_at,93020\_at,93021\_at |
| 7 | establishment and/or maintenance of chromatin architecture | 3 | 80 | 107 | 6246 | 0.028 | 0.013 | 2.189 | 0.157 | 160998\_at,93020\_at,93021\_at |
| 8 | chromatin assembly/disassembly | 3 | 48 | 51 | 2164 | 0.059 | 0.022 | 2.652 | 0.102 | 160998\_at,93020\_at,93021\_at |
| 9 | nucleosome assembly | 3 | 28 | 22 | 911 | 0.136 | 0.031 | 4.436 | 0.027 | 160998\_at,93020\_at,93021\_at |
| 4 | cell proliferation | 12 | 501 | 248 | 13100 | 0.048 | 0.038 | 1.265 | 0.242 | 160127\_at,94881\_at,96728\_at,98067\_at,104735\_at,95913\_at,92926\_at,94246\_at,97107\_at,96801\_at,101554\_at,101593\_at |
| 5 | cell cycle | 10 | 435 | 209 | 11544 | 0.048 | 0.038 | 1.27 | 0.264 | 160127\_at,94881\_at,96728\_at,98067\_at,104735\_at,95913\_at,92926\_at,94246\_at,97107\_at,96801\_at |
| 6 | M phase | 2 | 74 | 170 | 9498 | 0.012 | 0.008 | 1.51 | 0.383 | 104735\_at,160127\_at |
| 7 | M phase of mitotic cell cycle | 2 | 57 | 107 | 6246 | 0.019 | 0.009 | 2.047 | 0.255 | 104735\_at,160127\_at |
| 8 | mitosis | 2 | 57 | 51 | 2164 | 0.039 | 0.026 | 1.489 | 0.391 | 104735\_at,160127\_at |
| 6 | mitotic cell cycle | 3 | 173 | 170 | 9498 | 0.018 | 0.018 | 0.969 | 0.602 | 95913\_at,104735\_at,160127\_at |
| 7 | M phase of mitotic cell cycle | 2 | 57 | 107 | 6246 | 0.019 | 0.009 | 2.047 | 0.255 | 104735\_at,160127\_at |
| 8 | mitosis | 2 | 57 | 51 | 2164 | 0.039 | 0.026 | 1.489 | 0.391 | 104735\_at,160127\_at |
| 6 | regulation of cell cycle | 7 | 204 | 170 | 9498 | 0.041 | 0.021 | 1.917 | 0.074 | 160127\_at,92926\_at,94246\_at,97107\_at,94881\_at,96801\_at,98067\_at |
| 7 | cell cycle arrest | 3 | 20 | 107 | 6246 | 0.028 | 0.003 | 8.762 | 0.005 | 94881\_at,96801\_at,98067\_at |
| 5 | cytokinesis | 1 | 5 | 209 | 11544 | 0.005 | 0 | 11.116 | 0.087 | 104735\_at |
| 5 | regulation of cell proliferation | 2 | 38 | 209 | 11544 | 0.01 | 0.003 | 2.909 | 0.15 | 101554\_at,101593\_at |
| 6 | positive regulation of cell proliferation | 1 | 10 | 170 | 9498 | 0.006 | 0.001 | 5.6 | 0.165 | 101593\_at |
| 4 | transport | 20 | 1083 | 248 | 13100 | 0.081 | 0.083 | 0.976 | 0.581 | 101568\_at,104063\_at,160361\_at,160885\_at,92847\_s\_at,93252\_at,93278\_at,96876\_at,96936\_at,98569\_at,98887\_at,102737\_at,101398\_at,93347\_at,97984\_i\_at,160343\_at,97515\_at,161250\_at,101554\_at,99909\_at |
| 5 | carbohydrate transport | 1 | 28 | 209 | 11544 | 0.005 | 0.002 | 1.967 | 0.401 | 102737\_at |
| 6 | monosaccharide transport | 1 | 13 | 170 | 9498 | 0.006 | 0.001 | 4.292 | 0.209 | 102737\_at |
| 7 | hexose transport | 1 | 13 | 107 | 6246 | 0.009 | 0.002 | 4.495 | 0.201 | 102737\_at |
| 8 | glucose transport | 1 | 13 | 51 | 2164 | 0.02 | 0.006 | 3.263 | 0.267 | 102737\_at |
| 5 | intracellular transport | 12 | 351 | 209 | 11544 | 0.057 | 0.03 | 1.888 | 0.026 | 104063\_at,101398\_at,92847\_s\_at,93252\_at,93347\_at,96936\_at,97984\_i\_at,98887\_at,160343\_at,97515\_at,161250\_at,101554\_at |
| 6 | Golgi vesicle transport | 1 | 12 | 170 | 9498 | 0.006 | 0.001 | 4.667 | 0.195 | 104063\_at |
| 7 | intra-Golgi transport | 1 | 4 | 107 | 6246 | 0.009 | 0.001 | 14.609 | 0.067 | 104063\_at |
| 6 | intracellular protein transport | 12 | 284 | 170 | 9498 | 0.071 | 0.03 | 2.361 | 0.005 | 101398\_at,104063\_at,92847\_s\_at,93252\_at,93347\_at,96936\_at,97984\_i\_at,98887\_at,160343\_at,97515\_at,161250\_at,101554\_at |
| 7 | protein targeting | 4 | 101 | 107 | 6246 | 0.037 | 0.016 | 2.312 | 0.094 | 160343\_at,97515\_at,161250\_at,101554\_at |
| 8 | protein-ER targeting | 1 | 6 | 51 | 2164 | 0.02 | 0.003 | 7.079 | 0.133 | 160343\_at |
| 9 | cotranslational membrane targeting | 1 | 3 | 22 | 911 | 0.045 | 0.003 | 13.815 | 0.071 | 160343\_at |
| 8 | protein-nucleus import | 2 | 32 | 51 | 2164 | 0.039 | 0.015 | 2.652 | 0.173 | 161250\_at,101554\_at |
| 9 | protein-nucleus import, docking | 1 | 10 | 22 | 911 | 0.045 | 0.011 | 4.139 | 0.218 | 161250\_at |
| 9 | protein-nucleus import, translocation | 1 | 4 | 22 | 911 | 0.045 | 0.004 | 10.353 | 0.093 | 101554\_at |
| 9 | ribosomal protein-nucleus import | 1 | 3 | 22 | 911 | 0.045 | 0.003 | 13.815 | 0.071 | 161250\_at |
| 5 | ion transport | 2 | 335 | 209 | 11544 | 0.01 | 0.029 | 0.33 | 0.985 | 160885\_at,99909\_at |
| 6 | cation transport | 1 | 236 | 170 | 9498 | 0.006 | 0.025 | 0.237 | 0.987 | 99909\_at |
| 7 | di-, tri-valent inorganic cation transport | 1 | 58 | 107 | 6246 | 0.009 | 0.009 | 1.006 | 0.635 | 99909\_at |
| 8 | calcium ion transport | 1 | 33 | 51 | 2164 | 0.02 | 0.015 | 1.286 | 0.548 | 99909\_at |
| 5 | protein transport | 12 | 297 | 209 | 11544 | 0.057 | 0.026 | 2.232 | 0.008 | 101398\_at,104063\_at,93252\_at,93347\_at,96936\_at,97984\_i\_at,98887\_at,92847\_s\_at,160343\_at,97515\_at,161250\_at,101554\_at |
| 6 | intracellular protein transport | 12 | 284 | 170 | 9498 | 0.071 | 0.03 | 2.361 | 0.005 | 101398\_at,104063\_at,92847\_s\_at,93252\_at,93347\_at,96936\_at,97984\_i\_at,98887\_at,160343\_at,97515\_at,161250\_at,101554\_at |
| 7 | protein targeting | 4 | 101 | 107 | 6246 | 0.037 | 0.016 | 2.312 | 0.094 | 160343\_at,97515\_at,161250\_at,101554\_at |
| 8 | protein-ER targeting | 1 | 6 | 51 | 2164 | 0.02 | 0.003 | 7.079 | 0.133 | 160343\_at |
| 9 | cotranslational membrane targeting | 1 | 3 | 22 | 911 | 0.045 | 0.003 | 13.815 | 0.071 | 160343\_at |
| 8 | protein-nucleus import | 2 | 32 | 51 | 2164 | 0.039 | 0.015 | 2.652 | 0.173 | 161250\_at,101554\_at |
| 9 | protein-nucleus import, docking | 1 | 10 | 22 | 911 | 0.045 | 0.011 | 4.139 | 0.218 | 161250\_at |
| 9 | protein-nucleus import, translocation | 1 | 4 | 22 | 911 | 0.045 | 0.004 | 10.353 | 0.093 | 101554\_at |
| 9 | ribosomal protein-nucleus import | 1 | 3 | 22 | 911 | 0.045 | 0.003 | 13.815 | 0.071 | 161250\_at |
| 3 | cell motility | 2 | 188 | 214 | 10726 | 0.009 | 0.018 | 0.533 | 0.893 | 93294\_at,100880\_at |
| 4 | cell migration | 1 | 53 | 248 | 13100 | 0.004 | 0.004 | 0.995 | 0.638 | 93294\_at |
| 4 | muscle contraction | 1 | 54 | 248 | 13100 | 0.004 | 0.004 | 0.978 | 0.644 | 100880\_at |
| 2 | development | 17 | 990 | 218 | 10540 | 0.078 | 0.094 | 0.83 | 0.824 | 100508\_at,104463\_at,99366\_at,101979\_at,102779\_at,161666\_f\_at,92653\_at,96596\_at,97973\_at,96592\_at,99532\_at,95913\_at,92780\_f\_at,93294\_at,101593\_at,100564\_at,99577\_at |
| 3 | cell differentiation | 8 | 137 | 214 | 10726 | 0.037 | 0.013 | 2.927 | 0.006 | 101979\_at,102779\_at,161666\_f\_at,92653\_at,96596\_at,97973\_at,96592\_at,99532\_at |
| 4 | cell fate commitment | 1 | 13 | 248 | 13100 | 0.004 | 0.001 | 4.071 | 0.22 | 97973\_at |
| 4 | lymphocytic blood cell differentiation | 2 | 6 | 248 | 13100 | 0.008 | 0 | 17.522 | 0.005 | 96592\_at,101979\_at |
| 5 | B-cell differentiation | 1 | 1 | 209 | 11544 | 0.005 | 0 | 53.111 | 0.018 | 96592\_at |
| 5 | T-cell differentiation | 1 | 2 | 209 | 11544 | 0.005 | 0 | 28.118 | 0.036 | 101979\_at |
| 6 | T-helper cell differentiation | 1 | 1 | 170 | 9498 | 0.006 | 0 | 53.455 | 0.018 | 101979\_at |
| 7 | T-helper 1 cell differentiation | 1 | 1 | 107 | 6246 | 0.009 | 0 | 58.438 | 0.017 | 101979\_at |
| 4 | myeloid blood cell differentiation | 1 | 7 | 248 | 13100 | 0.004 | 0.001 | 7.604 | 0.125 | 97973\_at |
| 5 | erythrocyte differentiation | 1 | 3 | 209 | 11544 | 0.005 | 0 | 18.385 | 0.053 | 97973\_at |
| 4 | osteoblast differentiation | 1 | 1 | 248 | 13100 | 0.004 | 0 | 50.375 | 0.019 | 99532\_at |
| 5 | regulation of osteoblast differentiation | 1 | 1 | 209 | 11544 | 0.005 | 0 | 53.111 | 0.018 | 99532\_at |
| 6 | negative regulation of osteoblast differentiation | 1 | 1 | 170 | 9498 | 0.006 | 0 | 53.455 | 0.018 | 99532\_at |
| 3 | embryonic development | 2 | 52 | 214 | 10726 | 0.009 | 0.005 | 1.928 | 0.278 | 95913\_at,92780\_f\_at |
| 4 | embryonic development (sensu Animalia) | 1 | 18 | 248 | 13100 | 0.004 | 0.001 | 2.942 | 0.291 | 95913\_at |
| 5 | gastrulation | 1 | 12 | 209 | 11544 | 0.005 | 0.001 | 4.596 | 0.197 | 95913\_at |
| 6 | gastrulation (sensu Deuterostoma) | 1 | 3 | 170 | 9498 | 0.006 | 0 | 18.375 | 0.053 | 95913\_at |
| 7 | gastrulation (sensu Mammalia) | 1 | 3 | 107 | 6246 | 0.009 | 0 | 19.479 | 0.051 | 95913\_at |
| 4 | embryonic pattern specification | 1 | 7 | 248 | 13100 | 0.004 | 0.001 | 7.604 | 0.125 | 92780\_f\_at |
| 5 | patterning of blood vessels | 1 | 3 | 209 | 11544 | 0.005 | 0 | 18.385 | 0.053 | 92780\_f\_at |
| 3 | morphogenesis | 8 | 594 | 214 | 10726 | 0.037 | 0.055 | 0.675 | 0.912 | 93294\_at,92780\_f\_at,101593\_at,97973\_at,96592\_at,101979\_at,92653\_at,99532\_at |
| 4 | organogenesis | 8 | 544 | 248 | 13100 | 0.032 | 0.042 | 0.777 | 0.814 | 93294\_at,92780\_f\_at,101593\_at,97973\_at,96592\_at,101979\_at,92653\_at,99532\_at |
| 5 | blood vessel development | 2 | 61 | 209 | 11544 | 0.01 | 0.005 | 1.812 | 0.303 | 93294\_at,92780\_f\_at |
| 6 | angiogenesis | 2 | 50 | 170 | 9498 | 0.012 | 0.005 | 2.236 | 0.225 | 93294\_at,92780\_f\_at |
| 5 | patterning of blood vessels | 1 | 3 | 209 | 11544 | 0.005 | 0 | 18.385 | 0.053 | 92780\_f\_at |
| 5 | hemopoiesis | 4 | 22 | 209 | 11544 | 0.019 | 0.002 | 10.021 | 0.001 | 101593\_at,97973\_at,96592\_at,101979\_at |
| 6 | T-helper cell differentiation | 1 | 1 | 170 | 9498 | 0.006 | 0 | 53.455 | 0.018 | 101979\_at |
| 7 | T-helper 1 cell differentiation | 1 | 1 | 107 | 6246 | 0.009 | 0 | 58.438 | 0.017 | 101979\_at |
| 5 | B-cell differentiation | 1 | 1 | 209 | 11544 | 0.005 | 0 | 53.111 | 0.018 | 96592\_at |
| 5 | T-cell differentiation | 1 | 2 | 209 | 11544 | 0.005 | 0 | 28.118 | 0.036 | 101979\_at |
| 6 | T-helper cell differentiation | 1 | 1 | 170 | 9498 | 0.006 | 0 | 53.455 | 0.018 | 101979\_at |
| 7 | T-helper 1 cell differentiation | 1 | 1 | 107 | 6246 | 0.009 | 0 | 58.438 | 0.017 | 101979\_at |
| 5 | erythrocyte differentiation | 1 | 3 | 209 | 11544 | 0.005 | 0 | 18.385 | 0.053 | 97973\_at |
| 5 | neurogenesis | 1 | 164 | 209 | 11544 | 0.005 | 0.014 | 0.336 | 0.951 | 92653\_at |
| 5 | skeletal development | 2 | 52 | 209 | 11544 | 0.01 | 0.004 | 2.127 | 0.242 | 93294\_at,99532\_at |
| 6 | ossification | 2 | 25 | 170 | 9498 | 0.012 | 0.003 | 4.471 | 0.073 | 93294\_at,99532\_at |
| 6 | negative regulation of osteoblast differentiation | 1 | 1 | 170 | 9498 | 0.006 | 0 | 53.455 | 0.018 | 99532\_at |
| 5 | regulation of osteoblast differentiation | 1 | 1 | 209 | 11544 | 0.005 | 0 | 53.111 | 0.018 | 99532\_at |
| 6 | negative regulation of osteoblast differentiation | 1 | 1 | 170 | 9498 | 0.006 | 0 | 53.455 | 0.018 | 99532\_at |
| 4 | lymphocytic blood cell differentiation | 2 | 6 | 248 | 13100 | 0.008 | 0 | 17.522 | 0.005 | 96592\_at,101979\_at |
| 5 | B-cell differentiation | 1 | 1 | 209 | 11544 | 0.005 | 0 | 53.111 | 0.018 | 96592\_at |
| 5 | T-cell differentiation | 1 | 2 | 209 | 11544 | 0.005 | 0 | 28.118 | 0.036 | 101979\_at |
| 6 | T-helper cell differentiation | 1 | 1 | 170 | 9498 | 0.006 | 0 | 53.455 | 0.018 | 101979\_at |
| 7 | T-helper 1 cell differentiation | 1 | 1 | 107 | 6246 | 0.009 | 0 | 58.438 | 0.017 | 101979\_at |
| 4 | myeloid blood cell differentiation | 1 | 7 | 248 | 13100 | 0.004 | 0.001 | 7.604 | 0.125 | 97973\_at |
| 5 | erythrocyte differentiation | 1 | 3 | 209 | 11544 | 0.005 | 0 | 18.385 | 0.053 | 97973\_at |
| 4 | osteoblast differentiation | 1 | 1 | 248 | 13100 | 0.004 | 0 | 50.375 | 0.019 | 99532\_at |
| 5 | regulation of osteoblast differentiation | 1 | 1 | 209 | 11544 | 0.005 | 0 | 53.111 | 0.018 | 99532\_at |
| 6 | negative regulation of osteoblast differentiation | 1 | 1 | 170 | 9498 | 0.006 | 0 | 53.455 | 0.018 | 99532\_at |
| 3 | pattern specification | 2 | 83 | 214 | 10726 | 0.009 | 0.008 | 1.208 | 0.496 | 92780\_f\_at,95913\_at |
| 4 | embryonic pattern specification | 1 | 7 | 248 | 13100 | 0.004 | 0.001 | 7.604 | 0.125 | 92780\_f\_at |
| 5 | patterning of blood vessels | 1 | 3 | 209 | 11544 | 0.005 | 0 | 18.385 | 0.053 | 92780\_f\_at |
| 4 | anterior/posterior pattern formation | 1 | 10 | 248 | 13100 | 0.004 | 0.001 | 5.303 | 0.174 | 95913\_at |
| 5 | anterior/posterior axis specification | 1 | 3 | 209 | 11544 | 0.005 | 0 | 18.385 | 0.053 | 95913\_at |
| 3 | pigmentation | 1 | 23 | 214 | 10726 | 0.005 | 0.002 | 2.182 | 0.371 | 100564\_at |
| 4 | pigment metabolism | 1 | 23 | 248 | 13100 | 0.004 | 0.002 | 2.29 | 0.356 | 100564\_at |
| 5 | melanin metabolism | 1 | 8 | 209 | 11544 | 0.005 | 0.001 | 6.928 | 0.136 | 100564\_at |
| 6 | melanin biosynthesis | 1 | 8 | 170 | 9498 | 0.006 | 0.001 | 7 | 0.135 | 100564\_at |
| 7 | melanin biosynthesis from tyrosine | 1 | 8 | 107 | 6246 | 0.009 | 0.001 | 7.305 | 0.129 | 100564\_at |
| 3 | reproduction | 1 | 99 | 214 | 10726 | 0.005 | 0.009 | 0.506 | 0.865 | 99577\_at |
| 4 | sexual reproduction | 1 | 99 | 248 | 13100 | 0.004 | 0.008 | 0.533 | 0.85 | 99577\_at |
| 5 | gametogenesis | 1 | 86 | 209 | 11544 | 0.005 | 0.007 | 0.642 | 0.793 | 99577\_at |
| 6 | germ-cell development | 1 | 9 | 170 | 9498 | 0.006 | 0.001 | 6.189 | 0.15 | 99577\_at |
| 2 | physiological processes | 123 | 5866 | 218 | 10540 | 0.564 | 0.557 | 1.014 | 0.437 | 102809\_s\_at,92653\_at,92926\_at,94246\_at,97107\_at,97973\_at,93347\_at,101059\_at,103899\_at,92780\_f\_at,93294\_at,92262\_at,92263\_at,102737\_at,94270\_at,160462\_f\_at,94522\_at,94835\_f\_at,160343\_at,93278\_at,160081\_at,94068\_at,97409\_at,160998\_at,93020\_at,93021\_at,160127\_at,94881\_at,96728\_at,98067\_at,104735\_at,95913\_at,96801\_at,101554\_at,101593\_at,101568\_at,104063\_at,160361\_at,160885\_at,92847\_s\_at,93252\_at,96876\_at,96936\_at,98569\_at,98887\_at,101398\_at,97984\_i\_at,97515\_at,161250\_at,99909\_at,100064\_f\_at,94799\_at,95478\_at,160428\_at,96058\_s\_at,100564\_at,101990\_at,160090\_f\_at,103326\_at,94502\_at,100880\_at,92401\_at,102936\_at,100535\_at,101979\_at,102779\_at,160366\_at,161666\_f\_at,95715\_at,95737\_at,96785\_at,95496\_at,100578\_at,100611\_at,99133\_at,101963\_at,96890\_at,101583\_at,93751\_at,101060\_at,103353\_f\_at,103574\_at,162044\_f\_at,94715\_at,96831\_at,103483\_at,94073\_at,160722\_at,160449\_at,102401\_at,103634\_at,104002\_at,104463\_at,160502\_at,160724\_at,160749\_at,160783\_at,92440\_at,93782\_at,93789\_s\_at,94821\_at,94823\_at,96703\_at,160898\_at,100629\_at,93543\_f\_at,102332\_at,94941\_at,97890\_at,101510\_at,99340\_at,103717\_at,94274\_at,100998\_at,104041\_at,92866\_at,94000\_at,94285\_at,96752\_at,97540\_f\_at,98472\_at,104597\_at,92198\_s\_at |
| 3 | cell growth and/or maintenance | 50 | 2128 | 214 | 10726 | 0.234 | 0.198 | 1.178 | 0.113 | 102809\_s\_at,92653\_at,92926\_at,94246\_at,97107\_at,97973\_at,93347\_at,101059\_at,103899\_at,92780\_f\_at,93294\_at,92262\_at,92263\_at,102737\_at,94270\_at,160462\_f\_at,94522\_at,94835\_f\_at,160343\_at,93278\_at,160081\_at,94068\_at,97409\_at,160998\_at,93020\_at,93021\_at,160127\_at,94881\_at,96728\_at,98067\_at,104735\_at,95913\_at,96801\_at,101554\_at,101593\_at,101568\_at,104063\_at,160361\_at,160885\_at,92847\_s\_at,93252\_at,96876\_at,96936\_at,98569\_at,98887\_at,101398\_at,97984\_i\_at,97515\_at,161250\_at,99909\_at |
| 4 | autophagy | 1 | 6 | 248 | 13100 | 0.004 | 0 | 8.761 | 0.108 | 93347\_at |
| 4 | cell growth | 6 | 51 | 248 | 13100 | 0.024 | 0.004 | 6.219 | 0 | 101059\_at,103899\_at,92780\_f\_at,93294\_at,92262\_at,92263\_at |
| 5 | regulation of cell growth | 6 | 38 | 209 | 11544 | 0.029 | 0.003 | 8.726 | 0 | 101059\_at,103899\_at,92780\_f\_at,93294\_at,92262\_at,92263\_at |
| 6 | negative regulation of cell growth | 2 | 2 | 170 | 9498 | 0.012 | 0 | 56 | 0 | 92262\_at,92263\_at |
| 4 | cell homeostasis | 2 | 41 | 248 | 13100 | 0.008 | 0.003 | 2.575 | 0.182 | 101059\_at,102737\_at |
| 5 | cell ion homeostasis | 2 | 37 | 209 | 11544 | 0.01 | 0.003 | 2.981 | 0.144 | 101059\_at,102737\_at |
| 6 | cation homeostasis | 2 | 36 | 170 | 9498 | 0.012 | 0.004 | 3.103 | 0.135 | 101059\_at,102737\_at |
| 7 | di-, tri-valent inorganic cation homeostasis | 1 | 29 | 107 | 6246 | 0.009 | 0.005 | 2.015 | 0.395 | 101059\_at |
| 8 | calcium ion homeostasis | 1 | 13 | 51 | 2164 | 0.02 | 0.006 | 3.263 | 0.267 | 101059\_at |
| 7 | monovalent inorganic cation homeostasis | 1 | 7 | 107 | 6246 | 0.009 | 0.001 | 8.348 | 0.114 | 102737\_at |
| 8 | hydrogen ion homeostasis | 1 | 7 | 51 | 2164 | 0.02 | 0.003 | 6.071 | 0.154 | 102737\_at |
| 9 | regulation of pH | 1 | 7 | 22 | 911 | 0.045 | 0.008 | 5.918 | 0.158 | 102737\_at |
| 4 | cell organization and biogenesis | 12 | 530 | 248 | 13100 | 0.048 | 0.04 | 1.196 | 0.304 | 94270\_at,160462\_f\_at,94522\_at,94835\_f\_at,160343\_at,93278\_at,160081\_at,94068\_at,97409\_at,160998\_at,93020\_at,93021\_at |
| 5 | cytoplasm organization and biogenesis | 9 | 380 | 209 | 11544 | 0.043 | 0.033 | 1.308 | 0.252 | 94270\_at,160462\_f\_at,94522\_at,94835\_f\_at,160343\_at,93278\_at,160081\_at,94068\_at,97409\_at |
| 6 | organelle organization and biogenesis | 6 | 318 | 170 | 9498 | 0.035 | 0.033 | 1.054 | 0.507 | 94270\_at,160462\_f\_at,94522\_at,94835\_f\_at,160343\_at,93278\_at |
| 7 | cytoskeleton organization and biogenesis | 4 | 262 | 107 | 6246 | 0.037 | 0.042 | 0.891 | 0.663 | 94270\_at,160462\_f\_at,94522\_at,94835\_f\_at |
| 8 | microtubule-based process | 3 | 119 | 51 | 2164 | 0.059 | 0.055 | 1.07 | 0.539 | 160462\_f\_at,94522\_at,94835\_f\_at |
| 9 | microtubule-based movement | 1 | 38 | 22 | 911 | 0.045 | 0.042 | 1.09 | 0.613 | 160462\_f\_at |
| 7 | ER organization and biogenesis | 1 | 6 | 107 | 6246 | 0.009 | 0.001 | 9.74 | 0.099 | 160343\_at |
| 8 | protein-ER targeting | 1 | 6 | 51 | 2164 | 0.02 | 0.003 | 7.079 | 0.133 | 160343\_at |
| 9 | cotranslational membrane targeting | 1 | 3 | 22 | 911 | 0.045 | 0.003 | 13.815 | 0.071 | 160343\_at |
| 7 | peroxisome organization and biogenesis | 1 | 17 | 107 | 6246 | 0.009 | 0.003 | 3.438 | 0.255 | 93278\_at |
| 6 | ribosome biogenesis and assembly | 3 | 60 | 170 | 9498 | 0.018 | 0.006 | 2.793 | 0.092 | 160081\_at,94068\_at,97409\_at |
| 7 | ribosome biogenesis | 3 | 60 | 107 | 6246 | 0.028 | 0.01 | 2.918 | 0.083 | 160081\_at,94068\_at,97409\_at |
| 5 | nuclear organization and biogenesis | 3 | 112 | 209 | 11544 | 0.014 | 0.01 | 1.479 | 0.331 | 160998\_at,93020\_at,93021\_at |
| 6 | chromosome organization and biogenesis (sensu Eukarya) | 3 | 108 | 170 | 9498 | 0.018 | 0.011 | 1.552 | 0.305 | 160998\_at,93020\_at,93021\_at |
| 7 | establishment and/or maintenance of chromatin architecture | 3 | 80 | 107 | 6246 | 0.028 | 0.013 | 2.189 | 0.157 | 160998\_at,93020\_at,93021\_at |
| 8 | chromatin assembly/disassembly | 3 | 48 | 51 | 2164 | 0.059 | 0.022 | 2.652 | 0.102 | 160998\_at,93020\_at,93021\_at |
| 9 | nucleosome assembly | 3 | 28 | 22 | 911 | 0.136 | 0.031 | 4.436 | 0.027 | 160998\_at,93020\_at,93021\_at |
| 4 | cell proliferation | 12 | 501 | 248 | 13100 | 0.048 | 0.038 | 1.265 | 0.242 | 160127\_at,94881\_at,96728\_at,98067\_at,104735\_at,95913\_at,92926\_at,94246\_at,97107\_at,96801\_at,101554\_at,101593\_at |
| 5 | cell cycle | 10 | 435 | 209 | 11544 | 0.048 | 0.038 | 1.27 | 0.264 | 160127\_at,94881\_at,96728\_at,98067\_at,104735\_at,95913\_at,92926\_at,94246\_at,97107\_at,96801\_at |
| 6 | M phase | 2 | 74 | 170 | 9498 | 0.012 | 0.008 | 1.51 | 0.383 | 104735\_at,160127\_at |
| 7 | M phase of mitotic cell cycle | 2 | 57 | 107 | 6246 | 0.019 | 0.009 | 2.047 | 0.255 | 104735\_at,160127\_at |
| 8 | mitosis | 2 | 57 | 51 | 2164 | 0.039 | 0.026 | 1.489 | 0.391 | 104735\_at,160127\_at |
| 6 | mitotic cell cycle | 3 | 173 | 170 | 9498 | 0.018 | 0.018 | 0.969 | 0.602 | 95913\_at,104735\_at,160127\_at |
| 7 | M phase of mitotic cell cycle | 2 | 57 | 107 | 6246 | 0.019 | 0.009 | 2.047 | 0.255 | 104735\_at,160127\_at |
| 8 | mitosis | 2 | 57 | 51 | 2164 | 0.039 | 0.026 | 1.489 | 0.391 | 104735\_at,160127\_at |
| 6 | regulation of cell cycle | 7 | 204 | 170 | 9498 | 0.041 | 0.021 | 1.917 | 0.074 | 160127\_at,92926\_at,94246\_at,97107\_at,94881\_at,96801\_at,98067\_at |
| 7 | cell cycle arrest | 3 | 20 | 107 | 6246 | 0.028 | 0.003 | 8.762 | 0.005 | 94881\_at,96801\_at,98067\_at |
| 5 | cytokinesis | 1 | 5 | 209 | 11544 | 0.005 | 0 | 11.116 | 0.087 | 104735\_at |
| 5 | regulation of cell proliferation | 2 | 38 | 209 | 11544 | 0.01 | 0.003 | 2.909 | 0.15 | 101554\_at,101593\_at |
| 6 | positive regulation of cell proliferation | 1 | 10 | 170 | 9498 | 0.006 | 0.001 | 5.6 | 0.165 | 101593\_at |
| 4 | transport | 20 | 1083 | 248 | 13100 | 0.081 | 0.083 | 0.976 | 0.581 | 101568\_at,104063\_at,160361\_at,160885\_at,92847\_s\_at,93252\_at,93278\_at,96876\_at,96936\_at,98569\_at,98887\_at,102737\_at,101398\_at,93347\_at,97984\_i\_at,160343\_at,97515\_at,161250\_at,101554\_at,99909\_at |
| 5 | carbohydrate transport | 1 | 28 | 209 | 11544 | 0.005 | 0.002 | 1.967 | 0.401 | 102737\_at |
| 6 | monosaccharide transport | 1 | 13 | 170 | 9498 | 0.006 | 0.001 | 4.292 | 0.209 | 102737\_at |
| 7 | hexose transport | 1 | 13 | 107 | 6246 | 0.009 | 0.002 | 4.495 | 0.201 | 102737\_at |
| 8 | glucose transport | 1 | 13 | 51 | 2164 | 0.02 | 0.006 | 3.263 | 0.267 | 102737\_at |
| 5 | intracellular transport | 12 | 351 | 209 | 11544 | 0.057 | 0.03 | 1.888 | 0.026 | 104063\_at,101398\_at,92847\_s\_at,93252\_at,93347\_at,96936\_at,97984\_i\_at,98887\_at,160343\_at,97515\_at,161250\_at,101554\_at |
| 6 | Golgi vesicle transport | 1 | 12 | 170 | 9498 | 0.006 | 0.001 | 4.667 | 0.195 | 104063\_at |
| 7 | intra-Golgi transport | 1 | 4 | 107 | 6246 | 0.009 | 0.001 | 14.609 | 0.067 | 104063\_at |
| 6 | intracellular protein transport | 12 | 284 | 170 | 9498 | 0.071 | 0.03 | 2.361 | 0.005 | 101398\_at,104063\_at,92847\_s\_at,93252\_at,93347\_at,96936\_at,97984\_i\_at,98887\_at,160343\_at,97515\_at,161250\_at,101554\_at |
| 7 | protein targeting | 4 | 101 | 107 | 6246 | 0.037 | 0.016 | 2.312 | 0.094 | 160343\_at,97515\_at,161250\_at,101554\_at |
| 8 | protein-ER targeting | 1 | 6 | 51 | 2164 | 0.02 | 0.003 | 7.079 | 0.133 | 160343\_at |
| 9 | cotranslational membrane targeting | 1 | 3 | 22 | 911 | 0.045 | 0.003 | 13.815 | 0.071 | 160343\_at |
| 8 | protein-nucleus import | 2 | 32 | 51 | 2164 | 0.039 | 0.015 | 2.652 | 0.173 | 161250\_at,101554\_at |
| 9 | protein-nucleus import, docking | 1 | 10 | 22 | 911 | 0.045 | 0.011 | 4.139 | 0.218 | 161250\_at |
| 9 | protein-nucleus import, translocation | 1 | 4 | 22 | 911 | 0.045 | 0.004 | 10.353 | 0.093 | 101554\_at |
| 9 | ribosomal protein-nucleus import | 1 | 3 | 22 | 911 | 0.045 | 0.003 | 13.815 | 0.071 | 161250\_at |
| 5 | ion transport | 2 | 335 | 209 | 11544 | 0.01 | 0.029 | 0.33 | 0.985 | 160885\_at,99909\_at |
| 6 | cation transport | 1 | 236 | 170 | 9498 | 0.006 | 0.025 | 0.237 | 0.987 | 99909\_at |
| 7 | di-, tri-valent inorganic cation transport | 1 | 58 | 107 | 6246 | 0.009 | 0.009 | 1.006 | 0.635 | 99909\_at |
| 8 | calcium ion transport | 1 | 33 | 51 | 2164 | 0.02 | 0.015 | 1.286 | 0.548 | 99909\_at |
| 5 | protein transport | 12 | 297 | 209 | 11544 | 0.057 | 0.026 | 2.232 | 0.008 | 101398\_at,104063\_at,93252\_at,93347\_at,96936\_at,97984\_i\_at,98887\_at,92847\_s\_at,160343\_at,97515\_at,161250\_at,101554\_at |
| 6 | intracellular protein transport | 12 | 284 | 170 | 9498 | 0.071 | 0.03 | 2.361 | 0.005 | 101398\_at,104063\_at,92847\_s\_at,93252\_at,93347\_at,96936\_at,97984\_i\_at,98887\_at,160343\_at,97515\_at,161250\_at,101554\_at |
| 7 | protein targeting | 4 | 101 | 107 | 6246 | 0.037 | 0.016 | 2.312 | 0.094 | 160343\_at,97515\_at,161250\_at,101554\_at |
| 8 | protein-ER targeting | 1 | 6 | 51 | 2164 | 0.02 | 0.003 | 7.079 | 0.133 | 160343\_at |
| 9 | cotranslational membrane targeting | 1 | 3 | 22 | 911 | 0.045 | 0.003 | 13.815 | 0.071 | 160343\_at |
| 8 | protein-nucleus import | 2 | 32 | 51 | 2164 | 0.039 | 0.015 | 2.652 | 0.173 | 161250\_at,101554\_at |
| 9 | protein-nucleus import, docking | 1 | 10 | 22 | 911 | 0.045 | 0.011 | 4.139 | 0.218 | 161250\_at |
| 9 | protein-nucleus import, translocation | 1 | 4 | 22 | 911 | 0.045 | 0.004 | 10.353 | 0.093 | 101554\_at |
| 9 | ribosomal protein-nucleus import | 1 | 3 | 22 | 911 | 0.045 | 0.003 | 13.815 | 0.071 | 161250\_at |
| 3 | circulation | 2 | 40 | 214 | 10726 | 0.009 | 0.004 | 2.507 | 0.19 | 102737\_at,100064\_f\_at |
| 4 | regulation of blood pressure | 1 | 18 | 248 | 13100 | 0.004 | 0.001 | 2.942 | 0.291 | 102737\_at |
| 4 | regulation of heart rate | 1 | 19 | 248 | 13100 | 0.004 | 0.001 | 2.779 | 0.305 | 100064\_f\_at |
| 3 | hemostasis | 2 | 44 | 214 | 10726 | 0.009 | 0.004 | 2.28 | 0.219 | 94799\_at,95478\_at |
| 4 | blood coagulation | 2 | 42 | 248 | 13100 | 0.008 | 0.003 | 2.511 | 0.189 | 94799\_at,95478\_at |
| 3 | metabolism | 78 | 3908 | 214 | 10726 | 0.364 | 0.364 | 1 | 0.524 | 160428\_at,96058\_s\_at,97515\_at,100564\_at,101990\_at,160090\_f\_at,103326\_at,94502\_at,100880\_at,92401\_at,102936\_at,100535\_at,101979\_at,102779\_at,160081\_at,160366\_at,161666\_f\_at,94068\_at,95715\_at,95737\_at,96785\_at,95496\_at,100578\_at,100611\_at,99133\_at,101963\_at,96890\_at,101583\_at,93751\_at,93278\_at,96801\_at,101060\_at,103353\_f\_at,103574\_at,162044\_f\_at,92653\_at,94715\_at,96831\_at,93294\_at,103483\_at,94073\_at,160722\_at,160449\_at,101059\_at,102401\_at,103634\_at,104002\_at,104463\_at,160502\_at,160724\_at,160749\_at,160783\_at,92440\_at,92926\_at,93782\_at,93789\_s\_at,94246\_at,94821\_at,94823\_at,97973\_at,99909\_at,96703\_at,160898\_at,100629\_at,93543\_f\_at,102332\_at,102809\_s\_at,94941\_at,97107\_at,97409\_at,97890\_at,160343\_at,161250\_at,101554\_at,101510\_at,99340\_at,103717\_at,94274\_at |
| 4 | pigment metabolism | 1 | 23 | 248 | 13100 | 0.004 | 0.002 | 2.29 | 0.356 | 100564\_at |
| 5 | melanin metabolism | 1 | 8 | 209 | 11544 | 0.005 | 0.001 | 6.928 | 0.136 | 100564\_at |
| 6 | melanin biosynthesis | 1 | 8 | 170 | 9498 | 0.006 | 0.001 | 7 | 0.135 | 100564\_at |
| 7 | melanin biosynthesis from tyrosine | 1 | 8 | 107 | 6246 | 0.009 | 0.001 | 7.305 | 0.129 | 100564\_at |
| 4 | alcohol metabolism | 5 | 167 | 248 | 13100 | 0.02 | 0.013 | 1.581 | 0.21 | 101990\_at,160090\_f\_at,160428\_at,103326\_at,94502\_at |
| 5 | alcohol catabolism | 4 | 58 | 209 | 11544 | 0.019 | 0.005 | 3.813 | 0.021 | 101990\_at,160090\_f\_at,160428\_at,103326\_at |
| 6 | monosaccharide catabolism | 4 | 58 | 170 | 9498 | 0.024 | 0.006 | 3.851 | 0.02 | 101990\_at,160090\_f\_at,160428\_at,103326\_at |
| 7 | hexose catabolism | 4 | 58 | 107 | 6246 | 0.037 | 0.009 | 4.024 | 0.017 | 101990\_at,160090\_f\_at,160428\_at,103326\_at |
| 8 | glucose catabolism | 4 | 58 | 51 | 2164 | 0.078 | 0.027 | 2.926 | 0.046 | 101990\_at,160090\_f\_at,160428\_at,103326\_at |
| 9 | glycolysis | 3 | 52 | 22 | 911 | 0.136 | 0.057 | 2.389 | 0.126 | 101990\_at,160090\_f\_at,160428\_at |
| 9 | pentose-phosphate shunt | 1 | 7 | 22 | 911 | 0.045 | 0.008 | 5.918 | 0.158 | 103326\_at |
| 10 | pentose-phosphate shunt, non-oxidative branch | 1 | 1 | 4 | 197 | 0.25 | 0.005 | 49.213 | 0.02 | 103326\_at |
| 5 | sphingosine metabolism | 1 | 3 | 209 | 11544 | 0.005 | 0 | 18.385 | 0.053 | 94502\_at |
| 4 | amine metabolism | 2 | 148 | 248 | 13100 | 0.008 | 0.011 | 0.713 | 0.774 | 94502\_at,100880\_at |
| 5 | sphingosine metabolism | 1 | 3 | 209 | 11544 | 0.005 | 0 | 18.385 | 0.053 | 94502\_at |
| 5 | amino acid metabolism | 1 | 97 | 209 | 11544 | 0.005 | 0.008 | 0.569 | 0.831 | 100880\_at |
| 4 | biosynthesis | 15 | 652 | 248 | 13100 | 0.06 | 0.05 | 1.215 | 0.254 | 92401\_at,102936\_at,97515\_at,100535\_at,101979\_at,102779\_at,160081\_at,160366\_at,161666\_f\_at,94068\_at,95715\_at,95737\_at,96785\_at,95496\_at,100578\_at |
| 5 | lipid biosynthesis | 3 | 124 | 209 | 11544 | 0.014 | 0.011 | 1.336 | 0.39 | 92401\_at,102936\_at,97515\_at |
| 6 | fatty acid biosynthesis | 1 | 40 | 170 | 9498 | 0.006 | 0.004 | 1.397 | 0.515 | 92401\_at |
| 7 | eicosanoid biosynthesis | 1 | 19 | 107 | 6246 | 0.009 | 0.003 | 3.076 | 0.28 | 92401\_at |
| 8 | leukotriene biosynthesis | 1 | 10 | 51 | 2164 | 0.02 | 0.005 | 4.245 | 0.213 | 92401\_at |
| 6 | membrane lipid biosynthesis | 1 | 20 | 170 | 9498 | 0.006 | 0.002 | 2.787 | 0.303 | 102936\_at |
| 7 | sphingolipid biosynthesis | 1 | 5 | 107 | 6246 | 0.009 | 0.001 | 11.688 | 0.083 | 102936\_at |
| 6 | steroid biosynthesis | 1 | 45 | 170 | 9498 | 0.006 | 0.005 | 1.241 | 0.557 | 97515\_at |
| 5 | macromolecule biosynthesis | 10 | 322 | 209 | 11544 | 0.048 | 0.028 | 1.716 | 0.068 | 100535\_at,101979\_at,102779\_at,160081\_at,160366\_at,161666\_f\_at,94068\_at,95715\_at,95737\_at,96785\_at |
| 6 | protein biosynthesis | 10 | 322 | 170 | 9498 | 0.059 | 0.034 | 1.735 | 0.063 | 100535\_at,101979\_at,102779\_at,160081\_at,160366\_at,161666\_f\_at,94068\_at,95715\_at,95737\_at,96785\_at |
| 7 | amino acid activation | 1 | 36 | 107 | 6246 | 0.009 | 0.006 | 1.623 | 0.464 | 96785\_at |
| 7 | cytokine biosynthesis | 1 | 4 | 107 | 6246 | 0.009 | 0.001 | 14.609 | 0.067 | 101979\_at |
| 8 | interferon-gamma biosynthesis | 1 | 1 | 51 | 2164 | 0.02 | 0 | 42.63 | 0.024 | 101979\_at |
| 7 | regulation of protein biosynthesis | 1 | 7 | 107 | 6246 | 0.009 | 0.001 | 8.348 | 0.114 | 100535\_at |
| 7 | regulation of translation | 1 | 24 | 107 | 6246 | 0.009 | 0.004 | 2.435 | 0.34 | 100535\_at |
| 8 | regulation of translational initiation | 1 | 13 | 51 | 2164 | 0.02 | 0.006 | 3.263 | 0.267 | 100535\_at |
| 7 | translational initiation | 2 | 34 | 107 | 6246 | 0.019 | 0.005 | 3.436 | 0.115 | 160366\_at,100535\_at |
| 8 | regulation of translational initiation | 1 | 13 | 51 | 2164 | 0.02 | 0.006 | 3.263 | 0.267 | 100535\_at |
| 5 | nucleotide biosynthesis | 2 | 74 | 209 | 11544 | 0.01 | 0.006 | 1.493 | 0.389 | 95496\_at,100578\_at |
| 6 | nucleoside monophosphate biosynthesis | 1 | 15 | 170 | 9498 | 0.006 | 0.002 | 3.722 | 0.237 | 100578\_at |
| 7 | purine nucleoside monophosphate biosynthesis | 1 | 10 | 107 | 6246 | 0.009 | 0.002 | 5.844 | 0.159 | 100578\_at |
| 8 | purine ribonucleoside monophosphate biosynthesis | 1 | 10 | 51 | 2164 | 0.02 | 0.005 | 4.245 | 0.213 | 100578\_at |
| 9 | GMP biosynthesis | 1 | 2 | 22 | 911 | 0.045 | 0.002 | 20.659 | 0.048 | 100578\_at |
| 6 | purine nucleotide biosynthesis | 1 | 45 | 170 | 9498 | 0.006 | 0.005 | 1.241 | 0.557 | 100578\_at |
| 7 | purine nucleoside monophosphate biosynthesis | 1 | 10 | 107 | 6246 | 0.009 | 0.002 | 5.844 | 0.159 | 100578\_at |
| 8 | purine ribonucleoside monophosphate biosynthesis | 1 | 10 | 51 | 2164 | 0.02 | 0.005 | 4.245 | 0.213 | 100578\_at |
| 9 | GMP biosynthesis | 1 | 2 | 22 | 911 | 0.045 | 0.002 | 20.659 | 0.048 | 100578\_at |
| 4 | carbohydrate metabolism | 6 | 231 | 248 | 13100 | 0.024 | 0.018 | 1.372 | 0.274 | 100611\_at,99133\_at,101990\_at,160090\_f\_at,160428\_at,103326\_at |
| 5 | main pathways of carbohydrate metabolism | 4 | 84 | 209 | 11544 | 0.019 | 0.007 | 2.629 | 0.066 | 101990\_at,160090\_f\_at,160428\_at,103326\_at |
| 6 | tricarboxylic acid cycle | 1 | 19 | 170 | 9498 | 0.006 | 0.002 | 2.94 | 0.291 | 160428\_at |
| 4 | catabolism | 9 | 631 | 248 | 13100 | 0.036 | 0.048 | 0.753 | 0.85 | 101990\_at,160090\_f\_at,160428\_at,103326\_at,100611\_at,101963\_at,96890\_at,101583\_at,93751\_at |
| 5 | alcohol catabolism | 4 | 58 | 209 | 11544 | 0.019 | 0.005 | 3.813 | 0.021 | 101990\_at,160090\_f\_at,160428\_at,103326\_at |
| 6 | monosaccharide catabolism | 4 | 58 | 170 | 9498 | 0.024 | 0.006 | 3.851 | 0.02 | 101990\_at,160090\_f\_at,160428\_at,103326\_at |
| 7 | hexose catabolism | 4 | 58 | 107 | 6246 | 0.037 | 0.009 | 4.024 | 0.017 | 101990\_at,160090\_f\_at,160428\_at,103326\_at |
| 8 | glucose catabolism | 4 | 58 | 51 | 2164 | 0.078 | 0.027 | 2.926 | 0.046 | 101990\_at,160090\_f\_at,160428\_at,103326\_at |
| 9 | glycolysis | 3 | 52 | 22 | 911 | 0.136 | 0.057 | 2.389 | 0.126 | 101990\_at,160090\_f\_at,160428\_at |
| 9 | pentose-phosphate shunt | 1 | 7 | 22 | 911 | 0.045 | 0.008 | 5.918 | 0.158 | 103326\_at |
| 10 | pentose-phosphate shunt, non-oxidative branch | 1 | 1 | 4 | 197 | 0.25 | 0.005 | 49.213 | 0.02 | 103326\_at |
| 5 | cell wall catabolism | 1 | 5 | 209 | 11544 | 0.005 | 0 | 11.116 | 0.087 | 100611\_at |
| 5 | macromolecule catabolism | 4 | 470 | 209 | 11544 | 0.019 | 0.041 | 0.47 | 0.973 | 101963\_at,96890\_at,101583\_at,93751\_at |
| 6 | protein catabolism | 4 | 466 | 170 | 9498 | 0.024 | 0.049 | 0.48 | 0.97 | 101963\_at,96890\_at,101583\_at,93751\_at |
| 7 | proteolysis and peptidolysis | 4 | 457 | 107 | 6246 | 0.037 | 0.073 | 0.511 | 0.959 | 101963\_at,96890\_at,101583\_at,93751\_at |
| 8 | ATP-dependent proteolysis | 1 | 1 | 51 | 2164 | 0.02 | 0 | 42.63 | 0.024 | 96890\_at |
| 8 | modification-dependent protein catabolism | 2 | 122 | 51 | 2164 | 0.039 | 0.056 | 0.696 | 0.794 | 101583\_at,93751\_at |
| 9 | ubiquitin-dependent protein catabolism | 2 | 120 | 22 | 911 | 0.091 | 0.132 | 0.69 | 0.81 | 101583\_at,93751\_at |
| 4 | coenzymes and prosthetic group metabolism | 2 | 95 | 248 | 13100 | 0.008 | 0.007 | 1.112 | 0.54 | 93278\_at,96801\_at |
| 5 | coenzyme metabolism | 2 | 80 | 209 | 11544 | 0.01 | 0.007 | 1.381 | 0.427 | 93278\_at,96801\_at |
| 6 | acyl-CoA metabolism | 1 | NA | 170 | 9498 | 0.006 | NA | NA | NA | 93278\_at |
| 6 | ATP metabolism | 1 | 26 | 170 | 9498 | 0.006 | 0.003 | 2.146 | 0.375 | 96801\_at |
| 4 | electron transport | 8 | 313 | 248 | 13100 | 0.032 | 0.024 | 1.35 | 0.243 | 101060\_at,103353\_f\_at,103574\_at,160366\_at,162044\_f\_at,92653\_at,94715\_at,96831\_at |
| 4 | lipid metabolism | 5 | 285 | 248 | 13100 | 0.02 | 0.022 | 0.926 | 0.631 | 92401\_at,102936\_at,97515\_at,93278\_at,94502\_at |
| 5 | lipid biosynthesis | 3 | 124 | 209 | 11544 | 0.014 | 0.011 | 1.336 | 0.39 | 92401\_at,102936\_at,97515\_at |
| 6 | fatty acid biosynthesis | 1 | 40 | 170 | 9498 | 0.006 | 0.004 | 1.397 | 0.515 | 92401\_at |
| 7 | eicosanoid biosynthesis | 1 | 19 | 107 | 6246 | 0.009 | 0.003 | 3.076 | 0.28 | 92401\_at |
| 8 | leukotriene biosynthesis | 1 | 10 | 51 | 2164 | 0.02 | 0.005 | 4.245 | 0.213 | 92401\_at |
| 6 | membrane lipid biosynthesis | 1 | 20 | 170 | 9498 | 0.006 | 0.002 | 2.787 | 0.303 | 102936\_at |
| 7 | sphingolipid biosynthesis | 1 | 5 | 107 | 6246 | 0.009 | 0.001 | 11.688 | 0.083 | 102936\_at |
| 6 | steroid biosynthesis | 1 | 45 | 170 | 9498 | 0.006 | 0.005 | 1.241 | 0.557 | 97515\_at |
| 5 | fatty acid metabolism | 2 | 85 | 209 | 11544 | 0.01 | 0.007 | 1.3 | 0.457 | 92401\_at,93278\_at |
| 6 | fatty acid biosynthesis | 1 | 40 | 170 | 9498 | 0.006 | 0.004 | 1.397 | 0.515 | 92401\_at |
| 7 | eicosanoid biosynthesis | 1 | 19 | 107 | 6246 | 0.009 | 0.003 | 3.076 | 0.28 | 92401\_at |
| 8 | leukotriene biosynthesis | 1 | 10 | 51 | 2164 | 0.02 | 0.005 | 4.245 | 0.213 | 92401\_at |
| 6 | acyl-CoA metabolism | 1 | NA | 170 | 9498 | 0.006 | NA | NA | NA | 93278\_at |
| 6 | eicosanoid metabolism | 1 | 23 | 170 | 9498 | 0.006 | 0.002 | 2.43 | 0.34 | 92401\_at |
| 7 | eicosanoid biosynthesis | 1 | 19 | 107 | 6246 | 0.009 | 0.003 | 3.076 | 0.28 | 92401\_at |
| 8 | leukotriene biosynthesis | 1 | 10 | 51 | 2164 | 0.02 | 0.005 | 4.245 | 0.213 | 92401\_at |
| 7 | leukotriene metabolism | 1 | 10 | 107 | 6246 | 0.009 | 0.002 | 5.844 | 0.159 | 92401\_at |
| 8 | leukotriene biosynthesis | 1 | 10 | 51 | 2164 | 0.02 | 0.005 | 4.245 | 0.213 | 92401\_at |
| 5 | membrane lipid metabolism | 2 | 46 | 209 | 11544 | 0.01 | 0.004 | 2.405 | 0.202 | 102936\_at,94502\_at |
| 6 | membrane lipid biosynthesis | 1 | 20 | 170 | 9498 | 0.006 | 0.002 | 2.787 | 0.303 | 102936\_at |
| 7 | sphingolipid biosynthesis | 1 | 5 | 107 | 6246 | 0.009 | 0.001 | 11.688 | 0.083 | 102936\_at |
| 6 | sphingolipid metabolism | 2 | 14 | 170 | 9498 | 0.012 | 0.001 | 8 | 0.025 | 94502\_at,102936\_at |
| 7 | sphingolipid biosynthesis | 1 | 5 | 107 | 6246 | 0.009 | 0.001 | 11.688 | 0.083 | 102936\_at |
| 7 | sphingoid metabolism | 1 | 10 | 107 | 6246 | 0.009 | 0.002 | 5.844 | 0.159 | 94502\_at |
| 8 | sphinganine metabolism | 1 | 1 | 51 | 2164 | 0.02 | 0 | 42.63 | 0.024 | 94502\_at |
| 9 | sphinganine-1-phosphate metabolism | 1 | 1 | 22 | 911 | 0.045 | 0.001 | 41.318 | 0.024 | 94502\_at |
| 5 | sphingosine metabolism | 1 | 3 | 209 | 11544 | 0.005 | 0 | 18.385 | 0.053 | 94502\_at |
| 4 | nucleobase, nucleoside, nucleotide and nucleic acid metabolism | 27 | 1530 | 248 | 13100 | 0.109 | 0.117 | 0.932 | 0.682 | 93294\_at,103483\_at,94073\_at,95496\_at,160722\_at,160449\_at,101059\_at,102401\_at,103634\_at,104002\_at,104463\_at,160502\_at,160724\_at,160749\_at,160783\_at,92440\_at,92653\_at,92926\_at,93782\_at,93789\_s\_at,94246\_at,94821\_at,94823\_at,97973\_at,99909\_at,96703\_at,160898\_at |
| 5 | DNA metabolism | 3 | 302 | 209 | 11544 | 0.014 | 0.026 | 0.549 | 0.914 | 93294\_at,103483\_at,94073\_at |
| 6 | DNA repair | 2 | 99 | 170 | 9498 | 0.012 | 0.01 | 1.129 | 0.532 | 103483\_at,94073\_at |
| 7 | nucleotide-excision repair | 2 | 20 | 107 | 6246 | 0.019 | 0.003 | 5.841 | 0.045 | 103483\_at,94073\_at |
| 8 | transcription-coupled nucleotide-excision repair | 1 | 1 | 51 | 2164 | 0.02 | 0 | 42.63 | 0.024 | 94073\_at |
| 5 | nucleoside metabolism | 1 | 15 | 209 | 11544 | 0.005 | 0.001 | 3.677 | 0.24 | 95496\_at |
| 5 | RNA metabolism | 1 | 132 | 209 | 11544 | 0.005 | 0.011 | 0.418 | 0.912 | 160722\_at |
| 6 | RNA processing | 1 | 126 | 170 | 9498 | 0.006 | 0.013 | 0.443 | 0.899 | 160722\_at |
| 5 | transcription | 23 | 1086 | 209 | 11544 | 0.11 | 0.094 | 1.17 | 0.243 | 160449\_at,94073\_at,101059\_at,102401\_at,103634\_at,104002\_at,104463\_at,160502\_at,160724\_at,160749\_at,160783\_at,92440\_at,92653\_at,92926\_at,93782\_at,93789\_s\_at,94246\_at,94821\_at,94823\_at,97973\_at,99909\_at,96703\_at,160898\_at |
| 6 | regulation of transcription | 21 | 1026 | 170 | 9498 | 0.124 | 0.108 | 1.144 | 0.289 | 160449\_at,101059\_at,102401\_at,103634\_at,104002\_at,104463\_at,160502\_at,160724\_at,160749\_at,160783\_at,92440\_at,92653\_at,92926\_at,93782\_at,93789\_s\_at,94246\_at,94821\_at,94823\_at,97973\_at,99909\_at,96703\_at |
| 7 | negative regulation of transcription | 1 | 40 | 107 | 6246 | 0.009 | 0.006 | 1.461 | 0.5 | 160449\_at |
| 8 | negative regulation of transcription, DNA-dependent | 1 | 27 | 51 | 2164 | 0.02 | 0.012 | 1.571 | 0.477 | 160449\_at |
| 9 | negative regulation of transcription from Pol II promoter | 1 | 24 | 22 | 911 | 0.045 | 0.026 | 1.726 | 0.448 | 160449\_at |
| 7 | regulation of transcription, DNA-dependent | 21 | 1013 | 107 | 6246 | 0.196 | 0.162 | 1.21 | 0.2 | 101059\_at,102401\_at,103634\_at,104002\_at,104463\_at,160502\_at,160724\_at,160749\_at,160783\_at,92440\_at,92653\_at,92926\_at,93782\_at,93789\_s\_at,94246\_at,94821\_at,94823\_at,97973\_at,99909\_at,160449\_at,96703\_at |
| 8 | negative regulation of transcription, DNA-dependent | 1 | 27 | 51 | 2164 | 0.02 | 0.012 | 1.571 | 0.477 | 160449\_at |
| 9 | negative regulation of transcription from Pol II promoter | 1 | 24 | 22 | 911 | 0.045 | 0.026 | 1.726 | 0.448 | 160449\_at |
| 8 | regulation of transcription from Pol II promoter | 2 | 72 | 51 | 2164 | 0.039 | 0.033 | 1.179 | 0.512 | 96703\_at,160449\_at |
| 9 | negative regulation of transcription from Pol II promoter | 1 | 24 | 22 | 911 | 0.045 | 0.026 | 1.726 | 0.448 | 160449\_at |
| 6 | transcription, DNA-dependent | 23 | 1046 | 170 | 9498 | 0.135 | 0.11 | 1.228 | 0.174 | 101059\_at,102401\_at,103634\_at,104002\_at,104463\_at,160502\_at,160724\_at,160749\_at,160783\_at,92440\_at,92653\_at,92926\_at,93782\_at,93789\_s\_at,94246\_at,94821\_at,94823\_at,97973\_at,99909\_at,160449\_at,96703\_at,160898\_at,94073\_at |
| 7 | regulation of transcription, DNA-dependent | 21 | 1013 | 107 | 6246 | 0.196 | 0.162 | 1.21 | 0.2 | 101059\_at,102401\_at,103634\_at,104002\_at,104463\_at,160502\_at,160724\_at,160749\_at,160783\_at,92440\_at,92653\_at,92926\_at,93782\_at,93789\_s\_at,94246\_at,94821\_at,94823\_at,97973\_at,99909\_at,160449\_at,96703\_at |
| 8 | negative regulation of transcription, DNA-dependent | 1 | 27 | 51 | 2164 | 0.02 | 0.012 | 1.571 | 0.477 | 160449\_at |
| 9 | negative regulation of transcription from Pol II promoter | 1 | 24 | 22 | 911 | 0.045 | 0.026 | 1.726 | 0.448 | 160449\_at |
| 8 | regulation of transcription from Pol II promoter | 2 | 72 | 51 | 2164 | 0.039 | 0.033 | 1.179 | 0.512 | 96703\_at,160449\_at |
| 9 | negative regulation of transcription from Pol II promoter | 1 | 24 | 22 | 911 | 0.045 | 0.026 | 1.726 | 0.448 | 160449\_at |
| 7 | transcription from Pol II promoter | 4 | 104 | 107 | 6246 | 0.037 | 0.017 | 2.245 | 0.102 | 160898\_at,94073\_at,96703\_at,160449\_at |
| 8 | regulation of transcription from Pol II promoter | 2 | 72 | 51 | 2164 | 0.039 | 0.033 | 1.179 | 0.512 | 96703\_at,160449\_at |
| 9 | negative regulation of transcription from Pol II promoter | 1 | 24 | 22 | 911 | 0.045 | 0.026 | 1.726 | 0.448 | 160449\_at |
| 4 | oxygen and reactive oxygen species metabolism | 2 | 34 | 248 | 13100 | 0.008 | 0.003 | 3.1 | 0.135 | 100629\_at,93543\_f\_at |
| 5 | glutathione conjugation reaction | 2 | 15 | 209 | 11544 | 0.01 | 0.001 | 7.362 | 0.029 | 100629\_at,93543\_f\_at |
| 4 | phosphorus metabolism | 6 | 488 | 248 | 13100 | 0.024 | 0.037 | 0.649 | 0.905 | 102332\_at,102809\_s\_at,94941\_at,97107\_at,97409\_at,97890\_at |
| 5 | phosphate metabolism | 6 | 488 | 209 | 11544 | 0.029 | 0.042 | 0.679 | 0.882 | 102332\_at,102809\_s\_at,94941\_at,97107\_at,97409\_at,97890\_at |
| 6 | phosphorylation | 6 | 395 | 170 | 9498 | 0.035 | 0.042 | 0.849 | 0.716 | 102332\_at,102809\_s\_at,94941\_at,97107\_at,97409\_at,97890\_at |
| 7 | protein amino acid phosphorylation | 6 | 379 | 107 | 6246 | 0.056 | 0.061 | 0.924 | 0.639 | 102332\_at,102809\_s\_at,94941\_at,97107\_at,97409\_at,97890\_at |
| 4 | protein metabolism | 29 | 1458 | 248 | 13100 | 0.117 | 0.111 | 1.051 | 0.418 | 160343\_at,97515\_at,161250\_at,101554\_at,100535\_at,101979\_at,102779\_at,160081\_at,160366\_at,161666\_f\_at,94068\_at,95715\_at,95737\_at,96785\_at,101963\_at,96890\_at,101583\_at,93751\_at,101510\_at,96831\_at,99340\_at,103717\_at,94274\_at,102332\_at,102809\_s\_at,94941\_at,97107\_at,97409\_at,97890\_at |
| 5 | protein folding | 2 | 56 | 209 | 11544 | 0.01 | 0.005 | 1.973 | 0.269 | 101510\_at,96831\_at |
| 5 | protein localization | 1 | 5 | 209 | 11544 | 0.005 | 0 | 11.116 | 0.087 | 99340\_at |
| 5 | protein modification | 9 | 654 | 209 | 11544 | 0.043 | 0.057 | 0.76 | 0.844 | 101583\_at,103717\_at,94274\_at,102332\_at,102809\_s\_at,94941\_at,97107\_at,97409\_at,97890\_at |
| 6 | ubiquitin cycle | 3 | 56 | 170 | 9498 | 0.018 | 0.006 | 2.992 | 0.078 | 101583\_at,103717\_at,94274\_at |
| 3 | pathogenesis | 1 | 10 | 214 | 10726 | 0.005 | 0.001 | 5.022 | 0.183 | 102737\_at |
| 3 | respiratory gaseous exchange | 2 | 10 | 214 | 10726 | 0.009 | 0.001 | 10.054 | 0.016 | 101059\_at,102737\_at |
| 3 | response to external stimulus | 15 | 666 | 214 | 10726 | 0.07 | 0.062 | 1.129 | 0.351 | 100998\_at,104041\_at,92866\_at,94000\_at,94285\_at,96752\_at,97540\_f\_at,98472\_at,102401\_at,103634\_at,104597\_at,93252\_at,94799\_at,92198\_s\_at,101568\_at |
| 4 | response to biotic stimulus | 15 | 516 | 248 | 13100 | 0.06 | 0.039 | 1.535 | 0.066 | 100998\_at,104041\_at,92866\_at,94000\_at,94285\_at,96752\_at,97540\_f\_at,98472\_at,102401\_at,103634\_at,104597\_at,93252\_at,94799\_at,92198\_s\_at,101568\_at |
| 5 | defense response | 15 | 471 | 209 | 11544 | 0.072 | 0.041 | 1.759 | 0.024 | 100998\_at,104041\_at,92866\_at,94000\_at,94285\_at,96752\_at,97540\_f\_at,98472\_at,102401\_at,103634\_at,104597\_at,93252\_at,94799\_at,92198\_s\_at,101568\_at |
| 6 | immune response | 13 | 362 | 170 | 9498 | 0.076 | 0.038 | 2.007 | 0.013 | 100998\_at,102401\_at,103634\_at,104597\_at,92866\_at,93252\_at,94000\_at,94285\_at,97540\_f\_at,98472\_at,94799\_at,92198\_s\_at,101568\_at |
| 7 | acute-phase response | 1 | 23 | 107 | 6246 | 0.009 | 0.004 | 2.541 | 0.328 | 94799\_at |
| 7 | antigen presentation | 5 | 26 | 107 | 6246 | 0.047 | 0.004 | 11.233 | 0 | 97540\_f\_at,98472\_at,100998\_at,92866\_at,94285\_at |
| 8 | antigen presentation, endogenous antigen | 2 | 15 | 51 | 2164 | 0.039 | 0.007 | 5.659 | 0.047 | 97540\_f\_at,98472\_at |
| 8 | antigen presentation, exogenous antigen | 3 | 11 | 51 | 2164 | 0.059 | 0.005 | 11.579 | 0.002 | 100998\_at,92866\_at,94285\_at |
| 7 | antigen processing | 5 | 27 | 107 | 6246 | 0.047 | 0.004 | 10.817 | 0 | 97540\_f\_at,98472\_at,100998\_at,92866\_at,94285\_at |
| 8 | antigen processing, endogenous antigen via MHC class I | 2 | 15 | 51 | 2164 | 0.039 | 0.007 | 5.659 | 0.047 | 97540\_f\_at,98472\_at |
| 8 | antigen processing, exogenous antigen via MHC class II | 3 | 12 | 51 | 2164 | 0.059 | 0.006 | 10.598 | 0.002 | 100998\_at,92866\_at,94285\_at |
| 7 | humoral immune response | 2 | 103 | 107 | 6246 | 0.019 | 0.016 | 1.133 | 0.53 | 92198\_s\_at,101568\_at |
| 8 | humoral defense mechanism (sensu Vertebrata) | 2 | 39 | 51 | 2164 | 0.039 | 0.018 | 2.176 | 0.234 | 92198\_s\_at,101568\_at |
| 9 | complement activation | 2 | 38 | 22 | 911 | 0.091 | 0.042 | 2.18 | 0.233 | 92198\_s\_at,101568\_at |
| 10 | complement activation, classical pathway | 1 | 17 | 4 | 197 | 0.25 | 0.086 | 2.897 | 0.305 | 92198\_s\_at |
| 10 | immediate hypersensitivity response | 1 | 4 | 4 | 197 | 0.25 | 0.02 | 12.315 | 0.079 | 101568\_at |
| 3 | response to stress | 1 | 400 | 214 | 10726 | 0.005 | 0.037 | 0.125 | 1 | 96831\_at |
| 3 | secretion | 2 | 16 | 214 | 10726 | 0.009 | 0.001 | 6.275 | 0.04 | 101398\_at,97984\_i\_at |
| 4 | protein secretion | 2 | 8 | 248 | 13100 | 0.008 | 0.001 | 13.213 | 0.009 | 101398\_at,97984\_i\_at |

  
