# Supplementary material for: Molecular Signatures of Proliferation and Quiescence in Hematopoietic Stem Cells
Source: PLoS Biol. 2004 Sep 28;2(10):e301. doi: 10.1371/journal.pbio.0020301 (PMC520599; doi:10.1371/journal.pbio.0020301)
Supplement: Table S31 — (31 KB HTML). [file pbio.0020301.st031.html]

   Significant Tom Day 1   

# Significant Tom Day 1

|  |  |  |  |  |  |  |  |  |  |  |  |
| --- | --- | --- | --- | --- | --- | --- | --- | --- | --- | --- | --- |
| GOLevel | GOTerm | ProbeCount | ArrayCount | ListGOLevelCount | ArrayGoLevelCount | ListFq | ArrayFq | FoldChange | H-Pvalue | ProbeIds | GeneNames |
| 5 | cell-cell adhesion | 3 | 44 | 209 | 11544 | 0.014 | 0.004 | 3.766 | 0.045 | 104041\_at,96752\_at,96785\_at | RIKEN cDNA 1810009A16 gene,intercellular adhesion molecule,H2-K region expressed gene 2 |
| 10 | regulation of BMP signaling pathway | 1 | 2 | 4 | 197 | 0.25 | 0.01 | 24.631 | 0.04 | 99532\_at | transducer of ErbB-2.1 |
| 8 | FGF receptor signaling pathway | 1 | 2 | 51 | 2164 | 0.02 | 0.001 | 21.315 | 0.047 | 93294\_at | connective tissue growth factor |
| 7 | G-protein signaling, coupled to IP3 second messenger (phospholipase C activating) | 2 | 13 | 107 | 6246 | 0.019 | 0.002 | 8.986 | 0.02 | 99909\_at,99340\_at | transient receptor potential cation channel, subfamily C, member 6,guanine nucleotide binding protein, beta 2, related sequence 1 |
| 7 | MAPKKK cascade | 3 | 18 | 107 | 6246 | 0.028 | 0.003 | 9.736 | 0.003 | 101979\_at,102779\_at,161666\_f\_at | growth arrest and DNA-damage-inducible 45 gamma,growth arrest and DNA-damage-inducible 45 beta,growth arrest and DNA-damage-inducible 45 beta |
| 8 | activation of MAPKK | 3 | 3 | 51 | 2164 | 0.059 | 0.001 | 42.317 | 0 | 101979\_at,102779\_at,161666\_f\_at | growth arrest and DNA-damage-inducible 45 gamma,growth arrest and DNA-damage-inducible 45 beta,growth arrest and DNA-damage-inducible 45 beta |
| 3 | cell differentiation | 8 | 137 | 214 | 10726 | 0.037 | 0.013 | 2.927 | 0.006 | 101979\_at,102779\_at,161666\_f\_at,92653\_at,96596\_at,97973\_at,96592\_at,99532\_at | growth arrest and DNA-damage-inducible 45 gamma,growth arrest and DNA-damage-inducible 45 beta,growth arrest and DNA-damage-inducible 45 beta,RIKEN cDNA D530037H12 gene,N-myc downstream regulated-like,T-cell acute lymphocytic leukemia 1,phosphatidylinositol 3-kinase, regulatory subunit, polypeptide 1 (p85 alpha),transducer of ErbB-2.1 |
| 4 | lymphocytic blood cell differentiation | 2 | 6 | 248 | 13100 | 0.008 | 0 | 17.522 | 0.005 | 96592\_at,101979\_at | phosphatidylinositol 3-kinase, regulatory subunit, polypeptide 1 (p85 alpha),growth arrest and DNA-damage-inducible 45 gamma |
| 5 | B-cell differentiation | 1 | 1 | 209 | 11544 | 0.005 | 0 | 53.111 | 0.018 | 96592\_at | phosphatidylinositol 3-kinase, regulatory subunit, polypeptide 1 (p85 alpha) |
| 5 | T-cell differentiation | 1 | 2 | 209 | 11544 | 0.005 | 0 | 28.118 | 0.036 | 101979\_at | growth arrest and DNA-damage-inducible 45 gamma |
| 6 | T-helper cell differentiation | 1 | 1 | 170 | 9498 | 0.006 | 0 | 53.455 | 0.018 | 101979\_at | growth arrest and DNA-damage-inducible 45 gamma |
| 7 | T-helper 1 cell differentiation | 1 | 1 | 107 | 6246 | 0.009 | 0 | 58.438 | 0.017 | 101979\_at | growth arrest and DNA-damage-inducible 45 gamma |
| 4 | osteoblast differentiation | 1 | 1 | 248 | 13100 | 0.004 | 0 | 50.375 | 0.019 | 99532\_at | transducer of ErbB-2.1 |
| 5 | regulation of osteoblast differentiation | 1 | 1 | 209 | 11544 | 0.005 | 0 | 53.111 | 0.018 | 99532\_at | transducer of ErbB-2.1 |
| 6 | negative regulation of osteoblast differentiation | 1 | 1 | 170 | 9498 | 0.006 | 0 | 53.455 | 0.018 | 99532\_at | transducer of ErbB-2.1 |
| 4 | cell growth | 6 | 51 | 248 | 13100 | 0.024 | 0.004 | 6.219 | 0 | 101059\_at,103899\_at,92780\_f\_at,93294\_at,92262\_at,92263\_at | necdin,RIKEN cDNA 4930558F19 gene,NA,connective tissue growth factor,wild-type p53-induced gene 1,B-cell receptor-associated protein 37 |
| 5 | regulation of cell growth | 6 | 38 | 209 | 11544 | 0.029 | 0.003 | 8.726 | 0 | 101059\_at,103899\_at,92780\_f\_at,93294\_at,92262\_at,92263\_at | necdin,RIKEN cDNA 4930558F19 gene,NA,connective tissue growth factor,wild-type p53-induced gene 1,B-cell receptor-associated protein 37 |
| 6 | negative regulation of cell growth | 2 | 2 | 170 | 9498 | 0.012 | 0 | 56 | 0 | 92262\_at,92263\_at | wild-type p53-induced gene 1,B-cell receptor-associated protein 37 |
| 9 | nucleosome assembly | 3 | 28 | 22 | 911 | 0.136 | 0.031 | 4.436 | 0.027 | 160998\_at,93020\_at,93021\_at | DNA segment, Chr 4, ERATO Doi 89, expressed,reduced expression 3,reduced expression 3 |
| 7 | cell cycle arrest | 3 | 20 | 107 | 6246 | 0.028 | 0.003 | 8.762 | 0.005 | 94881\_at,96801\_at,98067\_at | cyclin-dependent kinase inhibitor 1A (P21),adenylate kinase 1,cyclin-dependent kinase inhibitor 1A (P21) |
| 5 | intracellular transport | 12 | 351 | 209 | 11544 | 0.057 | 0.03 | 1.888 | 0.026 | 104063\_at,101398\_at,92847\_s\_at,93252\_at,93347\_at,96936\_at,97984\_i\_at,98887\_at,160343\_at,97515\_at,161250\_at,101554\_at | Src activating and signaling molecule,syntaxin binding protein 2,mannose-6-phosphate receptor, cation dependent,B-cell receptor-associated protein 31,RAB24, member RAS oncogene family,coatomer protein complex, subunit gamma 1,kinesin 2,N-ethylmaleimide sensitive fusion protein attachment protein alpha,signal recognition particle 19,hydroxysteroid (17-beta) dehydrogenase 4,importin 4,nuclear factor of kappa light chain gene enhancer in B-cells inhibitor, alpha |
| 6 | intracellular protein transport | 12 | 284 | 170 | 9498 | 0.071 | 0.03 | 2.361 | 0.005 | 101398\_at,104063\_at,92847\_s\_at,93252\_at,93347\_at,96936\_at,97984\_i\_at,98887\_at,160343\_at,97515\_at,161250\_at,101554\_at | syntaxin binding protein 2,Src activating and signaling molecule,mannose-6-phosphate receptor, cation dependent,B-cell receptor-associated protein 31,RAB24, member RAS oncogene family,coatomer protein complex, subunit gamma 1,kinesin 2,N-ethylmaleimide sensitive fusion protein attachment protein alpha,signal recognition particle 19,hydroxysteroid (17-beta) dehydrogenase 4,importin 4,nuclear factor of kappa light chain gene enhancer in B-cells inhibitor, alpha |
| 5 | protein transport | 12 | 297 | 209 | 11544 | 0.057 | 0.026 | 2.232 | 0.008 | 101398\_at,104063\_at,93252\_at,93347\_at,96936\_at,97984\_i\_at,98887\_at,92847\_s\_at,160343\_at,97515\_at,161250\_at,101554\_at | syntaxin binding protein 2,Src activating and signaling molecule,B-cell receptor-associated protein 31,RAB24, member RAS oncogene family,coatomer protein complex, subunit gamma 1,kinesin 2,N-ethylmaleimide sensitive fusion protein attachment protein alpha,mannose-6-phosphate receptor, cation dependent,signal recognition particle 19,hydroxysteroid (17-beta) dehydrogenase 4,importin 4,nuclear factor of kappa light chain gene enhancer in B-cells inhibitor, alpha |
| 6 | intracellular protein transport | 12 | 284 | 170 | 9498 | 0.071 | 0.03 | 2.361 | 0.005 | 101398\_at,104063\_at,92847\_s\_at,93252\_at,93347\_at,96936\_at,97984\_i\_at,98887\_at,160343\_at,97515\_at,161250\_at,101554\_at | syntaxin binding protein 2,Src activating and signaling molecule,mannose-6-phosphate receptor, cation dependent,B-cell receptor-associated protein 31,RAB24, member RAS oncogene family,coatomer protein complex, subunit gamma 1,kinesin 2,N-ethylmaleimide sensitive fusion protein attachment protein alpha,signal recognition particle 19,hydroxysteroid (17-beta) dehydrogenase 4,importin 4,nuclear factor of kappa light chain gene enhancer in B-cells inhibitor, alpha |
| 3 | cell differentiation | 8 | 137 | 214 | 10726 | 0.037 | 0.013 | 2.927 | 0.006 | 101979\_at,102779\_at,161666\_f\_at,92653\_at,96596\_at,97973\_at,96592\_at,99532\_at | growth arrest and DNA-damage-inducible 45 gamma,growth arrest and DNA-damage-inducible 45 beta,growth arrest and DNA-damage-inducible 45 beta,RIKEN cDNA D530037H12 gene,N-myc downstream regulated-like,T-cell acute lymphocytic leukemia 1,phosphatidylinositol 3-kinase, regulatory subunit, polypeptide 1 (p85 alpha),transducer of ErbB-2.1 |
| 4 | lymphocytic blood cell differentiation | 2 | 6 | 248 | 13100 | 0.008 | 0 | 17.522 | 0.005 | 96592\_at,101979\_at | phosphatidylinositol 3-kinase, regulatory subunit, polypeptide 1 (p85 alpha),growth arrest and DNA-damage-inducible 45 gamma |
| 5 | B-cell differentiation | 1 | 1 | 209 | 11544 | 0.005 | 0 | 53.111 | 0.018 | 96592\_at | phosphatidylinositol 3-kinase, regulatory subunit, polypeptide 1 (p85 alpha) |
| 5 | T-cell differentiation | 1 | 2 | 209 | 11544 | 0.005 | 0 | 28.118 | 0.036 | 101979\_at | growth arrest and DNA-damage-inducible 45 gamma |
| 6 | T-helper cell differentiation | 1 | 1 | 170 | 9498 | 0.006 | 0 | 53.455 | 0.018 | 101979\_at | growth arrest and DNA-damage-inducible 45 gamma |
| 7 | T-helper 1 cell differentiation | 1 | 1 | 107 | 6246 | 0.009 | 0 | 58.438 | 0.017 | 101979\_at | growth arrest and DNA-damage-inducible 45 gamma |
| 4 | osteoblast differentiation | 1 | 1 | 248 | 13100 | 0.004 | 0 | 50.375 | 0.019 | 99532\_at | transducer of ErbB-2.1 |
| 5 | regulation of osteoblast differentiation | 1 | 1 | 209 | 11544 | 0.005 | 0 | 53.111 | 0.018 | 99532\_at | transducer of ErbB-2.1 |
| 6 | negative regulation of osteoblast differentiation | 1 | 1 | 170 | 9498 | 0.006 | 0 | 53.455 | 0.018 | 99532\_at | transducer of ErbB-2.1 |
| 5 | hemopoiesis | 4 | 22 | 209 | 11544 | 0.019 | 0.002 | 10.021 | 0.001 | 101593\_at,97973\_at,96592\_at,101979\_at | cysteine rich protein 2,T-cell acute lymphocytic leukemia 1,phosphatidylinositol 3-kinase, regulatory subunit, polypeptide 1 (p85 alpha),growth arrest and DNA-damage-inducible 45 gamma |
| 6 | T-helper cell differentiation | 1 | 1 | 170 | 9498 | 0.006 | 0 | 53.455 | 0.018 | 101979\_at | growth arrest and DNA-damage-inducible 45 gamma |
| 7 | T-helper 1 cell differentiation | 1 | 1 | 107 | 6246 | 0.009 | 0 | 58.438 | 0.017 | 101979\_at | growth arrest and DNA-damage-inducible 45 gamma |
| 5 | B-cell differentiation | 1 | 1 | 209 | 11544 | 0.005 | 0 | 53.111 | 0.018 | 96592\_at | phosphatidylinositol 3-kinase, regulatory subunit, polypeptide 1 (p85 alpha) |
| 5 | T-cell differentiation | 1 | 2 | 209 | 11544 | 0.005 | 0 | 28.118 | 0.036 | 101979\_at | growth arrest and DNA-damage-inducible 45 gamma |
| 6 | T-helper cell differentiation | 1 | 1 | 170 | 9498 | 0.006 | 0 | 53.455 | 0.018 | 101979\_at | growth arrest and DNA-damage-inducible 45 gamma |
| 7 | T-helper 1 cell differentiation | 1 | 1 | 107 | 6246 | 0.009 | 0 | 58.438 | 0.017 | 101979\_at | growth arrest and DNA-damage-inducible 45 gamma |
| 6 | negative regulation of osteoblast differentiation | 1 | 1 | 170 | 9498 | 0.006 | 0 | 53.455 | 0.018 | 99532\_at | transducer of ErbB-2.1 |
| 5 | regulation of osteoblast differentiation | 1 | 1 | 209 | 11544 | 0.005 | 0 | 53.111 | 0.018 | 99532\_at | transducer of ErbB-2.1 |
| 6 | negative regulation of osteoblast differentiation | 1 | 1 | 170 | 9498 | 0.006 | 0 | 53.455 | 0.018 | 99532\_at | transducer of ErbB-2.1 |
| 4 | lymphocytic blood cell differentiation | 2 | 6 | 248 | 13100 | 0.008 | 0 | 17.522 | 0.005 | 96592\_at,101979\_at | phosphatidylinositol 3-kinase, regulatory subunit, polypeptide 1 (p85 alpha),growth arrest and DNA-damage-inducible 45 gamma |
| 5 | B-cell differentiation | 1 | 1 | 209 | 11544 | 0.005 | 0 | 53.111 | 0.018 | 96592\_at | phosphatidylinositol 3-kinase, regulatory subunit, polypeptide 1 (p85 alpha) |
| 5 | T-cell differentiation | 1 | 2 | 209 | 11544 | 0.005 | 0 | 28.118 | 0.036 | 101979\_at | growth arrest and DNA-damage-inducible 45 gamma |
| 6 | T-helper cell differentiation | 1 | 1 | 170 | 9498 | 0.006 | 0 | 53.455 | 0.018 | 101979\_at | growth arrest and DNA-damage-inducible 45 gamma |
| 7 | T-helper 1 cell differentiation | 1 | 1 | 107 | 6246 | 0.009 | 0 | 58.438 | 0.017 | 101979\_at | growth arrest and DNA-damage-inducible 45 gamma |
| 4 | osteoblast differentiation | 1 | 1 | 248 | 13100 | 0.004 | 0 | 50.375 | 0.019 | 99532\_at | transducer of ErbB-2.1 |
| 5 | regulation of osteoblast differentiation | 1 | 1 | 209 | 11544 | 0.005 | 0 | 53.111 | 0.018 | 99532\_at | transducer of ErbB-2.1 |
| 6 | negative regulation of osteoblast differentiation | 1 | 1 | 170 | 9498 | 0.006 | 0 | 53.455 | 0.018 | 99532\_at | transducer of ErbB-2.1 |
| 4 | cell growth | 6 | 51 | 248 | 13100 | 0.024 | 0.004 | 6.219 | 0 | 101059\_at,103899\_at,92780\_f\_at,93294\_at,92262\_at,92263\_at | necdin,RIKEN cDNA 4930558F19 gene,NA,connective tissue growth factor,wild-type p53-induced gene 1,B-cell receptor-associated protein 37 |
| 5 | regulation of cell growth | 6 | 38 | 209 | 11544 | 0.029 | 0.003 | 8.726 | 0 | 101059\_at,103899\_at,92780\_f\_at,93294\_at,92262\_at,92263\_at | necdin,RIKEN cDNA 4930558F19 gene,NA,connective tissue growth factor,wild-type p53-induced gene 1,B-cell receptor-associated protein 37 |
| 6 | negative regulation of cell growth | 2 | 2 | 170 | 9498 | 0.012 | 0 | 56 | 0 | 92262\_at,92263\_at | wild-type p53-induced gene 1,B-cell receptor-associated protein 37 |
| 9 | nucleosome assembly | 3 | 28 | 22 | 911 | 0.136 | 0.031 | 4.436 | 0.027 | 160998\_at,93020\_at,93021\_at | DNA segment, Chr 4, ERATO Doi 89, expressed,reduced expression 3,reduced expression 3 |
| 7 | cell cycle arrest | 3 | 20 | 107 | 6246 | 0.028 | 0.003 | 8.762 | 0.005 | 94881\_at,96801\_at,98067\_at | cyclin-dependent kinase inhibitor 1A (P21),adenylate kinase 1,cyclin-dependent kinase inhibitor 1A (P21) |
| 5 | intracellular transport | 12 | 351 | 209 | 11544 | 0.057 | 0.03 | 1.888 | 0.026 | 104063\_at,101398\_at,92847\_s\_at,93252\_at,93347\_at,96936\_at,97984\_i\_at,98887\_at,160343\_at,97515\_at,161250\_at,101554\_at | Src activating and signaling molecule,syntaxin binding protein 2,mannose-6-phosphate receptor, cation dependent,B-cell receptor-associated protein 31,RAB24, member RAS oncogene family,coatomer protein complex, subunit gamma 1,kinesin 2,N-ethylmaleimide sensitive fusion protein attachment protein alpha,signal recognition particle 19,hydroxysteroid (17-beta) dehydrogenase 4,importin 4,nuclear factor of kappa light chain gene enhancer in B-cells inhibitor, alpha |
| 6 | intracellular protein transport | 12 | 284 | 170 | 9498 | 0.071 | 0.03 | 2.361 | 0.005 | 101398\_at,104063\_at,92847\_s\_at,93252\_at,93347\_at,96936\_at,97984\_i\_at,98887\_at,160343\_at,97515\_at,161250\_at,101554\_at | syntaxin binding protein 2,Src activating and signaling molecule,mannose-6-phosphate receptor, cation dependent,B-cell receptor-associated protein 31,RAB24, member RAS oncogene family,coatomer protein complex, subunit gamma 1,kinesin 2,N-ethylmaleimide sensitive fusion protein attachment protein alpha,signal recognition particle 19,hydroxysteroid (17-beta) dehydrogenase 4,importin 4,nuclear factor of kappa light chain gene enhancer in B-cells inhibitor, alpha |
| 5 | protein transport | 12 | 297 | 209 | 11544 | 0.057 | 0.026 | 2.232 | 0.008 | 101398\_at,104063\_at,93252\_at,93347\_at,96936\_at,97984\_i\_at,98887\_at,92847\_s\_at,160343\_at,97515\_at,161250\_at,101554\_at | syntaxin binding protein 2,Src activating and signaling molecule,B-cell receptor-associated protein 31,RAB24, member RAS oncogene family,coatomer protein complex, subunit gamma 1,kinesin 2,N-ethylmaleimide sensitive fusion protein attachment protein alpha,mannose-6-phosphate receptor, cation dependent,signal recognition particle 19,hydroxysteroid (17-beta) dehydrogenase 4,importin 4,nuclear factor of kappa light chain gene enhancer in B-cells inhibitor, alpha |
| 6 | intracellular protein transport | 12 | 284 | 170 | 9498 | 0.071 | 0.03 | 2.361 | 0.005 | 101398\_at,104063\_at,92847\_s\_at,93252\_at,93347\_at,96936\_at,97984\_i\_at,98887\_at,160343\_at,97515\_at,161250\_at,101554\_at | syntaxin binding protein 2,Src activating and signaling molecule,mannose-6-phosphate receptor, cation dependent,B-cell receptor-associated protein 31,RAB24, member RAS oncogene family,coatomer protein complex, subunit gamma 1,kinesin 2,N-ethylmaleimide sensitive fusion protein attachment protein alpha,signal recognition particle 19,hydroxysteroid (17-beta) dehydrogenase 4,importin 4,nuclear factor of kappa light chain gene enhancer in B-cells inhibitor, alpha |
| 5 | alcohol catabolism | 4 | 58 | 209 | 11544 | 0.019 | 0.005 | 3.813 | 0.021 | 101990\_at,160090\_f\_at,160428\_at,103326\_at | lactate dehydrogenase 2, B chain,aldolase 1, A isoform,succinate-Coenzyme A ligase, GDP-forming, beta subunit,RIKEN cDNA E130107N23 gene |
| 6 | monosaccharide catabolism | 4 | 58 | 170 | 9498 | 0.024 | 0.006 | 3.851 | 0.02 | 101990\_at,160090\_f\_at,160428\_at,103326\_at | lactate dehydrogenase 2, B chain,aldolase 1, A isoform,succinate-Coenzyme A ligase, GDP-forming, beta subunit,RIKEN cDNA E130107N23 gene |
| 7 | hexose catabolism | 4 | 58 | 107 | 6246 | 0.037 | 0.009 | 4.024 | 0.017 | 101990\_at,160090\_f\_at,160428\_at,103326\_at | lactate dehydrogenase 2, B chain,aldolase 1, A isoform,succinate-Coenzyme A ligase, GDP-forming, beta subunit,RIKEN cDNA E130107N23 gene |
| 8 | glucose catabolism | 4 | 58 | 51 | 2164 | 0.078 | 0.027 | 2.926 | 0.046 | 101990\_at,160090\_f\_at,160428\_at,103326\_at | lactate dehydrogenase 2, B chain,aldolase 1, A isoform,succinate-Coenzyme A ligase, GDP-forming, beta subunit,RIKEN cDNA E130107N23 gene |
| 10 | pentose-phosphate shunt, non-oxidative branch | 1 | 1 | 4 | 197 | 0.25 | 0.005 | 49.213 | 0.02 | 103326\_at | RIKEN cDNA E130107N23 gene |
| 8 | interferon-gamma biosynthesis | 1 | 1 | 51 | 2164 | 0.02 | 0 | 42.63 | 0.024 | 101979\_at | growth arrest and DNA-damage-inducible 45 gamma |
| 9 | GMP biosynthesis | 1 | 2 | 22 | 911 | 0.045 | 0.002 | 20.659 | 0.048 | 100578\_at | inosine 5'-phosphate dehydrogenase 2 |
| 9 | GMP biosynthesis | 1 | 2 | 22 | 911 | 0.045 | 0.002 | 20.659 | 0.048 | 100578\_at | inosine 5'-phosphate dehydrogenase 2 |
| 5 | alcohol catabolism | 4 | 58 | 209 | 11544 | 0.019 | 0.005 | 3.813 | 0.021 | 101990\_at,160090\_f\_at,160428\_at,103326\_at | lactate dehydrogenase 2, B chain,aldolase 1, A isoform,succinate-Coenzyme A ligase, GDP-forming, beta subunit,RIKEN cDNA E130107N23 gene |
| 6 | monosaccharide catabolism | 4 | 58 | 170 | 9498 | 0.024 | 0.006 | 3.851 | 0.02 | 101990\_at,160090\_f\_at,160428\_at,103326\_at | lactate dehydrogenase 2, B chain,aldolase 1, A isoform,succinate-Coenzyme A ligase, GDP-forming, beta subunit,RIKEN cDNA E130107N23 gene |
| 7 | hexose catabolism | 4 | 58 | 107 | 6246 | 0.037 | 0.009 | 4.024 | 0.017 | 101990\_at,160090\_f\_at,160428\_at,103326\_at | lactate dehydrogenase 2, B chain,aldolase 1, A isoform,succinate-Coenzyme A ligase, GDP-forming, beta subunit,RIKEN cDNA E130107N23 gene |
| 8 | glucose catabolism | 4 | 58 | 51 | 2164 | 0.078 | 0.027 | 2.926 | 0.046 | 101990\_at,160090\_f\_at,160428\_at,103326\_at | lactate dehydrogenase 2, B chain,aldolase 1, A isoform,succinate-Coenzyme A ligase, GDP-forming, beta subunit,RIKEN cDNA E130107N23 gene |
| 10 | pentose-phosphate shunt, non-oxidative branch | 1 | 1 | 4 | 197 | 0.25 | 0.005 | 49.213 | 0.02 | 103326\_at | RIKEN cDNA E130107N23 gene |
| 8 | ATP-dependent proteolysis | 1 | 1 | 51 | 2164 | 0.02 | 0 | 42.63 | 0.024 | 96890\_at | RIKEN cDNA 1300002A08 gene |
| 6 | sphingolipid metabolism | 2 | 14 | 170 | 9498 | 0.012 | 0.001 | 8 | 0.025 | 94502\_at,102936\_at | DNA segment, Chr 13, Wayne State University 50, expressed,UDP-Gal:betaGlcNAc beta 1,4-galactosyltransferase, polypeptide 6 |
| 8 | sphinganine metabolism | 1 | 1 | 51 | 2164 | 0.02 | 0 | 42.63 | 0.024 | 94502\_at | DNA segment, Chr 13, Wayne State University 50, expressed |
| 9 | sphinganine-1-phosphate metabolism | 1 | 1 | 22 | 911 | 0.045 | 0.001 | 41.318 | 0.024 | 94502\_at | DNA segment, Chr 13, Wayne State University 50, expressed |
| 7 | nucleotide-excision repair | 2 | 20 | 107 | 6246 | 0.019 | 0.003 | 5.841 | 0.045 | 103483\_at,94073\_at | excision repair cross-complementing rodent repair deficiency,complementation group 5,polymerase (RNA) II (DNA directed) polypeptide G |
| 8 | transcription-coupled nucleotide-excision repair | 1 | 1 | 51 | 2164 | 0.02 | 0 | 42.63 | 0.024 | 94073\_at | polymerase (RNA) II (DNA directed) polypeptide G |
| 5 | glutathione conjugation reaction | 2 | 15 | 209 | 11544 | 0.01 | 0.001 | 7.362 | 0.029 | 100629\_at,93543\_f\_at | glutathione S-transferase, mu 5,glutathione S-transferase, mu 1 |
| 3 | respiratory gaseous exchange | 2 | 10 | 214 | 10726 | 0.009 | 0.001 | 10.054 | 0.016 | 101059\_at,102737\_at | necdin,endothelin 1 |
| 5 | defense response | 15 | 471 | 209 | 11544 | 0.072 | 0.041 | 1.759 | 0.024 | 100998\_at,104041\_at,92866\_at,94000\_at,94285\_at,96752\_at,97540\_f\_at,98472\_at,102401\_at,103634\_at,104597\_at,93252\_at,94799\_at,92198\_s\_at,101568\_at | histocompatibility 2, class II antigen A, beta 1,RIKEN cDNA 1810009A16 gene,histocompatibility 2, class II antigen A, alpha,CD8 antigen, beta chain,histocompatibility 2, class II antigen E beta,intercellular adhesion molecule,histocompatibility 2, D region locus 1,histocompatibility 2, T region locus 23,interferon regulatory factor 1,interferon dependent positive acting transcription factor 3 gamma,guanylate nucleotide binding protein 2,B-cell receptor-associated protein 31,coagulation factor VIII,decay accelerating factor 2,proline synthetase co-transcribed |
| 6 | immune response | 13 | 362 | 170 | 9498 | 0.076 | 0.038 | 2.007 | 0.013 | 100998\_at,102401\_at,103634\_at,104597\_at,92866\_at,93252\_at,94000\_at,94285\_at,97540\_f\_at,98472\_at,94799\_at,92198\_s\_at,101568\_at | histocompatibility 2, class II antigen A, beta 1,interferon regulatory factor 1,interferon dependent positive acting transcription factor 3 gamma,guanylate nucleotide binding protein 2,histocompatibility 2, class II antigen A, alpha,B-cell receptor-associated protein 31,CD8 antigen, beta chain,histocompatibility 2, class II antigen E beta,histocompatibility 2, D region locus 1,histocompatibility 2, T region locus 23,coagulation factor VIII,decay accelerating factor 2,proline synthetase co-transcribed |
| 7 | antigen presentation | 5 | 26 | 107 | 6246 | 0.047 | 0.004 | 11.233 | 0 | 97540\_f\_at,98472\_at,100998\_at,92866\_at,94285\_at | histocompatibility 2, D region locus 1,histocompatibility 2, T region locus 23,histocompatibility 2, class II antigen A, beta 1,histocompatibility 2, class II antigen A, alpha,histocompatibility 2, class II antigen E beta |
| 8 | antigen presentation, endogenous antigen | 2 | 15 | 51 | 2164 | 0.039 | 0.007 | 5.659 | 0.047 | 97540\_f\_at,98472\_at | histocompatibility 2, D region locus 1,histocompatibility 2, T region locus 23 |
| 8 | antigen presentation, exogenous antigen | 3 | 11 | 51 | 2164 | 0.059 | 0.005 | 11.579 | 0.002 | 100998\_at,92866\_at,94285\_at | histocompatibility 2, class II antigen A, beta 1,histocompatibility 2, class II antigen A, alpha,histocompatibility 2, class II antigen E beta |
| 7 | antigen processing | 5 | 27 | 107 | 6246 | 0.047 | 0.004 | 10.817 | 0 | 97540\_f\_at,98472\_at,100998\_at,92866\_at,94285\_at | histocompatibility 2, D region locus 1,histocompatibility 2, T region locus 23,histocompatibility 2, class II antigen A, beta 1,histocompatibility 2, class II antigen A, alpha,histocompatibility 2, class II antigen E beta |
| 8 | antigen processing, endogenous antigen via MHC class I | 2 | 15 | 51 | 2164 | 0.039 | 0.007 | 5.659 | 0.047 | 97540\_f\_at,98472\_at | histocompatibility 2, D region locus 1,histocompatibility 2, T region locus 23 |
| 8 | antigen processing, exogenous antigen via MHC class II | 3 | 12 | 51 | 2164 | 0.059 | 0.006 | 10.598 | 0.002 | 100998\_at,92866\_at,94285\_at | histocompatibility 2, class II antigen A, beta 1,histocompatibility 2, class II antigen A, alpha,histocompatibility 2, class II antigen E beta |
| 3 | secretion | 2 | 16 | 214 | 10726 | 0.009 | 0.001 | 6.275 | 0.04 | 101398\_at,97984\_i\_at | syntaxin binding protein 2,kinesin 2 |
| 4 | protein secretion | 2 | 8 | 248 | 13100 | 0.008 | 0.001 | 13.213 | 0.009 | 101398\_at,97984\_i\_at | syntaxin binding protein 2,kinesin 2 |

  
