# Supplementary material for: Molecular Signatures of Proliferation and Quiescence in Hematopoietic Stem Cells
Source: PLoS Biol. 2004 Sep 28;2(10):e301. doi: 10.1371/journal.pbio.0020301 (PMC520599; doi:10.1371/journal.pbio.0020301)
Supplement: Table S32 — (136 KB HTML). [file pbio.0020301.st032.html]

   Full Tom Day 10   

# Full Tom Day 10

|  |  |  |  |  |  |  |  |  |  |  |
| --- | --- | --- | --- | --- | --- | --- | --- | --- | --- | --- |
| GOLevel | GOTerm | ProbeCount | ArrayCount | ListGOLevelCount | ArrayGoLevelCount | ListFq | ArrayFq | FoldChange | H-Pvalue | ProbeIds |
| 0 | Gene\_Ontology | 276 | NA | 276 | 0 | 1 | NA | NA | NA | 103259\_at,102242\_at,162384\_f\_at,102382\_at,101778\_at,161112\_at,100134\_at,104206\_at,104692\_at,92877\_at,93199\_at,96784\_at,103518\_at,98862\_at,92945\_at,100348\_at,102024\_at,102063\_at,104388\_at,160495\_at,162206\_f\_at,92232\_at,93315\_at,93718\_at,94042\_f\_at,94331\_at,95023\_at,96300\_f\_at,97509\_f\_at,99100\_at,101144\_at,102658\_at,102663\_at,161689\_f\_at,93914\_at,94928\_at,95295\_s\_at,92986\_g\_at,102787\_at,103656\_at,93193\_at,94192\_at,103596\_at,99953\_at,96818\_at,101186\_at,100293\_at,104256\_at,104257\_g\_at,104425\_at,160698\_s\_at,160965\_at,93319\_at,96333\_g\_at,96511\_s\_at,94264\_at,161667\_r\_at,94006\_at,100635\_at,96056\_at,97319\_at,94236\_at,95287\_at,100988\_at,103812\_at,160920\_at,161980\_f\_at,94003\_at,97285\_f\_at,102580\_r\_at,161610\_at,162204\_r\_at,97118\_at,97497\_at,100710\_at,101697\_f\_at,104533\_at,104714\_at,161551\_f\_at,92400\_at,93104\_at,94397\_at,94932\_at,104263\_at,100606\_at,103408\_at,100877\_at,92542\_at,95489\_at,97593\_f\_at,104683\_at,160287\_at,160461\_f\_at,95119\_at,98461\_at,160140\_at,104471\_at,100939\_at,98882\_s\_at,98884\_r\_at,94012\_at,101844\_at,95081\_at,100616\_at,93701\_at,98534\_at,94011\_at,95573\_at,95574\_f\_at,95502\_at,101014\_at,101073\_at,102781\_at,99187\_f\_at,99188\_at,95694\_at,95917\_at,94483\_at,104150\_at,160512\_at,101079\_at,103314\_at,103704\_at,103845\_at,160592\_at,161785\_f\_at,92339\_at,93408\_at,93414\_at,93570\_at,93627\_at,95155\_at,95444\_at,95586\_at,95607\_at,95647\_f\_at,100492\_at,101449\_at,101036\_at,104453\_at,94979\_at,95655\_at,162041\_f\_at,160832\_at,96534\_at,98926\_at,100921\_at,160151\_i\_at,161530\_r\_at,94341\_at,95382\_at,95387\_f\_at,160762\_at,160099\_at,100429\_at,104340\_at,160834\_at,160182\_at,93520\_at,95355\_at,92991\_at,92992\_i\_at,92993\_r\_at,92918\_at,98018\_at,101432\_at,102991\_s\_at,96650\_at,99617\_at,99045\_at,162092\_f\_at,104217\_at,104285\_at,99184\_at,97386\_at,102313\_at,160425\_at,102101\_f\_at,104048\_at,104144\_at,160283\_at,160977\_at,93975\_at,94484\_at,95501\_at,96577\_i\_at,96578\_r\_at,96845\_at,97083\_at,100136\_at,94818\_at,97936\_at,102980\_at,93852\_at,98435\_at,162228\_f\_at,103562\_f\_at,104677\_at,161039\_at,92634\_at,93509\_at,95564\_at,96176\_at,99085\_at,99086\_g\_at,160088\_at,161005\_at,162260\_at,93424\_at,93440\_at,94951\_at,98533\_at,99985\_at,104165\_at,97897\_at,160174\_at,100030\_at,104219\_f\_at,103545\_at,101889\_s\_at,101943\_at,95536\_at,104701\_at,100094\_at,100486\_at,102895\_at,103015\_at,103504\_at,160220\_at,160396\_at,160781\_r\_at,161113\_at,161187\_f\_at,161333\_f\_at,162114\_f\_at,92233\_at,92300\_at,92737\_at,94689\_at,95521\_s\_at,96196\_i\_at,96197\_f\_at,96481\_at,96817\_at,98002\_at,98465\_f\_at,98767\_at,98818\_at,99103\_at,99665\_at,101836\_at,92758\_at,94980\_at,104364\_at,93274\_at,93311\_at,97384\_at,97429\_at,99458\_i\_at,160742\_at,102279\_at,103582\_r\_at,161396\_f\_at,104572\_at,161745\_f\_at,92571\_at,103422\_at,93909\_f\_at,98000\_at,100973\_i\_at,103080\_at,93321\_at,94224\_s\_at,97710\_f\_at,100333\_at,103033\_at |
| 1 | biological\_process | 276 | 6769 | 276 | 6769 | 1 | 1 | 1 | 1 | 103259\_at,102242\_at,162384\_f\_at,102382\_at,101778\_at,161112\_at,100134\_at,104206\_at,104692\_at,92877\_at,93199\_at,96784\_at,103518\_at,98862\_at,92945\_at,100348\_at,102024\_at,102063\_at,104388\_at,160495\_at,162206\_f\_at,92232\_at,93315\_at,93718\_at,94042\_f\_at,94331\_at,95023\_at,96300\_f\_at,97509\_f\_at,99100\_at,101144\_at,102658\_at,102663\_at,161689\_f\_at,93914\_at,94928\_at,95295\_s\_at,92986\_g\_at,102787\_at,103656\_at,93193\_at,94192\_at,103596\_at,99953\_at,96818\_at,101186\_at,100293\_at,104256\_at,104257\_g\_at,104425\_at,160698\_s\_at,160965\_at,93319\_at,96333\_g\_at,96511\_s\_at,94264\_at,161667\_r\_at,94006\_at,100635\_at,96056\_at,97319\_at,94236\_at,95287\_at,100988\_at,103812\_at,160920\_at,161980\_f\_at,94003\_at,97285\_f\_at,102580\_r\_at,161610\_at,162204\_r\_at,97118\_at,97497\_at,100710\_at,101697\_f\_at,104533\_at,104714\_at,161551\_f\_at,92400\_at,93104\_at,94397\_at,94932\_at,104263\_at,100606\_at,103408\_at,100877\_at,92542\_at,95489\_at,97593\_f\_at,104683\_at,160287\_at,160461\_f\_at,95119\_at,98461\_at,160140\_at,104471\_at,100939\_at,98882\_s\_at,98884\_r\_at,94012\_at,101844\_at,95081\_at,100616\_at,93701\_at,98534\_at,94011\_at,95573\_at,95574\_f\_at,95502\_at,101014\_at,101073\_at,102781\_at,99187\_f\_at,99188\_at,95694\_at,95917\_at,94483\_at,104150\_at,160512\_at,101079\_at,103314\_at,103704\_at,103845\_at,160592\_at,161785\_f\_at,92339\_at,93408\_at,93414\_at,93570\_at,93627\_at,95155\_at,95444\_at,95586\_at,95607\_at,95647\_f\_at,100492\_at,101449\_at,101036\_at,104453\_at,94979\_at,95655\_at,162041\_f\_at,160832\_at,96534\_at,98926\_at,100921\_at,160151\_i\_at,161530\_r\_at,94341\_at,95382\_at,95387\_f\_at,160762\_at,160099\_at,100429\_at,104340\_at,160834\_at,160182\_at,93520\_at,95355\_at,92991\_at,92992\_i\_at,92993\_r\_at,92918\_at,98018\_at,101432\_at,102991\_s\_at,96650\_at,99617\_at,99045\_at,162092\_f\_at,104217\_at,104285\_at,99184\_at,97386\_at,102313\_at,160425\_at,102101\_f\_at,104048\_at,104144\_at,160283\_at,160977\_at,93975\_at,94484\_at,95501\_at,96577\_i\_at,96578\_r\_at,96845\_at,97083\_at,100136\_at,94818\_at,97936\_at,102980\_at,93852\_at,98435\_at,162228\_f\_at,103562\_f\_at,104677\_at,161039\_at,92634\_at,93509\_at,95564\_at,96176\_at,99085\_at,99086\_g\_at,160088\_at,161005\_at,162260\_at,93424\_at,93440\_at,94951\_at,98533\_at,99985\_at,104165\_at,97897\_at,160174\_at,100030\_at,104219\_f\_at,103545\_at,101889\_s\_at,101943\_at,95536\_at,104701\_at,100094\_at,100486\_at,102895\_at,103015\_at,103504\_at,160220\_at,160396\_at,160781\_r\_at,161113\_at,161187\_f\_at,161333\_f\_at,162114\_f\_at,92233\_at,92300\_at,92737\_at,94689\_at,95521\_s\_at,96196\_i\_at,96197\_f\_at,96481\_at,96817\_at,98002\_at,98465\_f\_at,98767\_at,98818\_at,99103\_at,99665\_at,101836\_at,92758\_at,94980\_at,104364\_at,93274\_at,93311\_at,97384\_at,97429\_at,99458\_i\_at,160742\_at,102279\_at,103582\_r\_at,161396\_f\_at,104572\_at,161745\_f\_at,92571\_at,103422\_at,93909\_f\_at,98000\_at,100973\_i\_at,103080\_at,93321\_at,94224\_s\_at,97710\_f\_at,100333\_at,103033\_at |
| 2 | behavior | 4 | 63 | 428 | 10540 | 0.009 | 0.006 | 1.564 | 0.253 | 103259\_at,102242\_at,162384\_f\_at,102382\_at |
| 3 | mechanosensory behavior | 1 | 2 | 415 | 10726 | 0.002 | 0 | 12.684 | 0.076 | 103259\_at |
| 3 | rhythmic behavior | 3 | 17 | 415 | 10726 | 0.007 | 0.002 | 4.576 | 0.026 | 102242\_at,162384\_f\_at,102382\_at |
| 4 | circadian rhythm | 2 | 14 | 482 | 13100 | 0.004 | 0.001 | 3.879 | 0.092 | 102242\_at,102382\_at |
| 2 | cellular process | 147 | 3616 | 428 | 10540 | 0.343 | 0.343 | 1.001 | 0.512 | 101778\_at,161112\_at,100134\_at,104206\_at,104692\_at,92877\_at,93199\_at,96784\_at,103518\_at,98862\_at,92945\_at,100348\_at,102024\_at,102063\_at,102242\_at,102382\_at,104388\_at,160495\_at,162206\_f\_at,92232\_at,93315\_at,93718\_at,94042\_f\_at,94331\_at,95023\_at,96300\_f\_at,97509\_f\_at,99100\_at,101144\_at,102658\_at,102663\_at,161689\_f\_at,93914\_at,94928\_at,95295\_s\_at,92986\_g\_at,102787\_at,103656\_at,93193\_at,94192\_at,103596\_at,99953\_at,96818\_at,101186\_at,100293\_at,104256\_at,104257\_g\_at,104425\_at,160698\_s\_at,160965\_at,93319\_at,96333\_g\_at,96511\_s\_at,94264\_at,161667\_r\_at,94006\_at,100635\_at,96056\_at,97319\_at,94236\_at,95287\_at,100988\_at,103812\_at,160920\_at,161980\_f\_at,94003\_at,97285\_f\_at,102580\_r\_at,161610\_at,162204\_r\_at,97118\_at,97497\_at,103259\_at,100710\_at,101697\_f\_at,104533\_at,104714\_at,161551\_f\_at,92400\_at,93104\_at,94397\_at,94932\_at,104263\_at,100606\_at,103408\_at,100877\_at,92542\_at,95489\_at,97593\_f\_at,104683\_at,160287\_at,160461\_f\_at,95119\_at,98461\_at,160140\_at,104471\_at,100939\_at,98882\_s\_at,98884\_r\_at,94012\_at,101844\_at,95081\_at,100616\_at,93701\_at,98534\_at,94011\_at,95573\_at,95574\_f\_at,95502\_at,101014\_at,101073\_at,102781\_at,99187\_f\_at,99188\_at,95694\_at,95917\_at,94483\_at,104150\_at,160512\_at,101079\_at,103314\_at,103704\_at,103845\_at,160592\_at,161785\_f\_at,92339\_at,93408\_at,93414\_at,93570\_at,93627\_at,95155\_at,95444\_at,95586\_at,95607\_at,95647\_f\_at,100492\_at,101449\_at,101036\_at,104453\_at,94979\_at,95655\_at,162041\_f\_at,160832\_at,96534\_at,98926\_at,100921\_at,160151\_i\_at |
| 3 | cell communication | 60 | 1550 | 415 | 10726 | 0.145 | 0.145 | 1 | 0.521 | 101778\_at,161112\_at,100134\_at,104206\_at,104692\_at,92877\_at,93199\_at,96784\_at,103518\_at,98862\_at,92945\_at,100348\_at,102024\_at,102063\_at,102242\_at,102382\_at,104388\_at,160495\_at,162206\_f\_at,92232\_at,93315\_at,93718\_at,94042\_f\_at,94331\_at,95023\_at,96300\_f\_at,97509\_f\_at,99100\_at,101144\_at,102658\_at,102663\_at,161689\_f\_at,93914\_at,94928\_at,95295\_s\_at,92986\_g\_at,102787\_at,103656\_at,93193\_at,94192\_at,103596\_at,99953\_at,96818\_at,101186\_at,100293\_at,104256\_at,104257\_g\_at,104425\_at,160698\_s\_at,160965\_at,93319\_at,96333\_g\_at,96511\_s\_at,94264\_at,161667\_r\_at,94006\_at,100635\_at,96056\_at,97319\_at,94236\_at |
| 4 | cell adhesion | 7 | 322 | 482 | 13100 | 0.015 | 0.025 | 0.591 | 0.955 | 100134\_at,104206\_at,104692\_at,92877\_at,93199\_at,96784\_at,103518\_at |
| 5 | cell-cell adhesion | 2 | 44 | 409 | 11544 | 0.005 | 0.004 | 1.283 | 0.465 | 103518\_at,96784\_at |
| 6 | homophilic cell adhesion | 2 | 31 | 354 | 9498 | 0.006 | 0.003 | 1.733 | 0.322 | 103518\_at,96784\_at |
| 4 | cell-cell signaling | 4 | 123 | 482 | 13100 | 0.008 | 0.009 | 0.884 | 0.668 | 101778\_at,161112\_at,98862\_at,92945\_at |
| 5 | transmission of nerve impulse | 1 | 82 | 409 | 11544 | 0.002 | 0.007 | 0.344 | 0.949 | 92945\_at |
| 6 | synaptic transmission | 1 | 80 | 354 | 9498 | 0.003 | 0.008 | 0.335 | 0.953 | 92945\_at |
| 4 | signal transduction | 52 | 1199 | 482 | 13100 | 0.108 | 0.092 | 1.179 | 0.119 | 100348\_at,102024\_at,102063\_at,102242\_at,102382\_at,104388\_at,160495\_at,162206\_f\_at,92232\_at,93315\_at,93718\_at,94042\_f\_at,94331\_at,95023\_at,96300\_f\_at,97509\_f\_at,98862\_at,99100\_at,101144\_at,102658\_at,102663\_at,161689\_f\_at,93914\_at,94928\_at,100134\_at,95295\_s\_at,92986\_g\_at,102787\_at,103518\_at,103656\_at,93193\_at,94192\_at,103596\_at,99953\_at,96818\_at,101186\_at,100293\_at,104256\_at,104257\_g\_at,104425\_at,160698\_s\_at,160965\_at,93319\_at,96333\_g\_at,96511\_s\_at,94264\_at,161667\_r\_at,94006\_at,100635\_at,96056\_at,97319\_at,94236\_at |
| 5 | cell surface receptor linked signal transduction | 20 | 621 | 409 | 11544 | 0.049 | 0.054 | 0.909 | 0.704 | 101144\_at,102658\_at,102663\_at,161689\_f\_at,93914\_at,94928\_at,100134\_at,95295\_s\_at,92986\_g\_at,102787\_at,103518\_at,103656\_at,93193\_at,94042\_f\_at,94192\_at,103596\_at,99953\_at,96818\_at,98862\_at,101186\_at |
| 6 | enzyme linked receptor protein signaling pathway | 3 | 131 | 354 | 9498 | 0.008 | 0.014 | 0.614 | 0.872 | 100134\_at,95295\_s\_at,92986\_g\_at |
| 7 | transmembrane receptor protein serine/threonine kinase signaling pathway | 1 | 39 | 209 | 6246 | 0.005 | 0.006 | 0.766 | 0.736 | 100134\_at |
| 8 | TGFbeta receptor signaling pathway | 1 | 31 | 77 | 2164 | 0.013 | 0.014 | 0.906 | 0.677 | 100134\_at |
| 9 | regulation of TGFbeta receptor signaling pathway | 1 | 4 | 41 | 911 | 0.024 | 0.004 | 5.556 | 0.168 | 100134\_at |
| 7 | transmembrane receptor protein tyrosine kinase signaling pathway | 1 | 61 | 209 | 6246 | 0.005 | 0.01 | 0.489 | 0.876 | 95295\_s\_at |
| 7 | transmembrane receptor protein tyrosine phosphatase signaling pathway | 1 | 30 | 209 | 6246 | 0.005 | 0.005 | 0.996 | 0.641 | 92986\_g\_at |
| 6 | G-protein coupled receptor protein signaling pathway | 8 | 355 | 354 | 9498 | 0.023 | 0.037 | 0.605 | 0.958 | 102787\_at,103518\_at,103656\_at,93193\_at,94042\_f\_at,94192\_at,103596\_at,99953\_at |
| 7 | G-protein signaling, coupled to IP3 second messenger (phospholipase C activating) | 1 | 13 | 209 | 6246 | 0.005 | 0.002 | 2.298 | 0.358 | 103596\_at |
| 8 | protein kinase C activation | 1 | 5 | 77 | 2164 | 0.013 | 0.002 | 5.623 | 0.166 | 103596\_at |
| 7 | neuropeptide signaling pathway | 3 | 45 | 209 | 6246 | 0.014 | 0.007 | 1.993 | 0.19 | 102787\_at,103518\_at,99953\_at |
| 6 | N signaling pathway | 1 | 5 | 354 | 9498 | 0.003 | 0.001 | 5.321 | 0.173 | 96818\_at |
| 6 | Wnt receptor signaling pathway | 2 | 30 | 354 | 9498 | 0.006 | 0.003 | 1.788 | 0.308 | 98862\_at,101186\_at |
| 7 | frizzled-2 signaling pathway | 1 | 16 | 209 | 6246 | 0.005 | 0.003 | 1.867 | 0.42 | 98862\_at |
| 7 | regulation of Wnt receptor signaling pathway | 1 | 4 | 209 | 6246 | 0.005 | 0.001 | 7.469 | 0.127 | 101186\_at |
| 5 | intracellular signaling cascade | 24 | 485 | 409 | 11544 | 0.059 | 0.042 | 1.397 | 0.062 | 100293\_at,103596\_at,104256\_at,104257\_g\_at,104425\_at,160698\_s\_at,160965\_at,162206\_f\_at,92232\_at,93319\_at,94331\_at,96333\_g\_at,96511\_s\_at,99100\_at,99953\_at,94264\_at,93315\_at,161667\_r\_at,94006\_at,100635\_at,93718\_at,96056\_at,97319\_at,94236\_at |
| 6 | protein kinase cascade | 5 | 50 | 354 | 9498 | 0.014 | 0.005 | 2.684 | 0.038 | 94264\_at,99100\_at,93315\_at,161667\_r\_at,94006\_at |
| 7 | JAK-STAT cascade | 1 | 10 | 209 | 6246 | 0.005 | 0.002 | 2.987 | 0.289 | 99100\_at |
| 7 | MAPKKK cascade | 2 | 18 | 209 | 6246 | 0.01 | 0.003 | 3.323 | 0.12 | 93315\_at,161667\_r\_at |
| 8 | activation of MAPKK | 1 | 3 | 77 | 2164 | 0.013 | 0.001 | 9.345 | 0.103 | 161667\_r\_at |
| 7 | NIK-I-kappaB/NF-kappaB cascade | 1 | 7 | 209 | 6246 | 0.005 | 0.001 | 4.268 | 0.212 | 94006\_at |
| 6 | small GTPase mediated signal transduction | 6 | 135 | 354 | 9498 | 0.017 | 0.014 | 1.193 | 0.39 | 100635\_at,93718\_at,96056\_at,97319\_at,99953\_at,94236\_at |
| 7 | RAC protein signal transduction | 1 | 4 | 209 | 6246 | 0.005 | 0.001 | 7.469 | 0.127 | 94236\_at |
| 3 | cell death | 8 | 207 | 415 | 10726 | 0.019 | 0.019 | 0.999 | 0.552 | 95287\_at,100988\_at,103812\_at,160920\_at,161667\_r\_at,161980\_f\_at,94003\_at,97285\_f\_at |
| 4 | programmed cell death | 7 | 192 | 482 | 13100 | 0.015 | 0.015 | 0.99 | 0.564 | 100988\_at,103812\_at,160920\_at,161667\_r\_at,161980\_f\_at,94003\_at,97285\_f\_at |
| 5 | apoptosis | 7 | 192 | 409 | 11544 | 0.017 | 0.017 | 1.029 | 0.524 | 100988\_at,103812\_at,160920\_at,161667\_r\_at,161980\_f\_at,94003\_at,97285\_f\_at |
| 6 | anti-apoptosis | 1 | 31 | 354 | 9498 | 0.003 | 0.003 | 0.865 | 0.693 | 161980\_f\_at |
| 3 | cell differentiation | 9 | 137 | 415 | 10726 | 0.022 | 0.013 | 1.699 | 0.084 | 100348\_at,102580\_r\_at,161610\_at,161667\_r\_at,162204\_r\_at,97118\_at,97497\_at,103259\_at,92986\_g\_at |
| 4 | epidermal cell differentiation | 1 | 3 | 482 | 13100 | 0.002 | 0 | 9 | 0.106 | 103259\_at |
| 5 | hair cell differentiation | 1 | 3 | 409 | 11544 | 0.002 | 0 | 9.385 | 0.103 | 103259\_at |
| 4 | vasculogenesis | 1 | 7 | 482 | 13100 | 0.002 | 0.001 | 3.906 | 0.231 | 92986\_g\_at |
| 3 | cell growth and/or maintenance | 93 | 2128 | 415 | 10726 | 0.224 | 0.198 | 1.13 | 0.102 | 100710\_at,101697\_f\_at,104533\_at,104714\_at,161551\_f\_at,92400\_at,93104\_at,94264\_at,94397\_at,94932\_at,96056\_at,96511\_s\_at,162206\_f\_at,92232\_at,104263\_at,100606\_at,103408\_at,100877\_at,92542\_at,94236\_at,95489\_at,97593\_f\_at,104683\_at,160287\_at,160461\_f\_at,95119\_at,98461\_at,160140\_at,104471\_at,100939\_at,98882\_s\_at,98884\_r\_at,94012\_at,101844\_at,95081\_at,100616\_at,93701\_at,98534\_at,94011\_at,95573\_at,95574\_f\_at,95502\_at,100293\_at,100348\_at,101014\_at,101073\_at,94003\_at,94928\_at,96300\_f\_at,102781\_at,160495\_at,99187\_f\_at,99188\_at,95694\_at,95917\_at,94483\_at,104150\_at,94331\_at,160512\_at,101079\_at,102658\_at,103314\_at,103704\_at,103845\_at,160592\_at,161689\_f\_at,161785\_f\_at,92339\_at,92945\_at,93408\_at,93414\_at,93570\_at,93627\_at,95155\_at,95444\_at,95586\_at,95607\_at,95647\_f\_at,96333\_g\_at,100492\_at,100635\_at,101449\_at,93718\_at,101036\_at,104453\_at,95023\_at,94979\_at,95655\_at,162041\_f\_at,103812\_at,160832\_at,96534\_at,98926\_at |
| 4 | cell growth | 2 | 51 | 482 | 13100 | 0.004 | 0.004 | 1.067 | 0.565 | 162206\_f\_at,92232\_at |
| 5 | regulation of cell growth | 2 | 38 | 409 | 11544 | 0.005 | 0.003 | 1.486 | 0.392 | 162206\_f\_at,92232\_at |
| 4 | cell homeostasis | 3 | 41 | 482 | 13100 | 0.006 | 0.003 | 1.987 | 0.191 | 104263\_at,100606\_at,103408\_at |
| 5 | cell ion homeostasis | 3 | 37 | 409 | 11544 | 0.007 | 0.003 | 2.283 | 0.142 | 104263\_at,100606\_at,103408\_at |
| 6 | cation homeostasis | 3 | 36 | 354 | 9498 | 0.008 | 0.004 | 2.235 | 0.149 | 104263\_at,100606\_at,103408\_at |
| 7 | di-, tri-valent inorganic cation homeostasis | 3 | 29 | 209 | 6246 | 0.014 | 0.005 | 3.093 | 0.071 | 104263\_at,100606\_at,103408\_at |
| 8 | calcium ion homeostasis | 1 | 13 | 77 | 2164 | 0.013 | 0.006 | 2.161 | 0.376 | 104263\_at |
| 8 | copper ion homeostasis | 1 | 1 | 77 | 2164 | 0.013 | 0 | 28.239 | 0.036 | 100606\_at |
| 8 | iron ion homeostasis | 1 | 15 | 77 | 2164 | 0.013 | 0.007 | 1.874 | 0.42 | 103408\_at |
| 4 | cell organization and biogenesis | 25 | 530 | 482 | 13100 | 0.052 | 0.04 | 1.282 | 0.121 | 100877\_at,92542\_at,94236\_at,95489\_at,97593\_f\_at,104683\_at,160287\_at,160461\_f\_at,95119\_at,98461\_at,160140\_at,104471\_at,100939\_at,98882\_s\_at,98884\_r\_at,94012\_at,101844\_at,95081\_at,100616\_at,93701\_at,98534\_at,94011\_at,95573\_at,95574\_f\_at,95502\_at |
| 5 | cytoplasm organization and biogenesis | 18 | 380 | 409 | 11544 | 0.044 | 0.033 | 1.337 | 0.129 | 100877\_at,92542\_at,94236\_at,95489\_at,97593\_f\_at,104683\_at,160287\_at,160461\_f\_at,95119\_at,98461\_at,160140\_at,104471\_at,100939\_at,98882\_s\_at,98884\_r\_at,94012\_at,101844\_at,95081\_at |
| 6 | organelle organization and biogenesis | 17 | 318 | 354 | 9498 | 0.048 | 0.033 | 1.434 | 0.086 | 100877\_at,92542\_at,94236\_at,95489\_at,97593\_f\_at,104683\_at,160287\_at,160461\_f\_at,95119\_at,98461\_at,160140\_at,104471\_at,100939\_at,98882\_s\_at,98884\_r\_at,94012\_at,101844\_at |
| 7 | cytoskeleton organization and biogenesis | 15 | 262 | 209 | 6246 | 0.072 | 0.042 | 1.711 | 0.029 | 100877\_at,92542\_at,94236\_at,95489\_at,97593\_f\_at,104683\_at,160287\_at,160461\_f\_at,95119\_at,98461\_at,160140\_at,104471\_at,100939\_at,98882\_s\_at,98884\_r\_at |
| 8 | actin filament-based process | 3 | 42 | 77 | 2164 | 0.039 | 0.019 | 2.007 | 0.186 | 94236\_at,95489\_at,97593\_f\_at |
| 9 | actin cytoskeleton organization and biogenesis | 3 | 42 | 41 | 911 | 0.073 | 0.046 | 1.587 | 0.292 | 94236\_at,95489\_at,97593\_f\_at |
| 8 | microtubule-based process | 10 | 119 | 77 | 2164 | 0.13 | 0.055 | 2.362 | 0.008 | 104683\_at,160287\_at,160461\_f\_at,95119\_at,98461\_at,160140\_at,104471\_at,100939\_at,98882\_s\_at,98884\_r\_at |
| 9 | microtubule cytoskeleton organization and biogenesis | 3 | 12 | 41 | 911 | 0.073 | 0.013 | 5.556 | 0.014 | 104683\_at,160140\_at,104471\_at |
| 10 | microtubule nucleation | 1 | 3 | 14 | 197 | 0.071 | 0.015 | 4.69 | 0.199 | 160140\_at |
| 11 | tubulin folding | 1 | 2 | 5 | 34 | 0.2 | 0.059 | 3.4 | 0.276 | 160140\_at |
| 12 | alpha-tubulin folding | 1 | 1 | 2 | 8 | 0.5 | 0.125 | 4 | 0.25 | 160140\_at |
| 10 | microtubule stabilization | 1 | 1 | 14 | 197 | 0.071 | 0.005 | 14.061 | 0.071 | 104471\_at |
| 9 | microtubule-based movement | 5 | 38 | 41 | 911 | 0.122 | 0.042 | 2.924 | 0.024 | 100939\_at,104683\_at,160461\_f\_at,98882\_s\_at,98884\_r\_at |
| 10 | axon cargo transport | 2 | 4 | 14 | 197 | 0.143 | 0.02 | 7.037 | 0.026 | 98882\_s\_at,98884\_r\_at |
| 11 | retrograde axon cargo transport | 2 | 4 | 5 | 34 | 0.4 | 0.118 | 3.4 | 0.094 | 98882\_s\_at,98884\_r\_at |
| 7 | mitochondrion organization and biogenesis | 1 | 13 | 209 | 6246 | 0.005 | 0.002 | 2.298 | 0.358 | 94012\_at |
| 8 | protein-mitochondrial targeting | 1 | 6 | 77 | 2164 | 0.013 | 0.003 | 4.69 | 0.196 | 94012\_at |
| 9 | mitochondrial translocation | 1 | 6 | 41 | 911 | 0.024 | 0.007 | 3.701 | 0.242 | 94012\_at |
| 7 | peroxisome organization and biogenesis | 1 | 17 | 209 | 6246 | 0.005 | 0.003 | 1.757 | 0.44 | 101844\_at |
| 6 | ribosome biogenesis and assembly | 1 | 60 | 354 | 9498 | 0.003 | 0.006 | 0.446 | 0.898 | 95081\_at |
| 7 | ribosome biogenesis | 1 | 60 | 209 | 6246 | 0.005 | 0.01 | 0.497 | 0.872 | 95081\_at |
| 8 | rRNA processing | 1 | 14 | 77 | 2164 | 0.013 | 0.006 | 2.008 | 0.399 | 95081\_at |
| 5 | nuclear organization and biogenesis | 8 | 112 | 409 | 11544 | 0.02 | 0.01 | 2.016 | 0.045 | 100616\_at,93701\_at,104471\_at,98534\_at,94011\_at,95573\_at,95574\_f\_at,95502\_at |
| 6 | chromosome organization and biogenesis (sensu Eukarya) | 8 | 108 | 354 | 9498 | 0.023 | 0.011 | 1.988 | 0.048 | 100616\_at,93701\_at,104471\_at,98534\_at,94011\_at,95573\_at,95574\_f\_at,95502\_at |
| 7 | establishment and/or maintenance of chromatin architecture | 8 | 80 | 209 | 6246 | 0.038 | 0.013 | 2.988 | 0.005 | 93701\_at,100616\_at,104471\_at,98534\_at,94011\_at,95573\_at,95574\_f\_at,95502\_at |
| 8 | chromatin assembly/disassembly | 2 | 48 | 77 | 2164 | 0.026 | 0.022 | 1.171 | 0.516 | 93701\_at,100616\_at |
| 9 | nucleosome assembly | 1 | 28 | 41 | 911 | 0.024 | 0.031 | 0.793 | 0.73 | 100616\_at |
| 8 | chromatin modification | 7 | 36 | 77 | 2164 | 0.091 | 0.017 | 5.463 | 0 | 104471\_at,98534\_at,93701\_at,94011\_at,95573\_at,95574\_f\_at,95502\_at |
| 9 | non-covalent chromatin modification | 5 | 13 | 41 | 911 | 0.122 | 0.014 | 8.546 | 0 | 93701\_at,94011\_at,95573\_at,95574\_f\_at,95502\_at |
| 10 | chromatin modeling | 5 | 13 | 14 | 197 | 0.357 | 0.066 | 5.412 | 0.001 | 93701\_at,94011\_at,95573\_at,95574\_f\_at,95502\_at |
| 11 | chromatin silencing | 1 | 2 | 5 | 34 | 0.2 | 0.059 | 3.4 | 0.276 | 95502\_at |
| 4 | cell proliferation | 22 | 501 | 482 | 13100 | 0.046 | 0.038 | 1.194 | 0.224 | 100293\_at,100348\_at,101014\_at,101073\_at,93104\_at,94003\_at,94928\_at,94932\_at,96300\_f\_at,102781\_at,160495\_at,99187\_f\_at,99188\_at,95694\_at,95917\_at,104714\_at,94264\_at,94483\_at,96511\_s\_at,104150\_at,94331\_at,160512\_at |
| 5 | cell cycle | 13 | 435 | 409 | 11544 | 0.032 | 0.038 | 0.843 | 0.775 | 102781\_at,160495\_at,94003\_at,99187\_f\_at,99188\_at,95694\_at,95917\_at,100348\_at,104714\_at,94264\_at,94483\_at,94932\_at,96511\_s\_at |
| 6 | DNA replication and chromosome cycle | 1 | 113 | 354 | 9498 | 0.003 | 0.012 | 0.237 | 0.987 | 95694\_at |
| 7 | DNA replication | 1 | 94 | 209 | 6246 | 0.005 | 0.015 | 0.318 | 0.96 | 95694\_at |
| 8 | DNA dependent DNA replication | 1 | 31 | 77 | 2164 | 0.013 | 0.014 | 0.906 | 0.677 | 95694\_at |
| 9 | DNA topological change | 1 | 8 | 41 | 911 | 0.024 | 0.009 | 2.778 | 0.309 | 95694\_at |
| 9 | DNA unwinding | 1 | 4 | 41 | 911 | 0.024 | 0.004 | 5.556 | 0.168 | 95694\_at |
| 6 | M phase | 2 | 74 | 354 | 9498 | 0.006 | 0.008 | 0.725 | 0.769 | 99187\_f\_at,99188\_at |
| 7 | M phase of mitotic cell cycle | 2 | 57 | 209 | 6246 | 0.01 | 0.009 | 1.048 | 0.574 | 99187\_f\_at,99188\_at |
| 8 | mitosis | 2 | 57 | 77 | 2164 | 0.026 | 0.026 | 0.986 | 0.61 | 99187\_f\_at,99188\_at |
| 6 | mitotic cell cycle | 3 | 173 | 354 | 9498 | 0.008 | 0.018 | 0.465 | 0.959 | 95917\_at,99187\_f\_at,99188\_at |
| 7 | M phase of mitotic cell cycle | 2 | 57 | 209 | 6246 | 0.01 | 0.009 | 1.048 | 0.574 | 99187\_f\_at,99188\_at |
| 8 | mitosis | 2 | 57 | 77 | 2164 | 0.026 | 0.026 | 0.986 | 0.61 | 99187\_f\_at,99188\_at |
| 6 | regulation of cell cycle | 8 | 204 | 354 | 9498 | 0.023 | 0.021 | 1.052 | 0.493 | 100348\_at,104714\_at,94264\_at,94483\_at,94932\_at,96511\_s\_at,99187\_f\_at,99188\_at |
| 5 | regulation of cell proliferation | 3 | 38 | 409 | 11544 | 0.007 | 0.003 | 2.228 | 0.151 | 104150\_at,94331\_at,93104\_at |
| 6 | negative regulation of cell proliferation | 1 | 17 | 354 | 9498 | 0.003 | 0.002 | 1.575 | 0.476 | 93104\_at |
| 5 | T-cell proliferation | 1 | 4 | 409 | 11544 | 0.002 | 0 | 6.971 | 0.134 | 160512\_at |
| 4 | transport | 40 | 1083 | 482 | 13100 | 0.083 | 0.083 | 1.004 | 0.515 | 100710\_at,101079\_at,102658\_at,103314\_at,103704\_at,103845\_at,160592\_at,161551\_f\_at,161689\_f\_at,161785\_f\_at,92339\_at,92945\_at,93408\_at,93414\_at,93570\_at,93627\_at,94012\_at,95155\_at,95444\_at,95586\_at,95607\_at,95647\_f\_at,96333\_g\_at,100492\_at,100635\_at,101449\_at,93718\_at,101036\_at,104453\_at,95023\_at,104150\_at,94979\_at,95655\_at,162041\_f\_at,103812\_at,103408\_at,160832\_at,96534\_at,98926\_at,101073\_at |
| 5 | amine/polyamine transport | 1 | 19 | 409 | 11544 | 0.002 | 0.002 | 1.479 | 0.496 | 93570\_at |
| 6 | amino acid transport | 1 | 19 | 354 | 9498 | 0.003 | 0.002 | 1.41 | 0.514 | 93570\_at |
| 5 | intracellular transport | 14 | 351 | 409 | 11544 | 0.034 | 0.03 | 1.126 | 0.363 | 100492\_at,100635\_at,101449\_at,93718\_at,94012\_at,95155\_at,95444\_at,96333\_g\_at,101036\_at,103704\_at,104453\_at,95023\_at,101079\_at,104150\_at |
| 6 | intracellular protein transport | 14 | 284 | 354 | 9498 | 0.04 | 0.03 | 1.323 | 0.175 | 100492\_at,100635\_at,101449\_at,93718\_at,94012\_at,95155\_at,95444\_at,96333\_g\_at,101036\_at,103704\_at,104453\_at,95023\_at,101079\_at,104150\_at |
| 7 | protein targeting | 8 | 101 | 209 | 6246 | 0.038 | 0.016 | 2.367 | 0.019 | 101036\_at,103704\_at,104453\_at,94012\_at,95023\_at,95444\_at,101079\_at,104150\_at |
| 8 | protein-mitochondrial targeting | 1 | 6 | 77 | 2164 | 0.013 | 0.003 | 4.69 | 0.196 | 94012\_at |
| 9 | mitochondrial translocation | 1 | 6 | 41 | 911 | 0.024 | 0.007 | 3.701 | 0.242 | 94012\_at |
| 8 | protein-nucleus import | 2 | 32 | 77 | 2164 | 0.026 | 0.015 | 1.756 | 0.316 | 101079\_at,104150\_at |
| 9 | protein-nucleus import, translocation | 1 | 4 | 41 | 911 | 0.024 | 0.004 | 5.556 | 0.168 | 104150\_at |
| 6 | nucleocytoplasmic transport | 2 | 40 | 354 | 9498 | 0.006 | 0.004 | 1.342 | 0.443 | 101079\_at,104150\_at |
| 7 | RNA-nucleus export | 1 | 7 | 209 | 6246 | 0.005 | 0.001 | 4.268 | 0.212 | 101079\_at |
| 8 | mRNA-nucleus export | 1 | 4 | 77 | 2164 | 0.013 | 0.002 | 7.022 | 0.135 | 101079\_at |
| 5 | ion transport | 9 | 335 | 409 | 11544 | 0.022 | 0.029 | 0.758 | 0.845 | 92945\_at,93570\_at,94979\_at,95586\_at,95655\_at,162041\_f\_at,103812\_at,103845\_at,103408\_at |
| 6 | anion transport | 4 | 79 | 354 | 9498 | 0.011 | 0.008 | 1.358 | 0.34 | 162041\_f\_at,103812\_at,93570\_at,95655\_at |
| 7 | inorganic anion transport | 3 | 50 | 209 | 6246 | 0.014 | 0.008 | 1.792 | 0.234 | 103812\_at,93570\_at,95655\_at |
| 8 | chloride transport | 3 | 39 | 77 | 2164 | 0.039 | 0.018 | 2.162 | 0.159 | 103812\_at,93570\_at,95655\_at |
| 6 | cation transport | 5 | 236 | 354 | 9498 | 0.014 | 0.025 | 0.568 | 0.943 | 94979\_at,103845\_at,103408\_at,92945\_at,93570\_at |
| 7 | di-, tri-valent inorganic cation transport | 3 | 58 | 209 | 6246 | 0.014 | 0.009 | 1.545 | 0.307 | 94979\_at,103845\_at,103408\_at |
| 8 | calcium ion transport | 1 | 33 | 77 | 2164 | 0.013 | 0.015 | 0.852 | 0.7 | 94979\_at |
| 8 | transition metal ion transport | 2 | 25 | 77 | 2164 | 0.026 | 0.012 | 2.248 | 0.223 | 103845\_at,103408\_at |
| 9 | copper ion transport | 1 | 5 | 41 | 911 | 0.024 | 0.005 | 4.443 | 0.206 | 103845\_at |
| 9 | iron ion transport | 1 | 11 | 41 | 911 | 0.024 | 0.012 | 2.021 | 0.399 | 103408\_at |
| 7 | metal ion transport | 5 | 184 | 209 | 6246 | 0.024 | 0.029 | 0.812 | 0.744 | 94979\_at,103845\_at,103408\_at,92945\_at,93570\_at |
| 8 | calcium ion transport | 1 | 33 | 77 | 2164 | 0.013 | 0.015 | 0.852 | 0.7 | 94979\_at |
| 8 | transition metal ion transport | 2 | 25 | 77 | 2164 | 0.026 | 0.012 | 2.248 | 0.223 | 103845\_at,103408\_at |
| 9 | copper ion transport | 1 | 5 | 41 | 911 | 0.024 | 0.005 | 4.443 | 0.206 | 103845\_at |
| 9 | iron ion transport | 1 | 11 | 41 | 911 | 0.024 | 0.012 | 2.021 | 0.399 | 103408\_at |
| 8 | potassium ion transport | 1 | 94 | 77 | 2164 | 0.013 | 0.043 | 0.299 | 0.969 | 92945\_at |
| 8 | sodium ion transport | 1 | 43 | 77 | 2164 | 0.013 | 0.02 | 0.654 | 0.793 | 93570\_at |
| 5 | lipid transport | 5 | 40 | 409 | 11544 | 0.012 | 0.003 | 3.522 | 0.013 | 103314\_at,160832\_at,161785\_f\_at,95607\_at,96534\_at |
| 5 | protein transport | 14 | 297 | 409 | 11544 | 0.034 | 0.026 | 1.33 | 0.17 | 100635\_at,93718\_at,94012\_at,95155\_at,95444\_at,96333\_g\_at,100492\_at,101449\_at,101036\_at,103704\_at,104453\_at,95023\_at,101079\_at,104150\_at |
| 6 | intracellular protein transport | 14 | 284 | 354 | 9498 | 0.04 | 0.03 | 1.323 | 0.175 | 100492\_at,100635\_at,101449\_at,93718\_at,94012\_at,95155\_at,95444\_at,96333\_g\_at,101036\_at,103704\_at,104453\_at,95023\_at,101079\_at,104150\_at |
| 7 | protein targeting | 8 | 101 | 209 | 6246 | 0.038 | 0.016 | 2.367 | 0.019 | 101036\_at,103704\_at,104453\_at,94012\_at,95023\_at,95444\_at,101079\_at,104150\_at |
| 8 | protein-mitochondrial targeting | 1 | 6 | 77 | 2164 | 0.013 | 0.003 | 4.69 | 0.196 | 94012\_at |
| 9 | mitochondrial translocation | 1 | 6 | 41 | 911 | 0.024 | 0.007 | 3.701 | 0.242 | 94012\_at |
| 8 | protein-nucleus import | 2 | 32 | 77 | 2164 | 0.026 | 0.015 | 1.756 | 0.316 | 101079\_at,104150\_at |
| 9 | protein-nucleus import, translocation | 1 | 4 | 41 | 911 | 0.024 | 0.004 | 5.556 | 0.168 | 104150\_at |
| 5 | secretory pathway | 1 | 31 | 409 | 11544 | 0.002 | 0.003 | 0.907 | 0.674 | 98926\_at |
| 6 | exocytosis | 1 | 12 | 354 | 9498 | 0.003 | 0.001 | 2.238 | 0.366 | 98926\_at |
| 7 | calcium ion dependent exocytosis | 1 | 3 | 209 | 6246 | 0.005 | 0 | 9.958 | 0.097 | 98926\_at |
| 7 | regulation of exocytosis | 1 | 2 | 209 | 6246 | 0.005 | 0 | 14.938 | 0.066 | 98926\_at |
| 5 | vesicle-mediated transport | 5 | 112 | 409 | 11544 | 0.012 | 0.01 | 1.26 | 0.365 | 100492\_at,98926\_at,101073\_at,160832\_at,96534\_at |
| 6 | exocytosis | 1 | 12 | 354 | 9498 | 0.003 | 0.001 | 2.238 | 0.366 | 98926\_at |
| 7 | calcium ion dependent exocytosis | 1 | 3 | 209 | 6246 | 0.005 | 0 | 9.958 | 0.097 | 98926\_at |
| 7 | regulation of exocytosis | 1 | 2 | 209 | 6246 | 0.005 | 0 | 14.938 | 0.066 | 98926\_at |
| 6 | endocytosis | 3 | 61 | 354 | 9498 | 0.008 | 0.006 | 1.319 | 0.398 | 101073\_at,160832\_at,96534\_at |
| 3 | cell motility | 6 | 188 | 415 | 10726 | 0.014 | 0.018 | 0.825 | 0.741 | 160512\_at,94236\_at,95489\_at,97593\_f\_at,100921\_at,160151\_i\_at |
| 4 | cell migration | 2 | 53 | 482 | 13100 | 0.004 | 0.004 | 1.025 | 0.586 | 160512\_at,94236\_at |
| 5 | regulation of cell migration | 2 | 17 | 409 | 11544 | 0.005 | 0.001 | 3.327 | 0.12 | 160512\_at,94236\_at |
| 6 | negative regulation of cell migration | 1 | 3 | 354 | 9498 | 0.003 | 0 | 8.812 | 0.108 | 94236\_at |
| 4 | muscle contraction | 4 | 54 | 482 | 13100 | 0.008 | 0.004 | 2.015 | 0.137 | 95489\_at,97593\_f\_at,100921\_at,160151\_i\_at |
| 5 | regulation of muscle contraction | 1 | 19 | 409 | 11544 | 0.002 | 0.002 | 1.479 | 0.496 | 100921\_at |
| 5 | smooth muscle contraction | 1 | 3 | 409 | 11544 | 0.002 | 0 | 9.385 | 0.103 | 160151\_i\_at |
| 2 | development | 36 | 990 | 428 | 10540 | 0.084 | 0.094 | 0.895 | 0.785 | 102580\_r\_at,103518\_at,161112\_at,161530\_r\_at,162204\_r\_at,94341\_at,95382\_at,95387\_f\_at,95489\_at,97118\_at,97497\_at,97593\_f\_at,98862\_at,100348\_at,161610\_at,161667\_r\_at,103259\_at,92986\_g\_at,95917\_at,97509\_f\_at,101186\_at,94003\_at,101778\_at,100134\_at,100877\_at,100921\_at,92542\_at,93718\_at,160140\_at,160512\_at,160762\_at,160099\_at,104263\_at,100429\_at,104340\_at,160834\_at |
| 3 | cell differentiation | 9 | 137 | 415 | 10726 | 0.022 | 0.013 | 1.699 | 0.084 | 100348\_at,102580\_r\_at,161610\_at,161667\_r\_at,162204\_r\_at,97118\_at,97497\_at,103259\_at,92986\_g\_at |
| 4 | epidermal cell differentiation | 1 | 3 | 482 | 13100 | 0.002 | 0 | 9 | 0.106 | 103259\_at |
| 5 | hair cell differentiation | 1 | 3 | 409 | 11544 | 0.002 | 0 | 9.385 | 0.103 | 103259\_at |
| 4 | vasculogenesis | 1 | 7 | 482 | 13100 | 0.002 | 0.001 | 3.906 | 0.231 | 92986\_g\_at |
| 3 | embryonic development | 3 | 52 | 415 | 10726 | 0.007 | 0.005 | 1.491 | 0.327 | 95917\_at,103259\_at,97509\_f\_at |
| 4 | embryonic development (sensu Animalia) | 1 | 18 | 482 | 13100 | 0.002 | 0.001 | 1.511 | 0.491 | 95917\_at |
| 5 | gastrulation | 1 | 12 | 409 | 11544 | 0.002 | 0.001 | 2.346 | 0.351 | 95917\_at |
| 6 | gastrulation (sensu Deuterostoma) | 1 | 3 | 354 | 9498 | 0.003 | 0 | 8.812 | 0.108 | 95917\_at |
| 7 | gastrulation (sensu Mammalia) | 1 | 3 | 209 | 6246 | 0.005 | 0 | 9.958 | 0.097 | 95917\_at |
| 4 | embryonic morphogenesis | 2 | 19 | 482 | 13100 | 0.004 | 0.001 | 2.862 | 0.153 | 103259\_at,97509\_f\_at |
| 5 | ear morphogenesis | 2 | 3 | 409 | 11544 | 0.005 | 0 | 18.808 | 0.004 | 103259\_at,97509\_f\_at |
| 6 | inner ear morphogenesis | 2 | 3 | 354 | 9498 | 0.006 | 0 | 17.656 | 0.004 | 103259\_at,97509\_f\_at |
| 3 | morphogenesis | 21 | 594 | 415 | 10726 | 0.051 | 0.055 | 0.914 | 0.699 | 103259\_at,97509\_f\_at,101186\_at,102580\_r\_at,94003\_at,98862\_at,101778\_at,92986\_g\_at,100134\_at,95382\_at,100877\_at,100921\_at,92542\_at,161530\_r\_at,93718\_at,95387\_f\_at,160140\_at,160512\_at,160762\_at,160099\_at,104263\_at |
| 4 | embryonic morphogenesis | 2 | 19 | 482 | 13100 | 0.004 | 0.001 | 2.862 | 0.153 | 103259\_at,97509\_f\_at |
| 5 | ear morphogenesis | 2 | 3 | 409 | 11544 | 0.005 | 0 | 18.808 | 0.004 | 103259\_at,97509\_f\_at |
| 6 | inner ear morphogenesis | 2 | 3 | 354 | 9498 | 0.006 | 0 | 17.656 | 0.004 | 103259\_at,97509\_f\_at |
| 4 | organogenesis | 21 | 544 | 482 | 13100 | 0.044 | 0.042 | 1.049 | 0.442 | 101186\_at,102580\_r\_at,94003\_at,98862\_at,103259\_at,97509\_f\_at,101778\_at,92986\_g\_at,100134\_at,95382\_at,100877\_at,100921\_at,92542\_at,161530\_r\_at,93718\_at,95387\_f\_at,160140\_at,160512\_at,160762\_at,160099\_at,104263\_at |
| 5 | ear morphogenesis | 2 | 3 | 409 | 11544 | 0.005 | 0 | 18.808 | 0.004 | 103259\_at,97509\_f\_at |
| 6 | inner ear morphogenesis | 2 | 3 | 354 | 9498 | 0.006 | 0 | 17.656 | 0.004 | 103259\_at,97509\_f\_at |
| 5 | blood vessel development | 3 | 61 | 409 | 11544 | 0.007 | 0.005 | 1.388 | 0.368 | 101778\_at,92986\_g\_at,100134\_at |
| 6 | angiogenesis | 1 | 50 | 354 | 9498 | 0.003 | 0.005 | 0.536 | 0.851 | 100134\_at |
| 5 | heart development | 2 | 31 | 409 | 11544 | 0.005 | 0.003 | 1.818 | 0.301 | 100134\_at,92986\_g\_at |
| 5 | lymph gland development | 1 | 20 | 409 | 11544 | 0.002 | 0.002 | 1.41 | 0.514 | 95382\_at |
| 5 | midgut development | 1 | 1 | 409 | 11544 | 0.002 | 0 | 27.111 | 0.035 | 101186\_at |
| 6 | visceral mesoderm/endoderm interaction | 1 | 1 | 354 | 9498 | 0.003 | 0 | 25.636 | 0.037 | 101186\_at |
| 5 | muscle development | 3 | 75 | 409 | 11544 | 0.007 | 0.006 | 1.128 | 0.499 | 100877\_at,100921\_at,92542\_at |
| 5 | neurogenesis | 9 | 164 | 409 | 11544 | 0.022 | 0.014 | 1.548 | 0.128 | 102580\_r\_at,161530\_r\_at,93718\_at,95387\_f\_at,160140\_at,160512\_at,160762\_at,97509\_f\_at,160099\_at |
| 6 | axonogenesis | 1 | 34 | 354 | 9498 | 0.003 | 0.004 | 0.788 | 0.726 | 160140\_at |
| 6 | central nervous system development | 3 | 39 | 354 | 9498 | 0.008 | 0.004 | 2.061 | 0.177 | 160512\_at,160762\_at,97509\_f\_at |
| 7 | brain development | 3 | 29 | 209 | 6246 | 0.014 | 0.005 | 3.093 | 0.071 | 160512\_at,160762\_at,97509\_f\_at |
| 6 | peripheral nervous system development | 1 | 24 | 354 | 9498 | 0.003 | 0.003 | 1.115 | 0.599 | 160099\_at |
| 7 | sensory organ development | 1 | 16 | 209 | 6246 | 0.005 | 0.003 | 1.867 | 0.42 | 160099\_at |
| 5 | salivary gland development | 1 | 10 | 409 | 11544 | 0.002 | 0.001 | 2.805 | 0.303 | 97509\_f\_at |
| 6 | salivary gland morphogenesis | 1 | 7 | 354 | 9498 | 0.003 | 0.001 | 3.811 | 0.234 | 97509\_f\_at |
| 5 | skeletal development | 1 | 52 | 409 | 11544 | 0.002 | 0.004 | 0.542 | 0.847 | 104263\_at |
| 6 | ossification | 1 | 25 | 354 | 9498 | 0.003 | 0.003 | 1.072 | 0.614 | 104263\_at |
| 7 | bone mineralization | 1 | 4 | 209 | 6246 | 0.005 | 0.001 | 7.469 | 0.127 | 104263\_at |
| 4 | vasculogenesis | 1 | 7 | 482 | 13100 | 0.002 | 0.001 | 3.906 | 0.231 | 92986\_g\_at |
| 3 | pattern specification | 4 | 83 | 415 | 10726 | 0.01 | 0.008 | 1.245 | 0.401 | 161112\_at,95917\_at,162204\_r\_at,97497\_at |
| 4 | anterior/posterior pattern formation | 1 | 10 | 482 | 13100 | 0.002 | 0.001 | 2.724 | 0.313 | 95917\_at |
| 5 | anterior/posterior axis specification | 1 | 3 | 409 | 11544 | 0.002 | 0 | 9.385 | 0.103 | 95917\_at |
| 4 | compartment specification | 2 | 8 | 482 | 13100 | 0.004 | 0.001 | 6.803 | 0.033 | 162204\_r\_at,97497\_at |
| 3 | pigmentation | 1 | 23 | 415 | 10726 | 0.002 | 0.002 | 1.126 | 0.597 | 100429\_at |
| 4 | pigment metabolism | 1 | 23 | 482 | 13100 | 0.002 | 0.002 | 1.176 | 0.578 | 100429\_at |
| 5 | heme metabolism | 1 | 15 | 409 | 11544 | 0.002 | 0.001 | 1.877 | 0.418 | 100429\_at |
| 6 | heme biosynthesis | 1 | 11 | 354 | 9498 | 0.003 | 0.001 | 2.431 | 0.342 | 100429\_at |
| 3 | regulation of gene expression, epigenetic | 2 | 28 | 415 | 10726 | 0.005 | 0.003 | 1.847 | 0.295 | 104340\_at,160834\_at |
| 4 | DNA methylation | 2 | 21 | 482 | 13100 | 0.004 | 0.002 | 2.594 | 0.18 | 104340\_at,160834\_at |
| 3 | reproduction | 1 | 99 | 415 | 10726 | 0.002 | 0.009 | 0.261 | 0.98 | 102580\_r\_at |
| 4 | sexual reproduction | 1 | 99 | 482 | 13100 | 0.002 | 0.008 | 0.274 | 0.976 | 102580\_r\_at |
| 5 | gametogenesis | 1 | 86 | 409 | 11544 | 0.002 | 0.007 | 0.328 | 0.956 | 102580\_r\_at |
| 6 | male gamete generation | 1 | NA | 354 | 9498 | 0.003 | NA | NA | NA | 102580\_r\_at |
| 7 | spermatogenesis | 1 | 66 | 209 | 6246 | 0.005 | 0.011 | 0.452 | 0.895 | 102580\_r\_at |
| 2 | obsolete biological process | 2 | 3 | 428 | 10540 | 0.005 | 0 | 16.679 | 0.005 | 160182\_at,93520\_at |
| 3 | mRNA splicing | 2 | 54 | 415 | 10726 | 0.005 | 0.005 | 0.958 | 0.624 | 160182\_at,93520\_at |
| 2 | physiological processes | 239 | 5866 | 428 | 10540 | 0.558 | 0.557 | 1.003 | 0.489 | 100710\_at,101697\_f\_at,104533\_at,104714\_at,161551\_f\_at,92400\_at,93104\_at,94264\_at,94397\_at,94932\_at,96056\_at,96511\_s\_at,162206\_f\_at,92232\_at,104263\_at,100606\_at,103408\_at,100877\_at,92542\_at,94236\_at,95489\_at,97593\_f\_at,104683\_at,160287\_at,160461\_f\_at,95119\_at,98461\_at,160140\_at,104471\_at,100939\_at,98882\_s\_at,98884\_r\_at,94012\_at,101844\_at,95081\_at,100616\_at,93701\_at,98534\_at,94011\_at,95573\_at,95574\_f\_at,95502\_at,100293\_at,100348\_at,101014\_at,101073\_at,94003\_at,94928\_at,96300\_f\_at,102781\_at,160495\_at,99187\_f\_at,99188\_at,95694\_at,95917\_at,94483\_at,104150\_at,94331\_at,160512\_at,101079\_at,102658\_at,103314\_at,103704\_at,103845\_at,160592\_at,161689\_f\_at,161785\_f\_at,92339\_at,92945\_at,93408\_at,93414\_at,93570\_at,93627\_at,95155\_at,95444\_at,95586\_at,95607\_at,95647\_f\_at,96333\_g\_at,100492\_at,100635\_at,101449\_at,93718\_at,101036\_at,104453\_at,95023\_at,94979\_at,95655\_at,162041\_f\_at,103812\_at,160832\_at,96534\_at,98926\_at,93193\_at,95355\_at,92991\_at,92992\_i\_at,92993\_r\_at,92918\_at,94192\_at,98018\_at,101432\_at,102787\_at,102991\_s\_at,96650\_at,99617\_at,100429\_at,99045\_at,162092\_f\_at,104217\_at,104285\_at,99184\_at,97386\_at,102313\_at,101186\_at,160425\_at,102101\_f\_at,104048\_at,104144\_at,160283\_at,160977\_at,161667\_r\_at,93975\_at,94484\_at,95501\_at,96577\_i\_at,96578\_r\_at,96845\_at,97083\_at,100136\_at,96784\_at,93315\_at,94818\_at,97936\_at,102980\_at,93852\_at,98435\_at,162228\_f\_at,103562\_f\_at,104677\_at,161039\_at,92634\_at,93509\_at,95564\_at,96176\_at,99085\_at,99086\_g\_at,160088\_at,161005\_at,162260\_at,93424\_at,93440\_at,94951\_at,98533\_at,99985\_at,104165\_at,97897\_at,160174\_at,100030\_at,104219\_f\_at,160182\_at,103545\_at,101889\_s\_at,101943\_at,95536\_at,104701\_at,100094\_at,100486\_at,102024\_at,102242\_at,102382\_at,102580\_r\_at,102895\_at,103015\_at,103259\_at,103504\_at,160220\_at,160396\_at,160781\_r\_at,160834\_at,161113\_at,161187\_f\_at,161333\_f\_at,162114\_f\_at,162204\_r\_at,92233\_at,92300\_at,92737\_at,94689\_at,95521\_s\_at,96196\_i\_at,96197\_f\_at,96481\_at,96817\_at,97118\_at,97497\_at,98002\_at,98465\_f\_at,98767\_at,98818\_at,99100\_at,99103\_at,99665\_at,101836\_at,92758\_at,92986\_g\_at,94980\_at,102063\_at,104364\_at,160698\_s\_at,93274\_at,93311\_at,95295\_s\_at,97384\_at,97429\_at,97509\_f\_at,99458\_i\_at,160742\_at,102279\_at,103582\_r\_at,161396\_f\_at,161112\_at,104388\_at,104572\_at,93199\_at,160099\_at,161745\_f\_at,92571\_at,103422\_at,104692\_at,93909\_f\_at,98000\_at,100973\_i\_at,103080\_at,93321\_at,94224\_s\_at,97710\_f\_at,100333\_at,103033\_at |
| 3 | cell growth and/or maintenance | 93 | 2128 | 415 | 10726 | 0.224 | 0.198 | 1.13 | 0.102 | 100710\_at,101697\_f\_at,104533\_at,104714\_at,161551\_f\_at,92400\_at,93104\_at,94264\_at,94397\_at,94932\_at,96056\_at,96511\_s\_at,162206\_f\_at,92232\_at,104263\_at,100606\_at,103408\_at,100877\_at,92542\_at,94236\_at,95489\_at,97593\_f\_at,104683\_at,160287\_at,160461\_f\_at,95119\_at,98461\_at,160140\_at,104471\_at,100939\_at,98882\_s\_at,98884\_r\_at,94012\_at,101844\_at,95081\_at,100616\_at,93701\_at,98534\_at,94011\_at,95573\_at,95574\_f\_at,95502\_at,100293\_at,100348\_at,101014\_at,101073\_at,94003\_at,94928\_at,96300\_f\_at,102781\_at,160495\_at,99187\_f\_at,99188\_at,95694\_at,95917\_at,94483\_at,104150\_at,94331\_at,160512\_at,101079\_at,102658\_at,103314\_at,103704\_at,103845\_at,160592\_at,161689\_f\_at,161785\_f\_at,92339\_at,92945\_at,93408\_at,93414\_at,93570\_at,93627\_at,95155\_at,95444\_at,95586\_at,95607\_at,95647\_f\_at,96333\_g\_at,100492\_at,100635\_at,101449\_at,93718\_at,101036\_at,104453\_at,95023\_at,94979\_at,95655\_at,162041\_f\_at,103812\_at,160832\_at,96534\_at,98926\_at |
| 4 | cell growth | 2 | 51 | 482 | 13100 | 0.004 | 0.004 | 1.067 | 0.565 | 162206\_f\_at,92232\_at |
| 5 | regulation of cell growth | 2 | 38 | 409 | 11544 | 0.005 | 0.003 | 1.486 | 0.392 | 162206\_f\_at,92232\_at |
| 4 | cell homeostasis | 3 | 41 | 482 | 13100 | 0.006 | 0.003 | 1.987 | 0.191 | 104263\_at,100606\_at,103408\_at |
| 5 | cell ion homeostasis | 3 | 37 | 409 | 11544 | 0.007 | 0.003 | 2.283 | 0.142 | 104263\_at,100606\_at,103408\_at |
| 6 | cation homeostasis | 3 | 36 | 354 | 9498 | 0.008 | 0.004 | 2.235 | 0.149 | 104263\_at,100606\_at,103408\_at |
| 7 | di-, tri-valent inorganic cation homeostasis | 3 | 29 | 209 | 6246 | 0.014 | 0.005 | 3.093 | 0.071 | 104263\_at,100606\_at,103408\_at |
| 8 | calcium ion homeostasis | 1 | 13 | 77 | 2164 | 0.013 | 0.006 | 2.161 | 0.376 | 104263\_at |
| 8 | copper ion homeostasis | 1 | 1 | 77 | 2164 | 0.013 | 0 | 28.239 | 0.036 | 100606\_at |
| 8 | iron ion homeostasis | 1 | 15 | 77 | 2164 | 0.013 | 0.007 | 1.874 | 0.42 | 103408\_at |
| 4 | cell organization and biogenesis | 25 | 530 | 482 | 13100 | 0.052 | 0.04 | 1.282 | 0.121 | 100877\_at,92542\_at,94236\_at,95489\_at,97593\_f\_at,104683\_at,160287\_at,160461\_f\_at,95119\_at,98461\_at,160140\_at,104471\_at,100939\_at,98882\_s\_at,98884\_r\_at,94012\_at,101844\_at,95081\_at,100616\_at,93701\_at,98534\_at,94011\_at,95573\_at,95574\_f\_at,95502\_at |
| 5 | cytoplasm organization and biogenesis | 18 | 380 | 409 | 11544 | 0.044 | 0.033 | 1.337 | 0.129 | 100877\_at,92542\_at,94236\_at,95489\_at,97593\_f\_at,104683\_at,160287\_at,160461\_f\_at,95119\_at,98461\_at,160140\_at,104471\_at,100939\_at,98882\_s\_at,98884\_r\_at,94012\_at,101844\_at,95081\_at |
| 6 | organelle organization and biogenesis | 17 | 318 | 354 | 9498 | 0.048 | 0.033 | 1.434 | 0.086 | 100877\_at,92542\_at,94236\_at,95489\_at,97593\_f\_at,104683\_at,160287\_at,160461\_f\_at,95119\_at,98461\_at,160140\_at,104471\_at,100939\_at,98882\_s\_at,98884\_r\_at,94012\_at,101844\_at |
| 7 | cytoskeleton organization and biogenesis | 15 | 262 | 209 | 6246 | 0.072 | 0.042 | 1.711 | 0.029 | 100877\_at,92542\_at,94236\_at,95489\_at,97593\_f\_at,104683\_at,160287\_at,160461\_f\_at,95119\_at,98461\_at,160140\_at,104471\_at,100939\_at,98882\_s\_at,98884\_r\_at |
| 8 | actin filament-based process | 3 | 42 | 77 | 2164 | 0.039 | 0.019 | 2.007 | 0.186 | 94236\_at,95489\_at,97593\_f\_at |
| 9 | actin cytoskeleton organization and biogenesis | 3 | 42 | 41 | 911 | 0.073 | 0.046 | 1.587 | 0.292 | 94236\_at,95489\_at,97593\_f\_at |
| 8 | microtubule-based process | 10 | 119 | 77 | 2164 | 0.13 | 0.055 | 2.362 | 0.008 | 104683\_at,160287\_at,160461\_f\_at,95119\_at,98461\_at,160140\_at,104471\_at,100939\_at,98882\_s\_at,98884\_r\_at |
| 9 | microtubule cytoskeleton organization and biogenesis | 3 | 12 | 41 | 911 | 0.073 | 0.013 | 5.556 | 0.014 | 104683\_at,160140\_at,104471\_at |
| 10 | microtubule nucleation | 1 | 3 | 14 | 197 | 0.071 | 0.015 | 4.69 | 0.199 | 160140\_at |
| 11 | tubulin folding | 1 | 2 | 5 | 34 | 0.2 | 0.059 | 3.4 | 0.276 | 160140\_at |
| 12 | alpha-tubulin folding | 1 | 1 | 2 | 8 | 0.5 | 0.125 | 4 | 0.25 | 160140\_at |
| 10 | microtubule stabilization | 1 | 1 | 14 | 197 | 0.071 | 0.005 | 14.061 | 0.071 | 104471\_at |
| 9 | microtubule-based movement | 5 | 38 | 41 | 911 | 0.122 | 0.042 | 2.924 | 0.024 | 100939\_at,104683\_at,160461\_f\_at,98882\_s\_at,98884\_r\_at |
| 10 | axon cargo transport | 2 | 4 | 14 | 197 | 0.143 | 0.02 | 7.037 | 0.026 | 98882\_s\_at,98884\_r\_at |
| 11 | retrograde axon cargo transport | 2 | 4 | 5 | 34 | 0.4 | 0.118 | 3.4 | 0.094 | 98882\_s\_at,98884\_r\_at |
| 7 | mitochondrion organization and biogenesis | 1 | 13 | 209 | 6246 | 0.005 | 0.002 | 2.298 | 0.358 | 94012\_at |
| 8 | protein-mitochondrial targeting | 1 | 6 | 77 | 2164 | 0.013 | 0.003 | 4.69 | 0.196 | 94012\_at |
| 9 | mitochondrial translocation | 1 | 6 | 41 | 911 | 0.024 | 0.007 | 3.701 | 0.242 | 94012\_at |
| 7 | peroxisome organization and biogenesis | 1 | 17 | 209 | 6246 | 0.005 | 0.003 | 1.757 | 0.44 | 101844\_at |
| 6 | ribosome biogenesis and assembly | 1 | 60 | 354 | 9498 | 0.003 | 0.006 | 0.446 | 0.898 | 95081\_at |
| 7 | ribosome biogenesis | 1 | 60 | 209 | 6246 | 0.005 | 0.01 | 0.497 | 0.872 | 95081\_at |
| 8 | rRNA processing | 1 | 14 | 77 | 2164 | 0.013 | 0.006 | 2.008 | 0.399 | 95081\_at |
| 5 | nuclear organization and biogenesis | 8 | 112 | 409 | 11544 | 0.02 | 0.01 | 2.016 | 0.045 | 100616\_at,93701\_at,104471\_at,98534\_at,94011\_at,95573\_at,95574\_f\_at,95502\_at |
| 6 | chromosome organization and biogenesis (sensu Eukarya) | 8 | 108 | 354 | 9498 | 0.023 | 0.011 | 1.988 | 0.048 | 100616\_at,93701\_at,104471\_at,98534\_at,94011\_at,95573\_at,95574\_f\_at,95502\_at |
| 7 | establishment and/or maintenance of chromatin architecture | 8 | 80 | 209 | 6246 | 0.038 | 0.013 | 2.988 | 0.005 | 93701\_at,100616\_at,104471\_at,98534\_at,94011\_at,95573\_at,95574\_f\_at,95502\_at |
| 8 | chromatin assembly/disassembly | 2 | 48 | 77 | 2164 | 0.026 | 0.022 | 1.171 | 0.516 | 93701\_at,100616\_at |
| 9 | nucleosome assembly | 1 | 28 | 41 | 911 | 0.024 | 0.031 | 0.793 | 0.73 | 100616\_at |
| 8 | chromatin modification | 7 | 36 | 77 | 2164 | 0.091 | 0.017 | 5.463 | 0 | 104471\_at,98534\_at,93701\_at,94011\_at,95573\_at,95574\_f\_at,95502\_at |
| 9 | non-covalent chromatin modification | 5 | 13 | 41 | 911 | 0.122 | 0.014 | 8.546 | 0 | 93701\_at,94011\_at,95573\_at,95574\_f\_at,95502\_at |
| 10 | chromatin modeling | 5 | 13 | 14 | 197 | 0.357 | 0.066 | 5.412 | 0.001 | 93701\_at,94011\_at,95573\_at,95574\_f\_at,95502\_at |
| 11 | chromatin silencing | 1 | 2 | 5 | 34 | 0.2 | 0.059 | 3.4 | 0.276 | 95502\_at |
| 4 | cell proliferation | 22 | 501 | 482 | 13100 | 0.046 | 0.038 | 1.194 | 0.224 | 100293\_at,100348\_at,101014\_at,101073\_at,93104\_at,94003\_at,94928\_at,94932\_at,96300\_f\_at,102781\_at,160495\_at,99187\_f\_at,99188\_at,95694\_at,95917\_at,104714\_at,94264\_at,94483\_at,96511\_s\_at,104150\_at,94331\_at,160512\_at |
| 5 | cell cycle | 13 | 435 | 409 | 11544 | 0.032 | 0.038 | 0.843 | 0.775 | 102781\_at,160495\_at,94003\_at,99187\_f\_at,99188\_at,95694\_at,95917\_at,100348\_at,104714\_at,94264\_at,94483\_at,94932\_at,96511\_s\_at |
| 6 | DNA replication and chromosome cycle | 1 | 113 | 354 | 9498 | 0.003 | 0.012 | 0.237 | 0.987 | 95694\_at |
| 7 | DNA replication | 1 | 94 | 209 | 6246 | 0.005 | 0.015 | 0.318 | 0.96 | 95694\_at |
| 8 | DNA dependent DNA replication | 1 | 31 | 77 | 2164 | 0.013 | 0.014 | 0.906 | 0.677 | 95694\_at |
| 9 | DNA topological change | 1 | 8 | 41 | 911 | 0.024 | 0.009 | 2.778 | 0.309 | 95694\_at |
| 9 | DNA unwinding | 1 | 4 | 41 | 911 | 0.024 | 0.004 | 5.556 | 0.168 | 95694\_at |
| 6 | M phase | 2 | 74 | 354 | 9498 | 0.006 | 0.008 | 0.725 | 0.769 | 99187\_f\_at,99188\_at |
| 7 | M phase of mitotic cell cycle | 2 | 57 | 209 | 6246 | 0.01 | 0.009 | 1.048 | 0.574 | 99187\_f\_at,99188\_at |
| 8 | mitosis | 2 | 57 | 77 | 2164 | 0.026 | 0.026 | 0.986 | 0.61 | 99187\_f\_at,99188\_at |
| 6 | mitotic cell cycle | 3 | 173 | 354 | 9498 | 0.008 | 0.018 | 0.465 | 0.959 | 95917\_at,99187\_f\_at,99188\_at |
| 7 | M phase of mitotic cell cycle | 2 | 57 | 209 | 6246 | 0.01 | 0.009 | 1.048 | 0.574 | 99187\_f\_at,99188\_at |
| 8 | mitosis | 2 | 57 | 77 | 2164 | 0.026 | 0.026 | 0.986 | 0.61 | 99187\_f\_at,99188\_at |
| 6 | regulation of cell cycle | 8 | 204 | 354 | 9498 | 0.023 | 0.021 | 1.052 | 0.493 | 100348\_at,104714\_at,94264\_at,94483\_at,94932\_at,96511\_s\_at,99187\_f\_at,99188\_at |
| 5 | regulation of cell proliferation | 3 | 38 | 409 | 11544 | 0.007 | 0.003 | 2.228 | 0.151 | 104150\_at,94331\_at,93104\_at |
| 6 | negative regulation of cell proliferation | 1 | 17 | 354 | 9498 | 0.003 | 0.002 | 1.575 | 0.476 | 93104\_at |
| 5 | T-cell proliferation | 1 | 4 | 409 | 11544 | 0.002 | 0 | 6.971 | 0.134 | 160512\_at |
| 4 | transport | 40 | 1083 | 482 | 13100 | 0.083 | 0.083 | 1.004 | 0.515 | 100710\_at,101079\_at,102658\_at,103314\_at,103704\_at,103845\_at,160592\_at,161551\_f\_at,161689\_f\_at,161785\_f\_at,92339\_at,92945\_at,93408\_at,93414\_at,93570\_at,93627\_at,94012\_at,95155\_at,95444\_at,95586\_at,95607\_at,95647\_f\_at,96333\_g\_at,100492\_at,100635\_at,101449\_at,93718\_at,101036\_at,104453\_at,95023\_at,104150\_at,94979\_at,95655\_at,162041\_f\_at,103812\_at,103408\_at,160832\_at,96534\_at,98926\_at,101073\_at |
| 5 | amine/polyamine transport | 1 | 19 | 409 | 11544 | 0.002 | 0.002 | 1.479 | 0.496 | 93570\_at |
| 6 | amino acid transport | 1 | 19 | 354 | 9498 | 0.003 | 0.002 | 1.41 | 0.514 | 93570\_at |
| 5 | intracellular transport | 14 | 351 | 409 | 11544 | 0.034 | 0.03 | 1.126 | 0.363 | 100492\_at,100635\_at,101449\_at,93718\_at,94012\_at,95155\_at,95444\_at,96333\_g\_at,101036\_at,103704\_at,104453\_at,95023\_at,101079\_at,104150\_at |
| 6 | intracellular protein transport | 14 | 284 | 354 | 9498 | 0.04 | 0.03 | 1.323 | 0.175 | 100492\_at,100635\_at,101449\_at,93718\_at,94012\_at,95155\_at,95444\_at,96333\_g\_at,101036\_at,103704\_at,104453\_at,95023\_at,101079\_at,104150\_at |
| 7 | protein targeting | 8 | 101 | 209 | 6246 | 0.038 | 0.016 | 2.367 | 0.019 | 101036\_at,103704\_at,104453\_at,94012\_at,95023\_at,95444\_at,101079\_at,104150\_at |
| 8 | protein-mitochondrial targeting | 1 | 6 | 77 | 2164 | 0.013 | 0.003 | 4.69 | 0.196 | 94012\_at |
| 9 | mitochondrial translocation | 1 | 6 | 41 | 911 | 0.024 | 0.007 | 3.701 | 0.242 | 94012\_at |
| 8 | protein-nucleus import | 2 | 32 | 77 | 2164 | 0.026 | 0.015 | 1.756 | 0.316 | 101079\_at,104150\_at |
| 9 | protein-nucleus import, translocation | 1 | 4 | 41 | 911 | 0.024 | 0.004 | 5.556 | 0.168 | 104150\_at |
| 6 | nucleocytoplasmic transport | 2 | 40 | 354 | 9498 | 0.006 | 0.004 | 1.342 | 0.443 | 101079\_at,104150\_at |
| 7 | RNA-nucleus export | 1 | 7 | 209 | 6246 | 0.005 | 0.001 | 4.268 | 0.212 | 101079\_at |
| 8 | mRNA-nucleus export | 1 | 4 | 77 | 2164 | 0.013 | 0.002 | 7.022 | 0.135 | 101079\_at |
| 5 | ion transport | 9 | 335 | 409 | 11544 | 0.022 | 0.029 | 0.758 | 0.845 | 92945\_at,93570\_at,94979\_at,95586\_at,95655\_at,162041\_f\_at,103812\_at,103845\_at,103408\_at |
| 6 | anion transport | 4 | 79 | 354 | 9498 | 0.011 | 0.008 | 1.358 | 0.34 | 162041\_f\_at,103812\_at,93570\_at,95655\_at |
| 7 | inorganic anion transport | 3 | 50 | 209 | 6246 | 0.014 | 0.008 | 1.792 | 0.234 | 103812\_at,93570\_at,95655\_at |
| 8 | chloride transport | 3 | 39 | 77 | 2164 | 0.039 | 0.018 | 2.162 | 0.159 | 103812\_at,93570\_at,95655\_at |
| 6 | cation transport | 5 | 236 | 354 | 9498 | 0.014 | 0.025 | 0.568 | 0.943 | 94979\_at,103845\_at,103408\_at,92945\_at,93570\_at |
| 7 | di-, tri-valent inorganic cation transport | 3 | 58 | 209 | 6246 | 0.014 | 0.009 | 1.545 | 0.307 | 94979\_at,103845\_at,103408\_at |
| 8 | calcium ion transport | 1 | 33 | 77 | 2164 | 0.013 | 0.015 | 0.852 | 0.7 | 94979\_at |
| 8 | transition metal ion transport | 2 | 25 | 77 | 2164 | 0.026 | 0.012 | 2.248 | 0.223 | 103845\_at,103408\_at |
| 9 | copper ion transport | 1 | 5 | 41 | 911 | 0.024 | 0.005 | 4.443 | 0.206 | 103845\_at |
| 9 | iron ion transport | 1 | 11 | 41 | 911 | 0.024 | 0.012 | 2.021 | 0.399 | 103408\_at |
| 7 | metal ion transport | 5 | 184 | 209 | 6246 | 0.024 | 0.029 | 0.812 | 0.744 | 94979\_at,103845\_at,103408\_at,92945\_at,93570\_at |
| 8 | calcium ion transport | 1 | 33 | 77 | 2164 | 0.013 | 0.015 | 0.852 | 0.7 | 94979\_at |
| 8 | transition metal ion transport | 2 | 25 | 77 | 2164 | 0.026 | 0.012 | 2.248 | 0.223 | 103845\_at,103408\_at |
| 9 | copper ion transport | 1 | 5 | 41 | 911 | 0.024 | 0.005 | 4.443 | 0.206 | 103845\_at |
| 9 | iron ion transport | 1 | 11 | 41 | 911 | 0.024 | 0.012 | 2.021 | 0.399 | 103408\_at |
| 8 | potassium ion transport | 1 | 94 | 77 | 2164 | 0.013 | 0.043 | 0.299 | 0.969 | 92945\_at |
| 8 | sodium ion transport | 1 | 43 | 77 | 2164 | 0.013 | 0.02 | 0.654 | 0.793 | 93570\_at |
| 5 | lipid transport | 5 | 40 | 409 | 11544 | 0.012 | 0.003 | 3.522 | 0.013 | 103314\_at,160832\_at,161785\_f\_at,95607\_at,96534\_at |
| 5 | protein transport | 14 | 297 | 409 | 11544 | 0.034 | 0.026 | 1.33 | 0.17 | 100635\_at,93718\_at,94012\_at,95155\_at,95444\_at,96333\_g\_at,100492\_at,101449\_at,101036\_at,103704\_at,104453\_at,95023\_at,101079\_at,104150\_at |
| 6 | intracellular protein transport | 14 | 284 | 354 | 9498 | 0.04 | 0.03 | 1.323 | 0.175 | 100492\_at,100635\_at,101449\_at,93718\_at,94012\_at,95155\_at,95444\_at,96333\_g\_at,101036\_at,103704\_at,104453\_at,95023\_at,101079\_at,104150\_at |
| 7 | protein targeting | 8 | 101 | 209 | 6246 | 0.038 | 0.016 | 2.367 | 0.019 | 101036\_at,103704\_at,104453\_at,94012\_at,95023\_at,95444\_at,101079\_at,104150\_at |
| 8 | protein-mitochondrial targeting | 1 | 6 | 77 | 2164 | 0.013 | 0.003 | 4.69 | 0.196 | 94012\_at |
| 9 | mitochondrial translocation | 1 | 6 | 41 | 911 | 0.024 | 0.007 | 3.701 | 0.242 | 94012\_at |
| 8 | protein-nucleus import | 2 | 32 | 77 | 2164 | 0.026 | 0.015 | 1.756 | 0.316 | 101079\_at,104150\_at |
| 9 | protein-nucleus import, translocation | 1 | 4 | 41 | 911 | 0.024 | 0.004 | 5.556 | 0.168 | 104150\_at |
| 5 | secretory pathway | 1 | 31 | 409 | 11544 | 0.002 | 0.003 | 0.907 | 0.674 | 98926\_at |
| 6 | exocytosis | 1 | 12 | 354 | 9498 | 0.003 | 0.001 | 2.238 | 0.366 | 98926\_at |
| 7 | calcium ion dependent exocytosis | 1 | 3 | 209 | 6246 | 0.005 | 0 | 9.958 | 0.097 | 98926\_at |
| 7 | regulation of exocytosis | 1 | 2 | 209 | 6246 | 0.005 | 0 | 14.938 | 0.066 | 98926\_at |
| 5 | vesicle-mediated transport | 5 | 112 | 409 | 11544 | 0.012 | 0.01 | 1.26 | 0.365 | 100492\_at,98926\_at,101073\_at,160832\_at,96534\_at |
| 6 | exocytosis | 1 | 12 | 354 | 9498 | 0.003 | 0.001 | 2.238 | 0.366 | 98926\_at |
| 7 | calcium ion dependent exocytosis | 1 | 3 | 209 | 6246 | 0.005 | 0 | 9.958 | 0.097 | 98926\_at |
| 7 | regulation of exocytosis | 1 | 2 | 209 | 6246 | 0.005 | 0 | 14.938 | 0.066 | 98926\_at |
| 6 | endocytosis | 3 | 61 | 354 | 9498 | 0.008 | 0.006 | 1.319 | 0.398 | 101073\_at,160832\_at,96534\_at |
| 3 | circulation | 5 | 40 | 415 | 10726 | 0.012 | 0.004 | 3.231 | 0.018 | 93193\_at,95355\_at,92991\_at,92992\_i\_at,92993\_r\_at |
| 4 | regulation of blood pressure | 2 | 18 | 482 | 13100 | 0.004 | 0.001 | 3.029 | 0.14 | 93193\_at,95355\_at |
| 4 | regulation of heart rate | 3 | 19 | 482 | 13100 | 0.006 | 0.001 | 4.29 | 0.031 | 92991\_at,92992\_i\_at,92993\_r\_at |
| 3 | hemostasis | 3 | 44 | 415 | 10726 | 0.007 | 0.004 | 1.763 | 0.242 | 92918\_at,94192\_at,98018\_at |
| 4 | blood coagulation | 3 | 42 | 482 | 13100 | 0.006 | 0.003 | 1.938 | 0.2 | 92918\_at,94192\_at,98018\_at |
| 3 | lactation | 1 | 2 | 415 | 10726 | 0.002 | 0 | 12.684 | 0.076 | 104263\_at |
| 3 | metabolism | 164 | 3908 | 415 | 10726 | 0.395 | 0.364 | 1.085 | 0.101 | 101432\_at,102787\_at,102991\_s\_at,92918\_at,96650\_at,99617\_at,100429\_at,99045\_at,162092\_f\_at,104217\_at,160832\_at,96534\_at,104285\_at,99184\_at,97386\_at,92400\_at,102313\_at,101844\_at,101186\_at,161785\_f\_at,95607\_at,160425\_at,101697\_f\_at,102101\_f\_at,104048\_at,104144\_at,160283\_at,160977\_at,161667\_r\_at,93975\_at,94484\_at,95501\_at,96300\_f\_at,96577\_i\_at,96578\_r\_at,96845\_at,97083\_at,100136\_at,96784\_at,93315\_at,94818\_at,97936\_at,102980\_at,93852\_at,98435\_at,162228\_f\_at,103562\_f\_at,104677\_at,161039\_at,92634\_at,104471\_at,93509\_at,95564\_at,96176\_at,99085\_at,99086\_g\_at,160088\_at,161005\_at,162260\_at,93424\_at,93440\_at,94951\_at,98533\_at,99985\_at,101073\_at,95647\_f\_at,103314\_at,104165\_at,100606\_at,95694\_at,97897\_at,93701\_at,100616\_at,98534\_at,94011\_at,95573\_at,95574\_f\_at,95502\_at,160174\_at,100030\_at,104219\_f\_at,95081\_at,101079\_at,160182\_at,103545\_at,101889\_s\_at,101943\_at,95536\_at,104701\_at,100094\_at,100486\_at,100939\_at,102024\_at,102242\_at,102382\_at,102580\_r\_at,102895\_at,103015\_at,103259\_at,103504\_at,104714\_at,160220\_at,160396\_at,160495\_at,160781\_r\_at,160834\_at,161113\_at,161187\_f\_at,161333\_f\_at,162114\_f\_at,162204\_r\_at,92233\_at,92300\_at,92737\_at,92991\_at,92992\_i\_at,92993\_r\_at,94331\_at,94397\_at,94689\_at,95521\_s\_at,96196\_i\_at,96197\_f\_at,96481\_at,96817\_at,97118\_at,97497\_at,98002\_at,98465\_f\_at,98767\_at,98818\_at,99100\_at,99103\_at,99665\_at,101014\_at,92339\_at,101836\_at,92758\_at,92986\_g\_at,94980\_at,102063\_at,104364\_at,104533\_at,160698\_s\_at,93274\_at,93311\_at,94483\_at,95295\_s\_at,97384\_at,97429\_at,97509\_f\_at,99458\_i\_at,160742\_at,101036\_at,103704\_at,104453\_at,94012\_at,95023\_at,95444\_at,104150\_at,102279\_at,103582\_r\_at,161396\_f\_at,161112\_at |
| 4 | pigment metabolism | 1 | 23 | 482 | 13100 | 0.002 | 0.002 | 1.176 | 0.578 | 100429\_at |
| 5 | heme metabolism | 1 | 15 | 409 | 11544 | 0.002 | 0.001 | 1.877 | 0.418 | 100429\_at |
| 6 | heme biosynthesis | 1 | 11 | 354 | 9498 | 0.003 | 0.001 | 2.431 | 0.342 | 100429\_at |
| 4 | alcohol metabolism | 6 | 167 | 482 | 13100 | 0.012 | 0.013 | 0.976 | 0.582 | 99045\_at,162092\_f\_at,104217\_at,160832\_at,96534\_at,104285\_at |
| 5 | alcohol catabolism | 1 | 58 | 409 | 11544 | 0.002 | 0.005 | 0.486 | 0.877 | 99045\_at |
| 6 | monosaccharide catabolism | 1 | 58 | 354 | 9498 | 0.003 | 0.006 | 0.462 | 0.89 | 99045\_at |
| 7 | hexose catabolism | 1 | 58 | 209 | 6246 | 0.005 | 0.009 | 0.515 | 0.862 | 99045\_at |
| 8 | glucose catabolism | 1 | 58 | 77 | 2164 | 0.013 | 0.027 | 0.485 | 0.881 | 99045\_at |
| 9 | glycolysis | 1 | 52 | 41 | 911 | 0.024 | 0.057 | 0.427 | 0.915 | 99045\_at |
| 5 | monosaccharide metabolism | 2 | 108 | 409 | 11544 | 0.005 | 0.009 | 0.522 | 0.9 | 99045\_at,162092\_f\_at |
| 6 | monosaccharide catabolism | 1 | 58 | 354 | 9498 | 0.003 | 0.006 | 0.462 | 0.89 | 99045\_at |
| 7 | hexose catabolism | 1 | 58 | 209 | 6246 | 0.005 | 0.009 | 0.515 | 0.862 | 99045\_at |
| 8 | glucose catabolism | 1 | 58 | 77 | 2164 | 0.013 | 0.027 | 0.485 | 0.881 | 99045\_at |
| 9 | glycolysis | 1 | 52 | 41 | 911 | 0.024 | 0.057 | 0.427 | 0.915 | 99045\_at |
| 6 | hexose metabolism | 2 | 107 | 354 | 9498 | 0.006 | 0.011 | 0.501 | 0.913 | 99045\_at,162092\_f\_at |
| 7 | hexose catabolism | 1 | 58 | 209 | 6246 | 0.005 | 0.009 | 0.515 | 0.862 | 99045\_at |
| 8 | glucose catabolism | 1 | 58 | 77 | 2164 | 0.013 | 0.027 | 0.485 | 0.881 | 99045\_at |
| 9 | glycolysis | 1 | 52 | 41 | 911 | 0.024 | 0.057 | 0.427 | 0.915 | 99045\_at |
| 7 | myo-inositol metabolism | 1 | 6 | 209 | 6246 | 0.005 | 0.001 | 4.979 | 0.185 | 162092\_f\_at |
| 5 | polyol metabolism | 1 | 7 | 409 | 11544 | 0.002 | 0.001 | 4 | 0.223 | 104217\_at |
| 6 | glycerol metabolism | 1 | 7 | 354 | 9498 | 0.003 | 0.001 | 3.811 | 0.234 | 104217\_at |
| 5 | sterol metabolism | 3 | 36 | 409 | 11544 | 0.007 | 0.003 | 2.349 | 0.134 | 160832\_at,96534\_at,104285\_at |
| 6 | cholesterol metabolism | 3 | 33 | 354 | 9498 | 0.008 | 0.003 | 2.441 | 0.123 | 160832\_at,96534\_at,104285\_at |
| 7 | cholesterol biosynthesis | 1 | 17 | 209 | 6246 | 0.005 | 0.003 | 1.757 | 0.44 | 104285\_at |
| 4 | amine metabolism | 3 | 148 | 482 | 13100 | 0.006 | 0.011 | 0.55 | 0.913 | 99184\_at,97386\_at,92400\_at |
| 5 | amine catabolism | 1 | 30 | 409 | 11544 | 0.002 | 0.003 | 0.938 | 0.662 | 99184\_at |
| 6 | amino acid catabolism | 1 | 24 | 354 | 9498 | 0.003 | 0.003 | 1.115 | 0.599 | 99184\_at |
| 7 | serine family amino acid catabolism | 1 | 4 | 209 | 6246 | 0.005 | 0.001 | 7.469 | 0.127 | 99184\_at |
| 8 | cysteine catabolism | 1 | 2 | 77 | 2164 | 0.013 | 0.001 | 14.12 | 0.07 | 99184\_at |
| 9 | L-cysteine catabolism | 1 | 2 | 41 | 911 | 0.024 | 0.002 | 11.086 | 0.088 | 99184\_at |
| 10 | L-cysteine catabolism to taurine | 1 | 2 | 14 | 197 | 0.071 | 0.01 | 7.037 | 0.137 | 99184\_at |
| 5 | amino acid metabolism | 1 | 97 | 409 | 11544 | 0.002 | 0.008 | 0.29 | 0.97 | 99184\_at |
| 6 | amino acid catabolism | 1 | 24 | 354 | 9498 | 0.003 | 0.003 | 1.115 | 0.599 | 99184\_at |
| 7 | serine family amino acid catabolism | 1 | 4 | 209 | 6246 | 0.005 | 0.001 | 7.469 | 0.127 | 99184\_at |
| 8 | cysteine catabolism | 1 | 2 | 77 | 2164 | 0.013 | 0.001 | 14.12 | 0.07 | 99184\_at |
| 9 | L-cysteine catabolism | 1 | 2 | 41 | 911 | 0.024 | 0.002 | 11.086 | 0.088 | 99184\_at |
| 10 | L-cysteine catabolism to taurine | 1 | 2 | 14 | 197 | 0.071 | 0.01 | 7.037 | 0.137 | 99184\_at |
| 5 | amino sugar metabolism | 1 | 9 | 409 | 11544 | 0.002 | 0.001 | 3.128 | 0.277 | 97386\_at |
| 6 | glucosamine metabolism | 1 | 6 | 354 | 9498 | 0.003 | 0.001 | 4.476 | 0.204 | 97386\_at |
| 7 | N-acetylglucosamine metabolism | 1 | 6 | 209 | 6246 | 0.005 | 0.001 | 4.979 | 0.185 | 97386\_at |
| 5 | aminoglycan metabolism | 1 | 7 | 409 | 11544 | 0.002 | 0.001 | 4 | 0.223 | 92400\_at |
| 6 | aminoglycan biosynthesis | 1 | 3 | 354 | 9498 | 0.003 | 0 | 8.812 | 0.108 | 92400\_at |
| 7 | glycosaminoglycan biosynthesis | 1 | 3 | 209 | 6246 | 0.005 | 0 | 9.958 | 0.097 | 92400\_at |
| 4 | amino acid and derivative metabolism | 1 | 134 | 482 | 13100 | 0.002 | 0.01 | 0.202 | 0.994 | 99184\_at |
| 5 | amino acid metabolism | 1 | 97 | 409 | 11544 | 0.002 | 0.008 | 0.29 | 0.97 | 99184\_at |
| 6 | amino acid catabolism | 1 | 24 | 354 | 9498 | 0.003 | 0.003 | 1.115 | 0.599 | 99184\_at |
| 7 | serine family amino acid catabolism | 1 | 4 | 209 | 6246 | 0.005 | 0.001 | 7.469 | 0.127 | 99184\_at |
| 8 | cysteine catabolism | 1 | 2 | 77 | 2164 | 0.013 | 0.001 | 14.12 | 0.07 | 99184\_at |
| 9 | L-cysteine catabolism | 1 | 2 | 41 | 911 | 0.024 | 0.002 | 11.086 | 0.088 | 99184\_at |
| 10 | L-cysteine catabolism to taurine | 1 | 2 | 14 | 197 | 0.071 | 0.01 | 7.037 | 0.137 | 99184\_at |
| 5 | amino acid derivative metabolism | 1 | 50 | 409 | 11544 | 0.002 | 0.004 | 0.564 | 0.836 | 99184\_at |
| 6 | taurine metabolism | 1 | 2 | 354 | 9498 | 0.003 | 0 | 13.429 | 0.073 | 99184\_at |
| 4 | aromatic compound metabolism | 2 | 65 | 482 | 13100 | 0.004 | 0.005 | 0.837 | 0.696 | 102313\_at,101844\_at |
| 5 | aromatic compound biosynthesis | 1 | 7 | 409 | 11544 | 0.002 | 0.001 | 4 | 0.223 | 102313\_at |
| 6 | pteridine and derivative biosynthesis | 1 | 7 | 354 | 9498 | 0.003 | 0.001 | 3.811 | 0.234 | 102313\_at |
| 7 | tetrahydrobiopterin biosynthesis | 1 | 4 | 209 | 6246 | 0.005 | 0.001 | 7.469 | 0.127 | 102313\_at |
| 5 | folic acid and derivative metabolism | 1 | 6 | 409 | 11544 | 0.002 | 0.001 | 4.692 | 0.195 | 101844\_at |
| 6 | tetrahydrofolate metabolism | 1 | 1 | 354 | 9498 | 0.003 | 0 | 25.636 | 0.037 | 101844\_at |
| 4 | biosynthesis | 32 | 652 | 482 | 13100 | 0.066 | 0.05 | 1.334 | 0.059 | 102313\_at,104285\_at,92400\_at,101186\_at,100429\_at,161785\_f\_at,95607\_at,160425\_at,102991\_s\_at,101697\_f\_at,102101\_f\_at,104048\_at,104144\_at,160283\_at,160977\_at,161667\_r\_at,93975\_at,94484\_at,95501\_at,96300\_f\_at,96577\_i\_at,96578\_r\_at,96845\_at,97083\_at,100136\_at,96784\_at,93315\_at,94818\_at,97936\_at,102980\_at,93852\_at,98435\_at |
| 5 | aromatic compound biosynthesis | 1 | 7 | 409 | 11544 | 0.002 | 0.001 | 4 | 0.223 | 102313\_at |
| 6 | pteridine and derivative biosynthesis | 1 | 7 | 354 | 9498 | 0.003 | 0.001 | 3.811 | 0.234 | 102313\_at |
| 7 | tetrahydrobiopterin biosynthesis | 1 | 4 | 209 | 6246 | 0.005 | 0.001 | 7.469 | 0.127 | 102313\_at |
| 5 | carbohydrate biosynthesis | 2 | 48 | 409 | 11544 | 0.005 | 0.004 | 1.175 | 0.511 | 92400\_at,101186\_at |
| 6 | aminoglycan biosynthesis | 1 | 3 | 354 | 9498 | 0.003 | 0 | 8.812 | 0.108 | 92400\_at |
| 7 | glycosaminoglycan biosynthesis | 1 | 3 | 209 | 6246 | 0.005 | 0 | 9.958 | 0.097 | 92400\_at |
| 6 | proteoglycan biosynthesis | 2 | 4 | 354 | 9498 | 0.006 | 0 | 13.452 | 0.008 | 101186\_at,92400\_at |
| 7 | heparan sulfate proteoglycan biosynthesis | 1 | 3 | 209 | 6246 | 0.005 | 0 | 9.958 | 0.097 | 92400\_at |
| 5 | coenzymes and prosthetic group biosynthesis | 1 | 51 | 409 | 11544 | 0.002 | 0.004 | 0.552 | 0.842 | 100429\_at |
| 6 | porphyrin biosynthesis | 1 | 11 | 354 | 9498 | 0.003 | 0.001 | 2.431 | 0.342 | 100429\_at |
| 6 | heme biosynthesis | 1 | 11 | 354 | 9498 | 0.003 | 0.001 | 2.431 | 0.342 | 100429\_at |
| 5 | hormone biosynthesis | 2 | 19 | 409 | 11544 | 0.005 | 0.002 | 2.964 | 0.144 | 161785\_f\_at,95607\_at |
| 6 | C21-steroid hormone biosynthesis | 2 | 17 | 354 | 9498 | 0.006 | 0.002 | 3.156 | 0.131 | 161785\_f\_at,95607\_at |
| 5 | lipid biosynthesis | 4 | 124 | 409 | 11544 | 0.01 | 0.011 | 0.911 | 0.645 | 160425\_at,102991\_s\_at,161785\_f\_at,95607\_at |
| 6 | isoprenoid biosynthesis | 1 | 9 | 354 | 9498 | 0.003 | 0.001 | 2.968 | 0.29 | 160425\_at |
| 6 | steroid biosynthesis | 3 | 45 | 354 | 9498 | 0.008 | 0.005 | 1.787 | 0.235 | 102991\_s\_at,161785\_f\_at,95607\_at |
| 6 | C21-steroid hormone biosynthesis | 2 | 17 | 354 | 9498 | 0.006 | 0.002 | 3.156 | 0.131 | 161785\_f\_at,95607\_at |
| 5 | macromolecule biosynthesis | 22 | 322 | 409 | 11544 | 0.054 | 0.028 | 1.929 | 0.002 | 101697\_f\_at,102101\_f\_at,104048\_at,104144\_at,160283\_at,160977\_at,161667\_r\_at,93975\_at,94484\_at,95501\_at,96300\_f\_at,96577\_i\_at,96578\_r\_at,96845\_at,97083\_at,100136\_at,96784\_at,93315\_at,94818\_at,97936\_at,102980\_at,93852\_at |
| 6 | protein biosynthesis | 22 | 322 | 354 | 9498 | 0.062 | 0.034 | 1.833 | 0.004 | 101697\_f\_at,102101\_f\_at,104048\_at,104144\_at,160283\_at,160977\_at,161667\_r\_at,93975\_at,94484\_at,95501\_at,96300\_f\_at,96577\_i\_at,96578\_r\_at,96845\_at,97083\_at,100136\_at,96784\_at,93315\_at,94818\_at,97936\_at,102980\_at,93852\_at |
| 7 | amino acid activation | 3 | 36 | 209 | 6246 | 0.014 | 0.006 | 2.491 | 0.118 | 100136\_at,96784\_at,104048\_at |
| 8 | cysteinyl-tRNA aminoacylation | 1 | 1 | 77 | 2164 | 0.013 | 0 | 28.239 | 0.036 | 104048\_at |
| 7 | cytokine biosynthesis | 1 | 4 | 209 | 6246 | 0.005 | 0.001 | 7.469 | 0.127 | 93315\_at |
| 8 | regulation of cytokine biosynthesis | 1 | 3 | 77 | 2164 | 0.013 | 0.001 | 9.345 | 0.103 | 93315\_at |
| 7 | glycoprotein biosynthesis | 2 | 52 | 209 | 6246 | 0.01 | 0.008 | 1.149 | 0.524 | 94818\_at,97936\_at |
| 8 | protein amino acid glycosylation | 2 | 52 | 77 | 2164 | 0.026 | 0.024 | 1.081 | 0.559 | 94818\_at,97936\_at |
| 9 | O-linked glycosylation | 2 | 4 | 41 | 911 | 0.049 | 0.004 | 11.112 | 0.011 | 94818\_at,97936\_at |
| 7 | lipoprotein biosynthesis | 2 | 16 | 209 | 6246 | 0.01 | 0.003 | 3.738 | 0.098 | 102980\_at,93852\_at |
| 8 | protein lipidation | 2 | 16 | 77 | 2164 | 0.026 | 0.007 | 3.514 | 0.109 | 102980\_at,93852\_at |
| 9 | protein myristoylation | 1 | 3 | 41 | 911 | 0.024 | 0.003 | 7.413 | 0.129 | 102980\_at |
| 10 | protein amino acid myristoylation | 1 | 3 | 14 | 197 | 0.071 | 0.015 | 4.69 | 0.199 | 102980\_at |
| 11 | N-terminal protein myristoylation | 1 | 3 | 5 | 34 | 0.2 | 0.088 | 2.267 | 0.389 | 102980\_at |
| 12 | N-terminal peptidyl-glycine N-myristoylation | 1 | 3 | 2 | 8 | 0.5 | 0.375 | 1.333 | 0.643 | 102980\_at |
| 9 | protein prenylation | 1 | 10 | 41 | 911 | 0.024 | 0.011 | 2.221 | 0.371 | 93852\_at |
| 10 | protein amino acid prenylation | 1 | 6 | 14 | 197 | 0.071 | 0.03 | 2.345 | 0.361 | 93852\_at |
| 7 | regulation of translation | 1 | 24 | 209 | 6246 | 0.005 | 0.004 | 1.245 | 0.559 | 101697\_f\_at |
| 8 | regulation of translational initiation | 1 | 13 | 77 | 2164 | 0.013 | 0.006 | 2.161 | 0.376 | 101697\_f\_at |
| 7 | translational elongation | 2 | 22 | 209 | 6246 | 0.01 | 0.004 | 2.719 | 0.167 | 104144\_at,94484\_at |
| 7 | translational initiation | 4 | 34 | 209 | 6246 | 0.019 | 0.005 | 3.518 | 0.026 | 93975\_at,96845\_at,97083\_at,101697\_f\_at |
| 8 | regulation of translational initiation | 1 | 13 | 77 | 2164 | 0.013 | 0.006 | 2.161 | 0.376 | 101697\_f\_at |
| 5 | nucleotide biosynthesis | 1 | 74 | 409 | 11544 | 0.002 | 0.006 | 0.381 | 0.931 | 98435\_at |
| 6 | purine nucleotide biosynthesis | 1 | 45 | 354 | 9498 | 0.003 | 0.005 | 0.595 | 0.82 | 98435\_at |
| 4 | carbohydrate metabolism | 6 | 231 | 482 | 13100 | 0.012 | 0.018 | 0.706 | 0.858 | 162228\_f\_at,99045\_at,162092\_f\_at,97386\_at,92400\_at,101186\_at |
| 5 | monosaccharide metabolism | 2 | 108 | 409 | 11544 | 0.005 | 0.009 | 0.522 | 0.9 | 99045\_at,162092\_f\_at |
| 6 | monosaccharide catabolism | 1 | 58 | 354 | 9498 | 0.003 | 0.006 | 0.462 | 0.89 | 99045\_at |
| 7 | hexose catabolism | 1 | 58 | 209 | 6246 | 0.005 | 0.009 | 0.515 | 0.862 | 99045\_at |
| 8 | glucose catabolism | 1 | 58 | 77 | 2164 | 0.013 | 0.027 | 0.485 | 0.881 | 99045\_at |
| 9 | glycolysis | 1 | 52 | 41 | 911 | 0.024 | 0.057 | 0.427 | 0.915 | 99045\_at |
| 6 | hexose metabolism | 2 | 107 | 354 | 9498 | 0.006 | 0.011 | 0.501 | 0.913 | 99045\_at,162092\_f\_at |
| 7 | hexose catabolism | 1 | 58 | 209 | 6246 | 0.005 | 0.009 | 0.515 | 0.862 | 99045\_at |
| 8 | glucose catabolism | 1 | 58 | 77 | 2164 | 0.013 | 0.027 | 0.485 | 0.881 | 99045\_at |
| 9 | glycolysis | 1 | 52 | 41 | 911 | 0.024 | 0.057 | 0.427 | 0.915 | 99045\_at |
| 7 | myo-inositol metabolism | 1 | 6 | 209 | 6246 | 0.005 | 0.001 | 4.979 | 0.185 | 162092\_f\_at |
| 5 | amino sugar metabolism | 1 | 9 | 409 | 11544 | 0.002 | 0.001 | 3.128 | 0.277 | 97386\_at |
| 6 | glucosamine metabolism | 1 | 6 | 354 | 9498 | 0.003 | 0.001 | 4.476 | 0.204 | 97386\_at |
| 7 | N-acetylglucosamine metabolism | 1 | 6 | 209 | 6246 | 0.005 | 0.001 | 4.979 | 0.185 | 97386\_at |
| 5 | aminoglycan metabolism | 1 | 7 | 409 | 11544 | 0.002 | 0.001 | 4 | 0.223 | 92400\_at |
| 6 | aminoglycan biosynthesis | 1 | 3 | 354 | 9498 | 0.003 | 0 | 8.812 | 0.108 | 92400\_at |
| 7 | glycosaminoglycan biosynthesis | 1 | 3 | 209 | 6246 | 0.005 | 0 | 9.958 | 0.097 | 92400\_at |
| 5 | carbohydrate biosynthesis | 2 | 48 | 409 | 11544 | 0.005 | 0.004 | 1.175 | 0.511 | 92400\_at,101186\_at |
| 6 | aminoglycan biosynthesis | 1 | 3 | 354 | 9498 | 0.003 | 0 | 8.812 | 0.108 | 92400\_at |
| 7 | glycosaminoglycan biosynthesis | 1 | 3 | 209 | 6246 | 0.005 | 0 | 9.958 | 0.097 | 92400\_at |
| 6 | proteoglycan biosynthesis | 2 | 4 | 354 | 9498 | 0.006 | 0 | 13.452 | 0.008 | 101186\_at,92400\_at |
| 7 | heparan sulfate proteoglycan biosynthesis | 1 | 3 | 209 | 6246 | 0.005 | 0 | 9.958 | 0.097 | 92400\_at |
| 4 | catabolism | 14 | 631 | 482 | 13100 | 0.029 | 0.048 | 0.603 | 0.987 | 99045\_at,99184\_at,103562\_f\_at,104677\_at,161039\_at,92634\_at,92918\_at,104471\_at,93509\_at,95564\_at,96176\_at,99085\_at,99086\_g\_at,96650\_at |
| 5 | alcohol catabolism | 1 | 58 | 409 | 11544 | 0.002 | 0.005 | 0.486 | 0.877 | 99045\_at |
| 6 | monosaccharide catabolism | 1 | 58 | 354 | 9498 | 0.003 | 0.006 | 0.462 | 0.89 | 99045\_at |
| 7 | hexose catabolism | 1 | 58 | 209 | 6246 | 0.005 | 0.009 | 0.515 | 0.862 | 99045\_at |
| 8 | glucose catabolism | 1 | 58 | 77 | 2164 | 0.013 | 0.027 | 0.485 | 0.881 | 99045\_at |
| 9 | glycolysis | 1 | 52 | 41 | 911 | 0.024 | 0.057 | 0.427 | 0.915 | 99045\_at |
| 5 | amine catabolism | 1 | 30 | 409 | 11544 | 0.002 | 0.003 | 0.938 | 0.662 | 99184\_at |
| 6 | amino acid catabolism | 1 | 24 | 354 | 9498 | 0.003 | 0.003 | 1.115 | 0.599 | 99184\_at |
| 7 | serine family amino acid catabolism | 1 | 4 | 209 | 6246 | 0.005 | 0.001 | 7.469 | 0.127 | 99184\_at |
| 8 | cysteine catabolism | 1 | 2 | 77 | 2164 | 0.013 | 0.001 | 14.12 | 0.07 | 99184\_at |
| 9 | L-cysteine catabolism | 1 | 2 | 41 | 911 | 0.024 | 0.002 | 11.086 | 0.088 | 99184\_at |
| 10 | L-cysteine catabolism to taurine | 1 | 2 | 14 | 197 | 0.071 | 0.01 | 7.037 | 0.137 | 99184\_at |
| 5 | cell wall catabolism | 1 | 5 | 409 | 11544 | 0.002 | 0 | 5.674 | 0.165 | 103562\_f\_at |
| 5 | macromolecule catabolism | 11 | 470 | 409 | 11544 | 0.027 | 0.041 | 0.661 | 0.949 | 104677\_at,161039\_at,92634\_at,92918\_at,104471\_at,93509\_at,95564\_at,96176\_at,99085\_at,99086\_g\_at,96650\_at |
| 6 | protein catabolism | 10 | 466 | 354 | 9498 | 0.028 | 0.049 | 0.576 | 0.982 | 104677\_at,161039\_at,92634\_at,92918\_at,104471\_at,93509\_at,95564\_at,96176\_at,99085\_at,99086\_g\_at |
| 7 | proteolysis and peptidolysis | 10 | 457 | 209 | 6246 | 0.048 | 0.073 | 0.654 | 0.949 | 104677\_at,161039\_at,92634\_at,92918\_at,104471\_at,93509\_at,95564\_at,96176\_at,99085\_at,99086\_g\_at |
| 8 | modification-dependent protein catabolism | 6 | 122 | 77 | 2164 | 0.078 | 0.056 | 1.382 | 0.265 | 104471\_at,93509\_at,95564\_at,96176\_at,99085\_at,99086\_g\_at |
| 9 | ubiquitin-dependent protein catabolism | 6 | 120 | 41 | 911 | 0.146 | 0.132 | 1.111 | 0.46 | 104471\_at,93509\_at,95564\_at,96176\_at,99085\_at,99086\_g\_at |
| 6 | RNA catabolism | 1 | 3 | 354 | 9498 | 0.003 | 0 | 8.812 | 0.108 | 96650\_at |
| 7 | mRNA catabolism | 1 | 1 | 209 | 6246 | 0.005 | 0 | 29.875 | 0.033 | 96650\_at |
| 8 | mRNA catabolism, deadenylation-dependent | 1 | 1 | 77 | 2164 | 0.013 | 0 | 28.239 | 0.036 | 96650\_at |
| 4 | electron transport | 10 | 313 | 482 | 13100 | 0.021 | 0.024 | 0.869 | 0.72 | 100429\_at,160088\_at,161005\_at,162260\_at,93424\_at,93440\_at,94951\_at,97083\_at,98533\_at,99985\_at |
| 5 | thioredoxin pathway | 1 | 3 | 409 | 11544 | 0.002 | 0 | 9.385 | 0.103 | 99985\_at |
| 4 | lipid metabolism | 11 | 285 | 482 | 13100 | 0.023 | 0.022 | 1.049 | 0.479 | 101073\_at,160425\_at,102991\_s\_at,161785\_f\_at,95607\_at,95647\_f\_at,98533\_at,103314\_at,160832\_at,96534\_at,104285\_at |
| 5 | lipid biosynthesis | 4 | 124 | 409 | 11544 | 0.01 | 0.011 | 0.911 | 0.645 | 160425\_at,102991\_s\_at,161785\_f\_at,95607\_at |
| 6 | isoprenoid biosynthesis | 1 | 9 | 354 | 9498 | 0.003 | 0.001 | 2.968 | 0.29 | 160425\_at |
| 6 | steroid biosynthesis | 3 | 45 | 354 | 9498 | 0.008 | 0.005 | 1.787 | 0.235 | 102991\_s\_at,161785\_f\_at,95607\_at |
| 6 | C21-steroid hormone biosynthesis | 2 | 17 | 354 | 9498 | 0.006 | 0.002 | 3.156 | 0.131 | 161785\_f\_at,95607\_at |
| 5 | fatty acid metabolism | 2 | 85 | 409 | 11544 | 0.005 | 0.007 | 0.664 | 0.809 | 95647\_f\_at,98533\_at |
| 5 | steroid metabolism | 7 | 74 | 409 | 11544 | 0.017 | 0.006 | 2.669 | 0.016 | 103314\_at,160832\_at,96534\_at,104285\_at,102991\_s\_at,161785\_f\_at,95607\_at |
| 6 | cholesterol metabolism | 3 | 33 | 354 | 9498 | 0.008 | 0.003 | 2.441 | 0.123 | 160832\_at,96534\_at,104285\_at |
| 7 | cholesterol biosynthesis | 1 | 17 | 209 | 6246 | 0.005 | 0.003 | 1.757 | 0.44 | 104285\_at |
| 6 | steroid biosynthesis | 3 | 45 | 354 | 9498 | 0.008 | 0.005 | 1.787 | 0.235 | 102991\_s\_at,161785\_f\_at,95607\_at |
| 6 | C21-steroid hormone biosynthesis | 2 | 17 | 354 | 9498 | 0.006 | 0.002 | 3.156 | 0.131 | 161785\_f\_at,95607\_at |
| 5 | sterol metabolism | 3 | 36 | 409 | 11544 | 0.007 | 0.003 | 2.349 | 0.134 | 160832\_at,96534\_at,104285\_at |
| 6 | cholesterol metabolism | 3 | 33 | 354 | 9498 | 0.008 | 0.003 | 2.441 | 0.123 | 160832\_at,96534\_at,104285\_at |
| 7 | cholesterol biosynthesis | 1 | 17 | 209 | 6246 | 0.005 | 0.003 | 1.757 | 0.44 | 104285\_at |
| 4 | nitrogen metabolism | 1 | 20 | 482 | 13100 | 0.002 | 0.002 | 1.353 | 0.528 | 104165\_at |
| 4 | nucleobase, nucleoside, nucleotide and nucleic acid metabolism | 73 | 1530 | 482 | 13100 | 0.151 | 0.117 | 1.297 | 0.011 | 100606\_at,95694\_at,97897\_at,93701\_at,100616\_at,104471\_at,98534\_at,94011\_at,95573\_at,95574\_f\_at,95502\_at,160174\_at,162228\_f\_at,100030\_at,98435\_at,96650\_at,104219\_f\_at,95081\_at,101079\_at,160182\_at,103545\_at,101889\_s\_at,101943\_at,95536\_at,104701\_at,100094\_at,100486\_at,100939\_at,101186\_at,102024\_at,102242\_at,102382\_at,102580\_r\_at,102895\_at,103015\_at,103259\_at,103504\_at,104714\_at,160220\_at,160396\_at,160495\_at,160781\_r\_at,160834\_at,161113\_at,161187\_f\_at,161333\_f\_at,162114\_f\_at,162204\_r\_at,92233\_at,92300\_at,92737\_at,92991\_at,92992\_i\_at,92993\_r\_at,94331\_at,94397\_at,94689\_at,95521\_s\_at,96196\_i\_at,96197\_f\_at,96481\_at,96817\_at,97118\_at,97497\_at,98002\_at,98465\_f\_at,98767\_at,98818\_at,99100\_at,99103\_at,99665\_at,101014\_at,92339\_at |
| 5 | DNA metabolism | 12 | 302 | 409 | 11544 | 0.029 | 0.026 | 1.122 | 0.384 | 95694\_at,97897\_at,93701\_at,100616\_at,104471\_at,98534\_at,94011\_at,95573\_at,95574\_f\_at,95502\_at,160174\_at,162228\_f\_at |
| 6 | DNA packaging | 9 | 93 | 354 | 9498 | 0.025 | 0.01 | 2.597 | 0.008 | 97897\_at,93701\_at,100616\_at,104471\_at,98534\_at,94011\_at,95573\_at,95574\_f\_at,95502\_at |
| 7 | establishment and/or maintenance of chromatin architecture | 8 | 80 | 209 | 6246 | 0.038 | 0.013 | 2.988 | 0.005 | 93701\_at,100616\_at,104471\_at,98534\_at,94011\_at,95573\_at,95574\_f\_at,95502\_at |
| 8 | chromatin assembly/disassembly | 2 | 48 | 77 | 2164 | 0.026 | 0.022 | 1.171 | 0.516 | 93701\_at,100616\_at |
| 9 | nucleosome assembly | 1 | 28 | 41 | 911 | 0.024 | 0.031 | 0.793 | 0.73 | 100616\_at |
| 8 | chromatin modification | 7 | 36 | 77 | 2164 | 0.091 | 0.017 | 5.463 | 0 | 104471\_at,98534\_at,93701\_at,94011\_at,95573\_at,95574\_f\_at,95502\_at |
| 9 | non-covalent chromatin modification | 5 | 13 | 41 | 911 | 0.122 | 0.014 | 8.546 | 0 | 93701\_at,94011\_at,95573\_at,95574\_f\_at,95502\_at |
| 10 | chromatin modeling | 5 | 13 | 14 | 197 | 0.357 | 0.066 | 5.412 | 0.001 | 93701\_at,94011\_at,95573\_at,95574\_f\_at,95502\_at |
| 11 | chromatin silencing | 1 | 2 | 5 | 34 | 0.2 | 0.059 | 3.4 | 0.276 | 95502\_at |
| 6 | DNA repair | 2 | 99 | 354 | 9498 | 0.006 | 0.01 | 0.542 | 0.889 | 160174\_at,162228\_f\_at |
| 5 | nucleoside metabolism | 1 | 15 | 409 | 11544 | 0.002 | 0.001 | 1.877 | 0.418 | 100030\_at |
| 5 | nucleotide metabolism | 1 | 95 | 409 | 11544 | 0.002 | 0.008 | 0.296 | 0.968 | 98435\_at |
| 6 | purine nucleotide biosynthesis | 1 | 45 | 354 | 9498 | 0.003 | 0.005 | 0.595 | 0.82 | 98435\_at |
| 6 | purine nucleotide metabolism | 1 | 54 | 354 | 9498 | 0.003 | 0.006 | 0.496 | 0.872 | 98435\_at |
| 6 | purine nucleotide biosynthesis | 1 | 45 | 354 | 9498 | 0.003 | 0.005 | 0.595 | 0.82 | 98435\_at |
| 5 | nucleotide biosynthesis | 1 | 74 | 409 | 11544 | 0.002 | 0.006 | 0.381 | 0.931 | 98435\_at |
| 6 | purine nucleotide biosynthesis | 1 | 45 | 354 | 9498 | 0.003 | 0.005 | 0.595 | 0.82 | 98435\_at |
| 5 | RNA metabolism | 7 | 132 | 409 | 11544 | 0.017 | 0.011 | 1.497 | 0.188 | 96650\_at,104219\_f\_at,160174\_at,95081\_at,101079\_at,160182\_at,103545\_at |
| 6 | RNA catabolism | 1 | 3 | 354 | 9498 | 0.003 | 0 | 8.812 | 0.108 | 96650\_at |
| 7 | mRNA catabolism | 1 | 1 | 209 | 6246 | 0.005 | 0 | 29.875 | 0.033 | 96650\_at |
| 8 | mRNA catabolism, deadenylation-dependent | 1 | 1 | 77 | 2164 | 0.013 | 0 | 28.239 | 0.036 | 96650\_at |
| 6 | RNA processing | 6 | 126 | 354 | 9498 | 0.017 | 0.013 | 1.277 | 0.329 | 104219\_f\_at,160174\_at,95081\_at,101079\_at,160182\_at,103545\_at |
| 7 | mRNA processing | 2 | 84 | 209 | 6246 | 0.01 | 0.013 | 0.712 | 0.778 | 101079\_at,160182\_at |
| 8 | nuclear mRNA splicing, via spliceosome | 1 | NA | 77 | 2164 | 0.013 | NA | NA | NA | 160182\_at |
| 9 | spliceosome assembly | 1 | 19 | 41 | 911 | 0.024 | 0.021 | 1.169 | 0.587 | 160182\_at |
| 10 | mRNA splice site selection | 1 | 16 | 14 | 197 | 0.071 | 0.081 | 0.879 | 0.707 | 160182\_at |
| 7 | tRNA processing | 1 | 6 | 209 | 6246 | 0.005 | 0.001 | 4.979 | 0.185 | 103545\_at |
| 5 | transcription | 59 | 1086 | 409 | 11544 | 0.144 | 0.094 | 1.533 | 0.001 | 101889\_s\_at,101943\_at,160174\_at,95536\_at,104701\_at,95502\_at,100094\_at,100486\_at,100939\_at,101186\_at,102024\_at,102242\_at,102382\_at,102580\_r\_at,102895\_at,103015\_at,103259\_at,103504\_at,104471\_at,104714\_at,160220\_at,160396\_at,160495\_at,160781\_r\_at,160834\_at,161113\_at,161187\_f\_at,161333\_f\_at,162114\_f\_at,162204\_r\_at,92233\_at,92300\_at,92737\_at,92991\_at,92992\_i\_at,92993\_r\_at,94331\_at,94397\_at,94689\_at,95521\_s\_at,95573\_at,95574\_f\_at,96196\_i\_at,96197\_f\_at,96481\_at,96817\_at,97118\_at,97497\_at,98002\_at,98465\_f\_at,98534\_at,98767\_at,98818\_at,99100\_at,99103\_at,99665\_at,101014\_at,92339\_at,95081\_at |
| 6 | regulation of transcription | 54 | 1026 | 354 | 9498 | 0.153 | 0.108 | 1.412 | 0.005 | 104701\_at,95502\_at,100094\_at,100486\_at,100939\_at,101186\_at,101889\_s\_at,102024\_at,102242\_at,102382\_at,102580\_r\_at,102895\_at,103015\_at,103259\_at,103504\_at,104471\_at,104714\_at,160220\_at,160396\_at,160495\_at,160781\_r\_at,160834\_at,161113\_at,161187\_f\_at,161333\_f\_at,162114\_f\_at,162204\_r\_at,92233\_at,92300\_at,92737\_at,92991\_at,92992\_i\_at,92993\_r\_at,94331\_at,94397\_at,94689\_at,95521\_s\_at,95573\_at,95574\_f\_at,96196\_i\_at,96197\_f\_at,96481\_at,96817\_at,97118\_at,97497\_at,98002\_at,98465\_f\_at,98534\_at,98767\_at,98818\_at,99100\_at,99103\_at,99665\_at,101014\_at |
| 7 | negative regulation of transcription | 2 | 40 | 209 | 6246 | 0.01 | 0.006 | 1.495 | 0.389 | 104701\_at,95502\_at |
| 8 | negative regulation of transcription, DNA-dependent | 2 | 27 | 77 | 2164 | 0.026 | 0.012 | 2.081 | 0.249 | 104701\_at,95502\_at |
| 7 | regulation of transcription, DNA-dependent | 54 | 1013 | 209 | 6246 | 0.258 | 0.162 | 1.593 | 0 | 100094\_at,100486\_at,100939\_at,101186\_at,101889\_s\_at,102024\_at,102242\_at,102382\_at,102580\_r\_at,102895\_at,103015\_at,103259\_at,103504\_at,104471\_at,104701\_at,104714\_at,160220\_at,160396\_at,160495\_at,160781\_r\_at,160834\_at,161113\_at,161187\_f\_at,161333\_f\_at,162114\_f\_at,162204\_r\_at,92233\_at,92300\_at,92737\_at,92991\_at,92992\_i\_at,92993\_r\_at,94331\_at,94397\_at,94689\_at,95502\_at,95521\_s\_at,95573\_at,95574\_f\_at,96196\_i\_at,96197\_f\_at,96481\_at,96817\_at,97118\_at,97497\_at,98002\_at,98465\_f\_at,98534\_at,98767\_at,98818\_at,99100\_at,99103\_at,99665\_at,101014\_at |
| 8 | negative regulation of transcription, DNA-dependent | 2 | 27 | 77 | 2164 | 0.026 | 0.012 | 2.081 | 0.249 | 104701\_at,95502\_at |
| 8 | regulation of transcription from Pol II promoter | 5 | 72 | 77 | 2164 | 0.065 | 0.033 | 1.952 | 0.11 | 101014\_at,92991\_at,92992\_i\_at,92993\_r\_at,99100\_at |
| 6 | transcription, DNA-dependent | 56 | 1046 | 354 | 9498 | 0.158 | 0.11 | 1.436 | 0.003 | 100094\_at,100486\_at,100939\_at,101186\_at,101889\_s\_at,102024\_at,102242\_at,102382\_at,102580\_r\_at,102895\_at,103015\_at,103259\_at,103504\_at,104471\_at,104701\_at,104714\_at,160220\_at,160396\_at,160495\_at,160781\_r\_at,160834\_at,161113\_at,161187\_f\_at,161333\_f\_at,162114\_f\_at,162204\_r\_at,92233\_at,92300\_at,92737\_at,92991\_at,92992\_i\_at,92993\_r\_at,94331\_at,94397\_at,94689\_at,95502\_at,95521\_s\_at,95573\_at,95574\_f\_at,96196\_i\_at,96197\_f\_at,96481\_at,96817\_at,97118\_at,97497\_at,98002\_at,98465\_f\_at,98534\_at,98767\_at,98818\_at,99100\_at,99103\_at,99665\_at,101014\_at,92339\_at,95081\_at |
| 7 | regulation of transcription, DNA-dependent | 54 | 1013 | 209 | 6246 | 0.258 | 0.162 | 1.593 | 0 | 100094\_at,100486\_at,100939\_at,101186\_at,101889\_s\_at,102024\_at,102242\_at,102382\_at,102580\_r\_at,102895\_at,103015\_at,103259\_at,103504\_at,104471\_at,104701\_at,104714\_at,160220\_at,160396\_at,160495\_at,160781\_r\_at,160834\_at,161113\_at,161187\_f\_at,161333\_f\_at,162114\_f\_at,162204\_r\_at,92233\_at,92300\_at,92737\_at,92991\_at,92992\_i\_at,92993\_r\_at,94331\_at,94397\_at,94689\_at,95502\_at,95521\_s\_at,95573\_at,95574\_f\_at,96196\_i\_at,96197\_f\_at,96481\_at,96817\_at,97118\_at,97497\_at,98002\_at,98465\_f\_at,98534\_at,98767\_at,98818\_at,99100\_at,99103\_at,99665\_at,101014\_at |
| 8 | negative regulation of transcription, DNA-dependent | 2 | 27 | 77 | 2164 | 0.026 | 0.012 | 2.081 | 0.249 | 104701\_at,95502\_at |
| 8 | regulation of transcription from Pol II promoter | 5 | 72 | 77 | 2164 | 0.065 | 0.033 | 1.952 | 0.11 | 101014\_at,92991\_at,92992\_i\_at,92993\_r\_at,99100\_at |
| 7 | transcription from Pol I promoter | 2 | 20 | 209 | 6246 | 0.01 | 0.003 | 2.991 | 0.143 | 92339\_at,95081\_at |
| 8 | rRNA processing | 1 | 14 | 77 | 2164 | 0.013 | 0.006 | 2.008 | 0.399 | 95081\_at |
| 7 | transcription from Pol II promoter | 5 | 104 | 209 | 6246 | 0.024 | 0.017 | 1.437 | 0.268 | 99100\_at,101014\_at,92991\_at,92992\_i\_at,92993\_r\_at |
| 8 | regulation of transcription from Pol II promoter | 5 | 72 | 77 | 2164 | 0.065 | 0.033 | 1.952 | 0.11 | 101014\_at,92991\_at,92992\_i\_at,92993\_r\_at,99100\_at |
| 4 | phosphorus metabolism | 18 | 488 | 482 | 13100 | 0.037 | 0.037 | 1.002 | 0.531 | 101836\_at,92758\_at,92986\_g\_at,94980\_at,102063\_at,104364\_at,104533\_at,160698\_s\_at,93274\_at,93311\_at,93315\_at,93424\_at,94483\_at,95295\_s\_at,97384\_at,97429\_at,97509\_f\_at,99458\_i\_at |
| 5 | phosphate metabolism | 18 | 488 | 409 | 11544 | 0.044 | 0.042 | 1.041 | 0.465 | 101836\_at,92758\_at,92986\_g\_at,94980\_at,102063\_at,104364\_at,104533\_at,160698\_s\_at,93274\_at,93311\_at,93315\_at,93424\_at,94483\_at,95295\_s\_at,97384\_at,97429\_at,97509\_f\_at,99458\_i\_at |
| 6 | dephosphorylation | 4 | 92 | 354 | 9498 | 0.011 | 0.01 | 1.166 | 0.45 | 101836\_at,92758\_at,92986\_g\_at,94980\_at |
| 7 | protein amino acid dephosphorylation | 4 | 92 | 209 | 6246 | 0.019 | 0.015 | 1.299 | 0.371 | 101836\_at,92758\_at,92986\_g\_at,94980\_at |
| 6 | phosphorylation | 14 | 395 | 354 | 9498 | 0.04 | 0.042 | 0.951 | 0.617 | 102063\_at,104364\_at,104533\_at,160698\_s\_at,93274\_at,93311\_at,93315\_at,93424\_at,94483\_at,95295\_s\_at,97384\_at,97429\_at,97509\_f\_at,99458\_i\_at |
| 7 | protein amino acid phosphorylation | 14 | 379 | 209 | 6246 | 0.067 | 0.061 | 1.104 | 0.39 | 102063\_at,104364\_at,104533\_at,160698\_s\_at,93274\_at,93311\_at,93315\_at,93424\_at,94483\_at,95295\_s\_at,97384\_at,97429\_at,97509\_f\_at,99458\_i\_at |
| 4 | protein metabolism | 63 | 1458 | 482 | 13100 | 0.131 | 0.111 | 1.174 | 0.098 | 160742\_at,101036\_at,103704\_at,104453\_at,94012\_at,95023\_at,95444\_at,101079\_at,104150\_at,101697\_f\_at,102101\_f\_at,104048\_at,104144\_at,160283\_at,160977\_at,161667\_r\_at,93975\_at,94484\_at,95501\_at,96300\_f\_at,96577\_i\_at,96578\_r\_at,96845\_at,97083\_at,100136\_at,96784\_at,93315\_at,94818\_at,97936\_at,102980\_at,93852\_at,104677\_at,161039\_at,92634\_at,92918\_at,104471\_at,93509\_at,95564\_at,96176\_at,99085\_at,99086\_g\_at,102279\_at,103582\_r\_at,161396\_f\_at,101836\_at,92758\_at,92986\_g\_at,94980\_at,102063\_at,104364\_at,104533\_at,160698\_s\_at,93274\_at,93311\_at,93424\_at,94483\_at,95295\_s\_at,97384\_at,97429\_at,97509\_f\_at,99458\_i\_at,97897\_at,161112\_at |
| 5 | protein folding | 1 | 56 | 409 | 11544 | 0.002 | 0.005 | 0.503 | 0.868 | 102279\_at |
| 5 | protein modification | 30 | 654 | 409 | 11544 | 0.073 | 0.057 | 1.295 | 0.088 | 103582\_r\_at,160742\_at,161396\_f\_at,99085\_at,99086\_g\_at,94818\_at,97936\_at,102980\_at,93852\_at,101836\_at,92758\_at,92986\_g\_at,94980\_at,102063\_at,104364\_at,104533\_at,160698\_s\_at,93274\_at,93311\_at,93315\_at,93424\_at,94483\_at,95295\_s\_at,97384\_at,97429\_at,97509\_f\_at,99458\_i\_at,97897\_at,104471\_at,161112\_at |
| 6 | protein amino acid acetylation | 1 | 6 | 354 | 9498 | 0.003 | 0.001 | 4.476 | 0.204 | 97897\_at |
| 7 | internal protein amino acid acetylation | 1 | 4 | 209 | 6246 | 0.005 | 0.001 | 7.469 | 0.127 | 97897\_at |
| 6 | protein amino acid deacetylation | 1 | 3 | 354 | 9498 | 0.003 | 0 | 8.812 | 0.108 | 104471\_at |
| 6 | protein processing | 1 | 6 | 354 | 9498 | 0.003 | 0.001 | 4.476 | 0.204 | 161112\_at |
| 7 | protein splicing | 1 | 5 | 209 | 6246 | 0.005 | 0.001 | 5.975 | 0.157 | 161112\_at |
| 6 | ubiquitin cycle | 5 | 56 | 354 | 9498 | 0.014 | 0.006 | 2.393 | 0.057 | 103582\_r\_at,161396\_f\_at,99085\_at,99086\_g\_at,104471\_at |
| 7 | protein deubiquitination | 2 | 7 | 209 | 6246 | 0.01 | 0.001 | 8.545 | 0.021 | 99085\_at,99086\_g\_at |
| 7 | protein ubiquitination | 1 | 5 | 209 | 6246 | 0.005 | 0.001 | 5.975 | 0.157 | 104471\_at |
| 8 | protein polyubiquitination | 1 | 1 | 77 | 2164 | 0.013 | 0 | 28.239 | 0.036 | 104471\_at |
| 3 | pathogenesis | 2 | 10 | 415 | 10726 | 0.005 | 0.001 | 5.183 | 0.055 | 101073\_at,94928\_at |
| 4 | necrosis | 1 | 3 | 482 | 13100 | 0.002 | 0 | 9 | 0.106 | 94928\_at |
| 3 | respiratory gaseous exchange | 1 | 10 | 415 | 10726 | 0.002 | 0.001 | 2.591 | 0.326 | 93193\_at |
| 3 | response to external stimulus | 25 | 666 | 415 | 10726 | 0.06 | 0.062 | 0.97 | 0.594 | 101432\_at,104388\_at,104572\_at,160512\_at,93199\_at,160099\_at,161745\_f\_at,92571\_at,103422\_at,104692\_at,93909\_f\_at,98000\_at,100973\_i\_at,103080\_at,93321\_at,94224\_s\_at,96511\_s\_at,97710\_f\_at,98002\_at,98465\_f\_at,100333\_at,99100\_at,103033\_at,93315\_at,94928\_at |
| 4 | perception of external stimulus | 1 | 84 | 482 | 13100 | 0.002 | 0.006 | 0.323 | 0.958 | 101432\_at |
| 5 | perception of abiotic stimulus | 1 | 72 | 409 | 11544 | 0.002 | 0.006 | 0.391 | 0.926 | 101432\_at |
| 6 | perception of light | 1 | 41 | 354 | 9498 | 0.003 | 0.004 | 0.653 | 0.79 | 101432\_at |
| 7 | vision | 1 | 34 | 209 | 6246 | 0.005 | 0.005 | 0.879 | 0.687 | 101432\_at |
| 4 | response to abiotic stimulus | 8 | 195 | 482 | 13100 | 0.017 | 0.015 | 1.115 | 0.428 | 101432\_at,104388\_at,104572\_at,160512\_at,93199\_at,160099\_at,161745\_f\_at,92571\_at |
| 5 | perception of abiotic stimulus | 1 | 72 | 409 | 11544 | 0.002 | 0.006 | 0.391 | 0.926 | 101432\_at |
| 6 | perception of light | 1 | 41 | 354 | 9498 | 0.003 | 0.004 | 0.653 | 0.79 | 101432\_at |
| 7 | vision | 1 | 34 | 209 | 6246 | 0.005 | 0.005 | 0.879 | 0.687 | 101432\_at |
| 5 | response to chemical substance | 4 | 90 | 409 | 11544 | 0.01 | 0.008 | 1.254 | 0.396 | 104388\_at,104572\_at,160512\_at,93199\_at |
| 6 | chemotaxis | 4 | 60 | 354 | 9498 | 0.011 | 0.006 | 1.788 | 0.184 | 104388\_at,104572\_at,160512\_at,93199\_at |
| 7 | immune cell chemotaxis | 1 | 3 | 209 | 6246 | 0.005 | 0 | 9.958 | 0.097 | 93199\_at |
| 8 | neutrophil chemotaxis | 1 | 1 | 77 | 2164 | 0.013 | 0 | 28.239 | 0.036 | 93199\_at |
| 5 | response to temperature | 3 | 28 | 409 | 11544 | 0.007 | 0.002 | 3.016 | 0.075 | 160099\_at,161745\_f\_at,92571\_at |
| 6 | response to heat | 3 | 27 | 354 | 9498 | 0.008 | 0.003 | 2.982 | 0.078 | 160099\_at,161745\_f\_at,92571\_at |
| 4 | response to biotic stimulus | 21 | 516 | 482 | 13100 | 0.044 | 0.039 | 1.106 | 0.348 | 103422\_at,104692\_at,93909\_f\_at,98000\_at,100973\_i\_at,103080\_at,104388\_at,104572\_at,160512\_at,93321\_at,94224\_s\_at,96511\_s\_at,97710\_f\_at,98002\_at,98465\_f\_at,100333\_at,99100\_at,103033\_at,93315\_at,94928\_at,93199\_at |
| 5 | defense response | 21 | 471 | 409 | 11544 | 0.051 | 0.041 | 1.258 | 0.165 | 103422\_at,104692\_at,93909\_f\_at,98000\_at,100973\_i\_at,103080\_at,104388\_at,104572\_at,160512\_at,93321\_at,94224\_s\_at,96511\_s\_at,97710\_f\_at,98002\_at,98465\_f\_at,100333\_at,99100\_at,103033\_at,93315\_at,94928\_at,93199\_at |
| 6 | immune response | 18 | 362 | 354 | 9498 | 0.051 | 0.038 | 1.334 | 0.13 | 100973\_i\_at,103080\_at,104388\_at,104572\_at,160512\_at,93321\_at,94224\_s\_at,96511\_s\_at,97710\_f\_at,98002\_at,98465\_f\_at,100333\_at,99100\_at,103033\_at,104692\_at,93315\_at,94928\_at,93199\_at |
| 7 | acute-phase response | 2 | 23 | 209 | 6246 | 0.01 | 0.004 | 2.601 | 0.179 | 100333\_at,99100\_at |
| 7 | cell activation | 2 | 19 | 209 | 6246 | 0.01 | 0.003 | 3.148 | 0.131 | 96511\_s\_at,160512\_at |
| 8 | lymphocyte activation | 2 | 19 | 77 | 2164 | 0.026 | 0.009 | 2.958 | 0.145 | 96511\_s\_at,160512\_at |
| 9 | lymphocyte differentiation | 1 | 5 | 41 | 911 | 0.024 | 0.005 | 4.443 | 0.206 | 96511\_s\_at |
| 10 | thymocyte differentiation | 1 | 2 | 14 | 197 | 0.071 | 0.01 | 7.037 | 0.137 | 96511\_s\_at |
| 9 | T-cell activation | 2 | 9 | 41 | 911 | 0.049 | 0.01 | 4.937 | 0.058 | 96511\_s\_at,160512\_at |
| 7 | humoral immune response | 1 | 103 | 209 | 6246 | 0.005 | 0.016 | 0.29 | 0.971 | 103033\_at |
| 8 | humoral defense mechanism (sensu Vertebrata) | 1 | 39 | 77 | 2164 | 0.013 | 0.018 | 0.721 | 0.76 | 103033\_at |
| 9 | complement activation | 1 | 38 | 41 | 911 | 0.024 | 0.042 | 0.585 | 0.833 | 103033\_at |
| 7 | innate immune response | 4 | 70 | 209 | 6246 | 0.019 | 0.011 | 1.707 | 0.206 | 104692\_at,93315\_at,94928\_at,93199\_at |
| 8 | inflammatory response | 4 | 70 | 77 | 2164 | 0.052 | 0.032 | 1.606 | 0.236 | 104692\_at,93315\_at,94928\_at,93199\_at |
| 8 | neutrophil chemotaxis | 1 | 1 | 77 | 2164 | 0.013 | 0 | 28.239 | 0.036 | 93199\_at |
| 5 | T-cell proliferation | 1 | 4 | 409 | 11544 | 0.002 | 0 | 6.971 | 0.134 | 160512\_at |

  
