# Supplementary material for: Molecular Signatures of Proliferation and Quiescence in Hematopoietic Stem Cells
Source: PLoS Biol. 2004 Sep 28;2(10):e301. doi: 10.1371/journal.pbio.0020301 (PMC520599; doi:10.1371/journal.pbio.0020301)
Supplement: Table S33 — (45 KB HTML). [file pbio.0020301.st033.html]

   Significant Tom Day 10   

# Significant Tom Day 10

|  |  |  |  |  |  |  |  |  |  |  |  |
| --- | --- | --- | --- | --- | --- | --- | --- | --- | --- | --- | --- |
| GOLevel | GOTerm | ProbeCount | ArrayCount | ListGOLevelCount | ArrayGoLevelCount | ListFq | ArrayFq | FoldChange | H-Pvalue | ProbeIds | GeneNames |
| 3 | rhythmic behavior | 3 | 17 | 415 | 10726 | 0.007 | 0.002 | 4.576 | 0.026 | 102242\_at,162384\_f\_at,102382\_at | period homolog 3 (Drosophila),CCR4 carbon catabolite repression 4-like (S. cerevisiae),aryl hydrocarbon receptor nuclear translocator-like |
| 6 | protein kinase cascade | 5 | 50 | 354 | 9498 | 0.014 | 0.005 | 2.684 | 0.038 | 94264\_at,99100\_at,93315\_at,161667\_r\_at,94006\_at | v-raf-1 leukemia viral oncogene 1,signal transducer and activator of transcription 3,mitogen activated protein kinase kinase 3,RIKEN cDNA 1110001M20 gene,5-azacytidine induced gene 2 |
| 8 | copper ion homeostasis | 1 | 1 | 77 | 2164 | 0.013 | 0 | 28.239 | 0.036 | 100606\_at | prion protein |
| 7 | cytoskeleton organization and biogenesis | 15 | 262 | 209 | 6246 | 0.072 | 0.042 | 1.711 | 0.029 | 100877\_at,92542\_at,94236\_at,95489\_at,97593\_f\_at,104683\_at,160287\_at,160461\_f\_at,95119\_at,98461\_at,160140\_at,104471\_at,100939\_at,98882\_s\_at,98884\_r\_at | RIKEN cDNA 1810058I24 gene,DNA segment, Chr 4, Wayne State University 53, expressed,nischarin,flightless I homolog (Drosophila),flightless I homolog (Drosophila),expressed sequence AA407558,microtubule-associated protein 1 light chain 3,RIKEN cDNA 2310057H16 gene,RIKEN cDNA 1110038D17 gene,RIKEN cDNA 1200014P03 gene,RIKEN cDNA D230016N13 gene,histone deacetylase 6,expressed sequence AI449432,nuclear distribution gene E-like homolog 1 (A. nidulans),nuclear distribution gene E-like homolog 1 (A. nidulans) |
| 8 | microtubule-based process | 10 | 119 | 77 | 2164 | 0.13 | 0.055 | 2.362 | 0.008 | 104683\_at,160287\_at,160461\_f\_at,95119\_at,98461\_at,160140\_at,104471\_at,100939\_at,98882\_s\_at,98884\_r\_at | expressed sequence AA407558,microtubule-associated protein 1 light chain 3,RIKEN cDNA 2310057H16 gene,RIKEN cDNA 1110038D17 gene,RIKEN cDNA 1200014P03 gene,RIKEN cDNA D230016N13 gene,histone deacetylase 6,expressed sequence AI449432,nuclear distribution gene E-like homolog 1 (A. nidulans),nuclear distribution gene E-like homolog 1 (A. nidulans) |
| 9 | microtubule cytoskeleton organization and biogenesis | 3 | 12 | 41 | 911 | 0.073 | 0.013 | 5.556 | 0.014 | 104683\_at,160140\_at,104471\_at | expressed sequence AA407558,RIKEN cDNA D230016N13 gene,histone deacetylase 6 |
| 9 | microtubule-based movement | 5 | 38 | 41 | 911 | 0.122 | 0.042 | 2.924 | 0.024 | 100939\_at,104683\_at,160461\_f\_at,98882\_s\_at,98884\_r\_at | expressed sequence AI449432,expressed sequence AA407558,RIKEN cDNA 2310057H16 gene,nuclear distribution gene E-like homolog 1 (A. nidulans),nuclear distribution gene E-like homolog 1 (A. nidulans) |
| 10 | axon cargo transport | 2 | 4 | 14 | 197 | 0.143 | 0.02 | 7.037 | 0.026 | 98882\_s\_at,98884\_r\_at | nuclear distribution gene E-like homolog 1 (A. nidulans),nuclear distribution gene E-like homolog 1 (A. nidulans) |
| 5 | nuclear organization and biogenesis | 8 | 112 | 409 | 11544 | 0.02 | 0.01 | 2.016 | 0.045 | 100616\_at,93701\_at,104471\_at,98534\_at,94011\_at,95573\_at,95574\_f\_at,95502\_at | centromere autoantigen A,SWI/SNF related, matrix associated, actin dependent regulator of chromatin, subfamily a, member 5,histone deacetylase 6,Sin3-associated polypeptide 18,RIKEN cDNA 2310022K01 gene,bromodomain adjacent to zinc finger domain, 2A,CGG triplet repeat binding protein 1,sirtuin 2 (silent mating type information regulation 2, homolog) 2 (S. cerevisiae) |
| 6 | chromosome organization and biogenesis (sensu Eukarya) | 8 | 108 | 354 | 9498 | 0.023 | 0.011 | 1.988 | 0.048 | 100616\_at,93701\_at,104471\_at,98534\_at,94011\_at,95573\_at,95574\_f\_at,95502\_at | centromere autoantigen A,SWI/SNF related, matrix associated, actin dependent regulator of chromatin, subfamily a, member 5,histone deacetylase 6,Sin3-associated polypeptide 18,RIKEN cDNA 2310022K01 gene,bromodomain adjacent to zinc finger domain, 2A,CGG triplet repeat binding protein 1,sirtuin 2 (silent mating type information regulation 2, homolog) 2 (S. cerevisiae) |
| 7 | establishment and/or maintenance of chromatin architecture | 8 | 80 | 209 | 6246 | 0.038 | 0.013 | 2.988 | 0.005 | 93701\_at,100616\_at,104471\_at,98534\_at,94011\_at,95573\_at,95574\_f\_at,95502\_at | SWI/SNF related, matrix associated, actin dependent regulator of chromatin, subfamily a, member 5,centromere autoantigen A,histone deacetylase 6,Sin3-associated polypeptide 18,RIKEN cDNA 2310022K01 gene,bromodomain adjacent to zinc finger domain, 2A,CGG triplet repeat binding protein 1,sirtuin 2 (silent mating type information regulation 2, homolog) 2 (S. cerevisiae) |
| 8 | chromatin modification | 7 | 36 | 77 | 2164 | 0.091 | 0.017 | 5.463 | 0 | 104471\_at,98534\_at,93701\_at,94011\_at,95573\_at,95574\_f\_at,95502\_at | histone deacetylase 6,Sin3-associated polypeptide 18,SWI/SNF related, matrix associated, actin dependent regulator of chromatin, subfamily a, member 5,RIKEN cDNA 2310022K01 gene,bromodomain adjacent to zinc finger domain, 2A,CGG triplet repeat binding protein 1,sirtuin 2 (silent mating type information regulation 2, homolog) 2 (S. cerevisiae) |
| 9 | non-covalent chromatin modification | 5 | 13 | 41 | 911 | 0.122 | 0.014 | 8.546 | 0 | 93701\_at,94011\_at,95573\_at,95574\_f\_at,95502\_at | SWI/SNF related, matrix associated, actin dependent regulator of chromatin, subfamily a, member 5,RIKEN cDNA 2310022K01 gene,bromodomain adjacent to zinc finger domain, 2A,CGG triplet repeat binding protein 1,sirtuin 2 (silent mating type information regulation 2, homolog) 2 (S. cerevisiae) |
| 10 | chromatin modeling | 5 | 13 | 14 | 197 | 0.357 | 0.066 | 5.412 | 0.001 | 93701\_at,94011\_at,95573\_at,95574\_f\_at,95502\_at | SWI/SNF related, matrix associated, actin dependent regulator of chromatin, subfamily a, member 5,RIKEN cDNA 2310022K01 gene,bromodomain adjacent to zinc finger domain, 2A,CGG triplet repeat binding protein 1,sirtuin 2 (silent mating type information regulation 2, homolog) 2 (S. cerevisiae) |
| 7 | protein targeting | 8 | 101 | 209 | 6246 | 0.038 | 0.016 | 2.367 | 0.019 | 101036\_at,103704\_at,104453\_at,94012\_at,95023\_at,95444\_at,101079\_at,104150\_at | translocase of outer mitochondrial membrane 20 homolog (yeast),RIKEN cDNA 2010305K11 gene,RIKEN cDNA 2310079P12 gene,translocase of inner mitochondrial membrane 13 homolog a (yeast),hypothetical protein LOC214597,RIKEN cDNA 4930579A11 gene,nuclear RNA export factor 1 homolog (S. cerevisiae),RIKEN cDNA 2810008P14 gene |
| 5 | lipid transport | 5 | 40 | 409 | 11544 | 0.012 | 0.003 | 3.522 | 0.013 | 103314\_at,160832\_at,161785\_f\_at,95607\_at,96534\_at | DNA segment, Chr 13, ERATO Doi 275, expressed,low density lipoprotein receptor,hypothetical protein MGC28888,START domain containing 3,very low density lipoprotein receptor |
| 7 | protein targeting | 8 | 101 | 209 | 6246 | 0.038 | 0.016 | 2.367 | 0.019 | 101036\_at,103704\_at,104453\_at,94012\_at,95023\_at,95444\_at,101079\_at,104150\_at | translocase of outer mitochondrial membrane 20 homolog (yeast),RIKEN cDNA 2010305K11 gene,RIKEN cDNA 2310079P12 gene,translocase of inner mitochondrial membrane 13 homolog a (yeast),hypothetical protein LOC214597,RIKEN cDNA 4930579A11 gene,nuclear RNA export factor 1 homolog (S. cerevisiae),RIKEN cDNA 2810008P14 gene |
| 5 | ear morphogenesis | 2 | 3 | 409 | 11544 | 0.005 | 0 | 18.808 | 0.004 | 103259\_at,97509\_f\_at | growth factor independent 1,fibroblast growth factor receptor 1 |
| 6 | inner ear morphogenesis | 2 | 3 | 354 | 9498 | 0.006 | 0 | 17.656 | 0.004 | 103259\_at,97509\_f\_at | growth factor independent 1,fibroblast growth factor receptor 1 |
| 5 | ear morphogenesis | 2 | 3 | 409 | 11544 | 0.005 | 0 | 18.808 | 0.004 | 103259\_at,97509\_f\_at | growth factor independent 1,fibroblast growth factor receptor 1 |
| 6 | inner ear morphogenesis | 2 | 3 | 354 | 9498 | 0.006 | 0 | 17.656 | 0.004 | 103259\_at,97509\_f\_at | growth factor independent 1,fibroblast growth factor receptor 1 |
| 5 | ear morphogenesis | 2 | 3 | 409 | 11544 | 0.005 | 0 | 18.808 | 0.004 | 103259\_at,97509\_f\_at | growth factor independent 1,fibroblast growth factor receptor 1 |
| 6 | inner ear morphogenesis | 2 | 3 | 354 | 9498 | 0.006 | 0 | 17.656 | 0.004 | 103259\_at,97509\_f\_at | growth factor independent 1,fibroblast growth factor receptor 1 |
| 5 | midgut development | 1 | 1 | 409 | 11544 | 0.002 | 0 | 27.111 | 0.035 | 101186\_at | per-pentamer repeat gene |
| 6 | visceral mesoderm/endoderm interaction | 1 | 1 | 354 | 9498 | 0.003 | 0 | 25.636 | 0.037 | 101186\_at | per-pentamer repeat gene |
| 4 | compartment specification | 2 | 8 | 482 | 13100 | 0.004 | 0.001 | 6.803 | 0.033 | 162204\_r\_at,97497\_at | Notch gene homolog 1 (Drosophila),Notch gene homolog 1 (Drosophila) |
| 2 | obsolete biological process | 2 | 3 | 428 | 10540 | 0.005 | 0 | 16.679 | 0.005 | 160182\_at,93520\_at | splicing factor, arginine/serine-rich 6,serine/arginine repetitive matrix 1 |
| 8 | copper ion homeostasis | 1 | 1 | 77 | 2164 | 0.013 | 0 | 28.239 | 0.036 | 100606\_at | prion protein |
| 7 | cytoskeleton organization and biogenesis | 15 | 262 | 209 | 6246 | 0.072 | 0.042 | 1.711 | 0.029 | 100877\_at,92542\_at,94236\_at,95489\_at,97593\_f\_at,104683\_at,160287\_at,160461\_f\_at,95119\_at,98461\_at,160140\_at,104471\_at,100939\_at,98882\_s\_at,98884\_r\_at | RIKEN cDNA 1810058I24 gene,DNA segment, Chr 4, Wayne State University 53, expressed,nischarin,flightless I homolog (Drosophila),flightless I homolog (Drosophila),expressed sequence AA407558,microtubule-associated protein 1 light chain 3,RIKEN cDNA 2310057H16 gene,RIKEN cDNA 1110038D17 gene,RIKEN cDNA 1200014P03 gene,RIKEN cDNA D230016N13 gene,histone deacetylase 6,expressed sequence AI449432,nuclear distribution gene E-like homolog 1 (A. nidulans),nuclear distribution gene E-like homolog 1 (A. nidulans) |
| 8 | microtubule-based process | 10 | 119 | 77 | 2164 | 0.13 | 0.055 | 2.362 | 0.008 | 104683\_at,160287\_at,160461\_f\_at,95119\_at,98461\_at,160140\_at,104471\_at,100939\_at,98882\_s\_at,98884\_r\_at | expressed sequence AA407558,microtubule-associated protein 1 light chain 3,RIKEN cDNA 2310057H16 gene,RIKEN cDNA 1110038D17 gene,RIKEN cDNA 1200014P03 gene,RIKEN cDNA D230016N13 gene,histone deacetylase 6,expressed sequence AI449432,nuclear distribution gene E-like homolog 1 (A. nidulans),nuclear distribution gene E-like homolog 1 (A. nidulans) |
| 9 | microtubule cytoskeleton organization and biogenesis | 3 | 12 | 41 | 911 | 0.073 | 0.013 | 5.556 | 0.014 | 104683\_at,160140\_at,104471\_at | expressed sequence AA407558,RIKEN cDNA D230016N13 gene,histone deacetylase 6 |
| 9 | microtubule-based movement | 5 | 38 | 41 | 911 | 0.122 | 0.042 | 2.924 | 0.024 | 100939\_at,104683\_at,160461\_f\_at,98882\_s\_at,98884\_r\_at | expressed sequence AI449432,expressed sequence AA407558,RIKEN cDNA 2310057H16 gene,nuclear distribution gene E-like homolog 1 (A. nidulans),nuclear distribution gene E-like homolog 1 (A. nidulans) |
| 10 | axon cargo transport | 2 | 4 | 14 | 197 | 0.143 | 0.02 | 7.037 | 0.026 | 98882\_s\_at,98884\_r\_at | nuclear distribution gene E-like homolog 1 (A. nidulans),nuclear distribution gene E-like homolog 1 (A. nidulans) |
| 5 | nuclear organization and biogenesis | 8 | 112 | 409 | 11544 | 0.02 | 0.01 | 2.016 | 0.045 | 100616\_at,93701\_at,104471\_at,98534\_at,94011\_at,95573\_at,95574\_f\_at,95502\_at | centromere autoantigen A,SWI/SNF related, matrix associated, actin dependent regulator of chromatin, subfamily a, member 5,histone deacetylase 6,Sin3-associated polypeptide 18,RIKEN cDNA 2310022K01 gene,bromodomain adjacent to zinc finger domain, 2A,CGG triplet repeat binding protein 1,sirtuin 2 (silent mating type information regulation 2, homolog) 2 (S. cerevisiae) |
| 6 | chromosome organization and biogenesis (sensu Eukarya) | 8 | 108 | 354 | 9498 | 0.023 | 0.011 | 1.988 | 0.048 | 100616\_at,93701\_at,104471\_at,98534\_at,94011\_at,95573\_at,95574\_f\_at,95502\_at | centromere autoantigen A,SWI/SNF related, matrix associated, actin dependent regulator of chromatin, subfamily a, member 5,histone deacetylase 6,Sin3-associated polypeptide 18,RIKEN cDNA 2310022K01 gene,bromodomain adjacent to zinc finger domain, 2A,CGG triplet repeat binding protein 1,sirtuin 2 (silent mating type information regulation 2, homolog) 2 (S. cerevisiae) |
| 7 | establishment and/or maintenance of chromatin architecture | 8 | 80 | 209 | 6246 | 0.038 | 0.013 | 2.988 | 0.005 | 93701\_at,100616\_at,104471\_at,98534\_at,94011\_at,95573\_at,95574\_f\_at,95502\_at | SWI/SNF related, matrix associated, actin dependent regulator of chromatin, subfamily a, member 5,centromere autoantigen A,histone deacetylase 6,Sin3-associated polypeptide 18,RIKEN cDNA 2310022K01 gene,bromodomain adjacent to zinc finger domain, 2A,CGG triplet repeat binding protein 1,sirtuin 2 (silent mating type information regulation 2, homolog) 2 (S. cerevisiae) |
| 8 | chromatin modification | 7 | 36 | 77 | 2164 | 0.091 | 0.017 | 5.463 | 0 | 104471\_at,98534\_at,93701\_at,94011\_at,95573\_at,95574\_f\_at,95502\_at | histone deacetylase 6,Sin3-associated polypeptide 18,SWI/SNF related, matrix associated, actin dependent regulator of chromatin, subfamily a, member 5,RIKEN cDNA 2310022K01 gene,bromodomain adjacent to zinc finger domain, 2A,CGG triplet repeat binding protein 1,sirtuin 2 (silent mating type information regulation 2, homolog) 2 (S. cerevisiae) |
| 9 | non-covalent chromatin modification | 5 | 13 | 41 | 911 | 0.122 | 0.014 | 8.546 | 0 | 93701\_at,94011\_at,95573\_at,95574\_f\_at,95502\_at | SWI/SNF related, matrix associated, actin dependent regulator of chromatin, subfamily a, member 5,RIKEN cDNA 2310022K01 gene,bromodomain adjacent to zinc finger domain, 2A,CGG triplet repeat binding protein 1,sirtuin 2 (silent mating type information regulation 2, homolog) 2 (S. cerevisiae) |
| 10 | chromatin modeling | 5 | 13 | 14 | 197 | 0.357 | 0.066 | 5.412 | 0.001 | 93701\_at,94011\_at,95573\_at,95574\_f\_at,95502\_at | SWI/SNF related, matrix associated, actin dependent regulator of chromatin, subfamily a, member 5,RIKEN cDNA 2310022K01 gene,bromodomain adjacent to zinc finger domain, 2A,CGG triplet repeat binding protein 1,sirtuin 2 (silent mating type information regulation 2, homolog) 2 (S. cerevisiae) |
| 7 | protein targeting | 8 | 101 | 209 | 6246 | 0.038 | 0.016 | 2.367 | 0.019 | 101036\_at,103704\_at,104453\_at,94012\_at,95023\_at,95444\_at,101079\_at,104150\_at | translocase of outer mitochondrial membrane 20 homolog (yeast),RIKEN cDNA 2010305K11 gene,RIKEN cDNA 2310079P12 gene,translocase of inner mitochondrial membrane 13 homolog a (yeast),hypothetical protein LOC214597,RIKEN cDNA 4930579A11 gene,nuclear RNA export factor 1 homolog (S. cerevisiae),RIKEN cDNA 2810008P14 gene |
| 5 | lipid transport | 5 | 40 | 409 | 11544 | 0.012 | 0.003 | 3.522 | 0.013 | 103314\_at,160832\_at,161785\_f\_at,95607\_at,96534\_at | DNA segment, Chr 13, ERATO Doi 275, expressed,low density lipoprotein receptor,hypothetical protein MGC28888,START domain containing 3,very low density lipoprotein receptor |
| 7 | protein targeting | 8 | 101 | 209 | 6246 | 0.038 | 0.016 | 2.367 | 0.019 | 101036\_at,103704\_at,104453\_at,94012\_at,95023\_at,95444\_at,101079\_at,104150\_at | translocase of outer mitochondrial membrane 20 homolog (yeast),RIKEN cDNA 2010305K11 gene,RIKEN cDNA 2310079P12 gene,translocase of inner mitochondrial membrane 13 homolog a (yeast),hypothetical protein LOC214597,RIKEN cDNA 4930579A11 gene,nuclear RNA export factor 1 homolog (S. cerevisiae),RIKEN cDNA 2810008P14 gene |
| 3 | circulation | 5 | 40 | 415 | 10726 | 0.012 | 0.004 | 3.231 | 0.018 | 93193\_at,95355\_at,92991\_at,92992\_i\_at,92993\_r\_at | adrenergic receptor, beta 2,angiotensin II, type I receptor-associated protein,trans-acting transcription factor 4,RIKEN cDNA 5730497N03 gene,RIKEN cDNA 5730497N03 gene |
| 4 | regulation of heart rate | 3 | 19 | 482 | 13100 | 0.006 | 0.001 | 4.29 | 0.031 | 92991\_at,92992\_i\_at,92993\_r\_at | trans-acting transcription factor 4,RIKEN cDNA 5730497N03 gene,RIKEN cDNA 5730497N03 gene |
| 6 | tetrahydrofolate metabolism | 1 | 1 | 354 | 9498 | 0.003 | 0 | 25.636 | 0.037 | 101844\_at | pipecolic acid oxidase |
| 6 | proteoglycan biosynthesis | 2 | 4 | 354 | 9498 | 0.006 | 0 | 13.452 | 0.008 | 101186\_at,92400\_at | per-pentamer repeat gene,N-deacetylase/N-sulfotransferase (heparan glucosaminyl) 2 |
| 5 | macromolecule biosynthesis | 22 | 322 | 409 | 11544 | 0.054 | 0.028 | 1.929 | 0.002 | 101697\_f\_at,102101\_f\_at,104048\_at,104144\_at,160283\_at,160977\_at,161667\_r\_at,93975\_at,94484\_at,95501\_at,96300\_f\_at,96577\_i\_at,96578\_r\_at,96845\_at,97083\_at,100136\_at,96784\_at,93315\_at,94818\_at,97936\_at,102980\_at,93852\_at | NA,proteolipid protein (myelin),cysteinyl-tRNA synthetase,GTP binding protein 2,RIKEN cDNA 2410005K20 gene,Rho guanine nucleotide exchange factor (GEF) 5,RIKEN cDNA 1110001M20 gene,RIKEN cDNA 1300002F13 gene,Hbs1-like (S. cerevisiae),RIKEN cDNA 2410001C21 gene,ribosomal protein S27,RIKEN cDNA 4932431F02 gene,RIKEN cDNA 4932431F02 gene,RIKEN cDNA 2310024J23 gene,DNA segment, Chr 2, ERATO Doi 303, expressed,lysosomal membrane glycoprotein 2,RIKEN cDNA 2900037I21 gene,mitogen activated protein kinase kinase 3,O-linked N-acetylglucosamine (GlcNAc) transferase (UDP-N-acetylglucosamine:polypeptide-N-acetylglucosaminyl transferase),RIKEN cDNA 2810407L07 gene,N-myristoyltransferase 1,myocyte enhancer factor 2A |
| 6 | protein biosynthesis | 22 | 322 | 354 | 9498 | 0.062 | 0.034 | 1.833 | 0.004 | 101697\_f\_at,102101\_f\_at,104048\_at,104144\_at,160283\_at,160977\_at,161667\_r\_at,93975\_at,94484\_at,95501\_at,96300\_f\_at,96577\_i\_at,96578\_r\_at,96845\_at,97083\_at,100136\_at,96784\_at,93315\_at,94818\_at,97936\_at,102980\_at,93852\_at | NA,proteolipid protein (myelin),cysteinyl-tRNA synthetase,GTP binding protein 2,RIKEN cDNA 2410005K20 gene,Rho guanine nucleotide exchange factor (GEF) 5,RIKEN cDNA 1110001M20 gene,RIKEN cDNA 1300002F13 gene,Hbs1-like (S. cerevisiae),RIKEN cDNA 2410001C21 gene,ribosomal protein S27,RIKEN cDNA 4932431F02 gene,RIKEN cDNA 4932431F02 gene,RIKEN cDNA 2310024J23 gene,DNA segment, Chr 2, ERATO Doi 303, expressed,lysosomal membrane glycoprotein 2,RIKEN cDNA 2900037I21 gene,mitogen activated protein kinase kinase 3,O-linked N-acetylglucosamine (GlcNAc) transferase (UDP-N-acetylglucosamine:polypeptide-N-acetylglucosaminyl transferase),RIKEN cDNA 2810407L07 gene,N-myristoyltransferase 1,myocyte enhancer factor 2A |
| 8 | cysteinyl-tRNA aminoacylation | 1 | 1 | 77 | 2164 | 0.013 | 0 | 28.239 | 0.036 | 104048\_at | cysteinyl-tRNA synthetase |
| 9 | O-linked glycosylation | 2 | 4 | 41 | 911 | 0.049 | 0.004 | 11.112 | 0.011 | 94818\_at,97936\_at | O-linked N-acetylglucosamine (GlcNAc) transferase (UDP-N-acetylglucosamine:polypeptide-N-acetylglucosaminyl transferase),RIKEN cDNA 2810407L07 gene |
| 7 | translational initiation | 4 | 34 | 209 | 6246 | 0.019 | 0.005 | 3.518 | 0.026 | 93975\_at,96845\_at,97083\_at,101697\_f\_at | RIKEN cDNA 1300002F13 gene,RIKEN cDNA 2310024J23 gene,DNA segment, Chr 2, ERATO Doi 303, expressed,NA |
| 6 | proteoglycan biosynthesis | 2 | 4 | 354 | 9498 | 0.006 | 0 | 13.452 | 0.008 | 101186\_at,92400\_at | per-pentamer repeat gene,N-deacetylase/N-sulfotransferase (heparan glucosaminyl) 2 |
| 7 | mRNA catabolism | 1 | 1 | 209 | 6246 | 0.005 | 0 | 29.875 | 0.033 | 96650\_at | AU RNA binding protein/enoyl-coenzyme A hydratase |
| 8 | mRNA catabolism, deadenylation-dependent | 1 | 1 | 77 | 2164 | 0.013 | 0 | 28.239 | 0.036 | 96650\_at | AU RNA binding protein/enoyl-coenzyme A hydratase |
| 5 | steroid metabolism | 7 | 74 | 409 | 11544 | 0.017 | 0.006 | 2.669 | 0.016 | 103314\_at,160832\_at,96534\_at,104285\_at,102991\_s\_at,161785\_f\_at,95607\_at | DNA segment, Chr 13, ERATO Doi 275, expressed,low density lipoprotein receptor,very low density lipoprotein receptor,3-hydroxy-3-methylglutaryl-Coenzyme A reductase,procollagen, type XI, alpha 2,hypothetical protein MGC28888,START domain containing 3 |
| 4 | nucleobase, nucleoside, nucleotide and nucleic acid metabolism | 73 | 1530 | 482 | 13100 | 0.151 | 0.117 | 1.297 | 0.011 | 100606\_at,95694\_at,97897\_at,93701\_at,100616\_at,104471\_at,98534\_at,94011\_at,95573\_at,95574\_f\_at,95502\_at,160174\_at,162228\_f\_at,100030\_at,98435\_at,96650\_at,104219\_f\_at,95081\_at,101079\_at,160182\_at,103545\_at,101889\_s\_at,101943\_at,95536\_at,104701\_at,100094\_at,100486\_at,100939\_at,101186\_at,102024\_at,102242\_at,102382\_at,102580\_r\_at,102895\_at,103015\_at,103259\_at,103504\_at,104714\_at,160220\_at,160396\_at,160495\_at,160781\_r\_at,160834\_at,161113\_at,161187\_f\_at,161333\_f\_at,162114\_f\_at,162204\_r\_at,92233\_at,92300\_at,92737\_at,92991\_at,92992\_i\_at,92993\_r\_at,94331\_at,94397\_at,94689\_at,95521\_s\_at,96196\_i\_at,96197\_f\_at,96481\_at,96817\_at,97118\_at,97497\_at,98002\_at,98465\_f\_at,98767\_at,98818\_at,99100\_at,99103\_at,99665\_at,101014\_at,92339\_at | prion protein,topoisomerase (DNA) I,expressed sequence C78339,SWI/SNF related, matrix associated, actin dependent regulator of chromatin, subfamily a, member 5,centromere autoantigen A,histone deacetylase 6,Sin3-associated polypeptide 18,RIKEN cDNA 2310022K01 gene,bromodomain adjacent to zinc finger domain, 2A,CGG triplet repeat binding protein 1,sirtuin 2 (silent mating type information regulation 2, homolog) 2 (S. cerevisiae),RIKEN cDNA 0610041O14 gene,START domain containing 3,uridine phosphorylase,adenylosuccinate synthetase 1, muscle,AU RNA binding protein/enoyl-coenzyme A hydratase,poly(rC) binding protein 2,RIKEN cDNA 2310032N20 gene,nuclear RNA export factor 1 homolog (S. cerevisiae),splicing factor, arginine/serine-rich 6,RIKEN cDNA 2610019E17 gene,RAR-related orphan receptor alpha,transcription elongation factor B (SIII), polypeptide 3,transcription elongation factor B (SIII), polypeptide 3,basic helix-loop-helix domain containing, class B2,suppressor of Ty 5 homolog (S. cerevisiae),enhancer of zeste homolog 1 (Drosophila),expressed sequence AI449432,per-pentamer repeat gene,nuclear receptor coactivator 3,period homolog 3 (Drosophila),aryl hydrocarbon receptor nuclear translocator-like,homeo box A6,RIKEN cDNA 4921518A06 gene,B-cell leukemia/lymphoma 6,growth factor independent 1,single-stranded DNA binding protein 2,expressed sequence AA959601,zinc finger protein 110,RIKEN cDNA 0610013I17 gene,aryl-hydrocarbon receptor,NA,RIKEN cDNA 1110032C13 gene,estrogen receptor 1 (alpha),RIKEN cDNA 5730589K01 gene,DNA segment, Chr 1, ERATO Doi 161, expressed,Trf (TATA binding protein-related factor)-proximal protein homolog (Drosophila),Notch gene homolog 1 (Drosophila),RIKEN cDNA 1810007M14 gene,max binding protein,interferon regulatory factor 4,trans-acting transcription factor 4,RIKEN cDNA 5730497N03 gene,RIKEN cDNA 5730497N03 gene,signal transducer and activator of transcription 6,RIKEN cDNA 1200014O24 gene,expressed sequence C79248,Zinc finger protein 68,RIKEN cDNA 5730589K01 gene,RIKEN cDNA 5730589K01 gene,expressed sequence C80638,RIKEN cDNA 2700067D09 gene,expressed sequence AI159700,Notch gene homolog 1 (Drosophila),interferon consensus sequence binding protein 1,interferon activated gene 204,YY1 transcription factor,nuclear receptor subfamily 3, group C, member 1,signal transducer and activator of transcription 3,interferon regulatory factor 3,special AT-rich sequence binding protein 1,interferon (alpha and beta) receptor 2,TATA box binding protein (Tbp)-associated factor, RNA polymerase I, A |
| 6 | DNA packaging | 9 | 93 | 354 | 9498 | 0.025 | 0.01 | 2.597 | 0.008 | 97897\_at,93701\_at,100616\_at,104471\_at,98534\_at,94011\_at,95573\_at,95574\_f\_at,95502\_at | expressed sequence C78339,SWI/SNF related, matrix associated, actin dependent regulator of chromatin, subfamily a, member 5,centromere autoantigen A,histone deacetylase 6,Sin3-associated polypeptide 18,RIKEN cDNA 2310022K01 gene,bromodomain adjacent to zinc finger domain, 2A,CGG triplet repeat binding protein 1,sirtuin 2 (silent mating type information regulation 2, homolog) 2 (S. cerevisiae) |
| 7 | establishment and/or maintenance of chromatin architecture | 8 | 80 | 209 | 6246 | 0.038 | 0.013 | 2.988 | 0.005 | 93701\_at,100616\_at,104471\_at,98534\_at,94011\_at,95573\_at,95574\_f\_at,95502\_at | SWI/SNF related, matrix associated, actin dependent regulator of chromatin, subfamily a, member 5,centromere autoantigen A,histone deacetylase 6,Sin3-associated polypeptide 18,RIKEN cDNA 2310022K01 gene,bromodomain adjacent to zinc finger domain, 2A,CGG triplet repeat binding protein 1,sirtuin 2 (silent mating type information regulation 2, homolog) 2 (S. cerevisiae) |
| 8 | chromatin modification | 7 | 36 | 77 | 2164 | 0.091 | 0.017 | 5.463 | 0 | 104471\_at,98534\_at,93701\_at,94011\_at,95573\_at,95574\_f\_at,95502\_at | histone deacetylase 6,Sin3-associated polypeptide 18,SWI/SNF related, matrix associated, actin dependent regulator of chromatin, subfamily a, member 5,RIKEN cDNA 2310022K01 gene,bromodomain adjacent to zinc finger domain, 2A,CGG triplet repeat binding protein 1,sirtuin 2 (silent mating type information regulation 2, homolog) 2 (S. cerevisiae) |
| 9 | non-covalent chromatin modification | 5 | 13 | 41 | 911 | 0.122 | 0.014 | 8.546 | 0 | 93701\_at,94011\_at,95573\_at,95574\_f\_at,95502\_at | SWI/SNF related, matrix associated, actin dependent regulator of chromatin, subfamily a, member 5,RIKEN cDNA 2310022K01 gene,bromodomain adjacent to zinc finger domain, 2A,CGG triplet repeat binding protein 1,sirtuin 2 (silent mating type information regulation 2, homolog) 2 (S. cerevisiae) |
| 10 | chromatin modeling | 5 | 13 | 14 | 197 | 0.357 | 0.066 | 5.412 | 0.001 | 93701\_at,94011\_at,95573\_at,95574\_f\_at,95502\_at | SWI/SNF related, matrix associated, actin dependent regulator of chromatin, subfamily a, member 5,RIKEN cDNA 2310022K01 gene,bromodomain adjacent to zinc finger domain, 2A,CGG triplet repeat binding protein 1,sirtuin 2 (silent mating type information regulation 2, homolog) 2 (S. cerevisiae) |
| 7 | mRNA catabolism | 1 | 1 | 209 | 6246 | 0.005 | 0 | 29.875 | 0.033 | 96650\_at | AU RNA binding protein/enoyl-coenzyme A hydratase |
| 8 | mRNA catabolism, deadenylation-dependent | 1 | 1 | 77 | 2164 | 0.013 | 0 | 28.239 | 0.036 | 96650\_at | AU RNA binding protein/enoyl-coenzyme A hydratase |
| 5 | transcription | 59 | 1086 | 409 | 11544 | 0.144 | 0.094 | 1.533 | 0.001 | 101889\_s\_at,101943\_at,160174\_at,95536\_at,104701\_at,95502\_at,100094\_at,100486\_at,100939\_at,101186\_at,102024\_at,102242\_at,102382\_at,102580\_r\_at,102895\_at,103015\_at,103259\_at,103504\_at,104471\_at,104714\_at,160220\_at,160396\_at,160495\_at,160781\_r\_at,160834\_at,161113\_at,161187\_f\_at,161333\_f\_at,162114\_f\_at,162204\_r\_at,92233\_at,92300\_at,92737\_at,92991\_at,92992\_i\_at,92993\_r\_at,94331\_at,94397\_at,94689\_at,95521\_s\_at,95573\_at,95574\_f\_at,96196\_i\_at,96197\_f\_at,96481\_at,96817\_at,97118\_at,97497\_at,98002\_at,98465\_f\_at,98534\_at,98767\_at,98818\_at,99100\_at,99103\_at,99665\_at,101014\_at,92339\_at,95081\_at | RAR-related orphan receptor alpha,transcription elongation factor B (SIII), polypeptide 3,RIKEN cDNA 0610041O14 gene,transcription elongation factor B (SIII), polypeptide 3,basic helix-loop-helix domain containing, class B2,sirtuin 2 (silent mating type information regulation 2, homolog) 2 (S. cerevisiae),suppressor of Ty 5 homolog (S. cerevisiae),enhancer of zeste homolog 1 (Drosophila),expressed sequence AI449432,per-pentamer repeat gene,nuclear receptor coactivator 3,period homolog 3 (Drosophila),aryl hydrocarbon receptor nuclear translocator-like,homeo box A6,RIKEN cDNA 4921518A06 gene,B-cell leukemia/lymphoma 6,growth factor independent 1,single-stranded DNA binding protein 2,histone deacetylase 6,expressed sequence AA959601,zinc finger protein 110,RIKEN cDNA 0610013I17 gene,aryl-hydrocarbon receptor,NA,RIKEN cDNA 1110032C13 gene,estrogen receptor 1 (alpha),RIKEN cDNA 5730589K01 gene,DNA segment, Chr 1, ERATO Doi 161, expressed,Trf (TATA binding protein-related factor)-proximal protein homolog (Drosophila),Notch gene homolog 1 (Drosophila),RIKEN cDNA 1810007M14 gene,max binding protein,interferon regulatory factor 4,trans-acting transcription factor 4,RIKEN cDNA 5730497N03 gene,RIKEN cDNA 5730497N03 gene,signal transducer and activator of transcription 6,RIKEN cDNA 1200014O24 gene,expressed sequence C79248,Zinc finger protein 68,bromodomain adjacent to zinc finger domain, 2A,CGG triplet repeat binding protein 1,RIKEN cDNA 5730589K01 gene,RIKEN cDNA 5730589K01 gene,expressed sequence C80638,RIKEN cDNA 2700067D09 gene,expressed sequence AI159700,Notch gene homolog 1 (Drosophila),interferon consensus sequence binding protein 1,interferon activated gene 204,Sin3-associated polypeptide 18,YY1 transcription factor,nuclear receptor subfamily 3, group C, member 1,signal transducer and activator of transcription 3,interferon regulatory factor 3,special AT-rich sequence binding protein 1,interferon (alpha and beta) receptor 2,TATA box binding protein (Tbp)-associated factor, RNA polymerase I, A,RIKEN cDNA 2310032N20 gene |
| 6 | regulation of transcription | 54 | 1026 | 354 | 9498 | 0.153 | 0.108 | 1.412 | 0.005 | 104701\_at,95502\_at,100094\_at,100486\_at,100939\_at,101186\_at,101889\_s\_at,102024\_at,102242\_at,102382\_at,102580\_r\_at,102895\_at,103015\_at,103259\_at,103504\_at,104471\_at,104714\_at,160220\_at,160396\_at,160495\_at,160781\_r\_at,160834\_at,161113\_at,161187\_f\_at,161333\_f\_at,162114\_f\_at,162204\_r\_at,92233\_at,92300\_at,92737\_at,92991\_at,92992\_i\_at,92993\_r\_at,94331\_at,94397\_at,94689\_at,95521\_s\_at,95573\_at,95574\_f\_at,96196\_i\_at,96197\_f\_at,96481\_at,96817\_at,97118\_at,97497\_at,98002\_at,98465\_f\_at,98534\_at,98767\_at,98818\_at,99100\_at,99103\_at,99665\_at,101014\_at | basic helix-loop-helix domain containing, class B2,sirtuin 2 (silent mating type information regulation 2, homolog) 2 (S. cerevisiae),suppressor of Ty 5 homolog (S. cerevisiae),enhancer of zeste homolog 1 (Drosophila),expressed sequence AI449432,per-pentamer repeat gene,RAR-related orphan receptor alpha,nuclear receptor coactivator 3,period homolog 3 (Drosophila),aryl hydrocarbon receptor nuclear translocator-like,homeo box A6,RIKEN cDNA 4921518A06 gene,B-cell leukemia/lymphoma 6,growth factor independent 1,single-stranded DNA binding protein 2,histone deacetylase 6,expressed sequence AA959601,zinc finger protein 110,RIKEN cDNA 0610013I17 gene,aryl-hydrocarbon receptor,NA,RIKEN cDNA 1110032C13 gene,estrogen receptor 1 (alpha),RIKEN cDNA 5730589K01 gene,DNA segment, Chr 1, ERATO Doi 161, expressed,Trf (TATA binding protein-related factor)-proximal protein homolog (Drosophila),Notch gene homolog 1 (Drosophila),RIKEN cDNA 1810007M14 gene,max binding protein,interferon regulatory factor 4,trans-acting transcription factor 4,RIKEN cDNA 5730497N03 gene,RIKEN cDNA 5730497N03 gene,signal transducer and activator of transcription 6,RIKEN cDNA 1200014O24 gene,expressed sequence C79248,Zinc finger protein 68,bromodomain adjacent to zinc finger domain, 2A,CGG triplet repeat binding protein 1,RIKEN cDNA 5730589K01 gene,RIKEN cDNA 5730589K01 gene,expressed sequence C80638,RIKEN cDNA 2700067D09 gene,expressed sequence AI159700,Notch gene homolog 1 (Drosophila),interferon consensus sequence binding protein 1,interferon activated gene 204,Sin3-associated polypeptide 18,YY1 transcription factor,nuclear receptor subfamily 3, group C, member 1,signal transducer and activator of transcription 3,interferon regulatory factor 3,special AT-rich sequence binding protein 1,interferon (alpha and beta) receptor 2 |
| 7 | regulation of transcription, DNA-dependent | 54 | 1013 | 209 | 6246 | 0.258 | 0.162 | 1.593 | 0 | 100094\_at,100486\_at,100939\_at,101186\_at,101889\_s\_at,102024\_at,102242\_at,102382\_at,102580\_r\_at,102895\_at,103015\_at,103259\_at,103504\_at,104471\_at,104701\_at,104714\_at,160220\_at,160396\_at,160495\_at,160781\_r\_at,160834\_at,161113\_at,161187\_f\_at,161333\_f\_at,162114\_f\_at,162204\_r\_at,92233\_at,92300\_at,92737\_at,92991\_at,92992\_i\_at,92993\_r\_at,94331\_at,94397\_at,94689\_at,95502\_at,95521\_s\_at,95573\_at,95574\_f\_at,96196\_i\_at,96197\_f\_at,96481\_at,96817\_at,97118\_at,97497\_at,98002\_at,98465\_f\_at,98534\_at,98767\_at,98818\_at,99100\_at,99103\_at,99665\_at,101014\_at | suppressor of Ty 5 homolog (S. cerevisiae),enhancer of zeste homolog 1 (Drosophila),expressed sequence AI449432,per-pentamer repeat gene,RAR-related orphan receptor alpha,nuclear receptor coactivator 3,period homolog 3 (Drosophila),aryl hydrocarbon receptor nuclear translocator-like,homeo box A6,RIKEN cDNA 4921518A06 gene,B-cell leukemia/lymphoma 6,growth factor independent 1,single-stranded DNA binding protein 2,histone deacetylase 6,basic helix-loop-helix domain containing, class B2,expressed sequence AA959601,zinc finger protein 110,RIKEN cDNA 0610013I17 gene,aryl-hydrocarbon receptor,NA,RIKEN cDNA 1110032C13 gene,estrogen receptor 1 (alpha),RIKEN cDNA 5730589K01 gene,DNA segment, Chr 1, ERATO Doi 161, expressed,Trf (TATA binding protein-related factor)-proximal protein homolog (Drosophila),Notch gene homolog 1 (Drosophila),RIKEN cDNA 1810007M14 gene,max binding protein,interferon regulatory factor 4,trans-acting transcription factor 4,RIKEN cDNA 5730497N03 gene,RIKEN cDNA 5730497N03 gene,signal transducer and activator of transcription 6,RIKEN cDNA 1200014O24 gene,expressed sequence C79248,sirtuin 2 (silent mating type information regulation 2, homolog) 2 (S. cerevisiae),Zinc finger protein 68,bromodomain adjacent to zinc finger domain, 2A,CGG triplet repeat binding protein 1,RIKEN cDNA 5730589K01 gene,RIKEN cDNA 5730589K01 gene,expressed sequence C80638,RIKEN cDNA 2700067D09 gene,expressed sequence AI159700,Notch gene homolog 1 (Drosophila),interferon consensus sequence binding protein 1,interferon activated gene 204,Sin3-associated polypeptide 18,YY1 transcription factor,nuclear receptor subfamily 3, group C, member 1,signal transducer and activator of transcription 3,interferon regulatory factor 3,special AT-rich sequence binding protein 1,interferon (alpha and beta) receptor 2 |
| 6 | transcription, DNA-dependent | 56 | 1046 | 354 | 9498 | 0.158 | 0.11 | 1.436 | 0.003 | 100094\_at,100486\_at,100939\_at,101186\_at,101889\_s\_at,102024\_at,102242\_at,102382\_at,102580\_r\_at,102895\_at,103015\_at,103259\_at,103504\_at,104471\_at,104701\_at,104714\_at,160220\_at,160396\_at,160495\_at,160781\_r\_at,160834\_at,161113\_at,161187\_f\_at,161333\_f\_at,162114\_f\_at,162204\_r\_at,92233\_at,92300\_at,92737\_at,92991\_at,92992\_i\_at,92993\_r\_at,94331\_at,94397\_at,94689\_at,95502\_at,95521\_s\_at,95573\_at,95574\_f\_at,96196\_i\_at,96197\_f\_at,96481\_at,96817\_at,97118\_at,97497\_at,98002\_at,98465\_f\_at,98534\_at,98767\_at,98818\_at,99100\_at,99103\_at,99665\_at,101014\_at,92339\_at,95081\_at | suppressor of Ty 5 homolog (S. cerevisiae),enhancer of zeste homolog 1 (Drosophila),expressed sequence AI449432,per-pentamer repeat gene,RAR-related orphan receptor alpha,nuclear receptor coactivator 3,period homolog 3 (Drosophila),aryl hydrocarbon receptor nuclear translocator-like,homeo box A6,RIKEN cDNA 4921518A06 gene,B-cell leukemia/lymphoma 6,growth factor independent 1,single-stranded DNA binding protein 2,histone deacetylase 6,basic helix-loop-helix domain containing, class B2,expressed sequence AA959601,zinc finger protein 110,RIKEN cDNA 0610013I17 gene,aryl-hydrocarbon receptor,NA,RIKEN cDNA 1110032C13 gene,estrogen receptor 1 (alpha),RIKEN cDNA 5730589K01 gene,DNA segment, Chr 1, ERATO Doi 161, expressed,Trf (TATA binding protein-related factor)-proximal protein homolog (Drosophila),Notch gene homolog 1 (Drosophila),RIKEN cDNA 1810007M14 gene,max binding protein,interferon regulatory factor 4,trans-acting transcription factor 4,RIKEN cDNA 5730497N03 gene,RIKEN cDNA 5730497N03 gene,signal transducer and activator of transcription 6,RIKEN cDNA 1200014O24 gene,expressed sequence C79248,sirtuin 2 (silent mating type information regulation 2, homolog) 2 (S. cerevisiae),Zinc finger protein 68,bromodomain adjacent to zinc finger domain, 2A,CGG triplet repeat binding protein 1,RIKEN cDNA 5730589K01 gene,RIKEN cDNA 5730589K01 gene,expressed sequence C80638,RIKEN cDNA 2700067D09 gene,expressed sequence AI159700,Notch gene homolog 1 (Drosophila),interferon consensus sequence binding protein 1,interferon activated gene 204,Sin3-associated polypeptide 18,YY1 transcription factor,nuclear receptor subfamily 3, group C, member 1,signal transducer and activator of transcription 3,interferon regulatory factor 3,special AT-rich sequence binding protein 1,interferon (alpha and beta) receptor 2,TATA box binding protein (Tbp)-associated factor, RNA polymerase I, A,RIKEN cDNA 2310032N20 gene |
| 7 | regulation of transcription, DNA-dependent | 54 | 1013 | 209 | 6246 | 0.258 | 0.162 | 1.593 | 0 | 100094\_at,100486\_at,100939\_at,101186\_at,101889\_s\_at,102024\_at,102242\_at,102382\_at,102580\_r\_at,102895\_at,103015\_at,103259\_at,103504\_at,104471\_at,104701\_at,104714\_at,160220\_at,160396\_at,160495\_at,160781\_r\_at,160834\_at,161113\_at,161187\_f\_at,161333\_f\_at,162114\_f\_at,162204\_r\_at,92233\_at,92300\_at,92737\_at,92991\_at,92992\_i\_at,92993\_r\_at,94331\_at,94397\_at,94689\_at,95502\_at,95521\_s\_at,95573\_at,95574\_f\_at,96196\_i\_at,96197\_f\_at,96481\_at,96817\_at,97118\_at,97497\_at,98002\_at,98465\_f\_at,98534\_at,98767\_at,98818\_at,99100\_at,99103\_at,99665\_at,101014\_at | suppressor of Ty 5 homolog (S. cerevisiae),enhancer of zeste homolog 1 (Drosophila),expressed sequence AI449432,per-pentamer repeat gene,RAR-related orphan receptor alpha,nuclear receptor coactivator 3,period homolog 3 (Drosophila),aryl hydrocarbon receptor nuclear translocator-like,homeo box A6,RIKEN cDNA 4921518A06 gene,B-cell leukemia/lymphoma 6,growth factor independent 1,single-stranded DNA binding protein 2,histone deacetylase 6,basic helix-loop-helix domain containing, class B2,expressed sequence AA959601,zinc finger protein 110,RIKEN cDNA 0610013I17 gene,aryl-hydrocarbon receptor,NA,RIKEN cDNA 1110032C13 gene,estrogen receptor 1 (alpha),RIKEN cDNA 5730589K01 gene,DNA segment, Chr 1, ERATO Doi 161, expressed,Trf (TATA binding protein-related factor)-proximal protein homolog (Drosophila),Notch gene homolog 1 (Drosophila),RIKEN cDNA 1810007M14 gene,max binding protein,interferon regulatory factor 4,trans-acting transcription factor 4,RIKEN cDNA 5730497N03 gene,RIKEN cDNA 5730497N03 gene,signal transducer and activator of transcription 6,RIKEN cDNA 1200014O24 gene,expressed sequence C79248,sirtuin 2 (silent mating type information regulation 2, homolog) 2 (S. cerevisiae),Zinc finger protein 68,bromodomain adjacent to zinc finger domain, 2A,CGG triplet repeat binding protein 1,RIKEN cDNA 5730589K01 gene,RIKEN cDNA 5730589K01 gene,expressed sequence C80638,RIKEN cDNA 2700067D09 gene,expressed sequence AI159700,Notch gene homolog 1 (Drosophila),interferon consensus sequence binding protein 1,interferon activated gene 204,Sin3-associated polypeptide 18,YY1 transcription factor,nuclear receptor subfamily 3, group C, member 1,signal transducer and activator of transcription 3,interferon regulatory factor 3,special AT-rich sequence binding protein 1,interferon (alpha and beta) receptor 2 |
| 7 | protein deubiquitination | 2 | 7 | 209 | 6246 | 0.01 | 0.001 | 8.545 | 0.021 | 99085\_at,99086\_g\_at | ubiquitin specific protease 3,ubiquitin specific protease 3 |
| 8 | protein polyubiquitination | 1 | 1 | 77 | 2164 | 0.013 | 0 | 28.239 | 0.036 | 104471\_at | histone deacetylase 6 |
| 8 | neutrophil chemotaxis | 1 | 1 | 77 | 2164 | 0.013 | 0 | 28.239 | 0.036 | 93199\_at | NA |
| 8 | neutrophil chemotaxis | 1 | 1 | 77 | 2164 | 0.013 | 0 | 28.239 | 0.036 | 93199\_at | NA |

  
