# Supplementary material for: Molecular Signatures of Proliferation and Quiescence in Hematopoietic Stem Cells
Source: PLoS Biol. 2004 Sep 28;2(10):e301. doi: 10.1371/journal.pbio.0020301 (PMC520599; doi:10.1371/journal.pbio.0020301)
Supplement: Table S36 — (52 KB HTML). [file pbio.0020301.st036.html]

   Full Tom Day 0   

# Full Tom Day 0

|  |  |  |  |  |  |  |  |  |  |  |
| --- | --- | --- | --- | --- | --- | --- | --- | --- | --- | --- |
| GOLevel | GOTerm | ProbeCount | ArrayCount | ListGOLevelCount | ArrayGoLevelCount | ListFq | ArrayFq | FoldChange | H-Pvalue | ProbeIds |
| 0 | Gene\_Ontology | 89 | NA | 89 | 0 | 1 | NA | NA | NA | 102356\_at,92558\_at,96886\_at,93705\_at,102286\_at,102884\_at,102021\_at,98254\_f\_at,102225\_at,102896\_at,96912\_s\_at,103392\_at,101426\_at,92672\_at,98418\_at,100582\_at,93714\_f\_at,95893\_at,96530\_at,103217\_at,160792\_at,93093\_at,95102\_at,98045\_s\_at,101072\_at,103990\_at,160901\_at,92644\_s\_at,92270\_at,100473\_at,98007\_at,94288\_at,98977\_at,160273\_at,104598\_at,92310\_at,92323\_at,103427\_at,104396\_at,104398\_at,104719\_at,160564\_at,95157\_at,97181\_f\_at,99451\_at,92256\_at,96186\_at,102933\_at,93682\_at,92248\_at,103832\_at,93500\_at,100533\_s\_at,101515\_at,160526\_s\_at,97817\_at,95133\_at,160547\_s\_at,160346\_at,97798\_at,161683\_r\_at,94834\_at,96732\_at,96848\_at,97336\_at,97943\_at,94948\_at,94345\_at,102209\_at,102955\_at,104562\_at,161211\_r\_at,96940\_at,98083\_at,160362\_at,93179\_at,94929\_at,98431\_at,103348\_at,98369\_f\_at,160104\_at,104362\_at,101897\_g\_at,93120\_f\_at,93907\_f\_at,98088\_at,100322\_at,102156\_f\_at,93584\_at |
| 1 | biological\_process | 89 | 6769 | 89 | 6769 | 1 | 1 | 1 | 1 | 102356\_at,92558\_at,96886\_at,93705\_at,102286\_at,102884\_at,102021\_at,98254\_f\_at,102225\_at,102896\_at,96912\_s\_at,103392\_at,101426\_at,92672\_at,98418\_at,100582\_at,93714\_f\_at,95893\_at,96530\_at,103217\_at,160792\_at,93093\_at,95102\_at,98045\_s\_at,101072\_at,103990\_at,160901\_at,92644\_s\_at,92270\_at,100473\_at,98007\_at,94288\_at,98977\_at,160273\_at,104598\_at,92310\_at,92323\_at,103427\_at,104396\_at,104398\_at,104719\_at,160564\_at,95157\_at,97181\_f\_at,99451\_at,92256\_at,96186\_at,102933\_at,93682\_at,92248\_at,103832\_at,93500\_at,100533\_s\_at,101515\_at,160526\_s\_at,97817\_at,95133\_at,160547\_s\_at,160346\_at,97798\_at,161683\_r\_at,94834\_at,96732\_at,96848\_at,97336\_at,97943\_at,94948\_at,94345\_at,102209\_at,102955\_at,104562\_at,161211\_r\_at,96940\_at,98083\_at,160362\_at,93179\_at,94929\_at,98431\_at,103348\_at,98369\_f\_at,160104\_at,104362\_at,101897\_g\_at,93120\_f\_at,93907\_f\_at,98088\_at,100322\_at,102156\_f\_at,93584\_at |
| 2 | cellular process | 47 | 3616 | 135 | 10540 | 0.348 | 0.343 | 1.015 | 0.483 | 102356\_at,92558\_at,96886\_at,93705\_at,102286\_at,102884\_at,102021\_at,98254\_f\_at,102225\_at,102896\_at,96912\_s\_at,103392\_at,101426\_at,92672\_at,98418\_at,100582\_at,93714\_f\_at,95893\_at,96530\_at,103217\_at,160792\_at,93093\_at,95102\_at,98045\_s\_at,101072\_at,103990\_at,160901\_at,92644\_s\_at,92270\_at,100473\_at,98007\_at,94288\_at,98977\_at,160273\_at,104598\_at,92310\_at,92323\_at,103427\_at,104396\_at,104398\_at,104719\_at,160564\_at,95157\_at,97181\_f\_at,99451\_at,92256\_at,96186\_at |
| 3 | cell communication | 19 | 1550 | 123 | 10726 | 0.154 | 0.145 | 1.069 | 0.415 | 102356\_at,92558\_at,96886\_at,93705\_at,102286\_at,102884\_at,102021\_at,98254\_f\_at,102225\_at,102896\_at,96912\_s\_at,103392\_at,101426\_at,92672\_at,98418\_at,100582\_at,93714\_f\_at,95893\_at,96530\_at |
| 4 | cell adhesion | 3 | 322 | 149 | 13100 | 0.02 | 0.025 | 0.819 | 0.713 | 102356\_at,92558\_at,96886\_at |
| 5 | cell-cell adhesion | 1 | 44 | 132 | 11544 | 0.008 | 0.004 | 1.99 | 0.398 | 92558\_at |
| 5 | cell-matrix adhesion | 1 | 50 | 132 | 11544 | 0.008 | 0.004 | 1.751 | 0.438 | 102356\_at |
| 4 | cell-cell signaling | 1 | 123 | 149 | 13100 | 0.007 | 0.009 | 0.715 | 0.757 | 93705\_at |
| 5 | transmission of nerve impulse | 1 | 82 | 132 | 11544 | 0.008 | 0.007 | 1.068 | 0.612 | 93705\_at |
| 6 | synaptic transmission | 1 | 80 | 92 | 9498 | 0.011 | 0.008 | 1.291 | 0.542 | 93705\_at |
| 4 | signal transduction | 16 | 1199 | 149 | 13100 | 0.107 | 0.092 | 1.173 | 0.288 | 102286\_at,102884\_at,102021\_at,98254\_f\_at,102225\_at,102896\_at,96912\_s\_at,103392\_at,101426\_at,92672\_at,102356\_at,98418\_at,100582\_at,93714\_f\_at,95893\_at,96530\_at |
| 5 | cell surface receptor linked signal transduction | 10 | 621 | 132 | 11544 | 0.076 | 0.054 | 1.408 | 0.173 | 102021\_at,98254\_f\_at,102225\_at,102896\_at,96912\_s\_at,103392\_at,101426\_at,92672\_at,102356\_at,98418\_at |
| 6 | enzyme linked receptor protein signaling pathway | 2 | 131 | 92 | 9498 | 0.022 | 0.014 | 1.577 | 0.363 | 102225\_at,102896\_at |
| 7 | transmembrane receptor protein tyrosine kinase signaling pathway | 2 | 61 | 71 | 6246 | 0.028 | 0.01 | 2.883 | 0.152 | 102225\_at,102896\_at |
| 6 | G-protein coupled receptor protein signaling pathway | 4 | 355 | 92 | 9498 | 0.043 | 0.037 | 1.163 | 0.452 | 96912\_s\_at,103392\_at,101426\_at,92672\_at |
| 7 | G-protein signaling, coupled to cyclic nucleotide second messenger | 1 | 33 | 71 | 6246 | 0.014 | 0.005 | 2.667 | 0.315 | 103392\_at |
| 8 | G-protein signaling, coupled to cAMP nucleotide second messenger | 1 | 27 | 18 | 2164 | 0.056 | 0.012 | 4.452 | 0.203 | 103392\_at |
| 9 | G-protein signaling, adenylate cyclase activating pathway | 1 | 17 | 5 | 911 | 0.2 | 0.019 | 10.718 | 0.09 | 103392\_at |
| 10 | adenylate cyclase activation | 1 | 8 | 2 | 197 | 0.5 | 0.041 | 12.312 | 0.08 | 103392\_at |
| 7 | G-protein signaling, coupled to IP3 second messenger (phospholipase C activating) | 1 | 13 | 71 | 6246 | 0.014 | 0.002 | 6.769 | 0.138 | 101426\_at |
| 8 | protein kinase C activation | 1 | 5 | 18 | 2164 | 0.056 | 0.002 | 24.052 | 0.041 | 101426\_at |
| 7 | neuropeptide signaling pathway | 1 | 45 | 71 | 6246 | 0.014 | 0.007 | 1.956 | 0.403 | 92672\_at |
| 6 | integrin-mediated signaling pathway | 1 | 45 | 92 | 9498 | 0.011 | 0.005 | 2.293 | 0.355 | 102356\_at |
| 6 | Wnt receptor signaling pathway | 1 | 30 | 92 | 9498 | 0.011 | 0.003 | 3.44 | 0.254 | 98418\_at |
| 7 | frizzled signaling pathway | 1 | 7 | 71 | 6246 | 0.014 | 0.001 | 12.571 | 0.077 | 98418\_at |
| 5 | intracellular signaling cascade | 10 | 485 | 132 | 11544 | 0.076 | 0.042 | 1.803 | 0.051 | 100582\_at,102286\_at,102884\_at,103392\_at,92672\_at,93714\_f\_at,95893\_at,98418\_at,102896\_at,96530\_at |
| 6 | protein kinase cascade | 1 | 50 | 92 | 9498 | 0.011 | 0.005 | 2.067 | 0.386 | 102896\_at |
| 7 | MAPKKK cascade | 1 | 18 | 71 | 6246 | 0.014 | 0.003 | 4.889 | 0.186 | 102896\_at |
| 6 | small GTPase mediated signal transduction | 3 | 135 | 92 | 9498 | 0.033 | 0.014 | 2.295 | 0.143 | 92672\_at,96530\_at,102896\_at |
| 7 | RAS protein signal transduction | 1 | 15 | 71 | 6246 | 0.014 | 0.002 | 5.867 | 0.158 | 102896\_at |
| 3 | cell death | 4 | 207 | 123 | 10726 | 0.033 | 0.019 | 1.685 | 0.214 | 103217\_at,160792\_at,93093\_at,95102\_at |
| 4 | programmed cell death | 4 | 192 | 149 | 13100 | 0.027 | 0.015 | 1.832 | 0.175 | 103217\_at,160792\_at,93093\_at,95102\_at |
| 5 | apoptosis | 4 | 192 | 132 | 11544 | 0.03 | 0.017 | 1.822 | 0.177 | 103217\_at,160792\_at,93093\_at,95102\_at |
| 6 | apoptotic program | 1 | 16 | 92 | 9498 | 0.011 | 0.002 | 6.47 | 0.144 | 95102\_at |
| 6 | induction of apoptosis | 2 | 28 | 92 | 9498 | 0.022 | 0.003 | 7.369 | 0.03 | 103217\_at,95102\_at |
| 3 | cell differentiation | 1 | 137 | 123 | 10726 | 0.008 | 0.013 | 0.637 | 0.796 | 98045\_s\_at |
| 4 | cellular morphogenesis during differentiation | 1 | 4 | 149 | 13100 | 0.007 | 0 | 21.645 | 0.045 | 98045\_s\_at |
| 3 | cell growth and/or maintenance | 29 | 2128 | 123 | 10726 | 0.236 | 0.198 | 1.188 | 0.175 | 101072\_at,102286\_at,103990\_at,160901\_at,92644\_s\_at,93714\_f\_at,92270\_at,100473\_at,98007\_at,94288\_at,98977\_at,160273\_at,104598\_at,92310\_at,92323\_at,103427\_at,104396\_at,104398\_at,104719\_at,160564\_at,93705\_at,95157\_at,97181\_f\_at,99451\_at,96530\_at,92256\_at,96186\_at,100582\_at,98045\_s\_at |
| 4 | cell growth | 2 | 51 | 149 | 13100 | 0.013 | 0.004 | 3.45 | 0.114 | 93714\_f\_at,92270\_at |
| 5 | regulation of cell growth | 2 | 38 | 132 | 11544 | 0.015 | 0.003 | 4.605 | 0.07 | 93714\_f\_at,92270\_at |
| 6 | negative regulation of cell growth | 1 | 2 | 92 | 9498 | 0.011 | 0 | 51.762 | 0.019 | 92270\_at |
| 4 | cell organization and biogenesis | 5 | 530 | 149 | 13100 | 0.034 | 0.04 | 0.829 | 0.726 | 100473\_at,98007\_at,94288\_at,98977\_at,160273\_at |
| 5 | cytoplasm organization and biogenesis | 2 | 380 | 132 | 11544 | 0.015 | 0.033 | 0.46 | 0.935 | 100473\_at,98007\_at |
| 6 | organelle organization and biogenesis | 1 | 318 | 92 | 9498 | 0.011 | 0.033 | 0.325 | 0.957 | 100473\_at |
| 7 | cytoskeleton organization and biogenesis | 1 | 262 | 71 | 6246 | 0.014 | 0.042 | 0.336 | 0.953 | 100473\_at |
| 8 | actin filament-based process | 1 | 42 | 18 | 2164 | 0.056 | 0.019 | 2.862 | 0.298 | 100473\_at |
| 9 | actin cytoskeleton organization and biogenesis | 1 | 42 | 5 | 911 | 0.2 | 0.046 | 4.338 | 0.211 | 100473\_at |
| 10 | actin filament organization | 1 | 16 | 2 | 197 | 0.5 | 0.081 | 6.156 | 0.156 | 100473\_at |
| 6 | ribosome biogenesis and assembly | 1 | 60 | 92 | 9498 | 0.011 | 0.006 | 1.72 | 0.443 | 98007\_at |
| 7 | ribosome biogenesis | 1 | 60 | 71 | 6246 | 0.014 | 0.01 | 1.465 | 0.498 | 98007\_at |
| 5 | nuclear organization and biogenesis | 3 | 112 | 132 | 11544 | 0.023 | 0.01 | 2.343 | 0.137 | 94288\_at,98977\_at,160273\_at |
| 6 | chromosome organization and biogenesis (sensu Eukarya) | 3 | 108 | 92 | 9498 | 0.033 | 0.011 | 2.868 | 0.087 | 94288\_at,98977\_at,160273\_at |
| 7 | establishment and/or maintenance of chromatin architecture | 2 | 80 | 71 | 6246 | 0.028 | 0.013 | 2.199 | 0.231 | 160273\_at,94288\_at |
| 8 | chromatin assembly/disassembly | 2 | 48 | 18 | 2164 | 0.111 | 0.022 | 5.009 | 0.059 | 160273\_at,94288\_at |
| 9 | nucleosome assembly | 1 | 28 | 5 | 911 | 0.2 | 0.031 | 6.506 | 0.145 | 94288\_at |
| 8 | chromatin modification | 1 | 36 | 18 | 2164 | 0.056 | 0.017 | 3.339 | 0.262 | 160273\_at |
| 7 | telomere maintenance | 1 | 8 | 71 | 6246 | 0.014 | 0.001 | 11 | 0.087 | 98977\_at |
| 8 | telomerase-dependent telomere maintenance | 1 | 8 | 18 | 2164 | 0.056 | 0.004 | 15.016 | 0.065 | 98977\_at |
| 4 | cell proliferation | 8 | 501 | 149 | 13100 | 0.054 | 0.038 | 1.404 | 0.211 | 101072\_at,104598\_at,92310\_at,92323\_at,102286\_at,103990\_at,160901\_at,92644\_s\_at |
| 5 | cell cycle | 8 | 435 | 132 | 11544 | 0.061 | 0.038 | 1.609 | 0.125 | 101072\_at,104598\_at,92310\_at,92323\_at,102286\_at,103990\_at,160901\_at,92644\_s\_at |
| 6 | regulation of cell cycle | 4 | 204 | 92 | 9498 | 0.043 | 0.021 | 2.024 | 0.136 | 102286\_at,103990\_at,160901\_at,92644\_s\_at |
| 4 | transport | 14 | 1083 | 149 | 13100 | 0.094 | 0.083 | 1.137 | 0.349 | 103427\_at,104396\_at,104398\_at,104719\_at,160564\_at,93705\_at,95157\_at,97181\_f\_at,99451\_at,96530\_at,92256\_at,96186\_at,100582\_at,98045\_s\_at |
| 5 | amine/polyamine transport | 1 | 19 | 132 | 11544 | 0.008 | 0.002 | 4.594 | 0.196 | 104719\_at |
| 6 | amino acid transport | 1 | 19 | 92 | 9498 | 0.011 | 0.002 | 5.435 | 0.169 | 104719\_at |
| 5 | gas transport | 1 | 10 | 132 | 11544 | 0.008 | 0.001 | 8.713 | 0.109 | 97181\_f\_at |
| 6 | oxygen transport | 1 | 10 | 92 | 9498 | 0.011 | 0.001 | 10.352 | 0.093 | 97181\_f\_at |
| 5 | intracellular transport | 3 | 351 | 132 | 11544 | 0.023 | 0.03 | 0.747 | 0.77 | 95157\_at,96530\_at,92256\_at |
| 6 | intracellular protein transport | 3 | 284 | 92 | 9498 | 0.033 | 0.03 | 1.091 | 0.522 | 95157\_at,96530\_at,92256\_at |
| 7 | protein targeting | 1 | 101 | 71 | 6246 | 0.014 | 0.016 | 0.871 | 0.688 | 92256\_at |
| 5 | ion transport | 3 | 335 | 132 | 11544 | 0.023 | 0.029 | 0.783 | 0.742 | 104719\_at,93705\_at,99451\_at |
| 6 | anion transport | 1 | 79 | 92 | 9498 | 0.011 | 0.008 | 1.306 | 0.538 | 104719\_at |
| 7 | inorganic anion transport | 1 | 50 | 71 | 6246 | 0.014 | 0.008 | 1.758 | 0.437 | 104719\_at |
| 8 | chloride transport | 1 | 39 | 18 | 2164 | 0.056 | 0.018 | 3.083 | 0.28 | 104719\_at |
| 6 | cation transport | 2 | 236 | 92 | 9498 | 0.022 | 0.025 | 0.875 | 0.671 | 99451\_at,104719\_at |
| 7 | metal ion transport | 2 | 184 | 71 | 6246 | 0.028 | 0.029 | 0.956 | 0.624 | 104719\_at,99451\_at |
| 8 | potassium ion transport | 2 | 94 | 18 | 2164 | 0.111 | 0.043 | 2.558 | 0.182 | 104719\_at,99451\_at |
| 8 | sodium ion transport | 1 | 43 | 18 | 2164 | 0.056 | 0.02 | 2.796 | 0.304 | 104719\_at |
| 5 | lipid transport | 1 | 40 | 132 | 11544 | 0.008 | 0.003 | 2.184 | 0.369 | 96186\_at |
| 5 | protein transport | 3 | 297 | 132 | 11544 | 0.023 | 0.026 | 0.883 | 0.665 | 95157\_at,96530\_at,92256\_at |
| 6 | intracellular protein transport | 3 | 284 | 92 | 9498 | 0.033 | 0.03 | 1.091 | 0.522 | 95157\_at,96530\_at,92256\_at |
| 7 | protein targeting | 1 | 101 | 71 | 6246 | 0.014 | 0.016 | 0.871 | 0.688 | 92256\_at |
| 5 | vesicle-mediated transport | 2 | 112 | 132 | 11544 | 0.015 | 0.01 | 1.562 | 0.367 | 100582\_at,98045\_s\_at |
| 6 | endocytosis | 2 | 61 | 92 | 9498 | 0.022 | 0.006 | 3.386 | 0.118 | 100582\_at,98045\_s\_at |
| 7 | receptor mediated endocytosis | 2 | 9 | 71 | 6246 | 0.028 | 0.001 | 19.562 | 0.004 | 100582\_at,98045\_s\_at |
| 3 | cell motility | 1 | 188 | 123 | 10726 | 0.008 | 0.018 | 0.464 | 0.888 | 100473\_at |
| 4 | cell migration | 1 | 53 | 149 | 13100 | 0.007 | 0.004 | 1.657 | 0.455 | 100473\_at |
| 5 | axon guidance | 1 | 23 | 132 | 11544 | 0.008 | 0.002 | 3.809 | 0.233 | 100473\_at |
| 2 | development | 13 | 990 | 135 | 10540 | 0.096 | 0.094 | 1.025 | 0.506 | 100473\_at,102356\_at,102933\_at,93682\_at,98418\_at,98045\_s\_at,102225\_at,92248\_at,103832\_at,93500\_at,100533\_s\_at,101515\_at,160526\_s\_at |
| 3 | cell differentiation | 1 | 137 | 123 | 10726 | 0.008 | 0.013 | 0.637 | 0.796 | 98045\_s\_at |
| 4 | cellular morphogenesis during differentiation | 1 | 4 | 149 | 13100 | 0.007 | 0 | 21.645 | 0.045 | 98045\_s\_at |
| 3 | embryonic development | 1 | 52 | 123 | 10726 | 0.008 | 0.005 | 1.676 | 0.452 | 98045\_s\_at |
| 4 | embryonic development (sensu Animalia) | 1 | 18 | 149 | 13100 | 0.007 | 0.001 | 4.898 | 0.186 | 98045\_s\_at |
| 5 | embryonic development (sensu Mammalia) | 1 | 6 | 132 | 11544 | 0.008 | 0.001 | 14.577 | 0.067 | 98045\_s\_at |
| 3 | morphogenesis | 4 | 594 | 123 | 10726 | 0.033 | 0.055 | 0.587 | 0.915 | 102225\_at,100473\_at,92248\_at,103832\_at |
| 4 | organogenesis | 4 | 544 | 149 | 13100 | 0.027 | 0.042 | 0.647 | 0.872 | 102225\_at,100473\_at,92248\_at,103832\_at |
| 5 | neurogenesis | 2 | 164 | 132 | 11544 | 0.015 | 0.014 | 1.066 | 0.562 | 100473\_at,92248\_at |
| 5 | skeletal development | 1 | 52 | 132 | 11544 | 0.008 | 0.004 | 1.684 | 0.451 | 103832\_at |
| 6 | ossification | 1 | 25 | 92 | 9498 | 0.011 | 0.003 | 4.133 | 0.216 | 103832\_at |
| 3 | pigmentation | 1 | 23 | 123 | 10726 | 0.008 | 0.002 | 3.799 | 0.233 | 93500\_at |
| 4 | pigment metabolism | 1 | 23 | 149 | 13100 | 0.007 | 0.002 | 3.812 | 0.232 | 93500\_at |
| 5 | heme metabolism | 1 | 15 | 132 | 11544 | 0.008 | 0.001 | 5.831 | 0.159 | 93500\_at |
| 6 | heme biosynthesis | 1 | 11 | 92 | 9498 | 0.011 | 0.001 | 9.371 | 0.102 | 93500\_at |
| 3 | reproduction | 3 | 99 | 123 | 10726 | 0.024 | 0.009 | 2.642 | 0.105 | 100533\_s\_at,101515\_at,160526\_s\_at |
| 4 | sexual reproduction | 3 | 99 | 149 | 13100 | 0.02 | 0.008 | 2.663 | 0.103 | 100533\_s\_at,101515\_at,160526\_s\_at |
| 5 | gametogenesis | 3 | 86 | 132 | 11544 | 0.023 | 0.007 | 3.051 | 0.076 | 100533\_s\_at,101515\_at,160526\_s\_at |
| 6 | male gamete generation | 3 | NA | 92 | 9498 | 0.033 | NA | NA | NA | 100533\_s\_at,101515\_at,160526\_s\_at |
| 7 | spermatogenesis | 3 | 66 | 71 | 6246 | 0.042 | 0.011 | 3.997 | 0.039 | 100533\_s\_at,101515\_at,160526\_s\_at |
| 2 | obsolete biological process | 1 | 3 | 135 | 10540 | 0.007 | 0 | 26.464 | 0.038 | 97817\_at |
| 3 | mRNA splicing | 1 | 54 | 123 | 10726 | 0.008 | 0.005 | 1.616 | 0.464 | 97817\_at |
| 2 | physiological processes | 74 | 5866 | 135 | 10540 | 0.548 | 0.557 | 0.985 | 0.613 | 101072\_at,102286\_at,103990\_at,160901\_at,92644\_s\_at,93714\_f\_at,92270\_at,100473\_at,98007\_at,94288\_at,98977\_at,160273\_at,104598\_at,92310\_at,92323\_at,103427\_at,104396\_at,104398\_at,104719\_at,160564\_at,93705\_at,95157\_at,97181\_f\_at,99451\_at,96530\_at,92256\_at,96186\_at,100582\_at,98045\_s\_at,93500\_at,95133\_at,160547\_s\_at,160346\_at,97798\_at,161683\_r\_at,103392\_at,103217\_at,94834\_at,96732\_at,96848\_at,97336\_at,97943\_at,101515\_at,94948\_at,96886\_at,101426\_at,94345\_at,92248\_at,100533\_s\_at,102209\_at,102955\_at,104562\_at,160526\_s\_at,161211\_r\_at,96940\_at,98083\_at,160362\_at,93179\_at,94929\_at,98431\_at,102225\_at,103348\_at,95893\_at,98369\_f\_at,160104\_at,104362\_at,101897\_g\_at,93120\_f\_at,93907\_f\_at,98088\_at,100322\_at,102156\_f\_at,93584\_at,160792\_at |
| 3 | cell growth and/or maintenance | 29 | 2128 | 123 | 10726 | 0.236 | 0.198 | 1.188 | 0.175 | 101072\_at,102286\_at,103990\_at,160901\_at,92644\_s\_at,93714\_f\_at,92270\_at,100473\_at,98007\_at,94288\_at,98977\_at,160273\_at,104598\_at,92310\_at,92323\_at,103427\_at,104396\_at,104398\_at,104719\_at,160564\_at,93705\_at,95157\_at,97181\_f\_at,99451\_at,96530\_at,92256\_at,96186\_at,100582\_at,98045\_s\_at |
| 4 | cell growth | 2 | 51 | 149 | 13100 | 0.013 | 0.004 | 3.45 | 0.114 | 93714\_f\_at,92270\_at |
| 5 | regulation of cell growth | 2 | 38 | 132 | 11544 | 0.015 | 0.003 | 4.605 | 0.07 | 93714\_f\_at,92270\_at |
| 6 | negative regulation of cell growth | 1 | 2 | 92 | 9498 | 0.011 | 0 | 51.762 | 0.019 | 92270\_at |
| 4 | cell organization and biogenesis | 5 | 530 | 149 | 13100 | 0.034 | 0.04 | 0.829 | 0.726 | 100473\_at,98007\_at,94288\_at,98977\_at,160273\_at |
| 5 | cytoplasm organization and biogenesis | 2 | 380 | 132 | 11544 | 0.015 | 0.033 | 0.46 | 0.935 | 100473\_at,98007\_at |
| 6 | organelle organization and biogenesis | 1 | 318 | 92 | 9498 | 0.011 | 0.033 | 0.325 | 0.957 | 100473\_at |
| 7 | cytoskeleton organization and biogenesis | 1 | 262 | 71 | 6246 | 0.014 | 0.042 | 0.336 | 0.953 | 100473\_at |
| 8 | actin filament-based process | 1 | 42 | 18 | 2164 | 0.056 | 0.019 | 2.862 | 0.298 | 100473\_at |
| 9 | actin cytoskeleton organization and biogenesis | 1 | 42 | 5 | 911 | 0.2 | 0.046 | 4.338 | 0.211 | 100473\_at |
| 10 | actin filament organization | 1 | 16 | 2 | 197 | 0.5 | 0.081 | 6.156 | 0.156 | 100473\_at |
| 6 | ribosome biogenesis and assembly | 1 | 60 | 92 | 9498 | 0.011 | 0.006 | 1.72 | 0.443 | 98007\_at |
| 7 | ribosome biogenesis | 1 | 60 | 71 | 6246 | 0.014 | 0.01 | 1.465 | 0.498 | 98007\_at |
| 5 | nuclear organization and biogenesis | 3 | 112 | 132 | 11544 | 0.023 | 0.01 | 2.343 | 0.137 | 94288\_at,98977\_at,160273\_at |
| 6 | chromosome organization and biogenesis (sensu Eukarya) | 3 | 108 | 92 | 9498 | 0.033 | 0.011 | 2.868 | 0.087 | 94288\_at,98977\_at,160273\_at |
| 7 | establishment and/or maintenance of chromatin architecture | 2 | 80 | 71 | 6246 | 0.028 | 0.013 | 2.199 | 0.231 | 160273\_at,94288\_at |
| 8 | chromatin assembly/disassembly | 2 | 48 | 18 | 2164 | 0.111 | 0.022 | 5.009 | 0.059 | 160273\_at,94288\_at |
| 9 | nucleosome assembly | 1 | 28 | 5 | 911 | 0.2 | 0.031 | 6.506 | 0.145 | 94288\_at |
| 8 | chromatin modification | 1 | 36 | 18 | 2164 | 0.056 | 0.017 | 3.339 | 0.262 | 160273\_at |
| 7 | telomere maintenance | 1 | 8 | 71 | 6246 | 0.014 | 0.001 | 11 | 0.087 | 98977\_at |
| 8 | telomerase-dependent telomere maintenance | 1 | 8 | 18 | 2164 | 0.056 | 0.004 | 15.016 | 0.065 | 98977\_at |
| 4 | cell proliferation | 8 | 501 | 149 | 13100 | 0.054 | 0.038 | 1.404 | 0.211 | 101072\_at,104598\_at,92310\_at,92323\_at,102286\_at,103990\_at,160901\_at,92644\_s\_at |
| 5 | cell cycle | 8 | 435 | 132 | 11544 | 0.061 | 0.038 | 1.609 | 0.125 | 101072\_at,104598\_at,92310\_at,92323\_at,102286\_at,103990\_at,160901\_at,92644\_s\_at |
| 6 | regulation of cell cycle | 4 | 204 | 92 | 9498 | 0.043 | 0.021 | 2.024 | 0.136 | 102286\_at,103990\_at,160901\_at,92644\_s\_at |
| 4 | transport | 14 | 1083 | 149 | 13100 | 0.094 | 0.083 | 1.137 | 0.349 | 103427\_at,104396\_at,104398\_at,104719\_at,160564\_at,93705\_at,95157\_at,97181\_f\_at,99451\_at,96530\_at,92256\_at,96186\_at,100582\_at,98045\_s\_at |
| 5 | amine/polyamine transport | 1 | 19 | 132 | 11544 | 0.008 | 0.002 | 4.594 | 0.196 | 104719\_at |
| 6 | amino acid transport | 1 | 19 | 92 | 9498 | 0.011 | 0.002 | 5.435 | 0.169 | 104719\_at |
| 5 | gas transport | 1 | 10 | 132 | 11544 | 0.008 | 0.001 | 8.713 | 0.109 | 97181\_f\_at |
| 6 | oxygen transport | 1 | 10 | 92 | 9498 | 0.011 | 0.001 | 10.352 | 0.093 | 97181\_f\_at |
| 5 | intracellular transport | 3 | 351 | 132 | 11544 | 0.023 | 0.03 | 0.747 | 0.77 | 95157\_at,96530\_at,92256\_at |
| 6 | intracellular protein transport | 3 | 284 | 92 | 9498 | 0.033 | 0.03 | 1.091 | 0.522 | 95157\_at,96530\_at,92256\_at |
| 7 | protein targeting | 1 | 101 | 71 | 6246 | 0.014 | 0.016 | 0.871 | 0.688 | 92256\_at |
| 5 | ion transport | 3 | 335 | 132 | 11544 | 0.023 | 0.029 | 0.783 | 0.742 | 104719\_at,93705\_at,99451\_at |
| 6 | anion transport | 1 | 79 | 92 | 9498 | 0.011 | 0.008 | 1.306 | 0.538 | 104719\_at |
| 7 | inorganic anion transport | 1 | 50 | 71 | 6246 | 0.014 | 0.008 | 1.758 | 0.437 | 104719\_at |
| 8 | chloride transport | 1 | 39 | 18 | 2164 | 0.056 | 0.018 | 3.083 | 0.28 | 104719\_at |
| 6 | cation transport | 2 | 236 | 92 | 9498 | 0.022 | 0.025 | 0.875 | 0.671 | 99451\_at,104719\_at |
| 7 | metal ion transport | 2 | 184 | 71 | 6246 | 0.028 | 0.029 | 0.956 | 0.624 | 104719\_at,99451\_at |
| 8 | potassium ion transport | 2 | 94 | 18 | 2164 | 0.111 | 0.043 | 2.558 | 0.182 | 104719\_at,99451\_at |
| 8 | sodium ion transport | 1 | 43 | 18 | 2164 | 0.056 | 0.02 | 2.796 | 0.304 | 104719\_at |
| 5 | lipid transport | 1 | 40 | 132 | 11544 | 0.008 | 0.003 | 2.184 | 0.369 | 96186\_at |
| 5 | protein transport | 3 | 297 | 132 | 11544 | 0.023 | 0.026 | 0.883 | 0.665 | 95157\_at,96530\_at,92256\_at |
| 6 | intracellular protein transport | 3 | 284 | 92 | 9498 | 0.033 | 0.03 | 1.091 | 0.522 | 95157\_at,96530\_at,92256\_at |
| 7 | protein targeting | 1 | 101 | 71 | 6246 | 0.014 | 0.016 | 0.871 | 0.688 | 92256\_at |
| 5 | vesicle-mediated transport | 2 | 112 | 132 | 11544 | 0.015 | 0.01 | 1.562 | 0.367 | 100582\_at,98045\_s\_at |
| 6 | endocytosis | 2 | 61 | 92 | 9498 | 0.022 | 0.006 | 3.386 | 0.118 | 100582\_at,98045\_s\_at |
| 7 | receptor mediated endocytosis | 2 | 9 | 71 | 6246 | 0.028 | 0.001 | 19.562 | 0.004 | 100582\_at,98045\_s\_at |
| 3 | metabolism | 50 | 3908 | 123 | 10726 | 0.406 | 0.364 | 1.116 | 0.188 | 93500\_at,95133\_at,160547\_s\_at,160346\_at,97798\_at,161683\_r\_at,103392\_at,103217\_at,103990\_at,92256\_at,94834\_at,96732\_at,96848\_at,97336\_at,97943\_at,101515\_at,103427\_at,94948\_at,96886\_at,96186\_at,101426\_at,94345\_at,92248\_at,100533\_s\_at,101072\_at,102209\_at,102955\_at,104562\_at,160273\_at,160526\_s\_at,160901\_at,161211\_r\_at,92644\_s\_at,96940\_at,98083\_at,160362\_at,104598\_at,93179\_at,94929\_at,98431\_at,102225\_at,102286\_at,103348\_at,92310\_at,92323\_at,95893\_at,98007\_at,98369\_f\_at,160104\_at,104362\_at |
| 4 | pigment metabolism | 1 | 23 | 149 | 13100 | 0.007 | 0.002 | 3.812 | 0.232 | 93500\_at |
| 5 | heme metabolism | 1 | 15 | 132 | 11544 | 0.008 | 0.001 | 5.831 | 0.159 | 93500\_at |
| 6 | heme biosynthesis | 1 | 11 | 92 | 9498 | 0.011 | 0.001 | 9.371 | 0.102 | 93500\_at |
| 4 | alcohol metabolism | 1 | 167 | 149 | 13100 | 0.007 | 0.013 | 0.526 | 0.854 | 160547\_s\_at |
| 5 | alcohol catabolism | 1 | 58 | 132 | 11544 | 0.008 | 0.005 | 1.51 | 0.488 | 160547\_s\_at |
| 6 | monosaccharide catabolism | 1 | 58 | 92 | 9498 | 0.011 | 0.006 | 1.779 | 0.432 | 160547\_s\_at |
| 7 | hexose catabolism | 1 | 58 | 71 | 6246 | 0.014 | 0.009 | 1.516 | 0.486 | 160547\_s\_at |
| 8 | glucose catabolism | 1 | 58 | 18 | 2164 | 0.056 | 0.027 | 2.073 | 0.388 | 160547\_s\_at |
| 9 | glycolysis | 1 | 52 | 5 | 911 | 0.2 | 0.057 | 3.504 | 0.255 | 160547\_s\_at |
| 4 | amine metabolism | 1 | 148 | 149 | 13100 | 0.007 | 0.011 | 0.594 | 0.818 | 95133\_at |
| 5 | amine biosynthesis | 1 | 35 | 132 | 11544 | 0.008 | 0.003 | 2.502 | 0.332 | 95133\_at |
| 6 | amino acid biosynthesis | 1 | 25 | 92 | 9498 | 0.011 | 0.003 | 4.133 | 0.216 | 95133\_at |
| 7 | aspartate family amino acid biosynthesis | 1 | 6 | 71 | 6246 | 0.014 | 0.001 | 14.667 | 0.066 | 95133\_at |
| 8 | asparagine biosynthesis | 1 | 2 | 18 | 2164 | 0.056 | 0.001 | 60.391 | 0.017 | 95133\_at |
| 5 | amino acid metabolism | 1 | 97 | 132 | 11544 | 0.008 | 0.008 | 0.902 | 0.674 | 95133\_at |
| 6 | amino acid biosynthesis | 1 | 25 | 92 | 9498 | 0.011 | 0.003 | 4.133 | 0.216 | 95133\_at |
| 7 | aspartate family amino acid biosynthesis | 1 | 6 | 71 | 6246 | 0.014 | 0.001 | 14.667 | 0.066 | 95133\_at |
| 8 | asparagine biosynthesis | 1 | 2 | 18 | 2164 | 0.056 | 0.001 | 60.391 | 0.017 | 95133\_at |
| 6 | glutamine family amino acid metabolism | 1 | 15 | 92 | 9498 | 0.011 | 0.002 | 6.88 | 0.136 | 95133\_at |
| 7 | glutamine metabolism | 1 | 6 | 71 | 6246 | 0.014 | 0.001 | 14.667 | 0.066 | 95133\_at |
| 4 | biosynthesis | 6 | 652 | 149 | 13100 | 0.04 | 0.05 | 0.809 | 0.758 | 93500\_at,95133\_at,160346\_at,97798\_at,161683\_r\_at,103392\_at |
| 5 | amine biosynthesis | 1 | 35 | 132 | 11544 | 0.008 | 0.003 | 2.502 | 0.332 | 95133\_at |
| 6 | amino acid biosynthesis | 1 | 25 | 92 | 9498 | 0.011 | 0.003 | 4.133 | 0.216 | 95133\_at |
| 7 | aspartate family amino acid biosynthesis | 1 | 6 | 71 | 6246 | 0.014 | 0.001 | 14.667 | 0.066 | 95133\_at |
| 8 | asparagine biosynthesis | 1 | 2 | 18 | 2164 | 0.056 | 0.001 | 60.391 | 0.017 | 95133\_at |
| 5 | macromolecule biosynthesis | 3 | 322 | 132 | 11544 | 0.023 | 0.028 | 0.815 | 0.717 | 160346\_at,97798\_at,161683\_r\_at |
| 6 | protein biosynthesis | 3 | 322 | 92 | 9498 | 0.033 | 0.034 | 0.962 | 0.608 | 160346\_at,97798\_at,161683\_r\_at |
| 7 | lipoprotein biosynthesis | 1 | 16 | 71 | 6246 | 0.014 | 0.003 | 5.5 | 0.167 | 97798\_at |
| 8 | protein lipidation | 1 | 16 | 18 | 2164 | 0.056 | 0.007 | 7.518 | 0.126 | 97798\_at |
| 9 | protein prenylation | 1 | 10 | 5 | 911 | 0.2 | 0.011 | 18.215 | 0.054 | 97798\_at |
| 7 | translational elongation | 1 | 22 | 71 | 6246 | 0.014 | 0.004 | 4 | 0.223 | 161683\_r\_at |
| 5 | nucleotide biosynthesis | 1 | 74 | 132 | 11544 | 0.008 | 0.006 | 1.183 | 0.574 | 103392\_at |
| 6 | cyclic nucleotide biosynthesis | 1 | 8 | 92 | 9498 | 0.011 | 0.001 | 12.94 | 0.075 | 103392\_at |
| 7 | cAMP biosynthesis | 1 | 7 | 71 | 6246 | 0.014 | 0.001 | 12.571 | 0.077 | 103392\_at |
| 4 | catabolism | 9 | 631 | 149 | 13100 | 0.06 | 0.048 | 1.254 | 0.291 | 160547\_s\_at,103217\_at,103990\_at,92256\_at,94834\_at,96732\_at,96848\_at,97336\_at,97943\_at |
| 5 | alcohol catabolism | 1 | 58 | 132 | 11544 | 0.008 | 0.005 | 1.51 | 0.488 | 160547\_s\_at |
| 6 | monosaccharide catabolism | 1 | 58 | 92 | 9498 | 0.011 | 0.006 | 1.779 | 0.432 | 160547\_s\_at |
| 7 | hexose catabolism | 1 | 58 | 71 | 6246 | 0.014 | 0.009 | 1.516 | 0.486 | 160547\_s\_at |
| 8 | glucose catabolism | 1 | 58 | 18 | 2164 | 0.056 | 0.027 | 2.073 | 0.388 | 160547\_s\_at |
| 9 | glycolysis | 1 | 52 | 5 | 911 | 0.2 | 0.057 | 3.504 | 0.255 | 160547\_s\_at |
| 5 | macromolecule catabolism | 8 | 470 | 132 | 11544 | 0.061 | 0.041 | 1.489 | 0.17 | 103217\_at,103990\_at,92256\_at,94834\_at,96732\_at,96848\_at,97336\_at,97943\_at |
| 6 | protein catabolism | 8 | 466 | 92 | 9498 | 0.087 | 0.049 | 1.773 | 0.082 | 103217\_at,103990\_at,92256\_at,94834\_at,96732\_at,96848\_at,97336\_at,97943\_at |
| 7 | proteolysis and peptidolysis | 8 | 457 | 71 | 6246 | 0.113 | 0.073 | 1.54 | 0.145 | 103217\_at,103990\_at,92256\_at,94834\_at,96732\_at,96848\_at,97336\_at,97943\_at |
| 4 | electron transport | 4 | 313 | 149 | 13100 | 0.027 | 0.024 | 1.124 | 0.478 | 101515\_at,103427\_at,94948\_at,96886\_at |
| 4 | lipid metabolism | 3 | 285 | 149 | 13100 | 0.02 | 0.022 | 0.925 | 0.633 | 96186\_at,101515\_at,101426\_at |
| 5 | fatty acid metabolism | 1 | 85 | 132 | 11544 | 0.008 | 0.007 | 1.03 | 0.625 | 101515\_at |
| 6 | fatty acid oxidation | 1 | 5 | 92 | 9498 | 0.011 | 0.001 | 20.509 | 0.048 | 101515\_at |
| 7 | fatty acid beta-oxidation | 1 | 4 | 71 | 6246 | 0.014 | 0.001 | 22 | 0.045 | 101515\_at |
| 5 | membrane lipid metabolism | 1 | 46 | 132 | 11544 | 0.008 | 0.004 | 1.905 | 0.411 | 101426\_at |
| 6 | sphingolipid metabolism | 1 | 14 | 92 | 9498 | 0.011 | 0.001 | 7.395 | 0.127 | 101426\_at |
| 7 | sphingoid metabolism | 1 | 10 | 71 | 6246 | 0.014 | 0.002 | 8.8 | 0.108 | 101426\_at |
| 8 | ceramide metabolism | 1 | 9 | 18 | 2164 | 0.056 | 0.004 | 13.356 | 0.073 | 101426\_at |
| 4 | nucleobase, nucleoside, nucleotide and nucleic acid metabolism | 15 | 1530 | 149 | 13100 | 0.101 | 0.117 | 0.862 | 0.767 | 94345\_at,92248\_at,100533\_s\_at,101072\_at,102209\_at,102955\_at,103990\_at,104562\_at,160273\_at,160526\_s\_at,160901\_at,161211\_r\_at,92644\_s\_at,96940\_at,98083\_at |
| 5 | DNA metabolism | 1 | 302 | 132 | 11544 | 0.008 | 0.026 | 0.29 | 0.97 | 94345\_at |
| 6 | DNA repair | 1 | 99 | 92 | 9498 | 0.011 | 0.01 | 1.043 | 0.62 | 94345\_at |
| 5 | transcription | 14 | 1086 | 132 | 11544 | 0.106 | 0.094 | 1.127 | 0.36 | 92248\_at,100533\_s\_at,101072\_at,102209\_at,102955\_at,103990\_at,104562\_at,160273\_at,160526\_s\_at,160901\_at,161211\_r\_at,92644\_s\_at,96940\_at,98083\_at |
| 6 | regulation of transcription | 14 | 1026 | 92 | 9498 | 0.152 | 0.108 | 1.409 | 0.117 | 100533\_s\_at,101072\_at,102209\_at,102955\_at,103990\_at,104562\_at,160273\_at,160526\_s\_at,160901\_at,161211\_r\_at,92248\_at,92644\_s\_at,96940\_at,98083\_at |
| 7 | regulation of transcription, DNA-dependent | 14 | 1013 | 71 | 6246 | 0.197 | 0.162 | 1.216 | 0.253 | 100533\_s\_at,101072\_at,102209\_at,102955\_at,103990\_at,104562\_at,160273\_at,160526\_s\_at,160901\_at,161211\_r\_at,92248\_at,92644\_s\_at,96940\_at,98083\_at |
| 4 | one-carbon compound metabolism | 1 | 27 | 149 | 13100 | 0.007 | 0.002 | 3.257 | 0.266 | 160362\_at |
| 4 | phosphorus metabolism | 12 | 488 | 149 | 13100 | 0.081 | 0.037 | 2.162 | 0.01 | 104598\_at,93179\_at,94929\_at,98431\_at,102225\_at,102286\_at,103348\_at,92310\_at,92323\_at,95893\_at,98007\_at,98369\_f\_at |
| 5 | phosphate metabolism | 12 | 488 | 132 | 11544 | 0.091 | 0.042 | 2.151 | 0.01 | 104598\_at,93179\_at,94929\_at,98431\_at,102225\_at,102286\_at,103348\_at,92310\_at,92323\_at,95893\_at,98007\_at,98369\_f\_at |
| 6 | dephosphorylation | 4 | 92 | 92 | 9498 | 0.043 | 0.01 | 4.487 | 0.012 | 104598\_at,93179\_at,94929\_at,98431\_at |
| 7 | protein amino acid dephosphorylation | 4 | 92 | 71 | 6246 | 0.056 | 0.015 | 3.825 | 0.02 | 104598\_at,93179\_at,94929\_at,98431\_at |
| 6 | phosphorylation | 8 | 395 | 92 | 9498 | 0.087 | 0.042 | 2.091 | 0.037 | 102225\_at,102286\_at,103348\_at,92310\_at,92323\_at,95893\_at,98007\_at,98369\_f\_at |
| 7 | protein amino acid phosphorylation | 8 | 379 | 71 | 6246 | 0.113 | 0.061 | 1.857 | 0.064 | 102225\_at,102286\_at,103348\_at,92310\_at,92323\_at,95893\_at,98007\_at,98369\_f\_at |
| 4 | protein metabolism | 25 | 1458 | 149 | 13100 | 0.168 | 0.111 | 1.508 | 0.024 | 92256\_at,160346\_at,97798\_at,161683\_r\_at,103217\_at,103990\_at,94834\_at,96732\_at,96848\_at,97336\_at,97943\_at,160104\_at,104362\_at,104598\_at,93179\_at,94929\_at,98431\_at,102225\_at,102286\_at,103348\_at,92310\_at,92323\_at,95893\_at,98007\_at,98369\_f\_at |
| 5 | protein folding | 1 | 56 | 132 | 11544 | 0.008 | 0.005 | 1.563 | 0.476 | 160104\_at |
| 5 | protein modification | 14 | 654 | 132 | 11544 | 0.106 | 0.057 | 1.872 | 0.017 | 104362\_at,97798\_at,104598\_at,93179\_at,94929\_at,98431\_at,102225\_at,102286\_at,103348\_at,92310\_at,92323\_at,95893\_at,98007\_at,98369\_f\_at |
| 6 | ubiquitin cycle | 1 | 56 | 92 | 9498 | 0.011 | 0.006 | 1.842 | 0.421 | 104362\_at |
| 3 | response to external stimulus | 8 | 666 | 123 | 10726 | 0.065 | 0.062 | 1.048 | 0.499 | 104719\_at,101897\_g\_at,93120\_f\_at,93907\_f\_at,98088\_at,100322\_at,102156\_f\_at,93584\_at |
| 4 | perception of external stimulus | 1 | 84 | 149 | 13100 | 0.007 | 0.006 | 1.047 | 0.619 | 104719\_at |
| 5 | perception of abiotic stimulus | 1 | 72 | 132 | 11544 | 0.008 | 0.006 | 1.215 | 0.564 | 104719\_at |
| 6 | perception of sound | 1 | 17 | 92 | 9498 | 0.011 | 0.002 | 6.073 | 0.153 | 104719\_at |
| 7 | hearing | 1 | 17 | 71 | 6246 | 0.014 | 0.003 | 5.176 | 0.177 | 104719\_at |
| 4 | response to biotic stimulus | 7 | 516 | 149 | 13100 | 0.047 | 0.039 | 1.193 | 0.372 | 101897\_g\_at,93120\_f\_at,93907\_f\_at,98088\_at,100322\_at,102156\_f\_at,93584\_at |
| 5 | defense response | 7 | 471 | 132 | 11544 | 0.053 | 0.041 | 1.3 | 0.293 | 101897\_g\_at,93120\_f\_at,93907\_f\_at,98088\_at,100322\_at,102156\_f\_at,93584\_at |
| 6 | immune response | 5 | 362 | 92 | 9498 | 0.054 | 0.038 | 1.426 | 0.273 | 93120\_f\_at,98088\_at,100322\_at,102156\_f\_at,93584\_at |
| 7 | antigen presentation | 1 | 26 | 71 | 6246 | 0.014 | 0.004 | 3.385 | 0.258 | 93120\_f\_at |
| 8 | antigen presentation, endogenous antigen | 1 | 15 | 18 | 2164 | 0.056 | 0.007 | 8.017 | 0.118 | 93120\_f\_at |
| 7 | antigen processing | 1 | 27 | 71 | 6246 | 0.014 | 0.004 | 3.259 | 0.266 | 93120\_f\_at |
| 8 | antigen processing, endogenous antigen via MHC class I | 1 | 15 | 18 | 2164 | 0.056 | 0.007 | 8.017 | 0.118 | 93120\_f\_at |
| 7 | humoral immune response | 3 | 103 | 71 | 6246 | 0.042 | 0.016 | 2.562 | 0.112 | 100322\_at,102156\_f\_at,93584\_at |
| 7 | innate immune response | 1 | 70 | 71 | 6246 | 0.014 | 0.011 | 1.256 | 0.553 | 98088\_at |
| 8 | inflammatory response | 1 | 70 | 18 | 2164 | 0.056 | 0.032 | 1.717 | 0.448 | 98088\_at |
| 3 | response to stress | 1 | 400 | 123 | 10726 | 0.008 | 0.037 | 0.218 | 0.991 | 160792\_at |

  
