# Supplementary material for: Molecular Signatures of Proliferation and Quiescence in Hematopoietic Stem Cells
Source: PLoS Biol. 2004 Sep 28;2(10):e301. doi: 10.1371/journal.pbio.0020301 (PMC520599; doi:10.1371/journal.pbio.0020301)
Supplement: Table S37 — (8 KB HTML). [file pbio.0020301.st037.html]

   Significant Tom Day 0   

# Significant Tom Day 0

|  |  |  |  |  |  |  |  |  |  |  |  |
| --- | --- | --- | --- | --- | --- | --- | --- | --- | --- | --- | --- |
| GOLevel | GOTerm | ProbeCount | ArrayCount | ListGOLevelCount | ArrayGoLevelCount | ListFq | ArrayFq | FoldChange | H-Pvalue | ProbeIds | GeneNames |
| 8 | protein kinase C activation | 1 | 5 | 18 | 2164 | 0.056 | 0.002 | 24.052 | 0.041 | 101426\_at | ceramide kinase |
| 6 | induction of apoptosis | 2 | 28 | 92 | 9498 | 0.022 | 0.003 | 7.369 | 0.03 | 103217\_at,95102\_at | CASP8 and FADD-like apoptosis regulator,scotin gene |
| 4 | cellular morphogenesis during differentiation | 1 | 4 | 149 | 13100 | 0.007 | 0 | 21.645 | 0.045 | 98045\_s\_at | disabled homolog 2 (Drosophila) |
| 6 | negative regulation of cell growth | 1 | 2 | 92 | 9498 | 0.011 | 0 | 51.762 | 0.019 | 92270\_at | trophinin |
| 7 | receptor mediated endocytosis | 2 | 9 | 71 | 6246 | 0.028 | 0.001 | 19.562 | 0.004 | 100582\_at,98045\_s\_at | sorting nexin 17,disabled homolog 2 (Drosophila) |
| 4 | cellular morphogenesis during differentiation | 1 | 4 | 149 | 13100 | 0.007 | 0 | 21.645 | 0.045 | 98045\_s\_at | disabled homolog 2 (Drosophila) |
| 7 | spermatogenesis | 3 | 66 | 71 | 6246 | 0.042 | 0.011 | 3.997 | 0.039 | 100533\_s\_at,101515\_at,160526\_s\_at | cAMP responsive element modulator,acyl-Coenzyme A oxidase 1, palmitoyl,cAMP responsive element modulator |
| 2 | obsolete biological process | 1 | 3 | 135 | 10540 | 0.007 | 0 | 26.464 | 0.038 | 97817\_at | small protein effector 1 of Cdc42 |
| 6 | negative regulation of cell growth | 1 | 2 | 92 | 9498 | 0.011 | 0 | 51.762 | 0.019 | 92270\_at | trophinin |
| 7 | receptor mediated endocytosis | 2 | 9 | 71 | 6246 | 0.028 | 0.001 | 19.562 | 0.004 | 100582\_at,98045\_s\_at | sorting nexin 17,disabled homolog 2 (Drosophila) |
| 8 | asparagine biosynthesis | 1 | 2 | 18 | 2164 | 0.056 | 0.001 | 60.391 | 0.017 | 95133\_at | asparagine synthetase |
| 8 | asparagine biosynthesis | 1 | 2 | 18 | 2164 | 0.056 | 0.001 | 60.391 | 0.017 | 95133\_at | asparagine synthetase |
| 8 | asparagine biosynthesis | 1 | 2 | 18 | 2164 | 0.056 | 0.001 | 60.391 | 0.017 | 95133\_at | asparagine synthetase |
| 6 | fatty acid oxidation | 1 | 5 | 92 | 9498 | 0.011 | 0.001 | 20.509 | 0.048 | 101515\_at | acyl-Coenzyme A oxidase 1, palmitoyl |
| 7 | fatty acid beta-oxidation | 1 | 4 | 71 | 6246 | 0.014 | 0.001 | 22 | 0.045 | 101515\_at | acyl-Coenzyme A oxidase 1, palmitoyl |
| 4 | phosphorus metabolism | 12 | 488 | 149 | 13100 | 0.081 | 0.037 | 2.162 | 0.01 | 104598\_at,93179\_at,94929\_at,98431\_at,102225\_at,102286\_at,103348\_at,92310\_at,92323\_at,95893\_at,98007\_at,98369\_f\_at | dual specificity phosphatase 1,RIKEN cDNA B830009D23 gene,protein tyrosine phosphatase, non-receptor type 1,dual specificity phosphatase 12,RIKEN cDNA 8430421H08 gene,raf-related oncogene,RIKEN cDNA 1110018F06 gene,serum-inducible kinase,mitogen-activated protein kinase 12,B lymphoid kinase,ribosomal protein S6 kinase, polypeptide 2,RIKEN cDNA 2610028J07 gene |
| 5 | phosphate metabolism | 12 | 488 | 132 | 11544 | 0.091 | 0.042 | 2.151 | 0.01 | 104598\_at,93179\_at,94929\_at,98431\_at,102225\_at,102286\_at,103348\_at,92310\_at,92323\_at,95893\_at,98007\_at,98369\_f\_at | dual specificity phosphatase 1,RIKEN cDNA B830009D23 gene,protein tyrosine phosphatase, non-receptor type 1,dual specificity phosphatase 12,RIKEN cDNA 8430421H08 gene,raf-related oncogene,RIKEN cDNA 1110018F06 gene,serum-inducible kinase,mitogen-activated protein kinase 12,B lymphoid kinase,ribosomal protein S6 kinase, polypeptide 2,RIKEN cDNA 2610028J07 gene |
| 6 | dephosphorylation | 4 | 92 | 92 | 9498 | 0.043 | 0.01 | 4.487 | 0.012 | 104598\_at,93179\_at,94929\_at,98431\_at | dual specificity phosphatase 1,RIKEN cDNA B830009D23 gene,protein tyrosine phosphatase, non-receptor type 1,dual specificity phosphatase 12 |
| 7 | protein amino acid dephosphorylation | 4 | 92 | 71 | 6246 | 0.056 | 0.015 | 3.825 | 0.02 | 104598\_at,93179\_at,94929\_at,98431\_at | dual specificity phosphatase 1,RIKEN cDNA B830009D23 gene,protein tyrosine phosphatase, non-receptor type 1,dual specificity phosphatase 12 |
| 6 | phosphorylation | 8 | 395 | 92 | 9498 | 0.087 | 0.042 | 2.091 | 0.037 | 102225\_at,102286\_at,103348\_at,92310\_at,92323\_at,95893\_at,98007\_at,98369\_f\_at | RIKEN cDNA 8430421H08 gene,raf-related oncogene,RIKEN cDNA 1110018F06 gene,serum-inducible kinase,mitogen-activated protein kinase 12,B lymphoid kinase,ribosomal protein S6 kinase, polypeptide 2,RIKEN cDNA 2610028J07 gene |
| 4 | protein metabolism | 25 | 1458 | 149 | 13100 | 0.168 | 0.111 | 1.508 | 0.024 | 92256\_at,160346\_at,97798\_at,161683\_r\_at,103217\_at,103990\_at,94834\_at,96732\_at,96848\_at,97336\_at,97943\_at,160104\_at,104362\_at,104598\_at,93179\_at,94929\_at,98431\_at,102225\_at,102286\_at,103348\_at,92310\_at,92323\_at,95893\_at,98007\_at,98369\_f\_at | cathepsin B,RIKEN cDNA 6330580J24 gene,RIKEN cDNA 4930504E06 gene,GTP binding protein 1,CASP8 and FADD-like apoptosis regulator,FBJ osteosarcoma oncogene B,cathepsin H,RIKEN cDNA 1500001L20 gene,inositol polyphosphate-5-phosphatase,cathepsin F,calpain 6,hydroxy-delta-5-steroid dehydrogenase, 3 beta- and steroid delta-isomerase 7,RIKEN cDNA B230113M03 gene,dual specificity phosphatase 1,RIKEN cDNA B830009D23 gene,protein tyrosine phosphatase, non-receptor type 1,dual specificity phosphatase 12,RIKEN cDNA 8430421H08 gene,raf-related oncogene,RIKEN cDNA 1110018F06 gene,serum-inducible kinase,mitogen-activated protein kinase 12,B lymphoid kinase,ribosomal protein S6 kinase, polypeptide 2,RIKEN cDNA 2610028J07 gene |
| 5 | protein modification | 14 | 654 | 132 | 11544 | 0.106 | 0.057 | 1.872 | 0.017 | 104362\_at,97798\_at,104598\_at,93179\_at,94929\_at,98431\_at,102225\_at,102286\_at,103348\_at,92310\_at,92323\_at,95893\_at,98007\_at,98369\_f\_at | RIKEN cDNA B230113M03 gene,RIKEN cDNA 4930504E06 gene,dual specificity phosphatase 1,RIKEN cDNA B830009D23 gene,protein tyrosine phosphatase, non-receptor type 1,dual specificity phosphatase 12,RIKEN cDNA 8430421H08 gene,raf-related oncogene,RIKEN cDNA 1110018F06 gene,serum-inducible kinase,mitogen-activated protein kinase 12,B lymphoid kinase,ribosomal protein S6 kinase, polypeptide 2,RIKEN cDNA 2610028J07 gene |

  
