# Supplementary material for: Molecular Signatures of Proliferation and Quiescence in Hematopoietic Stem Cells
Source: PLoS Biol. 2004 Sep 28;2(10):e301. doi: 10.1371/journal.pbio.0020301 (PMC520599; doi:10.1371/journal.pbio.0020301)
Supplement: Table S38 — (61 KB HTML). [file pbio.0020301.st038.html]

   Full Tom Day 30   

# Full Tom Day 30

|  |  |  |  |  |  |  |  |  |  |  |
| --- | --- | --- | --- | --- | --- | --- | --- | --- | --- | --- |
| GOLevel | GOTerm | ProbeCount | ArrayCount | ListGOLevelCount | ArrayGoLevelCount | ListFq | ArrayFq | FoldChange | H-Pvalue | ProbeIds |
| 0 | Gene\_Ontology | 88 | NA | 88 | 0 | 1 | NA | NA | NA | 96147\_at,160727\_at,100482\_at,104083\_at,95016\_at,94657\_at,100696\_at,93382\_at,94146\_at,99992\_at,102224\_at,104417\_at,102152\_f\_at,95291\_r\_at,96911\_at,102028\_at,97375\_at,160606\_r\_at,101843\_at,160776\_at,161348\_r\_at,97684\_at,94060\_at,100462\_at,99587\_at,100115\_at,104292\_at,160463\_at,96088\_at,104243\_r\_at,103091\_at,95618\_at,97813\_at,99964\_at,103958\_g\_at,94976\_at,103236\_at,104376\_at,95805\_at,99440\_at,98402\_at,95033\_at,102335\_at,102873\_at,161990\_f\_at,95012\_at,96076\_at,96669\_at,161184\_f\_at,103666\_at,160498\_at,92249\_g\_at,161392\_f\_at,98861\_at,162034\_r\_at,96790\_f\_at,104139\_at,102302\_at,94796\_at,97563\_f\_at,95599\_at,160789\_at,96270\_at,99970\_at,92821\_at,102789\_at,103052\_r\_at,103547\_at,104438\_at,104645\_at,160244\_at,161148\_f\_at,93425\_at,97901\_at,93702\_at,98385\_at,104287\_at,104363\_at,98426\_at,98906\_at,93875\_at,160651\_at,97125\_f\_at,98438\_f\_at,100972\_s\_at,103954\_at,100583\_at,101352\_g\_at |
| 1 | biological\_process | 88 | 6769 | 88 | 6769 | 1 | 1 | 1 | 1 | 96147\_at,160727\_at,100482\_at,104083\_at,95016\_at,94657\_at,100696\_at,93382\_at,94146\_at,99992\_at,102224\_at,104417\_at,102152\_f\_at,95291\_r\_at,96911\_at,102028\_at,97375\_at,160606\_r\_at,101843\_at,160776\_at,161348\_r\_at,97684\_at,94060\_at,100462\_at,99587\_at,100115\_at,104292\_at,160463\_at,96088\_at,104243\_r\_at,103091\_at,95618\_at,97813\_at,99964\_at,103958\_g\_at,94976\_at,103236\_at,104376\_at,95805\_at,99440\_at,98402\_at,95033\_at,102335\_at,102873\_at,161990\_f\_at,95012\_at,96076\_at,96669\_at,161184\_f\_at,103666\_at,160498\_at,92249\_g\_at,161392\_f\_at,98861\_at,162034\_r\_at,96790\_f\_at,104139\_at,102302\_at,94796\_at,97563\_f\_at,95599\_at,160789\_at,96270\_at,99970\_at,92821\_at,102789\_at,103052\_r\_at,103547\_at,104438\_at,104645\_at,160244\_at,161148\_f\_at,93425\_at,97901\_at,93702\_at,98385\_at,104287\_at,104363\_at,98426\_at,98906\_at,93875\_at,160651\_at,97125\_f\_at,98438\_f\_at,100972\_s\_at,103954\_at,100583\_at,101352\_g\_at |
| 2 | behavior | 2 | 63 | 138 | 10540 | 0.014 | 0.006 | 2.423 | 0.2 | 96147\_at,160727\_at |
| 3 | adult behavior | 1 | 14 | 138 | 10726 | 0.007 | 0.001 | 5.534 | 0.166 | 96147\_at |
| 3 | locomotory behavior | 1 | 23 | 138 | 10726 | 0.007 | 0.002 | 3.388 | 0.258 | 160727\_at |
| 2 | cellular process | 49 | 3616 | 138 | 10540 | 0.355 | 0.343 | 1.035 | 0.414 | 100482\_at,104083\_at,95016\_at,94657\_at,100696\_at,93382\_at,94146\_at,99992\_at,102224\_at,104417\_at,102152\_f\_at,95291\_r\_at,96911\_at,102028\_at,97375\_at,160606\_r\_at,101843\_at,160776\_at,161348\_r\_at,97684\_at,94060\_at,100462\_at,99587\_at,100115\_at,104292\_at,160463\_at,96088\_at,104243\_r\_at,160727\_at,103091\_at,95618\_at,97813\_at,99964\_at,103958\_g\_at,94976\_at,103236\_at,104376\_at,95805\_at,99440\_at,96147\_at,98402\_at,95033\_at,102335\_at,102873\_at,161990\_f\_at,95012\_at,96076\_at,96669\_at,161184\_f\_at |
| 3 | cell communication | 24 | 1550 | 138 | 10726 | 0.174 | 0.145 | 1.203 | 0.191 | 100482\_at,104083\_at,95016\_at,94657\_at,100696\_at,93382\_at,94146\_at,99992\_at,102224\_at,104417\_at,102152\_f\_at,95291\_r\_at,96911\_at,102028\_at,97375\_at,160606\_r\_at,101843\_at,160776\_at,161348\_r\_at,97684\_at,94060\_at,100462\_at,99587\_at,100115\_at |
| 4 | cell adhesion | 3 | 322 | 160 | 13100 | 0.019 | 0.025 | 0.763 | 0.757 | 100482\_at,104083\_at,95016\_at |
| 5 | cell-cell adhesion | 1 | 44 | 144 | 11544 | 0.007 | 0.004 | 1.822 | 0.425 | 104083\_at |
| 6 | homophilic cell adhesion | 1 | 31 | 108 | 9498 | 0.009 | 0.003 | 2.84 | 0.299 | 104083\_at |
| 4 | cell-cell signaling | 1 | 123 | 160 | 13100 | 0.006 | 0.009 | 0.666 | 0.781 | 94657\_at |
| 5 | transmission of nerve impulse | 1 | 82 | 144 | 11544 | 0.007 | 0.007 | 0.977 | 0.644 | 94657\_at |
| 6 | synaptic transmission | 1 | 80 | 108 | 9498 | 0.009 | 0.008 | 1.1 | 0.601 | 94657\_at |
| 4 | signal transduction | 21 | 1199 | 160 | 13100 | 0.131 | 0.092 | 1.434 | 0.058 | 100696\_at,93382\_at,94146\_at,99992\_at,102224\_at,104417\_at,102152\_f\_at,95291\_r\_at,96911\_at,94657\_at,102028\_at,97375\_at,160606\_r\_at,101843\_at,160776\_at,161348\_r\_at,97684\_at,94060\_at,100462\_at,99587\_at,100115\_at |
| 5 | cell surface receptor linked signal transduction | 10 | 621 | 144 | 11544 | 0.069 | 0.054 | 1.291 | 0.247 | 99992\_at,102224\_at,104417\_at,102152\_f\_at,95291\_r\_at,96911\_at,94657\_at,102028\_at,97375\_at,160606\_r\_at |
| 6 | enzyme linked receptor protein signaling pathway | 2 | 131 | 108 | 9498 | 0.019 | 0.014 | 1.343 | 0.441 | 102224\_at,104417\_at |
| 7 | transmembrane receptor protein tyrosine kinase signaling pathway | 2 | 61 | 79 | 6246 | 0.025 | 0.01 | 2.592 | 0.18 | 102224\_at,104417\_at |
| 6 | G-protein coupled receptor protein signaling pathway | 6 | 355 | 108 | 9498 | 0.056 | 0.037 | 1.486 | 0.217 | 102152\_f\_at,95291\_r\_at,96911\_at,94657\_at,102028\_at,97375\_at |
| 7 | gamma-aminobutyric acid signaling pathway | 1 | 14 | 79 | 6246 | 0.013 | 0.002 | 5.652 | 0.163 | 94657\_at |
| 7 | neuropeptide signaling pathway | 2 | 45 | 79 | 6246 | 0.025 | 0.007 | 3.517 | 0.11 | 102028\_at,97375\_at |
| 6 | integrin-mediated signaling pathway | 1 | 45 | 108 | 9498 | 0.009 | 0.005 | 1.954 | 0.403 | 160606\_r\_at |
| 5 | intracellular signaling cascade | 10 | 485 | 144 | 11544 | 0.069 | 0.042 | 1.653 | 0.082 | 101843\_at,102028\_at,160776\_at,161348\_r\_at,97684\_at,97375\_at,94060\_at,100462\_at,99587\_at,100115\_at |
| 6 | protein kinase cascade | 2 | 50 | 108 | 9498 | 0.019 | 0.005 | 3.521 | 0.11 | 97375\_at,94060\_at |
| 7 | JAK-STAT cascade | 1 | 10 | 79 | 6246 | 0.013 | 0.002 | 7.912 | 0.12 | 97375\_at |
| 7 | JNK cascade | 1 | 13 | 79 | 6246 | 0.013 | 0.002 | 6.087 | 0.153 | 94060\_at |
| 6 | small GTPase mediated signal transduction | 3 | 135 | 108 | 9498 | 0.028 | 0.014 | 1.955 | 0.198 | 100462\_at,99587\_at,100115\_at |
| 7 | Rho protein signal transduction | 1 | 20 | 79 | 6246 | 0.013 | 0.003 | 3.956 | 0.225 | 100115\_at |
| 5 | two-component signal transduction system (phosphorelay) | 1 | 15 | 144 | 11544 | 0.007 | 0.001 | 5.338 | 0.172 | 99587\_at |
| 3 | cell death | 1 | 207 | 138 | 10726 | 0.007 | 0.019 | 0.376 | 0.933 | 104292\_at |
| 4 | programmed cell death | 1 | 192 | 160 | 13100 | 0.006 | 0.015 | 0.426 | 0.907 | 104292\_at |
| 5 | apoptosis | 1 | 192 | 144 | 11544 | 0.007 | 0.017 | 0.417 | 0.912 | 104292\_at |
| 6 | apoptotic program | 1 | 16 | 108 | 9498 | 0.009 | 0.002 | 5.512 | 0.167 | 104292\_at |
| 3 | cell differentiation | 4 | 137 | 138 | 10726 | 0.029 | 0.013 | 2.27 | 0.1 | 160463\_at,96088\_at,104243\_r\_at,160727\_at |
| 4 | epidermal cell differentiation | 1 | 3 | 160 | 13100 | 0.006 | 0 | 27.174 | 0.036 | 104243\_r\_at |
| 5 | hair cell differentiation | 1 | 3 | 144 | 11544 | 0.007 | 0 | 26.692 | 0.037 | 104243\_r\_at |
| 4 | vasculogenesis | 1 | 7 | 160 | 13100 | 0.006 | 0.001 | 11.792 | 0.082 | 160727\_at |
| 3 | cell growth and/or maintenance | 25 | 2128 | 138 | 10726 | 0.181 | 0.198 | 0.913 | 0.727 | 103091\_at,95618\_at,97813\_at,99964\_at,103958\_g\_at,94976\_at,101843\_at,100115\_at,103236\_at,104376\_at,95805\_at,99440\_at,96147\_at,97375\_at,98402\_at,95033\_at,102335\_at,102873\_at,161990\_f\_at,94657\_at,95012\_at,96076\_at,96669\_at,100462\_at,99587\_at |
| 4 | cell homeostasis | 3 | 41 | 160 | 13100 | 0.019 | 0.003 | 5.99 | 0.014 | 99964\_at,103958\_g\_at,94976\_at |
| 5 | cell ion homeostasis | 3 | 37 | 144 | 11544 | 0.021 | 0.003 | 6.489 | 0.011 | 99964\_at,103958\_g\_at,94976\_at |
| 6 | cation homeostasis | 3 | 36 | 108 | 9498 | 0.028 | 0.004 | 7.33 | 0.008 | 99964\_at,103958\_g\_at,94976\_at |
| 7 | di-, tri-valent inorganic cation homeostasis | 3 | 29 | 79 | 6246 | 0.038 | 0.005 | 8.183 | 0.006 | 99964\_at,103958\_g\_at,94976\_at |
| 8 | calcium ion homeostasis | 1 | 13 | 23 | 2164 | 0.043 | 0.006 | 7.235 | 0.13 | 99964\_at |
| 8 | iron ion homeostasis | 2 | 15 | 23 | 2164 | 0.087 | 0.007 | 12.548 | 0.01 | 103958\_g\_at,94976\_at |
| 4 | cell organization and biogenesis | 4 | 530 | 160 | 13100 | 0.025 | 0.04 | 0.618 | 0.893 | 101843\_at,100115\_at,103236\_at,104376\_at |
| 5 | cell surface structure organization and biogenesis | 1 | 4 | 144 | 11544 | 0.007 | 0 | 19.829 | 0.049 | 101843\_at |
| 6 | formation of a cell surface projection | 1 | 4 | 108 | 9498 | 0.009 | 0 | 22.048 | 0.045 | 101843\_at |
| 7 | lamellipodium formation | 1 | 4 | 79 | 6246 | 0.013 | 0.001 | 19.781 | 0.05 | 101843\_at |
| 5 | cytoplasm organization and biogenesis | 1 | 380 | 144 | 11544 | 0.007 | 0.033 | 0.211 | 0.992 | 100115\_at |
| 6 | organelle organization and biogenesis | 1 | 318 | 108 | 9498 | 0.009 | 0.033 | 0.277 | 0.975 | 100115\_at |
| 7 | cytoskeleton organization and biogenesis | 1 | 262 | 79 | 6246 | 0.013 | 0.042 | 0.302 | 0.967 | 100115\_at |
| 8 | microtubule-based process | 1 | 119 | 23 | 2164 | 0.043 | 0.055 | 0.791 | 0.73 | 100115\_at |
| 5 | nuclear organization and biogenesis | 2 | 112 | 144 | 11544 | 0.014 | 0.01 | 1.432 | 0.409 | 103236\_at,104376\_at |
| 6 | chromosome organization and biogenesis (sensu Eukarya) | 2 | 108 | 108 | 9498 | 0.019 | 0.011 | 1.629 | 0.348 | 103236\_at,104376\_at |
| 7 | establishment and/or maintenance of chromatin architecture | 2 | 80 | 79 | 6246 | 0.025 | 0.013 | 1.977 | 0.269 | 103236\_at,104376\_at |
| 8 | chromatin modification | 2 | 36 | 23 | 2164 | 0.087 | 0.017 | 5.226 | 0.055 | 103236\_at,104376\_at |
| 4 | cell proliferation | 9 | 501 | 160 | 13100 | 0.056 | 0.038 | 1.471 | 0.16 | 95618\_at,95805\_at,99440\_at,103091\_at,96147\_at,97813\_at,97375\_at,98402\_at,95033\_at |
| 5 | cell cycle | 8 | 435 | 144 | 11544 | 0.056 | 0.038 | 1.475 | 0.177 | 95618\_at,95805\_at,99440\_at,103091\_at,96147\_at,97813\_at,97375\_at,98402\_at |
| 6 | DNA replication and chromosome cycle | 1 | 113 | 108 | 9498 | 0.009 | 0.012 | 0.778 | 0.727 | 99440\_at |
| 7 | DNA replication | 1 | 94 | 79 | 6246 | 0.013 | 0.015 | 0.841 | 0.7 | 99440\_at |
| 6 | regulation of cell cycle | 5 | 204 | 108 | 9498 | 0.046 | 0.021 | 2.155 | 0.083 | 103091\_at,96147\_at,97813\_at,97375\_at,98402\_at |
| 7 | cell cycle arrest | 2 | 20 | 79 | 6246 | 0.025 | 0.003 | 7.912 | 0.026 | 97375\_at,98402\_at |
| 5 | cytokinesis | 1 | 5 | 144 | 11544 | 0.007 | 0 | 16.14 | 0.061 | 95033\_at |
| 5 | regulation of cell proliferation | 1 | 38 | 144 | 11544 | 0.007 | 0.003 | 2.109 | 0.38 | 96147\_at |
| 4 | transport | 11 | 1083 | 160 | 13100 | 0.069 | 0.083 | 0.832 | 0.78 | 102335\_at,102873\_at,161990\_f\_at,94657\_at,95012\_at,96076\_at,96669\_at,100462\_at,99587\_at,97375\_at,103958\_g\_at |
| 5 | intracellular transport | 4 | 351 | 144 | 11544 | 0.028 | 0.03 | 0.914 | 0.642 | 100462\_at,96076\_at,96669\_at,99587\_at |
| 6 | intracellular protein transport | 4 | 284 | 108 | 9498 | 0.037 | 0.03 | 1.239 | 0.405 | 100462\_at,96076\_at,96669\_at,99587\_at |
| 7 | protein targeting | 1 | 101 | 79 | 6246 | 0.013 | 0.016 | 0.783 | 0.726 | 96669\_at |
| 5 | ion transport | 3 | 335 | 144 | 11544 | 0.021 | 0.029 | 0.718 | 0.793 | 102335\_at,94657\_at,97375\_at |
| 6 | anion transport | 1 | 79 | 108 | 9498 | 0.009 | 0.008 | 1.113 | 0.596 | 94657\_at |
| 7 | inorganic anion transport | 1 | 50 | 79 | 6246 | 0.013 | 0.008 | 1.581 | 0.472 | 94657\_at |
| 8 | chloride transport | 1 | 39 | 23 | 2164 | 0.043 | 0.018 | 2.413 | 0.343 | 94657\_at |
| 6 | cation transport | 2 | 236 | 108 | 9498 | 0.019 | 0.025 | 0.745 | 0.754 | 97375\_at,102335\_at |
| 7 | di-, tri-valent inorganic cation transport | 1 | 58 | 79 | 6246 | 0.013 | 0.009 | 1.363 | 0.524 | 97375\_at |
| 8 | calcium ion transport | 1 | 33 | 23 | 2164 | 0.043 | 0.015 | 2.851 | 0.299 | 97375\_at |
| 7 | metal ion transport | 2 | 184 | 79 | 6246 | 0.025 | 0.029 | 0.859 | 0.682 | 97375\_at,102335\_at |
| 8 | calcium ion transport | 1 | 33 | 23 | 2164 | 0.043 | 0.015 | 2.851 | 0.299 | 97375\_at |
| 8 | potassium ion transport | 1 | 94 | 23 | 2164 | 0.043 | 0.043 | 1.001 | 0.642 | 102335\_at |
| 5 | peptide transport | 1 | 5 | 144 | 11544 | 0.007 | 0 | 16.14 | 0.061 | 102873\_at |
| 6 | oligopeptide transport | 1 | 5 | 108 | 9498 | 0.009 | 0.001 | 17.472 | 0.056 | 102873\_at |
| 5 | protein transport | 4 | 297 | 144 | 11544 | 0.028 | 0.026 | 1.08 | 0.51 | 100462\_at,96669\_at,99587\_at,96076\_at |
| 6 | intracellular protein transport | 4 | 284 | 108 | 9498 | 0.037 | 0.03 | 1.239 | 0.405 | 100462\_at,96076\_at,96669\_at,99587\_at |
| 7 | protein targeting | 1 | 101 | 79 | 6246 | 0.013 | 0.016 | 0.783 | 0.726 | 96669\_at |
| 5 | vesicle-mediated transport | 3 | 112 | 144 | 11544 | 0.021 | 0.01 | 2.147 | 0.164 | 96076\_at,103958\_g\_at,100462\_at |
| 6 | endocytosis | 1 | 61 | 108 | 9498 | 0.009 | 0.006 | 1.442 | 0.503 | 103958\_g\_at |
| 6 | nonselective vesicle transport | 1 | 7 | 108 | 9498 | 0.009 | 0.001 | 12.514 | 0.077 | 100462\_at |
| 3 | cell motility | 2 | 188 | 138 | 10726 | 0.014 | 0.018 | 0.827 | 0.7 | 101843\_at,161184\_f\_at |
| 4 | cell migration | 1 | 53 | 160 | 13100 | 0.006 | 0.004 | 1.543 | 0.479 | 161184\_f\_at |
| 5 | regulation of cell migration | 1 | 17 | 144 | 11544 | 0.007 | 0.001 | 4.721 | 0.192 | 161184\_f\_at |
| 6 | negative regulation of cell migration | 1 | 3 | 108 | 9498 | 0.009 | 0 | 28.938 | 0.034 | 161184\_f\_at |
| 3 | membrane fusion | 1 | 4 | 138 | 10726 | 0.007 | 0 | 19.595 | 0.05 | 161184\_f\_at |
| 4 | plasma membrane fusion | 1 | 3 | 160 | 13100 | 0.006 | 0 | 27.174 | 0.036 | 161184\_f\_at |
| 2 | development | 15 | 990 | 138 | 10540 | 0.109 | 0.094 | 1.157 | 0.315 | 103666\_at,104243\_r\_at,104292\_at,160498\_at,99964\_at,160463\_at,96088\_at,160727\_at,102224\_at,97813\_at,95016\_at,161184\_f\_at,97375\_at,92249\_g\_at,99440\_at |
| 3 | cell differentiation | 4 | 137 | 138 | 10726 | 0.029 | 0.013 | 2.27 | 0.1 | 160463\_at,96088\_at,104243\_r\_at,160727\_at |
| 4 | epidermal cell differentiation | 1 | 3 | 160 | 13100 | 0.006 | 0 | 27.174 | 0.036 | 104243\_r\_at |
| 5 | hair cell differentiation | 1 | 3 | 144 | 11544 | 0.007 | 0 | 26.692 | 0.037 | 104243\_r\_at |
| 4 | vasculogenesis | 1 | 7 | 160 | 13100 | 0.006 | 0.001 | 11.792 | 0.082 | 160727\_at |
| 3 | morphogenesis | 11 | 594 | 138 | 10726 | 0.08 | 0.055 | 1.439 | 0.143 | 102224\_at,103666\_at,97813\_at,99964\_at,160727\_at,95016\_at,161184\_f\_at,97375\_at,92249\_g\_at,104243\_r\_at,99440\_at |
| 4 | organogenesis | 11 | 544 | 160 | 13100 | 0.069 | 0.042 | 1.655 | 0.07 | 102224\_at,103666\_at,97813\_at,99964\_at,160727\_at,95016\_at,161184\_f\_at,97375\_at,92249\_g\_at,104243\_r\_at,99440\_at |
| 5 | blood vessel development | 3 | 61 | 144 | 11544 | 0.021 | 0.005 | 3.945 | 0.04 | 160727\_at,95016\_at,161184\_f\_at |
| 6 | angiogenesis | 2 | 50 | 108 | 9498 | 0.019 | 0.005 | 3.521 | 0.11 | 95016\_at,161184\_f\_at |
| 7 | regulation of angiogenesis | 1 | 11 | 79 | 6246 | 0.013 | 0.002 | 7.193 | 0.131 | 161184\_f\_at |
| 8 | negative regulation of angiogenesis | 1 | 8 | 23 | 2164 | 0.043 | 0.004 | 11.751 | 0.082 | 161184\_f\_at |
| 5 | heart development | 1 | 31 | 144 | 11544 | 0.007 | 0.003 | 2.58 | 0.323 | 97375\_at |
| 5 | neurogenesis | 2 | 164 | 144 | 11544 | 0.014 | 0.014 | 0.977 | 0.61 | 92249\_g\_at,160727\_at |
| 6 | nerve maturation | 1 | 10 | 108 | 9498 | 0.009 | 0.001 | 8.819 | 0.108 | 160727\_at |
| 7 | nerve ensheathment | 1 | 10 | 79 | 6246 | 0.013 | 0.002 | 7.912 | 0.12 | 160727\_at |
| 5 | respiratory tube development | 2 | 10 | 144 | 11544 | 0.014 | 0.001 | 15.966 | 0.007 | 104243\_r\_at,99440\_at |
| 6 | lung development | 2 | 10 | 108 | 9498 | 0.019 | 0.001 | 17.638 | 0.005 | 104243\_r\_at,99440\_at |
| 5 | skeletal development | 2 | 52 | 144 | 11544 | 0.014 | 0.004 | 3.087 | 0.137 | 99964\_at,97375\_at |
| 6 | cartilage condensation | 1 | 5 | 108 | 9498 | 0.009 | 0.001 | 17.472 | 0.056 | 97375\_at |
| 4 | vasculogenesis | 1 | 7 | 160 | 13100 | 0.006 | 0.001 | 11.792 | 0.082 | 160727\_at |
| 3 | pattern specification | 1 | 83 | 138 | 10726 | 0.007 | 0.008 | 0.937 | 0.66 | 103666\_at |
| 2 | physiological processes | 72 | 5866 | 138 | 10540 | 0.522 | 0.557 | 0.937 | 0.82 | 103091\_at,95618\_at,97813\_at,99964\_at,103958\_g\_at,94976\_at,101843\_at,100115\_at,103236\_at,104376\_at,95805\_at,99440\_at,96147\_at,97375\_at,98402\_at,95033\_at,102335\_at,102873\_at,161990\_f\_at,94657\_at,95012\_at,96076\_at,96669\_at,100462\_at,99587\_at,104292\_at,161392\_f\_at,98861\_at,162034\_r\_at,96790\_f\_at,104139\_at,102302\_at,94796\_at,97563\_f\_at,95599\_at,160789\_at,160606\_r\_at,96270\_at,99970\_at,92821\_at,92249\_g\_at,161348\_r\_at,104243\_r\_at,102789\_at,103052\_r\_at,103547\_at,103666\_at,104438\_at,104645\_at,160244\_at,161148\_f\_at,93425\_at,97901\_at,93702\_at,98385\_at,102224\_at,104417\_at,161184\_f\_at,104287\_at,104363\_at,98426\_at,98906\_at,100696\_at,94146\_at,93875\_at,160651\_at,97125\_f\_at,98438\_f\_at,100972\_s\_at,103954\_at,100583\_at,101352\_g\_at |
| 3 | cell growth and/or maintenance | 25 | 2128 | 138 | 10726 | 0.181 | 0.198 | 0.913 | 0.727 | 103091\_at,95618\_at,97813\_at,99964\_at,103958\_g\_at,94976\_at,101843\_at,100115\_at,103236\_at,104376\_at,95805\_at,99440\_at,96147\_at,97375\_at,98402\_at,95033\_at,102335\_at,102873\_at,161990\_f\_at,94657\_at,95012\_at,96076\_at,96669\_at,100462\_at,99587\_at |
| 4 | cell homeostasis | 3 | 41 | 160 | 13100 | 0.019 | 0.003 | 5.99 | 0.014 | 99964\_at,103958\_g\_at,94976\_at |
| 5 | cell ion homeostasis | 3 | 37 | 144 | 11544 | 0.021 | 0.003 | 6.489 | 0.011 | 99964\_at,103958\_g\_at,94976\_at |
| 6 | cation homeostasis | 3 | 36 | 108 | 9498 | 0.028 | 0.004 | 7.33 | 0.008 | 99964\_at,103958\_g\_at,94976\_at |
| 7 | di-, tri-valent inorganic cation homeostasis | 3 | 29 | 79 | 6246 | 0.038 | 0.005 | 8.183 | 0.006 | 99964\_at,103958\_g\_at,94976\_at |
| 8 | calcium ion homeostasis | 1 | 13 | 23 | 2164 | 0.043 | 0.006 | 7.235 | 0.13 | 99964\_at |
| 8 | iron ion homeostasis | 2 | 15 | 23 | 2164 | 0.087 | 0.007 | 12.548 | 0.01 | 103958\_g\_at,94976\_at |
| 4 | cell organization and biogenesis | 4 | 530 | 160 | 13100 | 0.025 | 0.04 | 0.618 | 0.893 | 101843\_at,100115\_at,103236\_at,104376\_at |
| 5 | cell surface structure organization and biogenesis | 1 | 4 | 144 | 11544 | 0.007 | 0 | 19.829 | 0.049 | 101843\_at |
| 6 | formation of a cell surface projection | 1 | 4 | 108 | 9498 | 0.009 | 0 | 22.048 | 0.045 | 101843\_at |
| 7 | lamellipodium formation | 1 | 4 | 79 | 6246 | 0.013 | 0.001 | 19.781 | 0.05 | 101843\_at |
| 5 | cytoplasm organization and biogenesis | 1 | 380 | 144 | 11544 | 0.007 | 0.033 | 0.211 | 0.992 | 100115\_at |
| 6 | organelle organization and biogenesis | 1 | 318 | 108 | 9498 | 0.009 | 0.033 | 0.277 | 0.975 | 100115\_at |
| 7 | cytoskeleton organization and biogenesis | 1 | 262 | 79 | 6246 | 0.013 | 0.042 | 0.302 | 0.967 | 100115\_at |
| 8 | microtubule-based process | 1 | 119 | 23 | 2164 | 0.043 | 0.055 | 0.791 | 0.73 | 100115\_at |
| 5 | nuclear organization and biogenesis | 2 | 112 | 144 | 11544 | 0.014 | 0.01 | 1.432 | 0.409 | 103236\_at,104376\_at |
| 6 | chromosome organization and biogenesis (sensu Eukarya) | 2 | 108 | 108 | 9498 | 0.019 | 0.011 | 1.629 | 0.348 | 103236\_at,104376\_at |
| 7 | establishment and/or maintenance of chromatin architecture | 2 | 80 | 79 | 6246 | 0.025 | 0.013 | 1.977 | 0.269 | 103236\_at,104376\_at |
| 8 | chromatin modification | 2 | 36 | 23 | 2164 | 0.087 | 0.017 | 5.226 | 0.055 | 103236\_at,104376\_at |
| 4 | cell proliferation | 9 | 501 | 160 | 13100 | 0.056 | 0.038 | 1.471 | 0.16 | 95618\_at,95805\_at,99440\_at,103091\_at,96147\_at,97813\_at,97375\_at,98402\_at,95033\_at |
| 5 | cell cycle | 8 | 435 | 144 | 11544 | 0.056 | 0.038 | 1.475 | 0.177 | 95618\_at,95805\_at,99440\_at,103091\_at,96147\_at,97813\_at,97375\_at,98402\_at |
| 6 | DNA replication and chromosome cycle | 1 | 113 | 108 | 9498 | 0.009 | 0.012 | 0.778 | 0.727 | 99440\_at |
| 7 | DNA replication | 1 | 94 | 79 | 6246 | 0.013 | 0.015 | 0.841 | 0.7 | 99440\_at |
| 6 | regulation of cell cycle | 5 | 204 | 108 | 9498 | 0.046 | 0.021 | 2.155 | 0.083 | 103091\_at,96147\_at,97813\_at,97375\_at,98402\_at |
| 7 | cell cycle arrest | 2 | 20 | 79 | 6246 | 0.025 | 0.003 | 7.912 | 0.026 | 97375\_at,98402\_at |
| 5 | cytokinesis | 1 | 5 | 144 | 11544 | 0.007 | 0 | 16.14 | 0.061 | 95033\_at |
| 5 | regulation of cell proliferation | 1 | 38 | 144 | 11544 | 0.007 | 0.003 | 2.109 | 0.38 | 96147\_at |
| 4 | transport | 11 | 1083 | 160 | 13100 | 0.069 | 0.083 | 0.832 | 0.78 | 102335\_at,102873\_at,161990\_f\_at,94657\_at,95012\_at,96076\_at,96669\_at,100462\_at,99587\_at,97375\_at,103958\_g\_at |
| 5 | intracellular transport | 4 | 351 | 144 | 11544 | 0.028 | 0.03 | 0.914 | 0.642 | 100462\_at,96076\_at,96669\_at,99587\_at |
| 6 | intracellular protein transport | 4 | 284 | 108 | 9498 | 0.037 | 0.03 | 1.239 | 0.405 | 100462\_at,96076\_at,96669\_at,99587\_at |
| 7 | protein targeting | 1 | 101 | 79 | 6246 | 0.013 | 0.016 | 0.783 | 0.726 | 96669\_at |
| 5 | ion transport | 3 | 335 | 144 | 11544 | 0.021 | 0.029 | 0.718 | 0.793 | 102335\_at,94657\_at,97375\_at |
| 6 | anion transport | 1 | 79 | 108 | 9498 | 0.009 | 0.008 | 1.113 | 0.596 | 94657\_at |
| 7 | inorganic anion transport | 1 | 50 | 79 | 6246 | 0.013 | 0.008 | 1.581 | 0.472 | 94657\_at |
| 8 | chloride transport | 1 | 39 | 23 | 2164 | 0.043 | 0.018 | 2.413 | 0.343 | 94657\_at |
| 6 | cation transport | 2 | 236 | 108 | 9498 | 0.019 | 0.025 | 0.745 | 0.754 | 97375\_at,102335\_at |
| 7 | di-, tri-valent inorganic cation transport | 1 | 58 | 79 | 6246 | 0.013 | 0.009 | 1.363 | 0.524 | 97375\_at |
| 8 | calcium ion transport | 1 | 33 | 23 | 2164 | 0.043 | 0.015 | 2.851 | 0.299 | 97375\_at |
| 7 | metal ion transport | 2 | 184 | 79 | 6246 | 0.025 | 0.029 | 0.859 | 0.682 | 97375\_at,102335\_at |
| 8 | calcium ion transport | 1 | 33 | 23 | 2164 | 0.043 | 0.015 | 2.851 | 0.299 | 97375\_at |
| 8 | potassium ion transport | 1 | 94 | 23 | 2164 | 0.043 | 0.043 | 1.001 | 0.642 | 102335\_at |
| 5 | peptide transport | 1 | 5 | 144 | 11544 | 0.007 | 0 | 16.14 | 0.061 | 102873\_at |
| 6 | oligopeptide transport | 1 | 5 | 108 | 9498 | 0.009 | 0.001 | 17.472 | 0.056 | 102873\_at |
| 5 | protein transport | 4 | 297 | 144 | 11544 | 0.028 | 0.026 | 1.08 | 0.51 | 100462\_at,96669\_at,99587\_at,96076\_at |
| 6 | intracellular protein transport | 4 | 284 | 108 | 9498 | 0.037 | 0.03 | 1.239 | 0.405 | 100462\_at,96076\_at,96669\_at,99587\_at |
| 7 | protein targeting | 1 | 101 | 79 | 6246 | 0.013 | 0.016 | 0.783 | 0.726 | 96669\_at |
| 5 | vesicle-mediated transport | 3 | 112 | 144 | 11544 | 0.021 | 0.01 | 2.147 | 0.164 | 96076\_at,103958\_g\_at,100462\_at |
| 6 | endocytosis | 1 | 61 | 108 | 9498 | 0.009 | 0.006 | 1.442 | 0.503 | 103958\_g\_at |
| 6 | nonselective vesicle transport | 1 | 7 | 108 | 9498 | 0.009 | 0.001 | 12.514 | 0.077 | 100462\_at |
| 3 | metabolism | 53 | 3908 | 138 | 10726 | 0.384 | 0.364 | 1.054 | 0.344 | 104292\_at,161392\_f\_at,94976\_at,98861\_at,162034\_r\_at,96790\_f\_at,104139\_at,102302\_at,94796\_at,97563\_f\_at,95012\_at,95599\_at,160789\_at,98402\_at,103958\_g\_at,160606\_r\_at,96270\_at,99970\_at,92821\_at,161990\_f\_at,92249\_g\_at,99964\_at,161348\_r\_at,104243\_r\_at,104376\_at,102789\_at,103052\_r\_at,103091\_at,103236\_at,103547\_at,103666\_at,104438\_at,104645\_at,160244\_at,161148\_f\_at,93425\_at,95618\_at,96147\_at,97813\_at,97901\_at,99440\_at,99587\_at,93702\_at,98385\_at,102224\_at,104417\_at,161184\_f\_at,95805\_at,104287\_at,96669\_at,104363\_at,98426\_at,98906\_at |
| 4 | alcohol metabolism | 2 | 167 | 160 | 13100 | 0.012 | 0.013 | 0.98 | 0.608 | 162034\_r\_at,96790\_f\_at |
| 5 | alcohol catabolism | 1 | 58 | 144 | 11544 | 0.007 | 0.005 | 1.382 | 0.518 | 162034\_r\_at |
| 6 | monosaccharide catabolism | 1 | 58 | 108 | 9498 | 0.009 | 0.006 | 1.516 | 0.486 | 162034\_r\_at |
| 7 | hexose catabolism | 1 | 58 | 79 | 6246 | 0.013 | 0.009 | 1.363 | 0.524 | 162034\_r\_at |
| 8 | glucose catabolism | 1 | 58 | 23 | 2164 | 0.043 | 0.027 | 1.622 | 0.466 | 162034\_r\_at |
| 9 | glycolysis | 1 | 52 | 6 | 911 | 0.167 | 0.057 | 2.92 | 0.298 | 162034\_r\_at |
| 5 | monosaccharide metabolism | 2 | 108 | 144 | 11544 | 0.014 | 0.009 | 1.484 | 0.391 | 162034\_r\_at,96790\_f\_at |
| 6 | monosaccharide catabolism | 1 | 58 | 108 | 9498 | 0.009 | 0.006 | 1.516 | 0.486 | 162034\_r\_at |
| 7 | hexose catabolism | 1 | 58 | 79 | 6246 | 0.013 | 0.009 | 1.363 | 0.524 | 162034\_r\_at |
| 8 | glucose catabolism | 1 | 58 | 23 | 2164 | 0.043 | 0.027 | 1.622 | 0.466 | 162034\_r\_at |
| 9 | glycolysis | 1 | 52 | 6 | 911 | 0.167 | 0.057 | 2.92 | 0.298 | 162034\_r\_at |
| 6 | hexose metabolism | 2 | 107 | 108 | 9498 | 0.019 | 0.011 | 1.643 | 0.344 | 162034\_r\_at,96790\_f\_at |
| 7 | hexose catabolism | 1 | 58 | 79 | 6246 | 0.013 | 0.009 | 1.363 | 0.524 | 162034\_r\_at |
| 8 | glucose catabolism | 1 | 58 | 23 | 2164 | 0.043 | 0.027 | 1.622 | 0.466 | 162034\_r\_at |
| 9 | glycolysis | 1 | 52 | 6 | 911 | 0.167 | 0.057 | 2.92 | 0.298 | 162034\_r\_at |
| 7 | galactose metabolism | 1 | 13 | 79 | 6246 | 0.013 | 0.002 | 6.087 | 0.153 | 96790\_f\_at |
| 4 | amine metabolism | 3 | 148 | 160 | 13100 | 0.019 | 0.011 | 1.659 | 0.271 | 104139\_at,161392\_f\_at,102302\_at |
| 5 | 4-hydroxyproline metabolism | 1 | 5 | 144 | 11544 | 0.007 | 0 | 16.14 | 0.061 | 104139\_at |
| 6 | peptidyl-proline hydroxylation to 4-hydroxy-L-proline | 1 | 5 | 108 | 9498 | 0.009 | 0.001 | 17.472 | 0.056 | 104139\_at |
| 5 | amine biosynthesis | 1 | 35 | 144 | 11544 | 0.007 | 0.003 | 2.29 | 0.356 | 161392\_f\_at |
| 6 | amino acid biosynthesis | 1 | 25 | 108 | 9498 | 0.009 | 0.003 | 3.521 | 0.249 | 161392\_f\_at |
| 7 | glutamine family amino acid biosynthesis | 1 | 7 | 79 | 6246 | 0.013 | 0.001 | 11.304 | 0.085 | 161392\_f\_at |
| 8 | glutamine biosynthesis | 1 | 2 | 23 | 2164 | 0.043 | 0.001 | 47.261 | 0.021 | 161392\_f\_at |
| 9 | proline biosynthesis | 1 | 2 | 6 | 911 | 0.167 | 0.002 | 75.759 | 0.013 | 161392\_f\_at |
| 5 | amine catabolism | 1 | 30 | 144 | 11544 | 0.007 | 0.003 | 2.669 | 0.314 | 102302\_at |
| 6 | amino acid catabolism | 1 | 24 | 108 | 9498 | 0.009 | 0.003 | 3.66 | 0.24 | 102302\_at |
| 4 | biosynthesis | 7 | 652 | 160 | 13100 | 0.044 | 0.05 | 0.879 | 0.69 | 161392\_f\_at,94796\_at,97563\_f\_at,95012\_at,95599\_at,160789\_at,94976\_at |
| 5 | amine biosynthesis | 1 | 35 | 144 | 11544 | 0.007 | 0.003 | 2.29 | 0.356 | 161392\_f\_at |
| 6 | amino acid biosynthesis | 1 | 25 | 108 | 9498 | 0.009 | 0.003 | 3.521 | 0.249 | 161392\_f\_at |
| 7 | glutamine family amino acid biosynthesis | 1 | 7 | 79 | 6246 | 0.013 | 0.001 | 11.304 | 0.085 | 161392\_f\_at |
| 8 | glutamine biosynthesis | 1 | 2 | 23 | 2164 | 0.043 | 0.001 | 47.261 | 0.021 | 161392\_f\_at |
| 9 | proline biosynthesis | 1 | 2 | 6 | 911 | 0.167 | 0.002 | 75.759 | 0.013 | 161392\_f\_at |
| 5 | hormone biosynthesis | 1 | 19 | 144 | 11544 | 0.007 | 0.002 | 4.206 | 0.212 | 94796\_at |
| 6 | C21-steroid hormone biosynthesis | 1 | 17 | 108 | 9498 | 0.009 | 0.002 | 5.173 | 0.177 | 94796\_at |
| 5 | lipid biosynthesis | 1 | 124 | 144 | 11544 | 0.007 | 0.011 | 0.646 | 0.791 | 94796\_at |
| 6 | steroid biosynthesis | 1 | 45 | 108 | 9498 | 0.009 | 0.005 | 1.954 | 0.403 | 94796\_at |
| 6 | C21-steroid hormone biosynthesis | 1 | 17 | 108 | 9498 | 0.009 | 0.002 | 5.173 | 0.177 | 94796\_at |
| 5 | macromolecule biosynthesis | 5 | 322 | 144 | 11544 | 0.035 | 0.028 | 1.245 | 0.374 | 97563\_f\_at,95012\_at,95599\_at,160789\_at,94976\_at |
| 6 | protein biosynthesis | 5 | 322 | 108 | 9498 | 0.046 | 0.034 | 1.366 | 0.304 | 97563\_f\_at,95012\_at,95599\_at,160789\_at,94976\_at |
| 7 | amino acid activation | 1 | 36 | 79 | 6246 | 0.013 | 0.006 | 2.198 | 0.368 | 95012\_at |
| 7 | glycoprotein biosynthesis | 2 | 52 | 79 | 6246 | 0.025 | 0.008 | 3.04 | 0.14 | 95599\_at,160789\_at |
| 8 | protein amino acid glycosylation | 2 | 52 | 23 | 2164 | 0.087 | 0.024 | 3.619 | 0.104 | 95599\_at,160789\_at |
| 9 | N-linked glycosylation | 1 | 12 | 6 | 911 | 0.167 | 0.013 | 12.655 | 0.077 | 160789\_at |
| 7 | regulation of translation | 1 | 24 | 79 | 6246 | 0.013 | 0.004 | 3.297 | 0.264 | 94976\_at |
| 7 | translational elongation | 1 | 22 | 79 | 6246 | 0.013 | 0.004 | 3.597 | 0.245 | 97563\_f\_at |
| 4 | carbohydrate metabolism | 4 | 231 | 160 | 13100 | 0.025 | 0.018 | 1.418 | 0.312 | 160789\_at,98402\_at,162034\_r\_at,96790\_f\_at |
| 5 | monosaccharide metabolism | 2 | 108 | 144 | 11544 | 0.014 | 0.009 | 1.484 | 0.391 | 162034\_r\_at,96790\_f\_at |
| 6 | monosaccharide catabolism | 1 | 58 | 108 | 9498 | 0.009 | 0.006 | 1.516 | 0.486 | 162034\_r\_at |
| 7 | hexose catabolism | 1 | 58 | 79 | 6246 | 0.013 | 0.009 | 1.363 | 0.524 | 162034\_r\_at |
| 8 | glucose catabolism | 1 | 58 | 23 | 2164 | 0.043 | 0.027 | 1.622 | 0.466 | 162034\_r\_at |
| 9 | glycolysis | 1 | 52 | 6 | 911 | 0.167 | 0.057 | 2.92 | 0.298 | 162034\_r\_at |
| 6 | hexose metabolism | 2 | 107 | 108 | 9498 | 0.019 | 0.011 | 1.643 | 0.344 | 162034\_r\_at,96790\_f\_at |
| 7 | hexose catabolism | 1 | 58 | 79 | 6246 | 0.013 | 0.009 | 1.363 | 0.524 | 162034\_r\_at |
| 8 | glucose catabolism | 1 | 58 | 23 | 2164 | 0.043 | 0.027 | 1.622 | 0.466 | 162034\_r\_at |
| 9 | glycolysis | 1 | 52 | 6 | 911 | 0.167 | 0.057 | 2.92 | 0.298 | 162034\_r\_at |
| 7 | galactose metabolism | 1 | 13 | 79 | 6246 | 0.013 | 0.002 | 6.087 | 0.153 | 96790\_f\_at |
| 4 | catabolism | 7 | 631 | 160 | 13100 | 0.044 | 0.048 | 0.908 | 0.656 | 162034\_r\_at,102302\_at,103958\_g\_at,160606\_r\_at,96270\_at,99970\_at,92821\_at |
| 5 | alcohol catabolism | 1 | 58 | 144 | 11544 | 0.007 | 0.005 | 1.382 | 0.518 | 162034\_r\_at |
| 6 | monosaccharide catabolism | 1 | 58 | 108 | 9498 | 0.009 | 0.006 | 1.516 | 0.486 | 162034\_r\_at |
| 7 | hexose catabolism | 1 | 58 | 79 | 6246 | 0.013 | 0.009 | 1.363 | 0.524 | 162034\_r\_at |
| 8 | glucose catabolism | 1 | 58 | 23 | 2164 | 0.043 | 0.027 | 1.622 | 0.466 | 162034\_r\_at |
| 9 | glycolysis | 1 | 52 | 6 | 911 | 0.167 | 0.057 | 2.92 | 0.298 | 162034\_r\_at |
| 5 | amine catabolism | 1 | 30 | 144 | 11544 | 0.007 | 0.003 | 2.669 | 0.314 | 102302\_at |
| 6 | amino acid catabolism | 1 | 24 | 108 | 9498 | 0.009 | 0.003 | 3.66 | 0.24 | 102302\_at |
| 5 | macromolecule catabolism | 5 | 470 | 144 | 11544 | 0.035 | 0.041 | 0.853 | 0.703 | 103958\_g\_at,160606\_r\_at,96270\_at,99970\_at,92821\_at |
| 6 | protein catabolism | 5 | 466 | 108 | 9498 | 0.046 | 0.049 | 0.944 | 0.617 | 103958\_g\_at,160606\_r\_at,96270\_at,99970\_at,92821\_at |
| 7 | proteolysis and peptidolysis | 5 | 457 | 79 | 6246 | 0.063 | 0.073 | 0.865 | 0.696 | 103958\_g\_at,160606\_r\_at,96270\_at,99970\_at,92821\_at |
| 8 | modification-dependent protein catabolism | 1 | 122 | 23 | 2164 | 0.043 | 0.056 | 0.771 | 0.739 | 92821\_at |
| 9 | ubiquitin-dependent protein catabolism | 1 | 120 | 6 | 911 | 0.167 | 0.132 | 1.265 | 0.573 | 92821\_at |
| 4 | lipid metabolism | 3 | 285 | 160 | 13100 | 0.019 | 0.022 | 0.862 | 0.68 | 94796\_at,161990\_f\_at,98861\_at |
| 5 | lipid biosynthesis | 1 | 124 | 144 | 11544 | 0.007 | 0.011 | 0.646 | 0.791 | 94796\_at |
| 6 | steroid biosynthesis | 1 | 45 | 108 | 9498 | 0.009 | 0.005 | 1.954 | 0.403 | 94796\_at |
| 6 | C21-steroid hormone biosynthesis | 1 | 17 | 108 | 9498 | 0.009 | 0.002 | 5.173 | 0.177 | 94796\_at |
| 5 | fatty acid metabolism | 1 | 85 | 144 | 11544 | 0.007 | 0.007 | 0.943 | 0.657 | 161990\_f\_at |
| 5 | steroid metabolism | 2 | 74 | 144 | 11544 | 0.014 | 0.006 | 2.167 | 0.236 | 98861\_at,94796\_at |
| 6 | steroid biosynthesis | 1 | 45 | 108 | 9498 | 0.009 | 0.005 | 1.954 | 0.403 | 94796\_at |
| 6 | C21-steroid hormone biosynthesis | 1 | 17 | 108 | 9498 | 0.009 | 0.002 | 5.173 | 0.177 | 94796\_at |
| 4 | nucleobase, nucleoside, nucleotide and nucleic acid metabolism | 22 | 1530 | 160 | 13100 | 0.138 | 0.117 | 1.177 | 0.238 | 92249\_g\_at,99964\_at,161348\_r\_at,104243\_r\_at,104376\_at,102789\_at,103052\_r\_at,103091\_at,103236\_at,103547\_at,103666\_at,104438\_at,104645\_at,160244\_at,161148\_f\_at,93425\_at,95618\_at,96147\_at,97813\_at,97901\_at,99440\_at,99587\_at |
| 5 | transcription | 22 | 1086 | 144 | 11544 | 0.153 | 0.094 | 1.624 | 0.015 | 92249\_g\_at,99964\_at,161348\_r\_at,104243\_r\_at,104376\_at,102789\_at,103052\_r\_at,103091\_at,103236\_at,103547\_at,103666\_at,104438\_at,104645\_at,160244\_at,161148\_f\_at,93425\_at,95618\_at,96147\_at,97813\_at,97901\_at,99440\_at,99587\_at |
| 6 | regulation of transcription | 22 | 1026 | 108 | 9498 | 0.204 | 0.108 | 1.886 | 0.002 | 161348\_r\_at,104243\_r\_at,104376\_at,102789\_at,103052\_r\_at,103091\_at,103236\_at,103547\_at,103666\_at,104438\_at,104645\_at,160244\_at,161148\_f\_at,92249\_g\_at,93425\_at,95618\_at,96147\_at,97813\_at,97901\_at,99440\_at,99587\_at,99964\_at |
| 7 | negative regulation of transcription | 2 | 40 | 79 | 6246 | 0.025 | 0.006 | 3.956 | 0.091 | 104243\_r\_at,104376\_at |
| 8 | negative regulation of transcription, DNA-dependent | 2 | 27 | 23 | 2164 | 0.087 | 0.012 | 6.968 | 0.032 | 104243\_r\_at,104376\_at |
| 9 | negative regulation of transcription from Pol II promoter | 2 | 24 | 6 | 911 | 0.333 | 0.026 | 12.655 | 0.009 | 104243\_r\_at,104376\_at |
| 7 | regulation of transcription, DNA-dependent | 21 | 1013 | 79 | 6246 | 0.266 | 0.162 | 1.639 | 0.012 | 102789\_at,103052\_r\_at,103091\_at,103236\_at,103547\_at,103666\_at,104243\_r\_at,104376\_at,104438\_at,104645\_at,160244\_at,161148\_f\_at,92249\_g\_at,93425\_at,95618\_at,96147\_at,97813\_at,97901\_at,99440\_at,99587\_at,99964\_at |
| 8 | negative regulation of transcription, DNA-dependent | 2 | 27 | 23 | 2164 | 0.087 | 0.012 | 6.968 | 0.032 | 104243\_r\_at,104376\_at |
| 9 | negative regulation of transcription from Pol II promoter | 2 | 24 | 6 | 911 | 0.333 | 0.026 | 12.655 | 0.009 | 104243\_r\_at,104376\_at |
| 5 | two-component signal transduction system (phosphorelay) | 1 | 15 | 144 | 11544 | 0.007 | 0.001 | 5.338 | 0.172 | 99587\_at |
| 4 | phosphorus metabolism | 7 | 488 | 160 | 13100 | 0.044 | 0.037 | 1.174 | 0.387 | 93702\_at,98385\_at,99970\_at,102224\_at,104417\_at,161184\_f\_at,95805\_at |
| 5 | phosphate metabolism | 7 | 488 | 144 | 11544 | 0.049 | 0.042 | 1.15 | 0.408 | 93702\_at,98385\_at,99970\_at,102224\_at,104417\_at,161184\_f\_at,95805\_at |
| 6 | dephosphorylation | 3 | 92 | 108 | 9498 | 0.028 | 0.01 | 2.867 | 0.087 | 93702\_at,98385\_at,99970\_at |
| 7 | protein amino acid dephosphorylation | 3 | 92 | 79 | 6246 | 0.038 | 0.015 | 2.578 | 0.11 | 93702\_at,98385\_at,99970\_at |
| 6 | phosphorylation | 4 | 395 | 108 | 9498 | 0.037 | 0.042 | 0.891 | 0.663 | 102224\_at,104417\_at,161184\_f\_at,95805\_at |
| 7 | protein amino acid phosphorylation | 4 | 379 | 79 | 6246 | 0.051 | 0.061 | 0.834 | 0.715 | 102224\_at,104417\_at,161184\_f\_at,95805\_at |
| 4 | protein metabolism | 22 | 1458 | 160 | 13100 | 0.138 | 0.111 | 1.235 | 0.174 | 104139\_at,104287\_at,96669\_at,97563\_f\_at,95012\_at,95599\_at,160789\_at,94976\_at,103958\_g\_at,160606\_r\_at,96270\_at,99970\_at,92821\_at,104363\_at,98426\_at,98906\_at,93702\_at,98385\_at,102224\_at,104417\_at,161184\_f\_at,95805\_at |
| 5 | protein modification | 12 | 654 | 144 | 11544 | 0.083 | 0.057 | 1.471 | 0.116 | 104363\_at,98426\_at,98906\_at,95599\_at,160789\_at,93702\_at,98385\_at,99970\_at,102224\_at,104417\_at,161184\_f\_at,95805\_at |
| 6 | ubiquitin cycle | 2 | 56 | 108 | 9498 | 0.019 | 0.006 | 3.139 | 0.133 | 104363\_at,98906\_at |
| 7 | protein ubiquitination | 1 | 5 | 79 | 6246 | 0.013 | 0.001 | 15.825 | 0.062 | 98906\_at |
| 3 | pregnancy | 1 | 7 | 138 | 10726 | 0.007 | 0.001 | 11.154 | 0.087 | 98861\_at |
| 3 | response to external stimulus | 13 | 666 | 138 | 10726 | 0.094 | 0.062 | 1.517 | 0.087 | 100696\_at,97375\_at,94146\_at,93875\_at,160651\_at,97125\_f\_at,97813\_at,98438\_f\_at,100972\_s\_at,102873\_at,103954\_at,100583\_at,101352\_g\_at |
| 4 | perception of external stimulus | 2 | 84 | 160 | 13100 | 0.012 | 0.006 | 1.95 | 0.274 | 100696\_at,97375\_at |
| 5 | perception of abiotic stimulus | 1 | 72 | 144 | 11544 | 0.007 | 0.006 | 1.112 | 0.596 | 100696\_at |
| 6 | perception of light | 1 | 41 | 108 | 9498 | 0.009 | 0.004 | 2.144 | 0.375 | 100696\_at |
| 7 | vision | 1 | 34 | 79 | 6246 | 0.013 | 0.005 | 2.327 | 0.352 | 100696\_at |
| 5 | perception of mechanical stimulus | 1 | 3 | 144 | 11544 | 0.007 | 0 | 26.692 | 0.037 | 97375\_at |
| 6 | mechanosensory perception | 1 | 3 | 108 | 9498 | 0.009 | 0 | 28.938 | 0.034 | 97375\_at |
| 4 | response to abiotic stimulus | 3 | 195 | 160 | 13100 | 0.019 | 0.015 | 1.259 | 0.427 | 100696\_at,94146\_at,93875\_at |
| 5 | perception of abiotic stimulus | 1 | 72 | 144 | 11544 | 0.007 | 0.006 | 1.112 | 0.596 | 100696\_at |
| 6 | perception of light | 1 | 41 | 108 | 9498 | 0.009 | 0.004 | 2.144 | 0.375 | 100696\_at |
| 7 | vision | 1 | 34 | 79 | 6246 | 0.013 | 0.005 | 2.327 | 0.352 | 100696\_at |
| 5 | response to chemical substance | 1 | 90 | 144 | 11544 | 0.007 | 0.008 | 0.89 | 0.678 | 94146\_at |
| 6 | chemotaxis | 1 | 60 | 108 | 9498 | 0.009 | 0.006 | 1.465 | 0.498 | 94146\_at |
| 5 | response to temperature | 1 | 28 | 144 | 11544 | 0.007 | 0.002 | 2.856 | 0.297 | 93875\_at |
| 6 | response to heat | 1 | 27 | 108 | 9498 | 0.009 | 0.003 | 3.261 | 0.266 | 93875\_at |
| 4 | response to biotic stimulus | 10 | 516 | 160 | 13100 | 0.062 | 0.039 | 1.587 | 0.101 | 160651\_at,97125\_f\_at,97813\_at,98438\_f\_at,100972\_s\_at,102873\_at,94146\_at,103954\_at,100583\_at,101352\_g\_at |
| 5 | defense response | 10 | 471 | 144 | 11544 | 0.069 | 0.041 | 1.702 | 0.07 | 160651\_at,97125\_f\_at,97813\_at,98438\_f\_at,100972\_s\_at,102873\_at,94146\_at,103954\_at,100583\_at,101352\_g\_at |
| 6 | immune response | 8 | 362 | 108 | 9498 | 0.074 | 0.038 | 1.944 | 0.054 | 100972\_s\_at,102873\_at,94146\_at,97125\_f\_at,98438\_f\_at,103954\_at,100583\_at,101352\_g\_at |
| 7 | acute-phase response | 1 | 23 | 79 | 6246 | 0.013 | 0.004 | 3.44 | 0.254 | 103954\_at |
| 7 | antigen presentation | 2 | 26 | 79 | 6246 | 0.025 | 0.004 | 6.087 | 0.042 | 97125\_f\_at,98438\_f\_at |
| 8 | antigen presentation, endogenous antigen | 2 | 15 | 23 | 2164 | 0.087 | 0.007 | 12.548 | 0.01 | 97125\_f\_at,98438\_f\_at |
| 7 | antigen processing | 2 | 27 | 79 | 6246 | 0.025 | 0.004 | 5.861 | 0.045 | 97125\_f\_at,98438\_f\_at |
| 8 | antigen processing, endogenous antigen via MHC class I | 2 | 15 | 23 | 2164 | 0.087 | 0.007 | 12.548 | 0.01 | 97125\_f\_at,98438\_f\_at |
| 7 | humoral immune response | 2 | 103 | 79 | 6246 | 0.025 | 0.016 | 1.535 | 0.376 | 100583\_at,101352\_g\_at |
| 7 | innate immune response | 2 | 70 | 79 | 6246 | 0.025 | 0.011 | 2.259 | 0.222 | 103954\_at,94146\_at |
| 8 | inflammatory response | 2 | 70 | 23 | 2164 | 0.087 | 0.032 | 2.688 | 0.169 | 103954\_at,94146\_at |

  
