# Supplementary material for: Molecular Signatures of Proliferation and Quiescence in Hematopoietic Stem Cells
Source: PLoS Biol. 2004 Sep 28;2(10):e301. doi: 10.1371/journal.pbio.0020301 (PMC520599; doi:10.1371/journal.pbio.0020301)
Supplement: Table S40 — (65 KB HTML). [file pbio.0020301.st040.html]

   Full Tom Day 3   

# Full Tom Day 3

|  |  |  |  |  |  |  |  |  |  |  |
| --- | --- | --- | --- | --- | --- | --- | --- | --- | --- | --- |
| GOLevel | GOTerm | ProbeCount | ArrayCount | ListGOLevelCount | ArrayGoLevelCount | ListFq | ArrayFq | FoldChange | H-Pvalue | ProbeIds |
| 0 | Gene\_Ontology | 89 | NA | 89 | 0 | 1 | NA | NA | NA | 161232\_r\_at,102280\_at,161787\_f\_at,92593\_at,95292\_at,98140\_at,98993\_at,99475\_at,161134\_at,93459\_s\_at,99439\_at,97411\_at,97909\_at,103910\_at,93445\_at,96858\_at,93228\_at,98550\_at,99777\_s\_at,93333\_at,94788\_f\_at,95118\_r\_at,96710\_at,99457\_at,100062\_at,101958\_f\_at,103064\_at,103797\_at,103821\_at,160069\_at,160496\_s\_at,92478\_at,92647\_at,95527\_at,97095\_at,101920\_at,103207\_at,92551\_at,95612\_at,162009\_f\_at,94376\_s\_at,96891\_at,100225\_f\_at,102105\_f\_at,102134\_f\_at,161757\_f\_at,160518\_at,98956\_at,95611\_at,101445\_at,95732\_at,160595\_at,94534\_at,99658\_f\_at,101001\_at,102103\_f\_at,96916\_at,96629\_at,98922\_at,161492\_i\_at,102047\_at,95462\_at,161038\_at,98618\_at,98999\_at,104313\_at,99513\_at,95063\_at,95456\_r\_at,102848\_f\_at,93439\_f\_at,94078\_at,97492\_at,99631\_f\_at,99660\_f\_at,162327\_f\_at,98072\_r\_at,101589\_at,103944\_at,101957\_f\_at,101004\_f\_at,92782\_at,160377\_at,95131\_f\_at,99111\_at,103201\_at,97393\_at,103203\_f\_at,103204\_r\_at |
| 1 | biological\_process | 89 | 6769 | 89 | 6769 | 1 | 1 | 1 | 1 | 161232\_r\_at,102280\_at,161787\_f\_at,92593\_at,95292\_at,98140\_at,98993\_at,99475\_at,161134\_at,93459\_s\_at,99439\_at,97411\_at,97909\_at,103910\_at,93445\_at,96858\_at,93228\_at,98550\_at,99777\_s\_at,93333\_at,94788\_f\_at,95118\_r\_at,96710\_at,99457\_at,100062\_at,101958\_f\_at,103064\_at,103797\_at,103821\_at,160069\_at,160496\_s\_at,92478\_at,92647\_at,95527\_at,97095\_at,101920\_at,103207\_at,92551\_at,95612\_at,162009\_f\_at,94376\_s\_at,96891\_at,100225\_f\_at,102105\_f\_at,102134\_f\_at,161757\_f\_at,160518\_at,98956\_at,95611\_at,101445\_at,95732\_at,160595\_at,94534\_at,99658\_f\_at,101001\_at,102103\_f\_at,96916\_at,96629\_at,98922\_at,161492\_i\_at,102047\_at,95462\_at,161038\_at,98618\_at,98999\_at,104313\_at,99513\_at,95063\_at,95456\_r\_at,102848\_f\_at,93439\_f\_at,94078\_at,97492\_at,99631\_f\_at,99660\_f\_at,162327\_f\_at,98072\_r\_at,101589\_at,103944\_at,101957\_f\_at,101004\_f\_at,92782\_at,160377\_at,95131\_f\_at,99111\_at,103201\_at,97393\_at,103203\_f\_at,103204\_r\_at |
| 2 | behavior | 1 | 63 | 138 | 10540 | 0.007 | 0.006 | 1.212 | 0.565 | 161232\_r\_at |
| 3 | locomotory behavior | 1 | 23 | 139 | 10726 | 0.007 | 0.002 | 3.36 | 0.259 | 161232\_r\_at |
| 2 | cellular process | 49 | 3616 | 138 | 10540 | 0.355 | 0.343 | 1.035 | 0.414 | 102280\_at,161232\_r\_at,161787\_f\_at,92593\_at,95292\_at,98140\_at,98993\_at,99475\_at,161134\_at,93459\_s\_at,99439\_at,97411\_at,97909\_at,103910\_at,93445\_at,96858\_at,93228\_at,98550\_at,99777\_s\_at,93333\_at,94788\_f\_at,95118\_r\_at,96710\_at,99457\_at,100062\_at,101958\_f\_at,103064\_at,103797\_at,103821\_at,160069\_at,160496\_s\_at,92478\_at,92647\_at,95527\_at,97095\_at,101920\_at,103207\_at,92551\_at,95612\_at,162009\_f\_at,94376\_s\_at,96891\_at,100225\_f\_at,102105\_f\_at,102134\_f\_at,161757\_f\_at,160518\_at,98956\_at,95611\_at |
| 3 | cell communication | 13 | 1550 | 139 | 10726 | 0.094 | 0.145 | 0.647 | 0.973 | 102280\_at,161232\_r\_at,161787\_f\_at,92593\_at,95292\_at,98140\_at,98993\_at,99475\_at,161134\_at,93459\_s\_at,99439\_at,97411\_at,97909\_at |
| 4 | cell adhesion | 6 | 322 | 161 | 13100 | 0.037 | 0.025 | 1.516 | 0.205 | 102280\_at,161232\_r\_at,161787\_f\_at,92593\_at,95292\_at,98140\_at |
| 5 | cell-cell adhesion | 2 | 44 | 125 | 11544 | 0.016 | 0.004 | 4.199 | 0.082 | 102280\_at,98140\_at |
| 6 | homophilic cell adhesion | 2 | 31 | 108 | 9498 | 0.019 | 0.003 | 5.681 | 0.048 | 102280\_at,98140\_at |
| 5 | cell-matrix adhesion | 2 | 50 | 125 | 11544 | 0.016 | 0.004 | 3.695 | 0.102 | 161787\_f\_at,95292\_at |
| 4 | signal transduction | 9 | 1199 | 161 | 13100 | 0.056 | 0.092 | 0.611 | 0.965 | 98993\_at,99475\_at,161134\_at,93459\_s\_at,99439\_at,161787\_f\_at,95292\_at,97411\_at,97909\_at |
| 5 | cell surface receptor linked signal transduction | 5 | 621 | 125 | 11544 | 0.04 | 0.054 | 0.744 | 0.81 | 161134\_at,93459\_s\_at,99439\_at,161787\_f\_at,95292\_at |
| 6 | G-protein coupled receptor protein signaling pathway | 3 | 355 | 108 | 9498 | 0.028 | 0.037 | 0.743 | 0.774 | 161134\_at,93459\_s\_at,99439\_at |
| 6 | integrin-mediated signaling pathway | 2 | 45 | 108 | 9498 | 0.019 | 0.005 | 3.907 | 0.093 | 161787\_f\_at,95292\_at |
| 5 | intracellular signaling cascade | 3 | 485 | 125 | 11544 | 0.024 | 0.042 | 0.571 | 0.901 | 97411\_at,97909\_at,99475\_at |
| 3 | cell death | 4 | 207 | 139 | 10726 | 0.029 | 0.019 | 1.491 | 0.281 | 103910\_at,93445\_at,96858\_at,93228\_at |
| 4 | programmed cell death | 4 | 192 | 161 | 13100 | 0.025 | 0.015 | 1.694 | 0.211 | 103910\_at,93445\_at,96858\_at,93228\_at |
| 5 | apoptosis | 4 | 192 | 125 | 11544 | 0.032 | 0.017 | 1.924 | 0.155 | 103910\_at,93445\_at,96858\_at,93228\_at |
| 6 | anti-apoptosis | 1 | 31 | 108 | 9498 | 0.009 | 0.003 | 2.84 | 0.299 | 93228\_at |
| 6 | apoptotic program | 1 | 16 | 108 | 9498 | 0.009 | 0.002 | 5.512 | 0.167 | 96858\_at |
| 7 | apoptotic mitochondrial changes | 1 | 4 | 74 | 6246 | 0.014 | 0.001 | 21.109 | 0.047 | 96858\_at |
| 7 | apoptotic nuclear changes | 1 | 3 | 74 | 6246 | 0.014 | 0 | 28.146 | 0.035 | 96858\_at |
| 8 | DNA fragmentation | 1 | 3 | 25 | 2164 | 0.04 | 0.001 | 28.777 | 0.034 | 96858\_at |
| 3 | cell differentiation | 2 | 137 | 139 | 10726 | 0.014 | 0.013 | 1.127 | 0.533 | 93228\_at,99475\_at |
| 4 | lymphocytic blood cell differentiation | 1 | 6 | 161 | 13100 | 0.006 | 0 | 13.5 | 0.072 | 93228\_at |
| 4 | neuron differentiation | 1 | 5 | 161 | 13100 | 0.006 | 0 | 16.342 | 0.06 | 99475\_at |
| 5 | regulation of neuron differentiation | 1 | 1 | 125 | 11544 | 0.008 | 0 | 88.889 | 0.011 | 99475\_at |
| 6 | positive regulation of neuron differentiation | 1 | 1 | 108 | 9498 | 0.009 | 0 | 84.182 | 0.011 | 99475\_at |
| 3 | cell growth and/or maintenance | 38 | 2128 | 139 | 10726 | 0.273 | 0.198 | 1.378 | 0.02 | 97411\_at,98550\_at,99439\_at,99475\_at,99777\_s\_at,93333\_at,94788\_f\_at,95118\_r\_at,97909\_at,96710\_at,99457\_at,100062\_at,101958\_f\_at,103064\_at,103797\_at,103821\_at,160069\_at,160496\_s\_at,161134\_at,92478\_at,92647\_at,95527\_at,97095\_at,101920\_at,103207\_at,92551\_at,95612\_at,162009\_f\_at,94376\_s\_at,103910\_at,96891\_at,100225\_f\_at,102105\_f\_at,102134\_f\_at,161757\_f\_at,160518\_at,98956\_at,95611\_at |
| 4 | cell growth | 1 | 51 | 161 | 13100 | 0.006 | 0.004 | 1.596 | 0.468 | 99475\_at |
| 5 | regulation of cell growth | 1 | 38 | 125 | 11544 | 0.008 | 0.003 | 2.432 | 0.339 | 99475\_at |
| 4 | cell organization and biogenesis | 7 | 530 | 161 | 13100 | 0.043 | 0.04 | 1.075 | 0.478 | 99777\_s\_at,93333\_at,94788\_f\_at,95118\_r\_at,97909\_at,96710\_at,98550\_at |
| 5 | cytoplasm organization and biogenesis | 5 | 380 | 125 | 11544 | 0.04 | 0.033 | 1.215 | 0.394 | 99777\_s\_at,93333\_at,94788\_f\_at,95118\_r\_at,97909\_at |
| 6 | organelle organization and biogenesis | 5 | 318 | 108 | 9498 | 0.046 | 0.033 | 1.383 | 0.295 | 99777\_s\_at,93333\_at,94788\_f\_at,95118\_r\_at,97909\_at |
| 7 | cytoskeleton organization and biogenesis | 5 | 262 | 74 | 6246 | 0.068 | 0.042 | 1.611 | 0.198 | 99777\_s\_at,93333\_at,94788\_f\_at,95118\_r\_at,97909\_at |
| 8 | microtubule-based process | 4 | 119 | 25 | 2164 | 0.16 | 0.055 | 2.91 | 0.045 | 93333\_at,94788\_f\_at,95118\_r\_at,97909\_at |
| 9 | microtubule cytoskeleton organization and biogenesis | 1 | 12 | 14 | 911 | 0.071 | 0.013 | 5.424 | 0.171 | 93333\_at |
| 10 | microtubule nucleation | 1 | 3 | 4 | 197 | 0.25 | 0.015 | 16.415 | 0.06 | 93333\_at |
| 11 | tubulin folding | 1 | 2 | 2 | 34 | 0.5 | 0.059 | 8.501 | 0.116 | 93333\_at |
| 12 | chaperonin-mediated tubulin folding | 1 | 1 | 2 | 8 | 0.5 | 0.125 | 4 | 0.25 | 93333\_at |
| 5 | nuclear organization and biogenesis | 2 | 112 | 125 | 11544 | 0.016 | 0.01 | 1.649 | 0.343 | 96710\_at,98550\_at |
| 6 | chromosome organization and biogenesis (sensu Eukarya) | 2 | 108 | 108 | 9498 | 0.019 | 0.011 | 1.629 | 0.348 | 96710\_at,98550\_at |
| 7 | establishment and/or maintenance of chromatin architecture | 2 | 80 | 74 | 6246 | 0.027 | 0.013 | 2.11 | 0.245 | 96710\_at,98550\_at |
| 8 | chromatin assembly/disassembly | 2 | 48 | 25 | 2164 | 0.08 | 0.022 | 3.607 | 0.105 | 96710\_at,98550\_at |
| 9 | nucleosome assembly | 2 | 28 | 14 | 911 | 0.143 | 0.031 | 4.647 | 0.066 | 96710\_at,98550\_at |
| 4 | cell proliferation | 24 | 501 | 161 | 13100 | 0.149 | 0.038 | 3.898 | 0 | 99457\_at,100062\_at,101958\_f\_at,103064\_at,103797\_at,103821\_at,160069\_at,160496\_s\_at,161134\_at,92478\_at,92647\_at,95527\_at,97095\_at,101920\_at,103207\_at,92551\_at,95612\_at,98550\_at,162009\_f\_at,94376\_s\_at,103910\_at,96891\_at,97411\_at,99439\_at |
| 5 | cell cycle | 24 | 435 | 125 | 11544 | 0.192 | 0.038 | 5.096 | 0 | 100062\_at,101958\_f\_at,103064\_at,103797\_at,103821\_at,160069\_at,160496\_s\_at,161134\_at,92478\_at,92647\_at,95527\_at,97095\_at,101920\_at,103207\_at,92551\_at,95612\_at,98550\_at,162009\_f\_at,94376\_s\_at,99457\_at,103910\_at,96891\_at,97411\_at,99439\_at |
| 6 | DNA replication and chromosome cycle | 11 | 113 | 108 | 9498 | 0.102 | 0.012 | 8.559 | 0 | 92478\_at,100062\_at,101920\_at,103207\_at,160496\_s\_at,92551\_at,92647\_at,95527\_at,95612\_at,98550\_at,162009\_f\_at |
| 7 | chromosome segregation | 1 | 13 | 74 | 6246 | 0.014 | 0.002 | 6.495 | 0.144 | 92478\_at |
| 7 | DNA replication | 10 | 94 | 74 | 6246 | 0.135 | 0.015 | 8.979 | 0 | 100062\_at,101920\_at,103207\_at,160496\_s\_at,92551\_at,92647\_at,95527\_at,95612\_at,98550\_at,162009\_f\_at |
| 8 | DNA dependent DNA replication | 3 | 31 | 25 | 2164 | 0.12 | 0.014 | 8.374 | 0.005 | 100062\_at,160496\_s\_at,162009\_f\_at |
| 9 | DNA replication initiation | 2 | 10 | 14 | 911 | 0.143 | 0.011 | 13.011 | 0.009 | 100062\_at,160496\_s\_at |
| 9 | maintenance of fidelity during DNA dependent DNA replication | 1 | 7 | 14 | 911 | 0.071 | 0.008 | 9.301 | 0.103 | 162009\_f\_at |
| 10 | mismatch repair | 1 | 7 | 4 | 197 | 0.25 | 0.036 | 7.036 | 0.136 | 162009\_f\_at |
| 6 | M phase | 4 | 74 | 108 | 9498 | 0.037 | 0.008 | 4.755 | 0.01 | 92478\_at,97095\_at,94376\_s\_at,99457\_at |
| 7 | M phase of mitotic cell cycle | 2 | 57 | 74 | 6246 | 0.027 | 0.009 | 2.961 | 0.146 | 92478\_at,97095\_at |
| 8 | mitosis | 2 | 57 | 25 | 2164 | 0.08 | 0.026 | 3.037 | 0.139 | 92478\_at,97095\_at |
| 7 | nuclear division | 4 | 73 | 74 | 6246 | 0.054 | 0.012 | 4.624 | 0.011 | 92478\_at,97095\_at,94376\_s\_at,99457\_at |
| 8 | mitosis | 2 | 57 | 25 | 2164 | 0.08 | 0.026 | 3.037 | 0.139 | 92478\_at,97095\_at |
| 8 | meiosis | 2 | 23 | 25 | 2164 | 0.08 | 0.011 | 7.526 | 0.028 | 94376\_s\_at,99457\_at |
| 6 | mitotic cell cycle | 15 | 173 | 108 | 9498 | 0.139 | 0.018 | 7.627 | 0 | 92478\_at,97095\_at,103910\_at,103064\_at,96891\_at,100062\_at,101920\_at,103207\_at,160496\_s\_at,92551\_at,92647\_at,95527\_at,95612\_at,98550\_at,162009\_f\_at |
| 7 | M phase of mitotic cell cycle | 2 | 57 | 74 | 6246 | 0.027 | 0.009 | 2.961 | 0.146 | 92478\_at,97095\_at |
| 8 | mitosis | 2 | 57 | 25 | 2164 | 0.08 | 0.026 | 3.037 | 0.139 | 92478\_at,97095\_at |
| 7 | G1/S transition of mitotic cell cycle | 1 | 12 | 74 | 6246 | 0.014 | 0.002 | 7.036 | 0.133 | 103910\_at |
| 7 | G2/M transition of mitotic cell cycle | 1 | 3 | 74 | 6246 | 0.014 | 0 | 28.146 | 0.035 | 103064\_at |
| 7 | S phase of mitotic cell cycle | 11 | 95 | 74 | 6246 | 0.149 | 0.015 | 9.773 | 0 | 96891\_at,100062\_at,101920\_at,103207\_at,160496\_s\_at,92551\_at,92647\_at,95527\_at,95612\_at,98550\_at,162009\_f\_at |
| 8 | DNA dependent DNA replication | 3 | 31 | 25 | 2164 | 0.12 | 0.014 | 8.374 | 0.005 | 100062\_at,160496\_s\_at,162009\_f\_at |
| 9 | DNA replication initiation | 2 | 10 | 14 | 911 | 0.143 | 0.011 | 13.011 | 0.009 | 100062\_at,160496\_s\_at |
| 9 | maintenance of fidelity during DNA dependent DNA replication | 1 | 7 | 14 | 911 | 0.071 | 0.008 | 9.301 | 0.103 | 162009\_f\_at |
| 10 | mismatch repair | 1 | 7 | 4 | 197 | 0.25 | 0.036 | 7.036 | 0.136 | 162009\_f\_at |
| 7 | DNA replication | 10 | 94 | 74 | 6246 | 0.135 | 0.015 | 8.979 | 0 | 100062\_at,101920\_at,103207\_at,160496\_s\_at,92551\_at,92647\_at,95527\_at,95612\_at,98550\_at,162009\_f\_at |
| 8 | DNA dependent DNA replication | 3 | 31 | 25 | 2164 | 0.12 | 0.014 | 8.374 | 0.005 | 100062\_at,160496\_s\_at,162009\_f\_at |
| 9 | DNA replication initiation | 2 | 10 | 14 | 911 | 0.143 | 0.011 | 13.011 | 0.009 | 100062\_at,160496\_s\_at |
| 9 | maintenance of fidelity during DNA dependent DNA replication | 1 | 7 | 14 | 911 | 0.071 | 0.008 | 9.301 | 0.103 | 162009\_f\_at |
| 10 | mismatch repair | 1 | 7 | 4 | 197 | 0.25 | 0.036 | 7.036 | 0.136 | 162009\_f\_at |
| 6 | regulation of cell cycle | 4 | 204 | 108 | 9498 | 0.037 | 0.021 | 1.724 | 0.202 | 101958\_f\_at,97411\_at,99439\_at,103064\_at |
| 7 | cell cycle checkpoint | 1 | 10 | 74 | 6246 | 0.014 | 0.002 | 8.444 | 0.112 | 103064\_at |
| 8 | DNA damage response, signal transduction resulting in cell cycle arrest | 1 | 7 | 25 | 2164 | 0.04 | 0.003 | 12.384 | 0.078 | 103064\_at |
| 4 | transport | 8 | 1083 | 161 | 13100 | 0.05 | 0.083 | 0.601 | 0.961 | 100225\_f\_at,102105\_f\_at,102134\_f\_at,161757\_f\_at,94376\_s\_at,160518\_at,98956\_at,95611\_at |
| 5 | hydrogen transport | 1 | 50 | 125 | 11544 | 0.008 | 0.004 | 1.848 | 0.42 | 102134\_f\_at |
| 6 | proton transport | 1 | 44 | 108 | 9498 | 0.009 | 0.005 | 2 | 0.396 | 102134\_f\_at |
| 5 | intracellular transport | 3 | 351 | 125 | 11544 | 0.024 | 0.03 | 0.789 | 0.737 | 160518\_at,161757\_f\_at,98956\_at |
| 6 | Golgi vesicle transport | 1 | 12 | 108 | 9498 | 0.009 | 0.001 | 7.349 | 0.128 | 160518\_at |
| 7 | retrograde (Golgi to ER) transport | 1 | 1 | 74 | 6246 | 0.014 | 0 | 84.438 | 0.012 | 160518\_at |
| 6 | intracellular protein transport | 2 | 284 | 108 | 9498 | 0.019 | 0.03 | 0.619 | 0.838 | 161757\_f\_at,98956\_at |
| 7 | protein targeting | 2 | 101 | 74 | 6246 | 0.027 | 0.016 | 1.672 | 0.337 | 98956\_at,161757\_f\_at |
| 8 | protein-nucleus import | 1 | 32 | 25 | 2164 | 0.04 | 0.015 | 2.705 | 0.312 | 161757\_f\_at |
| 9 | NLS-bearing substrate-nucleus import | 1 | 2 | 14 | 911 | 0.071 | 0.002 | 32.468 | 0.031 | 161757\_f\_at |
| 9 | protein-nucleus import, docking | 1 | 10 | 14 | 911 | 0.071 | 0.011 | 6.505 | 0.144 | 161757\_f\_at |
| 5 | ion transport | 1 | 335 | 125 | 11544 | 0.008 | 0.029 | 0.276 | 0.975 | 102134\_f\_at |
| 5 | lipid transport | 1 | 40 | 125 | 11544 | 0.008 | 0.003 | 2.305 | 0.354 | 95611\_at |
| 5 | protein transport | 2 | 297 | 125 | 11544 | 0.016 | 0.026 | 0.622 | 0.836 | 161757\_f\_at,98956\_at |
| 6 | intracellular protein transport | 2 | 284 | 108 | 9498 | 0.019 | 0.03 | 0.619 | 0.838 | 161757\_f\_at,98956\_at |
| 7 | protein targeting | 2 | 101 | 74 | 6246 | 0.027 | 0.016 | 1.672 | 0.337 | 98956\_at,161757\_f\_at |
| 8 | protein-nucleus import | 1 | 32 | 25 | 2164 | 0.04 | 0.015 | 2.705 | 0.312 | 161757\_f\_at |
| 9 | NLS-bearing substrate-nucleus import | 1 | 2 | 14 | 911 | 0.071 | 0.002 | 32.468 | 0.031 | 161757\_f\_at |
| 9 | protein-nucleus import, docking | 1 | 10 | 14 | 911 | 0.071 | 0.011 | 6.505 | 0.144 | 161757\_f\_at |
| 3 | cell motility | 2 | 188 | 139 | 10726 | 0.014 | 0.018 | 0.821 | 0.704 | 161232\_r\_at,95292\_at |
| 4 | cell migration | 2 | 53 | 161 | 13100 | 0.012 | 0.004 | 3.067 | 0.138 | 161232\_r\_at,95292\_at |
| 2 | development | 8 | 990 | 138 | 10540 | 0.058 | 0.094 | 0.617 | 0.954 | 161232\_r\_at,161787\_f\_at,93459\_s\_at,93228\_at,99475\_at,160069\_at,95292\_at,101445\_at |
| 3 | cell differentiation | 2 | 137 | 139 | 10726 | 0.014 | 0.013 | 1.127 | 0.533 | 93228\_at,99475\_at |
| 4 | lymphocytic blood cell differentiation | 1 | 6 | 161 | 13100 | 0.006 | 0 | 13.5 | 0.072 | 93228\_at |
| 4 | neuron differentiation | 1 | 5 | 161 | 13100 | 0.006 | 0 | 16.342 | 0.06 | 99475\_at |
| 5 | regulation of neuron differentiation | 1 | 1 | 125 | 11544 | 0.008 | 0 | 88.889 | 0.011 | 99475\_at |
| 6 | positive regulation of neuron differentiation | 1 | 1 | 108 | 9498 | 0.009 | 0 | 84.182 | 0.011 | 99475\_at |
| 3 | growth | 1 | 8 | 139 | 10726 | 0.007 | 0.001 | 9.587 | 0.099 | 99475\_at |
| 4 | regulation of growth | 1 | 8 | 161 | 13100 | 0.006 | 0.001 | 10.18 | 0.094 | 99475\_at |
| 5 | regulation of body size | 1 | 5 | 125 | 11544 | 0.008 | 0 | 18.605 | 0.053 | 99475\_at |
| 3 | morphogenesis | 4 | 594 | 139 | 10726 | 0.029 | 0.055 | 0.52 | 0.954 | 160069\_at,95292\_at,161232\_r\_at,93228\_at |
| 4 | organogenesis | 4 | 544 | 161 | 13100 | 0.025 | 0.042 | 0.598 | 0.906 | 160069\_at,95292\_at,161232\_r\_at,93228\_at |
| 5 | heart development | 1 | 31 | 125 | 11544 | 0.008 | 0.003 | 2.974 | 0.287 | 95292\_at |
| 5 | neurogenesis | 1 | 164 | 125 | 11544 | 0.008 | 0.014 | 0.563 | 0.834 | 161232\_r\_at |
| 6 | central nervous system development | 1 | 39 | 108 | 9498 | 0.009 | 0.004 | 2.253 | 0.36 | 161232\_r\_at |
| 7 | brain development | 1 | 29 | 74 | 6246 | 0.014 | 0.005 | 2.912 | 0.293 | 161232\_r\_at |
| 5 | urogenital system development | 1 | 11 | 125 | 11544 | 0.008 | 0.001 | 8.421 | 0.113 | 93228\_at |
| 3 | regulation of gene expression, epigenetic | 2 | 28 | 139 | 10726 | 0.014 | 0.003 | 5.513 | 0.051 | 101445\_at,93228\_at |
| 4 | DNA methylation | 2 | 21 | 161 | 13100 | 0.012 | 0.002 | 7.763 | 0.027 | 101445\_at,93228\_at |
| 2 | obsolete biological process | 1 | 3 | 138 | 10540 | 0.007 | 0 | 25.893 | 0.039 | 95732\_at |
| 3 | mRNA splicing | 1 | 54 | 139 | 10726 | 0.007 | 0.005 | 1.429 | 0.506 | 95732\_at |
| 2 | physiological processes | 79 | 5866 | 138 | 10540 | 0.572 | 0.557 | 1.029 | 0.386 | 97411\_at,98550\_at,99439\_at,99475\_at,99777\_s\_at,93333\_at,94788\_f\_at,95118\_r\_at,97909\_at,96710\_at,99457\_at,100062\_at,101958\_f\_at,103064\_at,103797\_at,103821\_at,160069\_at,160496\_s\_at,161134\_at,92478\_at,92647\_at,95527\_at,97095\_at,101920\_at,103207\_at,92551\_at,95612\_at,162009\_f\_at,94376\_s\_at,103910\_at,96891\_at,100225\_f\_at,102105\_f\_at,102134\_f\_at,161757\_f\_at,160518\_at,98956\_at,95611\_at,160595\_at,94534\_at,99658\_f\_at,101001\_at,102103\_f\_at,96916\_at,96629\_at,98922\_at,161492\_i\_at,102047\_at,95462\_at,161038\_at,98618\_at,98999\_at,104313\_at,99513\_at,95063\_at,95456\_r\_at,102848\_f\_at,93439\_f\_at,94078\_at,96858\_at,97492\_at,99631\_f\_at,99660\_f\_at,162327\_f\_at,98072\_r\_at,101589\_at,103944\_at,101957\_f\_at,95732\_at,101004\_f\_at,92782\_at,101445\_at,160377\_at,95131\_f\_at,99111\_at,103201\_at,97393\_at,103203\_f\_at,103204\_r\_at |
| 3 | cell growth and/or maintenance | 38 | 2128 | 139 | 10726 | 0.273 | 0.198 | 1.378 | 0.02 | 97411\_at,98550\_at,99439\_at,99475\_at,99777\_s\_at,93333\_at,94788\_f\_at,95118\_r\_at,97909\_at,96710\_at,99457\_at,100062\_at,101958\_f\_at,103064\_at,103797\_at,103821\_at,160069\_at,160496\_s\_at,161134\_at,92478\_at,92647\_at,95527\_at,97095\_at,101920\_at,103207\_at,92551\_at,95612\_at,162009\_f\_at,94376\_s\_at,103910\_at,96891\_at,100225\_f\_at,102105\_f\_at,102134\_f\_at,161757\_f\_at,160518\_at,98956\_at,95611\_at |
| 4 | cell growth | 1 | 51 | 161 | 13100 | 0.006 | 0.004 | 1.596 | 0.468 | 99475\_at |
| 5 | regulation of cell growth | 1 | 38 | 125 | 11544 | 0.008 | 0.003 | 2.432 | 0.339 | 99475\_at |
| 4 | cell organization and biogenesis | 7 | 530 | 161 | 13100 | 0.043 | 0.04 | 1.075 | 0.478 | 99777\_s\_at,93333\_at,94788\_f\_at,95118\_r\_at,97909\_at,96710\_at,98550\_at |
| 5 | cytoplasm organization and biogenesis | 5 | 380 | 125 | 11544 | 0.04 | 0.033 | 1.215 | 0.394 | 99777\_s\_at,93333\_at,94788\_f\_at,95118\_r\_at,97909\_at |
| 6 | organelle organization and biogenesis | 5 | 318 | 108 | 9498 | 0.046 | 0.033 | 1.383 | 0.295 | 99777\_s\_at,93333\_at,94788\_f\_at,95118\_r\_at,97909\_at |
| 7 | cytoskeleton organization and biogenesis | 5 | 262 | 74 | 6246 | 0.068 | 0.042 | 1.611 | 0.198 | 99777\_s\_at,93333\_at,94788\_f\_at,95118\_r\_at,97909\_at |
| 8 | microtubule-based process | 4 | 119 | 25 | 2164 | 0.16 | 0.055 | 2.91 | 0.045 | 93333\_at,94788\_f\_at,95118\_r\_at,97909\_at |
| 9 | microtubule cytoskeleton organization and biogenesis | 1 | 12 | 14 | 911 | 0.071 | 0.013 | 5.424 | 0.171 | 93333\_at |
| 10 | microtubule nucleation | 1 | 3 | 4 | 197 | 0.25 | 0.015 | 16.415 | 0.06 | 93333\_at |
| 11 | tubulin folding | 1 | 2 | 2 | 34 | 0.5 | 0.059 | 8.501 | 0.116 | 93333\_at |
| 12 | chaperonin-mediated tubulin folding | 1 | 1 | 2 | 8 | 0.5 | 0.125 | 4 | 0.25 | 93333\_at |
| 5 | nuclear organization and biogenesis | 2 | 112 | 125 | 11544 | 0.016 | 0.01 | 1.649 | 0.343 | 96710\_at,98550\_at |
| 6 | chromosome organization and biogenesis (sensu Eukarya) | 2 | 108 | 108 | 9498 | 0.019 | 0.011 | 1.629 | 0.348 | 96710\_at,98550\_at |
| 7 | establishment and/or maintenance of chromatin architecture | 2 | 80 | 74 | 6246 | 0.027 | 0.013 | 2.11 | 0.245 | 96710\_at,98550\_at |
| 8 | chromatin assembly/disassembly | 2 | 48 | 25 | 2164 | 0.08 | 0.022 | 3.607 | 0.105 | 96710\_at,98550\_at |
| 9 | nucleosome assembly | 2 | 28 | 14 | 911 | 0.143 | 0.031 | 4.647 | 0.066 | 96710\_at,98550\_at |
| 4 | cell proliferation | 24 | 501 | 161 | 13100 | 0.149 | 0.038 | 3.898 | 0 | 99457\_at,100062\_at,101958\_f\_at,103064\_at,103797\_at,103821\_at,160069\_at,160496\_s\_at,161134\_at,92478\_at,92647\_at,95527\_at,97095\_at,101920\_at,103207\_at,92551\_at,95612\_at,98550\_at,162009\_f\_at,94376\_s\_at,103910\_at,96891\_at,97411\_at,99439\_at |
| 5 | cell cycle | 24 | 435 | 125 | 11544 | 0.192 | 0.038 | 5.096 | 0 | 100062\_at,101958\_f\_at,103064\_at,103797\_at,103821\_at,160069\_at,160496\_s\_at,161134\_at,92478\_at,92647\_at,95527\_at,97095\_at,101920\_at,103207\_at,92551\_at,95612\_at,98550\_at,162009\_f\_at,94376\_s\_at,99457\_at,103910\_at,96891\_at,97411\_at,99439\_at |
| 6 | DNA replication and chromosome cycle | 11 | 113 | 108 | 9498 | 0.102 | 0.012 | 8.559 | 0 | 92478\_at,100062\_at,101920\_at,103207\_at,160496\_s\_at,92551\_at,92647\_at,95527\_at,95612\_at,98550\_at,162009\_f\_at |
| 7 | chromosome segregation | 1 | 13 | 74 | 6246 | 0.014 | 0.002 | 6.495 | 0.144 | 92478\_at |
| 7 | DNA replication | 10 | 94 | 74 | 6246 | 0.135 | 0.015 | 8.979 | 0 | 100062\_at,101920\_at,103207\_at,160496\_s\_at,92551\_at,92647\_at,95527\_at,95612\_at,98550\_at,162009\_f\_at |
| 8 | DNA dependent DNA replication | 3 | 31 | 25 | 2164 | 0.12 | 0.014 | 8.374 | 0.005 | 100062\_at,160496\_s\_at,162009\_f\_at |
| 9 | DNA replication initiation | 2 | 10 | 14 | 911 | 0.143 | 0.011 | 13.011 | 0.009 | 100062\_at,160496\_s\_at |
| 9 | maintenance of fidelity during DNA dependent DNA replication | 1 | 7 | 14 | 911 | 0.071 | 0.008 | 9.301 | 0.103 | 162009\_f\_at |
| 10 | mismatch repair | 1 | 7 | 4 | 197 | 0.25 | 0.036 | 7.036 | 0.136 | 162009\_f\_at |
| 6 | M phase | 4 | 74 | 108 | 9498 | 0.037 | 0.008 | 4.755 | 0.01 | 92478\_at,97095\_at,94376\_s\_at,99457\_at |
| 7 | M phase of mitotic cell cycle | 2 | 57 | 74 | 6246 | 0.027 | 0.009 | 2.961 | 0.146 | 92478\_at,97095\_at |
| 8 | mitosis | 2 | 57 | 25 | 2164 | 0.08 | 0.026 | 3.037 | 0.139 | 92478\_at,97095\_at |
| 7 | nuclear division | 4 | 73 | 74 | 6246 | 0.054 | 0.012 | 4.624 | 0.011 | 92478\_at,97095\_at,94376\_s\_at,99457\_at |
| 8 | mitosis | 2 | 57 | 25 | 2164 | 0.08 | 0.026 | 3.037 | 0.139 | 92478\_at,97095\_at |
| 8 | meiosis | 2 | 23 | 25 | 2164 | 0.08 | 0.011 | 7.526 | 0.028 | 94376\_s\_at,99457\_at |
| 6 | mitotic cell cycle | 15 | 173 | 108 | 9498 | 0.139 | 0.018 | 7.627 | 0 | 92478\_at,97095\_at,103910\_at,103064\_at,96891\_at,100062\_at,101920\_at,103207\_at,160496\_s\_at,92551\_at,92647\_at,95527\_at,95612\_at,98550\_at,162009\_f\_at |
| 7 | M phase of mitotic cell cycle | 2 | 57 | 74 | 6246 | 0.027 | 0.009 | 2.961 | 0.146 | 92478\_at,97095\_at |
| 8 | mitosis | 2 | 57 | 25 | 2164 | 0.08 | 0.026 | 3.037 | 0.139 | 92478\_at,97095\_at |
| 7 | G1/S transition of mitotic cell cycle | 1 | 12 | 74 | 6246 | 0.014 | 0.002 | 7.036 | 0.133 | 103910\_at |
| 7 | G2/M transition of mitotic cell cycle | 1 | 3 | 74 | 6246 | 0.014 | 0 | 28.146 | 0.035 | 103064\_at |
| 7 | S phase of mitotic cell cycle | 11 | 95 | 74 | 6246 | 0.149 | 0.015 | 9.773 | 0 | 96891\_at,100062\_at,101920\_at,103207\_at,160496\_s\_at,92551\_at,92647\_at,95527\_at,95612\_at,98550\_at,162009\_f\_at |
| 8 | DNA dependent DNA replication | 3 | 31 | 25 | 2164 | 0.12 | 0.014 | 8.374 | 0.005 | 100062\_at,160496\_s\_at,162009\_f\_at |
| 9 | DNA replication initiation | 2 | 10 | 14 | 911 | 0.143 | 0.011 | 13.011 | 0.009 | 100062\_at,160496\_s\_at |
| 9 | maintenance of fidelity during DNA dependent DNA replication | 1 | 7 | 14 | 911 | 0.071 | 0.008 | 9.301 | 0.103 | 162009\_f\_at |
| 10 | mismatch repair | 1 | 7 | 4 | 197 | 0.25 | 0.036 | 7.036 | 0.136 | 162009\_f\_at |
| 7 | DNA replication | 10 | 94 | 74 | 6246 | 0.135 | 0.015 | 8.979 | 0 | 100062\_at,101920\_at,103207\_at,160496\_s\_at,92551\_at,92647\_at,95527\_at,95612\_at,98550\_at,162009\_f\_at |
| 8 | DNA dependent DNA replication | 3 | 31 | 25 | 2164 | 0.12 | 0.014 | 8.374 | 0.005 | 100062\_at,160496\_s\_at,162009\_f\_at |
| 9 | DNA replication initiation | 2 | 10 | 14 | 911 | 0.143 | 0.011 | 13.011 | 0.009 | 100062\_at,160496\_s\_at |
| 9 | maintenance of fidelity during DNA dependent DNA replication | 1 | 7 | 14 | 911 | 0.071 | 0.008 | 9.301 | 0.103 | 162009\_f\_at |
| 10 | mismatch repair | 1 | 7 | 4 | 197 | 0.25 | 0.036 | 7.036 | 0.136 | 162009\_f\_at |
| 6 | regulation of cell cycle | 4 | 204 | 108 | 9498 | 0.037 | 0.021 | 1.724 | 0.202 | 101958\_f\_at,97411\_at,99439\_at,103064\_at |
| 7 | cell cycle checkpoint | 1 | 10 | 74 | 6246 | 0.014 | 0.002 | 8.444 | 0.112 | 103064\_at |
| 8 | DNA damage response, signal transduction resulting in cell cycle arrest | 1 | 7 | 25 | 2164 | 0.04 | 0.003 | 12.384 | 0.078 | 103064\_at |
| 4 | transport | 8 | 1083 | 161 | 13100 | 0.05 | 0.083 | 0.601 | 0.961 | 100225\_f\_at,102105\_f\_at,102134\_f\_at,161757\_f\_at,94376\_s\_at,160518\_at,98956\_at,95611\_at |
| 5 | hydrogen transport | 1 | 50 | 125 | 11544 | 0.008 | 0.004 | 1.848 | 0.42 | 102134\_f\_at |
| 6 | proton transport | 1 | 44 | 108 | 9498 | 0.009 | 0.005 | 2 | 0.396 | 102134\_f\_at |
| 5 | intracellular transport | 3 | 351 | 125 | 11544 | 0.024 | 0.03 | 0.789 | 0.737 | 160518\_at,161757\_f\_at,98956\_at |
| 6 | Golgi vesicle transport | 1 | 12 | 108 | 9498 | 0.009 | 0.001 | 7.349 | 0.128 | 160518\_at |
| 7 | retrograde (Golgi to ER) transport | 1 | 1 | 74 | 6246 | 0.014 | 0 | 84.438 | 0.012 | 160518\_at |
| 6 | intracellular protein transport | 2 | 284 | 108 | 9498 | 0.019 | 0.03 | 0.619 | 0.838 | 161757\_f\_at,98956\_at |
| 7 | protein targeting | 2 | 101 | 74 | 6246 | 0.027 | 0.016 | 1.672 | 0.337 | 98956\_at,161757\_f\_at |
| 8 | protein-nucleus import | 1 | 32 | 25 | 2164 | 0.04 | 0.015 | 2.705 | 0.312 | 161757\_f\_at |
| 9 | NLS-bearing substrate-nucleus import | 1 | 2 | 14 | 911 | 0.071 | 0.002 | 32.468 | 0.031 | 161757\_f\_at |
| 9 | protein-nucleus import, docking | 1 | 10 | 14 | 911 | 0.071 | 0.011 | 6.505 | 0.144 | 161757\_f\_at |
| 5 | ion transport | 1 | 335 | 125 | 11544 | 0.008 | 0.029 | 0.276 | 0.975 | 102134\_f\_at |
| 5 | lipid transport | 1 | 40 | 125 | 11544 | 0.008 | 0.003 | 2.305 | 0.354 | 95611\_at |
| 5 | protein transport | 2 | 297 | 125 | 11544 | 0.016 | 0.026 | 0.622 | 0.836 | 161757\_f\_at,98956\_at |
| 6 | intracellular protein transport | 2 | 284 | 108 | 9498 | 0.019 | 0.03 | 0.619 | 0.838 | 161757\_f\_at,98956\_at |
| 7 | protein targeting | 2 | 101 | 74 | 6246 | 0.027 | 0.016 | 1.672 | 0.337 | 98956\_at,161757\_f\_at |
| 8 | protein-nucleus import | 1 | 32 | 25 | 2164 | 0.04 | 0.015 | 2.705 | 0.312 | 161757\_f\_at |
| 9 | NLS-bearing substrate-nucleus import | 1 | 2 | 14 | 911 | 0.071 | 0.002 | 32.468 | 0.031 | 161757\_f\_at |
| 9 | protein-nucleus import, docking | 1 | 10 | 14 | 911 | 0.071 | 0.011 | 6.505 | 0.144 | 161757\_f\_at |
| 3 | metabolism | 60 | 3908 | 139 | 10726 | 0.432 | 0.364 | 1.185 | 0.059 | 160595\_at,94534\_at,99658\_f\_at,101001\_at,102105\_f\_at,102103\_f\_at,96916\_at,96629\_at,98922\_at,161492\_i\_at,102047\_at,95462\_at,161038\_at,98618\_at,98999\_at,104313\_at,95611\_at,99513\_at,100225\_f\_at,95063\_at,95456\_r\_at,102848\_f\_at,93439\_f\_at,94078\_at,96858\_at,97492\_at,99631\_f\_at,99660\_f\_at,162327\_f\_at,98072\_r\_at,94376\_s\_at,100062\_at,101920\_at,103207\_at,160496\_s\_at,92551\_at,92647\_at,95527\_at,95612\_at,98550\_at,162009\_f\_at,101589\_at,96710\_at,103944\_at,101957\_f\_at,95732\_at,101004\_f\_at,92782\_at,101445\_at,101958\_f\_at,160377\_at,95131\_f\_at,99111\_at,103064\_at,103201\_at,103797\_at,97095\_at,97393\_at,98956\_at,161757\_f\_at |
| 4 | amine metabolism | 1 | 148 | 161 | 13100 | 0.006 | 0.011 | 0.55 | 0.841 | 101001\_at |
| 5 | biogenic amine metabolism | 1 | 35 | 125 | 11544 | 0.008 | 0.003 | 2.64 | 0.317 | 101001\_at |
| 6 | polyamine metabolism | 1 | 13 | 108 | 9498 | 0.009 | 0.001 | 6.759 | 0.138 | 101001\_at |
| 4 | biosynthesis | 11 | 652 | 161 | 13100 | 0.068 | 0.05 | 1.373 | 0.179 | 102105\_f\_at,102103\_f\_at,96916\_at,96629\_at,98922\_at,161492\_i\_at,102047\_at,95462\_at,161038\_at,98618\_at,98999\_at |
| 5 | lipid biosynthesis | 1 | 124 | 125 | 11544 | 0.008 | 0.011 | 0.745 | 0.743 | 102105\_f\_at |
| 6 | fatty acid biosynthesis | 1 | 40 | 108 | 9498 | 0.009 | 0.004 | 2.2 | 0.368 | 102105\_f\_at |
| 7 | eicosanoid biosynthesis | 1 | 19 | 74 | 6246 | 0.014 | 0.003 | 4.444 | 0.203 | 102105\_f\_at |
| 8 | prostanoid biosynthesis | 1 | 9 | 25 | 2164 | 0.04 | 0.004 | 9.615 | 0.099 | 102105\_f\_at |
| 9 | prostaglandin biosynthesis | 1 | 9 | 14 | 911 | 0.071 | 0.01 | 7.23 | 0.131 | 102105\_f\_at |
| 5 | macromolecule biosynthesis | 7 | 322 | 125 | 11544 | 0.056 | 0.028 | 2.008 | 0.061 | 102103\_f\_at,96916\_at,96629\_at,98922\_at,161492\_i\_at,102047\_at,95462\_at |
| 6 | protein biosynthesis | 7 | 322 | 108 | 9498 | 0.065 | 0.034 | 1.912 | 0.074 | 102103\_f\_at,96916\_at,96629\_at,98922\_at,161492\_i\_at,102047\_at,95462\_at |
| 7 | amino acid activation | 1 | 36 | 74 | 6246 | 0.014 | 0.006 | 2.345 | 0.35 | 96629\_at |
| 7 | glycoprotein biosynthesis | 2 | 52 | 74 | 6246 | 0.027 | 0.008 | 3.245 | 0.126 | 98922\_at,161492\_i\_at |
| 8 | protein amino acid glycosylation | 2 | 52 | 25 | 2164 | 0.08 | 0.024 | 3.329 | 0.12 | 98922\_at,161492\_i\_at |
| 9 | N-linked glycosylation | 1 | 12 | 14 | 911 | 0.071 | 0.013 | 5.424 | 0.171 | 161492\_i\_at |
| 7 | lipoprotein biosynthesis | 1 | 16 | 74 | 6246 | 0.014 | 0.003 | 5.277 | 0.174 | 102047\_at |
| 8 | protein lipidation | 1 | 16 | 25 | 2164 | 0.04 | 0.007 | 5.413 | 0.17 | 102047\_at |
| 9 | protein myristoylation | 1 | 3 | 14 | 911 | 0.071 | 0.003 | 21.711 | 0.045 | 102047\_at |
| 10 | protein amino acid myristoylation | 1 | 3 | 4 | 197 | 0.25 | 0.015 | 16.415 | 0.06 | 102047\_at |
| 11 | N-terminal protein myristoylation | 1 | 3 | 2 | 34 | 0.5 | 0.088 | 5.666 | 0.171 | 102047\_at |
| 12 | N-terminal peptidyl-glycine N-myristoylation | 1 | 3 | 2 | 8 | 0.5 | 0.375 | 1.333 | 0.643 | 102047\_at |
| 7 | regulation of translation | 1 | 24 | 74 | 6246 | 0.014 | 0.004 | 3.518 | 0.249 | 95462\_at |
| 8 | regulation of translational initiation | 1 | 13 | 25 | 2164 | 0.04 | 0.006 | 6.656 | 0.141 | 95462\_at |
| 5 | nucleotide biosynthesis | 3 | 74 | 125 | 11544 | 0.024 | 0.006 | 3.744 | 0.046 | 161038\_at,98618\_at,98999\_at |
| 6 | deoxyribonucleotide biosynthesis | 1 | 7 | 108 | 9498 | 0.009 | 0.001 | 12.514 | 0.077 | 98618\_at |
| 7 | deoxyribonucleoside diphosphate biosynthesis | 1 | 4 | 74 | 6246 | 0.014 | 0.001 | 21.109 | 0.047 | 98618\_at |
| 8 | pyrimidine deoxyribonucleoside diphosphate biosynthesis | 1 | 4 | 25 | 2164 | 0.04 | 0.002 | 21.622 | 0.045 | 98618\_at |
| 9 | dTDP biosynthesis | 1 | 4 | 14 | 911 | 0.071 | 0.004 | 16.271 | 0.06 | 98618\_at |
| 7 | deoxyribonucleoside triphosphate biosynthesis | 1 | 5 | 74 | 6246 | 0.014 | 0.001 | 16.887 | 0.058 | 98618\_at |
| 8 | pyrimidine deoxyribonucleoside triphosphate biosynthesis | 1 | 5 | 25 | 2164 | 0.04 | 0.002 | 17.316 | 0.056 | 98618\_at |
| 9 | dTTP biosynthesis | 1 | 5 | 14 | 911 | 0.071 | 0.005 | 13.011 | 0.075 | 98618\_at |
| 6 | nucleoside monophosphate biosynthesis | 1 | 15 | 108 | 9498 | 0.009 | 0.002 | 5.861 | 0.158 | 161038\_at |
| 7 | ribonucleoside monophosphate biosynthesis | 1 | 13 | 74 | 6246 | 0.014 | 0.002 | 6.495 | 0.144 | 161038\_at |
| 6 | purine nucleotide biosynthesis | 1 | 45 | 108 | 9498 | 0.009 | 0.005 | 1.954 | 0.403 | 98999\_at |
| 7 | purine ribonucleotide biosynthesis | 1 | 42 | 74 | 6246 | 0.014 | 0.007 | 2.01 | 0.395 | 98999\_at |
| 4 | carbohydrate metabolism | 2 | 231 | 161 | 13100 | 0.012 | 0.018 | 0.704 | 0.78 | 104313\_at,94534\_at |
| 5 | main pathways of carbohydrate metabolism | 1 | 84 | 125 | 11544 | 0.008 | 0.007 | 1.099 | 0.601 | 94534\_at |
| 6 | tricarboxylic acid cycle | 1 | 19 | 108 | 9498 | 0.009 | 0.002 | 4.63 | 0.195 | 94534\_at |
| 4 | catabolism | 5 | 631 | 161 | 13100 | 0.031 | 0.048 | 0.645 | 0.893 | 95611\_at,99513\_at,100225\_f\_at,95063\_at,95456\_r\_at |
| 5 | lipid catabolism | 2 | 44 | 125 | 11544 | 0.016 | 0.004 | 4.199 | 0.082 | 95611\_at,99513\_at |
| 6 | membrane lipid catabolism | 1 | 4 | 108 | 9498 | 0.009 | 0 | 22.048 | 0.045 | 99513\_at |
| 7 | phospholipid catabolism | 1 | 2 | 74 | 6246 | 0.014 | 0 | 42.219 | 0.024 | 99513\_at |
| 5 | macromolecule catabolism | 4 | 470 | 125 | 11544 | 0.032 | 0.041 | 0.786 | 0.755 | 100225\_f\_at,95063\_at,95456\_r\_at,99513\_at |
| 6 | protein catabolism | 4 | 466 | 108 | 9498 | 0.037 | 0.049 | 0.755 | 0.783 | 100225\_f\_at,95063\_at,95456\_r\_at,99513\_at |
| 7 | proteolysis and peptidolysis | 2 | 457 | 74 | 6246 | 0.027 | 0.073 | 0.369 | 0.976 | 95456\_r\_at,99513\_at |
| 4 | electron transport | 8 | 313 | 161 | 13100 | 0.05 | 0.024 | 2.08 | 0.04 | 102848\_f\_at,93439\_f\_at,94078\_at,96858\_at,97492\_at,99631\_f\_at,99660\_f\_at,162327\_f\_at |
| 5 | ATP synthesis coupled electron transport | 1 | 7 | 125 | 11544 | 0.008 | 0.001 | 13.115 | 0.073 | 162327\_f\_at |
| 6 | ATP synthesis coupled electron transport (sensu Eukarya) | 1 | 7 | 108 | 9498 | 0.009 | 0.001 | 12.514 | 0.077 | 162327\_f\_at |
| 7 | mitochondrial electron transport, NADH to ubiquinone | 1 | 6 | 74 | 6246 | 0.014 | 0.001 | 14.073 | 0.069 | 162327\_f\_at |
| 4 | lipid metabolism | 3 | 285 | 161 | 13100 | 0.019 | 0.022 | 0.856 | 0.684 | 95611\_at,102105\_f\_at,99513\_at |
| 5 | lipid biosynthesis | 1 | 124 | 125 | 11544 | 0.008 | 0.011 | 0.745 | 0.743 | 102105\_f\_at |
| 6 | fatty acid biosynthesis | 1 | 40 | 108 | 9498 | 0.009 | 0.004 | 2.2 | 0.368 | 102105\_f\_at |
| 7 | eicosanoid biosynthesis | 1 | 19 | 74 | 6246 | 0.014 | 0.003 | 4.444 | 0.203 | 102105\_f\_at |
| 8 | prostanoid biosynthesis | 1 | 9 | 25 | 2164 | 0.04 | 0.004 | 9.615 | 0.099 | 102105\_f\_at |
| 9 | prostaglandin biosynthesis | 1 | 9 | 14 | 911 | 0.071 | 0.01 | 7.23 | 0.131 | 102105\_f\_at |
| 5 | lipid catabolism | 2 | 44 | 125 | 11544 | 0.016 | 0.004 | 4.199 | 0.082 | 95611\_at,99513\_at |
| 6 | membrane lipid catabolism | 1 | 4 | 108 | 9498 | 0.009 | 0 | 22.048 | 0.045 | 99513\_at |
| 7 | phospholipid catabolism | 1 | 2 | 74 | 6246 | 0.014 | 0 | 42.219 | 0.024 | 99513\_at |
| 5 | fatty acid metabolism | 1 | 85 | 125 | 11544 | 0.008 | 0.007 | 1.087 | 0.605 | 102105\_f\_at |
| 6 | fatty acid biosynthesis | 1 | 40 | 108 | 9498 | 0.009 | 0.004 | 2.2 | 0.368 | 102105\_f\_at |
| 7 | eicosanoid biosynthesis | 1 | 19 | 74 | 6246 | 0.014 | 0.003 | 4.444 | 0.203 | 102105\_f\_at |
| 8 | prostanoid biosynthesis | 1 | 9 | 25 | 2164 | 0.04 | 0.004 | 9.615 | 0.099 | 102105\_f\_at |
| 9 | prostaglandin biosynthesis | 1 | 9 | 14 | 911 | 0.071 | 0.01 | 7.23 | 0.131 | 102105\_f\_at |
| 6 | eicosanoid metabolism | 1 | 23 | 108 | 9498 | 0.009 | 0.002 | 3.826 | 0.232 | 102105\_f\_at |
| 7 | eicosanoid biosynthesis | 1 | 19 | 74 | 6246 | 0.014 | 0.003 | 4.444 | 0.203 | 102105\_f\_at |
| 8 | prostanoid biosynthesis | 1 | 9 | 25 | 2164 | 0.04 | 0.004 | 9.615 | 0.099 | 102105\_f\_at |
| 9 | prostaglandin biosynthesis | 1 | 9 | 14 | 911 | 0.071 | 0.01 | 7.23 | 0.131 | 102105\_f\_at |
| 7 | prostanoid metabolism | 1 | 13 | 74 | 6246 | 0.014 | 0.002 | 6.495 | 0.144 | 102105\_f\_at |
| 8 | prostanoid biosynthesis | 1 | 9 | 25 | 2164 | 0.04 | 0.004 | 9.615 | 0.099 | 102105\_f\_at |
| 9 | prostaglandin biosynthesis | 1 | 9 | 14 | 911 | 0.071 | 0.01 | 7.23 | 0.131 | 102105\_f\_at |
| 8 | prostaglandin metabolism | 1 | 13 | 25 | 2164 | 0.04 | 0.006 | 6.656 | 0.141 | 102105\_f\_at |
| 9 | prostaglandin biosynthesis | 1 | 9 | 14 | 911 | 0.071 | 0.01 | 7.23 | 0.131 | 102105\_f\_at |
| 4 | nucleobase, nucleoside, nucleotide and nucleic acid metabolism | 25 | 1530 | 161 | 13100 | 0.155 | 0.117 | 1.33 | 0.084 | 98072\_r\_at,94376\_s\_at,100062\_at,101920\_at,103207\_at,160496\_s\_at,92551\_at,92647\_at,95527\_at,95612\_at,98550\_at,162009\_f\_at,101589\_at,96710\_at,103944\_at,101957\_f\_at,161038\_at,95732\_at,101004\_f\_at,92782\_at,101445\_at,101958\_f\_at,160377\_at,95131\_f\_at,99111\_at |
| 5 | DNA metabolism | 15 | 302 | 125 | 11544 | 0.12 | 0.026 | 4.587 | 0 | 94376\_s\_at,100062\_at,101920\_at,103207\_at,160496\_s\_at,92551\_at,92647\_at,95527\_at,95612\_at,98550\_at,162009\_f\_at,101589\_at,96710\_at,103944\_at,101957\_f\_at |
| 6 | DNA packaging | 3 | 93 | 108 | 9498 | 0.028 | 0.01 | 2.838 | 0.089 | 101589\_at,96710\_at,98550\_at |
| 7 | establishment and/or maintenance of chromatin architecture | 2 | 80 | 74 | 6246 | 0.027 | 0.013 | 2.11 | 0.245 | 96710\_at,98550\_at |
| 8 | chromatin assembly/disassembly | 2 | 48 | 25 | 2164 | 0.08 | 0.022 | 3.607 | 0.105 | 96710\_at,98550\_at |
| 9 | nucleosome assembly | 2 | 28 | 14 | 911 | 0.143 | 0.031 | 4.647 | 0.066 | 96710\_at,98550\_at |
| 6 | DNA recombination | 2 | 25 | 108 | 9498 | 0.019 | 0.003 | 7.042 | 0.032 | 103944\_at,92551\_at |
| 6 | DNA repair | 8 | 99 | 108 | 9498 | 0.074 | 0.01 | 7.108 | 0 | 101957\_f\_at,103944\_at,162009\_f\_at,92551\_at,92647\_at,94376\_s\_at,95527\_at,95612\_at |
| 7 | base-excision repair | 1 | 8 | 74 | 6246 | 0.014 | 0.001 | 10.555 | 0.091 | 101957\_f\_at |
| 5 | nucleoside metabolism | 1 | 15 | 125 | 11544 | 0.008 | 0.001 | 6.154 | 0.151 | 161038\_at |
| 5 | RNA metabolism | 2 | 132 | 125 | 11544 | 0.016 | 0.011 | 1.4 | 0.42 | 95732\_at,101004\_f\_at |
| 6 | RNA processing | 2 | 126 | 108 | 9498 | 0.019 | 0.013 | 1.396 | 0.421 | 95732\_at,101004\_f\_at |
| 7 | mRNA processing | 2 | 84 | 74 | 6246 | 0.027 | 0.013 | 2.01 | 0.262 | 95732\_at,101004\_f\_at |
| 8 | nuclear mRNA splicing, via spliceosome | 1 | NA | 25 | 2164 | 0.04 | NA | NA | NA | 101004\_f\_at |
| 9 | spliceosome assembly | 1 | 19 | 14 | 911 | 0.071 | 0.021 | 3.424 | 0.257 | 101004\_f\_at |
| 10 | mRNA splice site selection | 1 | 16 | 4 | 197 | 0.25 | 0.081 | 3.078 | 0.289 | 101004\_f\_at |
| 5 | transcription | 8 | 1086 | 125 | 11544 | 0.064 | 0.094 | 0.68 | 0.912 | 92782\_at,100062\_at,101445\_at,101958\_f\_at,160377\_at,160496\_s\_at,95131\_f\_at,99111\_at |
| 6 | regulation of transcription | 8 | 1026 | 108 | 9498 | 0.074 | 0.108 | 0.686 | 0.909 | 92782\_at,100062\_at,101445\_at,101958\_f\_at,160377\_at,160496\_s\_at,95131\_f\_at,99111\_at |
| 7 | regulation of transcription, DNA-dependent | 7 | 1013 | 74 | 6246 | 0.095 | 0.162 | 0.583 | 0.967 | 100062\_at,101445\_at,101958\_f\_at,160377\_at,160496\_s\_at,95131\_f\_at,99111\_at |
| 4 | phosphorus metabolism | 6 | 488 | 161 | 13100 | 0.037 | 0.037 | 1.001 | 0.558 | 162327\_f\_at,103064\_at,103201\_at,103797\_at,97095\_at,97393\_at |
| 5 | phosphate metabolism | 6 | 488 | 125 | 11544 | 0.048 | 0.042 | 1.136 | 0.435 | 162327\_f\_at,103064\_at,103201\_at,103797\_at,97095\_at,97393\_at |
| 6 | phosphorylation | 6 | 395 | 108 | 9498 | 0.056 | 0.042 | 1.336 | 0.293 | 162327\_f\_at,103064\_at,103201\_at,103797\_at,97095\_at,97393\_at |
| 7 | mitochondrial electron transport, NADH to ubiquinone | 1 | 6 | 74 | 6246 | 0.014 | 0.001 | 14.073 | 0.069 | 162327\_f\_at |
| 7 | protein amino acid phosphorylation | 5 | 379 | 74 | 6246 | 0.068 | 0.061 | 1.114 | 0.469 | 103064\_at,103201\_at,103797\_at,97095\_at,97393\_at |
| 6 | ATP synthesis coupled electron transport (sensu Eukarya) | 1 | 7 | 108 | 9498 | 0.009 | 0.001 | 12.514 | 0.077 | 162327\_f\_at |
| 7 | mitochondrial electron transport, NADH to ubiquinone | 1 | 6 | 74 | 6246 | 0.014 | 0.001 | 14.073 | 0.069 | 162327\_f\_at |
| 5 | ATP synthesis coupled electron transport | 1 | 7 | 125 | 11544 | 0.008 | 0.001 | 13.115 | 0.073 | 162327\_f\_at |
| 6 | ATP synthesis coupled electron transport (sensu Eukarya) | 1 | 7 | 108 | 9498 | 0.009 | 0.001 | 12.514 | 0.077 | 162327\_f\_at |
| 7 | mitochondrial electron transport, NADH to ubiquinone | 1 | 6 | 74 | 6246 | 0.014 | 0.001 | 14.073 | 0.069 | 162327\_f\_at |
| 4 | protein metabolism | 19 | 1458 | 161 | 13100 | 0.118 | 0.111 | 1.06 | 0.429 | 98956\_at,161757\_f\_at,102103\_f\_at,96916\_at,96629\_at,98922\_at,161492\_i\_at,102047\_at,95462\_at,100225\_f\_at,95063\_at,95456\_r\_at,99513\_at,103064\_at,103201\_at,103797\_at,97095\_at,97393\_at,101957\_f\_at |
| 5 | protein modification | 9 | 654 | 125 | 11544 | 0.072 | 0.057 | 1.271 | 0.277 | 98922\_at,161492\_i\_at,102047\_at,103064\_at,103201\_at,103797\_at,97095\_at,97393\_at,101957\_f\_at |
| 6 | protein amino acid ADP-ribosylation | 1 | 12 | 108 | 9498 | 0.009 | 0.001 | 7.349 | 0.128 | 101957\_f\_at |
| 3 | response to endogenous stimulus | 9 | 119 | 139 | 10726 | 0.065 | 0.011 | 5.839 | 0 | 103064\_at,101957\_f\_at,103944\_at,162009\_f\_at,92551\_at,92647\_at,94376\_s\_at,95527\_at,95612\_at |
| 4 | response to DNA damage stimulus | 9 | 119 | 161 | 13100 | 0.056 | 0.009 | 6.156 | 0 | 103064\_at,101957\_f\_at,103944\_at,162009\_f\_at,92551\_at,92647\_at,94376\_s\_at,95527\_at,95612\_at |
| 3 | response to external stimulus | 2 | 666 | 139 | 10726 | 0.014 | 0.062 | 0.232 | 0.999 | 103203\_f\_at,103204\_r\_at |
| 4 | response to biotic stimulus | 2 | 516 | 161 | 13100 | 0.012 | 0.039 | 0.315 | 0.989 | 103203\_f\_at,103204\_r\_at |
| 5 | defense response | 2 | 471 | 125 | 11544 | 0.016 | 0.041 | 0.392 | 0.966 | 103203\_f\_at,103204\_r\_at |
| 6 | immune response | 2 | 362 | 108 | 9498 | 0.019 | 0.038 | 0.486 | 0.922 | 103203\_f\_at,103204\_r\_at |

  
