# Supplementary material for: Molecular Signatures of Proliferation and Quiescence in Hematopoietic Stem Cells
Source: PLoS Biol. 2004 Sep 28;2(10):e301. doi: 10.1371/journal.pbio.0020301 (PMC520599; doi:10.1371/journal.pbio.0020301)
Supplement: Table S41 — (32 KB HTML). [file pbio.0020301.st041.html]

   Significant Tom Day 3   

# Significant Tom Day 3

|  |  |  |  |  |  |  |  |  |  |  |  |
| --- | --- | --- | --- | --- | --- | --- | --- | --- | --- | --- | --- |
| GOLevel | GOTerm | ProbeCount | ArrayCount | ListGOLevelCount | ArrayGoLevelCount | ListFq | ArrayFq | FoldChange | H-Pvalue | ProbeIds | GeneNames |
| 6 | homophilic cell adhesion | 2 | 31 | 108 | 9498 | 0.019 | 0.003 | 5.681 | 0.048 | 102280\_at,98140\_at | protocadherin 7,cadherin 1 |
| 7 | apoptotic mitochondrial changes | 1 | 4 | 74 | 6246 | 0.014 | 0.001 | 21.109 | 0.047 | 96858\_at | programmed cell death 8 |
| 7 | apoptotic nuclear changes | 1 | 3 | 74 | 6246 | 0.014 | 0 | 28.146 | 0.035 | 96858\_at | programmed cell death 8 |
| 8 | DNA fragmentation | 1 | 3 | 25 | 2164 | 0.04 | 0.001 | 28.777 | 0.034 | 96858\_at | programmed cell death 8 |
| 5 | regulation of neuron differentiation | 1 | 1 | 125 | 11544 | 0.008 | 0 | 88.889 | 0.011 | 99475\_at | suppressor of cytokine signaling 2 |
| 6 | positive regulation of neuron differentiation | 1 | 1 | 108 | 9498 | 0.009 | 0 | 84.182 | 0.011 | 99475\_at | suppressor of cytokine signaling 2 |
| 3 | cell growth and/or maintenance | 38 | 2128 | 139 | 10726 | 0.273 | 0.198 | 1.378 | 0.02 | 97411\_at,98550\_at,99439\_at,99475\_at,99777\_s\_at,93333\_at,94788\_f\_at,95118\_r\_at,97909\_at,96710\_at,99457\_at,100062\_at,101958\_f\_at,103064\_at,103797\_at,103821\_at,160069\_at,160496\_s\_at,161134\_at,92478\_at,92647\_at,95527\_at,97095\_at,101920\_at,103207\_at,92551\_at,95612\_at,162009\_f\_at,94376\_s\_at,103910\_at,96891\_at,100225\_f\_at,102105\_f\_at,102134\_f\_at,161757\_f\_at,160518\_at,98956\_at,95611\_at | ect2 oncogene,SET translocation,MAS1 oncogene,suppressor of cytokine signaling 2,RIKEN cDNA 6230416J20 gene,tubulin cofactor a,tubulin, beta 5,kinesin family member 22, pseudogene,stathmin 1,histone H2A.F/Z variant,antigen identified by monoclonal antibody Ki 67,minichromosome maintenance deficient 3 (S. cerevisiae),transcription factor Dp 1,checkpoint kinase 1 homolog (S. pombe),cell division cycle 71 homolog (S. cerevisiae)-like 1,cell division cycle 6 homolog (S. cerevisiae),geminin,minichromosome maintenance deficient 3 (S. cerevisiae),RIKEN cDNA 2610201A13 gene,stromal antigen 1,retinoblastoma binding protein 4,chromatin assembly factor 1, subunit A (p150),budding uninhibited by benzimidazoles 1 homolog (S. cerevisiae),polymerase (DNA directed), epsilon 2 (p59 subunit),polymerase (DNA directed), alpha 1,ligase I, DNA, ATP-dependent,replication factor C (activator 1) 5,mutS homolog 2 (E. coli),meiotic recombination 11 homolog A (S. cerevisiae),TAF10 RNA polymerase II, TATA box binding protein (TBP)-associated factor,acidic nuclear phosphoprotein 32 family, member B,proteasome (prosome, macropain) 26S subunit, ATPase 3,prostaglandin D2 synthase (brain),ATP synthase, H+ transporting, mitochondrial F0 complex, subunit c (subunit 9), isoform 2,karyopherin (importin) beta 3,RIKEN cDNA 1110060F11 gene,translocating chain-associating membrane protein 1,lipoprotein lipase |
| 8 | microtubule-based process | 4 | 119 | 25 | 2164 | 0.16 | 0.055 | 2.91 | 0.045 | 93333\_at,94788\_f\_at,95118\_r\_at,97909\_at | tubulin cofactor a,tubulin, beta 5,kinesin family member 22, pseudogene,stathmin 1 |
| 4 | cell proliferation | 24 | 501 | 161 | 13100 | 0.149 | 0.038 | 3.898 | 0 | 99457\_at,100062\_at,101958\_f\_at,103064\_at,103797\_at,103821\_at,160069\_at,160496\_s\_at,161134\_at,92478\_at,92647\_at,95527\_at,97095\_at,101920\_at,103207\_at,92551\_at,95612\_at,98550\_at,162009\_f\_at,94376\_s\_at,103910\_at,96891\_at,97411\_at,99439\_at | antigen identified by monoclonal antibody Ki 67,minichromosome maintenance deficient 3 (S. cerevisiae),transcription factor Dp 1,checkpoint kinase 1 homolog (S. pombe),cell division cycle 71 homolog (S. cerevisiae)-like 1,cell division cycle 6 homolog (S. cerevisiae),geminin,minichromosome maintenance deficient 3 (S. cerevisiae),RIKEN cDNA 2610201A13 gene,stromal antigen 1,retinoblastoma binding protein 4,chromatin assembly factor 1, subunit A (p150),budding uninhibited by benzimidazoles 1 homolog (S. cerevisiae),polymerase (DNA directed), epsilon 2 (p59 subunit),polymerase (DNA directed), alpha 1,ligase I, DNA, ATP-dependent,replication factor C (activator 1) 5,SET translocation,mutS homolog 2 (E. coli),meiotic recombination 11 homolog A (S. cerevisiae),TAF10 RNA polymerase II, TATA box binding protein (TBP)-associated factor,acidic nuclear phosphoprotein 32 family, member B,ect2 oncogene,MAS1 oncogene |
| 5 | cell cycle | 24 | 435 | 125 | 11544 | 0.192 | 0.038 | 5.096 | 0 | 100062\_at,101958\_f\_at,103064\_at,103797\_at,103821\_at,160069\_at,160496\_s\_at,161134\_at,92478\_at,92647\_at,95527\_at,97095\_at,101920\_at,103207\_at,92551\_at,95612\_at,98550\_at,162009\_f\_at,94376\_s\_at,99457\_at,103910\_at,96891\_at,97411\_at,99439\_at | minichromosome maintenance deficient 3 (S. cerevisiae),transcription factor Dp 1,checkpoint kinase 1 homolog (S. pombe),cell division cycle 71 homolog (S. cerevisiae)-like 1,cell division cycle 6 homolog (S. cerevisiae),geminin,minichromosome maintenance deficient 3 (S. cerevisiae),RIKEN cDNA 2610201A13 gene,stromal antigen 1,retinoblastoma binding protein 4,chromatin assembly factor 1, subunit A (p150),budding uninhibited by benzimidazoles 1 homolog (S. cerevisiae),polymerase (DNA directed), epsilon 2 (p59 subunit),polymerase (DNA directed), alpha 1,ligase I, DNA, ATP-dependent,replication factor C (activator 1) 5,SET translocation,mutS homolog 2 (E. coli),meiotic recombination 11 homolog A (S. cerevisiae),antigen identified by monoclonal antibody Ki 67,TAF10 RNA polymerase II, TATA box binding protein (TBP)-associated factor,acidic nuclear phosphoprotein 32 family, member B,ect2 oncogene,MAS1 oncogene |
| 6 | DNA replication and chromosome cycle | 11 | 113 | 108 | 9498 | 0.102 | 0.012 | 8.559 | 0 | 92478\_at,100062\_at,101920\_at,103207\_at,160496\_s\_at,92551\_at,92647\_at,95527\_at,95612\_at,98550\_at,162009\_f\_at | stromal antigen 1,minichromosome maintenance deficient 3 (S. cerevisiae),polymerase (DNA directed), epsilon 2 (p59 subunit),polymerase (DNA directed), alpha 1,minichromosome maintenance deficient 3 (S. cerevisiae),ligase I, DNA, ATP-dependent,retinoblastoma binding protein 4,chromatin assembly factor 1, subunit A (p150),replication factor C (activator 1) 5,SET translocation,mutS homolog 2 (E. coli) |
| 7 | DNA replication | 10 | 94 | 74 | 6246 | 0.135 | 0.015 | 8.979 | 0 | 100062\_at,101920\_at,103207\_at,160496\_s\_at,92551\_at,92647\_at,95527\_at,95612\_at,98550\_at,162009\_f\_at | minichromosome maintenance deficient 3 (S. cerevisiae),polymerase (DNA directed), epsilon 2 (p59 subunit),polymerase (DNA directed), alpha 1,minichromosome maintenance deficient 3 (S. cerevisiae),ligase I, DNA, ATP-dependent,retinoblastoma binding protein 4,chromatin assembly factor 1, subunit A (p150),replication factor C (activator 1) 5,SET translocation,mutS homolog 2 (E. coli) |
| 8 | DNA dependent DNA replication | 3 | 31 | 25 | 2164 | 0.12 | 0.014 | 8.374 | 0.005 | 100062\_at,160496\_s\_at,162009\_f\_at | minichromosome maintenance deficient 3 (S. cerevisiae),minichromosome maintenance deficient 3 (S. cerevisiae),mutS homolog 2 (E. coli) |
| 9 | DNA replication initiation | 2 | 10 | 14 | 911 | 0.143 | 0.011 | 13.011 | 0.009 | 100062\_at,160496\_s\_at | minichromosome maintenance deficient 3 (S. cerevisiae),minichromosome maintenance deficient 3 (S. cerevisiae) |
| 6 | M phase | 4 | 74 | 108 | 9498 | 0.037 | 0.008 | 4.755 | 0.01 | 92478\_at,97095\_at,94376\_s\_at,99457\_at | stromal antigen 1,budding uninhibited by benzimidazoles 1 homolog (S. cerevisiae),meiotic recombination 11 homolog A (S. cerevisiae),antigen identified by monoclonal antibody Ki 67 |
| 7 | nuclear division | 4 | 73 | 74 | 6246 | 0.054 | 0.012 | 4.624 | 0.011 | 92478\_at,97095\_at,94376\_s\_at,99457\_at | stromal antigen 1,budding uninhibited by benzimidazoles 1 homolog (S. cerevisiae),meiotic recombination 11 homolog A (S. cerevisiae),antigen identified by monoclonal antibody Ki 67 |
| 8 | meiosis | 2 | 23 | 25 | 2164 | 0.08 | 0.011 | 7.526 | 0.028 | 94376\_s\_at,99457\_at | meiotic recombination 11 homolog A (S. cerevisiae),antigen identified by monoclonal antibody Ki 67 |
| 6 | mitotic cell cycle | 15 | 173 | 108 | 9498 | 0.139 | 0.018 | 7.627 | 0 | 92478\_at,97095\_at,103910\_at,103064\_at,96891\_at,100062\_at,101920\_at,103207\_at,160496\_s\_at,92551\_at,92647\_at,95527\_at,95612\_at,98550\_at,162009\_f\_at | stromal antigen 1,budding uninhibited by benzimidazoles 1 homolog (S. cerevisiae),TAF10 RNA polymerase II, TATA box binding protein (TBP)-associated factor,checkpoint kinase 1 homolog (S. pombe),acidic nuclear phosphoprotein 32 family, member B,minichromosome maintenance deficient 3 (S. cerevisiae),polymerase (DNA directed), epsilon 2 (p59 subunit),polymerase (DNA directed), alpha 1,minichromosome maintenance deficient 3 (S. cerevisiae),ligase I, DNA, ATP-dependent,retinoblastoma binding protein 4,chromatin assembly factor 1, subunit A (p150),replication factor C (activator 1) 5,SET translocation,mutS homolog 2 (E. coli) |
| 7 | G2/M transition of mitotic cell cycle | 1 | 3 | 74 | 6246 | 0.014 | 0 | 28.146 | 0.035 | 103064\_at | checkpoint kinase 1 homolog (S. pombe) |
| 7 | S phase of mitotic cell cycle | 11 | 95 | 74 | 6246 | 0.149 | 0.015 | 9.773 | 0 | 96891\_at,100062\_at,101920\_at,103207\_at,160496\_s\_at,92551\_at,92647\_at,95527\_at,95612\_at,98550\_at,162009\_f\_at | acidic nuclear phosphoprotein 32 family, member B,minichromosome maintenance deficient 3 (S. cerevisiae),polymerase (DNA directed), epsilon 2 (p59 subunit),polymerase (DNA directed), alpha 1,minichromosome maintenance deficient 3 (S. cerevisiae),ligase I, DNA, ATP-dependent,retinoblastoma binding protein 4,chromatin assembly factor 1, subunit A (p150),replication factor C (activator 1) 5,SET translocation,mutS homolog 2 (E. coli) |
| 8 | DNA dependent DNA replication | 3 | 31 | 25 | 2164 | 0.12 | 0.014 | 8.374 | 0.005 | 100062\_at,160496\_s\_at,162009\_f\_at | minichromosome maintenance deficient 3 (S. cerevisiae),minichromosome maintenance deficient 3 (S. cerevisiae),mutS homolog 2 (E. coli) |
| 9 | DNA replication initiation | 2 | 10 | 14 | 911 | 0.143 | 0.011 | 13.011 | 0.009 | 100062\_at,160496\_s\_at | minichromosome maintenance deficient 3 (S. cerevisiae),minichromosome maintenance deficient 3 (S. cerevisiae) |
| 7 | DNA replication | 10 | 94 | 74 | 6246 | 0.135 | 0.015 | 8.979 | 0 | 100062\_at,101920\_at,103207\_at,160496\_s\_at,92551\_at,92647\_at,95527\_at,95612\_at,98550\_at,162009\_f\_at | minichromosome maintenance deficient 3 (S. cerevisiae),polymerase (DNA directed), epsilon 2 (p59 subunit),polymerase (DNA directed), alpha 1,minichromosome maintenance deficient 3 (S. cerevisiae),ligase I, DNA, ATP-dependent,retinoblastoma binding protein 4,chromatin assembly factor 1, subunit A (p150),replication factor C (activator 1) 5,SET translocation,mutS homolog 2 (E. coli) |
| 8 | DNA dependent DNA replication | 3 | 31 | 25 | 2164 | 0.12 | 0.014 | 8.374 | 0.005 | 100062\_at,160496\_s\_at,162009\_f\_at | minichromosome maintenance deficient 3 (S. cerevisiae),minichromosome maintenance deficient 3 (S. cerevisiae),mutS homolog 2 (E. coli) |
| 9 | DNA replication initiation | 2 | 10 | 14 | 911 | 0.143 | 0.011 | 13.011 | 0.009 | 100062\_at,160496\_s\_at | minichromosome maintenance deficient 3 (S. cerevisiae),minichromosome maintenance deficient 3 (S. cerevisiae) |
| 7 | retrograde (Golgi to ER) transport | 1 | 1 | 74 | 6246 | 0.014 | 0 | 84.438 | 0.012 | 160518\_at | RIKEN cDNA 1110060F11 gene |
| 9 | NLS-bearing substrate-nucleus import | 1 | 2 | 14 | 911 | 0.071 | 0.002 | 32.468 | 0.031 | 161757\_f\_at | karyopherin (importin) beta 3 |
| 9 | NLS-bearing substrate-nucleus import | 1 | 2 | 14 | 911 | 0.071 | 0.002 | 32.468 | 0.031 | 161757\_f\_at | karyopherin (importin) beta 3 |
| 5 | regulation of neuron differentiation | 1 | 1 | 125 | 11544 | 0.008 | 0 | 88.889 | 0.011 | 99475\_at | suppressor of cytokine signaling 2 |
| 6 | positive regulation of neuron differentiation | 1 | 1 | 108 | 9498 | 0.009 | 0 | 84.182 | 0.011 | 99475\_at | suppressor of cytokine signaling 2 |
| 4 | DNA methylation | 2 | 21 | 161 | 13100 | 0.012 | 0.002 | 7.763 | 0.027 | 101445\_at,93228\_at | DNA methyltransferase (cytosine-5) 1,helicase, lymphoid specific |
| 2 | obsolete biological process | 1 | 3 | 138 | 10540 | 0.007 | 0 | 25.893 | 0.039 | 95732\_at | RIKEN cDNA 1110005L13 gene |
| 3 | cell growth and/or maintenance | 38 | 2128 | 139 | 10726 | 0.273 | 0.198 | 1.378 | 0.02 | 97411\_at,98550\_at,99439\_at,99475\_at,99777\_s\_at,93333\_at,94788\_f\_at,95118\_r\_at,97909\_at,96710\_at,99457\_at,100062\_at,101958\_f\_at,103064\_at,103797\_at,103821\_at,160069\_at,160496\_s\_at,161134\_at,92478\_at,92647\_at,95527\_at,97095\_at,101920\_at,103207\_at,92551\_at,95612\_at,162009\_f\_at,94376\_s\_at,103910\_at,96891\_at,100225\_f\_at,102105\_f\_at,102134\_f\_at,161757\_f\_at,160518\_at,98956\_at,95611\_at | ect2 oncogene,SET translocation,MAS1 oncogene,suppressor of cytokine signaling 2,RIKEN cDNA 6230416J20 gene,tubulin cofactor a,tubulin, beta 5,kinesin family member 22, pseudogene,stathmin 1,histone H2A.F/Z variant,antigen identified by monoclonal antibody Ki 67,minichromosome maintenance deficient 3 (S. cerevisiae),transcription factor Dp 1,checkpoint kinase 1 homolog (S. pombe),cell division cycle 71 homolog (S. cerevisiae)-like 1,cell division cycle 6 homolog (S. cerevisiae),geminin,minichromosome maintenance deficient 3 (S. cerevisiae),RIKEN cDNA 2610201A13 gene,stromal antigen 1,retinoblastoma binding protein 4,chromatin assembly factor 1, subunit A (p150),budding uninhibited by benzimidazoles 1 homolog (S. cerevisiae),polymerase (DNA directed), epsilon 2 (p59 subunit),polymerase (DNA directed), alpha 1,ligase I, DNA, ATP-dependent,replication factor C (activator 1) 5,mutS homolog 2 (E. coli),meiotic recombination 11 homolog A (S. cerevisiae),TAF10 RNA polymerase II, TATA box binding protein (TBP)-associated factor,acidic nuclear phosphoprotein 32 family, member B,proteasome (prosome, macropain) 26S subunit, ATPase 3,prostaglandin D2 synthase (brain),ATP synthase, H+ transporting, mitochondrial F0 complex, subunit c (subunit 9), isoform 2,karyopherin (importin) beta 3,RIKEN cDNA 1110060F11 gene,translocating chain-associating membrane protein 1,lipoprotein lipase |
| 8 | microtubule-based process | 4 | 119 | 25 | 2164 | 0.16 | 0.055 | 2.91 | 0.045 | 93333\_at,94788\_f\_at,95118\_r\_at,97909\_at | tubulin cofactor a,tubulin, beta 5,kinesin family member 22, pseudogene,stathmin 1 |
| 4 | cell proliferation | 24 | 501 | 161 | 13100 | 0.149 | 0.038 | 3.898 | 0 | 99457\_at,100062\_at,101958\_f\_at,103064\_at,103797\_at,103821\_at,160069\_at,160496\_s\_at,161134\_at,92478\_at,92647\_at,95527\_at,97095\_at,101920\_at,103207\_at,92551\_at,95612\_at,98550\_at,162009\_f\_at,94376\_s\_at,103910\_at,96891\_at,97411\_at,99439\_at | antigen identified by monoclonal antibody Ki 67,minichromosome maintenance deficient 3 (S. cerevisiae),transcription factor Dp 1,checkpoint kinase 1 homolog (S. pombe),cell division cycle 71 homolog (S. cerevisiae)-like 1,cell division cycle 6 homolog (S. cerevisiae),geminin,minichromosome maintenance deficient 3 (S. cerevisiae),RIKEN cDNA 2610201A13 gene,stromal antigen 1,retinoblastoma binding protein 4,chromatin assembly factor 1, subunit A (p150),budding uninhibited by benzimidazoles 1 homolog (S. cerevisiae),polymerase (DNA directed), epsilon 2 (p59 subunit),polymerase (DNA directed), alpha 1,ligase I, DNA, ATP-dependent,replication factor C (activator 1) 5,SET translocation,mutS homolog 2 (E. coli),meiotic recombination 11 homolog A (S. cerevisiae),TAF10 RNA polymerase II, TATA box binding protein (TBP)-associated factor,acidic nuclear phosphoprotein 32 family, member B,ect2 oncogene,MAS1 oncogene |
| 5 | cell cycle | 24 | 435 | 125 | 11544 | 0.192 | 0.038 | 5.096 | 0 | 100062\_at,101958\_f\_at,103064\_at,103797\_at,103821\_at,160069\_at,160496\_s\_at,161134\_at,92478\_at,92647\_at,95527\_at,97095\_at,101920\_at,103207\_at,92551\_at,95612\_at,98550\_at,162009\_f\_at,94376\_s\_at,99457\_at,103910\_at,96891\_at,97411\_at,99439\_at | minichromosome maintenance deficient 3 (S. cerevisiae),transcription factor Dp 1,checkpoint kinase 1 homolog (S. pombe),cell division cycle 71 homolog (S. cerevisiae)-like 1,cell division cycle 6 homolog (S. cerevisiae),geminin,minichromosome maintenance deficient 3 (S. cerevisiae),RIKEN cDNA 2610201A13 gene,stromal antigen 1,retinoblastoma binding protein 4,chromatin assembly factor 1, subunit A (p150),budding uninhibited by benzimidazoles 1 homolog (S. cerevisiae),polymerase (DNA directed), epsilon 2 (p59 subunit),polymerase (DNA directed), alpha 1,ligase I, DNA, ATP-dependent,replication factor C (activator 1) 5,SET translocation,mutS homolog 2 (E. coli),meiotic recombination 11 homolog A (S. cerevisiae),antigen identified by monoclonal antibody Ki 67,TAF10 RNA polymerase II, TATA box binding protein (TBP)-associated factor,acidic nuclear phosphoprotein 32 family, member B,ect2 oncogene,MAS1 oncogene |
| 6 | DNA replication and chromosome cycle | 11 | 113 | 108 | 9498 | 0.102 | 0.012 | 8.559 | 0 | 92478\_at,100062\_at,101920\_at,103207\_at,160496\_s\_at,92551\_at,92647\_at,95527\_at,95612\_at,98550\_at,162009\_f\_at | stromal antigen 1,minichromosome maintenance deficient 3 (S. cerevisiae),polymerase (DNA directed), epsilon 2 (p59 subunit),polymerase (DNA directed), alpha 1,minichromosome maintenance deficient 3 (S. cerevisiae),ligase I, DNA, ATP-dependent,retinoblastoma binding protein 4,chromatin assembly factor 1, subunit A (p150),replication factor C (activator 1) 5,SET translocation,mutS homolog 2 (E. coli) |
| 7 | DNA replication | 10 | 94 | 74 | 6246 | 0.135 | 0.015 | 8.979 | 0 | 100062\_at,101920\_at,103207\_at,160496\_s\_at,92551\_at,92647\_at,95527\_at,95612\_at,98550\_at,162009\_f\_at | minichromosome maintenance deficient 3 (S. cerevisiae),polymerase (DNA directed), epsilon 2 (p59 subunit),polymerase (DNA directed), alpha 1,minichromosome maintenance deficient 3 (S. cerevisiae),ligase I, DNA, ATP-dependent,retinoblastoma binding protein 4,chromatin assembly factor 1, subunit A (p150),replication factor C (activator 1) 5,SET translocation,mutS homolog 2 (E. coli) |
| 8 | DNA dependent DNA replication | 3 | 31 | 25 | 2164 | 0.12 | 0.014 | 8.374 | 0.005 | 100062\_at,160496\_s\_at,162009\_f\_at | minichromosome maintenance deficient 3 (S. cerevisiae),minichromosome maintenance deficient 3 (S. cerevisiae),mutS homolog 2 (E. coli) |
| 9 | DNA replication initiation | 2 | 10 | 14 | 911 | 0.143 | 0.011 | 13.011 | 0.009 | 100062\_at,160496\_s\_at | minichromosome maintenance deficient 3 (S. cerevisiae),minichromosome maintenance deficient 3 (S. cerevisiae) |
| 6 | M phase | 4 | 74 | 108 | 9498 | 0.037 | 0.008 | 4.755 | 0.01 | 92478\_at,97095\_at,94376\_s\_at,99457\_at | stromal antigen 1,budding uninhibited by benzimidazoles 1 homolog (S. cerevisiae),meiotic recombination 11 homolog A (S. cerevisiae),antigen identified by monoclonal antibody Ki 67 |
| 7 | nuclear division | 4 | 73 | 74 | 6246 | 0.054 | 0.012 | 4.624 | 0.011 | 92478\_at,97095\_at,94376\_s\_at,99457\_at | stromal antigen 1,budding uninhibited by benzimidazoles 1 homolog (S. cerevisiae),meiotic recombination 11 homolog A (S. cerevisiae),antigen identified by monoclonal antibody Ki 67 |
| 8 | meiosis | 2 | 23 | 25 | 2164 | 0.08 | 0.011 | 7.526 | 0.028 | 94376\_s\_at,99457\_at | meiotic recombination 11 homolog A (S. cerevisiae),antigen identified by monoclonal antibody Ki 67 |
| 6 | mitotic cell cycle | 15 | 173 | 108 | 9498 | 0.139 | 0.018 | 7.627 | 0 | 92478\_at,97095\_at,103910\_at,103064\_at,96891\_at,100062\_at,101920\_at,103207\_at,160496\_s\_at,92551\_at,92647\_at,95527\_at,95612\_at,98550\_at,162009\_f\_at | stromal antigen 1,budding uninhibited by benzimidazoles 1 homolog (S. cerevisiae),TAF10 RNA polymerase II, TATA box binding protein (TBP)-associated factor,checkpoint kinase 1 homolog (S. pombe),acidic nuclear phosphoprotein 32 family, member B,minichromosome maintenance deficient 3 (S. cerevisiae),polymerase (DNA directed), epsilon 2 (p59 subunit),polymerase (DNA directed), alpha 1,minichromosome maintenance deficient 3 (S. cerevisiae),ligase I, DNA, ATP-dependent,retinoblastoma binding protein 4,chromatin assembly factor 1, subunit A (p150),replication factor C (activator 1) 5,SET translocation,mutS homolog 2 (E. coli) |
| 7 | G2/M transition of mitotic cell cycle | 1 | 3 | 74 | 6246 | 0.014 | 0 | 28.146 | 0.035 | 103064\_at | checkpoint kinase 1 homolog (S. pombe) |
| 7 | S phase of mitotic cell cycle | 11 | 95 | 74 | 6246 | 0.149 | 0.015 | 9.773 | 0 | 96891\_at,100062\_at,101920\_at,103207\_at,160496\_s\_at,92551\_at,92647\_at,95527\_at,95612\_at,98550\_at,162009\_f\_at | acidic nuclear phosphoprotein 32 family, member B,minichromosome maintenance deficient 3 (S. cerevisiae),polymerase (DNA directed), epsilon 2 (p59 subunit),polymerase (DNA directed), alpha 1,minichromosome maintenance deficient 3 (S. cerevisiae),ligase I, DNA, ATP-dependent,retinoblastoma binding protein 4,chromatin assembly factor 1, subunit A (p150),replication factor C (activator 1) 5,SET translocation,mutS homolog 2 (E. coli) |
| 8 | DNA dependent DNA replication | 3 | 31 | 25 | 2164 | 0.12 | 0.014 | 8.374 | 0.005 | 100062\_at,160496\_s\_at,162009\_f\_at | minichromosome maintenance deficient 3 (S. cerevisiae),minichromosome maintenance deficient 3 (S. cerevisiae),mutS homolog 2 (E. coli) |
| 9 | DNA replication initiation | 2 | 10 | 14 | 911 | 0.143 | 0.011 | 13.011 | 0.009 | 100062\_at,160496\_s\_at | minichromosome maintenance deficient 3 (S. cerevisiae),minichromosome maintenance deficient 3 (S. cerevisiae) |
| 7 | DNA replication | 10 | 94 | 74 | 6246 | 0.135 | 0.015 | 8.979 | 0 | 100062\_at,101920\_at,103207\_at,160496\_s\_at,92551\_at,92647\_at,95527\_at,95612\_at,98550\_at,162009\_f\_at | minichromosome maintenance deficient 3 (S. cerevisiae),polymerase (DNA directed), epsilon 2 (p59 subunit),polymerase (DNA directed), alpha 1,minichromosome maintenance deficient 3 (S. cerevisiae),ligase I, DNA, ATP-dependent,retinoblastoma binding protein 4,chromatin assembly factor 1, subunit A (p150),replication factor C (activator 1) 5,SET translocation,mutS homolog 2 (E. coli) |
| 8 | DNA dependent DNA replication | 3 | 31 | 25 | 2164 | 0.12 | 0.014 | 8.374 | 0.005 | 100062\_at,160496\_s\_at,162009\_f\_at | minichromosome maintenance deficient 3 (S. cerevisiae),minichromosome maintenance deficient 3 (S. cerevisiae),mutS homolog 2 (E. coli) |
| 9 | DNA replication initiation | 2 | 10 | 14 | 911 | 0.143 | 0.011 | 13.011 | 0.009 | 100062\_at,160496\_s\_at | minichromosome maintenance deficient 3 (S. cerevisiae),minichromosome maintenance deficient 3 (S. cerevisiae) |
| 7 | retrograde (Golgi to ER) transport | 1 | 1 | 74 | 6246 | 0.014 | 0 | 84.438 | 0.012 | 160518\_at | RIKEN cDNA 1110060F11 gene |
| 9 | NLS-bearing substrate-nucleus import | 1 | 2 | 14 | 911 | 0.071 | 0.002 | 32.468 | 0.031 | 161757\_f\_at | karyopherin (importin) beta 3 |
| 9 | NLS-bearing substrate-nucleus import | 1 | 2 | 14 | 911 | 0.071 | 0.002 | 32.468 | 0.031 | 161757\_f\_at | karyopherin (importin) beta 3 |
| 9 | protein myristoylation | 1 | 3 | 14 | 911 | 0.071 | 0.003 | 21.711 | 0.045 | 102047\_at | N-myristoyltransferase 1 |
| 5 | nucleotide biosynthesis | 3 | 74 | 125 | 11544 | 0.024 | 0.006 | 3.744 | 0.046 | 161038\_at,98618\_at,98999\_at | phosphoribosyl pyrophosphate synthetase 2,deoxythymidylate kinase,adenylosuccinate lyase |
| 7 | deoxyribonucleoside diphosphate biosynthesis | 1 | 4 | 74 | 6246 | 0.014 | 0.001 | 21.109 | 0.047 | 98618\_at | deoxythymidylate kinase |
| 8 | pyrimidine deoxyribonucleoside diphosphate biosynthesis | 1 | 4 | 25 | 2164 | 0.04 | 0.002 | 21.622 | 0.045 | 98618\_at | deoxythymidylate kinase |
| 6 | membrane lipid catabolism | 1 | 4 | 108 | 9498 | 0.009 | 0 | 22.048 | 0.045 | 99513\_at | phospholipase A2, group IVA (cytosolic, calcium-dependent) |
| 7 | phospholipid catabolism | 1 | 2 | 74 | 6246 | 0.014 | 0 | 42.219 | 0.024 | 99513\_at | phospholipase A2, group IVA (cytosolic, calcium-dependent) |
| 4 | electron transport | 8 | 313 | 161 | 13100 | 0.05 | 0.024 | 2.08 | 0.04 | 102848\_f\_at,93439\_f\_at,94078\_at,96858\_at,97492\_at,99631\_f\_at,99660\_f\_at,162327\_f\_at | histocompatibility 2, class II antigen E beta,PRKC, apoptosis, WT1, regulator,RIKEN cDNA 1110020P15 gene,programmed cell death 8,RIKEN cDNA 0610040B21 gene,cytochrome c oxidase, subunit VI a, polypeptide 1,cytochrome c oxidase, subunit VIIc,NADH dehydrogenase (ubiquinone) flavoprotein 2 |
| 6 | membrane lipid catabolism | 1 | 4 | 108 | 9498 | 0.009 | 0 | 22.048 | 0.045 | 99513\_at | phospholipase A2, group IVA (cytosolic, calcium-dependent) |
| 7 | phospholipid catabolism | 1 | 2 | 74 | 6246 | 0.014 | 0 | 42.219 | 0.024 | 99513\_at | phospholipase A2, group IVA (cytosolic, calcium-dependent) |
| 5 | DNA metabolism | 15 | 302 | 125 | 11544 | 0.12 | 0.026 | 4.587 | 0 | 94376\_s\_at,100062\_at,101920\_at,103207\_at,160496\_s\_at,92551\_at,92647\_at,95527\_at,95612\_at,98550\_at,162009\_f\_at,101589\_at,96710\_at,103944\_at,101957\_f\_at | meiotic recombination 11 homolog A (S. cerevisiae),minichromosome maintenance deficient 3 (S. cerevisiae),polymerase (DNA directed), epsilon 2 (p59 subunit),polymerase (DNA directed), alpha 1,minichromosome maintenance deficient 3 (S. cerevisiae),ligase I, DNA, ATP-dependent,retinoblastoma binding protein 4,chromatin assembly factor 1, subunit A (p150),replication factor C (activator 1) 5,SET translocation,mutS homolog 2 (E. coli),high mobility group nucleosomal binding domain 2,histone H2A.F/Z variant,RAD51-like 1 (S. cerevisiae),ADP-ribosyltransferase (NAD+; poly (ADP-ribose) polymerase) 1 |
| 6 | DNA recombination | 2 | 25 | 108 | 9498 | 0.019 | 0.003 | 7.042 | 0.032 | 103944\_at,92551\_at | RAD51-like 1 (S. cerevisiae),ligase I, DNA, ATP-dependent |
| 6 | DNA repair | 8 | 99 | 108 | 9498 | 0.074 | 0.01 | 7.108 | 0 | 101957\_f\_at,103944\_at,162009\_f\_at,92551\_at,92647\_at,94376\_s\_at,95527\_at,95612\_at | ADP-ribosyltransferase (NAD+; poly (ADP-ribose) polymerase) 1,RAD51-like 1 (S. cerevisiae),mutS homolog 2 (E. coli),ligase I, DNA, ATP-dependent,retinoblastoma binding protein 4,meiotic recombination 11 homolog A (S. cerevisiae),chromatin assembly factor 1, subunit A (p150),replication factor C (activator 1) 5 |
| 3 | response to endogenous stimulus | 9 | 119 | 139 | 10726 | 0.065 | 0.011 | 5.839 | 0 | 103064\_at,101957\_f\_at,103944\_at,162009\_f\_at,92551\_at,92647\_at,94376\_s\_at,95527\_at,95612\_at | checkpoint kinase 1 homolog (S. pombe),ADP-ribosyltransferase (NAD+; poly (ADP-ribose) polymerase) 1,RAD51-like 1 (S. cerevisiae),mutS homolog 2 (E. coli),ligase I, DNA, ATP-dependent,retinoblastoma binding protein 4,meiotic recombination 11 homolog A (S. cerevisiae),chromatin assembly factor 1, subunit A (p150),replication factor C (activator 1) 5 |
| 4 | response to DNA damage stimulus | 9 | 119 | 161 | 13100 | 0.056 | 0.009 | 6.156 | 0 | 103064\_at,101957\_f\_at,103944\_at,162009\_f\_at,92551\_at,92647\_at,94376\_s\_at,95527\_at,95612\_at | checkpoint kinase 1 homolog (S. pombe),ADP-ribosyltransferase (NAD+; poly (ADP-ribose) polymerase) 1,RAD51-like 1 (S. cerevisiae),mutS homolog 2 (E. coli),ligase I, DNA, ATP-dependent,retinoblastoma binding protein 4,meiotic recombination 11 homolog A (S. cerevisiae),chromatin assembly factor 1, subunit A (p150),replication factor C (activator 1) 5 |

  
