# Supplementary material for: Molecular Signatures of Proliferation and Quiescence in Hematopoietic Stem Cells
Source: PLoS Biol. 2004 Sep 28;2(10):e301. doi: 10.1371/journal.pbio.0020301 (PMC520599; doi:10.1371/journal.pbio.0020301)
Supplement: Table S42 — (215 KB HTML). [file pbio.0020301.st042.html]

   Full Tom Day 6   

# Full Tom Day 6

|  |  |  |  |  |  |  |  |  |  |  |
| --- | --- | --- | --- | --- | --- | --- | --- | --- | --- | --- |
| GOLevel | GOTerm | ProbeCount | ArrayCount | ListGOLevelCount | ArrayGoLevelCount | ListFq | ArrayFq | FoldChange | H-Pvalue | ProbeIds |
| 0 | Gene\_Ontology | 360 | NA | 360 | 0 | 1 | NA | NA | NA | 160551\_at,102838\_at,104212\_at,93784\_at,94267\_i\_at,94268\_f\_at,94931\_at,96743\_at,101254\_at,103605\_g\_at,98595\_at,101096\_s\_at,99991\_at,104423\_at,101741\_at,161872\_f\_at,93838\_at,100057\_at,160297\_at,160299\_at,160485\_r\_at,160543\_at,94110\_f\_at,96734\_at,97412\_at,97807\_at,99156\_at,97055\_s\_at,101440\_at,102821\_s\_at,104135\_at,160536\_at,94394\_at,94506\_at,98975\_at,100116\_at,100990\_g\_at,101521\_at,160412\_at,160876\_at,93764\_at,95413\_at,96861\_at,99129\_at,100331\_g\_at,104297\_at,104301\_at,97828\_at,104762\_r\_at,96089\_at,93456\_r\_at,98075\_at,93548\_at,92831\_at,104476\_at,94933\_at,93593\_f\_at,92778\_i\_at,96634\_at,102197\_at,101407\_at,93812\_at,93815\_at,98524\_f\_at,100400\_at,160872\_f\_at,161004\_at,94789\_r\_at,97276\_at,94862\_i\_at,97979\_at,101061\_at,93062\_at,94014\_at,94210\_at,96849\_at,97477\_at,97478\_at,104322\_at,96956\_at,102126\_at,160266\_r\_at,160531\_at,92646\_at,96291\_f\_at,96292\_r\_at,96293\_at,160503\_at,99151\_at,101954\_at,93251\_at,93833\_s\_at,98039\_at,94897\_at,96775\_at,93112\_at,98587\_at,95660\_at,92770\_at,95491\_at,95891\_at,100128\_at,102853\_at,160538\_at,160659\_at,94294\_at,99522\_at,99632\_at,100156\_at,100612\_at,101065\_at,101067\_at,102001\_at,102631\_at,103418\_at,104738\_at,93041\_at,96289\_at,104733\_at,92788\_f\_at,103805\_at,100459\_at,100618\_f\_at,103534\_at,104380\_at,160076\_at,160256\_at,160856\_at,161487\_f\_at,93084\_at,93734\_i\_at,93735\_f\_at,93993\_at,94277\_at,94323\_at,95061\_at,95091\_at,95441\_at,95690\_at,96048\_at,96336\_at,96668\_at,96892\_at,97248\_at,97374\_at,98930\_at,99147\_at,97424\_at,93533\_at,99128\_at,160125\_at,160126\_at,160203\_at,92798\_at,92799\_g\_at,92800\_i\_at,93596\_i\_at,95656\_i\_at,96611\_at,93014\_at,92636\_f\_at,96670\_at,96947\_at,98959\_at,93970\_at,100917\_at,95654\_at,161147\_f\_at,93952\_r\_at,94892\_r\_at,101866\_at,101964\_at,160293\_at,95561\_at,96696\_at,96627\_at,161897\_f\_at,93258\_at,94275\_at,100073\_at,160550\_i\_at,100576\_at,100577\_at,102409\_at,93008\_at,93999\_at,94313\_at,95049\_at,96029\_at,97200\_f\_at,92874\_f\_at,103881\_at,103939\_at,160314\_at,160711\_at,92589\_at,93029\_at,93754\_at,94276\_at,95408\_at,95426\_at,95634\_at,95635\_g\_at,95636\_at,95693\_at,96268\_at,96678\_at,96948\_at,97449\_at,97820\_at,98527\_at,98966\_at,99566\_at,99613\_at,AFFX-GapdhMur/M32599\_3\_at,AFFX-GapdhMur/M32599\_5\_at,97279\_at,97419\_at,104567\_at,92540\_f\_at,160844\_at,97318\_at,93139\_at,99056\_at,97179\_at,103683\_at,104147\_at,103334\_at,98910\_at,93582\_at,102194\_at,95760\_at,96909\_at,102970\_at,92388\_at,101097\_at,101680\_at,102019\_at,160431\_at,92578\_at,93579\_at,94252\_at,94494\_at,94870\_f\_at,94912\_at,95067\_at,95498\_at,97342\_at,97751\_f\_at,97824\_at,97884\_at,98120\_at,98904\_at,99594\_at,95677\_at,92565\_at,93236\_s\_at,93237\_s\_at,95497\_at,92625\_at,92824\_at,97538\_at,99148\_at,93991\_at,95053\_s\_at,93542\_at,100539\_at,94025\_at,95448\_at,100543\_s\_at,101562\_at,104541\_at,96093\_at,96733\_at,99655\_at,93519\_s\_at,100512\_at,100733\_at,101486\_at,101558\_s\_at,101992\_at,102791\_at,92547\_at,93085\_at,93988\_at,94263\_f\_at,94841\_at,96952\_at,97459\_at,98557\_f\_at,93203\_f\_at,94372\_at,162417\_at,103581\_at,100059\_at,100550\_f\_at,100568\_at,103619\_at,103671\_at,93742\_at,93820\_at,95045\_at,95696\_at,96112\_at,97013\_f\_at,98613\_at,99618\_at,100079\_at,94062\_at,96267\_at,96899\_at,96902\_at,95064\_at,95485\_at,99106\_at,160135\_at,94034\_at,99544\_at,96081\_at,101105\_at,93095\_at,96699\_at,93559\_at,160107\_at,160723\_at,93117\_at,99182\_at,160426\_at,93551\_at,98081\_at,95479\_at,95480\_at,102412\_at,103319\_at,103654\_at,95132\_r\_at,95460\_at,97164\_at,98516\_at,160324\_at,97819\_at,99583\_at,97758\_at,96052\_at,104080\_at,98934\_at,100089\_at,101207\_at,160416\_at,160456\_at,92829\_at,98153\_at,99546\_at,93101\_s\_at,97460\_at,95015\_at,103038\_at,93277\_at,93078\_at,96231\_at |
| 1 | biological\_process | 360 | 6769 | 360 | 6769 | 1 | 1 | 1 | 1 | 160551\_at,102838\_at,104212\_at,93784\_at,94267\_i\_at,94268\_f\_at,94931\_at,96743\_at,101254\_at,103605\_g\_at,98595\_at,101096\_s\_at,99991\_at,104423\_at,101741\_at,161872\_f\_at,93838\_at,100057\_at,160297\_at,160299\_at,160485\_r\_at,160543\_at,94110\_f\_at,96734\_at,97412\_at,97807\_at,99156\_at,97055\_s\_at,101440\_at,102821\_s\_at,104135\_at,160536\_at,94394\_at,94506\_at,98975\_at,100116\_at,100990\_g\_at,101521\_at,160412\_at,160876\_at,93764\_at,95413\_at,96861\_at,99129\_at,100331\_g\_at,104297\_at,104301\_at,97828\_at,104762\_r\_at,96089\_at,93456\_r\_at,98075\_at,93548\_at,92831\_at,104476\_at,94933\_at,93593\_f\_at,92778\_i\_at,96634\_at,102197\_at,101407\_at,93812\_at,93815\_at,98524\_f\_at,100400\_at,160872\_f\_at,161004\_at,94789\_r\_at,97276\_at,94862\_i\_at,97979\_at,101061\_at,93062\_at,94014\_at,94210\_at,96849\_at,97477\_at,97478\_at,104322\_at,96956\_at,102126\_at,160266\_r\_at,160531\_at,92646\_at,96291\_f\_at,96292\_r\_at,96293\_at,160503\_at,99151\_at,101954\_at,93251\_at,93833\_s\_at,98039\_at,94897\_at,96775\_at,93112\_at,98587\_at,95660\_at,92770\_at,95491\_at,95891\_at,100128\_at,102853\_at,160538\_at,160659\_at,94294\_at,99522\_at,99632\_at,100156\_at,100612\_at,101065\_at,101067\_at,102001\_at,102631\_at,103418\_at,104738\_at,93041\_at,96289\_at,104733\_at,92788\_f\_at,103805\_at,100459\_at,100618\_f\_at,103534\_at,104380\_at,160076\_at,160256\_at,160856\_at,161487\_f\_at,93084\_at,93734\_i\_at,93735\_f\_at,93993\_at,94277\_at,94323\_at,95061\_at,95091\_at,95441\_at,95690\_at,96048\_at,96336\_at,96668\_at,96892\_at,97248\_at,97374\_at,98930\_at,99147\_at,97424\_at,93533\_at,99128\_at,160125\_at,160126\_at,160203\_at,92798\_at,92799\_g\_at,92800\_i\_at,93596\_i\_at,95656\_i\_at,96611\_at,93014\_at,92636\_f\_at,96670\_at,96947\_at,98959\_at,93970\_at,100917\_at,95654\_at,161147\_f\_at,93952\_r\_at,94892\_r\_at,101866\_at,101964\_at,160293\_at,95561\_at,96696\_at,96627\_at,161897\_f\_at,93258\_at,94275\_at,100073\_at,160550\_i\_at,100576\_at,100577\_at,102409\_at,93008\_at,93999\_at,94313\_at,95049\_at,96029\_at,97200\_f\_at,92874\_f\_at,103881\_at,103939\_at,160314\_at,160711\_at,92589\_at,93029\_at,93754\_at,94276\_at,95408\_at,95426\_at,95634\_at,95635\_g\_at,95636\_at,95693\_at,96268\_at,96678\_at,96948\_at,97449\_at,97820\_at,98527\_at,98966\_at,99566\_at,99613\_at,AFFX-GapdhMur/M32599\_3\_at,AFFX-GapdhMur/M32599\_5\_at,97279\_at,97419\_at,104567\_at,92540\_f\_at,160844\_at,97318\_at,93139\_at,99056\_at,97179\_at,103683\_at,104147\_at,103334\_at,98910\_at,93582\_at,102194\_at,95760\_at,96909\_at,102970\_at,92388\_at,101097\_at,101680\_at,102019\_at,160431\_at,92578\_at,93579\_at,94252\_at,94494\_at,94870\_f\_at,94912\_at,95067\_at,95498\_at,97342\_at,97751\_f\_at,97824\_at,97884\_at,98120\_at,98904\_at,99594\_at,95677\_at,92565\_at,93236\_s\_at,93237\_s\_at,95497\_at,92625\_at,92824\_at,97538\_at,99148\_at,93991\_at,95053\_s\_at,93542\_at,100539\_at,94025\_at,95448\_at,100543\_s\_at,101562\_at,104541\_at,96093\_at,96733\_at,99655\_at,93519\_s\_at,100512\_at,100733\_at,101486\_at,101558\_s\_at,101992\_at,102791\_at,92547\_at,93085\_at,93988\_at,94263\_f\_at,94841\_at,96952\_at,97459\_at,98557\_f\_at,93203\_f\_at,94372\_at,162417\_at,103581\_at,100059\_at,100550\_f\_at,100568\_at,103619\_at,103671\_at,93742\_at,93820\_at,95045\_at,95696\_at,96112\_at,97013\_f\_at,98613\_at,99618\_at,100079\_at,94062\_at,96267\_at,96899\_at,96902\_at,95064\_at,95485\_at,99106\_at,160135\_at,94034\_at,99544\_at,96081\_at,101105\_at,93095\_at,96699\_at,93559\_at,160107\_at,160723\_at,93117\_at,99182\_at,160426\_at,93551\_at,98081\_at,95479\_at,95480\_at,102412\_at,103319\_at,103654\_at,95132\_r\_at,95460\_at,97164\_at,98516\_at,160324\_at,97819\_at,99583\_at,97758\_at,96052\_at,104080\_at,98934\_at,100089\_at,101207\_at,160416\_at,160456\_at,92829\_at,98153\_at,99546\_at,93101\_s\_at,97460\_at,95015\_at,103038\_at,93277\_at,93078\_at,96231\_at |
| 2 | behavior | 1 | 63 | 537 | 10540 | 0.002 | 0.006 | 0.311 | 0.963 | 160551\_at |
| 3 | behavioral fear response | 1 | 5 | 509 | 10726 | 0.002 | 0 | 4.17 | 0.216 | 160551\_at |
| 3 | learning and/or memory | 1 | 9 | 509 | 10726 | 0.002 | 0.001 | 2.333 | 0.354 | 160551\_at |
| 4 | learning | 1 | 3 | 695 | 13100 | 0.001 | 0 | 6.261 | 0.151 | 160551\_at |
| 2 | cellular process | 167 | 3616 | 537 | 10540 | 0.311 | 0.343 | 0.906 | 0.952 | 102838\_at,104212\_at,93784\_at,94267\_i\_at,94268\_f\_at,94931\_at,96743\_at,160551\_at,101254\_at,103605\_g\_at,98595\_at,101096\_s\_at,99991\_at,104423\_at,101741\_at,161872\_f\_at,93838\_at,100057\_at,160297\_at,160299\_at,160485\_r\_at,160543\_at,94110\_f\_at,96734\_at,97412\_at,97807\_at,99156\_at,97055\_s\_at,101440\_at,102821\_s\_at,104135\_at,160536\_at,94394\_at,94506\_at,98975\_at,100116\_at,100990\_g\_at,101521\_at,160412\_at,160876\_at,93764\_at,95413\_at,96861\_at,99129\_at,100331\_g\_at,104297\_at,104301\_at,97828\_at,104762\_r\_at,96089\_at,93456\_r\_at,98075\_at,93548\_at,92831\_at,104476\_at,94933\_at,93593\_f\_at,92778\_i\_at,96634\_at,102197\_at,101407\_at,93812\_at,93815\_at,98524\_f\_at,100400\_at,160872\_f\_at,161004\_at,94789\_r\_at,97276\_at,94862\_i\_at,97979\_at,101061\_at,93062\_at,94014\_at,94210\_at,96849\_at,97477\_at,97478\_at,104322\_at,96956\_at,102126\_at,160266\_r\_at,160531\_at,92646\_at,96291\_f\_at,96292\_r\_at,96293\_at,160503\_at,99151\_at,101954\_at,93251\_at,93833\_s\_at,98039\_at,94897\_at,96775\_at,93112\_at,98587\_at,95660\_at,92770\_at,95491\_at,95891\_at,100128\_at,102853\_at,160538\_at,160659\_at,94294\_at,99522\_at,99632\_at,100156\_at,100612\_at,101065\_at,101067\_at,102001\_at,102631\_at,103418\_at,104738\_at,93041\_at,96289\_at,104733\_at,92788\_f\_at,103805\_at,100459\_at,100618\_f\_at,103534\_at,104380\_at,160076\_at,160256\_at,160856\_at,161487\_f\_at,93084\_at,93734\_i\_at,93735\_f\_at,93993\_at,94277\_at,94323\_at,95061\_at,95091\_at,95441\_at,95690\_at,96048\_at,96336\_at,96668\_at,96892\_at,97248\_at,97374\_at,98930\_at,99147\_at,97424\_at,93533\_at,99128\_at,160125\_at,160126\_at,160203\_at,92798\_at,92799\_g\_at,92800\_i\_at,93596\_i\_at,95656\_i\_at,96611\_at,93014\_at,92636\_f\_at,96670\_at,96947\_at,98959\_at,93970\_at,100917\_at,95654\_at |
| 3 | cell communication | 36 | 1550 | 509 | 10726 | 0.071 | 0.145 | 0.489 | 1 | 102838\_at,104212\_at,93784\_at,94267\_i\_at,94268\_f\_at,94931\_at,96743\_at,160551\_at,101254\_at,103605\_g\_at,98595\_at,101096\_s\_at,99991\_at,104423\_at,101741\_at,161872\_f\_at,93838\_at,100057\_at,160297\_at,160299\_at,160485\_r\_at,160543\_at,94110\_f\_at,96734\_at,97412\_at,97807\_at,99156\_at,97055\_s\_at,101440\_at,102821\_s\_at,104135\_at,160536\_at,94394\_at,94506\_at,98975\_at,100116\_at |
| 4 | cell adhesion | 7 | 322 | 695 | 13100 | 0.01 | 0.025 | 0.41 | 0.999 | 102838\_at,104212\_at,93784\_at,94267\_i\_at,94268\_f\_at,94931\_at,96743\_at |
| 5 | cell-matrix adhesion | 1 | 50 | 600 | 11544 | 0.002 | 0.004 | 0.386 | 0.931 | 104212\_at |
| 4 | cell-cell signaling | 1 | 123 | 695 | 13100 | 0.001 | 0.009 | 0.153 | 0.999 | 160551\_at |
| 5 | transmission of nerve impulse | 1 | 82 | 600 | 11544 | 0.002 | 0.007 | 0.235 | 0.988 | 160551\_at |
| 6 | synaptic transmission | 1 | 80 | 466 | 9498 | 0.002 | 0.008 | 0.255 | 0.982 | 160551\_at |
| 7 | nerve-nerve synaptic transmission | 1 | 5 | 298 | 6246 | 0.003 | 0.001 | 4.2 | 0.217 | 160551\_at |
| 7 | regulation of synapse | 1 | 4 | 298 | 6246 | 0.003 | 0.001 | 5.25 | 0.178 | 160551\_at |
| 4 | signal transduction | 29 | 1199 | 695 | 13100 | 0.042 | 0.092 | 0.456 | 1 | 101254\_at,103605\_g\_at,98595\_at,101096\_s\_at,99991\_at,104423\_at,101741\_at,161872\_f\_at,93838\_at,104212\_at,100057\_at,160297\_at,160299\_at,160485\_r\_at,160543\_at,94110\_f\_at,96734\_at,97412\_at,97807\_at,99156\_at,97055\_s\_at,101440\_at,102821\_s\_at,104135\_at,160536\_at,94394\_at,94506\_at,98975\_at,100116\_at |
| 5 | cell surface receptor linked signal transduction | 7 | 621 | 600 | 11544 | 0.012 | 0.054 | 0.217 | 1 | 101096\_s\_at,99991\_at,104423\_at,101741\_at,161872\_f\_at,93838\_at,104212\_at |
| 6 | enzyme linked receptor protein signaling pathway | 1 | 131 | 466 | 9498 | 0.002 | 0.014 | 0.156 | 0.999 | 104423\_at |
| 7 | transmembrane receptor protein tyrosine phosphatase signaling pathway | 1 | 30 | 298 | 6246 | 0.003 | 0.005 | 0.7 | 0.77 | 104423\_at |
| 6 | G-protein coupled receptor protein signaling pathway | 3 | 355 | 466 | 9498 | 0.006 | 0.037 | 0.172 | 1 | 101741\_at,161872\_f\_at,93838\_at |
| 7 | neuropeptide signaling pathway | 1 | 45 | 298 | 6246 | 0.003 | 0.007 | 0.467 | 0.89 | 93838\_at |
| 6 | integrin-mediated signaling pathway | 1 | 45 | 466 | 9498 | 0.002 | 0.005 | 0.454 | 0.897 | 104212\_at |
| 5 | intracellular signaling cascade | 20 | 485 | 600 | 11544 | 0.033 | 0.042 | 0.793 | 0.886 | 100057\_at,160297\_at,160299\_at,160485\_r\_at,160543\_at,94110\_f\_at,96734\_at,97412\_at,97807\_at,99156\_at,97055\_s\_at,101254\_at,101440\_at,102821\_s\_at,104135\_at,160536\_at,94394\_at,94506\_at,98975\_at,100116\_at |
| 6 | protein kinase cascade | 1 | 50 | 466 | 9498 | 0.002 | 0.005 | 0.409 | 0.92 | 97055\_s\_at |
| 7 | JNK cascade | 1 | 13 | 298 | 6246 | 0.003 | 0.002 | 1.615 | 0.471 | 97055\_s\_at |
| 8 | activation of JUNKK | 1 | 3 | 130 | 2164 | 0.008 | 0.001 | 5.532 | 0.17 | 97055\_s\_at |
| 6 | small GTPase mediated signal transduction | 10 | 135 | 466 | 9498 | 0.021 | 0.014 | 1.51 | 0.126 | 101254\_at,101440\_at,102821\_s\_at,104135\_at,160536\_at,94394\_at,94506\_at,98975\_at,100116\_at,96734\_at |
| 7 | RAS protein signal transduction | 3 | 15 | 298 | 6246 | 0.01 | 0.002 | 4.196 | 0.032 | 101440\_at,160536\_at,98975\_at |
| 7 | Rho protein signal transduction | 2 | 20 | 298 | 6246 | 0.007 | 0.003 | 2.097 | 0.247 | 100116\_at,96734\_at |
| 5 | two-component signal transduction system (phosphorelay) | 1 | 15 | 600 | 11544 | 0.002 | 0.001 | 1.285 | 0.551 | 94506\_at |
| 3 | cell death | 15 | 207 | 509 | 10726 | 0.029 | 0.019 | 1.527 | 0.068 | 100990\_g\_at,101521\_at,160412\_at,160876\_at,93764\_at,94267\_i\_at,94268\_f\_at,95413\_at,96861\_at,99129\_at,100331\_g\_at,93784\_at,104297\_at,104301\_at,97828\_at |
| 4 | programmed cell death | 15 | 192 | 695 | 13100 | 0.022 | 0.015 | 1.472 | 0.086 | 100990\_g\_at,101521\_at,160412\_at,160876\_at,93764\_at,94267\_i\_at,94268\_f\_at,95413\_at,96861\_at,99129\_at,100331\_g\_at,93784\_at,104297\_at,104301\_at,97828\_at |
| 5 | apoptosis | 15 | 192 | 600 | 11544 | 0.025 | 0.017 | 1.503 | 0.075 | 100990\_g\_at,101521\_at,160412\_at,160876\_at,93764\_at,94267\_i\_at,94268\_f\_at,95413\_at,96861\_at,99129\_at,100331\_g\_at,93784\_at,104297\_at,104301\_at,97828\_at |
| 6 | anti-apoptosis | 3 | 31 | 466 | 9498 | 0.006 | 0.003 | 1.975 | 0.193 | 100331\_g\_at,101521\_at,93784\_at |
| 6 | apoptotic program | 3 | 16 | 466 | 9498 | 0.006 | 0.002 | 3.833 | 0.041 | 104297\_at,104301\_at,96861\_at |
| 7 | apoptotic mitochondrial changes | 1 | 4 | 298 | 6246 | 0.003 | 0.001 | 5.25 | 0.178 | 96861\_at |
| 7 | apoptotic nuclear changes | 1 | 3 | 298 | 6246 | 0.003 | 0 | 7 | 0.136 | 96861\_at |
| 8 | DNA fragmentation | 1 | 3 | 130 | 2164 | 0.008 | 0.001 | 5.532 | 0.17 | 96861\_at |
| 6 | induction of apoptosis | 1 | 28 | 466 | 9498 | 0.002 | 0.003 | 0.729 | 0.756 | 97828\_at |
| 3 | cell differentiation | 6 | 137 | 509 | 10726 | 0.012 | 0.013 | 0.923 | 0.638 | 104762\_r\_at,96089\_at,93456\_r\_at,98075\_at,93548\_at,92831\_at |
| 4 | cell fate commitment | 1 | 13 | 695 | 13100 | 0.001 | 0.001 | 1.455 | 0.508 | 93456\_r\_at |
| 5 | cell fate determination | 1 | 4 | 600 | 11544 | 0.002 | 0 | 4.771 | 0.192 | 93456\_r\_at |
| 6 | mesoderm cell fate determination | 1 | 1 | 466 | 9498 | 0.002 | 0 | 19.545 | 0.049 | 93456\_r\_at |
| 4 | epidermal cell differentiation | 1 | 3 | 695 | 13100 | 0.001 | 0 | 6.261 | 0.151 | 98075\_at |
| 5 | hair cell differentiation | 1 | 3 | 600 | 11544 | 0.002 | 0 | 6.423 | 0.148 | 98075\_at |
| 4 | lymphocytic blood cell differentiation | 1 | 6 | 695 | 13100 | 0.001 | 0 | 3.13 | 0.279 | 93548\_at |
| 4 | myeloid blood cell differentiation | 2 | 7 | 695 | 13100 | 0.003 | 0.001 | 5.434 | 0.049 | 93548\_at,92831\_at |
| 5 | erythrocyte differentiation | 1 | 3 | 600 | 11544 | 0.002 | 0 | 6.423 | 0.148 | 92831\_at |
| 3 | cell growth and/or maintenance | 130 | 2128 | 509 | 10726 | 0.255 | 0.198 | 1.287 | 0.001 | 101254\_at,104476\_at,160536\_at,94394\_at,94506\_at,94933\_at,97412\_at,93593\_f\_at,92778\_i\_at,96634\_at,102197\_at,101407\_at,93812\_at,93815\_at,93784\_at,98524\_f\_at,96734\_at,100116\_at,100400\_at,160872\_f\_at,161004\_at,94789\_r\_at,97276\_at,94862\_i\_at,97979\_at,101061\_at,93062\_at,94014\_at,94210\_at,96849\_at,97477\_at,97478\_at,104322\_at,96956\_at,102126\_at,160266\_r\_at,160531\_at,92646\_at,96291\_f\_at,96292\_r\_at,96293\_at,160503\_at,99151\_at,101954\_at,93251\_at,93833\_s\_at,98039\_at,94897\_at,96775\_at,93112\_at,98587\_at,95660\_at,104762\_r\_at,92770\_at,95491\_at,95891\_at,100128\_at,102853\_at,160538\_at,160659\_at,94294\_at,99522\_at,99632\_at,100156\_at,100612\_at,101065\_at,101067\_at,102001\_at,102631\_at,103418\_at,104738\_at,93041\_at,96289\_at,98975\_at,104733\_at,92788\_f\_at,99129\_at,103805\_at,100459\_at,100618\_f\_at,102821\_s\_at,103534\_at,104380\_at,160076\_at,160256\_at,160543\_at,160856\_at,160876\_at,161487\_f\_at,92831\_at,93084\_at,93734\_i\_at,93735\_f\_at,93993\_at,94277\_at,94323\_at,95061\_at,95091\_at,95441\_at,95690\_at,96048\_at,96336\_at,96668\_at,96892\_at,97248\_at,97374\_at,98930\_at,99147\_at,97424\_at,93533\_at,99128\_at,160125\_at,160126\_at,160203\_at,92798\_at,92799\_g\_at,92800\_i\_at,93596\_i\_at,95656\_i\_at,96611\_at,93014\_at,92636\_f\_at,96670\_at,96947\_at,98959\_at,99156\_at,93970\_at,100917\_at,95654\_at,160551\_at |
| 4 | cell growth | 3 | 51 | 695 | 13100 | 0.004 | 0.004 | 1.111 | 0.513 | 93593\_f\_at,92778\_i\_at,96634\_at |
| 5 | regulation of cell growth | 2 | 38 | 600 | 11544 | 0.003 | 0.003 | 1.012 | 0.595 | 92778\_i\_at,96634\_at |
| 4 | cell homeostasis | 4 | 41 | 695 | 13100 | 0.006 | 0.003 | 1.84 | 0.171 | 102197\_at,101407\_at,93812\_at,93815\_at |
| 5 | cell ion homeostasis | 2 | 37 | 600 | 11544 | 0.003 | 0.003 | 1.037 | 0.58 | 102197\_at,101407\_at |
| 6 | cation homeostasis | 2 | 36 | 466 | 9498 | 0.004 | 0.004 | 1.132 | 0.533 | 102197\_at,101407\_at |
| 7 | di-, tri-valent inorganic cation homeostasis | 2 | 29 | 298 | 6246 | 0.007 | 0.005 | 1.446 | 0.406 | 102197\_at,101407\_at |
| 8 | calcium ion homeostasis | 1 | 13 | 130 | 2164 | 0.008 | 0.006 | 1.28 | 0.554 | 102197\_at |
| 8 | iron ion homeostasis | 1 | 15 | 130 | 2164 | 0.008 | 0.007 | 1.11 | 0.606 | 101407\_at |
| 5 | regulation of cell volume | 2 | 1 | 600 | 11544 | 0.003 | 0 | 37 | 0 | 93812\_at,93815\_at |
| 4 | cell organization and biogenesis | 39 | 530 | 695 | 13100 | 0.056 | 0.04 | 1.387 | 0.024 | 93784\_at,98524\_f\_at,96734\_at,100116\_at,100400\_at,160872\_f\_at,161004\_at,94789\_r\_at,97276\_at,101254\_at,94862\_i\_at,97979\_at,101061\_at,93062\_at,94014\_at,94210\_at,96849\_at,97477\_at,97478\_at,104322\_at,96956\_at,102126\_at,160266\_r\_at,160531\_at,92646\_at,96291\_f\_at,96292\_r\_at,96293\_at,160503\_at,99151\_at,101954\_at,93251\_at,93833\_s\_at,98039\_at,94897\_at,96775\_at,93112\_at,98587\_at,95660\_at |
| 5 | cellular morphogenesis | 2 | 49 | 600 | 11544 | 0.003 | 0.004 | 0.785 | 0.731 | 93784\_at,98524\_f\_at |
| 6 | regulation of cell shape | 2 | 22 | 466 | 9498 | 0.004 | 0.002 | 1.849 | 0.294 | 93784\_at,98524\_f\_at |
| 5 | cytoplasm organization and biogenesis | 29 | 380 | 600 | 11544 | 0.048 | 0.033 | 1.468 | 0.024 | 96734\_at,100116\_at,100400\_at,160872\_f\_at,161004\_at,94789\_r\_at,97276\_at,101254\_at,94862\_i\_at,97979\_at,101061\_at,93062\_at,94014\_at,94210\_at,96849\_at,97477\_at,97478\_at,104322\_at,96956\_at,102126\_at,160266\_r\_at,160531\_at,92646\_at,96291\_f\_at,96292\_r\_at,96293\_at,98524\_f\_at,160503\_at,99151\_at |
| 6 | organelle organization and biogenesis | 18 | 318 | 466 | 9498 | 0.039 | 0.033 | 1.154 | 0.298 | 100116\_at,100400\_at,160872\_f\_at,161004\_at,94789\_r\_at,97276\_at,101254\_at,94862\_i\_at,97979\_at,101061\_at,93062\_at,94014\_at,94210\_at,96849\_at,97477\_at,97478\_at,104322\_at,96956\_at |
| 7 | cytoskeleton organization and biogenesis | 9 | 262 | 298 | 6246 | 0.03 | 0.042 | 0.72 | 0.886 | 100116\_at,100400\_at,160872\_f\_at,161004\_at,94789\_r\_at,97276\_at,101254\_at,94862\_i\_at,97979\_at |
| 8 | microtubule-based process | 9 | 119 | 130 | 2164 | 0.069 | 0.055 | 1.259 | 0.283 | 100116\_at,100400\_at,160872\_f\_at,161004\_at,94789\_r\_at,97276\_at,101254\_at,94862\_i\_at,97979\_at |
| 9 | microtubule cytoskeleton organization and biogenesis | 1 | 12 | 66 | 911 | 0.015 | 0.013 | 1.15 | 0.597 | 101254\_at |
| 10 | M-phase specific microtubule process | 1 | 2 | 5 | 197 | 0.2 | 0.01 | 19.704 | 0.05 | 101254\_at |
| 11 | spindle assembly | 1 | 1 | 4 | 34 | 0.25 | 0.029 | 8.501 | 0.118 | 101254\_at |
| 12 | mitotic spindle assembly | 1 | 1 | 1 | 8 | 1 | 0.125 | 8 | 0.125 | 101254\_at |
| 9 | microtubule-based movement | 2 | 38 | 66 | 911 | 0.03 | 0.042 | 0.726 | 0.779 | 94862\_i\_at,97979\_at |
| 7 | ER organization and biogenesis | 1 | 6 | 298 | 6246 | 0.003 | 0.001 | 3.5 | 0.254 | 101061\_at |
| 8 | protein-ER targeting | 1 | 6 | 130 | 2164 | 0.008 | 0.003 | 2.776 | 0.311 | 101061\_at |
| 9 | cotranslational membrane targeting | 1 | 3 | 66 | 911 | 0.015 | 0.003 | 4.605 | 0.202 | 101061\_at |
| 7 | mitochondrion organization and biogenesis | 6 | 13 | 298 | 6246 | 0.02 | 0.002 | 9.678 | 0 | 93062\_at,94014\_at,94210\_at,96849\_at,97477\_at,97478\_at |
| 8 | mitochondrial genome maintenance | 1 | 4 | 130 | 2164 | 0.008 | 0.002 | 4.157 | 0.22 | 93062\_at |
| 8 | protein-mitochondrial targeting | 5 | 6 | 130 | 2164 | 0.038 | 0.003 | 13.884 | 0 | 94014\_at,94210\_at,96849\_at,97477\_at,97478\_at |
| 9 | mitochondrial translocation | 5 | 6 | 66 | 911 | 0.076 | 0.007 | 11.496 | 0 | 94014\_at,94210\_at,96849\_at,97477\_at,97478\_at |
| 7 | peroxisome organization and biogenesis | 2 | 17 | 298 | 6246 | 0.007 | 0.003 | 2.467 | 0.193 | 104322\_at,96956\_at |
| 6 | ribosome biogenesis and assembly | 10 | 60 | 466 | 9498 | 0.021 | 0.006 | 3.396 | 0.001 | 102126\_at,160266\_r\_at,160531\_at,92646\_at,96291\_f\_at,96292\_r\_at,96293\_at,98524\_f\_at,160503\_at,99151\_at |
| 7 | ribosome biogenesis | 10 | 60 | 298 | 6246 | 0.034 | 0.01 | 3.492 | 0 | 102126\_at,160266\_r\_at,160531\_at,92646\_at,96291\_f\_at,96292\_r\_at,96293\_at,98524\_f\_at,160503\_at,99151\_at |
| 8 | rRNA processing | 3 | 14 | 130 | 2164 | 0.023 | 0.006 | 3.567 | 0.047 | 160503\_at,160531\_at,99151\_at |
| 5 | nuclear organization and biogenesis | 9 | 112 | 600 | 11544 | 0.015 | 0.01 | 1.546 | 0.128 | 101954\_at,93251\_at,93833\_s\_at,98039\_at,94897\_at,96775\_at,93112\_at,98587\_at,95660\_at |
| 6 | chromosome organization and biogenesis (sensu Eukarya) | 9 | 108 | 466 | 9498 | 0.019 | 0.011 | 1.698 | 0.083 | 101954\_at,93251\_at,93833\_s\_at,98039\_at,94897\_at,96775\_at,93112\_at,98587\_at,95660\_at |
| 7 | establishment and/or maintenance of chromatin architecture | 7 | 80 | 298 | 6246 | 0.023 | 0.013 | 1.834 | 0.086 | 94897\_at,96775\_at,101954\_at,93112\_at,93833\_s\_at,98587\_at,95660\_at |
| 8 | chromatin assembly/disassembly | 5 | 48 | 130 | 2164 | 0.038 | 0.022 | 1.734 | 0.158 | 96775\_at,101954\_at,93112\_at,93833\_s\_at,98587\_at |
| 9 | nucleosome assembly | 4 | 28 | 66 | 911 | 0.061 | 0.031 | 1.972 | 0.138 | 101954\_at,93112\_at,93833\_s\_at,98587\_at |
| 8 | chromatin modification | 1 | 36 | 130 | 2164 | 0.008 | 0.017 | 0.462 | 0.895 | 95660\_at |
| 9 | non-covalent chromatin modification | 1 | 13 | 66 | 911 | 0.015 | 0.014 | 1.062 | 0.626 | 95660\_at |
| 10 | chromatin modeling | 1 | 13 | 5 | 197 | 0.2 | 0.066 | 3.031 | 0.292 | 95660\_at |
| 4 | cell proliferation | 35 | 501 | 695 | 13100 | 0.05 | 0.038 | 1.317 | 0.058 | 104762\_r\_at,92770\_at,94933\_at,95491\_at,95891\_at,100128\_at,102853\_at,104476\_at,160538\_at,160659\_at,93112\_at,94294\_at,99522\_at,99632\_at,100156\_at,100612\_at,101065\_at,101067\_at,102001\_at,102631\_at,103418\_at,104738\_at,93041\_at,96289\_at,98975\_at,101254\_at,104733\_at,92788\_f\_at,99129\_at,160536\_at,94394\_at,94506\_at,97412\_at,103805\_at,93784\_at |
| 5 | cell cycle | 32 | 435 | 600 | 11544 | 0.053 | 0.038 | 1.415 | 0.03 | 100128\_at,102853\_at,104476\_at,160538\_at,160659\_at,92770\_at,93112\_at,94294\_at,99522\_at,99632\_at,100156\_at,100612\_at,101065\_at,101067\_at,102001\_at,102631\_at,103418\_at,104738\_at,93041\_at,96289\_at,98975\_at,101254\_at,104733\_at,92788\_f\_at,99129\_at,160536\_at,94394\_at,94506\_at,94933\_at,95891\_at,97412\_at,103805\_at |
| 6 | DNA replication and chromosome cycle | 14 | 113 | 466 | 9498 | 0.03 | 0.012 | 2.524 | 0.001 | 102853\_at,99632\_at,100156\_at,100612\_at,101065\_at,101067\_at,102001\_at,102631\_at,103418\_at,104738\_at,93041\_at,93112\_at,96289\_at,98975\_at |
| 7 | chromosome segregation | 2 | 13 | 298 | 6246 | 0.007 | 0.002 | 3.226 | 0.125 | 102853\_at,99632\_at |
| 8 | mitotic chromosome segregation | 1 | 1 | 130 | 2164 | 0.008 | 0 | 16.717 | 0.06 | 99632\_at |
| 7 | DNA replication | 12 | 94 | 298 | 6246 | 0.04 | 0.015 | 2.676 | 0.002 | 100156\_at,100612\_at,101065\_at,101067\_at,102001\_at,102631\_at,103418\_at,104738\_at,93041\_at,93112\_at,96289\_at,98975\_at |
| 8 | DNA dependent DNA replication | 7 | 31 | 130 | 2164 | 0.054 | 0.014 | 3.758 | 0.002 | 100156\_at,93041\_at,93112\_at,103418\_at,98975\_at,101065\_at,101067\_at |
| 9 | DNA replication initiation | 3 | 10 | 66 | 911 | 0.045 | 0.011 | 4.139 | 0.03 | 100156\_at,93041\_at,93112\_at |
| 9 | DNA strand elongation | 1 | 1 | 66 | 911 | 0.015 | 0.001 | 13.773 | 0.072 | 103418\_at |
| 9 | DNA topological change | 1 | 8 | 66 | 911 | 0.015 | 0.009 | 1.726 | 0.453 | 98975\_at |
| 9 | DNA unwinding | 1 | 4 | 66 | 911 | 0.015 | 0.004 | 3.451 | 0.26 | 93112\_at |
| 9 | regulation of DNA replication | 2 | 1 | 66 | 911 | 0.03 | 0.001 | 27.545 | 0 | 101065\_at,101067\_at |
| 6 | M phase | 8 | 74 | 466 | 9498 | 0.017 | 0.008 | 2.204 | 0.028 | 101254\_at,100128\_at,102853\_at,104733\_at,92788\_f\_at,94294\_at,99632\_at,99129\_at |
| 7 | M phase of mitotic cell cycle | 8 | 57 | 298 | 6246 | 0.027 | 0.009 | 2.941 | 0.005 | 100128\_at,101254\_at,102853\_at,104733\_at,92788\_f\_at,94294\_at,99632\_at,99129\_at |
| 8 | mitosis | 8 | 57 | 130 | 2164 | 0.062 | 0.026 | 2.336 | 0.019 | 100128\_at,101254\_at,102853\_at,104733\_at,92788\_f\_at,94294\_at,99632\_at,99129\_at |
| 9 | mitotic metaphase/anaphase transition | 1 | 1 | 66 | 911 | 0.015 | 0.001 | 13.773 | 0.072 | 99632\_at |
| 10 | regulation of mitotic metaphase/anaphase transition | 1 | 1 | 5 | 197 | 0.2 | 0.005 | 39.37 | 0.025 | 99632\_at |
| 11 | negative regulation of mitotic metaphase/anaphase transition | 1 | 1 | 4 | 34 | 0.25 | 0.029 | 8.501 | 0.118 | 99632\_at |
| 9 | regulation of mitosis | 2 | 3 | 66 | 911 | 0.03 | 0.003 | 9.21 | 0.015 | 99632\_at,99129\_at |
| 10 | regulation of mitotic metaphase/anaphase transition | 1 | 1 | 5 | 197 | 0.2 | 0.005 | 39.37 | 0.025 | 99632\_at |
| 11 | negative regulation of mitotic metaphase/anaphase transition | 1 | 1 | 4 | 34 | 0.25 | 0.029 | 8.501 | 0.118 | 99632\_at |
| 10 | mitotic checkpoint | 2 | 3 | 5 | 197 | 0.4 | 0.015 | 26.264 | 0.002 | 99129\_at,99632\_at |
| 11 | mitotic spindle checkpoint | 2 | 2 | 4 | 34 | 0.5 | 0.059 | 8.501 | 0.011 | 99129\_at,99632\_at |
| 7 | nuclear division | 8 | 73 | 298 | 6246 | 0.027 | 0.012 | 2.297 | 0.022 | 100128\_at,101254\_at,102853\_at,104733\_at,92788\_f\_at,94294\_at,99632\_at,99129\_at |
| 8 | mitosis | 8 | 57 | 130 | 2164 | 0.062 | 0.026 | 2.336 | 0.019 | 100128\_at,101254\_at,102853\_at,104733\_at,92788\_f\_at,94294\_at,99632\_at,99129\_at |
| 9 | mitotic metaphase/anaphase transition | 1 | 1 | 66 | 911 | 0.015 | 0.001 | 13.773 | 0.072 | 99632\_at |
| 10 | regulation of mitotic metaphase/anaphase transition | 1 | 1 | 5 | 197 | 0.2 | 0.005 | 39.37 | 0.025 | 99632\_at |
| 11 | negative regulation of mitotic metaphase/anaphase transition | 1 | 1 | 4 | 34 | 0.25 | 0.029 | 8.501 | 0.118 | 99632\_at |
| 9 | regulation of mitosis | 2 | 3 | 66 | 911 | 0.03 | 0.003 | 9.21 | 0.015 | 99632\_at,99129\_at |
| 10 | regulation of mitotic metaphase/anaphase transition | 1 | 1 | 5 | 197 | 0.2 | 0.005 | 39.37 | 0.025 | 99632\_at |
| 11 | negative regulation of mitotic metaphase/anaphase transition | 1 | 1 | 4 | 34 | 0.25 | 0.029 | 8.501 | 0.118 | 99632\_at |
| 10 | mitotic checkpoint | 2 | 3 | 5 | 197 | 0.4 | 0.015 | 26.264 | 0.002 | 99129\_at,99632\_at |
| 11 | mitotic spindle checkpoint | 2 | 2 | 4 | 34 | 0.5 | 0.059 | 8.501 | 0.011 | 99129\_at,99632\_at |
| 8 | meiosis | 1 | 23 | 130 | 2164 | 0.008 | 0.011 | 0.723 | 0.761 | 102853\_at |
| 6 | mitotic cell cycle | 9 | 173 | 466 | 9498 | 0.019 | 0.018 | 1.06 | 0.477 | 100128\_at,101254\_at,102853\_at,104733\_at,92788\_f\_at,94294\_at,99632\_at,99129\_at,104738\_at |
| 7 | M phase of mitotic cell cycle | 8 | 57 | 298 | 6246 | 0.027 | 0.009 | 2.941 | 0.005 | 100128\_at,101254\_at,102853\_at,104733\_at,92788\_f\_at,94294\_at,99632\_at,99129\_at |
| 8 | mitosis | 8 | 57 | 130 | 2164 | 0.062 | 0.026 | 2.336 | 0.019 | 100128\_at,101254\_at,102853\_at,104733\_at,92788\_f\_at,94294\_at,99632\_at,99129\_at |
| 9 | mitotic metaphase/anaphase transition | 1 | 1 | 66 | 911 | 0.015 | 0.001 | 13.773 | 0.072 | 99632\_at |
| 10 | regulation of mitotic metaphase/anaphase transition | 1 | 1 | 5 | 197 | 0.2 | 0.005 | 39.37 | 0.025 | 99632\_at |
| 11 | negative regulation of mitotic metaphase/anaphase transition | 1 | 1 | 4 | 34 | 0.25 | 0.029 | 8.501 | 0.118 | 99632\_at |
| 9 | regulation of mitosis | 2 | 3 | 66 | 911 | 0.03 | 0.003 | 9.21 | 0.015 | 99632\_at,99129\_at |
| 10 | regulation of mitotic metaphase/anaphase transition | 1 | 1 | 5 | 197 | 0.2 | 0.005 | 39.37 | 0.025 | 99632\_at |
| 11 | negative regulation of mitotic metaphase/anaphase transition | 1 | 1 | 4 | 34 | 0.25 | 0.029 | 8.501 | 0.118 | 99632\_at |
| 10 | mitotic checkpoint | 2 | 3 | 5 | 197 | 0.4 | 0.015 | 26.264 | 0.002 | 99129\_at,99632\_at |
| 11 | mitotic spindle checkpoint | 2 | 2 | 4 | 34 | 0.5 | 0.059 | 8.501 | 0.011 | 99129\_at,99632\_at |
| 7 | G2 phase of mitotic cell cycle | 1 | 3 | 298 | 6246 | 0.003 | 0 | 7 | 0.136 | 104738\_at |
| 6 | regulation of cell cycle | 12 | 204 | 466 | 9498 | 0.026 | 0.021 | 1.199 | 0.3 | 101254\_at,160536\_at,92770\_at,94294\_at,94394\_at,94506\_at,94933\_at,95891\_at,97412\_at,99129\_at,99632\_at,103805\_at |
| 7 | cell cycle checkpoint | 3 | 10 | 298 | 6246 | 0.01 | 0.002 | 6.294 | 0.01 | 99129\_at,99632\_at,103805\_at |
| 8 | DNA damage response, signal transduction resulting in cell cycle arrest | 1 | 7 | 130 | 2164 | 0.008 | 0.003 | 2.381 | 0.352 | 103805\_at |
| 5 | cytokinesis | 1 | 5 | 600 | 11544 | 0.002 | 0 | 3.884 | 0.234 | 104733\_at |
| 5 | regulation of cell proliferation | 1 | 38 | 600 | 11544 | 0.002 | 0.003 | 0.508 | 0.869 | 93784\_at |
| 4 | transport | 64 | 1083 | 695 | 13100 | 0.092 | 0.083 | 1.114 | 0.195 | 100459\_at,100618\_f\_at,102821\_s\_at,103534\_at,104322\_at,104380\_at,160076\_at,160256\_at,160543\_at,160856\_at,160876\_at,161487\_f\_at,92831\_at,93084\_at,93734\_i\_at,93735\_f\_at,93993\_at,94014\_at,94210\_at,94277\_at,94323\_at,95061\_at,95091\_at,95441\_at,95690\_at,96048\_at,96336\_at,96668\_at,96849\_at,96892\_at,97248\_at,97374\_at,97477\_at,97478\_at,98930\_at,99147\_at,97424\_at,93533\_at,99128\_at,160125\_at,160126\_at,160203\_at,92798\_at,92799\_g\_at,92800\_i\_at,93596\_i\_at,95656\_i\_at,96611\_at,93014\_at,101254\_at,94506\_at,92636\_f\_at,96670\_at,96734\_at,96947\_at,98959\_at,99156\_at,101061\_at,93970\_at,100917\_at,93812\_at,93815\_at,95654\_at,160551\_at |
| 5 | amine/polyamine transport | 1 | 19 | 600 | 11544 | 0.002 | 0.002 | 1.012 | 0.638 | 97424\_at |
| 6 | amino acid transport | 1 | 19 | 466 | 9498 | 0.002 | 0.002 | 1.075 | 0.616 | 97424\_at |
| 7 | acidic amino acid transport | 1 | 1 | 298 | 6246 | 0.003 | 0 | 21 | 0.048 | 97424\_at |
| 8 | L-glutamate transport | 1 | 1 | 130 | 2164 | 0.008 | 0 | 16.717 | 0.06 | 97424\_at |
| 5 | carbohydrate transport | 1 | 28 | 600 | 11544 | 0.002 | 0.002 | 0.687 | 0.776 | 104380\_at |
| 6 | nucleotide-sugar transport | 1 | 2 | 466 | 9498 | 0.002 | 0 | 10.238 | 0.096 | 104380\_at |
| 5 | gas transport | 1 | 10 | 600 | 11544 | 0.002 | 0.001 | 1.92 | 0.414 | 103534\_at |
| 6 | oxygen transport | 1 | 10 | 466 | 9498 | 0.002 | 0.001 | 2.048 | 0.395 | 103534\_at |
| 5 | hydrogen transport | 13 | 50 | 600 | 11544 | 0.022 | 0.004 | 5.005 | 0 | 93533\_at,99128\_at,160125\_at,160126\_at,160203\_at,161487\_f\_at,92798\_at,92799\_g\_at,92800\_i\_at,93596\_i\_at,95656\_i\_at,96611\_at,93014\_at |
| 6 | proton transport | 12 | 44 | 466 | 9498 | 0.026 | 0.005 | 5.562 | 0 | 160125\_at,160126\_at,160203\_at,161487\_f\_at,92798\_at,92799\_g\_at,92800\_i\_at,93596\_i\_at,95656\_i\_at,96611\_at,99128\_at,93014\_at |
| 7 | energy coupled proton transport, down the electrochemical gradient | 2 | 5 | 298 | 6246 | 0.007 | 0.001 | 8.387 | 0.021 | 93014\_at,95656\_i\_at |
| 8 | ATP synthesis coupled proton transport | 2 | 5 | 130 | 2164 | 0.015 | 0.002 | 6.658 | 0.032 | 93014\_at,95656\_i\_at |
| 5 | intracellular transport | 31 | 351 | 600 | 11544 | 0.052 | 0.03 | 1.699 | 0.003 | 101254\_at,102821\_s\_at,104322\_at,160076\_at,160256\_at,160543\_at,160876\_at,93993\_at,94014\_at,94210\_at,94277\_at,94323\_at,94506\_at,95091\_at,95441\_at,96668\_at,96849\_at,97477\_at,97478\_at,98930\_at,99147\_at,92636\_f\_at,96670\_at,96734\_at,96947\_at,98959\_at,99156\_at,101061\_at,93970\_at,100618\_f\_at,93084\_at |
| 6 | intracellular protein transport | 29 | 284 | 466 | 9498 | 0.062 | 0.03 | 2.081 | 0 | 101254\_at,102821\_s\_at,104322\_at,160076\_at,160256\_at,160543\_at,160876\_at,93993\_at,94014\_at,94210\_at,94277\_at,94323\_at,94506\_at,95091\_at,95441\_at,96668\_at,96849\_at,97477\_at,97478\_at,98930\_at,99147\_at,92636\_f\_at,96670\_at,96734\_at,96947\_at,98959\_at,99156\_at,101061\_at,93970\_at |
| 7 | protein targeting | 17 | 101 | 298 | 6246 | 0.057 | 0.016 | 3.528 | 0 | 92636\_f\_at,94014\_at,94210\_at,95441\_at,96668\_at,96670\_at,96734\_at,96849\_at,96947\_at,97477\_at,97478\_at,98959\_at,99156\_at,101061\_at,101254\_at,93970\_at,94323\_at |
| 8 | protein-ER targeting | 1 | 6 | 130 | 2164 | 0.008 | 0.003 | 2.776 | 0.311 | 101061\_at |
| 9 | cotranslational membrane targeting | 1 | 3 | 66 | 911 | 0.015 | 0.003 | 4.605 | 0.202 | 101061\_at |
| 8 | protein-mitochondrial targeting | 5 | 6 | 130 | 2164 | 0.038 | 0.003 | 13.884 | 0 | 94014\_at,94210\_at,96849\_at,97477\_at,97478\_at |
| 9 | mitochondrial translocation | 5 | 6 | 66 | 911 | 0.076 | 0.007 | 11.496 | 0 | 94014\_at,94210\_at,96849\_at,97477\_at,97478\_at |
| 8 | protein-nucleus export | 1 | 2 | 130 | 2164 | 0.008 | 0.001 | 8.359 | 0.117 | 101254\_at |
| 8 | protein-nucleus import | 2 | 32 | 130 | 2164 | 0.015 | 0.015 | 1.04 | 0.582 | 93970\_at,94323\_at |
| 6 | mitochondrial transport | 2 | 12 | 466 | 9498 | 0.004 | 0.001 | 3.405 | 0.115 | 100618\_f\_at,93084\_at |
| 6 | nucleocytoplasmic transport | 3 | 40 | 466 | 9498 | 0.006 | 0.004 | 1.53 | 0.313 | 101254\_at,93970\_at,94323\_at |
| 7 | RNA-nucleus export | 1 | 7 | 298 | 6246 | 0.003 | 0.001 | 3 | 0.29 | 101254\_at |
| 5 | ion transport | 9 | 335 | 600 | 11544 | 0.015 | 0.029 | 0.517 | 0.992 | 100917\_at,161487\_f\_at,93812\_at,93815\_at,95654\_at,160551\_at,95061\_at,92831\_at,160856\_at |
| 6 | anion transport | 5 | 79 | 466 | 9498 | 0.011 | 0.008 | 1.29 | 0.346 | 160551\_at,93812\_at,93815\_at,95654\_at,95061\_at |
| 7 | inorganic anion transport | 3 | 50 | 298 | 6246 | 0.01 | 0.008 | 1.257 | 0.429 | 93812\_at,93815\_at,95654\_at |
| 8 | chloride transport | 3 | 39 | 130 | 2164 | 0.023 | 0.018 | 1.281 | 0.419 | 93812\_at,93815\_at,95654\_at |
| 7 | organic anion transport | 1 | 15 | 298 | 6246 | 0.003 | 0.002 | 1.4 | 0.52 | 95061\_at |
| 6 | cation transport | 3 | 236 | 466 | 9498 | 0.006 | 0.025 | 0.259 | 0.999 | 100917\_at,92831\_at,160856\_at |
| 7 | di-, tri-valent inorganic cation transport | 1 | 58 | 298 | 6246 | 0.003 | 0.009 | 0.362 | 0.942 | 92831\_at |
| 8 | transition metal ion transport | 1 | 25 | 130 | 2164 | 0.008 | 0.012 | 0.666 | 0.789 | 92831\_at |
| 9 | iron ion transport | 1 | 11 | 66 | 911 | 0.015 | 0.012 | 1.255 | 0.565 | 92831\_at |
| 7 | metal ion transport | 2 | 184 | 298 | 6246 | 0.007 | 0.029 | 0.228 | 0.999 | 92831\_at,160856\_at |
| 8 | transition metal ion transport | 1 | 25 | 130 | 2164 | 0.008 | 0.012 | 0.666 | 0.789 | 92831\_at |
| 9 | iron ion transport | 1 | 11 | 66 | 911 | 0.015 | 0.012 | 1.255 | 0.565 | 92831\_at |
| 8 | sodium ion transport | 1 | 43 | 130 | 2164 | 0.008 | 0.02 | 0.387 | 0.932 | 160856\_at |
| 5 | protein transport | 29 | 297 | 600 | 11544 | 0.048 | 0.026 | 1.878 | 0.001 | 101254\_at,102821\_s\_at,104322\_at,160076\_at,160256\_at,160543\_at,160876\_at,93993\_at,94014\_at,94210\_at,94277\_at,94323\_at,94506\_at,95091\_at,95441\_at,96668\_at,96849\_at,97477\_at,97478\_at,98930\_at,99147\_at,92636\_f\_at,96670\_at,96734\_at,96947\_at,98959\_at,99156\_at,101061\_at,93970\_at |
| 6 | intracellular protein transport | 29 | 284 | 466 | 9498 | 0.062 | 0.03 | 2.081 | 0 | 101254\_at,102821\_s\_at,104322\_at,160076\_at,160256\_at,160543\_at,160876\_at,93993\_at,94014\_at,94210\_at,94277\_at,94323\_at,94506\_at,95091\_at,95441\_at,96668\_at,96849\_at,97477\_at,97478\_at,98930\_at,99147\_at,92636\_f\_at,96670\_at,96734\_at,96947\_at,98959\_at,99156\_at,101061\_at,93970\_at |
| 7 | protein targeting | 17 | 101 | 298 | 6246 | 0.057 | 0.016 | 3.528 | 0 | 92636\_f\_at,94014\_at,94210\_at,95441\_at,96668\_at,96670\_at,96734\_at,96849\_at,96947\_at,97477\_at,97478\_at,98959\_at,99156\_at,101061\_at,101254\_at,93970\_at,94323\_at |
| 8 | protein-ER targeting | 1 | 6 | 130 | 2164 | 0.008 | 0.003 | 2.776 | 0.311 | 101061\_at |
| 9 | cotranslational membrane targeting | 1 | 3 | 66 | 911 | 0.015 | 0.003 | 4.605 | 0.202 | 101061\_at |
| 8 | protein-mitochondrial targeting | 5 | 6 | 130 | 2164 | 0.038 | 0.003 | 13.884 | 0 | 94014\_at,94210\_at,96849\_at,97477\_at,97478\_at |
| 9 | mitochondrial translocation | 5 | 6 | 66 | 911 | 0.076 | 0.007 | 11.496 | 0 | 94014\_at,94210\_at,96849\_at,97477\_at,97478\_at |
| 8 | protein-nucleus export | 1 | 2 | 130 | 2164 | 0.008 | 0.001 | 8.359 | 0.117 | 101254\_at |
| 8 | protein-nucleus import | 2 | 32 | 130 | 2164 | 0.015 | 0.015 | 1.04 | 0.582 | 93970\_at,94323\_at |
| 5 | vesicle-mediated transport | 1 | 112 | 600 | 11544 | 0.002 | 0.01 | 0.172 | 0.998 | 96734\_at |
| 6 | endocytosis | 1 | 61 | 466 | 9498 | 0.002 | 0.006 | 0.335 | 0.954 | 96734\_at |
| 7 | regulation of endocytosis | 1 | 7 | 298 | 6246 | 0.003 | 0.001 | 3 | 0.29 | 96734\_at |
| 3 | cell motility | 3 | 188 | 509 | 10726 | 0.006 | 0.018 | 0.336 | 0.994 | 94267\_i\_at,94268\_f\_at,93533\_at |
| 4 | cell migration | 2 | 53 | 695 | 13100 | 0.003 | 0.004 | 0.711 | 0.78 | 94267\_i\_at,94268\_f\_at |
| 5 | substrate-bound cell migration | 2 | 3 | 600 | 11544 | 0.003 | 0 | 12.808 | 0.008 | 94267\_i\_at,94268\_f\_at |
| 6 | substrate-bound cell migration, cell attachment to substrate | 2 | 1 | 466 | 9498 | 0.004 | 0 | 39 | 0 | 94267\_i\_at,94268\_f\_at |
| 4 | muscle contraction | 1 | 54 | 695 | 13100 | 0.001 | 0.004 | 0.35 | 0.948 | 93533\_at |
| 5 | regulation of muscle contraction | 1 | 19 | 600 | 11544 | 0.002 | 0.002 | 1.012 | 0.638 | 93533\_at |
| 2 | development | 31 | 990 | 537 | 10540 | 0.058 | 0.094 | 0.615 | 0.999 | 104297\_at,104301\_at,161147\_f\_at,93952\_r\_at,94892\_r\_at,94897\_at,98075\_at,104762\_r\_at,96089\_at,93456\_r\_at,93548\_at,92831\_at,96634\_at,101866\_at,92778\_i\_at,101964\_at,93784\_at,98524\_f\_at,160293\_at,95561\_at,96696\_at,94267\_i\_at,94268\_f\_at,96627\_at,93533\_at,161897\_f\_at,93258\_at,94275\_at,100073\_at,160550\_i\_at,100576\_at |
| 3 | cell differentiation | 6 | 137 | 509 | 10726 | 0.012 | 0.013 | 0.923 | 0.638 | 104762\_r\_at,96089\_at,93456\_r\_at,98075\_at,93548\_at,92831\_at |
| 4 | cell fate commitment | 1 | 13 | 695 | 13100 | 0.001 | 0.001 | 1.455 | 0.508 | 93456\_r\_at |
| 5 | cell fate determination | 1 | 4 | 600 | 11544 | 0.002 | 0 | 4.771 | 0.192 | 93456\_r\_at |
| 6 | mesoderm cell fate determination | 1 | 1 | 466 | 9498 | 0.002 | 0 | 19.545 | 0.049 | 93456\_r\_at |
| 4 | epidermal cell differentiation | 1 | 3 | 695 | 13100 | 0.001 | 0 | 6.261 | 0.151 | 98075\_at |
| 5 | hair cell differentiation | 1 | 3 | 600 | 11544 | 0.002 | 0 | 6.423 | 0.148 | 98075\_at |
| 4 | lymphocytic blood cell differentiation | 1 | 6 | 695 | 13100 | 0.001 | 0 | 3.13 | 0.279 | 93548\_at |
| 4 | myeloid blood cell differentiation | 2 | 7 | 695 | 13100 | 0.003 | 0.001 | 5.434 | 0.049 | 93548\_at,92831\_at |
| 5 | erythrocyte differentiation | 1 | 3 | 600 | 11544 | 0.002 | 0 | 6.423 | 0.148 | 92831\_at |
| 3 | aging | 1 | 4 | 509 | 10726 | 0.002 | 0 | 5.297 | 0.177 | 96634\_at |
| 3 | embryonic development | 2 | 52 | 509 | 10726 | 0.004 | 0.005 | 0.81 | 0.714 | 101866\_at,92778\_i\_at |
| 4 | embryonic development (sensu Animalia) | 1 | 18 | 695 | 13100 | 0.001 | 0.001 | 1.051 | 0.625 | 101866\_at |
| 5 | gastrulation | 1 | 12 | 600 | 11544 | 0.002 | 0.001 | 1.606 | 0.473 | 101866\_at |
| 4 | embryonic pattern specification | 1 | 7 | 695 | 13100 | 0.001 | 0.001 | 2.717 | 0.317 | 92778\_i\_at |
| 5 | patterning of blood vessels | 1 | 3 | 600 | 11544 | 0.002 | 0 | 6.423 | 0.148 | 92778\_i\_at |
| 3 | growth | 1 | 8 | 509 | 10726 | 0.002 | 0.001 | 2.613 | 0.322 | 101964\_at |
| 4 | regulation of growth | 1 | 8 | 695 | 13100 | 0.001 | 0.001 | 2.361 | 0.354 | 101964\_at |
| 3 | morphogenesis | 14 | 594 | 509 | 10726 | 0.028 | 0.055 | 0.497 | 0.999 | 93784\_at,98524\_f\_at,160293\_at,95561\_at,96696\_at,94267\_i\_at,94268\_f\_at,92778\_i\_at,96627\_at,93548\_at,92831\_at,93533\_at,161897\_f\_at,98075\_at |
| 4 | organogenesis | 12 | 544 | 695 | 13100 | 0.017 | 0.042 | 0.416 | 1 | 160293\_at,95561\_at,96696\_at,94267\_i\_at,94268\_f\_at,92778\_i\_at,96627\_at,93548\_at,92831\_at,93533\_at,161897\_f\_at,98075\_at |
| 5 | blood vessel development | 3 | 61 | 600 | 11544 | 0.005 | 0.005 | 0.947 | 0.621 | 94267\_i\_at,94268\_f\_at,92778\_i\_at |
| 6 | angiogenesis | 3 | 50 | 466 | 9498 | 0.006 | 0.005 | 1.224 | 0.447 | 94267\_i\_at,94268\_f\_at,92778\_i\_at |
| 5 | patterning of blood vessels | 1 | 3 | 600 | 11544 | 0.002 | 0 | 6.423 | 0.148 | 92778\_i\_at |
| 5 | hemopoiesis | 3 | 22 | 600 | 11544 | 0.005 | 0.002 | 2.618 | 0.103 | 96627\_at,93548\_at,92831\_at |
| 5 | erythrocyte differentiation | 1 | 3 | 600 | 11544 | 0.002 | 0 | 6.423 | 0.148 | 92831\_at |
| 5 | muscle development | 1 | 75 | 600 | 11544 | 0.002 | 0.006 | 0.257 | 0.982 | 93533\_at |
| 5 | neurogenesis | 1 | 164 | 600 | 11544 | 0.002 | 0.014 | 0.118 | 1 | 161897\_f\_at |
| 5 | respiratory tube development | 1 | 10 | 600 | 11544 | 0.002 | 0.001 | 1.92 | 0.414 | 98075\_at |
| 6 | lung development | 1 | 10 | 466 | 9498 | 0.002 | 0.001 | 2.048 | 0.395 | 98075\_at |
| 5 | skeletal development | 1 | 52 | 600 | 11544 | 0.002 | 0.004 | 0.371 | 0.938 | 93548\_at |
| 6 | ossification | 1 | 25 | 466 | 9498 | 0.002 | 0.003 | 0.817 | 0.716 | 93548\_at |
| 4 | lymphocytic blood cell differentiation | 1 | 6 | 695 | 13100 | 0.001 | 0 | 3.13 | 0.279 | 93548\_at |
| 4 | myeloid blood cell differentiation | 2 | 7 | 695 | 13100 | 0.003 | 0.001 | 5.434 | 0.049 | 93548\_at,92831\_at |
| 5 | erythrocyte differentiation | 1 | 3 | 600 | 11544 | 0.002 | 0 | 6.423 | 0.148 | 92831\_at |
| 3 | pigmentation | 2 | 23 | 509 | 10726 | 0.004 | 0.002 | 1.836 | 0.299 | 93258\_at,94275\_at |
| 4 | pigment metabolism | 2 | 23 | 695 | 13100 | 0.003 | 0.002 | 1.636 | 0.347 | 93258\_at,94275\_at |
| 5 | heme metabolism | 2 | 15 | 600 | 11544 | 0.003 | 0.001 | 2.562 | 0.182 | 93258\_at,94275\_at |
| 6 | heme biosynthesis | 2 | 11 | 466 | 9498 | 0.004 | 0.001 | 3.698 | 0.099 | 93258\_at,94275\_at |
| 3 | regulation of gene expression, epigenetic | 1 | 28 | 509 | 10726 | 0.002 | 0.003 | 0.751 | 0.744 | 100073\_at |
| 4 | DNA methylation | 1 | 21 | 695 | 13100 | 0.001 | 0.002 | 0.9 | 0.682 | 100073\_at |
| 3 | reproduction | 6 | 99 | 509 | 10726 | 0.012 | 0.009 | 1.277 | 0.33 | 101964\_at,160550\_i\_at,100576\_at,93952\_r\_at,94892\_r\_at,94897\_at |
| 4 | sexual reproduction | 6 | 99 | 695 | 13100 | 0.009 | 0.008 | 1.142 | 0.43 | 101964\_at,160550\_i\_at,100576\_at,93952\_r\_at,94892\_r\_at,94897\_at |
| 5 | fertilization | 1 | 14 | 600 | 11544 | 0.002 | 0.001 | 1.38 | 0.527 | 101964\_at |
| 6 | fertilization (sensu Animalia) | 1 | 14 | 466 | 9498 | 0.002 | 0.001 | 1.463 | 0.506 | 101964\_at |
| 5 | gametogenesis | 5 | 86 | 600 | 11544 | 0.008 | 0.007 | 1.118 | 0.465 | 160550\_i\_at,100576\_at,93952\_r\_at,94892\_r\_at,94897\_at |
| 6 | female gamete generation | 1 | 6 | 466 | 9498 | 0.002 | 0.001 | 3.413 | 0.261 | 160550\_i\_at |
| 6 | male gamete generation | 4 | NA | 466 | 9498 | 0.009 | NA | NA | NA | 100576\_at,93952\_r\_at,94892\_r\_at,94897\_at |
| 7 | spermatogenesis | 4 | 66 | 298 | 6246 | 0.013 | 0.011 | 1.27 | 0.387 | 100576\_at,93952\_r\_at,94892\_r\_at,94897\_at |
| 3 | sex determination | 1 | 1 | 509 | 10726 | 0.002 | 0 | 21.778 | 0.047 | 160550\_i\_at |
| 2 | obsolete biological process | 8 | 3 | 537 | 10540 | 0.015 | 0 | 53.214 | 0 | 100577\_at,102409\_at,93008\_at,93999\_at,94313\_at,95049\_at,96029\_at,97200\_f\_at |
| 3 | mRNA splicing | 8 | 54 | 509 | 10726 | 0.016 | 0.005 | 3.125 | 0.004 | 100577\_at,102409\_at,93008\_at,93999\_at,94313\_at,95049\_at,96029\_at,97200\_f\_at |
| 2 | physiological processes | 330 | 5866 | 537 | 10540 | 0.615 | 0.557 | 1.104 | 0.003 | 101254\_at,104476\_at,160536\_at,94394\_at,94506\_at,94933\_at,97412\_at,93593\_f\_at,92778\_i\_at,96634\_at,102197\_at,101407\_at,93812\_at,93815\_at,93784\_at,98524\_f\_at,96734\_at,100116\_at,100400\_at,160872\_f\_at,161004\_at,94789\_r\_at,97276\_at,94862\_i\_at,97979\_at,101061\_at,93062\_at,94014\_at,94210\_at,96849\_at,97477\_at,97478\_at,104322\_at,96956\_at,102126\_at,160266\_r\_at,160531\_at,92646\_at,96291\_f\_at,96292\_r\_at,96293\_at,160503\_at,99151\_at,101954\_at,93251\_at,93833\_s\_at,98039\_at,94897\_at,96775\_at,93112\_at,98587\_at,95660\_at,104762\_r\_at,92770\_at,95491\_at,95891\_at,100128\_at,102853\_at,160538\_at,160659\_at,94294\_at,99522\_at,99632\_at,100156\_at,100612\_at,101065\_at,101067\_at,102001\_at,102631\_at,103418\_at,104738\_at,93041\_at,96289\_at,98975\_at,104733\_at,92788\_f\_at,99129\_at,103805\_at,100459\_at,100618\_f\_at,102821\_s\_at,103534\_at,104380\_at,160076\_at,160256\_at,160543\_at,160856\_at,160876\_at,161487\_f\_at,92831\_at,93084\_at,93734\_i\_at,93735\_f\_at,93993\_at,94277\_at,94323\_at,95061\_at,95091\_at,95441\_at,95690\_at,96048\_at,96336\_at,96668\_at,96892\_at,97248\_at,97374\_at,98930\_at,99147\_at,97424\_at,93533\_at,99128\_at,160125\_at,160126\_at,160203\_at,92798\_at,92799\_g\_at,92800\_i\_at,93596\_i\_at,95656\_i\_at,96611\_at,93014\_at,92636\_f\_at,96670\_at,96947\_at,98959\_at,99156\_at,93970\_at,100917\_at,95654\_at,160551\_at,101741\_at,93838\_at,92874\_f\_at,103881\_at,103939\_at,104297\_at,104301\_at,160293\_at,160314\_at,160711\_at,92589\_at,93029\_at,93754\_at,94276\_at,95408\_at,95426\_at,95634\_at,95635\_g\_at,95636\_at,95693\_at,96268\_at,96678\_at,96948\_at,97449\_at,97820\_at,98527\_at,98966\_at,99566\_at,99613\_at,93258\_at,94275\_at,AFFX-GapdhMur/M32599\_3\_at,AFFX-GapdhMur/M32599\_5\_at,97279\_at,96627\_at,97419\_at,104567\_at,92540\_f\_at,160844\_at,97318\_at,93139\_at,99056\_at,97179\_at,103683\_at,161897\_f\_at,104147\_at,103334\_at,98910\_at,93582\_at,102194\_at,95760\_at,96909\_at,102970\_at,92388\_at,101097\_at,101680\_at,102019\_at,160431\_at,92578\_at,93579\_at,94252\_at,94494\_at,94870\_f\_at,94912\_at,95067\_at,95498\_at,97342\_at,97751\_f\_at,97824\_at,97884\_at,98120\_at,98904\_at,99594\_at,95677\_at,92565\_at,93236\_s\_at,93237\_s\_at,95497\_at,92625\_at,92824\_at,97538\_at,99148\_at,93991\_at,95053\_s\_at,93542\_at,100576\_at,100539\_at,94025\_at,95448\_at,100543\_s\_at,101562\_at,104541\_at,95561\_at,96093\_at,96733\_at,99655\_at,93519\_s\_at,100512\_at,100733\_at,101486\_at,101558\_s\_at,101992\_at,102791\_at,92547\_at,93085\_at,93988\_at,94263\_f\_at,94841\_at,96952\_at,97459\_at,98557\_f\_at,93203\_f\_at,94372\_at,162417\_at,103581\_at,100057\_at,100059\_at,100550\_f\_at,100568\_at,103619\_at,103671\_at,93742\_at,93820\_at,95045\_at,95696\_at,96112\_at,96861\_at,97013\_f\_at,98613\_at,99618\_at,100079\_at,94062\_at,96267\_at,96899\_at,96902\_at,95064\_at,95485\_at,99106\_at,160135\_at,94034\_at,99544\_at,96081\_at,101105\_at,93095\_at,96699\_at,93559\_at,160107\_at,160723\_at,93117\_at,96696\_at,100577\_at,102409\_at,93008\_at,93999\_at,95049\_at,96029\_at,97200\_f\_at,99182\_at,160426\_at,93551\_at,98081\_at,95479\_at,95480\_at,161147\_f\_at,98075\_at,93548\_at,102412\_at,103319\_at,103654\_at,95132\_r\_at,95460\_at,97164\_at,98516\_at,160324\_at,100331\_g\_at,97819\_at,99583\_at,97758\_at,104423\_at,96052\_at,104080\_at,98595\_at,98934\_at,100089\_at,101207\_at,160416\_at,160456\_at,92829\_at,98153\_at,99546\_at,93101\_s\_at,97460\_at,101440\_at,95015\_at,161872\_f\_at,103038\_at,93277\_at,102838\_at,93078\_at,96231\_at |
| 3 | cell growth and/or maintenance | 130 | 2128 | 509 | 10726 | 0.255 | 0.198 | 1.287 | 0.001 | 101254\_at,104476\_at,160536\_at,94394\_at,94506\_at,94933\_at,97412\_at,93593\_f\_at,92778\_i\_at,96634\_at,102197\_at,101407\_at,93812\_at,93815\_at,93784\_at,98524\_f\_at,96734\_at,100116\_at,100400\_at,160872\_f\_at,161004\_at,94789\_r\_at,97276\_at,94862\_i\_at,97979\_at,101061\_at,93062\_at,94014\_at,94210\_at,96849\_at,97477\_at,97478\_at,104322\_at,96956\_at,102126\_at,160266\_r\_at,160531\_at,92646\_at,96291\_f\_at,96292\_r\_at,96293\_at,160503\_at,99151\_at,101954\_at,93251\_at,93833\_s\_at,98039\_at,94897\_at,96775\_at,93112\_at,98587\_at,95660\_at,104762\_r\_at,92770\_at,95491\_at,95891\_at,100128\_at,102853\_at,160538\_at,160659\_at,94294\_at,99522\_at,99632\_at,100156\_at,100612\_at,101065\_at,101067\_at,102001\_at,102631\_at,103418\_at,104738\_at,93041\_at,96289\_at,98975\_at,104733\_at,92788\_f\_at,99129\_at,103805\_at,100459\_at,100618\_f\_at,102821\_s\_at,103534\_at,104380\_at,160076\_at,160256\_at,160543\_at,160856\_at,160876\_at,161487\_f\_at,92831\_at,93084\_at,93734\_i\_at,93735\_f\_at,93993\_at,94277\_at,94323\_at,95061\_at,95091\_at,95441\_at,95690\_at,96048\_at,96336\_at,96668\_at,96892\_at,97248\_at,97374\_at,98930\_at,99147\_at,97424\_at,93533\_at,99128\_at,160125\_at,160126\_at,160203\_at,92798\_at,92799\_g\_at,92800\_i\_at,93596\_i\_at,95656\_i\_at,96611\_at,93014\_at,92636\_f\_at,96670\_at,96947\_at,98959\_at,99156\_at,93970\_at,100917\_at,95654\_at,160551\_at |
| 4 | cell growth | 3 | 51 | 695 | 13100 | 0.004 | 0.004 | 1.111 | 0.513 | 93593\_f\_at,92778\_i\_at,96634\_at |
| 5 | regulation of cell growth | 2 | 38 | 600 | 11544 | 0.003 | 0.003 | 1.012 | 0.595 | 92778\_i\_at,96634\_at |
| 4 | cell homeostasis | 4 | 41 | 695 | 13100 | 0.006 | 0.003 | 1.84 | 0.171 | 102197\_at,101407\_at,93812\_at,93815\_at |
| 5 | cell ion homeostasis | 2 | 37 | 600 | 11544 | 0.003 | 0.003 | 1.037 | 0.58 | 102197\_at,101407\_at |
| 6 | cation homeostasis | 2 | 36 | 466 | 9498 | 0.004 | 0.004 | 1.132 | 0.533 | 102197\_at,101407\_at |
| 7 | di-, tri-valent inorganic cation homeostasis | 2 | 29 | 298 | 6246 | 0.007 | 0.005 | 1.446 | 0.406 | 102197\_at,101407\_at |
| 8 | calcium ion homeostasis | 1 | 13 | 130 | 2164 | 0.008 | 0.006 | 1.28 | 0.554 | 102197\_at |
| 8 | iron ion homeostasis | 1 | 15 | 130 | 2164 | 0.008 | 0.007 | 1.11 | 0.606 | 101407\_at |
| 5 | regulation of cell volume | 2 | 1 | 600 | 11544 | 0.003 | 0 | 37 | 0 | 93812\_at,93815\_at |
| 4 | cell organization and biogenesis | 39 | 530 | 695 | 13100 | 0.056 | 0.04 | 1.387 | 0.024 | 93784\_at,98524\_f\_at,96734\_at,100116\_at,100400\_at,160872\_f\_at,161004\_at,94789\_r\_at,97276\_at,101254\_at,94862\_i\_at,97979\_at,101061\_at,93062\_at,94014\_at,94210\_at,96849\_at,97477\_at,97478\_at,104322\_at,96956\_at,102126\_at,160266\_r\_at,160531\_at,92646\_at,96291\_f\_at,96292\_r\_at,96293\_at,160503\_at,99151\_at,101954\_at,93251\_at,93833\_s\_at,98039\_at,94897\_at,96775\_at,93112\_at,98587\_at,95660\_at |
| 5 | cellular morphogenesis | 2 | 49 | 600 | 11544 | 0.003 | 0.004 | 0.785 | 0.731 | 93784\_at,98524\_f\_at |
| 6 | regulation of cell shape | 2 | 22 | 466 | 9498 | 0.004 | 0.002 | 1.849 | 0.294 | 93784\_at,98524\_f\_at |
| 5 | cytoplasm organization and biogenesis | 29 | 380 | 600 | 11544 | 0.048 | 0.033 | 1.468 | 0.024 | 96734\_at,100116\_at,100400\_at,160872\_f\_at,161004\_at,94789\_r\_at,97276\_at,101254\_at,94862\_i\_at,97979\_at,101061\_at,93062\_at,94014\_at,94210\_at,96849\_at,97477\_at,97478\_at,104322\_at,96956\_at,102126\_at,160266\_r\_at,160531\_at,92646\_at,96291\_f\_at,96292\_r\_at,96293\_at,98524\_f\_at,160503\_at,99151\_at |
| 6 | organelle organization and biogenesis | 18 | 318 | 466 | 9498 | 0.039 | 0.033 | 1.154 | 0.298 | 100116\_at,100400\_at,160872\_f\_at,161004\_at,94789\_r\_at,97276\_at,101254\_at,94862\_i\_at,97979\_at,101061\_at,93062\_at,94014\_at,94210\_at,96849\_at,97477\_at,97478\_at,104322\_at,96956\_at |
| 7 | cytoskeleton organization and biogenesis | 9 | 262 | 298 | 6246 | 0.03 | 0.042 | 0.72 | 0.886 | 100116\_at,100400\_at,160872\_f\_at,161004\_at,94789\_r\_at,97276\_at,101254\_at,94862\_i\_at,97979\_at |
| 8 | microtubule-based process | 9 | 119 | 130 | 2164 | 0.069 | 0.055 | 1.259 | 0.283 | 100116\_at,100400\_at,160872\_f\_at,161004\_at,94789\_r\_at,97276\_at,101254\_at,94862\_i\_at,97979\_at |
| 9 | microtubule cytoskeleton organization and biogenesis | 1 | 12 | 66 | 911 | 0.015 | 0.013 | 1.15 | 0.597 | 101254\_at |
| 10 | M-phase specific microtubule process | 1 | 2 | 5 | 197 | 0.2 | 0.01 | 19.704 | 0.05 | 101254\_at |
| 11 | spindle assembly | 1 | 1 | 4 | 34 | 0.25 | 0.029 | 8.501 | 0.118 | 101254\_at |
| 12 | mitotic spindle assembly | 1 | 1 | 1 | 8 | 1 | 0.125 | 8 | 0.125 | 101254\_at |
| 9 | microtubule-based movement | 2 | 38 | 66 | 911 | 0.03 | 0.042 | 0.726 | 0.779 | 94862\_i\_at,97979\_at |
| 7 | ER organization and biogenesis | 1 | 6 | 298 | 6246 | 0.003 | 0.001 | 3.5 | 0.254 | 101061\_at |
| 8 | protein-ER targeting | 1 | 6 | 130 | 2164 | 0.008 | 0.003 | 2.776 | 0.311 | 101061\_at |
| 9 | cotranslational membrane targeting | 1 | 3 | 66 | 911 | 0.015 | 0.003 | 4.605 | 0.202 | 101061\_at |
| 7 | mitochondrion organization and biogenesis | 6 | 13 | 298 | 6246 | 0.02 | 0.002 | 9.678 | 0 | 93062\_at,94014\_at,94210\_at,96849\_at,97477\_at,97478\_at |
| 8 | mitochondrial genome maintenance | 1 | 4 | 130 | 2164 | 0.008 | 0.002 | 4.157 | 0.22 | 93062\_at |
| 8 | protein-mitochondrial targeting | 5 | 6 | 130 | 2164 | 0.038 | 0.003 | 13.884 | 0 | 94014\_at,94210\_at,96849\_at,97477\_at,97478\_at |
| 9 | mitochondrial translocation | 5 | 6 | 66 | 911 | 0.076 | 0.007 | 11.496 | 0 | 94014\_at,94210\_at,96849\_at,97477\_at,97478\_at |
| 7 | peroxisome organization and biogenesis | 2 | 17 | 298 | 6246 | 0.007 | 0.003 | 2.467 | 0.193 | 104322\_at,96956\_at |
| 6 | ribosome biogenesis and assembly | 10 | 60 | 466 | 9498 | 0.021 | 0.006 | 3.396 | 0.001 | 102126\_at,160266\_r\_at,160531\_at,92646\_at,96291\_f\_at,96292\_r\_at,96293\_at,98524\_f\_at,160503\_at,99151\_at |
| 7 | ribosome biogenesis | 10 | 60 | 298 | 6246 | 0.034 | 0.01 | 3.492 | 0 | 102126\_at,160266\_r\_at,160531\_at,92646\_at,96291\_f\_at,96292\_r\_at,96293\_at,98524\_f\_at,160503\_at,99151\_at |
| 8 | rRNA processing | 3 | 14 | 130 | 2164 | 0.023 | 0.006 | 3.567 | 0.047 | 160503\_at,160531\_at,99151\_at |
| 5 | nuclear organization and biogenesis | 9 | 112 | 600 | 11544 | 0.015 | 0.01 | 1.546 | 0.128 | 101954\_at,93251\_at,93833\_s\_at,98039\_at,94897\_at,96775\_at,93112\_at,98587\_at,95660\_at |
| 6 | chromosome organization and biogenesis (sensu Eukarya) | 9 | 108 | 466 | 9498 | 0.019 | 0.011 | 1.698 | 0.083 | 101954\_at,93251\_at,93833\_s\_at,98039\_at,94897\_at,96775\_at,93112\_at,98587\_at,95660\_at |
| 7 | establishment and/or maintenance of chromatin architecture | 7 | 80 | 298 | 6246 | 0.023 | 0.013 | 1.834 | 0.086 | 94897\_at,96775\_at,101954\_at,93112\_at,93833\_s\_at,98587\_at,95660\_at |
| 8 | chromatin assembly/disassembly | 5 | 48 | 130 | 2164 | 0.038 | 0.022 | 1.734 | 0.158 | 96775\_at,101954\_at,93112\_at,93833\_s\_at,98587\_at |
| 9 | nucleosome assembly | 4 | 28 | 66 | 911 | 0.061 | 0.031 | 1.972 | 0.138 | 101954\_at,93112\_at,93833\_s\_at,98587\_at |
| 8 | chromatin modification | 1 | 36 | 130 | 2164 | 0.008 | 0.017 | 0.462 | 0.895 | 95660\_at |
| 9 | non-covalent chromatin modification | 1 | 13 | 66 | 911 | 0.015 | 0.014 | 1.062 | 0.626 | 95660\_at |
| 10 | chromatin modeling | 1 | 13 | 5 | 197 | 0.2 | 0.066 | 3.031 | 0.292 | 95660\_at |
| 4 | cell proliferation | 35 | 501 | 695 | 13100 | 0.05 | 0.038 | 1.317 | 0.058 | 104762\_r\_at,92770\_at,94933\_at,95491\_at,95891\_at,100128\_at,102853\_at,104476\_at,160538\_at,160659\_at,93112\_at,94294\_at,99522\_at,99632\_at,100156\_at,100612\_at,101065\_at,101067\_at,102001\_at,102631\_at,103418\_at,104738\_at,93041\_at,96289\_at,98975\_at,101254\_at,104733\_at,92788\_f\_at,99129\_at,160536\_at,94394\_at,94506\_at,97412\_at,103805\_at,93784\_at |
| 5 | cell cycle | 32 | 435 | 600 | 11544 | 0.053 | 0.038 | 1.415 | 0.03 | 100128\_at,102853\_at,104476\_at,160538\_at,160659\_at,92770\_at,93112\_at,94294\_at,99522\_at,99632\_at,100156\_at,100612\_at,101065\_at,101067\_at,102001\_at,102631\_at,103418\_at,104738\_at,93041\_at,96289\_at,98975\_at,101254\_at,104733\_at,92788\_f\_at,99129\_at,160536\_at,94394\_at,94506\_at,94933\_at,95891\_at,97412\_at,103805\_at |
| 6 | DNA replication and chromosome cycle | 14 | 113 | 466 | 9498 | 0.03 | 0.012 | 2.524 | 0.001 | 102853\_at,99632\_at,100156\_at,100612\_at,101065\_at,101067\_at,102001\_at,102631\_at,103418\_at,104738\_at,93041\_at,93112\_at,96289\_at,98975\_at |
| 7 | chromosome segregation | 2 | 13 | 298 | 6246 | 0.007 | 0.002 | 3.226 | 0.125 | 102853\_at,99632\_at |
| 8 | mitotic chromosome segregation | 1 | 1 | 130 | 2164 | 0.008 | 0 | 16.717 | 0.06 | 99632\_at |
| 7 | DNA replication | 12 | 94 | 298 | 6246 | 0.04 | 0.015 | 2.676 | 0.002 | 100156\_at,100612\_at,101065\_at,101067\_at,102001\_at,102631\_at,103418\_at,104738\_at,93041\_at,93112\_at,96289\_at,98975\_at |
| 8 | DNA dependent DNA replication | 7 | 31 | 130 | 2164 | 0.054 | 0.014 | 3.758 | 0.002 | 100156\_at,93041\_at,93112\_at,103418\_at,98975\_at,101065\_at,101067\_at |
| 9 | DNA replication initiation | 3 | 10 | 66 | 911 | 0.045 | 0.011 | 4.139 | 0.03 | 100156\_at,93041\_at,93112\_at |
| 9 | DNA strand elongation | 1 | 1 | 66 | 911 | 0.015 | 0.001 | 13.773 | 0.072 | 103418\_at |
| 9 | DNA topological change | 1 | 8 | 66 | 911 | 0.015 | 0.009 | 1.726 | 0.453 | 98975\_at |
| 9 | DNA unwinding | 1 | 4 | 66 | 911 | 0.015 | 0.004 | 3.451 | 0.26 | 93112\_at |
| 9 | regulation of DNA replication | 2 | 1 | 66 | 911 | 0.03 | 0.001 | 27.545 | 0 | 101065\_at,101067\_at |
| 6 | M phase | 8 | 74 | 466 | 9498 | 0.017 | 0.008 | 2.204 | 0.028 | 101254\_at,100128\_at,102853\_at,104733\_at,92788\_f\_at,94294\_at,99632\_at,99129\_at |
| 7 | M phase of mitotic cell cycle | 8 | 57 | 298 | 6246 | 0.027 | 0.009 | 2.941 | 0.005 | 100128\_at,101254\_at,102853\_at,104733\_at,92788\_f\_at,94294\_at,99632\_at,99129\_at |
| 8 | mitosis | 8 | 57 | 130 | 2164 | 0.062 | 0.026 | 2.336 | 0.019 | 100128\_at,101254\_at,102853\_at,104733\_at,92788\_f\_at,94294\_at,99632\_at,99129\_at |
| 9 | mitotic metaphase/anaphase transition | 1 | 1 | 66 | 911 | 0.015 | 0.001 | 13.773 | 0.072 | 99632\_at |
| 10 | regulation of mitotic metaphase/anaphase transition | 1 | 1 | 5 | 197 | 0.2 | 0.005 | 39.37 | 0.025 | 99632\_at |
| 11 | negative regulation of mitotic metaphase/anaphase transition | 1 | 1 | 4 | 34 | 0.25 | 0.029 | 8.501 | 0.118 | 99632\_at |
| 9 | regulation of mitosis | 2 | 3 | 66 | 911 | 0.03 | 0.003 | 9.21 | 0.015 | 99632\_at,99129\_at |
| 10 | regulation of mitotic metaphase/anaphase transition | 1 | 1 | 5 | 197 | 0.2 | 0.005 | 39.37 | 0.025 | 99632\_at |
| 11 | negative regulation of mitotic metaphase/anaphase transition | 1 | 1 | 4 | 34 | 0.25 | 0.029 | 8.501 | 0.118 | 99632\_at |
| 10 | mitotic checkpoint | 2 | 3 | 5 | 197 | 0.4 | 0.015 | 26.264 | 0.002 | 99129\_at,99632\_at |
| 11 | mitotic spindle checkpoint | 2 | 2 | 4 | 34 | 0.5 | 0.059 | 8.501 | 0.011 | 99129\_at,99632\_at |
| 7 | nuclear division | 8 | 73 | 298 | 6246 | 0.027 | 0.012 | 2.297 | 0.022 | 100128\_at,101254\_at,102853\_at,104733\_at,92788\_f\_at,94294\_at,99632\_at,99129\_at |
| 8 | mitosis | 8 | 57 | 130 | 2164 | 0.062 | 0.026 | 2.336 | 0.019 | 100128\_at,101254\_at,102853\_at,104733\_at,92788\_f\_at,94294\_at,99632\_at,99129\_at |
| 9 | mitotic metaphase/anaphase transition | 1 | 1 | 66 | 911 | 0.015 | 0.001 | 13.773 | 0.072 | 99632\_at |
| 10 | regulation of mitotic metaphase/anaphase transition | 1 | 1 | 5 | 197 | 0.2 | 0.005 | 39.37 | 0.025 | 99632\_at |
| 11 | negative regulation of mitotic metaphase/anaphase transition | 1 | 1 | 4 | 34 | 0.25 | 0.029 | 8.501 | 0.118 | 99632\_at |
| 9 | regulation of mitosis | 2 | 3 | 66 | 911 | 0.03 | 0.003 | 9.21 | 0.015 | 99632\_at,99129\_at |
| 10 | regulation of mitotic metaphase/anaphase transition | 1 | 1 | 5 | 197 | 0.2 | 0.005 | 39.37 | 0.025 | 99632\_at |
| 11 | negative regulation of mitotic metaphase/anaphase transition | 1 | 1 | 4 | 34 | 0.25 | 0.029 | 8.501 | 0.118 | 99632\_at |
| 10 | mitotic checkpoint | 2 | 3 | 5 | 197 | 0.4 | 0.015 | 26.264 | 0.002 | 99129\_at,99632\_at |
| 11 | mitotic spindle checkpoint | 2 | 2 | 4 | 34 | 0.5 | 0.059 | 8.501 | 0.011 | 99129\_at,99632\_at |
| 8 | meiosis | 1 | 23 | 130 | 2164 | 0.008 | 0.011 | 0.723 | 0.761 | 102853\_at |
| 6 | mitotic cell cycle | 9 | 173 | 466 | 9498 | 0.019 | 0.018 | 1.06 | 0.477 | 100128\_at,101254\_at,102853\_at,104733\_at,92788\_f\_at,94294\_at,99632\_at,99129\_at,104738\_at |
| 7 | M phase of mitotic cell cycle | 8 | 57 | 298 | 6246 | 0.027 | 0.009 | 2.941 | 0.005 | 100128\_at,101254\_at,102853\_at,104733\_at,92788\_f\_at,94294\_at,99632\_at,99129\_at |
| 8 | mitosis | 8 | 57 | 130 | 2164 | 0.062 | 0.026 | 2.336 | 0.019 | 100128\_at,101254\_at,102853\_at,104733\_at,92788\_f\_at,94294\_at,99632\_at,99129\_at |
| 9 | mitotic metaphase/anaphase transition | 1 | 1 | 66 | 911 | 0.015 | 0.001 | 13.773 | 0.072 | 99632\_at |
| 10 | regulation of mitotic metaphase/anaphase transition | 1 | 1 | 5 | 197 | 0.2 | 0.005 | 39.37 | 0.025 | 99632\_at |
| 11 | negative regulation of mitotic metaphase/anaphase transition | 1 | 1 | 4 | 34 | 0.25 | 0.029 | 8.501 | 0.118 | 99632\_at |
| 9 | regulation of mitosis | 2 | 3 | 66 | 911 | 0.03 | 0.003 | 9.21 | 0.015 | 99632\_at,99129\_at |
| 10 | regulation of mitotic metaphase/anaphase transition | 1 | 1 | 5 | 197 | 0.2 | 0.005 | 39.37 | 0.025 | 99632\_at |
| 11 | negative regulation of mitotic metaphase/anaphase transition | 1 | 1 | 4 | 34 | 0.25 | 0.029 | 8.501 | 0.118 | 99632\_at |
| 10 | mitotic checkpoint | 2 | 3 | 5 | 197 | 0.4 | 0.015 | 26.264 | 0.002 | 99129\_at,99632\_at |
| 11 | mitotic spindle checkpoint | 2 | 2 | 4 | 34 | 0.5 | 0.059 | 8.501 | 0.011 | 99129\_at,99632\_at |
| 7 | G2 phase of mitotic cell cycle | 1 | 3 | 298 | 6246 | 0.003 | 0 | 7 | 0.136 | 104738\_at |
| 6 | regulation of cell cycle | 12 | 204 | 466 | 9498 | 0.026 | 0.021 | 1.199 | 0.3 | 101254\_at,160536\_at,92770\_at,94294\_at,94394\_at,94506\_at,94933\_at,95891\_at,97412\_at,99129\_at,99632\_at,103805\_at |
| 7 | cell cycle checkpoint | 3 | 10 | 298 | 6246 | 0.01 | 0.002 | 6.294 | 0.01 | 99129\_at,99632\_at,103805\_at |
| 8 | DNA damage response, signal transduction resulting in cell cycle arrest | 1 | 7 | 130 | 2164 | 0.008 | 0.003 | 2.381 | 0.352 | 103805\_at |
| 5 | cytokinesis | 1 | 5 | 600 | 11544 | 0.002 | 0 | 3.884 | 0.234 | 104733\_at |
| 5 | regulation of cell proliferation | 1 | 38 | 600 | 11544 | 0.002 | 0.003 | 0.508 | 0.869 | 93784\_at |
| 4 | transport | 64 | 1083 | 695 | 13100 | 0.092 | 0.083 | 1.114 | 0.195 | 100459\_at,100618\_f\_at,102821\_s\_at,103534\_at,104322\_at,104380\_at,160076\_at,160256\_at,160543\_at,160856\_at,160876\_at,161487\_f\_at,92831\_at,93084\_at,93734\_i\_at,93735\_f\_at,93993\_at,94014\_at,94210\_at,94277\_at,94323\_at,95061\_at,95091\_at,95441\_at,95690\_at,96048\_at,96336\_at,96668\_at,96849\_at,96892\_at,97248\_at,97374\_at,97477\_at,97478\_at,98930\_at,99147\_at,97424\_at,93533\_at,99128\_at,160125\_at,160126\_at,160203\_at,92798\_at,92799\_g\_at,92800\_i\_at,93596\_i\_at,95656\_i\_at,96611\_at,93014\_at,101254\_at,94506\_at,92636\_f\_at,96670\_at,96734\_at,96947\_at,98959\_at,99156\_at,101061\_at,93970\_at,100917\_at,93812\_at,93815\_at,95654\_at,160551\_at |
| 5 | amine/polyamine transport | 1 | 19 | 600 | 11544 | 0.002 | 0.002 | 1.012 | 0.638 | 97424\_at |
| 6 | amino acid transport | 1 | 19 | 466 | 9498 | 0.002 | 0.002 | 1.075 | 0.616 | 97424\_at |
| 7 | acidic amino acid transport | 1 | 1 | 298 | 6246 | 0.003 | 0 | 21 | 0.048 | 97424\_at |
| 8 | L-glutamate transport | 1 | 1 | 130 | 2164 | 0.008 | 0 | 16.717 | 0.06 | 97424\_at |
| 5 | carbohydrate transport | 1 | 28 | 600 | 11544 | 0.002 | 0.002 | 0.687 | 0.776 | 104380\_at |
| 6 | nucleotide-sugar transport | 1 | 2 | 466 | 9498 | 0.002 | 0 | 10.238 | 0.096 | 104380\_at |
| 5 | gas transport | 1 | 10 | 600 | 11544 | 0.002 | 0.001 | 1.92 | 0.414 | 103534\_at |
| 6 | oxygen transport | 1 | 10 | 466 | 9498 | 0.002 | 0.001 | 2.048 | 0.395 | 103534\_at |
| 5 | hydrogen transport | 13 | 50 | 600 | 11544 | 0.022 | 0.004 | 5.005 | 0 | 93533\_at,99128\_at,160125\_at,160126\_at,160203\_at,161487\_f\_at,92798\_at,92799\_g\_at,92800\_i\_at,93596\_i\_at,95656\_i\_at,96611\_at,93014\_at |
| 6 | proton transport | 12 | 44 | 466 | 9498 | 0.026 | 0.005 | 5.562 | 0 | 160125\_at,160126\_at,160203\_at,161487\_f\_at,92798\_at,92799\_g\_at,92800\_i\_at,93596\_i\_at,95656\_i\_at,96611\_at,99128\_at,93014\_at |
| 7 | energy coupled proton transport, down the electrochemical gradient | 2 | 5 | 298 | 6246 | 0.007 | 0.001 | 8.387 | 0.021 | 93014\_at,95656\_i\_at |
| 8 | ATP synthesis coupled proton transport | 2 | 5 | 130 | 2164 | 0.015 | 0.002 | 6.658 | 0.032 | 93014\_at,95656\_i\_at |
| 5 | intracellular transport | 31 | 351 | 600 | 11544 | 0.052 | 0.03 | 1.699 | 0.003 | 101254\_at,102821\_s\_at,104322\_at,160076\_at,160256\_at,160543\_at,160876\_at,93993\_at,94014\_at,94210\_at,94277\_at,94323\_at,94506\_at,95091\_at,95441\_at,96668\_at,96849\_at,97477\_at,97478\_at,98930\_at,99147\_at,92636\_f\_at,96670\_at,96734\_at,96947\_at,98959\_at,99156\_at,101061\_at,93970\_at,100618\_f\_at,93084\_at |
| 6 | intracellular protein transport | 29 | 284 | 466 | 9498 | 0.062 | 0.03 | 2.081 | 0 | 101254\_at,102821\_s\_at,104322\_at,160076\_at,160256\_at,160543\_at,160876\_at,93993\_at,94014\_at,94210\_at,94277\_at,94323\_at,94506\_at,95091\_at,95441\_at,96668\_at,96849\_at,97477\_at,97478\_at,98930\_at,99147\_at,92636\_f\_at,96670\_at,96734\_at,96947\_at,98959\_at,99156\_at,101061\_at,93970\_at |
| 7 | protein targeting | 17 | 101 | 298 | 6246 | 0.057 | 0.016 | 3.528 | 0 | 92636\_f\_at,94014\_at,94210\_at,95441\_at,96668\_at,96670\_at,96734\_at,96849\_at,96947\_at,97477\_at,97478\_at,98959\_at,99156\_at,101061\_at,101254\_at,93970\_at,94323\_at |
| 8 | protein-ER targeting | 1 | 6 | 130 | 2164 | 0.008 | 0.003 | 2.776 | 0.311 | 101061\_at |
| 9 | cotranslational membrane targeting | 1 | 3 | 66 | 911 | 0.015 | 0.003 | 4.605 | 0.202 | 101061\_at |
| 8 | protein-mitochondrial targeting | 5 | 6 | 130 | 2164 | 0.038 | 0.003 | 13.884 | 0 | 94014\_at,94210\_at,96849\_at,97477\_at,97478\_at |
| 9 | mitochondrial translocation | 5 | 6 | 66 | 911 | 0.076 | 0.007 | 11.496 | 0 | 94014\_at,94210\_at,96849\_at,97477\_at,97478\_at |
| 8 | protein-nucleus export | 1 | 2 | 130 | 2164 | 0.008 | 0.001 | 8.359 | 0.117 | 101254\_at |
| 8 | protein-nucleus import | 2 | 32 | 130 | 2164 | 0.015 | 0.015 | 1.04 | 0.582 | 93970\_at,94323\_at |
| 6 | mitochondrial transport | 2 | 12 | 466 | 9498 | 0.004 | 0.001 | 3.405 | 0.115 | 100618\_f\_at,93084\_at |
| 6 | nucleocytoplasmic transport | 3 | 40 | 466 | 9498 | 0.006 | 0.004 | 1.53 | 0.313 | 101254\_at,93970\_at,94323\_at |
| 7 | RNA-nucleus export | 1 | 7 | 298 | 6246 | 0.003 | 0.001 | 3 | 0.29 | 101254\_at |
| 5 | ion transport | 9 | 335 | 600 | 11544 | 0.015 | 0.029 | 0.517 | 0.992 | 100917\_at,161487\_f\_at,93812\_at,93815\_at,95654\_at,160551\_at,95061\_at,92831\_at,160856\_at |
| 6 | anion transport | 5 | 79 | 466 | 9498 | 0.011 | 0.008 | 1.29 | 0.346 | 160551\_at,93812\_at,93815\_at,95654\_at,95061\_at |
| 7 | inorganic anion transport | 3 | 50 | 298 | 6246 | 0.01 | 0.008 | 1.257 | 0.429 | 93812\_at,93815\_at,95654\_at |
| 8 | chloride transport | 3 | 39 | 130 | 2164 | 0.023 | 0.018 | 1.281 | 0.419 | 93812\_at,93815\_at,95654\_at |
| 7 | organic anion transport | 1 | 15 | 298 | 6246 | 0.003 | 0.002 | 1.4 | 0.52 | 95061\_at |
| 6 | cation transport | 3 | 236 | 466 | 9498 | 0.006 | 0.025 | 0.259 | 0.999 | 100917\_at,92831\_at,160856\_at |
| 7 | di-, tri-valent inorganic cation transport | 1 | 58 | 298 | 6246 | 0.003 | 0.009 | 0.362 | 0.942 | 92831\_at |
| 8 | transition metal ion transport | 1 | 25 | 130 | 2164 | 0.008 | 0.012 | 0.666 | 0.789 | 92831\_at |
| 9 | iron ion transport | 1 | 11 | 66 | 911 | 0.015 | 0.012 | 1.255 | 0.565 | 92831\_at |
| 7 | metal ion transport | 2 | 184 | 298 | 6246 | 0.007 | 0.029 | 0.228 | 0.999 | 92831\_at,160856\_at |
| 8 | transition metal ion transport | 1 | 25 | 130 | 2164 | 0.008 | 0.012 | 0.666 | 0.789 | 92831\_at |
| 9 | iron ion transport | 1 | 11 | 66 | 911 | 0.015 | 0.012 | 1.255 | 0.565 | 92831\_at |
| 8 | sodium ion transport | 1 | 43 | 130 | 2164 | 0.008 | 0.02 | 0.387 | 0.932 | 160856\_at |
| 5 | protein transport | 29 | 297 | 600 | 11544 | 0.048 | 0.026 | 1.878 | 0.001 | 101254\_at,102821\_s\_at,104322\_at,160076\_at,160256\_at,160543\_at,160876\_at,93993\_at,94014\_at,94210\_at,94277\_at,94323\_at,94506\_at,95091\_at,95441\_at,96668\_at,96849\_at,97477\_at,97478\_at,98930\_at,99147\_at,92636\_f\_at,96670\_at,96734\_at,96947\_at,98959\_at,99156\_at,101061\_at,93970\_at |
| 6 | intracellular protein transport | 29 | 284 | 466 | 9498 | 0.062 | 0.03 | 2.081 | 0 | 101254\_at,102821\_s\_at,104322\_at,160076\_at,160256\_at,160543\_at,160876\_at,93993\_at,94014\_at,94210\_at,94277\_at,94323\_at,94506\_at,95091\_at,95441\_at,96668\_at,96849\_at,97477\_at,97478\_at,98930\_at,99147\_at,92636\_f\_at,96670\_at,96734\_at,96947\_at,98959\_at,99156\_at,101061\_at,93970\_at |
| 7 | protein targeting | 17 | 101 | 298 | 6246 | 0.057 | 0.016 | 3.528 | 0 | 92636\_f\_at,94014\_at,94210\_at,95441\_at,96668\_at,96670\_at,96734\_at,96849\_at,96947\_at,97477\_at,97478\_at,98959\_at,99156\_at,101061\_at,101254\_at,93970\_at,94323\_at |
| 8 | protein-ER targeting | 1 | 6 | 130 | 2164 | 0.008 | 0.003 | 2.776 | 0.311 | 101061\_at |
| 9 | cotranslational membrane targeting | 1 | 3 | 66 | 911 | 0.015 | 0.003 | 4.605 | 0.202 | 101061\_at |
| 8 | protein-mitochondrial targeting | 5 | 6 | 130 | 2164 | 0.038 | 0.003 | 13.884 | 0 | 94014\_at,94210\_at,96849\_at,97477\_at,97478\_at |
| 9 | mitochondrial translocation | 5 | 6 | 66 | 911 | 0.076 | 0.007 | 11.496 | 0 | 94014\_at,94210\_at,96849\_at,97477\_at,97478\_at |
| 8 | protein-nucleus export | 1 | 2 | 130 | 2164 | 0.008 | 0.001 | 8.359 | 0.117 | 101254\_at |
| 8 | protein-nucleus import | 2 | 32 | 130 | 2164 | 0.015 | 0.015 | 1.04 | 0.582 | 93970\_at,94323\_at |
| 5 | vesicle-mediated transport | 1 | 112 | 600 | 11544 | 0.002 | 0.01 | 0.172 | 0.998 | 96734\_at |
| 6 | endocytosis | 1 | 61 | 466 | 9498 | 0.002 | 0.006 | 0.335 | 0.954 | 96734\_at |
| 7 | regulation of endocytosis | 1 | 7 | 298 | 6246 | 0.003 | 0.001 | 3 | 0.29 | 96734\_at |
| 3 | circulation | 2 | 40 | 509 | 10726 | 0.004 | 0.004 | 1.054 | 0.572 | 101741\_at,93838\_at |
| 4 | regulation of blood pressure | 2 | 18 | 695 | 13100 | 0.003 | 0.001 | 2.102 | 0.247 | 101741\_at,93838\_at |
| 3 | digestion | 1 | 12 | 509 | 10726 | 0.002 | 0.001 | 1.75 | 0.442 | 92874\_f\_at |
| 3 | hemostasis | 1 | 44 | 509 | 10726 | 0.002 | 0.004 | 0.478 | 0.883 | 93838\_at |
| 4 | blood coagulation | 1 | 42 | 695 | 13100 | 0.001 | 0.003 | 0.449 | 0.899 | 93838\_at |
| 3 | metabolism | 260 | 3908 | 509 | 10726 | 0.511 | 0.364 | 1.402 | 0 | 103881\_at,103939\_at,104297\_at,104301\_at,160293\_at,160314\_at,160711\_at,92589\_at,93029\_at,93754\_at,94276\_at,95408\_at,95426\_at,95634\_at,95635\_g\_at,95636\_at,95693\_at,96268\_at,96678\_at,96948\_at,97449\_at,97820\_at,98527\_at,98966\_at,99566\_at,99613\_at,93258\_at,94275\_at,AFFX-GapdhMur/M32599\_3\_at,AFFX-GapdhMur/M32599\_5\_at,97279\_at,96627\_at,97419\_at,104567\_at,92540\_f\_at,160844\_at,97318\_at,93139\_at,99056\_at,96336\_at,97179\_at,103683\_at,161897\_f\_at,104147\_at,103334\_at,160125\_at,160126\_at,92798\_at,92799\_g\_at,92800\_i\_at,93596\_i\_at,95656\_i\_at,96611\_at,99128\_at,93014\_at,98910\_at,93582\_at,102194\_at,95760\_at,96909\_at,102970\_at,92388\_at,101097\_at,101680\_at,102019\_at,160431\_at,92578\_at,92646\_at,93062\_at,93579\_at,94252\_at,94494\_at,94870\_f\_at,94912\_at,95067\_at,95498\_at,96291\_f\_at,96292\_r\_at,96293\_at,97342\_at,97751\_f\_at,97824\_at,97884\_at,98120\_at,98524\_f\_at,98904\_at,99594\_at,95677\_at,92565\_at,93236\_s\_at,93237\_s\_at,95497\_at,92625\_at,92824\_at,97538\_at,99148\_at,93991\_at,95053\_s\_at,93542\_at,100576\_at,100539\_at,93734\_i\_at,93735\_f\_at,94025\_at,95448\_at,100543\_s\_at,101562\_at,104541\_at,92874\_f\_at,95561\_at,96093\_at,96733\_at,99655\_at,93519\_s\_at,100512\_at,100733\_at,101486\_at,101558\_s\_at,101992\_at,102791\_at,92547\_at,93085\_at,93988\_at,94263\_f\_at,94841\_at,96892\_at,96952\_at,97459\_at,98557\_f\_at,98975\_at,93203\_f\_at,94372\_at,162417\_at,103581\_at,100057\_at,100059\_at,100156\_at,100550\_f\_at,100568\_at,103619\_at,103671\_at,93041\_at,93742\_at,93820\_at,95045\_at,95696\_at,96112\_at,96861\_at,96947\_at,97013\_f\_at,98613\_at,99618\_at,100079\_at,94062\_at,96267\_at,96899\_at,96902\_at,95064\_at,95485\_at,99106\_at,160135\_at,94034\_at,96289\_at,99544\_at,101254\_at,102631\_at,96081\_at,100612\_at,101065\_at,101067\_at,102001\_at,103418\_at,104738\_at,93112\_at,101105\_at,93095\_at,93251\_at,96699\_at,98039\_at,94897\_at,96775\_at,101954\_at,93833\_s\_at,98587\_at,95660\_at,100459\_at,102853\_at,93559\_at,160107\_at,160723\_at,93117\_at,96696\_at,99151\_at,160503\_at,160531\_at,100577\_at,102409\_at,93008\_at,93999\_at,95049\_at,96029\_at,97200\_f\_at,99182\_at,160426\_at,93551\_at,98081\_at,95479\_at,95480\_at,161147\_f\_at,98075\_at,93548\_at,102412\_at,103319\_at,103654\_at,104476\_at,160659\_at,94506\_at,95132\_r\_at,95460\_at,97164\_at,98516\_at,160324\_at,100331\_g\_at,97819\_at,99583\_at,97758\_at,104423\_at,96052\_at,100128\_at,104080\_at,160538\_at,98595\_at,98934\_at,99522\_at,92636\_f\_at,94014\_at,94210\_at,95441\_at,96668\_at,96670\_at,96734\_at,96849\_at,97477\_at,97478\_at,98959\_at,99156\_at,101061\_at,93970\_at,94323\_at,100089\_at,101207\_at,160416\_at,160456\_at,92829\_at,98153\_at,99546\_at,93101\_s\_at,97460\_at,101440\_at,95015\_at |
| 4 | pigment metabolism | 2 | 23 | 695 | 13100 | 0.003 | 0.002 | 1.636 | 0.347 | 93258\_at,94275\_at |
| 5 | heme metabolism | 2 | 15 | 600 | 11544 | 0.003 | 0.001 | 2.562 | 0.182 | 93258\_at,94275\_at |
| 6 | heme biosynthesis | 2 | 11 | 466 | 9498 | 0.004 | 0.001 | 3.698 | 0.099 | 93258\_at,94275\_at |
| 4 | alcohol metabolism | 10 | 167 | 695 | 13100 | 0.014 | 0.013 | 1.129 | 0.393 | 99566\_at,96268\_at,AFFX-GapdhMur/M32599\_3\_at,AFFX-GapdhMur/M32599\_5\_at,97279\_at,97820\_at,95634\_at,95635\_g\_at,95636\_at,96627\_at |
| 5 | alcohol biosynthesis | 1 | 18 | 600 | 11544 | 0.002 | 0.002 | 1.071 | 0.618 | 99566\_at |
| 6 | monosaccharide biosynthesis | 1 | 18 | 466 | 9498 | 0.002 | 0.002 | 1.132 | 0.596 | 99566\_at |
| 7 | hexose biosynthesis | 1 | 18 | 298 | 6246 | 0.003 | 0.003 | 1.167 | 0.586 | 99566\_at |
| 8 | gluconeogenesis | 1 | 14 | 130 | 2164 | 0.008 | 0.006 | 1.189 | 0.581 | 99566\_at |
| 5 | alcohol catabolism | 5 | 58 | 600 | 11544 | 0.008 | 0.005 | 1.659 | 0.182 | 96268\_at,99566\_at,AFFX-GapdhMur/M32599\_3\_at,AFFX-GapdhMur/M32599\_5\_at,97279\_at |
| 6 | monosaccharide catabolism | 5 | 58 | 466 | 9498 | 0.011 | 0.006 | 1.756 | 0.154 | 96268\_at,99566\_at,AFFX-GapdhMur/M32599\_3\_at,AFFX-GapdhMur/M32599\_5\_at,97279\_at |
| 7 | hexose catabolism | 5 | 58 | 298 | 6246 | 0.017 | 0.009 | 1.806 | 0.142 | 96268\_at,99566\_at,AFFX-GapdhMur/M32599\_3\_at,AFFX-GapdhMur/M32599\_5\_at,97279\_at |
| 8 | glucose catabolism | 5 | 58 | 130 | 2164 | 0.038 | 0.027 | 1.435 | 0.267 | 96268\_at,99566\_at,AFFX-GapdhMur/M32599\_3\_at,AFFX-GapdhMur/M32599\_5\_at,97279\_at |
| 9 | glycolysis | 4 | 52 | 66 | 911 | 0.061 | 0.057 | 1.062 | 0.53 | 96268\_at,99566\_at,AFFX-GapdhMur/M32599\_3\_at,AFFX-GapdhMur/M32599\_5\_at |
| 9 | pentose-phosphate shunt | 2 | 7 | 66 | 911 | 0.03 | 0.008 | 3.945 | 0.086 | 97279\_at,99566\_at |
| 5 | monosaccharide metabolism | 6 | 108 | 600 | 11544 | 0.01 | 0.009 | 1.068 | 0.494 | 99566\_at,96268\_at,AFFX-GapdhMur/M32599\_3\_at,AFFX-GapdhMur/M32599\_5\_at,97279\_at,97820\_at |
| 6 | monosaccharide biosynthesis | 1 | 18 | 466 | 9498 | 0.002 | 0.002 | 1.132 | 0.596 | 99566\_at |
| 7 | hexose biosynthesis | 1 | 18 | 298 | 6246 | 0.003 | 0.003 | 1.167 | 0.586 | 99566\_at |
| 8 | gluconeogenesis | 1 | 14 | 130 | 2164 | 0.008 | 0.006 | 1.189 | 0.581 | 99566\_at |
| 6 | monosaccharide catabolism | 5 | 58 | 466 | 9498 | 0.011 | 0.006 | 1.756 | 0.154 | 96268\_at,99566\_at,AFFX-GapdhMur/M32599\_3\_at,AFFX-GapdhMur/M32599\_5\_at,97279\_at |
| 7 | hexose catabolism | 5 | 58 | 298 | 6246 | 0.017 | 0.009 | 1.806 | 0.142 | 96268\_at,99566\_at,AFFX-GapdhMur/M32599\_3\_at,AFFX-GapdhMur/M32599\_5\_at,97279\_at |
| 8 | glucose catabolism | 5 | 58 | 130 | 2164 | 0.038 | 0.027 | 1.435 | 0.267 | 96268\_at,99566\_at,AFFX-GapdhMur/M32599\_3\_at,AFFX-GapdhMur/M32599\_5\_at,97279\_at |
| 9 | glycolysis | 4 | 52 | 66 | 911 | 0.061 | 0.057 | 1.062 | 0.53 | 96268\_at,99566\_at,AFFX-GapdhMur/M32599\_3\_at,AFFX-GapdhMur/M32599\_5\_at |
| 9 | pentose-phosphate shunt | 2 | 7 | 66 | 911 | 0.03 | 0.008 | 3.945 | 0.086 | 97279\_at,99566\_at |
| 6 | hexose metabolism | 6 | 107 | 466 | 9498 | 0.013 | 0.011 | 1.143 | 0.429 | 99566\_at,96268\_at,AFFX-GapdhMur/M32599\_3\_at,AFFX-GapdhMur/M32599\_5\_at,97279\_at,97820\_at |
| 7 | hexose biosynthesis | 1 | 18 | 298 | 6246 | 0.003 | 0.003 | 1.167 | 0.586 | 99566\_at |
| 8 | gluconeogenesis | 1 | 14 | 130 | 2164 | 0.008 | 0.006 | 1.189 | 0.581 | 99566\_at |
| 7 | hexose catabolism | 5 | 58 | 298 | 6246 | 0.017 | 0.009 | 1.806 | 0.142 | 96268\_at,99566\_at,AFFX-GapdhMur/M32599\_3\_at,AFFX-GapdhMur/M32599\_5\_at,97279\_at |
| 8 | glucose catabolism | 5 | 58 | 130 | 2164 | 0.038 | 0.027 | 1.435 | 0.267 | 96268\_at,99566\_at,AFFX-GapdhMur/M32599\_3\_at,AFFX-GapdhMur/M32599\_5\_at,97279\_at |
| 9 | glycolysis | 4 | 52 | 66 | 911 | 0.061 | 0.057 | 1.062 | 0.53 | 96268\_at,99566\_at,AFFX-GapdhMur/M32599\_3\_at,AFFX-GapdhMur/M32599\_5\_at |
| 9 | pentose-phosphate shunt | 2 | 7 | 66 | 911 | 0.03 | 0.008 | 3.945 | 0.086 | 97279\_at,99566\_at |
| 7 | galactose metabolism | 1 | 13 | 298 | 6246 | 0.003 | 0.002 | 1.615 | 0.471 | 97820\_at |
| 5 | sterol metabolism | 4 | 36 | 600 | 11544 | 0.007 | 0.003 | 2.138 | 0.115 | 95634\_at,95635\_g\_at,95636\_at,96627\_at |
| 6 | cholesterol metabolism | 4 | 33 | 466 | 9498 | 0.009 | 0.003 | 2.473 | 0.076 | 95634\_at,95635\_g\_at,95636\_at,96627\_at |
| 7 | cholesterol biosynthesis | 4 | 17 | 298 | 6246 | 0.013 | 0.003 | 4.934 | 0.007 | 95634\_at,95635\_g\_at,95636\_at,96627\_at |
| 6 | sterol biosynthesis | 4 | 20 | 466 | 9498 | 0.009 | 0.002 | 4.066 | 0.015 | 96627\_at,95634\_at,95635\_g\_at,95636\_at |
| 7 | cholesterol biosynthesis | 4 | 17 | 298 | 6246 | 0.013 | 0.003 | 4.934 | 0.007 | 95634\_at,95635\_g\_at,95636\_at,96627\_at |
| 4 | aldehyde metabolism | 1 | 4 | 695 | 13100 | 0.001 | 0 | 4.645 | 0.196 | 95693\_at |
| 5 | glyoxylate metabolism | 1 | 2 | 600 | 11544 | 0.002 | 0 | 9.824 | 0.101 | 95693\_at |
| 6 | glyoxylate cycle | 1 | 2 | 466 | 9498 | 0.002 | 0 | 10.238 | 0.096 | 95693\_at |
| 4 | amine metabolism | 11 | 148 | 695 | 13100 | 0.016 | 0.011 | 1.401 | 0.163 | 97419\_at,104567\_at,92589\_at,92540\_f\_at,160844\_at,95408\_at,97318\_at,93139\_at,99056\_at,97279\_at,96336\_at |
| 5 | amine biosynthesis | 4 | 35 | 600 | 11544 | 0.007 | 0.003 | 2.201 | 0.106 | 97419\_at,104567\_at,92589\_at,92540\_f\_at |
| 6 | amino acid biosynthesis | 3 | 25 | 466 | 9498 | 0.006 | 0.003 | 2.449 | 0.122 | 97419\_at,104567\_at,92589\_at |
| 7 | aspartate family amino acid biosynthesis | 1 | 6 | 298 | 6246 | 0.003 | 0.001 | 3.5 | 0.254 | 97419\_at |
| 8 | methionine biosynthesis | 1 | 4 | 130 | 2164 | 0.008 | 0.002 | 4.157 | 0.22 | 97419\_at |
| 7 | glutamine family amino acid biosynthesis | 1 | 7 | 298 | 6246 | 0.003 | 0.001 | 3 | 0.29 | 104567\_at |
| 8 | arginine biosynthesis | 1 | 4 | 130 | 2164 | 0.008 | 0.002 | 4.157 | 0.22 | 104567\_at |
| 7 | serine family amino acid biosynthesis | 1 | 8 | 298 | 6246 | 0.003 | 0.001 | 2.625 | 0.324 | 92589\_at |
| 8 | L-serine biosynthesis | 1 | 7 | 130 | 2164 | 0.008 | 0.003 | 2.381 | 0.352 | 92589\_at |
| 6 | biogenic amine biosynthesis | 1 | 10 | 466 | 9498 | 0.002 | 0.001 | 2.048 | 0.395 | 92540\_f\_at |
| 7 | polyamine biosynthesis | 1 | 6 | 298 | 6246 | 0.003 | 0.001 | 3.5 | 0.254 | 92540\_f\_at |
| 8 | spermidine biosynthesis | 1 | 3 | 130 | 2164 | 0.008 | 0.001 | 5.532 | 0.17 | 92540\_f\_at |
| 5 | amine catabolism | 4 | 30 | 600 | 11544 | 0.007 | 0.003 | 2.565 | 0.068 | 160844\_at,95408\_at,97318\_at,104567\_at |
| 6 | amino acid catabolism | 4 | 24 | 466 | 9498 | 0.009 | 0.003 | 3.391 | 0.028 | 160844\_at,95408\_at,97318\_at,104567\_at |
| 7 | aromatic amino acid family catabolism | 2 | 8 | 298 | 6246 | 0.007 | 0.001 | 5.242 | 0.052 | 160844\_at,95408\_at |
| 8 | phenylalanine catabolism | 2 | 6 | 130 | 2164 | 0.015 | 0.003 | 5.552 | 0.046 | 160844\_at,95408\_at |
| 7 | D-amino acid catabolism | 1 | 1 | 298 | 6246 | 0.003 | 0 | 21 | 0.048 | 97318\_at |
| 7 | glutamine family amino acid catabolism | 1 | 7 | 298 | 6246 | 0.003 | 0.001 | 3 | 0.29 | 104567\_at |
| 8 | arginine catabolism | 1 | 6 | 130 | 2164 | 0.008 | 0.003 | 2.776 | 0.311 | 104567\_at |
| 5 | amino acid metabolism | 9 | 97 | 600 | 11544 | 0.015 | 0.008 | 1.786 | 0.065 | 93139\_at,97419\_at,104567\_at,92589\_at,160844\_at,95408\_at,97318\_at,99056\_at,97279\_at |
| 6 | amino acid biosynthesis | 3 | 25 | 466 | 9498 | 0.006 | 0.003 | 2.449 | 0.122 | 97419\_at,104567\_at,92589\_at |
| 7 | aspartate family amino acid biosynthesis | 1 | 6 | 298 | 6246 | 0.003 | 0.001 | 3.5 | 0.254 | 97419\_at |
| 8 | methionine biosynthesis | 1 | 4 | 130 | 2164 | 0.008 | 0.002 | 4.157 | 0.22 | 97419\_at |
| 7 | glutamine family amino acid biosynthesis | 1 | 7 | 298 | 6246 | 0.003 | 0.001 | 3 | 0.29 | 104567\_at |
| 8 | arginine biosynthesis | 1 | 4 | 130 | 2164 | 0.008 | 0.002 | 4.157 | 0.22 | 104567\_at |
| 7 | serine family amino acid biosynthesis | 1 | 8 | 298 | 6246 | 0.003 | 0.001 | 2.625 | 0.324 | 92589\_at |
| 8 | L-serine biosynthesis | 1 | 7 | 130 | 2164 | 0.008 | 0.003 | 2.381 | 0.352 | 92589\_at |
| 6 | amino acid catabolism | 4 | 24 | 466 | 9498 | 0.009 | 0.003 | 3.391 | 0.028 | 160844\_at,95408\_at,97318\_at,104567\_at |
| 7 | aromatic amino acid family catabolism | 2 | 8 | 298 | 6246 | 0.007 | 0.001 | 5.242 | 0.052 | 160844\_at,95408\_at |
| 8 | phenylalanine catabolism | 2 | 6 | 130 | 2164 | 0.015 | 0.003 | 5.552 | 0.046 | 160844\_at,95408\_at |
| 7 | D-amino acid catabolism | 1 | 1 | 298 | 6246 | 0.003 | 0 | 21 | 0.048 | 97318\_at |
| 7 | glutamine family amino acid catabolism | 1 | 7 | 298 | 6246 | 0.003 | 0.001 | 3 | 0.29 | 104567\_at |
| 8 | arginine catabolism | 1 | 6 | 130 | 2164 | 0.008 | 0.003 | 2.776 | 0.311 | 104567\_at |
| 6 | aromatic amino acid family metabolism | 3 | 22 | 466 | 9498 | 0.006 | 0.002 | 2.776 | 0.091 | 95408\_at,160844\_at,99056\_at |
| 7 | aromatic amino acid family catabolism | 2 | 8 | 298 | 6246 | 0.007 | 0.001 | 5.242 | 0.052 | 160844\_at,95408\_at |
| 8 | phenylalanine catabolism | 2 | 6 | 130 | 2164 | 0.015 | 0.003 | 5.552 | 0.046 | 160844\_at,95408\_at |
| 7 | L-phenylalanine metabolism | 3 | 7 | 298 | 6246 | 0.01 | 0.001 | 8.991 | 0.003 | 99056\_at,160844\_at,95408\_at |
| 8 | phenylalanine catabolism | 2 | 6 | 130 | 2164 | 0.015 | 0.003 | 5.552 | 0.046 | 160844\_at,95408\_at |
| 6 | branched chain family amino acid metabolism | 1 | 4 | 466 | 9498 | 0.002 | 0 | 5.119 | 0.182 | 97279\_at |
| 7 | valine metabolism | 1 | 1 | 298 | 6246 | 0.003 | 0 | 21 | 0.048 | 97279\_at |
| 6 | serine family amino acid metabolism | 1 | 12 | 466 | 9498 | 0.002 | 0.001 | 1.706 | 0.453 | 92589\_at |
| 7 | serine family amino acid biosynthesis | 1 | 8 | 298 | 6246 | 0.003 | 0.001 | 2.625 | 0.324 | 92589\_at |
| 8 | L-serine biosynthesis | 1 | 7 | 130 | 2164 | 0.008 | 0.003 | 2.381 | 0.352 | 92589\_at |
| 7 | L-serine metabolism | 1 | 7 | 298 | 6246 | 0.003 | 0.001 | 3 | 0.29 | 92589\_at |
| 8 | L-serine biosynthesis | 1 | 7 | 130 | 2164 | 0.008 | 0.003 | 2.381 | 0.352 | 92589\_at |
| 5 | creatine metabolism | 1 | 3 | 600 | 11544 | 0.002 | 0 | 6.423 | 0.148 | 96336\_at |
| 6 | creatine biosynthesis | 1 | 3 | 466 | 9498 | 0.002 | 0 | 6.719 | 0.14 | 96336\_at |
| 4 | aromatic compound metabolism | 8 | 65 | 695 | 13100 | 0.012 | 0.005 | 2.321 | 0.021 | 95408\_at,160844\_at,99056\_at,97179\_at,96948\_at,103683\_at,161897\_f\_at,160293\_at |
| 5 | aromatic compound biosynthesis | 4 | 7 | 600 | 11544 | 0.007 | 0.001 | 10.934 | 0 | 97179\_at,160844\_at,96948\_at,99056\_at |
| 6 | pteridine and derivative biosynthesis | 4 | 7 | 466 | 9498 | 0.009 | 0.001 | 11.595 | 0 | 97179\_at,160844\_at,96948\_at,99056\_at |
| 7 | Mo-molybdopterin cofactor biosynthesis | 1 | 3 | 298 | 6246 | 0.003 | 0 | 7 | 0.136 | 97179\_at |
| 7 | tetrahydrobiopterin biosynthesis | 3 | 4 | 298 | 6246 | 0.01 | 0.001 | 15.734 | 0 | 160844\_at,96948\_at,99056\_at |
| 5 | nucleobase metabolism | 3 | 12 | 600 | 11544 | 0.005 | 0.001 | 4.808 | 0.022 | 103683\_at,161897\_f\_at,160293\_at |
| 6 | nucleobase biosynthesis | 1 | 8 | 466 | 9498 | 0.002 | 0.001 | 2.56 | 0.331 | 103683\_at |
| 7 | pyrimidine base biosynthesis | 1 | 7 | 298 | 6246 | 0.003 | 0.001 | 3 | 0.29 | 103683\_at |
| 8 | -primede novo-prime pyrimidine base biosynthesis | 1 | 7 | 130 | 2164 | 0.008 | 0.003 | 2.381 | 0.352 | 103683\_at |
| 6 | purine base metabolism | 1 | 4 | 466 | 9498 | 0.002 | 0 | 5.119 | 0.182 | 161897\_f\_at |
| 6 | pyrimidine base metabolism | 2 | 8 | 466 | 9498 | 0.004 | 0.001 | 5.107 | 0.055 | 160293\_at,103683\_at |
| 7 | pyrimidine base biosynthesis | 1 | 7 | 298 | 6246 | 0.003 | 0.001 | 3 | 0.29 | 103683\_at |
| 8 | -primede novo-prime pyrimidine base biosynthesis | 1 | 7 | 130 | 2164 | 0.008 | 0.003 | 2.381 | 0.352 | 103683\_at |
| 4 | biosynthesis | 70 | 652 | 695 | 13100 | 0.101 | 0.05 | 2.024 | 0 | 95634\_at,95635\_g\_at,95636\_at,99566\_at,97419\_at,104567\_at,92589\_at,92540\_f\_at,97179\_at,160844\_at,96948\_at,99056\_at,103683\_at,104147\_at,103334\_at,160125\_at,160126\_at,92798\_at,92799\_g\_at,92800\_i\_at,93596\_i\_at,95656\_i\_at,96611\_at,99128\_at,93014\_at,98910\_at,93582\_at,93258\_at,94275\_at,102194\_at,95760\_at,96909\_at,102970\_at,92388\_at,94276\_at,96627\_at,101097\_at,101680\_at,102019\_at,160431\_at,92578\_at,92646\_at,93062\_at,93579\_at,94252\_at,94494\_at,94870\_f\_at,94912\_at,95067\_at,95498\_at,96291\_f\_at,96292\_r\_at,96293\_at,97342\_at,97751\_f\_at,97824\_at,97884\_at,98120\_at,98524\_f\_at,98904\_at,99594\_at,95677\_at,103881\_at,92565\_at,161897\_f\_at,93236\_s\_at,93237\_s\_at,95497\_at,92625\_at,92824\_at |
| 5 | alcohol biosynthesis | 1 | 18 | 600 | 11544 | 0.002 | 0.002 | 1.071 | 0.618 | 99566\_at |
| 6 | monosaccharide biosynthesis | 1 | 18 | 466 | 9498 | 0.002 | 0.002 | 1.132 | 0.596 | 99566\_at |
| 7 | hexose biosynthesis | 1 | 18 | 298 | 6246 | 0.003 | 0.003 | 1.167 | 0.586 | 99566\_at |
| 8 | gluconeogenesis | 1 | 14 | 130 | 2164 | 0.008 | 0.006 | 1.189 | 0.581 | 99566\_at |
| 5 | amine biosynthesis | 4 | 35 | 600 | 11544 | 0.007 | 0.003 | 2.201 | 0.106 | 97419\_at,104567\_at,92589\_at,92540\_f\_at |
| 6 | amino acid biosynthesis | 3 | 25 | 466 | 9498 | 0.006 | 0.003 | 2.449 | 0.122 | 97419\_at,104567\_at,92589\_at |
| 7 | aspartate family amino acid biosynthesis | 1 | 6 | 298 | 6246 | 0.003 | 0.001 | 3.5 | 0.254 | 97419\_at |
| 8 | methionine biosynthesis | 1 | 4 | 130 | 2164 | 0.008 | 0.002 | 4.157 | 0.22 | 97419\_at |
| 7 | glutamine family amino acid biosynthesis | 1 | 7 | 298 | 6246 | 0.003 | 0.001 | 3 | 0.29 | 104567\_at |
| 8 | arginine biosynthesis | 1 | 4 | 130 | 2164 | 0.008 | 0.002 | 4.157 | 0.22 | 104567\_at |
| 7 | serine family amino acid biosynthesis | 1 | 8 | 298 | 6246 | 0.003 | 0.001 | 2.625 | 0.324 | 92589\_at |
| 8 | L-serine biosynthesis | 1 | 7 | 130 | 2164 | 0.008 | 0.003 | 2.381 | 0.352 | 92589\_at |
| 6 | biogenic amine biosynthesis | 1 | 10 | 466 | 9498 | 0.002 | 0.001 | 2.048 | 0.395 | 92540\_f\_at |
| 7 | polyamine biosynthesis | 1 | 6 | 298 | 6246 | 0.003 | 0.001 | 3.5 | 0.254 | 92540\_f\_at |
| 8 | spermidine biosynthesis | 1 | 3 | 130 | 2164 | 0.008 | 0.001 | 5.532 | 0.17 | 92540\_f\_at |
| 5 | aromatic compound biosynthesis | 4 | 7 | 600 | 11544 | 0.007 | 0.001 | 10.934 | 0 | 97179\_at,160844\_at,96948\_at,99056\_at |
| 6 | pteridine and derivative biosynthesis | 4 | 7 | 466 | 9498 | 0.009 | 0.001 | 11.595 | 0 | 97179\_at,160844\_at,96948\_at,99056\_at |
| 7 | Mo-molybdopterin cofactor biosynthesis | 1 | 3 | 298 | 6246 | 0.003 | 0 | 7 | 0.136 | 97179\_at |
| 7 | tetrahydrobiopterin biosynthesis | 3 | 4 | 298 | 6246 | 0.01 | 0.001 | 15.734 | 0 | 160844\_at,96948\_at,99056\_at |
| 5 | carbohydrate biosynthesis | 3 | 48 | 600 | 11544 | 0.005 | 0.004 | 1.202 | 0.458 | 104147\_at,99566\_at,103334\_at |
| 6 | monosaccharide biosynthesis | 1 | 18 | 466 | 9498 | 0.002 | 0.002 | 1.132 | 0.596 | 99566\_at |
| 7 | hexose biosynthesis | 1 | 18 | 298 | 6246 | 0.003 | 0.003 | 1.167 | 0.586 | 99566\_at |
| 8 | gluconeogenesis | 1 | 14 | 130 | 2164 | 0.008 | 0.006 | 1.189 | 0.581 | 99566\_at |
| 6 | polysaccharide biosynthesis | 1 | 14 | 466 | 9498 | 0.002 | 0.001 | 1.463 | 0.506 | 103334\_at |
| 7 | glucan biosynthesis | 1 | 9 | 298 | 6246 | 0.003 | 0.001 | 2.333 | 0.356 | 103334\_at |
| 8 | glycogen biosynthesis | 1 | 9 | 130 | 2164 | 0.008 | 0.004 | 1.849 | 0.428 | 103334\_at |
| 5 | coenzymes and prosthetic group biosynthesis | 15 | 51 | 600 | 11544 | 0.025 | 0.004 | 5.656 | 0 | 97179\_at,160125\_at,160126\_at,92798\_at,92799\_g\_at,92800\_i\_at,93596\_i\_at,95656\_i\_at,96611\_at,99128\_at,93014\_at,98910\_at,93582\_at,93258\_at,94275\_at |
| 6 | coenzyme biosynthesis | 13 | 40 | 466 | 9498 | 0.028 | 0.004 | 6.627 | 0 | 97179\_at,160125\_at,160126\_at,92798\_at,92799\_g\_at,92800\_i\_at,93596\_i\_at,95656\_i\_at,96611\_at,99128\_at,93014\_at,98910\_at,93582\_at |
| 7 | Mo-molybdopterin cofactor biosynthesis | 1 | 3 | 298 | 6246 | 0.003 | 0 | 7 | 0.136 | 97179\_at |
| 7 | ATP biosynthesis | 10 | 24 | 298 | 6246 | 0.034 | 0.004 | 8.74 | 0 | 160125\_at,160126\_at,92798\_at,92799\_g\_at,92800\_i\_at,93596\_i\_at,95656\_i\_at,96611\_at,99128\_at,93014\_at |
| 8 | ATP synthesis coupled proton transport | 2 | 5 | 130 | 2164 | 0.015 | 0.002 | 6.658 | 0.032 | 93014\_at,95656\_i\_at |
| 7 | lipoic acid biosynthesis | 1 | 1 | 298 | 6246 | 0.003 | 0 | 21 | 0.048 | 98910\_at |
| 8 | lipoate biosynthesis | 1 | 1 | 130 | 2164 | 0.008 | 0 | 16.717 | 0.06 | 98910\_at |
| 7 | quinone cofactor biosynthesis | 1 | 1 | 298 | 6246 | 0.003 | 0 | 21 | 0.048 | 93582\_at |
| 8 | ubiquinone biosynthesis | 1 | 1 | 130 | 2164 | 0.008 | 0 | 16.717 | 0.06 | 93582\_at |
| 6 | porphyrin biosynthesis | 2 | 11 | 466 | 9498 | 0.004 | 0.001 | 3.698 | 0.099 | 93258\_at,94275\_at |
| 6 | heme biosynthesis | 2 | 11 | 466 | 9498 | 0.004 | 0.001 | 3.698 | 0.099 | 93258\_at,94275\_at |
| 5 | lipid biosynthesis | 11 | 124 | 600 | 11544 | 0.018 | 0.011 | 1.707 | 0.058 | 102194\_at,95760\_at,96909\_at,99566\_at,102970\_at,92388\_at,94276\_at,96627\_at,95634\_at,95635\_g\_at,95636\_at |
| 6 | fatty acid biosynthesis | 6 | 40 | 466 | 9498 | 0.013 | 0.004 | 3.059 | 0.013 | 102194\_at,95760\_at,96909\_at,99566\_at,102970\_at,92388\_at |
| 7 | eicosanoid biosynthesis | 2 | 19 | 298 | 6246 | 0.007 | 0.003 | 2.207 | 0.229 | 102970\_at,92388\_at |
| 8 | leukotriene biosynthesis | 1 | 10 | 130 | 2164 | 0.008 | 0.005 | 1.665 | 0.463 | 102970\_at |
| 8 | prostanoid biosynthesis | 1 | 9 | 130 | 2164 | 0.008 | 0.004 | 1.849 | 0.428 | 92388\_at |
| 9 | prostaglandin biosynthesis | 1 | 9 | 66 | 911 | 0.015 | 0.01 | 1.533 | 0.493 | 92388\_at |
| 6 | steroid biosynthesis | 5 | 45 | 466 | 9498 | 0.011 | 0.005 | 2.264 | 0.068 | 94276\_at,96627\_at,95634\_at,95635\_g\_at,95636\_at |
| 7 | cholesterol biosynthesis | 4 | 17 | 298 | 6246 | 0.013 | 0.003 | 4.934 | 0.007 | 95634\_at,95635\_g\_at,95636\_at,96627\_at |
| 6 | sterol biosynthesis | 4 | 20 | 466 | 9498 | 0.009 | 0.002 | 4.066 | 0.015 | 96627\_at,95634\_at,95635\_g\_at,95636\_at |
| 7 | cholesterol biosynthesis | 4 | 17 | 298 | 6246 | 0.013 | 0.003 | 4.934 | 0.007 | 95634\_at,95635\_g\_at,95636\_at,96627\_at |
| 5 | macromolecule biosynthesis | 28 | 322 | 600 | 11544 | 0.047 | 0.028 | 1.673 | 0.005 | 101097\_at,101680\_at,102019\_at,160431\_at,92578\_at,92646\_at,93062\_at,93579\_at,94252\_at,94494\_at,94870\_f\_at,94912\_at,95067\_at,95498\_at,96291\_f\_at,96292\_r\_at,96293\_at,97342\_at,97751\_f\_at,97824\_at,97884\_at,98120\_at,98524\_f\_at,98904\_at,99594\_at,95677\_at,103881\_at,92565\_at |
| 6 | protein biosynthesis | 28 | 322 | 466 | 9498 | 0.06 | 0.034 | 1.773 | 0.002 | 101097\_at,101680\_at,102019\_at,160431\_at,92578\_at,92646\_at,93062\_at,93579\_at,94252\_at,94494\_at,94870\_f\_at,94912\_at,95067\_at,95498\_at,96291\_f\_at,96292\_r\_at,96293\_at,97342\_at,97751\_f\_at,97824\_at,97884\_at,98120\_at,98524\_f\_at,98904\_at,99594\_at,95677\_at,103881\_at,92565\_at |
| 7 | amino acid activation | 2 | 36 | 298 | 6246 | 0.007 | 0.006 | 1.165 | 0.518 | 95677\_at,94494\_at |
| 8 | phenylalanyl-tRNA aminoacylation | 1 | 2 | 130 | 2164 | 0.008 | 0.001 | 8.359 | 0.117 | 94494\_at |
| 7 | lipoprotein biosynthesis | 1 | 16 | 298 | 6246 | 0.003 | 0.003 | 1.312 | 0.543 | 103881\_at |
| 8 | protein lipidation | 1 | 16 | 130 | 2164 | 0.008 | 0.007 | 1.041 | 0.63 | 103881\_at |
| 9 | protein prenylation | 1 | 10 | 66 | 911 | 0.015 | 0.011 | 1.38 | 0.53 | 103881\_at |
| 7 | regulation of protein biosynthesis | 1 | 7 | 298 | 6246 | 0.003 | 0.001 | 3 | 0.29 | 92565\_at |
| 7 | regulation of translation | 1 | 24 | 298 | 6246 | 0.003 | 0.004 | 0.875 | 0.691 | 92565\_at |
| 7 | translational elongation | 1 | 22 | 298 | 6246 | 0.003 | 0.004 | 0.955 | 0.66 | 94252\_at |
| 5 | nucleotide biosynthesis | 17 | 74 | 600 | 11544 | 0.028 | 0.006 | 4.42 | 0 | 161897\_f\_at,93236\_s\_at,93237\_s\_at,95497\_at,160125\_at,160126\_at,92798\_at,92799\_g\_at,92800\_i\_at,93596\_i\_at,95656\_i\_at,96611\_at,99128\_at,93014\_at,92625\_at,92824\_at,103683\_at |
| 6 | deoxyribonucleotide biosynthesis | 2 | 7 | 466 | 9498 | 0.004 | 0.001 | 5.797 | 0.043 | 93236\_s\_at,93237\_s\_at |
| 7 | deoxyribonucleoside monophosphate biosynthesis | 2 | 2 | 298 | 6246 | 0.007 | 0 | 20.969 | 0.002 | 93236\_s\_at,93237\_s\_at |
| 8 | pyrimidine deoxyribonucleoside monophosphate biosynthesis | 2 | 2 | 130 | 2164 | 0.015 | 0.001 | 16.717 | 0.004 | 93236\_s\_at,93237\_s\_at |
| 9 | dTMP biosynthesis | 2 | 2 | 66 | 911 | 0.03 | 0.002 | 13.773 | 0.005 | 93236\_s\_at,93237\_s\_at |
| 6 | nucleoside monophosphate biosynthesis | 3 | 15 | 466 | 9498 | 0.006 | 0.002 | 4.076 | 0.034 | 93236\_s\_at,93237\_s\_at,161897\_f\_at |
| 7 | deoxyribonucleoside monophosphate biosynthesis | 2 | 2 | 298 | 6246 | 0.007 | 0 | 20.969 | 0.002 | 93236\_s\_at,93237\_s\_at |
| 8 | pyrimidine deoxyribonucleoside monophosphate biosynthesis | 2 | 2 | 130 | 2164 | 0.015 | 0.001 | 16.717 | 0.004 | 93236\_s\_at,93237\_s\_at |
| 9 | dTMP biosynthesis | 2 | 2 | 66 | 911 | 0.03 | 0.002 | 13.773 | 0.005 | 93236\_s\_at,93237\_s\_at |
| 7 | ribonucleoside monophosphate biosynthesis | 1 | 13 | 298 | 6246 | 0.003 | 0.002 | 1.615 | 0.471 | 161897\_f\_at |
| 6 | nucleoside triphosphate biosynthesis | 12 | 36 | 466 | 9498 | 0.026 | 0.004 | 6.794 | 0 | 160125\_at,160126\_at,92798\_at,92799\_g\_at,92800\_i\_at,93596\_i\_at,95656\_i\_at,96611\_at,99128\_at,93014\_at,92625\_at,92824\_at |
| 7 | purine nucleoside triphosphate biosynthesis | 12 | 31 | 298 | 6246 | 0.04 | 0.005 | 8.119 | 0 | 160125\_at,160126\_at,92798\_at,92799\_g\_at,92800\_i\_at,93596\_i\_at,95656\_i\_at,96611\_at,99128\_at,93014\_at,92625\_at,92824\_at |
| 8 | purine ribonucleoside triphosphate biosynthesis | 12 | 31 | 130 | 2164 | 0.092 | 0.014 | 6.442 | 0 | 160125\_at,160126\_at,92798\_at,92799\_g\_at,92800\_i\_at,93596\_i\_at,95656\_i\_at,96611\_at,99128\_at,93014\_at,92625\_at,92824\_at |
| 9 | GTP biosynthesis | 2 | 7 | 66 | 911 | 0.03 | 0.008 | 3.945 | 0.086 | 92625\_at,92824\_at |
| 8 | ATP synthesis coupled proton transport | 2 | 5 | 130 | 2164 | 0.015 | 0.002 | 6.658 | 0.032 | 93014\_at,95656\_i\_at |
| 7 | ATP biosynthesis | 10 | 24 | 298 | 6246 | 0.034 | 0.004 | 8.74 | 0 | 160125\_at,160126\_at,92798\_at,92799\_g\_at,92800\_i\_at,93596\_i\_at,95656\_i\_at,96611\_at,99128\_at,93014\_at |
| 8 | ATP synthesis coupled proton transport | 2 | 5 | 130 | 2164 | 0.015 | 0.002 | 6.658 | 0.032 | 93014\_at,95656\_i\_at |
| 7 | ribonucleoside triphosphate biosynthesis | 12 | 31 | 298 | 6246 | 0.04 | 0.005 | 8.119 | 0 | 160125\_at,160126\_at,92798\_at,92799\_g\_at,92800\_i\_at,93596\_i\_at,95656\_i\_at,96611\_at,99128\_at,93014\_at,92625\_at,92824\_at |
| 8 | purine ribonucleoside triphosphate biosynthesis | 12 | 31 | 130 | 2164 | 0.092 | 0.014 | 6.442 | 0 | 160125\_at,160126\_at,92798\_at,92799\_g\_at,92800\_i\_at,93596\_i\_at,95656\_i\_at,96611\_at,99128\_at,93014\_at,92625\_at,92824\_at |
| 9 | GTP biosynthesis | 2 | 7 | 66 | 911 | 0.03 | 0.008 | 3.945 | 0.086 | 92625\_at,92824\_at |
| 8 | ATP synthesis coupled proton transport | 2 | 5 | 130 | 2164 | 0.015 | 0.002 | 6.658 | 0.032 | 93014\_at,95656\_i\_at |
| 8 | pyrimidine ribonucleoside triphosphate biosynthesis | 2 | 7 | 130 | 2164 | 0.015 | 0.003 | 4.762 | 0.062 | 92625\_at,92824\_at |
| 9 | CTP biosynthesis | 2 | 7 | 66 | 911 | 0.03 | 0.008 | 3.945 | 0.086 | 92625\_at,92824\_at |
| 9 | UTP biosynthesis | 2 | 7 | 66 | 911 | 0.03 | 0.008 | 3.945 | 0.086 | 92625\_at,92824\_at |
| 7 | ATP biosynthesis | 10 | 24 | 298 | 6246 | 0.034 | 0.004 | 8.74 | 0 | 160125\_at,160126\_at,92798\_at,92799\_g\_at,92800\_i\_at,93596\_i\_at,95656\_i\_at,96611\_at,99128\_at,93014\_at |
| 8 | ATP synthesis coupled proton transport | 2 | 5 | 130 | 2164 | 0.015 | 0.002 | 6.658 | 0.032 | 93014\_at,95656\_i\_at |
| 6 | pyrimidine nucleotide biosynthesis | 1 | 22 | 466 | 9498 | 0.002 | 0.002 | 0.927 | 0.67 | 103683\_at |
| 4 | carbohydrate metabolism | 14 | 231 | 695 | 13100 | 0.02 | 0.018 | 1.142 | 0.343 | 97538\_at,99566\_at,96268\_at,AFFX-GapdhMur/M32599\_3\_at,AFFX-GapdhMur/M32599\_5\_at,97279\_at,97820\_at,104147\_at,103334\_at,95693\_at,99148\_at,93029\_at,93991\_at,95053\_s\_at |
| 5 | monosaccharide metabolism | 6 | 108 | 600 | 11544 | 0.01 | 0.009 | 1.068 | 0.494 | 99566\_at,96268\_at,AFFX-GapdhMur/M32599\_3\_at,AFFX-GapdhMur/M32599\_5\_at,97279\_at,97820\_at |
| 6 | monosaccharide biosynthesis | 1 | 18 | 466 | 9498 | 0.002 | 0.002 | 1.132 | 0.596 | 99566\_at |
| 7 | hexose biosynthesis | 1 | 18 | 298 | 6246 | 0.003 | 0.003 | 1.167 | 0.586 | 99566\_at |
| 8 | gluconeogenesis | 1 | 14 | 130 | 2164 | 0.008 | 0.006 | 1.189 | 0.581 | 99566\_at |
| 6 | monosaccharide catabolism | 5 | 58 | 466 | 9498 | 0.011 | 0.006 | 1.756 | 0.154 | 96268\_at,99566\_at,AFFX-GapdhMur/M32599\_3\_at,AFFX-GapdhMur/M32599\_5\_at,97279\_at |
| 7 | hexose catabolism | 5 | 58 | 298 | 6246 | 0.017 | 0.009 | 1.806 | 0.142 | 96268\_at,99566\_at,AFFX-GapdhMur/M32599\_3\_at,AFFX-GapdhMur/M32599\_5\_at,97279\_at |
| 8 | glucose catabolism | 5 | 58 | 130 | 2164 | 0.038 | 0.027 | 1.435 | 0.267 | 96268\_at,99566\_at,AFFX-GapdhMur/M32599\_3\_at,AFFX-GapdhMur/M32599\_5\_at,97279\_at |
| 9 | glycolysis | 4 | 52 | 66 | 911 | 0.061 | 0.057 | 1.062 | 0.53 | 96268\_at,99566\_at,AFFX-GapdhMur/M32599\_3\_at,AFFX-GapdhMur/M32599\_5\_at |
| 9 | pentose-phosphate shunt | 2 | 7 | 66 | 911 | 0.03 | 0.008 | 3.945 | 0.086 | 97279\_at,99566\_at |
| 6 | hexose metabolism | 6 | 107 | 466 | 9498 | 0.013 | 0.011 | 1.143 | 0.429 | 99566\_at,96268\_at,AFFX-GapdhMur/M32599\_3\_at,AFFX-GapdhMur/M32599\_5\_at,97279\_at,97820\_at |
| 7 | hexose biosynthesis | 1 | 18 | 298 | 6246 | 0.003 | 0.003 | 1.167 | 0.586 | 99566\_at |
| 8 | gluconeogenesis | 1 | 14 | 130 | 2164 | 0.008 | 0.006 | 1.189 | 0.581 | 99566\_at |
| 7 | hexose catabolism | 5 | 58 | 298 | 6246 | 0.017 | 0.009 | 1.806 | 0.142 | 96268\_at,99566\_at,AFFX-GapdhMur/M32599\_3\_at,AFFX-GapdhMur/M32599\_5\_at,97279\_at |
| 8 | glucose catabolism | 5 | 58 | 130 | 2164 | 0.038 | 0.027 | 1.435 | 0.267 | 96268\_at,99566\_at,AFFX-GapdhMur/M32599\_3\_at,AFFX-GapdhMur/M32599\_5\_at,97279\_at |
| 9 | glycolysis | 4 | 52 | 66 | 911 | 0.061 | 0.057 | 1.062 | 0.53 | 96268\_at,99566\_at,AFFX-GapdhMur/M32599\_3\_at,AFFX-GapdhMur/M32599\_5\_at |
| 9 | pentose-phosphate shunt | 2 | 7 | 66 | 911 | 0.03 | 0.008 | 3.945 | 0.086 | 97279\_at,99566\_at |
| 7 | galactose metabolism | 1 | 13 | 298 | 6246 | 0.003 | 0.002 | 1.615 | 0.471 | 97820\_at |
| 5 | carbohydrate biosynthesis | 3 | 48 | 600 | 11544 | 0.005 | 0.004 | 1.202 | 0.458 | 104147\_at,99566\_at,103334\_at |
| 6 | monosaccharide biosynthesis | 1 | 18 | 466 | 9498 | 0.002 | 0.002 | 1.132 | 0.596 | 99566\_at |
| 7 | hexose biosynthesis | 1 | 18 | 298 | 6246 | 0.003 | 0.003 | 1.167 | 0.586 | 99566\_at |
| 8 | gluconeogenesis | 1 | 14 | 130 | 2164 | 0.008 | 0.006 | 1.189 | 0.581 | 99566\_at |
| 6 | polysaccharide biosynthesis | 1 | 14 | 466 | 9498 | 0.002 | 0.001 | 1.463 | 0.506 | 103334\_at |
| 7 | glucan biosynthesis | 1 | 9 | 298 | 6246 | 0.003 | 0.001 | 2.333 | 0.356 | 103334\_at |
| 8 | glycogen biosynthesis | 1 | 9 | 130 | 2164 | 0.008 | 0.004 | 1.849 | 0.428 | 103334\_at |
| 5 | main pathways of carbohydrate metabolism | 10 | 84 | 600 | 11544 | 0.017 | 0.007 | 2.29 | 0.012 | 95693\_at,99566\_at,96268\_at,AFFX-GapdhMur/M32599\_3\_at,AFFX-GapdhMur/M32599\_5\_at,97279\_at,99148\_at,93029\_at,93991\_at,95053\_s\_at |
| 6 | glyoxylate cycle | 1 | 2 | 466 | 9498 | 0.002 | 0 | 10.238 | 0.096 | 95693\_at |
| 6 | TCA intermediate metabolism | 1 | 4 | 466 | 9498 | 0.002 | 0 | 5.119 | 0.182 | 99148\_at |
| 7 | fumarate metabolism | 1 | 1 | 298 | 6246 | 0.003 | 0 | 21 | 0.048 | 99148\_at |
| 6 | tricarboxylic acid cycle | 6 | 19 | 466 | 9498 | 0.013 | 0.002 | 6.44 | 0 | 93029\_at,93991\_at,95053\_s\_at,95693\_at,96268\_at,99148\_at |
| 4 | catabolism | 44 | 631 | 695 | 13100 | 0.063 | 0.048 | 1.314 | 0.038 | 93542\_at,96268\_at,99566\_at,AFFX-GapdhMur/M32599\_3\_at,AFFX-GapdhMur/M32599\_5\_at,97279\_at,160844\_at,95408\_at,97318\_at,104567\_at,100576\_at,100539\_at,93734\_i\_at,93735\_f\_at,94025\_at,95448\_at,100543\_s\_at,101562\_at,104541\_at,92874\_f\_at,95561\_at,96093\_at,96733\_at,99655\_at,93519\_s\_at,100512\_at,100733\_at,101486\_at,101558\_s\_at,101992\_at,102791\_at,92547\_at,93085\_at,93988\_at,94263\_f\_at,94841\_at,96892\_at,96952\_at,97459\_at,98557\_f\_at,98975\_at,93203\_f\_at,94372\_at,162417\_at |
| 5 | alcohol catabolism | 5 | 58 | 600 | 11544 | 0.008 | 0.005 | 1.659 | 0.182 | 96268\_at,99566\_at,AFFX-GapdhMur/M32599\_3\_at,AFFX-GapdhMur/M32599\_5\_at,97279\_at |
| 6 | monosaccharide catabolism | 5 | 58 | 466 | 9498 | 0.011 | 0.006 | 1.756 | 0.154 | 96268\_at,99566\_at,AFFX-GapdhMur/M32599\_3\_at,AFFX-GapdhMur/M32599\_5\_at,97279\_at |
| 7 | hexose catabolism | 5 | 58 | 298 | 6246 | 0.017 | 0.009 | 1.806 | 0.142 | 96268\_at,99566\_at,AFFX-GapdhMur/M32599\_3\_at,AFFX-GapdhMur/M32599\_5\_at,97279\_at |
| 8 | glucose catabolism | 5 | 58 | 130 | 2164 | 0.038 | 0.027 | 1.435 | 0.267 | 96268\_at,99566\_at,AFFX-GapdhMur/M32599\_3\_at,AFFX-GapdhMur/M32599\_5\_at,97279\_at |
| 9 | glycolysis | 4 | 52 | 66 | 911 | 0.061 | 0.057 | 1.062 | 0.53 | 96268\_at,99566\_at,AFFX-GapdhMur/M32599\_3\_at,AFFX-GapdhMur/M32599\_5\_at |
| 9 | pentose-phosphate shunt | 2 | 7 | 66 | 911 | 0.03 | 0.008 | 3.945 | 0.086 | 97279\_at,99566\_at |
| 5 | amine catabolism | 4 | 30 | 600 | 11544 | 0.007 | 0.003 | 2.565 | 0.068 | 160844\_at,95408\_at,97318\_at,104567\_at |
| 6 | amino acid catabolism | 4 | 24 | 466 | 9498 | 0.009 | 0.003 | 3.391 | 0.028 | 160844\_at,95408\_at,97318\_at,104567\_at |
| 7 | aromatic amino acid family catabolism | 2 | 8 | 298 | 6246 | 0.007 | 0.001 | 5.242 | 0.052 | 160844\_at,95408\_at |
| 8 | phenylalanine catabolism | 2 | 6 | 130 | 2164 | 0.015 | 0.003 | 5.552 | 0.046 | 160844\_at,95408\_at |
| 7 | D-amino acid catabolism | 1 | 1 | 298 | 6246 | 0.003 | 0 | 21 | 0.048 | 97318\_at |
| 7 | glutamine family amino acid catabolism | 1 | 7 | 298 | 6246 | 0.003 | 0.001 | 3 | 0.29 | 104567\_at |
| 8 | arginine catabolism | 1 | 6 | 130 | 2164 | 0.008 | 0.003 | 2.776 | 0.311 | 104567\_at |
| 5 | lipid catabolism | 2 | 44 | 600 | 11544 | 0.003 | 0.004 | 0.874 | 0.675 | 100576\_at,100539\_at |
| 6 | fatty acid catabolism | 1 | 1 | 466 | 9498 | 0.002 | 0 | 19.545 | 0.049 | 100539\_at |
| 5 | macromolecule catabolism | 29 | 470 | 600 | 11544 | 0.048 | 0.041 | 1.187 | 0.192 | 93734\_i\_at,93735\_f\_at,94025\_at,95448\_at,100543\_s\_at,101562\_at,104541\_at,92874\_f\_at,95561\_at,96093\_at,96733\_at,99655\_at,93519\_s\_at,100512\_at,100733\_at,101486\_at,101558\_s\_at,101992\_at,102791\_at,92547\_at,93085\_at,93988\_at,94263\_f\_at,94841\_at,96892\_at,96952\_at,97459\_at,98557\_f\_at,98975\_at |
| 6 | protein catabolism | 29 | 466 | 466 | 9498 | 0.062 | 0.049 | 1.268 | 0.11 | 93734\_i\_at,93735\_f\_at,94025\_at,95448\_at,100543\_s\_at,101562\_at,104541\_at,92874\_f\_at,95561\_at,96093\_at,96733\_at,99655\_at,93519\_s\_at,100512\_at,100733\_at,101486\_at,101558\_s\_at,101992\_at,102791\_at,92547\_at,93085\_at,93988\_at,94263\_f\_at,94841\_at,96892\_at,96952\_at,97459\_at,98557\_f\_at,98975\_at |
| 7 | proteolysis and peptidolysis | 27 | 457 | 298 | 6246 | 0.091 | 0.073 | 1.238 | 0.143 | 100543\_s\_at,101562\_at,104541\_at,92874\_f\_at,95448\_at,95561\_at,96093\_at,96733\_at,99655\_at,93519\_s\_at,100512\_at,100733\_at,101486\_at,101558\_s\_at,101992\_at,102791\_at,92547\_at,93085\_at,93988\_at,94025\_at,94263\_f\_at,94841\_at,96892\_at,96952\_at,97459\_at,98557\_f\_at,98975\_at |
| 8 | modification-dependent protein catabolism | 18 | 122 | 130 | 2164 | 0.138 | 0.056 | 2.456 | 0 | 93519\_s\_at,100512\_at,100733\_at,101486\_at,101558\_s\_at,101992\_at,102791\_at,92547\_at,93085\_at,93988\_at,94025\_at,94263\_f\_at,94841\_at,96892\_at,96952\_at,97459\_at,98557\_f\_at,98975\_at |
| 9 | NEDD8 class-dependent protein catabolism | 1 | 2 | 66 | 911 | 0.015 | 0.002 | 6.886 | 0.14 | 93519\_s\_at |
| 9 | ubiquitin-dependent protein catabolism | 17 | 120 | 66 | 911 | 0.258 | 0.132 | 1.956 | 0.003 | 100512\_at,100733\_at,101486\_at,101558\_s\_at,101992\_at,102791\_at,92547\_at,93085\_at,93988\_at,94025\_at,94263\_f\_at,94841\_at,96892\_at,96952\_at,97459\_at,98557\_f\_at,98975\_at |
| 5 | nucleotide catabolism | 3 | 10 | 600 | 11544 | 0.005 | 0.001 | 5.747 | 0.013 | 93203\_f\_at,94372\_at,162417\_at |
| 6 | deoxyribonucleotide catabolism | 1 | 2 | 466 | 9498 | 0.002 | 0 | 10.238 | 0.096 | 94372\_at |
| 7 | deoxyribonucleoside triphosphate catabolism | 1 | 2 | 298 | 6246 | 0.003 | 0 | 10.5 | 0.093 | 94372\_at |
| 8 | purine deoxyribonucleoside triphosphate catabolism | 1 | 2 | 130 | 2164 | 0.008 | 0.001 | 8.359 | 0.117 | 94372\_at |
| 9 | dGTP catabolism | 1 | 2 | 66 | 911 | 0.015 | 0.002 | 6.886 | 0.14 | 94372\_at |
| 6 | nucleoside diphosphate catabolism | 1 | 6 | 466 | 9498 | 0.002 | 0.001 | 3.413 | 0.261 | 162417\_at |
| 7 | ribonucleoside diphosphate catabolism | 1 | 6 | 298 | 6246 | 0.003 | 0.001 | 3.5 | 0.254 | 162417\_at |
| 4 | coenzymes and prosthetic group metabolism | 16 | 95 | 695 | 13100 | 0.023 | 0.007 | 3.175 | 0 | 97179\_at,160125\_at,160126\_at,92798\_at,92799\_g\_at,92800\_i\_at,93596\_i\_at,95656\_i\_at,96611\_at,99128\_at,93014\_at,98910\_at,93582\_at,93258\_at,94275\_at,103581\_at |
| 5 | coenzymes and prosthetic group biosynthesis | 15 | 51 | 600 | 11544 | 0.025 | 0.004 | 5.656 | 0 | 97179\_at,160125\_at,160126\_at,92798\_at,92799\_g\_at,92800\_i\_at,93596\_i\_at,95656\_i\_at,96611\_at,99128\_at,93014\_at,98910\_at,93582\_at,93258\_at,94275\_at |
| 6 | coenzyme biosynthesis | 13 | 40 | 466 | 9498 | 0.028 | 0.004 | 6.627 | 0 | 97179\_at,160125\_at,160126\_at,92798\_at,92799\_g\_at,92800\_i\_at,93596\_i\_at,95656\_i\_at,96611\_at,99128\_at,93014\_at,98910\_at,93582\_at |
| 7 | Mo-molybdopterin cofactor biosynthesis | 1 | 3 | 298 | 6246 | 0.003 | 0 | 7 | 0.136 | 97179\_at |
| 7 | ATP biosynthesis | 10 | 24 | 298 | 6246 | 0.034 | 0.004 | 8.74 | 0 | 160125\_at,160126\_at,92798\_at,92799\_g\_at,92800\_i\_at,93596\_i\_at,95656\_i\_at,96611\_at,99128\_at,93014\_at |
| 8 | ATP synthesis coupled proton transport | 2 | 5 | 130 | 2164 | 0.015 | 0.002 | 6.658 | 0.032 | 93014\_at,95656\_i\_at |
| 7 | lipoic acid biosynthesis | 1 | 1 | 298 | 6246 | 0.003 | 0 | 21 | 0.048 | 98910\_at |
| 8 | lipoate biosynthesis | 1 | 1 | 130 | 2164 | 0.008 | 0 | 16.717 | 0.06 | 98910\_at |
| 7 | quinone cofactor biosynthesis | 1 | 1 | 298 | 6246 | 0.003 | 0 | 21 | 0.048 | 93582\_at |
| 8 | ubiquinone biosynthesis | 1 | 1 | 130 | 2164 | 0.008 | 0 | 16.717 | 0.06 | 93582\_at |
| 6 | porphyrin biosynthesis | 2 | 11 | 466 | 9498 | 0.004 | 0.001 | 3.698 | 0.099 | 93258\_at,94275\_at |
| 6 | heme biosynthesis | 2 | 11 | 466 | 9498 | 0.004 | 0.001 | 3.698 | 0.099 | 93258\_at,94275\_at |
| 5 | coenzyme metabolism | 14 | 80 | 600 | 11544 | 0.023 | 0.007 | 3.367 | 0 | 97179\_at,160125\_at,160126\_at,92798\_at,92799\_g\_at,92800\_i\_at,93596\_i\_at,95656\_i\_at,96611\_at,99128\_at,93014\_at,98910\_at,93582\_at,103581\_at |
| 6 | coenzyme biosynthesis | 13 | 40 | 466 | 9498 | 0.028 | 0.004 | 6.627 | 0 | 97179\_at,160125\_at,160126\_at,92798\_at,92799\_g\_at,92800\_i\_at,93596\_i\_at,95656\_i\_at,96611\_at,99128\_at,93014\_at,98910\_at,93582\_at |
| 7 | Mo-molybdopterin cofactor biosynthesis | 1 | 3 | 298 | 6246 | 0.003 | 0 | 7 | 0.136 | 97179\_at |
| 7 | ATP biosynthesis | 10 | 24 | 298 | 6246 | 0.034 | 0.004 | 8.74 | 0 | 160125\_at,160126\_at,92798\_at,92799\_g\_at,92800\_i\_at,93596\_i\_at,95656\_i\_at,96611\_at,99128\_at,93014\_at |
| 8 | ATP synthesis coupled proton transport | 2 | 5 | 130 | 2164 | 0.015 | 0.002 | 6.658 | 0.032 | 93014\_at,95656\_i\_at |
| 7 | lipoic acid biosynthesis | 1 | 1 | 298 | 6246 | 0.003 | 0 | 21 | 0.048 | 98910\_at |
| 8 | lipoate biosynthesis | 1 | 1 | 130 | 2164 | 0.008 | 0 | 16.717 | 0.06 | 98910\_at |
| 7 | quinone cofactor biosynthesis | 1 | 1 | 298 | 6246 | 0.003 | 0 | 21 | 0.048 | 93582\_at |
| 8 | ubiquinone biosynthesis | 1 | 1 | 130 | 2164 | 0.008 | 0 | 16.717 | 0.06 | 93582\_at |
| 6 | acyl-CoA metabolism | 1 | NA | 466 | 9498 | 0.002 | NA | NA | NA | 103581\_at |
| 4 | electron transport | 25 | 313 | 695 | 13100 | 0.036 | 0.024 | 1.506 | 0.027 | 100057\_at,100059\_at,100156\_at,100550\_f\_at,100568\_at,103619\_at,103671\_at,92388\_at,93041\_at,93742\_at,93820\_at,95045\_at,95053\_s\_at,95696\_at,96112\_at,96861\_at,96947\_at,97013\_f\_at,98613\_at,99618\_at,100079\_at,94062\_at,96267\_at,96899\_at,96902\_at |
| 5 | ATP synthesis coupled electron transport | 5 | 7 | 600 | 11544 | 0.008 | 0.001 | 13.656 | 0 | 100079\_at,94062\_at,96267\_at,96899\_at,96902\_at |
| 6 | ATP synthesis coupled electron transport (sensu Eukarya) | 5 | 7 | 466 | 9498 | 0.011 | 0.001 | 14.5 | 0 | 100079\_at,94062\_at,96267\_at,96899\_at,96902\_at |
| 7 | mitochondrial electron transport, NADH to ubiquinone | 5 | 6 | 298 | 6246 | 0.017 | 0.001 | 17.479 | 0 | 100079\_at,94062\_at,96267\_at,96899\_at,96902\_at |
| 4 | energy pathways | 13 | 104 | 695 | 13100 | 0.019 | 0.008 | 2.356 | 0.003 | 92798\_at,92799\_g\_at,92800\_i\_at,95693\_at,99566\_at,96268\_at,AFFX-GapdhMur/M32599\_3\_at,AFFX-GapdhMur/M32599\_5\_at,97279\_at,99148\_at,93029\_at,93991\_at,95053\_s\_at |
| 5 | energy derivation by oxidation of organic compounds | 10 | 100 | 600 | 11544 | 0.017 | 0.009 | 1.925 | 0.035 | 95693\_at,99566\_at,96268\_at,AFFX-GapdhMur/M32599\_3\_at,AFFX-GapdhMur/M32599\_5\_at,97279\_at,99148\_at,93029\_at,93991\_at,95053\_s\_at |
| 6 | glyoxylate cycle | 1 | 2 | 466 | 9498 | 0.002 | 0 | 10.238 | 0.096 | 95693\_at |
| 6 | TCA intermediate metabolism | 1 | 4 | 466 | 9498 | 0.002 | 0 | 5.119 | 0.182 | 99148\_at |
| 7 | fumarate metabolism | 1 | 1 | 298 | 6246 | 0.003 | 0 | 21 | 0.048 | 99148\_at |
| 6 | tricarboxylic acid cycle | 6 | 19 | 466 | 9498 | 0.013 | 0.002 | 6.44 | 0 | 93029\_at,93991\_at,95053\_s\_at,95693\_at,96268\_at,99148\_at |
| 6 | cellular respiration | 1 | 1 | 466 | 9498 | 0.002 | 0 | 19.545 | 0.049 | 95053\_s\_at |
| 7 | aerobic respiration | 1 | 1 | 298 | 6246 | 0.003 | 0 | 21 | 0.048 | 95053\_s\_at |
| 5 | main pathways of carbohydrate metabolism | 10 | 84 | 600 | 11544 | 0.017 | 0.007 | 2.29 | 0.012 | 95693\_at,99566\_at,96268\_at,AFFX-GapdhMur/M32599\_3\_at,AFFX-GapdhMur/M32599\_5\_at,97279\_at,99148\_at,93029\_at,93991\_at,95053\_s\_at |
| 6 | glyoxylate cycle | 1 | 2 | 466 | 9498 | 0.002 | 0 | 10.238 | 0.096 | 95693\_at |
| 6 | TCA intermediate metabolism | 1 | 4 | 466 | 9498 | 0.002 | 0 | 5.119 | 0.182 | 99148\_at |
| 7 | fumarate metabolism | 1 | 1 | 298 | 6246 | 0.003 | 0 | 21 | 0.048 | 99148\_at |
| 6 | tricarboxylic acid cycle | 6 | 19 | 466 | 9498 | 0.013 | 0.002 | 6.44 | 0 | 93029\_at,93991\_at,95053\_s\_at,95693\_at,96268\_at,99148\_at |
| 4 | lipid metabolism | 20 | 285 | 695 | 13100 | 0.029 | 0.022 | 1.323 | 0.123 | 102194\_at,95760\_at,96909\_at,99566\_at,102970\_at,92388\_at,94276\_at,96627\_at,95634\_at,95635\_g\_at,95636\_at,100576\_at,100539\_at,93754\_at,95064\_at,95426\_at,95485\_at,98527\_at,103581\_at,99106\_at |
| 5 | lipid biosynthesis | 11 | 124 | 600 | 11544 | 0.018 | 0.011 | 1.707 | 0.058 | 102194\_at,95760\_at,96909\_at,99566\_at,102970\_at,92388\_at,94276\_at,96627\_at,95634\_at,95635\_g\_at,95636\_at |
| 6 | fatty acid biosynthesis | 6 | 40 | 466 | 9498 | 0.013 | 0.004 | 3.059 | 0.013 | 102194\_at,95760\_at,96909\_at,99566\_at,102970\_at,92388\_at |
| 7 | eicosanoid biosynthesis | 2 | 19 | 298 | 6246 | 0.007 | 0.003 | 2.207 | 0.229 | 102970\_at,92388\_at |
| 8 | leukotriene biosynthesis | 1 | 10 | 130 | 2164 | 0.008 | 0.005 | 1.665 | 0.463 | 102970\_at |
| 8 | prostanoid biosynthesis | 1 | 9 | 130 | 2164 | 0.008 | 0.004 | 1.849 | 0.428 | 92388\_at |
| 9 | prostaglandin biosynthesis | 1 | 9 | 66 | 911 | 0.015 | 0.01 | 1.533 | 0.493 | 92388\_at |
| 6 | steroid biosynthesis | 5 | 45 | 466 | 9498 | 0.011 | 0.005 | 2.264 | 0.068 | 94276\_at,96627\_at,95634\_at,95635\_g\_at,95636\_at |
| 7 | cholesterol biosynthesis | 4 | 17 | 298 | 6246 | 0.013 | 0.003 | 4.934 | 0.007 | 95634\_at,95635\_g\_at,95636\_at,96627\_at |
| 6 | sterol biosynthesis | 4 | 20 | 466 | 9498 | 0.009 | 0.002 | 4.066 | 0.015 | 96627\_at,95634\_at,95635\_g\_at,95636\_at |
| 7 | cholesterol biosynthesis | 4 | 17 | 298 | 6246 | 0.013 | 0.003 | 4.934 | 0.007 | 95634\_at,95635\_g\_at,95636\_at,96627\_at |
| 5 | lipid catabolism | 2 | 44 | 600 | 11544 | 0.003 | 0.004 | 0.874 | 0.675 | 100576\_at,100539\_at |
| 6 | fatty acid catabolism | 1 | 1 | 466 | 9498 | 0.002 | 0 | 19.545 | 0.049 | 100539\_at |
| 5 | fatty acid metabolism | 14 | 85 | 600 | 11544 | 0.023 | 0.007 | 3.17 | 0 | 100539\_at,93754\_at,95064\_at,95426\_at,95485\_at,98527\_at,102194\_at,95760\_at,96909\_at,99566\_at,102970\_at,92388\_at,103581\_at,99106\_at |
| 6 | fatty acid biosynthesis | 6 | 40 | 466 | 9498 | 0.013 | 0.004 | 3.059 | 0.013 | 102194\_at,95760\_at,96909\_at,99566\_at,102970\_at,92388\_at |
| 7 | eicosanoid biosynthesis | 2 | 19 | 298 | 6246 | 0.007 | 0.003 | 2.207 | 0.229 | 102970\_at,92388\_at |
| 8 | leukotriene biosynthesis | 1 | 10 | 130 | 2164 | 0.008 | 0.005 | 1.665 | 0.463 | 102970\_at |
| 8 | prostanoid biosynthesis | 1 | 9 | 130 | 2164 | 0.008 | 0.004 | 1.849 | 0.428 | 92388\_at |
| 9 | prostaglandin biosynthesis | 1 | 9 | 66 | 911 | 0.015 | 0.01 | 1.533 | 0.493 | 92388\_at |
| 6 | fatty acid catabolism | 1 | 1 | 466 | 9498 | 0.002 | 0 | 19.545 | 0.049 | 100539\_at |
| 6 | acyl-CoA metabolism | 1 | NA | 466 | 9498 | 0.002 | NA | NA | NA | 103581\_at |
| 6 | fatty acid oxidation | 1 | 5 | 466 | 9498 | 0.002 | 0.001 | 4.057 | 0.222 | 99106\_at |
| 7 | fatty acid beta-oxidation | 1 | 4 | 298 | 6246 | 0.003 | 0.001 | 5.25 | 0.178 | 99106\_at |
| 6 | long-chain fatty acid metabolism | 1 | 1 | 466 | 9498 | 0.002 | 0 | 19.545 | 0.049 | 103581\_at |
| 4 | nitrogen metabolism | 2 | 20 | 695 | 13100 | 0.003 | 0.002 | 1.882 | 0.287 | 160135\_at,104567\_at |
| 5 | urea cycle | 1 | 7 | 600 | 11544 | 0.002 | 0.001 | 2.738 | 0.312 | 104567\_at |
| 4 | nucleobase, nucleoside, nucleotide and nucleic acid metabolism | 87 | 1530 | 695 | 13100 | 0.125 | 0.117 | 1.072 | 0.256 | 94034\_at,96289\_at,99544\_at,103683\_at,161897\_f\_at,160293\_at,101254\_at,102631\_at,96081\_at,100156\_at,100612\_at,101065\_at,101067\_at,102001\_at,103418\_at,104738\_at,93041\_at,93112\_at,98975\_at,101105\_at,93095\_at,93251\_at,96699\_at,98039\_at,94897\_at,96775\_at,101954\_at,93833\_s\_at,98587\_at,95660\_at,100459\_at,102853\_at,93559\_at,160107\_at,95497\_at,93236\_s\_at,93237\_s\_at,160125\_at,160126\_at,92798\_at,92799\_g\_at,92800\_i\_at,93596\_i\_at,95656\_i\_at,96611\_at,99128\_at,93014\_at,92625\_at,92824\_at,93203\_f\_at,94372\_at,162417\_at,160723\_at,93117\_at,96696\_at,99151\_at,160503\_at,160531\_at,100577\_at,102409\_at,93008\_at,93999\_at,95049\_at,96029\_at,97200\_f\_at,99182\_at,160426\_at,92565\_at,93551\_at,98081\_at,95479\_at,95480\_at,161147\_f\_at,98075\_at,93548\_at,102412\_at,103319\_at,103654\_at,104476\_at,160659\_at,94506\_at,95132\_r\_at,95460\_at,97164\_at,98516\_at,160324\_at,93519\_s\_at |
| 5 | nucleobase metabolism | 3 | 12 | 600 | 11544 | 0.005 | 0.001 | 4.808 | 0.022 | 103683\_at,161897\_f\_at,160293\_at |
| 6 | nucleobase biosynthesis | 1 | 8 | 466 | 9498 | 0.002 | 0.001 | 2.56 | 0.331 | 103683\_at |
| 7 | pyrimidine base biosynthesis | 1 | 7 | 298 | 6246 | 0.003 | 0.001 | 3 | 0.29 | 103683\_at |
| 8 | -primede novo-prime pyrimidine base biosynthesis | 1 | 7 | 130 | 2164 | 0.008 | 0.003 | 2.381 | 0.352 | 103683\_at |
| 6 | purine base metabolism | 1 | 4 | 466 | 9498 | 0.002 | 0 | 5.119 | 0.182 | 161897\_f\_at |
| 6 | pyrimidine base metabolism | 2 | 8 | 466 | 9498 | 0.004 | 0.001 | 5.107 | 0.055 | 160293\_at,103683\_at |
| 7 | pyrimidine base biosynthesis | 1 | 7 | 298 | 6246 | 0.003 | 0.001 | 3 | 0.29 | 103683\_at |
| 8 | -primede novo-prime pyrimidine base biosynthesis | 1 | 7 | 130 | 2164 | 0.008 | 0.003 | 2.381 | 0.352 | 103683\_at |
| 5 | DNA metabolism | 28 | 302 | 600 | 11544 | 0.047 | 0.026 | 1.784 | 0.002 | 101254\_at,102631\_at,96081\_at,100156\_at,100612\_at,101065\_at,101067\_at,102001\_at,103418\_at,104738\_at,93041\_at,93112\_at,96289\_at,98975\_at,101105\_at,93095\_at,93251\_at,96699\_at,98039\_at,94897\_at,96775\_at,101954\_at,93833\_s\_at,98587\_at,95660\_at,100459\_at,102853\_at,93559\_at |
| 6 | DNA integration | 1 | 3 | 466 | 9498 | 0.002 | 0 | 6.719 | 0.14 | 101105\_at |
| 6 | DNA packaging | 11 | 93 | 466 | 9498 | 0.024 | 0.01 | 2.412 | 0.006 | 93095\_at,93251\_at,96699\_at,98039\_at,94897\_at,96775\_at,101954\_at,93112\_at,93833\_s\_at,98587\_at,95660\_at |
| 7 | establishment and/or maintenance of chromatin architecture | 7 | 80 | 298 | 6246 | 0.023 | 0.013 | 1.834 | 0.086 | 94897\_at,96775\_at,101954\_at,93112\_at,93833\_s\_at,98587\_at,95660\_at |
| 8 | chromatin assembly/disassembly | 5 | 48 | 130 | 2164 | 0.038 | 0.022 | 1.734 | 0.158 | 96775\_at,101954\_at,93112\_at,93833\_s\_at,98587\_at |
| 9 | nucleosome assembly | 4 | 28 | 66 | 911 | 0.061 | 0.031 | 1.972 | 0.138 | 101954\_at,93112\_at,93833\_s\_at,98587\_at |
| 8 | chromatin modification | 1 | 36 | 130 | 2164 | 0.008 | 0.017 | 0.462 | 0.895 | 95660\_at |
| 9 | non-covalent chromatin modification | 1 | 13 | 66 | 911 | 0.015 | 0.014 | 1.062 | 0.626 | 95660\_at |
| 10 | chromatin modeling | 1 | 13 | 5 | 197 | 0.2 | 0.066 | 3.031 | 0.292 | 95660\_at |
| 6 | DNA recombination | 1 | 25 | 466 | 9498 | 0.002 | 0.003 | 0.817 | 0.716 | 101105\_at |
| 7 | provirus integration | 1 | 1 | 298 | 6246 | 0.003 | 0 | 21 | 0.048 | 101105\_at |
| 6 | DNA repair | 3 | 99 | 466 | 9498 | 0.006 | 0.01 | 0.618 | 0.871 | 100459\_at,102853\_at,93559\_at |
| 5 | nucleoside metabolism | 3 | 15 | 600 | 11544 | 0.005 | 0.001 | 3.846 | 0.04 | 160107\_at,161897\_f\_at,95497\_at |
| 5 | nucleotide metabolism | 24 | 95 | 600 | 11544 | 0.04 | 0.008 | 4.86 | 0 | 94034\_at,96289\_at,161897\_f\_at,93236\_s\_at,93237\_s\_at,95497\_at,160125\_at,160126\_at,92798\_at,92799\_g\_at,92800\_i\_at,93596\_i\_at,95656\_i\_at,96611\_at,99128\_at,93014\_at,92625\_at,92824\_at,103683\_at,93203\_f\_at,94372\_at,162417\_at,102001\_at,160107\_at |
| 6 | deoxyribonucleotide biosynthesis | 2 | 7 | 466 | 9498 | 0.004 | 0.001 | 5.797 | 0.043 | 93236\_s\_at,93237\_s\_at |
| 7 | deoxyribonucleoside monophosphate biosynthesis | 2 | 2 | 298 | 6246 | 0.007 | 0 | 20.969 | 0.002 | 93236\_s\_at,93237\_s\_at |
| 8 | pyrimidine deoxyribonucleoside monophosphate biosynthesis | 2 | 2 | 130 | 2164 | 0.015 | 0.001 | 16.717 | 0.004 | 93236\_s\_at,93237\_s\_at |
| 9 | dTMP biosynthesis | 2 | 2 | 66 | 911 | 0.03 | 0.002 | 13.773 | 0.005 | 93236\_s\_at,93237\_s\_at |
| 6 | nucleoside monophosphate biosynthesis | 3 | 15 | 466 | 9498 | 0.006 | 0.002 | 4.076 | 0.034 | 93236\_s\_at,93237\_s\_at,161897\_f\_at |
| 7 | deoxyribonucleoside monophosphate biosynthesis | 2 | 2 | 298 | 6246 | 0.007 | 0 | 20.969 | 0.002 | 93236\_s\_at,93237\_s\_at |
| 8 | pyrimidine deoxyribonucleoside monophosphate biosynthesis | 2 | 2 | 130 | 2164 | 0.015 | 0.001 | 16.717 | 0.004 | 93236\_s\_at,93237\_s\_at |
| 9 | dTMP biosynthesis | 2 | 2 | 66 | 911 | 0.03 | 0.002 | 13.773 | 0.005 | 93236\_s\_at,93237\_s\_at |
| 7 | ribonucleoside monophosphate biosynthesis | 1 | 13 | 298 | 6246 | 0.003 | 0.002 | 1.615 | 0.471 | 161897\_f\_at |
| 6 | nucleoside triphosphate biosynthesis | 12 | 36 | 466 | 9498 | 0.026 | 0.004 | 6.794 | 0 | 160125\_at,160126\_at,92798\_at,92799\_g\_at,92800\_i\_at,93596\_i\_at,95656\_i\_at,96611\_at,99128\_at,93014\_at,92625\_at,92824\_at |
| 7 | purine nucleoside triphosphate biosynthesis | 12 | 31 | 298 | 6246 | 0.04 | 0.005 | 8.119 | 0 | 160125\_at,160126\_at,92798\_at,92799\_g\_at,92800\_i\_at,93596\_i\_at,95656\_i\_at,96611\_at,99128\_at,93014\_at,92625\_at,92824\_at |
| 8 | purine ribonucleoside triphosphate biosynthesis | 12 | 31 | 130 | 2164 | 0.092 | 0.014 | 6.442 | 0 | 160125\_at,160126\_at,92798\_at,92799\_g\_at,92800\_i\_at,93596\_i\_at,95656\_i\_at,96611\_at,99128\_at,93014\_at,92625\_at,92824\_at |
| 9 | GTP biosynthesis | 2 | 7 | 66 | 911 | 0.03 | 0.008 | 3.945 | 0.086 | 92625\_at,92824\_at |
| 8 | ATP synthesis coupled proton transport | 2 | 5 | 130 | 2164 | 0.015 | 0.002 | 6.658 | 0.032 | 93014\_at,95656\_i\_at |
| 7 | ATP biosynthesis | 10 | 24 | 298 | 6246 | 0.034 | 0.004 | 8.74 | 0 | 160125\_at,160126\_at,92798\_at,92799\_g\_at,92800\_i\_at,93596\_i\_at,95656\_i\_at,96611\_at,99128\_at,93014\_at |
| 8 | ATP synthesis coupled proton transport | 2 | 5 | 130 | 2164 | 0.015 | 0.002 | 6.658 | 0.032 | 93014\_at,95656\_i\_at |
| 7 | ribonucleoside triphosphate biosynthesis | 12 | 31 | 298 | 6246 | 0.04 | 0.005 | 8.119 | 0 | 160125\_at,160126\_at,92798\_at,92799\_g\_at,92800\_i\_at,93596\_i\_at,95656\_i\_at,96611\_at,99128\_at,93014\_at,92625\_at,92824\_at |
| 8 | purine ribonucleoside triphosphate biosynthesis | 12 | 31 | 130 | 2164 | 0.092 | 0.014 | 6.442 | 0 | 160125\_at,160126\_at,92798\_at,92799\_g\_at,92800\_i\_at,93596\_i\_at,95656\_i\_at,96611\_at,99128\_at,93014\_at,92625\_at,92824\_at |
| 9 | GTP biosynthesis | 2 | 7 | 66 | 911 | 0.03 | 0.008 | 3.945 | 0.086 | 92625\_at,92824\_at |
| 8 | ATP synthesis coupled proton transport | 2 | 5 | 130 | 2164 | 0.015 | 0.002 | 6.658 | 0.032 | 93014\_at,95656\_i\_at |
| 8 | pyrimidine ribonucleoside triphosphate biosynthesis | 2 | 7 | 130 | 2164 | 0.015 | 0.003 | 4.762 | 0.062 | 92625\_at,92824\_at |
| 9 | CTP biosynthesis | 2 | 7 | 66 | 911 | 0.03 | 0.008 | 3.945 | 0.086 | 92625\_at,92824\_at |
| 9 | UTP biosynthesis | 2 | 7 | 66 | 911 | 0.03 | 0.008 | 3.945 | 0.086 | 92625\_at,92824\_at |
| 7 | ATP biosynthesis | 10 | 24 | 298 | 6246 | 0.034 | 0.004 | 8.74 | 0 | 160125\_at,160126\_at,92798\_at,92799\_g\_at,92800\_i\_at,93596\_i\_at,95656\_i\_at,96611\_at,99128\_at,93014\_at |
| 8 | ATP synthesis coupled proton transport | 2 | 5 | 130 | 2164 | 0.015 | 0.002 | 6.658 | 0.032 | 93014\_at,95656\_i\_at |
| 6 | pyrimidine nucleotide biosynthesis | 1 | 22 | 466 | 9498 | 0.002 | 0.002 | 0.927 | 0.67 | 103683\_at |
| 6 | deoxyribonucleotide catabolism | 1 | 2 | 466 | 9498 | 0.002 | 0 | 10.238 | 0.096 | 94372\_at |
| 7 | deoxyribonucleoside triphosphate catabolism | 1 | 2 | 298 | 6246 | 0.003 | 0 | 10.5 | 0.093 | 94372\_at |
| 8 | purine deoxyribonucleoside triphosphate catabolism | 1 | 2 | 130 | 2164 | 0.008 | 0.001 | 8.359 | 0.117 | 94372\_at |
| 9 | dGTP catabolism | 1 | 2 | 66 | 911 | 0.015 | 0.002 | 6.886 | 0.14 | 94372\_at |
| 6 | nucleoside diphosphate catabolism | 1 | 6 | 466 | 9498 | 0.002 | 0.001 | 3.413 | 0.261 | 162417\_at |
| 7 | ribonucleoside diphosphate catabolism | 1 | 6 | 298 | 6246 | 0.003 | 0.001 | 3.5 | 0.254 | 162417\_at |
| 6 | deoxyribonucleotide metabolism | 4 | 11 | 466 | 9498 | 0.009 | 0.001 | 7.397 | 0.001 | 93236\_s\_at,93237\_s\_at,94372\_at,102001\_at |
| 7 | deoxyribonucleoside monophosphate biosynthesis | 2 | 2 | 298 | 6246 | 0.007 | 0 | 20.969 | 0.002 | 93236\_s\_at,93237\_s\_at |
| 8 | pyrimidine deoxyribonucleoside monophosphate biosynthesis | 2 | 2 | 130 | 2164 | 0.015 | 0.001 | 16.717 | 0.004 | 93236\_s\_at,93237\_s\_at |
| 9 | dTMP biosynthesis | 2 | 2 | 66 | 911 | 0.03 | 0.002 | 13.773 | 0.005 | 93236\_s\_at,93237\_s\_at |
| 7 | deoxyribonucleoside triphosphate catabolism | 1 | 2 | 298 | 6246 | 0.003 | 0 | 10.5 | 0.093 | 94372\_at |
| 8 | purine deoxyribonucleoside triphosphate catabolism | 1 | 2 | 130 | 2164 | 0.008 | 0.001 | 8.359 | 0.117 | 94372\_at |
| 9 | dGTP catabolism | 1 | 2 | 66 | 911 | 0.015 | 0.002 | 6.886 | 0.14 | 94372\_at |
| 7 | deoxyribonucleoside diphosphate metabolism | 1 | 6 | 298 | 6246 | 0.003 | 0.001 | 3.5 | 0.254 | 102001\_at |
| 6 | deoxyribonucleotide biosynthesis | 2 | 7 | 466 | 9498 | 0.004 | 0.001 | 5.797 | 0.043 | 93236\_s\_at,93237\_s\_at |
| 7 | deoxyribonucleoside monophosphate biosynthesis | 2 | 2 | 298 | 6246 | 0.007 | 0 | 20.969 | 0.002 | 93236\_s\_at,93237\_s\_at |
| 8 | pyrimidine deoxyribonucleoside monophosphate biosynthesis | 2 | 2 | 130 | 2164 | 0.015 | 0.001 | 16.717 | 0.004 | 93236\_s\_at,93237\_s\_at |
| 9 | dTMP biosynthesis | 2 | 2 | 66 | 911 | 0.03 | 0.002 | 13.773 | 0.005 | 93236\_s\_at,93237\_s\_at |
| 6 | deoxyribonucleotide catabolism | 1 | 2 | 466 | 9498 | 0.002 | 0 | 10.238 | 0.096 | 94372\_at |
| 7 | deoxyribonucleoside triphosphate catabolism | 1 | 2 | 298 | 6246 | 0.003 | 0 | 10.5 | 0.093 | 94372\_at |
| 8 | purine deoxyribonucleoside triphosphate catabolism | 1 | 2 | 130 | 2164 | 0.008 | 0.001 | 8.359 | 0.117 | 94372\_at |
| 9 | dGTP catabolism | 1 | 2 | 66 | 911 | 0.015 | 0.002 | 6.886 | 0.14 | 94372\_at |
| 6 | purine nucleotide metabolism | 1 | 54 | 466 | 9498 | 0.002 | 0.006 | 0.378 | 0.934 | 160107\_at |
| 7 | purine salvage | 1 | 5 | 298 | 6246 | 0.003 | 0.001 | 4.2 | 0.217 | 160107\_at |
| 5 | nucleotide biosynthesis | 17 | 74 | 600 | 11544 | 0.028 | 0.006 | 4.42 | 0 | 161897\_f\_at,93236\_s\_at,93237\_s\_at,95497\_at,160125\_at,160126\_at,92798\_at,92799\_g\_at,92800\_i\_at,93596\_i\_at,95656\_i\_at,96611\_at,99128\_at,93014\_at,92625\_at,92824\_at,103683\_at |
| 6 | deoxyribonucleotide biosynthesis | 2 | 7 | 466 | 9498 | 0.004 | 0.001 | 5.797 | 0.043 | 93236\_s\_at,93237\_s\_at |
| 7 | deoxyribonucleoside monophosphate biosynthesis | 2 | 2 | 298 | 6246 | 0.007 | 0 | 20.969 | 0.002 | 93236\_s\_at,93237\_s\_at |
| 8 | pyrimidine deoxyribonucleoside monophosphate biosynthesis | 2 | 2 | 130 | 2164 | 0.015 | 0.001 | 16.717 | 0.004 | 93236\_s\_at,93237\_s\_at |
| 9 | dTMP biosynthesis | 2 | 2 | 66 | 911 | 0.03 | 0.002 | 13.773 | 0.005 | 93236\_s\_at,93237\_s\_at |
| 6 | nucleoside monophosphate biosynthesis | 3 | 15 | 466 | 9498 | 0.006 | 0.002 | 4.076 | 0.034 | 93236\_s\_at,93237\_s\_at,161897\_f\_at |
| 7 | deoxyribonucleoside monophosphate biosynthesis | 2 | 2 | 298 | 6246 | 0.007 | 0 | 20.969 | 0.002 | 93236\_s\_at,93237\_s\_at |
| 8 | pyrimidine deoxyribonucleoside monophosphate biosynthesis | 2 | 2 | 130 | 2164 | 0.015 | 0.001 | 16.717 | 0.004 | 93236\_s\_at,93237\_s\_at |
| 9 | dTMP biosynthesis | 2 | 2 | 66 | 911 | 0.03 | 0.002 | 13.773 | 0.005 | 93236\_s\_at,93237\_s\_at |
| 7 | ribonucleoside monophosphate biosynthesis | 1 | 13 | 298 | 6246 | 0.003 | 0.002 | 1.615 | 0.471 | 161897\_f\_at |
| 6 | nucleoside triphosphate biosynthesis | 12 | 36 | 466 | 9498 | 0.026 | 0.004 | 6.794 | 0 | 160125\_at,160126\_at,92798\_at,92799\_g\_at,92800\_i\_at,93596\_i\_at,95656\_i\_at,96611\_at,99128\_at,93014\_at,92625\_at,92824\_at |
| 7 | purine nucleoside triphosphate biosynthesis | 12 | 31 | 298 | 6246 | 0.04 | 0.005 | 8.119 | 0 | 160125\_at,160126\_at,92798\_at,92799\_g\_at,92800\_i\_at,93596\_i\_at,95656\_i\_at,96611\_at,99128\_at,93014\_at,92625\_at,92824\_at |
| 8 | purine ribonucleoside triphosphate biosynthesis | 12 | 31 | 130 | 2164 | 0.092 | 0.014 | 6.442 | 0 | 160125\_at,160126\_at,92798\_at,92799\_g\_at,92800\_i\_at,93596\_i\_at,95656\_i\_at,96611\_at,99128\_at,93014\_at,92625\_at,92824\_at |
| 9 | GTP biosynthesis | 2 | 7 | 66 | 911 | 0.03 | 0.008 | 3.945 | 0.086 | 92625\_at,92824\_at |
| 8 | ATP synthesis coupled proton transport | 2 | 5 | 130 | 2164 | 0.015 | 0.002 | 6.658 | 0.032 | 93014\_at,95656\_i\_at |
| 7 | ATP biosynthesis | 10 | 24 | 298 | 6246 | 0.034 | 0.004 | 8.74 | 0 | 160125\_at,160126\_at,92798\_at,92799\_g\_at,92800\_i\_at,93596\_i\_at,95656\_i\_at,96611\_at,99128\_at,93014\_at |
| 8 | ATP synthesis coupled proton transport | 2 | 5 | 130 | 2164 | 0.015 | 0.002 | 6.658 | 0.032 | 93014\_at,95656\_i\_at |
| 7 | ribonucleoside triphosphate biosynthesis | 12 | 31 | 298 | 6246 | 0.04 | 0.005 | 8.119 | 0 | 160125\_at,160126\_at,92798\_at,92799\_g\_at,92800\_i\_at,93596\_i\_at,95656\_i\_at,96611\_at,99128\_at,93014\_at,92625\_at,92824\_at |
| 8 | purine ribonucleoside triphosphate biosynthesis | 12 | 31 | 130 | 2164 | 0.092 | 0.014 | 6.442 | 0 | 160125\_at,160126\_at,92798\_at,92799\_g\_at,92800\_i\_at,93596\_i\_at,95656\_i\_at,96611\_at,99128\_at,93014\_at,92625\_at,92824\_at |
| 9 | GTP biosynthesis | 2 | 7 | 66 | 911 | 0.03 | 0.008 | 3.945 | 0.086 | 92625\_at,92824\_at |
| 8 | ATP synthesis coupled proton transport | 2 | 5 | 130 | 2164 | 0.015 | 0.002 | 6.658 | 0.032 | 93014\_at,95656\_i\_at |
| 8 | pyrimidine ribonucleoside triphosphate biosynthesis | 2 | 7 | 130 | 2164 | 0.015 | 0.003 | 4.762 | 0.062 | 92625\_at,92824\_at |
| 9 | CTP biosynthesis | 2 | 7 | 66 | 911 | 0.03 | 0.008 | 3.945 | 0.086 | 92625\_at,92824\_at |
| 9 | UTP biosynthesis | 2 | 7 | 66 | 911 | 0.03 | 0.008 | 3.945 | 0.086 | 92625\_at,92824\_at |
| 7 | ATP biosynthesis | 10 | 24 | 298 | 6246 | 0.034 | 0.004 | 8.74 | 0 | 160125\_at,160126\_at,92798\_at,92799\_g\_at,92800\_i\_at,93596\_i\_at,95656\_i\_at,96611\_at,99128\_at,93014\_at |
| 8 | ATP synthesis coupled proton transport | 2 | 5 | 130 | 2164 | 0.015 | 0.002 | 6.658 | 0.032 | 93014\_at,95656\_i\_at |
| 6 | pyrimidine nucleotide biosynthesis | 1 | 22 | 466 | 9498 | 0.002 | 0.002 | 0.927 | 0.67 | 103683\_at |
| 5 | nucleotide catabolism | 3 | 10 | 600 | 11544 | 0.005 | 0.001 | 5.747 | 0.013 | 93203\_f\_at,94372\_at,162417\_at |
| 6 | deoxyribonucleotide catabolism | 1 | 2 | 466 | 9498 | 0.002 | 0 | 10.238 | 0.096 | 94372\_at |
| 7 | deoxyribonucleoside triphosphate catabolism | 1 | 2 | 298 | 6246 | 0.003 | 0 | 10.5 | 0.093 | 94372\_at |
| 8 | purine deoxyribonucleoside triphosphate catabolism | 1 | 2 | 130 | 2164 | 0.008 | 0.001 | 8.359 | 0.117 | 94372\_at |
| 9 | dGTP catabolism | 1 | 2 | 66 | 911 | 0.015 | 0.002 | 6.886 | 0.14 | 94372\_at |
| 6 | nucleoside diphosphate catabolism | 1 | 6 | 466 | 9498 | 0.002 | 0.001 | 3.413 | 0.261 | 162417\_at |
| 7 | ribonucleoside diphosphate catabolism | 1 | 6 | 298 | 6246 | 0.003 | 0.001 | 3.5 | 0.254 | 162417\_at |
| 5 | RNA metabolism | 15 | 132 | 600 | 11544 | 0.025 | 0.011 | 2.187 | 0.004 | 160723\_at,93117\_at,96696\_at,99151\_at,160503\_at,160531\_at,100577\_at,102409\_at,93008\_at,93999\_at,95049\_at,96029\_at,97200\_f\_at,99182\_at,96289\_at |
| 6 | RNA processing | 14 | 126 | 466 | 9498 | 0.03 | 0.013 | 2.264 | 0.003 | 160723\_at,93117\_at,96696\_at,99151\_at,160503\_at,160531\_at,100577\_at,102409\_at,93008\_at,93999\_at,95049\_at,96029\_at,97200\_f\_at,99182\_at |
| 7 | mRNA processing | 7 | 84 | 298 | 6246 | 0.023 | 0.013 | 1.746 | 0.105 | 100577\_at,102409\_at,93008\_at,93999\_at,95049\_at,96029\_at,97200\_f\_at |
| 8 | nuclear mRNA splicing, via spliceosome | 2 | NA | 130 | 2164 | 0.015 | NA | NA | NA | 100577\_at,95049\_at |
| 9 | spliceosome assembly | 2 | 19 | 66 | 911 | 0.03 | 0.021 | 1.453 | 0.406 | 100577\_at,95049\_at |
| 7 | tRNA processing | 1 | 6 | 298 | 6246 | 0.003 | 0.001 | 3.5 | 0.254 | 99182\_at |
| 6 | tRNA metabolism | 2 | 7 | 466 | 9498 | 0.004 | 0.001 | 5.797 | 0.043 | 96289\_at,99182\_at |
| 7 | tRNA processing | 1 | 6 | 298 | 6246 | 0.003 | 0.001 | 3.5 | 0.254 | 99182\_at |
| 5 | transcription | 27 | 1086 | 600 | 11544 | 0.045 | 0.094 | 0.478 | 1 | 160426\_at,92565\_at,93551\_at,98081\_at,95479\_at,95480\_at,161147\_f\_at,98075\_at,93548\_at,100156\_at,102412\_at,103319\_at,103654\_at,104476\_at,160659\_at,93008\_at,93041\_at,93112\_at,93251\_at,94506\_at,95132\_r\_at,95460\_at,97164\_at,98039\_at,98516\_at,160324\_at,93519\_s\_at |
| 6 | regulation of transcription | 25 | 1026 | 466 | 9498 | 0.054 | 0.108 | 0.497 | 1 | 95479\_at,95480\_at,161147\_f\_at,98075\_at,93548\_at,100156\_at,102412\_at,103319\_at,103654\_at,104476\_at,160659\_at,92565\_at,93008\_at,93041\_at,93112\_at,93251\_at,94506\_at,95132\_r\_at,95460\_at,97164\_at,98039\_at,98516\_at,93551\_at,160324\_at,93519\_s\_at |
| 7 | negative regulation of transcription | 4 | 40 | 298 | 6246 | 0.013 | 0.006 | 2.097 | 0.121 | 95479\_at,95480\_at,161147\_f\_at,98075\_at |
| 8 | negative regulation of transcription, DNA-dependent | 2 | 27 | 130 | 2164 | 0.015 | 0.012 | 1.232 | 0.489 | 161147\_f\_at,98075\_at |
| 9 | negative regulation of transcription from Pol II promoter | 2 | 24 | 66 | 911 | 0.03 | 0.026 | 1.15 | 0.53 | 161147\_f\_at,98075\_at |
| 7 | positive regulation of transcription | 1 | 20 | 298 | 6246 | 0.003 | 0.003 | 1.05 | 0.624 | 93548\_at |
| 8 | positive regulation of transcription, DNA-dependent | 1 | 15 | 130 | 2164 | 0.008 | 0.007 | 1.11 | 0.606 | 93548\_at |
| 9 | positive regulation of transcription from Pol II promoter | 1 | 15 | 66 | 911 | 0.015 | 0.016 | 0.92 | 0.679 | 93548\_at |
| 7 | regulation of transcription, DNA-dependent | 23 | 1013 | 298 | 6246 | 0.077 | 0.162 | 0.476 | 1 | 100156\_at,102412\_at,103319\_at,103654\_at,104476\_at,160659\_at,161147\_f\_at,92565\_at,93008\_at,93041\_at,93112\_at,93251\_at,94506\_at,95132\_r\_at,95460\_at,97164\_at,98039\_at,98075\_at,98516\_at,93548\_at,93551\_at,160324\_at,93519\_s\_at |
| 8 | negative regulation of transcription, DNA-dependent | 2 | 27 | 130 | 2164 | 0.015 | 0.012 | 1.232 | 0.489 | 161147\_f\_at,98075\_at |
| 9 | negative regulation of transcription from Pol II promoter | 2 | 24 | 66 | 911 | 0.03 | 0.026 | 1.15 | 0.53 | 161147\_f\_at,98075\_at |
| 8 | positive regulation of transcription, DNA-dependent | 1 | 15 | 130 | 2164 | 0.008 | 0.007 | 1.11 | 0.606 | 93548\_at |
| 9 | positive regulation of transcription from Pol II promoter | 1 | 15 | 66 | 911 | 0.015 | 0.016 | 0.92 | 0.679 | 93548\_at |
| 8 | regulation of transcription from Pol I promoter | 2 | 2 | 130 | 2164 | 0.015 | 0.001 | 16.717 | 0.004 | 103654\_at,93551\_at |
| 8 | regulation of transcription from Pol II promoter | 5 | 72 | 130 | 2164 | 0.038 | 0.033 | 1.156 | 0.437 | 160324\_at,93519\_s\_at,161147\_f\_at,98075\_at,93548\_at |
| 9 | negative regulation of transcription from Pol II promoter | 2 | 24 | 66 | 911 | 0.03 | 0.026 | 1.15 | 0.53 | 161147\_f\_at,98075\_at |
| 9 | positive regulation of transcription from Pol II promoter | 1 | 15 | 66 | 911 | 0.015 | 0.016 | 0.92 | 0.679 | 93548\_at |
| 6 | transcription, DNA-dependent | 23 | 1046 | 466 | 9498 | 0.049 | 0.11 | 0.448 | 1 | 100156\_at,102412\_at,103319\_at,103654\_at,104476\_at,160659\_at,161147\_f\_at,92565\_at,93008\_at,93041\_at,93112\_at,93251\_at,94506\_at,95132\_r\_at,95460\_at,97164\_at,98039\_at,98075\_at,98516\_at,93548\_at,93551\_at,160324\_at,93519\_s\_at |
| 7 | regulation of transcription, DNA-dependent | 23 | 1013 | 298 | 6246 | 0.077 | 0.162 | 0.476 | 1 | 100156\_at,102412\_at,103319\_at,103654\_at,104476\_at,160659\_at,161147\_f\_at,92565\_at,93008\_at,93041\_at,93112\_at,93251\_at,94506\_at,95132\_r\_at,95460\_at,97164\_at,98039\_at,98075\_at,98516\_at,93548\_at,93551\_at,160324\_at,93519\_s\_at |
| 8 | negative regulation of transcription, DNA-dependent | 2 | 27 | 130 | 2164 | 0.015 | 0.012 | 1.232 | 0.489 | 161147\_f\_at,98075\_at |
| 9 | negative regulation of transcription from Pol II promoter | 2 | 24 | 66 | 911 | 0.03 | 0.026 | 1.15 | 0.53 | 161147\_f\_at,98075\_at |
| 8 | positive regulation of transcription, DNA-dependent | 1 | 15 | 130 | 2164 | 0.008 | 0.007 | 1.11 | 0.606 | 93548\_at |
| 9 | positive regulation of transcription from Pol II promoter | 1 | 15 | 66 | 911 | 0.015 | 0.016 | 0.92 | 0.679 | 93548\_at |
| 8 | regulation of transcription from Pol I promoter | 2 | 2 | 130 | 2164 | 0.015 | 0.001 | 16.717 | 0.004 | 103654\_at,93551\_at |
| 8 | regulation of transcription from Pol II promoter | 5 | 72 | 130 | 2164 | 0.038 | 0.033 | 1.156 | 0.437 | 160324\_at,93519\_s\_at,161147\_f\_at,98075\_at,93548\_at |
| 9 | negative regulation of transcription from Pol II promoter | 2 | 24 | 66 | 911 | 0.03 | 0.026 | 1.15 | 0.53 | 161147\_f\_at,98075\_at |
| 9 | positive regulation of transcription from Pol II promoter | 1 | 15 | 66 | 911 | 0.015 | 0.016 | 0.92 | 0.679 | 93548\_at |
| 7 | transcription from Pol II promoter | 7 | 104 | 298 | 6246 | 0.023 | 0.017 | 1.411 | 0.227 | 93551\_at,160324\_at,93519\_s\_at,161147\_f\_at,98075\_at,93548\_at,98516\_at |
| 8 | regulation of transcription from Pol II promoter | 5 | 72 | 130 | 2164 | 0.038 | 0.033 | 1.156 | 0.437 | 160324\_at,93519\_s\_at,161147\_f\_at,98075\_at,93548\_at |
| 9 | negative regulation of transcription from Pol II promoter | 2 | 24 | 66 | 911 | 0.03 | 0.026 | 1.15 | 0.53 | 161147\_f\_at,98075\_at |
| 9 | positive regulation of transcription from Pol II promoter | 1 | 15 | 66 | 911 | 0.015 | 0.016 | 0.92 | 0.679 | 93548\_at |
| 8 | transcription initiation from Pol II promoter | 1 | 7 | 130 | 2164 | 0.008 | 0.003 | 2.381 | 0.352 | 98516\_at |
| 7 | transcription from Pol III promoter | 1 | 2 | 298 | 6246 | 0.003 | 0 | 10.5 | 0.093 | 93551\_at |
| 5 | two-component signal transduction system (phosphorelay) | 1 | 15 | 600 | 11544 | 0.002 | 0.001 | 1.285 | 0.551 | 94506\_at |
| 5 | two-component signal transduction system (phosphorelay) | 1 | 15 | 600 | 11544 | 0.002 | 0.001 | 1.285 | 0.551 | 94506\_at |
| 4 | oxygen and reactive oxygen species metabolism | 6 | 34 | 695 | 13100 | 0.009 | 0.003 | 3.319 | 0.008 | 100331\_g\_at,97819\_at,99583\_at,97758\_at,100059\_at,97013\_f\_at |
| 5 | glutathione conjugation reaction | 2 | 15 | 600 | 11544 | 0.003 | 0.001 | 2.562 | 0.182 | 97819\_at,99583\_at |
| 5 | response to oxidative stress | 2 | 10 | 600 | 11544 | 0.003 | 0.001 | 3.828 | 0.092 | 100331\_g\_at,97758\_at |
| 5 | superoxide metabolism | 2 | 10 | 600 | 11544 | 0.003 | 0.001 | 3.828 | 0.092 | 100059\_at,97013\_f\_at |
| 4 | phosphorus metabolism | 17 | 488 | 695 | 13100 | 0.024 | 0.037 | 0.657 | 0.979 | 104423\_at,96052\_at,93014\_at,95656\_i\_at,100079\_at,94062\_at,96267\_at,96899\_at,96902\_at,100128\_at,104080\_at,160538\_at,160659\_at,98595\_at,98934\_at,99522\_at,99544\_at |
| 5 | phosphate metabolism | 17 | 488 | 600 | 11544 | 0.028 | 0.042 | 0.67 | 0.973 | 104423\_at,96052\_at,93014\_at,95656\_i\_at,100079\_at,94062\_at,96267\_at,96899\_at,96902\_at,100128\_at,104080\_at,160538\_at,160659\_at,98595\_at,98934\_at,99522\_at,99544\_at |
| 6 | dephosphorylation | 2 | 92 | 466 | 9498 | 0.004 | 0.01 | 0.443 | 0.945 | 104423\_at,96052\_at |
| 7 | protein amino acid dephosphorylation | 2 | 92 | 298 | 6246 | 0.007 | 0.015 | 0.456 | 0.939 | 104423\_at,96052\_at |
| 6 | phosphorylation | 15 | 395 | 466 | 9498 | 0.032 | 0.042 | 0.774 | 0.88 | 93014\_at,95656\_i\_at,100079\_at,94062\_at,96267\_at,96899\_at,96902\_at,100128\_at,104080\_at,160538\_at,160659\_at,98595\_at,98934\_at,99522\_at,99544\_at |
| 7 | mitochondrial electron transport, NADH to ubiquinone | 5 | 6 | 298 | 6246 | 0.017 | 0.001 | 17.479 | 0 | 100079\_at,94062\_at,96267\_at,96899\_at,96902\_at |
| 7 | protein amino acid phosphorylation | 8 | 379 | 298 | 6246 | 0.027 | 0.061 | 0.442 | 0.998 | 100128\_at,104080\_at,160538\_at,160659\_at,98595\_at,98934\_at,99522\_at,99544\_at |
| 8 | peptidyl-tyrosine phosphorylation | 1 | 4 | 130 | 2164 | 0.008 | 0.002 | 4.157 | 0.22 | 104080\_at |
| 6 | ATP synthesis coupled electron transport (sensu Eukarya) | 5 | 7 | 466 | 9498 | 0.011 | 0.001 | 14.5 | 0 | 100079\_at,94062\_at,96267\_at,96899\_at,96902\_at |
| 7 | mitochondrial electron transport, NADH to ubiquinone | 5 | 6 | 298 | 6246 | 0.017 | 0.001 | 17.479 | 0 | 100079\_at,94062\_at,96267\_at,96899\_at,96902\_at |
| 5 | ATP synthesis coupled electron transport | 5 | 7 | 600 | 11544 | 0.008 | 0.001 | 13.656 | 0 | 100079\_at,94062\_at,96267\_at,96899\_at,96902\_at |
| 6 | ATP synthesis coupled electron transport (sensu Eukarya) | 5 | 7 | 466 | 9498 | 0.011 | 0.001 | 14.5 | 0 | 100079\_at,94062\_at,96267\_at,96899\_at,96902\_at |
| 7 | mitochondrial electron transport, NADH to ubiquinone | 5 | 6 | 298 | 6246 | 0.017 | 0.001 | 17.479 | 0 | 100079\_at,94062\_at,96267\_at,96899\_at,96902\_at |
| 4 | protein metabolism | 94 | 1458 | 695 | 13100 | 0.135 | 0.111 | 1.215 | 0.025 | 92636\_f\_at,94014\_at,94210\_at,95441\_at,96668\_at,96670\_at,96734\_at,96849\_at,96947\_at,97477\_at,97478\_at,98959\_at,99156\_at,101061\_at,101254\_at,93970\_at,94323\_at,101097\_at,101680\_at,102019\_at,160431\_at,92578\_at,92646\_at,93062\_at,93579\_at,94252\_at,94494\_at,94870\_f\_at,94912\_at,95067\_at,95498\_at,96291\_f\_at,96292\_r\_at,96293\_at,97342\_at,97751\_f\_at,97824\_at,97884\_at,98120\_at,98524\_f\_at,98904\_at,99594\_at,95677\_at,103881\_at,92565\_at,93734\_i\_at,93735\_f\_at,94025\_at,95448\_at,100543\_s\_at,101562\_at,104541\_at,92874\_f\_at,95561\_at,96093\_at,96733\_at,99655\_at,93519\_s\_at,100512\_at,100733\_at,101486\_at,101558\_s\_at,101992\_at,102791\_at,92547\_at,93085\_at,93988\_at,94263\_f\_at,94841\_at,96892\_at,96952\_at,97459\_at,98557\_f\_at,98975\_at,100089\_at,101207\_at,160416\_at,160456\_at,92829\_at,98153\_at,99546\_at,93101\_s\_at,97460\_at,104423\_at,96052\_at,100128\_at,104080\_at,160538\_at,160659\_at,98595\_at,98934\_at,99522\_at,99544\_at,101440\_at |
| 5 | protein folding | 7 | 56 | 600 | 11544 | 0.012 | 0.005 | 2.406 | 0.025 | 100089\_at,101207\_at,160416\_at,160456\_at,92829\_at,98153\_at,99546\_at |
| 5 | protein localization | 1 | 5 | 600 | 11544 | 0.002 | 0 | 3.884 | 0.234 | 93519\_s\_at |
| 5 | protein modification | 15 | 654 | 600 | 11544 | 0.025 | 0.057 | 0.441 | 1 | 93101\_s\_at,97460\_at,103881\_at,104423\_at,96052\_at,100128\_at,104080\_at,160538\_at,160659\_at,98595\_at,98934\_at,99522\_at,99544\_at,101440\_at,92547\_at |
| 6 | protein amino acid ADP-ribosylation | 1 | 12 | 466 | 9498 | 0.002 | 0.001 | 1.706 | 0.453 | 101440\_at |
| 6 | ubiquitin cycle | 3 | 56 | 466 | 9498 | 0.006 | 0.006 | 1.092 | 0.523 | 92547\_at,93101\_s\_at,97460\_at |
| 4 | vitamin metabolism | 1 | 22 | 695 | 13100 | 0.001 | 0.002 | 0.857 | 0.699 | 96678\_at |
| 5 | fat-soluble vitamin metabolism | 1 | 2 | 600 | 11544 | 0.002 | 0 | 9.824 | 0.101 | 96678\_at |
| 6 | vitamin A metabolism | 1 | 2 | 466 | 9498 | 0.002 | 0 | 10.238 | 0.096 | 96678\_at |
| 7 | retinal metabolism | 1 | 1 | 298 | 6246 | 0.003 | 0 | 21 | 0.048 | 96678\_at |
| 4 | xenobiotic metabolism | 1 | 20 | 695 | 13100 | 0.001 | 0.002 | 0.941 | 0.664 | 95015\_at |
| 3 | response to external stimulus | 17 | 666 | 509 | 10726 | 0.033 | 0.062 | 0.538 | 0.999 | 161872\_f\_at,103038\_at,100079\_at,94210\_at,97449\_at,160293\_at,93277\_at,100331\_g\_at,97758\_at,102838\_at,93078\_at,102791\_at,93085\_at,96093\_at,96231\_at,102970\_at,93838\_at |
| 4 | perception of external stimulus | 5 | 84 | 695 | 13100 | 0.007 | 0.006 | 1.122 | 0.462 | 161872\_f\_at,103038\_at,100079\_at,94210\_at,97449\_at |
| 5 | perception of abiotic stimulus | 5 | 72 | 600 | 11544 | 0.008 | 0.006 | 1.335 | 0.319 | 161872\_f\_at,103038\_at,100079\_at,94210\_at,97449\_at |
| 6 | perception of light | 2 | 41 | 466 | 9498 | 0.004 | 0.004 | 0.993 | 0.605 | 161872\_f\_at,103038\_at |
| 7 | phototransduction | 1 | 17 | 298 | 6246 | 0.003 | 0.003 | 1.235 | 0.565 | 161872\_f\_at |
| 7 | vision | 2 | 34 | 298 | 6246 | 0.007 | 0.005 | 1.233 | 0.488 | 103038\_at,161872\_f\_at |
| 6 | perception of sound | 3 | 17 | 466 | 9498 | 0.006 | 0.002 | 3.598 | 0.048 | 100079\_at,94210\_at,97449\_at |
| 7 | hearing | 3 | 17 | 298 | 6246 | 0.01 | 0.003 | 3.702 | 0.044 | 100079\_at,94210\_at,97449\_at |
| 4 | response to abiotic stimulus | 7 | 195 | 695 | 13100 | 0.01 | 0.015 | 0.676 | 0.898 | 161872\_f\_at,103038\_at,100079\_at,94210\_at,97449\_at,160293\_at,93277\_at |
| 5 | perception of abiotic stimulus | 5 | 72 | 600 | 11544 | 0.008 | 0.006 | 1.335 | 0.319 | 161872\_f\_at,103038\_at,100079\_at,94210\_at,97449\_at |
| 6 | perception of light | 2 | 41 | 466 | 9498 | 0.004 | 0.004 | 0.993 | 0.605 | 161872\_f\_at,103038\_at |
| 7 | phototransduction | 1 | 17 | 298 | 6246 | 0.003 | 0.003 | 1.235 | 0.565 | 161872\_f\_at |
| 7 | vision | 2 | 34 | 298 | 6246 | 0.007 | 0.005 | 1.233 | 0.488 | 103038\_at,161872\_f\_at |
| 6 | perception of sound | 3 | 17 | 466 | 9498 | 0.006 | 0.002 | 3.598 | 0.048 | 100079\_at,94210\_at,97449\_at |
| 7 | hearing | 3 | 17 | 298 | 6246 | 0.01 | 0.003 | 3.702 | 0.044 | 100079\_at,94210\_at,97449\_at |
| 5 | response to chemical substance | 1 | 90 | 600 | 11544 | 0.002 | 0.008 | 0.214 | 0.992 | 160293\_at |
| 6 | chemotaxis | 1 | 60 | 466 | 9498 | 0.002 | 0.006 | 0.34 | 0.952 | 160293\_at |
| 5 | response to temperature | 1 | 28 | 600 | 11544 | 0.002 | 0.002 | 0.687 | 0.776 | 93277\_at |
| 6 | response to heat | 1 | 27 | 466 | 9498 | 0.002 | 0.003 | 0.757 | 0.743 | 93277\_at |
| 4 | response to biotic stimulus | 10 | 516 | 695 | 13100 | 0.014 | 0.039 | 0.365 | 1 | 100331\_g\_at,97758\_at,102838\_at,93078\_at,102791\_at,93085\_at,96093\_at,96231\_at,102970\_at,93838\_at |
| 5 | response to oxidative stress | 2 | 10 | 600 | 11544 | 0.003 | 0.001 | 3.828 | 0.092 | 100331\_g\_at,97758\_at |
| 5 | defense response | 8 | 471 | 600 | 11544 | 0.013 | 0.041 | 0.327 | 1 | 102838\_at,93078\_at,102791\_at,93085\_at,96093\_at,96231\_at,102970\_at,93838\_at |
| 6 | immune response | 6 | 362 | 466 | 9498 | 0.013 | 0.038 | 0.338 | 1 | 102791\_at,93085\_at,96093\_at,96231\_at,102970\_at,93838\_at |
| 7 | acute-phase response | 2 | 23 | 298 | 6246 | 0.007 | 0.004 | 1.823 | 0.301 | 96093\_at,96231\_at |
| 7 | innate immune response | 2 | 70 | 298 | 6246 | 0.007 | 0.011 | 0.599 | 0.854 | 102970\_at,93838\_at |
| 8 | inflammatory response | 2 | 70 | 130 | 2164 | 0.015 | 0.032 | 0.475 | 0.932 | 102970\_at,93838\_at |

  
