# Supplementary material for: Molecular Signatures of Proliferation and Quiescence in Hematopoietic Stem Cells
Source: PLoS Biol. 2004 Sep 28;2(10):e301. doi: 10.1371/journal.pbio.0020301 (PMC520599; doi:10.1371/journal.pbio.0020301)
Supplement: Table S43 — (199 KB HTML). [file pbio.0020301.st043.html]

   Significant Tom Day 6   

# Significant Tom Day 6

|  |  |  |  |  |  |  |  |  |  |  |  |
| --- | --- | --- | --- | --- | --- | --- | --- | --- | --- | --- | --- |
| GOLevel | GOTerm | ProbeCount | ArrayCount | ListGOLevelCount | ArrayGoLevelCount | ListFq | ArrayFq | FoldChange | H-Pvalue | ProbeIds | GeneNames |
| 7 | RAS protein signal transduction | 3 | 15 | 298 | 6246 | 0.01 | 0.002 | 4.196 | 0.032 | 101440\_at,160536\_at,98975\_at | RIKEN cDNA 2900006F19 gene,Harvey rat sarcoma virus oncogene 1,RIKEN cDNA 2410008G02 gene |
| 6 | apoptotic program | 3 | 16 | 466 | 9498 | 0.006 | 0.002 | 3.833 | 0.041 | 104297\_at,104301\_at,96861\_at | RIKEN cDNA 2510001A17 gene,RIKEN cDNA 2610001E06 gene,mitochondrial ribosomal protein L50 |
| 6 | mesoderm cell fate determination | 1 | 1 | 466 | 9498 | 0.002 | 0 | 19.545 | 0.049 | 93456\_r\_at | bone morphogenetic protein 4 |
| 4 | myeloid blood cell differentiation | 2 | 7 | 695 | 13100 | 0.003 | 0.001 | 5.434 | 0.049 | 93548\_at,92831\_at | RIKEN cDNA 1190006C12 gene,sideroflexin 1 |
| 3 | cell growth and/or maintenance | 130 | 2128 | 509 | 10726 | 0.255 | 0.198 | 1.287 | 0.001 | 101254\_at,104476\_at,160536\_at,94394\_at,94506\_at,94933\_at,97412\_at,93593\_f\_at,92778\_i\_at,96634\_at,102197\_at,101407\_at,93812\_at,93815\_at,93784\_at,98524\_f\_at,96734\_at,100116\_at,100400\_at,160872\_f\_at,161004\_at,94789\_r\_at,97276\_at,94862\_i\_at,97979\_at,101061\_at,93062\_at,94014\_at,94210\_at,96849\_at,97477\_at,97478\_at,104322\_at,96956\_at,102126\_at,160266\_r\_at,160531\_at,92646\_at,96291\_f\_at,96292\_r\_at,96293\_at,160503\_at,99151\_at,101954\_at,93251\_at,93833\_s\_at,98039\_at,94897\_at,96775\_at,93112\_at,98587\_at,95660\_at,104762\_r\_at,92770\_at,95491\_at,95891\_at,100128\_at,102853\_at,160538\_at,160659\_at,94294\_at,99522\_at,99632\_at,100156\_at,100612\_at,101065\_at,101067\_at,102001\_at,102631\_at,103418\_at,104738\_at,93041\_at,96289\_at,98975\_at,104733\_at,92788\_f\_at,99129\_at,103805\_at,100459\_at,100618\_f\_at,102821\_s\_at,103534\_at,104380\_at,160076\_at,160256\_at,160543\_at,160856\_at,160876\_at,161487\_f\_at,92831\_at,93084\_at,93734\_i\_at,93735\_f\_at,93993\_at,94277\_at,94323\_at,95061\_at,95091\_at,95441\_at,95690\_at,96048\_at,96336\_at,96668\_at,96892\_at,97248\_at,97374\_at,98930\_at,99147\_at,97424\_at,93533\_at,99128\_at,160125\_at,160126\_at,160203\_at,92798\_at,92799\_g\_at,92800\_i\_at,93596\_i\_at,95656\_i\_at,96611\_at,93014\_at,92636\_f\_at,96670\_at,96947\_at,98959\_at,99156\_at,93970\_at,100917\_at,95654\_at,160551\_at | RAN, member RAS oncogene family,retinoblastoma-like 1 (p107),Harvey rat sarcoma virus oncogene 1,Harvey rat sarcoma oncogene, subgroup R,cleavage and polyadenylation specific factor 5,cDNA sequence BC008155,RIKEN cDNA 3300001G02 gene,epithelial membrane protein 3,NA,RIKEN cDNA 5730469M10 gene,nucleobindin 2,Friedreich ataxia,chloride channel, nucleotide-sensitive, 1A,RIKEN cDNA 0610041L09 gene,craniofacial development protein 1,RIKEN cDNA 2210039B01 gene,synaptojanin 2 binding protein,RIKEN cDNA 2810417H13 gene,RIKEN cDNA 4921531G14 gene,RIKEN cDNA 2310008H09 gene,RIKEN cDNA 1700097N02 gene,tubulin, beta 5,cytoskeleton-associated protein 1,dynein, cytoplasmic, light chain 2A,protein phosphatase 1, regulatory (inhibitor) subunit 7,signal sequence receptor, beta,mitochondrial ribosomal protein L39,RIKEN cDNA 2510048O06 gene,translocase of inner mitochondrial membrane 9 homolog (yeast),translocase of inner mitochondrial membrane 8 homolog a (yeast),translocase of inner mitochondrial membrane 8 homolog b (yeast),golgi phosphoprotein 2,cytoskeleton associated protein 2,RIKEN cDNA 0610038D11 gene,ribosomal protein S12,RIKEN cDNA 1110064N10 gene,B-cell receptor-associated protein 37,mitochondrial ribosomal protein L23,NA,NA,RIKEN cDNA 2410015N17 gene,fibrillarin,RIKEN cDNA 2610002K22 gene,H2A histone family, member Z,4-nitrophenylphosphatase domain and non-neuronal SNAP25-like protein homolog 1 (C. elegans),histone 1, H2bl,RIKEN cDNA 2410015M20 gene,glutathione peroxidase 4,chromobox homolog 1 (Drosophila HP1 beta),minichromosome maintenance deficient 2 mitotin (S. cerevisiae),nucleosome assembly protein 1-like 1,RIKEN cDNA 0610025L15 gene,RIKEN cDNA 1500015J03 gene,S100 calcium binding protein A6 (calcyclin),Parkinson disease (autosomal recessive, early onset) 7,NA,cell division cycle 2 homolog A (S. pombe),chondroitin sulfate proteoglycan 6,cyclin-dependent kinase 4,RIKEN cDNA 2310057G13 gene,cyclin B2,germ cell-specific gene 2,MAD2 (mitotic arrest deficient, homolog)-like 1 (yeast),minichromosome maintenance deficient 5, cell division cycle 46 (S. cerevisiae),ribonucleotide reductase M1,proliferating cell nuclear antigen,RIKEN cDNA 2010005E08 gene,ribonucleotide reductase M2,Bloom syndrome homolog (human),expressed sequence AU040575,zuotin related factor 2,minichromosome maintenance deficient 4 homolog (S. cerevisiae),stomatin (Epb7.2)-like 2,RIKEN cDNA 2410008G02 gene,centrin 2,centrin 3,CD40 ligand-activated specific transcript 3,nibrin,RAD50 homolog (S. cerevisiae),solute carrier family 25 (mitochondrial carrier; adenine nucleotide translocator), member 5,RAS-like, family 2, locus 9,hemoglobin, beta adult minor chain,solute carrier family 35 (CMP-sialic acid transporter), member 1,metaxin 2,RIKEN cDNA 1110020J08 gene,sorting nexin 3,ubiquitin-like 4,B-cell receptor-associated protein 29,ATP synthase, H+ transporting, mitochondrial F0 complex, subunit c (subunit 9), isoform 1,sideroflexin 1,solute carrier family 25 (mitochondrial carrier; adenine nucleotide translocator), member 4,proteasome (prosome, macropain) 26S subunit, ATPase 3,proteasome (prosome, macropain) 26S subunit, ATPase 3,RIKEN cDNA 1300009F09 gene,metaxin 1,RIKEN cDNA D630012G11 gene,breast carcinoma amplified sequence 2,SEC13 related gene (S. cerevisiae),translocase of inner mitochondrial membrane 23 homolog (yeast),RIKEN cDNA 1110030L07 gene,heat-responsive protein 12,glycine amidinotransferase (L-arginine:glycine amidinotransferase),translocator of inner mitochondrial membrane b,proteasome (prosome, macropain) subunit, alpha type 1,diazepam binding inhibitor,RIKEN cDNA 2810025M15 gene,coatomer protein complex, subunit epsilon,RIKEN cDNA 2700001K05 gene,ADP-ribosylation-like factor 6 interacting protein 5,RIKEN cDNA 1500011L16 gene,ATP synthase, H+ transporting, mitochondrial F1 complex, O subunit,DNA segment, Chr 11, ERATO Doi 497, expressed,mitogen-activated protein kinase kinase 1 interacting protein 1,RIKEN cDNA 5330419I01 gene,ATP synthase, H+ transporting, mitochondrial F1 complex, gamma polypeptide 1,ATP synthase, H+ transporting, mitochondrial F1 complex, gamma polypeptide 1,ATP synthase, H+ transporting, mitochondrial F1 complex, gamma polypeptide 1,RIKEN cDNA 2410043G19 gene,ATPase, H+ transporting, V1 subunit F,NADH-ubiquinone oxidoreductase subunit B14.7,ATP synthase, H+ transporting, mitochondrial F0 complex, subunit g,SEC61, gamma subunit (S. cerevisiae),RIKEN cDNA 0610025I19 gene,RIKEN cDNA 0610009I16 gene,RIKEN cDNA D930048L02 gene,RIKEN cDNA 2700099C19 gene,importin 7,RIKEN cDNA 2410080P20 gene,chloride intracellular channel 1,voltage-dependent anion channel 3 |
| 5 | regulation of cell volume | 2 | 1 | 600 | 11544 | 0.003 | 0 | 37 | 0 | 93812\_at,93815\_at | chloride channel, nucleotide-sensitive, 1A,RIKEN cDNA 0610041L09 gene |
| 4 | cell organization and biogenesis | 39 | 530 | 695 | 13100 | 0.056 | 0.04 | 1.387 | 0.024 | 93784\_at,98524\_f\_at,96734\_at,100116\_at,100400\_at,160872\_f\_at,161004\_at,94789\_r\_at,97276\_at,101254\_at,94862\_i\_at,97979\_at,101061\_at,93062\_at,94014\_at,94210\_at,96849\_at,97477\_at,97478\_at,104322\_at,96956\_at,102126\_at,160266\_r\_at,160531\_at,92646\_at,96291\_f\_at,96292\_r\_at,96293\_at,160503\_at,99151\_at,101954\_at,93251\_at,93833\_s\_at,98039\_at,94897\_at,96775\_at,93112\_at,98587\_at,95660\_at | craniofacial development protein 1,RIKEN cDNA 2210039B01 gene,synaptojanin 2 binding protein,RIKEN cDNA 2810417H13 gene,RIKEN cDNA 4921531G14 gene,RIKEN cDNA 2310008H09 gene,RIKEN cDNA 1700097N02 gene,tubulin, beta 5,cytoskeleton-associated protein 1,RAN, member RAS oncogene family,dynein, cytoplasmic, light chain 2A,protein phosphatase 1, regulatory (inhibitor) subunit 7,signal sequence receptor, beta,mitochondrial ribosomal protein L39,RIKEN cDNA 2510048O06 gene,translocase of inner mitochondrial membrane 9 homolog (yeast),translocase of inner mitochondrial membrane 8 homolog a (yeast),translocase of inner mitochondrial membrane 8 homolog b (yeast),golgi phosphoprotein 2,cytoskeleton associated protein 2,RIKEN cDNA 0610038D11 gene,ribosomal protein S12,RIKEN cDNA 1110064N10 gene,B-cell receptor-associated protein 37,mitochondrial ribosomal protein L23,NA,NA,RIKEN cDNA 2410015N17 gene,fibrillarin,RIKEN cDNA 2610002K22 gene,H2A histone family, member Z,4-nitrophenylphosphatase domain and non-neuronal SNAP25-like protein homolog 1 (C. elegans),histone 1, H2bl,RIKEN cDNA 2410015M20 gene,glutathione peroxidase 4,chromobox homolog 1 (Drosophila HP1 beta),minichromosome maintenance deficient 2 mitotin (S. cerevisiae),nucleosome assembly protein 1-like 1,RIKEN cDNA 0610025L15 gene |
| 5 | cytoplasm organization and biogenesis | 29 | 380 | 600 | 11544 | 0.048 | 0.033 | 1.468 | 0.024 | 96734\_at,100116\_at,100400\_at,160872\_f\_at,161004\_at,94789\_r\_at,97276\_at,101254\_at,94862\_i\_at,97979\_at,101061\_at,93062\_at,94014\_at,94210\_at,96849\_at,97477\_at,97478\_at,104322\_at,96956\_at,102126\_at,160266\_r\_at,160531\_at,92646\_at,96291\_f\_at,96292\_r\_at,96293\_at,98524\_f\_at,160503\_at,99151\_at | synaptojanin 2 binding protein,RIKEN cDNA 2810417H13 gene,RIKEN cDNA 4921531G14 gene,RIKEN cDNA 2310008H09 gene,RIKEN cDNA 1700097N02 gene,tubulin, beta 5,cytoskeleton-associated protein 1,RAN, member RAS oncogene family,dynein, cytoplasmic, light chain 2A,protein phosphatase 1, regulatory (inhibitor) subunit 7,signal sequence receptor, beta,mitochondrial ribosomal protein L39,RIKEN cDNA 2510048O06 gene,translocase of inner mitochondrial membrane 9 homolog (yeast),translocase of inner mitochondrial membrane 8 homolog a (yeast),translocase of inner mitochondrial membrane 8 homolog b (yeast),golgi phosphoprotein 2,cytoskeleton associated protein 2,RIKEN cDNA 0610038D11 gene,ribosomal protein S12,RIKEN cDNA 1110064N10 gene,B-cell receptor-associated protein 37,mitochondrial ribosomal protein L23,NA,NA,RIKEN cDNA 2410015N17 gene,RIKEN cDNA 2210039B01 gene,fibrillarin,RIKEN cDNA 2610002K22 gene |
| 7 | mitochondrion organization and biogenesis | 6 | 13 | 298 | 6246 | 0.02 | 0.002 | 9.678 | 0 | 93062\_at,94014\_at,94210\_at,96849\_at,97477\_at,97478\_at | mitochondrial ribosomal protein L39,RIKEN cDNA 2510048O06 gene,translocase of inner mitochondrial membrane 9 homolog (yeast),translocase of inner mitochondrial membrane 8 homolog a (yeast),translocase of inner mitochondrial membrane 8 homolog b (yeast),golgi phosphoprotein 2 |
| 8 | protein-mitochondrial targeting | 5 | 6 | 130 | 2164 | 0.038 | 0.003 | 13.884 | 0 | 94014\_at,94210\_at,96849\_at,97477\_at,97478\_at | RIKEN cDNA 2510048O06 gene,translocase of inner mitochondrial membrane 9 homolog (yeast),translocase of inner mitochondrial membrane 8 homolog a (yeast),translocase of inner mitochondrial membrane 8 homolog b (yeast),golgi phosphoprotein 2 |
| 9 | mitochondrial translocation | 5 | 6 | 66 | 911 | 0.076 | 0.007 | 11.496 | 0 | 94014\_at,94210\_at,96849\_at,97477\_at,97478\_at | RIKEN cDNA 2510048O06 gene,translocase of inner mitochondrial membrane 9 homolog (yeast),translocase of inner mitochondrial membrane 8 homolog a (yeast),translocase of inner mitochondrial membrane 8 homolog b (yeast),golgi phosphoprotein 2 |
| 6 | ribosome biogenesis and assembly | 10 | 60 | 466 | 9498 | 0.021 | 0.006 | 3.396 | 0.001 | 102126\_at,160266\_r\_at,160531\_at,92646\_at,96291\_f\_at,96292\_r\_at,96293\_at,98524\_f\_at,160503\_at,99151\_at | ribosomal protein S12,RIKEN cDNA 1110064N10 gene,B-cell receptor-associated protein 37,mitochondrial ribosomal protein L23,NA,NA,RIKEN cDNA 2410015N17 gene,RIKEN cDNA 2210039B01 gene,fibrillarin,RIKEN cDNA 2610002K22 gene |
| 7 | ribosome biogenesis | 10 | 60 | 298 | 6246 | 0.034 | 0.01 | 3.492 | 0 | 102126\_at,160266\_r\_at,160531\_at,92646\_at,96291\_f\_at,96292\_r\_at,96293\_at,98524\_f\_at,160503\_at,99151\_at | ribosomal protein S12,RIKEN cDNA 1110064N10 gene,B-cell receptor-associated protein 37,mitochondrial ribosomal protein L23,NA,NA,RIKEN cDNA 2410015N17 gene,RIKEN cDNA 2210039B01 gene,fibrillarin,RIKEN cDNA 2610002K22 gene |
| 8 | rRNA processing | 3 | 14 | 130 | 2164 | 0.023 | 0.006 | 3.567 | 0.047 | 160503\_at,160531\_at,99151\_at | fibrillarin,B-cell receptor-associated protein 37,RIKEN cDNA 2610002K22 gene |
| 5 | cell cycle | 32 | 435 | 600 | 11544 | 0.053 | 0.038 | 1.415 | 0.03 | 100128\_at,102853\_at,104476\_at,160538\_at,160659\_at,92770\_at,93112\_at,94294\_at,99522\_at,99632\_at,100156\_at,100612\_at,101065\_at,101067\_at,102001\_at,102631\_at,103418\_at,104738\_at,93041\_at,96289\_at,98975\_at,101254\_at,104733\_at,92788\_f\_at,99129\_at,160536\_at,94394\_at,94506\_at,94933\_at,95891\_at,97412\_at,103805\_at | cell division cycle 2 homolog A (S. pombe),chondroitin sulfate proteoglycan 6,retinoblastoma-like 1 (p107),cyclin-dependent kinase 4,RIKEN cDNA 2310057G13 gene,S100 calcium binding protein A6 (calcyclin),minichromosome maintenance deficient 2 mitotin (S. cerevisiae),cyclin B2,germ cell-specific gene 2,MAD2 (mitotic arrest deficient, homolog)-like 1 (yeast),minichromosome maintenance deficient 5, cell division cycle 46 (S. cerevisiae),ribonucleotide reductase M1,proliferating cell nuclear antigen,RIKEN cDNA 2010005E08 gene,ribonucleotide reductase M2,Bloom syndrome homolog (human),expressed sequence AU040575,zuotin related factor 2,minichromosome maintenance deficient 4 homolog (S. cerevisiae),stomatin (Epb7.2)-like 2,RIKEN cDNA 2410008G02 gene,RAN, member RAS oncogene family,centrin 2,centrin 3,CD40 ligand-activated specific transcript 3,Harvey rat sarcoma virus oncogene 1,Harvey rat sarcoma oncogene, subgroup R,cleavage and polyadenylation specific factor 5,cDNA sequence BC008155,NA,RIKEN cDNA 3300001G02 gene,nibrin |
| 6 | DNA replication and chromosome cycle | 14 | 113 | 466 | 9498 | 0.03 | 0.012 | 2.524 | 0.001 | 102853\_at,99632\_at,100156\_at,100612\_at,101065\_at,101067\_at,102001\_at,102631\_at,103418\_at,104738\_at,93041\_at,93112\_at,96289\_at,98975\_at | chondroitin sulfate proteoglycan 6,MAD2 (mitotic arrest deficient, homolog)-like 1 (yeast),minichromosome maintenance deficient 5, cell division cycle 46 (S. cerevisiae),ribonucleotide reductase M1,proliferating cell nuclear antigen,RIKEN cDNA 2010005E08 gene,ribonucleotide reductase M2,Bloom syndrome homolog (human),expressed sequence AU040575,zuotin related factor 2,minichromosome maintenance deficient 4 homolog (S. cerevisiae),minichromosome maintenance deficient 2 mitotin (S. cerevisiae),stomatin (Epb7.2)-like 2,RIKEN cDNA 2410008G02 gene |
| 7 | DNA replication | 12 | 94 | 298 | 6246 | 0.04 | 0.015 | 2.676 | 0.002 | 100156\_at,100612\_at,101065\_at,101067\_at,102001\_at,102631\_at,103418\_at,104738\_at,93041\_at,93112\_at,96289\_at,98975\_at | minichromosome maintenance deficient 5, cell division cycle 46 (S. cerevisiae),ribonucleotide reductase M1,proliferating cell nuclear antigen,RIKEN cDNA 2010005E08 gene,ribonucleotide reductase M2,Bloom syndrome homolog (human),expressed sequence AU040575,zuotin related factor 2,minichromosome maintenance deficient 4 homolog (S. cerevisiae),minichromosome maintenance deficient 2 mitotin (S. cerevisiae),stomatin (Epb7.2)-like 2,RIKEN cDNA 2410008G02 gene |
| 8 | DNA dependent DNA replication | 7 | 31 | 130 | 2164 | 0.054 | 0.014 | 3.758 | 0.002 | 100156\_at,93041\_at,93112\_at,103418\_at,98975\_at,101065\_at,101067\_at | minichromosome maintenance deficient 5, cell division cycle 46 (S. cerevisiae),minichromosome maintenance deficient 4 homolog (S. cerevisiae),minichromosome maintenance deficient 2 mitotin (S. cerevisiae),expressed sequence AU040575,RIKEN cDNA 2410008G02 gene,proliferating cell nuclear antigen,RIKEN cDNA 2010005E08 gene |
| 9 | DNA replication initiation | 3 | 10 | 66 | 911 | 0.045 | 0.011 | 4.139 | 0.03 | 100156\_at,93041\_at,93112\_at | minichromosome maintenance deficient 5, cell division cycle 46 (S. cerevisiae),minichromosome maintenance deficient 4 homolog (S. cerevisiae),minichromosome maintenance deficient 2 mitotin (S. cerevisiae) |
| 9 | regulation of DNA replication | 2 | 1 | 66 | 911 | 0.03 | 0.001 | 27.545 | 0 | 101065\_at,101067\_at | proliferating cell nuclear antigen,RIKEN cDNA 2010005E08 gene |
| 6 | M phase | 8 | 74 | 466 | 9498 | 0.017 | 0.008 | 2.204 | 0.028 | 101254\_at,100128\_at,102853\_at,104733\_at,92788\_f\_at,94294\_at,99632\_at,99129\_at | RAN, member RAS oncogene family,cell division cycle 2 homolog A (S. pombe),chondroitin sulfate proteoglycan 6,centrin 2,centrin 3,cyclin B2,MAD2 (mitotic arrest deficient, homolog)-like 1 (yeast),CD40 ligand-activated specific transcript 3 |
| 7 | M phase of mitotic cell cycle | 8 | 57 | 298 | 6246 | 0.027 | 0.009 | 2.941 | 0.005 | 100128\_at,101254\_at,102853\_at,104733\_at,92788\_f\_at,94294\_at,99632\_at,99129\_at | cell division cycle 2 homolog A (S. pombe),RAN, member RAS oncogene family,chondroitin sulfate proteoglycan 6,centrin 2,centrin 3,cyclin B2,MAD2 (mitotic arrest deficient, homolog)-like 1 (yeast),CD40 ligand-activated specific transcript 3 |
| 8 | mitosis | 8 | 57 | 130 | 2164 | 0.062 | 0.026 | 2.336 | 0.019 | 100128\_at,101254\_at,102853\_at,104733\_at,92788\_f\_at,94294\_at,99632\_at,99129\_at | cell division cycle 2 homolog A (S. pombe),RAN, member RAS oncogene family,chondroitin sulfate proteoglycan 6,centrin 2,centrin 3,cyclin B2,MAD2 (mitotic arrest deficient, homolog)-like 1 (yeast),CD40 ligand-activated specific transcript 3 |
| 10 | regulation of mitotic metaphase/anaphase transition | 1 | 1 | 5 | 197 | 0.2 | 0.005 | 39.37 | 0.025 | 99632\_at | MAD2 (mitotic arrest deficient, homolog)-like 1 (yeast) |
| 9 | regulation of mitosis | 2 | 3 | 66 | 911 | 0.03 | 0.003 | 9.21 | 0.015 | 99632\_at,99129\_at | MAD2 (mitotic arrest deficient, homolog)-like 1 (yeast),CD40 ligand-activated specific transcript 3 |
| 10 | regulation of mitotic metaphase/anaphase transition | 1 | 1 | 5 | 197 | 0.2 | 0.005 | 39.37 | 0.025 | 99632\_at | MAD2 (mitotic arrest deficient, homolog)-like 1 (yeast) |
| 10 | mitotic checkpoint | 2 | 3 | 5 | 197 | 0.4 | 0.015 | 26.264 | 0.002 | 99129\_at,99632\_at | CD40 ligand-activated specific transcript 3,MAD2 (mitotic arrest deficient, homolog)-like 1 (yeast) |
| 11 | mitotic spindle checkpoint | 2 | 2 | 4 | 34 | 0.5 | 0.059 | 8.501 | 0.011 | 99129\_at,99632\_at | CD40 ligand-activated specific transcript 3,MAD2 (mitotic arrest deficient, homolog)-like 1 (yeast) |
| 7 | nuclear division | 8 | 73 | 298 | 6246 | 0.027 | 0.012 | 2.297 | 0.022 | 100128\_at,101254\_at,102853\_at,104733\_at,92788\_f\_at,94294\_at,99632\_at,99129\_at | cell division cycle 2 homolog A (S. pombe),RAN, member RAS oncogene family,chondroitin sulfate proteoglycan 6,centrin 2,centrin 3,cyclin B2,MAD2 (mitotic arrest deficient, homolog)-like 1 (yeast),CD40 ligand-activated specific transcript 3 |
| 8 | mitosis | 8 | 57 | 130 | 2164 | 0.062 | 0.026 | 2.336 | 0.019 | 100128\_at,101254\_at,102853\_at,104733\_at,92788\_f\_at,94294\_at,99632\_at,99129\_at | cell division cycle 2 homolog A (S. pombe),RAN, member RAS oncogene family,chondroitin sulfate proteoglycan 6,centrin 2,centrin 3,cyclin B2,MAD2 (mitotic arrest deficient, homolog)-like 1 (yeast),CD40 ligand-activated specific transcript 3 |
| 10 | regulation of mitotic metaphase/anaphase transition | 1 | 1 | 5 | 197 | 0.2 | 0.005 | 39.37 | 0.025 | 99632\_at | MAD2 (mitotic arrest deficient, homolog)-like 1 (yeast) |
| 9 | regulation of mitosis | 2 | 3 | 66 | 911 | 0.03 | 0.003 | 9.21 | 0.015 | 99632\_at,99129\_at | MAD2 (mitotic arrest deficient, homolog)-like 1 (yeast),CD40 ligand-activated specific transcript 3 |
| 10 | regulation of mitotic metaphase/anaphase transition | 1 | 1 | 5 | 197 | 0.2 | 0.005 | 39.37 | 0.025 | 99632\_at | MAD2 (mitotic arrest deficient, homolog)-like 1 (yeast) |
| 10 | mitotic checkpoint | 2 | 3 | 5 | 197 | 0.4 | 0.015 | 26.264 | 0.002 | 99129\_at,99632\_at | CD40 ligand-activated specific transcript 3,MAD2 (mitotic arrest deficient, homolog)-like 1 (yeast) |
| 11 | mitotic spindle checkpoint | 2 | 2 | 4 | 34 | 0.5 | 0.059 | 8.501 | 0.011 | 99129\_at,99632\_at | CD40 ligand-activated specific transcript 3,MAD2 (mitotic arrest deficient, homolog)-like 1 (yeast) |
| 7 | M phase of mitotic cell cycle | 8 | 57 | 298 | 6246 | 0.027 | 0.009 | 2.941 | 0.005 | 100128\_at,101254\_at,102853\_at,104733\_at,92788\_f\_at,94294\_at,99632\_at,99129\_at | cell division cycle 2 homolog A (S. pombe),RAN, member RAS oncogene family,chondroitin sulfate proteoglycan 6,centrin 2,centrin 3,cyclin B2,MAD2 (mitotic arrest deficient, homolog)-like 1 (yeast),CD40 ligand-activated specific transcript 3 |
| 8 | mitosis | 8 | 57 | 130 | 2164 | 0.062 | 0.026 | 2.336 | 0.019 | 100128\_at,101254\_at,102853\_at,104733\_at,92788\_f\_at,94294\_at,99632\_at,99129\_at | cell division cycle 2 homolog A (S. pombe),RAN, member RAS oncogene family,chondroitin sulfate proteoglycan 6,centrin 2,centrin 3,cyclin B2,MAD2 (mitotic arrest deficient, homolog)-like 1 (yeast),CD40 ligand-activated specific transcript 3 |
| 10 | regulation of mitotic metaphase/anaphase transition | 1 | 1 | 5 | 197 | 0.2 | 0.005 | 39.37 | 0.025 | 99632\_at | MAD2 (mitotic arrest deficient, homolog)-like 1 (yeast) |
| 9 | regulation of mitosis | 2 | 3 | 66 | 911 | 0.03 | 0.003 | 9.21 | 0.015 | 99632\_at,99129\_at | MAD2 (mitotic arrest deficient, homolog)-like 1 (yeast),CD40 ligand-activated specific transcript 3 |
| 10 | regulation of mitotic metaphase/anaphase transition | 1 | 1 | 5 | 197 | 0.2 | 0.005 | 39.37 | 0.025 | 99632\_at | MAD2 (mitotic arrest deficient, homolog)-like 1 (yeast) |
| 10 | mitotic checkpoint | 2 | 3 | 5 | 197 | 0.4 | 0.015 | 26.264 | 0.002 | 99129\_at,99632\_at | CD40 ligand-activated specific transcript 3,MAD2 (mitotic arrest deficient, homolog)-like 1 (yeast) |
| 11 | mitotic spindle checkpoint | 2 | 2 | 4 | 34 | 0.5 | 0.059 | 8.501 | 0.011 | 99129\_at,99632\_at | CD40 ligand-activated specific transcript 3,MAD2 (mitotic arrest deficient, homolog)-like 1 (yeast) |
| 7 | cell cycle checkpoint | 3 | 10 | 298 | 6246 | 0.01 | 0.002 | 6.294 | 0.01 | 99129\_at,99632\_at,103805\_at | CD40 ligand-activated specific transcript 3,MAD2 (mitotic arrest deficient, homolog)-like 1 (yeast),nibrin |
| 7 | acidic amino acid transport | 1 | 1 | 298 | 6246 | 0.003 | 0 | 21 | 0.048 | 97424\_at | ADP-ribosylation-like factor 6 interacting protein 5 |
| 5 | hydrogen transport | 13 | 50 | 600 | 11544 | 0.022 | 0.004 | 5.005 | 0 | 93533\_at,99128\_at,160125\_at,160126\_at,160203\_at,161487\_f\_at,92798\_at,92799\_g\_at,92800\_i\_at,93596\_i\_at,95656\_i\_at,96611\_at,93014\_at | RIKEN cDNA 1500011L16 gene,ATP synthase, H+ transporting, mitochondrial F1 complex, O subunit,DNA segment, Chr 11, ERATO Doi 497, expressed,mitogen-activated protein kinase kinase 1 interacting protein 1,RIKEN cDNA 5330419I01 gene,ATP synthase, H+ transporting, mitochondrial F0 complex, subunit c (subunit 9), isoform 1,ATP synthase, H+ transporting, mitochondrial F1 complex, gamma polypeptide 1,ATP synthase, H+ transporting, mitochondrial F1 complex, gamma polypeptide 1,ATP synthase, H+ transporting, mitochondrial F1 complex, gamma polypeptide 1,RIKEN cDNA 2410043G19 gene,ATPase, H+ transporting, V1 subunit F,NADH-ubiquinone oxidoreductase subunit B14.7,ATP synthase, H+ transporting, mitochondrial F0 complex, subunit g |
| 6 | proton transport | 12 | 44 | 466 | 9498 | 0.026 | 0.005 | 5.562 | 0 | 160125\_at,160126\_at,160203\_at,161487\_f\_at,92798\_at,92799\_g\_at,92800\_i\_at,93596\_i\_at,95656\_i\_at,96611\_at,99128\_at,93014\_at | DNA segment, Chr 11, ERATO Doi 497, expressed,mitogen-activated protein kinase kinase 1 interacting protein 1,RIKEN cDNA 5330419I01 gene,ATP synthase, H+ transporting, mitochondrial F0 complex, subunit c (subunit 9), isoform 1,ATP synthase, H+ transporting, mitochondrial F1 complex, gamma polypeptide 1,ATP synthase, H+ transporting, mitochondrial F1 complex, gamma polypeptide 1,ATP synthase, H+ transporting, mitochondrial F1 complex, gamma polypeptide 1,RIKEN cDNA 2410043G19 gene,ATPase, H+ transporting, V1 subunit F,NADH-ubiquinone oxidoreductase subunit B14.7,ATP synthase, H+ transporting, mitochondrial F1 complex, O subunit,ATP synthase, H+ transporting, mitochondrial F0 complex, subunit g |
| 7 | energy coupled proton transport, down the electrochemical gradient | 2 | 5 | 298 | 6246 | 0.007 | 0.001 | 8.387 | 0.021 | 93014\_at,95656\_i\_at | ATP synthase, H+ transporting, mitochondrial F0 complex, subunit g,ATPase, H+ transporting, V1 subunit F |
| 8 | ATP synthesis coupled proton transport | 2 | 5 | 130 | 2164 | 0.015 | 0.002 | 6.658 | 0.032 | 93014\_at,95656\_i\_at | ATP synthase, H+ transporting, mitochondrial F0 complex, subunit g,ATPase, H+ transporting, V1 subunit F |
| 5 | intracellular transport | 31 | 351 | 600 | 11544 | 0.052 | 0.03 | 1.699 | 0.003 | 101254\_at,102821\_s\_at,104322\_at,160076\_at,160256\_at,160543\_at,160876\_at,93993\_at,94014\_at,94210\_at,94277\_at,94323\_at,94506\_at,95091\_at,95441\_at,96668\_at,96849\_at,97477\_at,97478\_at,98930\_at,99147\_at,92636\_f\_at,96670\_at,96734\_at,96947\_at,98959\_at,99156\_at,101061\_at,93970\_at,100618\_f\_at,93084\_at | RAN, member RAS oncogene family,RAS-like, family 2, locus 9,cytoskeleton associated protein 2,metaxin 2,RIKEN cDNA 1110020J08 gene,sorting nexin 3,B-cell receptor-associated protein 29,RIKEN cDNA 1300009F09 gene,RIKEN cDNA 2510048O06 gene,translocase of inner mitochondrial membrane 9 homolog (yeast),metaxin 1,RIKEN cDNA D630012G11 gene,cleavage and polyadenylation specific factor 5,SEC13 related gene (S. cerevisiae),translocase of inner mitochondrial membrane 23 homolog (yeast),translocator of inner mitochondrial membrane b,translocase of inner mitochondrial membrane 8 homolog a (yeast),translocase of inner mitochondrial membrane 8 homolog b (yeast),golgi phosphoprotein 2,coatomer protein complex, subunit epsilon,RIKEN cDNA 2700001K05 gene,SEC61, gamma subunit (S. cerevisiae),RIKEN cDNA 0610025I19 gene,synaptojanin 2 binding protein,RIKEN cDNA 0610009I16 gene,RIKEN cDNA D930048L02 gene,RIKEN cDNA 2700099C19 gene,signal sequence receptor, beta,importin 7,solute carrier family 25 (mitochondrial carrier; adenine nucleotide translocator), member 5,solute carrier family 25 (mitochondrial carrier; adenine nucleotide translocator), member 4 |
| 6 | intracellular protein transport | 29 | 284 | 466 | 9498 | 0.062 | 0.03 | 2.081 | 0 | 101254\_at,102821\_s\_at,104322\_at,160076\_at,160256\_at,160543\_at,160876\_at,93993\_at,94014\_at,94210\_at,94277\_at,94323\_at,94506\_at,95091\_at,95441\_at,96668\_at,96849\_at,97477\_at,97478\_at,98930\_at,99147\_at,92636\_f\_at,96670\_at,96734\_at,96947\_at,98959\_at,99156\_at,101061\_at,93970\_at | RAN, member RAS oncogene family,RAS-like, family 2, locus 9,cytoskeleton associated protein 2,metaxin 2,RIKEN cDNA 1110020J08 gene,sorting nexin 3,B-cell receptor-associated protein 29,RIKEN cDNA 1300009F09 gene,RIKEN cDNA 2510048O06 gene,translocase of inner mitochondrial membrane 9 homolog (yeast),metaxin 1,RIKEN cDNA D630012G11 gene,cleavage and polyadenylation specific factor 5,SEC13 related gene (S. cerevisiae),translocase of inner mitochondrial membrane 23 homolog (yeast),translocator of inner mitochondrial membrane b,translocase of inner mitochondrial membrane 8 homolog a (yeast),translocase of inner mitochondrial membrane 8 homolog b (yeast),golgi phosphoprotein 2,coatomer protein complex, subunit epsilon,RIKEN cDNA 2700001K05 gene,SEC61, gamma subunit (S. cerevisiae),RIKEN cDNA 0610025I19 gene,synaptojanin 2 binding protein,RIKEN cDNA 0610009I16 gene,RIKEN cDNA D930048L02 gene,RIKEN cDNA 2700099C19 gene,signal sequence receptor, beta,importin 7 |
| 7 | protein targeting | 17 | 101 | 298 | 6246 | 0.057 | 0.016 | 3.528 | 0 | 92636\_f\_at,94014\_at,94210\_at,95441\_at,96668\_at,96670\_at,96734\_at,96849\_at,96947\_at,97477\_at,97478\_at,98959\_at,99156\_at,101061\_at,101254\_at,93970\_at,94323\_at | SEC61, gamma subunit (S. cerevisiae),RIKEN cDNA 2510048O06 gene,translocase of inner mitochondrial membrane 9 homolog (yeast),translocase of inner mitochondrial membrane 23 homolog (yeast),translocator of inner mitochondrial membrane b,RIKEN cDNA 0610025I19 gene,synaptojanin 2 binding protein,translocase of inner mitochondrial membrane 8 homolog a (yeast),RIKEN cDNA 0610009I16 gene,translocase of inner mitochondrial membrane 8 homolog b (yeast),golgi phosphoprotein 2,RIKEN cDNA D930048L02 gene,RIKEN cDNA 2700099C19 gene,signal sequence receptor, beta,RAN, member RAS oncogene family,importin 7,RIKEN cDNA D630012G11 gene |
| 8 | protein-mitochondrial targeting | 5 | 6 | 130 | 2164 | 0.038 | 0.003 | 13.884 | 0 | 94014\_at,94210\_at,96849\_at,97477\_at,97478\_at | RIKEN cDNA 2510048O06 gene,translocase of inner mitochondrial membrane 9 homolog (yeast),translocase of inner mitochondrial membrane 8 homolog a (yeast),translocase of inner mitochondrial membrane 8 homolog b (yeast),golgi phosphoprotein 2 |
| 9 | mitochondrial translocation | 5 | 6 | 66 | 911 | 0.076 | 0.007 | 11.496 | 0 | 94014\_at,94210\_at,96849\_at,97477\_at,97478\_at | RIKEN cDNA 2510048O06 gene,translocase of inner mitochondrial membrane 9 homolog (yeast),translocase of inner mitochondrial membrane 8 homolog a (yeast),translocase of inner mitochondrial membrane 8 homolog b (yeast),golgi phosphoprotein 2 |
| 5 | protein transport | 29 | 297 | 600 | 11544 | 0.048 | 0.026 | 1.878 | 0.001 | 101254\_at,102821\_s\_at,104322\_at,160076\_at,160256\_at,160543\_at,160876\_at,93993\_at,94014\_at,94210\_at,94277\_at,94323\_at,94506\_at,95091\_at,95441\_at,96668\_at,96849\_at,97477\_at,97478\_at,98930\_at,99147\_at,92636\_f\_at,96670\_at,96734\_at,96947\_at,98959\_at,99156\_at,101061\_at,93970\_at | RAN, member RAS oncogene family,RAS-like, family 2, locus 9,cytoskeleton associated protein 2,metaxin 2,RIKEN cDNA 1110020J08 gene,sorting nexin 3,B-cell receptor-associated protein 29,RIKEN cDNA 1300009F09 gene,RIKEN cDNA 2510048O06 gene,translocase of inner mitochondrial membrane 9 homolog (yeast),metaxin 1,RIKEN cDNA D630012G11 gene,cleavage and polyadenylation specific factor 5,SEC13 related gene (S. cerevisiae),translocase of inner mitochondrial membrane 23 homolog (yeast),translocator of inner mitochondrial membrane b,translocase of inner mitochondrial membrane 8 homolog a (yeast),translocase of inner mitochondrial membrane 8 homolog b (yeast),golgi phosphoprotein 2,coatomer protein complex, subunit epsilon,RIKEN cDNA 2700001K05 gene,SEC61, gamma subunit (S. cerevisiae),RIKEN cDNA 0610025I19 gene,synaptojanin 2 binding protein,RIKEN cDNA 0610009I16 gene,RIKEN cDNA D930048L02 gene,RIKEN cDNA 2700099C19 gene,signal sequence receptor, beta,importin 7 |
| 6 | intracellular protein transport | 29 | 284 | 466 | 9498 | 0.062 | 0.03 | 2.081 | 0 | 101254\_at,102821\_s\_at,104322\_at,160076\_at,160256\_at,160543\_at,160876\_at,93993\_at,94014\_at,94210\_at,94277\_at,94323\_at,94506\_at,95091\_at,95441\_at,96668\_at,96849\_at,97477\_at,97478\_at,98930\_at,99147\_at,92636\_f\_at,96670\_at,96734\_at,96947\_at,98959\_at,99156\_at,101061\_at,93970\_at | RAN, member RAS oncogene family,RAS-like, family 2, locus 9,cytoskeleton associated protein 2,metaxin 2,RIKEN cDNA 1110020J08 gene,sorting nexin 3,B-cell receptor-associated protein 29,RIKEN cDNA 1300009F09 gene,RIKEN cDNA 2510048O06 gene,translocase of inner mitochondrial membrane 9 homolog (yeast),metaxin 1,RIKEN cDNA D630012G11 gene,cleavage and polyadenylation specific factor 5,SEC13 related gene (S. cerevisiae),translocase of inner mitochondrial membrane 23 homolog (yeast),translocator of inner mitochondrial membrane b,translocase of inner mitochondrial membrane 8 homolog a (yeast),translocase of inner mitochondrial membrane 8 homolog b (yeast),golgi phosphoprotein 2,coatomer protein complex, subunit epsilon,RIKEN cDNA 2700001K05 gene,SEC61, gamma subunit (S. cerevisiae),RIKEN cDNA 0610025I19 gene,synaptojanin 2 binding protein,RIKEN cDNA 0610009I16 gene,RIKEN cDNA D930048L02 gene,RIKEN cDNA 2700099C19 gene,signal sequence receptor, beta,importin 7 |
| 7 | protein targeting | 17 | 101 | 298 | 6246 | 0.057 | 0.016 | 3.528 | 0 | 92636\_f\_at,94014\_at,94210\_at,95441\_at,96668\_at,96670\_at,96734\_at,96849\_at,96947\_at,97477\_at,97478\_at,98959\_at,99156\_at,101061\_at,101254\_at,93970\_at,94323\_at | SEC61, gamma subunit (S. cerevisiae),RIKEN cDNA 2510048O06 gene,translocase of inner mitochondrial membrane 9 homolog (yeast),translocase of inner mitochondrial membrane 23 homolog (yeast),translocator of inner mitochondrial membrane b,RIKEN cDNA 0610025I19 gene,synaptojanin 2 binding protein,translocase of inner mitochondrial membrane 8 homolog a (yeast),RIKEN cDNA 0610009I16 gene,translocase of inner mitochondrial membrane 8 homolog b (yeast),golgi phosphoprotein 2,RIKEN cDNA D930048L02 gene,RIKEN cDNA 2700099C19 gene,signal sequence receptor, beta,RAN, member RAS oncogene family,importin 7,RIKEN cDNA D630012G11 gene |
| 8 | protein-mitochondrial targeting | 5 | 6 | 130 | 2164 | 0.038 | 0.003 | 13.884 | 0 | 94014\_at,94210\_at,96849\_at,97477\_at,97478\_at | RIKEN cDNA 2510048O06 gene,translocase of inner mitochondrial membrane 9 homolog (yeast),translocase of inner mitochondrial membrane 8 homolog a (yeast),translocase of inner mitochondrial membrane 8 homolog b (yeast),golgi phosphoprotein 2 |
| 9 | mitochondrial translocation | 5 | 6 | 66 | 911 | 0.076 | 0.007 | 11.496 | 0 | 94014\_at,94210\_at,96849\_at,97477\_at,97478\_at | RIKEN cDNA 2510048O06 gene,translocase of inner mitochondrial membrane 9 homolog (yeast),translocase of inner mitochondrial membrane 8 homolog a (yeast),translocase of inner mitochondrial membrane 8 homolog b (yeast),golgi phosphoprotein 2 |
| 5 | substrate-bound cell migration | 2 | 3 | 600 | 11544 | 0.003 | 0 | 12.808 | 0.008 | 94267\_i\_at,94268\_f\_at | ubiquitin-like 5,ubiquitin-like 5 |
| 6 | substrate-bound cell migration, cell attachment to substrate | 2 | 1 | 466 | 9498 | 0.004 | 0 | 39 | 0 | 94267\_i\_at,94268\_f\_at | ubiquitin-like 5,ubiquitin-like 5 |
| 6 | mesoderm cell fate determination | 1 | 1 | 466 | 9498 | 0.002 | 0 | 19.545 | 0.049 | 93456\_r\_at | bone morphogenetic protein 4 |
| 4 | myeloid blood cell differentiation | 2 | 7 | 695 | 13100 | 0.003 | 0.001 | 5.434 | 0.049 | 93548\_at,92831\_at | RIKEN cDNA 1190006C12 gene,sideroflexin 1 |
| 4 | myeloid blood cell differentiation | 2 | 7 | 695 | 13100 | 0.003 | 0.001 | 5.434 | 0.049 | 93548\_at,92831\_at | RIKEN cDNA 1190006C12 gene,sideroflexin 1 |
| 3 | sex determination | 1 | 1 | 509 | 10726 | 0.002 | 0 | 21.778 | 0.047 | 160550\_i\_at | mago-nashi homolog, proliferation-associated (Drosophila) |
| 2 | obsolete biological process | 8 | 3 | 537 | 10540 | 0.015 | 0 | 53.214 | 0 | 100577\_at,102409\_at,93008\_at,93999\_at,94313\_at,95049\_at,96029\_at,97200\_f\_at | small nuclear ribonucleoprotein D1,RIKEN cDNA 2010003I05 gene,U6 snRNA-associated SM-like protein 4,small nuclear ribonucleoprotein polypeptide G,U1 small nuclear ribonucleoprotein 1C,small nuclear ribonucleoprotein D2,splicing factor 3a, subunit 3, 60kDa,small nuclear ribonucleoprotein E |
| 3 | mRNA splicing | 8 | 54 | 509 | 10726 | 0.016 | 0.005 | 3.125 | 0.004 | 100577\_at,102409\_at,93008\_at,93999\_at,94313\_at,95049\_at,96029\_at,97200\_f\_at | small nuclear ribonucleoprotein D1,RIKEN cDNA 2010003I05 gene,U6 snRNA-associated SM-like protein 4,small nuclear ribonucleoprotein polypeptide G,U1 small nuclear ribonucleoprotein 1C,small nuclear ribonucleoprotein D2,splicing factor 3a, subunit 3, 60kDa,small nuclear ribonucleoprotein E |
| 2 | physiological processes | 330 | 5866 | 537 | 10540 | 0.615 | 0.557 | 1.104 | 0.003 | 101254\_at,104476\_at,160536\_at,94394\_at,94506\_at,94933\_at,97412\_at,93593\_f\_at,92778\_i\_at,96634\_at,102197\_at,101407\_at,93812\_at,93815\_at,93784\_at,98524\_f\_at,96734\_at,100116\_at,100400\_at,160872\_f\_at,161004\_at,94789\_r\_at,97276\_at,94862\_i\_at,97979\_at,101061\_at,93062\_at,94014\_at,94210\_at,96849\_at,97477\_at,97478\_at,104322\_at,96956\_at,102126\_at,160266\_r\_at,160531\_at,92646\_at,96291\_f\_at,96292\_r\_at,96293\_at,160503\_at,99151\_at,101954\_at,93251\_at,93833\_s\_at,98039\_at,94897\_at,96775\_at,93112\_at,98587\_at,95660\_at,104762\_r\_at,92770\_at,95491\_at,95891\_at,100128\_at,102853\_at,160538\_at,160659\_at,94294\_at,99522\_at,99632\_at,100156\_at,100612\_at,101065\_at,101067\_at,102001\_at,102631\_at,103418\_at,104738\_at,93041\_at,96289\_at,98975\_at,104733\_at,92788\_f\_at,99129\_at,103805\_at,100459\_at,100618\_f\_at,102821\_s\_at,103534\_at,104380\_at,160076\_at,160256\_at,160543\_at,160856\_at,160876\_at,161487\_f\_at,92831\_at,93084\_at,93734\_i\_at,93735\_f\_at,93993\_at,94277\_at,94323\_at,95061\_at,95091\_at,95441\_at,95690\_at,96048\_at,96336\_at,96668\_at,96892\_at,97248\_at,97374\_at,98930\_at,99147\_at,97424\_at,93533\_at,99128\_at,160125\_at,160126\_at,160203\_at,92798\_at,92799\_g\_at,92800\_i\_at,93596\_i\_at,95656\_i\_at,96611\_at,93014\_at,92636\_f\_at,96670\_at,96947\_at,98959\_at,99156\_at,93970\_at,100917\_at,95654\_at,160551\_at,101741\_at,93838\_at,92874\_f\_at,103881\_at,103939\_at,104297\_at,104301\_at,160293\_at,160314\_at,160711\_at,92589\_at,93029\_at,93754\_at,94276\_at,95408\_at,95426\_at,95634\_at,95635\_g\_at,95636\_at,95693\_at,96268\_at,96678\_at,96948\_at,97449\_at,97820\_at,98527\_at,98966\_at,99566\_at,99613\_at,93258\_at,94275\_at,AFFX-GapdhMur/M32599\_3\_at,AFFX-GapdhMur/M32599\_5\_at,97279\_at,96627\_at,97419\_at,104567\_at,92540\_f\_at,160844\_at,97318\_at,93139\_at,99056\_at,97179\_at,103683\_at,161897\_f\_at,104147\_at,103334\_at,98910\_at,93582\_at,102194\_at,95760\_at,96909\_at,102970\_at,92388\_at,101097\_at,101680\_at,102019\_at,160431\_at,92578\_at,93579\_at,94252\_at,94494\_at,94870\_f\_at,94912\_at,95067\_at,95498\_at,97342\_at,97751\_f\_at,97824\_at,97884\_at,98120\_at,98904\_at,99594\_at,95677\_at,92565\_at,93236\_s\_at,93237\_s\_at,95497\_at,92625\_at,92824\_at,97538\_at,99148\_at,93991\_at,95053\_s\_at,93542\_at,100576\_at,100539\_at,94025\_at,95448\_at,100543\_s\_at,101562\_at,104541\_at,95561\_at,96093\_at,96733\_at,99655\_at,93519\_s\_at,100512\_at,100733\_at,101486\_at,101558\_s\_at,101992\_at,102791\_at,92547\_at,93085\_at,93988\_at,94263\_f\_at,94841\_at,96952\_at,97459\_at,98557\_f\_at,93203\_f\_at,94372\_at,162417\_at,103581\_at,100057\_at,100059\_at,100550\_f\_at,100568\_at,103619\_at,103671\_at,93742\_at,93820\_at,95045\_at,95696\_at,96112\_at,96861\_at,97013\_f\_at,98613\_at,99618\_at,100079\_at,94062\_at,96267\_at,96899\_at,96902\_at,95064\_at,95485\_at,99106\_at,160135\_at,94034\_at,99544\_at,96081\_at,101105\_at,93095\_at,96699\_at,93559\_at,160107\_at,160723\_at,93117\_at,96696\_at,100577\_at,102409\_at,93008\_at,93999\_at,95049\_at,96029\_at,97200\_f\_at,99182\_at,160426\_at,93551\_at,98081\_at,95479\_at,95480\_at,161147\_f\_at,98075\_at,93548\_at,102412\_at,103319\_at,103654\_at,95132\_r\_at,95460\_at,97164\_at,98516\_at,160324\_at,100331\_g\_at,97819\_at,99583\_at,97758\_at,104423\_at,96052\_at,104080\_at,98595\_at,98934\_at,100089\_at,101207\_at,160416\_at,160456\_at,92829\_at,98153\_at,99546\_at,93101\_s\_at,97460\_at,101440\_at,95015\_at,161872\_f\_at,103038\_at,93277\_at,102838\_at,93078\_at,96231\_at | RAN, member RAS oncogene family,retinoblastoma-like 1 (p107),Harvey rat sarcoma virus oncogene 1,Harvey rat sarcoma oncogene, subgroup R,cleavage and polyadenylation specific factor 5,cDNA sequence BC008155,RIKEN cDNA 3300001G02 gene,epithelial membrane protein 3,NA,RIKEN cDNA 5730469M10 gene,nucleobindin 2,Friedreich ataxia,chloride channel, nucleotide-sensitive, 1A,RIKEN cDNA 0610041L09 gene,craniofacial development protein 1,RIKEN cDNA 2210039B01 gene,synaptojanin 2 binding protein,RIKEN cDNA 2810417H13 gene,RIKEN cDNA 4921531G14 gene,RIKEN cDNA 2310008H09 gene,RIKEN cDNA 1700097N02 gene,tubulin, beta 5,cytoskeleton-associated protein 1,dynein, cytoplasmic, light chain 2A,protein phosphatase 1, regulatory (inhibitor) subunit 7,signal sequence receptor, beta,mitochondrial ribosomal protein L39,RIKEN cDNA 2510048O06 gene,translocase of inner mitochondrial membrane 9 homolog (yeast),translocase of inner mitochondrial membrane 8 homolog a (yeast),translocase of inner mitochondrial membrane 8 homolog b (yeast),golgi phosphoprotein 2,cytoskeleton associated protein 2,RIKEN cDNA 0610038D11 gene,ribosomal protein S12,RIKEN cDNA 1110064N10 gene,B-cell receptor-associated protein 37,mitochondrial ribosomal protein L23,NA,NA,RIKEN cDNA 2410015N17 gene,fibrillarin,RIKEN cDNA 2610002K22 gene,H2A histone family, member Z,4-nitrophenylphosphatase domain and non-neuronal SNAP25-like protein homolog 1 (C. elegans),histone 1, H2bl,RIKEN cDNA 2410015M20 gene,glutathione peroxidase 4,chromobox homolog 1 (Drosophila HP1 beta),minichromosome maintenance deficient 2 mitotin (S. cerevisiae),nucleosome assembly protein 1-like 1,RIKEN cDNA 0610025L15 gene,RIKEN cDNA 1500015J03 gene,S100 calcium binding protein A6 (calcyclin),Parkinson disease (autosomal recessive, early onset) 7,NA,cell division cycle 2 homolog A (S. pombe),chondroitin sulfate proteoglycan 6,cyclin-dependent kinase 4,RIKEN cDNA 2310057G13 gene,cyclin B2,germ cell-specific gene 2,MAD2 (mitotic arrest deficient, homolog)-like 1 (yeast),minichromosome maintenance deficient 5, cell division cycle 46 (S. cerevisiae),ribonucleotide reductase M1,proliferating cell nuclear antigen,RIKEN cDNA 2010005E08 gene,ribonucleotide reductase M2,Bloom syndrome homolog (human),expressed sequence AU040575,zuotin related factor 2,minichromosome maintenance deficient 4 homolog (S. cerevisiae),stomatin (Epb7.2)-like 2,RIKEN cDNA 2410008G02 gene,centrin 2,centrin 3,CD40 ligand-activated specific transcript 3,nibrin,RAD50 homolog (S. cerevisiae),solute carrier family 25 (mitochondrial carrier; adenine nucleotide translocator), member 5,RAS-like, family 2, locus 9,hemoglobin, beta adult minor chain,solute carrier family 35 (CMP-sialic acid transporter), member 1,metaxin 2,RIKEN cDNA 1110020J08 gene,sorting nexin 3,ubiquitin-like 4,B-cell receptor-associated protein 29,ATP synthase, H+ transporting, mitochondrial F0 complex, subunit c (subunit 9), isoform 1,sideroflexin 1,solute carrier family 25 (mitochondrial carrier; adenine nucleotide translocator), member 4,proteasome (prosome, macropain) 26S subunit, ATPase 3,proteasome (prosome, macropain) 26S subunit, ATPase 3,RIKEN cDNA 1300009F09 gene,metaxin 1,RIKEN cDNA D630012G11 gene,breast carcinoma amplified sequence 2,SEC13 related gene (S. cerevisiae),translocase of inner mitochondrial membrane 23 homolog (yeast),RIKEN cDNA 1110030L07 gene,heat-responsive protein 12,glycine amidinotransferase (L-arginine:glycine amidinotransferase),translocator of inner mitochondrial membrane b,proteasome (prosome, macropain) subunit, alpha type 1,diazepam binding inhibitor,RIKEN cDNA 2810025M15 gene,coatomer protein complex, subunit epsilon,RIKEN cDNA 2700001K05 gene,ADP-ribosylation-like factor 6 interacting protein 5,RIKEN cDNA 1500011L16 gene,ATP synthase, H+ transporting, mitochondrial F1 complex, O subunit,DNA segment, Chr 11, ERATO Doi 497, expressed,mitogen-activated protein kinase kinase 1 interacting protein 1,RIKEN cDNA 5330419I01 gene,ATP synthase, H+ transporting, mitochondrial F1 complex, gamma polypeptide 1,ATP synthase, H+ transporting, mitochondrial F1 complex, gamma polypeptide 1,ATP synthase, H+ transporting, mitochondrial F1 complex, gamma polypeptide 1,RIKEN cDNA 2410043G19 gene,ATPase, H+ transporting, V1 subunit F,NADH-ubiquinone oxidoreductase subunit B14.7,ATP synthase, H+ transporting, mitochondrial F0 complex, subunit g,SEC61, gamma subunit (S. cerevisiae),RIKEN cDNA 0610025I19 gene,RIKEN cDNA 0610009I16 gene,RIKEN cDNA D930048L02 gene,RIKEN cDNA 2700099C19 gene,importin 7,RIKEN cDNA 2410080P20 gene,chloride intracellular channel 1,voltage-dependent anion channel 3,proteasome (prosome, macropain) subunit, beta type 5, pseudogene,RIKEN cDNA 2700038C09 gene,COP9 (constitutive photomorphogenic) homolog, subunit 7a (Arabidopsis thaliana),inorganic pyrophosphatase 2,RIKEN cDNA 2610509I15 gene,RIKEN cDNA 2510001A17 gene,RIKEN cDNA 2610001E06 gene,RIKEN cDNA 2700038L12 gene,RIKEN cDNA 2010317E03 gene,2,4-dienoyl CoA reductase 1, mitochondrial,expressed sequence AI480570,isocitrate dehydrogenase 3 (NAD+), gamma,enoyl coenzyme A hydratase 1, peroxisomal,hydroxysteroid (17-beta) dehydrogenase 12,RIKEN cDNA 2310003F16 gene,enoyl Coenzyme A hydratase, short chain, 1, mitochondrial,RIKEN cDNA 0610010K14 gene,RIKEN cDNA 0610010K14 gene,RIKEN cDNA 0610010K14 gene,NA,succinate-CoA ligase, GDP-forming, alpha subunit,dehydrogenase/reductase (SDR family) member 4,quininoid dihydropteridine reductase,NA,galactokinase 1,dodecenoyl-Coenzyme A delta isomerase (3,2 trans-enoyl-Coenyme A isomerase),dihydrolipoamide branched chain transacylase E2,triosephosphate isomerase,methylmalonyl-Coenzyme A mutase,hydroxymethylbilane synthase,uroporphyrinogen decarboxylase,glyceraldehyde-3-phosphate dehydrogenase,glyceraldehyde-3-phosphate dehydrogenase,RIKEN cDNA 6430402H10 gene,phenylalkylamine Ca2+ antagonist (emopamil) binding protein,RIKEN cDNA 2310010I22 gene,RIKEN cDNA 3110052F15 gene,spermidine synthase,6-pyruvoyl-tetrahydropterin synthase,histidyl tRNA synthetase 2,NA,6-pyruvoyl-tetrahydropterin synthase/dimerization cofactor of hepatocyte nuclear factor 1 alpha (TCF1),expressed sequence C76683,dihydroorotate dehydrogenase,phosphoribosyl pyrophosphate synthetase 1,N-acetylneuraminic acid synthase (sialic acid synthase),calcitonin gene-related peptide-receptor component protein,RIKEN cDNA 1110014K08 gene,demethyl-Q 7,RIKEN cDNA 2810432D09 gene,RIKEN cDNA 1110011K10 gene,NADH dehydrogenase (ubiquinone) 1, alpha/beta subcomplex, 1,proteasome (prosome, macropain) 26S subunit, ATPase 3, interacting protein,NA,DNA segment, Chr 9, Wayne State University 149,ribosomal protein L27a,mitochondrial ribosomal protein L13,mitochondrial ribosomal protein L12,small inducible cytokine subfamily E, member 1,RIKEN cDNA 5830427H10 gene,eukaryotic translation initiation factor 2, subunit 3, structural gene X-linked,phenylalanine-tRNA synthetase-like,RIKEN cDNA 2310075M17 gene,mitochondrial ribosomal protein S21,mitochondrial ribosomal protein L2,mitochondrial ribosomal protein S15,mitochondrial ribosomal protein S14,NA,DNA segment, Chr 11, ERATO Doi 175, expressed,mitochondrial ribosomal protein S11,mitochondrial ribosomal protein L27,mitochondrial ribosomal protein L35,mitochondrial ribosomal protein L51,U5 snRNP-specific protein (Prp8-binding),RIKEN cDNA 1110005A23 gene,thymidylate synthase,thymidylate synthase,RIKEN cDNA 1110005A05 gene,expressed in non-metastatic cells 2, protein,expressed in non-metastatic cells 6, protein,beta-glucuronidase,fumarate hydratase 1,malate dehydrogenase, mitochondrial,succinate dehydrogenase complex, subunit B, iron sulfur (Ip),phosphotriesterase related,platelet-activating factor acetylhydrolase, isoform 1b, alpha1 subunit,brain acyl-CoA hydrolase,proteasome (prosome, macropain) subunit, beta type 3,proteasome (prosome, macropain) 26S subunit, ATPase 2,bromodomain containing 7,heat shock protein 4,proteinase 3,RIKEN cDNA 1700013H19 gene,open reading frame 11,RAP1, GTP-GDP dissociation stimulator 1,signal peptidase complex,neural precursor cell expressed, developmentally down-regulated gene 8,ubiquitin carboxyl-terminal esterase L5,proteasome (prosome, macropain) subunit, alpha type 2,proteasome (prosome, macropain) subunit, beta type 10,proteasome (prosome, macropain) subunit, beta type 5,proteasome (prosome, macropain) subunit, beta type 6,proteosome (prosome, macropain) subunit, beta type 8 (large multifunctional protease 7),huntingtin interacting protein 2,proteosome (prosome, macropain) subunit, beta type 9 (large multifunctional protease 2),proteasome (prosome, macropain) subunit, alpha type 7,proteasome (prosome, macropain) subunit, beta type 7,proteasome (prosome, macropain) subunit, alpha type 5,proteasome (prosome, macropain) subunit, alpha type 6,proteasome (prosome, macropain) subunit, alpha type 4,proteasome (prosome, macropain) subunit, beta type 4,RIKEN cDNA 3230402J05 gene,nudix (nucleoside diphosphate linked moiety X)-type motif 1,RIKEN cDNA 1500001M20 gene,cytosolic acyl-CoA thioesterase 1,RIKEN cDNA 2510027N19 gene,cytochrome b-245, alpha polypeptide,cytochrome c oxidase, subunit VIc,ATP-binding cassette, sub-family E (OABP), member 1,cytochrome b5 outer mitochondrial membrane precursor,HIV-1 tat interactive protein 2, homolog (human),RIKEN cDNA 5730449L18 gene,cytochrome c oxidase, subunit VIIa 2,RIKEN cDNA 0610012D09 gene,thioredoxin-like 2,electron transferring flavoprotein, alpha polypeptide,mitochondrial ribosomal protein L50,cytochrome b-245, alpha polypeptide,RIKEN cDNA 2700085E05 gene,ubiquinol-cytochrome c reductase subunit,NADH dehydrogenase (ubiquinone) 1 beta subcomplex, 9,NADH dehydrogenase (ubiquinone) flavoprotein 2,NADH dehydrogenase (ubiquinone) flavoprotein 1,NADH dehydrogenase (ubiquinone) Fe-S protein 3,RIKEN cDNA 2900091E11 gene,acetyl-Coenzyme A acyltransferase 2 (mitochondrial 3-oxoacyl-Coenzyme A thiolase),L-3-hydroxyacyl-Coenzyme A dehydrogenase, short chain,COP9 (constitutive photomorphogenic) homolog, subunit 6 (Arabidopsis thaliana),DNA segment, Chr 16, ERATO Doi 502, expressed,small fragment nuclease,deoxyguanosine kinase,thymidine kinase 1,barrier to autointegration factor 1,high mobility group box 1,high mobility group nucleosomal binding domain 1,apurinic/apyrimidinic endonuclease 1,hypoxanthine guanine phosphoribosyl transferase,RIKEN cDNA 1500001M20 gene,heterogeneous nuclear ribonucleoprotein A2/B1,NA,small nuclear ribonucleoprotein D1,RIKEN cDNA 2010003I05 gene,U6 snRNA-associated SM-like protein 4,small nuclear ribonucleoprotein polypeptide G,small nuclear ribonucleoprotein D2,splicing factor 3a, subunit 3, 60kDa,small nuclear ribonucleoprotein E,RIKEN cDNA 2610511E03 gene,RNA polymerase 1-1,RIKEN cDNA 2510029B14 gene,RNA polymerase 1-3,nuclear DNA binding protein,NA,RIKEN cDNA 2310061C15 gene,RIKEN cDNA G431001I09 gene,RIKEN cDNA 1190006C12 gene,expressed sequence AW541137,proteasome (prosome, macropain) 26S subunit, non-ATPase, 10,nucleosome binding protein 1,NADH dehydrogenase (ubiquinone) 1 beta subcomplex, 2,COP9 (constitutive photomorphogenic) homolog, subunit 5 (Arabidopsis thaliana),RIKEN cDNA 2610207P08 gene,NA,replication protein A3,thioredoxin peroxidase, pseudogene 1,glutathione S-transferase omega 1,glutathione S-transferase, pi 2,peroxiredoxin 1,RIKEN cDNA 2810047L02 gene,acid phosphatase 1, soluble,PDGFA associated protein 1,interleukin-1 receptor-associated kinase 1,RIKEN cDNA 0610007P06 gene,peptidylprolyl isomerase C,peptidylprolyl isomerase A,FK506 binding protein 3,RIKEN cDNA 1100001J08 gene,heat shock protein 1 (chaperonin 10),chaperonin subunit 3 (gamma),FK506 binding protein 2,neural precursor cell expressed, developmentally down-regulted gene 4,ubiquitin-conjugating enzyme E2R 2,RIKEN cDNA 2900006F19 gene,aldo-keto reductase family 1, member C13,RIKEN cDNA 1110049G11 gene,guanylate cyclase activator 1a (retina),heat shock protein 1 (chaperonin),selectin, lymphocyte,lymphocyte antigen 6 complex, locus A,RIKEN cDNA 2010012D11 gene |
| 3 | cell growth and/or maintenance | 130 | 2128 | 509 | 10726 | 0.255 | 0.198 | 1.287 | 0.001 | 101254\_at,104476\_at,160536\_at,94394\_at,94506\_at,94933\_at,97412\_at,93593\_f\_at,92778\_i\_at,96634\_at,102197\_at,101407\_at,93812\_at,93815\_at,93784\_at,98524\_f\_at,96734\_at,100116\_at,100400\_at,160872\_f\_at,161004\_at,94789\_r\_at,97276\_at,94862\_i\_at,97979\_at,101061\_at,93062\_at,94014\_at,94210\_at,96849\_at,97477\_at,97478\_at,104322\_at,96956\_at,102126\_at,160266\_r\_at,160531\_at,92646\_at,96291\_f\_at,96292\_r\_at,96293\_at,160503\_at,99151\_at,101954\_at,93251\_at,93833\_s\_at,98039\_at,94897\_at,96775\_at,93112\_at,98587\_at,95660\_at,104762\_r\_at,92770\_at,95491\_at,95891\_at,100128\_at,102853\_at,160538\_at,160659\_at,94294\_at,99522\_at,99632\_at,100156\_at,100612\_at,101065\_at,101067\_at,102001\_at,102631\_at,103418\_at,104738\_at,93041\_at,96289\_at,98975\_at,104733\_at,92788\_f\_at,99129\_at,103805\_at,100459\_at,100618\_f\_at,102821\_s\_at,103534\_at,104380\_at,160076\_at,160256\_at,160543\_at,160856\_at,160876\_at,161487\_f\_at,92831\_at,93084\_at,93734\_i\_at,93735\_f\_at,93993\_at,94277\_at,94323\_at,95061\_at,95091\_at,95441\_at,95690\_at,96048\_at,96336\_at,96668\_at,96892\_at,97248\_at,97374\_at,98930\_at,99147\_at,97424\_at,93533\_at,99128\_at,160125\_at,160126\_at,160203\_at,92798\_at,92799\_g\_at,92800\_i\_at,93596\_i\_at,95656\_i\_at,96611\_at,93014\_at,92636\_f\_at,96670\_at,96947\_at,98959\_at,99156\_at,93970\_at,100917\_at,95654\_at,160551\_at | RAN, member RAS oncogene family,retinoblastoma-like 1 (p107),Harvey rat sarcoma virus oncogene 1,Harvey rat sarcoma oncogene, subgroup R,cleavage and polyadenylation specific factor 5,cDNA sequence BC008155,RIKEN cDNA 3300001G02 gene,epithelial membrane protein 3,NA,RIKEN cDNA 5730469M10 gene,nucleobindin 2,Friedreich ataxia,chloride channel, nucleotide-sensitive, 1A,RIKEN cDNA 0610041L09 gene,craniofacial development protein 1,RIKEN cDNA 2210039B01 gene,synaptojanin 2 binding protein,RIKEN cDNA 2810417H13 gene,RIKEN cDNA 4921531G14 gene,RIKEN cDNA 2310008H09 gene,RIKEN cDNA 1700097N02 gene,tubulin, beta 5,cytoskeleton-associated protein 1,dynein, cytoplasmic, light chain 2A,protein phosphatase 1, regulatory (inhibitor) subunit 7,signal sequence receptor, beta,mitochondrial ribosomal protein L39,RIKEN cDNA 2510048O06 gene,translocase of inner mitochondrial membrane 9 homolog (yeast),translocase of inner mitochondrial membrane 8 homolog a (yeast),translocase of inner mitochondrial membrane 8 homolog b (yeast),golgi phosphoprotein 2,cytoskeleton associated protein 2,RIKEN cDNA 0610038D11 gene,ribosomal protein S12,RIKEN cDNA 1110064N10 gene,B-cell receptor-associated protein 37,mitochondrial ribosomal protein L23,NA,NA,RIKEN cDNA 2410015N17 gene,fibrillarin,RIKEN cDNA 2610002K22 gene,H2A histone family, member Z,4-nitrophenylphosphatase domain and non-neuronal SNAP25-like protein homolog 1 (C. elegans),histone 1, H2bl,RIKEN cDNA 2410015M20 gene,glutathione peroxidase 4,chromobox homolog 1 (Drosophila HP1 beta),minichromosome maintenance deficient 2 mitotin (S. cerevisiae),nucleosome assembly protein 1-like 1,RIKEN cDNA 0610025L15 gene,RIKEN cDNA 1500015J03 gene,S100 calcium binding protein A6 (calcyclin),Parkinson disease (autosomal recessive, early onset) 7,NA,cell division cycle 2 homolog A (S. pombe),chondroitin sulfate proteoglycan 6,cyclin-dependent kinase 4,RIKEN cDNA 2310057G13 gene,cyclin B2,germ cell-specific gene 2,MAD2 (mitotic arrest deficient, homolog)-like 1 (yeast),minichromosome maintenance deficient 5, cell division cycle 46 (S. cerevisiae),ribonucleotide reductase M1,proliferating cell nuclear antigen,RIKEN cDNA 2010005E08 gene,ribonucleotide reductase M2,Bloom syndrome homolog (human),expressed sequence AU040575,zuotin related factor 2,minichromosome maintenance deficient 4 homolog (S. cerevisiae),stomatin (Epb7.2)-like 2,RIKEN cDNA 2410008G02 gene,centrin 2,centrin 3,CD40 ligand-activated specific transcript 3,nibrin,RAD50 homolog (S. cerevisiae),solute carrier family 25 (mitochondrial carrier; adenine nucleotide translocator), member 5,RAS-like, family 2, locus 9,hemoglobin, beta adult minor chain,solute carrier family 35 (CMP-sialic acid transporter), member 1,metaxin 2,RIKEN cDNA 1110020J08 gene,sorting nexin 3,ubiquitin-like 4,B-cell receptor-associated protein 29,ATP synthase, H+ transporting, mitochondrial F0 complex, subunit c (subunit 9), isoform 1,sideroflexin 1,solute carrier family 25 (mitochondrial carrier; adenine nucleotide translocator), member 4,proteasome (prosome, macropain) 26S subunit, ATPase 3,proteasome (prosome, macropain) 26S subunit, ATPase 3,RIKEN cDNA 1300009F09 gene,metaxin 1,RIKEN cDNA D630012G11 gene,breast carcinoma amplified sequence 2,SEC13 related gene (S. cerevisiae),translocase of inner mitochondrial membrane 23 homolog (yeast),RIKEN cDNA 1110030L07 gene,heat-responsive protein 12,glycine amidinotransferase (L-arginine:glycine amidinotransferase),translocator of inner mitochondrial membrane b,proteasome (prosome, macropain) subunit, alpha type 1,diazepam binding inhibitor,RIKEN cDNA 2810025M15 gene,coatomer protein complex, subunit epsilon,RIKEN cDNA 2700001K05 gene,ADP-ribosylation-like factor 6 interacting protein 5,RIKEN cDNA 1500011L16 gene,ATP synthase, H+ transporting, mitochondrial F1 complex, O subunit,DNA segment, Chr 11, ERATO Doi 497, expressed,mitogen-activated protein kinase kinase 1 interacting protein 1,RIKEN cDNA 5330419I01 gene,ATP synthase, H+ transporting, mitochondrial F1 complex, gamma polypeptide 1,ATP synthase, H+ transporting, mitochondrial F1 complex, gamma polypeptide 1,ATP synthase, H+ transporting, mitochondrial F1 complex, gamma polypeptide 1,RIKEN cDNA 2410043G19 gene,ATPase, H+ transporting, V1 subunit F,NADH-ubiquinone oxidoreductase subunit B14.7,ATP synthase, H+ transporting, mitochondrial F0 complex, subunit g,SEC61, gamma subunit (S. cerevisiae),RIKEN cDNA 0610025I19 gene,RIKEN cDNA 0610009I16 gene,RIKEN cDNA D930048L02 gene,RIKEN cDNA 2700099C19 gene,importin 7,RIKEN cDNA 2410080P20 gene,chloride intracellular channel 1,voltage-dependent anion channel 3 |
| 5 | regulation of cell volume | 2 | 1 | 600 | 11544 | 0.003 | 0 | 37 | 0 | 93812\_at,93815\_at | chloride channel, nucleotide-sensitive, 1A,RIKEN cDNA 0610041L09 gene |
| 4 | cell organization and biogenesis | 39 | 530 | 695 | 13100 | 0.056 | 0.04 | 1.387 | 0.024 | 93784\_at,98524\_f\_at,96734\_at,100116\_at,100400\_at,160872\_f\_at,161004\_at,94789\_r\_at,97276\_at,101254\_at,94862\_i\_at,97979\_at,101061\_at,93062\_at,94014\_at,94210\_at,96849\_at,97477\_at,97478\_at,104322\_at,96956\_at,102126\_at,160266\_r\_at,160531\_at,92646\_at,96291\_f\_at,96292\_r\_at,96293\_at,160503\_at,99151\_at,101954\_at,93251\_at,93833\_s\_at,98039\_at,94897\_at,96775\_at,93112\_at,98587\_at,95660\_at | craniofacial development protein 1,RIKEN cDNA 2210039B01 gene,synaptojanin 2 binding protein,RIKEN cDNA 2810417H13 gene,RIKEN cDNA 4921531G14 gene,RIKEN cDNA 2310008H09 gene,RIKEN cDNA 1700097N02 gene,tubulin, beta 5,cytoskeleton-associated protein 1,RAN, member RAS oncogene family,dynein, cytoplasmic, light chain 2A,protein phosphatase 1, regulatory (inhibitor) subunit 7,signal sequence receptor, beta,mitochondrial ribosomal protein L39,RIKEN cDNA 2510048O06 gene,translocase of inner mitochondrial membrane 9 homolog (yeast),translocase of inner mitochondrial membrane 8 homolog a (yeast),translocase of inner mitochondrial membrane 8 homolog b (yeast),golgi phosphoprotein 2,cytoskeleton associated protein 2,RIKEN cDNA 0610038D11 gene,ribosomal protein S12,RIKEN cDNA 1110064N10 gene,B-cell receptor-associated protein 37,mitochondrial ribosomal protein L23,NA,NA,RIKEN cDNA 2410015N17 gene,fibrillarin,RIKEN cDNA 2610002K22 gene,H2A histone family, member Z,4-nitrophenylphosphatase domain and non-neuronal SNAP25-like protein homolog 1 (C. elegans),histone 1, H2bl,RIKEN cDNA 2410015M20 gene,glutathione peroxidase 4,chromobox homolog 1 (Drosophila HP1 beta),minichromosome maintenance deficient 2 mitotin (S. cerevisiae),nucleosome assembly protein 1-like 1,RIKEN cDNA 0610025L15 gene |
| 5 | cytoplasm organization and biogenesis | 29 | 380 | 600 | 11544 | 0.048 | 0.033 | 1.468 | 0.024 | 96734\_at,100116\_at,100400\_at,160872\_f\_at,161004\_at,94789\_r\_at,97276\_at,101254\_at,94862\_i\_at,97979\_at,101061\_at,93062\_at,94014\_at,94210\_at,96849\_at,97477\_at,97478\_at,104322\_at,96956\_at,102126\_at,160266\_r\_at,160531\_at,92646\_at,96291\_f\_at,96292\_r\_at,96293\_at,98524\_f\_at,160503\_at,99151\_at | synaptojanin 2 binding protein,RIKEN cDNA 2810417H13 gene,RIKEN cDNA 4921531G14 gene,RIKEN cDNA 2310008H09 gene,RIKEN cDNA 1700097N02 gene,tubulin, beta 5,cytoskeleton-associated protein 1,RAN, member RAS oncogene family,dynein, cytoplasmic, light chain 2A,protein phosphatase 1, regulatory (inhibitor) subunit 7,signal sequence receptor, beta,mitochondrial ribosomal protein L39,RIKEN cDNA 2510048O06 gene,translocase of inner mitochondrial membrane 9 homolog (yeast),translocase of inner mitochondrial membrane 8 homolog a (yeast),translocase of inner mitochondrial membrane 8 homolog b (yeast),golgi phosphoprotein 2,cytoskeleton associated protein 2,RIKEN cDNA 0610038D11 gene,ribosomal protein S12,RIKEN cDNA 1110064N10 gene,B-cell receptor-associated protein 37,mitochondrial ribosomal protein L23,NA,NA,RIKEN cDNA 2410015N17 gene,RIKEN cDNA 2210039B01 gene,fibrillarin,RIKEN cDNA 2610002K22 gene |
| 7 | mitochondrion organization and biogenesis | 6 | 13 | 298 | 6246 | 0.02 | 0.002 | 9.678 | 0 | 93062\_at,94014\_at,94210\_at,96849\_at,97477\_at,97478\_at | mitochondrial ribosomal protein L39,RIKEN cDNA 2510048O06 gene,translocase of inner mitochondrial membrane 9 homolog (yeast),translocase of inner mitochondrial membrane 8 homolog a (yeast),translocase of inner mitochondrial membrane 8 homolog b (yeast),golgi phosphoprotein 2 |
| 8 | protein-mitochondrial targeting | 5 | 6 | 130 | 2164 | 0.038 | 0.003 | 13.884 | 0 | 94014\_at,94210\_at,96849\_at,97477\_at,97478\_at | RIKEN cDNA 2510048O06 gene,translocase of inner mitochondrial membrane 9 homolog (yeast),translocase of inner mitochondrial membrane 8 homolog a (yeast),translocase of inner mitochondrial membrane 8 homolog b (yeast),golgi phosphoprotein 2 |
| 9 | mitochondrial translocation | 5 | 6 | 66 | 911 | 0.076 | 0.007 | 11.496 | 0 | 94014\_at,94210\_at,96849\_at,97477\_at,97478\_at | RIKEN cDNA 2510048O06 gene,translocase of inner mitochondrial membrane 9 homolog (yeast),translocase of inner mitochondrial membrane 8 homolog a (yeast),translocase of inner mitochondrial membrane 8 homolog b (yeast),golgi phosphoprotein 2 |
| 6 | ribosome biogenesis and assembly | 10 | 60 | 466 | 9498 | 0.021 | 0.006 | 3.396 | 0.001 | 102126\_at,160266\_r\_at,160531\_at,92646\_at,96291\_f\_at,96292\_r\_at,96293\_at,98524\_f\_at,160503\_at,99151\_at | ribosomal protein S12,RIKEN cDNA 1110064N10 gene,B-cell receptor-associated protein 37,mitochondrial ribosomal protein L23,NA,NA,RIKEN cDNA 2410015N17 gene,RIKEN cDNA 2210039B01 gene,fibrillarin,RIKEN cDNA 2610002K22 gene |
| 7 | ribosome biogenesis | 10 | 60 | 298 | 6246 | 0.034 | 0.01 | 3.492 | 0 | 102126\_at,160266\_r\_at,160531\_at,92646\_at,96291\_f\_at,96292\_r\_at,96293\_at,98524\_f\_at,160503\_at,99151\_at | ribosomal protein S12,RIKEN cDNA 1110064N10 gene,B-cell receptor-associated protein 37,mitochondrial ribosomal protein L23,NA,NA,RIKEN cDNA 2410015N17 gene,RIKEN cDNA 2210039B01 gene,fibrillarin,RIKEN cDNA 2610002K22 gene |
| 8 | rRNA processing | 3 | 14 | 130 | 2164 | 0.023 | 0.006 | 3.567 | 0.047 | 160503\_at,160531\_at,99151\_at | fibrillarin,B-cell receptor-associated protein 37,RIKEN cDNA 2610002K22 gene |
| 5 | cell cycle | 32 | 435 | 600 | 11544 | 0.053 | 0.038 | 1.415 | 0.03 | 100128\_at,102853\_at,104476\_at,160538\_at,160659\_at,92770\_at,93112\_at,94294\_at,99522\_at,99632\_at,100156\_at,100612\_at,101065\_at,101067\_at,102001\_at,102631\_at,103418\_at,104738\_at,93041\_at,96289\_at,98975\_at,101254\_at,104733\_at,92788\_f\_at,99129\_at,160536\_at,94394\_at,94506\_at,94933\_at,95891\_at,97412\_at,103805\_at | cell division cycle 2 homolog A (S. pombe),chondroitin sulfate proteoglycan 6,retinoblastoma-like 1 (p107),cyclin-dependent kinase 4,RIKEN cDNA 2310057G13 gene,S100 calcium binding protein A6 (calcyclin),minichromosome maintenance deficient 2 mitotin (S. cerevisiae),cyclin B2,germ cell-specific gene 2,MAD2 (mitotic arrest deficient, homolog)-like 1 (yeast),minichromosome maintenance deficient 5, cell division cycle 46 (S. cerevisiae),ribonucleotide reductase M1,proliferating cell nuclear antigen,RIKEN cDNA 2010005E08 gene,ribonucleotide reductase M2,Bloom syndrome homolog (human),expressed sequence AU040575,zuotin related factor 2,minichromosome maintenance deficient 4 homolog (S. cerevisiae),stomatin (Epb7.2)-like 2,RIKEN cDNA 2410008G02 gene,RAN, member RAS oncogene family,centrin 2,centrin 3,CD40 ligand-activated specific transcript 3,Harvey rat sarcoma virus oncogene 1,Harvey rat sarcoma oncogene, subgroup R,cleavage and polyadenylation specific factor 5,cDNA sequence BC008155,NA,RIKEN cDNA 3300001G02 gene,nibrin |
| 6 | DNA replication and chromosome cycle | 14 | 113 | 466 | 9498 | 0.03 | 0.012 | 2.524 | 0.001 | 102853\_at,99632\_at,100156\_at,100612\_at,101065\_at,101067\_at,102001\_at,102631\_at,103418\_at,104738\_at,93041\_at,93112\_at,96289\_at,98975\_at | chondroitin sulfate proteoglycan 6,MAD2 (mitotic arrest deficient, homolog)-like 1 (yeast),minichromosome maintenance deficient 5, cell division cycle 46 (S. cerevisiae),ribonucleotide reductase M1,proliferating cell nuclear antigen,RIKEN cDNA 2010005E08 gene,ribonucleotide reductase M2,Bloom syndrome homolog (human),expressed sequence AU040575,zuotin related factor 2,minichromosome maintenance deficient 4 homolog (S. cerevisiae),minichromosome maintenance deficient 2 mitotin (S. cerevisiae),stomatin (Epb7.2)-like 2,RIKEN cDNA 2410008G02 gene |
| 7 | DNA replication | 12 | 94 | 298 | 6246 | 0.04 | 0.015 | 2.676 | 0.002 | 100156\_at,100612\_at,101065\_at,101067\_at,102001\_at,102631\_at,103418\_at,104738\_at,93041\_at,93112\_at,96289\_at,98975\_at | minichromosome maintenance deficient 5, cell division cycle 46 (S. cerevisiae),ribonucleotide reductase M1,proliferating cell nuclear antigen,RIKEN cDNA 2010005E08 gene,ribonucleotide reductase M2,Bloom syndrome homolog (human),expressed sequence AU040575,zuotin related factor 2,minichromosome maintenance deficient 4 homolog (S. cerevisiae),minichromosome maintenance deficient 2 mitotin (S. cerevisiae),stomatin (Epb7.2)-like 2,RIKEN cDNA 2410008G02 gene |
| 8 | DNA dependent DNA replication | 7 | 31 | 130 | 2164 | 0.054 | 0.014 | 3.758 | 0.002 | 100156\_at,93041\_at,93112\_at,103418\_at,98975\_at,101065\_at,101067\_at | minichromosome maintenance deficient 5, cell division cycle 46 (S. cerevisiae),minichromosome maintenance deficient 4 homolog (S. cerevisiae),minichromosome maintenance deficient 2 mitotin (S. cerevisiae),expressed sequence AU040575,RIKEN cDNA 2410008G02 gene,proliferating cell nuclear antigen,RIKEN cDNA 2010005E08 gene |
| 9 | DNA replication initiation | 3 | 10 | 66 | 911 | 0.045 | 0.011 | 4.139 | 0.03 | 100156\_at,93041\_at,93112\_at | minichromosome maintenance deficient 5, cell division cycle 46 (S. cerevisiae),minichromosome maintenance deficient 4 homolog (S. cerevisiae),minichromosome maintenance deficient 2 mitotin (S. cerevisiae) |
| 9 | regulation of DNA replication | 2 | 1 | 66 | 911 | 0.03 | 0.001 | 27.545 | 0 | 101065\_at,101067\_at | proliferating cell nuclear antigen,RIKEN cDNA 2010005E08 gene |
| 6 | M phase | 8 | 74 | 466 | 9498 | 0.017 | 0.008 | 2.204 | 0.028 | 101254\_at,100128\_at,102853\_at,104733\_at,92788\_f\_at,94294\_at,99632\_at,99129\_at | RAN, member RAS oncogene family,cell division cycle 2 homolog A (S. pombe),chondroitin sulfate proteoglycan 6,centrin 2,centrin 3,cyclin B2,MAD2 (mitotic arrest deficient, homolog)-like 1 (yeast),CD40 ligand-activated specific transcript 3 |
| 7 | M phase of mitotic cell cycle | 8 | 57 | 298 | 6246 | 0.027 | 0.009 | 2.941 | 0.005 | 100128\_at,101254\_at,102853\_at,104733\_at,92788\_f\_at,94294\_at,99632\_at,99129\_at | cell division cycle 2 homolog A (S. pombe),RAN, member RAS oncogene family,chondroitin sulfate proteoglycan 6,centrin 2,centrin 3,cyclin B2,MAD2 (mitotic arrest deficient, homolog)-like 1 (yeast),CD40 ligand-activated specific transcript 3 |
| 8 | mitosis | 8 | 57 | 130 | 2164 | 0.062 | 0.026 | 2.336 | 0.019 | 100128\_at,101254\_at,102853\_at,104733\_at,92788\_f\_at,94294\_at,99632\_at,99129\_at | cell division cycle 2 homolog A (S. pombe),RAN, member RAS oncogene family,chondroitin sulfate proteoglycan 6,centrin 2,centrin 3,cyclin B2,MAD2 (mitotic arrest deficient, homolog)-like 1 (yeast),CD40 ligand-activated specific transcript 3 |
| 10 | regulation of mitotic metaphase/anaphase transition | 1 | 1 | 5 | 197 | 0.2 | 0.005 | 39.37 | 0.025 | 99632\_at | MAD2 (mitotic arrest deficient, homolog)-like 1 (yeast) |
| 9 | regulation of mitosis | 2 | 3 | 66 | 911 | 0.03 | 0.003 | 9.21 | 0.015 | 99632\_at,99129\_at | MAD2 (mitotic arrest deficient, homolog)-like 1 (yeast),CD40 ligand-activated specific transcript 3 |
| 10 | regulation of mitotic metaphase/anaphase transition | 1 | 1 | 5 | 197 | 0.2 | 0.005 | 39.37 | 0.025 | 99632\_at | MAD2 (mitotic arrest deficient, homolog)-like 1 (yeast) |
| 10 | mitotic checkpoint | 2 | 3 | 5 | 197 | 0.4 | 0.015 | 26.264 | 0.002 | 99129\_at,99632\_at | CD40 ligand-activated specific transcript 3,MAD2 (mitotic arrest deficient, homolog)-like 1 (yeast) |
| 11 | mitotic spindle checkpoint | 2 | 2 | 4 | 34 | 0.5 | 0.059 | 8.501 | 0.011 | 99129\_at,99632\_at | CD40 ligand-activated specific transcript 3,MAD2 (mitotic arrest deficient, homolog)-like 1 (yeast) |
| 7 | nuclear division | 8 | 73 | 298 | 6246 | 0.027 | 0.012 | 2.297 | 0.022 | 100128\_at,101254\_at,102853\_at,104733\_at,92788\_f\_at,94294\_at,99632\_at,99129\_at | cell division cycle 2 homolog A (S. pombe),RAN, member RAS oncogene family,chondroitin sulfate proteoglycan 6,centrin 2,centrin 3,cyclin B2,MAD2 (mitotic arrest deficient, homolog)-like 1 (yeast),CD40 ligand-activated specific transcript 3 |
| 8 | mitosis | 8 | 57 | 130 | 2164 | 0.062 | 0.026 | 2.336 | 0.019 | 100128\_at,101254\_at,102853\_at,104733\_at,92788\_f\_at,94294\_at,99632\_at,99129\_at | cell division cycle 2 homolog A (S. pombe),RAN, member RAS oncogene family,chondroitin sulfate proteoglycan 6,centrin 2,centrin 3,cyclin B2,MAD2 (mitotic arrest deficient, homolog)-like 1 (yeast),CD40 ligand-activated specific transcript 3 |
| 10 | regulation of mitotic metaphase/anaphase transition | 1 | 1 | 5 | 197 | 0.2 | 0.005 | 39.37 | 0.025 | 99632\_at | MAD2 (mitotic arrest deficient, homolog)-like 1 (yeast) |
| 9 | regulation of mitosis | 2 | 3 | 66 | 911 | 0.03 | 0.003 | 9.21 | 0.015 | 99632\_at,99129\_at | MAD2 (mitotic arrest deficient, homolog)-like 1 (yeast),CD40 ligand-activated specific transcript 3 |
| 10 | regulation of mitotic metaphase/anaphase transition | 1 | 1 | 5 | 197 | 0.2 | 0.005 | 39.37 | 0.025 | 99632\_at | MAD2 (mitotic arrest deficient, homolog)-like 1 (yeast) |
| 10 | mitotic checkpoint | 2 | 3 | 5 | 197 | 0.4 | 0.015 | 26.264 | 0.002 | 99129\_at,99632\_at | CD40 ligand-activated specific transcript 3,MAD2 (mitotic arrest deficient, homolog)-like 1 (yeast) |
| 11 | mitotic spindle checkpoint | 2 | 2 | 4 | 34 | 0.5 | 0.059 | 8.501 | 0.011 | 99129\_at,99632\_at | CD40 ligand-activated specific transcript 3,MAD2 (mitotic arrest deficient, homolog)-like 1 (yeast) |
| 7 | M phase of mitotic cell cycle | 8 | 57 | 298 | 6246 | 0.027 | 0.009 | 2.941 | 0.005 | 100128\_at,101254\_at,102853\_at,104733\_at,92788\_f\_at,94294\_at,99632\_at,99129\_at | cell division cycle 2 homolog A (S. pombe),RAN, member RAS oncogene family,chondroitin sulfate proteoglycan 6,centrin 2,centrin 3,cyclin B2,MAD2 (mitotic arrest deficient, homolog)-like 1 (yeast),CD40 ligand-activated specific transcript 3 |
| 8 | mitosis | 8 | 57 | 130 | 2164 | 0.062 | 0.026 | 2.336 | 0.019 | 100128\_at,101254\_at,102853\_at,104733\_at,92788\_f\_at,94294\_at,99632\_at,99129\_at | cell division cycle 2 homolog A (S. pombe),RAN, member RAS oncogene family,chondroitin sulfate proteoglycan 6,centrin 2,centrin 3,cyclin B2,MAD2 (mitotic arrest deficient, homolog)-like 1 (yeast),CD40 ligand-activated specific transcript 3 |
| 10 | regulation of mitotic metaphase/anaphase transition | 1 | 1 | 5 | 197 | 0.2 | 0.005 | 39.37 | 0.025 | 99632\_at | MAD2 (mitotic arrest deficient, homolog)-like 1 (yeast) |
| 9 | regulation of mitosis | 2 | 3 | 66 | 911 | 0.03 | 0.003 | 9.21 | 0.015 | 99632\_at,99129\_at | MAD2 (mitotic arrest deficient, homolog)-like 1 (yeast),CD40 ligand-activated specific transcript 3 |
| 10 | regulation of mitotic metaphase/anaphase transition | 1 | 1 | 5 | 197 | 0.2 | 0.005 | 39.37 | 0.025 | 99632\_at | MAD2 (mitotic arrest deficient, homolog)-like 1 (yeast) |
| 10 | mitotic checkpoint | 2 | 3 | 5 | 197 | 0.4 | 0.015 | 26.264 | 0.002 | 99129\_at,99632\_at | CD40 ligand-activated specific transcript 3,MAD2 (mitotic arrest deficient, homolog)-like 1 (yeast) |
| 11 | mitotic spindle checkpoint | 2 | 2 | 4 | 34 | 0.5 | 0.059 | 8.501 | 0.011 | 99129\_at,99632\_at | CD40 ligand-activated specific transcript 3,MAD2 (mitotic arrest deficient, homolog)-like 1 (yeast) |
| 7 | cell cycle checkpoint | 3 | 10 | 298 | 6246 | 0.01 | 0.002 | 6.294 | 0.01 | 99129\_at,99632\_at,103805\_at | CD40 ligand-activated specific transcript 3,MAD2 (mitotic arrest deficient, homolog)-like 1 (yeast),nibrin |
| 7 | acidic amino acid transport | 1 | 1 | 298 | 6246 | 0.003 | 0 | 21 | 0.048 | 97424\_at | ADP-ribosylation-like factor 6 interacting protein 5 |
| 5 | hydrogen transport | 13 | 50 | 600 | 11544 | 0.022 | 0.004 | 5.005 | 0 | 93533\_at,99128\_at,160125\_at,160126\_at,160203\_at,161487\_f\_at,92798\_at,92799\_g\_at,92800\_i\_at,93596\_i\_at,95656\_i\_at,96611\_at,93014\_at | RIKEN cDNA 1500011L16 gene,ATP synthase, H+ transporting, mitochondrial F1 complex, O subunit,DNA segment, Chr 11, ERATO Doi 497, expressed,mitogen-activated protein kinase kinase 1 interacting protein 1,RIKEN cDNA 5330419I01 gene,ATP synthase, H+ transporting, mitochondrial F0 complex, subunit c (subunit 9), isoform 1,ATP synthase, H+ transporting, mitochondrial F1 complex, gamma polypeptide 1,ATP synthase, H+ transporting, mitochondrial F1 complex, gamma polypeptide 1,ATP synthase, H+ transporting, mitochondrial F1 complex, gamma polypeptide 1,RIKEN cDNA 2410043G19 gene,ATPase, H+ transporting, V1 subunit F,NADH-ubiquinone oxidoreductase subunit B14.7,ATP synthase, H+ transporting, mitochondrial F0 complex, subunit g |
| 6 | proton transport | 12 | 44 | 466 | 9498 | 0.026 | 0.005 | 5.562 | 0 | 160125\_at,160126\_at,160203\_at,161487\_f\_at,92798\_at,92799\_g\_at,92800\_i\_at,93596\_i\_at,95656\_i\_at,96611\_at,99128\_at,93014\_at | DNA segment, Chr 11, ERATO Doi 497, expressed,mitogen-activated protein kinase kinase 1 interacting protein 1,RIKEN cDNA 5330419I01 gene,ATP synthase, H+ transporting, mitochondrial F0 complex, subunit c (subunit 9), isoform 1,ATP synthase, H+ transporting, mitochondrial F1 complex, gamma polypeptide 1,ATP synthase, H+ transporting, mitochondrial F1 complex, gamma polypeptide 1,ATP synthase, H+ transporting, mitochondrial F1 complex, gamma polypeptide 1,RIKEN cDNA 2410043G19 gene,ATPase, H+ transporting, V1 subunit F,NADH-ubiquinone oxidoreductase subunit B14.7,ATP synthase, H+ transporting, mitochondrial F1 complex, O subunit,ATP synthase, H+ transporting, mitochondrial F0 complex, subunit g |
| 7 | energy coupled proton transport, down the electrochemical gradient | 2 | 5 | 298 | 6246 | 0.007 | 0.001 | 8.387 | 0.021 | 93014\_at,95656\_i\_at | ATP synthase, H+ transporting, mitochondrial F0 complex, subunit g,ATPase, H+ transporting, V1 subunit F |
| 8 | ATP synthesis coupled proton transport | 2 | 5 | 130 | 2164 | 0.015 | 0.002 | 6.658 | 0.032 | 93014\_at,95656\_i\_at | ATP synthase, H+ transporting, mitochondrial F0 complex, subunit g,ATPase, H+ transporting, V1 subunit F |
| 5 | intracellular transport | 31 | 351 | 600 | 11544 | 0.052 | 0.03 | 1.699 | 0.003 | 101254\_at,102821\_s\_at,104322\_at,160076\_at,160256\_at,160543\_at,160876\_at,93993\_at,94014\_at,94210\_at,94277\_at,94323\_at,94506\_at,95091\_at,95441\_at,96668\_at,96849\_at,97477\_at,97478\_at,98930\_at,99147\_at,92636\_f\_at,96670\_at,96734\_at,96947\_at,98959\_at,99156\_at,101061\_at,93970\_at,100618\_f\_at,93084\_at | RAN, member RAS oncogene family,RAS-like, family 2, locus 9,cytoskeleton associated protein 2,metaxin 2,RIKEN cDNA 1110020J08 gene,sorting nexin 3,B-cell receptor-associated protein 29,RIKEN cDNA 1300009F09 gene,RIKEN cDNA 2510048O06 gene,translocase of inner mitochondrial membrane 9 homolog (yeast),metaxin 1,RIKEN cDNA D630012G11 gene,cleavage and polyadenylation specific factor 5,SEC13 related gene (S. cerevisiae),translocase of inner mitochondrial membrane 23 homolog (yeast),translocator of inner mitochondrial membrane b,translocase of inner mitochondrial membrane 8 homolog a (yeast),translocase of inner mitochondrial membrane 8 homolog b (yeast),golgi phosphoprotein 2,coatomer protein complex, subunit epsilon,RIKEN cDNA 2700001K05 gene,SEC61, gamma subunit (S. cerevisiae),RIKEN cDNA 0610025I19 gene,synaptojanin 2 binding protein,RIKEN cDNA 0610009I16 gene,RIKEN cDNA D930048L02 gene,RIKEN cDNA 2700099C19 gene,signal sequence receptor, beta,importin 7,solute carrier family 25 (mitochondrial carrier; adenine nucleotide translocator), member 5,solute carrier family 25 (mitochondrial carrier; adenine nucleotide translocator), member 4 |
| 6 | intracellular protein transport | 29 | 284 | 466 | 9498 | 0.062 | 0.03 | 2.081 | 0 | 101254\_at,102821\_s\_at,104322\_at,160076\_at,160256\_at,160543\_at,160876\_at,93993\_at,94014\_at,94210\_at,94277\_at,94323\_at,94506\_at,95091\_at,95441\_at,96668\_at,96849\_at,97477\_at,97478\_at,98930\_at,99147\_at,92636\_f\_at,96670\_at,96734\_at,96947\_at,98959\_at,99156\_at,101061\_at,93970\_at | RAN, member RAS oncogene family,RAS-like, family 2, locus 9,cytoskeleton associated protein 2,metaxin 2,RIKEN cDNA 1110020J08 gene,sorting nexin 3,B-cell receptor-associated protein 29,RIKEN cDNA 1300009F09 gene,RIKEN cDNA 2510048O06 gene,translocase of inner mitochondrial membrane 9 homolog (yeast),metaxin 1,RIKEN cDNA D630012G11 gene,cleavage and polyadenylation specific factor 5,SEC13 related gene (S. cerevisiae),translocase of inner mitochondrial membrane 23 homolog (yeast),translocator of inner mitochondrial membrane b,translocase of inner mitochondrial membrane 8 homolog a (yeast),translocase of inner mitochondrial membrane 8 homolog b (yeast),golgi phosphoprotein 2,coatomer protein complex, subunit epsilon,RIKEN cDNA 2700001K05 gene,SEC61, gamma subunit (S. cerevisiae),RIKEN cDNA 0610025I19 gene,synaptojanin 2 binding protein,RIKEN cDNA 0610009I16 gene,RIKEN cDNA D930048L02 gene,RIKEN cDNA 2700099C19 gene,signal sequence receptor, beta,importin 7 |
| 7 | protein targeting | 17 | 101 | 298 | 6246 | 0.057 | 0.016 | 3.528 | 0 | 92636\_f\_at,94014\_at,94210\_at,95441\_at,96668\_at,96670\_at,96734\_at,96849\_at,96947\_at,97477\_at,97478\_at,98959\_at,99156\_at,101061\_at,101254\_at,93970\_at,94323\_at | SEC61, gamma subunit (S. cerevisiae),RIKEN cDNA 2510048O06 gene,translocase of inner mitochondrial membrane 9 homolog (yeast),translocase of inner mitochondrial membrane 23 homolog (yeast),translocator of inner mitochondrial membrane b,RIKEN cDNA 0610025I19 gene,synaptojanin 2 binding protein,translocase of inner mitochondrial membrane 8 homolog a (yeast),RIKEN cDNA 0610009I16 gene,translocase of inner mitochondrial membrane 8 homolog b (yeast),golgi phosphoprotein 2,RIKEN cDNA D930048L02 gene,RIKEN cDNA 2700099C19 gene,signal sequence receptor, beta,RAN, member RAS oncogene family,importin 7,RIKEN cDNA D630012G11 gene |
| 8 | protein-mitochondrial targeting | 5 | 6 | 130 | 2164 | 0.038 | 0.003 | 13.884 | 0 | 94014\_at,94210\_at,96849\_at,97477\_at,97478\_at | RIKEN cDNA 2510048O06 gene,translocase of inner mitochondrial membrane 9 homolog (yeast),translocase of inner mitochondrial membrane 8 homolog a (yeast),translocase of inner mitochondrial membrane 8 homolog b (yeast),golgi phosphoprotein 2 |
| 9 | mitochondrial translocation | 5 | 6 | 66 | 911 | 0.076 | 0.007 | 11.496 | 0 | 94014\_at,94210\_at,96849\_at,97477\_at,97478\_at | RIKEN cDNA 2510048O06 gene,translocase of inner mitochondrial membrane 9 homolog (yeast),translocase of inner mitochondrial membrane 8 homolog a (yeast),translocase of inner mitochondrial membrane 8 homolog b (yeast),golgi phosphoprotein 2 |
| 5 | protein transport | 29 | 297 | 600 | 11544 | 0.048 | 0.026 | 1.878 | 0.001 | 101254\_at,102821\_s\_at,104322\_at,160076\_at,160256\_at,160543\_at,160876\_at,93993\_at,94014\_at,94210\_at,94277\_at,94323\_at,94506\_at,95091\_at,95441\_at,96668\_at,96849\_at,97477\_at,97478\_at,98930\_at,99147\_at,92636\_f\_at,96670\_at,96734\_at,96947\_at,98959\_at,99156\_at,101061\_at,93970\_at | RAN, member RAS oncogene family,RAS-like, family 2, locus 9,cytoskeleton associated protein 2,metaxin 2,RIKEN cDNA 1110020J08 gene,sorting nexin 3,B-cell receptor-associated protein 29,RIKEN cDNA 1300009F09 gene,RIKEN cDNA 2510048O06 gene,translocase of inner mitochondrial membrane 9 homolog (yeast),metaxin 1,RIKEN cDNA D630012G11 gene,cleavage and polyadenylation specific factor 5,SEC13 related gene (S. cerevisiae),translocase of inner mitochondrial membrane 23 homolog (yeast),translocator of inner mitochondrial membrane b,translocase of inner mitochondrial membrane 8 homolog a (yeast),translocase of inner mitochondrial membrane 8 homolog b (yeast),golgi phosphoprotein 2,coatomer protein complex, subunit epsilon,RIKEN cDNA 2700001K05 gene,SEC61, gamma subunit (S. cerevisiae),RIKEN cDNA 0610025I19 gene,synaptojanin 2 binding protein,RIKEN cDNA 0610009I16 gene,RIKEN cDNA D930048L02 gene,RIKEN cDNA 2700099C19 gene,signal sequence receptor, beta,importin 7 |
| 6 | intracellular protein transport | 29 | 284 | 466 | 9498 | 0.062 | 0.03 | 2.081 | 0 | 101254\_at,102821\_s\_at,104322\_at,160076\_at,160256\_at,160543\_at,160876\_at,93993\_at,94014\_at,94210\_at,94277\_at,94323\_at,94506\_at,95091\_at,95441\_at,96668\_at,96849\_at,97477\_at,97478\_at,98930\_at,99147\_at,92636\_f\_at,96670\_at,96734\_at,96947\_at,98959\_at,99156\_at,101061\_at,93970\_at | RAN, member RAS oncogene family,RAS-like, family 2, locus 9,cytoskeleton associated protein 2,metaxin 2,RIKEN cDNA 1110020J08 gene,sorting nexin 3,B-cell receptor-associated protein 29,RIKEN cDNA 1300009F09 gene,RIKEN cDNA 2510048O06 gene,translocase of inner mitochondrial membrane 9 homolog (yeast),metaxin 1,RIKEN cDNA D630012G11 gene,cleavage and polyadenylation specific factor 5,SEC13 related gene (S. cerevisiae),translocase of inner mitochondrial membrane 23 homolog (yeast),translocator of inner mitochondrial membrane b,translocase of inner mitochondrial membrane 8 homolog a (yeast),translocase of inner mitochondrial membrane 8 homolog b (yeast),golgi phosphoprotein 2,coatomer protein complex, subunit epsilon,RIKEN cDNA 2700001K05 gene,SEC61, gamma subunit (S. cerevisiae),RIKEN cDNA 0610025I19 gene,synaptojanin 2 binding protein,RIKEN cDNA 0610009I16 gene,RIKEN cDNA D930048L02 gene,RIKEN cDNA 2700099C19 gene,signal sequence receptor, beta,importin 7 |
| 7 | protein targeting | 17 | 101 | 298 | 6246 | 0.057 | 0.016 | 3.528 | 0 | 92636\_f\_at,94014\_at,94210\_at,95441\_at,96668\_at,96670\_at,96734\_at,96849\_at,96947\_at,97477\_at,97478\_at,98959\_at,99156\_at,101061\_at,101254\_at,93970\_at,94323\_at | SEC61, gamma subunit (S. cerevisiae),RIKEN cDNA 2510048O06 gene,translocase of inner mitochondrial membrane 9 homolog (yeast),translocase of inner mitochondrial membrane 23 homolog (yeast),translocator of inner mitochondrial membrane b,RIKEN cDNA 0610025I19 gene,synaptojanin 2 binding protein,translocase of inner mitochondrial membrane 8 homolog a (yeast),RIKEN cDNA 0610009I16 gene,translocase of inner mitochondrial membrane 8 homolog b (yeast),golgi phosphoprotein 2,RIKEN cDNA D930048L02 gene,RIKEN cDNA 2700099C19 gene,signal sequence receptor, beta,RAN, member RAS oncogene family,importin 7,RIKEN cDNA D630012G11 gene |
| 8 | protein-mitochondrial targeting | 5 | 6 | 130 | 2164 | 0.038 | 0.003 | 13.884 | 0 | 94014\_at,94210\_at,96849\_at,97477\_at,97478\_at | RIKEN cDNA 2510048O06 gene,translocase of inner mitochondrial membrane 9 homolog (yeast),translocase of inner mitochondrial membrane 8 homolog a (yeast),translocase of inner mitochondrial membrane 8 homolog b (yeast),golgi phosphoprotein 2 |
| 9 | mitochondrial translocation | 5 | 6 | 66 | 911 | 0.076 | 0.007 | 11.496 | 0 | 94014\_at,94210\_at,96849\_at,97477\_at,97478\_at | RIKEN cDNA 2510048O06 gene,translocase of inner mitochondrial membrane 9 homolog (yeast),translocase of inner mitochondrial membrane 8 homolog a (yeast),translocase of inner mitochondrial membrane 8 homolog b (yeast),golgi phosphoprotein 2 |
| 3 | metabolism | 260 | 3908 | 509 | 10726 | 0.511 | 0.364 | 1.402 | 0 | 103881\_at,103939\_at,104297\_at,104301\_at,160293\_at,160314\_at,160711\_at,92589\_at,93029\_at,93754\_at,94276\_at,95408\_at,95426\_at,95634\_at,95635\_g\_at,95636\_at,95693\_at,96268\_at,96678\_at,96948\_at,97449\_at,97820\_at,98527\_at,98966\_at,99566\_at,99613\_at,93258\_at,94275\_at,AFFX-GapdhMur/M32599\_3\_at,AFFX-GapdhMur/M32599\_5\_at,97279\_at,96627\_at,97419\_at,104567\_at,92540\_f\_at,160844\_at,97318\_at,93139\_at,99056\_at,96336\_at,97179\_at,103683\_at,161897\_f\_at,104147\_at,103334\_at,160125\_at,160126\_at,92798\_at,92799\_g\_at,92800\_i\_at,93596\_i\_at,95656\_i\_at,96611\_at,99128\_at,93014\_at,98910\_at,93582\_at,102194\_at,95760\_at,96909\_at,102970\_at,92388\_at,101097\_at,101680\_at,102019\_at,160431\_at,92578\_at,92646\_at,93062\_at,93579\_at,94252\_at,94494\_at,94870\_f\_at,94912\_at,95067\_at,95498\_at,96291\_f\_at,96292\_r\_at,96293\_at,97342\_at,97751\_f\_at,97824\_at,97884\_at,98120\_at,98524\_f\_at,98904\_at,99594\_at,95677\_at,92565\_at,93236\_s\_at,93237\_s\_at,95497\_at,92625\_at,92824\_at,97538\_at,99148\_at,93991\_at,95053\_s\_at,93542\_at,100576\_at,100539\_at,93734\_i\_at,93735\_f\_at,94025\_at,95448\_at,100543\_s\_at,101562\_at,104541\_at,92874\_f\_at,95561\_at,96093\_at,96733\_at,99655\_at,93519\_s\_at,100512\_at,100733\_at,101486\_at,101558\_s\_at,101992\_at,102791\_at,92547\_at,93085\_at,93988\_at,94263\_f\_at,94841\_at,96892\_at,96952\_at,97459\_at,98557\_f\_at,98975\_at,93203\_f\_at,94372\_at,162417\_at,103581\_at,100057\_at,100059\_at,100156\_at,100550\_f\_at,100568\_at,103619\_at,103671\_at,93041\_at,93742\_at,93820\_at,95045\_at,95696\_at,96112\_at,96861\_at,96947\_at,97013\_f\_at,98613\_at,99618\_at,100079\_at,94062\_at,96267\_at,96899\_at,96902\_at,95064\_at,95485\_at,99106\_at,160135\_at,94034\_at,96289\_at,99544\_at,101254\_at,102631\_at,96081\_at,100612\_at,101065\_at,101067\_at,102001\_at,103418\_at,104738\_at,93112\_at,101105\_at,93095\_at,93251\_at,96699\_at,98039\_at,94897\_at,96775\_at,101954\_at,93833\_s\_at,98587\_at,95660\_at,100459\_at,102853\_at,93559\_at,160107\_at,160723\_at,93117\_at,96696\_at,99151\_at,160503\_at,160531\_at,100577\_at,102409\_at,93008\_at,93999\_at,95049\_at,96029\_at,97200\_f\_at,99182\_at,160426\_at,93551\_at,98081\_at,95479\_at,95480\_at,161147\_f\_at,98075\_at,93548\_at,102412\_at,103319\_at,103654\_at,104476\_at,160659\_at,94506\_at,95132\_r\_at,95460\_at,97164\_at,98516\_at,160324\_at,100331\_g\_at,97819\_at,99583\_at,97758\_at,104423\_at,96052\_at,100128\_at,104080\_at,160538\_at,98595\_at,98934\_at,99522\_at,92636\_f\_at,94014\_at,94210\_at,95441\_at,96668\_at,96670\_at,96734\_at,96849\_at,97477\_at,97478\_at,98959\_at,99156\_at,101061\_at,93970\_at,94323\_at,100089\_at,101207\_at,160416\_at,160456\_at,92829\_at,98153\_at,99546\_at,93101\_s\_at,97460\_at,101440\_at,95015\_at | inorganic pyrophosphatase 2,RIKEN cDNA 2610509I15 gene,RIKEN cDNA 2510001A17 gene,RIKEN cDNA 2610001E06 gene,RIKEN cDNA 2700038L12 gene,RIKEN cDNA 2010317E03 gene,2,4-dienoyl CoA reductase 1, mitochondrial,expressed sequence AI480570,isocitrate dehydrogenase 3 (NAD+), gamma,enoyl coenzyme A hydratase 1, peroxisomal,hydroxysteroid (17-beta) dehydrogenase 12,RIKEN cDNA 2310003F16 gene,enoyl Coenzyme A hydratase, short chain, 1, mitochondrial,RIKEN cDNA 0610010K14 gene,RIKEN cDNA 0610010K14 gene,RIKEN cDNA 0610010K14 gene,NA,succinate-CoA ligase, GDP-forming, alpha subunit,dehydrogenase/reductase (SDR family) member 4,quininoid dihydropteridine reductase,NA,galactokinase 1,dodecenoyl-Coenzyme A delta isomerase (3,2 trans-enoyl-Coenyme A isomerase),dihydrolipoamide branched chain transacylase E2,triosephosphate isomerase,methylmalonyl-Coenzyme A mutase,hydroxymethylbilane synthase,uroporphyrinogen decarboxylase,glyceraldehyde-3-phosphate dehydrogenase,glyceraldehyde-3-phosphate dehydrogenase,RIKEN cDNA 6430402H10 gene,phenylalkylamine Ca2+ antagonist (emopamil) binding protein,RIKEN cDNA 2310010I22 gene,RIKEN cDNA 3110052F15 gene,spermidine synthase,6-pyruvoyl-tetrahydropterin synthase,histidyl tRNA synthetase 2,NA,6-pyruvoyl-tetrahydropterin synthase/dimerization cofactor of hepatocyte nuclear factor 1 alpha (TCF1),glycine amidinotransferase (L-arginine:glycine amidinotransferase),expressed sequence C76683,dihydroorotate dehydrogenase,phosphoribosyl pyrophosphate synthetase 1,N-acetylneuraminic acid synthase (sialic acid synthase),calcitonin gene-related peptide-receptor component protein,DNA segment, Chr 11, ERATO Doi 497, expressed,mitogen-activated protein kinase kinase 1 interacting protein 1,ATP synthase, H+ transporting, mitochondrial F1 complex, gamma polypeptide 1,ATP synthase, H+ transporting, mitochondrial F1 complex, gamma polypeptide 1,ATP synthase, H+ transporting, mitochondrial F1 complex, gamma polypeptide 1,RIKEN cDNA 2410043G19 gene,ATPase, H+ transporting, V1 subunit F,NADH-ubiquinone oxidoreductase subunit B14.7,ATP synthase, H+ transporting, mitochondrial F1 complex, O subunit,ATP synthase, H+ transporting, mitochondrial F0 complex, subunit g,RIKEN cDNA 1110014K08 gene,demethyl-Q 7,RIKEN cDNA 2810432D09 gene,RIKEN cDNA 1110011K10 gene,NADH dehydrogenase (ubiquinone) 1, alpha/beta subcomplex, 1,proteasome (prosome, macropain) 26S subunit, ATPase 3, interacting protein,NA,DNA segment, Chr 9, Wayne State University 149,ribosomal protein L27a,mitochondrial ribosomal protein L13,mitochondrial ribosomal protein L12,small inducible cytokine subfamily E, member 1,mitochondrial ribosomal protein L23,mitochondrial ribosomal protein L39,RIKEN cDNA 5830427H10 gene,eukaryotic translation initiation factor 2, subunit 3, structural gene X-linked,phenylalanine-tRNA synthetase-like,RIKEN cDNA 2310075M17 gene,mitochondrial ribosomal protein S21,mitochondrial ribosomal protein L2,mitochondrial ribosomal protein S15,NA,NA,RIKEN cDNA 2410015N17 gene,mitochondrial ribosomal protein S14,NA,DNA segment, Chr 11, ERATO Doi 175, expressed,mitochondrial ribosomal protein S11,mitochondrial ribosomal protein L27,RIKEN cDNA 2210039B01 gene,mitochondrial ribosomal protein L35,mitochondrial ribosomal protein L51,U5 snRNP-specific protein (Prp8-binding),RIKEN cDNA 1110005A23 gene,thymidylate synthase,thymidylate synthase,RIKEN cDNA 1110005A05 gene,expressed in non-metastatic cells 2, protein,expressed in non-metastatic cells 6, protein,beta-glucuronidase,fumarate hydratase 1,malate dehydrogenase, mitochondrial,succinate dehydrogenase complex, subunit B, iron sulfur (Ip),phosphotriesterase related,platelet-activating factor acetylhydrolase, isoform 1b, alpha1 subunit,brain acyl-CoA hydrolase,proteasome (prosome, macropain) 26S subunit, ATPase 3,proteasome (prosome, macropain) 26S subunit, ATPase 3,proteasome (prosome, macropain) subunit, beta type 3,proteasome (prosome, macropain) 26S subunit, ATPase 2,bromodomain containing 7,heat shock protein 4,proteinase 3,COP9 (constitutive photomorphogenic) homolog, subunit 7a (Arabidopsis thaliana),RIKEN cDNA 1700013H19 gene,open reading frame 11,RAP1, GTP-GDP dissociation stimulator 1,signal peptidase complex,neural precursor cell expressed, developmentally down-regulated gene 8,ubiquitin carboxyl-terminal esterase L5,proteasome (prosome, macropain) subunit, alpha type 2,proteasome (prosome, macropain) subunit, beta type 10,proteasome (prosome, macropain) subunit, beta type 5,proteasome (prosome, macropain) subunit, beta type 6,proteosome (prosome, macropain) subunit, beta type 8 (large multifunctional protease 7),huntingtin interacting protein 2,proteosome (prosome, macropain) subunit, beta type 9 (large multifunctional protease 2),proteasome (prosome, macropain) subunit, alpha type 7,proteasome (prosome, macropain) subunit, beta type 7,proteasome (prosome, macropain) subunit, alpha type 5,proteasome (prosome, macropain) subunit, alpha type 1,proteasome (prosome, macropain) subunit, alpha type 6,proteasome (prosome, macropain) subunit, alpha type 4,proteasome (prosome, macropain) subunit, beta type 4,RIKEN cDNA 2410008G02 gene,RIKEN cDNA 3230402J05 gene,nudix (nucleoside diphosphate linked moiety X)-type motif 1,RIKEN cDNA 1500001M20 gene,cytosolic acyl-CoA thioesterase 1,RIKEN cDNA 2510027N19 gene,cytochrome b-245, alpha polypeptide,minichromosome maintenance deficient 5, cell division cycle 46 (S. cerevisiae),cytochrome c oxidase, subunit VIc,ATP-binding cassette, sub-family E (OABP), member 1,cytochrome b5 outer mitochondrial membrane precursor,HIV-1 tat interactive protein 2, homolog (human),minichromosome maintenance deficient 4 homolog (S. cerevisiae),RIKEN cDNA 5730449L18 gene,cytochrome c oxidase, subunit VIIa 2,RIKEN cDNA 0610012D09 gene,thioredoxin-like 2,electron transferring flavoprotein, alpha polypeptide,mitochondrial ribosomal protein L50,RIKEN cDNA 0610009I16 gene,cytochrome b-245, alpha polypeptide,RIKEN cDNA 2700085E05 gene,ubiquinol-cytochrome c reductase subunit,NADH dehydrogenase (ubiquinone) 1 beta subcomplex, 9,NADH dehydrogenase (ubiquinone) flavoprotein 2,NADH dehydrogenase (ubiquinone) flavoprotein 1,NADH dehydrogenase (ubiquinone) Fe-S protein 3,RIKEN cDNA 2900091E11 gene,acetyl-Coenzyme A acyltransferase 2 (mitochondrial 3-oxoacyl-Coenzyme A thiolase),L-3-hydroxyacyl-Coenzyme A dehydrogenase, short chain,COP9 (constitutive photomorphogenic) homolog, subunit 6 (Arabidopsis thaliana),DNA segment, Chr 16, ERATO Doi 502, expressed,small fragment nuclease,stomatin (Epb7.2)-like 2,deoxyguanosine kinase,RAN, member RAS oncogene family,Bloom syndrome homolog (human),thymidine kinase 1,ribonucleotide reductase M1,proliferating cell nuclear antigen,RIKEN cDNA 2010005E08 gene,ribonucleotide reductase M2,expressed sequence AU040575,zuotin related factor 2,minichromosome maintenance deficient 2 mitotin (S. cerevisiae),barrier to autointegration factor 1,high mobility group box 1,4-nitrophenylphosphatase domain and non-neuronal SNAP25-like protein homolog 1 (C. elegans),high mobility group nucleosomal binding domain 1,RIKEN cDNA 2410015M20 gene,glutathione peroxidase 4,chromobox homolog 1 (Drosophila HP1 beta),H2A histone family, member Z,histone 1, H2bl,nucleosome assembly protein 1-like 1,RIKEN cDNA 0610025L15 gene,RAD50 homolog (S. cerevisiae),chondroitin sulfate proteoglycan 6,apurinic/apyrimidinic endonuclease 1,hypoxanthine guanine phosphoribosyl transferase,RIKEN cDNA 1500001M20 gene,heterogeneous nuclear ribonucleoprotein A2/B1,NA,RIKEN cDNA 2610002K22 gene,fibrillarin,B-cell receptor-associated protein 37,small nuclear ribonucleoprotein D1,RIKEN cDNA 2010003I05 gene,U6 snRNA-associated SM-like protein 4,small nuclear ribonucleoprotein polypeptide G,small nuclear ribonucleoprotein D2,splicing factor 3a, subunit 3, 60kDa,small nuclear ribonucleoprotein E,RIKEN cDNA 2610511E03 gene,RNA polymerase 1-1,RIKEN cDNA 2510029B14 gene,RNA polymerase 1-3,nuclear DNA binding protein,NA,RIKEN cDNA 2310061C15 gene,RIKEN cDNA G431001I09 gene,RIKEN cDNA 1190006C12 gene,expressed sequence AW541137,proteasome (prosome, macropain) 26S subunit, non-ATPase, 10,nucleosome binding protein 1,retinoblastoma-like 1 (p107),RIKEN cDNA 2310057G13 gene,cleavage and polyadenylation specific factor 5,NADH dehydrogenase (ubiquinone) 1 beta subcomplex, 2,COP9 (constitutive photomorphogenic) homolog, subunit 5 (Arabidopsis thaliana),RIKEN cDNA 2610207P08 gene,NA,replication protein A3,thioredoxin peroxidase, pseudogene 1,glutathione S-transferase omega 1,glutathione S-transferase, pi 2,peroxiredoxin 1,RIKEN cDNA 2810047L02 gene,acid phosphatase 1, soluble,cell division cycle 2 homolog A (S. pombe),PDGFA associated protein 1,cyclin-dependent kinase 4,interleukin-1 receptor-associated kinase 1,RIKEN cDNA 0610007P06 gene,germ cell-specific gene 2,SEC61, gamma subunit (S. cerevisiae),RIKEN cDNA 2510048O06 gene,translocase of inner mitochondrial membrane 9 homolog (yeast),translocase of inner mitochondrial membrane 23 homolog (yeast),translocator of inner mitochondrial membrane b,RIKEN cDNA 0610025I19 gene,synaptojanin 2 binding protein,translocase of inner mitochondrial membrane 8 homolog a (yeast),translocase of inner mitochondrial membrane 8 homolog b (yeast),golgi phosphoprotein 2,RIKEN cDNA D930048L02 gene,RIKEN cDNA 2700099C19 gene,signal sequence receptor, beta,importin 7,RIKEN cDNA D630012G11 gene,peptidylprolyl isomerase C,peptidylprolyl isomerase A,FK506 binding protein 3,RIKEN cDNA 1100001J08 gene,heat shock protein 1 (chaperonin 10),chaperonin subunit 3 (gamma),FK506 binding protein 2,neural precursor cell expressed, developmentally down-regulted gene 4,ubiquitin-conjugating enzyme E2R 2,RIKEN cDNA 2900006F19 gene,aldo-keto reductase family 1, member C13 |
| 7 | cholesterol biosynthesis | 4 | 17 | 298 | 6246 | 0.013 | 0.003 | 4.934 | 0.007 | 95634\_at,95635\_g\_at,95636\_at,96627\_at | RIKEN cDNA 0610010K14 gene,RIKEN cDNA 0610010K14 gene,RIKEN cDNA 0610010K14 gene,phenylalkylamine Ca2+ antagonist (emopamil) binding protein |
| 6 | sterol biosynthesis | 4 | 20 | 466 | 9498 | 0.009 | 0.002 | 4.066 | 0.015 | 96627\_at,95634\_at,95635\_g\_at,95636\_at | phenylalkylamine Ca2+ antagonist (emopamil) binding protein,RIKEN cDNA 0610010K14 gene,RIKEN cDNA 0610010K14 gene,RIKEN cDNA 0610010K14 gene |
| 7 | cholesterol biosynthesis | 4 | 17 | 298 | 6246 | 0.013 | 0.003 | 4.934 | 0.007 | 95634\_at,95635\_g\_at,95636\_at,96627\_at | RIKEN cDNA 0610010K14 gene,RIKEN cDNA 0610010K14 gene,RIKEN cDNA 0610010K14 gene,phenylalkylamine Ca2+ antagonist (emopamil) binding protein |
| 6 | amino acid catabolism | 4 | 24 | 466 | 9498 | 0.009 | 0.003 | 3.391 | 0.028 | 160844\_at,95408\_at,97318\_at,104567\_at | 6-pyruvoyl-tetrahydropterin synthase,RIKEN cDNA 2310003F16 gene,histidyl tRNA synthetase 2,RIKEN cDNA 3110052F15 gene |
| 8 | phenylalanine catabolism | 2 | 6 | 130 | 2164 | 0.015 | 0.003 | 5.552 | 0.046 | 160844\_at,95408\_at | 6-pyruvoyl-tetrahydropterin synthase,RIKEN cDNA 2310003F16 gene |
| 7 | D-amino acid catabolism | 1 | 1 | 298 | 6246 | 0.003 | 0 | 21 | 0.048 | 97318\_at | histidyl tRNA synthetase 2 |
| 6 | amino acid catabolism | 4 | 24 | 466 | 9498 | 0.009 | 0.003 | 3.391 | 0.028 | 160844\_at,95408\_at,97318\_at,104567\_at | 6-pyruvoyl-tetrahydropterin synthase,RIKEN cDNA 2310003F16 gene,histidyl tRNA synthetase 2,RIKEN cDNA 3110052F15 gene |
| 8 | phenylalanine catabolism | 2 | 6 | 130 | 2164 | 0.015 | 0.003 | 5.552 | 0.046 | 160844\_at,95408\_at | 6-pyruvoyl-tetrahydropterin synthase,RIKEN cDNA 2310003F16 gene |
| 7 | D-amino acid catabolism | 1 | 1 | 298 | 6246 | 0.003 | 0 | 21 | 0.048 | 97318\_at | histidyl tRNA synthetase 2 |
| 8 | phenylalanine catabolism | 2 | 6 | 130 | 2164 | 0.015 | 0.003 | 5.552 | 0.046 | 160844\_at,95408\_at | 6-pyruvoyl-tetrahydropterin synthase,RIKEN cDNA 2310003F16 gene |
| 7 | L-phenylalanine metabolism | 3 | 7 | 298 | 6246 | 0.01 | 0.001 | 8.991 | 0.003 | 99056\_at,160844\_at,95408\_at | 6-pyruvoyl-tetrahydropterin synthase/dimerization cofactor of hepatocyte nuclear factor 1 alpha (TCF1),6-pyruvoyl-tetrahydropterin synthase,RIKEN cDNA 2310003F16 gene |
| 8 | phenylalanine catabolism | 2 | 6 | 130 | 2164 | 0.015 | 0.003 | 5.552 | 0.046 | 160844\_at,95408\_at | 6-pyruvoyl-tetrahydropterin synthase,RIKEN cDNA 2310003F16 gene |
| 7 | valine metabolism | 1 | 1 | 298 | 6246 | 0.003 | 0 | 21 | 0.048 | 97279\_at | RIKEN cDNA 6430402H10 gene |
| 4 | aromatic compound metabolism | 8 | 65 | 695 | 13100 | 0.012 | 0.005 | 2.321 | 0.021 | 95408\_at,160844\_at,99056\_at,97179\_at,96948\_at,103683\_at,161897\_f\_at,160293\_at | RIKEN cDNA 2310003F16 gene,6-pyruvoyl-tetrahydropterin synthase,6-pyruvoyl-tetrahydropterin synthase/dimerization cofactor of hepatocyte nuclear factor 1 alpha (TCF1),expressed sequence C76683,quininoid dihydropteridine reductase,dihydroorotate dehydrogenase,phosphoribosyl pyrophosphate synthetase 1,RIKEN cDNA 2700038L12 gene |
| 5 | aromatic compound biosynthesis | 4 | 7 | 600 | 11544 | 0.007 | 0.001 | 10.934 | 0 | 97179\_at,160844\_at,96948\_at,99056\_at | expressed sequence C76683,6-pyruvoyl-tetrahydropterin synthase,quininoid dihydropteridine reductase,6-pyruvoyl-tetrahydropterin synthase/dimerization cofactor of hepatocyte nuclear factor 1 alpha (TCF1) |
| 6 | pteridine and derivative biosynthesis | 4 | 7 | 466 | 9498 | 0.009 | 0.001 | 11.595 | 0 | 97179\_at,160844\_at,96948\_at,99056\_at | expressed sequence C76683,6-pyruvoyl-tetrahydropterin synthase,quininoid dihydropteridine reductase,6-pyruvoyl-tetrahydropterin synthase/dimerization cofactor of hepatocyte nuclear factor 1 alpha (TCF1) |
| 7 | tetrahydrobiopterin biosynthesis | 3 | 4 | 298 | 6246 | 0.01 | 0.001 | 15.734 | 0 | 160844\_at,96948\_at,99056\_at | 6-pyruvoyl-tetrahydropterin synthase,quininoid dihydropteridine reductase,6-pyruvoyl-tetrahydropterin synthase/dimerization cofactor of hepatocyte nuclear factor 1 alpha (TCF1) |
| 5 | nucleobase metabolism | 3 | 12 | 600 | 11544 | 0.005 | 0.001 | 4.808 | 0.022 | 103683\_at,161897\_f\_at,160293\_at | dihydroorotate dehydrogenase,phosphoribosyl pyrophosphate synthetase 1,RIKEN cDNA 2700038L12 gene |
| 4 | biosynthesis | 70 | 652 | 695 | 13100 | 0.101 | 0.05 | 2.024 | 0 | 95634\_at,95635\_g\_at,95636\_at,99566\_at,97419\_at,104567\_at,92589\_at,92540\_f\_at,97179\_at,160844\_at,96948\_at,99056\_at,103683\_at,104147\_at,103334\_at,160125\_at,160126\_at,92798\_at,92799\_g\_at,92800\_i\_at,93596\_i\_at,95656\_i\_at,96611\_at,99128\_at,93014\_at,98910\_at,93582\_at,93258\_at,94275\_at,102194\_at,95760\_at,96909\_at,102970\_at,92388\_at,94276\_at,96627\_at,101097\_at,101680\_at,102019\_at,160431\_at,92578\_at,92646\_at,93062\_at,93579\_at,94252\_at,94494\_at,94870\_f\_at,94912\_at,95067\_at,95498\_at,96291\_f\_at,96292\_r\_at,96293\_at,97342\_at,97751\_f\_at,97824\_at,97884\_at,98120\_at,98524\_f\_at,98904\_at,99594\_at,95677\_at,103881\_at,92565\_at,161897\_f\_at,93236\_s\_at,93237\_s\_at,95497\_at,92625\_at,92824\_at | RIKEN cDNA 0610010K14 gene,RIKEN cDNA 0610010K14 gene,RIKEN cDNA 0610010K14 gene,triosephosphate isomerase,RIKEN cDNA 2310010I22 gene,RIKEN cDNA 3110052F15 gene,expressed sequence AI480570,spermidine synthase,expressed sequence C76683,6-pyruvoyl-tetrahydropterin synthase,quininoid dihydropteridine reductase,6-pyruvoyl-tetrahydropterin synthase/dimerization cofactor of hepatocyte nuclear factor 1 alpha (TCF1),dihydroorotate dehydrogenase,N-acetylneuraminic acid synthase (sialic acid synthase),calcitonin gene-related peptide-receptor component protein,DNA segment, Chr 11, ERATO Doi 497, expressed,mitogen-activated protein kinase kinase 1 interacting protein 1,ATP synthase, H+ transporting, mitochondrial F1 complex, gamma polypeptide 1,ATP synthase, H+ transporting, mitochondrial F1 complex, gamma polypeptide 1,ATP synthase, H+ transporting, mitochondrial F1 complex, gamma polypeptide 1,RIKEN cDNA 2410043G19 gene,ATPase, H+ transporting, V1 subunit F,NADH-ubiquinone oxidoreductase subunit B14.7,ATP synthase, H+ transporting, mitochondrial F1 complex, O subunit,ATP synthase, H+ transporting, mitochondrial F0 complex, subunit g,RIKEN cDNA 1110014K08 gene,demethyl-Q 7,hydroxymethylbilane synthase,uroporphyrinogen decarboxylase,RIKEN cDNA 2810432D09 gene,RIKEN cDNA 1110011K10 gene,NADH dehydrogenase (ubiquinone) 1, alpha/beta subcomplex, 1,proteasome (prosome, macropain) 26S subunit, ATPase 3, interacting protein,NA,hydroxysteroid (17-beta) dehydrogenase 12,phenylalkylamine Ca2+ antagonist (emopamil) binding protein,DNA segment, Chr 9, Wayne State University 149,ribosomal protein L27a,mitochondrial ribosomal protein L13,mitochondrial ribosomal protein L12,small inducible cytokine subfamily E, member 1,mitochondrial ribosomal protein L23,mitochondrial ribosomal protein L39,RIKEN cDNA 5830427H10 gene,eukaryotic translation initiation factor 2, subunit 3, structural gene X-linked,phenylalanine-tRNA synthetase-like,RIKEN cDNA 2310075M17 gene,mitochondrial ribosomal protein S21,mitochondrial ribosomal protein L2,mitochondrial ribosomal protein S15,NA,NA,RIKEN cDNA 2410015N17 gene,mitochondrial ribosomal protein S14,NA,DNA segment, Chr 11, ERATO Doi 175, expressed,mitochondrial ribosomal protein S11,mitochondrial ribosomal protein L27,RIKEN cDNA 2210039B01 gene,mitochondrial ribosomal protein L35,mitochondrial ribosomal protein L51,U5 snRNP-specific protein (Prp8-binding),inorganic pyrophosphatase 2,RIKEN cDNA 1110005A23 gene,phosphoribosyl pyrophosphate synthetase 1,thymidylate synthase,thymidylate synthase,RIKEN cDNA 1110005A05 gene,expressed in non-metastatic cells 2, protein,expressed in non-metastatic cells 6, protein |
| 5 | aromatic compound biosynthesis | 4 | 7 | 600 | 11544 | 0.007 | 0.001 | 10.934 | 0 | 97179\_at,160844\_at,96948\_at,99056\_at | expressed sequence C76683,6-pyruvoyl-tetrahydropterin synthase,quininoid dihydropteridine reductase,6-pyruvoyl-tetrahydropterin synthase/dimerization cofactor of hepatocyte nuclear factor 1 alpha (TCF1) |
| 6 | pteridine and derivative biosynthesis | 4 | 7 | 466 | 9498 | 0.009 | 0.001 | 11.595 | 0 | 97179\_at,160844\_at,96948\_at,99056\_at | expressed sequence C76683,6-pyruvoyl-tetrahydropterin synthase,quininoid dihydropteridine reductase,6-pyruvoyl-tetrahydropterin synthase/dimerization cofactor of hepatocyte nuclear factor 1 alpha (TCF1) |
| 7 | tetrahydrobiopterin biosynthesis | 3 | 4 | 298 | 6246 | 0.01 | 0.001 | 15.734 | 0 | 160844\_at,96948\_at,99056\_at | 6-pyruvoyl-tetrahydropterin synthase,quininoid dihydropteridine reductase,6-pyruvoyl-tetrahydropterin synthase/dimerization cofactor of hepatocyte nuclear factor 1 alpha (TCF1) |
| 5 | coenzymes and prosthetic group biosynthesis | 15 | 51 | 600 | 11544 | 0.025 | 0.004 | 5.656 | 0 | 97179\_at,160125\_at,160126\_at,92798\_at,92799\_g\_at,92800\_i\_at,93596\_i\_at,95656\_i\_at,96611\_at,99128\_at,93014\_at,98910\_at,93582\_at,93258\_at,94275\_at | expressed sequence C76683,DNA segment, Chr 11, ERATO Doi 497, expressed,mitogen-activated protein kinase kinase 1 interacting protein 1,ATP synthase, H+ transporting, mitochondrial F1 complex, gamma polypeptide 1,ATP synthase, H+ transporting, mitochondrial F1 complex, gamma polypeptide 1,ATP synthase, H+ transporting, mitochondrial F1 complex, gamma polypeptide 1,RIKEN cDNA 2410043G19 gene,ATPase, H+ transporting, V1 subunit F,NADH-ubiquinone oxidoreductase subunit B14.7,ATP synthase, H+ transporting, mitochondrial F1 complex, O subunit,ATP synthase, H+ transporting, mitochondrial F0 complex, subunit g,RIKEN cDNA 1110014K08 gene,demethyl-Q 7,hydroxymethylbilane synthase,uroporphyrinogen decarboxylase |
| 6 | coenzyme biosynthesis | 13 | 40 | 466 | 9498 | 0.028 | 0.004 | 6.627 | 0 | 97179\_at,160125\_at,160126\_at,92798\_at,92799\_g\_at,92800\_i\_at,93596\_i\_at,95656\_i\_at,96611\_at,99128\_at,93014\_at,98910\_at,93582\_at | expressed sequence C76683,DNA segment, Chr 11, ERATO Doi 497, expressed,mitogen-activated protein kinase kinase 1 interacting protein 1,ATP synthase, H+ transporting, mitochondrial F1 complex, gamma polypeptide 1,ATP synthase, H+ transporting, mitochondrial F1 complex, gamma polypeptide 1,ATP synthase, H+ transporting, mitochondrial F1 complex, gamma polypeptide 1,RIKEN cDNA 2410043G19 gene,ATPase, H+ transporting, V1 subunit F,NADH-ubiquinone oxidoreductase subunit B14.7,ATP synthase, H+ transporting, mitochondrial F1 complex, O subunit,ATP synthase, H+ transporting, mitochondrial F0 complex, subunit g,RIKEN cDNA 1110014K08 gene,demethyl-Q 7 |
| 7 | ATP biosynthesis | 10 | 24 | 298 | 6246 | 0.034 | 0.004 | 8.74 | 0 | 160125\_at,160126\_at,92798\_at,92799\_g\_at,92800\_i\_at,93596\_i\_at,95656\_i\_at,96611\_at,99128\_at,93014\_at | DNA segment, Chr 11, ERATO Doi 497, expressed,mitogen-activated protein kinase kinase 1 interacting protein 1,ATP synthase, H+ transporting, mitochondrial F1 complex, gamma polypeptide 1,ATP synthase, H+ transporting, mitochondrial F1 complex, gamma polypeptide 1,ATP synthase, H+ transporting, mitochondrial F1 complex, gamma polypeptide 1,RIKEN cDNA 2410043G19 gene,ATPase, H+ transporting, V1 subunit F,NADH-ubiquinone oxidoreductase subunit B14.7,ATP synthase, H+ transporting, mitochondrial F1 complex, O subunit,ATP synthase, H+ transporting, mitochondrial F0 complex, subunit g |
| 8 | ATP synthesis coupled proton transport | 2 | 5 | 130 | 2164 | 0.015 | 0.002 | 6.658 | 0.032 | 93014\_at,95656\_i\_at | ATP synthase, H+ transporting, mitochondrial F0 complex, subunit g,ATPase, H+ transporting, V1 subunit F |
| 7 | lipoic acid biosynthesis | 1 | 1 | 298 | 6246 | 0.003 | 0 | 21 | 0.048 | 98910\_at | RIKEN cDNA 1110014K08 gene |
| 7 | quinone cofactor biosynthesis | 1 | 1 | 298 | 6246 | 0.003 | 0 | 21 | 0.048 | 93582\_at | demethyl-Q 7 |
| 6 | fatty acid biosynthesis | 6 | 40 | 466 | 9498 | 0.013 | 0.004 | 3.059 | 0.013 | 102194\_at,95760\_at,96909\_at,99566\_at,102970\_at,92388\_at | RIKEN cDNA 2810432D09 gene,RIKEN cDNA 1110011K10 gene,NADH dehydrogenase (ubiquinone) 1, alpha/beta subcomplex, 1,triosephosphate isomerase,proteasome (prosome, macropain) 26S subunit, ATPase 3, interacting protein,NA |
| 7 | cholesterol biosynthesis | 4 | 17 | 298 | 6246 | 0.013 | 0.003 | 4.934 | 0.007 | 95634\_at,95635\_g\_at,95636\_at,96627\_at | RIKEN cDNA 0610010K14 gene,RIKEN cDNA 0610010K14 gene,RIKEN cDNA 0610010K14 gene,phenylalkylamine Ca2+ antagonist (emopamil) binding protein |
| 6 | sterol biosynthesis | 4 | 20 | 466 | 9498 | 0.009 | 0.002 | 4.066 | 0.015 | 96627\_at,95634\_at,95635\_g\_at,95636\_at | phenylalkylamine Ca2+ antagonist (emopamil) binding protein,RIKEN cDNA 0610010K14 gene,RIKEN cDNA 0610010K14 gene,RIKEN cDNA 0610010K14 gene |
| 7 | cholesterol biosynthesis | 4 | 17 | 298 | 6246 | 0.013 | 0.003 | 4.934 | 0.007 | 95634\_at,95635\_g\_at,95636\_at,96627\_at | RIKEN cDNA 0610010K14 gene,RIKEN cDNA 0610010K14 gene,RIKEN cDNA 0610010K14 gene,phenylalkylamine Ca2+ antagonist (emopamil) binding protein |
| 5 | macromolecule biosynthesis | 28 | 322 | 600 | 11544 | 0.047 | 0.028 | 1.673 | 0.005 | 101097\_at,101680\_at,102019\_at,160431\_at,92578\_at,92646\_at,93062\_at,93579\_at,94252\_at,94494\_at,94870\_f\_at,94912\_at,95067\_at,95498\_at,96291\_f\_at,96292\_r\_at,96293\_at,97342\_at,97751\_f\_at,97824\_at,97884\_at,98120\_at,98524\_f\_at,98904\_at,99594\_at,95677\_at,103881\_at,92565\_at | DNA segment, Chr 9, Wayne State University 149,ribosomal protein L27a,mitochondrial ribosomal protein L13,mitochondrial ribosomal protein L12,small inducible cytokine subfamily E, member 1,mitochondrial ribosomal protein L23,mitochondrial ribosomal protein L39,RIKEN cDNA 5830427H10 gene,eukaryotic translation initiation factor 2, subunit 3, structural gene X-linked,phenylalanine-tRNA synthetase-like,RIKEN cDNA 2310075M17 gene,mitochondrial ribosomal protein S21,mitochondrial ribosomal protein L2,mitochondrial ribosomal protein S15,NA,NA,RIKEN cDNA 2410015N17 gene,mitochondrial ribosomal protein S14,NA,DNA segment, Chr 11, ERATO Doi 175, expressed,mitochondrial ribosomal protein S11,mitochondrial ribosomal protein L27,RIKEN cDNA 2210039B01 gene,mitochondrial ribosomal protein L35,mitochondrial ribosomal protein L51,U5 snRNP-specific protein (Prp8-binding),inorganic pyrophosphatase 2,RIKEN cDNA 1110005A23 gene |
| 6 | protein biosynthesis | 28 | 322 | 466 | 9498 | 0.06 | 0.034 | 1.773 | 0.002 | 101097\_at,101680\_at,102019\_at,160431\_at,92578\_at,92646\_at,93062\_at,93579\_at,94252\_at,94494\_at,94870\_f\_at,94912\_at,95067\_at,95498\_at,96291\_f\_at,96292\_r\_at,96293\_at,97342\_at,97751\_f\_at,97824\_at,97884\_at,98120\_at,98524\_f\_at,98904\_at,99594\_at,95677\_at,103881\_at,92565\_at | DNA segment, Chr 9, Wayne State University 149,ribosomal protein L27a,mitochondrial ribosomal protein L13,mitochondrial ribosomal protein L12,small inducible cytokine subfamily E, member 1,mitochondrial ribosomal protein L23,mitochondrial ribosomal protein L39,RIKEN cDNA 5830427H10 gene,eukaryotic translation initiation factor 2, subunit 3, structural gene X-linked,phenylalanine-tRNA synthetase-like,RIKEN cDNA 2310075M17 gene,mitochondrial ribosomal protein S21,mitochondrial ribosomal protein L2,mitochondrial ribosomal protein S15,NA,NA,RIKEN cDNA 2410015N17 gene,mitochondrial ribosomal protein S14,NA,DNA segment, Chr 11, ERATO Doi 175, expressed,mitochondrial ribosomal protein S11,mitochondrial ribosomal protein L27,RIKEN cDNA 2210039B01 gene,mitochondrial ribosomal protein L35,mitochondrial ribosomal protein L51,U5 snRNP-specific protein (Prp8-binding),inorganic pyrophosphatase 2,RIKEN cDNA 1110005A23 gene |
| 5 | nucleotide biosynthesis | 17 | 74 | 600 | 11544 | 0.028 | 0.006 | 4.42 | 0 | 161897\_f\_at,93236\_s\_at,93237\_s\_at,95497\_at,160125\_at,160126\_at,92798\_at,92799\_g\_at,92800\_i\_at,93596\_i\_at,95656\_i\_at,96611\_at,99128\_at,93014\_at,92625\_at,92824\_at,103683\_at | phosphoribosyl pyrophosphate synthetase 1,thymidylate synthase,thymidylate synthase,RIKEN cDNA 1110005A05 gene,DNA segment, Chr 11, ERATO Doi 497, expressed,mitogen-activated protein kinase kinase 1 interacting protein 1,ATP synthase, H+ transporting, mitochondrial F1 complex, gamma polypeptide 1,ATP synthase, H+ transporting, mitochondrial F1 complex, gamma polypeptide 1,ATP synthase, H+ transporting, mitochondrial F1 complex, gamma polypeptide 1,RIKEN cDNA 2410043G19 gene,ATPase, H+ transporting, V1 subunit F,NADH-ubiquinone oxidoreductase subunit B14.7,ATP synthase, H+ transporting, mitochondrial F1 complex, O subunit,ATP synthase, H+ transporting, mitochondrial F0 complex, subunit g,expressed in non-metastatic cells 2, protein,expressed in non-metastatic cells 6, protein,dihydroorotate dehydrogenase |
| 6 | deoxyribonucleotide biosynthesis | 2 | 7 | 466 | 9498 | 0.004 | 0.001 | 5.797 | 0.043 | 93236\_s\_at,93237\_s\_at | thymidylate synthase,thymidylate synthase |
| 7 | deoxyribonucleoside monophosphate biosynthesis | 2 | 2 | 298 | 6246 | 0.007 | 0 | 20.969 | 0.002 | 93236\_s\_at,93237\_s\_at | thymidylate synthase,thymidylate synthase |
| 8 | pyrimidine deoxyribonucleoside monophosphate biosynthesis | 2 | 2 | 130 | 2164 | 0.015 | 0.001 | 16.717 | 0.004 | 93236\_s\_at,93237\_s\_at | thymidylate synthase,thymidylate synthase |
| 9 | dTMP biosynthesis | 2 | 2 | 66 | 911 | 0.03 | 0.002 | 13.773 | 0.005 | 93236\_s\_at,93237\_s\_at | thymidylate synthase,thymidylate synthase |
| 6 | nucleoside monophosphate biosynthesis | 3 | 15 | 466 | 9498 | 0.006 | 0.002 | 4.076 | 0.034 | 93236\_s\_at,93237\_s\_at,161897\_f\_at | thymidylate synthase,thymidylate synthase,phosphoribosyl pyrophosphate synthetase 1 |
| 7 | deoxyribonucleoside monophosphate biosynthesis | 2 | 2 | 298 | 6246 | 0.007 | 0 | 20.969 | 0.002 | 93236\_s\_at,93237\_s\_at | thymidylate synthase,thymidylate synthase |
| 8 | pyrimidine deoxyribonucleoside monophosphate biosynthesis | 2 | 2 | 130 | 2164 | 0.015 | 0.001 | 16.717 | 0.004 | 93236\_s\_at,93237\_s\_at | thymidylate synthase,thymidylate synthase |
| 9 | dTMP biosynthesis | 2 | 2 | 66 | 911 | 0.03 | 0.002 | 13.773 | 0.005 | 93236\_s\_at,93237\_s\_at | thymidylate synthase,thymidylate synthase |
| 6 | nucleoside triphosphate biosynthesis | 12 | 36 | 466 | 9498 | 0.026 | 0.004 | 6.794 | 0 | 160125\_at,160126\_at,92798\_at,92799\_g\_at,92800\_i\_at,93596\_i\_at,95656\_i\_at,96611\_at,99128\_at,93014\_at,92625\_at,92824\_at | DNA segment, Chr 11, ERATO Doi 497, expressed,mitogen-activated protein kinase kinase 1 interacting protein 1,ATP synthase, H+ transporting, mitochondrial F1 complex, gamma polypeptide 1,ATP synthase, H+ transporting, mitochondrial F1 complex, gamma polypeptide 1,ATP synthase, H+ transporting, mitochondrial F1 complex, gamma polypeptide 1,RIKEN cDNA 2410043G19 gene,ATPase, H+ transporting, V1 subunit F,NADH-ubiquinone oxidoreductase subunit B14.7,ATP synthase, H+ transporting, mitochondrial F1 complex, O subunit,ATP synthase, H+ transporting, mitochondrial F0 complex, subunit g,expressed in non-metastatic cells 2, protein,expressed in non-metastatic cells 6, protein |
| 7 | purine nucleoside triphosphate biosynthesis | 12 | 31 | 298 | 6246 | 0.04 | 0.005 | 8.119 | 0 | 160125\_at,160126\_at,92798\_at,92799\_g\_at,92800\_i\_at,93596\_i\_at,95656\_i\_at,96611\_at,99128\_at,93014\_at,92625\_at,92824\_at | DNA segment, Chr 11, ERATO Doi 497, expressed,mitogen-activated protein kinase kinase 1 interacting protein 1,ATP synthase, H+ transporting, mitochondrial F1 complex, gamma polypeptide 1,ATP synthase, H+ transporting, mitochondrial F1 complex, gamma polypeptide 1,ATP synthase, H+ transporting, mitochondrial F1 complex, gamma polypeptide 1,RIKEN cDNA 2410043G19 gene,ATPase, H+ transporting, V1 subunit F,NADH-ubiquinone oxidoreductase subunit B14.7,ATP synthase, H+ transporting, mitochondrial F1 complex, O subunit,ATP synthase, H+ transporting, mitochondrial F0 complex, subunit g,expressed in non-metastatic cells 2, protein,expressed in non-metastatic cells 6, protein |
| 8 | purine ribonucleoside triphosphate biosynthesis | 12 | 31 | 130 | 2164 | 0.092 | 0.014 | 6.442 | 0 | 160125\_at,160126\_at,92798\_at,92799\_g\_at,92800\_i\_at,93596\_i\_at,95656\_i\_at,96611\_at,99128\_at,93014\_at,92625\_at,92824\_at | DNA segment, Chr 11, ERATO Doi 497, expressed,mitogen-activated protein kinase kinase 1 interacting protein 1,ATP synthase, H+ transporting, mitochondrial F1 complex, gamma polypeptide 1,ATP synthase, H+ transporting, mitochondrial F1 complex, gamma polypeptide 1,ATP synthase, H+ transporting, mitochondrial F1 complex, gamma polypeptide 1,RIKEN cDNA 2410043G19 gene,ATPase, H+ transporting, V1 subunit F,NADH-ubiquinone oxidoreductase subunit B14.7,ATP synthase, H+ transporting, mitochondrial F1 complex, O subunit,ATP synthase, H+ transporting, mitochondrial F0 complex, subunit g,expressed in non-metastatic cells 2, protein,expressed in non-metastatic cells 6, protein |
| 8 | ATP synthesis coupled proton transport | 2 | 5 | 130 | 2164 | 0.015 | 0.002 | 6.658 | 0.032 | 93014\_at,95656\_i\_at | ATP synthase, H+ transporting, mitochondrial F0 complex, subunit g,ATPase, H+ transporting, V1 subunit F |
| 7 | ATP biosynthesis | 10 | 24 | 298 | 6246 | 0.034 | 0.004 | 8.74 | 0 | 160125\_at,160126\_at,92798\_at,92799\_g\_at,92800\_i\_at,93596\_i\_at,95656\_i\_at,96611\_at,99128\_at,93014\_at | DNA segment, Chr 11, ERATO Doi 497, expressed,mitogen-activated protein kinase kinase 1 interacting protein 1,ATP synthase, H+ transporting, mitochondrial F1 complex, gamma polypeptide 1,ATP synthase, H+ transporting, mitochondrial F1 complex, gamma polypeptide 1,ATP synthase, H+ transporting, mitochondrial F1 complex, gamma polypeptide 1,RIKEN cDNA 2410043G19 gene,ATPase, H+ transporting, V1 subunit F,NADH-ubiquinone oxidoreductase subunit B14.7,ATP synthase, H+ transporting, mitochondrial F1 complex, O subunit,ATP synthase, H+ transporting, mitochondrial F0 complex, subunit g |
| 8 | ATP synthesis coupled proton transport | 2 | 5 | 130 | 2164 | 0.015 | 0.002 | 6.658 | 0.032 | 93014\_at,95656\_i\_at | ATP synthase, H+ transporting, mitochondrial F0 complex, subunit g,ATPase, H+ transporting, V1 subunit F |
| 7 | ribonucleoside triphosphate biosynthesis | 12 | 31 | 298 | 6246 | 0.04 | 0.005 | 8.119 | 0 | 160125\_at,160126\_at,92798\_at,92799\_g\_at,92800\_i\_at,93596\_i\_at,95656\_i\_at,96611\_at,99128\_at,93014\_at,92625\_at,92824\_at | DNA segment, Chr 11, ERATO Doi 497, expressed,mitogen-activated protein kinase kinase 1 interacting protein 1,ATP synthase, H+ transporting, mitochondrial F1 complex, gamma polypeptide 1,ATP synthase, H+ transporting, mitochondrial F1 complex, gamma polypeptide 1,ATP synthase, H+ transporting, mitochondrial F1 complex, gamma polypeptide 1,RIKEN cDNA 2410043G19 gene,ATPase, H+ transporting, V1 subunit F,NADH-ubiquinone oxidoreductase subunit B14.7,ATP synthase, H+ transporting, mitochondrial F1 complex, O subunit,ATP synthase, H+ transporting, mitochondrial F0 complex, subunit g,expressed in non-metastatic cells 2, protein,expressed in non-metastatic cells 6, protein |
| 8 | purine ribonucleoside triphosphate biosynthesis | 12 | 31 | 130 | 2164 | 0.092 | 0.014 | 6.442 | 0 | 160125\_at,160126\_at,92798\_at,92799\_g\_at,92800\_i\_at,93596\_i\_at,95656\_i\_at,96611\_at,99128\_at,93014\_at,92625\_at,92824\_at | DNA segment, Chr 11, ERATO Doi 497, expressed,mitogen-activated protein kinase kinase 1 interacting protein 1,ATP synthase, H+ transporting, mitochondrial F1 complex, gamma polypeptide 1,ATP synthase, H+ transporting, mitochondrial F1 complex, gamma polypeptide 1,ATP synthase, H+ transporting, mitochondrial F1 complex, gamma polypeptide 1,RIKEN cDNA 2410043G19 gene,ATPase, H+ transporting, V1 subunit F,NADH-ubiquinone oxidoreductase subunit B14.7,ATP synthase, H+ transporting, mitochondrial F1 complex, O subunit,ATP synthase, H+ transporting, mitochondrial F0 complex, subunit g,expressed in non-metastatic cells 2, protein,expressed in non-metastatic cells 6, protein |
| 8 | ATP synthesis coupled proton transport | 2 | 5 | 130 | 2164 | 0.015 | 0.002 | 6.658 | 0.032 | 93014\_at,95656\_i\_at | ATP synthase, H+ transporting, mitochondrial F0 complex, subunit g,ATPase, H+ transporting, V1 subunit F |
| 7 | ATP biosynthesis | 10 | 24 | 298 | 6246 | 0.034 | 0.004 | 8.74 | 0 | 160125\_at,160126\_at,92798\_at,92799\_g\_at,92800\_i\_at,93596\_i\_at,95656\_i\_at,96611\_at,99128\_at,93014\_at | DNA segment, Chr 11, ERATO Doi 497, expressed,mitogen-activated protein kinase kinase 1 interacting protein 1,ATP synthase, H+ transporting, mitochondrial F1 complex, gamma polypeptide 1,ATP synthase, H+ transporting, mitochondrial F1 complex, gamma polypeptide 1,ATP synthase, H+ transporting, mitochondrial F1 complex, gamma polypeptide 1,RIKEN cDNA 2410043G19 gene,ATPase, H+ transporting, V1 subunit F,NADH-ubiquinone oxidoreductase subunit B14.7,ATP synthase, H+ transporting, mitochondrial F1 complex, O subunit,ATP synthase, H+ transporting, mitochondrial F0 complex, subunit g |
| 8 | ATP synthesis coupled proton transport | 2 | 5 | 130 | 2164 | 0.015 | 0.002 | 6.658 | 0.032 | 93014\_at,95656\_i\_at | ATP synthase, H+ transporting, mitochondrial F0 complex, subunit g,ATPase, H+ transporting, V1 subunit F |
| 5 | main pathways of carbohydrate metabolism | 10 | 84 | 600 | 11544 | 0.017 | 0.007 | 2.29 | 0.012 | 95693\_at,99566\_at,96268\_at,AFFX-GapdhMur/M32599\_3\_at,AFFX-GapdhMur/M32599\_5\_at,97279\_at,99148\_at,93029\_at,93991\_at,95053\_s\_at | NA,triosephosphate isomerase,succinate-CoA ligase, GDP-forming, alpha subunit,glyceraldehyde-3-phosphate dehydrogenase,glyceraldehyde-3-phosphate dehydrogenase,RIKEN cDNA 6430402H10 gene,fumarate hydratase 1,isocitrate dehydrogenase 3 (NAD+), gamma,malate dehydrogenase, mitochondrial,succinate dehydrogenase complex, subunit B, iron sulfur (Ip) |
| 7 | fumarate metabolism | 1 | 1 | 298 | 6246 | 0.003 | 0 | 21 | 0.048 | 99148\_at | fumarate hydratase 1 |
| 6 | tricarboxylic acid cycle | 6 | 19 | 466 | 9498 | 0.013 | 0.002 | 6.44 | 0 | 93029\_at,93991\_at,95053\_s\_at,95693\_at,96268\_at,99148\_at | isocitrate dehydrogenase 3 (NAD+), gamma,malate dehydrogenase, mitochondrial,succinate dehydrogenase complex, subunit B, iron sulfur (Ip),NA,succinate-CoA ligase, GDP-forming, alpha subunit,fumarate hydratase 1 |
| 4 | catabolism | 44 | 631 | 695 | 13100 | 0.063 | 0.048 | 1.314 | 0.038 | 93542\_at,96268\_at,99566\_at,AFFX-GapdhMur/M32599\_3\_at,AFFX-GapdhMur/M32599\_5\_at,97279\_at,160844\_at,95408\_at,97318\_at,104567\_at,100576\_at,100539\_at,93734\_i\_at,93735\_f\_at,94025\_at,95448\_at,100543\_s\_at,101562\_at,104541\_at,92874\_f\_at,95561\_at,96093\_at,96733\_at,99655\_at,93519\_s\_at,100512\_at,100733\_at,101486\_at,101558\_s\_at,101992\_at,102791\_at,92547\_at,93085\_at,93988\_at,94263\_f\_at,94841\_at,96892\_at,96952\_at,97459\_at,98557\_f\_at,98975\_at,93203\_f\_at,94372\_at,162417\_at | phosphotriesterase related,succinate-CoA ligase, GDP-forming, alpha subunit,triosephosphate isomerase,glyceraldehyde-3-phosphate dehydrogenase,glyceraldehyde-3-phosphate dehydrogenase,RIKEN cDNA 6430402H10 gene,6-pyruvoyl-tetrahydropterin synthase,RIKEN cDNA 2310003F16 gene,histidyl tRNA synthetase 2,RIKEN cDNA 3110052F15 gene,platelet-activating factor acetylhydrolase, isoform 1b, alpha1 subunit,brain acyl-CoA hydrolase,proteasome (prosome, macropain) 26S subunit, ATPase 3,proteasome (prosome, macropain) 26S subunit, ATPase 3,proteasome (prosome, macropain) subunit, beta type 3,proteasome (prosome, macropain) 26S subunit, ATPase 2,bromodomain containing 7,heat shock protein 4,proteinase 3,COP9 (constitutive photomorphogenic) homolog, subunit 7a (Arabidopsis thaliana),RIKEN cDNA 1700013H19 gene,open reading frame 11,RAP1, GTP-GDP dissociation stimulator 1,signal peptidase complex,neural precursor cell expressed, developmentally down-regulated gene 8,ubiquitin carboxyl-terminal esterase L5,proteasome (prosome, macropain) subunit, alpha type 2,proteasome (prosome, macropain) subunit, beta type 10,proteasome (prosome, macropain) subunit, beta type 5,proteasome (prosome, macropain) subunit, beta type 6,proteosome (prosome, macropain) subunit, beta type 8 (large multifunctional protease 7),huntingtin interacting protein 2,proteosome (prosome, macropain) subunit, beta type 9 (large multifunctional protease 2),proteasome (prosome, macropain) subunit, alpha type 7,proteasome (prosome, macropain) subunit, beta type 7,proteasome (prosome, macropain) subunit, alpha type 5,proteasome (prosome, macropain) subunit, alpha type 1,proteasome (prosome, macropain) subunit, alpha type 6,proteasome (prosome, macropain) subunit, alpha type 4,proteasome (prosome, macropain) subunit, beta type 4,RIKEN cDNA 2410008G02 gene,RIKEN cDNA 3230402J05 gene,nudix (nucleoside diphosphate linked moiety X)-type motif 1,RIKEN cDNA 1500001M20 gene |
| 6 | amino acid catabolism | 4 | 24 | 466 | 9498 | 0.009 | 0.003 | 3.391 | 0.028 | 160844\_at,95408\_at,97318\_at,104567\_at | 6-pyruvoyl-tetrahydropterin synthase,RIKEN cDNA 2310003F16 gene,histidyl tRNA synthetase 2,RIKEN cDNA 3110052F15 gene |
| 8 | phenylalanine catabolism | 2 | 6 | 130 | 2164 | 0.015 | 0.003 | 5.552 | 0.046 | 160844\_at,95408\_at | 6-pyruvoyl-tetrahydropterin synthase,RIKEN cDNA 2310003F16 gene |
| 7 | D-amino acid catabolism | 1 | 1 | 298 | 6246 | 0.003 | 0 | 21 | 0.048 | 97318\_at | histidyl tRNA synthetase 2 |
| 6 | fatty acid catabolism | 1 | 1 | 466 | 9498 | 0.002 | 0 | 19.545 | 0.049 | 100539\_at | brain acyl-CoA hydrolase |
| 8 | modification-dependent protein catabolism | 18 | 122 | 130 | 2164 | 0.138 | 0.056 | 2.456 | 0 | 93519\_s\_at,100512\_at,100733\_at,101486\_at,101558\_s\_at,101992\_at,102791\_at,92547\_at,93085\_at,93988\_at,94025\_at,94263\_f\_at,94841\_at,96892\_at,96952\_at,97459\_at,98557\_f\_at,98975\_at | neural precursor cell expressed, developmentally down-regulated gene 8,ubiquitin carboxyl-terminal esterase L5,proteasome (prosome, macropain) subunit, alpha type 2,proteasome (prosome, macropain) subunit, beta type 10,proteasome (prosome, macropain) subunit, beta type 5,proteasome (prosome, macropain) subunit, beta type 6,proteosome (prosome, macropain) subunit, beta type 8 (large multifunctional protease 7),huntingtin interacting protein 2,proteosome (prosome, macropain) subunit, beta type 9 (large multifunctional protease 2),proteasome (prosome, macropain) subunit, alpha type 7,proteasome (prosome, macropain) subunit, beta type 3,proteasome (prosome, macropain) subunit, beta type 7,proteasome (prosome, macropain) subunit, alpha type 5,proteasome (prosome, macropain) subunit, alpha type 1,proteasome (prosome, macropain) subunit, alpha type 6,proteasome (prosome, macropain) subunit, alpha type 4,proteasome (prosome, macropain) subunit, beta type 4,RIKEN cDNA 2410008G02 gene |
| 9 | ubiquitin-dependent protein catabolism | 17 | 120 | 66 | 911 | 0.258 | 0.132 | 1.956 | 0.003 | 100512\_at,100733\_at,101486\_at,101558\_s\_at,101992\_at,102791\_at,92547\_at,93085\_at,93988\_at,94025\_at,94263\_f\_at,94841\_at,96892\_at,96952\_at,97459\_at,98557\_f\_at,98975\_at | ubiquitin carboxyl-terminal esterase L5,proteasome (prosome, macropain) subunit, alpha type 2,proteasome (prosome, macropain) subunit, beta type 10,proteasome (prosome, macropain) subunit, beta type 5,proteasome (prosome, macropain) subunit, beta type 6,proteosome (prosome, macropain) subunit, beta type 8 (large multifunctional protease 7),huntingtin interacting protein 2,proteosome (prosome, macropain) subunit, beta type 9 (large multifunctional protease 2),proteasome (prosome, macropain) subunit, alpha type 7,proteasome (prosome, macropain) subunit, beta type 3,proteasome (prosome, macropain) subunit, beta type 7,proteasome (prosome, macropain) subunit, alpha type 5,proteasome (prosome, macropain) subunit, alpha type 1,proteasome (prosome, macropain) subunit, alpha type 6,proteasome (prosome, macropain) subunit, alpha type 4,proteasome (prosome, macropain) subunit, beta type 4,RIKEN cDNA 2410008G02 gene |
| 5 | nucleotide catabolism | 3 | 10 | 600 | 11544 | 0.005 | 0.001 | 5.747 | 0.013 | 93203\_f\_at,94372\_at,162417\_at | RIKEN cDNA 3230402J05 gene,nudix (nucleoside diphosphate linked moiety X)-type motif 1,RIKEN cDNA 1500001M20 gene |
| 4 | coenzymes and prosthetic group metabolism | 16 | 95 | 695 | 13100 | 0.023 | 0.007 | 3.175 | 0 | 97179\_at,160125\_at,160126\_at,92798\_at,92799\_g\_at,92800\_i\_at,93596\_i\_at,95656\_i\_at,96611\_at,99128\_at,93014\_at,98910\_at,93582\_at,93258\_at,94275\_at,103581\_at | expressed sequence C76683,DNA segment, Chr 11, ERATO Doi 497, expressed,mitogen-activated protein kinase kinase 1 interacting protein 1,ATP synthase, H+ transporting, mitochondrial F1 complex, gamma polypeptide 1,ATP synthase, H+ transporting, mitochondrial F1 complex, gamma polypeptide 1,ATP synthase, H+ transporting, mitochondrial F1 complex, gamma polypeptide 1,RIKEN cDNA 2410043G19 gene,ATPase, H+ transporting, V1 subunit F,NADH-ubiquinone oxidoreductase subunit B14.7,ATP synthase, H+ transporting, mitochondrial F1 complex, O subunit,ATP synthase, H+ transporting, mitochondrial F0 complex, subunit g,RIKEN cDNA 1110014K08 gene,demethyl-Q 7,hydroxymethylbilane synthase,uroporphyrinogen decarboxylase,cytosolic acyl-CoA thioesterase 1 |
| 5 | coenzymes and prosthetic group biosynthesis | 15 | 51 | 600 | 11544 | 0.025 | 0.004 | 5.656 | 0 | 97179\_at,160125\_at,160126\_at,92798\_at,92799\_g\_at,92800\_i\_at,93596\_i\_at,95656\_i\_at,96611\_at,99128\_at,93014\_at,98910\_at,93582\_at,93258\_at,94275\_at | expressed sequence C76683,DNA segment, Chr 11, ERATO Doi 497, expressed,mitogen-activated protein kinase kinase 1 interacting protein 1,ATP synthase, H+ transporting, mitochondrial F1 complex, gamma polypeptide 1,ATP synthase, H+ transporting, mitochondrial F1 complex, gamma polypeptide 1,ATP synthase, H+ transporting, mitochondrial F1 complex, gamma polypeptide 1,RIKEN cDNA 2410043G19 gene,ATPase, H+ transporting, V1 subunit F,NADH-ubiquinone oxidoreductase subunit B14.7,ATP synthase, H+ transporting, mitochondrial F1 complex, O subunit,ATP synthase, H+ transporting, mitochondrial F0 complex, subunit g,RIKEN cDNA 1110014K08 gene,demethyl-Q 7,hydroxymethylbilane synthase,uroporphyrinogen decarboxylase |
| 6 | coenzyme biosynthesis | 13 | 40 | 466 | 9498 | 0.028 | 0.004 | 6.627 | 0 | 97179\_at,160125\_at,160126\_at,92798\_at,92799\_g\_at,92800\_i\_at,93596\_i\_at,95656\_i\_at,96611\_at,99128\_at,93014\_at,98910\_at,93582\_at | expressed sequence C76683,DNA segment, Chr 11, ERATO Doi 497, expressed,mitogen-activated protein kinase kinase 1 interacting protein 1,ATP synthase, H+ transporting, mitochondrial F1 complex, gamma polypeptide 1,ATP synthase, H+ transporting, mitochondrial F1 complex, gamma polypeptide 1,ATP synthase, H+ transporting, mitochondrial F1 complex, gamma polypeptide 1,RIKEN cDNA 2410043G19 gene,ATPase, H+ transporting, V1 subunit F,NADH-ubiquinone oxidoreductase subunit B14.7,ATP synthase, H+ transporting, mitochondrial F1 complex, O subunit,ATP synthase, H+ transporting, mitochondrial F0 complex, subunit g,RIKEN cDNA 1110014K08 gene,demethyl-Q 7 |
| 7 | ATP biosynthesis | 10 | 24 | 298 | 6246 | 0.034 | 0.004 | 8.74 | 0 | 160125\_at,160126\_at,92798\_at,92799\_g\_at,92800\_i\_at,93596\_i\_at,95656\_i\_at,96611\_at,99128\_at,93014\_at | DNA segment, Chr 11, ERATO Doi 497, expressed,mitogen-activated protein kinase kinase 1 interacting protein 1,ATP synthase, H+ transporting, mitochondrial F1 complex, gamma polypeptide 1,ATP synthase, H+ transporting, mitochondrial F1 complex, gamma polypeptide 1,ATP synthase, H+ transporting, mitochondrial F1 complex, gamma polypeptide 1,RIKEN cDNA 2410043G19 gene,ATPase, H+ transporting, V1 subunit F,NADH-ubiquinone oxidoreductase subunit B14.7,ATP synthase, H+ transporting, mitochondrial F1 complex, O subunit,ATP synthase, H+ transporting, mitochondrial F0 complex, subunit g |
| 8 | ATP synthesis coupled proton transport | 2 | 5 | 130 | 2164 | 0.015 | 0.002 | 6.658 | 0.032 | 93014\_at,95656\_i\_at | ATP synthase, H+ transporting, mitochondrial F0 complex, subunit g,ATPase, H+ transporting, V1 subunit F |
| 7 | lipoic acid biosynthesis | 1 | 1 | 298 | 6246 | 0.003 | 0 | 21 | 0.048 | 98910\_at | RIKEN cDNA 1110014K08 gene |
| 7 | quinone cofactor biosynthesis | 1 | 1 | 298 | 6246 | 0.003 | 0 | 21 | 0.048 | 93582\_at | demethyl-Q 7 |
| 5 | coenzyme metabolism | 14 | 80 | 600 | 11544 | 0.023 | 0.007 | 3.367 | 0 | 97179\_at,160125\_at,160126\_at,92798\_at,92799\_g\_at,92800\_i\_at,93596\_i\_at,95656\_i\_at,96611\_at,99128\_at,93014\_at,98910\_at,93582\_at,103581\_at | expressed sequence C76683,DNA segment, Chr 11, ERATO Doi 497, expressed,mitogen-activated protein kinase kinase 1 interacting protein 1,ATP synthase, H+ transporting, mitochondrial F1 complex, gamma polypeptide 1,ATP synthase, H+ transporting, mitochondrial F1 complex, gamma polypeptide 1,ATP synthase, H+ transporting, mitochondrial F1 complex, gamma polypeptide 1,RIKEN cDNA 2410043G19 gene,ATPase, H+ transporting, V1 subunit F,NADH-ubiquinone oxidoreductase subunit B14.7,ATP synthase, H+ transporting, mitochondrial F1 complex, O subunit,ATP synthase, H+ transporting, mitochondrial F0 complex, subunit g,RIKEN cDNA 1110014K08 gene,demethyl-Q 7,cytosolic acyl-CoA thioesterase 1 |
| 6 | coenzyme biosynthesis | 13 | 40 | 466 | 9498 | 0.028 | 0.004 | 6.627 | 0 | 97179\_at,160125\_at,160126\_at,92798\_at,92799\_g\_at,92800\_i\_at,93596\_i\_at,95656\_i\_at,96611\_at,99128\_at,93014\_at,98910\_at,93582\_at | expressed sequence C76683,DNA segment, Chr 11, ERATO Doi 497, expressed,mitogen-activated protein kinase kinase 1 interacting protein 1,ATP synthase, H+ transporting, mitochondrial F1 complex, gamma polypeptide 1,ATP synthase, H+ transporting, mitochondrial F1 complex, gamma polypeptide 1,ATP synthase, H+ transporting, mitochondrial F1 complex, gamma polypeptide 1,RIKEN cDNA 2410043G19 gene,ATPase, H+ transporting, V1 subunit F,NADH-ubiquinone oxidoreductase subunit B14.7,ATP synthase, H+ transporting, mitochondrial F1 complex, O subunit,ATP synthase, H+ transporting, mitochondrial F0 complex, subunit g,RIKEN cDNA 1110014K08 gene,demethyl-Q 7 |
| 7 | ATP biosynthesis | 10 | 24 | 298 | 6246 | 0.034 | 0.004 | 8.74 | 0 | 160125\_at,160126\_at,92798\_at,92799\_g\_at,92800\_i\_at,93596\_i\_at,95656\_i\_at,96611\_at,99128\_at,93014\_at | DNA segment, Chr 11, ERATO Doi 497, expressed,mitogen-activated protein kinase kinase 1 interacting protein 1,ATP synthase, H+ transporting, mitochondrial F1 complex, gamma polypeptide 1,ATP synthase, H+ transporting, mitochondrial F1 complex, gamma polypeptide 1,ATP synthase, H+ transporting, mitochondrial F1 complex, gamma polypeptide 1,RIKEN cDNA 2410043G19 gene,ATPase, H+ transporting, V1 subunit F,NADH-ubiquinone oxidoreductase subunit B14.7,ATP synthase, H+ transporting, mitochondrial F1 complex, O subunit,ATP synthase, H+ transporting, mitochondrial F0 complex, subunit g |
| 8 | ATP synthesis coupled proton transport | 2 | 5 | 130 | 2164 | 0.015 | 0.002 | 6.658 | 0.032 | 93014\_at,95656\_i\_at | ATP synthase, H+ transporting, mitochondrial F0 complex, subunit g,ATPase, H+ transporting, V1 subunit F |
| 7 | lipoic acid biosynthesis | 1 | 1 | 298 | 6246 | 0.003 | 0 | 21 | 0.048 | 98910\_at | RIKEN cDNA 1110014K08 gene |
| 7 | quinone cofactor biosynthesis | 1 | 1 | 298 | 6246 | 0.003 | 0 | 21 | 0.048 | 93582\_at | demethyl-Q 7 |
| 4 | electron transport | 25 | 313 | 695 | 13100 | 0.036 | 0.024 | 1.506 | 0.027 | 100057\_at,100059\_at,100156\_at,100550\_f\_at,100568\_at,103619\_at,103671\_at,92388\_at,93041\_at,93742\_at,93820\_at,95045\_at,95053\_s\_at,95696\_at,96112\_at,96861\_at,96947\_at,97013\_f\_at,98613\_at,99618\_at,100079\_at,94062\_at,96267\_at,96899\_at,96902\_at | RIKEN cDNA 2510027N19 gene,cytochrome b-245, alpha polypeptide,minichromosome maintenance deficient 5, cell division cycle 46 (S. cerevisiae),cytochrome c oxidase, subunit VIc,ATP-binding cassette, sub-family E (OABP), member 1,cytochrome b5 outer mitochondrial membrane precursor,HIV-1 tat interactive protein 2, homolog (human),NA,minichromosome maintenance deficient 4 homolog (S. cerevisiae),RIKEN cDNA 5730449L18 gene,cytochrome c oxidase, subunit VIIa 2,RIKEN cDNA 0610012D09 gene,succinate dehydrogenase complex, subunit B, iron sulfur (Ip),thioredoxin-like 2,electron transferring flavoprotein, alpha polypeptide,mitochondrial ribosomal protein L50,RIKEN cDNA 0610009I16 gene,cytochrome b-245, alpha polypeptide,RIKEN cDNA 2700085E05 gene,ubiquinol-cytochrome c reductase subunit,NADH dehydrogenase (ubiquinone) 1 beta subcomplex, 9,NADH dehydrogenase (ubiquinone) flavoprotein 2,NADH dehydrogenase (ubiquinone) flavoprotein 1,NADH dehydrogenase (ubiquinone) Fe-S protein 3,RIKEN cDNA 2900091E11 gene |
| 5 | ATP synthesis coupled electron transport | 5 | 7 | 600 | 11544 | 0.008 | 0.001 | 13.656 | 0 | 100079\_at,94062\_at,96267\_at,96899\_at,96902\_at | NADH dehydrogenase (ubiquinone) 1 beta subcomplex, 9,NADH dehydrogenase (ubiquinone) flavoprotein 2,NADH dehydrogenase (ubiquinone) flavoprotein 1,NADH dehydrogenase (ubiquinone) Fe-S protein 3,RIKEN cDNA 2900091E11 gene |
| 6 | ATP synthesis coupled electron transport (sensu Eukarya) | 5 | 7 | 466 | 9498 | 0.011 | 0.001 | 14.5 | 0 | 100079\_at,94062\_at,96267\_at,96899\_at,96902\_at | NADH dehydrogenase (ubiquinone) 1 beta subcomplex, 9,NADH dehydrogenase (ubiquinone) flavoprotein 2,NADH dehydrogenase (ubiquinone) flavoprotein 1,NADH dehydrogenase (ubiquinone) Fe-S protein 3,RIKEN cDNA 2900091E11 gene |
| 7 | mitochondrial electron transport, NADH to ubiquinone | 5 | 6 | 298 | 6246 | 0.017 | 0.001 | 17.479 | 0 | 100079\_at,94062\_at,96267\_at,96899\_at,96902\_at | NADH dehydrogenase (ubiquinone) 1 beta subcomplex, 9,NADH dehydrogenase (ubiquinone) flavoprotein 2,NADH dehydrogenase (ubiquinone) flavoprotein 1,NADH dehydrogenase (ubiquinone) Fe-S protein 3,RIKEN cDNA 2900091E11 gene |
| 4 | energy pathways | 13 | 104 | 695 | 13100 | 0.019 | 0.008 | 2.356 | 0.003 | 92798\_at,92799\_g\_at,92800\_i\_at,95693\_at,99566\_at,96268\_at,AFFX-GapdhMur/M32599\_3\_at,AFFX-GapdhMur/M32599\_5\_at,97279\_at,99148\_at,93029\_at,93991\_at,95053\_s\_at | ATP synthase, H+ transporting, mitochondrial F1 complex, gamma polypeptide 1,ATP synthase, H+ transporting, mitochondrial F1 complex, gamma polypeptide 1,ATP synthase, H+ transporting, mitochondrial F1 complex, gamma polypeptide 1,NA,triosephosphate isomerase,succinate-CoA ligase, GDP-forming, alpha subunit,glyceraldehyde-3-phosphate dehydrogenase,glyceraldehyde-3-phosphate dehydrogenase,RIKEN cDNA 6430402H10 gene,fumarate hydratase 1,isocitrate dehydrogenase 3 (NAD+), gamma,malate dehydrogenase, mitochondrial,succinate dehydrogenase complex, subunit B, iron sulfur (Ip) |
| 5 | energy derivation by oxidation of organic compounds | 10 | 100 | 600 | 11544 | 0.017 | 0.009 | 1.925 | 0.035 | 95693\_at,99566\_at,96268\_at,AFFX-GapdhMur/M32599\_3\_at,AFFX-GapdhMur/M32599\_5\_at,97279\_at,99148\_at,93029\_at,93991\_at,95053\_s\_at | NA,triosephosphate isomerase,succinate-CoA ligase, GDP-forming, alpha subunit,glyceraldehyde-3-phosphate dehydrogenase,glyceraldehyde-3-phosphate dehydrogenase,RIKEN cDNA 6430402H10 gene,fumarate hydratase 1,isocitrate dehydrogenase 3 (NAD+), gamma,malate dehydrogenase, mitochondrial,succinate dehydrogenase complex, subunit B, iron sulfur (Ip) |
| 7 | fumarate metabolism | 1 | 1 | 298 | 6246 | 0.003 | 0 | 21 | 0.048 | 99148\_at | fumarate hydratase 1 |
| 6 | tricarboxylic acid cycle | 6 | 19 | 466 | 9498 | 0.013 | 0.002 | 6.44 | 0 | 93029\_at,93991\_at,95053\_s\_at,95693\_at,96268\_at,99148\_at | isocitrate dehydrogenase 3 (NAD+), gamma,malate dehydrogenase, mitochondrial,succinate dehydrogenase complex, subunit B, iron sulfur (Ip),NA,succinate-CoA ligase, GDP-forming, alpha subunit,fumarate hydratase 1 |
| 6 | cellular respiration | 1 | 1 | 466 | 9498 | 0.002 | 0 | 19.545 | 0.049 | 95053\_s\_at | succinate dehydrogenase complex, subunit B, iron sulfur (Ip) |
| 7 | aerobic respiration | 1 | 1 | 298 | 6246 | 0.003 | 0 | 21 | 0.048 | 95053\_s\_at | succinate dehydrogenase complex, subunit B, iron sulfur (Ip) |
| 5 | main pathways of carbohydrate metabolism | 10 | 84 | 600 | 11544 | 0.017 | 0.007 | 2.29 | 0.012 | 95693\_at,99566\_at,96268\_at,AFFX-GapdhMur/M32599\_3\_at,AFFX-GapdhMur/M32599\_5\_at,97279\_at,99148\_at,93029\_at,93991\_at,95053\_s\_at | NA,triosephosphate isomerase,succinate-CoA ligase, GDP-forming, alpha subunit,glyceraldehyde-3-phosphate dehydrogenase,glyceraldehyde-3-phosphate dehydrogenase,RIKEN cDNA 6430402H10 gene,fumarate hydratase 1,isocitrate dehydrogenase 3 (NAD+), gamma,malate dehydrogenase, mitochondrial,succinate dehydrogenase complex, subunit B, iron sulfur (Ip) |
| 7 | fumarate metabolism | 1 | 1 | 298 | 6246 | 0.003 | 0 | 21 | 0.048 | 99148\_at | fumarate hydratase 1 |
| 6 | tricarboxylic acid cycle | 6 | 19 | 466 | 9498 | 0.013 | 0.002 | 6.44 | 0 | 93029\_at,93991\_at,95053\_s\_at,95693\_at,96268\_at,99148\_at | isocitrate dehydrogenase 3 (NAD+), gamma,malate dehydrogenase, mitochondrial,succinate dehydrogenase complex, subunit B, iron sulfur (Ip),NA,succinate-CoA ligase, GDP-forming, alpha subunit,fumarate hydratase 1 |
| 6 | fatty acid biosynthesis | 6 | 40 | 466 | 9498 | 0.013 | 0.004 | 3.059 | 0.013 | 102194\_at,95760\_at,96909\_at,99566\_at,102970\_at,92388\_at | RIKEN cDNA 2810432D09 gene,RIKEN cDNA 1110011K10 gene,NADH dehydrogenase (ubiquinone) 1, alpha/beta subcomplex, 1,triosephosphate isomerase,proteasome (prosome, macropain) 26S subunit, ATPase 3, interacting protein,NA |
| 7 | cholesterol biosynthesis | 4 | 17 | 298 | 6246 | 0.013 | 0.003 | 4.934 | 0.007 | 95634\_at,95635\_g\_at,95636\_at,96627\_at | RIKEN cDNA 0610010K14 gene,RIKEN cDNA 0610010K14 gene,RIKEN cDNA 0610010K14 gene,phenylalkylamine Ca2+ antagonist (emopamil) binding protein |
| 6 | sterol biosynthesis | 4 | 20 | 466 | 9498 | 0.009 | 0.002 | 4.066 | 0.015 | 96627\_at,95634\_at,95635\_g\_at,95636\_at | phenylalkylamine Ca2+ antagonist (emopamil) binding protein,RIKEN cDNA 0610010K14 gene,RIKEN cDNA 0610010K14 gene,RIKEN cDNA 0610010K14 gene |
| 7 | cholesterol biosynthesis | 4 | 17 | 298 | 6246 | 0.013 | 0.003 | 4.934 | 0.007 | 95634\_at,95635\_g\_at,95636\_at,96627\_at | RIKEN cDNA 0610010K14 gene,RIKEN cDNA 0610010K14 gene,RIKEN cDNA 0610010K14 gene,phenylalkylamine Ca2+ antagonist (emopamil) binding protein |
| 6 | fatty acid catabolism | 1 | 1 | 466 | 9498 | 0.002 | 0 | 19.545 | 0.049 | 100539\_at | brain acyl-CoA hydrolase |
| 5 | fatty acid metabolism | 14 | 85 | 600 | 11544 | 0.023 | 0.007 | 3.17 | 0 | 100539\_at,93754\_at,95064\_at,95426\_at,95485\_at,98527\_at,102194\_at,95760\_at,96909\_at,99566\_at,102970\_at,92388\_at,103581\_at,99106\_at | brain acyl-CoA hydrolase,enoyl coenzyme A hydratase 1, peroxisomal,acetyl-Coenzyme A acyltransferase 2 (mitochondrial 3-oxoacyl-Coenzyme A thiolase),enoyl Coenzyme A hydratase, short chain, 1, mitochondrial,L-3-hydroxyacyl-Coenzyme A dehydrogenase, short chain,dodecenoyl-Coenzyme A delta isomerase (3,2 trans-enoyl-Coenyme A isomerase),RIKEN cDNA 2810432D09 gene,RIKEN cDNA 1110011K10 gene,NADH dehydrogenase (ubiquinone) 1, alpha/beta subcomplex, 1,triosephosphate isomerase,proteasome (prosome, macropain) 26S subunit, ATPase 3, interacting protein,NA,cytosolic acyl-CoA thioesterase 1,COP9 (constitutive photomorphogenic) homolog, subunit 6 (Arabidopsis thaliana) |
| 6 | fatty acid biosynthesis | 6 | 40 | 466 | 9498 | 0.013 | 0.004 | 3.059 | 0.013 | 102194\_at,95760\_at,96909\_at,99566\_at,102970\_at,92388\_at | RIKEN cDNA 2810432D09 gene,RIKEN cDNA 1110011K10 gene,NADH dehydrogenase (ubiquinone) 1, alpha/beta subcomplex, 1,triosephosphate isomerase,proteasome (prosome, macropain) 26S subunit, ATPase 3, interacting protein,NA |
| 6 | fatty acid catabolism | 1 | 1 | 466 | 9498 | 0.002 | 0 | 19.545 | 0.049 | 100539\_at | brain acyl-CoA hydrolase |
| 6 | long-chain fatty acid metabolism | 1 | 1 | 466 | 9498 | 0.002 | 0 | 19.545 | 0.049 | 103581\_at | cytosolic acyl-CoA thioesterase 1 |
| 5 | nucleobase metabolism | 3 | 12 | 600 | 11544 | 0.005 | 0.001 | 4.808 | 0.022 | 103683\_at,161897\_f\_at,160293\_at | dihydroorotate dehydrogenase,phosphoribosyl pyrophosphate synthetase 1,RIKEN cDNA 2700038L12 gene |
| 5 | DNA metabolism | 28 | 302 | 600 | 11544 | 0.047 | 0.026 | 1.784 | 0.002 | 101254\_at,102631\_at,96081\_at,100156\_at,100612\_at,101065\_at,101067\_at,102001\_at,103418\_at,104738\_at,93041\_at,93112\_at,96289\_at,98975\_at,101105\_at,93095\_at,93251\_at,96699\_at,98039\_at,94897\_at,96775\_at,101954\_at,93833\_s\_at,98587\_at,95660\_at,100459\_at,102853\_at,93559\_at | RAN, member RAS oncogene family,Bloom syndrome homolog (human),thymidine kinase 1,minichromosome maintenance deficient 5, cell division cycle 46 (S. cerevisiae),ribonucleotide reductase M1,proliferating cell nuclear antigen,RIKEN cDNA 2010005E08 gene,ribonucleotide reductase M2,expressed sequence AU040575,zuotin related factor 2,minichromosome maintenance deficient 4 homolog (S. cerevisiae),minichromosome maintenance deficient 2 mitotin (S. cerevisiae),stomatin (Epb7.2)-like 2,RIKEN cDNA 2410008G02 gene,barrier to autointegration factor 1,high mobility group box 1,4-nitrophenylphosphatase domain and non-neuronal SNAP25-like protein homolog 1 (C. elegans),high mobility group nucleosomal binding domain 1,RIKEN cDNA 2410015M20 gene,glutathione peroxidase 4,chromobox homolog 1 (Drosophila HP1 beta),H2A histone family, member Z,histone 1, H2bl,nucleosome assembly protein 1-like 1,RIKEN cDNA 0610025L15 gene,RAD50 homolog (S. cerevisiae),chondroitin sulfate proteoglycan 6,apurinic/apyrimidinic endonuclease 1 |
| 6 | DNA packaging | 11 | 93 | 466 | 9498 | 0.024 | 0.01 | 2.412 | 0.006 | 93095\_at,93251\_at,96699\_at,98039\_at,94897\_at,96775\_at,101954\_at,93112\_at,93833\_s\_at,98587\_at,95660\_at | high mobility group box 1,4-nitrophenylphosphatase domain and non-neuronal SNAP25-like protein homolog 1 (C. elegans),high mobility group nucleosomal binding domain 1,RIKEN cDNA 2410015M20 gene,glutathione peroxidase 4,chromobox homolog 1 (Drosophila HP1 beta),H2A histone family, member Z,minichromosome maintenance deficient 2 mitotin (S. cerevisiae),histone 1, H2bl,nucleosome assembly protein 1-like 1,RIKEN cDNA 0610025L15 gene |
| 7 | provirus integration | 1 | 1 | 298 | 6246 | 0.003 | 0 | 21 | 0.048 | 101105\_at | barrier to autointegration factor 1 |
| 5 | nucleoside metabolism | 3 | 15 | 600 | 11544 | 0.005 | 0.001 | 3.846 | 0.04 | 160107\_at,161897\_f\_at,95497\_at | hypoxanthine guanine phosphoribosyl transferase,phosphoribosyl pyrophosphate synthetase 1,RIKEN cDNA 1110005A05 gene |
| 5 | nucleotide metabolism | 24 | 95 | 600 | 11544 | 0.04 | 0.008 | 4.86 | 0 | 94034\_at,96289\_at,161897\_f\_at,93236\_s\_at,93237\_s\_at,95497\_at,160125\_at,160126\_at,92798\_at,92799\_g\_at,92800\_i\_at,93596\_i\_at,95656\_i\_at,96611\_at,99128\_at,93014\_at,92625\_at,92824\_at,103683\_at,93203\_f\_at,94372\_at,162417\_at,102001\_at,160107\_at | small fragment nuclease,stomatin (Epb7.2)-like 2,phosphoribosyl pyrophosphate synthetase 1,thymidylate synthase,thymidylate synthase,RIKEN cDNA 1110005A05 gene,DNA segment, Chr 11, ERATO Doi 497, expressed,mitogen-activated protein kinase kinase 1 interacting protein 1,ATP synthase, H+ transporting, mitochondrial F1 complex, gamma polypeptide 1,ATP synthase, H+ transporting, mitochondrial F1 complex, gamma polypeptide 1,ATP synthase, H+ transporting, mitochondrial F1 complex, gamma polypeptide 1,RIKEN cDNA 2410043G19 gene,ATPase, H+ transporting, V1 subunit F,NADH-ubiquinone oxidoreductase subunit B14.7,ATP synthase, H+ transporting, mitochondrial F1 complex, O subunit,ATP synthase, H+ transporting, mitochondrial F0 complex, subunit g,expressed in non-metastatic cells 2, protein,expressed in non-metastatic cells 6, protein,dihydroorotate dehydrogenase,RIKEN cDNA 3230402J05 gene,nudix (nucleoside diphosphate linked moiety X)-type motif 1,RIKEN cDNA 1500001M20 gene,ribonucleotide reductase M2,hypoxanthine guanine phosphoribosyl transferase |
| 6 | deoxyribonucleotide biosynthesis | 2 | 7 | 466 | 9498 | 0.004 | 0.001 | 5.797 | 0.043 | 93236\_s\_at,93237\_s\_at | thymidylate synthase,thymidylate synthase |
| 7 | deoxyribonucleoside monophosphate biosynthesis | 2 | 2 | 298 | 6246 | 0.007 | 0 | 20.969 | 0.002 | 93236\_s\_at,93237\_s\_at | thymidylate synthase,thymidylate synthase |
| 8 | pyrimidine deoxyribonucleoside monophosphate biosynthesis | 2 | 2 | 130 | 2164 | 0.015 | 0.001 | 16.717 | 0.004 | 93236\_s\_at,93237\_s\_at | thymidylate synthase,thymidylate synthase |
| 9 | dTMP biosynthesis | 2 | 2 | 66 | 911 | 0.03 | 0.002 | 13.773 | 0.005 | 93236\_s\_at,93237\_s\_at | thymidylate synthase,thymidylate synthase |
| 6 | nucleoside monophosphate biosynthesis | 3 | 15 | 466 | 9498 | 0.006 | 0.002 | 4.076 | 0.034 | 93236\_s\_at,93237\_s\_at,161897\_f\_at | thymidylate synthase,thymidylate synthase,phosphoribosyl pyrophosphate synthetase 1 |
| 7 | deoxyribonucleoside monophosphate biosynthesis | 2 | 2 | 298 | 6246 | 0.007 | 0 | 20.969 | 0.002 | 93236\_s\_at,93237\_s\_at | thymidylate synthase,thymidylate synthase |
| 8 | pyrimidine deoxyribonucleoside monophosphate biosynthesis | 2 | 2 | 130 | 2164 | 0.015 | 0.001 | 16.717 | 0.004 | 93236\_s\_at,93237\_s\_at | thymidylate synthase,thymidylate synthase |
| 9 | dTMP biosynthesis | 2 | 2 | 66 | 911 | 0.03 | 0.002 | 13.773 | 0.005 | 93236\_s\_at,93237\_s\_at | thymidylate synthase,thymidylate synthase |
| 6 | nucleoside triphosphate biosynthesis | 12 | 36 | 466 | 9498 | 0.026 | 0.004 | 6.794 | 0 | 160125\_at,160126\_at,92798\_at,92799\_g\_at,92800\_i\_at,93596\_i\_at,95656\_i\_at,96611\_at,99128\_at,93014\_at,92625\_at,92824\_at | DNA segment, Chr 11, ERATO Doi 497, expressed,mitogen-activated protein kinase kinase 1 interacting protein 1,ATP synthase, H+ transporting, mitochondrial F1 complex, gamma polypeptide 1,ATP synthase, H+ transporting, mitochondrial F1 complex, gamma polypeptide 1,ATP synthase, H+ transporting, mitochondrial F1 complex, gamma polypeptide 1,RIKEN cDNA 2410043G19 gene,ATPase, H+ transporting, V1 subunit F,NADH-ubiquinone oxidoreductase subunit B14.7,ATP synthase, H+ transporting, mitochondrial F1 complex, O subunit,ATP synthase, H+ transporting, mitochondrial F0 complex, subunit g,expressed in non-metastatic cells 2, protein,expressed in non-metastatic cells 6, protein |
| 7 | purine nucleoside triphosphate biosynthesis | 12 | 31 | 298 | 6246 | 0.04 | 0.005 | 8.119 | 0 | 160125\_at,160126\_at,92798\_at,92799\_g\_at,92800\_i\_at,93596\_i\_at,95656\_i\_at,96611\_at,99128\_at,93014\_at,92625\_at,92824\_at | DNA segment, Chr 11, ERATO Doi 497, expressed,mitogen-activated protein kinase kinase 1 interacting protein 1,ATP synthase, H+ transporting, mitochondrial F1 complex, gamma polypeptide 1,ATP synthase, H+ transporting, mitochondrial F1 complex, gamma polypeptide 1,ATP synthase, H+ transporting, mitochondrial F1 complex, gamma polypeptide 1,RIKEN cDNA 2410043G19 gene,ATPase, H+ transporting, V1 subunit F,NADH-ubiquinone oxidoreductase subunit B14.7,ATP synthase, H+ transporting, mitochondrial F1 complex, O subunit,ATP synthase, H+ transporting, mitochondrial F0 complex, subunit g,expressed in non-metastatic cells 2, protein,expressed in non-metastatic cells 6, protein |
| 8 | purine ribonucleoside triphosphate biosynthesis | 12 | 31 | 130 | 2164 | 0.092 | 0.014 | 6.442 | 0 | 160125\_at,160126\_at,92798\_at,92799\_g\_at,92800\_i\_at,93596\_i\_at,95656\_i\_at,96611\_at,99128\_at,93014\_at,92625\_at,92824\_at | DNA segment, Chr 11, ERATO Doi 497, expressed,mitogen-activated protein kinase kinase 1 interacting protein 1,ATP synthase, H+ transporting, mitochondrial F1 complex, gamma polypeptide 1,ATP synthase, H+ transporting, mitochondrial F1 complex, gamma polypeptide 1,ATP synthase, H+ transporting, mitochondrial F1 complex, gamma polypeptide 1,RIKEN cDNA 2410043G19 gene,ATPase, H+ transporting, V1 subunit F,NADH-ubiquinone oxidoreductase subunit B14.7,ATP synthase, H+ transporting, mitochondrial F1 complex, O subunit,ATP synthase, H+ transporting, mitochondrial F0 complex, subunit g,expressed in non-metastatic cells 2, protein,expressed in non-metastatic cells 6, protein |
| 8 | ATP synthesis coupled proton transport | 2 | 5 | 130 | 2164 | 0.015 | 0.002 | 6.658 | 0.032 | 93014\_at,95656\_i\_at | ATP synthase, H+ transporting, mitochondrial F0 complex, subunit g,ATPase, H+ transporting, V1 subunit F |
| 7 | ATP biosynthesis | 10 | 24 | 298 | 6246 | 0.034 | 0.004 | 8.74 | 0 | 160125\_at,160126\_at,92798\_at,92799\_g\_at,92800\_i\_at,93596\_i\_at,95656\_i\_at,96611\_at,99128\_at,93014\_at | DNA segment, Chr 11, ERATO Doi 497, expressed,mitogen-activated protein kinase kinase 1 interacting protein 1,ATP synthase, H+ transporting, mitochondrial F1 complex, gamma polypeptide 1,ATP synthase, H+ transporting, mitochondrial F1 complex, gamma polypeptide 1,ATP synthase, H+ transporting, mitochondrial F1 complex, gamma polypeptide 1,RIKEN cDNA 2410043G19 gene,ATPase, H+ transporting, V1 subunit F,NADH-ubiquinone oxidoreductase subunit B14.7,ATP synthase, H+ transporting, mitochondrial F1 complex, O subunit,ATP synthase, H+ transporting, mitochondrial F0 complex, subunit g |
| 8 | ATP synthesis coupled proton transport | 2 | 5 | 130 | 2164 | 0.015 | 0.002 | 6.658 | 0.032 | 93014\_at,95656\_i\_at | ATP synthase, H+ transporting, mitochondrial F0 complex, subunit g,ATPase, H+ transporting, V1 subunit F |
| 7 | ribonucleoside triphosphate biosynthesis | 12 | 31 | 298 | 6246 | 0.04 | 0.005 | 8.119 | 0 | 160125\_at,160126\_at,92798\_at,92799\_g\_at,92800\_i\_at,93596\_i\_at,95656\_i\_at,96611\_at,99128\_at,93014\_at,92625\_at,92824\_at | DNA segment, Chr 11, ERATO Doi 497, expressed,mitogen-activated protein kinase kinase 1 interacting protein 1,ATP synthase, H+ transporting, mitochondrial F1 complex, gamma polypeptide 1,ATP synthase, H+ transporting, mitochondrial F1 complex, gamma polypeptide 1,ATP synthase, H+ transporting, mitochondrial F1 complex, gamma polypeptide 1,RIKEN cDNA 2410043G19 gene,ATPase, H+ transporting, V1 subunit F,NADH-ubiquinone oxidoreductase subunit B14.7,ATP synthase, H+ transporting, mitochondrial F1 complex, O subunit,ATP synthase, H+ transporting, mitochondrial F0 complex, subunit g,expressed in non-metastatic cells 2, protein,expressed in non-metastatic cells 6, protein |
| 8 | purine ribonucleoside triphosphate biosynthesis | 12 | 31 | 130 | 2164 | 0.092 | 0.014 | 6.442 | 0 | 160125\_at,160126\_at,92798\_at,92799\_g\_at,92800\_i\_at,93596\_i\_at,95656\_i\_at,96611\_at,99128\_at,93014\_at,92625\_at,92824\_at | DNA segment, Chr 11, ERATO Doi 497, expressed,mitogen-activated protein kinase kinase 1 interacting protein 1,ATP synthase, H+ transporting, mitochondrial F1 complex, gamma polypeptide 1,ATP synthase, H+ transporting, mitochondrial F1 complex, gamma polypeptide 1,ATP synthase, H+ transporting, mitochondrial F1 complex, gamma polypeptide 1,RIKEN cDNA 2410043G19 gene,ATPase, H+ transporting, V1 subunit F,NADH-ubiquinone oxidoreductase subunit B14.7,ATP synthase, H+ transporting, mitochondrial F1 complex, O subunit,ATP synthase, H+ transporting, mitochondrial F0 complex, subunit g,expressed in non-metastatic cells 2, protein,expressed in non-metastatic cells 6, protein |
| 8 | ATP synthesis coupled proton transport | 2 | 5 | 130 | 2164 | 0.015 | 0.002 | 6.658 | 0.032 | 93014\_at,95656\_i\_at | ATP synthase, H+ transporting, mitochondrial F0 complex, subunit g,ATPase, H+ transporting, V1 subunit F |
| 7 | ATP biosynthesis | 10 | 24 | 298 | 6246 | 0.034 | 0.004 | 8.74 | 0 | 160125\_at,160126\_at,92798\_at,92799\_g\_at,92800\_i\_at,93596\_i\_at,95656\_i\_at,96611\_at,99128\_at,93014\_at | DNA segment, Chr 11, ERATO Doi 497, expressed,mitogen-activated protein kinase kinase 1 interacting protein 1,ATP synthase, H+ transporting, mitochondrial F1 complex, gamma polypeptide 1,ATP synthase, H+ transporting, mitochondrial F1 complex, gamma polypeptide 1,ATP synthase, H+ transporting, mitochondrial F1 complex, gamma polypeptide 1,RIKEN cDNA 2410043G19 gene,ATPase, H+ transporting, V1 subunit F,NADH-ubiquinone oxidoreductase subunit B14.7,ATP synthase, H+ transporting, mitochondrial F1 complex, O subunit,ATP synthase, H+ transporting, mitochondrial F0 complex, subunit g |
| 8 | ATP synthesis coupled proton transport | 2 | 5 | 130 | 2164 | 0.015 | 0.002 | 6.658 | 0.032 | 93014\_at,95656\_i\_at | ATP synthase, H+ transporting, mitochondrial F0 complex, subunit g,ATPase, H+ transporting, V1 subunit F |
| 6 | deoxyribonucleotide metabolism | 4 | 11 | 466 | 9498 | 0.009 | 0.001 | 7.397 | 0.001 | 93236\_s\_at,93237\_s\_at,94372\_at,102001\_at | thymidylate synthase,thymidylate synthase,nudix (nucleoside diphosphate linked moiety X)-type motif 1,ribonucleotide reductase M2 |
| 7 | deoxyribonucleoside monophosphate biosynthesis | 2 | 2 | 298 | 6246 | 0.007 | 0 | 20.969 | 0.002 | 93236\_s\_at,93237\_s\_at | thymidylate synthase,thymidylate synthase |
| 8 | pyrimidine deoxyribonucleoside monophosphate biosynthesis | 2 | 2 | 130 | 2164 | 0.015 | 0.001 | 16.717 | 0.004 | 93236\_s\_at,93237\_s\_at | thymidylate synthase,thymidylate synthase |
| 9 | dTMP biosynthesis | 2 | 2 | 66 | 911 | 0.03 | 0.002 | 13.773 | 0.005 | 93236\_s\_at,93237\_s\_at | thymidylate synthase,thymidylate synthase |
| 6 | deoxyribonucleotide biosynthesis | 2 | 7 | 466 | 9498 | 0.004 | 0.001 | 5.797 | 0.043 | 93236\_s\_at,93237\_s\_at | thymidylate synthase,thymidylate synthase |
| 7 | deoxyribonucleoside monophosphate biosynthesis | 2 | 2 | 298 | 6246 | 0.007 | 0 | 20.969 | 0.002 | 93236\_s\_at,93237\_s\_at | thymidylate synthase,thymidylate synthase |
| 8 | pyrimidine deoxyribonucleoside monophosphate biosynthesis | 2 | 2 | 130 | 2164 | 0.015 | 0.001 | 16.717 | 0.004 | 93236\_s\_at,93237\_s\_at | thymidylate synthase,thymidylate synthase |
| 9 | dTMP biosynthesis | 2 | 2 | 66 | 911 | 0.03 | 0.002 | 13.773 | 0.005 | 93236\_s\_at,93237\_s\_at | thymidylate synthase,thymidylate synthase |
| 5 | nucleotide biosynthesis | 17 | 74 | 600 | 11544 | 0.028 | 0.006 | 4.42 | 0 | 161897\_f\_at,93236\_s\_at,93237\_s\_at,95497\_at,160125\_at,160126\_at,92798\_at,92799\_g\_at,92800\_i\_at,93596\_i\_at,95656\_i\_at,96611\_at,99128\_at,93014\_at,92625\_at,92824\_at,103683\_at | phosphoribosyl pyrophosphate synthetase 1,thymidylate synthase,thymidylate synthase,RIKEN cDNA 1110005A05 gene,DNA segment, Chr 11, ERATO Doi 497, expressed,mitogen-activated protein kinase kinase 1 interacting protein 1,ATP synthase, H+ transporting, mitochondrial F1 complex, gamma polypeptide 1,ATP synthase, H+ transporting, mitochondrial F1 complex, gamma polypeptide 1,ATP synthase, H+ transporting, mitochondrial F1 complex, gamma polypeptide 1,RIKEN cDNA 2410043G19 gene,ATPase, H+ transporting, V1 subunit F,NADH-ubiquinone oxidoreductase subunit B14.7,ATP synthase, H+ transporting, mitochondrial F1 complex, O subunit,ATP synthase, H+ transporting, mitochondrial F0 complex, subunit g,expressed in non-metastatic cells 2, protein,expressed in non-metastatic cells 6, protein,dihydroorotate dehydrogenase |
| 6 | deoxyribonucleotide biosynthesis | 2 | 7 | 466 | 9498 | 0.004 | 0.001 | 5.797 | 0.043 | 93236\_s\_at,93237\_s\_at | thymidylate synthase,thymidylate synthase |
| 7 | deoxyribonucleoside monophosphate biosynthesis | 2 | 2 | 298 | 6246 | 0.007 | 0 | 20.969 | 0.002 | 93236\_s\_at,93237\_s\_at | thymidylate synthase,thymidylate synthase |
| 8 | pyrimidine deoxyribonucleoside monophosphate biosynthesis | 2 | 2 | 130 | 2164 | 0.015 | 0.001 | 16.717 | 0.004 | 93236\_s\_at,93237\_s\_at | thymidylate synthase,thymidylate synthase |
| 9 | dTMP biosynthesis | 2 | 2 | 66 | 911 | 0.03 | 0.002 | 13.773 | 0.005 | 93236\_s\_at,93237\_s\_at | thymidylate synthase,thymidylate synthase |
| 6 | nucleoside monophosphate biosynthesis | 3 | 15 | 466 | 9498 | 0.006 | 0.002 | 4.076 | 0.034 | 93236\_s\_at,93237\_s\_at,161897\_f\_at | thymidylate synthase,thymidylate synthase,phosphoribosyl pyrophosphate synthetase 1 |
| 7 | deoxyribonucleoside monophosphate biosynthesis | 2 | 2 | 298 | 6246 | 0.007 | 0 | 20.969 | 0.002 | 93236\_s\_at,93237\_s\_at | thymidylate synthase,thymidylate synthase |
| 8 | pyrimidine deoxyribonucleoside monophosphate biosynthesis | 2 | 2 | 130 | 2164 | 0.015 | 0.001 | 16.717 | 0.004 | 93236\_s\_at,93237\_s\_at | thymidylate synthase,thymidylate synthase |
| 9 | dTMP biosynthesis | 2 | 2 | 66 | 911 | 0.03 | 0.002 | 13.773 | 0.005 | 93236\_s\_at,93237\_s\_at | thymidylate synthase,thymidylate synthase |
| 6 | nucleoside triphosphate biosynthesis | 12 | 36 | 466 | 9498 | 0.026 | 0.004 | 6.794 | 0 | 160125\_at,160126\_at,92798\_at,92799\_g\_at,92800\_i\_at,93596\_i\_at,95656\_i\_at,96611\_at,99128\_at,93014\_at,92625\_at,92824\_at | DNA segment, Chr 11, ERATO Doi 497, expressed,mitogen-activated protein kinase kinase 1 interacting protein 1,ATP synthase, H+ transporting, mitochondrial F1 complex, gamma polypeptide 1,ATP synthase, H+ transporting, mitochondrial F1 complex, gamma polypeptide 1,ATP synthase, H+ transporting, mitochondrial F1 complex, gamma polypeptide 1,RIKEN cDNA 2410043G19 gene,ATPase, H+ transporting, V1 subunit F,NADH-ubiquinone oxidoreductase subunit B14.7,ATP synthase, H+ transporting, mitochondrial F1 complex, O subunit,ATP synthase, H+ transporting, mitochondrial F0 complex, subunit g,expressed in non-metastatic cells 2, protein,expressed in non-metastatic cells 6, protein |
| 7 | purine nucleoside triphosphate biosynthesis | 12 | 31 | 298 | 6246 | 0.04 | 0.005 | 8.119 | 0 | 160125\_at,160126\_at,92798\_at,92799\_g\_at,92800\_i\_at,93596\_i\_at,95656\_i\_at,96611\_at,99128\_at,93014\_at,92625\_at,92824\_at | DNA segment, Chr 11, ERATO Doi 497, expressed,mitogen-activated protein kinase kinase 1 interacting protein 1,ATP synthase, H+ transporting, mitochondrial F1 complex, gamma polypeptide 1,ATP synthase, H+ transporting, mitochondrial F1 complex, gamma polypeptide 1,ATP synthase, H+ transporting, mitochondrial F1 complex, gamma polypeptide 1,RIKEN cDNA 2410043G19 gene,ATPase, H+ transporting, V1 subunit F,NADH-ubiquinone oxidoreductase subunit B14.7,ATP synthase, H+ transporting, mitochondrial F1 complex, O subunit,ATP synthase, H+ transporting, mitochondrial F0 complex, subunit g,expressed in non-metastatic cells 2, protein,expressed in non-metastatic cells 6, protein |
| 8 | purine ribonucleoside triphosphate biosynthesis | 12 | 31 | 130 | 2164 | 0.092 | 0.014 | 6.442 | 0 | 160125\_at,160126\_at,92798\_at,92799\_g\_at,92800\_i\_at,93596\_i\_at,95656\_i\_at,96611\_at,99128\_at,93014\_at,92625\_at,92824\_at | DNA segment, Chr 11, ERATO Doi 497, expressed,mitogen-activated protein kinase kinase 1 interacting protein 1,ATP synthase, H+ transporting, mitochondrial F1 complex, gamma polypeptide 1,ATP synthase, H+ transporting, mitochondrial F1 complex, gamma polypeptide 1,ATP synthase, H+ transporting, mitochondrial F1 complex, gamma polypeptide 1,RIKEN cDNA 2410043G19 gene,ATPase, H+ transporting, V1 subunit F,NADH-ubiquinone oxidoreductase subunit B14.7,ATP synthase, H+ transporting, mitochondrial F1 complex, O subunit,ATP synthase, H+ transporting, mitochondrial F0 complex, subunit g,expressed in non-metastatic cells 2, protein,expressed in non-metastatic cells 6, protein |
| 8 | ATP synthesis coupled proton transport | 2 | 5 | 130 | 2164 | 0.015 | 0.002 | 6.658 | 0.032 | 93014\_at,95656\_i\_at | ATP synthase, H+ transporting, mitochondrial F0 complex, subunit g,ATPase, H+ transporting, V1 subunit F |
| 7 | ATP biosynthesis | 10 | 24 | 298 | 6246 | 0.034 | 0.004 | 8.74 | 0 | 160125\_at,160126\_at,92798\_at,92799\_g\_at,92800\_i\_at,93596\_i\_at,95656\_i\_at,96611\_at,99128\_at,93014\_at | DNA segment, Chr 11, ERATO Doi 497, expressed,mitogen-activated protein kinase kinase 1 interacting protein 1,ATP synthase, H+ transporting, mitochondrial F1 complex, gamma polypeptide 1,ATP synthase, H+ transporting, mitochondrial F1 complex, gamma polypeptide 1,ATP synthase, H+ transporting, mitochondrial F1 complex, gamma polypeptide 1,RIKEN cDNA 2410043G19 gene,ATPase, H+ transporting, V1 subunit F,NADH-ubiquinone oxidoreductase subunit B14.7,ATP synthase, H+ transporting, mitochondrial F1 complex, O subunit,ATP synthase, H+ transporting, mitochondrial F0 complex, subunit g |
| 8 | ATP synthesis coupled proton transport | 2 | 5 | 130 | 2164 | 0.015 | 0.002 | 6.658 | 0.032 | 93014\_at,95656\_i\_at | ATP synthase, H+ transporting, mitochondrial F0 complex, subunit g,ATPase, H+ transporting, V1 subunit F |
| 7 | ribonucleoside triphosphate biosynthesis | 12 | 31 | 298 | 6246 | 0.04 | 0.005 | 8.119 | 0 | 160125\_at,160126\_at,92798\_at,92799\_g\_at,92800\_i\_at,93596\_i\_at,95656\_i\_at,96611\_at,99128\_at,93014\_at,92625\_at,92824\_at | DNA segment, Chr 11, ERATO Doi 497, expressed,mitogen-activated protein kinase kinase 1 interacting protein 1,ATP synthase, H+ transporting, mitochondrial F1 complex, gamma polypeptide 1,ATP synthase, H+ transporting, mitochondrial F1 complex, gamma polypeptide 1,ATP synthase, H+ transporting, mitochondrial F1 complex, gamma polypeptide 1,RIKEN cDNA 2410043G19 gene,ATPase, H+ transporting, V1 subunit F,NADH-ubiquinone oxidoreductase subunit B14.7,ATP synthase, H+ transporting, mitochondrial F1 complex, O subunit,ATP synthase, H+ transporting, mitochondrial F0 complex, subunit g,expressed in non-metastatic cells 2, protein,expressed in non-metastatic cells 6, protein |
| 8 | purine ribonucleoside triphosphate biosynthesis | 12 | 31 | 130 | 2164 | 0.092 | 0.014 | 6.442 | 0 | 160125\_at,160126\_at,92798\_at,92799\_g\_at,92800\_i\_at,93596\_i\_at,95656\_i\_at,96611\_at,99128\_at,93014\_at,92625\_at,92824\_at | DNA segment, Chr 11, ERATO Doi 497, expressed,mitogen-activated protein kinase kinase 1 interacting protein 1,ATP synthase, H+ transporting, mitochondrial F1 complex, gamma polypeptide 1,ATP synthase, H+ transporting, mitochondrial F1 complex, gamma polypeptide 1,ATP synthase, H+ transporting, mitochondrial F1 complex, gamma polypeptide 1,RIKEN cDNA 2410043G19 gene,ATPase, H+ transporting, V1 subunit F,NADH-ubiquinone oxidoreductase subunit B14.7,ATP synthase, H+ transporting, mitochondrial F1 complex, O subunit,ATP synthase, H+ transporting, mitochondrial F0 complex, subunit g,expressed in non-metastatic cells 2, protein,expressed in non-metastatic cells 6, protein |
| 8 | ATP synthesis coupled proton transport | 2 | 5 | 130 | 2164 | 0.015 | 0.002 | 6.658 | 0.032 | 93014\_at,95656\_i\_at | ATP synthase, H+ transporting, mitochondrial F0 complex, subunit g,ATPase, H+ transporting, V1 subunit F |
| 7 | ATP biosynthesis | 10 | 24 | 298 | 6246 | 0.034 | 0.004 | 8.74 | 0 | 160125\_at,160126\_at,92798\_at,92799\_g\_at,92800\_i\_at,93596\_i\_at,95656\_i\_at,96611\_at,99128\_at,93014\_at | DNA segment, Chr 11, ERATO Doi 497, expressed,mitogen-activated protein kinase kinase 1 interacting protein 1,ATP synthase, H+ transporting, mitochondrial F1 complex, gamma polypeptide 1,ATP synthase, H+ transporting, mitochondrial F1 complex, gamma polypeptide 1,ATP synthase, H+ transporting, mitochondrial F1 complex, gamma polypeptide 1,RIKEN cDNA 2410043G19 gene,ATPase, H+ transporting, V1 subunit F,NADH-ubiquinone oxidoreductase subunit B14.7,ATP synthase, H+ transporting, mitochondrial F1 complex, O subunit,ATP synthase, H+ transporting, mitochondrial F0 complex, subunit g |
| 8 | ATP synthesis coupled proton transport | 2 | 5 | 130 | 2164 | 0.015 | 0.002 | 6.658 | 0.032 | 93014\_at,95656\_i\_at | ATP synthase, H+ transporting, mitochondrial F0 complex, subunit g,ATPase, H+ transporting, V1 subunit F |
| 5 | nucleotide catabolism | 3 | 10 | 600 | 11544 | 0.005 | 0.001 | 5.747 | 0.013 | 93203\_f\_at,94372\_at,162417\_at | RIKEN cDNA 3230402J05 gene,nudix (nucleoside diphosphate linked moiety X)-type motif 1,RIKEN cDNA 1500001M20 gene |
| 5 | RNA metabolism | 15 | 132 | 600 | 11544 | 0.025 | 0.011 | 2.187 | 0.004 | 160723\_at,93117\_at,96696\_at,99151\_at,160503\_at,160531\_at,100577\_at,102409\_at,93008\_at,93999\_at,95049\_at,96029\_at,97200\_f\_at,99182\_at,96289\_at | RIKEN cDNA 1500001M20 gene,heterogeneous nuclear ribonucleoprotein A2/B1,NA,RIKEN cDNA 2610002K22 gene,fibrillarin,B-cell receptor-associated protein 37,small nuclear ribonucleoprotein D1,RIKEN cDNA 2010003I05 gene,U6 snRNA-associated SM-like protein 4,small nuclear ribonucleoprotein polypeptide G,small nuclear ribonucleoprotein D2,splicing factor 3a, subunit 3, 60kDa,small nuclear ribonucleoprotein E,RIKEN cDNA 2610511E03 gene,stomatin (Epb7.2)-like 2 |
| 6 | RNA processing | 14 | 126 | 466 | 9498 | 0.03 | 0.013 | 2.264 | 0.003 | 160723\_at,93117\_at,96696\_at,99151\_at,160503\_at,160531\_at,100577\_at,102409\_at,93008\_at,93999\_at,95049\_at,96029\_at,97200\_f\_at,99182\_at | RIKEN cDNA 1500001M20 gene,heterogeneous nuclear ribonucleoprotein A2/B1,NA,RIKEN cDNA 2610002K22 gene,fibrillarin,B-cell receptor-associated protein 37,small nuclear ribonucleoprotein D1,RIKEN cDNA 2010003I05 gene,U6 snRNA-associated SM-like protein 4,small nuclear ribonucleoprotein polypeptide G,small nuclear ribonucleoprotein D2,splicing factor 3a, subunit 3, 60kDa,small nuclear ribonucleoprotein E,RIKEN cDNA 2610511E03 gene |
| 6 | tRNA metabolism | 2 | 7 | 466 | 9498 | 0.004 | 0.001 | 5.797 | 0.043 | 96289\_at,99182\_at | stomatin (Epb7.2)-like 2,RIKEN cDNA 2610511E03 gene |
| 8 | regulation of transcription from Pol I promoter | 2 | 2 | 130 | 2164 | 0.015 | 0.001 | 16.717 | 0.004 | 103654\_at,93551\_at | nucleosome binding protein 1,RIKEN cDNA 2510029B14 gene |
| 8 | regulation of transcription from Pol I promoter | 2 | 2 | 130 | 2164 | 0.015 | 0.001 | 16.717 | 0.004 | 103654\_at,93551\_at | nucleosome binding protein 1,RIKEN cDNA 2510029B14 gene |
| 4 | oxygen and reactive oxygen species metabolism | 6 | 34 | 695 | 13100 | 0.009 | 0.003 | 3.319 | 0.008 | 100331\_g\_at,97819\_at,99583\_at,97758\_at,100059\_at,97013\_f\_at | thioredoxin peroxidase, pseudogene 1,glutathione S-transferase omega 1,glutathione S-transferase, pi 2,peroxiredoxin 1,cytochrome b-245, alpha polypeptide,cytochrome b-245, alpha polypeptide |
| 7 | mitochondrial electron transport, NADH to ubiquinone | 5 | 6 | 298 | 6246 | 0.017 | 0.001 | 17.479 | 0 | 100079\_at,94062\_at,96267\_at,96899\_at,96902\_at | NADH dehydrogenase (ubiquinone) 1 beta subcomplex, 9,NADH dehydrogenase (ubiquinone) flavoprotein 2,NADH dehydrogenase (ubiquinone) flavoprotein 1,NADH dehydrogenase (ubiquinone) Fe-S protein 3,RIKEN cDNA 2900091E11 gene |
| 6 | ATP synthesis coupled electron transport (sensu Eukarya) | 5 | 7 | 466 | 9498 | 0.011 | 0.001 | 14.5 | 0 | 100079\_at,94062\_at,96267\_at,96899\_at,96902\_at | NADH dehydrogenase (ubiquinone) 1 beta subcomplex, 9,NADH dehydrogenase (ubiquinone) flavoprotein 2,NADH dehydrogenase (ubiquinone) flavoprotein 1,NADH dehydrogenase (ubiquinone) Fe-S protein 3,RIKEN cDNA 2900091E11 gene |
| 7 | mitochondrial electron transport, NADH to ubiquinone | 5 | 6 | 298 | 6246 | 0.017 | 0.001 | 17.479 | 0 | 100079\_at,94062\_at,96267\_at,96899\_at,96902\_at | NADH dehydrogenase (ubiquinone) 1 beta subcomplex, 9,NADH dehydrogenase (ubiquinone) flavoprotein 2,NADH dehydrogenase (ubiquinone) flavoprotein 1,NADH dehydrogenase (ubiquinone) Fe-S protein 3,RIKEN cDNA 2900091E11 gene |
| 5 | ATP synthesis coupled electron transport | 5 | 7 | 600 | 11544 | 0.008 | 0.001 | 13.656 | 0 | 100079\_at,94062\_at,96267\_at,96899\_at,96902\_at | NADH dehydrogenase (ubiquinone) 1 beta subcomplex, 9,NADH dehydrogenase (ubiquinone) flavoprotein 2,NADH dehydrogenase (ubiquinone) flavoprotein 1,NADH dehydrogenase (ubiquinone) Fe-S protein 3,RIKEN cDNA 2900091E11 gene |
| 6 | ATP synthesis coupled electron transport (sensu Eukarya) | 5 | 7 | 466 | 9498 | 0.011 | 0.001 | 14.5 | 0 | 100079\_at,94062\_at,96267\_at,96899\_at,96902\_at | NADH dehydrogenase (ubiquinone) 1 beta subcomplex, 9,NADH dehydrogenase (ubiquinone) flavoprotein 2,NADH dehydrogenase (ubiquinone) flavoprotein 1,NADH dehydrogenase (ubiquinone) Fe-S protein 3,RIKEN cDNA 2900091E11 gene |
| 7 | mitochondrial electron transport, NADH to ubiquinone | 5 | 6 | 298 | 6246 | 0.017 | 0.001 | 17.479 | 0 | 100079\_at,94062\_at,96267\_at,96899\_at,96902\_at | NADH dehydrogenase (ubiquinone) 1 beta subcomplex, 9,NADH dehydrogenase (ubiquinone) flavoprotein 2,NADH dehydrogenase (ubiquinone) flavoprotein 1,NADH dehydrogenase (ubiquinone) Fe-S protein 3,RIKEN cDNA 2900091E11 gene |
| 4 | protein metabolism | 94 | 1458 | 695 | 13100 | 0.135 | 0.111 | 1.215 | 0.025 | 92636\_f\_at,94014\_at,94210\_at,95441\_at,96668\_at,96670\_at,96734\_at,96849\_at,96947\_at,97477\_at,97478\_at,98959\_at,99156\_at,101061\_at,101254\_at,93970\_at,94323\_at,101097\_at,101680\_at,102019\_at,160431\_at,92578\_at,92646\_at,93062\_at,93579\_at,94252\_at,94494\_at,94870\_f\_at,94912\_at,95067\_at,95498\_at,96291\_f\_at,96292\_r\_at,96293\_at,97342\_at,97751\_f\_at,97824\_at,97884\_at,98120\_at,98524\_f\_at,98904\_at,99594\_at,95677\_at,103881\_at,92565\_at,93734\_i\_at,93735\_f\_at,94025\_at,95448\_at,100543\_s\_at,101562\_at,104541\_at,92874\_f\_at,95561\_at,96093\_at,96733\_at,99655\_at,93519\_s\_at,100512\_at,100733\_at,101486\_at,101558\_s\_at,101992\_at,102791\_at,92547\_at,93085\_at,93988\_at,94263\_f\_at,94841\_at,96892\_at,96952\_at,97459\_at,98557\_f\_at,98975\_at,100089\_at,101207\_at,160416\_at,160456\_at,92829\_at,98153\_at,99546\_at,93101\_s\_at,97460\_at,104423\_at,96052\_at,100128\_at,104080\_at,160538\_at,160659\_at,98595\_at,98934\_at,99522\_at,99544\_at,101440\_at | SEC61, gamma subunit (S. cerevisiae),RIKEN cDNA 2510048O06 gene,translocase of inner mitochondrial membrane 9 homolog (yeast),translocase of inner mitochondrial membrane 23 homolog (yeast),translocator of inner mitochondrial membrane b,RIKEN cDNA 0610025I19 gene,synaptojanin 2 binding protein,translocase of inner mitochondrial membrane 8 homolog a (yeast),RIKEN cDNA 0610009I16 gene,translocase of inner mitochondrial membrane 8 homolog b (yeast),golgi phosphoprotein 2,RIKEN cDNA D930048L02 gene,RIKEN cDNA 2700099C19 gene,signal sequence receptor, beta,RAN, member RAS oncogene family,importin 7,RIKEN cDNA D630012G11 gene,DNA segment, Chr 9, Wayne State University 149,ribosomal protein L27a,mitochondrial ribosomal protein L13,mitochondrial ribosomal protein L12,small inducible cytokine subfamily E, member 1,mitochondrial ribosomal protein L23,mitochondrial ribosomal protein L39,RIKEN cDNA 5830427H10 gene,eukaryotic translation initiation factor 2, subunit 3, structural gene X-linked,phenylalanine-tRNA synthetase-like,RIKEN cDNA 2310075M17 gene,mitochondrial ribosomal protein S21,mitochondrial ribosomal protein L2,mitochondrial ribosomal protein S15,NA,NA,RIKEN cDNA 2410015N17 gene,mitochondrial ribosomal protein S14,NA,DNA segment, Chr 11, ERATO Doi 175, expressed,mitochondrial ribosomal protein S11,mitochondrial ribosomal protein L27,RIKEN cDNA 2210039B01 gene,mitochondrial ribosomal protein L35,mitochondrial ribosomal protein L51,U5 snRNP-specific protein (Prp8-binding),inorganic pyrophosphatase 2,RIKEN cDNA 1110005A23 gene,proteasome (prosome, macropain) 26S subunit, ATPase 3,proteasome (prosome, macropain) 26S subunit, ATPase 3,proteasome (prosome, macropain) subunit, beta type 3,proteasome (prosome, macropain) 26S subunit, ATPase 2,bromodomain containing 7,heat shock protein 4,proteinase 3,COP9 (constitutive photomorphogenic) homolog, subunit 7a (Arabidopsis thaliana),RIKEN cDNA 1700013H19 gene,open reading frame 11,RAP1, GTP-GDP dissociation stimulator 1,signal peptidase complex,neural precursor cell expressed, developmentally down-regulated gene 8,ubiquitin carboxyl-terminal esterase L5,proteasome (prosome, macropain) subunit, alpha type 2,proteasome (prosome, macropain) subunit, beta type 10,proteasome (prosome, macropain) subunit, beta type 5,proteasome (prosome, macropain) subunit, beta type 6,proteosome (prosome, macropain) subunit, beta type 8 (large multifunctional protease 7),huntingtin interacting protein 2,proteosome (prosome, macropain) subunit, beta type 9 (large multifunctional protease 2),proteasome (prosome, macropain) subunit, alpha type 7,proteasome (prosome, macropain) subunit, beta type 7,proteasome (prosome, macropain) subunit, alpha type 5,proteasome (prosome, macropain) subunit, alpha type 1,proteasome (prosome, macropain) subunit, alpha type 6,proteasome (prosome, macropain) subunit, alpha type 4,proteasome (prosome, macropain) subunit, beta type 4,RIKEN cDNA 2410008G02 gene,peptidylprolyl isomerase C,peptidylprolyl isomerase A,FK506 binding protein 3,RIKEN cDNA 1100001J08 gene,heat shock protein 1 (chaperonin 10),chaperonin subunit 3 (gamma),FK506 binding protein 2,neural precursor cell expressed, developmentally down-regulted gene 4,ubiquitin-conjugating enzyme E2R 2,RIKEN cDNA 2810047L02 gene,acid phosphatase 1, soluble,cell division cycle 2 homolog A (S. pombe),PDGFA associated protein 1,cyclin-dependent kinase 4,RIKEN cDNA 2310057G13 gene,interleukin-1 receptor-associated kinase 1,RIKEN cDNA 0610007P06 gene,germ cell-specific gene 2,deoxyguanosine kinase,RIKEN cDNA 2900006F19 gene |
| 5 | protein folding | 7 | 56 | 600 | 11544 | 0.012 | 0.005 | 2.406 | 0.025 | 100089\_at,101207\_at,160416\_at,160456\_at,92829\_at,98153\_at,99546\_at | peptidylprolyl isomerase C,peptidylprolyl isomerase A,FK506 binding protein 3,RIKEN cDNA 1100001J08 gene,heat shock protein 1 (chaperonin 10),chaperonin subunit 3 (gamma),FK506 binding protein 2 |
| 7 | retinal metabolism | 1 | 1 | 298 | 6246 | 0.003 | 0 | 21 | 0.048 | 96678\_at | dehydrogenase/reductase (SDR family) member 4 |
| 6 | perception of sound | 3 | 17 | 466 | 9498 | 0.006 | 0.002 | 3.598 | 0.048 | 100079\_at,94210\_at,97449\_at | NADH dehydrogenase (ubiquinone) 1 beta subcomplex, 9,translocase of inner mitochondrial membrane 9 homolog (yeast),NA |
| 7 | hearing | 3 | 17 | 298 | 6246 | 0.01 | 0.003 | 3.702 | 0.044 | 100079\_at,94210\_at,97449\_at | NADH dehydrogenase (ubiquinone) 1 beta subcomplex, 9,translocase of inner mitochondrial membrane 9 homolog (yeast),NA |
| 6 | perception of sound | 3 | 17 | 466 | 9498 | 0.006 | 0.002 | 3.598 | 0.048 | 100079\_at,94210\_at,97449\_at | NADH dehydrogenase (ubiquinone) 1 beta subcomplex, 9,translocase of inner mitochondrial membrane 9 homolog (yeast),NA |
| 7 | hearing | 3 | 17 | 298 | 6246 | 0.01 | 0.003 | 3.702 | 0.044 | 100079\_at,94210\_at,97449\_at | NADH dehydrogenase (ubiquinone) 1 beta subcomplex, 9,translocase of inner mitochondrial membrane 9 homolog (yeast),NA |

  
