# Supplementary material for: Molecular Signatures of Proliferation and Quiescence in Hematopoietic Stem Cells
Source: PLoS Biol. 2004 Sep 28;2(10):e301. doi: 10.1371/journal.pbio.0020301 (PMC520599; doi:10.1371/journal.pbio.0020301)
Supplement: Table S44 — (244 KB HTML). [file pbio.0020301.st044.html]

   Full Up in Fetal Liver   

# Full Up in Fetal Liver

|  |  |  |  |  |  |  |  |  |  |  |
| --- | --- | --- | --- | --- | --- | --- | --- | --- | --- | --- |
| GOLevel | GOTerm | ProbeCount | ArrayCount | ListGOLevelCount | ArrayGoLevelCount | ListFq | ArrayFq | FoldChange | H-Pvalue | ProbeIds |
| 0 | Gene\_Ontology | 684 | NA | 684 | 0 | 1 | NA | NA | NA | 96147\_at,103693\_at,103699\_i\_at,160726\_at,160727\_at,93619\_at,99535\_at,94420\_f\_at,97925\_at,100134\_at,100327\_at,100482\_at,101392\_at,102356\_at,103816\_at,104083\_at,104206\_at,104407\_at,104694\_at,160430\_at,160469\_at,92558\_at,92559\_at,92877\_at,93063\_at,94454\_at,95511\_at,96752\_at,96886\_at,97519\_at,98849\_at,97312\_at,103518\_at,103736\_at,101217\_at,93705\_at,94657\_at,98011\_at,98324\_at,100122\_at,100514\_at,101465\_at,102994\_at,104389\_at,160495\_at,160941\_at,161609\_at,162206\_f\_at,92484\_at,93016\_at,93315\_at,94331\_at,95023\_at,96252\_at,96852\_at,97844\_at,98427\_s\_at,98504\_at,98508\_s\_at,99100\_at,99475\_at,101144\_at,102255\_at,102658\_at,102663\_at,161689\_f\_at,93874\_s\_at,93914\_at,94928\_at,98254\_f\_at,101457\_at,160252\_at,96488\_at,96489\_at,96764\_at,92216\_at,102983\_at,102984\_g\_at,102032\_at,99532\_at,160440\_at,102224\_at,104417\_at,102787\_at,102794\_at,103362\_at,103658\_r\_at,104445\_at,161788\_f\_at,92268\_at,93193\_at,93459\_s\_at,94192\_at,95474\_at,95477\_at,96912\_s\_at,99416\_at,102321\_at,96532\_at,103596\_at,97375\_at,99364\_at,99366\_at,101186\_at,100024\_at,102750\_at,102957\_at,104256\_at,104257\_g\_at,160296\_at,160490\_at,160632\_at,160911\_at,160965\_at,92302\_at,93017\_at,93714\_f\_at,94109\_at,94556\_at,96513\_at,96736\_at,98946\_at,99467\_at,94264\_at,100951\_at,94060\_at,161666\_f\_at,97740\_at,94448\_at,99070\_at,100561\_at,100635\_at,101030\_at,103062\_at,103901\_at,104114\_at,104117\_at,104179\_at,104725\_at,160082\_s\_at,160757\_at,160993\_at,92185\_at,92805\_s\_at,94031\_at,94106\_at,94319\_at,94505\_at,95418\_at,96530\_at,97058\_f\_at,97224\_at,97319\_at,98927\_at,99032\_at,92854\_at,96238\_at,95287\_at,102823\_at,102824\_g\_at,97949\_at,100988\_at,102064\_at,102734\_at,102905\_at,103812\_at,104100\_at,160920\_at,161067\_at,161980\_f\_at,93064\_at,93093\_at,95412\_at,96255\_at,97285\_f\_at,97890\_at,98436\_s\_at,98868\_at,92925\_at,95102\_at,98945\_at,97825\_at,104761\_at,160301\_at,160464\_s\_at,161610\_at,92653\_at,93550\_at,94752\_s\_at,94809\_at,96088\_at,96596\_at,97118\_at,97498\_at,97973\_at,103531\_f\_at,100130\_at,101475\_at,102364\_at,102920\_at,103990\_at,104477\_at,104576\_at,160829\_at,160901\_at,161551\_f\_at,92926\_at,93104\_at,93666\_at,95348\_at,95617\_at,95618\_at,96771\_at,96810\_at,97484\_at,98110\_at,98756\_at,101998\_at,102752\_at,97426\_at,103899\_at,92778\_i\_at,92780\_f\_at,96633\_s\_at,92263\_at,92270\_at,104263\_at,100606\_at,101118\_at,103959\_at,160676\_at,94976\_at,96299\_at,101578\_f\_at,92542\_at,94270\_at,98968\_at,160065\_s\_at,95489\_at,98127\_at,96278\_at,99013\_f\_at,100342\_i\_at,160287\_at,160288\_at,160679\_at,161615\_f\_at,93729\_at,94835\_f\_at,95119\_at,98461\_at,98882\_s\_at,98884\_r\_at,160373\_i\_at,96945\_at,96779\_f\_at,97409\_at,101848\_g\_at,160399\_r\_at,93020\_at,162138\_s\_at,104376\_at,101015\_s\_at,92770\_at,102781\_at,103416\_at,104598\_at,160127\_at,160359\_at,94820\_r\_at,94881\_at,94882\_at,95471\_at,95805\_at,96728\_at,97504\_at,98067\_at,98478\_at,99187\_f\_at,99188\_at,99529\_f\_at,100307\_at,101930\_at,103500\_at,160603\_at,95613\_at,104735\_at,95917\_at,92638\_at,94483\_at,96600\_at,102292\_at,100088\_at,95033\_at,104149\_at,160511\_at,100567\_at,100944\_at,101079\_at,101568\_at,101787\_f\_at,103035\_at,103065\_at,103376\_s\_at,103427\_at,103739\_at,103913\_at,104019\_at,104461\_at,104534\_at,104719\_at,160683\_at,160684\_at,161696\_f\_at,161990\_f\_at,92392\_at,92847\_s\_at,93316\_at,93320\_at,93414\_at,93626\_at,95377\_at,95444\_at,95586\_at,95695\_at,96019\_at,96076\_at,96079\_at,96669\_at,96725\_at,96876\_at,97181\_f\_at,97458\_at,97812\_at,99500\_at,92648\_at,160124\_r\_at,160202\_at,94043\_at,96951\_at,100499\_at,101934\_at,160149\_at,161026\_s\_at,99350\_at,99354\_s\_at,99358\_at,101023\_f\_at,104453\_at,92256\_at,103686\_at,94254\_at,94255\_g\_at,94256\_at,94464\_at,94465\_g\_at,95655\_at,162041\_f\_at,93471\_at,102854\_s\_at,103935\_at,96186\_at,96534\_at,98114\_at,92531\_at,160795\_at,98926\_at,103258\_at,104522\_at,97551\_at,161184\_f\_at,160151\_i\_at,94004\_at,100992\_at,102381\_at,102644\_at,103086\_at,103328\_at,160483\_at,160857\_at,94473\_at,102940\_at,92248\_at,92249\_g\_at,160099\_at,160834\_at,100522\_s\_at,100523\_r\_at,100533\_s\_at,101515\_at,160526\_s\_at,92888\_s\_at,102094\_f\_at,160199\_at,160200\_at,93267\_at,93520\_at,96777\_at,92992\_i\_at,92993\_r\_at,103499\_at,93083\_at,94383\_at,98018\_at,100068\_at,102768\_i\_at,160092\_at,161401\_f\_at,93241\_r\_at,94815\_at,95135\_at,95440\_at,97451\_at,98931\_at,160338\_at,101990\_at,161946\_r\_at,94439\_at,97833\_at,97834\_g\_at,99045\_at,AFFX-MUR\_b2\_at,104337\_f\_at,98984\_f\_at,94415\_at,93177\_at,160084\_at,101490\_at,102302\_at,99184\_at,96657\_at,92586\_at,102360\_at,101000\_at,101001\_at,102313\_at,160335\_at,103471\_at,99849\_at,97829\_at,101681\_f\_at,103674\_f\_at,103994\_at,104049\_at,104144\_at,160111\_at,160112\_at,160130\_at,160976\_at,160977\_at,161342\_r\_at,92855\_at,93975\_at,94499\_at,95070\_at,95737\_at,96325\_at,100136\_at,101590\_at,95681\_f\_at,96628\_at,160137\_at,94433\_at,160579\_at,94818\_at,93852\_at,97798\_at,102322\_at,94872\_at,99133\_at,93165\_at,100622\_at,95062\_at,100905\_at,101963\_at,103222\_at,103223\_at,104186\_at,104188\_at,104677\_at,160655\_at,160718\_at,161270\_i\_at,94695\_at,94834\_at,94861\_at,96738\_at,97111\_at,97336\_at,97665\_i\_at,99970\_at,103713\_at,160205\_f\_at,92660\_f\_at,92821\_at,93464\_at,94917\_at,95563\_at,95564\_at,96176\_at,99102\_at,93026\_at,101585\_at,102125\_f\_at,103353\_f\_at,103922\_f\_at,160088\_at,160383\_at,160391\_at,160588\_at,160611\_at,162044\_f\_at,93421\_at,93424\_at,93440\_at,93997\_at,94948\_at,95425\_at,96603\_at,97496\_f\_at,98533\_at,99985\_at,161760\_s\_at,96609\_at,104165\_at,97897\_at,94343\_at,94345\_at,94346\_at,100030\_at,104404\_at,94041\_at,104456\_at,160374\_r\_at,92568\_at,101943\_at,160739\_at,104701\_at,102425\_at,103288\_at,100011\_at,100032\_at,100924\_at,101502\_at,102069\_at,102209\_at,102371\_at,102384\_at,102657\_at,102789\_at,102955\_at,102996\_at,103015\_at,103321\_at,103440\_at,103497\_at,103501\_at,103547\_at,103634\_at,103720\_at,103774\_at,104070\_at,104155\_f\_at,104156\_r\_at,104562\_at,104605\_at,104645\_at,160138\_at,160246\_at,160313\_at,160397\_at,160502\_at,160605\_s\_at,160724\_at,160783\_at,160894\_at,161084\_at,161113\_at,161847\_r\_at,162010\_r\_at,92195\_at,92440\_at,92562\_at,92564\_at,92908\_at,93528\_s\_at,93728\_at,93793\_at,94356\_at,94408\_at,94469\_at,94689\_at,94821\_at,95521\_s\_at,95522\_i\_at,95671\_at,95673\_s\_at,96192\_at,96561\_at,96836\_r\_at,96961\_at,97355\_at,97859\_at,98083\_at,98468\_r\_at,98988\_at,99024\_at,99076\_at,99103\_at,99602\_at,99603\_g\_at,99622\_at,92344\_at,96703\_at,98122\_at,93543\_f\_at,100595\_at,101836\_at,93179\_at,93285\_at,94980\_at,98580\_at,100417\_at,101936\_at,102332\_at,103020\_s\_at,103021\_r\_at,103451\_at,103969\_at,93274\_at,93311\_at,97429\_at,98369\_f\_at,102414\_i\_at,102415\_r\_at,102279\_at,160103\_at,160104\_at,93731\_at,103584\_at,98906\_at,102912\_at,104572\_at,93875\_at,94817\_at,95360\_at,97915\_at,97918\_at,100154\_at,100306\_at,100600\_at,100998\_at,101876\_s\_at,101878\_at,101886\_f\_at,103422\_at,160651\_at,92866\_at,93078\_at,93088\_at,93120\_f\_at,93865\_s\_at,93907\_f\_at,94286\_at,97125\_f\_at,97540\_f\_at,97541\_f\_at,98000\_at,98438\_f\_at,98472\_at,99378\_f\_at,99379\_f\_at,100973\_i\_at,103202\_at,104597\_at,98088\_at,103467\_g\_at,100583\_at,102156\_f\_at,102161\_f\_at,102372\_at,93086\_at,95057\_at,95058\_f\_at |
| 1 | biological\_process | 684 | 6769 | 684 | 6769 | 1 | 1 | 1 | 1 | 96147\_at,103693\_at,103699\_i\_at,160726\_at,160727\_at,93619\_at,99535\_at,94420\_f\_at,97925\_at,100134\_at,100327\_at,100482\_at,101392\_at,102356\_at,103816\_at,104083\_at,104206\_at,104407\_at,104694\_at,160430\_at,160469\_at,92558\_at,92559\_at,92877\_at,93063\_at,94454\_at,95511\_at,96752\_at,96886\_at,97519\_at,98849\_at,97312\_at,103518\_at,103736\_at,101217\_at,93705\_at,94657\_at,98011\_at,98324\_at,100122\_at,100514\_at,101465\_at,102994\_at,104389\_at,160495\_at,160941\_at,161609\_at,162206\_f\_at,92484\_at,93016\_at,93315\_at,94331\_at,95023\_at,96252\_at,96852\_at,97844\_at,98427\_s\_at,98504\_at,98508\_s\_at,99100\_at,99475\_at,101144\_at,102255\_at,102658\_at,102663\_at,161689\_f\_at,93874\_s\_at,93914\_at,94928\_at,98254\_f\_at,101457\_at,160252\_at,96488\_at,96489\_at,96764\_at,92216\_at,102983\_at,102984\_g\_at,102032\_at,99532\_at,160440\_at,102224\_at,104417\_at,102787\_at,102794\_at,103362\_at,103658\_r\_at,104445\_at,161788\_f\_at,92268\_at,93193\_at,93459\_s\_at,94192\_at,95474\_at,95477\_at,96912\_s\_at,99416\_at,102321\_at,96532\_at,103596\_at,97375\_at,99364\_at,99366\_at,101186\_at,100024\_at,102750\_at,102957\_at,104256\_at,104257\_g\_at,160296\_at,160490\_at,160632\_at,160911\_at,160965\_at,92302\_at,93017\_at,93714\_f\_at,94109\_at,94556\_at,96513\_at,96736\_at,98946\_at,99467\_at,94264\_at,100951\_at,94060\_at,161666\_f\_at,97740\_at,94448\_at,99070\_at,100561\_at,100635\_at,101030\_at,103062\_at,103901\_at,104114\_at,104117\_at,104179\_at,104725\_at,160082\_s\_at,160757\_at,160993\_at,92185\_at,92805\_s\_at,94031\_at,94106\_at,94319\_at,94505\_at,95418\_at,96530\_at,97058\_f\_at,97224\_at,97319\_at,98927\_at,99032\_at,92854\_at,96238\_at,95287\_at,102823\_at,102824\_g\_at,97949\_at,100988\_at,102064\_at,102734\_at,102905\_at,103812\_at,104100\_at,160920\_at,161067\_at,161980\_f\_at,93064\_at,93093\_at,95412\_at,96255\_at,97285\_f\_at,97890\_at,98436\_s\_at,98868\_at,92925\_at,95102\_at,98945\_at,97825\_at,104761\_at,160301\_at,160464\_s\_at,161610\_at,92653\_at,93550\_at,94752\_s\_at,94809\_at,96088\_at,96596\_at,97118\_at,97498\_at,97973\_at,103531\_f\_at,100130\_at,101475\_at,102364\_at,102920\_at,103990\_at,104477\_at,104576\_at,160829\_at,160901\_at,161551\_f\_at,92926\_at,93104\_at,93666\_at,95348\_at,95617\_at,95618\_at,96771\_at,96810\_at,97484\_at,98110\_at,98756\_at,101998\_at,102752\_at,97426\_at,103899\_at,92778\_i\_at,92780\_f\_at,96633\_s\_at,92263\_at,92270\_at,104263\_at,100606\_at,101118\_at,103959\_at,160676\_at,94976\_at,96299\_at,101578\_f\_at,92542\_at,94270\_at,98968\_at,160065\_s\_at,95489\_at,98127\_at,96278\_at,99013\_f\_at,100342\_i\_at,160287\_at,160288\_at,160679\_at,161615\_f\_at,93729\_at,94835\_f\_at,95119\_at,98461\_at,98882\_s\_at,98884\_r\_at,160373\_i\_at,96945\_at,96779\_f\_at,97409\_at,101848\_g\_at,160399\_r\_at,93020\_at,162138\_s\_at,104376\_at,101015\_s\_at,92770\_at,102781\_at,103416\_at,104598\_at,160127\_at,160359\_at,94820\_r\_at,94881\_at,94882\_at,95471\_at,95805\_at,96728\_at,97504\_at,98067\_at,98478\_at,99187\_f\_at,99188\_at,99529\_f\_at,100307\_at,101930\_at,103500\_at,160603\_at,95613\_at,104735\_at,95917\_at,92638\_at,94483\_at,96600\_at,102292\_at,100088\_at,95033\_at,104149\_at,160511\_at,100567\_at,100944\_at,101079\_at,101568\_at,101787\_f\_at,103035\_at,103065\_at,103376\_s\_at,103427\_at,103739\_at,103913\_at,104019\_at,104461\_at,104534\_at,104719\_at,160683\_at,160684\_at,161696\_f\_at,161990\_f\_at,92392\_at,92847\_s\_at,93316\_at,93320\_at,93414\_at,93626\_at,95377\_at,95444\_at,95586\_at,95695\_at,96019\_at,96076\_at,96079\_at,96669\_at,96725\_at,96876\_at,97181\_f\_at,97458\_at,97812\_at,99500\_at,92648\_at,160124\_r\_at,160202\_at,94043\_at,96951\_at,100499\_at,101934\_at,160149\_at,161026\_s\_at,99350\_at,99354\_s\_at,99358\_at,101023\_f\_at,104453\_at,92256\_at,103686\_at,94254\_at,94255\_g\_at,94256\_at,94464\_at,94465\_g\_at,95655\_at,162041\_f\_at,93471\_at,102854\_s\_at,103935\_at,96186\_at,96534\_at,98114\_at,92531\_at,160795\_at,98926\_at,103258\_at,104522\_at,97551\_at,161184\_f\_at,160151\_i\_at,94004\_at,100992\_at,102381\_at,102644\_at,103086\_at,103328\_at,160483\_at,160857\_at,94473\_at,102940\_at,92248\_at,92249\_g\_at,160099\_at,160834\_at,100522\_s\_at,100523\_r\_at,100533\_s\_at,101515\_at,160526\_s\_at,92888\_s\_at,102094\_f\_at,160199\_at,160200\_at,93267\_at,93520\_at,96777\_at,92992\_i\_at,92993\_r\_at,103499\_at,93083\_at,94383\_at,98018\_at,100068\_at,102768\_i\_at,160092\_at,161401\_f\_at,93241\_r\_at,94815\_at,95135\_at,95440\_at,97451\_at,98931\_at,160338\_at,101990\_at,161946\_r\_at,94439\_at,97833\_at,97834\_g\_at,99045\_at,AFFX-MUR\_b2\_at,104337\_f\_at,98984\_f\_at,94415\_at,93177\_at,160084\_at,101490\_at,102302\_at,99184\_at,96657\_at,92586\_at,102360\_at,101000\_at,101001\_at,102313\_at,160335\_at,103471\_at,99849\_at,97829\_at,101681\_f\_at,103674\_f\_at,103994\_at,104049\_at,104144\_at,160111\_at,160112\_at,160130\_at,160976\_at,160977\_at,161342\_r\_at,92855\_at,93975\_at,94499\_at,95070\_at,95737\_at,96325\_at,100136\_at,101590\_at,95681\_f\_at,96628\_at,160137\_at,94433\_at,160579\_at,94818\_at,93852\_at,97798\_at,102322\_at,94872\_at,99133\_at,93165\_at,100622\_at,95062\_at,100905\_at,101963\_at,103222\_at,103223\_at,104186\_at,104188\_at,104677\_at,160655\_at,160718\_at,161270\_i\_at,94695\_at,94834\_at,94861\_at,96738\_at,97111\_at,97336\_at,97665\_i\_at,99970\_at,103713\_at,160205\_f\_at,92660\_f\_at,92821\_at,93464\_at,94917\_at,95563\_at,95564\_at,96176\_at,99102\_at,93026\_at,101585\_at,102125\_f\_at,103353\_f\_at,103922\_f\_at,160088\_at,160383\_at,160391\_at,160588\_at,160611\_at,162044\_f\_at,93421\_at,93424\_at,93440\_at,93997\_at,94948\_at,95425\_at,96603\_at,97496\_f\_at,98533\_at,99985\_at,161760\_s\_at,96609\_at,104165\_at,97897\_at,94343\_at,94345\_at,94346\_at,100030\_at,104404\_at,94041\_at,104456\_at,160374\_r\_at,92568\_at,101943\_at,160739\_at,104701\_at,102425\_at,103288\_at,100011\_at,100032\_at,100924\_at,101502\_at,102069\_at,102209\_at,102371\_at,102384\_at,102657\_at,102789\_at,102955\_at,102996\_at,103015\_at,103321\_at,103440\_at,103497\_at,103501\_at,103547\_at,103634\_at,103720\_at,103774\_at,104070\_at,104155\_f\_at,104156\_r\_at,104562\_at,104605\_at,104645\_at,160138\_at,160246\_at,160313\_at,160397\_at,160502\_at,160605\_s\_at,160724\_at,160783\_at,160894\_at,161084\_at,161113\_at,161847\_r\_at,162010\_r\_at,92195\_at,92440\_at,92562\_at,92564\_at,92908\_at,93528\_s\_at,93728\_at,93793\_at,94356\_at,94408\_at,94469\_at,94689\_at,94821\_at,95521\_s\_at,95522\_i\_at,95671\_at,95673\_s\_at,96192\_at,96561\_at,96836\_r\_at,96961\_at,97355\_at,97859\_at,98083\_at,98468\_r\_at,98988\_at,99024\_at,99076\_at,99103\_at,99602\_at,99603\_g\_at,99622\_at,92344\_at,96703\_at,98122\_at,93543\_f\_at,100595\_at,101836\_at,93179\_at,93285\_at,94980\_at,98580\_at,100417\_at,101936\_at,102332\_at,103020\_s\_at,103021\_r\_at,103451\_at,103969\_at,93274\_at,93311\_at,97429\_at,98369\_f\_at,102414\_i\_at,102415\_r\_at,102279\_at,160103\_at,160104\_at,93731\_at,103584\_at,98906\_at,102912\_at,104572\_at,93875\_at,94817\_at,95360\_at,97915\_at,97918\_at,100154\_at,100306\_at,100600\_at,100998\_at,101876\_s\_at,101878\_at,101886\_f\_at,103422\_at,160651\_at,92866\_at,93078\_at,93088\_at,93120\_f\_at,93865\_s\_at,93907\_f\_at,94286\_at,97125\_f\_at,97540\_f\_at,97541\_f\_at,98000\_at,98438\_f\_at,98472\_at,99378\_f\_at,99379\_f\_at,100973\_i\_at,103202\_at,104597\_at,98088\_at,103467\_g\_at,100583\_at,102156\_f\_at,102161\_f\_at,102372\_at,93086\_at,95057\_at,95058\_f\_at |
| 2 | behavior | 9 | 63 | 1057 | 10540 | 0.009 | 0.006 | 1.423 | 0.176 | 96147\_at,103693\_at,103699\_i\_at,160726\_at,160727\_at,93619\_at,99535\_at,94420\_f\_at,97925\_at |
| 3 | adult behavior | 3 | 14 | 1040 | 10726 | 0.003 | 0.001 | 2.198 | 0.148 | 96147\_at,103693\_at,103699\_i\_at |
| 4 | adult locomotory behavior | 2 | 10 | 1185 | 13100 | 0.002 | 0.001 | 2.224 | 0.227 | 103693\_at,103699\_i\_at |
| 5 | adult walking behavior | 2 | 5 | 1042 | 11544 | 0.002 | 0 | 4.465 | 0.068 | 103693\_at,103699\_i\_at |
| 3 | locomotory behavior | 4 | 23 | 1040 | 10726 | 0.004 | 0.002 | 1.799 | 0.178 | 160726\_at,160727\_at,103693\_at,103699\_i\_at |
| 4 | adult locomotory behavior | 2 | 10 | 1185 | 13100 | 0.002 | 0.001 | 2.224 | 0.227 | 103693\_at,103699\_i\_at |
| 5 | adult walking behavior | 2 | 5 | 1042 | 11544 | 0.002 | 0 | 4.465 | 0.068 | 103693\_at,103699\_i\_at |
| 3 | rhythmic behavior | 4 | 17 | 1040 | 10726 | 0.004 | 0.002 | 2.437 | 0.075 | 93619\_at,99535\_at,94420\_f\_at,97925\_at |
| 4 | circadian rhythm | 3 | 14 | 1185 | 13100 | 0.003 | 0.001 | 2.364 | 0.127 | 93619\_at,94420\_f\_at,97925\_at |
| 2 | cellular process | 370 | 3616 | 1057 | 10540 | 0.35 | 0.343 | 1.02 | 0.319 | 100134\_at,100327\_at,100482\_at,101392\_at,102356\_at,103816\_at,104083\_at,104206\_at,104407\_at,104694\_at,160430\_at,160469\_at,92558\_at,92559\_at,92877\_at,93063\_at,94454\_at,95511\_at,96752\_at,96886\_at,97519\_at,98849\_at,97312\_at,103518\_at,103736\_at,101217\_at,93705\_at,94657\_at,98011\_at,98324\_at,100122\_at,100514\_at,101465\_at,102994\_at,104389\_at,160495\_at,160941\_at,161609\_at,162206\_f\_at,92484\_at,93016\_at,93315\_at,93619\_at,94331\_at,95023\_at,96252\_at,96852\_at,97844\_at,98427\_s\_at,98504\_at,98508\_s\_at,99100\_at,99475\_at,101144\_at,102255\_at,102658\_at,102663\_at,161689\_f\_at,93874\_s\_at,93914\_at,94928\_at,98254\_f\_at,101457\_at,160252\_at,96488\_at,96489\_at,96764\_at,92216\_at,102983\_at,102984\_g\_at,102032\_at,99532\_at,160440\_at,102224\_at,104417\_at,102787\_at,102794\_at,103362\_at,103658\_r\_at,104445\_at,161788\_f\_at,92268\_at,93193\_at,93459\_s\_at,94192\_at,95474\_at,95477\_at,96912\_s\_at,99416\_at,102321\_at,96532\_at,103596\_at,97375\_at,99364\_at,99366\_at,101186\_at,100024\_at,102750\_at,102957\_at,104256\_at,104257\_g\_at,160296\_at,160490\_at,160632\_at,160911\_at,160965\_at,92302\_at,93017\_at,93714\_f\_at,94109\_at,94556\_at,96513\_at,96736\_at,98946\_at,99467\_at,94264\_at,100951\_at,94060\_at,161666\_f\_at,97740\_at,94448\_at,99070\_at,100561\_at,100635\_at,101030\_at,103062\_at,103901\_at,104114\_at,104117\_at,104179\_at,104725\_at,160082\_s\_at,160757\_at,160993\_at,92185\_at,92805\_s\_at,94031\_at,94106\_at,94319\_at,94505\_at,95418\_at,96530\_at,97058\_f\_at,97224\_at,97319\_at,98927\_at,99032\_at,92854\_at,96238\_at,95287\_at,102823\_at,102824\_g\_at,97949\_at,100988\_at,102064\_at,102734\_at,102905\_at,103812\_at,104100\_at,160920\_at,161067\_at,161980\_f\_at,93064\_at,93093\_at,95412\_at,96255\_at,97285\_f\_at,97890\_at,98436\_s\_at,98868\_at,92925\_at,95102\_at,98945\_at,97825\_at,104761\_at,160301\_at,160464\_s\_at,161610\_at,92653\_at,93550\_at,94752\_s\_at,94809\_at,96088\_at,96596\_at,97118\_at,97498\_at,97973\_at,103531\_f\_at,160726\_at,160727\_at,100130\_at,101475\_at,102364\_at,102920\_at,103990\_at,104477\_at,104576\_at,160829\_at,160901\_at,161551\_f\_at,92926\_at,93104\_at,93666\_at,95348\_at,95617\_at,95618\_at,96771\_at,96810\_at,97484\_at,98110\_at,98756\_at,101998\_at,102752\_at,97426\_at,103899\_at,92778\_i\_at,92780\_f\_at,96633\_s\_at,92263\_at,92270\_at,104263\_at,100606\_at,101118\_at,103959\_at,160676\_at,94976\_at,96299\_at,101578\_f\_at,92542\_at,94270\_at,98968\_at,160065\_s\_at,95489\_at,98127\_at,96278\_at,99013\_f\_at,100342\_i\_at,160287\_at,160288\_at,160679\_at,161615\_f\_at,93729\_at,94835\_f\_at,95119\_at,98461\_at,98882\_s\_at,98884\_r\_at,160373\_i\_at,96945\_at,96779\_f\_at,97409\_at,101848\_g\_at,160399\_r\_at,93020\_at,162138\_s\_at,104376\_at,101015\_s\_at,92770\_at,102781\_at,103416\_at,104598\_at,160127\_at,160359\_at,94820\_r\_at,94881\_at,94882\_at,95471\_at,95805\_at,96728\_at,97504\_at,98067\_at,98478\_at,99187\_f\_at,99188\_at,99529\_f\_at,100307\_at,101930\_at,103500\_at,160603\_at,95613\_at,104735\_at,95917\_at,92638\_at,94483\_at,96147\_at,96600\_at,102292\_at,100088\_at,95033\_at,104149\_at,160511\_at,100567\_at,100944\_at,101079\_at,101568\_at,101787\_f\_at,103035\_at,103065\_at,103376\_s\_at,103427\_at,103739\_at,103913\_at,104019\_at,104461\_at,104534\_at,104719\_at,160683\_at,160684\_at,161696\_f\_at,161990\_f\_at,92392\_at,92847\_s\_at,93316\_at,93320\_at,93414\_at,93626\_at,95377\_at,95444\_at,95586\_at,95695\_at,96019\_at,96076\_at,96079\_at,96669\_at,96725\_at,96876\_at,97181\_f\_at,97458\_at,97812\_at,99500\_at,92648\_at,160124\_r\_at,160202\_at,94043\_at,96951\_at,100499\_at,101934\_at,160149\_at,161026\_s\_at,99350\_at,99354\_s\_at,99358\_at,101023\_f\_at,104453\_at,92256\_at,103686\_at,94254\_at,94255\_g\_at,94256\_at,94464\_at,94465\_g\_at,95655\_at,162041\_f\_at,93471\_at,102854\_s\_at,103935\_at,96186\_at,96534\_at,98114\_at,92531\_at,160795\_at,98926\_at,103258\_at,104522\_at,97551\_at,103693\_at,103699\_i\_at,161184\_f\_at,160151\_i\_at,94004\_at |
| 3 | cell communication | 149 | 1550 | 1040 | 10726 | 0.143 | 0.145 | 0.991 | 0.563 | 100134\_at,100327\_at,100482\_at,101392\_at,102356\_at,103816\_at,104083\_at,104206\_at,104407\_at,104694\_at,160430\_at,160469\_at,92558\_at,92559\_at,92877\_at,93063\_at,94454\_at,95511\_at,96752\_at,96886\_at,97519\_at,98849\_at,97312\_at,103518\_at,103736\_at,101217\_at,93705\_at,94657\_at,98011\_at,98324\_at,100122\_at,100514\_at,101465\_at,102994\_at,104389\_at,160495\_at,160941\_at,161609\_at,162206\_f\_at,92484\_at,93016\_at,93315\_at,93619\_at,94331\_at,95023\_at,96252\_at,96852\_at,97844\_at,98427\_s\_at,98504\_at,98508\_s\_at,99100\_at,99475\_at,101144\_at,102255\_at,102658\_at,102663\_at,161689\_f\_at,93874\_s\_at,93914\_at,94928\_at,98254\_f\_at,101457\_at,160252\_at,96488\_at,96489\_at,96764\_at,92216\_at,102983\_at,102984\_g\_at,102032\_at,99532\_at,160440\_at,102224\_at,104417\_at,102787\_at,102794\_at,103362\_at,103658\_r\_at,104445\_at,161788\_f\_at,92268\_at,93193\_at,93459\_s\_at,94192\_at,95474\_at,95477\_at,96912\_s\_at,99416\_at,102321\_at,96532\_at,103596\_at,97375\_at,99364\_at,99366\_at,101186\_at,100024\_at,102750\_at,102957\_at,104256\_at,104257\_g\_at,160296\_at,160490\_at,160632\_at,160911\_at,160965\_at,92302\_at,93017\_at,93714\_f\_at,94109\_at,94556\_at,96513\_at,96736\_at,98946\_at,99467\_at,94264\_at,100951\_at,94060\_at,161666\_f\_at,97740\_at,94448\_at,99070\_at,100561\_at,100635\_at,101030\_at,103062\_at,103901\_at,104114\_at,104117\_at,104179\_at,104725\_at,160082\_s\_at,160757\_at,160993\_at,92185\_at,92805\_s\_at,94031\_at,94106\_at,94319\_at,94505\_at,95418\_at,96530\_at,97058\_f\_at,97224\_at,97319\_at,98927\_at,99032\_at,92854\_at,96238\_at |
| 4 | cell adhesion | 24 | 322 | 1185 | 13100 | 0.02 | 0.025 | 0.824 | 0.867 | 100134\_at,100327\_at,100482\_at,101392\_at,102356\_at,103816\_at,104083\_at,104206\_at,104407\_at,104694\_at,160430\_at,160469\_at,92558\_at,92559\_at,92877\_at,93063\_at,94454\_at,95511\_at,96752\_at,96886\_at,97519\_at,98849\_at,97312\_at,103518\_at |
| 5 | cell-cell adhesion | 6 | 44 | 1042 | 11544 | 0.006 | 0.004 | 1.512 | 0.202 | 92558\_at,92559\_at,96752\_at,97312\_at,103518\_at,104083\_at |
| 6 | heterophilic cell adhesion | 1 | 2 | 880 | 9498 | 0.001 | 0 | 5.429 | 0.177 | 97312\_at |
| 6 | homophilic cell adhesion | 2 | 31 | 880 | 9498 | 0.002 | 0.003 | 0.696 | 0.796 | 103518\_at,104083\_at |
| 5 | cell-matrix adhesion | 2 | 50 | 1042 | 11544 | 0.002 | 0.004 | 0.443 | 0.948 | 102356\_at,95511\_at |
| 4 | cell-cell signaling | 5 | 123 | 1185 | 13100 | 0.004 | 0.009 | 0.449 | 0.989 | 103736\_at,101217\_at,93705\_at,94657\_at,98011\_at |
| 5 | transmission of nerve impulse | 4 | 82 | 1042 | 11544 | 0.004 | 0.007 | 0.541 | 0.946 | 101217\_at,93705\_at,94657\_at,98011\_at |
| 6 | synaptic transmission | 4 | 80 | 880 | 9498 | 0.005 | 0.008 | 0.54 | 0.946 | 101217\_at,93705\_at,94657\_at,98011\_at |
| 4 | response to extracellular stimulus | 1 | 1 | 1185 | 13100 | 0.001 | 0 | 10.5 | 0.09 | 98324\_at |
| 5 | cellular response to starvation | 1 | 1 | 1042 | 11544 | 0.001 | 0 | 10.667 | 0.09 | 98324\_at |
| 4 | signal transduction | 129 | 1199 | 1185 | 13100 | 0.109 | 0.092 | 1.189 | 0.019 | 100122\_at,100514\_at,101465\_at,102994\_at,103736\_at,104389\_at,104407\_at,160495\_at,160941\_at,161609\_at,162206\_f\_at,92484\_at,93016\_at,93315\_at,93619\_at,94331\_at,95023\_at,96252\_at,96852\_at,97844\_at,98427\_s\_at,98504\_at,98508\_s\_at,99100\_at,99475\_at,101144\_at,102255\_at,102658\_at,102663\_at,161689\_f\_at,93874\_s\_at,93914\_at,94928\_at,98254\_f\_at,101457\_at,160252\_at,96488\_at,96489\_at,96764\_at,92216\_at,102983\_at,102984\_g\_at,102032\_at,99532\_at,160440\_at,100134\_at,102224\_at,104417\_at,102787\_at,102794\_at,103362\_at,103518\_at,103658\_r\_at,104445\_at,161788\_f\_at,92268\_at,93193\_at,93459\_s\_at,94192\_at,95474\_at,95477\_at,96912\_s\_at,98011\_at,99416\_at,102321\_at,96532\_at,103596\_at,101217\_at,94657\_at,97375\_at,102356\_at,95511\_at,160430\_at,99364\_at,99366\_at,101186\_at,100024\_at,102750\_at,102957\_at,104256\_at,104257\_g\_at,160296\_at,160490\_at,160632\_at,160911\_at,160965\_at,92302\_at,93017\_at,93714\_f\_at,94109\_at,94556\_at,96513\_at,96736\_at,98946\_at,99467\_at,94264\_at,100951\_at,94060\_at,161666\_f\_at,97740\_at,94448\_at,99070\_at,100561\_at,100635\_at,101030\_at,103062\_at,103901\_at,104114\_at,104117\_at,104179\_at,104725\_at,160082\_s\_at,160757\_at,160993\_at,92185\_at,92805\_s\_at,94031\_at,94106\_at,94319\_at,94505\_at,95418\_at,96530\_at,97058\_f\_at,97224\_at,97319\_at,98927\_at,99032\_at,92854\_at,96238\_at |
| 5 | cell surface receptor linked signal transduction | 57 | 621 | 1042 | 11544 | 0.055 | 0.054 | 1.017 | 0.467 | 101144\_at,102255\_at,102658\_at,102663\_at,161689\_f\_at,93874\_s\_at,93914\_at,94928\_at,98254\_f\_at,101457\_at,102994\_at,160252\_at,96488\_at,96489\_at,96764\_at,92216\_at,102983\_at,102984\_g\_at,102032\_at,99532\_at,160440\_at,100134\_at,102224\_at,104417\_at,100122\_at,100514\_at,102787\_at,102794\_at,103362\_at,103518\_at,103658\_r\_at,104445\_at,161609\_at,161788\_f\_at,92268\_at,93193\_at,93459\_s\_at,94192\_at,95474\_at,95477\_at,96912\_s\_at,97844\_at,98011\_at,99416\_at,102321\_at,96532\_at,103596\_at,101217\_at,94657\_at,97375\_at,102356\_at,95511\_at,160430\_at,103736\_at,99364\_at,99366\_at,101186\_at |
| 6 | cytokine and chemokine mediated signaling pathway | 6 | 9 | 880 | 9498 | 0.007 | 0.001 | 7.179 | 0 | 101457\_at,102994\_at,160252\_at,96488\_at,96489\_at,96764\_at |
| 6 | enzyme linked receptor protein signaling pathway | 9 | 131 | 880 | 9498 | 0.01 | 0.014 | 0.742 | 0.868 | 92216\_at,102983\_at,102984\_g\_at,102032\_at,99532\_at,160440\_at,100134\_at,102224\_at,104417\_at |
| 7 | transmembrane receptor protein serine/threonine kinase signaling pathway | 7 | 39 | 538 | 6246 | 0.013 | 0.006 | 2.085 | 0.046 | 92216\_at,102983\_at,102984\_g\_at,102032\_at,99532\_at,160440\_at,100134\_at |
| 8 | TGFbeta receptor signaling pathway | 7 | 31 | 199 | 2164 | 0.035 | 0.014 | 2.455 | 0.019 | 92216\_at,102983\_at,102984\_g\_at,102032\_at,99532\_at,160440\_at,100134\_at |
| 9 | BMP receptor signaling pathway | 4 | 5 | 72 | 911 | 0.056 | 0.005 | 10.12 | 0 | 102983\_at,102984\_g\_at,102032\_at,99532\_at |
| 10 | regulation of BMP signaling pathway | 2 | 2 | 15 | 197 | 0.133 | 0.01 | 13.136 | 0.005 | 102032\_at,99532\_at |
| 11 | negative regulation of BMP signaling pathway | 2 | 2 | 5 | 34 | 0.4 | 0.059 | 6.8 | 0.018 | 102032\_at,99532\_at |
| 11 | positive regulation of BMP signaling pathway | 1 | 1 | 5 | 34 | 0.2 | 0.029 | 6.8 | 0.147 | 102032\_at |
| 9 | common-partner SMAD protein phosphorylation | 4 | 9 | 72 | 911 | 0.056 | 0.01 | 5.623 | 0.003 | 102983\_at,102984\_g\_at,160440\_at,92216\_at |
| 9 | regulation of TGFbeta receptor signaling pathway | 1 | 4 | 72 | 911 | 0.014 | 0.004 | 3.164 | 0.281 | 100134\_at |
| 9 | SMAD protein heteromerization | 2 | 5 | 72 | 911 | 0.028 | 0.005 | 5.06 | 0.053 | 102983\_at,102984\_g\_at |
| 9 | SMAD protein nuclear translocation | 2 | 3 | 72 | 911 | 0.028 | 0.003 | 8.444 | 0.018 | 160440\_at,99532\_at |
| 7 | transmembrane receptor protein tyrosine kinase signaling pathway | 2 | 61 | 538 | 6246 | 0.004 | 0.01 | 0.381 | 0.973 | 102224\_at,104417\_at |
| 6 | G-protein coupled receptor protein signaling pathway | 26 | 355 | 880 | 9498 | 0.03 | 0.037 | 0.791 | 0.92 | 100122\_at,100514\_at,102787\_at,102794\_at,103362\_at,103518\_at,103658\_r\_at,104445\_at,161609\_at,161788\_f\_at,92268\_at,93193\_at,93459\_s\_at,94192\_at,95474\_at,95477\_at,96912\_s\_at,97844\_at,98011\_at,99416\_at,102321\_at,96532\_at,103596\_at,101217\_at,94657\_at,97375\_at |
| 7 | G-protein signaling, coupled to cyclic nucleotide second messenger | 4 | 33 | 538 | 6246 | 0.007 | 0.005 | 1.407 | 0.316 | 103362\_at,102321\_at,161788\_f\_at,96532\_at |
| 8 | G-protein signaling, coupled to cAMP nucleotide second messenger | 3 | 27 | 199 | 2164 | 0.015 | 0.012 | 1.208 | 0.458 | 103362\_at,102321\_at,161788\_f\_at |
| 9 | G-protein signaling, adenylate cyclase activating pathway | 2 | 17 | 72 | 911 | 0.028 | 0.019 | 1.489 | 0.394 | 103362\_at,102321\_at |
| 10 | adenylate cyclase activation | 1 | 8 | 15 | 197 | 0.067 | 0.041 | 1.642 | 0.476 | 102321\_at |
| 9 | G-protein signaling, adenylate cyclase inhibiting pathway | 1 | 10 | 72 | 911 | 0.014 | 0.011 | 1.265 | 0.563 | 161788\_f\_at |
| 8 | G-protein signaling, coupled to cGMP nucleotide second messenger | 1 | 6 | 199 | 2164 | 0.005 | 0.003 | 1.816 | 0.44 | 96532\_at |
| 7 | G-protein signaling, coupled to IP3 second messenger (phospholipase C activating) | 1 | 13 | 538 | 6246 | 0.002 | 0.002 | 0.894 | 0.69 | 103596\_at |
| 8 | protein kinase C activation | 1 | 5 | 199 | 2164 | 0.005 | 0.002 | 2.177 | 0.383 | 103596\_at |
| 7 | gamma-aminobutyric acid signaling pathway | 2 | 14 | 538 | 6246 | 0.004 | 0.002 | 1.661 | 0.343 | 101217\_at,94657\_at |
| 7 | neuropeptide signaling pathway | 3 | 45 | 538 | 6246 | 0.006 | 0.007 | 0.775 | 0.757 | 102787\_at,103518\_at,97375\_at |
| 6 | integrin-mediated signaling pathway | 2 | 45 | 880 | 9498 | 0.002 | 0.005 | 0.479 | 0.93 | 102356\_at,95511\_at |
| 6 | Wnt receptor signaling pathway | 5 | 30 | 880 | 9498 | 0.006 | 0.003 | 1.797 | 0.139 | 160430\_at,103736\_at,99364\_at,99366\_at,101186\_at |
| 7 | frizzled-2 signaling pathway | 3 | 16 | 538 | 6246 | 0.006 | 0.003 | 2.18 | 0.154 | 103736\_at,99364\_at,99366\_at |
| 7 | regulation of Wnt receptor signaling pathway | 1 | 4 | 538 | 6246 | 0.002 | 0.001 | 2.906 | 0.303 | 101186\_at |
| 5 | intracellular signaling cascade | 67 | 485 | 1042 | 11544 | 0.064 | 0.042 | 1.531 | 0 | 100024\_at,101457\_at,101465\_at,102321\_at,102750\_at,102957\_at,102994\_at,103596\_at,104256\_at,104257\_g\_at,160296\_at,160490\_at,160632\_at,160911\_at,160965\_at,162206\_f\_at,92302\_at,93017\_at,93714\_f\_at,94109\_at,94331\_at,94556\_at,96488\_at,96489\_at,96513\_at,96736\_at,98504\_at,98946\_at,99100\_at,99467\_at,99475\_at,100514\_at,94264\_at,100951\_at,97375\_at,94060\_at,93315\_at,161666\_f\_at,97740\_at,94448\_at,99070\_at,100561\_at,100635\_at,101030\_at,103062\_at,103901\_at,104114\_at,104117\_at,104179\_at,104725\_at,160082\_s\_at,160757\_at,160993\_at,92185\_at,92805\_s\_at,93016\_at,94031\_at,94106\_at,94319\_at,94505\_at,95418\_at,96530\_at,97058\_f\_at,97224\_at,97319\_at,98927\_at,99032\_at |
| 6 | protein kinase cascade | 14 | 50 | 880 | 9498 | 0.016 | 0.005 | 3.025 | 0 | 100514\_at,94264\_at,100951\_at,101457\_at,96488\_at,96489\_at,97375\_at,99100\_at,94060\_at,93315\_at,161666\_f\_at,97740\_at,94448\_at,99070\_at |
| 7 | JAK-STAT cascade | 6 | 10 | 538 | 6246 | 0.011 | 0.002 | 6.969 | 0 | 100951\_at,101457\_at,96488\_at,96489\_at,97375\_at,99100\_at |
| 8 | STAT protein nuclear translocation | 3 | 3 | 199 | 2164 | 0.015 | 0.001 | 10.849 | 0.001 | 101457\_at,96488\_at,96489\_at |
| 8 | tyrosine phosphorylation of STAT protein | 3 | 2 | 199 | 2164 | 0.015 | 0.001 | 16.391 | 0 | 101457\_at,96488\_at,96489\_at |
| 7 | JNK cascade | 1 | 13 | 538 | 6246 | 0.002 | 0.002 | 0.894 | 0.69 | 94060\_at |
| 7 | MAPKKK cascade | 3 | 18 | 538 | 6246 | 0.006 | 0.003 | 1.938 | 0.198 | 93315\_at,161666\_f\_at,97740\_at |
| 8 | activation of MAPKK | 1 | 3 | 199 | 2164 | 0.005 | 0.001 | 3.619 | 0.251 | 161666\_f\_at |
| 8 | inactivation of MAPK | 1 | 4 | 199 | 2164 | 0.005 | 0.002 | 2.719 | 0.32 | 97740\_at |
| 7 | NIK-I-kappaB/NF-kappaB cascade | 2 | 7 | 538 | 6246 | 0.004 | 0.001 | 3.321 | 0.116 | 94448\_at,99070\_at |
| 8 | I-kappaB phosphorylation | 1 | 2 | 199 | 2164 | 0.005 | 0.001 | 5.467 | 0.176 | 99070\_at |
| 6 | small GTPase mediated signal transduction | 30 | 135 | 880 | 9498 | 0.034 | 0.014 | 2.399 | 0 | 100561\_at,100635\_at,101030\_at,103062\_at,103901\_at,104114\_at,104117\_at,104179\_at,104725\_at,160082\_s\_at,160757\_at,160911\_at,160993\_at,92185\_at,92302\_at,92805\_s\_at,93016\_at,94031\_at,94106\_at,94319\_at,94505\_at,95418\_at,96530\_at,97058\_f\_at,97224\_at,97319\_at,98927\_at,99032\_at,100514\_at,96736\_at |
| 7 | Rho protein signal transduction | 4 | 20 | 538 | 6246 | 0.007 | 0.003 | 2.322 | 0.087 | 100514\_at,101030\_at,94106\_at,96736\_at |
| 5 | two-component signal transduction system (phosphorelay) | 5 | 15 | 1042 | 11544 | 0.005 | 0.001 | 3.692 | 0.008 | 92854\_at,94031\_at,94319\_at,94505\_at,96238\_at |
| 3 | cell death | 33 | 207 | 1040 | 10726 | 0.032 | 0.019 | 1.644 | 0.003 | 95287\_at,102823\_at,102824\_g\_at,97949\_at,100988\_at,101457\_at,102064\_at,102734\_at,102905\_at,103812\_at,104100\_at,160920\_at,161067\_at,161666\_f\_at,161980\_f\_at,93063\_at,93064\_at,93093\_at,94448\_at,95412\_at,96252\_at,96255\_at,96488\_at,96489\_at,97285\_f\_at,97890\_at,98427\_s\_at,98436\_s\_at,98868\_at,92925\_at,95102\_at,98945\_at,97825\_at |
| 4 | cytolysis | 3 | 15 | 1185 | 13100 | 0.003 | 0.001 | 2.2 | 0.148 | 102823\_at,102824\_g\_at,97949\_at |
| 4 | programmed cell death | 29 | 192 | 1185 | 13100 | 0.024 | 0.015 | 1.669 | 0.004 | 100988\_at,101457\_at,102064\_at,102734\_at,102905\_at,103812\_at,104100\_at,160920\_at,161067\_at,161666\_f\_at,161980\_f\_at,93063\_at,93064\_at,93093\_at,94448\_at,95412\_at,96252\_at,96255\_at,96488\_at,96489\_at,97285\_f\_at,97890\_at,98427\_s\_at,98436\_s\_at,98868\_at,92925\_at,95102\_at,98945\_at,97825\_at |
| 5 | apoptosis | 29 | 192 | 1042 | 11544 | 0.028 | 0.017 | 1.673 | 0.004 | 100988\_at,101457\_at,102064\_at,102734\_at,102905\_at,103812\_at,104100\_at,160920\_at,161067\_at,161666\_f\_at,161980\_f\_at,93063\_at,93064\_at,93093\_at,94448\_at,95412\_at,96252\_at,96255\_at,96488\_at,96489\_at,97285\_f\_at,97890\_at,98427\_s\_at,98436\_s\_at,98868\_at,92925\_at,95102\_at,98945\_at,97825\_at |
| 6 | anti-apoptosis | 3 | 31 | 880 | 9498 | 0.003 | 0.003 | 1.046 | 0.558 | 102734\_at,161980\_f\_at,92925\_at |
| 6 | apoptotic program | 3 | 16 | 880 | 9498 | 0.003 | 0.002 | 2.03 | 0.18 | 95102\_at,98945\_at,94448\_at |
| 7 | caspase activation | 1 | 7 | 538 | 6246 | 0.002 | 0.001 | 1.661 | 0.468 | 94448\_at |
| 6 | induction of apoptosis | 6 | 28 | 880 | 9498 | 0.007 | 0.003 | 2.312 | 0.04 | 102064\_at,102905\_at,92925\_at,95102\_at,97825\_at,98436\_s\_at |
| 3 | cell differentiation | 24 | 137 | 1040 | 10726 | 0.023 | 0.013 | 1.807 | 0.003 | 100514\_at,101457\_at,104761\_at,160301\_at,160464\_s\_at,161610\_at,161666\_f\_at,92653\_at,93550\_at,94752\_s\_at,94809\_at,96088\_at,96488\_at,96489\_at,96596\_at,97118\_at,97498\_at,97973\_at,92925\_at,99475\_at,99532\_at,103531\_f\_at,160726\_at,160727\_at |
| 4 | cell fate commitment | 1 | 13 | 1185 | 13100 | 0.001 | 0.001 | 0.848 | 0.709 | 97973\_at |
| 4 | keratinocyte differentiation | 1 | 3 | 1185 | 13100 | 0.001 | 0 | 3.652 | 0.248 | 94809\_at |
| 4 | myeloid blood cell differentiation | 1 | 7 | 1185 | 13100 | 0.001 | 0.001 | 1.585 | 0.485 | 97973\_at |
| 5 | erythrocyte differentiation | 1 | 3 | 1042 | 11544 | 0.001 | 0 | 3.692 | 0.247 | 97973\_at |
| 4 | neuron differentiation | 2 | 5 | 1185 | 13100 | 0.002 | 0 | 4.447 | 0.068 | 92925\_at,99475\_at |
| 5 | regulation of neuron differentiation | 1 | 1 | 1042 | 11544 | 0.001 | 0 | 10.667 | 0.09 | 99475\_at |
| 6 | positive regulation of neuron differentiation | 1 | 1 | 880 | 9498 | 0.001 | 0 | 10.364 | 0.093 | 99475\_at |
| 4 | osteoblast differentiation | 1 | 1 | 1185 | 13100 | 0.001 | 0 | 10.5 | 0.09 | 99532\_at |
| 5 | regulation of osteoblast differentiation | 1 | 1 | 1042 | 11544 | 0.001 | 0 | 10.667 | 0.09 | 99532\_at |
| 6 | negative regulation of osteoblast differentiation | 1 | 1 | 880 | 9498 | 0.001 | 0 | 10.364 | 0.093 | 99532\_at |
| 4 | spermatid development | 1 | 11 | 1185 | 13100 | 0.001 | 0.001 | 1 | 0.648 | 103531\_f\_at |
| 4 | vasculogenesis | 2 | 7 | 1185 | 13100 | 0.002 | 0.001 | 3.189 | 0.127 | 160726\_at,160727\_at |
| 3 | cell growth and/or maintenance | 230 | 2128 | 1040 | 10726 | 0.221 | 0.198 | 1.115 | 0.03 | 100130\_at,101030\_at,101475\_at,102364\_at,102920\_at,103990\_at,104477\_at,104576\_at,160829\_at,160901\_at,161551\_f\_at,92653\_at,92926\_at,93104\_at,93666\_at,93714\_f\_at,94264\_at,94448\_at,94505\_at,94752\_s\_at,95348\_at,95617\_at,95618\_at,96513\_at,96771\_at,96810\_at,97484\_at,97973\_at,98110\_at,98756\_at,99467\_at,101998\_at,102752\_at,97426\_at,97498\_at,103899\_at,162206\_f\_at,92778\_i\_at,92780\_f\_at,94809\_at,96633\_s\_at,99475\_at,92263\_at,92270\_at,98324\_at,104263\_at,100606\_at,101118\_at,103959\_at,160676\_at,94976\_at,100514\_at,104725\_at,96299\_at,96736\_at,101578\_f\_at,92542\_at,94270\_at,98968\_at,160065\_s\_at,95489\_at,98127\_at,94106\_at,96278\_at,99013\_f\_at,100342\_i\_at,160287\_at,160288\_at,160679\_at,161615\_f\_at,93729\_at,94835\_f\_at,95119\_at,98461\_at,98882\_s\_at,98884\_r\_at,160373\_i\_at,96945\_at,96779\_f\_at,97409\_at,101848\_g\_at,160399\_r\_at,93020\_at,162138\_s\_at,104376\_at,101015\_s\_at,102994\_at,104761\_at,92770\_at,94928\_at,96852\_at,102781\_at,103416\_at,104598\_at,160127\_at,160359\_at,160495\_at,94820\_r\_at,94881\_at,94882\_at,95471\_at,95805\_at,96728\_at,97504\_at,97844\_at,98067\_at,98478\_at,99187\_f\_at,99188\_at,99529\_f\_at,100307\_at,101930\_at,103500\_at,160603\_at,95613\_at,104735\_at,95917\_at,92638\_at,94483\_at,96147\_at,96600\_at,100951\_at,102292\_at,97375\_at,100088\_at,95033\_at,104149\_at,94331\_at,101457\_at,160301\_at,96488\_at,96489\_at,102794\_at,160511\_at,100567\_at,100944\_at,101079\_at,101217\_at,101568\_at,101787\_f\_at,102658\_at,103035\_at,103065\_at,103376\_s\_at,103427\_at,103739\_at,103913\_at,104019\_at,104461\_at,104534\_at,104719\_at,160683\_at,160684\_at,161689\_f\_at,161696\_f\_at,161990\_f\_at,92392\_at,92847\_s\_at,93316\_at,93320\_at,93414\_at,93626\_at,93705\_at,94031\_at,94556\_at,94657\_at,95377\_at,95444\_at,95586\_at,95695\_at,96019\_at,96076\_at,96079\_at,96669\_at,96725\_at,96876\_at,97181\_f\_at,97458\_at,97812\_at,99500\_at,92648\_at,160124\_r\_at,160202\_at,94043\_at,96951\_at,100499\_at,100635\_at,101934\_at,102750\_at,103062\_at,104179\_at,160082\_s\_at,160149\_at,161026\_s\_at,92185\_at,92805\_s\_at,94319\_at,96530\_at,97058\_f\_at,97224\_at,98927\_at,99350\_at,99354\_s\_at,99358\_at,101023\_f\_at,104453\_at,92256\_at,95023\_at,103686\_at,94254\_at,94255\_g\_at,94256\_at,94464\_at,94465\_g\_at,95655\_at,162041\_f\_at,93471\_at,103812\_at,102854\_s\_at,103935\_at,96186\_at,96534\_at,98114\_at,92531\_at,160795\_at,98926\_at,103258\_at,104522\_at,93063\_at,97551\_at |
| 4 | autophagy | 1 | 6 | 1185 | 13100 | 0.001 | 0 | 1.826 | 0.434 | 101998\_at |
| 4 | cell growth | 13 | 51 | 1185 | 13100 | 0.011 | 0.004 | 2.82 | 0 | 102752\_at,97426\_at,97498\_at,103899\_at,162206\_f\_at,92778\_i\_at,92780\_f\_at,93714\_f\_at,94809\_at,96633\_s\_at,99475\_at,92263\_at,92270\_at |
| 5 | regulation of cell growth | 10 | 38 | 1042 | 11544 | 0.01 | 0.003 | 2.918 | 0.002 | 103899\_at,162206\_f\_at,92778\_i\_at,92780\_f\_at,93714\_f\_at,94809\_at,96633\_s\_at,99475\_at,92263\_at,92270\_at |
| 6 | negative regulation of cell growth | 2 | 2 | 880 | 9498 | 0.002 | 0 | 10.81 | 0.009 | 92263\_at,92270\_at |
| 4 | cell homeostasis | 7 | 41 | 1185 | 13100 | 0.006 | 0.003 | 1.888 | 0.073 | 98324\_at,104263\_at,100606\_at,101118\_at,103959\_at,160676\_at,94976\_at |
| 5 | cell glucose homeostasis | 1 | 2 | 1042 | 11544 | 0.001 | 0 | 5.647 | 0.172 | 98324\_at |
| 5 | cell ion homeostasis | 6 | 37 | 1042 | 11544 | 0.006 | 0.003 | 1.794 | 0.112 | 104263\_at,100606\_at,101118\_at,103959\_at,160676\_at,94976\_at |
| 6 | cation homeostasis | 6 | 36 | 880 | 9498 | 0.007 | 0.004 | 1.799 | 0.111 | 104263\_at,100606\_at,101118\_at,103959\_at,160676\_at,94976\_at |
| 7 | di-, tri-valent inorganic cation homeostasis | 6 | 29 | 538 | 6246 | 0.011 | 0.005 | 2.403 | 0.034 | 104263\_at,100606\_at,101118\_at,103959\_at,160676\_at,94976\_at |
| 8 | calcium ion homeostasis | 1 | 13 | 199 | 2164 | 0.005 | 0.006 | 0.837 | 0.716 | 104263\_at |
| 8 | copper ion homeostasis | 1 | 1 | 199 | 2164 | 0.005 | 0 | 10.935 | 0.092 | 100606\_at |
| 8 | iron ion homeostasis | 4 | 15 | 199 | 2164 | 0.02 | 0.007 | 2.9 | 0.042 | 101118\_at,103959\_at,160676\_at,94976\_at |
| 4 | cell organization and biogenesis | 35 | 530 | 1185 | 13100 | 0.03 | 0.04 | 0.73 | 0.984 | 100514\_at,104725\_at,96299\_at,96736\_at,101578\_f\_at,92542\_at,94270\_at,98968\_at,160065\_s\_at,95489\_at,98127\_at,94106\_at,96278\_at,99013\_f\_at,100342\_i\_at,160287\_at,160288\_at,160679\_at,161615\_f\_at,93729\_at,94835\_f\_at,95119\_at,98461\_at,98882\_s\_at,98884\_r\_at,160373\_i\_at,96945\_at,96779\_f\_at,97409\_at,101848\_g\_at,160399\_r\_at,93020\_at,162138\_s\_at,101475\_at,104376\_at |
| 5 | cellular morphogenesis | 3 | 49 | 1042 | 11544 | 0.003 | 0.004 | 0.679 | 0.831 | 100514\_at,104725\_at,96299\_at |
| 6 | regulation of cell shape | 3 | 22 | 880 | 9498 | 0.003 | 0.002 | 1.47 | 0.334 | 100514\_at,104725\_at,96299\_at |
| 5 | cytoplasm organization and biogenesis | 28 | 380 | 1042 | 11544 | 0.027 | 0.033 | 0.816 | 0.895 | 96736\_at,101578\_f\_at,92542\_at,94270\_at,98968\_at,104725\_at,160065\_s\_at,95489\_at,98127\_at,94106\_at,96278\_at,96299\_at,99013\_f\_at,100342\_i\_at,160287\_at,160288\_at,160679\_at,161615\_f\_at,93729\_at,94835\_f\_at,95119\_at,98461\_at,98882\_s\_at,98884\_r\_at,160373\_i\_at,96945\_at,96779\_f\_at,97409\_at |
| 6 | organelle organization and biogenesis | 25 | 318 | 880 | 9498 | 0.028 | 0.033 | 0.849 | 0.835 | 101578\_f\_at,92542\_at,94270\_at,98968\_at,104725\_at,160065\_s\_at,95489\_at,98127\_at,94106\_at,96278\_at,96299\_at,99013\_f\_at,100342\_i\_at,160287\_at,160288\_at,160679\_at,161615\_f\_at,93729\_at,94835\_f\_at,95119\_at,98461\_at,98882\_s\_at,98884\_r\_at,160373\_i\_at,96945\_at |
| 7 | cytoskeleton organization and biogenesis | 23 | 262 | 538 | 6246 | 0.043 | 0.042 | 1.019 | 0.495 | 101578\_f\_at,92542\_at,94270\_at,98968\_at,104725\_at,160065\_s\_at,95489\_at,98127\_at,94106\_at,96278\_at,96299\_at,99013\_f\_at,100342\_i\_at,160287\_at,160288\_at,160679\_at,161615\_f\_at,93729\_at,94835\_f\_at,95119\_at,98461\_at,98882\_s\_at,98884\_r\_at |
| 8 | actin filament-based process | 8 | 42 | 199 | 2164 | 0.04 | 0.019 | 2.071 | 0.034 | 104725\_at,160065\_s\_at,95489\_at,98127\_at,94106\_at,96278\_at,96299\_at,99013\_f\_at |
| 9 | actin cytoskeleton organization and biogenesis | 8 | 42 | 72 | 911 | 0.111 | 0.046 | 2.41 | 0.014 | 104725\_at,160065\_s\_at,95489\_at,98127\_at,94106\_at,96278\_at,96299\_at,99013\_f\_at |
| 10 | actin filament organization | 4 | 16 | 15 | 197 | 0.267 | 0.081 | 3.283 | 0.023 | 94106\_at,96278\_at,96299\_at,99013\_f\_at |
| 8 | microtubule-based process | 12 | 119 | 199 | 2164 | 0.06 | 0.055 | 1.097 | 0.413 | 100342\_i\_at,160287\_at,160288\_at,160679\_at,161615\_f\_at,93729\_at,94835\_f\_at,95119\_at,96299\_at,98461\_at,98882\_s\_at,98884\_r\_at |
| 9 | microtubule-based movement | 4 | 38 | 72 | 911 | 0.056 | 0.042 | 1.332 | 0.353 | 100342\_i\_at,96299\_at,98882\_s\_at,98884\_r\_at |
| 10 | axon cargo transport | 2 | 4 | 15 | 197 | 0.133 | 0.02 | 6.568 | 0.03 | 98882\_s\_at,98884\_r\_at |
| 11 | retrograde axon cargo transport | 2 | 4 | 5 | 34 | 0.4 | 0.118 | 3.4 | 0.094 | 98882\_s\_at,98884\_r\_at |
| 7 | ER organization and biogenesis | 1 | 6 | 538 | 6246 | 0.002 | 0.001 | 1.938 | 0.418 | 160373\_i\_at |
| 8 | protein-ER targeting | 1 | 6 | 199 | 2164 | 0.005 | 0.003 | 1.816 | 0.44 | 160373\_i\_at |
| 9 | cotranslational membrane targeting | 1 | 3 | 72 | 911 | 0.014 | 0.003 | 4.222 | 0.219 | 160373\_i\_at |
| 7 | vacuole organization and biogenesis | 1 | 7 | 538 | 6246 | 0.002 | 0.001 | 1.661 | 0.468 | 96945\_at |
| 6 | ribosome biogenesis and assembly | 2 | 60 | 880 | 9498 | 0.002 | 0.006 | 0.359 | 0.979 | 96779\_f\_at,97409\_at |
| 7 | ribosome biogenesis | 2 | 60 | 538 | 6246 | 0.004 | 0.01 | 0.387 | 0.971 | 96779\_f\_at,97409\_at |
| 5 | nuclear organization and biogenesis | 6 | 112 | 1042 | 11544 | 0.006 | 0.01 | 0.594 | 0.946 | 101848\_g\_at,160399\_r\_at,93020\_at,162138\_s\_at,101475\_at,104376\_at |
| 6 | chromosome organization and biogenesis (sensu Eukarya) | 6 | 108 | 880 | 9498 | 0.007 | 0.011 | 0.6 | 0.943 | 101848\_g\_at,160399\_r\_at,93020\_at,162138\_s\_at,101475\_at,104376\_at |
| 7 | establishment and/or maintenance of chromatin architecture | 5 | 80 | 538 | 6246 | 0.009 | 0.013 | 0.725 | 0.831 | 162138\_s\_at,160399\_r\_at,93020\_at,101475\_at,104376\_at |
| 8 | chromatin assembly/disassembly | 3 | 48 | 199 | 2164 | 0.015 | 0.022 | 0.68 | 0.833 | 162138\_s\_at,160399\_r\_at,93020\_at |
| 9 | nucleosome assembly | 2 | 28 | 72 | 911 | 0.028 | 0.031 | 0.904 | 0.665 | 160399\_r\_at,93020\_at |
| 8 | chromatin modification | 3 | 36 | 199 | 2164 | 0.015 | 0.017 | 0.906 | 0.657 | 101475\_at,104376\_at,162138\_s\_at |
| 4 | cell proliferation | 68 | 501 | 1185 | 13100 | 0.057 | 0.038 | 1.501 | 0 | 101015\_s\_at,102752\_at,102994\_at,104761\_at,92770\_at,93104\_at,94928\_at,96852\_at,97484\_at,102781\_at,103416\_at,104477\_at,104598\_at,160127\_at,160359\_at,160495\_at,94820\_r\_at,94881\_at,94882\_at,95471\_at,95617\_at,95618\_at,95805\_at,96728\_at,97504\_at,97844\_at,98067\_at,98478\_at,99187\_f\_at,99188\_at,99529\_f\_at,100307\_at,101930\_at,103500\_at,160603\_at,95613\_at,104735\_at,95917\_at,100130\_at,102364\_at,102920\_at,103990\_at,160901\_at,92638\_at,92926\_at,94264\_at,94483\_at,94505\_at,95348\_at,96147\_at,96513\_at,96600\_at,96771\_at,98756\_at,100951\_at,102292\_at,94809\_at,97375\_at,100088\_at,95033\_at,104149\_at,94331\_at,101457\_at,160301\_at,96488\_at,96489\_at,102794\_at,160511\_at |
| 5 | cell cycle | 51 | 435 | 1042 | 11544 | 0.049 | 0.038 | 1.299 | 0.031 | 102781\_at,103416\_at,104477\_at,104598\_at,160127\_at,160359\_at,160495\_at,92770\_at,94820\_r\_at,94881\_at,94882\_at,95471\_at,95617\_at,95618\_at,95805\_at,96728\_at,97504\_at,97844\_at,98067\_at,98478\_at,99187\_f\_at,99188\_at,99529\_f\_at,100307\_at,101930\_at,103500\_at,160603\_at,95613\_at,104735\_at,95917\_at,100130\_at,102364\_at,102752\_at,102920\_at,103990\_at,160901\_at,92638\_at,92926\_at,94264\_at,94483\_at,94505\_at,95348\_at,96147\_at,96513\_at,96600\_at,96771\_at,98756\_at,100951\_at,102292\_at,94809\_at,97375\_at |
| 6 | DNA replication and chromosome cycle | 5 | 113 | 880 | 9498 | 0.006 | 0.012 | 0.477 | 0.983 | 100307\_at,101930\_at,103500\_at,160603\_at,95613\_at |
| 7 | DNA replication | 5 | 94 | 538 | 6246 | 0.009 | 0.015 | 0.617 | 0.917 | 100307\_at,101930\_at,103500\_at,160603\_at,95613\_at |
| 6 | M phase | 5 | 74 | 880 | 9498 | 0.006 | 0.008 | 0.729 | 0.828 | 104735\_at,160127\_at,98478\_at,99187\_f\_at,99188\_at |
| 7 | M phase of mitotic cell cycle | 5 | 57 | 538 | 6246 | 0.009 | 0.009 | 1.018 | 0.552 | 104735\_at,160127\_at,98478\_at,99187\_f\_at,99188\_at |
| 8 | mitosis | 5 | 57 | 199 | 2164 | 0.025 | 0.026 | 0.954 | 0.614 | 104735\_at,160127\_at,98478\_at,99187\_f\_at,99188\_at |
| 6 | mitotic cell cycle | 6 | 173 | 880 | 9498 | 0.007 | 0.018 | 0.375 | 0.999 | 95917\_at,104735\_at,160127\_at,98478\_at,99187\_f\_at,99188\_at |
| 7 | M phase of mitotic cell cycle | 5 | 57 | 538 | 6246 | 0.009 | 0.009 | 1.018 | 0.552 | 104735\_at,160127\_at,98478\_at,99187\_f\_at,99188\_at |
| 8 | mitosis | 5 | 57 | 199 | 2164 | 0.025 | 0.026 | 0.954 | 0.614 | 104735\_at,160127\_at,98478\_at,99187\_f\_at,99188\_at |
| 6 | regulation of cell cycle | 32 | 204 | 880 | 9498 | 0.036 | 0.021 | 1.693 | 0.002 | 100130\_at,102364\_at,102752\_at,102920\_at,103990\_at,160127\_at,160901\_at,92638\_at,92770\_at,92926\_at,94264\_at,94483\_at,94505\_at,94820\_r\_at,95348\_at,96147\_at,96513\_at,96600\_at,96771\_at,97504\_at,98478\_at,98756\_at,99187\_f\_at,99188\_at,100951\_at,102292\_at,94809\_at,94881\_at,94882\_at,95471\_at,97375\_at,98067\_at |
| 7 | cell cycle arrest | 8 | 20 | 538 | 6246 | 0.015 | 0.003 | 4.647 | 0 | 100951\_at,102292\_at,94809\_at,94881\_at,94882\_at,95471\_at,97375\_at,98067\_at |
| 5 | cytokinesis | 3 | 5 | 1042 | 11544 | 0.003 | 0 | 6.698 | 0.006 | 100088\_at,104735\_at,95033\_at |
| 5 | regulation of cell proliferation | 9 | 38 | 1042 | 11544 | 0.009 | 0.003 | 2.626 | 0.006 | 104149\_at,94331\_at,96147\_at,101457\_at,160301\_at,93104\_at,94809\_at,96488\_at,96489\_at |
| 6 | negative regulation of cell proliferation | 6 | 17 | 880 | 9498 | 0.007 | 0.002 | 3.81 | 0.003 | 101457\_at,160301\_at,93104\_at,94809\_at,96488\_at,96489\_at |
| 5 | T-cell proliferation | 2 | 4 | 1042 | 11544 | 0.002 | 0 | 5.486 | 0.043 | 102794\_at,160511\_at |
| 4 | transport | 106 | 1083 | 1185 | 13100 | 0.089 | 0.083 | 1.082 | 0.201 | 100567\_at,100944\_at,101079\_at,101118\_at,101217\_at,101568\_at,101787\_f\_at,102658\_at,103035\_at,103065\_at,103376\_s\_at,103427\_at,103739\_at,103913\_at,104019\_at,104461\_at,104534\_at,104719\_at,160683\_at,160684\_at,161551\_f\_at,161689\_f\_at,161696\_f\_at,161990\_f\_at,92392\_at,92847\_s\_at,93316\_at,93320\_at,93414\_at,93626\_at,93705\_at,94031\_at,94556\_at,94657\_at,95377\_at,95444\_at,95586\_at,95695\_at,96019\_at,96076\_at,96079\_at,96669\_at,96725\_at,96876\_at,97181\_f\_at,97458\_at,97812\_at,99500\_at,92648\_at,160124\_r\_at,160202\_at,94043\_at,96951\_at,96945\_at,100499\_at,100635\_at,101934\_at,102750\_at,103062\_at,104179\_at,160082\_s\_at,160149\_at,161026\_s\_at,92185\_at,92805\_s\_at,94319\_at,94505\_at,96530\_at,97058\_f\_at,97224\_at,98927\_at,99350\_at,99354\_s\_at,99358\_at,101023\_f\_at,104453\_at,92256\_at,95023\_at,96736\_at,160373\_i\_at,104149\_at,100951\_at,103686\_at,94254\_at,94255\_g\_at,94256\_at,94464\_at,94465\_g\_at,95655\_at,162041\_f\_at,93471\_at,103812\_at,102854\_s\_at,103935\_at,97375\_at,96186\_at,96534\_at,98114\_at,92531\_at,160795\_at,98926\_at,103258\_at,103959\_at,104522\_at,93063\_at,97551\_at |
| 5 | amine/polyamine transport | 2 | 19 | 1042 | 11544 | 0.002 | 0.002 | 1.164 | 0.522 | 104719\_at,99500\_at |
| 6 | amino acid transport | 2 | 19 | 880 | 9498 | 0.002 | 0.002 | 1.135 | 0.537 | 104719\_at,99500\_at |
| 5 | carbohydrate transport | 1 | 28 | 1042 | 11544 | 0.001 | 0.002 | 0.395 | 0.929 | 92648\_at |
| 6 | monosaccharide transport | 1 | 13 | 880 | 9498 | 0.001 | 0.001 | 0.832 | 0.718 | 92648\_at |
| 7 | hexose transport | 1 | 13 | 538 | 6246 | 0.002 | 0.002 | 0.894 | 0.69 | 92648\_at |
| 8 | glucose transport | 1 | 13 | 199 | 2164 | 0.005 | 0.006 | 0.837 | 0.716 | 92648\_at |
| 5 | gas transport | 1 | 10 | 1042 | 11544 | 0.001 | 0.001 | 1.103 | 0.612 | 97181\_f\_at |
| 6 | oxygen transport | 1 | 10 | 880 | 9498 | 0.001 | 0.001 | 1.086 | 0.622 | 97181\_f\_at |
| 5 | hydrogen transport | 4 | 50 | 1042 | 11544 | 0.004 | 0.004 | 0.887 | 0.673 | 160124\_r\_at,160202\_at,94043\_at,96951\_at |
| 6 | proton transport | 4 | 44 | 880 | 9498 | 0.005 | 0.005 | 0.983 | 0.592 | 160124\_r\_at,160202\_at,94043\_at,96951\_at |
| 5 | intracellular transport | 39 | 351 | 1042 | 11544 | 0.037 | 0.03 | 1.231 | 0.101 | 96945\_at,100499\_at,100635\_at,101934\_at,102750\_at,103062\_at,103913\_at,104179\_at,160082\_s\_at,160149\_at,161026\_s\_at,92185\_at,92648\_at,92805\_s\_at,92847\_s\_at,94031\_at,94319\_at,94505\_at,94556\_at,95444\_at,96076\_at,96530\_at,96669\_at,97058\_f\_at,97224\_at,97458\_at,97812\_at,98927\_at,99350\_at,99354\_s\_at,99358\_at,101023\_f\_at,104453\_at,92256\_at,95023\_at,96736\_at,160373\_i\_at,101079\_at,104149\_at |
| 6 | Golgi vesicle transport | 1 | 12 | 880 | 9498 | 0.001 | 0.001 | 0.905 | 0.689 | 96945\_at |
| 7 | post-Golgi transport | 1 | 5 | 538 | 6246 | 0.002 | 0.001 | 2.325 | 0.363 | 96945\_at |
| 6 | intracellular protein transport | 39 | 284 | 880 | 9498 | 0.044 | 0.03 | 1.482 | 0.008 | 100499\_at,100635\_at,101934\_at,102750\_at,103062\_at,103913\_at,104179\_at,160082\_s\_at,160149\_at,161026\_s\_at,92185\_at,92648\_at,92805\_s\_at,92847\_s\_at,94031\_at,94319\_at,94505\_at,94556\_at,95444\_at,96076\_at,96530\_at,96669\_at,96945\_at,97058\_f\_at,97224\_at,97458\_at,97812\_at,98927\_at,99350\_at,99354\_s\_at,99358\_at,101023\_f\_at,104453\_at,92256\_at,95023\_at,96736\_at,160373\_i\_at,101079\_at,104149\_at |
| 7 | protein targeting | 12 | 101 | 538 | 6246 | 0.022 | 0.016 | 1.379 | 0.158 | 101023\_f\_at,103913\_at,104453\_at,92256\_at,95023\_at,95444\_at,96669\_at,96736\_at,160373\_i\_at,101079\_at,97458\_at,104149\_at |
| 8 | protein-ER targeting | 1 | 6 | 199 | 2164 | 0.005 | 0.003 | 1.816 | 0.44 | 160373\_i\_at |
| 9 | cotranslational membrane targeting | 1 | 3 | 72 | 911 | 0.014 | 0.003 | 4.222 | 0.219 | 160373\_i\_at |
| 8 | protein-nucleus import | 3 | 32 | 199 | 2164 | 0.015 | 0.015 | 1.02 | 0.576 | 101079\_at,97458\_at,104149\_at |
| 9 | protein-nucleus import, translocation | 1 | 4 | 72 | 911 | 0.014 | 0.004 | 3.164 | 0.281 | 104149\_at |
| 6 | nucleocytoplasmic transport | 3 | 40 | 880 | 9498 | 0.003 | 0.004 | 0.81 | 0.73 | 101079\_at,97458\_at,104149\_at |
| 7 | RNA-nucleus export | 1 | 7 | 538 | 6246 | 0.002 | 0.001 | 1.661 | 0.468 | 101079\_at |
| 8 | mRNA-nucleus export | 1 | 4 | 199 | 2164 | 0.005 | 0.002 | 2.719 | 0.32 | 101079\_at |
| 5 | ion transport | 28 | 335 | 1042 | 11544 | 0.027 | 0.029 | 0.926 | 0.696 | 100951\_at,101217\_at,101787\_f\_at,103686\_at,104461\_at,104719\_at,93705\_at,94254\_at,94255\_g\_at,94256\_at,94464\_at,94465\_g\_at,94657\_at,95377\_at,95586\_at,95655\_at,99500\_at,162041\_f\_at,93471\_at,103812\_at,103065\_at,96079\_at,100944\_at,102854\_s\_at,103935\_at,97375\_at,101118\_at,104019\_at |
| 6 | anion transport | 17 | 79 | 880 | 9498 | 0.019 | 0.008 | 2.322 | 0.001 | 162041\_f\_at,93471\_at,101217\_at,103812\_at,104719\_at,94254\_at,94255\_g\_at,94256\_at,94464\_at,94465\_g\_at,94657\_at,95377\_at,95655\_at,99500\_at,103065\_at,96079\_at,100944\_at |
| 7 | inorganic anion transport | 14 | 50 | 538 | 6246 | 0.026 | 0.008 | 3.248 | 0 | 101217\_at,103812\_at,104719\_at,94254\_at,94255\_g\_at,94256\_at,94464\_at,94465\_g\_at,94657\_at,95377\_at,95655\_at,99500\_at,103065\_at,96079\_at |
| 8 | chloride transport | 12 | 39 | 199 | 2164 | 0.06 | 0.018 | 3.346 | 0 | 101217\_at,103812\_at,104719\_at,94254\_at,94255\_g\_at,94256\_at,94464\_at,94465\_g\_at,94657\_at,95377\_at,95655\_at,99500\_at |
| 8 | phosphate transport | 2 | 6 | 199 | 2164 | 0.01 | 0.003 | 3.628 | 0.099 | 103065\_at,96079\_at |
| 7 | organic anion transport | 1 | 15 | 538 | 6246 | 0.002 | 0.002 | 0.775 | 0.741 | 100944\_at |
| 8 | dicarboxylic acid transport | 1 | 7 | 199 | 2164 | 0.005 | 0.003 | 1.557 | 0.491 | 100944\_at |
| 6 | cation transport | 12 | 236 | 880 | 9498 | 0.014 | 0.025 | 0.549 | 0.994 | 100951\_at,101787\_f\_at,102854\_s\_at,103686\_at,103935\_at,104461\_at,97375\_at,101118\_at,104719\_at,99500\_at,104019\_at,96079\_at |
| 7 | di-, tri-valent inorganic cation transport | 6 | 58 | 538 | 6246 | 0.011 | 0.009 | 1.2 | 0.383 | 100951\_at,103935\_at,104461\_at,97375\_at,102854\_s\_at,101118\_at |
| 8 | calcium ion transport | 4 | 33 | 199 | 2164 | 0.02 | 0.015 | 1.318 | 0.361 | 100951\_at,103935\_at,104461\_at,97375\_at |
| 8 | transition metal ion transport | 2 | 25 | 199 | 2164 | 0.01 | 0.012 | 0.87 | 0.685 | 102854\_s\_at,101118\_at |
| 9 | copper ion transport | 1 | 5 | 72 | 911 | 0.014 | 0.005 | 2.53 | 0.338 | 102854\_s\_at |
| 9 | iron ion transport | 1 | 11 | 72 | 911 | 0.014 | 0.012 | 1.151 | 0.598 | 101118\_at |
| 7 | metal ion transport | 11 | 184 | 538 | 6246 | 0.02 | 0.029 | 0.694 | 0.929 | 102854\_s\_at,100951\_at,103935\_at,104461\_at,97375\_at,101118\_at,101787\_f\_at,104719\_at,99500\_at,104019\_at,96079\_at |
| 8 | calcium ion transport | 4 | 33 | 199 | 2164 | 0.02 | 0.015 | 1.318 | 0.361 | 100951\_at,103935\_at,104461\_at,97375\_at |
| 8 | transition metal ion transport | 2 | 25 | 199 | 2164 | 0.01 | 0.012 | 0.87 | 0.685 | 102854\_s\_at,101118\_at |
| 9 | copper ion transport | 1 | 5 | 72 | 911 | 0.014 | 0.005 | 2.53 | 0.338 | 102854\_s\_at |
| 9 | iron ion transport | 1 | 11 | 72 | 911 | 0.014 | 0.012 | 1.151 | 0.598 | 101118\_at |
| 8 | potassium ion transport | 3 | 94 | 199 | 2164 | 0.015 | 0.043 | 0.347 | 0.994 | 101787\_f\_at,104719\_at,99500\_at |
| 8 | sodium ion transport | 4 | 43 | 199 | 2164 | 0.02 | 0.02 | 1.012 | 0.568 | 104019\_at,104719\_at,96079\_at,99500\_at |
| 5 | lipid transport | 4 | 40 | 1042 | 11544 | 0.004 | 0.003 | 1.107 | 0.493 | 93316\_at,96186\_at,96534\_at,98114\_at |
| 6 | sterol transport | 1 | 3 | 880 | 9498 | 0.001 | 0 | 3.562 | 0.253 | 98114\_at |
| 7 | cholesterol transport | 1 | 3 | 538 | 6246 | 0.002 | 0 | 3.875 | 0.237 | 98114\_at |
| 5 | neurotransmitter transport | 2 | 21 | 1042 | 11544 | 0.002 | 0.002 | 1.055 | 0.577 | 100499\_at,161696\_f\_at |
| 5 | peptide transport | 1 | 5 | 1042 | 11544 | 0.001 | 0 | 2.233 | 0.377 | 103035\_at |
| 6 | oligopeptide transport | 1 | 5 | 880 | 9498 | 0.001 | 0.001 | 2.151 | 0.385 | 103035\_at |
| 5 | protein transport | 40 | 297 | 1042 | 11544 | 0.038 | 0.026 | 1.492 | 0.007 | 100635\_at,102750\_at,103062\_at,103913\_at,104179\_at,160082\_s\_at,92531\_at,92648\_at,94031\_at,94319\_at,94505\_at,94556\_at,95444\_at,96669\_at,96945\_at,97058\_f\_at,97224\_at,97458\_at,97812\_at,98927\_at,99350\_at,99354\_s\_at,99358\_at,100499\_at,101934\_at,160149\_at,161026\_s\_at,92185\_at,92805\_s\_at,92847\_s\_at,96076\_at,96530\_at,101023\_f\_at,104453\_at,92256\_at,95023\_at,96736\_at,160373\_i\_at,101079\_at,104149\_at |
| 6 | intracellular protein transport | 39 | 284 | 880 | 9498 | 0.044 | 0.03 | 1.482 | 0.008 | 100499\_at,100635\_at,101934\_at,102750\_at,103062\_at,103913\_at,104179\_at,160082\_s\_at,160149\_at,161026\_s\_at,92185\_at,92648\_at,92805\_s\_at,92847\_s\_at,94031\_at,94319\_at,94505\_at,94556\_at,95444\_at,96076\_at,96530\_at,96669\_at,96945\_at,97058\_f\_at,97224\_at,97458\_at,97812\_at,98927\_at,99350\_at,99354\_s\_at,99358\_at,101023\_f\_at,104453\_at,92256\_at,95023\_at,96736\_at,160373\_i\_at,101079\_at,104149\_at |
| 7 | protein targeting | 12 | 101 | 538 | 6246 | 0.022 | 0.016 | 1.379 | 0.158 | 101023\_f\_at,103913\_at,104453\_at,92256\_at,95023\_at,95444\_at,96669\_at,96736\_at,160373\_i\_at,101079\_at,97458\_at,104149\_at |
| 8 | protein-ER targeting | 1 | 6 | 199 | 2164 | 0.005 | 0.003 | 1.816 | 0.44 | 160373\_i\_at |
| 9 | cotranslational membrane targeting | 1 | 3 | 72 | 911 | 0.014 | 0.003 | 4.222 | 0.219 | 160373\_i\_at |
| 8 | protein-nucleus import | 3 | 32 | 199 | 2164 | 0.015 | 0.015 | 1.02 | 0.576 | 101079\_at,97458\_at,104149\_at |
| 9 | protein-nucleus import, translocation | 1 | 4 | 72 | 911 | 0.014 | 0.004 | 3.164 | 0.281 | 104149\_at |
| 5 | secretory pathway | 4 | 31 | 1042 | 11544 | 0.004 | 0.003 | 1.428 | 0.305 | 160373\_i\_at,96945\_at,160795\_at,98926\_at |
| 6 | exocytosis | 3 | 12 | 880 | 9498 | 0.003 | 0.001 | 2.706 | 0.093 | 160795\_at,98926\_at,96945\_at |
| 7 | calcium ion dependent exocytosis | 1 | 3 | 538 | 6246 | 0.002 | 0 | 3.875 | 0.237 | 98926\_at |
| 7 | nonselective vesicle exocytosis | 1 | 2 | 538 | 6246 | 0.002 | 0 | 5.812 | 0.165 | 96945\_at |
| 8 | nonselective vesicle targeting | 1 | 1 | 199 | 2164 | 0.005 | 0 | 10.935 | 0.092 | 96945\_at |
| 7 | regulation of exocytosis | 1 | 2 | 538 | 6246 | 0.002 | 0 | 5.812 | 0.165 | 98926\_at |
| 5 | vesicle-mediated transport | 12 | 112 | 1042 | 11544 | 0.012 | 0.01 | 1.188 | 0.31 | 96076\_at,96945\_at,160795\_at,98926\_at,103258\_at,103959\_at,104522\_at,93063\_at,96534\_at,97551\_at,96736\_at,104179\_at |
| 6 | exocytosis | 3 | 12 | 880 | 9498 | 0.003 | 0.001 | 2.706 | 0.093 | 160795\_at,98926\_at,96945\_at |
| 7 | calcium ion dependent exocytosis | 1 | 3 | 538 | 6246 | 0.002 | 0 | 3.875 | 0.237 | 98926\_at |
| 7 | nonselective vesicle exocytosis | 1 | 2 | 538 | 6246 | 0.002 | 0 | 5.812 | 0.165 | 96945\_at |
| 8 | nonselective vesicle targeting | 1 | 1 | 199 | 2164 | 0.005 | 0 | 10.935 | 0.092 | 96945\_at |
| 7 | regulation of exocytosis | 1 | 2 | 538 | 6246 | 0.002 | 0 | 5.812 | 0.165 | 98926\_at |
| 6 | endocytosis | 7 | 61 | 880 | 9498 | 0.008 | 0.006 | 1.238 | 0.335 | 103258\_at,103959\_at,104522\_at,93063\_at,96534\_at,97551\_at,96736\_at |
| 7 | receptor mediated endocytosis | 1 | 9 | 538 | 6246 | 0.002 | 0.001 | 1.292 | 0.556 | 97551\_at |
| 7 | regulation of endocytosis | 1 | 7 | 538 | 6246 | 0.002 | 0.001 | 1.661 | 0.468 | 96736\_at |
| 6 | nonselective vesicle transport | 1 | 7 | 880 | 9498 | 0.001 | 0.001 | 1.541 | 0.494 | 104179\_at |
| 3 | cell motility | 9 | 188 | 1040 | 10726 | 0.009 | 0.018 | 0.493 | 0.996 | 96278\_at,103693\_at,103699\_i\_at,102794\_at,160511\_at,161184\_f\_at,95489\_at,160151\_i\_at,94004\_at |
| 4 | cell migration | 5 | 53 | 1185 | 13100 | 0.004 | 0.004 | 1.042 | 0.53 | 103693\_at,103699\_i\_at,102794\_at,160511\_at,161184\_f\_at |
| 5 | axon guidance | 2 | 23 | 1042 | 11544 | 0.002 | 0.002 | 0.965 | 0.628 | 103693\_at,103699\_i\_at |
| 5 | regulation of cell migration | 3 | 17 | 1042 | 11544 | 0.003 | 0.001 | 1.959 | 0.194 | 102794\_at,160511\_at,161184\_f\_at |
| 6 | negative regulation of cell migration | 1 | 3 | 880 | 9498 | 0.001 | 0 | 3.562 | 0.253 | 161184\_f\_at |
| 4 | muscle contraction | 3 | 54 | 1185 | 13100 | 0.003 | 0.004 | 0.614 | 0.878 | 95489\_at,160151\_i\_at,94004\_at |
| 5 | smooth muscle contraction | 2 | 3 | 1042 | 11544 | 0.002 | 0 | 7.385 | 0.023 | 160151\_i\_at,94004\_at |
| 3 | membrane fusion | 2 | 4 | 1040 | 10726 | 0.002 | 0 | 5.189 | 0.049 | 96945\_at,161184\_f\_at |
| 4 | plasma membrane fusion | 1 | 3 | 1185 | 13100 | 0.001 | 0 | 3.652 | 0.248 | 161184\_f\_at |
| 2 | development | 86 | 990 | 1057 | 10540 | 0.081 | 0.094 | 0.866 | 0.939 | 100992\_at,102356\_at,102381\_at,102644\_at,103086\_at,103328\_at,103518\_at,103693\_at,103699\_i\_at,103736\_at,160483\_at,160603\_at,160857\_at,93459\_s\_at,93550\_at,94473\_at,95489\_at,97118\_at,97498\_at,99364\_at,99366\_at,99416\_at,99529\_f\_at,100514\_at,101457\_at,104761\_at,160301\_at,160464\_s\_at,161610\_at,161666\_f\_at,92653\_at,94752\_s\_at,94809\_at,96088\_at,96488\_at,96489\_at,96596\_at,97973\_at,92925\_at,99475\_at,99532\_at,103531\_f\_at,160726\_at,160727\_at,96633\_s\_at,96852\_at,95917\_at,104576\_at,92778\_i\_at,92780\_f\_at,104725\_at,96299\_at,101186\_at,102224\_at,102983\_at,102984\_g\_at,160440\_at,100134\_at,160469\_at,161184\_f\_at,160430\_at,96771\_at,97375\_at,102032\_at,92638\_at,102940\_at,98427\_s\_at,92542\_at,92248\_at,92249\_g\_at,101475\_at,102794\_at,160511\_at,160099\_at,104263\_at,97519\_at,103362\_at,100024\_at,100951\_at,160834\_at,100522\_s\_at,100523\_r\_at,100533\_s\_at,101515\_at,160526\_s\_at,92888\_s\_at |
| 3 | cell differentiation | 24 | 137 | 1040 | 10726 | 0.023 | 0.013 | 1.807 | 0.003 | 100514\_at,101457\_at,104761\_at,160301\_at,160464\_s\_at,161610\_at,161666\_f\_at,92653\_at,93550\_at,94752\_s\_at,94809\_at,96088\_at,96488\_at,96489\_at,96596\_at,97118\_at,97498\_at,97973\_at,92925\_at,99475\_at,99532\_at,103531\_f\_at,160726\_at,160727\_at |
| 4 | cell fate commitment | 1 | 13 | 1185 | 13100 | 0.001 | 0.001 | 0.848 | 0.709 | 97973\_at |
| 4 | keratinocyte differentiation | 1 | 3 | 1185 | 13100 | 0.001 | 0 | 3.652 | 0.248 | 94809\_at |
| 4 | myeloid blood cell differentiation | 1 | 7 | 1185 | 13100 | 0.001 | 0.001 | 1.585 | 0.485 | 97973\_at |
| 5 | erythrocyte differentiation | 1 | 3 | 1042 | 11544 | 0.001 | 0 | 3.692 | 0.247 | 97973\_at |
| 4 | neuron differentiation | 2 | 5 | 1185 | 13100 | 0.002 | 0 | 4.447 | 0.068 | 92925\_at,99475\_at |
| 5 | regulation of neuron differentiation | 1 | 1 | 1042 | 11544 | 0.001 | 0 | 10.667 | 0.09 | 99475\_at |
| 6 | positive regulation of neuron differentiation | 1 | 1 | 880 | 9498 | 0.001 | 0 | 10.364 | 0.093 | 99475\_at |
| 4 | osteoblast differentiation | 1 | 1 | 1185 | 13100 | 0.001 | 0 | 10.5 | 0.09 | 99532\_at |
| 5 | regulation of osteoblast differentiation | 1 | 1 | 1042 | 11544 | 0.001 | 0 | 10.667 | 0.09 | 99532\_at |
| 6 | negative regulation of osteoblast differentiation | 1 | 1 | 880 | 9498 | 0.001 | 0 | 10.364 | 0.093 | 99532\_at |
| 4 | spermatid development | 1 | 11 | 1185 | 13100 | 0.001 | 0.001 | 1 | 0.648 | 103531\_f\_at |
| 4 | vasculogenesis | 2 | 7 | 1185 | 13100 | 0.002 | 0.001 | 3.189 | 0.127 | 160726\_at,160727\_at |
| 3 | aging | 1 | 4 | 1040 | 10726 | 0.001 | 0 | 2.595 | 0.335 | 96633\_s\_at |
| 3 | embryonic development | 5 | 52 | 1040 | 10726 | 0.005 | 0.005 | 0.992 | 0.577 | 96852\_at,95917\_at,104576\_at,92778\_i\_at,92780\_f\_at |
| 4 | embryonic development (sensu Animalia) | 2 | 18 | 1185 | 13100 | 0.002 | 0.001 | 1.234 | 0.494 | 96852\_at,95917\_at |
| 5 | gastrulation | 2 | 12 | 1042 | 11544 | 0.002 | 0.001 | 1.846 | 0.296 | 96852\_at,95917\_at |
| 6 | formation of primary germ layer | 1 | 4 | 880 | 9498 | 0.001 | 0 | 2.714 | 0.322 | 96852\_at |
| 7 | mesoderm formation | 1 | 4 | 538 | 6246 | 0.002 | 0.001 | 2.906 | 0.303 | 96852\_at |
| 6 | gastrulation (sensu Deuterostoma) | 1 | 3 | 880 | 9498 | 0.001 | 0 | 3.562 | 0.253 | 95917\_at |
| 7 | gastrulation (sensu Mammalia) | 1 | 3 | 538 | 6246 | 0.002 | 0 | 3.875 | 0.237 | 95917\_at |
| 4 | embryonic morphogenesis | 1 | 19 | 1185 | 13100 | 0.001 | 0.001 | 0.579 | 0.835 | 104576\_at |
| 5 | limb morphogenesis | 1 | 13 | 1042 | 11544 | 0.001 | 0.001 | 0.85 | 0.708 | 104576\_at |
| 4 | embryonic pattern specification | 2 | 7 | 1185 | 13100 | 0.002 | 0.001 | 3.189 | 0.127 | 92778\_i\_at,92780\_f\_at |
| 5 | patterning of blood vessels | 2 | 3 | 1042 | 11544 | 0.002 | 0 | 7.385 | 0.023 | 92778\_i\_at,92780\_f\_at |
| 3 | growth | 1 | 8 | 1040 | 10726 | 0.001 | 0.001 | 1.28 | 0.558 | 99475\_at |
| 4 | regulation of growth | 1 | 8 | 1185 | 13100 | 0.001 | 0.001 | 1.377 | 0.532 | 99475\_at |
| 5 | regulation of body size | 1 | 5 | 1042 | 11544 | 0.001 | 0 | 2.233 | 0.377 | 99475\_at |
| 3 | morphogenesis | 47 | 594 | 1040 | 10726 | 0.045 | 0.055 | 0.816 | 0.946 | 100514\_at,104725\_at,96299\_at,104576\_at,101186\_at,102224\_at,102381\_at,102644\_at,102983\_at,102984\_g\_at,103086\_at,103736\_at,160440\_at,160603\_at,96852\_at,160726\_at,160727\_at,100134\_at,92778\_i\_at,92780\_f\_at,160469\_at,161184\_f\_at,103531\_f\_at,160430\_at,96771\_at,97375\_at,102032\_at,97973\_at,92638\_at,102940\_at,98427\_s\_at,92542\_at,97498\_at,103693\_at,103699\_i\_at,160857\_at,92248\_at,92249\_g\_at,92653\_at,101475\_at,102794\_at,160511\_at,160099\_at,104263\_at,97519\_at,99532\_at,103362\_at |
| 4 | embryonic morphogenesis | 1 | 19 | 1185 | 13100 | 0.001 | 0.001 | 0.579 | 0.835 | 104576\_at |
| 5 | limb morphogenesis | 1 | 13 | 1042 | 11544 | 0.001 | 0.001 | 0.85 | 0.708 | 104576\_at |
| 4 | organogenesis | 44 | 544 | 1185 | 13100 | 0.037 | 0.042 | 0.894 | 0.807 | 101186\_at,102224\_at,102381\_at,102644\_at,102983\_at,102984\_g\_at,103086\_at,103736\_at,160440\_at,160603\_at,96852\_at,160726\_at,160727\_at,100134\_at,100514\_at,92778\_i\_at,92780\_f\_at,160469\_at,161184\_f\_at,103531\_f\_at,160430\_at,96771\_at,97375\_at,102032\_at,97973\_at,92638\_at,102940\_at,98427\_s\_at,92542\_at,97498\_at,103693\_at,103699\_i\_at,160857\_at,92248\_at,92249\_g\_at,92653\_at,101475\_at,102794\_at,160511\_at,160099\_at,104263\_at,97519\_at,99532\_at,103362\_at |
| 5 | blood vessel development | 8 | 61 | 1042 | 11544 | 0.008 | 0.005 | 1.455 | 0.182 | 160726\_at,160727\_at,100134\_at,100514\_at,92778\_i\_at,92780\_f\_at,160469\_at,161184\_f\_at |
| 6 | angiogenesis | 6 | 50 | 880 | 9498 | 0.007 | 0.005 | 1.297 | 0.316 | 100134\_at,100514\_at,92778\_i\_at,92780\_f\_at,160469\_at,161184\_f\_at |
| 7 | regulation of angiogenesis | 2 | 11 | 538 | 6246 | 0.004 | 0.002 | 2.114 | 0.244 | 160469\_at,161184\_f\_at |
| 8 | negative regulation of angiogenesis | 2 | 8 | 199 | 2164 | 0.01 | 0.004 | 2.716 | 0.163 | 160469\_at,161184\_f\_at |
| 5 | patterning of blood vessels | 2 | 3 | 1042 | 11544 | 0.002 | 0 | 7.385 | 0.023 | 92778\_i\_at,92780\_f\_at |
| 5 | gonad development | 1 | 5 | 1042 | 11544 | 0.001 | 0 | 2.233 | 0.377 | 103531\_f\_at |
| 6 | female gonad development | 1 | 4 | 880 | 9498 | 0.001 | 0 | 2.714 | 0.322 | 103531\_f\_at |
| 7 | ovarian follicle development | 1 | 1 | 538 | 6246 | 0.002 | 0 | 11.625 | 0.086 | 103531\_f\_at |
| 5 | heart development | 5 | 31 | 1042 | 11544 | 0.005 | 0.003 | 1.784 | 0.143 | 100134\_at,160430\_at,160603\_at,96771\_at,97375\_at |
| 5 | hemopoiesis | 2 | 22 | 1042 | 11544 | 0.002 | 0.002 | 1.005 | 0.603 | 102032\_at,97973\_at |
| 5 | erythrocyte differentiation | 1 | 3 | 1042 | 11544 | 0.001 | 0 | 3.692 | 0.247 | 97973\_at |
| 5 | histogenesis | 2 | 24 | 1042 | 11544 | 0.002 | 0.002 | 0.923 | 0.651 | 92638\_at,96852\_at |
| 6 | mesoderm development | 2 | 9 | 880 | 9498 | 0.002 | 0.001 | 2.389 | 0.2 | 92638\_at,96852\_at |
| 7 | mesoderm formation | 1 | 4 | 538 | 6246 | 0.002 | 0.001 | 2.906 | 0.303 | 96852\_at |
| 5 | lymph gland development | 2 | 20 | 1042 | 11544 | 0.002 | 0.002 | 1.11 | 0.55 | 102940\_at,98427\_s\_at |
| 5 | midgut development | 1 | 1 | 1042 | 11544 | 0.001 | 0 | 10.667 | 0.09 | 101186\_at |
| 6 | visceral mesoderm/endoderm interaction | 1 | 1 | 880 | 9498 | 0.001 | 0 | 10.364 | 0.093 | 101186\_at |
| 5 | muscle development | 2 | 75 | 1042 | 11544 | 0.002 | 0.006 | 0.295 | 0.993 | 92542\_at,97498\_at |
| 5 | neurogenesis | 14 | 164 | 1042 | 11544 | 0.013 | 0.014 | 0.946 | 0.627 | 103693\_at,103699\_i\_at,160857\_at,92248\_at,92249\_g\_at,92653\_at,101475\_at,102794\_at,160511\_at,160603\_at,160726\_at,160727\_at,96771\_at,160099\_at |
| 6 | central nervous system development | 4 | 39 | 880 | 9498 | 0.005 | 0.004 | 1.107 | 0.494 | 101475\_at,102794\_at,160511\_at,160603\_at |
| 7 | brain development | 4 | 29 | 538 | 6246 | 0.007 | 0.005 | 1.601 | 0.237 | 101475\_at,102794\_at,160511\_at,160603\_at |
| 6 | nerve maturation | 2 | 10 | 880 | 9498 | 0.002 | 0.001 | 2.162 | 0.236 | 160726\_at,160727\_at |
| 7 | nerve ensheathment | 2 | 10 | 538 | 6246 | 0.004 | 0.002 | 2.325 | 0.211 | 160726\_at,160727\_at |
| 6 | peripheral nervous system development | 2 | 24 | 880 | 9498 | 0.002 | 0.003 | 0.897 | 0.666 | 96771\_at,160099\_at |
| 7 | sensory organ development | 1 | 16 | 538 | 6246 | 0.002 | 0.003 | 0.727 | 0.764 | 160099\_at |
| 5 | skeletal development | 7 | 52 | 1042 | 11544 | 0.007 | 0.004 | 1.493 | 0.185 | 101475\_at,104263\_at,97375\_at,102032\_at,97519\_at,99532\_at,103362\_at |
| 6 | cartilage condensation | 1 | 5 | 880 | 9498 | 0.001 | 0.001 | 2.151 | 0.385 | 97375\_at |
| 6 | ossification | 5 | 25 | 880 | 9498 | 0.006 | 0.003 | 2.16 | 0.076 | 102032\_at,97519\_at,99532\_at,104263\_at,103362\_at |
| 7 | bone mineralization | 1 | 4 | 538 | 6246 | 0.002 | 0.001 | 2.906 | 0.303 | 104263\_at |
| 7 | regulation of bone formation | 1 | 2 | 538 | 6246 | 0.002 | 0 | 5.812 | 0.165 | 103362\_at |
| 6 | negative regulation of osteoblast differentiation | 1 | 1 | 880 | 9498 | 0.001 | 0 | 10.364 | 0.093 | 99532\_at |
| 5 | regulation of osteoblast differentiation | 1 | 1 | 1042 | 11544 | 0.001 | 0 | 10.667 | 0.09 | 99532\_at |
| 6 | negative regulation of osteoblast differentiation | 1 | 1 | 880 | 9498 | 0.001 | 0 | 10.364 | 0.093 | 99532\_at |
| 4 | vasculogenesis | 2 | 7 | 1185 | 13100 | 0.002 | 0.001 | 3.189 | 0.127 | 160726\_at,160727\_at |
| 4 | myeloid blood cell differentiation | 1 | 7 | 1185 | 13100 | 0.001 | 0.001 | 1.585 | 0.485 | 97973\_at |
| 5 | erythrocyte differentiation | 1 | 3 | 1042 | 11544 | 0.001 | 0 | 3.692 | 0.247 | 97973\_at |
| 4 | osteoblast differentiation | 1 | 1 | 1185 | 13100 | 0.001 | 0 | 10.5 | 0.09 | 99532\_at |
| 5 | regulation of osteoblast differentiation | 1 | 1 | 1042 | 11544 | 0.001 | 0 | 10.667 | 0.09 | 99532\_at |
| 6 | negative regulation of osteoblast differentiation | 1 | 1 | 880 | 9498 | 0.001 | 0 | 10.364 | 0.093 | 99532\_at |
| 3 | pattern specification | 8 | 83 | 1040 | 10726 | 0.008 | 0.008 | 0.994 | 0.562 | 100024\_at,102381\_at,102644\_at,103086\_at,92778\_i\_at,92780\_f\_at,95917\_at,100951\_at |
| 4 | embryonic pattern specification | 2 | 7 | 1185 | 13100 | 0.002 | 0.001 | 3.189 | 0.127 | 92778\_i\_at,92780\_f\_at |
| 5 | patterning of blood vessels | 2 | 3 | 1042 | 11544 | 0.002 | 0 | 7.385 | 0.023 | 92778\_i\_at,92780\_f\_at |
| 4 | anterior/posterior pattern formation | 1 | 10 | 1185 | 13100 | 0.001 | 0.001 | 1.105 | 0.613 | 95917\_at |
| 5 | anterior/posterior axis specification | 1 | 3 | 1042 | 11544 | 0.001 | 0 | 3.692 | 0.247 | 95917\_at |
| 4 | determination of symmetry | 1 | 6 | 1185 | 13100 | 0.001 | 0 | 1.826 | 0.434 | 100951\_at |
| 5 | determination of bilateral symmetry | 1 | 6 | 1042 | 11544 | 0.001 | 0.001 | 1.846 | 0.433 | 100951\_at |
| 6 | determination of left/right asymmetry | 1 | 6 | 880 | 9498 | 0.001 | 0.001 | 1.81 | 0.442 | 100951\_at |
| 3 | regulation of gene expression, epigenetic | 1 | 28 | 1040 | 10726 | 0.001 | 0.003 | 0.368 | 0.943 | 160834\_at |
| 4 | DNA methylation | 1 | 21 | 1185 | 13100 | 0.001 | 0.002 | 0.525 | 0.864 | 160834\_at |
| 3 | reproduction | 9 | 99 | 1040 | 10726 | 0.009 | 0.009 | 0.937 | 0.631 | 100522\_s\_at,100523\_r\_at,99529\_f\_at,96299\_at,100533\_s\_at,101515\_at,160526\_s\_at,92888\_s\_at,103531\_f\_at |
| 4 | sexual reproduction | 9 | 99 | 1185 | 13100 | 0.008 | 0.008 | 1.004 | 0.545 | 100522\_s\_at,100523\_r\_at,99529\_f\_at,96299\_at,100533\_s\_at,101515\_at,160526\_s\_at,92888\_s\_at,103531\_f\_at |
| 5 | fertilization | 2 | 14 | 1042 | 11544 | 0.002 | 0.001 | 1.587 | 0.365 | 100522\_s\_at,100523\_r\_at |
| 6 | fertilization (sensu Animalia) | 2 | 14 | 880 | 9498 | 0.002 | 0.001 | 1.544 | 0.377 | 100522\_s\_at,100523\_r\_at |
| 5 | gametogenesis | 7 | 86 | 1042 | 11544 | 0.007 | 0.007 | 0.902 | 0.668 | 99529\_f\_at,96299\_at,100533\_s\_at,101515\_at,160526\_s\_at,92888\_s\_at,103531\_f\_at |
| 6 | female gamete generation | 1 | 6 | 880 | 9498 | 0.001 | 0.001 | 1.81 | 0.442 | 96299\_at |
| 6 | male gamete generation | 5 | NA | 880 | 9498 | 0.006 | NA | NA | NA | 100533\_s\_at,101515\_at,160526\_s\_at,92888\_s\_at,103531\_f\_at |
| 7 | spermatogenesis | 5 | 66 | 538 | 6246 | 0.009 | 0.011 | 0.879 | 0.683 | 100533\_s\_at,101515\_at,160526\_s\_at,92888\_s\_at,103531\_f\_at |
| 4 | spermatid development | 1 | 11 | 1185 | 13100 | 0.001 | 0.001 | 1 | 0.648 | 103531\_f\_at |
| 2 | obsolete biological process | 7 | 3 | 1057 | 10540 | 0.007 | 0 | 23.643 | 0 | 101023\_f\_at,102094\_f\_at,160199\_at,160200\_at,93267\_at,93520\_at,96777\_at |
| 3 | mRNA splicing | 7 | 54 | 1040 | 10726 | 0.007 | 0.005 | 1.338 | 0.266 | 101023\_f\_at,102094\_f\_at,160199\_at,160200\_at,93267\_at,93520\_at,96777\_at |
| 2 | physiological processes | 585 | 5866 | 1057 | 10540 | 0.553 | 0.557 | 0.994 | 0.598 | 100130\_at,101030\_at,101475\_at,102364\_at,102920\_at,103990\_at,104477\_at,104576\_at,160829\_at,160901\_at,161551\_f\_at,92653\_at,92926\_at,93104\_at,93666\_at,93714\_f\_at,94264\_at,94448\_at,94505\_at,94752\_s\_at,95348\_at,95617\_at,95618\_at,96513\_at,96771\_at,96810\_at,97484\_at,97973\_at,98110\_at,98756\_at,99467\_at,101998\_at,102752\_at,97426\_at,97498\_at,103899\_at,162206\_f\_at,92778\_i\_at,92780\_f\_at,94809\_at,96633\_s\_at,99475\_at,92263\_at,92270\_at,98324\_at,104263\_at,100606\_at,101118\_at,103959\_at,160676\_at,94976\_at,100514\_at,104725\_at,96299\_at,96736\_at,101578\_f\_at,92542\_at,94270\_at,98968\_at,160065\_s\_at,95489\_at,98127\_at,94106\_at,96278\_at,99013\_f\_at,100342\_i\_at,160287\_at,160288\_at,160679\_at,161615\_f\_at,93729\_at,94835\_f\_at,95119\_at,98461\_at,98882\_s\_at,98884\_r\_at,160373\_i\_at,96945\_at,96779\_f\_at,97409\_at,101848\_g\_at,160399\_r\_at,93020\_at,162138\_s\_at,104376\_at,101015\_s\_at,102994\_at,104761\_at,92770\_at,94928\_at,96852\_at,102781\_at,103416\_at,104598\_at,160127\_at,160359\_at,160495\_at,94820\_r\_at,94881\_at,94882\_at,95471\_at,95805\_at,96728\_at,97504\_at,97844\_at,98067\_at,98478\_at,99187\_f\_at,99188\_at,99529\_f\_at,100307\_at,101930\_at,103500\_at,160603\_at,95613\_at,104735\_at,95917\_at,92638\_at,94483\_at,96147\_at,96600\_at,100951\_at,102292\_at,97375\_at,100088\_at,95033\_at,104149\_at,94331\_at,101457\_at,160301\_at,96488\_at,96489\_at,102794\_at,160511\_at,100567\_at,100944\_at,101079\_at,101217\_at,101568\_at,101787\_f\_at,102658\_at,103035\_at,103065\_at,103376\_s\_at,103427\_at,103739\_at,103913\_at,104019\_at,104461\_at,104534\_at,104719\_at,160683\_at,160684\_at,161689\_f\_at,161696\_f\_at,161990\_f\_at,92392\_at,92847\_s\_at,93316\_at,93320\_at,93414\_at,93626\_at,93705\_at,94031\_at,94556\_at,94657\_at,95377\_at,95444\_at,95586\_at,95695\_at,96019\_at,96076\_at,96079\_at,96669\_at,96725\_at,96876\_at,97181\_f\_at,97458\_at,97812\_at,99500\_at,92648\_at,160124\_r\_at,160202\_at,94043\_at,96951\_at,100499\_at,100635\_at,101934\_at,102750\_at,103062\_at,104179\_at,160082\_s\_at,160149\_at,161026\_s\_at,92185\_at,92805\_s\_at,94319\_at,96530\_at,97058\_f\_at,97224\_at,98927\_at,99350\_at,99354\_s\_at,99358\_at,101023\_f\_at,104453\_at,92256\_at,95023\_at,103686\_at,94254\_at,94255\_g\_at,94256\_at,94464\_at,94465\_g\_at,95655\_at,162041\_f\_at,93471\_at,103812\_at,102854\_s\_at,103935\_at,96186\_at,96534\_at,98114\_at,92531\_at,160795\_at,98926\_at,103258\_at,104522\_at,93063\_at,97551\_at,93193\_at,92992\_i\_at,92993\_r\_at,103499\_at,92268\_at,93083\_at,94192\_at,94383\_at,95474\_at,95477\_at,98018\_at,100068\_at,100522\_s\_at,100523\_r\_at,102768\_i\_at,102787\_at,160092\_at,161401\_f\_at,93017\_at,93241\_r\_at,94815\_at,95135\_at,95440\_at,97451\_at,98931\_at,160338\_at,101990\_at,161946\_r\_at,94439\_at,97833\_at,97834\_g\_at,99045\_at,AFFX-MUR\_b2\_at,104337\_f\_at,98984\_f\_at,98508\_s\_at,94415\_at,93177\_at,160084\_at,101490\_at,102302\_at,99184\_at,96657\_at,92586\_at,102360\_at,101000\_at,101001\_at,102313\_at,101186\_at,160335\_at,103471\_at,99849\_at,97829\_at,101681\_f\_at,103674\_f\_at,103994\_at,104049\_at,104144\_at,160111\_at,160112\_at,160130\_at,160976\_at,160977\_at,161342\_r\_at,161666\_f\_at,92855\_at,93975\_at,94499\_at,95070\_at,95737\_at,96325\_at,100136\_at,101590\_at,95681\_f\_at,96628\_at,93315\_at,160137\_at,94433\_at,160579\_at,94818\_at,93852\_at,97798\_at,102321\_at,102322\_at,94872\_at,99133\_at,93165\_at,100622\_at,95062\_at,100905\_at,101963\_at,102064\_at,102823\_at,102824\_g\_at,102905\_at,103222\_at,103223\_at,104186\_at,104188\_at,104677\_at,160655\_at,160718\_at,161270\_i\_at,94695\_at,94834\_at,94861\_at,96738\_at,97111\_at,97336\_at,97665\_i\_at,98436\_s\_at,99970\_at,103713\_at,160205\_f\_at,92660\_f\_at,92821\_at,93464\_at,94917\_at,95563\_at,95564\_at,96176\_at,99102\_at,93026\_at,101515\_at,101585\_at,102125\_f\_at,103353\_f\_at,103922\_f\_at,160088\_at,160383\_at,160391\_at,160588\_at,160611\_at,162044\_f\_at,93421\_at,93424\_at,93440\_at,93997\_at,94948\_at,95425\_at,96603\_at,96886\_at,97496\_f\_at,98533\_at,99985\_at,161760\_s\_at,96609\_at,104165\_at,97897\_at,94343\_at,94345\_at,94346\_at,100030\_at,104404\_at,160199\_at,160200\_at,94041\_at,102094\_f\_at,93267\_at,96777\_at,104456\_at,160374\_r\_at,92568\_at,101943\_at,160430\_at,160739\_at,92248\_at,92249\_g\_at,92484\_at,104701\_at,102425\_at,103288\_at,160440\_at,100011\_at,100032\_at,100533\_s\_at,100924\_at,101465\_at,101502\_at,102069\_at,102209\_at,102371\_at,102381\_at,102384\_at,102644\_at,102657\_at,102789\_at,102955\_at,102983\_at,102984\_g\_at,102996\_at,103015\_at,103086\_at,103321\_at,103328\_at,103440\_at,103497\_at,103501\_at,103547\_at,103634\_at,103720\_at,103774\_at,103901\_at,104070\_at,104155\_f\_at,104156\_r\_at,104562\_at,104605\_at,104645\_at,160138\_at,160246\_at,160313\_at,160397\_at,160483\_at,160502\_at,160526\_s\_at,160605\_s\_at,160724\_at,160783\_at,160834\_at,160894\_at,161084\_at,161113\_at,161847\_r\_at,162010\_r\_at,92195\_at,92216\_at,92440\_at,92562\_at,92564\_at,92854\_at,92908\_at,92925\_at,93528\_s\_at,93619\_at,93728\_at,93793\_at,94356\_at,94408\_at,94469\_at,94689\_at,94821\_at,95521\_s\_at,95522\_i\_at,95671\_at,95673\_s\_at,96192\_at,96238\_at,96561\_at,96836\_r\_at,96961\_at,97118\_at,97355\_at,97859\_at,98083\_at,98427\_s\_at,98468\_r\_at,98988\_at,99024\_at,99076\_at,99100\_at,99103\_at,99602\_at,99603\_g\_at,99622\_at,92344\_at,96703\_at,98122\_at,93543\_f\_at,100595\_at,101836\_at,92888\_s\_at,93179\_at,93285\_at,94980\_at,98580\_at,100417\_at,101936\_at,102224\_at,102332\_at,103020\_s\_at,103021\_r\_at,103451\_at,103969\_at,104417\_at,161067\_at,161184\_f\_at,93274\_at,93311\_at,97429\_at,97890\_at,97925\_at,98369\_f\_at,98504\_at,99070\_at,102414\_i\_at,102415\_r\_at,102279\_at,160103\_at,160104\_at,93731\_at,103584\_at,98906\_at,102912\_at,103531\_f\_at,104389\_at,104445\_at,104572\_at,160099\_at,93875\_at,94817\_at,95360\_at,97915\_at,97918\_at,100154\_at,100306\_at,100327\_at,100600\_at,100998\_at,101876\_s\_at,101878\_at,101886\_f\_at,103422\_at,104694\_at,160651\_at,92866\_at,93078\_at,93088\_at,93120\_f\_at,93865\_s\_at,93907\_f\_at,94286\_at,96752\_at,97125\_f\_at,97540\_f\_at,97541\_f\_at,98000\_at,98438\_f\_at,98472\_at,99378\_f\_at,99379\_f\_at,100973\_i\_at,102940\_at,103202\_at,104100\_at,104597\_at,98088\_at,103467\_g\_at,100583\_at,102156\_f\_at,102161\_f\_at,102372\_at,93086\_at,95057\_at,95058\_f\_at |
| 3 | cell growth and/or maintenance | 230 | 2128 | 1040 | 10726 | 0.221 | 0.198 | 1.115 | 0.03 | 100130\_at,101030\_at,101475\_at,102364\_at,102920\_at,103990\_at,104477\_at,104576\_at,160829\_at,160901\_at,161551\_f\_at,92653\_at,92926\_at,93104\_at,93666\_at,93714\_f\_at,94264\_at,94448\_at,94505\_at,94752\_s\_at,95348\_at,95617\_at,95618\_at,96513\_at,96771\_at,96810\_at,97484\_at,97973\_at,98110\_at,98756\_at,99467\_at,101998\_at,102752\_at,97426\_at,97498\_at,103899\_at,162206\_f\_at,92778\_i\_at,92780\_f\_at,94809\_at,96633\_s\_at,99475\_at,92263\_at,92270\_at,98324\_at,104263\_at,100606\_at,101118\_at,103959\_at,160676\_at,94976\_at,100514\_at,104725\_at,96299\_at,96736\_at,101578\_f\_at,92542\_at,94270\_at,98968\_at,160065\_s\_at,95489\_at,98127\_at,94106\_at,96278\_at,99013\_f\_at,100342\_i\_at,160287\_at,160288\_at,160679\_at,161615\_f\_at,93729\_at,94835\_f\_at,95119\_at,98461\_at,98882\_s\_at,98884\_r\_at,160373\_i\_at,96945\_at,96779\_f\_at,97409\_at,101848\_g\_at,160399\_r\_at,93020\_at,162138\_s\_at,104376\_at,101015\_s\_at,102994\_at,104761\_at,92770\_at,94928\_at,96852\_at,102781\_at,103416\_at,104598\_at,160127\_at,160359\_at,160495\_at,94820\_r\_at,94881\_at,94882\_at,95471\_at,95805\_at,96728\_at,97504\_at,97844\_at,98067\_at,98478\_at,99187\_f\_at,99188\_at,99529\_f\_at,100307\_at,101930\_at,103500\_at,160603\_at,95613\_at,104735\_at,95917\_at,92638\_at,94483\_at,96147\_at,96600\_at,100951\_at,102292\_at,97375\_at,100088\_at,95033\_at,104149\_at,94331\_at,101457\_at,160301\_at,96488\_at,96489\_at,102794\_at,160511\_at,100567\_at,100944\_at,101079\_at,101217\_at,101568\_at,101787\_f\_at,102658\_at,103035\_at,103065\_at,103376\_s\_at,103427\_at,103739\_at,103913\_at,104019\_at,104461\_at,104534\_at,104719\_at,160683\_at,160684\_at,161689\_f\_at,161696\_f\_at,161990\_f\_at,92392\_at,92847\_s\_at,93316\_at,93320\_at,93414\_at,93626\_at,93705\_at,94031\_at,94556\_at,94657\_at,95377\_at,95444\_at,95586\_at,95695\_at,96019\_at,96076\_at,96079\_at,96669\_at,96725\_at,96876\_at,97181\_f\_at,97458\_at,97812\_at,99500\_at,92648\_at,160124\_r\_at,160202\_at,94043\_at,96951\_at,100499\_at,100635\_at,101934\_at,102750\_at,103062\_at,104179\_at,160082\_s\_at,160149\_at,161026\_s\_at,92185\_at,92805\_s\_at,94319\_at,96530\_at,97058\_f\_at,97224\_at,98927\_at,99350\_at,99354\_s\_at,99358\_at,101023\_f\_at,104453\_at,92256\_at,95023\_at,103686\_at,94254\_at,94255\_g\_at,94256\_at,94464\_at,94465\_g\_at,95655\_at,162041\_f\_at,93471\_at,103812\_at,102854\_s\_at,103935\_at,96186\_at,96534\_at,98114\_at,92531\_at,160795\_at,98926\_at,103258\_at,104522\_at,93063\_at,97551\_at |
| 4 | autophagy | 1 | 6 | 1185 | 13100 | 0.001 | 0 | 1.826 | 0.434 | 101998\_at |
| 4 | cell growth | 13 | 51 | 1185 | 13100 | 0.011 | 0.004 | 2.82 | 0 | 102752\_at,97426\_at,97498\_at,103899\_at,162206\_f\_at,92778\_i\_at,92780\_f\_at,93714\_f\_at,94809\_at,96633\_s\_at,99475\_at,92263\_at,92270\_at |
| 5 | regulation of cell growth | 10 | 38 | 1042 | 11544 | 0.01 | 0.003 | 2.918 | 0.002 | 103899\_at,162206\_f\_at,92778\_i\_at,92780\_f\_at,93714\_f\_at,94809\_at,96633\_s\_at,99475\_at,92263\_at,92270\_at |
| 6 | negative regulation of cell growth | 2 | 2 | 880 | 9498 | 0.002 | 0 | 10.81 | 0.009 | 92263\_at,92270\_at |
| 4 | cell homeostasis | 7 | 41 | 1185 | 13100 | 0.006 | 0.003 | 1.888 | 0.073 | 98324\_at,104263\_at,100606\_at,101118\_at,103959\_at,160676\_at,94976\_at |
| 5 | cell glucose homeostasis | 1 | 2 | 1042 | 11544 | 0.001 | 0 | 5.647 | 0.172 | 98324\_at |
| 5 | cell ion homeostasis | 6 | 37 | 1042 | 11544 | 0.006 | 0.003 | 1.794 | 0.112 | 104263\_at,100606\_at,101118\_at,103959\_at,160676\_at,94976\_at |
| 6 | cation homeostasis | 6 | 36 | 880 | 9498 | 0.007 | 0.004 | 1.799 | 0.111 | 104263\_at,100606\_at,101118\_at,103959\_at,160676\_at,94976\_at |
| 7 | di-, tri-valent inorganic cation homeostasis | 6 | 29 | 538 | 6246 | 0.011 | 0.005 | 2.403 | 0.034 | 104263\_at,100606\_at,101118\_at,103959\_at,160676\_at,94976\_at |
| 8 | calcium ion homeostasis | 1 | 13 | 199 | 2164 | 0.005 | 0.006 | 0.837 | 0.716 | 104263\_at |
| 8 | copper ion homeostasis | 1 | 1 | 199 | 2164 | 0.005 | 0 | 10.935 | 0.092 | 100606\_at |
| 8 | iron ion homeostasis | 4 | 15 | 199 | 2164 | 0.02 | 0.007 | 2.9 | 0.042 | 101118\_at,103959\_at,160676\_at,94976\_at |
| 4 | cell organization and biogenesis | 35 | 530 | 1185 | 13100 | 0.03 | 0.04 | 0.73 | 0.984 | 100514\_at,104725\_at,96299\_at,96736\_at,101578\_f\_at,92542\_at,94270\_at,98968\_at,160065\_s\_at,95489\_at,98127\_at,94106\_at,96278\_at,99013\_f\_at,100342\_i\_at,160287\_at,160288\_at,160679\_at,161615\_f\_at,93729\_at,94835\_f\_at,95119\_at,98461\_at,98882\_s\_at,98884\_r\_at,160373\_i\_at,96945\_at,96779\_f\_at,97409\_at,101848\_g\_at,160399\_r\_at,93020\_at,162138\_s\_at,101475\_at,104376\_at |
| 5 | cellular morphogenesis | 3 | 49 | 1042 | 11544 | 0.003 | 0.004 | 0.679 | 0.831 | 100514\_at,104725\_at,96299\_at |
| 6 | regulation of cell shape | 3 | 22 | 880 | 9498 | 0.003 | 0.002 | 1.47 | 0.334 | 100514\_at,104725\_at,96299\_at |
| 5 | cytoplasm organization and biogenesis | 28 | 380 | 1042 | 11544 | 0.027 | 0.033 | 0.816 | 0.895 | 96736\_at,101578\_f\_at,92542\_at,94270\_at,98968\_at,104725\_at,160065\_s\_at,95489\_at,98127\_at,94106\_at,96278\_at,96299\_at,99013\_f\_at,100342\_i\_at,160287\_at,160288\_at,160679\_at,161615\_f\_at,93729\_at,94835\_f\_at,95119\_at,98461\_at,98882\_s\_at,98884\_r\_at,160373\_i\_at,96945\_at,96779\_f\_at,97409\_at |
| 6 | organelle organization and biogenesis | 25 | 318 | 880 | 9498 | 0.028 | 0.033 | 0.849 | 0.835 | 101578\_f\_at,92542\_at,94270\_at,98968\_at,104725\_at,160065\_s\_at,95489\_at,98127\_at,94106\_at,96278\_at,96299\_at,99013\_f\_at,100342\_i\_at,160287\_at,160288\_at,160679\_at,161615\_f\_at,93729\_at,94835\_f\_at,95119\_at,98461\_at,98882\_s\_at,98884\_r\_at,160373\_i\_at,96945\_at |
| 7 | cytoskeleton organization and biogenesis | 23 | 262 | 538 | 6246 | 0.043 | 0.042 | 1.019 | 0.495 | 101578\_f\_at,92542\_at,94270\_at,98968\_at,104725\_at,160065\_s\_at,95489\_at,98127\_at,94106\_at,96278\_at,96299\_at,99013\_f\_at,100342\_i\_at,160287\_at,160288\_at,160679\_at,161615\_f\_at,93729\_at,94835\_f\_at,95119\_at,98461\_at,98882\_s\_at,98884\_r\_at |
| 8 | actin filament-based process | 8 | 42 | 199 | 2164 | 0.04 | 0.019 | 2.071 | 0.034 | 104725\_at,160065\_s\_at,95489\_at,98127\_at,94106\_at,96278\_at,96299\_at,99013\_f\_at |
| 9 | actin cytoskeleton organization and biogenesis | 8 | 42 | 72 | 911 | 0.111 | 0.046 | 2.41 | 0.014 | 104725\_at,160065\_s\_at,95489\_at,98127\_at,94106\_at,96278\_at,96299\_at,99013\_f\_at |
| 10 | actin filament organization | 4 | 16 | 15 | 197 | 0.267 | 0.081 | 3.283 | 0.023 | 94106\_at,96278\_at,96299\_at,99013\_f\_at |
| 8 | microtubule-based process | 12 | 119 | 199 | 2164 | 0.06 | 0.055 | 1.097 | 0.413 | 100342\_i\_at,160287\_at,160288\_at,160679\_at,161615\_f\_at,93729\_at,94835\_f\_at,95119\_at,96299\_at,98461\_at,98882\_s\_at,98884\_r\_at |
| 9 | microtubule-based movement | 4 | 38 | 72 | 911 | 0.056 | 0.042 | 1.332 | 0.353 | 100342\_i\_at,96299\_at,98882\_s\_at,98884\_r\_at |
| 10 | axon cargo transport | 2 | 4 | 15 | 197 | 0.133 | 0.02 | 6.568 | 0.03 | 98882\_s\_at,98884\_r\_at |
| 11 | retrograde axon cargo transport | 2 | 4 | 5 | 34 | 0.4 | 0.118 | 3.4 | 0.094 | 98882\_s\_at,98884\_r\_at |
| 7 | ER organization and biogenesis | 1 | 6 | 538 | 6246 | 0.002 | 0.001 | 1.938 | 0.418 | 160373\_i\_at |
| 8 | protein-ER targeting | 1 | 6 | 199 | 2164 | 0.005 | 0.003 | 1.816 | 0.44 | 160373\_i\_at |
| 9 | cotranslational membrane targeting | 1 | 3 | 72 | 911 | 0.014 | 0.003 | 4.222 | 0.219 | 160373\_i\_at |
| 7 | vacuole organization and biogenesis | 1 | 7 | 538 | 6246 | 0.002 | 0.001 | 1.661 | 0.468 | 96945\_at |
| 6 | ribosome biogenesis and assembly | 2 | 60 | 880 | 9498 | 0.002 | 0.006 | 0.359 | 0.979 | 96779\_f\_at,97409\_at |
| 7 | ribosome biogenesis | 2 | 60 | 538 | 6246 | 0.004 | 0.01 | 0.387 | 0.971 | 96779\_f\_at,97409\_at |
| 5 | nuclear organization and biogenesis | 6 | 112 | 1042 | 11544 | 0.006 | 0.01 | 0.594 | 0.946 | 101848\_g\_at,160399\_r\_at,93020\_at,162138\_s\_at,101475\_at,104376\_at |
| 6 | chromosome organization and biogenesis (sensu Eukarya) | 6 | 108 | 880 | 9498 | 0.007 | 0.011 | 0.6 | 0.943 | 101848\_g\_at,160399\_r\_at,93020\_at,162138\_s\_at,101475\_at,104376\_at |
| 7 | establishment and/or maintenance of chromatin architecture | 5 | 80 | 538 | 6246 | 0.009 | 0.013 | 0.725 | 0.831 | 162138\_s\_at,160399\_r\_at,93020\_at,101475\_at,104376\_at |
| 8 | chromatin assembly/disassembly | 3 | 48 | 199 | 2164 | 0.015 | 0.022 | 0.68 | 0.833 | 162138\_s\_at,160399\_r\_at,93020\_at |
| 9 | nucleosome assembly | 2 | 28 | 72 | 911 | 0.028 | 0.031 | 0.904 | 0.665 | 160399\_r\_at,93020\_at |
| 8 | chromatin modification | 3 | 36 | 199 | 2164 | 0.015 | 0.017 | 0.906 | 0.657 | 101475\_at,104376\_at,162138\_s\_at |
| 4 | cell proliferation | 68 | 501 | 1185 | 13100 | 0.057 | 0.038 | 1.501 | 0 | 101015\_s\_at,102752\_at,102994\_at,104761\_at,92770\_at,93104\_at,94928\_at,96852\_at,97484\_at,102781\_at,103416\_at,104477\_at,104598\_at,160127\_at,160359\_at,160495\_at,94820\_r\_at,94881\_at,94882\_at,95471\_at,95617\_at,95618\_at,95805\_at,96728\_at,97504\_at,97844\_at,98067\_at,98478\_at,99187\_f\_at,99188\_at,99529\_f\_at,100307\_at,101930\_at,103500\_at,160603\_at,95613\_at,104735\_at,95917\_at,100130\_at,102364\_at,102920\_at,103990\_at,160901\_at,92638\_at,92926\_at,94264\_at,94483\_at,94505\_at,95348\_at,96147\_at,96513\_at,96600\_at,96771\_at,98756\_at,100951\_at,102292\_at,94809\_at,97375\_at,100088\_at,95033\_at,104149\_at,94331\_at,101457\_at,160301\_at,96488\_at,96489\_at,102794\_at,160511\_at |
| 5 | cell cycle | 51 | 435 | 1042 | 11544 | 0.049 | 0.038 | 1.299 | 0.031 | 102781\_at,103416\_at,104477\_at,104598\_at,160127\_at,160359\_at,160495\_at,92770\_at,94820\_r\_at,94881\_at,94882\_at,95471\_at,95617\_at,95618\_at,95805\_at,96728\_at,97504\_at,97844\_at,98067\_at,98478\_at,99187\_f\_at,99188\_at,99529\_f\_at,100307\_at,101930\_at,103500\_at,160603\_at,95613\_at,104735\_at,95917\_at,100130\_at,102364\_at,102752\_at,102920\_at,103990\_at,160901\_at,92638\_at,92926\_at,94264\_at,94483\_at,94505\_at,95348\_at,96147\_at,96513\_at,96600\_at,96771\_at,98756\_at,100951\_at,102292\_at,94809\_at,97375\_at |
| 6 | DNA replication and chromosome cycle | 5 | 113 | 880 | 9498 | 0.006 | 0.012 | 0.477 | 0.983 | 100307\_at,101930\_at,103500\_at,160603\_at,95613\_at |
| 7 | DNA replication | 5 | 94 | 538 | 6246 | 0.009 | 0.015 | 0.617 | 0.917 | 100307\_at,101930\_at,103500\_at,160603\_at,95613\_at |
| 6 | M phase | 5 | 74 | 880 | 9498 | 0.006 | 0.008 | 0.729 | 0.828 | 104735\_at,160127\_at,98478\_at,99187\_f\_at,99188\_at |
| 7 | M phase of mitotic cell cycle | 5 | 57 | 538 | 6246 | 0.009 | 0.009 | 1.018 | 0.552 | 104735\_at,160127\_at,98478\_at,99187\_f\_at,99188\_at |
| 8 | mitosis | 5 | 57 | 199 | 2164 | 0.025 | 0.026 | 0.954 | 0.614 | 104735\_at,160127\_at,98478\_at,99187\_f\_at,99188\_at |
| 6 | mitotic cell cycle | 6 | 173 | 880 | 9498 | 0.007 | 0.018 | 0.375 | 0.999 | 95917\_at,104735\_at,160127\_at,98478\_at,99187\_f\_at,99188\_at |
| 7 | M phase of mitotic cell cycle | 5 | 57 | 538 | 6246 | 0.009 | 0.009 | 1.018 | 0.552 | 104735\_at,160127\_at,98478\_at,99187\_f\_at,99188\_at |
| 8 | mitosis | 5 | 57 | 199 | 2164 | 0.025 | 0.026 | 0.954 | 0.614 | 104735\_at,160127\_at,98478\_at,99187\_f\_at,99188\_at |
| 6 | regulation of cell cycle | 32 | 204 | 880 | 9498 | 0.036 | 0.021 | 1.693 | 0.002 | 100130\_at,102364\_at,102752\_at,102920\_at,103990\_at,160127\_at,160901\_at,92638\_at,92770\_at,92926\_at,94264\_at,94483\_at,94505\_at,94820\_r\_at,95348\_at,96147\_at,96513\_at,96600\_at,96771\_at,97504\_at,98478\_at,98756\_at,99187\_f\_at,99188\_at,100951\_at,102292\_at,94809\_at,94881\_at,94882\_at,95471\_at,97375\_at,98067\_at |
| 7 | cell cycle arrest | 8 | 20 | 538 | 6246 | 0.015 | 0.003 | 4.647 | 0 | 100951\_at,102292\_at,94809\_at,94881\_at,94882\_at,95471\_at,97375\_at,98067\_at |
| 5 | cytokinesis | 3 | 5 | 1042 | 11544 | 0.003 | 0 | 6.698 | 0.006 | 100088\_at,104735\_at,95033\_at |
| 5 | regulation of cell proliferation | 9 | 38 | 1042 | 11544 | 0.009 | 0.003 | 2.626 | 0.006 | 104149\_at,94331\_at,96147\_at,101457\_at,160301\_at,93104\_at,94809\_at,96488\_at,96489\_at |
| 6 | negative regulation of cell proliferation | 6 | 17 | 880 | 9498 | 0.007 | 0.002 | 3.81 | 0.003 | 101457\_at,160301\_at,93104\_at,94809\_at,96488\_at,96489\_at |
| 5 | T-cell proliferation | 2 | 4 | 1042 | 11544 | 0.002 | 0 | 5.486 | 0.043 | 102794\_at,160511\_at |
| 4 | transport | 106 | 1083 | 1185 | 13100 | 0.089 | 0.083 | 1.082 | 0.201 | 100567\_at,100944\_at,101079\_at,101118\_at,101217\_at,101568\_at,101787\_f\_at,102658\_at,103035\_at,103065\_at,103376\_s\_at,103427\_at,103739\_at,103913\_at,104019\_at,104461\_at,104534\_at,104719\_at,160683\_at,160684\_at,161551\_f\_at,161689\_f\_at,161696\_f\_at,161990\_f\_at,92392\_at,92847\_s\_at,93316\_at,93320\_at,93414\_at,93626\_at,93705\_at,94031\_at,94556\_at,94657\_at,95377\_at,95444\_at,95586\_at,95695\_at,96019\_at,96076\_at,96079\_at,96669\_at,96725\_at,96876\_at,97181\_f\_at,97458\_at,97812\_at,99500\_at,92648\_at,160124\_r\_at,160202\_at,94043\_at,96951\_at,96945\_at,100499\_at,100635\_at,101934\_at,102750\_at,103062\_at,104179\_at,160082\_s\_at,160149\_at,161026\_s\_at,92185\_at,92805\_s\_at,94319\_at,94505\_at,96530\_at,97058\_f\_at,97224\_at,98927\_at,99350\_at,99354\_s\_at,99358\_at,101023\_f\_at,104453\_at,92256\_at,95023\_at,96736\_at,160373\_i\_at,104149\_at,100951\_at,103686\_at,94254\_at,94255\_g\_at,94256\_at,94464\_at,94465\_g\_at,95655\_at,162041\_f\_at,93471\_at,103812\_at,102854\_s\_at,103935\_at,97375\_at,96186\_at,96534\_at,98114\_at,92531\_at,160795\_at,98926\_at,103258\_at,103959\_at,104522\_at,93063\_at,97551\_at |
| 5 | amine/polyamine transport | 2 | 19 | 1042 | 11544 | 0.002 | 0.002 | 1.164 | 0.522 | 104719\_at,99500\_at |
| 6 | amino acid transport | 2 | 19 | 880 | 9498 | 0.002 | 0.002 | 1.135 | 0.537 | 104719\_at,99500\_at |
| 5 | carbohydrate transport | 1 | 28 | 1042 | 11544 | 0.001 | 0.002 | 0.395 | 0.929 | 92648\_at |
| 6 | monosaccharide transport | 1 | 13 | 880 | 9498 | 0.001 | 0.001 | 0.832 | 0.718 | 92648\_at |
| 7 | hexose transport | 1 | 13 | 538 | 6246 | 0.002 | 0.002 | 0.894 | 0.69 | 92648\_at |
| 8 | glucose transport | 1 | 13 | 199 | 2164 | 0.005 | 0.006 | 0.837 | 0.716 | 92648\_at |
| 5 | gas transport | 1 | 10 | 1042 | 11544 | 0.001 | 0.001 | 1.103 | 0.612 | 97181\_f\_at |
| 6 | oxygen transport | 1 | 10 | 880 | 9498 | 0.001 | 0.001 | 1.086 | 0.622 | 97181\_f\_at |
| 5 | hydrogen transport | 4 | 50 | 1042 | 11544 | 0.004 | 0.004 | 0.887 | 0.673 | 160124\_r\_at,160202\_at,94043\_at,96951\_at |
| 6 | proton transport | 4 | 44 | 880 | 9498 | 0.005 | 0.005 | 0.983 | 0.592 | 160124\_r\_at,160202\_at,94043\_at,96951\_at |
| 5 | intracellular transport | 39 | 351 | 1042 | 11544 | 0.037 | 0.03 | 1.231 | 0.101 | 96945\_at,100499\_at,100635\_at,101934\_at,102750\_at,103062\_at,103913\_at,104179\_at,160082\_s\_at,160149\_at,161026\_s\_at,92185\_at,92648\_at,92805\_s\_at,92847\_s\_at,94031\_at,94319\_at,94505\_at,94556\_at,95444\_at,96076\_at,96530\_at,96669\_at,97058\_f\_at,97224\_at,97458\_at,97812\_at,98927\_at,99350\_at,99354\_s\_at,99358\_at,101023\_f\_at,104453\_at,92256\_at,95023\_at,96736\_at,160373\_i\_at,101079\_at,104149\_at |
| 6 | Golgi vesicle transport | 1 | 12 | 880 | 9498 | 0.001 | 0.001 | 0.905 | 0.689 | 96945\_at |
| 7 | post-Golgi transport | 1 | 5 | 538 | 6246 | 0.002 | 0.001 | 2.325 | 0.363 | 96945\_at |
| 6 | intracellular protein transport | 39 | 284 | 880 | 9498 | 0.044 | 0.03 | 1.482 | 0.008 | 100499\_at,100635\_at,101934\_at,102750\_at,103062\_at,103913\_at,104179\_at,160082\_s\_at,160149\_at,161026\_s\_at,92185\_at,92648\_at,92805\_s\_at,92847\_s\_at,94031\_at,94319\_at,94505\_at,94556\_at,95444\_at,96076\_at,96530\_at,96669\_at,96945\_at,97058\_f\_at,97224\_at,97458\_at,97812\_at,98927\_at,99350\_at,99354\_s\_at,99358\_at,101023\_f\_at,104453\_at,92256\_at,95023\_at,96736\_at,160373\_i\_at,101079\_at,104149\_at |
| 7 | protein targeting | 12 | 101 | 538 | 6246 | 0.022 | 0.016 | 1.379 | 0.158 | 101023\_f\_at,103913\_at,104453\_at,92256\_at,95023\_at,95444\_at,96669\_at,96736\_at,160373\_i\_at,101079\_at,97458\_at,104149\_at |
| 8 | protein-ER targeting | 1 | 6 | 199 | 2164 | 0.005 | 0.003 | 1.816 | 0.44 | 160373\_i\_at |
| 9 | cotranslational membrane targeting | 1 | 3 | 72 | 911 | 0.014 | 0.003 | 4.222 | 0.219 | 160373\_i\_at |
| 8 | protein-nucleus import | 3 | 32 | 199 | 2164 | 0.015 | 0.015 | 1.02 | 0.576 | 101079\_at,97458\_at,104149\_at |
| 9 | protein-nucleus import, translocation | 1 | 4 | 72 | 911 | 0.014 | 0.004 | 3.164 | 0.281 | 104149\_at |
| 6 | nucleocytoplasmic transport | 3 | 40 | 880 | 9498 | 0.003 | 0.004 | 0.81 | 0.73 | 101079\_at,97458\_at,104149\_at |
| 7 | RNA-nucleus export | 1 | 7 | 538 | 6246 | 0.002 | 0.001 | 1.661 | 0.468 | 101079\_at |
| 8 | mRNA-nucleus export | 1 | 4 | 199 | 2164 | 0.005 | 0.002 | 2.719 | 0.32 | 101079\_at |
| 5 | ion transport | 28 | 335 | 1042 | 11544 | 0.027 | 0.029 | 0.926 | 0.696 | 100951\_at,101217\_at,101787\_f\_at,103686\_at,104461\_at,104719\_at,93705\_at,94254\_at,94255\_g\_at,94256\_at,94464\_at,94465\_g\_at,94657\_at,95377\_at,95586\_at,95655\_at,99500\_at,162041\_f\_at,93471\_at,103812\_at,103065\_at,96079\_at,100944\_at,102854\_s\_at,103935\_at,97375\_at,101118\_at,104019\_at |
| 6 | anion transport | 17 | 79 | 880 | 9498 | 0.019 | 0.008 | 2.322 | 0.001 | 162041\_f\_at,93471\_at,101217\_at,103812\_at,104719\_at,94254\_at,94255\_g\_at,94256\_at,94464\_at,94465\_g\_at,94657\_at,95377\_at,95655\_at,99500\_at,103065\_at,96079\_at,100944\_at |
| 7 | inorganic anion transport | 14 | 50 | 538 | 6246 | 0.026 | 0.008 | 3.248 | 0 | 101217\_at,103812\_at,104719\_at,94254\_at,94255\_g\_at,94256\_at,94464\_at,94465\_g\_at,94657\_at,95377\_at,95655\_at,99500\_at,103065\_at,96079\_at |
| 8 | chloride transport | 12 | 39 | 199 | 2164 | 0.06 | 0.018 | 3.346 | 0 | 101217\_at,103812\_at,104719\_at,94254\_at,94255\_g\_at,94256\_at,94464\_at,94465\_g\_at,94657\_at,95377\_at,95655\_at,99500\_at |
| 8 | phosphate transport | 2 | 6 | 199 | 2164 | 0.01 | 0.003 | 3.628 | 0.099 | 103065\_at,96079\_at |
| 7 | organic anion transport | 1 | 15 | 538 | 6246 | 0.002 | 0.002 | 0.775 | 0.741 | 100944\_at |
| 8 | dicarboxylic acid transport | 1 | 7 | 199 | 2164 | 0.005 | 0.003 | 1.557 | 0.491 | 100944\_at |
| 6 | cation transport | 12 | 236 | 880 | 9498 | 0.014 | 0.025 | 0.549 | 0.994 | 100951\_at,101787\_f\_at,102854\_s\_at,103686\_at,103935\_at,104461\_at,97375\_at,101118\_at,104719\_at,99500\_at,104019\_at,96079\_at |
| 7 | di-, tri-valent inorganic cation transport | 6 | 58 | 538 | 6246 | 0.011 | 0.009 | 1.2 | 0.383 | 100951\_at,103935\_at,104461\_at,97375\_at,102854\_s\_at,101118\_at |
| 8 | calcium ion transport | 4 | 33 | 199 | 2164 | 0.02 | 0.015 | 1.318 | 0.361 | 100951\_at,103935\_at,104461\_at,97375\_at |
| 8 | transition metal ion transport | 2 | 25 | 199 | 2164 | 0.01 | 0.012 | 0.87 | 0.685 | 102854\_s\_at,101118\_at |
| 9 | copper ion transport | 1 | 5 | 72 | 911 | 0.014 | 0.005 | 2.53 | 0.338 | 102854\_s\_at |
| 9 | iron ion transport | 1 | 11 | 72 | 911 | 0.014 | 0.012 | 1.151 | 0.598 | 101118\_at |
| 7 | metal ion transport | 11 | 184 | 538 | 6246 | 0.02 | 0.029 | 0.694 | 0.929 | 102854\_s\_at,100951\_at,103935\_at,104461\_at,97375\_at,101118\_at,101787\_f\_at,104719\_at,99500\_at,104019\_at,96079\_at |
| 8 | calcium ion transport | 4 | 33 | 199 | 2164 | 0.02 | 0.015 | 1.318 | 0.361 | 100951\_at,103935\_at,104461\_at,97375\_at |
| 8 | transition metal ion transport | 2 | 25 | 199 | 2164 | 0.01 | 0.012 | 0.87 | 0.685 | 102854\_s\_at,101118\_at |
| 9 | copper ion transport | 1 | 5 | 72 | 911 | 0.014 | 0.005 | 2.53 | 0.338 | 102854\_s\_at |
| 9 | iron ion transport | 1 | 11 | 72 | 911 | 0.014 | 0.012 | 1.151 | 0.598 | 101118\_at |
| 8 | potassium ion transport | 3 | 94 | 199 | 2164 | 0.015 | 0.043 | 0.347 | 0.994 | 101787\_f\_at,104719\_at,99500\_at |
| 8 | sodium ion transport | 4 | 43 | 199 | 2164 | 0.02 | 0.02 | 1.012 | 0.568 | 104019\_at,104719\_at,96079\_at,99500\_at |
| 5 | lipid transport | 4 | 40 | 1042 | 11544 | 0.004 | 0.003 | 1.107 | 0.493 | 93316\_at,96186\_at,96534\_at,98114\_at |
| 6 | sterol transport | 1 | 3 | 880 | 9498 | 0.001 | 0 | 3.562 | 0.253 | 98114\_at |
| 7 | cholesterol transport | 1 | 3 | 538 | 6246 | 0.002 | 0 | 3.875 | 0.237 | 98114\_at |
| 5 | neurotransmitter transport | 2 | 21 | 1042 | 11544 | 0.002 | 0.002 | 1.055 | 0.577 | 100499\_at,161696\_f\_at |
| 5 | peptide transport | 1 | 5 | 1042 | 11544 | 0.001 | 0 | 2.233 | 0.377 | 103035\_at |
| 6 | oligopeptide transport | 1 | 5 | 880 | 9498 | 0.001 | 0.001 | 2.151 | 0.385 | 103035\_at |
| 5 | protein transport | 40 | 297 | 1042 | 11544 | 0.038 | 0.026 | 1.492 | 0.007 | 100635\_at,102750\_at,103062\_at,103913\_at,104179\_at,160082\_s\_at,92531\_at,92648\_at,94031\_at,94319\_at,94505\_at,94556\_at,95444\_at,96669\_at,96945\_at,97058\_f\_at,97224\_at,97458\_at,97812\_at,98927\_at,99350\_at,99354\_s\_at,99358\_at,100499\_at,101934\_at,160149\_at,161026\_s\_at,92185\_at,92805\_s\_at,92847\_s\_at,96076\_at,96530\_at,101023\_f\_at,104453\_at,92256\_at,95023\_at,96736\_at,160373\_i\_at,101079\_at,104149\_at |
| 6 | intracellular protein transport | 39 | 284 | 880 | 9498 | 0.044 | 0.03 | 1.482 | 0.008 | 100499\_at,100635\_at,101934\_at,102750\_at,103062\_at,103913\_at,104179\_at,160082\_s\_at,160149\_at,161026\_s\_at,92185\_at,92648\_at,92805\_s\_at,92847\_s\_at,94031\_at,94319\_at,94505\_at,94556\_at,95444\_at,96076\_at,96530\_at,96669\_at,96945\_at,97058\_f\_at,97224\_at,97458\_at,97812\_at,98927\_at,99350\_at,99354\_s\_at,99358\_at,101023\_f\_at,104453\_at,92256\_at,95023\_at,96736\_at,160373\_i\_at,101079\_at,104149\_at |
| 7 | protein targeting | 12 | 101 | 538 | 6246 | 0.022 | 0.016 | 1.379 | 0.158 | 101023\_f\_at,103913\_at,104453\_at,92256\_at,95023\_at,95444\_at,96669\_at,96736\_at,160373\_i\_at,101079\_at,97458\_at,104149\_at |
| 8 | protein-ER targeting | 1 | 6 | 199 | 2164 | 0.005 | 0.003 | 1.816 | 0.44 | 160373\_i\_at |
| 9 | cotranslational membrane targeting | 1 | 3 | 72 | 911 | 0.014 | 0.003 | 4.222 | 0.219 | 160373\_i\_at |
| 8 | protein-nucleus import | 3 | 32 | 199 | 2164 | 0.015 | 0.015 | 1.02 | 0.576 | 101079\_at,97458\_at,104149\_at |
| 9 | protein-nucleus import, translocation | 1 | 4 | 72 | 911 | 0.014 | 0.004 | 3.164 | 0.281 | 104149\_at |
| 5 | secretory pathway | 4 | 31 | 1042 | 11544 | 0.004 | 0.003 | 1.428 | 0.305 | 160373\_i\_at,96945\_at,160795\_at,98926\_at |
| 6 | exocytosis | 3 | 12 | 880 | 9498 | 0.003 | 0.001 | 2.706 | 0.093 | 160795\_at,98926\_at,96945\_at |
| 7 | calcium ion dependent exocytosis | 1 | 3 | 538 | 6246 | 0.002 | 0 | 3.875 | 0.237 | 98926\_at |
| 7 | nonselective vesicle exocytosis | 1 | 2 | 538 | 6246 | 0.002 | 0 | 5.812 | 0.165 | 96945\_at |
| 8 | nonselective vesicle targeting | 1 | 1 | 199 | 2164 | 0.005 | 0 | 10.935 | 0.092 | 96945\_at |
| 7 | regulation of exocytosis | 1 | 2 | 538 | 6246 | 0.002 | 0 | 5.812 | 0.165 | 98926\_at |
| 5 | vesicle-mediated transport | 12 | 112 | 1042 | 11544 | 0.012 | 0.01 | 1.188 | 0.31 | 96076\_at,96945\_at,160795\_at,98926\_at,103258\_at,103959\_at,104522\_at,93063\_at,96534\_at,97551\_at,96736\_at,104179\_at |
| 6 | exocytosis | 3 | 12 | 880 | 9498 | 0.003 | 0.001 | 2.706 | 0.093 | 160795\_at,98926\_at,96945\_at |
| 7 | calcium ion dependent exocytosis | 1 | 3 | 538 | 6246 | 0.002 | 0 | 3.875 | 0.237 | 98926\_at |
| 7 | nonselective vesicle exocytosis | 1 | 2 | 538 | 6246 | 0.002 | 0 | 5.812 | 0.165 | 96945\_at |
| 8 | nonselective vesicle targeting | 1 | 1 | 199 | 2164 | 0.005 | 0 | 10.935 | 0.092 | 96945\_at |
| 7 | regulation of exocytosis | 1 | 2 | 538 | 6246 | 0.002 | 0 | 5.812 | 0.165 | 98926\_at |
| 6 | endocytosis | 7 | 61 | 880 | 9498 | 0.008 | 0.006 | 1.238 | 0.335 | 103258\_at,103959\_at,104522\_at,93063\_at,96534\_at,97551\_at,96736\_at |
| 7 | receptor mediated endocytosis | 1 | 9 | 538 | 6246 | 0.002 | 0.001 | 1.292 | 0.556 | 97551\_at |
| 7 | regulation of endocytosis | 1 | 7 | 538 | 6246 | 0.002 | 0.001 | 1.661 | 0.468 | 96736\_at |
| 6 | nonselective vesicle transport | 1 | 7 | 880 | 9498 | 0.001 | 0.001 | 1.541 | 0.494 | 104179\_at |
| 3 | circulation | 3 | 40 | 1040 | 10726 | 0.003 | 0.004 | 0.772 | 0.759 | 93193\_at,92992\_i\_at,92993\_r\_at |
| 4 | regulation of blood pressure | 1 | 18 | 1185 | 13100 | 0.001 | 0.001 | 0.613 | 0.819 | 93193\_at |
| 4 | regulation of heart rate | 2 | 19 | 1185 | 13100 | 0.002 | 0.001 | 1.166 | 0.523 | 92992\_i\_at,92993\_r\_at |
| 3 | hemostasis | 8 | 44 | 1040 | 10726 | 0.008 | 0.004 | 1.876 | 0.058 | 103499\_at,92268\_at,93083\_at,94192\_at,94383\_at,95474\_at,95477\_at,98018\_at |
| 4 | blood coagulation | 8 | 42 | 1185 | 13100 | 0.007 | 0.003 | 2.103 | 0.032 | 103499\_at,92268\_at,93083\_at,94192\_at,94383\_at,95474\_at,95477\_at,98018\_at |
| 3 | lactation | 1 | 2 | 1040 | 10726 | 0.001 | 0 | 5.053 | 0.185 | 104263\_at |
| 3 | metabolism | 372 | 3908 | 1040 | 10726 | 0.358 | 0.364 | 0.982 | 0.692 | 100068\_at,100522\_s\_at,100523\_r\_at,102768\_i\_at,102787\_at,102854\_s\_at,103739\_at,103935\_at,160092\_at,161401\_f\_at,93017\_at,93241\_r\_at,94815\_at,94976\_at,95135\_at,95440\_at,97451\_at,98931\_at,160338\_at,101990\_at,161946\_r\_at,94439\_at,97833\_at,97834\_g\_at,99045\_at,AFFX-MUR\_b2\_at,104337\_f\_at,98984\_f\_at,98508\_s\_at,96534\_at,94415\_at,93177\_at,160084\_at,101490\_at,102302\_at,99184\_at,96657\_at,92586\_at,102360\_at,101000\_at,101001\_at,160603\_at,102313\_at,101186\_at,160124\_r\_at,94043\_at,96951\_at,160335\_at,103471\_at,99849\_at,97829\_at,101681\_f\_at,102292\_at,103674\_f\_at,103994\_at,104049\_at,104144\_at,160111\_at,160112\_at,160130\_at,160976\_at,160977\_at,161342\_r\_at,161666\_f\_at,92855\_at,93975\_at,94499\_at,95070\_at,95737\_at,96325\_at,100136\_at,101590\_at,95681\_f\_at,96628\_at,96725\_at,93315\_at,160137\_at,94433\_at,160579\_at,94818\_at,93852\_at,97798\_at,96299\_at,102321\_at,102322\_at,94872\_at,99133\_at,100088\_at,93165\_at,100622\_at,95062\_at,100905\_at,101963\_at,102064\_at,102823\_at,102824\_g\_at,102905\_at,103222\_at,103223\_at,103959\_at,103990\_at,104186\_at,104188\_at,104677\_at,160655\_at,160676\_at,160718\_at,161270\_i\_at,92256\_at,94695\_at,94834\_at,94861\_at,96738\_at,97111\_at,97336\_at,97665\_i\_at,98436\_s\_at,99970\_at,103713\_at,160205\_f\_at,92660\_f\_at,92821\_at,93464\_at,94917\_at,95563\_at,95564\_at,96176\_at,99102\_at,93026\_at,101515\_at,101585\_at,102125\_f\_at,103353\_f\_at,103427\_at,103922\_f\_at,160088\_at,160383\_at,160391\_at,160588\_at,160611\_at,162044\_f\_at,92392\_at,92653\_at,93421\_at,93424\_at,93440\_at,93997\_at,94948\_at,95425\_at,96603\_at,96886\_at,97496\_f\_at,97498\_at,98533\_at,99985\_at,161760\_s\_at,96186\_at,161990\_f\_at,93320\_at,96609\_at,93316\_at,104165\_at,100606\_at,100307\_at,101930\_at,103500\_at,95613\_at,97897\_at,162138\_s\_at,160399\_r\_at,93020\_at,101475\_at,104376\_at,94343\_at,94345\_at,94346\_at,100030\_at,104404\_at,160199\_at,160200\_at,94041\_at,101023\_f\_at,101079\_at,102094\_f\_at,93267\_at,96777\_at,104456\_at,160374\_r\_at,92568\_at,101943\_at,160430\_at,160739\_at,92248\_at,92249\_g\_at,92484\_at,104701\_at,102425\_at,103288\_at,97484\_at,160440\_at,100011\_at,100032\_at,100130\_at,100533\_s\_at,100924\_at,101465\_at,101502\_at,102069\_at,102209\_at,102364\_at,102371\_at,102381\_at,102384\_at,102644\_at,102657\_at,102789\_at,102920\_at,102955\_at,102983\_at,102984\_g\_at,102994\_at,102996\_at,103015\_at,103086\_at,103321\_at,103328\_at,103440\_at,103497\_at,103501\_at,103547\_at,103634\_at,103720\_at,103774\_at,103901\_at,104070\_at,104155\_f\_at,104156\_r\_at,104477\_at,104562\_at,104605\_at,104645\_at,160138\_at,160246\_at,160313\_at,160397\_at,160483\_at,160495\_at,160502\_at,160526\_s\_at,160605\_s\_at,160724\_at,160783\_at,160834\_at,160894\_at,160901\_at,161084\_at,161113\_at,161847\_r\_at,162010\_r\_at,92195\_at,92216\_at,92440\_at,92562\_at,92564\_at,92854\_at,92908\_at,92925\_at,92926\_at,92992\_i\_at,92993\_r\_at,93528\_s\_at,93619\_at,93728\_at,93793\_at,94031\_at,94319\_at,94331\_at,94356\_at,94408\_at,94469\_at,94505\_at,94689\_at,94821\_at,95521\_s\_at,95522\_i\_at,95617\_at,95618\_at,95671\_at,95673\_s\_at,96147\_at,96192\_at,96238\_at,96561\_at,96836\_r\_at,96961\_at,97118\_at,97355\_at,97859\_at,97973\_at,98083\_at,98324\_at,98427\_s\_at,98468\_r\_at,98756\_at,98988\_at,99024\_at,99076\_at,99100\_at,99103\_at,99602\_at,99603\_g\_at,99622\_at,101015\_s\_at,92344\_at,96703\_at,98122\_at,93543\_f\_at,100595\_at,101836\_at,104598\_at,92638\_at,92888\_s\_at,93179\_at,93285\_at,94980\_at,98580\_at,100417\_at,101457\_at,101936\_at,102224\_at,102332\_at,103020\_s\_at,103021\_r\_at,103416\_at,103451\_at,103969\_at,104417\_at,161067\_at,161184\_f\_at,93274\_at,93311\_at,94483\_at,95805\_at,96488\_at,96489\_at,96771\_at,96852\_at,97409\_at,97429\_at,97890\_at,97925\_at,98369\_f\_at,98504\_at,99070\_at,102414\_i\_at,102415\_r\_at,103913\_at,104453\_at,95023\_at,95444\_at,96669\_at,96736\_at,160373\_i\_at,97458\_at,104149\_at,102279\_at,160103\_at,160104\_at,93731\_at,103584\_at,94809\_at,98906\_at |
| 4 | alcohol metabolism | 15 | 167 | 1185 | 13100 | 0.013 | 0.013 | 0.993 | 0.551 | 160338\_at,101990\_at,160092\_at,161946\_r\_at,94439\_at,94815\_at,97833\_at,97834\_g\_at,99045\_at,AFFX-MUR\_b2\_at,104337\_f\_at,98984\_f\_at,98508\_s\_at,96534\_at,102768\_i\_at |
| 5 | alcohol biosynthesis | 1 | 18 | 1042 | 11544 | 0.001 | 0.002 | 0.615 | 0.818 | 160338\_at |
| 6 | monosaccharide biosynthesis | 1 | 18 | 880 | 9498 | 0.001 | 0.002 | 0.6 | 0.827 | 160338\_at |
| 7 | hexose biosynthesis | 1 | 18 | 538 | 6246 | 0.002 | 0.003 | 0.646 | 0.803 | 160338\_at |
| 8 | myo-inositol biosynthesis | 1 | 2 | 199 | 2164 | 0.005 | 0.001 | 5.467 | 0.176 | 160338\_at |
| 5 | alcohol catabolism | 9 | 58 | 1042 | 11544 | 0.009 | 0.005 | 1.721 | 0.074 | 101990\_at,160092\_at,161946\_r\_at,94439\_at,94815\_at,97833\_at,97834\_g\_at,99045\_at,AFFX-MUR\_b2\_at |
| 6 | monosaccharide catabolism | 9 | 58 | 880 | 9498 | 0.01 | 0.006 | 1.674 | 0.084 | 101990\_at,160092\_at,161946\_r\_at,94439\_at,94815\_at,97833\_at,97834\_g\_at,99045\_at,AFFX-MUR\_b2\_at |
| 7 | hexose catabolism | 9 | 58 | 538 | 6246 | 0.017 | 0.009 | 1.801 | 0.058 | 101990\_at,160092\_at,161946\_r\_at,94439\_at,94815\_at,97833\_at,97834\_g\_at,99045\_at,AFFX-MUR\_b2\_at |
| 8 | glucose catabolism | 9 | 58 | 199 | 2164 | 0.045 | 0.027 | 1.688 | 0.079 | 101990\_at,160092\_at,161946\_r\_at,94439\_at,94815\_at,97833\_at,97834\_g\_at,99045\_at,AFFX-MUR\_b2\_at |
| 9 | glycolysis | 9 | 52 | 72 | 911 | 0.125 | 0.057 | 2.19 | 0.017 | 101990\_at,160092\_at,161946\_r\_at,94439\_at,94815\_at,97833\_at,97834\_g\_at,99045\_at,AFFX-MUR\_b2\_at |
| 5 | monosaccharide metabolism | 11 | 108 | 1042 | 11544 | 0.011 | 0.009 | 1.128 | 0.384 | 160338\_at,101990\_at,160092\_at,161946\_r\_at,94439\_at,94815\_at,97833\_at,97834\_g\_at,99045\_at,AFFX-MUR\_b2\_at,104337\_f\_at |
| 6 | monosaccharide biosynthesis | 1 | 18 | 880 | 9498 | 0.001 | 0.002 | 0.6 | 0.827 | 160338\_at |
| 7 | hexose biosynthesis | 1 | 18 | 538 | 6246 | 0.002 | 0.003 | 0.646 | 0.803 | 160338\_at |
| 8 | myo-inositol biosynthesis | 1 | 2 | 199 | 2164 | 0.005 | 0.001 | 5.467 | 0.176 | 160338\_at |
| 6 | monosaccharide catabolism | 9 | 58 | 880 | 9498 | 0.01 | 0.006 | 1.674 | 0.084 | 101990\_at,160092\_at,161946\_r\_at,94439\_at,94815\_at,97833\_at,97834\_g\_at,99045\_at,AFFX-MUR\_b2\_at |
| 7 | hexose catabolism | 9 | 58 | 538 | 6246 | 0.017 | 0.009 | 1.801 | 0.058 | 101990\_at,160092\_at,161946\_r\_at,94439\_at,94815\_at,97833\_at,97834\_g\_at,99045\_at,AFFX-MUR\_b2\_at |
| 8 | glucose catabolism | 9 | 58 | 199 | 2164 | 0.045 | 0.027 | 1.688 | 0.079 | 101990\_at,160092\_at,161946\_r\_at,94439\_at,94815\_at,97833\_at,97834\_g\_at,99045\_at,AFFX-MUR\_b2\_at |
| 9 | glycolysis | 9 | 52 | 72 | 911 | 0.125 | 0.057 | 2.19 | 0.017 | 101990\_at,160092\_at,161946\_r\_at,94439\_at,94815\_at,97833\_at,97834\_g\_at,99045\_at,AFFX-MUR\_b2\_at |
| 6 | pentose metabolism | 1 | 1 | 880 | 9498 | 0.001 | 0 | 10.364 | 0.093 | 104337\_f\_at |
| 7 | D-ribose metabolism | 1 | 1 | 538 | 6246 | 0.002 | 0 | 11.625 | 0.086 | 104337\_f\_at |
| 5 | polyol metabolism | 1 | 7 | 1042 | 11544 | 0.001 | 0.001 | 1.574 | 0.484 | 98984\_f\_at |
| 6 | glycerol metabolism | 1 | 7 | 880 | 9498 | 0.001 | 0.001 | 1.541 | 0.494 | 98984\_f\_at |
| 7 | glycerol-3-phosphate metabolism | 1 | 5 | 538 | 6246 | 0.002 | 0.001 | 2.325 | 0.363 | 98984\_f\_at |
| 5 | sphingosine metabolism | 1 | 3 | 1042 | 11544 | 0.001 | 0 | 3.692 | 0.247 | 98508\_s\_at |
| 5 | sterol metabolism | 2 | 36 | 1042 | 11544 | 0.002 | 0.003 | 0.615 | 0.849 | 96534\_at,102768\_i\_at |
| 6 | cholesterol metabolism | 1 | 33 | 880 | 9498 | 0.001 | 0.003 | 0.329 | 0.96 | 96534\_at |
| 6 | sterol biosynthesis | 1 | 20 | 880 | 9498 | 0.001 | 0.002 | 0.54 | 0.857 | 102768\_i\_at |
| 4 | amine metabolism | 16 | 148 | 1185 | 13100 | 0.014 | 0.011 | 1.195 | 0.263 | 98508\_s\_at,95135\_at,94415\_at,93177\_at,160084\_at,101490\_at,102302\_at,99184\_at,96657\_at,92586\_at,102360\_at,103739\_at,98931\_at,101000\_at,101001\_at,160603\_at |
| 5 | sphingosine metabolism | 1 | 3 | 1042 | 11544 | 0.001 | 0 | 3.692 | 0.247 | 98508\_s\_at |
| 5 | amine biosynthesis | 5 | 35 | 1042 | 11544 | 0.005 | 0.003 | 1.584 | 0.205 | 95135\_at,94415\_at,93177\_at,160084\_at,101490\_at |
| 6 | amino acid biosynthesis | 3 | 25 | 880 | 9498 | 0.003 | 0.003 | 1.297 | 0.412 | 95135\_at,94415\_at,93177\_at |
| 7 | aspartate family amino acid biosynthesis | 1 | 6 | 538 | 6246 | 0.002 | 0.001 | 1.938 | 0.418 | 95135\_at |
| 8 | asparagine biosynthesis | 1 | 2 | 199 | 2164 | 0.005 | 0.001 | 5.467 | 0.176 | 95135\_at |
| 7 | glutamine family amino acid biosynthesis | 1 | 7 | 538 | 6246 | 0.002 | 0.001 | 1.661 | 0.468 | 94415\_at |
| 8 | arginine biosynthesis | 1 | 4 | 199 | 2164 | 0.005 | 0.002 | 2.719 | 0.32 | 94415\_at |
| 7 | serine family amino acid biosynthesis | 1 | 8 | 538 | 6246 | 0.002 | 0.001 | 1.453 | 0.514 | 93177\_at |
| 8 | L-serine biosynthesis | 1 | 7 | 199 | 2164 | 0.005 | 0.003 | 1.557 | 0.491 | 93177\_at |
| 6 | biogenic amine biosynthesis | 2 | 10 | 880 | 9498 | 0.002 | 0.001 | 2.162 | 0.236 | 160084\_at,101490\_at |
| 7 | polyamine biosynthesis | 2 | 6 | 538 | 6246 | 0.004 | 0.001 | 3.875 | 0.088 | 160084\_at,101490\_at |
| 8 | spermidine biosynthesis | 1 | 3 | 199 | 2164 | 0.005 | 0.001 | 3.619 | 0.251 | 101490\_at |
| 8 | spermine biosynthesis | 1 | 2 | 199 | 2164 | 0.005 | 0.001 | 5.467 | 0.176 | 101490\_at |
| 5 | amine catabolism | 3 | 30 | 1042 | 11544 | 0.003 | 0.003 | 1.108 | 0.517 | 102302\_at,99184\_at,96657\_at |
| 6 | amino acid catabolism | 2 | 24 | 880 | 9498 | 0.002 | 0.003 | 0.897 | 0.666 | 102302\_at,99184\_at |
| 7 | serine family amino acid catabolism | 1 | 4 | 538 | 6246 | 0.002 | 0.001 | 2.906 | 0.303 | 99184\_at |
| 8 | cysteine catabolism | 1 | 2 | 199 | 2164 | 0.005 | 0.001 | 5.467 | 0.176 | 99184\_at |
| 9 | L-cysteine catabolism | 1 | 2 | 72 | 911 | 0.014 | 0.002 | 6.314 | 0.152 | 99184\_at |
| 10 | L-cysteine catabolism to taurine | 1 | 2 | 15 | 197 | 0.067 | 0.01 | 6.568 | 0.147 | 99184\_at |
| 6 | biogenic amine catabolism | 1 | 8 | 880 | 9498 | 0.001 | 0.001 | 1.357 | 0.541 | 96657\_at |
| 7 | polyamine catabolism | 1 | 1 | 538 | 6246 | 0.002 | 0 | 11.625 | 0.086 | 96657\_at |
| 8 | spermine catabolism | 1 | 1 | 199 | 2164 | 0.005 | 0 | 10.935 | 0.092 | 96657\_at |
| 5 | amino acid metabolism | 9 | 97 | 1042 | 11544 | 0.009 | 0.008 | 1.029 | 0.517 | 160084\_at,92586\_at,94415\_at,99184\_at,95135\_at,93177\_at,102302\_at,102360\_at,103739\_at |
| 6 | amino acid biosynthesis | 3 | 25 | 880 | 9498 | 0.003 | 0.003 | 1.297 | 0.412 | 95135\_at,94415\_at,93177\_at |
| 7 | aspartate family amino acid biosynthesis | 1 | 6 | 538 | 6246 | 0.002 | 0.001 | 1.938 | 0.418 | 95135\_at |
| 8 | asparagine biosynthesis | 1 | 2 | 199 | 2164 | 0.005 | 0.001 | 5.467 | 0.176 | 95135\_at |
| 7 | glutamine family amino acid biosynthesis | 1 | 7 | 538 | 6246 | 0.002 | 0.001 | 1.661 | 0.468 | 94415\_at |
| 8 | arginine biosynthesis | 1 | 4 | 199 | 2164 | 0.005 | 0.002 | 2.719 | 0.32 | 94415\_at |
| 7 | serine family amino acid biosynthesis | 1 | 8 | 538 | 6246 | 0.002 | 0.001 | 1.453 | 0.514 | 93177\_at |
| 8 | L-serine biosynthesis | 1 | 7 | 199 | 2164 | 0.005 | 0.003 | 1.557 | 0.491 | 93177\_at |
| 6 | amino acid catabolism | 2 | 24 | 880 | 9498 | 0.002 | 0.003 | 0.897 | 0.666 | 102302\_at,99184\_at |
| 7 | serine family amino acid catabolism | 1 | 4 | 538 | 6246 | 0.002 | 0.001 | 2.906 | 0.303 | 99184\_at |
| 8 | cysteine catabolism | 1 | 2 | 199 | 2164 | 0.005 | 0.001 | 5.467 | 0.176 | 99184\_at |
| 9 | L-cysteine catabolism | 1 | 2 | 72 | 911 | 0.014 | 0.002 | 6.314 | 0.152 | 99184\_at |
| 10 | L-cysteine catabolism to taurine | 1 | 2 | 15 | 197 | 0.067 | 0.01 | 6.568 | 0.147 | 99184\_at |
| 6 | aspartate family amino acid metabolism | 2 | 7 | 880 | 9498 | 0.002 | 0.001 | 3.068 | 0.132 | 95135\_at,102360\_at |
| 7 | aspartate family amino acid biosynthesis | 1 | 6 | 538 | 6246 | 0.002 | 0.001 | 1.938 | 0.418 | 95135\_at |
| 8 | asparagine biosynthesis | 1 | 2 | 199 | 2164 | 0.005 | 0.001 | 5.467 | 0.176 | 95135\_at |
| 7 | methionine metabolism | 1 | 5 | 538 | 6246 | 0.002 | 0.001 | 2.325 | 0.363 | 102360\_at |
| 6 | glutamine family amino acid metabolism | 3 | 15 | 880 | 9498 | 0.003 | 0.002 | 2.158 | 0.156 | 94415\_at,103739\_at,95135\_at |
| 7 | glutamine family amino acid biosynthesis | 1 | 7 | 538 | 6246 | 0.002 | 0.001 | 1.661 | 0.468 | 94415\_at |
| 8 | arginine biosynthesis | 1 | 4 | 199 | 2164 | 0.005 | 0.002 | 2.719 | 0.32 | 94415\_at |
| 7 | glutamine metabolism | 2 | 6 | 538 | 6246 | 0.004 | 0.001 | 3.875 | 0.088 | 103739\_at,95135\_at |
| 5 | aminoglycan metabolism | 1 | 7 | 1042 | 11544 | 0.001 | 0.001 | 1.574 | 0.484 | 98931\_at |
| 6 | aminoglycan catabolism | 1 | 3 | 880 | 9498 | 0.001 | 0 | 3.562 | 0.253 | 98931\_at |
| 7 | glycosaminoglycan catabolism | 1 | 3 | 538 | 6246 | 0.002 | 0 | 3.875 | 0.237 | 98931\_at |
| 6 | glycosaminoglycan metabolism | 1 | 7 | 880 | 9498 | 0.001 | 0.001 | 1.541 | 0.494 | 98931\_at |
| 7 | glycosaminoglycan catabolism | 1 | 3 | 538 | 6246 | 0.002 | 0 | 3.875 | 0.237 | 98931\_at |
| 5 | biogenic amine metabolism | 6 | 35 | 1042 | 11544 | 0.006 | 0.003 | 1.901 | 0.091 | 160084\_at,101490\_at,96657\_at,101000\_at,101001\_at,160603\_at |
| 6 | biogenic amine biosynthesis | 2 | 10 | 880 | 9498 | 0.002 | 0.001 | 2.162 | 0.236 | 160084\_at,101490\_at |
| 7 | polyamine biosynthesis | 2 | 6 | 538 | 6246 | 0.004 | 0.001 | 3.875 | 0.088 | 160084\_at,101490\_at |
| 8 | spermidine biosynthesis | 1 | 3 | 199 | 2164 | 0.005 | 0.001 | 3.619 | 0.251 | 101490\_at |
| 8 | spermine biosynthesis | 1 | 2 | 199 | 2164 | 0.005 | 0.001 | 5.467 | 0.176 | 101490\_at |
| 6 | biogenic amine catabolism | 1 | 8 | 880 | 9498 | 0.001 | 0.001 | 1.357 | 0.541 | 96657\_at |
| 7 | polyamine catabolism | 1 | 1 | 538 | 6246 | 0.002 | 0 | 11.625 | 0.086 | 96657\_at |
| 8 | spermine catabolism | 1 | 1 | 199 | 2164 | 0.005 | 0 | 10.935 | 0.092 | 96657\_at |
| 6 | polyamine metabolism | 5 | 13 | 880 | 9498 | 0.006 | 0.001 | 4.146 | 0.005 | 101000\_at,101001\_at,160084\_at,101490\_at,96657\_at |
| 7 | polyamine biosynthesis | 2 | 6 | 538 | 6246 | 0.004 | 0.001 | 3.875 | 0.088 | 160084\_at,101490\_at |
| 8 | spermidine biosynthesis | 1 | 3 | 199 | 2164 | 0.005 | 0.001 | 3.619 | 0.251 | 101490\_at |
| 8 | spermine biosynthesis | 1 | 2 | 199 | 2164 | 0.005 | 0.001 | 5.467 | 0.176 | 101490\_at |
| 7 | polyamine catabolism | 1 | 1 | 538 | 6246 | 0.002 | 0 | 11.625 | 0.086 | 96657\_at |
| 8 | spermine catabolism | 1 | 1 | 199 | 2164 | 0.005 | 0 | 10.935 | 0.092 | 96657\_at |
| 6 | thyroid hormone metabolism | 1 | 3 | 880 | 9498 | 0.001 | 0 | 3.562 | 0.253 | 160603\_at |
| 7 | thyroid hormone generation | 1 | 3 | 538 | 6246 | 0.002 | 0 | 3.875 | 0.237 | 160603\_at |
| 4 | amino acid and derivative metabolism | 14 | 134 | 1185 | 13100 | 0.012 | 0.01 | 1.154 | 0.326 | 160084\_at,92586\_at,94415\_at,99184\_at,95135\_at,93177\_at,102302\_at,102360\_at,103739\_at,101490\_at,96657\_at,101000\_at,101001\_at,160603\_at |
| 5 | amino acid metabolism | 9 | 97 | 1042 | 11544 | 0.009 | 0.008 | 1.029 | 0.517 | 160084\_at,92586\_at,94415\_at,99184\_at,95135\_at,93177\_at,102302\_at,102360\_at,103739\_at |
| 6 | amino acid biosynthesis | 3 | 25 | 880 | 9498 | 0.003 | 0.003 | 1.297 | 0.412 | 95135\_at,94415\_at,93177\_at |
| 7 | aspartate family amino acid biosynthesis | 1 | 6 | 538 | 6246 | 0.002 | 0.001 | 1.938 | 0.418 | 95135\_at |
| 8 | asparagine biosynthesis | 1 | 2 | 199 | 2164 | 0.005 | 0.001 | 5.467 | 0.176 | 95135\_at |
| 7 | glutamine family amino acid biosynthesis | 1 | 7 | 538 | 6246 | 0.002 | 0.001 | 1.661 | 0.468 | 94415\_at |
| 8 | arginine biosynthesis | 1 | 4 | 199 | 2164 | 0.005 | 0.002 | 2.719 | 0.32 | 94415\_at |
| 7 | serine family amino acid biosynthesis | 1 | 8 | 538 | 6246 | 0.002 | 0.001 | 1.453 | 0.514 | 93177\_at |
| 8 | L-serine biosynthesis | 1 | 7 | 199 | 2164 | 0.005 | 0.003 | 1.557 | 0.491 | 93177\_at |
| 6 | amino acid catabolism | 2 | 24 | 880 | 9498 | 0.002 | 0.003 | 0.897 | 0.666 | 102302\_at,99184\_at |
| 7 | serine family amino acid catabolism | 1 | 4 | 538 | 6246 | 0.002 | 0.001 | 2.906 | 0.303 | 99184\_at |
| 8 | cysteine catabolism | 1 | 2 | 199 | 2164 | 0.005 | 0.001 | 5.467 | 0.176 | 99184\_at |
| 9 | L-cysteine catabolism | 1 | 2 | 72 | 911 | 0.014 | 0.002 | 6.314 | 0.152 | 99184\_at |
| 10 | L-cysteine catabolism to taurine | 1 | 2 | 15 | 197 | 0.067 | 0.01 | 6.568 | 0.147 | 99184\_at |
| 6 | aspartate family amino acid metabolism | 2 | 7 | 880 | 9498 | 0.002 | 0.001 | 3.068 | 0.132 | 95135\_at,102360\_at |
| 7 | aspartate family amino acid biosynthesis | 1 | 6 | 538 | 6246 | 0.002 | 0.001 | 1.938 | 0.418 | 95135\_at |
| 8 | asparagine biosynthesis | 1 | 2 | 199 | 2164 | 0.005 | 0.001 | 5.467 | 0.176 | 95135\_at |
| 7 | methionine metabolism | 1 | 5 | 538 | 6246 | 0.002 | 0.001 | 2.325 | 0.363 | 102360\_at |
| 6 | glutamine family amino acid metabolism | 3 | 15 | 880 | 9498 | 0.003 | 0.002 | 2.158 | 0.156 | 94415\_at,103739\_at,95135\_at |
| 7 | glutamine family amino acid biosynthesis | 1 | 7 | 538 | 6246 | 0.002 | 0.001 | 1.661 | 0.468 | 94415\_at |
| 8 | arginine biosynthesis | 1 | 4 | 199 | 2164 | 0.005 | 0.002 | 2.719 | 0.32 | 94415\_at |
| 7 | glutamine metabolism | 2 | 6 | 538 | 6246 | 0.004 | 0.001 | 3.875 | 0.088 | 103739\_at,95135\_at |
| 5 | amino acid derivative metabolism | 7 | 50 | 1042 | 11544 | 0.007 | 0.004 | 1.552 | 0.161 | 160084\_at,101490\_at,96657\_at,101000\_at,101001\_at,160603\_at,99184\_at |
| 6 | biogenic amine biosynthesis | 2 | 10 | 880 | 9498 | 0.002 | 0.001 | 2.162 | 0.236 | 160084\_at,101490\_at |
| 7 | polyamine biosynthesis | 2 | 6 | 538 | 6246 | 0.004 | 0.001 | 3.875 | 0.088 | 160084\_at,101490\_at |
| 8 | spermidine biosynthesis | 1 | 3 | 199 | 2164 | 0.005 | 0.001 | 3.619 | 0.251 | 101490\_at |
| 8 | spermine biosynthesis | 1 | 2 | 199 | 2164 | 0.005 | 0.001 | 5.467 | 0.176 | 101490\_at |
| 6 | biogenic amine catabolism | 1 | 8 | 880 | 9498 | 0.001 | 0.001 | 1.357 | 0.541 | 96657\_at |
| 7 | polyamine catabolism | 1 | 1 | 538 | 6246 | 0.002 | 0 | 11.625 | 0.086 | 96657\_at |
| 8 | spermine catabolism | 1 | 1 | 199 | 2164 | 0.005 | 0 | 10.935 | 0.092 | 96657\_at |
| 6 | polyamine metabolism | 5 | 13 | 880 | 9498 | 0.006 | 0.001 | 4.146 | 0.005 | 101000\_at,101001\_at,160084\_at,101490\_at,96657\_at |
| 7 | polyamine biosynthesis | 2 | 6 | 538 | 6246 | 0.004 | 0.001 | 3.875 | 0.088 | 160084\_at,101490\_at |
| 8 | spermidine biosynthesis | 1 | 3 | 199 | 2164 | 0.005 | 0.001 | 3.619 | 0.251 | 101490\_at |
| 8 | spermine biosynthesis | 1 | 2 | 199 | 2164 | 0.005 | 0.001 | 5.467 | 0.176 | 101490\_at |
| 7 | polyamine catabolism | 1 | 1 | 538 | 6246 | 0.002 | 0 | 11.625 | 0.086 | 96657\_at |
| 8 | spermine catabolism | 1 | 1 | 199 | 2164 | 0.005 | 0 | 10.935 | 0.092 | 96657\_at |
| 6 | thyroid hormone metabolism | 1 | 3 | 880 | 9498 | 0.001 | 0 | 3.562 | 0.253 | 160603\_at |
| 7 | thyroid hormone generation | 1 | 3 | 538 | 6246 | 0.002 | 0 | 3.875 | 0.237 | 160603\_at |
| 6 | taurine metabolism | 1 | 2 | 880 | 9498 | 0.001 | 0 | 5.429 | 0.177 | 99184\_at |
| 5 | biogenic amine metabolism | 6 | 35 | 1042 | 11544 | 0.006 | 0.003 | 1.901 | 0.091 | 160084\_at,101490\_at,96657\_at,101000\_at,101001\_at,160603\_at |
| 6 | biogenic amine biosynthesis | 2 | 10 | 880 | 9498 | 0.002 | 0.001 | 2.162 | 0.236 | 160084\_at,101490\_at |
| 7 | polyamine biosynthesis | 2 | 6 | 538 | 6246 | 0.004 | 0.001 | 3.875 | 0.088 | 160084\_at,101490\_at |
| 8 | spermidine biosynthesis | 1 | 3 | 199 | 2164 | 0.005 | 0.001 | 3.619 | 0.251 | 101490\_at |
| 8 | spermine biosynthesis | 1 | 2 | 199 | 2164 | 0.005 | 0.001 | 5.467 | 0.176 | 101490\_at |
| 6 | biogenic amine catabolism | 1 | 8 | 880 | 9498 | 0.001 | 0.001 | 1.357 | 0.541 | 96657\_at |
| 7 | polyamine catabolism | 1 | 1 | 538 | 6246 | 0.002 | 0 | 11.625 | 0.086 | 96657\_at |
| 8 | spermine catabolism | 1 | 1 | 199 | 2164 | 0.005 | 0 | 10.935 | 0.092 | 96657\_at |
| 6 | polyamine metabolism | 5 | 13 | 880 | 9498 | 0.006 | 0.001 | 4.146 | 0.005 | 101000\_at,101001\_at,160084\_at,101490\_at,96657\_at |
| 7 | polyamine biosynthesis | 2 | 6 | 538 | 6246 | 0.004 | 0.001 | 3.875 | 0.088 | 160084\_at,101490\_at |
| 8 | spermidine biosynthesis | 1 | 3 | 199 | 2164 | 0.005 | 0.001 | 3.619 | 0.251 | 101490\_at |
| 8 | spermine biosynthesis | 1 | 2 | 199 | 2164 | 0.005 | 0.001 | 5.467 | 0.176 | 101490\_at |
| 7 | polyamine catabolism | 1 | 1 | 538 | 6246 | 0.002 | 0 | 11.625 | 0.086 | 96657\_at |
| 8 | spermine catabolism | 1 | 1 | 199 | 2164 | 0.005 | 0 | 10.935 | 0.092 | 96657\_at |
| 6 | thyroid hormone metabolism | 1 | 3 | 880 | 9498 | 0.001 | 0 | 3.562 | 0.253 | 160603\_at |
| 7 | thyroid hormone generation | 1 | 3 | 538 | 6246 | 0.002 | 0 | 3.875 | 0.237 | 160603\_at |
| 4 | aromatic compound metabolism | 1 | 65 | 1185 | 13100 | 0.001 | 0.005 | 0.169 | 0.998 | 102313\_at |
| 5 | aromatic compound biosynthesis | 1 | 7 | 1042 | 11544 | 0.001 | 0.001 | 1.574 | 0.484 | 102313\_at |
| 6 | pteridine and derivative biosynthesis | 1 | 7 | 880 | 9498 | 0.001 | 0.001 | 1.541 | 0.494 | 102313\_at |
| 7 | tetrahydrobiopterin biosynthesis | 1 | 4 | 538 | 6246 | 0.002 | 0.001 | 2.906 | 0.303 | 102313\_at |
| 4 | biosynthesis | 51 | 652 | 1185 | 13100 | 0.043 | 0.05 | 0.865 | 0.884 | 102313\_at,160338\_at,95135\_at,94415\_at,93177\_at,160084\_at,101490\_at,103739\_at,101186\_at,160124\_r\_at,94043\_at,96951\_at,160335\_at,103471\_at,99849\_at,98508\_s\_at,97829\_at,101681\_f\_at,102292\_at,103674\_f\_at,103994\_at,104049\_at,104144\_at,160111\_at,160112\_at,160130\_at,160976\_at,160977\_at,161342\_r\_at,161666\_f\_at,92855\_at,93975\_at,94499\_at,95070\_at,95737\_at,96325\_at,100136\_at,101590\_at,95681\_f\_at,96628\_at,96725\_at,93315\_at,160137\_at,94433\_at,160579\_at,94818\_at,93852\_at,97798\_at,94976\_at,96299\_at,102321\_at |
| 5 | alcohol biosynthesis | 1 | 18 | 1042 | 11544 | 0.001 | 0.002 | 0.615 | 0.818 | 160338\_at |
| 6 | monosaccharide biosynthesis | 1 | 18 | 880 | 9498 | 0.001 | 0.002 | 0.6 | 0.827 | 160338\_at |
| 7 | hexose biosynthesis | 1 | 18 | 538 | 6246 | 0.002 | 0.003 | 0.646 | 0.803 | 160338\_at |
| 8 | myo-inositol biosynthesis | 1 | 2 | 199 | 2164 | 0.005 | 0.001 | 5.467 | 0.176 | 160338\_at |
| 5 | amine biosynthesis | 5 | 35 | 1042 | 11544 | 0.005 | 0.003 | 1.584 | 0.205 | 95135\_at,94415\_at,93177\_at,160084\_at,101490\_at |
| 6 | amino acid biosynthesis | 3 | 25 | 880 | 9498 | 0.003 | 0.003 | 1.297 | 0.412 | 95135\_at,94415\_at,93177\_at |
| 7 | aspartate family amino acid biosynthesis | 1 | 6 | 538 | 6246 | 0.002 | 0.001 | 1.938 | 0.418 | 95135\_at |
| 8 | asparagine biosynthesis | 1 | 2 | 199 | 2164 | 0.005 | 0.001 | 5.467 | 0.176 | 95135\_at |
| 7 | glutamine family amino acid biosynthesis | 1 | 7 | 538 | 6246 | 0.002 | 0.001 | 1.661 | 0.468 | 94415\_at |
| 8 | arginine biosynthesis | 1 | 4 | 199 | 2164 | 0.005 | 0.002 | 2.719 | 0.32 | 94415\_at |
| 7 | serine family amino acid biosynthesis | 1 | 8 | 538 | 6246 | 0.002 | 0.001 | 1.453 | 0.514 | 93177\_at |
| 8 | L-serine biosynthesis | 1 | 7 | 199 | 2164 | 0.005 | 0.003 | 1.557 | 0.491 | 93177\_at |
| 6 | biogenic amine biosynthesis | 2 | 10 | 880 | 9498 | 0.002 | 0.001 | 2.162 | 0.236 | 160084\_at,101490\_at |
| 7 | polyamine biosynthesis | 2 | 6 | 538 | 6246 | 0.004 | 0.001 | 3.875 | 0.088 | 160084\_at,101490\_at |
| 8 | spermidine biosynthesis | 1 | 3 | 199 | 2164 | 0.005 | 0.001 | 3.619 | 0.251 | 101490\_at |
| 8 | spermine biosynthesis | 1 | 2 | 199 | 2164 | 0.005 | 0.001 | 5.467 | 0.176 | 101490\_at |
| 5 | aromatic compound biosynthesis | 1 | 7 | 1042 | 11544 | 0.001 | 0.001 | 1.574 | 0.484 | 102313\_at |
| 6 | pteridine and derivative biosynthesis | 1 | 7 | 880 | 9498 | 0.001 | 0.001 | 1.541 | 0.494 | 102313\_at |
| 7 | tetrahydrobiopterin biosynthesis | 1 | 4 | 538 | 6246 | 0.002 | 0.001 | 2.906 | 0.303 | 102313\_at |
| 5 | carbohydrate biosynthesis | 3 | 48 | 1042 | 11544 | 0.003 | 0.004 | 0.692 | 0.821 | 103739\_at,160338\_at,101186\_at |
| 6 | monosaccharide biosynthesis | 1 | 18 | 880 | 9498 | 0.001 | 0.002 | 0.6 | 0.827 | 160338\_at |
| 7 | hexose biosynthesis | 1 | 18 | 538 | 6246 | 0.002 | 0.003 | 0.646 | 0.803 | 160338\_at |
| 8 | myo-inositol biosynthesis | 1 | 2 | 199 | 2164 | 0.005 | 0.001 | 5.467 | 0.176 | 160338\_at |
| 6 | proteoglycan biosynthesis | 1 | 4 | 880 | 9498 | 0.001 | 0 | 2.714 | 0.322 | 101186\_at |
| 5 | coenzymes and prosthetic group biosynthesis | 4 | 51 | 1042 | 11544 | 0.004 | 0.004 | 0.869 | 0.688 | 160124\_r\_at,94043\_at,96951\_at,160335\_at |
| 6 | coenzyme biosynthesis | 4 | 40 | 880 | 9498 | 0.005 | 0.004 | 1.081 | 0.515 | 160124\_r\_at,94043\_at,96951\_at,160335\_at |
| 7 | ATP biosynthesis | 3 | 24 | 538 | 6246 | 0.006 | 0.004 | 1.453 | 0.342 | 160124\_r\_at,94043\_at,96951\_at |
| 7 | glutathione biosynthesis | 1 | 5 | 538 | 6246 | 0.002 | 0.001 | 2.325 | 0.363 | 160335\_at |
| 5 | lipid biosynthesis | 5 | 124 | 1042 | 11544 | 0.005 | 0.011 | 0.447 | 0.99 | 103471\_at,99849\_at,98508\_s\_at,160338\_at,97829\_at |
| 6 | fatty acid biosynthesis | 2 | 40 | 880 | 9498 | 0.002 | 0.004 | 0.539 | 0.896 | 103471\_at,99849\_at |
| 7 | eicosanoid biosynthesis | 1 | 19 | 538 | 6246 | 0.002 | 0.003 | 0.612 | 0.82 | 99849\_at |
| 8 | leukotriene biosynthesis | 1 | 10 | 199 | 2164 | 0.005 | 0.005 | 1.089 | 0.62 | 99849\_at |
| 6 | glycerolipid biosynthesis | 1 | 3 | 880 | 9498 | 0.001 | 0 | 3.562 | 0.253 | 98508\_s\_at |
| 7 | acylglycerol biosynthesis | 1 | 2 | 538 | 6246 | 0.002 | 0 | 5.812 | 0.165 | 98508\_s\_at |
| 8 | diacylglycerol biosynthesis | 1 | 2 | 199 | 2164 | 0.005 | 0.001 | 5.467 | 0.176 | 98508\_s\_at |
| 6 | membrane lipid biosynthesis | 2 | 20 | 880 | 9498 | 0.002 | 0.002 | 1.076 | 0.565 | 160338\_at,97829\_at |
| 7 | phospholipid biosynthesis | 2 | 14 | 538 | 6246 | 0.004 | 0.002 | 1.661 | 0.343 | 160338\_at,97829\_at |
| 5 | macromolecule biosynthesis | 32 | 322 | 1042 | 11544 | 0.031 | 0.028 | 1.101 | 0.309 | 101681\_f\_at,102292\_at,103674\_f\_at,103994\_at,104049\_at,104144\_at,160111\_at,160112\_at,160130\_at,160976\_at,160977\_at,161342\_r\_at,161666\_f\_at,92855\_at,93975\_at,94499\_at,95070\_at,95737\_at,96325\_at,100136\_at,101590\_at,95681\_f\_at,96628\_at,96725\_at,93315\_at,160137\_at,94433\_at,160579\_at,94818\_at,93852\_at,97798\_at,94976\_at |
| 6 | protein biosynthesis | 32 | 322 | 880 | 9498 | 0.036 | 0.034 | 1.073 | 0.364 | 101681\_f\_at,102292\_at,103674\_f\_at,103994\_at,104049\_at,104144\_at,160111\_at,160112\_at,160130\_at,160976\_at,160977\_at,161342\_r\_at,161666\_f\_at,92855\_at,93975\_at,94499\_at,95070\_at,95737\_at,96325\_at,100136\_at,101590\_at,95681\_f\_at,96628\_at,96725\_at,93315\_at,160137\_at,94433\_at,160579\_at,94818\_at,93852\_at,97798\_at,94976\_at |
| 7 | amino acid activation | 9 | 36 | 538 | 6246 | 0.017 | 0.006 | 2.905 | 0.003 | 100136\_at,101590\_at,95070\_at,95681\_f\_at,96325\_at,96628\_at,96725\_at,104049\_at,94499\_at |
| 8 | aspartyl-tRNA aminoacylation | 1 | 2 | 199 | 2164 | 0.005 | 0.001 | 5.467 | 0.176 | 95070\_at |
| 8 | cysteinyl-tRNA aminoacylation | 1 | 1 | 199 | 2164 | 0.005 | 0 | 10.935 | 0.092 | 104049\_at |
| 8 | lysyl-tRNA aminoacylation | 1 | 1 | 199 | 2164 | 0.005 | 0 | 10.935 | 0.092 | 96325\_at |
| 8 | phenylalanyl-tRNA aminoacylation | 1 | 2 | 199 | 2164 | 0.005 | 0.001 | 5.467 | 0.176 | 94499\_at |
| 7 | cytokine biosynthesis | 1 | 4 | 538 | 6246 | 0.002 | 0.001 | 2.906 | 0.303 | 93315\_at |
| 8 | regulation of cytokine biosynthesis | 1 | 3 | 199 | 2164 | 0.005 | 0.001 | 3.619 | 0.251 | 93315\_at |
| 7 | glycoprotein biosynthesis | 4 | 52 | 538 | 6246 | 0.007 | 0.008 | 0.892 | 0.667 | 160137\_at,94433\_at,160579\_at,94818\_at |
| 8 | protein amino acid glycosylation | 4 | 52 | 199 | 2164 | 0.02 | 0.024 | 0.836 | 0.719 | 160137\_at,94433\_at,160579\_at,94818\_at |
| 9 | N-linked glycosylation | 1 | 12 | 72 | 911 | 0.014 | 0.013 | 1.055 | 0.63 | 160579\_at |
| 9 | O-linked glycosylation | 1 | 4 | 72 | 911 | 0.014 | 0.004 | 3.164 | 0.281 | 94818\_at |
| 7 | lipoprotein biosynthesis | 2 | 16 | 538 | 6246 | 0.004 | 0.003 | 1.453 | 0.407 | 93852\_at,97798\_at |
| 8 | protein lipidation | 2 | 16 | 199 | 2164 | 0.01 | 0.007 | 1.36 | 0.441 | 93852\_at,97798\_at |
| 9 | protein prenylation | 2 | 10 | 72 | 911 | 0.028 | 0.011 | 2.53 | 0.184 | 93852\_at,97798\_at |
| 10 | protein amino acid prenylation | 1 | 6 | 15 | 197 | 0.067 | 0.03 | 2.189 | 0.382 | 93852\_at |
| 7 | regulation of protein biosynthesis | 1 | 7 | 538 | 6246 | 0.002 | 0.001 | 1.661 | 0.468 | 92855\_at |
| 7 | regulation of translation | 3 | 24 | 538 | 6246 | 0.006 | 0.004 | 1.453 | 0.342 | 92855\_at,94976\_at,161342\_r\_at |
| 8 | regulation of translational initiation | 1 | 13 | 199 | 2164 | 0.005 | 0.006 | 0.837 | 0.716 | 161342\_r\_at |
| 7 | translational elongation | 3 | 22 | 538 | 6246 | 0.006 | 0.004 | 1.585 | 0.293 | 103674\_f\_at,104144\_at,160130\_at |
| 7 | translational initiation | 5 | 34 | 538 | 6246 | 0.009 | 0.005 | 1.708 | 0.164 | 160111\_at,160112\_at,92855\_at,93975\_at,161342\_r\_at |
| 8 | regulation of translational initiation | 1 | 13 | 199 | 2164 | 0.005 | 0.006 | 0.837 | 0.716 | 161342\_r\_at |
| 5 | nitric oxide biosynthesis | 1 | 5 | 1042 | 11544 | 0.001 | 0 | 2.233 | 0.377 | 96299\_at |
| 6 | regulation of nitric oxide biosynthesis | 1 | 1 | 880 | 9498 | 0.001 | 0 | 10.364 | 0.093 | 96299\_at |
| 7 | negative regulation of nitric oxide biosynthesis | 1 | 1 | 538 | 6246 | 0.002 | 0 | 11.625 | 0.086 | 96299\_at |
| 5 | nucleotide biosynthesis | 1 | 74 | 1042 | 11544 | 0.001 | 0.006 | 0.15 | 0.999 | 102321\_at |
| 6 | cyclic nucleotide biosynthesis | 1 | 8 | 880 | 9498 | 0.001 | 0.001 | 1.357 | 0.541 | 102321\_at |
| 7 | cAMP biosynthesis | 1 | 7 | 538 | 6246 | 0.002 | 0.001 | 1.661 | 0.468 | 102321\_at |
| 4 | carbohydrate metabolism | 21 | 231 | 1185 | 13100 | 0.018 | 0.018 | 1.005 | 0.524 | 100522\_s\_at,100523\_r\_at,102322\_at,103739\_at,160579\_at,94872\_at,99133\_at,160338\_at,101990\_at,160092\_at,161946\_r\_at,94439\_at,94815\_at,97833\_at,97834\_g\_at,99045\_at,AFFX-MUR\_b2\_at,104337\_f\_at,98931\_at,101186\_at,100088\_at |
| 5 | monosaccharide metabolism | 11 | 108 | 1042 | 11544 | 0.011 | 0.009 | 1.128 | 0.384 | 160338\_at,101990\_at,160092\_at,161946\_r\_at,94439\_at,94815\_at,97833\_at,97834\_g\_at,99045\_at,AFFX-MUR\_b2\_at,104337\_f\_at |
| 6 | monosaccharide biosynthesis | 1 | 18 | 880 | 9498 | 0.001 | 0.002 | 0.6 | 0.827 | 160338\_at |
| 7 | hexose biosynthesis | 1 | 18 | 538 | 6246 | 0.002 | 0.003 | 0.646 | 0.803 | 160338\_at |
| 8 | myo-inositol biosynthesis | 1 | 2 | 199 | 2164 | 0.005 | 0.001 | 5.467 | 0.176 | 160338\_at |
| 6 | monosaccharide catabolism | 9 | 58 | 880 | 9498 | 0.01 | 0.006 | 1.674 | 0.084 | 101990\_at,160092\_at,161946\_r\_at,94439\_at,94815\_at,97833\_at,97834\_g\_at,99045\_at,AFFX-MUR\_b2\_at |
| 7 | hexose catabolism | 9 | 58 | 538 | 6246 | 0.017 | 0.009 | 1.801 | 0.058 | 101990\_at,160092\_at,161946\_r\_at,94439\_at,94815\_at,97833\_at,97834\_g\_at,99045\_at,AFFX-MUR\_b2\_at |
| 8 | glucose catabolism | 9 | 58 | 199 | 2164 | 0.045 | 0.027 | 1.688 | 0.079 | 101990\_at,160092\_at,161946\_r\_at,94439\_at,94815\_at,97833\_at,97834\_g\_at,99045\_at,AFFX-MUR\_b2\_at |
| 9 | glycolysis | 9 | 52 | 72 | 911 | 0.125 | 0.057 | 2.19 | 0.017 | 101990\_at,160092\_at,161946\_r\_at,94439\_at,94815\_at,97833\_at,97834\_g\_at,99045\_at,AFFX-MUR\_b2\_at |
| 6 | pentose metabolism | 1 | 1 | 880 | 9498 | 0.001 | 0 | 10.364 | 0.093 | 104337\_f\_at |
| 7 | D-ribose metabolism | 1 | 1 | 538 | 6246 | 0.002 | 0 | 11.625 | 0.086 | 104337\_f\_at |
| 5 | aminoglycan metabolism | 1 | 7 | 1042 | 11544 | 0.001 | 0.001 | 1.574 | 0.484 | 98931\_at |
| 6 | aminoglycan catabolism | 1 | 3 | 880 | 9498 | 0.001 | 0 | 3.562 | 0.253 | 98931\_at |
| 7 | glycosaminoglycan catabolism | 1 | 3 | 538 | 6246 | 0.002 | 0 | 3.875 | 0.237 | 98931\_at |
| 6 | glycosaminoglycan metabolism | 1 | 7 | 880 | 9498 | 0.001 | 0.001 | 1.541 | 0.494 | 98931\_at |
| 7 | glycosaminoglycan catabolism | 1 | 3 | 538 | 6246 | 0.002 | 0 | 3.875 | 0.237 | 98931\_at |
| 5 | carbohydrate biosynthesis | 3 | 48 | 1042 | 11544 | 0.003 | 0.004 | 0.692 | 0.821 | 103739\_at,160338\_at,101186\_at |
| 6 | monosaccharide biosynthesis | 1 | 18 | 880 | 9498 | 0.001 | 0.002 | 0.6 | 0.827 | 160338\_at |
| 7 | hexose biosynthesis | 1 | 18 | 538 | 6246 | 0.002 | 0.003 | 0.646 | 0.803 | 160338\_at |
| 8 | myo-inositol biosynthesis | 1 | 2 | 199 | 2164 | 0.005 | 0.001 | 5.467 | 0.176 | 160338\_at |
| 6 | proteoglycan biosynthesis | 1 | 4 | 880 | 9498 | 0.001 | 0 | 2.714 | 0.322 | 101186\_at |
| 5 | polysaccharide metabolism | 1 | 20 | 1042 | 11544 | 0.001 | 0.002 | 0.555 | 0.849 | 100088\_at |
| 6 | glucan metabolism | 1 | 14 | 880 | 9498 | 0.001 | 0.001 | 0.776 | 0.744 | 100088\_at |
| 7 | glycogen metabolism | 1 | 14 | 538 | 6246 | 0.002 | 0.002 | 0.83 | 0.717 | 100088\_at |
| 4 | catabolism | 52 | 631 | 1185 | 13100 | 0.044 | 0.048 | 0.911 | 0.784 | 101990\_at,160092\_at,161946\_r\_at,94439\_at,94815\_at,97833\_at,97834\_g\_at,99045\_at,AFFX-MUR\_b2\_at,102302\_at,99184\_at,96657\_at,93165\_at,100622\_at,95062\_at,100905\_at,101963\_at,102064\_at,102823\_at,102824\_g\_at,102905\_at,103222\_at,103223\_at,103959\_at,103990\_at,104186\_at,104188\_at,104677\_at,160655\_at,160676\_at,160718\_at,161270\_i\_at,92256\_at,94695\_at,94834\_at,94861\_at,96738\_at,97111\_at,97336\_at,97665\_i\_at,98436\_s\_at,99970\_at,103713\_at,160205\_f\_at,92660\_f\_at,92821\_at,93464\_at,94917\_at,95563\_at,95564\_at,96176\_at,99102\_at |
| 5 | alcohol catabolism | 9 | 58 | 1042 | 11544 | 0.009 | 0.005 | 1.721 | 0.074 | 101990\_at,160092\_at,161946\_r\_at,94439\_at,94815\_at,97833\_at,97834\_g\_at,99045\_at,AFFX-MUR\_b2\_at |
| 6 | monosaccharide catabolism | 9 | 58 | 880 | 9498 | 0.01 | 0.006 | 1.674 | 0.084 | 101990\_at,160092\_at,161946\_r\_at,94439\_at,94815\_at,97833\_at,97834\_g\_at,99045\_at,AFFX-MUR\_b2\_at |
| 7 | hexose catabolism | 9 | 58 | 538 | 6246 | 0.017 | 0.009 | 1.801 | 0.058 | 101990\_at,160092\_at,161946\_r\_at,94439\_at,94815\_at,97833\_at,97834\_g\_at,99045\_at,AFFX-MUR\_b2\_at |
| 8 | glucose catabolism | 9 | 58 | 199 | 2164 | 0.045 | 0.027 | 1.688 | 0.079 | 101990\_at,160092\_at,161946\_r\_at,94439\_at,94815\_at,97833\_at,97834\_g\_at,99045\_at,AFFX-MUR\_b2\_at |
| 9 | glycolysis | 9 | 52 | 72 | 911 | 0.125 | 0.057 | 2.19 | 0.017 | 101990\_at,160092\_at,161946\_r\_at,94439\_at,94815\_at,97833\_at,97834\_g\_at,99045\_at,AFFX-MUR\_b2\_at |
| 5 | amine catabolism | 3 | 30 | 1042 | 11544 | 0.003 | 0.003 | 1.108 | 0.517 | 102302\_at,99184\_at,96657\_at |
| 6 | amino acid catabolism | 2 | 24 | 880 | 9498 | 0.002 | 0.003 | 0.897 | 0.666 | 102302\_at,99184\_at |
| 7 | serine family amino acid catabolism | 1 | 4 | 538 | 6246 | 0.002 | 0.001 | 2.906 | 0.303 | 99184\_at |
| 8 | cysteine catabolism | 1 | 2 | 199 | 2164 | 0.005 | 0.001 | 5.467 | 0.176 | 99184\_at |
| 9 | L-cysteine catabolism | 1 | 2 | 72 | 911 | 0.014 | 0.002 | 6.314 | 0.152 | 99184\_at |
| 10 | L-cysteine catabolism to taurine | 1 | 2 | 15 | 197 | 0.067 | 0.01 | 6.568 | 0.147 | 99184\_at |
| 6 | biogenic amine catabolism | 1 | 8 | 880 | 9498 | 0.001 | 0.001 | 1.357 | 0.541 | 96657\_at |
| 7 | polyamine catabolism | 1 | 1 | 538 | 6246 | 0.002 | 0 | 11.625 | 0.086 | 96657\_at |
| 8 | spermine catabolism | 1 | 1 | 199 | 2164 | 0.005 | 0 | 10.935 | 0.092 | 96657\_at |
| 5 | cell wall catabolism | 1 | 5 | 1042 | 11544 | 0.001 | 0 | 2.233 | 0.377 | 93165\_at |
| 5 | lipid catabolism | 1 | 44 | 1042 | 11544 | 0.001 | 0.004 | 0.252 | 0.985 | 100622\_at |
| 5 | macromolecule catabolism | 38 | 470 | 1042 | 11544 | 0.036 | 0.041 | 0.896 | 0.789 | 95062\_at,100905\_at,101963\_at,102064\_at,102823\_at,102824\_g\_at,102905\_at,103222\_at,103223\_at,103959\_at,103990\_at,104186\_at,104188\_at,104677\_at,160655\_at,160676\_at,160718\_at,161270\_i\_at,92256\_at,94695\_at,94834\_at,94861\_at,96738\_at,97111\_at,97336\_at,97665\_i\_at,98436\_s\_at,99970\_at,103713\_at,160205\_f\_at,92660\_f\_at,92821\_at,93464\_at,94917\_at,95563\_at,95564\_at,96176\_at,99102\_at |
| 6 | protein catabolism | 38 | 466 | 880 | 9498 | 0.043 | 0.049 | 0.88 | 0.823 | 95062\_at,100905\_at,101963\_at,102064\_at,102823\_at,102824\_g\_at,102905\_at,103222\_at,103223\_at,103959\_at,103990\_at,104186\_at,104188\_at,104677\_at,160655\_at,160676\_at,160718\_at,161270\_i\_at,92256\_at,94695\_at,94834\_at,94861\_at,96738\_at,97111\_at,97336\_at,97665\_i\_at,98436\_s\_at,99970\_at,103713\_at,160205\_f\_at,92660\_f\_at,92821\_at,93464\_at,94917\_at,95563\_at,95564\_at,96176\_at,99102\_at |
| 7 | proteolysis and peptidolysis | 37 | 457 | 538 | 6246 | 0.069 | 0.073 | 0.94 | 0.684 | 100905\_at,101963\_at,102064\_at,102823\_at,102824\_g\_at,102905\_at,103222\_at,103223\_at,103959\_at,103990\_at,104186\_at,104188\_at,104677\_at,160655\_at,160676\_at,160718\_at,161270\_i\_at,92256\_at,94695\_at,94834\_at,94861\_at,96738\_at,97111\_at,97336\_at,97665\_i\_at,98436\_s\_at,99970\_at,103713\_at,160205\_f\_at,92660\_f\_at,92821\_at,93464\_at,94917\_at,95563\_at,95564\_at,96176\_at,99102\_at |
| 8 | modification-dependent protein catabolism | 10 | 122 | 199 | 2164 | 0.05 | 0.056 | 0.891 | 0.701 | 103713\_at,160205\_f\_at,92660\_f\_at,92821\_at,93464\_at,94917\_at,95563\_at,95564\_at,96176\_at,99102\_at |
| 9 | ubiquitin-dependent protein catabolism | 10 | 120 | 72 | 911 | 0.139 | 0.132 | 1.054 | 0.483 | 103713\_at,160205\_f\_at,92660\_f\_at,92821\_at,93464\_at,94917\_at,95563\_at,95564\_at,96176\_at,99102\_at |
| 4 | coenzymes and prosthetic group metabolism | 5 | 95 | 1185 | 13100 | 0.004 | 0.007 | 0.582 | 0.939 | 160124\_r\_at,94043\_at,96951\_at,160335\_at,93026\_at |
| 5 | coenzymes and prosthetic group biosynthesis | 4 | 51 | 1042 | 11544 | 0.004 | 0.004 | 0.869 | 0.688 | 160124\_r\_at,94043\_at,96951\_at,160335\_at |
| 6 | coenzyme biosynthesis | 4 | 40 | 880 | 9498 | 0.005 | 0.004 | 1.081 | 0.515 | 160124\_r\_at,94043\_at,96951\_at,160335\_at |
| 7 | ATP biosynthesis | 3 | 24 | 538 | 6246 | 0.006 | 0.004 | 1.453 | 0.342 | 160124\_r\_at,94043\_at,96951\_at |
| 7 | glutathione biosynthesis | 1 | 5 | 538 | 6246 | 0.002 | 0.001 | 2.325 | 0.363 | 160335\_at |
| 5 | coenzyme metabolism | 5 | 80 | 1042 | 11544 | 0.005 | 0.007 | 0.693 | 0.86 | 160124\_r\_at,94043\_at,96951\_at,160335\_at,93026\_at |
| 6 | coenzyme biosynthesis | 4 | 40 | 880 | 9498 | 0.005 | 0.004 | 1.081 | 0.515 | 160124\_r\_at,94043\_at,96951\_at,160335\_at |
| 7 | ATP biosynthesis | 3 | 24 | 538 | 6246 | 0.006 | 0.004 | 1.453 | 0.342 | 160124\_r\_at,94043\_at,96951\_at |
| 7 | glutathione biosynthesis | 1 | 5 | 538 | 6246 | 0.002 | 0.001 | 2.325 | 0.363 | 160335\_at |
| 6 | glutathione metabolism | 2 | 9 | 880 | 9498 | 0.002 | 0.001 | 2.389 | 0.2 | 93026\_at,160335\_at |
| 7 | glutathione biosynthesis | 1 | 5 | 538 | 6246 | 0.002 | 0.001 | 2.325 | 0.363 | 160335\_at |
| 4 | electron transport | 29 | 313 | 1185 | 13100 | 0.024 | 0.024 | 1.024 | 0.475 | 101515\_at,101585\_at,102125\_f\_at,102322\_at,103353\_f\_at,103427\_at,103922\_f\_at,160088\_at,160383\_at,160391\_at,160588\_at,160611\_at,162044\_f\_at,92392\_at,92653\_at,93421\_at,93424\_at,93440\_at,93997\_at,94948\_at,95425\_at,96603\_at,96886\_at,97496\_f\_at,97498\_at,98533\_at,98984\_f\_at,99849\_at,99985\_at |
| 5 | thioredoxin pathway | 1 | 3 | 1042 | 11544 | 0.001 | 0 | 3.692 | 0.247 | 99985\_at |
| 4 | lipid metabolism | 17 | 285 | 1185 | 13100 | 0.014 | 0.022 | 0.659 | 0.979 | 161760\_s\_at,96186\_at,103471\_at,99849\_at,98508\_s\_at,160338\_at,97829\_at,100622\_at,101515\_at,161990\_f\_at,93320\_at,95425\_at,98533\_at,96609\_at,93316\_at,96534\_at,102768\_i\_at |
| 5 | lipid biosynthesis | 5 | 124 | 1042 | 11544 | 0.005 | 0.011 | 0.447 | 0.99 | 103471\_at,99849\_at,98508\_s\_at,160338\_at,97829\_at |
| 6 | fatty acid biosynthesis | 2 | 40 | 880 | 9498 | 0.002 | 0.004 | 0.539 | 0.896 | 103471\_at,99849\_at |
| 7 | eicosanoid biosynthesis | 1 | 19 | 538 | 6246 | 0.002 | 0.003 | 0.612 | 0.82 | 99849\_at |
| 8 | leukotriene biosynthesis | 1 | 10 | 199 | 2164 | 0.005 | 0.005 | 1.089 | 0.62 | 99849\_at |
| 6 | glycerolipid biosynthesis | 1 | 3 | 880 | 9498 | 0.001 | 0 | 3.562 | 0.253 | 98508\_s\_at |
| 7 | acylglycerol biosynthesis | 1 | 2 | 538 | 6246 | 0.002 | 0 | 5.812 | 0.165 | 98508\_s\_at |
| 8 | diacylglycerol biosynthesis | 1 | 2 | 199 | 2164 | 0.005 | 0.001 | 5.467 | 0.176 | 98508\_s\_at |
| 6 | membrane lipid biosynthesis | 2 | 20 | 880 | 9498 | 0.002 | 0.002 | 1.076 | 0.565 | 160338\_at,97829\_at |
| 7 | phospholipid biosynthesis | 2 | 14 | 538 | 6246 | 0.004 | 0.002 | 1.661 | 0.343 | 160338\_at,97829\_at |
| 5 | lipid catabolism | 1 | 44 | 1042 | 11544 | 0.001 | 0.004 | 0.252 | 0.985 | 100622\_at |
| 5 | fatty acid metabolism | 8 | 85 | 1042 | 11544 | 0.008 | 0.007 | 1.043 | 0.505 | 101515\_at,161990\_f\_at,93320\_at,95425\_at,98533\_at,103471\_at,99849\_at,96609\_at |
| 6 | fatty acid biosynthesis | 2 | 40 | 880 | 9498 | 0.002 | 0.004 | 0.539 | 0.896 | 103471\_at,99849\_at |
| 7 | eicosanoid biosynthesis | 1 | 19 | 538 | 6246 | 0.002 | 0.003 | 0.612 | 0.82 | 99849\_at |
| 8 | leukotriene biosynthesis | 1 | 10 | 199 | 2164 | 0.005 | 0.005 | 1.089 | 0.62 | 99849\_at |
| 6 | fatty acid oxidation | 2 | 5 | 880 | 9498 | 0.002 | 0.001 | 4.283 | 0.071 | 96609\_at,101515\_at |
| 7 | fatty acid alpha-oxidation | 1 | 1 | 538 | 6246 | 0.002 | 0 | 11.625 | 0.086 | 96609\_at |
| 7 | fatty acid beta-oxidation | 1 | 4 | 538 | 6246 | 0.002 | 0.001 | 2.906 | 0.303 | 101515\_at |
| 5 | membrane lipid metabolism | 3 | 46 | 1042 | 11544 | 0.003 | 0.004 | 0.724 | 0.798 | 160338\_at,97829\_at,98508\_s\_at |
| 6 | membrane lipid biosynthesis | 2 | 20 | 880 | 9498 | 0.002 | 0.002 | 1.076 | 0.565 | 160338\_at,97829\_at |
| 7 | phospholipid biosynthesis | 2 | 14 | 538 | 6246 | 0.004 | 0.002 | 1.661 | 0.343 | 160338\_at,97829\_at |
| 6 | phospholipid metabolism | 2 | 31 | 880 | 9498 | 0.002 | 0.003 | 0.696 | 0.796 | 97829\_at,160338\_at |
| 7 | phospholipid biosynthesis | 2 | 14 | 538 | 6246 | 0.004 | 0.002 | 1.661 | 0.343 | 160338\_at,97829\_at |
| 6 | sphingolipid metabolism | 1 | 14 | 880 | 9498 | 0.001 | 0.001 | 0.776 | 0.744 | 98508\_s\_at |
| 7 | sphingoid metabolism | 1 | 10 | 538 | 6246 | 0.002 | 0.002 | 1.163 | 0.594 | 98508\_s\_at |
| 8 | ceramide metabolism | 1 | 9 | 199 | 2164 | 0.005 | 0.004 | 1.209 | 0.581 | 98508\_s\_at |
| 5 | sphingosine metabolism | 1 | 3 | 1042 | 11544 | 0.001 | 0 | 3.692 | 0.247 | 98508\_s\_at |
| 5 | steroid metabolism | 3 | 74 | 1042 | 11544 | 0.003 | 0.006 | 0.449 | 0.969 | 93316\_at,96534\_at,102768\_i\_at |
| 6 | cholesterol metabolism | 1 | 33 | 880 | 9498 | 0.001 | 0.003 | 0.329 | 0.96 | 96534\_at |
| 6 | sterol biosynthesis | 1 | 20 | 880 | 9498 | 0.001 | 0.002 | 0.54 | 0.857 | 102768\_i\_at |
| 5 | sterol metabolism | 2 | 36 | 1042 | 11544 | 0.002 | 0.003 | 0.615 | 0.849 | 96534\_at,102768\_i\_at |
| 6 | cholesterol metabolism | 1 | 33 | 880 | 9498 | 0.001 | 0.003 | 0.329 | 0.96 | 96534\_at |
| 6 | sterol biosynthesis | 1 | 20 | 880 | 9498 | 0.001 | 0.002 | 0.54 | 0.857 | 102768\_i\_at |
| 4 | nitrogen metabolism | 2 | 20 | 1185 | 13100 | 0.002 | 0.002 | 1.105 | 0.551 | 104165\_at,94415\_at |
| 5 | urea cycle | 1 | 7 | 1042 | 11544 | 0.001 | 0.001 | 1.574 | 0.484 | 94415\_at |
| 4 | nucleobase, nucleoside, nucleotide and nucleic acid metabolism | 161 | 1530 | 1185 | 13100 | 0.136 | 0.117 | 1.163 | 0.019 | 100606\_at,100307\_at,101930\_at,103500\_at,160603\_at,95613\_at,97897\_at,162138\_s\_at,160399\_r\_at,93020\_at,101475\_at,104376\_at,100905\_at,94343\_at,94345\_at,94346\_at,100030\_at,104404\_at,160199\_at,160200\_at,94041\_at,97665\_i\_at,101023\_f\_at,101079\_at,102094\_f\_at,93267\_at,96777\_at,104456\_at,160374\_r\_at,92568\_at,101943\_at,160430\_at,160739\_at,92248\_at,92249\_g\_at,92484\_at,104701\_at,102425\_at,103288\_at,97484\_at,160440\_at,100011\_at,100032\_at,100130\_at,100533\_s\_at,100924\_at,101186\_at,101465\_at,101502\_at,102069\_at,102209\_at,102364\_at,102371\_at,102381\_at,102384\_at,102644\_at,102657\_at,102789\_at,102920\_at,102955\_at,102983\_at,102984\_g\_at,102994\_at,102996\_at,103015\_at,103086\_at,103321\_at,103328\_at,103440\_at,103497\_at,103501\_at,103547\_at,103634\_at,103720\_at,103774\_at,103901\_at,103990\_at,104070\_at,104155\_f\_at,104156\_r\_at,104477\_at,104562\_at,104605\_at,104645\_at,160138\_at,160246\_at,160313\_at,160397\_at,160483\_at,160495\_at,160502\_at,160526\_s\_at,160605\_s\_at,160724\_at,160783\_at,160834\_at,160894\_at,160901\_at,161084\_at,161113\_at,161847\_r\_at,162010\_r\_at,92195\_at,92216\_at,92440\_at,92562\_at,92564\_at,92653\_at,92854\_at,92908\_at,92925\_at,92926\_at,92992\_i\_at,92993\_r\_at,93528\_s\_at,93619\_at,93728\_at,93793\_at,94031\_at,94319\_at,94331\_at,94356\_at,94408\_at,94469\_at,94505\_at,94689\_at,94821\_at,95521\_s\_at,95522\_i\_at,95617\_at,95618\_at,95671\_at,95673\_s\_at,96147\_at,96192\_at,96238\_at,96561\_at,96725\_at,96836\_r\_at,96961\_at,97118\_at,97355\_at,97859\_at,97973\_at,98083\_at,98324\_at,98427\_s\_at,98468\_r\_at,98756\_at,98988\_at,99024\_at,99076\_at,99100\_at,99103\_at,99602\_at,99603\_g\_at,99622\_at,101015\_s\_at,92344\_at,96703\_at,98122\_at |
| 5 | DNA metabolism | 15 | 302 | 1042 | 11544 | 0.014 | 0.026 | 0.55 | 0.997 | 100307\_at,101930\_at,103500\_at,160603\_at,95613\_at,97897\_at,162138\_s\_at,160399\_r\_at,93020\_at,101475\_at,104376\_at,100905\_at,94343\_at,94345\_at,94346\_at |
| 6 | DNA packaging | 6 | 93 | 880 | 9498 | 0.007 | 0.01 | 0.697 | 0.873 | 97897\_at,162138\_s\_at,160399\_r\_at,93020\_at,101475\_at,104376\_at |
| 7 | establishment and/or maintenance of chromatin architecture | 5 | 80 | 538 | 6246 | 0.009 | 0.013 | 0.725 | 0.831 | 162138\_s\_at,160399\_r\_at,93020\_at,101475\_at,104376\_at |
| 8 | chromatin assembly/disassembly | 3 | 48 | 199 | 2164 | 0.015 | 0.022 | 0.68 | 0.833 | 162138\_s\_at,160399\_r\_at,93020\_at |
| 9 | nucleosome assembly | 2 | 28 | 72 | 911 | 0.028 | 0.031 | 0.904 | 0.665 | 160399\_r\_at,93020\_at |
| 8 | chromatin modification | 3 | 36 | 199 | 2164 | 0.015 | 0.017 | 0.906 | 0.657 | 101475\_at,104376\_at,162138\_s\_at |
| 6 | DNA repair | 5 | 99 | 880 | 9498 | 0.006 | 0.01 | 0.545 | 0.959 | 100905\_at,94343\_at,94345\_at,94346\_at,95613\_at |
| 7 | base-excision repair | 1 | 8 | 538 | 6246 | 0.002 | 0.001 | 1.453 | 0.514 | 100905\_at |
| 5 | nucleoside metabolism | 1 | 15 | 1042 | 11544 | 0.001 | 0.001 | 0.738 | 0.758 | 100030\_at |
| 5 | RNA metabolism | 13 | 132 | 1042 | 11544 | 0.012 | 0.011 | 1.092 | 0.414 | 104404\_at,160199\_at,160200\_at,94041\_at,97665\_i\_at,101023\_f\_at,101079\_at,102094\_f\_at,93267\_at,96777\_at,104456\_at,160374\_r\_at,92568\_at |
| 6 | mRNA metabolism | 1 | 10 | 880 | 9498 | 0.001 | 0.001 | 1.086 | 0.622 | 104404\_at |
| 7 | mRNA cleavage | 1 | 3 | 538 | 6246 | 0.002 | 0 | 3.875 | 0.237 | 104404\_at |
| 7 | mRNA polyadenylation | 1 | 7 | 538 | 6246 | 0.002 | 0.001 | 1.661 | 0.468 | 104404\_at |
| 6 | RNA processing | 13 | 126 | 880 | 9498 | 0.015 | 0.013 | 1.113 | 0.385 | 160199\_at,160200\_at,94041\_at,97665\_i\_at,101023\_f\_at,101079\_at,102094\_f\_at,93267\_at,96777\_at,104404\_at,104456\_at,160374\_r\_at,92568\_at |
| 7 | mRNA processing | 8 | 84 | 538 | 6246 | 0.015 | 0.013 | 1.106 | 0.438 | 101023\_f\_at,101079\_at,102094\_f\_at,93267\_at,96777\_at,104404\_at,104456\_at,160374\_r\_at |
| 8 | mRNA modification | 1 | 7 | 199 | 2164 | 0.005 | 0.003 | 1.557 | 0.491 | 104456\_at |
| 8 | nuclear mRNA splicing, via spliceosome | 2 | NA | 199 | 2164 | 0.01 | NA | NA | NA | 101023\_f\_at,160374\_r\_at |
| 9 | spliceosome assembly | 2 | 19 | 72 | 911 | 0.028 | 0.021 | 1.332 | 0.451 | 101023\_f\_at,160374\_r\_at |
| 10 | mRNA splice site selection | 2 | 16 | 15 | 197 | 0.133 | 0.081 | 1.642 | 0.349 | 101023\_f\_at,160374\_r\_at |
| 7 | mRNA cleavage | 1 | 3 | 538 | 6246 | 0.002 | 0 | 3.875 | 0.237 | 104404\_at |
| 7 | mRNA polyadenylation | 1 | 7 | 538 | 6246 | 0.002 | 0.001 | 1.661 | 0.468 | 104404\_at |
| 7 | RNA modification | 2 | 10 | 538 | 6246 | 0.004 | 0.002 | 2.325 | 0.211 | 104456\_at,92568\_at |
| 8 | mRNA modification | 1 | 7 | 199 | 2164 | 0.005 | 0.003 | 1.557 | 0.491 | 104456\_at |
| 8 | RNA methylation | 1 | 2 | 199 | 2164 | 0.005 | 0.001 | 5.467 | 0.176 | 104456\_at |
| 8 | rRNA modification | 1 | 1 | 199 | 2164 | 0.005 | 0 | 10.935 | 0.092 | 92568\_at |
| 5 | transcription | 138 | 1086 | 1042 | 11544 | 0.132 | 0.094 | 1.408 | 0 | 101943\_at,160430\_at,160739\_at,92248\_at,92249\_g\_at,92484\_at,93267\_at,104701\_at,102425\_at,103288\_at,104376\_at,97484\_at,160440\_at,100011\_at,100032\_at,100130\_at,100307\_at,100533\_s\_at,100924\_at,101186\_at,101465\_at,101475\_at,101502\_at,101930\_at,102069\_at,102209\_at,102364\_at,102371\_at,102381\_at,102384\_at,102644\_at,102657\_at,102789\_at,102920\_at,102955\_at,102983\_at,102984\_g\_at,102994\_at,102996\_at,103015\_at,103086\_at,103321\_at,103328\_at,103440\_at,103497\_at,103501\_at,103547\_at,103634\_at,103720\_at,103774\_at,103901\_at,103990\_at,104070\_at,104155\_f\_at,104156\_r\_at,104477\_at,104562\_at,104605\_at,104645\_at,160138\_at,160246\_at,160313\_at,160397\_at,160483\_at,160495\_at,160502\_at,160526\_s\_at,160603\_at,160605\_s\_at,160724\_at,160783\_at,160834\_at,160894\_at,160901\_at,161084\_at,161113\_at,161847\_r\_at,162010\_r\_at,162138\_s\_at,92195\_at,92216\_at,92440\_at,92562\_at,92564\_at,92653\_at,92854\_at,92908\_at,92925\_at,92926\_at,92992\_i\_at,92993\_r\_at,93528\_s\_at,93619\_at,93728\_at,93793\_at,94031\_at,94319\_at,94331\_at,94356\_at,94408\_at,94469\_at,94505\_at,94689\_at,94821\_at,95521\_s\_at,95522\_i\_at,95617\_at,95618\_at,95671\_at,95673\_s\_at,96147\_at,96192\_at,96238\_at,96561\_at,96725\_at,96836\_r\_at,96961\_at,97118\_at,97355\_at,97859\_at,97973\_at,98083\_at,98324\_at,98427\_s\_at,98468\_r\_at,98756\_at,98988\_at,99024\_at,99076\_at,99100\_at,99103\_at,99602\_at,99603\_g\_at,99622\_at,101015\_s\_at,92344\_at,96703\_at,98122\_at |
| 6 | regulation of transcription | 135 | 1026 | 880 | 9498 | 0.153 | 0.108 | 1.42 | 0 | 160430\_at,104701\_at,102425\_at,103288\_at,104376\_at,97484\_at,160440\_at,100011\_at,100032\_at,100130\_at,100307\_at,100533\_s\_at,100924\_at,101186\_at,101465\_at,101475\_at,101502\_at,101930\_at,102069\_at,102209\_at,102364\_at,102371\_at,102381\_at,102384\_at,102644\_at,102657\_at,102789\_at,102920\_at,102955\_at,102983\_at,102984\_g\_at,102994\_at,102996\_at,103015\_at,103086\_at,103321\_at,103328\_at,103440\_at,103497\_at,103501\_at,103547\_at,103634\_at,103720\_at,103774\_at,103901\_at,103990\_at,104070\_at,104155\_f\_at,104156\_r\_at,104477\_at,104562\_at,104605\_at,104645\_at,160138\_at,160246\_at,160313\_at,160397\_at,160483\_at,160495\_at,160502\_at,160526\_s\_at,160603\_at,160605\_s\_at,160724\_at,160783\_at,160834\_at,160894\_at,160901\_at,161084\_at,161113\_at,161847\_r\_at,162010\_r\_at,162138\_s\_at,92195\_at,92216\_at,92248\_at,92249\_g\_at,92440\_at,92484\_at,92562\_at,92564\_at,92653\_at,92854\_at,92908\_at,92925\_at,92926\_at,92992\_i\_at,92993\_r\_at,93267\_at,93528\_s\_at,93619\_at,93728\_at,93793\_at,94031\_at,94319\_at,94331\_at,94356\_at,94408\_at,94469\_at,94505\_at,94689\_at,94821\_at,95521\_s\_at,95522\_i\_at,95617\_at,95618\_at,95671\_at,95673\_s\_at,96147\_at,96192\_at,96238\_at,96561\_at,96725\_at,96836\_r\_at,96961\_at,97118\_at,97355\_at,97859\_at,97973\_at,98083\_at,98324\_at,98427\_s\_at,98468\_r\_at,98756\_at,98988\_at,99024\_at,99076\_at,99100\_at,99103\_at,99602\_at,99603\_g\_at,99622\_at,101015\_s\_at,92344\_at,96703\_at |
| 7 | negative regulation of transcription | 5 | 40 | 538 | 6246 | 0.009 | 0.006 | 1.452 | 0.259 | 104701\_at,102425\_at,103288\_at,104376\_at,97484\_at |
| 8 | negative regulation of transcription, DNA-dependent | 5 | 27 | 199 | 2164 | 0.025 | 0.012 | 2.014 | 0.095 | 104701\_at,102425\_at,103288\_at,104376\_at,97484\_at |
| 9 | negative regulation of transcription from Pol II promoter | 4 | 24 | 72 | 911 | 0.056 | 0.026 | 2.109 | 0.114 | 102425\_at,103288\_at,104376\_at,97484\_at |
| 7 | positive regulation of transcription | 1 | 20 | 538 | 6246 | 0.002 | 0.003 | 0.581 | 0.835 | 160440\_at |
| 8 | positive regulation of transcription, DNA-dependent | 1 | 15 | 199 | 2164 | 0.005 | 0.007 | 0.726 | 0.766 | 160440\_at |
| 9 | positive regulation of transcription from Pol II promoter | 1 | 15 | 72 | 911 | 0.014 | 0.016 | 0.843 | 0.712 | 160440\_at |
| 7 | regulation of transcription, DNA-dependent | 135 | 1013 | 538 | 6246 | 0.251 | 0.162 | 1.547 | 0 | 100011\_at,100032\_at,100130\_at,100307\_at,100533\_s\_at,100924\_at,101186\_at,101465\_at,101475\_at,101502\_at,101930\_at,102069\_at,102209\_at,102364\_at,102371\_at,102381\_at,102384\_at,102425\_at,102644\_at,102657\_at,102789\_at,102920\_at,102955\_at,102983\_at,102984\_g\_at,102994\_at,102996\_at,103015\_at,103086\_at,103321\_at,103328\_at,103440\_at,103497\_at,103501\_at,103547\_at,103634\_at,103720\_at,103774\_at,103901\_at,103990\_at,104070\_at,104155\_f\_at,104156\_r\_at,104376\_at,104477\_at,104562\_at,104605\_at,104645\_at,104701\_at,160138\_at,160246\_at,160313\_at,160397\_at,160430\_at,160440\_at,160483\_at,160495\_at,160502\_at,160526\_s\_at,160603\_at,160605\_s\_at,160724\_at,160783\_at,160834\_at,160894\_at,160901\_at,161084\_at,161113\_at,161847\_r\_at,162010\_r\_at,162138\_s\_at,92195\_at,92216\_at,92248\_at,92249\_g\_at,92440\_at,92484\_at,92562\_at,92564\_at,92653\_at,92854\_at,92908\_at,92925\_at,92926\_at,92992\_i\_at,92993\_r\_at,93267\_at,93528\_s\_at,93619\_at,93728\_at,93793\_at,94031\_at,94319\_at,94331\_at,94356\_at,94408\_at,94469\_at,94505\_at,94689\_at,94821\_at,95521\_s\_at,95522\_i\_at,95617\_at,95618\_at,95671\_at,95673\_s\_at,96147\_at,96192\_at,96238\_at,96561\_at,96725\_at,96836\_r\_at,96961\_at,97118\_at,97355\_at,97484\_at,97859\_at,97973\_at,98083\_at,98324\_at,98427\_s\_at,98468\_r\_at,98756\_at,98988\_at,99024\_at,99076\_at,99100\_at,99103\_at,99602\_at,99603\_g\_at,99622\_at,103288\_at,101015\_s\_at,92344\_at,96703\_at |
| 8 | negative regulation of transcription, DNA-dependent | 5 | 27 | 199 | 2164 | 0.025 | 0.012 | 2.014 | 0.095 | 104701\_at,102425\_at,103288\_at,104376\_at,97484\_at |
| 9 | negative regulation of transcription from Pol II promoter | 4 | 24 | 72 | 911 | 0.056 | 0.026 | 2.109 | 0.114 | 102425\_at,103288\_at,104376\_at,97484\_at |
| 8 | positive regulation of transcription, DNA-dependent | 1 | 15 | 199 | 2164 | 0.005 | 0.007 | 0.726 | 0.766 | 160440\_at |
| 9 | positive regulation of transcription from Pol II promoter | 1 | 15 | 72 | 911 | 0.014 | 0.016 | 0.843 | 0.712 | 160440\_at |
| 8 | regulation of transcription from Pol II promoter | 13 | 72 | 199 | 2164 | 0.065 | 0.033 | 1.964 | 0.012 | 101015\_s\_at,102983\_at,102984\_g\_at,160440\_at,92344\_at,92992\_i\_at,92993\_r\_at,96703\_at,99100\_at,102425\_at,103288\_at,104376\_at,97484\_at |
| 9 | negative regulation of transcription from Pol II promoter | 4 | 24 | 72 | 911 | 0.056 | 0.026 | 2.109 | 0.114 | 102425\_at,103288\_at,104376\_at,97484\_at |
| 9 | positive regulation of transcription from Pol II promoter | 1 | 15 | 72 | 911 | 0.014 | 0.016 | 0.843 | 0.712 | 160440\_at |
| 6 | transcription, DNA-dependent | 136 | 1046 | 880 | 9498 | 0.155 | 0.11 | 1.403 | 0 | 100011\_at,100032\_at,100130\_at,100307\_at,100533\_s\_at,100924\_at,101186\_at,101465\_at,101475\_at,101502\_at,101930\_at,102069\_at,102209\_at,102364\_at,102371\_at,102381\_at,102384\_at,102425\_at,102644\_at,102657\_at,102789\_at,102920\_at,102955\_at,102983\_at,102984\_g\_at,102994\_at,102996\_at,103015\_at,103086\_at,103321\_at,103328\_at,103440\_at,103497\_at,103501\_at,103547\_at,103634\_at,103720\_at,103774\_at,103901\_at,103990\_at,104070\_at,104155\_f\_at,104156\_r\_at,104376\_at,104477\_at,104562\_at,104605\_at,104645\_at,104701\_at,160138\_at,160246\_at,160313\_at,160397\_at,160430\_at,160440\_at,160483\_at,160495\_at,160502\_at,160526\_s\_at,160603\_at,160605\_s\_at,160724\_at,160783\_at,160834\_at,160894\_at,160901\_at,161084\_at,161113\_at,161847\_r\_at,162010\_r\_at,162138\_s\_at,92195\_at,92216\_at,92248\_at,92249\_g\_at,92440\_at,92484\_at,92562\_at,92564\_at,92653\_at,92854\_at,92908\_at,92925\_at,92926\_at,92992\_i\_at,92993\_r\_at,93267\_at,93528\_s\_at,93619\_at,93728\_at,93793\_at,94031\_at,94319\_at,94331\_at,94356\_at,94408\_at,94469\_at,94505\_at,94689\_at,94821\_at,95521\_s\_at,95522\_i\_at,95617\_at,95618\_at,95671\_at,95673\_s\_at,96147\_at,96192\_at,96238\_at,96561\_at,96725\_at,96836\_r\_at,96961\_at,97118\_at,97355\_at,97484\_at,97859\_at,97973\_at,98083\_at,98324\_at,98427\_s\_at,98468\_r\_at,98756\_at,98988\_at,99024\_at,99076\_at,99100\_at,99103\_at,99602\_at,99603\_g\_at,99622\_at,103288\_at,101015\_s\_at,92344\_at,96703\_at,98122\_at |
| 7 | regulation of transcription, DNA-dependent | 135 | 1013 | 538 | 6246 | 0.251 | 0.162 | 1.547 | 0 | 100011\_at,100032\_at,100130\_at,100307\_at,100533\_s\_at,100924\_at,101186\_at,101465\_at,101475\_at,101502\_at,101930\_at,102069\_at,102209\_at,102364\_at,102371\_at,102381\_at,102384\_at,102425\_at,102644\_at,102657\_at,102789\_at,102920\_at,102955\_at,102983\_at,102984\_g\_at,102994\_at,102996\_at,103015\_at,103086\_at,103321\_at,103328\_at,103440\_at,103497\_at,103501\_at,103547\_at,103634\_at,103720\_at,103774\_at,103901\_at,103990\_at,104070\_at,104155\_f\_at,104156\_r\_at,104376\_at,104477\_at,104562\_at,104605\_at,104645\_at,104701\_at,160138\_at,160246\_at,160313\_at,160397\_at,160430\_at,160440\_at,160483\_at,160495\_at,160502\_at,160526\_s\_at,160603\_at,160605\_s\_at,160724\_at,160783\_at,160834\_at,160894\_at,160901\_at,161084\_at,161113\_at,161847\_r\_at,162010\_r\_at,162138\_s\_at,92195\_at,92216\_at,92248\_at,92249\_g\_at,92440\_at,92484\_at,92562\_at,92564\_at,92653\_at,92854\_at,92908\_at,92925\_at,92926\_at,92992\_i\_at,92993\_r\_at,93267\_at,93528\_s\_at,93619\_at,93728\_at,93793\_at,94031\_at,94319\_at,94331\_at,94356\_at,94408\_at,94469\_at,94505\_at,94689\_at,94821\_at,95521\_s\_at,95522\_i\_at,95617\_at,95618\_at,95671\_at,95673\_s\_at,96147\_at,96192\_at,96238\_at,96561\_at,96725\_at,96836\_r\_at,96961\_at,97118\_at,97355\_at,97484\_at,97859\_at,97973\_at,98083\_at,98324\_at,98427\_s\_at,98468\_r\_at,98756\_at,98988\_at,99024\_at,99076\_at,99100\_at,99103\_at,99602\_at,99603\_g\_at,99622\_at,103288\_at,101015\_s\_at,92344\_at,96703\_at |
| 8 | negative regulation of transcription, DNA-dependent | 5 | 27 | 199 | 2164 | 0.025 | 0.012 | 2.014 | 0.095 | 104701\_at,102425\_at,103288\_at,104376\_at,97484\_at |
| 9 | negative regulation of transcription from Pol II promoter | 4 | 24 | 72 | 911 | 0.056 | 0.026 | 2.109 | 0.114 | 102425\_at,103288\_at,104376\_at,97484\_at |
| 8 | positive regulation of transcription, DNA-dependent | 1 | 15 | 199 | 2164 | 0.005 | 0.007 | 0.726 | 0.766 | 160440\_at |
| 9 | positive regulation of transcription from Pol II promoter | 1 | 15 | 72 | 911 | 0.014 | 0.016 | 0.843 | 0.712 | 160440\_at |
| 8 | regulation of transcription from Pol II promoter | 13 | 72 | 199 | 2164 | 0.065 | 0.033 | 1.964 | 0.012 | 101015\_s\_at,102983\_at,102984\_g\_at,160440\_at,92344\_at,92992\_i\_at,92993\_r\_at,96703\_at,99100\_at,102425\_at,103288\_at,104376\_at,97484\_at |
| 9 | negative regulation of transcription from Pol II promoter | 4 | 24 | 72 | 911 | 0.056 | 0.026 | 2.109 | 0.114 | 102425\_at,103288\_at,104376\_at,97484\_at |
| 9 | positive regulation of transcription from Pol II promoter | 1 | 15 | 72 | 911 | 0.014 | 0.016 | 0.843 | 0.712 | 160440\_at |
| 7 | transcription from Pol II promoter | 14 | 104 | 538 | 6246 | 0.026 | 0.017 | 1.563 | 0.061 | 98122\_at,99100\_at,101015\_s\_at,102983\_at,102984\_g\_at,160440\_at,92344\_at,92992\_i\_at,92993\_r\_at,96703\_at,102425\_at,103288\_at,104376\_at,97484\_at |
| 8 | regulation of transcription from Pol II promoter | 13 | 72 | 199 | 2164 | 0.065 | 0.033 | 1.964 | 0.012 | 101015\_s\_at,102983\_at,102984\_g\_at,160440\_at,92344\_at,92992\_i\_at,92993\_r\_at,96703\_at,99100\_at,102425\_at,103288\_at,104376\_at,97484\_at |
| 9 | negative regulation of transcription from Pol II promoter | 4 | 24 | 72 | 911 | 0.056 | 0.026 | 2.109 | 0.114 | 102425\_at,103288\_at,104376\_at,97484\_at |
| 9 | positive regulation of transcription from Pol II promoter | 1 | 15 | 72 | 911 | 0.014 | 0.016 | 0.843 | 0.712 | 160440\_at |
| 5 | two-component signal transduction system (phosphorelay) | 5 | 15 | 1042 | 11544 | 0.005 | 0.001 | 3.692 | 0.008 | 92854\_at,94031\_at,94319\_at,94505\_at,96238\_at |
| 5 | two-component signal transduction system (phosphorelay) | 5 | 15 | 1042 | 11544 | 0.005 | 0.001 | 3.692 | 0.008 | 92854\_at,94031\_at,94319\_at,94505\_at,96238\_at |
| 4 | oxygen and reactive oxygen species metabolism | 1 | 34 | 1185 | 13100 | 0.001 | 0.003 | 0.323 | 0.96 | 93543\_f\_at |
| 5 | glutathione conjugation reaction | 1 | 15 | 1042 | 11544 | 0.001 | 0.001 | 0.738 | 0.758 | 93543\_f\_at |
| 4 | phosphorus metabolism | 49 | 488 | 1185 | 13100 | 0.041 | 0.037 | 1.11 | 0.239 | 100088\_at,100595\_at,101836\_at,104598\_at,92638\_at,92888\_s\_at,93179\_at,93285\_at,94980\_at,98508\_s\_at,98580\_at,99970\_at,100417\_at,101457\_at,101936\_at,102224\_at,102332\_at,103020\_s\_at,103021\_r\_at,103416\_at,103451\_at,103969\_at,104417\_at,161067\_at,161184\_f\_at,93274\_at,93311\_at,93315\_at,93421\_at,93424\_at,94483\_at,95805\_at,96488\_at,96489\_at,96771\_at,96852\_at,97409\_at,97429\_at,97890\_at,97925\_at,98369\_f\_at,98504\_at,99070\_at,102983\_at,102984\_g\_at,160440\_at,92216\_at,102414\_i\_at,102415\_r\_at |
| 5 | phosphate metabolism | 49 | 488 | 1042 | 11544 | 0.047 | 0.042 | 1.112 | 0.233 | 100088\_at,100595\_at,101836\_at,104598\_at,92638\_at,92888\_s\_at,93179\_at,93285\_at,94980\_at,98508\_s\_at,98580\_at,99970\_at,100417\_at,101457\_at,101936\_at,102224\_at,102332\_at,103020\_s\_at,103021\_r\_at,103416\_at,103451\_at,103969\_at,104417\_at,161067\_at,161184\_f\_at,93274\_at,93311\_at,93315\_at,93421\_at,93424\_at,94483\_at,95805\_at,96488\_at,96489\_at,96771\_at,96852\_at,97409\_at,97429\_at,97890\_at,97925\_at,98369\_f\_at,98504\_at,99070\_at,102983\_at,102984\_g\_at,160440\_at,92216\_at,102414\_i\_at,102415\_r\_at |
| 6 | dephosphorylation | 12 | 92 | 880 | 9498 | 0.014 | 0.01 | 1.408 | 0.142 | 100088\_at,100595\_at,101836\_at,104598\_at,92638\_at,92888\_s\_at,93179\_at,93285\_at,94980\_at,98508\_s\_at,98580\_at,99970\_at |
| 7 | protein amino acid dephosphorylation | 12 | 92 | 538 | 6246 | 0.022 | 0.015 | 1.514 | 0.095 | 100088\_at,100595\_at,101836\_at,104598\_at,92638\_at,92888\_s\_at,93179\_at,93285\_at,94980\_at,98508\_s\_at,98580\_at,99970\_at |
| 6 | phosphorylation | 37 | 395 | 880 | 9498 | 0.042 | 0.042 | 1.011 | 0.498 | 100417\_at,101457\_at,101936\_at,102224\_at,102332\_at,103020\_s\_at,103021\_r\_at,103416\_at,103451\_at,103969\_at,104417\_at,161067\_at,161184\_f\_at,93274\_at,93311\_at,93315\_at,93421\_at,93424\_at,94483\_at,95805\_at,96488\_at,96489\_at,96771\_at,96852\_at,97409\_at,97429\_at,97890\_at,97925\_at,98369\_f\_at,98504\_at,99070\_at,102983\_at,102984\_g\_at,160440\_at,92216\_at,102414\_i\_at,102415\_r\_at |
| 7 | protein amino acid phosphorylation | 37 | 379 | 538 | 6246 | 0.069 | 0.061 | 1.133 | 0.23 | 100417\_at,101457\_at,101936\_at,102224\_at,102332\_at,103020\_s\_at,103021\_r\_at,103416\_at,103451\_at,103969\_at,104417\_at,161067\_at,161184\_f\_at,93274\_at,93311\_at,93315\_at,93421\_at,93424\_at,94483\_at,95805\_at,96488\_at,96489\_at,96771\_at,96852\_at,97409\_at,97429\_at,97890\_at,97925\_at,98369\_f\_at,98504\_at,99070\_at,102983\_at,102984\_g\_at,160440\_at,92216\_at,102414\_i\_at,102415\_r\_at |
| 8 | I-kappaB phosphorylation | 1 | 2 | 199 | 2164 | 0.005 | 0.001 | 5.467 | 0.176 | 99070\_at |
| 8 | regulation of protein kinase activity | 2 | 6 | 199 | 2164 | 0.01 | 0.003 | 3.628 | 0.099 | 102414\_i\_at,102415\_r\_at |
| 9 | negative regulation of protein kinase activity | 2 | 6 | 72 | 911 | 0.028 | 0.007 | 4.215 | 0.075 | 102414\_i\_at,102415\_r\_at |
| 4 | protein metabolism | 137 | 1458 | 1185 | 13100 | 0.116 | 0.111 | 1.039 | 0.325 | 101023\_f\_at,103913\_at,104453\_at,92256\_at,95023\_at,95444\_at,96669\_at,96736\_at,160373\_i\_at,101079\_at,97458\_at,104149\_at,101681\_f\_at,102292\_at,103674\_f\_at,103994\_at,104049\_at,104144\_at,160111\_at,160112\_at,160130\_at,160976\_at,160977\_at,161342\_r\_at,161666\_f\_at,92855\_at,93975\_at,94499\_at,95070\_at,95737\_at,96325\_at,100136\_at,101590\_at,95681\_f\_at,96628\_at,96725\_at,93315\_at,160137\_at,94433\_at,160579\_at,94818\_at,93852\_at,97798\_at,94976\_at,95062\_at,100905\_at,101963\_at,102064\_at,102823\_at,102824\_g\_at,102905\_at,103222\_at,103223\_at,103959\_at,103990\_at,104186\_at,104188\_at,104677\_at,160655\_at,160676\_at,160718\_at,161270\_i\_at,94695\_at,94834\_at,94861\_at,96738\_at,97111\_at,97336\_at,97665\_i\_at,98436\_s\_at,99970\_at,103713\_at,160205\_f\_at,92660\_f\_at,92821\_at,93464\_at,94917\_at,95563\_at,95564\_at,96176\_at,99102\_at,102279\_at,160103\_at,160104\_at,93731\_at,103584\_at,94809\_at,98906\_at,100088\_at,100595\_at,101836\_at,104598\_at,92638\_at,92888\_s\_at,93179\_at,93285\_at,94980\_at,98508\_s\_at,98580\_at,100417\_at,101457\_at,101936\_at,102224\_at,102332\_at,103020\_s\_at,103021\_r\_at,103416\_at,103451\_at,103969\_at,104417\_at,161067\_at,161184\_f\_at,93274\_at,93311\_at,93421\_at,93424\_at,94483\_at,95805\_at,96488\_at,96489\_at,96771\_at,96852\_at,97409\_at,97429\_at,97890\_at,97925\_at,98369\_f\_at,98504\_at,99070\_at,102983\_at,102984\_g\_at,160440\_at,92216\_at,102414\_i\_at,102415\_r\_at,104070\_at,97897\_at |
| 5 | protein folding | 4 | 56 | 1042 | 11544 | 0.004 | 0.005 | 0.792 | 0.757 | 102279\_at,160103\_at,160104\_at,93731\_at |
| 5 | protein modification | 62 | 654 | 1042 | 11544 | 0.06 | 0.057 | 1.05 | 0.359 | 103584\_at,92660\_f\_at,94809\_at,98906\_at,160137\_at,94433\_at,160579\_at,94818\_at,93852\_at,97798\_at,100088\_at,100595\_at,101836\_at,104598\_at,92638\_at,92888\_s\_at,93179\_at,93285\_at,94980\_at,98508\_s\_at,98580\_at,99970\_at,100417\_at,101457\_at,101936\_at,102224\_at,102332\_at,103020\_s\_at,103021\_r\_at,103416\_at,103451\_at,103969\_at,104417\_at,161067\_at,161184\_f\_at,93274\_at,93311\_at,93315\_at,93421\_at,93424\_at,94483\_at,95805\_at,96488\_at,96489\_at,96771\_at,96852\_at,97409\_at,97429\_at,97890\_at,97925\_at,98369\_f\_at,98504\_at,99070\_at,102983\_at,102984\_g\_at,160440\_at,92216\_at,102414\_i\_at,102415\_r\_at,104070\_at,97897\_at,100905\_at |
| 6 | protein amino acid acetylation | 2 | 6 | 880 | 9498 | 0.002 | 0.001 | 3.603 | 0.1 | 104070\_at,97897\_at |
| 7 | internal protein amino acid acetylation | 1 | 4 | 538 | 6246 | 0.002 | 0.001 | 2.906 | 0.303 | 97897\_at |
| 6 | protein amino acid ADP-ribosylation | 1 | 12 | 880 | 9498 | 0.001 | 0.001 | 0.905 | 0.689 | 100905\_at |
| 6 | ubiquitin cycle | 4 | 56 | 880 | 9498 | 0.005 | 0.006 | 0.771 | 0.775 | 103584\_at,92660\_f\_at,94809\_at,98906\_at |
| 7 | protein ubiquitination | 1 | 5 | 538 | 6246 | 0.002 | 0.001 | 2.325 | 0.363 | 98906\_at |
| 3 | pathogenesis | 1 | 10 | 1040 | 10726 | 0.001 | 0.001 | 1.032 | 0.64 | 94928\_at |
| 4 | necrosis | 1 | 3 | 1185 | 13100 | 0.001 | 0 | 3.652 | 0.248 | 94928\_at |
| 3 | respiratory gaseous exchange | 1 | 10 | 1040 | 10726 | 0.001 | 0.001 | 1.032 | 0.64 | 93193\_at |
| 3 | response to endogenous stimulus | 8 | 119 | 1040 | 10726 | 0.008 | 0.011 | 0.693 | 0.901 | 102292\_at,102912\_at,103531\_f\_at,100905\_at,94343\_at,94345\_at,94346\_at,95613\_at |
| 4 | response to DNA damage stimulus | 8 | 119 | 1185 | 13100 | 0.007 | 0.009 | 0.743 | 0.854 | 102292\_at,102912\_at,103531\_f\_at,100905\_at,94343\_at,94345\_at,94346\_at,95613\_at |
| 3 | response to external stimulus | 72 | 666 | 1040 | 10726 | 0.069 | 0.062 | 1.115 | 0.174 | 98324\_at,104719\_at,97451\_at,100951\_at,97375\_at,102794\_at,104389\_at,104445\_at,104572\_at,160511\_at,103531\_f\_at,160099\_at,93875\_at,94817\_at,95360\_at,97915\_at,97918\_at,100154\_at,100306\_at,100327\_at,100600\_at,100998\_at,101876\_s\_at,101878\_at,101886\_f\_at,103035\_at,103258\_at,103422\_at,104694\_at,160651\_at,92866\_at,93078\_at,93088\_at,93120\_f\_at,93865\_s\_at,93907\_f\_at,94286\_at,96752\_at,97125\_f\_at,97540\_f\_at,97541\_f\_at,98000\_at,98438\_f\_at,98472\_at,99378\_f\_at,99379\_f\_at,100973\_i\_at,102940\_at,103202\_at,103634\_at,104100\_at,104597\_at,95348\_at,96513\_at,98088\_at,98468\_r\_at,103467\_g\_at,99100\_at,94448\_at,100583\_at,101475\_at,102156\_f\_at,102161\_f\_at,102372\_at,93086\_at,101568\_at,93315\_at,94928\_at,98988\_at,95057\_at,95058\_f\_at,160301\_at |
| 4 | response to extracellular stimulus | 1 | 1 | 1185 | 13100 | 0.001 | 0 | 10.5 | 0.09 | 98324\_at |
| 5 | cellular response to starvation | 1 | 1 | 1042 | 11544 | 0.001 | 0 | 10.667 | 0.09 | 98324\_at |
| 4 | perception of external stimulus | 4 | 84 | 1185 | 13100 | 0.003 | 0.006 | 0.527 | 0.953 | 104719\_at,97451\_at,100951\_at,97375\_at |
| 5 | perception of abiotic stimulus | 2 | 72 | 1042 | 11544 | 0.002 | 0.006 | 0.308 | 0.991 | 104719\_at,97451\_at |
| 6 | perception of sound | 2 | 17 | 880 | 9498 | 0.002 | 0.002 | 1.268 | 0.476 | 104719\_at,97451\_at |
| 7 | hearing | 2 | 17 | 538 | 6246 | 0.004 | 0.003 | 1.368 | 0.437 | 104719\_at,97451\_at |
| 5 | perception of mechanical stimulus | 2 | 3 | 1042 | 11544 | 0.002 | 0 | 7.385 | 0.023 | 100951\_at,97375\_at |
| 6 | mechanosensory perception | 2 | 3 | 880 | 9498 | 0.002 | 0 | 7.094 | 0.024 | 100951\_at,97375\_at |
| 4 | response to abiotic stimulus | 14 | 195 | 1185 | 13100 | 0.012 | 0.015 | 0.793 | 0.852 | 104719\_at,97451\_at,102794\_at,104389\_at,104445\_at,104572\_at,160511\_at,103531\_f\_at,160099\_at,93875\_at,94817\_at,95360\_at,97915\_at,97918\_at |
| 5 | perception of abiotic stimulus | 2 | 72 | 1042 | 11544 | 0.002 | 0.006 | 0.308 | 0.991 | 104719\_at,97451\_at |
| 6 | perception of sound | 2 | 17 | 880 | 9498 | 0.002 | 0.002 | 1.268 | 0.476 | 104719\_at,97451\_at |
| 7 | hearing | 2 | 17 | 538 | 6246 | 0.004 | 0.003 | 1.368 | 0.437 | 104719\_at,97451\_at |
| 5 | response to chemical substance | 5 | 90 | 1042 | 11544 | 0.005 | 0.008 | 0.615 | 0.918 | 102794\_at,104389\_at,104445\_at,104572\_at,160511\_at |
| 6 | chemotaxis | 5 | 60 | 880 | 9498 | 0.006 | 0.006 | 0.899 | 0.664 | 102794\_at,104389\_at,104445\_at,104572\_at,160511\_at |
| 5 | response to radiation | 1 | 46 | 1042 | 11544 | 0.001 | 0.004 | 0.241 | 0.987 | 103531\_f\_at |
| 5 | response to temperature | 6 | 28 | 1042 | 11544 | 0.006 | 0.002 | 2.37 | 0.036 | 160099\_at,93875\_at,94817\_at,95360\_at,97915\_at,97918\_at |
| 6 | response to heat | 6 | 27 | 880 | 9498 | 0.007 | 0.003 | 2.401 | 0.034 | 160099\_at,93875\_at,94817\_at,95360\_at,97915\_at,97918\_at |
| 4 | response to biotic stimulus | 59 | 516 | 1185 | 13100 | 0.05 | 0.039 | 1.264 | 0.035 | 100154\_at,100306\_at,100327\_at,100600\_at,100998\_at,101876\_s\_at,101878\_at,101886\_f\_at,102794\_at,103035\_at,103258\_at,103422\_at,104694\_at,160651\_at,92866\_at,93078\_at,93088\_at,93120\_f\_at,93865\_s\_at,93907\_f\_at,94286\_at,96752\_at,97125\_f\_at,97540\_f\_at,97541\_f\_at,98000\_at,98438\_f\_at,98472\_at,99378\_f\_at,99379\_f\_at,100973\_i\_at,102940\_at,103202\_at,103634\_at,104100\_at,104389\_at,104445\_at,104572\_at,104597\_at,160511\_at,95348\_at,96513\_at,98088\_at,98468\_r\_at,103467\_g\_at,99100\_at,94448\_at,100583\_at,101475\_at,102156\_f\_at,102161\_f\_at,102372\_at,93086\_at,101568\_at,93315\_at,94928\_at,98988\_at,95057\_at,95058\_f\_at |
| 5 | defense response | 57 | 471 | 1042 | 11544 | 0.055 | 0.041 | 1.341 | 0.013 | 100154\_at,100306\_at,100327\_at,100600\_at,100998\_at,101876\_s\_at,101878\_at,101886\_f\_at,102794\_at,103035\_at,103258\_at,103422\_at,104694\_at,160651\_at,92866\_at,93078\_at,93088\_at,93120\_f\_at,93865\_s\_at,93907\_f\_at,94286\_at,96752\_at,97125\_f\_at,97540\_f\_at,97541\_f\_at,98000\_at,98438\_f\_at,98472\_at,99378\_f\_at,99379\_f\_at,100973\_i\_at,102940\_at,103202\_at,103634\_at,104100\_at,104389\_at,104445\_at,104572\_at,104597\_at,160511\_at,95348\_at,96513\_at,98088\_at,98468\_r\_at,103467\_g\_at,99100\_at,94448\_at,100583\_at,101475\_at,102156\_f\_at,102161\_f\_at,102372\_at,93086\_at,101568\_at,93315\_at,94928\_at,98988\_at |
| 6 | immune response | 45 | 362 | 880 | 9498 | 0.051 | 0.038 | 1.342 | 0.025 | 100306\_at,100973\_i\_at,100998\_at,101886\_f\_at,102940\_at,103035\_at,103202\_at,103634\_at,104100\_at,104389\_at,104445\_at,104572\_at,104597\_at,160511\_at,92866\_at,93120\_f\_at,93865\_s\_at,94286\_at,95348\_at,96513\_at,97125\_f\_at,97540\_f\_at,97541\_f\_at,98088\_at,98438\_f\_at,98468\_r\_at,98472\_at,99378\_f\_at,99379\_f\_at,103467\_g\_at,99100\_at,93088\_at,102794\_at,94448\_at,100583\_at,101475\_at,102156\_f\_at,102161\_f\_at,102372\_at,93086\_at,101568\_at,104694\_at,93315\_at,94928\_at,98988\_at |
| 7 | acute-phase response | 2 | 23 | 538 | 6246 | 0.004 | 0.004 | 1.011 | 0.601 | 103467\_g\_at,99100\_at |
| 7 | antigen presentation | 12 | 26 | 538 | 6246 | 0.022 | 0.004 | 5.361 | 0 | 101886\_f\_at,93088\_at,93120\_f\_at,97125\_f\_at,97540\_f\_at,97541\_f\_at,98438\_f\_at,98472\_at,99379\_f\_at,100998\_at,92866\_at,94286\_at |
| 8 | antigen presentation, endogenous antigen | 9 | 15 | 199 | 2164 | 0.045 | 0.007 | 6.527 | 0 | 101886\_f\_at,93088\_at,93120\_f\_at,97125\_f\_at,97540\_f\_at,97541\_f\_at,98438\_f\_at,98472\_at,99379\_f\_at |
| 8 | antigen presentation, exogenous antigen | 3 | 11 | 199 | 2164 | 0.015 | 0.005 | 2.969 | 0.073 | 100998\_at,92866\_at,94286\_at |
| 7 | antigen processing | 12 | 27 | 538 | 6246 | 0.022 | 0.004 | 5.162 | 0 | 101886\_f\_at,93088\_at,93120\_f\_at,97125\_f\_at,97540\_f\_at,97541\_f\_at,98438\_f\_at,98472\_at,99379\_f\_at,100998\_at,92866\_at,94286\_at |
| 8 | antigen processing, endogenous antigen via MHC class I | 9 | 15 | 199 | 2164 | 0.045 | 0.007 | 6.527 | 0 | 101886\_f\_at,93088\_at,93120\_f\_at,97125\_f\_at,97540\_f\_at,97541\_f\_at,98438\_f\_at,98472\_at,99379\_f\_at |
| 8 | antigen processing, exogenous antigen via MHC class II | 3 | 12 | 199 | 2164 | 0.015 | 0.006 | 2.717 | 0.091 | 100998\_at,92866\_at,94286\_at |
| 7 | cell activation | 3 | 19 | 538 | 6246 | 0.006 | 0.003 | 1.836 | 0.221 | 96513\_at,102794\_at,160511\_at |
| 8 | lymphocyte activation | 3 | 19 | 199 | 2164 | 0.015 | 0.009 | 1.718 | 0.251 | 96513\_at,102794\_at,160511\_at |
| 9 | lymphocyte differentiation | 1 | 5 | 72 | 911 | 0.014 | 0.005 | 2.53 | 0.338 | 96513\_at |
| 10 | thymocyte differentiation | 1 | 2 | 15 | 197 | 0.067 | 0.01 | 6.568 | 0.147 | 96513\_at |
| 9 | T-cell activation | 3 | 9 | 72 | 911 | 0.042 | 0.01 | 4.218 | 0.028 | 96513\_at,102794\_at,160511\_at |
| 7 | cellular defense response | 2 | 31 | 538 | 6246 | 0.004 | 0.005 | 0.75 | 0.76 | 93088\_at,94448\_at |
| 8 | cellular defense response (sensu Vertebrata) | 1 | 4 | 199 | 2164 | 0.005 | 0.002 | 2.719 | 0.32 | 94448\_at |
| 7 | humoral immune response | 8 | 103 | 538 | 6246 | 0.015 | 0.016 | 0.902 | 0.673 | 100583\_at,101475\_at,102156\_f\_at,102161\_f\_at,102372\_at,93086\_at,94448\_at,101568\_at |
| 8 | humoral defense mechanism (sensu Vertebrata) | 2 | 39 | 199 | 2164 | 0.01 | 0.018 | 0.558 | 0.887 | 94448\_at,101568\_at |
| 9 | complement activation | 1 | 38 | 72 | 911 | 0.014 | 0.042 | 0.333 | 0.959 | 101568\_at |
| 10 | immediate hypersensitivity response | 1 | 4 | 15 | 197 | 0.067 | 0.02 | 3.284 | 0.273 | 101568\_at |
| 7 | innate immune response | 6 | 70 | 538 | 6246 | 0.011 | 0.011 | 0.995 | 0.567 | 104694\_at,93315\_at,94928\_at,95348\_at,98088\_at,98988\_at |
| 8 | inflammatory response | 6 | 70 | 199 | 2164 | 0.03 | 0.032 | 0.932 | 0.635 | 104694\_at,93315\_at,94928\_at,95348\_at,98088\_at,98988\_at |
| 5 | T-cell proliferation | 2 | 4 | 1042 | 11544 | 0.002 | 0 | 5.486 | 0.043 | 102794\_at,160511\_at |
| 5 | response to pest/pathogen/parasite | 18 | 213 | 1042 | 11544 | 0.017 | 0.018 | 0.936 | 0.652 | 103467\_g\_at,99100\_at,93088\_at,94448\_at,100583\_at,101475\_at,102156\_f\_at,102161\_f\_at,102372\_at,93086\_at,101568\_at,104694\_at,93315\_at,94928\_at,95348\_at,98088\_at,98988\_at,100327\_at |
| 6 | response to viruses | 1 | 1 | 880 | 9498 | 0.001 | 0 | 10.364 | 0.093 | 100327\_at |
| 5 | response to unfolded protein | 2 | 6 | 1042 | 11544 | 0.002 | 0.001 | 3.692 | 0.096 | 95057\_at,95058\_f\_at |
| 4 | response to wounding | 9 | 99 | 1185 | 13100 | 0.008 | 0.008 | 1.004 | 0.545 | 160301\_at,93088\_at,94448\_at,104694\_at,93315\_at,94928\_at,95348\_at,98088\_at,98988\_at |
| 3 | response to stress | 35 | 400 | 1040 | 10726 | 0.034 | 0.037 | 0.902 | 0.767 | 95057\_at,95058\_f\_at,102292\_at,102912\_at,103531\_f\_at,100905\_at,94343\_at,94345\_at,94346\_at,95613\_at,160099\_at,93875\_at,94817\_at,95360\_at,97915\_at,97918\_at,103467\_g\_at,99100\_at,93088\_at,94448\_at,100583\_at,101475\_at,102156\_f\_at,102161\_f\_at,102372\_at,93086\_at,101568\_at,104694\_at,93315\_at,94928\_at,95348\_at,98088\_at,98988\_at,100327\_at,160301\_at |
| 4 | response to DNA damage stimulus | 8 | 119 | 1185 | 13100 | 0.007 | 0.009 | 0.743 | 0.854 | 102292\_at,102912\_at,103531\_f\_at,100905\_at,94343\_at,94345\_at,94346\_at,95613\_at |
| 4 | response to wounding | 9 | 99 | 1185 | 13100 | 0.008 | 0.008 | 1.004 | 0.545 | 160301\_at,93088\_at,94448\_at,104694\_at,93315\_at,94928\_at,95348\_at,98088\_at,98988\_at |
| 3 | secretion | 2 | 16 | 1040 | 10726 | 0.002 | 0.001 | 1.289 | 0.469 | 103913\_at,92648\_at |
| 4 | protein secretion | 2 | 8 | 1185 | 13100 | 0.002 | 0.001 | 2.77 | 0.159 | 103913\_at,92648\_at |

  
