# Supplementary material for: Molecular Signatures of Proliferation and Quiescence in Hematopoietic Stem Cells
Source: PLoS Biol. 2004 Sep 28;2(10):e301. doi: 10.1371/journal.pbio.0020301 (PMC520599; doi:10.1371/journal.pbio.0020301)
Supplement: Table S45 — (14 KB HTML). [file pbio.0020301.st045.html]

   Significant Up in Fetal Liver   

# Significant Up in Fetal Liver

|  |  |  |  |  |  |  |  |  |  |  |  |
| --- | --- | --- | --- | --- | --- | --- | --- | --- | --- | --- | --- |
| GOLevel | GOTerm | ProbeCount | ArrayCount | ListGOLevelCount | ArrayGoLevelCount | ListFq | ArrayFq | FoldChange | H-Pvalue | ProbeIds | GeneNames |
| 4 | epidermal cell differentiation | 1 | 3 | 160 | 13100 | 0.006 | 0 | 27.174 | 0.036 | 104243\_r\_at | RIKEN cDNA 4930578F06 gene |
| 5 | hair cell differentiation | 1 | 3 | 144 | 11544 | 0.007 | 0 | 26.692 | 0.037 | 104243\_r\_at | RIKEN cDNA 4930578F06 gene |
| 4 | cell homeostasis | 3 | 41 | 160 | 13100 | 0.019 | 0.003 | 5.99 | 0.014 | 99964\_at,103958\_g\_at,94976\_at | vitamin D receptor,transferrin receptor,expressed sequence AL022610 |
| 5 | cell ion homeostasis | 3 | 37 | 144 | 11544 | 0.021 | 0.003 | 6.489 | 0.011 | 99964\_at,103958\_g\_at,94976\_at | vitamin D receptor,transferrin receptor,expressed sequence AL022610 |
| 6 | cation homeostasis | 3 | 36 | 108 | 9498 | 0.028 | 0.004 | 7.33 | 0.008 | 99964\_at,103958\_g\_at,94976\_at | vitamin D receptor,transferrin receptor,expressed sequence AL022610 |
| 7 | di-, tri-valent inorganic cation homeostasis | 3 | 29 | 79 | 6246 | 0.038 | 0.005 | 8.183 | 0.006 | 99964\_at,103958\_g\_at,94976\_at | vitamin D receptor,transferrin receptor,expressed sequence AL022610 |
| 8 | iron ion homeostasis | 2 | 15 | 23 | 2164 | 0.087 | 0.007 | 12.548 | 0.01 | 103958\_g\_at,94976\_at | transferrin receptor,expressed sequence AL022610 |
| 5 | cell surface structure organization and biogenesis | 1 | 4 | 144 | 11544 | 0.007 | 0 | 19.829 | 0.049 | 101843\_at | SH2-B PH domain containing signaling mediator 1 |
| 6 | formation of a cell surface projection | 1 | 4 | 108 | 9498 | 0.009 | 0 | 22.048 | 0.045 | 101843\_at | SH2-B PH domain containing signaling mediator 1 |
| 7 | lamellipodium formation | 1 | 4 | 79 | 6246 | 0.013 | 0.001 | 19.781 | 0.05 | 101843\_at | SH2-B PH domain containing signaling mediator 1 |
| 7 | cell cycle arrest | 2 | 20 | 79 | 6246 | 0.025 | 0.003 | 7.912 | 0.026 | 97375\_at,98402\_at | polycystic kidney disease 1 homolog,microtubule-actin crosslinking factor 1 |
| 6 | negative regulation of cell migration | 1 | 3 | 108 | 9498 | 0.009 | 0 | 28.938 | 0.034 | 161184\_f\_at | tyrosine kinase receptor 1 |
| 4 | plasma membrane fusion | 1 | 3 | 160 | 13100 | 0.006 | 0 | 27.174 | 0.036 | 161184\_f\_at | tyrosine kinase receptor 1 |
| 4 | epidermal cell differentiation | 1 | 3 | 160 | 13100 | 0.006 | 0 | 27.174 | 0.036 | 104243\_r\_at | RIKEN cDNA 4930578F06 gene |
| 5 | hair cell differentiation | 1 | 3 | 144 | 11544 | 0.007 | 0 | 26.692 | 0.037 | 104243\_r\_at | RIKEN cDNA 4930578F06 gene |
| 5 | blood vessel development | 3 | 61 | 144 | 11544 | 0.021 | 0.005 | 3.945 | 0.04 | 160727\_at,95016\_at,161184\_f\_at | RIKEN cDNA 2410002F23 gene,neuropilin,tyrosine kinase receptor 1 |
| 5 | respiratory tube development | 2 | 10 | 144 | 11544 | 0.014 | 0.001 | 15.966 | 0.007 | 104243\_r\_at,99440\_at | RIKEN cDNA 4930578F06 gene,nuclear factor I/B |
| 6 | lung development | 2 | 10 | 108 | 9498 | 0.019 | 0.001 | 17.638 | 0.005 | 104243\_r\_at,99440\_at | RIKEN cDNA 4930578F06 gene,nuclear factor I/B |
| 4 | cell homeostasis | 3 | 41 | 160 | 13100 | 0.019 | 0.003 | 5.99 | 0.014 | 99964\_at,103958\_g\_at,94976\_at | vitamin D receptor,transferrin receptor,expressed sequence AL022610 |
| 5 | cell ion homeostasis | 3 | 37 | 144 | 11544 | 0.021 | 0.003 | 6.489 | 0.011 | 99964\_at,103958\_g\_at,94976\_at | vitamin D receptor,transferrin receptor,expressed sequence AL022610 |
| 6 | cation homeostasis | 3 | 36 | 108 | 9498 | 0.028 | 0.004 | 7.33 | 0.008 | 99964\_at,103958\_g\_at,94976\_at | vitamin D receptor,transferrin receptor,expressed sequence AL022610 |
| 7 | di-, tri-valent inorganic cation homeostasis | 3 | 29 | 79 | 6246 | 0.038 | 0.005 | 8.183 | 0.006 | 99964\_at,103958\_g\_at,94976\_at | vitamin D receptor,transferrin receptor,expressed sequence AL022610 |
| 8 | iron ion homeostasis | 2 | 15 | 23 | 2164 | 0.087 | 0.007 | 12.548 | 0.01 | 103958\_g\_at,94976\_at | transferrin receptor,expressed sequence AL022610 |
| 5 | cell surface structure organization and biogenesis | 1 | 4 | 144 | 11544 | 0.007 | 0 | 19.829 | 0.049 | 101843\_at | SH2-B PH domain containing signaling mediator 1 |
| 6 | formation of a cell surface projection | 1 | 4 | 108 | 9498 | 0.009 | 0 | 22.048 | 0.045 | 101843\_at | SH2-B PH domain containing signaling mediator 1 |
| 7 | lamellipodium formation | 1 | 4 | 79 | 6246 | 0.013 | 0.001 | 19.781 | 0.05 | 101843\_at | SH2-B PH domain containing signaling mediator 1 |
| 7 | cell cycle arrest | 2 | 20 | 79 | 6246 | 0.025 | 0.003 | 7.912 | 0.026 | 97375\_at,98402\_at | polycystic kidney disease 1 homolog,microtubule-actin crosslinking factor 1 |
| 8 | glutamine biosynthesis | 1 | 2 | 23 | 2164 | 0.043 | 0.001 | 47.261 | 0.021 | 161392\_f\_at | leucine rich protein, B7 gene |
| 9 | proline biosynthesis | 1 | 2 | 6 | 911 | 0.167 | 0.002 | 75.759 | 0.013 | 161392\_f\_at | leucine rich protein, B7 gene |
| 8 | glutamine biosynthesis | 1 | 2 | 23 | 2164 | 0.043 | 0.001 | 47.261 | 0.021 | 161392\_f\_at | leucine rich protein, B7 gene |
| 9 | proline biosynthesis | 1 | 2 | 6 | 911 | 0.167 | 0.002 | 75.759 | 0.013 | 161392\_f\_at | leucine rich protein, B7 gene |
| 5 | transcription | 22 | 1086 | 144 | 11544 | 0.153 | 0.094 | 1.624 | 0.015 | 92249\_g\_at,99964\_at,161348\_r\_at,104243\_r\_at,104376\_at,102789\_at,103052\_r\_at,103091\_at,103236\_at,103547\_at,103666\_at,104438\_at,104645\_at,160244\_at,161148\_f\_at,93425\_at,95618\_at,96147\_at,97813\_at,97901\_at,99440\_at,99587\_at | nuclear receptor subfamily 4, group A, member 2,vitamin D receptor,PDZ and LIM domain 1 (elfin),RIKEN cDNA 4930578F06 gene,histone deacetylase 5,GATA binding protein 2,nuclear receptor subfamily 2, group F, member 2,avian reticuloendotheliosis viral (v-rel) oncogene related B,ring finger protein 1,expressed sequence AI573938,homeo box B5,zinc finger protein 30,Kruppel-like factor 7 (ubiquitous),feminization 1 homolog a (C. elegans),inhibitor of growth family, member 4,interferon regulatory factor 5,DNA segment, Chr 6, ERATO Doi 32, expressed,v-maf musculoaponeurotic fibrosarcoma oncogene family, protein G (avian),v-rel reticuloendotheliosis viral oncogene homolog A (avian),upstream binding transcription factor, RNA polymerase I,nuclear factor I/B,RAB7, member RAS oncogene family |
| 6 | regulation of transcription | 22 | 1026 | 108 | 9498 | 0.204 | 0.108 | 1.886 | 0.002 | 161348\_r\_at,104243\_r\_at,104376\_at,102789\_at,103052\_r\_at,103091\_at,103236\_at,103547\_at,103666\_at,104438\_at,104645\_at,160244\_at,161148\_f\_at,92249\_g\_at,93425\_at,95618\_at,96147\_at,97813\_at,97901\_at,99440\_at,99587\_at,99964\_at | PDZ and LIM domain 1 (elfin),RIKEN cDNA 4930578F06 gene,histone deacetylase 5,GATA binding protein 2,nuclear receptor subfamily 2, group F, member 2,avian reticuloendotheliosis viral (v-rel) oncogene related B,ring finger protein 1,expressed sequence AI573938,homeo box B5,zinc finger protein 30,Kruppel-like factor 7 (ubiquitous),feminization 1 homolog a (C. elegans),inhibitor of growth family, member 4,nuclear receptor subfamily 4, group A, member 2,interferon regulatory factor 5,DNA segment, Chr 6, ERATO Doi 32, expressed,v-maf musculoaponeurotic fibrosarcoma oncogene family, protein G (avian),v-rel reticuloendotheliosis viral oncogene homolog A (avian),upstream binding transcription factor, RNA polymerase I,nuclear factor I/B,RAB7, member RAS oncogene family,vitamin D receptor |
| 8 | negative regulation of transcription, DNA-dependent | 2 | 27 | 23 | 2164 | 0.087 | 0.012 | 6.968 | 0.032 | 104243\_r\_at,104376\_at | RIKEN cDNA 4930578F06 gene,histone deacetylase 5 |
| 9 | negative regulation of transcription from Pol II promoter | 2 | 24 | 6 | 911 | 0.333 | 0.026 | 12.655 | 0.009 | 104243\_r\_at,104376\_at | RIKEN cDNA 4930578F06 gene,histone deacetylase 5 |
| 7 | regulation of transcription, DNA-dependent | 21 | 1013 | 79 | 6246 | 0.266 | 0.162 | 1.639 | 0.012 | 102789\_at,103052\_r\_at,103091\_at,103236\_at,103547\_at,103666\_at,104243\_r\_at,104376\_at,104438\_at,104645\_at,160244\_at,161148\_f\_at,92249\_g\_at,93425\_at,95618\_at,96147\_at,97813\_at,97901\_at,99440\_at,99587\_at,99964\_at | GATA binding protein 2,nuclear receptor subfamily 2, group F, member 2,avian reticuloendotheliosis viral (v-rel) oncogene related B,ring finger protein 1,expressed sequence AI573938,homeo box B5,RIKEN cDNA 4930578F06 gene,histone deacetylase 5,zinc finger protein 30,Kruppel-like factor 7 (ubiquitous),feminization 1 homolog a (C. elegans),inhibitor of growth family, member 4,nuclear receptor subfamily 4, group A, member 2,interferon regulatory factor 5,DNA segment, Chr 6, ERATO Doi 32, expressed,v-maf musculoaponeurotic fibrosarcoma oncogene family, protein G (avian),v-rel reticuloendotheliosis viral oncogene homolog A (avian),upstream binding transcription factor, RNA polymerase I,nuclear factor I/B,RAB7, member RAS oncogene family,vitamin D receptor |
| 8 | negative regulation of transcription, DNA-dependent | 2 | 27 | 23 | 2164 | 0.087 | 0.012 | 6.968 | 0.032 | 104243\_r\_at,104376\_at | RIKEN cDNA 4930578F06 gene,histone deacetylase 5 |
| 9 | negative regulation of transcription from Pol II promoter | 2 | 24 | 6 | 911 | 0.333 | 0.026 | 12.655 | 0.009 | 104243\_r\_at,104376\_at | RIKEN cDNA 4930578F06 gene,histone deacetylase 5 |
| 5 | perception of mechanical stimulus | 1 | 3 | 144 | 11544 | 0.007 | 0 | 26.692 | 0.037 | 97375\_at | polycystic kidney disease 1 homolog |
| 6 | mechanosensory perception | 1 | 3 | 108 | 9498 | 0.009 | 0 | 28.938 | 0.034 | 97375\_at | polycystic kidney disease 1 homolog |
| 7 | antigen presentation | 2 | 26 | 79 | 6246 | 0.025 | 0.004 | 6.087 | 0.042 | 97125\_f\_at,98438\_f\_at | MHC (A.CA/J(H-2K-f) class I antigen,histocompatibility 2, Q region locus 7 |
| 8 | antigen presentation, endogenous antigen | 2 | 15 | 23 | 2164 | 0.087 | 0.007 | 12.548 | 0.01 | 97125\_f\_at,98438\_f\_at | MHC (A.CA/J(H-2K-f) class I antigen,histocompatibility 2, Q region locus 7 |
| 7 | antigen processing | 2 | 27 | 79 | 6246 | 0.025 | 0.004 | 5.861 | 0.045 | 97125\_f\_at,98438\_f\_at | MHC (A.CA/J(H-2K-f) class I antigen,histocompatibility 2, Q region locus 7 |
| 8 | antigen processing, endogenous antigen via MHC class I | 2 | 15 | 23 | 2164 | 0.087 | 0.007 | 12.548 | 0.01 | 97125\_f\_at,98438\_f\_at | MHC (A.CA/J(H-2K-f) class I antigen,histocompatibility 2, Q region locus 7 |

  
