# Supplementary material for: Molecular Signatures of Proliferation and Quiescence in Hematopoietic Stem Cells
Source: PLoS Biol. 2004 Sep 28;2(10):e301. doi: 10.1371/journal.pbio.0020301 (PMC520599; doi:10.1371/journal.pbio.0020301)
Supplement: Table S47 — This table gives the 0.25, 0.5, and 0.75 quartile of the gene correlations (Pearson's) to their TOM group mean shown in Figure 2A and 2C. (27 KB DOC). [file pbio.0020301.st047.doc]

| **Group** | **25^th^%** | **50^th^%** | **75^th^%** |
| --- | --- | --- | --- |
| Tom 0 | 0.787 | 0.883 | 0.935 |
| Tom 1 | 0.65 | 0.749 | 0.856 |
| Tom 3 | 0.867 | 0.922 | 0.953 |
| Tom 6 | 0.854 | 0.918 | 0.952 |
| Tom 10 | 0.811 | 0.879 | 0.929 |
| Tom 30 | 0.743 | 0.884 | 0.921 |
